# ARDS Clinical Practice Guideline 2021

**DOI:** 10.1186/s40560-022-00615-6

**Published:** 2022-07-08

**Authors:** Sadatomo Tasaka, Shinichiro Ohshimo, Muneyuki Takeuchi, Hideto Yasuda, Kazuya Ichikado, Kenji Tsushima, Moritoki Egi, Satoru Hashimoto, Nobuaki Shime, Osamu Saito, Shotaro Matsumoto, Eishu Nango, Yohei Okada, Kenichiro Hayashi, Masaaki Sakuraya, Mikio Nakajima, Satoshi Okamori, Shinya Miura, Tatsuma Fukuda, Tadashi Ishihara, Tetsuro Kamo, Tomoaki Yatabe, Yasuhiro Norisue, Yoshitaka Aoki, Yusuke Iizuka, Yutaka Kondo, Chihiro Narita, Daisuke Kawakami, Hiromu Okano, Jun Takeshita, Keisuke Anan, Satoru Robert Okazaki, Shunsuke Taito, Takuya Hayashi, Takuya Mayumi, Takero Terayama, Yoshifumi Kubota, Yoshinobu Abe, Yudai Iwasaki, Yuki Kishihara, Jun Kataoka, Tetsuro Nishimura, Hiroshi Yonekura, Koichi Ando, Takuo Yoshida, Tomoyuki Masuyama, Masamitsu Sanui, ARDS Clinical Practice Guideline 2021 committee from the Japanese Society of Intensive Care Medicine, the Japanese Respiratory Society, and the Japanese Society of Respiratory Care Medicine, Takuro Nakashima, Aiko Masunaga, Aiko Tanaka, Akihiko Inoue, Akiko Higashi, Atsushi Tanikawa, Atsushi Ujiro, Chihiro Takayama, Daisuke Kasugai, Daisuke Kawakami, Daisuke Ueno, Daizoh Satoh, Shinichi Kai, Kohei Ota, Yoshihiro Hagiwara, Jun Hamaguchi, Ryo Fujii, Takashi Hongo, Yuki Kishihara, Naohisa Masunaga, Ryohei Yamamoto, Satoru Robert Okazaki, Ryo Uchimido, Tetsuro Terayama, Satoshi Hokari, Hitoshi Sakamoto, Dongli, Emiko Nakataki, Erina Tabata, Seisuke Okazawa, Futoshi Kotajima, Go Ishimaru, Haruhiko Hoshino, Hideki Yoshida, Hidetaka Iwai, Hiroaki Nakagawa, Hiroko Sugimura, Hiromichi Narumiya, Hiromu Okano, Hiroshi Nakamura, Hiroshi Sugimoto, Hiroyuki Hashimoto, Hiroyuki Ito, Hisashi Dote, Hisashi Imahase, Hitoshi Sato, Masahiro Katsurada, Ichiro Osawa, Jun Kamei, Jun Maki, Jun Sugihara, Jun Takeshita, Junichi Fujimoto, Junichi Ishikawa, Junko Kosaka, Junpei Shibata, Katsuhiko Hashimoto, Yasushi Nakano, Kazuki Kikuyama, Kazushige Shimizu, Kazuya Okada, Keishi Kawano, Keisuke Anan, Keisuke Ota, Ken-ichi Kano, Kengo Asano, Kenichi Hondo, Kenji Ishii, Kensuke Fujita, Kenta Ogawa, Kentaro Ito, Kentaro Tokunaga, Kenzo Ishii, Kohei Kusumoto, Kohei Takimoto, Kohei Yamada, Koichi Naito, Koichi Yamashita, Koichi Yoshinaga, Kota Yamauchi, Maki Murata, Makiko Konda, Manabu Hamamoto, Masaharu Aga, Masahiro Kashiura, Masami Ishikawa, Masayuki Ozaki, Michihiko Kono, Michihito Kyo, Minoru Hayashi, Mitsuhiro Abe, Mitsunori Sato, Mizu Sakai, Motoshi Kainuma, Naoki Tominaga, Naoya Iguchi, Natsuki Nakagawa, Nobumasa Aoki, Norihiro Nishioka, Norihisa Miyashita, Nozomu Seki, Ryo Ikebe, Ryosuke Imai, Ryota Tate, Ryuhei Sato, Sachiko Miyakawa, Satoshi Kazuma, Satoshi Nakano, Satoshi Tetsumoto, Satoshi Yoshimura, Shigenori Yoshitake, Shin-etsu Hoshi, Shingo Ohki, Shintaro Sato, Shodai Yoshihiro, Shoichi Ihara, Shota Yamamoto, Shunichi Koide, Shunsuke Kimata, Shunsuke Saito, Shunsuke Yasuo, Shusuke Sekine, Soichiro Mimuro, Soichiro Wada, Sosuke Sugimura, Tadashi Ishihara, Tadashi Kaneko, Tadashi Nagato, Takaaki Maruhashi, Takahiro Tamura, Takanori Ohno, Takashi Ichiyama, Takashi Niwa, Takashi Ueji, Takayuki Ogura, Takeshi Kawasaki, Takeshi Tanaka, Takeshi Umegaki, Taku Furukawa, Taku Omura, Takumi Nagao, Takuya Mayumi, Takuya Taniguchi, Takuya Yoshida, Tatsutoshi Shimatani, Teppei Murata, Tetsuya Sato, Tohru Sawamoto, Yoshifumi Koukei, Tomohiro Takehara, Tomomi Ueda, Tomoya Katsuta, Tomoya Nishino, Toshiki Yokoyama, Ushio Higashijima, Wataru Iwanaga, Yasushi Inoue, Yoshiaki Iwashita, Yoshie Yamada, Yoshifumi Kubota, Yoshihiro Suido, Yoshihiro Tomioka, Yoshihisa Fujimoto, Yoshihito Fujita, Yoshikazu Yamaguchi, Yoshimi Nakamura, Yoshinobu Abe, Yoshitomo Eguchi, Yoshiyasu Oshima, Yosuke Fukuda, Yudai Iwasaki, Yuichi Yasufuku, Yuji Shono, Yuka Nakatani, Yuki Nakamori, Yukie Ito, Yuko Tanabe, Yusuke Nagamine, Yuta Nakamura, Yutaro Kurihara

**Affiliations:** 1 Department of Respiratory Medicine, Hirosaki University Graduate School of Medicine, 5 Zaifucho, Hirosaki, Aomori 036-8562 Japan; 2 Department of Emergency and Critical Care Medicine, Graduate School of Biomedical and Health Sciences, Hiroshima University, Hiroshima, Japan; 3 Department of Intensive Care Medicine, Osaka Women’s and Children’s Hospital, Osaka, Japan; 4 Department of Emergency and Critical Care Medicine, Saitama Medical Center, Jichi Medical University, Saitama, Japan; 5 Division of Respiratory Medicine, Saiseikai Kumamoto Hospital, Kumamoto, Japan; 6 International University of Health and Welfare, Tokyo, Japan; 7 Department of Anesthesiology, Kobe University Hospital, Hyogo, Japan; 8 Department of Anesthesiology and Intensive Care Medicine, Kyoto Prefectural University of Medicine, Kyoto, Japan; 9 Department of Emergency and Critical Care Medicine, Graduate School of Biomedical and Health Sciences, Hiroshima University, Hiroshima, Japan; 10 Department of Pediatric Emergency and Critical Care Medicine, Tokyo Metropolitan Children’s Medical Center, Tokyo, Japan; 11 Division of Critical Care Medicine, National Center for Child Health and Development, Tokyo, Japan; 12 Department of Family Medicine, Seibo International Catholic Hospital, Tokyo, Japan; 13 Department of Primary Care and Emergency Medicine, Graduate School of Medicine, Kyoto University, Kyoto, Japan; 14 Department of Pediatrics, The University of Tokyo Hospital, Tokyo, Japan; 15 Department of Emergency and Intensive Care Medicine, JA Hiroshima General Hospital, Hiroshima, Japan; 16 Emergency and Critical Care Center, Tokyo Metropolitan Hiroo Hospital, Tokyo, Japan; 17 Division of Pulmonary Medicine, Department of Medicine, Keio University School of Medicine, Tokyo, Japan; 18 Paediatric Intensive Care Unit, The Royal Children’s Hospital, Melbourne, Australia; 19 Department of Emergency and Critical Care Medicine, Graduate School of Medicine, University of the Ryukyus, Okinawa, Japan; 20 Department of Emergency and Critical Care Medicine, Urayasu Hospital, Juntendo University, Chiba, Japan; 21 Department of Critical Care Medicine, Tokyo Metropolitan Bokutoh Hospital, Tokyo, Japan; 22 Department of Anesthesiology, Nishichita General Hospital, Tokai, Japan; 23 Tokyo Bay Urayasu Ichikawa Medical Center, Chiba, Japan; 24 Department of Anesthesiology and Intensive Care Medicine, Hamamatsu University School of Medicine, Shizuoka, Japan; 25 Department of Anesthesiology and Critical Care Medicine, Jichi Medical University Saitama Medical Center, Saitama, Japan; 26 Department of Emergency and Critical Care Medicine, Juntendo University Urayasu Hospital, Chiba, Japan; 27 Department of Emergency Medicine, Shizuoka General Hospital, Shizuoka, Japan; 28 Department of Anesthesia and Critical Care, Kobe City Medical Center General Hospital, Hyogo, Japan; 29 Department of Critical Care and Emergency Medicine, National Hospital Organization Yokohama Medical Center, Kanagawa, Japan; 30 Department of Anesthesiology, Osaka Women’s and Children’s Hospital, Osaka, Japan; 31 Division of Respiratory Medicine, Saiseikai Kumamoto Hospital, Kyoto, Japan; 32 Department of Intensive Care Medicine, Kameda Medical Center, Chiba, Japan; 33 Division of Rehabilitation, Department of Clinical Practice and Support, Hiroshima University Hospital, Hiroshima, Japan; 34 Pediatric Emergency and Critical Care Center, Saitama Children’s Medical Center, Saitama, Japan; 35 Department of Cardiovascular Medicine, Graduate School of Medical Science, Kanazawa University, Kanazawa, Japan; 36 Department of Psychiatry, School of Medicine, National Defense Medical College, Saitama, Japan; 37 Kameda Medical Center Department of Infectious Diseases, Chiba, Japan; 38 Division of Emergency and Disaster Medicine Tohoku Medical and Pharmaceutical University, Miyagi, Japan; 39 Department of Anesthesiology and Perioperative Medicine, Tohoku University Graduate School of Medicine, Miyagi, Japan; 40 Department of Emergency Medicine, Japanese Red Cross Musashino Hospital, Tokyo, Japan; 41 Department of Critical Care Medicine, Nerima Hikarigaoka Hospital, Tokyo, Japan; 42 Department of Traumatology and Critical Care Medicine, Osaka City University Graduate School of Medicine, Osaka, Japan; 43 Department of Anesthesiology and Pain Medicine, Fujita Health University Bantane Hospital, Aichi, Japan; 44 Division of Respiratory Medicine and Allergology, Department of Medicine, Showa University School of Medicine, Tokyo, Japan; 45 Intensive Care Unit, Department of Anesthesiology, Jikei University School of Medicine, Tokyo, Japan; 46 Department of Emergency and Critical Care Medicine, Jichi Medical University Saitama Medical Center, Saitama, Japan

**Keywords:** ARDS, Acute lung injury, Systematic review, Clinical practice guideline

## Abstract

**Background:**

The joint committee of the Japanese Society of Intensive Care Medicine/Japanese Respiratory Society/Japanese Society of Respiratory Care Medicine on ARDS Clinical Practice Guideline has created and released the ARDS Clinical Practice Guideline 2021.

**Methods:**

The 2016 edition of the Clinical Practice Guideline covered clinical questions (CQs) that targeted only adults, but the present guideline includes 15 CQs for children in addition to 46 CQs for adults. As with the previous edition, we used a systematic review method with the Grading of Recommendations Assessment Development and Evaluation (GRADE) system as well as a degree of recommendation determination method. We also conducted systematic reviews that used meta-analyses of diagnostic accuracy and network meta-analyses as a new method.

**Results:**

Recommendations for adult patients with ARDS are described: we suggest against using serum C-reactive protein and procalcitonin levels to identify bacterial pneumonia as the underlying disease (GRADE 2D); we recommend limiting tidal volume to 4–8 mL/kg for mechanical ventilation (GRADE 1D); we recommend against managements targeting an excessively low SpO_2_ (PaO_2_) (GRADE 2D); we suggest against using transpulmonary pressure as a routine basis in positive end-expiratory pressure settings (GRADE 2B); we suggest implementing extracorporeal membrane oxygenation for those with severe ARDS (GRADE 2B); we suggest against using high-dose steroids (GRADE 2C); and we recommend using low-dose steroids (GRADE 1B). The recommendations for pediatric patients with ARDS are as follows: we suggest against using non-invasive respiratory support (non-invasive positive pressure ventilation/high-flow nasal cannula oxygen therapy) (GRADE 2D), we suggest placing pediatric patients with moderate ARDS in the prone position (GRADE 2D), we suggest against routinely implementing NO inhalation therapy (GRADE 2C), and we suggest against implementing daily sedation interruption for pediatric patients with respiratory failure (GRADE 2D).

**Conclusions:**

This article is a translated summary of the full version of the ARDS Clinical Practice Guideline 2021 published in Japanese (URL: https://www.jsicm.org/publication/guideline.html). The original text, which was written for Japanese healthcare professionals, may include different perspectives from healthcare professionals of other countries.

**Supplementary Information:**

The online version contains supplementary material available at 10.1186/s40560-022-00615-6.

## Background

In Japan, the Japanese Society of Respiratory Care Medicine published the first and second editions of the Clinical Practice Guideline for acute respiratory distress syndrome (ARDS) in 1999 [1] and 2004 [2], respectively, after which the Japanese Respiratory Society published the Guideline for ARDS Practice in 2005 [3] and 2010 [4]. These could be referred to as guidebooks, so to speak, in the form of narrative reviews. Fundamentally, clinical guidelines need to go through a process of objectively determining recommendations for clinical questions (CQ) based on systematic reviews (SR) for evaluating the quality of evidence for a CQ.

The Japanese Society of Respiratory Care Medicine and Japanese Society of Intensive Care Medicine jointly established the ARDS Clinical Guideline creation committee in July 2014 with the aim of providing SRs and recommendations using the Grading of Recommendations Assessment, Development and Evaluation (GRADE) system. At the same time, the Japanese Respiratory Society had established a guideline creation committee; therefore, the three societies and two committees decided to create a domestically integrated clinical guideline. Specifically, the Japanese Respiratory Society was responsible for Part 1, which described the narrative review section in the form of a so-called scope. The Japanese Society of Respiratory Care Medicine and Japanese Society of Intensive Care Medicine were primarily responsible for Part 2, which focused on the SRs and recommendations for the 13 CQs; however, the SR reviewers were recruited from all three societies. The completed ARDS Clinical Guideline 2016 [5] was highly regarded as an international standard by the GRADE system even in the Appraisal of Guidelines for Research and Evaluation II (AGREEII) by the Japan Council for Quality Health Care. Meanwhile, some issues, such as inconsistent descriptions between Parts 1 and 2, remained. Therefore, when creating the present guideline, a joint committee was formed from the preparatory stage to unify the intentions of the guideline creation policy and methods.

Treatment outcomes have been improving mainly due to advances in respiratory management, but the mortality rate of ARDS remains high at approximately 25–40% [6]. The primary objective of this guideline was to provide support so that healthcare professionals from multiple disciplines, including non-specialist physicians, could make appropriate decisions to improve the outcomes of patients with ARDS. Therefore, a diverse range of areas need to be covered, from diagnosis to respiratory management, drug therapy, and physical therapy. As such, both the content and scale far exceed the previous edition. We hope that this guideline will serve as the basis for a platform that disseminates evidence relating to ARDS across clinical department disciplines.

## Methods

### Composition of ARDS Clinical Practice Guideline Creation Committee

The ARDS clinical practice guideline creation committee is composed of the following.

- Governing committee: mainly responsible for managing and operating the overall clinical practice guideline, from CQ proposals mainly for adults to the creation of the final draft of the clinical practice guideline.
- Pediatric steering committee: responsible for managing and operating the overall clinical practice guideline, from CQ proposals in the pediatric area to the creation of the final draft of the clinical practice guideline.
- SR steering and governing committee (area-governing team, education team, methodology team): responsible for leading the SR team that implements SRs in accordance with PICO and the CQs determined by the governing committee and panel meeting and also responsible for determining the SR methodology and its education.
- Support team: plays various auxiliary roles across the overall clinical practice guideline creation process (e.g., panel meeting operation, creation of various publications, management of various created files).
- SR team: responsible for implementing SRs in accordance with PICO and the CQs determined by the governing committee and panel meeting, as well as creating evidence profiles to be explained later.
- Clinical practice guideline consultant: plays a supervisory and consultative role as a clinical practice guideline expert across the entire process.

### Setting of important CQs

The governing committee selected 61 (adult: 46; children: 15) CQs with reference to the previous guideline and public comments (July–September 2019). The CQs were divided into the following five areas, with an area manager assigned for each.

- How should the diagnosis and prognosis prediction of ARDS be conducted?(area A)
- Should non-invasive respiratory support be used for patients with ARDS?(area B)
- How should invasive respiratory support be conducted for patients with ARDS?(area C)
- What and how should treatment adjacent to ventilator use be conducted for patients with ARDS?(area D)
- What and how should drug and non-drug therapy be conducted for patients with ARDS?(area E)

### Analytic framework

To extract and organize the components of the CQs that should undergo SR in the abovementioned key clinical issues, we created an analytic framework according to the clinical flow and set key questions (KQ) 1–5 based on the following definitions. This analytic framework was proposed mainly by the general committee members and responsible committee members for each SR area, and it was decided with the approval of the panel meeting. The CQs that underwent SR for each KQ in these key clinical issues were formulated using the PICO format (alternatively, PECO, PICOT, or other formats). Please see the attachment for the list of CQs.

- Key clinical issues related to diagnosis/prediction

- KQ1 (comprehensive question): Does diagnosis/prediction improve final prognosis?
- KQ2: Who should be subject to diagnosis/prediction?
- KQ3: What is the diagnostic/predictive performance of diagnosis/prediction outcomes?
- KQ4: What harms are there in diagnosis/prediction?
- KQ5: Is there a link between intermediate and final outcomes?

- Key clinical issues related to intervention

- KQ1 (comprehensive question): Can intervention improve final prognosis?
- KQ2: Who should be subject to interventions?
- KQ3: What is the effect of interventions on outcomes?
- KQ4: What harms are there in interventions?
- KQ5: Is there a link between intermediate and final outcomes?

### Creation of PICO sheet

The patients, their diagnosis/prediction, interventions, outcomes, etc., in the CQs, which were organized and formulated above, were organized in a sheet called a PICO sheet. This PICO sheet was proposed mainly by the general committee members and the responsible committee members of each SR area, and it was decided with the approval of the panel meeting. As the targets of this guideline are patients with ARDS, so the patients were set as patients with ARDS as a general rule. Some of the clinical questions targeted patients with acute respiratory failure suspected with ARDS according to the analytic framework. Furthermore, in this guideline, there are CQs relating to clinical practice areas, where there were few reports of clinical trials targeting only patients with ARDS, but where the results of clinical trials targeting intensive care patients or mechanical ventilation patients are applicable to patients with ARDS; for such CQs, the target patients were set as patients with similar pathological conditions, such as those receiving intensive care or mechanical ventilation.

### Relative importance of outcomes

Outcomes involved listing beneficial (benefits) and harmful (harms) aspects (no more than seven aspects), and a score of 1–9 (1: least important; 9: most important) was relatively assigned according to the GRADE system. The scoring was proposed mainly by the general committee members and the responsible committee members of each SR area, and it was decided with the approval of the panel meeting.

*In terms of scoring, if only a given outcome can occur, then scores were given for cases, where each outcome is critical (7–9 points), important (4–6 points), or not important (1–3 points) for decision making. As a general rule for recommendation, decisions were made using outcomes that were evaluated as “critical.”

### SR policy

For each CQ, each SR team conducted a preliminary search and selected one of the following four policies.

- Evaluating existing SRs and adapt their results (see below).
- Conducting a new SR.
- No SR conduction due to very scarce relevant literature available.
- No SR conduction due to low clinical importance of the CQ.

GRADE-ADOLOPMENT (evaluation and adaptation of existing SRs).

When evaluating existing SRs and adapting their results, such results are adapted when the following criteria are met.

- The clinical question matches.
- A comprehensive search of research is conducted.
- The eligibility criteria for the selection and exclusion of research are clearly defined.
- The risk of bias of research is properly and critically examined (e.g., Cochrane risk of bias tool in interventional studies, ROBINS, in observational studies).
- A quantitative integration in which estimates effects (meta-analysis) is conducted (when appropriate).
- There is no problem with the quality of the article after evaluating with AMSTAR2 or ROBINS.
- If the publication of the existing SR is more than 2 years before the search formula creation start date of April 2020, then an additional search is conducted with the same search formula.
- For an intervention-based SR, if there is a newer randomized controlled trial (RCT) published than the existing SR, then a new SR is conducted as a general rule.
- The existing SR articles and evaluated results (PICO, AMSTAR2) are presented to the methodology team.
- The content is reviewed by two members of the methodology team, who will confirm whether those articles can truly be used. Those results will be presented to the governing committee, and an approval (or rejection) will be presented as the final decision.
- The use of the existing SR will be determined according to the final decision of the governing committee. Even if the existing SR cannot be used, the use of the search formula or risk of bias will be left to the discretion of each team.
- When using the existing articles for the SR of RCTs, whether observational studies will be included will be determined on the basis of the outcome. If the RCTs include many large-scale studies, where a sufficient sample size/event occurrence size is ensured for the harms, then there is no need to evaluate observational studies. If there is no evaluation for the outcomes of harms, then observational studies will also be examined.

### SR and meta-analysis

#### Step 1: Literature search

A literature search is conducted based on previous studies, previous ARDS guidelines, etc., from which key articles thought to be applicable to the research question are extracted, and a literature search formula is created so that such key articles are always included. When creating the literature search formula, a final decision was made under the supervision of the Kyoto Prefectural University of Medicine Library. As a general rule, the literature search was conducted using MEDLINE (PubMed), CENTRAL, and Ichushi-Web as the databases. As a general rule, the search period was not limited. The languages used were either English or Japanese.

#### Step 2: Primary screening

All titles and abstracts specified in Step 1 were downloaded. The automatic duplicate deletion function of the literature management software EndNote (Clarivate Analytics, USA) or Mendeley (Mendeley Ltd., UK) were used to remove duplicates, with duplicate articles further deleted manually. The article screening work was conducted online using Rayyan (https://rayyan.qcri.org/welcome). Two independent reviewers reviewed the titles and abstracts of the literature, excluding those which clearly were different on the following points: intervention studies were selected on the basis of research design, patients, intervention method, and language; and diagnostic/predictive studies were selected on the basis of research design, patients, index test, reference standard, availability of 2 × 2 table, and language. If there were conflicting opinions between the reviewers, a third reviewer conducted an evaluation, with a decision made after discussion.

#### Step 3: Secondary screening (full-text review)

The full text was ordered based on the screening results of Step 2, and the target literature was selected using the entire main text. Intervention studies were selected on the basis of research design, patients, and intervention method. Diagnostic/predictive studies were selected on the basis of research design, patients, index test, reference standard, and availability of a 2 × 2 table. If there were conflicting opinions between the reviewers, a third reviewer conducted an evaluation, with a decision made after discussion. A flow diagram was created for the literature search and extraction process through the primary and secondary screening.

#### Step 4: Data extraction

Each team created a sheet in advance for extracting information relating to the studies; information was then extracted based on this sheet. The data were listed separately for each outcome determined in each CQ. When there was insufficient data, the authors were contacted as necessary.

#### Step 5: Qualitative evaluation, quantitative integration

The quality of evidence was evaluated according to the method advocated by the GRADE working group. For primary studies to be incorporated in the meta-analysis for each CQ, the standard bias evaluation tools such as the Cochrane risk of bias tool (Ver. 1) and QUADAS-2 tool were used to grade the evidence across the four stages of high, moderate, low, and very low. For the meta-analysis of each outcome data, the Cochrane Review Manager (RevMan5) software ver. 5.4 (http://tech.cochrane.org/revman) or general statistical software such as STATA, R, and SAS were used to conduct an appropriate quantitative integration according to the type of estimated value of the effect.

After making a final decision of the quality of evidence and conducting a meta-analysis, a summary of finding table (SoF table) and GRADE evidence profile table were created. GRADEpro GDT (http://gdt.guidelinedevelopment.org/) was used for the creation of both tables. After the SoF table and evidence profile were created by each SR team, mutual peer review was then conducted by the guideline supervisor, education team, and support team, centered on the methodology team. For area B, “Should non-invasive respiratory support be used for patients with ARDS?” a network meta-analysis was conducted, and the quality of evidence evaluation was conducted based on the GRADE working group method.

#### Step 6: Creation of body of evidence

Recommendations for each CQ were determined by examining the quality of evidence and the balance between benefits and harms, as well as patient values, costs, and resources. Specifically, a table called the evidence-to-decision (EtoD) framework was created based on the evidence profile, and this was submitted to the panel meeting for drafting the recommendations.

### Creation of recommendations

When an SR was conducted, or when an existing SR was evaluated and its results were adapted, the committee members (guideline supervisor, governing committee SR area supervisor, responsible committee members of each area, methodology team, education team, support team) collaborated in advance of the decision of recommendation and created drafts of the evidence to decision table (EtoD table) and recommendation text. The EtoD table and recommendation text drafts underwent mutual peer review with other supervisors and the support team, centered on the methodology team, and their contents were refined. The Minds guideline creation manual 2017 [7] was referenced in the creation of the recommendation text draft. Afterwards, the four factors of certainty of evidence, balance of effects, values and acceptability, and feasibility were examined based on the draft, and the recommendations were formulated by the panel meeting using the following procedure.

When there is no reliable evidence or when the SR was not conducted.

When SR was conducted but there was no reliable evidence (e.g., lack of applicable literature), or when an SR was not conducted, this was not subject to GRADE evaluation (ungraded). In these cases, the responsible committee members of the area as well as members of the SR governing committee (SR governing committee of each area, methodology team, education team) collaborated to create a recommendation text draft with reference to the following criteria, and a policy of (1) or (2) was decided by the panel meeting.

1. Recommendation as good practice statement (GPS)
2. Description of current state of standard clinical practice without recommendation (in our practice statement).

### Good practice statement

When confident that the certainty of the net benefit of a medical practice shown by indirect evidence is at the same level as this example even without a formal literature search, or when it is thought that the task of collecting linked indirect evidence supporting the applicable recommendation would be difficult and unproductive, then we considered presenting this as a GPS [8, 9]. In these cases, the recommendation was “recommended,” and the quality of evidence was “ungraded” (ungraded recommendation) [8, 9]. The recommendation text clarified “Good practice statement.” The panel meeting decided whether to support the GPS by a “yes/no” vote, with use of the GPS decided at 90% agreement.

### In our practice statement

When there was insufficient evidence for a recommendation and a GPS was not applicable, but guidance based on current clinical practice patterns was considered appropriate, we provided descriptions in the current standard clinical practice as an “In our practice statement” [8, 9]. An “In our practice statement” was intended to describe current care and was not intended to be interpreted as a recommendation [8].

### Recommendation decision in panel meeting

The modified Delphi method was used for consensus building at the panel meeting.

#### Step 1: Advance voting

Each committee member independently gave a score of 1–9 (1: disagree, 9: agree) for the created recommendation draft. Committee members who gave a score of < 7 also provided the reasons for the decision. Voting was conducted online and anonymously. An independent member who was not involved in voting (support team or governing committee secretariat) aggregated the results and calculated the score median, inter-percentile range, inter-percentile range adjusted for symmetry, and disagreement index (DI).

#### Step 2: Panel meeting

Panel meetings were conducted based on the aggregated results as shown below to reach consensus.

1. When there was no agreement (Median < 6.5 or DI ≥ 1)
  Discussions were held within the committee, after which amendments were made to the EtoD and recommended text, and a second vote was held.
  Voting was conducted up to three times.

2. When there was agreement (Median ≥ 6.5 and DI < 1)

When a serious opinion was present during voting for a comment or recommendation presented by committee member, discussions were held within the committee, and a consensus was reached. CQs for which a consensus was not reached within the committee resulted in amendments to the EtoD and recommended text, after which a second vote was held.

### Strength of recommendation

The strength of recommendation shown by the GRADE system was classified into four categories: 1, recommended/recommended against, and 2, suggested/suggested against (Table 1).

**Table 1:** The categories of the strength of recommendation shown by the GRADE system

Recommendation	Example	Notation
Recommended	“We recommend conducting xx (medical practice).”	1
Suggested	“We suggest conducting xx (medical practice).”	2
Recommended against	“We recommend against conducting xx (medical practice).”	1
Suggested against	“We suggest against conducting xx (medical practice).”	2

In addition, the certainty of evidence was classified as shown in Table 2.

**Table 2:** The definitions of the certainly of evidence

Certainty	Definition	Notation
High	Highly confident that the effect estimates supports the recommendation	A
Moderate	Moderate confidence that the effect estimates supports the recommendation	B
Low	Limited confidence that the effect estimates supports the recommendation	C
Very low	Almost no confidence in the effect estimates to support the recommendation	D

### Creation of recommendation text and public comments

After the recommendation text and strength of recommendation were determined, the governing committee wrote the commentary text. Afterwards, public comments were solicited from June 11th to June 24th, 2021, on the websites of each academic society. There were 10 comments during the designated period, and descriptions were added in the text.

### Recommendations on COVID-19

Recommendations for COVID-19 were not included in this guideline, because there is insufficient evidence for their inclusion.

## Results

Below, the CQs and their recommendations are described for the five adult areas and the pediatric area. Please refer to Additional files 1, 2, 3, 4, 5, 6 for the search strategies, flow diagram, risk of bias summaries, forest plot, EP and EtD of each CQ.

**I. Area A: Diagnosis/severity of illness evaluation/type evaluation**

### CQ1: Should ARDS diagnoses be conducted for patients with acute respiratory failure?

#### Background

ARDS is a serious and urgent pathological condition that causes acute respiratory failure. Its diagnosis can affect the understanding of these pathological conditions, severity of illness evaluation, and treatment policy, and whether to confirm the diagnosis of ARDS is an important clinical question.

#### Recommendation

We recommend that ARDS should be suspected for patients with acute respiratory failure (GPS).

#### Supplementary item

Not only is the diagnosis of ARDS important for improving prognosis but also the diagnosis and treatment of diseases that causes ARDS.

#### Rationale

*Summary of evidence*:

There are no clinical studies that compare whether confirming or not confirming ARDS in a patient with acute respiratory failure directly improves patient outcomes. When ARDS is diagnosed, there is a possibility that the prognosis improves as a result of conducting the evidence-based recommendations presented in this guideline.

Balance of effects, acceptability, and feasibility determination:

The diagnosis of ARDS enables important clinical decision-making, such as understanding the pathological condition, evaluating illness severity, and deciding on a treatment policy; furthermore, there is a possibility that the prognosis improves as a result of conducting evidence-based recommendations presented in this guideline. Therefore, there are large benefits in ARDS diagnosis in patients with acute respiratory failure. ARDS diagnosis is based on the Berlin Definition proposed in 2012. The minimum requirements for the diagnostic items in addition to medical history and physical findings are chest imaging tests (simple chest X-rays, chest CT) and arterial blood gas analysis; the harm to patients and additional costs to medical institutions as a result of these actions are thought to be trivial.

### CQ2: Should blood brain natriuretic peptide (BNP) and NT-proBNP levels be used for identifying cardiogenic pulmonary edema as the causative disease of acute respiratory failure?

#### Background

Identifying cardiogenic pulmonary edema is important in ARDS clinical practice. Blood BNP and NT-proBNP levels are widely used as a supplementary diagnosis for heart failure, and whether to use these for identifying the causative disease of acute respiratory failure is an important clinical question.

#### Recommendation

We suggest using blood BNP or NT-proBNP levels for identifying cardiogenic pulmonary edema in patients with acute respiratory failure (weak recommendation/very low certainty of evidence: GRADE 2D).

#### Supplementary conditions

Changes in clinical conditions (e.g., patient characteristics, testing characteristics and timing, pre-test probability, patient/medical staff values) may change the balance of effects and result in a different option being recommended.

#### Rationale

*Summary of evidence*:

We found 6 studies on BNP and NT-proBNP levels in the blood. Blood BNP (cutoff value: 400–500 pg/mL) (3 studies [10–12]: *N* = 252) had an integrated sensitivity of 0.77 (95% confidence interval [CI]: 0.65–0.85) and integrated specificity of 0.62 (95% CI: 0.53–0.70). Blood BNP (cutoff value: 1000 pg/mL) (2 studies [11, 12]: N = 128) had an integrated sensitivity of 0.50 (95% CI: 0.36–0.64) and integrated specificity of 0.82 (95% CI: 0.72–0.89). Additionally, blood NT-proBNP (cutoff value: 4,000 pg/mL) (1 study [13]: *N* = 121) had an integrated sensitivity of 0.71 (95% CI: 0.52–0.85) and integrated specificity of 0.89 (95% CI: 0.80–0.94). The implementation of testing is generally supported when taking into account the balance between desirable and undesirable effects due to the implementation of testing.

*Certainty of evidence*:

The quality of evidence for the diagnostic performance of testing was judged as “very low” for almost all cases.

*Balance of effects, acceptability, and feasibility determination*:

This is already used in routine clinical practice, and there are no problems with acceptability and feasibility.

### CQ3: Should serum C-reactive protein (CRP) and procalcitonin (PCT) levels be used for identifying bacterial pneumonia as the underlying disease of ARDS?

#### Background

Pneumonia is a common causative disease of ARDS, most of which is bacterial pneumonia. Whether to use CRP and PCT for early recognition of this bacterial pneumonia is an important clinical question.

#### Recommendation

We suggest against identifying bacterial pneumonia as the underlying disease of ARDS only with serum CRP and PCT results (weak recommendation/very low certainty of evidence: GRADE 2D).

#### Supplementary conditions

Changes in clinical conditions (e.g., patient characteristics, testing characteristics and timing, pre-test probability, patient/medical staff values) may change the balance of effects and result in a different option being recommended.

#### Rationale

*Summary of evidence*:

We found 14 studies relating to serum CRP and 21 studies relating to serum PCT. Serum CRP (14 studies [14–27]: *N* = 3093) had a sensitivity of 0.76 (95% CI: 0.63–0.89) and specificity of 0.78 (fixed). Serum PCT (21 studies [14–26, 28–35]: *N* = 4,721) had a sensitivity of 0.64 (95% CI: 0.56–0.73) and specificity of 0.83 (fixed) (estimates using HSROC model, fixed at median value of specificity of primary studies).

*Certainty of evidence*:

In consideration of the risk of bias, indirectness, inconsistency, and inaccuracy, and certainty of evidence relating to the effects of testing was judged as “very low.”

*Balance of effects, acceptability, and feasibility determination*:

When comparing patients who are suspected of having bacterial pneumonia based on the results of serum CRP or serum PCT tests and those who are treated as if they have bacterial pneumonia regardless of the results of these tests, the latter may be more beneficial in situations, where the pre-test probability is estimated to be above a certain level. Complications from testing are thought to be negligible in terms of clinical decision-making. This is a widely used test with probably no problem regarding feasibility.

### CQ4: Should pneumococcal urinary antigen tests and sputum Gram staining be used for identifying pneumococcal pneumonia as the causative disease of ARDS?

#### Background

Pneumococcal pneumonia is a cause of acute respiratory failure and ARDS, and early medical intervention is important. Whether to use urinary pneumococcal capsular antigen tests and sputum Gram staining for conducting the early diagnosis of pneumococcal pneumonia is an important clinical question.

#### Recommendation

We suggest the use of pneumococcal urinary antigen testing and sputum Gram staining for identifying pneumococcal pneumonia as the causative disease of ARDS (weak recommendation/very low certainty of evidence: GRADE 2D.

#### Supplementary conditions

Changes in clinical conditions (e.g., patient characteristics, testing characteristics and timing, pre-test probability, patient/medical staff values) may change the balance of effects and result in a different option being recommended.

#### Rationale

*Summary of evidence*:

Pneumococcal urinary antigen testing (23 studies [36–58]: *N* = 10,900) had an integrated sensitivity of 0.65 (95% CI: 0.61–0.68) and integrated specificity of 0.91 (95% CI: 0.85–0.95). Sputum Gram staining (11 studies [55, 59–68]: *N* = 1794) had an integrated sensitivity of 0.69 (95% CI: 0.56–0.80) and integrated specificity of 0.91 (95% CI: 0.83–0.96).

*Certainty of evidence*:

The certainty of evidence relating to diagnostic performance for each test was judged as “very low.”

*Balance of effects, acceptability, and feasibility determination*:

When comparing the effects and harms of testing with the case, where the treatment policy was decided without testing, it is thought that the desirable effects of testing are large. This is a generally accepted medical practice with no problems regarding feasibility.

### CQ5: Should *Legionella* urinary antigen testing be used for identifying *Legionella* pneumonia as the causative disease of ARDS?

#### Background

*Legionella* pneumonia presents with rapid respiratory failure and is important as a causative disease of ARDS. Meanwhile, there is a possibility that an improved prognosis can be obtained with *Legionella* pneumonia with an appropriate administration of antibiotics, so whether to use *Legionella* urinary antigen testing is an important clinical question.

#### Recommendation

We suggest using *Legionella* urinary antigen testing for identifying *Legionella* pneumonia as the causative disease of ARDS (weak recommendation/very low certainty of evidence GRADE 2D).

#### Supplementary conditions

Changes in clinical conditions (e.g., patient characteristics, testing characteristics and timing, pre-test probability, patient/medical staff values) may change the balance of effects and result in a different option being recommended.

#### Rationale

*Summary of evidence*:

We found 21 studies [41, 69–88] relating to *Legionella* urinary antigen testing (*N* = 11,724); the integrated sensitivity was 0.79 (95% CI: 0.71–0.85) and the integrated specificity was 1.00 (95% CI: 0.99–1.00).

*Certainty of evidence*:

Considering the risk of bias, indirectness, and inconsistency for both sensitivity and specificity, the certainty of evidence was judged to be “very low.”

*Balance of effects, acceptability, and feasibility determination*:

When comparing the effects and harms of *Legionella* urinary antigen testing with the case, where the treatment policy was decided without testing, the desirable effects of testing are thought to be large. This is a generally accepted medical practice with no problems regarding feasibility.

### CQ6: Should antigen and PCR tests of the pharyngeal swabs and serum antibody tests be used to identify *Mycoplasma* pneumonia as the causative disease of ARDS?

#### Background

*Mycoplasma* pneumonia is relatively common as a form of community-acquired pneumonia and can rarely progress to ARDS and present with serious respiratory failure. Antigen and PCR tests of the pharyngeal swabs and serum antibody tests are generally used for the diagnosis of *Mycoplasma* pneumonia. Verifying the benefits and harms due to the implementation of these tests is an important issue in ARDS clinical practice.

#### Recommendation

We cannot provide a specific recommendation regarding whether to use antigen and PCR tests of the pharyngeal swabs or serum antibody tests for identifying *Mycoplasma* pneumonia as a causative disease of ARDS. The current status of these tests is that they are used based on the experience of clinicians (in our practice statement).

#### Supplementary conditions

None.

#### Rationale

*Summary of evidence*:

No applicable studies.

Since there is no high-quality evidence that investigated the diagnostic accuracy of tests, no clear recommendations can be made for this CQ. Therefore, we provided a description of the current clinical practice rather than an evidence-based recommendation for this CQ.

*Certainty of evidence*:

Since there are no applicable studies, the quality of evidence cannot be evaluated.

*Balance of effects, acceptability, and feasibility determination*:

The balance between benefits and harms is unclear. It is thought that there are no problems with regard to acceptability and feasibility, since these tests are conducted in daily clinical practice.

### CQ7: Should antigen tests of the pharyngeal/nasopharyngeal swabs and PCR tests of the bronchoalveolar lavage fluid be used for identifying influenza pneumonia as the causative disease of ARDS?

#### Background

It has been reported that the administration of neuraminidase inhibitors reduced hospitalization, pneumonia onset, and mortality rate in influenza virus infections. Antigen tests based on rapid diagnostic kits using pharyngeal swabs or nasopharyngeal swabs are commonly used in the diagnosis of influenza virus infections. Meanwhile, there are some reports that indicated that antigen tests using upper respiratory tract specimens could not diagnose novel *Influenza* A (H1N1) pneumonia, with diagnoses made using PCR tests of bronchoalveolar lavage (BAL) fluid. Diagnosing influenza pneumonia with these tests and conducting therapeutic intervention based on the administration of the appropriate antiviral medication is important.

#### Recommendation

We cannot provide a specific recommendation regarding whether to use antigen tests of the pharyngeal swabs and/or PCR tests of the BAL fluid for identifying influenza pneumonia as the causative disease of ARDS. The current status of these tests is that they are used based on the experience of clinicians (in our practice statement).

#### Supplementary conditions

None.

#### Rationale

*Summary of evidence*:

No applicable studies.

*Certainty of evidence*:

Since there are no applicable studies, the quality of evidence cannot be evaluated.

*Balance of effects, acceptability, and feasibility determination*:

The balance between desirable effects and undesirable effects is unclear. It is thought that there are no problems regarding acceptability and feasibility.

### CQ8: Should PCR tests of the bronchoalveolar lavage fluid and blood antigenemia methods be used for identifying cytomegalovirus pneumonia as the causative disease of ARDS?

#### Background

The cytomegalovirus (CMV) pneumonia blood antigenemia method and CMV-PCR test of the BAL fluid are used for the early diagnosis of CMV infection and determination of the effect of treatment, and they are widely used as an index for monitoring, determining the effect of antiviral agents, and the discontinuation period.

#### Recommendation

We suggest using PCR tests of BAL fluid and blood antigenemia methods for identifying CMV pneumonia as the causative disease of ARDS (weak recommendation/very low certainty of evidence: GRADE 2D).

#### Supplementary conditions

Changes in clinical conditions may change the balance of effects and result in a different option being recommended.

#### Rationale

*Summary of evidence*:

PCR tests using BAL fluid (5 studies [89–93]: *N* = 353) had an integrated sensitivity of 0.94 (95% CI: 0.86–0.97) and integrated specificity of 0.84 (95% CI: 0.52–0.96).

Blood antigenemia methods (3 studies [94–96]: *N* = 91) had an integrated sensitivity of 0.67 (95% CI: 0.54–0.79) and integrated specificity of 0.87 (95% CI: 0.73–0.95) when the definition of “positive” was at least one positive cell per 200,000. Furthermore, the frequency of adverse events with BAL was as follows: mortality, 0.000% (95% CI: 0.000–0.035).

*Certainty of evidence*:

For both PCR tests of the BAL fluid and blood antigenemia methods, the certainty of evidence was judged as “very low” after considering the risk of bias, etc. The certainty of evidence regarding the harm of BAL was judged as “moderate” or “low.”

*Balance of effects, acceptability, and feasibility determination*:

Benefits included appropriate treatment based on diagnosis, and harms included unnecessary treatment due to misdiagnosis (false positive) and adverse events. When compared with cases, where the treatment policy was decided without testing, the benefits were thought to be large. This is a feasible medical practice.

### CQ9: Should serum β-D-glucan be used for identifying *Pneumocystis* pneumonia as the causative disease of ARDS?

#### Background

*Pneumocystis* pneumonia (PCP) causes acute respiratory failure, and it is anticipated that early diagnosis with serum β-D-glucan testing would contribute to the improved prognosis of patients. Meanwhile, misdiagnosis with PCP is thought to worsen patient prognosis through the administration of unnecessary drugs and its side effects, as well as the lack of implementation of treatments for other causative diseases. Verifying the benefits and harms of test implementation is an important issue in ARDS clinical practice.

#### Recommendation

We suggest using serum β-d-glucan tests for identifying PCP as the causative disease of ARDS (weak recommendation/very low certainty of evidence: GRADE 2D).

#### Supplementary conditions

Changes in clinical conditions (e.g., patient characteristics, testing characteristics and timing, pre-test probability, patient/medical staff values) may change the balance of effects and result in a different option being recommended.

#### Rationale

*Summary of evidence*:

When setting a cutoff value (80 pg/mL) for serum β-d-glucan (3 studies [97–99]: *N* = 148), the integrated sensitivity and integrated specificity of PCP diagnosis were 0.84 (95% CI: 0.66–0.93) and 0.79 (95% CI: 0.69–0.87), respectively.

*Certainty of evidence*:

The certainty of evidence relating to the diagnostic performance of PCP tests using serum β-d-glucan was judged as “very low.”

*Balance of effects, acceptability, and feasibility determination*:

The implementation of testing is generally supported when taking into account the balance between benefits and harms due to the implementation of testing. Meanwhile, the certainty of evidence relating to testing in general is very low. There is no problem with feasibility, and it is a generally accepted method.

### CQ10: Should serum β-d-glucan and galactomannan antigens of the blood or BAL fluid be used for identifying invasive pulmonary aspergillosis as the causative disease of ARDS?

#### Background

Invasive pulmonary aspergillosis (IPA) rapidly progresses and presents with respiratory failure and can also result in fatal outcomes. Therefore, whether to use biomarkers for identifying the causative disease of ARDS is an important clinical question.

#### Recommendation

We suggest against identifying IPA as the causative disease of ARDS using only serum β-d-glucan test results (weak recommendation/very low certainty of evidence: GRADE 2D).

We suggest identifying IPA as the causative disease of ARDS using galactomannan antigen tests in blood and BAL fluid (weak recommendation/very low certainty of evidence: GRADE 2D).

#### Supplementary conditions

Changes in clinical conditions (e.g., patient characteristics, testing characteristics and timing, pre-test probability, patient/medical staff values) may change the balance of effects and result in a different option being recommended.

#### Rationale

*Summary of evidence*:

When setting a cutoff value of 80 pg/mL for serum β-d-glucan (9 studies [100–108]: *N* = 757), the integrated sensitivity was 0.70 (95% CI: 0.49–0.85) and the integrated specificity was 0.73 (95% CI: 0.58–0.84). Additionally, when setting a cutoff value of 1.0 optical density index (ODI) for blood galactomannan antigen tests (8 studies [109–116]: *N* = 145), the integrated sensitivity was 0.76 (95% CI: 0.60–0.91) and the integrated specificity was 0.88 (95% CI: 0.79–0.94).

*Certainty of evidence*:

Considering the risk of bias, indirectness, inconsistency, and inaccuracy for both sensitivity and specificity in either test, the certainty of evidence was judged to be “very low.”

*Balance of effects, acceptability, and feasibility determination*:

It was thought that there were limited clinical conditions that could benefit from serum β-d-glucan tests, whereas there were many clinical conditions for galactomannan antigen tests. Both tests are generally accepted medical practices with no problems with regard to feasibility.

### CQ11: Should plain chest X-rays, chest high-resolution CT, and interferon γ release assays be used for identifying miliary tuberculosis as the causative disease of ARDS?

#### Background

Not only can miliary tuberculosis be the cause of ARDS and be fatal, the disease also requires prevention measures for airborne infection. Therefore, whether chest imaging tests or interferon γ release assays (IGRAs) should be conducted for identifying miliary tuberculosis as the causative disease of ARDS is an important question.

#### Recommendation

We cannot provide a specific recommendation regarding whether to use plain chest X-rays, chest high-resolution CT (HRCT), and IGRAs for identifying miliary tuberculosis as the causative disease of ARDS. Plain chest X-ray is conducted for almost all patients with respiratory failure; HRCT and IGRA are currently conducted based on the experience of clinicians (in our practice statement).

#### Rationale

*Summary of evidence*:

No applicable studies.

*Certainty of evidence*:

Since there are no applicable studies, the quality of evidence cannot be evaluated.

*Balance of effects, acceptability, and feasibility determination*:

The balance between benefits and harms is unclear. It is thought that there are no problems with regard to acceptability and feasibility.

### CQ12: Should lung pathology or chest CT imaging be used for predicting prognosis of ARDS patients?

#### Background

ARDS typically presents with pathologically diffuse alveolar damage, but the Berlin Definition does not necessarily refer to pathological conditions. Whether to use lung pathology and chest CT imaging for predicting prognosis of patients with ARDS is an important clinical question.

#### Recommendation

We suggest against using only lung pathology or only chest CT imaging for predicting prognosis of patients with ARDS (weak recommendation/very low certainty of evidence: GRADE 2D).

#### Supplementary conditions

Changes in clinical conditions (e.g., patient characteristics, testing characteristics and timing, pre-test probability, patient/medical staff values, decision-making following test results) may result in a different option being recommended.

#### Rationale

*Summary of evidence*:

Lung biopsies (11 studies [117–127]: *N* = 616) had an integrated sensitivity of 0.42 (95% CI: 0.21–0.57) and integrated specificity of 0.69 (fixed). Chest CT (6 studies [128–133]: *N* = 409) had an integrated sensitivity of 0.62 (95% CI: 0.32–0.91) and an integrated specificity of 0.76 (fixed).

*Certainty of evidence*:

In consideration of the risk of bias, indirectness, inconsistency, and inaccuracy for both lung biopsies and chest CT, the certainty of evidence was judged as “very low.”

*Balance of effects, acceptability, and feasibility determination*:

When considering the net benefits after including adverse events associated with tests, it is thought that there was a very limited number of clinical conditions, where determining the treatment policy using lung biopsies or chest CT test results is useful. It was judged that a general statement could not be made regarding the feasibility of lung biopsies and chest CT tests.

### CQ13: Should PaO_2_/FIO_2_ (*P/F*) ratio be used for predicting prognosis of patients with ARDS?

#### Background

The Berlin Definition stipulated the severity of ARDS according to the *P/F* ratio, but its prognosis prediction performance is unclear. Whether to use the *P/F* ratio for ARDS patient prognosis prediction was thought to be clinically important.

#### Recommendation

We suggest against ARDS patient prognosis prediction using only *P/F* ratio (cutoff: 100, 200) results (weak recommendation/very low certainty of evidence: GRADE 2D).

#### Supplementary conditions

The *P/F* ratio is used to determine the severity of illness and treatment policy in clinical practice, but this recommendation does not reject the use of the *P/F* ratio in such circumstances. Additionally, changes in clinical conditions (e.g., patient characteristics, testing characteristics and timing, pre-test probability, patient/medical staff values) may change the balance of effects and result in a different option being recommended.

#### Rationale

*Summary of evidence*:

When the cutoff value is 100 (19 studies [129, 134–151]: *N* = 15,040), the integrated sensitivity was 0.43 (95% CI: 0.37–0.50) and integrated specificity was 0.70 (95% CI: 0.66–0.74). When the cutoff value was 200 (20 studies [129, 134, 135, 137–150, 152–154]: *N* = 15,489), the integrated sensitivity was 0.85 (95% CI: 0.81–0.88) and integrated specificity was 0.24 (95% CI: 0.21–0.29).

*Certainty of evidence*:

In consideration of the risk of bias, inconsistency, and inaccuracy, the certainty of evidence was judged as “very low.”

*Balance of effects, acceptability, and feasibility determination*:

When considering the net benefits, there was a very limited number of clinical conditions, where determining the treatment policy using *P/F* ratio test results was useful. The *P/F* ratio is a commonly conducted medical practice and is thought to be feasible.

**II. Area B: Non-invasive respiratory support**

### CQ 14: Should non-invasive respiratory support be used for patients with ARDS?

#### Background

Non-invasive respiratory support such as non-invasive positive pressure ventilation (NPPV) and high-flow nasal cannula oxygen therapy (HFNC) is expected to avoid complications due to tracheal intubation, but delays in tracheal intubation have been reported to increase mortality rate. However, NPPV and HFNC are not established treatments in the respiratory management of patients with ARDS.

#### Recommendation

We suggest conducting non-invasive respiratory support (NPPV, HFNC) instead of conventional oxygen therapy as an initial respiratory management for adult patients with acute respiratory failure suspected of having ARDS if there are no contraindications for non-invasive respiratory support or if organ failure other than respiratory failure is absent.

NPPV (weak recommendation/moderate certainty of evidence: GRADE 2B).

HFNC (weak recommendation/moderate certainty of evidence: GRADE 2B).

We suggest conducting non-invasive respiratory support (NPPV, HFNC) over tracheal intubation.

NPPV (weak recommendation/moderate certainty of evidence: GRADE 2B).

HFNC (weak recommendation/moderate certainty of evidence: GRADE 2B).

#### Supplementary item

Delays in necessary tracheal intubation could increase mortality, so patients should be carefully managed in an environment, where tracheal intubation could be conducted after the start of non-invasive respiratory support.

Contraindications for non-invasive respiratory support include the inability to protect the airway, high risk of vomiting, impaired consciousness, uncooperative patients, and unstable hemodynamics.

#### Rationale

*Summary of evidence*:

This CQ included examinations using network meta-analyses (see CQ15–18 for details). The surface under the cumulative ranking curve (SUCRA), which was quantified as the average rating, was used to plot the SUCRA values of each device (from a minimum of 0 to maximum of 100), with short-term mortality on the horizontal axis and pneumonia on the vertical axis.

In order of descending favorability, the ranking of short-term mortality and pneumonia was 1. HFNC, 2. NPPV, 3. mechanical ventilation (MV), and 4. Oxygen therapy. The SUCRA values for short-term mortality were 72.0 for HFNC, 66.3 for NPPV, 49.9 for MV, and 11.7 for oxygen therapy; the SUCRA values for pneumonia were 92.3 for HFNC, 71.9 for NPPV, 33.1 for oxygen therapy, and 2.7 for MV.

Considering the ranking results, there may be little difference in the effects of reducing mortality between NPPV and HFNC among the types of non-invasive respiratory support.

### CQ 15: Should NPPV be used over oxygen therapy for patients with ARDS?

#### Background

NPPV is used for patients with acute respiratory failure to avoid complications due to tracheal intubation, but delays in tracheal intubation increase mortality risk. NPPV is effective for cardiogenic pulmonary edema and acute exacerbations of chronic obstruction pulmonary disease (COPD), but NPPV is not an established treatment for patients with ARDS.

#### Recommendation

We suggest conducting NPPV over conventional oxygen therapy as an initial respiratory management for adult patients with acute respiratory failure suspected of having ARDS if there are no contraindications for non-invasive respiratory support or if organ failure other than respiratory failure is absent (weak recommendation/moderate certainty of evidence: GRADE 2B).

#### Supplementary item

Because delays in necessary tracheal intubation could increase mortality, patients should be carefully managed in an environment, where tracheal intubation can be conducted after the start of NPPV.

Contraindications for non-invasive respiratory support include the inability to protect the airway, high risk of vomiting, impaired consciousness, uncooperative patients, and unstable hemodynamics.

#### Rationale

*Summary of evidence*:

Network meta-analysis was conducted using 19 RCTs (*N* = 2777) [155–173]. Short-term mortality (direct comparison 14 RCTs [155, 158, 160, 162–171, 173]: *N* = 1494) occurred in 107 fewer/1000 patients (166 fewer–31 fewer patients); tracheal intubation (direct comparison 14 RCTs [155, 158, 160, 163–171, 173]: *N* = 1495) was performed in 135 fewer/1000 patients (188 fewer–69 fewer patients); pneumonia (direct comparison 8 RCTs: *N* = 804) occurred in 50 fewer/1000 patients (75 fewer–11 fewer patients. From the above, the desirable effect of NPPV was judged to be “moderate.”

There were 2 RCTs [167, 170] that reported on skin disorders, which was an important outcome relating to harm; this did not occur with conventional oxygen therapy, whereas 3.2–25.5% of patients undergoing NPPV experienced skin disorders. From the above, the undesirable effect of NPPV was judged to be “trivial.”

*Certainty of evidence*:

The certainty of evidence and direction of all effects were consistent, and the certainty of evidence for the overall outcome was judged as “moderate” adopting the highest certainty of evidence.

*Values, balance of effects, acceptability, and feasibility determination*:

A dedicated device is needed for NPPV, and this may increase the burden on nurses; however, this is already implemented in the daily clinical practice of many acute care hospitals. As such, even when considering cost, harmfulness, etc., it was judged that it was “probably yes” acceptable and feasible.

### CQ 16: Should HFNC be used over conventional oxygen therapy for patients with ARDS?

#### Background

HFNC is used for patients with acute respiratory failure to avoid complications due to tracheal intubation, but delays in tracheal intubation increase mortality risk. However, HFNC is not an established treatment in the respiratory management of patients with ARDS.

#### Recommendation

We suggest conducting HFNC over oxygen therapy as an initial respiratory management for adult patients with acute respiratory failure suspected of having ARDS if there are no contraindications for non-invasive respiratory support or if organ failure other than respiratory failure is absent (weak recommendation/moderate certainty of evidence: GRADE 2B).

#### Supplementary item

Because delays in necessary tracheal intubation could increase mortality, patients should be carefully managed in an environment, where tracheal intubation can be conducted after the start of HFNC.

Contraindications for non-invasive respiratory support include the inability to protect the airway, high risk of vomiting, impaired consciousness, uncooperative patients, and unstable hemodynamics.

#### Rationale

*Summary of evidence*:

Network meta-analysis was conducted using 19 RCTs (*N* = 2777) [155–173]. Short-term mortality direct comparison 2 RCTs: *N* = 976) occurred in 117 fewer/1000 patients (215 fewer–44 more patients); intubation (direct comparison 4 RCTs [156, 159–161]: *N* = 1176) was performed in 89 fewer/1000 patients (187 fewer–127 more); pneumonia (direct comparison 1 RCT [160]: *N* = 200) occurred in 82 fewer/1000 patients (110 fewer–4 more patients). From the above, the desirable effect of HFNC was judged to be “moderate.”

*Certainty of evidence*:

The direction of all effects was consistent, and the certainty of evidence for the overall outcome was judged as “moderate” adopting the highest certainty of evidence.

*Values, balance of effects, acceptability, and feasibility determination*:

The balance of effects of HFNC was judged as “probably favors the intervention.” A dedicated device is needed for HFNC, and a large amount of oxygen is needed compared with oxygen therapy, but this is already implemented in the daily clinical practice of many acute care hospitals, so it was judged that it was “probably yes” acceptable and feasible.

### CQ 17: Should NPPV be used prior to conducting tracheal intubation in patients with ARDS?

#### Background

NPPV is used for patients with acute respiratory failure to avoid complications due to tracheal intubation, but delays in tracheal intubation increase mortality risk. NPPV is effective for cardiogenic pulmonary edema and acute exacerbations of COPD, but NPPV is not an established treatment for patients with ARDS.

#### Recommendation

We suggest conducting NPPV over tracheal intubation as an initial respiratory management for adult patients with acute respiratory failure suspected of having ARDS if there are no contraindications for non-invasive respiratory support or if organ failure other than respiratory failure is absent (weak recommendation/moderate certainty of evidence: GRADE 2B).

#### Supplementary item

Because delays in necessary tracheal intubation could increase mortality, patients should be carefully managed in an environment, where tracheal intubation can be conducted after the start of NPPV.

Contraindications for non-invasive respiratory support include the inability to protect the airway, high risk of vomiting, impaired consciousness, uncooperative patients, and unstable hemodynamics.

#### Rationale

*Summary of evidence*:

Network meta-analysis was conducted using 19 RCTs (*N* = 2777) [155–173]. Short-term mortality (direct comparison 2 RCTs [157, 171]: *N* = 129) occurred in 31 fewer/1000 patients (203 fewer–296 more patients); pneumonia (direct comparison 2 RCTs [157, 171]: *N* = 129) occurred in 401 fewer/1000 patients (450 fewer–295 fewer patients). From the above, the desirable effect of NPPV was judged to be “moderate.”

There was 1 RCT [157] that reported on skin disorders, which was an important outcome relating to harm; there were more cases of skin disorders with NPPV (3/32 [9.4%] vs. 0/32 [0%]). From the above, the undesirable effect of NPPV was judged to be “trivial.”

*Certainty of evidence*:

The direction of all effects was consistent, and the certainty of evidence for the overall outcome was judged as “moderate” adopting the highest certainty of evidence.

*Values, balance of effects, acceptability, and feasibility determination*:

The balance of effects of NPPV was judged as “probably favors the intervention.” A dedicated device is needed for NPPV, but this is relatively cheap compared with a ventilator. It is already implemented in the daily clinical practice of many acute care hospitals, so it was judged that it was “probably yes” acceptable and feasible.

### CQ 18: Should HFNC be used prior to conducting tracheal intubation in patients with ARDS?

#### Background

HFNC is used for patients with acute respiratory failure to avoid complications due to tracheal intubation, but delays in tracheal intubation increase mortality risk. However, HFNC is not an established treatment in the respiratory management of patients with ARDS.

#### Recommendation

We suggest conducting HFNC over tracheal intubation as an initial respiratory management for adult patients with acute respiratory failure suspected of having ARDS if there are no contraindications for non-invasive respiratory support or if organ failure other than respiratory failure is absent (weak recommendation/moderate certainty of evidence: GRADE 2B).

#### Supplementary item

Because delays in necessary tracheal intubation could increase mortality, patients should be carefully managed in an environment, where tracheal intubation can be conducted after the start of HFNC.

Contraindications for non-invasive respiratory support include the inability to protect the airway, high risk of vomiting, impaired consciousness, uncooperative patients, and unstable hemodynamics.

#### Rationale

*Summary of evidence*:

Network meta-analysis was conducted using 19 RCTs (*N* = 2777) [155–173]. Short-term mortality (no direct comparison) occurred in 56 fewer/1000 patients (248 fewer–386 more patients); pneumonia (no direct comparison) occurred in 440 fewer/1000 patients (478 fewer–295 fewer patients). From the above, the desirable effect of HFNC was judged to be “moderate.”

The risk of aspiration during HFNC is unclear, but pneumonia incidence decreased, and this was not an undesirable effect. Positive pressure due to HFNC (60 L/min) was approximately 4 cmH_2_O, and the possibility of lung damage was low. From the above, the undesirable effect of HFNC was judged to be “trivial.”

*Certainty of evidence*:

The direction of all effects was consistent, and the certainty of evidence for the overall outcome was judged as “moderate” adopting the highest certainty of evidence.

*Values, balance of effects, acceptability, and feasibility determination*:

The balance of effects of HFNC was judged as “probably favors the intervention.” A dedicated device is needed for HFNC, but this is relatively cheap compared with a ventilator. It is already implemented in the daily clinical practice of many acute care hospitals, so it was judged that it was “probably yes” acceptable and feasible.

**III. Area C: Invasive respiratory support**

### CQ 19: Should low tidal volume be used in mechanically ventilated adult patients with ARDS?

#### Background

Several studies have suggested that mechanical ventilation may cause lung damage and bleeding. Limiting tidal volume is one of the lung protection strategies. Its effects have been verified in several large-scale RCTs, the results of which, however, are inconsistent.

#### Recommendation

We recommend limiting tidal volume to 4–8 mL/kg for mechanically ventilated adult patients with ARDS (strong recommendation/very low certainty of evidence: GRADE 1D).

#### Supplementary item

The criteria for adoption of the studies used in the SR were 4–8 mL/kg for the low tidal volume ventilation group and > 8 mL/kg for the conventional tidal volume ventilation group, from which the present results were obtained, so the above recommendations were made. The SR results had a very low certainty of evidence, but low tidal volume is thought to be commonly used in daily clinical practice, so the recommendation was changed to “strong recommendation” as a result of re-voting at the panel meeting.

#### Rationale

*Summary of evidence*:

By limiting tidal volume, short-term mortality (11 RCTs [174–184]: *N* = 1795) occurred in 85 fewer/1000 patients (95% CI: 138 fewer–24 fewer), ventilator-free days (VFD) (4 RCTs [178, 180, 181, 183]: *N* = 1045) was extended by an average of 3.28 days (95% CI: 0.73 days shorter–5.82 days longer), and barotrauma (7 RCTs [174–176, 178, 179, 181, 183]: *N* = 1551) was 1 more/1000 patients (95% CI: 27 fewer–37 more). From the above, the predicted desirable effect was judged as “moderate.” Meanwhile, there were no significant outcomes for harms, and the undesirable effects were judged as “do not know.” From the above, the balance of effects and harms was judged to be “probably favors the intervention.”

*Certainty of evidence*:

Because the direction of outcomes among desirable effects was not the same, the certainty of evidence for the overall outcome was judged as “very low,” which was the lowest certainty of evidence of all the outcomes.

*Values, balance of effects, acceptability, and feasibility determination*:

The increased burden and cost to the patient of limiting the amount of ventilation is considered to be small, and the feasibility was judged as “yes”.

### CQ 20: Should high levels of positive end-expiratory pressure (PEEP) be used for mechanically ventilated adult patients with ARDS?

#### Background

The use of high levels of PEEP is a strategy for reducing ventilator-associated lung injury (VALI).

#### Recommendation

We suggest using high levels of PEEP for mechanically ventilated adult patients with ARDS.

(weak recommendation/very low certainty of evidence: GRADE 2D).

#### Supplementary item

In the studies included in this SR, the PEEP values on day 1 in the intervention group ranged from 10 to 16.3 cmH_2_O and from 6.5 to 12.0 cmH_2_O in the control group. The most common methods of setting PEEP in the intervention group were setting by Pflex and setting by ARDSnet PEEP table.

#### Rationale

*Summary of evidence*:

With the use of high levels of PEEP, short-term mortality (15 RCTs [174, 180, 183, 185–196]: *N* = 4108) occurred in 55fewer/1000 patients (95% CI: 103 fewer–0 fewer patients), VFD (11 RCTs [180, 183, 185, 186, 188, 189, 191–195]: *N* = 2988) was extended by an average of 1.82 days (95% CI: 0.37 days shorter–4.01 days longer), and the length of hospitalization (6 RCTs [187, 190–194]: *N* = 2392) was extended by an average of 0.86 days (95% CI: 3.08 days shorter–4.8 days longer). From the above, the predicted desirable effect was judged as “moderate.” Meanwhile, barotrauma (10 RCTs [174, 183, 185–188, 191–193, 195]: *N* = 2861) occurred in 1 more/1000 patients (95% CI: 23 fewer–39 more patients). From the above, the undesirable effect was judged as “trivial.” Therefore, the balance of effects and harms due to intervention was judged as “probably favors the intervention.”

*Certainty of evidence*:

Because the directions of desired and undesirable effects were not consistent, the certainty of evidence for the overall outcome was judged as “very low” by adopting the lowest certainty of evidence.

*Values, balance of effects, acceptability, and feasibility determination*:

Because the increased burden on patients and cost of using higher PEEP is considered to be small, the cost was judged as “small” compared with the benefit.

### CQ21: Should plateau pressure be limited for mechanically ventilated adult patients with ARDS?

#### Background

Increased airway pressure is thought to be one of the factors causing VALI, and limiting plateau pressure is expected to suppress the development of VALI [178, 197]. Meanwhile, limiting the plateau pressure may induce adverse events, such as hypercapnia.

#### Recommendation

We suggest plateau pressure restrictions when using a ventilator on adult patients with ARDS (weak recommendation/very low certainty of evidence: GRADE 2D).

#### Supplementary item

The present results may not suggest that desirable effects would always outweigh the undesirable effects, and if the tidal volume or transpulmonary pressure is appropriately restricted, then it may not be possible to state that high plateau pressure is always harmful.

#### Rationale

*Summary of evidence*:

A total of 6 RCTs [175, 178, 181, 183, 187, 188] (*N* = 2882) were found. By limiting plateau pressure, the following outcomes were found to be beneficial: short-term mortality occurred in 6 more/1000 patients (95% CI: 54 fewer–81 more patients) [178, 183, 187, 188], long-term mortality occurred in 4 fewer/1000 patients (95% CI: 99 fewer–118 more patients) [175, 181, 188], VFD was extended by an average of 0.82 days (95% CI: 4 days shorter–5.65 days longer) [181, 183, 188], and barotrauma occurred in 11 fewer/1000 patients (95% CI: 30 fewer–12 more patients) [175, 178, 181, 187, 188]. Therefore, the desirable effect of the intervention was judged to be trivial. Meanwhile, with regard to harmful outcomes, there were no reports of death due to hypercapnia. In the RCT [187] included in this meta-analysis, adverse events included refractory acidosis (42/508) and deaths due to refractory acidosis (38/508). Therefore, the undesirable effects of the intervention was judged as “trivial.” Therefore, the balance of effects and harms was judged as “do not know.”

*Certainty of evidence*:

Because the direction of outcomes in desired and undesirable effects was inconsistent, the certainty of evidence for the overall outcome was judged as “very low” by adopting the lowest certainty of evidence.

*Balance of effects, acceptability, and feasibility determination*:

This is already used in daily clinical practice and is thought to have sufficiently high acceptability and feasibility.

### CQ22: Which, between pressure-control ventilation (PCV) and volume-control ventilation (VCV), is desirable for mechanical ventilation in adult patients with ARDS?

#### Background

The ventilation modes of commonly used ventilators are VCV and PCV, but it is unclear which between the ventilation modes is more beneficial for patient prognosis.

#### Recommendation

From the results of this SR, we cannot provide a recommendation on which ventilation mode between PCV and VCV should be used for adult patients with ARDS (in our practice statement).

#### Supplementary item

VCV has the advantage of detecting changes in compliance and airway resistance and may be considered for use in facilities that are familiar with its use. The panel voted to make this an “in our practice statement” without providing a recommendation.

#### Rationale

*Summary of evidence*:

The use of PCV as a ventilation mode resulted in the length of mechanical ventilation shortening by an average of 4.1 days (95% CI: 6.84 days shorter–1.37 days shorter) [198–200], and mortality decreasing by 149/1000 patients (95% CI: 270 fewer–11 more patients) [198–200]. From the above results, the desirable effect of the intervention was judged to be “moderate.” Meanwhile, VALI was not reported in the reports adopted in this SR. Therefore, we cited the meta-analysis of Chacko et al. [201], which included 2 RCTs, which reported that VALI occurred in 23/1000 patients (95% CI: 12 fewer–72 more patients) due to PCV, and the undesirable effect was judged to be trivial. As a result, it was judged that the effect of the intervention was probably greater than the harm.

*Certainty of evidence*:

Because the direction of desirable and undesirable effects was inconsistent, the certainty of evidence for the overall outcome was judged as “very low” by adopting the lowest certainty of evidence.

*Balance of effects, acceptability, and feasibility determination*:

Since the potential for PCV to increase VALI is small, the benefits of PCV were considered to outweigh the harms. In addition, the use of PCV does not increase costs or burdens, so it is considered to be sufficiently feasible.

### CQ23: Should airway pressure release ventilation (APRV) be used in the mechanical ventilation of adult patients with ARDS?

#### Background

APRV is frequently used for ARDS, because it can maintain high airway pressures, but it is unclear if APRV is more beneficial than commonly used ventilation modes, such as VCV and PCV.

#### Recommendation

We cannot provide a recommendation on whether APRV or conventional ventilation modes should be used in adult patients with ARDS. APRV could be considered when using mechanical ventilation with spontaneous respiration (in our practice statement).

#### Supplementary item

The subgroup analysis that excluded inverse ratio ventilation showed desirable effects on VFD, mortality, and barotrauma in the APRV group. However, the panel meeting could not come to an agreement about making recommendation including acceptability and feasibility and decided to present this as an in our practice statement.

#### Rationale

*Summary of evidence*:

A meta-analysis was conducted with 7 RCTs [202–208]. APRV resulted in VFD being extended by an average of 3.64 days (0.02 days shorter–7.3 days longer) [202–208], mortality being decreased by 72/1000 patients (125 fewer–5 more patients) [202–208], and barotrauma occurring in 23 fewer/1000 patients (41 fewer–42 more patients) [202, 203, 208]. From the above results, the desirable effect due to the intervention was judged as “moderate.” Meanwhile, no clear harmful outcomes were observed, and the undesirable effect was judged as “do not know.” From the above results, the effect of APRV was judged to be probably favours. The same results were obtained in a meta-analysis of only classical APRV.

*Certainty of evidence*:

Because the direction of the desirable and undesirable effects was consistent in both the overall analysis and the analysis using only classical APRV, the certainty of evidence for the overall outcome was judged as “very low.”

*Balance of effects, acceptability, and feasibility determination*:

Since it is difficult to conduct a typical APRV with other than a specific model of ventilator, additional costs are required. In addition, the appropriate use of APRVs may require skilled staff, and therefore, the use of APRVs should be addressed on a facility-specific basis.

### CQ24: Which is preferable for ventilatory management of adult patients with ARDS: synchronized intermittent mandatory ventilation (SIMV) or assisted controlled ventilation (A/C)?

#### Background

SIMV and A/C are available in standard ventilators, but it is unclear which between the two is more effective.

#### Recommendation

We cannot provide a recommendation from the results of the present SR on which mode should be used in adult patients with ARDS (in our practice statement).

#### Supplementary item

Considering the risks of increased asynchrony and extended time until ventilator removal, A/C is used more often than SIMV.

#### Rationale

*Summary of evidence*:

With SIMV use, the length of mechanical ventilation decreased by 0 days (7.41 fewer days–7.41 more days) [209], and mortality decreased by 100/1000 patients (272 fewer–308 more patients) [209]. From the above results, the expected desirable effect was judged as “small.” Meanwhile, there was an observational study that showed a high incidence of asynchrony with the use of SIMV and a study demonstrating that the time to ventilator weaning was longer when SIMV was used as a ventilator weaning mode [210–212]. From the above, the expected undesirable effect was judged as “do not know.” Therefore, the balance of effects and harms was judged to be “probably favors the intervention.”

*Certainty of evidence*:

The only RCT included in the meta-analysis had a small sample size of 40, and mortality and length of mechanical ventilation were secondary outcomes [209]. There was a trend toward higher midazolam use in the A/C group, which may have influenced delirium, asynchrony, ventilator days, and mortality. These findings made it difficult to make recommendations for the use of SIMV and A/C in this meta-analysis.

*Balance of effects, acceptability, and feasibility determination*:

It is thought that the costs of changing the mode of mechanical ventilation are low.

### CQ25: When using mechanical ventilation in adult patients with ARDS with spontaneous breathing, is pressure support ventilation (PSV) or A/C preferred?

#### Background

It is unclear which is more effective, PSV or A/C, which is commonly used in patients with preserved spontaneous breathing.

#### Recommendation

We cannot provide a recommendation on whether PSV or A/C should be used in adults with ARDS. It is common practice to select the ventilation mode based on the individual patient’s condition and status (in our practice statement).

#### Supplementary item

The appropriate mode should be selected considering the presence of spontaneous breathing, use of sedatives or muscle relaxants, and severity of ARDS in each patient.

#### Rationale

Asynchrony between the patient and ventilator can lead to lung injury from mechanical ventilation, which can adversely affect hemodynamics and ultimately lead to patient death.

PSV is a ventilation mode that preserves the patient’s spontaneous breathing and provides support during inspiration. Compared with the A/C mode, PSV may be less likely to cause patient–ventilator asynchrony and result in lung injury. Additionally, the dose of sedatives could also be reduced due to the increased comfort of breathing in PSV. However, because PSV is a ventilation mode that relies on spontaneous breathing, if the patient’s own spontaneous respiratory effort is too strong, it may increase the tidal volume and compromise the lung-protective ventilation.

*Summary of evidence*:

No applicable studies.

*Certainty of evidence*:

The quality of the evidence cannot be assessed because of the lack of relevant studies.

*Balance of effects, acceptability, and feasibility determination*:

Both ventilation modes are included in standard ventilators, and the required costs and resources are probably low.

### CQ26: Should we perform a recruitment maneuver when ventilating an adult patient with ARDS?

#### Background

The recruitment maneuver is a low-cost intervention that can be implemented at bedside and may improve oxygenation and lung compliance, reducing the need for rescue therapies, such as veno-venous extracorporeal membrane oxygenation (VV-ECMO). Meanwhile, positive pressure ventilation at high pressure may cause circulatory failure and barotrauma.

#### Recommendation

We suggest against the routine use of the recruitment maneuver in adult patients with ARDS.

(weak recommendation/very low certainty of evidence: GRADE 2D).

#### Supplementary item

Adequate education and training are required to perform the recruitment maneuver. If the circulation is adequately monitored and critical situations, such as cardiopulmonary arrest, can be adequately addressed, a recruitment maneuver may be performed to improve the *P/F* ratio and avoid rescue therapy.

#### Rationale

*Summary of evidence*:

When conducting the recruitment maneuver, VFD was extended by an average of 0.91 days (1.56 days shorter–3.37 days longer) [191–193, 213–215], 28-day mortality decreased by 16/1000 patients (61 fewer–41 more patients) [174, 187, 190–193, 213–218], and the intensive care unit (ICU) length of stay was shortened by an average of 1.03 days (2.58 days shorter–0.53 days longer) [187, 190–193, 213–218]. From the above, the desirable effect was judged to be “trivial.” Meanwhile, with regard to harm outcomes, barotrauma occurred in 4 fewer/1000 patients (27 fewer–35 more patients) [174, 187, 190–193, 213–215, 217, 218], and circulatory failure occurred in 90 more/1000 patients (3 fewer–218 more patients) [191, 193, 219]. From the above, harms of the intervention were judged to be small. The balance of desired effects and harms was judged to be “probably favors the comparison.”

*Certainty of evidence*:

Because the outcome directions for the desirable and undesirable effects were not consistent, the certainty of the evidence for the overall outcome was judged to be “very low.”

*Balance of effects, acceptability, and feasibility determination*:

The burden and cost of the recruitment procedure is not significant, as it only requires education and training of medical staff. In addition, there is a tendency that circulatory failure including cardiac arrest increases. Implementation of the maneuver should be carefully considered.

### CQ27: Are ventilator weaning protocols useful in patients with mechanical ventilation?

#### Background

Prolonged ventilation management may increase adverse events, such as ventilator-associated pneumonia, whereas premature extubation could increase reintubation and mortality rate [220]. Therefore, it is important to recognize the timing of ventilator weaning at an early stage and in a reliable manner. Ventilator weaning is often done at the discretion of the clinician, but protocolized weaning may be effective in reducing ventilator days [220].

#### Recommendation

We suggest protocolized ventilator weaning in adult patients with ARDS (weak recommendation/very low certainty of evidence: GRADE 2D).

#### Supplementary item

It is important to educate medical staff when introducing a protocol and making new changes to ventilator settings with multidisciplinary medical staff. It is also necessary to pay close attention to patient monitoring.

#### Rationale

*Summary of evidence*:

Because there were no RCTs that included only patients with ARDS, we included studies of patients who were on mechanical ventilation for more than 24 h [221–233]. With the use of the protocol, in-hospital mortality occurred in 10 more/1000 patients (30 fewer–57 more) [222, 224–227, 229, 233], ICU mortality occurred in 47 fewer/1000 patients (80 fewer–2 more patients) [223, 225, 228, 233], duration of mechanical ventilation was shortened by an average of 29.33 h (47.98 h shorter–10.69 h shorter) [221–231, 233], hospital stay was shortened by an average of 1.19 days (2.92 days shorter–0.55 days longer) [222, 224, 227, 229, 230], ICU length of stay was shortened by an average of 17.84 h (29.67 h shorter–6.02 h shorter) [222, 223, 225, 227–231, 233], tracheostomy was performed in 34 fewer/1000 patients (58 fewer–1 fewer patients) [222, 226–229, 231], and reintubation was performed in 6 fewer/1000 patients (36 fewer–36 more patients) [221, 222, 224, 225, 227, 228, 231, 233]. Therefore, the expected desirable effect was judged to be “trivial.” Meanwhile, there were no harm outcomes, so the undesirable effect was judged to be “do not know.” From the above results, we concluded that the effects were probably favors the intervention.

*Certainty of evidence*:

The direction of the outcomes within the desirable effect was inconsistent, so the certainty of the evidence for the overall outcome was judged to be “very low.”

*Balance of effects, acceptability, and feasibility determination*:

Except for the use of special ventilatory modes, the costs of implementing the protocol are trivial. Meanwhile, creating protocols and training medical staff may take time and have associated costs.

### CQ28: Should high-frequency oscillatory ventilation (HFOV) be used for adult patients with ARDS?

#### Background

It is important to prevent VALI in adult patients with ARDS. HFOV is a mechanical ventilation mode that restricts the tidal volume and enables lung recruitment, and it is thought to be a type of lung protective ventilation. However, it is not a common mechanical ventilation method for adult patients with ARDS, and there is a need to verify its effectiveness and safety.

#### Recommendation

We suggest against implementing HFOV for adult patients with moderate or severe ARDS (weak recommendation/high certainty of evidence: GRADE 2A).

#### Supplementary item

This recommendation is for the use of HFOV in adult patients with typical moderate to severe ARDS (*P/F* ratio ≤ 200 mmHg).

#### Rationale

*Summary of evidence*:

Mechanical ventilation by HFOV affected the following items defined as important outcomes: refractory hypoxemia, 38 more/273 patients to 19 fewer/275 patients [234].Therefore, the desirable effect was judged as “trivial.” Meanwhile, the effects on harmful outcomes were as follows: short-term mortality occurred in 27 more /1000 patients (5 RCTs: *N* = 1612; 95% CI: 61 fewer–133 more patients) [234–238]; in-hospital mortality occurred in 64 more/1000 patients (2 RCTs: *N* = 1343; 95% CI: 47 fewer–215 more patients) [234, 237]; ICU length of stay was extended by an average of 1.5 days (1 RCT: *N* = 795; 95% CI: 0.71 days shorter–3.71 days longer) [237]; ventilator-free days were shortened by an average of 0.5 days (1 RCT: *N* = 795; 95% CI: 1.71 days shorter–0.71 days longer) [237]; and barotrauma occurred in 30 more/1000 patients (4 RCTs: *N* = 784; 95% CI: 15 fewer–96 more patients) [234–236, 238]. From these, the undesirable effect was judged to be “small.” From the above results, the balance of effects was judged to be that “probably favors the comparison.”

*Certainty of evidence*:

The direction of outcomes of desirable effects and undesirable effects was consistent, and the certainty of evidence for the overall outcome was judged as “high” by adopting the highest of all the outcomes.

*Values, balance of effects, acceptability, and feasibility determination*:

The use of HFOV in adult patients requires a specialized mechanical ventilator and skilled medical staff. Judging from the overall balance of equipment, human cost, and effectiveness, there would be little benefit in introducing HFOV to a facility that is not familiar with its use.

### CQ29: Should driving pressure be used as an index when implementing mechanical ventilation in adult patients with ARDS?

#### Background

Driving pressure is associated with survival in the ventilatory management of patients with ARDS [239]. Limiting the driving pressure may reduce VALI rate [240]. Meanwhile, limiting driving pressure may increase harms, such as hypercapnia and acid–base imbalance. Therefore, there is clinical significance in investigating the usefulness of setting driving pressure as an index when conducting mechanical ventilation in patients with ARDS.

#### Recommendation

We cannot provide a recommendation on whether driving pressure should be used as an index when conducting mechanical ventilation in adult patients with ARDS. There is increased importance of restricting driving pressure in ensuring standard lung protective ventilation that restricts tidal volume or plateau pressure based on ideal weight while paying attention to the harms, such as hypercapnia and acid–base imbalance (in our practice statement).

#### Rationale

*Summary of evidence*:

A total of 13 observational studies met the inclusion criteria, and there were no RCTs that met the inclusion criteria [145, 239–250]. Low driving pressure is expected to reduce VALI rate; meanwhile, there are concerns of harms, such as increased respiratory rate, atelectasis, hypercapnia, and respiratory acidosis. Low driving pressure is associated with prevention of lung overextension or hyperinflation, decrease in VALI rate, and decrease in mortality rate. However, although driving pressure is a prognostic indicator, it is unclear whether using driving pressure as a marker for ventilation settings improves outcomes for patients with ARDS. The balance between the positive and negative effects of using driving pressure as an index for management is unclear, and no clear recommendations can be made in this CQ. For this reason, this CQ is not an evidence-based recommendation, but rather an in our practice statement.

*Certainty of evidence*:

Since there are no relevant studies, the quality of evidence cannot be evaluated.

*Values, balance of effects, acceptability, and feasibility determination*:

Since there is no cost to buy additional equipment to measure driving pressure, it can be implemented with education and manpower.

### CQ30: Is low SpO_2_ (PaO_2_) a target for management in adult patients with ARDS?

#### Background

Oxygen therapy is essential in adult patients with ARDS, and SpO_2_ monitoring during treatment is essential. Recent RCTs have shown that management that targets lower SpO_2_ improves mortality, whereas some studies have shown an increase in mortality, and the effect of management targeting low SpO_2_ is inconsistent [251]. Therefore, it is necessary to verify the benefits and harms of management targeting low SpO_2_ in patients with ARDS and to define a target SpO_2_ value.

#### Recommendation

We suggest against conducting management that targets an excessively low SpO_2_ (PaO_2_) in adult patients with ARDS (weak recommendation/very low certainty of evidence: GRADE 2D).

#### Supplementary item

The optimal SpO_2_ (PaO_2_) is currently unknown. Management that avoids excessively low or high oxygen levels should be conducted.

#### Rationale

*Summary of evidence*:

There were 6 RCTs [252–257] (*N* = 2337) comparing management of adult ventilated patients with low SpO_2_ (PaO_2_) versus high SpO_2_ (PaO_2_) goals. Favorable outcomes of management targeting low SpO_2_ included long-term mortality (18 fewer/1000 patients; 95% CI: 77 fewer–51 more patients) [252–257], VFD (0.25 days longer; 95% CI: 1.76 days shorter–2.27 days longer) [252, 255, 256], and ICU-acquired weakness (ICU-AW) (51 fewer/1000 patients; 95% CI: 80 fewer–4 more patients) [252]. As a result, the desirable effect was judged to be “small.” Meanwhile, the results were as follows for harmful outcomes: new arrythmia (26 more/1000 patients; 85% CI: 7 fewer–84 more patients) [252, 253] and intestinal ischemia (21 more/1000 patients; 95% CI: 20 fewer–866 more patients) [252, 253]. As a result, the undesirable effect was judged to be “small.” Based on the above results, the balance of effects was judged to be “do not know.”

*Certainty of evidence*:

Because the direction of outcomes was inconsistent, the certainty of evidence for the overall outcome was judged as “very low” by adopting the lowest of all the outcomes.

*Values, balance of effects, acceptability, and feasibility determination*:

Intervention is change in SpO_2_ target and has no cost. Management of patients with chronic obstructive pulmonary disease and others with low SpO_2_ is common and may be feasible in patients with ARDS.

**IV. Area D: Treatment adjacent to ventilator use**

### CQ31: Should neuromuscular blockers be used at an early phase in adult patients with moderate or severe ARDS?

BackgroundThe use of neuromuscular blockers for adult patients with moderate or severe ARDS has been suggested to improve the prognosis of patients with ARDS by reducing VALI rate [258, 259]. However, there are concerns, such as delays in early mobilization, ICU-AW, and impaired quality of line (QOL).

#### Recommendation

We suggest administering neuromuscular blockers at an early phase for adult patients with moderate or severe ARDS (weak recommendation/very low certainty of evidence: GRADE 2D).

#### Supplementary item

The administration of neuromuscular blockers should be limited to early-onset patients with moderate or worse ARDS for no longer 48 h. The drug mainly used in international RCTs (cisatracurium) is not available in Japan. Alternative drugs in Japan include the aminosteroid neuromuscular blockers (rocuronium and vecuronium). Care should be taken when using aminosteroids, because the risk of ICU-AW could increase. Also, particular attention should be given to concomitant use with corticosteroids.

#### Rationale

*Summary of evidence*:

There were 5 RCTs [258–262] that were in line with PICO. As for the effect estimates, survival (5 RCTs: *N* = 1461) [258–262] had a risk difference of 84 more/1000 patients (95% CI: 39 fewer–236 more/1000 patients), QOL (EQ-5D, etc.) (1 RCT: *N* = 246) [259] had a mean of 0.02 points lower (95% CI: 0.09 less–0.05 more points), and barotrauma (4 RCTs: *N* = 1,437) [258–261] had a risk difference of 31 fewer/1000 patients (95% CI: 46 fewer–9 fewer/1000 patients). The desirable effect due to intervention was judged as “moderate”. ICU-AW (4 RCTs: *N* = 747) [258–261] had a risk difference of 25 more/1000 patients (95% CI: 58 fewer–125 more/1000 patients). The undesirable effects were judged as “small.” From the above, the balance of effects was judged as “probably favors the intervention.”

*Certainty of evidence*:

The certainty of evidence of the overall outcome used the lowest certainty of evidence and was judged as “very low.”

*Values, balance of effects, acceptability, and feasibility determination:*

Neuromuscular blockers are drugs that are easy to acquire and use, and they have minimal costs. They are already used in clinical practice and are thought to be sufficiently feasible.

### CQ32: Should transpulmonary pressure be used when setting PEEP in patients with ARDS?

#### Background

Excessive PEEP in patients with ARDS enhanced VALI and also has a negative effect on hemodynamics [189, 263]. Meanwhile, excessively low PEEP has also been reported to increase lung collapse, which may enhance VALI and worsen oxygenation [189, 263]. Nevertheless, there is no established method for setting an appropriate PEEP. Clarifying a useful method for PEEP settings is a key clinical issue.

#### Recommendation

We suggest against using transpulmonary pressure as a routine basis in PEEP settings for patients with ARDS.

(weak recommendation/moderate certainty of evidence: GRADE 2B).

#### Supplementary item

The results of this SR indicated that transpulmonary pressure-based PEEP setting is associated with decreased 28-day mortality and long-term mortality. However, because its measurement requires a specific ventilator, it is difficult to recommend this procedure for routine measures. Therefore, the above recommendation was given. This recommendation does not reject the use of transpulmonary pressure under circumstances, where the esophageal pressure could be measured.

#### Rationale

*Summary of evidence*:

The results of this SR showed that there were 2 RCTs [189, 263] that compared transpulmonary pressure-based PEEP settings and conventional ARDSnet table-based PEEP decision methods in patients with ARDS. As for the effect estimates for short-term mortality (2 RCTs: *N* = 261) [189, 263] with transpulmonary pressure-based PEEP settings, the risk difference was 85 fewer/1000 patients (95% CI: 225 fewer–247 more/1000 patients) [189, 263]; and for long-term mortality (2 RCTs: *N* = 259) [189, 263], the risk difference was 72 fewer/1000 patients (95% CI: 207 fewer–148 more/1000 patients). From the above, the desirable effect transpulmonary pressure-based PEEP setting was judged as “small.” As for harms, the frequency of pneumothorax or barotrauma did not increase with transpulmonary pressure-based PEEP settings (pneumothorax: 2/98 vs. 3/102, absolute difference of 0.9% (− 3.4–5.2%); barotrauma: 5/98 vs. 6/102, absolute difference of 0.8% (5.5–7.1%); it was also reported that there were no complications associated with esophageal pressure balloon placement [263]. Therefore, the undesirable effect was judged as “small.” From the above, the balance of effects was judged as “do not know.”

*Certainty of evidence*:

The certainty of evidence of the overall outcome adopted the lowest certainty of evidence of all outcomes and was judged as “moderate.”

*Values, balance of effects, acceptability, and feasibility determination*:

Measuring transpulmonary pressure requires a specific ventilator, so its implementation is difficult for some facilities.

### CQ33: Should electrical impedance tomography (EIT) be used in PEEP settings for patients with ARDS?

#### Background

There is no established method regarding appropriate PEEP settings in patients with ARDS. EIT, which visualizes gas distribution in the lungs by measuring impedance changes in the body, can be used to extract the ventilation status of each lung site, and PEEP could be appropriately set by searching for PEEP, where the lungs are uniformly aerated [264]. Clarifying a useful method for PEEP settings is a key clinical issue.

#### Recommendation

We cannot recommend the use of EIT in PEEP settings for patients with ARDS in our daily practice (in our practice statement).

#### Supplementary item

As of February 2021, EIT has not been released in Japan. There is no evidence that compares EIT to conventional PEEP decision methods for setting the appropriate PEEP, but there is a possibility that EIT may help to set the appropriate PEEP for uniform lung ventilation.

#### Rationale

*Summary of evidence*:

A literature research was systematically conducted, but there were no studies that compared EIT-based PEEP decisions and other methods as an important outcome. Therefore, a clear recommendation cannot be suggested for this CQ. This CQ is not an evidence-based recommendation but instead a description of the current state of clinical practice.

*Certainty of evidence*:

Since there are no applicable studies, the quality of evidence cannot be evaluated.

*Values, balance of effects, acceptability, and feasibility determination*:

As of February 2021, the EIT has not been released in Japan and is an experimental device. When it is released in the future, the cost is expected to be excessive.

### CQ34: Should pulmonary ultrasound be used for PEEP settings in patients with ARDS?

#### Background

In patients with ARDS, excessive PEEP can increase VALI and adversely affect circulatory dynamics [189, 263]. On the other hand, extremely low PEEP also increases collapsed lung, and lower PEEP also has been reported to affect VALI and worsen oxygenation [189, 263]. There is no established method for setting an appropriate PEEP. Clarifying a useful method for PEEP settings is a key clinical issue.

#### Recommendation

It is not common to use pulmonary ultrasound in the PEEP settings of patients with ARDS (in our practice statement).

#### Supplementary item

Evaluating the non-ventilated area of the lung with pulmonary ultrasound requires technique and skill, and there is also no evidence that compares this with usual PEEP setting methods. There is also the possibility of excessive PEEP levels based on the results of pulmonary ultrasound. Meanwhile, pulmonary ultrasound can help assess PEEP configuration, because it can assess collapsed lung recruitment due to elevated PEEP, is non-invasive, and does not require additional costs.

#### Rationale

*Summary of evidence*:

No applicable studies.

*Balance of effects, acceptability, feasibility determination*:

Ultrasounds are equipped in each facility that conducts clinical practice for ARDS, and it is thought that additional costs of resources are not needed. Pulmonary ultrasound itself is non-invasive and does not require additional costs, so it is easy to implement; however, it is thought that evaluating lung aeration with pulmonary ultrasound requires technique and skill, and it cannot be said that this method is unequivocally feasible.

### CQ35: Should prone positioning be used in adult patients with moderate or severe ARDS?

#### Background

Changing the position of patients with ARDS from supine to prone position is thought to be a remedy for severe hypoxemia, because it improves oxygenation. Additionally, it has been suggested that prone positioning has the effects of preventing the onset or progression of VALI, and it has the possibility of improving patient prognosis [265]. The prone positioning itself does not require special equipment, but complications could occur by changing the body repositioning and long-term management [265]. Given these contexts, clarifying the benefits and harms of the prone positioning in patients with ARDS is a key clinical issue.

#### Recommendation

We suggest performing the prone positioning in adult patients with moderate or severe ARDS for long periods of time (weak recommendation/very low certainty of evidence: GRADE 2D).

#### Supplementary item

Prone positioning needs to be done in hospitals with experienced staffs. Additionally, long implementation times (over 12 h) should be considered when placing patients in the prone position.

#### Rationale

*Summary of evidence*:

The results of SR for beneficial outcomes showed that the effect estimates for short-term mortality (7 RCTs: *N* = 2118) [265–271] was 69 fewer/1000 patients in the prone position when compared to patients not in the prone position (95% CI: 135 fewer–13 more/1000 patients), mechanical ventilation duration (3 RCTs: *N* = 871) [267, 268, 270] was shortened by an average of 0.48 days (95% CI: 1.61 days shorter–0.65 days longer), and ICU length of stay (3 RCTs: *N* = 518) [269–271] was extended by an average of 1.03 days (95% CI: 1.7 days shorter–3.75 days longer), and the desirable effect due to intervention was judged to be “moderate.” Meanwhile, as for harmful outcomes, the effect estimates for tracheal tube problems (2 RCTs: *N* = 808) [265, 271] was 83 more/1000 patients in the prone position when compared to patients not in the prone position (95% CI: 30 more–151 more/1000 patients), and the number of pressure-induced skin disorders and ulcers (2 RCTs: *N* = 344) [266, 268] increased by 114/1000 patients (95% CI: 19 more–237 more/1000 patients), and the predicted undesirable effect was judged to be “small.” From the above, the balance of effects was judged as “Probably favors the intervention.”

*Certainty of evidence*:

Because the direction of desirable and undesirable effects is not consistent, the certainty of evidence for the overall outcome was judged as “very low,” which was the lowest of all the outcomes.

*Balance of effects, acceptability, and feasibility determination*:

Repositioning the patient to the prone position requires more manpower than usual, but there is no need to purchase additional expensive equipment, so cost is minimal. Meanwhile, there are concerns of the onset of tracheal tube displacement or dislocation and skin damage due to the prone position, but its harms are trivial. Although there is no evidence in Japan, this is already implemented in clinical practice. Even when considering costs, it is thought to be feasible if conducted in a facility, where there are well-trained staff in ARDS management.

### CQ36: Should ECMO be conducted in adult patients with severe ARDS?

#### Background

Introducing ECMO to patients with severe ARDS is expected to reduce the mortality rate through rescue effects for severe respiratory failure, where maintaining oxygenation is difficult with normal mechanical ventilation [272, 273]. ECMO may also minimize VALI. However, its effectiveness has not been established due to the uncertainty of indications and timing as well as the presence of many complications. Clarifying the relationship between ECMO introduction and mortality as well as adverse events are key clinical issues.

#### Recommendation

We suggest implementing ECMO for adult patients with severe ARDS (weak recommendation/moderate certainty of evidence: GRADE 2B).

#### Supplementary item

It is desirable to consult an experienced facility or specialist regarding ECMO indications and patient transport in cases of severe hypoxemia or hypercapnia that is resistant to standard lung protective ventilation or adjuvant therapies, such as muscle relaxants and prone positioning.

#### Rationale

*Summary of evidence*:

There were 2 RCTs [272, 273] that comparatively investigated whether ECMO or standard treatment with conventional mechanical ventilation should be conducted for severe patients with ARDS. For beneficial outcomes, the effect estimates for 60-day mortality (2 RCTs: *N* = 429) [272, 273] was 133 fewer/1000 patients with ECMO implementation (95% CI: 204 fewer–43 fewer /1000 patients), and the desirable effect due to intervention was judged as “moderate.” Meanwhile, for harmful outcomes, the effect estimates for stroke (1 RCTs: *N* = 249) [272] was 40 fewer/1000 patients (95% CI: 58 fewer–25 more/1000 patients). However, considering the possibility that there were 25 more patients/1000 patients due to intervention, the undesirable effect was judged as “small.” As an “important” outcome, bleeding-related complications tended to increase. Therefore, the balance of effects was judged as “probably favors the intervention.”

*Certainty of evidence*:

Because the direction of desirable and undesirable effects is not consistent, the certainty of evidence for the overall outcome was judged as “moderate,” which was the highest of all the outcomes.

*Balance of effects, acceptability, and feasibility determination*:

There are cost concerns with ECMO implementation. The introduction and management of ECMO requires staff from multiple disciplines with specialized knowledge and skill, and human resource must also be considered. In the Western countries, when a patient indicated for ECMO was present in a facility, where ECMO cannot be conducted, the patient is transferred to an ECMO center, thereby centralizing ECMO procedures. Attempts are also made in Japan toward this centralization of ECMO treatment.

### CQ37: Should early tracheostomy be performed in adult patients with ARDS?

#### Background

Tracheostomy has the advantage of reducing the administration of sedatives or analgesics as well as avoiding vocal cord injury when compared to tracheal intubation [274, 275]. However, tracheostomy may be associated with bleeding or removal difficulties due to airway narrowing. It has also been reported that early tracheostomy within 14 days after intubation may improve prognosis [276]. Therefore, clarifying whether early tracheostomy improves patient prognosis in patients with ARDS, where the length of mechanical ventilation can be extended to a long period of time is a key clinical issue.

#### Recommendation

We suggest performing early tracheostomy in adult patients with ARDS (weak recommendation/very low certainty of evidence: GRADE 2D).

#### Supplementary item

There is no clear definition of early tracheostomy, with many studies defining this from within 48 h to within 10 days after the initiation of mechanical ventilation, and early tracheostomy could be considered if the general condition of the patient is stable. Meanwhile, there are concerns with early tracheostomy that it may be conducted on patients who did not require it in the first place. Additionally, the tracheostomy itself may involve high levels of risk in patients with ARDS with high oxygen concentrations, airway pressure, or PEEP; therefore, careful consideration is needed for adaptation to the patient in this recommendation.

#### Rationale

*Summary of evidence*:

As for beneficial outcomes, the effect estimates for short-term mortality (14 RCTs: *N* = 2887) [277–290] was 37 fewer/1000 patients with early tracheostomy compared to patients without early tracheostomy (95% CI: 70 fewer–0 fewer/1000 patients), ventilator-associated pneumonia (VAP) (8 RCTs: *N* = 1527) [280, 282–285, 287–289] was 67 fewer/1000 patients (95% CI: 120 fewer–5 more/1000 patients), and VFD (4 RCTs: *N* = 1243) [282, 283, 285, 287] was extended by an average of 1.2 days (95% CI: 0.57 days longer–1.82 days longer). From the above, the desirable effect due to intervention was judged as “small.” Meanwhile, for the harmful outcomes, the effect estimates associated with tracheostomy-related bleeding (5 RCTs: *N* = 1715) [279, 282, 283, 285, 286] was 7 more/1000 patients with early tracheostomy compared to patients without early tracheostomy (95% CI: 5 fewer–30 more/1000 patients), and tracheostomy infection (4 RCTs: *N* = 816) [279, 282, 283, 285] was 12 more/1000 patients (95% CI: 6 fewer–54 more/1000 patients), and the predicted harm was judged as “trivial.” Therefore, the balance of effects and harms was judged as “probably favors the intervention.”

*Certainty of evidence*:

Because the direction of desirable and undesirable effects is not consistent, the certainty of evidence for the overall outcome was judged as “very low,” which was the lowest of all the outcomes.

*Balance of effects, acceptability, and feasibility determination*:

Tracheostomy has relatively low costs, is a procedure within the scope of usual clinical practice, and does not require the purchase of additional equipment or the additional human resources, so the adverse effects due to intervention are thought to be small. Meanwhile, early tracheostomy may result in unnecessary tracheostomies being conducted.

### CQ38: Should a VAP prevention bundle be routinely conducted for adult patients with ARDS?

#### Background

VAP is pneumonia that newly occurs 48 h after the initiation of mechanical ventilation and onwards, and it also includes pneumonia that occurred within 48 h following patient withdrawal from a ventilator [291]. VAP is one of the most important complications in mechanically ventilated patients, and it is important to implement appropriate preventive measures for VAP. The VAP prevention bundle, which involves conducting a series of care measures as a set, has been proposed as a preventive method for VAP, but its effects and certainty of evidence are unclear.

#### Recommendation

We recommend conducting a VAP prevention bundle routinely in adult patients with ARDS on a ventilator (GPS).

#### Supplementary item

Bundle contents should be considered in each facility.

#### Rationale

*Summary of evidence*:

The predicted desirable effect due to VAP prevention bundle implementation was judged as moderate. We were not able to find any RCTs that directly compared whether to perform VAP prevention bundles for adult patients with ARDS requiring mechanical ventilation. However, multiple observational studies reported that the occurrence of VAP was reduced through the implementation of VAP prevention bundles, and the balance of effects was judged as “probably favors the intervention.”

*Balance of effects, acceptability, and feasibility determination*:

Additionally, the balance of desirable and undesirable effects was judged as “probably favors the intervention.” The VAP prevention bundle has been taken up in the joint action on medical safety in Japan and has spread nationwide, so it would be accepted by major stakeholders. There are many facilities implementing VAP prevention bundles as part of daily clinical practice, and it was judged to be feasible.

**V. Area E: Drug therapy/non-drug therapy**

### CQ39: Should thrombomodulin be used in patients with ARDS?

#### Background

It has been reported that thrombomodulin, which is used for disseminated intravascular coagulation treatment, may have anti-inflammatory effects as well as anticoagulant effects [292–294]. Therefore, thrombomodulin may be effective in suppressing inflammation in ARDS.

#### Recommendation

We cannot provide a recommendation for the administration of thrombomodulin in patients with ARDS. (in our practice statement).

#### Rationale

*Summary of evidence*:

We conducted an SR, but found no RCTs to assess the effect of thrombomodulin in only patients with ARDS.

*Certainty of evidence*:

Since there are no applicable studies, the quality of evidence cannot be evaluated.

*Balance of effects, acceptability, and feasibility determination*:

Since there are no applicable studies, the balance of effects is unknown. Considering the lack of evidence and its off-label use, thrombomodulin use in patients with ARDS is likely unacceptable. Additionally, ethics committee approval will be needed when administering thrombomodulin for ARDS, since this such use will not be covered by the national health insurance. Therefore, an unequivocal statement cannot be made regarding feasibility. The cost of 6 days of thrombomodulin administration is approximately $2100 (¥240,000). There is a possibility of increased burdens on patients, hospitals, and payment institutions.

### CQ40: Should nitric oxide inhalation be used for patients with ARDS?

#### Background

It has been reported that nitric oxide (NO) inhalation, which has a pulmonary vessel dilatation effect, was effective in improving oxygenation in ARDS; meanwhile, it has also been reported that it did not improve the length of mechanical ventilation or mortality [295]. NO is sometimes used as a rescue therapy when oxygenation does not improve [296]; therefore, conducting an SR regarding NO inhalation is a key clinical issue.

#### Recommendation

We suggest against using NO inhalation in patients with ARDS (weak recommendation/very low certainty of evidence: GRADE 2D).

#### Supplementary item

This recommendation does not reject the use of NO inhalation as rescue therapy in facilities that already use this treatment.

#### Rationale

*Summary of evidence*:

As a result of implementation of NO inhalation, the length of mechanical ventilation (2 RCTs: *N* = 77) [297, 298] shortened by an average of 1.77 days (95% CI: 3.55 days shorter–0.02 days longer) and ICU length of stay (1 RCT: *N* = 40) [297] shortened by an average of 0.7 days (95% CI: 38.28 days shorter–36.88 days longer). Therefore, it was thought that there were minimal predicted desirable effects (judged as “trivial” benefit).

Meanwhile, as a result of implementation of NO inhalation, mortality (7 RCTs: *N* = 878) [296–302] occurred in 40 more/1000 patients (95% CI: 16 fewer–109 more patients) and renal dysfunction (1 RCT: *N* = 180) [300] in 132 more/1000 patients (95% CI: 10 more–373 more patients). As a result, the predicted undesirable effect was judged as “moderate.”

*Certainty of evidence*:

Although NO inhalation increased the harms (mortality and renal dysfunction), the length of mechanical ventilation and ICU length of stay were shortened. Because the direction of desirable and undesirable effects is not consistent, the certainty of evidence was judged as “very low” that was the lowest among all outcomes.

*Balance of effects, acceptability, and feasibility determination*:

Given that mortality increased when NO was used, the harms were judged to outweigh the benefits. Given the need for ethics committee approval and specialized equipment, it is predicted that there are a limited number of medical institutions, where this is feasible.

### CQ41: Should sivelestat be used for patients with ARDS?

#### Background.

The pathogenesis of ARDS is a permeability pulmonary edema due to non-specific inflammation, where neutrophil elastase is involved as an important mediator [303, 304]. It is an important clinical issue to test whether sivelestat, a neutrophil elastase inhibitor, is effective in ARDS.

#### Recommendation

We suggest against using sivelestat in patients with ARDS.

(weak recommendation/very low certainty of evidence: GRADE 2D).

#### Rationale

*Summary of evidence*:

As a result of sivelestat use, mortality (4 RCTs [305–308]: *N* = 557) occurred in 5 fewer/1000 patients (95% CI: 67 fewer–78 more patients), length of mechanical ventilation (2 RCTs [307, 309]: *N* = 38) was shortened by an average of 4.7 days (95% CI: 13.11 days shorter–3.64 days shorter), and the ICU length of stay (1 RCT [305]: *N* = 24) was shortened by an average of 5 days (95% CI: 21.26 days shorter–11.26 days shorter). The predicted desirable effect of sivelestat was judged as “small,” and the harms were also thought to be “small.”

*Certainty of evidence*:

There was no effect of the intervention on mortality, whereas there was a beneficial effect of the intervention on the lengths of mechanical ventilation and ICU stay. Therefore, the direction of desirable and undesirable effects is not consistent, and the certainty of evidence was judged as “very low.”

*Balance of effects, acceptability, and feasibility determination*:

The balance of effects of intervention was judged as “do not know.” The benefits of the intervention were not clear, whereas the costs could increase. Thus, the intervention was judged to be likely unacceptable. The drug is readily accessible, and the feasibility of the intervention was set as high.

### CQ42: Should corticosteroids be used for adult patients with ARDS?

#### Background

The pathogenesis of ARDS is a permeability pulmonary edema due to non-specific inflammation [310], and whether anti-inflammatory corticosteroids improve clinical outcomes is an important clinical question.

#### Recommendation

We suggest against using high-dose corticosteroids for adult patients with ARDS.

(weak recommendation/very low certainty of evidence: GRADE 2C).

We recommend using low-dose corticosteroids for adult patients with ARDS.

(strong recommendation/moderate certainty of evidence: GRADE 1B).

#### Supplementary item

The types and dose of steroids for ARDS vary from study to study. The high dose is approximately 30 mg/kg in terms of methylprednisolone, and the low dose is approximately 1–2 mg/kg. Sub-group analyses and previous meta-analyses suggested the usefulness of starting low-dose steroid administration at an early stage and continuing this for at least 7 days [311].

#### Rationale

*Summary of evidence*:

High-dose corticosteroids

For desirable effects, long-term mortality (1 RCT [312]: *N* = 99) decreased by 32/1000 patients (95% CI: 196 fewer–183 more patients), so this was judged as “trivial.” For undesirable effects, infection (1 RCT [312]: *N* = 99) occurred in 58 more/1000 patients (95% CI: 46 fewer–353 more patients), so this was judged as “small.”

Low-dose corticosteroids

For desirable effects, long-term mortality (5 RCTs [313–317]: *N* = 769) decreased by 105/1000 patients (95% CI: 178 fewer–7 fewer patients), infection (5 RCTs [313–317]: *N* = 769) occurred in 50 fewer/1000 patients (95% CI: 97 fewer–6 more patients), and VFD (5 RCTs [313–317]: *N* = 767) increased by an average of 4.75 days (95% CI: 2.97 days longer–6.54 days longer), hospital length of stay (2 RCTs [314, 315]: *N* = 271) shortened by an average of 5.04 days (95% CI: 9.43 days shorter–0.65 days shorter), and ICU length of stay (2 RCTs [314, 315]: *N* = 271) shortened by an average of 5.23 days (95% CI: 9.64 days shorter–0.82 days shorter), so this was judged as “moderate.” No undesirable effects were observed at low doses. Therefore, when considered together with the description in the additional considerations, it was judged to be “trivial.”

*Certainty of evidence*:

High-dose corticosteroids

Because the direction of desirable effects and undesirable effects was not consistent, the certainty of evidence was judged as “very low,” which was the lowest of all the outcomes.

Low-dose corticosteroids

As the direction of desirable effects and undesirable effects was consistent, the certainty of evidence was judged as “moderate,” which was the highest of all the outcomes.

*Balance of effects, acceptability, and feasibility determination*:

High-dose steroid administration can be harmful, and is, therefore, thought to be unacceptable. Effects of the intervention such as decreased mortality, VFD extension, shortened ICU length of stay, and shortened in-hospital stay could be expected for low-dose steroid administration, and it was judged as economically acceptable. Steroid administration is covered by insurance for septicemia and severe pneumonia, which are the major causes of ARDS, and its feasibility was judged to be high.

### CQ43: Should early rehabilitation intervention be conducted for adult patients with ARDS?

#### Background

Post-intensive care syndrome (PICS) is more likely to be a problem for patients with severe illnesses, such as ARDS [318]. Early rehabilitation is provided during ICU stay to prevent PICS. It has been reported that early rehabilitation improved physical function in severely ill patients and mechanically ventilated patients [319, 320]. Therefore, whether early rehabilitation intervention should be conducted for patients with ARDS is an important clinical question.

#### Recommendation

We suggest conducting early (i.e., within 72 h) rehabilitation for adult patients with ARDS (weak recommendation/low certainty of evidence: GRADE 2C).

#### Rationale

*Summary of evidence*:

With implementation of early rehabilitation, mortality (6 RCTs [321–326]: *N* = 883) decreased by 2/1000 patients (95% CI: 46 fewer–57 more patients), ICU-AW (2 RCTs [326, 327]; *N* = 211) occurred in 238 fewer/1000 patients (95% CI: 325 fewer–114 fewer patients), and length of mechanical ventilation (6 RCTs [321–323, 325–327]; *N* = 652) was shortened by an average of 2.39 days (95% CI: 3.73 days shorter–1.06 days shorter). Additionally, the number of individuals experiencing adverse events that were thought to be undesirable effects (2 RCTs [323, 324]; *N* = 612) decreased by 20/1000 patients (95% CI: 42 fewer–23 more patients). From the above, the predicted desirable effect was judged as “moderate.”

Meanwhile, harms involved in conducting early rehabilitation included one case each of unplanned tracheal tube extubation, accidental removal of the arterial catheter, and allergy due to electrical stimulation pad reported in RCTs in the intervention group. Therefore, the undesirable effect of early rehabilitation was judged as “trivial.”

*Certainty of evidence*:

The certainty of evidence was “low” as for all outcomes.

*Values, balance of effects, acceptability, and feasibility determination*:

Feasibility is thought to be high in facilities that are familiar with the implementation of early rehabilitation; facilities that have a physiotherapist, occupational therapist, and speech therapist in charge of intensive care; and environments with sufficient manpower.

### CQ44: Should non-sedative or light-sedative management be conducted for adult patients with ARDS?

#### Background

Sufficient sedation may sometimes be necessary for the management of mechanical ventilation, but high-dose sedatives may increase the duration of mechanical ventilation and the risk of delirium. Minimal to no sedation is expected to have a beneficial effect or avoid complications.

#### Recommendation

We suggest conducting no sedation or minimal sedation management for adult patients with ARDS (weak recommendation/very low certainty of evidence: GRADE 2D).

#### Supplementary item

In contrast to deep sedation, where subjects do not respond to stimuli, light sedation refers to sedation, where subjects could be awakened by physical or verbal stimuli. This recommendation is based on the results of clinical studies excluding patients requiring deep sedation and should be noted during implementation. Because there were also no studies focused only on patients with ARDS, care must be taken in indications for the interventions.

#### Rationale

*Summary of evidence*:

No studies were identified that included only patients with ARDS. As a result of non-sedative/light-sedative management, the number of patients who survived and got discharged (2 RCTs [328, 329]; *N* = 242) increased by 28/1000 patients (90 fewer–172 more patients), and ventilator-free days (2 RCTs [329, 330]; *N* = 1287) was extended by an average of 2 days (0.91 days shorter–4.88 days longer). From the above, the predicted desirable effect was judged as “small.”

Meanwhile, as a result of non-sedative/light-sedative management, the fraction of those undergoing tracheostomy (3 RCTs [328–330]: *N* = 1416) decreased by 4/1000 patients (24 fewer–25 more patients), and accidental extubation (3 RCTs [328–330]; *N* = 1416) increased by 33/1000 patients (2 more–76 more patients). From the above, the predicted undesirable effect was judged as “small.”

*Certainty of evidence*:

Because the direction of desirable and undesirable effects is not consistent, the certainty of evidence was judged as “very low,” which was the lowest of all the outcomes.

*Values, balance of effects, acceptability, and feasibility determination*:

When considering in terms of point estimates, the direction of predicted desirable effect of this intervention was almost consistent, and the direction can be changed when the upper and lower limits of the confidence interval were considered. From the above, the balance of benefits and harms was judged as “do not konw.” However, the number of patients who survived and got discharged increased by up to 172/1000 patients, and costs may also be reduced. Therefore, it was judged that the effects of intervention likely outweighed the harms.

### CQ45: Should restrictive fluid management strategies be implemented for adult patients with ARDS?

#### Background

Pulmonary edemas caused by vascular endothelial damage and vascular hyperpermeability are problems in ARDS, but the direct cause of death in ARDS is not hypoxemia but non-lung organ failure [331]. In other words, there is room for debate on whether restrictive fluid management, which can lead to organ perfusion disorders, should be used in fluid management in ARDS with the aim of improving lung oxygenation.

#### Recommendation

We suggest performing the restrictive fluid management strategies for adult patients with ARDS (weak recommendation/moderate certainty of evidence: GRADE 2B).

#### Supplementary item

There are several methods of restrictive fluid management, such as reduced infusion and use of diuretics, and an appropriate decision should be made according to the patient’s condition.

#### Rationale

*Summary of evidence*:

As a result of restrictive fluid management, mortality (6 RCTs [332–337]: *N* = 1214) decreased by 28/1000 patients (95% CI: 74 fewer–23 more patients), and VFD (1 RCT [337]: *N* = 1000) was extended by an average of 2.5 days (95% CI: 1.12 days longer–3.88 days longer). The number of days where hemodynamic events assumed to be harmful did not occur (1 RCT [337]: *N* = 1000) was shortened by an average of 0.3 days (95% CI: 0.57 days shorter–0.03 days shorter) as a result of restrictive infusion management, so the predicted desirable effect was judged to be “small.”

Meanwhile, as a result of restrictive fluid management, the proportion of late-onset cognitive dysfunction, which is an important outcome, occurred in 117 more/1000 patients (1 RCT [338]; certainty of evidence, “low”). Therefore, the predicted undesirable effect was judged to be “trivial.”

*Certainty of evidence*:

Because the direction of desirable and undesirable effects is the same, the certainty of evidence was judged to be “moderate,” which was the highest of all the outcomes.

*Values, balance of effects, acceptability, and feasibility determination*:

The predicted desirable effect of this intervention was judged to outweigh the harms. Since the possibility that the judgment may differ depending on the restrictive fluid management method, it was judged that this would likely be accepted.

### CQ46: Should enteral nutrition with high ω3 fatty acid content be given to patients with ARDS?

#### Background

Experiments with sepsis and septic acute lung injury model animals showed that the administration of fats with high ω3 fatty acid content could suppress pulmonary vascular permeability, pulmonary edema, and pulmonary hypertension [339]. Clarifying the effectiveness of giving enteral nutrition with high ω3 fatty acid content, such as fish oil is an important clinical issue.

#### Recommendation

We suggest giving enteral nutrition with high ω3 fatty acid content to patients with ARDS (weak recommendation/very low certainty of evidence: GRADE 2D).

#### Supplementary item

Enteral nutritional supplements with high ω3 fatty acid content have been withdrawn from the market in Japan, and providing enteral nutrition with high ω3 fatty acid content requires the addition of ω3 fatty acid preparations to normal enteral nutrition.

#### Rationale

*Summary of evidence*:

As a result of using ω3 fatty acid preparations, mortality (7 RCTs [340–342]: *N* = 672) decreased by 42/1000 patients (95% CI: 117 fewer–77 more patients), and the *P/F* ratio (3 RCTs [343–345]: *N* = 113) increased by 11.84 (95% CI: 31.2 lower–54.88 higher). From this, the predicted desirable effect was judged to be “moderate.”

Meanwhile, as a result of using ω3 fatty acid preparations, gastrointestinal intolerance (1 RCT [344]: *N* = 272) occurred in 77 more/1000 patients (95% CI: 21 fewer–228 more patients). From this, the predicted undesirable effect was judged to be “small.”

*Certainty of evidence*:

Because the direction of desirable and undesirable effects is not consistent, the certainty of evidence was judged as “very low,” which was the lowest of all the outcomes.

*Values, balance of effects, acceptability, and feasibility determination*:

Regarding values, the predicted desirable effect of this intervention is likely significant compared with the undesirable effect when considering not only the point estimates but also the upper and lower limits of the confidence interval. Regarding acceptability, benefits from the intervention outweigh the harms, and there is the possibility of cost reduction; however, the time and effort needed for intervention would increase, so it cannot be unequivocally stated one way or another. Regarding feasibility, enteral nutrition itself is a common treatment, and common enteral preparations are easily available, but there are limits to hospitals that adopt ω3 fatty acid preparations, so it was judged that feasibility cannot be unequivocally stated one way or another.

**VI. Children**

As in adults, the mechanism of ARDS onset in children is also pulmonary edema due to hyperpermeability of pulmonary capillaries. However, its causes, background diseases, and respiratory characteristics (physiological, anatomical, immunological) are different from those of adults [346, 347]. Therefore, conceptualizing pediatric ARDS (PARDS) separately from adult ARDS is justified. A total of 15 CQs were selected in this area, leading to 3 weak recommendations for and 6 weak recommendations against interventions, and 6 in our practice statements (Table 3). Particular attention must be paid to the following 2 points.

1. Definition

**Table 3:** Comparison of pediatric recommendation to adult one

CQ number		Question	Direction of recommendation	
PCQ	Adult		Pediatric	Adult
1	14	Should non-invasive respiratory support (NPPV/HFNC) be used for pediatric patients with ARDS?	− 1	+1
2	19	Should tidal volume be restricted in pediatric patients with ARDS?	0	+2
3	20	Should high PEEP be used in pediatric patients with moderate to severe ARDS?	0	+1
4	21	Should plateau pressure be restricted in pediatric patients with ARDS?	0	+1
5	27	Should a protocol be used when liberating pediatric patients with acute respiratory failure from mechanical ventilator?	+1	+1
6	28	Should HFOV be used for pediatric patients with moderate to severe ARDS?	− 1	− 1
7	23	Should APRV be used for pediatric patients with ARDS?	− 1	0
8	30	How should target SpO2 values be set in pediatric patients with ARDS?(Adult: Is low SpO2 (PaO2) a target for management in adult patients with ARDS? )	0	− 1
9	31	Should muscle relaxants be used at an early stage in pediatric patients with moderate or severe ARDS?	0	+1
10	35	Should pediatric patients with moderate to severe ARDS be placed in the prone position?	+1	+1
11	40	Should nitric oxide inhalation therapy be used in pediatric patients with ARDS?	− 1	− 1
12	N/A	Should surfactant be used in pediatric patients with ARDS?	− 1	N/A
13	42	Should steroids be used in pediatric patients with ARDS?	0	high dose: − 1low dose: +2
14	N/A	Should a protocol be used for the sedation of pediatric respiratory failure patients?	+1	N/A
15	44	Should daily sedation interruption (DSI) be implemented for pediatric respiratory failure patients?(Adult: Should non-sedative or light-sedative management be conducted for adult patients with ARDS?)	− 1	+1

+2, strong recommendation for using an intervention; +1, weak recommendation for using an intervention; 0, no recommendation; − 1, weak recommendation against using an intervention; *N/A* not applicable, *CQ* clinical question, *PCQ* pediatric clinical question

The Pediatric Acute Lung Injury Consensus Conference (PALICC) definition were proposed in 2015 as a definition of PARDS [348]. The frequency of PARDS according to the PALICC definition was reported as 6.1% of mechanically ventilated patients in the pediatric intensive care unit (PICU), and the mortality rate was reported as 17.1% [349]. Important differences between the PALICC definition and the Berlin definition [350] include the following: SpO2 could be used in diagnosis; oxygenation index, in preference to the P/F ratio, is the primary metric of lung disease severity to define PARDS for patients treated with invasive mechanical ventilation; infiltration shadows on chest imaging do not have to be bilateral; patient groups who may develop ARDS are defined as at risk, etc. There may be insufficient evidence to conclude whether the PALICC or the Berlin definition is more useful for the decision-making of therapeutic interventions in PARDS. PaO2 measurements are not easy in children, so SpO2 could be used instead. It is known that some PARDS diagnosed by the PALICC definition could be overlooked by the Berlin definition, suggesting the superiority of the PALICC definition [348, 349]. Meanwhile, PALICC definition does not have a long history of use, and many studies have been using the Berlin definition. In the extraction of articles for the systematic review in this guideline, we included PARDS diagnosed by any one of the PALICC definition, Berlin definition, and American–European Consensus Conference Criteria.

2. Patients

- Age

There is no definite threshold for the age to distinguish children and adults. PALICC also does not define an upper or lower age limit. Meanwhile, respiratory disorders in newborns due to perinatal abnormalities such as respiratory distress syndrome, meconium aspiration syndrome, respiratory disorders secondary to congenital malformations (e.g., congenital diaphragmatic hernia) are excluded. Like in the PALICC definition, the upper and lower age limits for children were not set in this guideline; when a study included children based on the definition for age in each study, it was included, whereas children with lung injuries associated with congenital lung disease and perinatal abnormalities were excluded.

b) Pathology

Although this is an ARDS guideline, the scope of patients (= P) was expanded according to the contents of CQs. We decided to extract as many articles as possible that contained information on PARDS at the literature search stage. In definitions other than PALICC, ARDS cannot be diagnosed if invasive positive pressure ventilation is not conducted; therefore, for pediatric CQ (PCQ) 1, all patients with acute respiratory failure were targeted, but articles only including bronchiolitis were excluded. For PCQ5, PCQ14, and PCQ15, as there were few differences between ARDS and non-ARDS patients, all mechanically ventilated children were included. For the other PCQs, articles in which at least half of the causes of acute respiratory failure were ARDS, were included.

### PCQ1: Should non-invasive respiratory support (NPPV/HFNC) be used for pediatric patients with ARDS?

#### Background

Non-invasive respiratory support such as NPPV or HFNC for patients with ARDS is expected to avoid complications due to tracheal intubation. Meanwhile, there are concerns that the application of non-invasive respiratory support could delay tracheal intubation and, if applied to patients with severe ARDS, could increase mortality rate. Furthermore, there are tolerability issues in children, so non-invasive respiratory support is not an established treatment in the respiratory management of patients with ARDS, and clarifying its effectiveness is a key clinical issue.

*Note*: The target population in the SR of this CQ was not limited to just patients with ARDS and included all pediatric cases with acute respiratory failure, but populations that targeted only acute bronchiolitis were excluded.

#### Recommendation

We suggest against using non-invasive respiratory support (NPPV/HFNC) in pediatric patients with ARDS.

(weak recommendation/ very low certainty of evidence: GRADE 2D).

#### Supplementary item

This recommendation does not reject the use of non-invasive respiratory support in the early stage of pediatric acute respiratory failure in cases other than ARDS.

#### Rationale

*Summary of evidence*:

As for beneficial outcomes, the tracheal intubation rate (4 RCTs [351–354]; *N* = 276) was lower by 46/1000 patients (95% CI: 150 fewer to 227 more patients). The length of hospital stay (2 RCTs [353, 354]; *N* = 92) was shortened by an average of 0.25 days (95% CI: 3.86 days shorter to 3.37 days longer). The predicted desirable effect was judged as “trivial.” As for harmful outcomes due to intervention, the mortality rate (4 RCTs [351–353, 355]; *N* = 870) increased by 65/1000 patients (95% CI: 61 fewer to 545 more patients), and ventilator-free days (1 RCT [353]; *N* = 42) was shortened by an average of 6 days (95% CI: 13.37 days shorter to 1.37 days longer). Pressure ulcers occurred in 11.6% of the intervention group and was not observed in the non-intervention group. The predicted undesirable effect was judged to be “small.” Considering the importance of clinical intubation rate and mortality rate, the balance of effects was judged as “probably favors the comparison.”

*Certainty of evidence*:

The direction of desirable and undesirable effects were inconsistent, so the certainty of evidence was judged to be “very low.”

*Balance of effects, acceptability, and feasibility determination*:

When focusing on the possibility that mortality rate increases, undesirable effects outweigh desirable effects. Therefore, the balance of effects was set as “probably favors the comparison.” Acceptability was set as “varies” due to the possibility that interface wearing or positive pressure may not be accepted in children. Feasibility in pediatric intensive care units is high, but specialized knowledge is also necessary, and this was set as “varies.”

### PCQ2: Should tidal volume be restricted in pediatric patients with ARDS?

#### Background

Mechanical ventilation could be used in patients with severe ARDS to buy time as a rescue therapy for ARDS, but the mortality rate remains high. It has also been suggested in several studies that mechanical ventilation itself could be the cause of lung injury [174, 176]. Under such circumstances, tidal volume restriction could be a part of lung protective strategies for reducing adverse events due to mechanical ventilation. Whether to restrict tidal volume during mechanical ventilation for pediatric patients with ARDS is a key clinical issue.

#### Recommendation

We cannot provide a recommendation regarding tidal volume restrictions for pediatric patients with ARDS. It is generally accepted not to exceed tidal volume in accordance with the treatment strategy of adult patients with ARDS (in our practice statement).

#### Supplementary item

Currently, it is unclear whether to use standard weight or actual weight for the tidal volume per body weight. There is a reference physiological tidal volume value of < 8 mL/kg [356].

#### Rationale

*Summary of evidence*:

No applicable studies.

*Certainty of evidence*:

There are no applicable studies, so a description of the certainty of evidence cannot be provided.

*Balance of effects, acceptability, and feasibility determination*:

Strain is considered to be an important factor in VALI [357]. Restricting tidal volume so as not to overextend the alveoli is thought to reduce strain and alleviate lung injury [357]. Meanwhile, restricting tidal volume is associated with harmful effects, such as hypercapnia. However, there are no studies that directly evaluated these aspects, and it is not possible to determine the balance of effects. Restricting tidal volume is possible by changing the settings of the ventilator, and it is thought to be sufficiently acceptable and feasible.

### PCQ3: Should high PEEP be used in pediatric patients with moderate to severe ARDS?

#### Background

Mechanical ventilation is often needed in patients with ARDS, but ventilator use can induce VALI [174]. Therefore, the mechanical ventilation of these patients should be provided based on lung protective strategies. Application of high-level PEEP is a strategy that aims to reduce VALI [358]. Meanwhile, high PEEP is thought to have adverse effects on hemodynamics. Whether to use high PEEP for mechanical ventilation in pediatric patients with moderate to severe ARDS is a key clinical issue.

#### Recommendation

We could not provide a recommendation on whether to use high PEEP in pediatric patients with moderate to severe ARDS, but high PEEP is commonly used based on clinician’s experience and findings from adult patients with ARDS (in our practice statement).

#### Supplementary item

Care should be taken to avoid excessive plateau pressure when using high PEEP (especially > 10 cmH_2_O). Additionally, careful monitoring of adverse events, such as hemodynamic instability is needed. The effects of PEEP differ according to lung pathophysiology, and high PEEP does not necessarily improve oxygenation or lung injury.

#### Rationale

*Summary of evidence*:

No applicable studies.

*Certainty of evidence*:

There are no applicable studies, so the certainty of evidence could not be evaluated.

*Balance of effects, acceptability, and feasibility determination*:

There are no applicable studies, and the balance of effects could not be determined. PEEP can be controlled by changing the ventilator settings, which is considered acceptable or feasible.

### PCQ4: Should plateau pressure be restricted in pediatric patients with ARDS?

#### Background

There are concerns that mechanical ventilation in adult patients with ARDS could lead not only to extended ventilator use time due to VALI but also increased mortality rate. Increased airway pressure is thought to be one of the factors that cause VALI, and it is expected that VALI is suppressed by restricting plateau pressure [359]. Meanwhile, restricting plateau pressure may induce adverse events, such as hypercapnia. Whether to restrict plateau pressure in the mechanical ventilation of pediatric patients with ARDS is a key clinical issue.

#### Recommendation

We cannot provide a recommendation regarding plateau pressure restrictions for pediatric patients with ARDS, but respiratory management with restrictions on plateau pressure is implemented in accordance with the treatment strategy of adult patients with ARDS (in our practice statement).

#### Supplementary item

The standard has been ≤ 28 cmH_2_O (more pressure may be required in conditions with decreased chest wall compliance) [360]. It should be noted that restricting plateau pressure alone is not sufficient in controlling overdistention if there is strong spontaneous breathing. It should also be noted that the plateau pressure and maximum airway pressure are different even in the case of pressure-control ventilation.

#### Rationale

*Summary of evidence*:

No applicable studies.

*Certainty of evidence*:

There are no applicable studies, so the certainty of evidence could not be evaluated.

*Balance of effects, acceptability, and feasibility determination*:

Plateau pressure restrictions in patients with ARDS could suppress VALI, but there are concerns that changes in blood acid–base balance associated with hypercapnia could result in adverse effects on hemodynamics and tissue metabolism. However, there are no applicable studies, and it is not possible to determine the balance of effects. Plateau pressure restrictions could be implemented by changing only the ventilator settings, which is considered to be sufficiently acceptable and feasible.

### PCQ5: Should a protocol be used when liberating pediatric patients with acute respiratory failure from mechanical ventilator?

#### Background

Prolonged mechanical ventilation increases the incidence of adverse events; meanwhile, premature extubation increases the reintubation rate and mortality [361]. Whether to use a protocol when liberating pediatric patients with acute respiratory failure from mechanical ventilator is a key clinical issue.

#### Recommendation

We suggest to use a protocol when liberating pediatric patients with acute respiratory failure who have been mechanically ventilated for more than 24 h from ventilator. (weak recommendation/ very low certainty of evidence: GRADE 2D).

#### Supplementary item

None.

#### Rationale

*Summary of evidence*:

Results of the 2 adopted RCTs [362, 363] (*N* = 479) showed that, for beneficial outcomes, 30-day mortality (1 RCT [362]; *N* = 260) decreased by 1/1000 patients (95% CI: 14 fewer to 88 more patients) and duration of mechanical ventilation (1 RCT [363]; *N* = 219) was shortened by an average of 2.9 days (95% CI: 5.91 days shorter to 0.11 days longer). As a result, the desirable effect was judged as “small.”

There were no reports on critical harmful outcomes; for other harmful outcomes, reintubation (2 RCTs [362, 363]; *N* = 479) decreased by 2/1000 patients (95% CI: 48 fewer to 103 more patients) and unplanned extubation (1 RCT [362]; *N* = 260) occurred in 41 fewer/1000 patients (95% CI: 57 fewer to 19 more patients). As a result, the undesirable effect was judged as “trivial.”

From the above, the balance of effects was judged as “probably favors the intervention.”

*Certainty of evidence*:

For the desirable effects, 30-day mortality and duration of mechanical ventilation were both set with a certainty of evidence of “very low.” For the undesirable effects, there were no reports of critical outcomes. As the direction of desirable and undesirable effects was consistent, the certainty of evidence of all outcomes was judged as “very low.”

*Balance of effects, acceptability, and feasibility determination*:

The balance of effects was judged as “probably favors the intervention,” and it was judged that patients and families would accept this method. It is thought that its implementation including evaluations of sedation level may not be easy in ICUs that are not specialized for children. Therefore, the feasibility of this intervention varies from facility to facility.

### PCQ6: Should HFOV be used for pediatric patients with moderate to severe ARDS?

#### Background

HFOV is a mechanical ventilation mode, where both lung recruitment and restricted tidal volume can be achieved. Though it cannot be said to be a common mechanical ventilation mode for children. Whether to use HFOV for pediatric patients with moderate to severe ARDS is a key clinical issue.

#### Recommendation

We suggest against implementing HFOV for pediatric patients with ARDS.

(weak recommendation/very low certainty of evidence: GRADE 2D).

#### Supplementary item

This recommendation does not reject the implementation of this procedure in facilities that already have an HFOV-specialized ventilator and are familiar with its use.

#### Rationale

*Summary of evidence*:

Results of the 4 adopted RCTs [364–367] (*N* = 292) showed that, for beneficial outcomes, mortality (4 RCTs [364–367]; *N* = 292) decreased by 12/1000 patients (95% CI: 108 fewer to 116 more patients), and ventilator-free days (1 RCT [364]; *N* = 102) was shortened by an average of 0 days (95% CI: 0.82 days shorter to 0.82 days longer). From the above, the desirable effect was judged as “trivial.” For harmful outcomes, hemodynamic instability (2 RCTs [365, 367]; *N* = 76) occurred in 53 more/1000 patients (95% CI: 18 fewer to 689 more patients), and the undesirable effect was judged as “small.” Therefore, the balance of effects was judged as “probably favors the comparison.”

*Certainty of evidence*:

The direction of desirable and undesirable effects was not the same, and the certainty of evidence of the overall outcome was judged as “very low.”

*Balance of effects, acceptability, and feasibility determination*:

The balance of effects was judged as “probably favors the comparison,” and it was judged that patients and families would accept this method. HFOV cannot be implemented without a specialized ventilator. Moreover, in facilities that do not use HFOV on a regular basis, purchasing the dedicated ventilators and providing training on how to use them will increase on-site burden. Therefore, feasibility was judged as likely low.

### PCQ7: Should APRV be used for pediatric patients with ARDS?

#### Background

Alongside treatment of underlying diseases, mechanical ventilation in patients with ARDS is important [368]. The optimal ventilation mode in mechanical ventilation is not clear. APRV is used in some cases of pediatric ARDS to maintain a high airway pressure, but its effectiveness in children is unclear. Therefore, whether to use APRV in pediatric patients with ARDS is a key clinical issue.

#### Recommendation

We suggest against implementing APRV as a ventilation mode in pediatric patients with ARDS (weak recommendation/very low certainty of evidence: GRADE 2D).

#### Supplementary item

This recommendation does not reject the implementation of APRV in facilities familiar with its use.

#### Rationale

*Summary of evidence*:

SR results showed that there was 1 adopted RCT [369] (*N* = 52) in line with PICO. For beneficial outcomes, developmental prognosis (PCPC after 180 days) showed 0 change (95% CI: 0.4 worsened to 0.4 improved), ventilator-free days shortened by an average of 4.5 days (95% CI: 10.35 days shorter to 1.35 days longer), and length of hospital stay was shortened by an average of 5 days (95% CI: 10.73 days shorter to 0.73 days longer). Twenty-eight-day mortality rate was initially assumed as a desirable effect, but as described later, mortality rate increased, so this was considered as an undesirable effect. Therefore, the desirable effect due to intervention was judged as “trivial.” For harmful outcomes, 28-day mortality rate increased by 269/1000 patients (95% CI: 8 fewer to 845 more patients) for the intervention compared to the control, and exacerbated hemodynamics was observed in 77 fewer/1000 patients (95% CI: 111 fewer to 231 more patients). Therefore, the undesirable effect was judged as “moderate.” From the above, the balance of benefits and harms was judged as “probably favors the comparison.”

*Certainty of evidence*:

The direction of desirable and undesirable effects was not the same, and the certainty of evidence for the overall outcome adopted the certainty of the outcome with the lowest certainty and set as “very low.”

*Balance of effects, acceptability, and feasibility determination*:

The balance of effects was judged as “probably favors the comparison.” There was the possibility that this had a negative effect on mortality rate, and intervention may not be acceptable. APRV is feasible if a specific ventilator is available, but if not, then feasibility is low, and the overall feasibility is set as "varies".

### PCQ8: How should target SpO_2_ values be set in pediatric patients with ARDS?

#### Background

Management with high oxygen concentration has been reported to induce adverse events that promote pulmonary fibrogenesis [370]. Meanwhile, it is unclear whether low SpO_2_ management improves survival rate or worsens prognosis through adverse events. Therefore, the target SpO_2_ value that should be set in pediatric patients with ARDS is a key clinical issue.

#### Recommendation

We cannot provide a specific target SpO_2_ value for pediatric patients with ARDS, but target SpO_2_ value are set so as to avoid excessively high or low oxygen levels, which could cause organ damage (in our practice statement).

#### Supplementary item

None.

#### Rationale

*Summary of evidence*:

No applicable studies.

*Certainty of evidence*:

There are no applicable studies, so a description of the certainty of evidence cannot be provided.

*Balance of effects, acceptability, and feasibility determination*:

There are no applicable studies, and it is not possible to judge the balance of effects. Management with a specific SpO_2_ target is possible with only changes to ventilator settings and is already implemented in daily clinical practice, so it is thought that its acceptability and feasibility are sufficiently high.

### PCQ9: Should muscle relaxants be used at an early stage in pediatric patients with moderate or severe ARDS?

#### Background

The use of muscle relaxants for patients with ARDS has been reported to avoid excessive stress to alveoli, reduce barotrauma, and improve oxygenation, suggesting an improved prognosis [371]. However, it has also often been reported that complications could occur due to muscle relaxants, which reduce or eliminate spontaneous breathing [371]. Whether to use muscle relaxants at an early stage for pediatric patients with moderate or severe ARDS is a key clinical issue.

#### Recommendation

We cannot provide a recommendation on the early use of muscle relaxants for pediatric patients with moderate or severe ARDS, but muscle relaxants have been used at an early stage in accordance with the treatment strategy of adult patients with moderate or worse ARDS (in our practice statement).

#### Supplementary item

None.

#### Rationale

*Summary of evidence*:

No applicable studies.

*Certainty of evidence*:

There are no applicable studies, so no description of the certainty of evidence can be provided.

*Balance of effects, acceptability, and feasibility determination*:

Muscle relaxants in patients with ARDS requiring mechanical ventilation are thought to have the advantages of decreased patient–ventilator asynchrony, decreased oxygen consumption, increased respiratory compliance and functional residual capacity, and prevention of local alveolar overdistention due to strong spontaneous breathing [371]. Meanwhile, there are concerns of harmful effects, such as the onset of ICU-AW due to the use of muscle relaxants. However, there are no studies that directly evaluated these aspects, so it is not possible to judge the balance of effects. Muscle relaxants themselves are used in daily clinical practice, and it is thought that there are no problems with acceptability or feasibility.

### PCQ10: Should pediatric patients with moderate to severe ARDS be placed in the prone position?

#### Background

The prone position may be effective as treatment for moderate to severe ARDS [372], as it improves oxygenation and prevents VALI, but there is room for debate on its clinical effects based on previous RCTs and meta-analyses [265, 373, 374]. The prone position can be implemented without special equipment, but complications can occur with repositioning of the patients and prolonged management. Therefore, whether to place pediatric patients with moderate to severe ARDS in the prone position is a key clinical issue.

#### Recommendation

We suggest placing pediatric patients with moderate to severe ARDS in the prone position (weak recommendation/very low certainty of evidence: GRADE 2D).

#### Supplementary item

Placing children in the prone position requires the facility to be familiar with the procedure, and applications need to be considered for each facility, including the degree of sedation.

#### Rationale

*Summary of evidence*:

There was 1 adopted RCT [375] (*N* = 101; patients with *P/F* ratio < 300 targeted). For beneficial outcomes, 28-day mortality decreased by 2/1000 patients (95% CI: 59 fewer to 217 more patients), poor developmental prognosis decreased by 103/1000 patients (95% CI: 174 fewer to 75 more patients), and ventilator-free days was shortened by an average of 0.3 days (95% CI: 3.63 days shorter to 3.03 days longer). As a result, the desirable effect due to intervention was judged as “small.” For harmful outcomes, endotracheal tube troubles occurred in 22 fewer/1000 patients (95% CI: 78 fewer to 175 more patients), and the predicted undesirable effect was judged as “trivial.” From the above, for whether benefits due to intervention would outweigh harms, it was judged that “probably favors the intervention.”

*Certainty of evidence*:

The direction of outcomes for desirable and undesirable effects were not the same, and the certainty of evidence was judged to be “very low.”

*Balance of effects, acceptability, and feasibility determination*:

The balance of effects was judged as “probably favors the intervention.” It is thought that there are not many human resources needed for repositioning small children to the prone position, but the facility will need to be familiar with the procedure for safe management, so it was judged that acceptability and feasibility of this procedure was likely possible.

### PCQ11: Should nitric oxide inhalation therapy be used in pediatric patients with ARDS?

#### Background

Various pathological conditions such as hypoxic pulmonary vasospasm and ventilation–perfusion imbalance are involved in ARDS [376]. There is a possibility that NO inhalation, which has a pulmonary vessel dilatation effect [377], may be effective, but its clinical effects vary according to reports. Therefore, whether to use NO inhalation therapy for pediatric patients with ARDS is a key clinical issue.

#### Recommendation

We suggest against routinely implementing NO inhalation therapy for pediatric patients with ARDS (weak recommendation/very low certainty of evidence: GRADE 2C).

#### Supplementary item

This treatment can be acceptable in limited circumstances. Examples include temporary use until the introduction of extracorporeal membrane oxygenation, and situations, where there are no other treatments and a high mortality rate is predicted. It should be noted that NO inhalation therapy for ARDS is not covered by insurance.

#### Rationale

*Summary of evidence*:

Meta-analysis results of the 3 adopted RCTs [378–380] (*N* = 177) showed that, for beneficial outcomes, mortality rate (3 RCTs [378–380]; *N* = 177) decreased by 54/1000 patients (95% CI: 237 fewer to 409 more patients), and 28-day ventilator-free days (2 RCTs [378, 379]; *N* = 71) were extended by an average of 4.90 days (95% CI: 0.78 days longer to 9.03 days longer). However, there is the possibility that mortality increased by 409/1000 patients, so the desirable effect was judged as “small.” For harmful outcomes, serious side effects (3 RCTs [378–380]; *N* = 177) decreased by 10/1000 patients (95% CI: 21 fewer to 99 more patients). However, there is the possibility that it could increase 99/1000 patients, so the undesirable effect was judged as “trivial.” Therefore, the balance of effects was judged as “probably favors the intervention.”

*Certainty of evidence*:

The direction of critical outcomes was the same, and the certainty of evidence was judged as “low.”

*Balance of effects, acceptability, and feasibility determination*:

The balance of effects was judged as “probably favors the intervention,” and it was judged that there were no problems with patient and family acceptability. However, the predicted net effect was uncertain, and there are many facilities, where the feasibility of this treatment would be low, so we decided not to propose that this be implemented routinely.

### PCQ12: Should surfactant be used in pediatric patients with ARDS?

#### Background

Various factors such as alveolar epithelial injury and alveolar surfactant dysfunction are involved in the pathophysiology of ARDS [376]. Surfactant administration can be effective, but its clinical effects vary according to reports. Therefore, whether to use surfactant on pediatric patients with ARDS is a key clinical issue.

#### Recommendation

We suggest against using surfactants for pediatric patients with ARDS (weak recommendation/very low certainty of evidence: GRADE 2D).

#### Supplementary item

None.

#### Rationale

*Summary of evidence*:

Meta-analysis results of the 9 incorporated RCTs [381–389] showed that, for the beneficial outcomes due to surfactant use, mortality (9 RCTs [381–389]; *N* = 658) decreased by 64/1000 patients (95% CI: 130 fewer to 45 more patients), 28-day ventilator-free days (2 RCTs [388, 389]; *N* = 261) were shortened by an average of 0.65 days (95% CI: 5.26 days shorter to 3.95 days longer), and the length of hospital stay (2 RCTs [387, 388]; *N* = 194) was shortened by an average of 1.67 days (95% CI: 7.82 days shorter to 4.49 days longer). Therefore, the desirable effect was judged as “small.” For harmful outcomes, drug-related adverse events (7 RCTs [381, 382, 385–389]; *N* = 589) occurred in 180 more/1000 patients (95% CI: 81 more to 360 more patients). Many of the drug-related adverse events were transient hypoxemia and hemodynamic compromises, and few were serious, so the undesirable effect was judged as “small.” Therefore, the balance of effects was judged as “does not favor either the intervention or the comparison.”

*Certainty of evidence*:

The direction of critical outcomes was not the same, and the certainty of evidence was set as “very low.”

*Balance of effects, acceptability, and feasibility determination*:

The balance of effects was judged as “does not favor either the intervention or the comparison.” Given that surfactants are expensive drugs, interventions could increase drug-related adverse events, and resources are needed for implementation, it was judged that acceptability was “probably no,” and feasibility was “probably no.”

### PCQ13: Should steroids be used in pediatric patients with ARDS?

#### Background

ARDS is a pathological condition caused by inflammation that has spread to the lungs due to various causes [376]. Steroids have an suppressing effect on inflammation mediators, but they can also decrease the immune system. Therefore, whether to use steroids is a key clinical issue.

#### Recommendation

We cannot provide a recommendation on the administration of steroids for pediatric patients with ARDS. Steroid administration is examined in consideration of patient background and pathophysiology (in our practice statement).

#### Supplementary item

This is a description of the current clinical practice, and this does not reject the use of steroids.

#### Rationale

*Summary of evidence*:

There was only 1 RCT [390] (*N* = 35). For beneficial outcomes, the mortality rate decreased by 88/1000 patients (95% CI: 110 fewer to 344 more patients), ventilator-free days was extended by an average of 1.32 days (95% CI: 3.32 days shorter to 5.96 days longer), and length of hospital stay was shortened by an average of 6.87 days (95% CI: 15.32 days shorter to 1.58 days longer). The confidence intervals were large, and there was the possibility that mortality rate increased by 344 patients, so the desirable effect was judged as “trivial.” For harmful outcomes, the infectious disease incidence decreased by 219/1000 patients (95% CI: 296 fewer to 175 more patients), and hyperglycemia increased by 142/1000 patients (95% CI: 138 fewer to 684 more patients). The critical harmful outcomes of developmental prognosis and presence of myopathy/neuropathy were not evaluated, and the undesirable effect was judged as “varies.” The balance of effects was judged as “varies.”

*Quality of evidence*:

The sample size of the applicable study was extremely small, and patient backgrounds also varied significantly. Therefore, it was thought difficult to provide a recommendation from this meta-analysis.

*Balance of effects, acceptability, and feasibility determination*:

The balance of effects was judged as “varies.” As steroids are frequently used in daily clinical practice, it was thought that there were no problems with feasibility. However, the critical undesirable effects of developmental prognosis and presence of myopathy/neuropathy were not evaluated. Therefore, the acceptability of this was thought to vary by stakeholder.

### PCQ14: Should a protocol be used for the sedation of pediatric respiratory failure patients?

#### Background

The introduction of sedation protocols is expected to shorten the length of mechanical ventilation and reduce the risk of onset of delirium and withdrawal symptoms, but its usefulness in the mechanical ventilation of pediatric patients with ARDS has not been established. Therefore, whether to use a protocol for the sedation of pediatric patients with ARDS is a key clinical issue.

As sedation protocols are interventions for patients with various diseases to be liberated from mechanical ventilation, target populations were not limited to patients with ARDS but included all mechanically ventilated children.

#### Recommendation

We suggest the use of protocols for the sedation of pediatric respiratory failure patients (weak recommendation/moderate certainty of evidence: GRADE 2B).

#### Supplementary item

The sedation protocol examined was based on an algorithm in which nurses assess and adjust sedation and analgesia on a scale.

#### Rationale

*Summary of evidence*:

There was 1 RCT [391] (*N* = 2449) that compared a sedation protocol group with a normal clinical practice group. For beneficial outcomes, mortality was lower by 13/1000 patients (95% CI: 25 fewer to 4 more patients), length of mechanical ventilation changed by an average of 0 days (95% CI: 0.46 days shorter to 0.46 days longer), and length of hospital stay was shortened by an average of 2 days (95% CI: 3.04 days shorter to 0.96 days longer). There were no reports of the developmental prognosis. Therefore, the desirable effect was judged as “small.” Meanwhile, for harmful outcomes, there were no reports of absolute numbers of adverse events, such as unplanned extubation or worsening of respiratory status. The report (Curley 2015 [391]) taken up in the meta-analysis indicated that withdrawal syndrome was more by 29/1000 patients (95% CI: 4 fewer to 60 more patients), and the undesirable effect was judged as “trivial.” Therefore, the balance of effects was judged as “probably favors the intervention.”

*Certainty of evidence*:

The direction of critical outcomes was the same, and the certainty of evidence was judged as “moderate.”

*Balance of effects, acceptability, and feasibility determination*:

The balance of effects was judged as “probably favors the intervention,” and it was judged that there were no major problems with patient and family acceptability. There is a possibility that sedation/analgesia scale introduction for children in ICUs with few pediatric patients would be a burden, and feasibility was judged as “varies.”

### PCQ15: Should daily sedation interruption (DSI) be implemented for pediatric respiratory failure patients?

#### Background

DSI is expected to shorten the length of mechanical ventilation and reduce the risk of delirium and withdrawal symptoms, but this is not an established treatment in pediatric patients with ARDS. Therefore, whether to implement DSI in pediatric patients with ARDS is a key clinical issue.

As DSI is an intervention for patients ventilated with various diseases to be liberated from mechanical ventilation, target populations were not limited to patients with ARDS, but included all mechanically ventilated children.

#### Recommendation

We suggest against implementing DSI for pediatric respiratory failure patients (weak recommendation/very low certainty of evidence: GRADE 2D).

#### Supplementary item

There is currently insufficient evidence for providing a recommendation for DSI. It is important to evaluate on a daily basis whether withdrawal from a ventilator is possible.

#### Rationale

*Summary of evidence*:

There were 3 RCTs [392–394] (*N* = 261) that compared the use of DSI in sedation management. For beneficial outcomes, the length of mechanical ventilation was shortened by an average of 1.48 days (95% CI: 3.48 days shorter to 0.51 days longer), and the length of hospital stay was shortened by an average of 3.4 days (95% CI: 8.84 days shorter to 2.04 days longer); there were no reports of developmental prognosis. From the above, the desirable effect was judged as “small.” Meanwhile, for harmful outcomes, mortality increased by 166/1000 patients (95% CI: 92 fewer to 1000 more patients), and unplanned extubation decreased by 26/1000 patients (95% CI: 41 fewer to 39 more patients). From the above, the undesirable effect was judged as “moderate.” As a result, the balance of effects was judged as “probably favors the comparison.” The causal relationship between intervention and increased mortality in the present meta-analysis is unclear.

*Certainty of evidence*:

The direction of outcomes was not the same, and the certainty of evidence was set as “very low.”

*Balance of effects, acceptability, and feasibility determination*:

The balance of effects was judged as “probably favors the comparison.” The personnel costs after DSI are large in ICUs that have few opportunities to hospitalize pediatric patients. Additionally, the increased discomfort in children due to DSI may result in situations, where family acceptability is difficult. Therefore, acceptability and feasibility were set as “cannot be unequivocally stated.”

## Conclusions

Continuing from the 2016 edition, we created a clinical practice guideline using the GRADE system. Considering our experience from the previous edition, we established a creation governing committee in close collaboration with the three academic societies from the preparation stage. There were many more CQs, so there were also more SR reviewers, and we were forced to increase the complexity of our operations. Therefore, we established an SR governing committee, with responsible committee members assigned to each area; we also formed a support team, which provided operational support for the governing committee. There were some changes in the SR committee members, but committee members who were involved in the creation of the 2016 edition played a leading role, and we were able to complete the SR work almost as planned. Some of the recommendation contents, such as those for steroid administration, became more in-depth than existing guidelines; it is thought that we were able to provide a certain degree of originality as a result. Meanwhile, many of the newly set CQs did not have RCTs; in addition, despite the fact that this was a clinical practice guideline for medical professionals in Japan, it could be seen that there was almost no evidence from Japan, and this has reaffirmed the need to provide evidence from Japan. We would like to reflect on the points clarified in the present creation process, and we would like to create a better clinical practice guideline in the next revision.

## Supplementary Information

**Additional file 1.** Contains Modified Preferred Reporting items of Systematic Reviews and Meta-Analyses (PRISMA) flow-chart, risk of bias summary, forest plots, evidence profiles, and evidence to decision table for CQ1–13 (area A) according to the GRADE system

**Additional file 2.** Contains Modified Preferred Reporting items of Systematic Reviews and Meta-Analyses (PRISMA) flow-chart, risk of bias summary, forest plots, evidence profiles, and evidence to decision table for CQ14–18 (area B) according to the GRADE system

**Additional file 3.** Contains Modified Preferred Reporting items of Systematic Reviews and Meta-Analyses (PRISMA) flow-chart, risk of bias summary, forest plots, evidence profiles, and evidence to decision table for CQ19–30 (area C) according to the GRADE system

**Additional file 4.** Contains Modified Preferred Reporting items of Systematic Reviews and Meta-Analyses (PRISMA) flow-chart, risk of bias summary, forest plots, evidence profiles, and evidence to decision table for CQ31–38 (area D) according to the GRADE system

**Additional file 5.** Contains Modified Preferred Reporting items of Systematic Reviews and Meta-Analyses (PRISMA) flow-chart, risk of bias summary, forest plots, evidence profiles, and evidence to decision table for CQ39–48 (area E) according to the GRADE system

**Additional file 6.** Contains Modified Preferred Reporting items of Systematic Reviews and Meta-Analyses (PRISMA) flow-chart, risk of bias summary, forest plots, evidence profiles, and evidence to decision table for PCQ1–15 (children) according to the GRADE system

**Additional file 7.** Contains tables disclosing intellectual and financial conflicts of interest for each person who participated in the creation of this guideline

## Data Availability

Additional file 1 to 6 contains Modified Preferred Reporting Items of Systematic Reviews and Meta-Analyses (PRISMA) flow-chart, risk of bias table, forest plots, evidence profiles, and evidence to decision table for each CQ according to the GRADE system. Additional file 7 contains tables disclosing intellectual and financial conflicts of interest for each person who participated in the creation of this guideline.

## Abbreviations

A/C: Assisted controlled ventilation
AGREEII: Appraisal of Guidelines for Research and Evaluation II
APRV: Airway pressure release ventilation
ARDS: Acute respiratory distress syndrome
BAL: Bronchoalveolar lavage
BNP: Brain natriuretic peptide
CI: Confidence interval
CMV: Cytomegalovirus
COPD: Chronic obstructive pulmonary disease
CQ: Clinical question
CRP: C-reactive protein
DI: Disagreement index
DSI: Daily sedation interruption
EIT: Electrical impedance tomography
GRADE: Grading of Recommendations Assessment, Development and Evaluation
GPS: Good practice statement
HFNC: High-flow nasal cannula oxygen therapy
HFOV: High-frequency oscillatory ventilation
HRCT: High-resolution CT
ICU: Intensive care unit
ICU-AW: ICU-acquired weakness
IGRA: Interferon γ release assay
IPA: Invasive pulmonary aspergillosis
KQ: Key question
MV: Mechanical ventilation
NO: Nitric oxide
NPPV: Non-invasive positive pressure ventilation
ODI: Optical density index
PALICC: Pediatric Acute Lung Injury Consensus Conference Criteria
PARDS: Pediatric ARDS
PCP: *Pneumocystis* Pneumonia
PCT: Procalcitonin
PCV: Pressure-control ventilation
PCQ: Pediatric clinical question
PEEP: Positive end-expiratory pressure
*P/F*: PaO_2_/FIO_2_
PICS: Post-intensive care syndrome
PICU: Pediatric intensive care unit
QOL: Quality of line
RCT: Randomized controlled trial
SIMV: Synchronized intermittent mandatory ventilation
SR: Systematic review
SUCRA: Surface under the cumulative ranking curve
VALI: Ventilator-associated lung injury
VAP: Ventilator-associated pneumonia
VCV: Volume-control ventilation
VFD: Ventilator-free days
VV-ECMO: Veno-venous extracorporeal membrane oxygenation
