# Supplementary material for: ARDS Clinical Practice Guideline 2021
Source: J Intensive Care. 2022 Jul 8;10:32. doi: 10.1186/s40560-022-00615-6 (PMC9263056; doi:10.1186/s40560-022-00615-6)
Supplement: Supplementary file 1 — Additional file 1. Contains Modified Preferred Reporting items of Systematic Reviews and Meta-Analyses (PRISMA) flow-chart, risk of bias summary, forest plots, evidence profiles, and evidence to decision table for CQ1–13 (area A) according to the GRADE system [file 40560_2022_615_MOESM1_ESM.docx]

Additional file 1

Modified Preferred Reporting items of Systematic Reviews and Meta-Analyses (PRISMA) flow-chart, risk of bias summary, forest plots, evidence profiles, and evidence to decision table for CQ1-13 (area A) according to the GRADE system

Table of contents

1. CQ1
   1. Search strategy p.4
   2. Flow diagram p.4
   3. Risk of bias p.4
   4. Forest plot p.4
   5. Evidence Profile p.4
   6. Evidence-to-Decision table p.5
2. CQ2
   1. Search strategy p.13
   2. Flow diagram p.15
   3. Risk of bias p.16
   4. Forest plot p.17
   5. Evidence Profile p.18
   6. Evidence-to-Decision table p.24
3. CQ3
   1. Search strategy p.38
   2. Flow diagram p.39
   3. Risk of bias p.40
   4. Forest plot p.41
   5. Evidence Profile p.41
   6. Evidence-to-Decision table p.46
4. CQ4
   1. Search strategy p.58
   2. Flow diagram p.60
   3. Risk of bias p.62
   4. Forest plot p.63
   5. Evidence Profile p.64
   6. Evidence-to-Decision table p.68
5. CQ5
   1. Search strategy p.80
   2. Flow diagram p.82
   3. Risk of bias p.83
   4. Forest plot p.84
   5. Evidence Profile p.85
   6. Evidence-to-Decision table p.87
6. CQ6
   1. Search strategy p.98
   2. Flow diagram p.99
   3. Risk of bias p.100
   4. Forest plot p.100
   5. Evidence Profile p.100
   6. Evidence-to-Decision table p.101
7. CQ7
   1. Search strategy p.111
   2. Flow diagram p.112
   3. Risk of bias p.113
   4. Forest plot p.113
   5. Evidence Profile p.113
   6. Evidence-to-Decision table p.114
8. CQ8
   1. Search strategy p.124
   2. Flow diagram p.127
   3. Risk of bias p.129
   4. Forest plot p.130
   5. Evidence Profile p.133
   6. Evidence-to-Decision table p.139
9. CQ9
   1. Search strategy p.154
   2. Flow diagram p.157
   3. Risk of bias p.158
   4. Forest plot p.159
   5. Evidence Profile p.160
   6. Evidence-to-Decision table p.162
10. CQ10
    1. Search strategy p.173
    2. Flow diagram p.178
    3. Risk of bias p.181
    4. Forest plot p.184
    5. Evidence Profile p.186
    6. Evidence-to-Decision table p.200
11. CQ11
    1. Search strategy p.222
    2. Flow diagram p.224
    3. Risk of bias p.225
    4. Forest plot p.225
    5. Evidence Profile p.225
    6. Evidence-to-Decision table p.226
12. CQ12
    1. Search strategy p.236
    2. Flow diagram p.241
    3. Risk of bias p.244
    4. Forest plot p.245
    5. Evidence Profile p.252
    6. Evidence-to-Decision table p.261
13. CQ13
    1. Search strategy p.276
    2. Flow diagram p.277
    3. Risk of bias p.278
    4. Forest plot p.279
    5. Evidence Profile p.281
    6. Evidence-to-Decision table p.285

**CQ1 Should ARDS diagnoses be conducted (for acute respiratory failure patients)?**

1. Search strategy

Not applicable

1. Flow diagram

Not applicable

1. Risk of bias

Not applicable

1. Forest plot

Not applicable

1. Evidence Profile

Not applicable

1. Evidence-to-Decision table

| Question | |
| --- | --- |
| **CQ1：Should ARDS diagnoses be conducted (for acute respiratory failure patients)?** | |
| **Population:** | Patients with acute respiratory failure |
| **Intervention:** | Diagnose ARDS |
| **Comparison:** | Not diagnose ARDS |
| **Main outcomes:** | Health-related quality of life after discharge, long-term survival and death (longer than 3 months), short-term survival and death (less than 3 months, in hospital, in intensive care unit [ICU]), serious adverse events (death, serious sequelae) |
| **setting:** | Situation equivalent to the emergency room or ICU |
| **perspective:** | Individual |
| **background:** | ARDS is a serious and urgent condition, and its diagnosis and treatment are extremely important.  ARDS is non-cardiogenic pulmonary edema caused by increased permeability of the vascular endothelium and alveolar epithelium following a causative disease or injury. The diagnosis of ARDS in clinical practice is based on the Berlin definition proposed in 2012. In other words, the diagnosis is based on the following four criteria: 1) acute onset, 2) bilateral shadows on chest imaging, 3) the condition cannot be explained by left heart failure alone, and 4) hypoxemia.  The diagnosis of ARDS has a significant impact on the understanding of the condition, evaluation of the severity of the disease, and treatment strategy. Furthermore, the implementation of the recommendations presented in this guideline will potentially improve the prognosis. |
| **conflict of interest:** | None |

**What is a good practice statement?**

A good practice statement (GPS) is evidence that is presented as a “strong recommendation” when the net benefit of a medical intervention is so certain, as indicated by indirect evidence, that it would be ethically difficult not to perform the intervention without a formal literature search. It is also considered a GPS when it would be difficult and unproductive to collect linked indirect evidence to support the recommendation. An example of this would be the difference in outcomes between jumping from an airplane with or without a parachute.

In this guideline, the following criteria should be considered for presentation as GPS. In this case, the recommendation would be “Strong,” and the Quality of Evidence would be “Ungraded” (Ungraded Recommendation). The recommendation statement should be clearly stated as (GPS: Good practice statement).

**★Questions to determine if a CQ is a good practice statement**

Seven criteria for judging a CQ as a GPS

| **Question** | **Answer** |
| --- | --- |
| 1. Is the statement clear and feasible? | Yes, Rationale: Making a diagnosis of ARDS is inexpensive, has few complications, and is easily implemented. |
| 1. Is the message really necessary? | Yes, Rationale: The diagnosis of ARDS may have a significant impact on understanding the disease, assessing its severity, and treatment strategies. |
| 1. Is the net benefit significant and unquestionable? | Yes, Rationale: The diagnosis of ARDS may have a significant impact on understanding the disease, assessing its severity, and treatment strategies, potentially contributing to patient benefit. Specifically, diagnosis of ARDS and implementation of the recommendations in this guideline may improve prognosis. |
| 1. Is it difficult to collect and summarize evidence on clinical questions? | Yes, Rationale: There are no clinical studies comparing the diagnosis of ARDS with or without ARDS in patients with suspected acute respiratory failure. |
| 1. Are there any specific issues (e.g., fairness) that should be considered? | No, Rationale: No problem. |
| 1. Is the rationale clearly presented? | Yes, Rationale: Diagnosing ARDS is important because implementing the recommendations in this guideline for patients with ARDS may improve their prognosis. |
| 1. Should the evaluation be based on a formal GRADE? | No. Rationale: Because the evidence is difficult to summarize, and there seems to be no need to consider the balance of benefits and harms, fairness, and costs. |

# Assessment

| Problem Is the problem a priority? | | |
| --- | --- | --- |
| Judgment | Research evidence | ADDITIONAL considerations |
| ○ No  ○ Probable no  ○ Probable yes  ● Yes  ○ Varied  ○ Do not know | Acute respiratory failure is a serious and urgent condition, and its diagnosis and treatment are extremely important.  ARDS is non-cardiogenic pulmonary edema caused by increased permeability of the vascular endothelium and alveolar epithelium following a causative disease or injury. The diagnosis of ARDS in clinical practice is based on the Berlin definition proposed in 2012. Namely, the diagnosis is based on the following four criteria: 1) acute onset, 2) bilateral shadows on chest imaging, 3) the condition cannot be explained by left heart failure alone, and 4) hypoxemia.  The diagnosis of ARDS has a significant impact on the understanding of the condition, evaluation of the disease severity, and treatment strategy. Furthermore, the implementation of the recommendations presented in this guideline will potentially improve the prognosis.  For these reasons, this issue is of high priority. |  |
| Desirable effects How substantial are the desirable anticipated effects? | | |
| Judgment | Research evidence | ADDITIONAL considerations |
| ○ Trivial  ○ Small  ○ Moderate  ● Large  ○ Varies  ○ Do not know | Although there is no relevant evidence, once ARDS is diagnosed, implementing the recommendations in this guideline can improve prognosis and have a significant effect. |  |
| Undesirable effects How substantial are the undesirable anticipated effects? | | |
| Judgment | Research evidence | ADDITIONAL considerations |
| ○ Large  ○ Moderate  ○ Small  ● Trivial  ○ Varies  ○ Do not know | The diagnosis of ARDS as defined by the Berlin definition is made primarily by history and physical examination, chest imaging studies (plain chest radiographs, chest computed tomography (CT)), and arterial blood gas analysis. These tests are commonly performed medical procedures and are considered harmless. |  |
| Certainty of evidence What is the overall certainty of the evidence of effects? | | |
| Judgment | Research evidence | ADDITIONAL considerations |
| ○ Very low  ○ Low  ○ Moderate  ● High  ○ No included studies | No clinical studies have compared whether diagnosing patients with acute respiratory failure with or without ARDS directly improved patient outcomes compared to not diagnosing ARDS. |  |
| Values Is there important uncertainty about or variability in how much people value the main outcomes? | | |
| Judgment | Research evidence | ADDITIONAL considerations |
| ○ Important uncertainty or variability  ○ Possibly important uncertainty or variability  ○ Probably no important uncertainty or variability  ● No important uncertainty or variability | The diagnosis of ARDS could potentially lead to patient benefits, such as improved life expectancy. There seems to be little variation in the magnitude of this value among people. |  |
| Balance of effects Does the balance between desirable and undesirable effects favor the intervention or the comparison? | | |
| Judgment | Research evidence | ADDITIONAL considerations |
| ○ Favors the comparison  ○ Probably favors the comparison  ○ Does not favor either the intervention or the comparison  ○ Probably favors the intervention  ● Favors the intervention  ○ Varies  ○ Do not know | Comparing the benefits and harms, the benefits of making a diagnosis of ARDS are considered significant. |  |
| Acceptability Is the intervention acceptable to key stakeholders? | | |
| Judgment | Research evidence | ADDITIONAL considerations |
| ○ No  ○ Probably no  ● Probably yes  ○ Yes  ○ Varies  ○ Do not know | It is a commonly medical practiced and probably acceptable. |  |
| Feasibility Is the intervention feasible to implement? | | |
| Judgment | Research evidence | ADDITIONAL considerations |
| ○ No  ○ Probably no  ○ Probably yes  ● Yes  ○ Varies  ○ Do not know | It seems feasible because it is already being implemented in daily clinical practice. |  |

# Summary of Judgment

|  | **Judgment** | | | | | | |
| --- | --- | --- | --- | --- | --- | --- | --- |
| **PROBLEM** | No | Probably no | Probably yes | Yes |  | Varies | Do not know |
| **DESIRABLE EFFECTS** | Trivial | Small | Moderate | Large |  | Varies | Do not know |
| **UNDESIRABLE EFFECTS** | Large | Moderate | Small | Trivial |  | Varies | Do not know |
| **CERTAINTY OF EVIDENCE** | Very low | Low | Moderate | High |  |  | No included studies |
| **VALUES** | Important uncertainty or variability | Possibly important uncertainty or variability | Probably no important uncertainty or variability | No important uncertainty or variability |  |  |  |
| **BALANCE OF EFFECTS** | Favors the comparison | Probably favors the comparison | Does not favor either the intervention or the comparison | Probably favors the intervention | Favors the intervention | Varies | Do not know |
| **ACCEPTABILITY** | No | Probably no | Probably yes | Yes |  | Varies | Do not know |
| **FEASIBILITY** | No | Probably no | Probably yes | Yes |  | Varies | Do not know |

# Type of Recommendation

| Strong recommendation against the intervention | Conditional recommendation against the intervention | Conditional recommendation for either the intervention or the comparison | Conditional recommendation for the intervention | Strong recommendation for the intervention |
| --- | --- | --- | --- | --- |
| ○ | ○ | ○ | ○ | ● |

# Conclusions

| Recommendation |
| --- |
| **We strongly recommend that the diagnosis of ARDS be made in patients with acute respiratory failure (GPS).**  **Note: In addition to the diagnosis of ARDS, diagnosis and treatment of the disease causing ARDS is important to improve the prognosis.** |
|  |
| Justification |
| **Question**  Should ARDS diagnoses be conducted (for acute respiratory failure patients)?  **Patients**  Patients with acute respiratory failure  **Intervention**  Diagnose ARDS  **Comparison**  Not diagnose ARDS  **Explanation：**  There are many diseases and conditions that cause acute respiratory failure, of which ARDS is one of the most common. There are no clinical studies comparing the presence or absence of a diagnosis of ARDS in patients with acute respiratory failure, making it difficult to provide sifficient evidence for this CQ. However, the diagnosis of ARDS in patients with acute respiratory failure can lead to the provision of appropriate treatment (e.g., lung protection strategies) and improve prognosis. Therefore, a diagnosis of ARDS can be considered useful with a high degree of certainty. The panel members unanimously approved the statement as a “GPS” after a thorough discussion about (1) is the statement clear and actionable, (2) is the message really necessary, (3) is the net benefit significant and unquestionable, (4) is it difficult to collect and summarize evidence for the clinical question, (5) are there specific issues to consider (e.g., fairness), (6) is the rationale clearly presented, and (7) whether a formal GRADE-based evaluation should be done.  **Summary of evidence**：  No clinical studies have compared whether diagnosing patients with acute respiratory failure with or without ARDS directly improves patient outcomes compared to not diagnosing ARDS. For patients diagnosed with ARDS, the evidence-based recommendations presented in this guideline may improve outcomes.  **Certainty of the evidence**：  High  **Values, balance of effects, acceptability, feasibility**：  The diagnosis of ARDS allows us to make critical clinical decisions, including understanding the pathogenesis, assessing the severity of the disease, and deciding a treatment strategy. Furthermore, implementing the recommendations presented in this guideline may improve the prognosis. Therefore, the benefit of diagnosing ARDS in patients with acute respiratory failure is significant.  The diagnosis of ARDS is based on the Berlin definition proposed in 2012. The minimum diagnostic items required are a history and physical examination, as well as chest imaging (plain chest radiograph, chest CT) and arterial blood gas analysis, which may cause little harm to the patient and little additional cost to the healthcare provider.  **Panel meeting**  In a preliminary vote, the “draft recommended text” was unanimously supported by the modified Delphi method. At the panel meeting, it was suggested that the statement of intent in the recommended text be changed to make it easier for clinicians to understand. The final consensus was reached after the preliminary vote.  **Additional considerations**：  In addition to the diagnosis of ARDS, diagnosis and treatment of the disease causing ARDS is important to improve prognosis. |

| Subgroup considerations |
| --- |
| None |
| Implementation considerations |
| It is already being implemented in routine clinical practice, and there appear to be no particular problems in terms of cost, toxicity, or acceptability. |

| Monitoring and evaluation |
| --- |
| After the publication of this guideline, it is necessary to collect information on how the diagnosis of ARDS is linked to treatment decisions in clinical practice through questionnaires. |
| Research priorities |
| Appropriate treatment of patients with ARDS is known to improve their prognosis. However, no clinical studies have compared whether the diagnosis of ARDS directly improves the prognosis of patients with acute respiratory failure. Furthermore, it is ethically difficult to conduct such comparative intervention studies. |

**CQ2 Should blood brain natriuretic peptide (BNP) and NT-proBNP levels be used for identifying cardiogenic pulmonary edema as a cause of acute respiratory failure?**

1.Search strategy

MEDLINE via PubMed （Search date: 2020/6/2）

| #1 | Natriuretic Peptide, Brain[mh] OR BNP OR B type natriuretic peptide OR brain natriuretic peptide |
| --- | --- |
| #2 | Respiratory distress syndrome, adult[mh] OR shock lung OR acute respiratory distress syndrome OR Adult respiratory distress syndrome |
| #3 | ARDS OR ALI |
| #4 | Respiratory Insufficiency[mh] OR Respiratory Failure OR Respiratory Depression OR Ventilatory Depression OR Depressions, Ventilatory OR acute respiratory failure |
| #5 | Acute lung injury[mh] OR Acute lung injuries OR lung injury |
| #6 | severe acute respiratory syndrome[mh] OR severe acute respiratory syndrome |
| #7 | Pulmonary edema[mh] OR Cardiogenic pulmonary edema |
| #8 | #2 OR #3 OR #4 OR #5 OR #6 OR #7 |
| #9 | #1 AND #8 |
| #10 | animals[mh] NOT humans[mh] |
| #11 | #9 NOT #10 |

CENTRAL （Search date: 2020/6/2）

| #1 | MeSH descriptor: [Natriuretic Peptide, Brain] explode all trees |
| --- | --- |
| #2 | bnp |
| #3 | b type natriuretic peptide |
| #4 | natriuretic peptide, brain |
| #5 | #1 OR #2 OR #3 OR #4 |
| #6 | MeSH descriptor: [Respiratory Distress Syndrome, Adult] explode all trees |
| #7 | MeSH descriptor: [Respiratory Insufficiency] explode all trees |
| #8 | MeSH descriptor: [Severe Acute Respiratory Syndrome] explode all trees |
| #9 | MeSH descriptor: [Lung Injury] explode all trees |
| #10 | respiratory distress syndrome adult |
| #11 | respiratory distress syndrome acute |
| #12 | respiratory insufficiency |
| #13 | severe acute respiratory syndrome |
| #14 | lung injury |
| #15 | #6 OR #7 OR #8 OR #9 OR #10 OR #11 OR #12 OR #13 OR #14 |
| #16 | #5 and #15 |

1. Flow diagram

**Identification**

4 Studies included in qualitative synthesis

143 Full-text articles assessed for eligibility

1204 records after duplicates removed

1211 records identified through database searching

1211 records identified through database searching

Medline via PubMed (n=808)

CENTRAL (n=73)

Clinicaltrial.gov　 (n=330)

0 additional records identified through other sources

4 Studies included in quantitative synthesis

(meta-analysis)*

*3 studies and 1 study were included in the meta-analysis of BNP and NT-proBNP, respectively.

139 Full-text articles excluded, with reasons:

・Wrong language (n=14)

・Wrong study design (n=16)

・Wrong population (n=100)

・Others (n=9)

Duplicates

n=7

1061 records excluded

**Included**

**Eligibility**

**Screening**

1. Risk of bias

1. Forest plot

Index test: Blood BNP (Cutoff 400–500 pg/mL)

Index test: Blood BNP (Cutoff 1000 pg/mL)

Index test: Blood NT-proBNP (Cutoff 4000 pg/mL)

1. Evidence Profile

Index test: Blood BNP (Cutoff 400–500 pg/mL)

| | Sensitivity | 0.77 (95% CI: 0.65 to 0.85) | | --- | --- | | Specificity | 0.62 (95% CI: 0.53 to 0.70) | |  | | Prior probability | 5% | 10% | 20% | | --- | --- | --- | --- | |  |
| --- | --- | --- | --- | --- | --- | --- | --- | --- | --- | --- | --- |

| Outcome | No. of studies (patients) | Study design | Assessment of certainty | | | | | Prevalence in 1000 patients | | | Certainty of the evidence |
| --- | --- | --- | --- | --- | --- | --- | --- | --- | --- | --- | --- |
|  |  |  | Risk of bias | Indirectness | Inconsistency | Imprecision | Publication bias | Prior probability 5% | Prior probability 10% | Prior probability 20% |  |
| True Positive | 3 (252) | Cross-sectional study, Cohort study | Very serious ^a^ | Not serious ^b^ | Serious ^c^ | Very serious ^d^ | None | 38 (32 to 43) | 77 (65 to 85) | 153 (130 to 170) | ⨁◯◯◯ Very low |
| False Negative |  |  |  |  |  |  |  | 12 (7 to 18) | 23 (15 to 35) | 47 (30 to 70) |  |
| True Negative | 3 (252) | Cross-sectional study, Cohort study | Very serious ^a^ | Not serious ^b^ | Serious ^c^ | Not serious ^e^ | None | 590 (504 to 669) | 559 (478 to 634) | 497 (425 to 563) | ⨁◯◯◯ Very low |
| False Positive |  |  |  |  |  |  |  | 360 (281 to 446) | 341 (266 to 422) | 303 (237 to 375) |  |

a. Regarding the risk of bias associated with the QUADAS-2 tool, the proportion of studies judged to have high or unknown risk of bias was 1/3 for patient selection, 2/3 for index test, 0/3 for reference standard, and 2/3 for flow and timing. Thus, the proportion of studies with high or unknown risk in two or more categories was approximately more than 2/3, which was judged to be “Very serious.”

b. The applicability was associated with a low risk of bias for each study. Therefore, it was judged as being “Not serious.”

c. The variability in the results of each study included in the systematic review was visually assessed using forest plots. In three studies, although there were differences in the point estimates and confidence intervals, the cause could not be determined as subgroup or sensitivity analyses were not conducted to explain the heterogeneity. Therefore, it was judged as being “Serious.”

d. The results may be imprecise because the total number of patients included in the systematic review was below the optimal informative threshold. Additionally, we examined the net benefit of the test (difference between true positives and weighted false positives) if one false positive was considered acceptable for every 0.1 true positive. When the prevalence rate was set at 5%, the net benefit of the test differed at the upper and lower limits of the confidence interval of the integrated sensitivity, which could have changed the clinical judgment. Consequently, the overall imprecision was judged to be “Very serious.”

e. The total number of patients included in the systematic review is above the threshold for optimal information content. Additionally, the net benefit of the test (difference between true negatives and weighted false negatives) is examined if one false negative per seven true negatives is considered acceptable. When the prevalence was set at 10–20%, the net benefit of the test did not differ between the upper and lower limits of the confidence interval for the integrated specificity, and clinical judgment was not expected to change. Therefore, the overall uncertainty was judged as being “Not serious.”

Index test: Blood BNP (Cutoff 1000 pg/mL)

| | Sensitivity | 0.50 (95% CI: 0.36 to 0.64) | | --- | --- | | Specificity | 0.82 (95% CI: 0.72 to 0.89) | |  | | Prior probability | 5% | 10% | 20% | | --- | --- | --- | --- | |  |
| --- | --- | --- | --- | --- | --- | --- | --- | --- | --- | --- | --- |

| Outcome | No. of studies (patients) | Study design | Assessment of certainty | | | | | Prevalence in 1000 patients | | | Certainty of the evidence |
| --- | --- | --- | --- | --- | --- | --- | --- | --- | --- | --- | --- |
|  |  |  | Risk of bias | Indirectness | Inconsistency | Imprecision | Publication bias | Prior probability 5% | Prior probability 10% | Prior probability 20% |  |
| True Positive | 2 (128) | Cross-sectional study, Cohort study | Serious ^a^ | Not serious ^b^ | Serious ^c^ | Serious ^d^ | None | 25 (18 to 32) | 50 (36 to 64) | 100 (71 to 129) | ⨁◯◯◯ Very low |
| False Negative |  |  |  |  |  |  |  | 25 (18 to 32) | 50 (36 to 64) | 100 (71 to 129) |  |
| True Negative | 2 (128) | Cross-sectional study, Cohort study | Serious ^a^ | Not serious ^b^ | Serious ^c^ | Not serious ^e^ | None | 780 (689 to 845) | 739 (653 to 800) | 657 (580 to 711) | ⨁⨁◯◯ Low |
| False Positive |  |  |  |  |  |  |  | 170 (105 to 261) | 161 (100 to 247) | 143 (89 to 220) |  |

a. Regarding the risk of bias in the QUADAS-2 tool, the proportion of studies judged to have a high or unknown risk of bias was 1/2 for patient selection, 1/2 for index test, 0/2 for reference standard, and 1/2 for flow and timing. Thus, a certain proportion of studies was determined as being at high or unknown risk, which was judged to be “Serious.”

b. The applicability was associated with a low risk of bias for each study. Therefore, it was judged as being “Not serious.”

c. The variability in the results of each study included in the systematic review was visually assessed using forest plots. In the three studies, although there were differences in the point estimates and confidence intervals, we could not determine the cause because we could not conduct subgroup or sensitivity analyses to explain the heterogeneity. Therefore, it was judged as being “Serious.”

d. The results may be imprecise because the total number of patients included in the systematic review was below the optimal informative threshold. Additionally, the net benefit of the test (difference between true positives and weighted false positives) is examined, if one false positive was considered acceptable for every 0.2 true positive. When the prevalence rate was set at 5% and 10%, the net benefit of the test did not differ between the upper and lower limits of the confidence interval of the integrated sensitivity, which did not affect the clinical judgment. Consequently, the overall imprecision was judged to be “Serious.”

e. The total number of patients included in the systematic review was above the threshold for optimal information content. Additionally, the net benefit of the test (difference between true negatives and weighted false negatives) was examined if 10 false-negatives per one true negative was considered acceptable. When the prevalence was set at 10–20%, the net benefit of the test did not differ between the upper and lower limits of the confidence interval for the integrated specificity, and clinical judgment was not expected to change. Therefore, the overall uncertainty was judged to be “Not serious.”

Index test: Blood NT-proBNP (Cutoff 4000 pg/mL)

| | Sensitivity | 0.71 (95% CI: 0.53 to 0.85) | | --- | --- | | Specificity | 0.89 (95% CI: 0.80 to 0.94) | |  | | Prior probability | 5% | 10% | 20% | | --- | --- | --- | --- | |  |
| --- | --- | --- | --- | --- | --- | --- | --- | --- | --- | --- | --- |

| Outcome | No. of studies (patients) | Study design | Assessment of certainty | | | | | Prevalence in 1000 patients | | | Certainty of the evidence |
| --- | --- | --- | --- | --- | --- | --- | --- | --- | --- | --- | --- |
|  |  |  | Risk of bias | Indirectness | Inconsistency | Imprecision | Publication bias | Prior probability 5% | Prior probability 10% | Prior probability 20% |  |
| True Positive | 1 (121) | Cross-sectional study, Cohort study | Very serious ^a^ | Not serious ^b^ | Not serious ^c^ | Serious ^d^ | None | 36 (27 to 43) | 71 (53 to 85) | 142 (106 to 170) | ⨁◯◯◯ Very low |
| False Negative |  |  |  |  |  |  |  | 14 (7 to 23) | 29 (15 to 47) | 58 (30 to 94) |  |
| True Negative | 1 (121) | Cross-sectional study, Cohort study | Very serious ^a^ | Not serious ^b^ | Not serious ^c^ | Serious ^e^ | None | 845 (760 to 893) | 801 (720 to 846) | 712 (640 to 752) | ⨁◯◯◯ Very low |
| False Positive |  |  |  |  |  |  |  | 105 (57 to 190) | 99 (54 to 180) | 88 (48 to 160) |  |

a. Regarding the risk of bias in the QUADAS-2 tool, the domains judged to have a high risk or unknown risk were patient selection and index test. Patients fulfilling both acute respiratory distress syndrome (ARDS) and cardiogenic pulmonary edema (CPE) diagnoses were excluded, and no cutoff value for the index test was set in advance. Therefore, it was judged to be “Very serious.”

b. The applicability was associated with a risk of bias for all studies. Therefore, it was judged as being “Not serious.”

c. It was judged as being “Not serious”; however, there was only one study.

d. The results may be imprecise because the total number of patients included in the systematic review was below the optimal informative threshold. Additionally, the net benefit of the test (difference between true positives and weighted false positives) was examined, if one false positive was considered acceptable for every 0.2 true positive. When the prevalence rate was set at 5% and 10%, the net benefit of the test did not differ between the upper and lower limits of the confidence interval of the integrated sensitivity, which did not affect the clinical judgment. Consequently, the overall imprecision was judged to be “Serious.”

e. The total number of patients included in the systematic review is above the threshold for optimal information content. Additionally, the net benefit of the test (difference between true negatives and weighted false negatives) is examined if 10 false negatives per one true negative are considered acceptable. When the prevalence is set at 10–20%, the net benefit of the test does not differ between the upper and lower ends of the confidence interval for the integrated specificity, and clinical judgment is not expected to change. Therefore, the overall uncertainty was judged to be “Not serious.”

1. Evidence to Decision Table

| Question | |
| --- | --- |
| **CQ2： Should blood brain natriuretic peptide (BNP) and NT-proBNP levels be used for identifying cardiogenic pulmonary edema as the causative disease of acute respiratory failure?** | |
| **Population:** | Patients with acute respiratory failure |
| **Target condition:** | Cardiogenic pulmonary edema |
| **Index test:** | Blood BNP, blood NT-proBNP |
| **Purpose/role of the test:** | Differential diagnosis of cardiogenic pulmonary edema in acute respiratory distress syndrome (ARDS) management |
| **setting:** | Situation equivalent to emergency room (ER) or intensive care unit (ICU) |
| **Main outcomes:** | overall survival, serious adverse events from testing |
| **Medical practice based on test results:** | If positive (suspected cardiogenic pulmonary edema), the mainstay of treatment is the management of heart failure, and the adjustment of preload and afterload based on cardiac function. If negative, fluid management specific to heart failure will not be used, but will focus on identifying other causes and conditions of respiratory failure. |
| **perspective:** | Individual |
| **background:** | In the diagnostic criteria for ARDS based on the Berlin definition, “ARDS is acute respiratory failure with bilateral infiltrative shadows that cannot be explained by cardiogenic pulmonary edema.” Therefore, it is important to differentiate cardiogenic pulmonary edema in the course of ARDS treatment. BNP and NT-proBNP can be measured by blood tests and are widely used as adjunctive diagnostic tools in heart failure treatment. These peptides are secreted in response to stretch stress on the myocardium (mainly the ventricles). Approximately 10% are also secreted in the atria and are mildly elevated in patients with atrial fibrillation and renal dysfunction.  In the treatment of patients with acute respiratory failure, if the diagnosis of cardiogenic pulmonary edema is correct, appropriate use of diuretics and vasodilators can improve prognosis. If the diagnosis of cardiogenic pulmonary edema is correctly ruled out, other causes of respiratory failure should be aggressively investigated. On the contrary, misdiagnosis is detrimental because it may lead to inappropriate administration of medications or miss other causes of respiratory failure.  Therefore, the implementation of blood BNP and NT-proBNP tests has a significant impact on the treatment of patients with acute respiratory failure, and this issue was considered clinically important. Therefore, we posed the question, “Should blood brain natriuretic peptide (BNP) and NT-proBNP levels be used for identifying cardiogenic pulmonary edema as a cause of acute respiratory failure?” |
| **conflict of interest:** | None |

# Assessment

| Problem Is the problem a priority? | | |
| --- | --- | --- |
| Judgment | Research evidence | ADDITIONAL considerations |
| ● Yes  ○ Probably yes  ○ Probably no  ○ No  ○ Varies  ○ Do not know | In the treatment of patients with acute respiratory failure, if the diagnosis of cardiogenic pulmonary edema is made correctly, the prognosis can be improved with the use of appropriate diuretics and vasodilators. If the diagnosis of cardiogenic pulmonary edema is correctly ruled out, other causes of respiratory failure should be aggressively investigated. On the contrary, misdiagnosis may be detrimental because it may lead to inappropriate drug administration or miss other causes of respiratory failure.  Therefore, this issue was considered clinically important because the implementation of blood BNP and NT-proBNP tests would significantly impact the treatment of patients with acute respiratory failure. Therefore, this clinical question was considered to be of high priority. |  |
| Test accuracy How accurate is the test? | | |
| Judgment | Research evidence | ADDITIONAL considerations |
| ○ Very accurate  ○ Accurate  ○ Inaccurate  ○ Very inaccurate  ● Varies  ○ Do not know | The results of the systematic review and meta-analysis showed the following.  The accuracy of the test was judged to be “Varies” because the accuracy of the test varied depending on the cutoff of the test.  **Blood BNP**  <Cutoff 400-500 pg/mL>  (three studies, 252 patients)  Integrated sensitivity: 0.77 (95% CI: 0.65-0.85)  Integrated specificity: 0.62 (95% CI: 0.53-0.70) (Bivariate model)   | BNP | Prevalence in 1000 patients | | | Certainty of the evidence | | --- | --- | --- | --- | --- | | Prior probability | 5% | 10% | 20% |  | | True positive | 38 (32-43) | 77 (65-85) | 153 (130-170) | Very low | | False negative | 12 (7-18) | 23 (15-35) | 47 (30-70) | | True negative | 590 (504 -669) | 559 (478-634) | 497 (425-563) | Very low | | False positive | 360 (281-446) | 341 (266-422) | 303 (237-375) |   **Blood BNP**  <Cutoff 1,000 pg/mL>  (two studies, 128 patients)  Integrated sensitivity: 0.50 (95% CI: 0.36-0.64)  Integrated specificity: 0.82 (95% CI: 0.72-0.89) (Bivariate model)   | BNP | Prevalence in 1000 patients | | | Certainty of the evidence | | --- | --- | --- | --- | --- | | Prior probability | 5% | 10% | 20% |  | | True positive | 25 (18-32) | 50 (36-64) | 100 (71-129) | Very low | | False negative | 25 (18-32) | 50 (36-64) | 100 (71-129) | | True negative | 780 (689-845) | 739 (653-800) | 657 (580-711) | Low | | False positive | 170 (105-261) | 161 (100-247) | 143 (89-220) |   **Blood NT-proBNP**  <Cutoff 4,000 pg/mL>  (one study, 121 patients)  Integrated sensitivity: 0.71 (95% CI: 0.53-0.85)  Integrated specificity: 0.89 (95% CI: 0.80-0.94)   | NT-proBNP | Prevalence in 1000 patients | | | Certainty of the evidence | | --- | --- | --- | --- | --- | | Prior probability | 5% | 10% | 20% |  | | True positive | 36 (27-43) | 71 (53-85) | 142 (106- 170) | Very low | | False negative | 14 (7-23) | 29 (15-47) | 58 (30-94) | | True negative | 845 (760-893) | 801 (720-846) | 712 (640-752) | Low | | False positive | 105 (57-190) | 99 (54-180) | 88 (48-160) | |  |
| Desirable effects How substantial are the desirable anticipated effects? | | |
| Judgment | Research evidence | ADDITIONAL considerations |
| ○ Large  ○ Moderate  ○ Small  ○ Trivial  ● Varies  ○ Do not know | Since the number of true positives obtained as a desirable effect depends on the cutoff of the test, it was judged to be “Varies.”  The number of patients who would benefit from appropriate treatment in a sample of 1000 patients (the number of true positives).  **Blood BNP　<Cutoff 400-500 pg/mL>**   | Prior probability | 5% | 10% | 20% | | --- | --- | --- | --- | | Treatment based on test results | 38 | 77 | 153 | | Treatment of all patients regardless of test results | 50 | 100 | 200 | | No treatment for all patients regardless of test results | 0 | 0 | 0 |   **Blood BNP　<Cutoff 1,000 pg/mL>**   | Prior probability | 5% | 10% | 20% | | --- | --- | --- | --- | | Treatment based on test results | 25 | 50 | 100 | | Treatment of all patients regardless of test results | 50 | 100 | 200 | | No treatment for all patients regardless of test results | 0 | 0 | 0 |   **Blood NT-proBNP　<Cutoff 4,000 pg/mL>**   | Prior probability | 5% | 10% | 20% | | --- | --- | --- | --- | | Treatment based on test results | 36 | 71 | 142 | | Treatment of all patients regardless of test results | 50 | 100 | 200 | | No treatment for all patients regardless of test results | 0 | 0 | 0 | | If all patients were to be treated regardless of the test results, we considered that the number of patients who would benefit from treatment would = 1000x (pre-test probability).  If all patients were not to be treated regardless of the test results, we considered the number of patients who would benefit from the treatment to be zero. |
| Undesirable effectsHow substantial are the undesirable anticipated effects? | | |
| Judgment | Research evidence | ADDITIONAL considerations |
| ○ Large  ○ Moderate  ○ Small  ● Trivial  ○ Varies  ○ Do not know | The relative clinical weighting of false positives to true positives was set at 0.1.  After examining the number of people who would be harmed by unnecessary treatment in a sample of 1000 patients, the undesirable effect was determined to be “Trivial.”  **Blood BNP　<Cutoff 400-500 pg/mL>**   | Prior probability | 5% | 10% | 20% | | --- | --- | --- | --- | | Treatment based on test results | 36 | 34 | 30 | | Treatment of all patients regardless of test results | 95 | 90 | 80 | | No treatment for all patients regardless of test results | 0 | 0 | 0 |   **Blood BNP　<Cutoff 1,000 pg/mL>**   | Prior probability | 5% | 10% | 20% | | --- | --- | --- | --- | | Treatment based on test results | 17 | 16 | 14 | | Treatment of all patients regardless of test results | 95 | 90 | 80 | | No treatment for all patients regardless of test results | 0 | 0 | 0 |   **Blood NT-proBNP　<Cutoff 4,000 pg/mL>**   | Prior probability | 5% | 10% | 20% | | --- | --- | --- | --- | | Treatment based on test results | 10 | 10 | 9 | | Treatment of all patients regardless of test results | 95 | 90 | 80 | | No treatment for all patients regardless of test results | 0 | 0 | 0 | | If patients were treated based on test results, the number of people who would be harmed by unnecessary treatment was calculated as (number of false positives) x (clinical weighting).  If all patients were treated regardless of test results, the number of false positives was considered to be (1 - pre-test probability) x1000, and if all patients were not treated, the number was considered to be zero. The number of people who would be harmed by unnecessary treatment was calculated by multiplying these by a clinical weighting. |
| Certainty of evidence What is the overall certainty of the evidence of test accuracy? | | |
| Judgment | Research evidence | ADDITIONAL considerations |
| ● Very low  ○ Low  ○ Moderate  ○ High  ○ No included studies | The certainty of the evidence was judged to be “Very low” by adopting the certainty of the evidence with the lowest certainty. |  |
| Certainty of the evidence of test’s effects What is the overall certainty of the evidence for any critical or important direct benefits, adverse effects, or burden of the test? | | |
| Judgment | Research evidence | ADDITIONAL considerations |
| ○ Very low  ○ Low  ○ Moderate  ○ High  ● No included studies  ○ Do not know | No evidence examining the direct effects of the tests. |  |
| Certainty of evidence of management’s effects What is the overall certainty of the evidence of effects of the management that is guided by the test results? | | |
| Judgment | Research evidence | ADDITIONAL considerations |
| ○ Very low  ○ Low  ○ Moderate  ○ High  ● No included studies  ○ Do not know | In general, treatment of cardiogenic pulmonary edema with fluid management is considered “good medical practice” and is expected to improve overall survival and other outcomes. On the contrary, if patients receive unnecessary treatment due to false positives, undesirable effects (such as adverse drug events) can be expected. However, no studies have included evidence on the impact of treatment on final outcomes such as overall survival. |  |
| Certainty of evidence of test result/management How certain is the link between test results and management decisions? | | |
| Judgment | Research evidence | ADDITIONAL considerations |
| ○ Very low  ○ Low  ○ Moderate  ○ High  ● No included studies  ○ Do not know | Since treatment of cardiogenic pulmonary edema is usually initiated promptly based on the test results, it seems reasonable to assume that there is a high degree of certainty regarding the relationship between the test results and management decisions. However, no studies have been included as evidence. |  |
| Certainty of effects What is the overall certainty of the evidence of effects of the test? | | |
| Judgment | Research evidence | ADDITIONAL considerations |
| ● Very low  ○ Low  ○ Moderate  ○ High  ○ No included studies  ○ Do not know | Since the certainty of the evidence of the test’s accuracy is “Very low,” the certainty of the evidence of effects of the test becomes “Very low.” |  |
| Values Is there important uncertainty about or variability in how much people value the main outcomes? | | |
| Judgment | Research evidence | ADDITIONAL considerations |
| ○ Important uncertainty or variability  ● Possibly important uncertainty or variability  ○ Probably no important uncertainty or variability  ○ No important uncertainty or variability | The value of desirable effects, such as true positives, and undesirable effects, such as false positives and adverse events, may vary according to the values and experiences of individual healthcare providers and patients.  (The relative clinical weighting of false positives to true positives may vary depending on the values and experiences of individual health care providers and patients.) |  |
| Balance of effects Does the balance between desirable and undesirable effects favor the intervention or the comparison? | | |
| Judgment | Research evidence | ADDITIONAL considerations |
| ○ Favors the test  ● Probably favors the test  ○ Does not favor either the test or the comparison  ○ Probably favors the comparison  ○ Favors the comparison  ○ Varies  ○ Do not know | The net benefit of the test was calculated, considering the benefit of a true positive diagnosis and appropriate treatment, the harm of a false positive diagnosis and unnecessary treatment, and the adverse events of the test.  **Blood BNP**  **<Cutoff 400-500 pg/mL>**  Net benefit  (The number of patients who benefit from the test when performed on 1000 patients.)   | Prior probability | 5% | 10% | 20% | | --- | --- | --- | --- | | Treatment based on test results | 2 | 43 | 124 | | Treatment of all patients regardless of test results | -45 | 10 | 120 | | No treatment for all patients regardless of test results | 0 | 0 | 0 |   The relative clinical weight of false positives to true positives was set at 0.1, and the net benefit was calculated with a prior probability of 5-20%. Serious adverse events of the test were considered to be negligible.  We compared the net benefit of treating patients based on the test results, treating all patients without testing, and not treating any patients without testing.  Many clinical situations would likely benefit from testing.  **Blood BNP**  **<Cutoff 1,000 pg/mL>**  Net benefit  (The number of patients who benefit from the test when performed on 1000 patients.)   | Prior probability | 5% | 10% | 20% | | --- | --- | --- | --- | | Treatment based on test results | 8 | 34 | 86 | | Treatment of all patients regardless of test results | -45 | 10 | 120 | | No treatment for all patients regardless of test results | 0 | 0 | 0 |   The relative clinical weight of false positives to true positives was set at 0.1, and the net benefit was calculated with a prior probability of 5-20%. Serious adverse events of the test were considered to be negligible.  We compared the net benefit of treating patients based on the test results, treating all patients without testing, and not treating any patients without testing.  When the prior probability is assumed to be 5% or 10%, it is estimated that there is a net benefit from performing the test. However, if we assume a prior probability of 20%, it is estimated that there is a net benefit from treating all patients without testing rather than performing the test.  **Blood NT-proBNP**  **<Cutoff 4,000 pg/mL>**  Net benefit  (The number of patients who benefit from the test when performed on 1000 patients.)   | Prior probability | 5% | 10% | 20% | | --- | --- | --- | --- | | Treatment based on test results | 25 | 61 | 133 | | Treatment of all patients regardless of test results | -45 | 10 | 120 | | No treatment for all patients regardless of test results | 0 | 0 | 0 |   The relative clinical weight of false positives to true positives was set at 0.1, and the net benefit was calculated with a prior probability of 5-20%. Serious adverse events of the test were considered to be negligible.  We compared the net benefit of treating patients based on the test results, treating all patients without testing, and not treating any patients without testing.  There are likely many clinical situations that would benefit from testing.  Therefore, the testing is probably desirable. However, if the cutoff value for blood BNP is set at 1000 pg/mL and the prior prevalence is greater than 20%, it may be beneficial to treat all patients without testing. |  |
| Acceptability Is the intervention acceptable to key stakeholders? | | |
| Judgment | Research evidence | ADDITIONAL considerations |
| ● Yes  ○ Probably yes  ○ Probably no  ○ No  ○ Varies  ○ Do not know | It is a commonly practiced medical procedure and probably acceptable. |  |
| Feasibility Is the intervention feasible to implement? | | |
| Judgment | Research evidence | ADDITIONAL considerations |
| ● Yes  ○ Probably yes  ○ Probably no  ○ No  ○ Varies  ○ Do not know | It seems feasible because it is already being implemented in daily clinical practice. |  |

# Summary of Judgment

|  | **Judgment** | | | | | | |
| --- | --- | --- | --- | --- | --- | --- | --- |
| **PROBLEM** | No | Probably no | Probably yes | Yes |  | Varies | Do not know |
| **DESIRABLE EFFECTS** | Trivial | Small | Moderate | Large |  | Varies | Do not know |
| **UNDESIRABLE EFFECTS** | Large | Moderate | Small | Trivial |  | Varies | Do not know |
| **CERTAINTY OF EVIDENCE OF TEST ACCURACY** | Very low | Low | Moderate | High |  |  | No included studies |
| **CERTAINTY OF THE EVIDENCE OF TEST’S EFFECTS** | Very low | Low | Moderate | High |  |  | No included studies |
| **CERTAINTY OF THE EVIDENCE OF MANAGEMENT’S EFFECTS** | Very low | Low | Moderate | High |  |  | No included studies |
| **CERTAINTY OF THE EVIDENCE OF TEST RESULT/MANAGEMENT** | Very low | Low | Moderate | High |  |  | No included studies |
| **CERTAINTY OF EFFECT** | Very low | Low | Moderate | High |  |  | No included studies |
| **VALUES** | Important uncertainty or variability | Possibly important uncertainty or variability | Probably no important uncertainty or variability | No important uncertainty or variability |  |  |  |
| **BALANCE OF EFFECTS** | Favors the comparison | Probably favors the comparison | Does not favor either the test or the comparison | Probably favors the test | Favors the test | Varies | Do not know |
| **ACCEPTABILITY** | No | Probably no | Probably yes | Yes |  | Varies | Do not know |
| **FEASIBILITY** | No | Probably no | Probably yes | Yes |  | Varies | Do not know |

# Type of Recommendation

| Strong recommendation against the test | Conditional recommendation against the test | Conditional recommendation for either the test or the comparison | Conditional recommendation for the test | Strong recommendation for the test |
| --- | --- | --- | --- | --- |
| ○ | ○ | ○ | ● | ○ |

# Conclusions

| Recommendation |
| --- |
| **We conditionally recommend the use of blood BNP or NT-proBNP in patients with acute respiratory failure to differentiate cardiogenic pulmonary edema.**  **(Conditional recommendation/very low certainty of the evidence: GRADE: 2D)**  **Note: If the clinical situation (characteristics of the target patient, characteristics and timing of the test, prior probability, values of the patient and health care providers) changes, the balance of effects may change, and different options may be recommended.** |
|  |
| Justification |
| **Question**  Should blood brain natriuretic peptide (BNP) and NT-proBNP levels be used for identifying cardiogenic pulmonary edema as the causative disease of acute respiratory failure?  **Patients**  Patients with acute respiratory failure  **Index test**  Blood BNP, blood NT-proBNP  **Purpose, role, and setting of the test**  Differential diagnosis of cardiogenic pulmonary edema in ER, ICU, or equivalent  **Medical practice based on test results**  If positive, treatment is focused on the management of heart failure, with preload and afterload adjustments based on cardiac function being the mainstay of therapy. If negative, fluid management specific to heart failure will not be used, and more emphasis will be placed on differentiating other causes and conditions of respiratory failure.  **Summary of evidence**：  Blood BNP (Cutoff 400-500 pg/mL) (three studies, 252 patients)  Integrated sensitivity: 0.77 (95% CI: 0.65-0.85), Integrated specificity: 0.62 (95% CI: 0.53-0.70)  Blood BNP (Cutoff 1,000 pg/mL) (two studies, 128 patients)  Integrated sensitivity: 0.50 (95% CI: 0.36-0.64), Integrated specificity: 0.82 (95% CI: 0.72-0.89)  Blood NT-proBNP (Cutoff 4,000 pg/mL) (one study, 121 patients)  Integrated sensitivity: 0.71 (95% CI: 0.53-0.85), Integrated specificity: 0.89 (95% CI: 0.80-0.94)  **Certainty of the evidence**：  Very low  **Values, balance of effects, acceptability, feasibility**：  The balance of desirable and undesirable effects of BNP and NT-proBNP testing in patients with acute respiratory failure generally supports the use of these tests. On the contrary, the certainty of the evidence for the tests is “Very low” or “Low.” Feasibility is not a problem, and is a generally accepted medical practice.  **Panel meeting**  In the preliminary vote, the median score of “Draft Recommended Text” was 7, and the disagreement index by the modified Delphi method was 0.164.  At the panel meeting, there was a question about whether BNP or NT-proBNP should be measured, and it was discussed that both are useful based on the evidence. There was also discussion about the fact that insurance reimbursement does not allow both tests to be performed at the same time. In the end, a consensus was reached based on the results of the preliminary vote.  **Additional considerations**：  If the prior probability is high (around 30%), treating all patients regardless of the test result may be more beneficial than treating patients based on the test result. Moreover, the balance of effects depends on the prior probability, the clinical weighting of false positives, and the performance of the test. Therefore, if the clinical situation (characteristics of the patient, characteristics and timing of the test, prior probability, the clinical weighting of false positives, and other values held by patients and caregivers) changes, the balance of effects may change and different options may be recommended. |

| Subgroup considerations |
| --- |
| None |
| Implementation considerations |
| Both BNP and NT pro-BNP levels may be elevated in patients with atrial fibrillation, chronic heart failure, and impaired renal function. Other conditions at the time of testing should be considered when interpreting results. It should be noted that the above tests cannot be performed at the same time for insurance purposes in Japan. |

| Monitoring and evaluation |
| --- |
| After the publication of this medical guideline, it is necessary to collect and monitor information on the status of clinical use and problems in conducting the test utilizing tools such as questionnaires. |
| Research priorities |
| The evidence is less certain, and more diagnostic accuracy studies in ICU patients are warranted. If possible, randomized controlled trials are needed to evaluate whether testing improves important patient outcomes. |

**CQ3** Should serum C-reactive protein (CRP) and procalcitonin (PCT) levels be used for identifying the causative disease of ARDS (bacterial pneumonia)?

1.Search strategy

MEDLINE via PubMed （Search date: 2020/6/10）

| #1 | Pneumonia/diagnosis[Mesh] |
| --- | --- |
| #2 | C-reactive protein[MH] |
| #3 | CRP[tiab] |
| #4 | Procalcitonin[Mesh] |
| #5 | Calcitonin[Mesh] |
| #6 | psep[TIAB] |
| #7 | presepsin[TIAB] |
| #8 | presepsin protein, human [NM] |
| #9 | "soluble CD14 subtype"[tiab] |
| #10 | sCD14-ST[tiab] |
| #11 | #2 OR #3 OR #4 OR #5 OR #6 OR #7 OR #8 OR #9 OR #10 |
| #12 | #1 AND #11 |

CENTRAL （Search date: 2020/6/10）

| #1 | [mh Pneumonia] |
| --- | --- |
| #2 | [mh "C-reactive protein"] |
| #3 | CRP:ti,ab |
| #4 | [mh Procalcitonin] |
| #5 | [mh Calcitonin] |
| #6 | psep:ti,ab |
| #7 | presepsin:ti,ab |
| #8 | presepsin protein, human:kw |
| #9 | "soluble CD14 subtype":ti,ab |
| #10 | "sCD14-ST":ti,ab |
| #11 | #2 OR #3 OR #4 OR #5 OR #6 OR #7 OR #8 OR #9 OR #10 |
| #12 | #1 AND #11 |

1. Flow diagram

**Identification**

24 Studies included in qualitative synthesis

36 Full-text articles assessed for eligibility

648 records after duplicates removed

695 records identified through database searching

695 records identified through database searching

Medline via PubMed (n=583)

CENTRAL (n=112)

0 additional records identified through other sources

612 records excluded

12 Full-text articles excluded, with reasons:

・Wrong study design (n=4)

・Wrong population (n=7)

・Other (n=3)

No reply from the author

despite inquiry (n=2)

22 Studies included in quantitative synthesis (meta-analysis)

Duplicates

n=47

**Included**

**Eligibility**

**Screening**

1. Risk of bias

Serum CRP Serum PCT


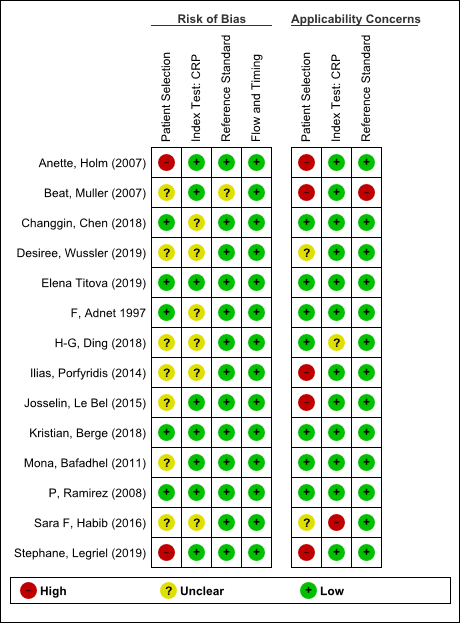

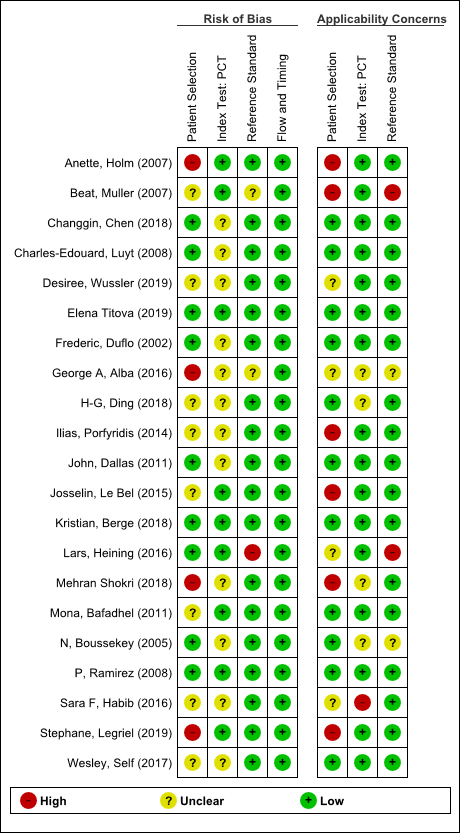


1. Forest plot

Serum CRP


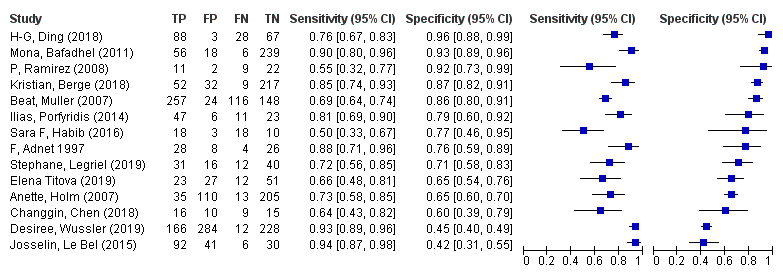


Serum PCT


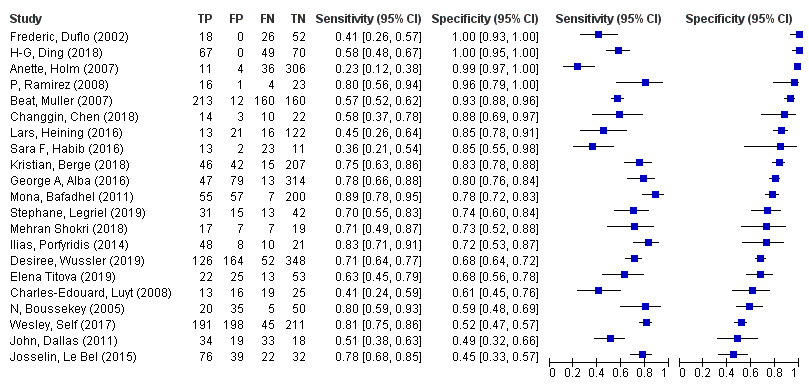


1. Evidence profile

Index test: Serum CRP

| | Sensitivity ^1^ | 0.76 (95% CI: 0.63 to 0.89) | | --- | --- | | Specificity ^1^ | 0.78 (fixed) | |  | | Prior probability | 5% | 10% | 20% | | --- | --- | --- | --- | |  |
| --- | --- | --- | --- | --- | --- | --- | --- | --- | --- | --- | --- |

| Outcome | No. of studies (patients) | Study design | Assessment of certainty | | | | | Prevalence in 1000 patients | | | Certainty of the evidence |
| --- | --- | --- | --- | --- | --- | --- | --- | --- | --- | --- | --- |
|  |  |  | Risk of bias | Indirectness | Inconsistency | Imprecision | Publication bias | Prior probability 5% | Prior probability 10% | Prior probability 20% |  |
| True Positive | 14 (3093) | Cross-sectional study, Cohort study | Serious ^a^ | Serious ^b^ | Very serious ^c^ | Serious ^d^ | None | 34 (32 to 44.5) | 76 (63 to 89) | 152 (126 to 178) | ⨁◯◯◯ Very low |
| False Negative |  |  |  |  |  |  |  | 12 (5.5 to 18) | 24 (11 to 37) | 48 (22 to 74) |  |
| True Negative | 14 (3093) | Cross-sectional study, Cohort study | Serious ^a^ | Serious ^b^ | Very serious ^c^ | None ^e^ | None | 741 | 702 | 624 | ⨁◯◯◯ Very low |
| False Positive |  |  |  |  |  |  |  | 209 | 198 | 176 |  |

1. It was calculated using the HSROC model. For sensitivity calculation, specificity was fixed at 0.765, which is the median of primary studies. For this reason, no confidence interval is given for the specificity.

a. Regarding the risk of bias in the QUADAS-2 tool, the proportion of studies judged to have a high or unknown risk of bias was 9/14 for patient selection, 6/14 for index test, 1/14 for reference standard, and 0/14 for flow and timing. Thus, a certain proportion of studies was determined to be high or unknown risk, which was judged to be “Serious.”

b. Regarding the concerns about the applicability in the QUADAS-2 tool, the proportion of studies judged to have a high or unknown concern regarding the applicability was 5/14 for patient selection, 2/14 for index test, 0/14 for reference standard. Thus, the applicability was considered to be maintained because less than one categories had a proportion of more than half of the study with high or unknown concerns about the applicability. However, in this systematic review, the literature search was conducted not only for patients with ARDS but also for those with acute respiratory failure or bacterial pneumonia (pneumonia). Owing to this gap from the ideal systematic review question that should be set, there are limitations to the direct application of the results to the clinical situation assumed by guideline readers. Therefore, the overall applicability was judged be "Serious.”

c. We visually assessed the variability in the results of each study included in the systematic review using forest plots.

d. The total number of patients included in the systematic review was above the threshold for optimal information content. Additionally, the net benefit of the test (difference between true positives and weighted false positives) was examined, if three false positives were considered acceptable for every one true positive. When the prevalence rate was set at 5% and 10%, the net benefit of the test differed, which could have changed the clinical judgment. Consequently, the overall imprecision was judged to be “Serious.”

e. The imprecision was not assessed because the specificity was fixed when estimating sensitivity using the HSROC model.

Index test: Serum PCT

| | Sensitivity ^1^ | 0.64 (95% CI: 0.56 to 0.73) | | --- | --- | | Specificity ^1^ | 0.83 (fixed) | |  | | Prior probability | 5% | 10% | 20% | | --- | --- | --- | --- | |  |
| --- | --- | --- | --- | --- | --- | --- | --- | --- | --- | --- | --- |

| Outcome | No. of studies (patients) | Study design | Assessment of certainty | | | | | Prevalence in 1000 patients | | | Certainty of the evidence |
| --- | --- | --- | --- | --- | --- | --- | --- | --- | --- | --- | --- |
|  |  |  | Risk of bias | Indirectness | Inconsistency | Imprecision | Publication bias | Prior probability 5% | Prior probability 10% | Prior probability 20% |  |
| True Positive | 21 (4721) | Cross-sectional study, Cohort study | Serious ^a^ | Serious ^b^ | Very serious ^c^ | Serious ^d^ | None | 32 (28 to 37) | 64 (56 to 73) | 128 (112 to 146) | ⨁◯◯◯ Very low |
| False Negative |  |  |  |  |  |  |  | 18 (13 to 22) | 36 (27 to 44) | 72 (54 to 88) |  |
| True Negative | 21 (4721) | Cross-sectional study, Cohort study | Serious ^a^ | Serious ^b^ | Very serious ^c^ | None ^e^ | None | 789 | 747 | 664 | ⨁◯◯◯ Very low |
| False Positive |  |  |  |  |  |  |  | 161 | 153 | 136 |  |

1. It was calculated using the hierarchical summary receiver operating characteristic (HSROC) model. For sensitivity calculation, specificity is fixed at 0.78, which is the median of primary studies. For this reason, no confidence interval is given for the specificity.

a. Regarding the risk of bias in the QUADAS-2 tool, the proportion of studies judged to have a high or unknown risk of bias was 12/21 for patient selection, 12/21 for index test, 3/21 for reference standard, and 0/21 for flow and timing. Thus, a certain proportion of studies was determined to be high or unknown risk, which was judged to be “Serious.”

b. Regarding the concerns about the applicability in the QUADAS-2 tool, the proportion of studies judged to have a high or unknown concern about the applicability was 9/21 for patient selection, 5/21 for index test, 4/21 for reference standard. Thus, the applicability was considered to be maintained because less than one category had a proportion of more than half of the study with high or unknown concerns about applicability. However, in this systematic review, we conducted a literature search not only for patients with ARDS but also for patients with acute respiratory failure or bacterial pneumonia (pneumonia). Owing to this gap from the ideal systematic review question that should be set, the results to the clinical situation assumed by guideline readers have limited direct applicability. Therefore, we judged the overall applicability to be "Serious.”

c. We visually assessed the variability in the results of each study included in the systematic review using forest plots.

d. The total number of patients included in the systematic review was above the threshold for optimal information content. Additionally, we examined the net benefit of the test (difference between true positives and weighted false positives) if three false positives were considered acceptable for every one true positive. When the prevalence rate was set at 5% and 10%, the net benefit of the test differed, which could have changed the clinical judgment. Consequently, the overall imprecision was judged to be “Serious.”

e. The imprecision was not assessed as the specificity was fixed when estimating sensitivity using the HSROC model.

1. Evidence-to-Decision table

| Question | |
| --- | --- |
| **CQ3： Should serum C-reactive protein (CRP) and procalcitonin (PCT) levels be used for identifying the causative disease of ARDS (bacterial pneumonia)?** | |
| **Population:** | Patients with ARDS or acute respiratory failure |
| **Target condition:** | Bacterial pneumonia |
| **Index test:** | Serum CRP, serum PCT |
| **Purpose/role of the test:** | Differential diagnosis of bacterial pneumonia in ARDS management |
| **setting:** | Situation equivalent to emergency room (ER) or intensive care unit (ICU) |
| **Main outcomes:** | Overall survival, serious adverse events from testing |
| **Medical practice based on test results:** | If positive (suspected bacterial pneumonia), perform further diagnostic testing and initiate treatment with appropriate antibiotic selection. If negative, avoid unnecessary antimicrobial therapy and perform additional testing and follow-up with a different diagnostic target. |
| **perspective:** | Individual |
| **background:** | Pneumonia is the most frequent cause of ARDS (about 30-60%), and bacterial pneumonia is the most common cause. CRP and PCT are proteins and peptides produced by inflammatory reactions. These are measured in the ER and ICU, and if elevated, bacterial pneumonia is suspected, and further testing and treatment are indicated. On the contrary, misdiagnosis may lead to unnecessary administration of antimicrobial agents, which may lead to the development of resistant bacteria and adverse drug reactions. In addition, inappropriately narrowing the spectrum of antimicrobial agents may lead to poor patient prognosis. Therefore, we posed the question, “Should serum CRP and PCT be used to differentiate the cause of ARDS (bacterial pneumonia)?” |
| **conflict of interest:** | None |

# Assessment

| Problem Is the problem a priority? | | |
| --- | --- | --- |
| Judgment | Research evidence | ADDITIONAL considerations |
| ● Yes  ○ Probably yes  ○ Probably no  ○ No  ○ Varies  ○ Do not know | Bacterial pneumonia may cause acute respiratory failure, and early therapeutic intervention is important. Early diagnosis by testing is expected to provide appropriate therapeutic intervention and potentially contribute to improved patient prognosis. On the contrary, misdiagnosis may result in the unnecessary administration of antimicrobial agents, which may lead to the development of resistant bacteria and adverse drug reactions. In addition, inappropriately narrowing the spectrum of antimicrobial agents may lead to poor patient prognosis. Examining the benefits and harms of testing is an important issue in ARDS care. Therefore, this clinical question was considered to be of high priority. |  |
| Test accuracy How accurate is the test? | | |
| Judgment | Research evidence | ADDITIONAL considerations |
| ○ Very accurate  ● Accurate  ○ Inaccurate  ○ Very inaccurate  ○ Varies  ○ Do not know | The results of the systematic review and meta-analysis showed the following.  The accuracy of the test was judged to be “Accurate.”  **Serum CRP**  (14 studies, 3093 patients)  Integrated sensitivity: 0.76 (95% CI: 0.63-0.89)  Integrated specificity: 0.78 (fixed)  (Estimated using the hierarchical summary receiver operating characteristic (HSROC) model, fixed at the median specificity of the primary study)   | CRP | Prevalence in 1000 patients | | | Certainty of the evidence | | --- | --- | --- | --- | --- | | Prior probability | 5% | 10% | 20% |  | | True positive | 38 (32-44.5) | 76 (63-89) | 152 (126-178) | Very low | | False negative | 12 (5.5-18) | 24 (11-37) | 48 (22-74) | | True negative | 741 | 702 | 624 | Very low | | False positive | 209 | 198 | 176 |   **Serum PCT**  (21 studies, 4721 patients)  Integrated sensitivity: 0.64 (95% CI: 0.56-0.73)  Integrated specificity: 0.83 (fixed)  (Estimated using the HSROC model, fixed at the median specificity of the primary study)   | PCT | Prevalence in 1000 patients | | | Certainty of the evidence | | --- | --- | --- | --- | --- | | Prior probability | 5% | 10% | 20% |  | | True positive | 32 (28-37) | 64 (56-73) | 128 (112-146) | Very low | | False negative | 18 (13-22) | 36 (27-44) | 72 (54-88) | | True negative | 789 | 747 | 664 | Very low | | False positive | 161 | 153 | 136 | |  |
| Desirable effects How substantial are the desirable anticipated effects? | | |
| Judgment | Research evidence | ADDITIONAL considerations |
| ○ Large  ● Moderate  ○ Small  ○ Trivial  ○ Varies  ○ Do not know | The number of patients who would benefit from appropriate treatment in a sample of 1000 patients (the number of true positives).  **Serum CRP**   | Prior probability | 5% | 10% | 20% | | --- | --- | --- | --- | | Treatment based on test results | 38 | 76 | 152 | | Treatment of all patients regardless of test results | 50 | 100 | 200 | | No treatment for all patients regardless of test results | 0 | 0 | 0 |   **Serum PCT**   | Prior probability | 5% | 10% | 20% | | --- | --- | --- | --- | | Treatment based on test results | 32 | 64 | 128 | | Treatment of all patients regardless of test results | 50 | 100 | 200 | | No treatment for all patients regardless of test results | 0 | 0 | 0 | | If all patients were to be treated regardless of the test results, the number of patients who would benefit from treatment was calculated as 1000 x (prior probability).  If all patients were not to be treated regardless of the test results, the number of patients who would benefit from the treatment was considered to be zero. |
| Undesirable effectsHow substantial are the undesirable anticipated effects? | | |
| Judgment | Research evidence | ADDITIONAL considerations |
| ○ Large  ○ Moderate  ○ Small  ● Trivial  ○ Varies  ○ Do not know | The relative clinical weighting of false positives to true positives was set at 0.05.  The number of patients who would be harmed by unnecessary treatment in a sample of 1000 patients.  **Serum CRP**   | Prior probability | 5% | 10% | 20% | | --- | --- | --- | --- | | Treatment based on test results | 10 | 10 | 9 | | Treatment of all patients regardless of test results | 48 | 45 | 40 | | No treatment for all patients regardless of test results | 0 | 0 | 0 |   **Serum PCT**   | Prior probability | 5% | 10% | 20% | | --- | --- | --- | --- | | Treatment based on test results | 8 | 8 | 7 | | Treatment of all patients regardless of test results | 48 | 45 | 40 | | No treatment for all patients regardless of test results | 0 | 0 | 0 | | If patients were treated based on test results, the number of patients who would be harmed by unnecessary treatment was calculated as (number of false positives) x (clinical weighting).  If all patients were treated regardless of the test results, the number of false positives was considered to be (1 - prior probability) x 1000, and if all patients were not treated, the number was considered to be zero. The number of patients who would be harmed by unnecessary treatment was calculated by multiplying these by the clinical weighting. |
| Certainty of evidence What is the overall certainty of the evidence of test accuracy? | | |
| Judgment | Research evidence | ADDITIONAL considerations |
| ● Very low  ○ Low  ○ Moderate  ○ High  ○ No included study | The certainty of the evidence was judged to be “Very low” by adopting the certainty of the evidence with the lowest certainty. |  |
| Certainty of the evidence of test’s effects What is the overall certainty of the evidence for any critical or important direct benefits, adverse effects, or burden of the test? | | |
| Judgment | Research evidence | ADDITIONAL considerations |
| ○ Very low  ○ Low  ○ Moderate  ○ High  ● No included study  ○ Do not know | No evidence examining the direct effects of the tests. |  |
| Certainty of evidence of management’s effects What is the overall certainty of the evidence of effects of the management that is guided by the test results? | | |
| Judgment | Research evidence | ADDITIONAL considerations |
| ○ Very low  ○ Low  ○ Moderate  ○ High  ● No included study  ○ Do not know | In general, treatments such as administration of antimicrobial agents based on true positive results are considered “good medical practice and is expected to improve life expectancy and other outcomes. On the contrary, if patients receive unnecessary treatment due to false positives, undesirable effects (such as adverse drug events) can occur. However, no studies have included evidence on the impact of treatment on final outcomes such as overall survival. |  |
| Certainty of evidence of test result/management How certain is the link between test results and management decisions? | | |
| Judgment | Research evidence | ADDITIONAL considerations |
| ○ Very low  ○ Low  ○ Moderate  ○ High  ● No included study  ○ Do not know | Since treatment of bacterial pneumonia is usually initiated promptly based on the test results, it seems reasonable to assume that there is a high degree of certainty regarding the relationship between the test results and management decisions. However, no studies have been included as evidence. |  |
| Certainty of effects What is the overall certainty of the evidence of effects of the test? | | |
| Judgment | Research evidence | ADDITIONAL considerations |
| ● Very low  ○ Low  ○ Moderate  ○ High  ○ No included study  ○ Do not know | Since the certainty of the evidence of the test’s accuracy is “Very low,” the certainty of the evidence of effects of the test becomes “Very low.” |  |
| Values Is there important uncertainty about or variability in how much people value the main outcomes? | | |
| Judgment | Research evidence | ADDITIONAL considerations |
| ○ Important uncertainty or variability  ● Possibly important uncertainty or variability  ○ Probably no important uncertainty or variability  ○ No important uncertainty or variability | The value of desirable effects such as true positives and undesirable effects such as false positives and adverse events may vary according to the values and experiences of individual healthcare providers and patients.  (The relative clinical weighting of false positives to true positives may vary depending on the values and experiences of individual health care providers and patients.) |  |
| Balance of effects Does the balance between desirable and undesirable effects favor the intervention or the comparison? | | |
| Judgment | Research evidence | ADDITIONAL considerations |
| ○ Favors the test  ○ Probably favors the test  ○ Does not favor either the test or the comparison  ● Probably favors the comparison  ○ Favors the comparison  ○ Varies  ○ Do not know | The net benefit of the test was calculated, considering the benefit of appropriate treatment due to true positive diagnosis, the harm of unnecessary treatment due to false positive diagnosis, and the adverse events of the test.  **Serum CRP**  Net benefit  (The number of patients who benefit from the test when performed on 1000 patients.)   | Prior probability | 5% | 10% | 20% | | --- | --- | --- | --- | | Treatment based on test results | 28 | 66 | 143 | | Treatment of all patients regardless of test results | 3 | 55 | 160 | | No treatment for all patients regardless of test results | 0 | 0 | 0 |   The relative clinical weight of false positives to true positives was set at 0.05, and the net benefit was calculated with a prior probability of 5-20%. Serious adverse events of the test were considered to be negligible. We compared the net benefit of treating patients based on the test results, treating all patients without testing, and not treating any patients without testing.  Clinical situations where there would be a net benefit from testing appeared to be limited, with a prior probability of 10% or less.  **Serum PCT**  Net benefit  (The number of patients who benefit from the test when performed on 1000 patients.)   | Prior probability | 5% | 10% | 20% | | --- | --- | --- | --- | | Treatment based on test results | 24 | 56 | 121 | | Treatment of all patients regardless of test results | 3 | 55 | 160 | | No treatment for all patients regardless of test results | 0 | 0 | 0 |   The relative clinical weight of false positives to true positives was set at 0.05, and the net benefit was calculated with a prior probability of 5-20%. Serious adverse events of the test were considered to be negligible. We compared the net benefit of treating patients based on the test results, treating all patients without testing, and not treating any patients without testing.  Clinical situations where there would be a net benefit from testing appeared to be limited, with a prior probability of 10% or less.  Based on these results, the balance of effects was judged to be “Probably favors the comparison.” | The net benefit (net benefit) was calculated as (number of true positives receiving appropriate treatment) - (number of false positives receiving unnecessary treatment) × (clinical weighting) - (number of serious adverse events of the test) for a sample of 1000 patients.  If serious adverse events were considered negligible, they were calculated as zero. |
| Acceptability Is the intervention acceptable to key stakeholders? | | |
| Judgment | Research evidence | ADDITIONAL considerations |
| ● Yes  ○ Probably yes  ○ Probably no  ○ No  ○ Varies  ○ Do not know | It is a commonly practiced medical procedure and probably acceptable. |  |
| Feasibility Is the intervention feasible to implement? | | |
| Judgment | Research evidence | ADDITIONAL considerations |
| ○ Yes  ● Probably yes  ○ Probably no  ○ No  ○ Varies  ○ Do not know | Some facilities may not be able to measure these tests in their own facilities, but these are commonly practiced medical procedures and probably feasible. No evidence was considered. |  |

# Summary of Judgment

|  | **Judgment** | | | | | | |
| --- | --- | --- | --- | --- | --- | --- | --- |
| **PROBLEM** | No | Probably no | Probably yes | Yes |  | Varies | Do not know |
| **DESIRABLE EFFECTS** | Trivial | Small | Moderate | Large |  | Varies | Do not know |
| **UNDESIRABLE EFFECTS** | Large | Moderate | Small | Trivial |  | Varies | Do not know |
| **CERTAINTY OF EVIDENCE OF TEST ACCURACY** | Very low | Low | Moderate | High |  |  | No included study |
| **CERTAINTY OF THE EVIDENCE OF TEST’S EFFECTS** | Very low | Low | Moderate | High |  |  | No included study |
| **CERTAINTY OF THE EVIDENCE OF MANAGEMENT’S EFFECTS** | Very low | Low | Moderate | High |  |  | No included study |
| **CERTAINTY OF THE EVIDENCE OF TEST RESULT/MANAGEMENT** | Very low | Low | Moderate | High |  |  | No included study |
| **CERTAINTY OF EFFECT** | Very low | Low | Moderate | High |  |  | No included study |
| **VALUES** | Important uncertainty or variability | Possibly important uncertainty or variability | Probably no important uncertainty or variability | No important uncertainty or variability |  |  |  |
| **BALANCE OF EFFECTS** | Favors the comparison | Probably favors the comparison | Does not favor either the test or the comparison | Probably favors the test | Favors the test | Varies | Do not know |
| **ACCEPTABILITY** | No | Probably no | Probably yes | Yes |  | Varies | Do not know |
| **FEASIBILITY** | No | Probably no | Probably yes | Yes |  | Varies | Do not know |

# Type of Recommendation

| Strong recommendation against the test | Conditional recommendation against the test | Conditional recommendation for either the test or the comparison | Conditional recommendation for the test | Strong recommendation for the test |
| --- | --- | --- | --- | --- |
| ○ | ● | ○ | ○ | ○ |

# Conclusions

| Recommendation |
| --- |
| **We conditionally recommend that the causative disease of ARDS (bacterial pneumonia) should not be differentiated based solely on the results of serum CRP and serum PCT**  **(Conditional recommendation/very low certainty of the evidence: GRADE: 2D).**  **Note: If the clinical situation (characteristics of the target patient, characteristics and timing of the test, prior probability, values of the patient and health care providers) changes, the balance of effects may change, and different options may be recommended.** |
|  |
| Justification |
| **Question**  Should serum C-reactive protein (CRP) and procalcitonin (PCT) levels be used for identifying the causative disease of ARDS (bacterial pneumonia)?  **Patients**  Patients with ARDS  **Index test**  Serum CRP, serum PCT  **Purpose, role, and setting of the test**  Differential diagnosis in ARDS management in ER, ICU, or equivalent  **Medical practice based on test results**  If positive (suspected bacterial pneumonia), perform further diagnostic tests and initiate treatment with appropriate antibiotic selection. If negative, avoid unnecessary antimicrobial therapy and perform additional testing and follow-up on a different diagnostic target.  **Summary of evidence**：  Serum CRP (14 studies, 3093 patients)  Integrated sensitivity: 0.76 (95% CI: 0.63-0.89), Specificity: 0.78 (fixed)  Serum PCT (21 studies, 4721 patients)  Integrated sensitivity: 0.64 (95% CI: 0.56-0.73), Specificity: 0.83 (fixed)  (Estimated using the HSROC model, fixed at the median specificity of the primary study).  **Certainty of the evidence**：  Very low  **Values, balance of effects, acceptability, feasibility**：  When comparing the management of diagnosing and treating bacterial pneumonia based on the results of serum CRP and serum PCT tests with the management of diagnosing and treating bacterial pneumonia regardless of the results of these tests, the latter management is likely to be beneficial in more clinical situations. We considered the adverse events of the test to be negligible. Feasibility is probably not a problem, and it is a generally accepted medical practice.  **Panel meeting**  In the preliminary vote, the median score of “recommended text proposal” was 8, and the disagreement index was 0.292 by the modified Delphi method.  In the panel meeting, there was a discussion on the expression of the recommended text. Some changes were made to the Japanese wording of the recommendation text, and agreement was finally reached with the results of the preliminary voting.  **Additional considerations**：  If the prior probability is more than 15-20%, it may be reasonable to treat the patient as having bacterial pneumonia regardless of the test results. However, in situations where bacterial pneumonia is not actively suspected with a prior probability of 10% or less, it seems reasonable to diagnose and treat bacterial pneumonia based on the serum CRP and serum PCT levels. In addition, the balance of effects depends on the prior probability, the clinical weighting of false positives, and the performance of the test. Therefore, if the clinical situation (characteristics of the patient, characteristics and timing of the test, prior probability, the clinical weighting of false positives, and other values held by patients and caregivers) changes, the balance of effects may change, and different options may be recommended. |

| Subgroup considerations |
| --- |
| None |
| Implementation considerations |
| In this meta-analysis, the integration was based on the HSROC model, and no clear cutoff was defined. |

| Monitoring and evaluation |
| --- |
| After the publication of this medical guideline, it is necessary to collect and monitor information on the status of clinical use and problems in conducting the test utilizing tools such as questionnaires. |
| Research priorities |
| The certainty of the results of this meta-analysis is very low, and further studies with greater certainty on the diagnostic performance of CRP and PCT for bacterial pneumonia are warranted. |

**CQ4 Should pneumococcal urinary antigen tests and sputum Gram staining be used for identifying pneumococcal pneumonia as the causative disease of ARDS?**

In this CQ, the results of previously published systematic reviews and meta-analyses on the diagnostic performance of Gram stain tests were reused.

Ogawa H, Kitsios GD, Iwata M, Terasawa T. Sputum Gram Stain for Bacterial Pathogen Diagnosis in Community-acquired Pneumonia: A Systematic Review and Bayesian Meta-analysis of Diagnostic Accuracy and Yield. Clin Infect Dis. 2020;71(3):499-513. doi:10.1093/cid/ciz876

1.Search strategy

Urinary antigen test

MEDLINE via PubMed （Search date: 2020/6/3）

| #1 | pneumococcal infections[mh] |
| --- | --- |
| #2 | "Pneumonia, Pneumococcal"[mh] |
| #3 | streptococcus pneumonia* [tiab] |
| #4 | #1 OR #2 OR #3 |
| #5 | "Antigens"[Mesh] |
| #6 | antigen[tiab] |
| #7 | #5 OR #6 |
| #8 | urine[mh] |
| #9 | urin*[tiab] |
| #10 | #8 OR #9 |
| #11 | #7 AND #10 |
| #12 | "Sputum"[Mesh] |
| #13 | "Blood Culture"[Mesh] |
| #14 | "Polymerase Chain Reaction"[Mesh] |
| #15 | #11 OR #12 OR #13 OR #14 |
| #16 | #4 AND #15 |
| #17 | animals[mh] NOT (animals[mh] AND humans[mh]) |
| #18 | #16 NOT #17 |

CENTRAL （Search date: 2020/6/3）

| #1 | [mh “pneumococcal infections”] |
| --- | --- |
| #2 | [mh “Pneumonia, Pneumococcal”] |
| #3 | streptococcus pneumonia*:ti,ab |
| #4 | penumococcal:ti,ab |
| #5 | #1 OR #2 OR #3 OR #4 |
| #6 | [mh Antigens] |
| #7 | antigen*:ti,ab |
| #8 | #6 OR #7 |
| #9 | [mh Urine] |
| #10 | urin*:ti,ab |
| #11 | #9 OR #10 |
| #12 | #8 AND #11 |
| #13 | #5 AND #12 |
| #14 | [mh animals] NOT ([mh animals] AND [mh humans]) |
| #15 | #13 NOT #14 |

Sputum Gram stain

See "Electronic search strategies (supplemental material)" in the literature below.

Ogawa H, Kitsios GD, Iwata M, Terasawa T. Sputum Gram Stain for Bacterial Pathogen Diagnosis in Community-acquired Pneumonia: A Systematic Review and Bayesian Meta-analysis of Diagnostic Accuracy and Yield. Clin Infect Dis. 2020;71(3):499-513. doi:10.1093/cid/ciz876

1. Flow diagram

Sputum Gram stain

See " Figure 1" in the literature below.

Ogawa H, Kitsios GD, Iwata M, Terasawa T. Sputum Gram Stain for Bacterial Pathogen Diagnosis in Community-acquired Pneumonia: A Systematic Review and Bayesian Meta-analysis of Diagnostic Accuracy and Yield. Clin Infect Dis. 2020;71(3):499-513. doi:10.1093/cid/ciz876

Urinary antigen test

**Identification**

23 Studies included in qualitative synthesis

65 Full-text articles assessed for eligibility

2150 records after duplicates removed

2179 records identified through database searching

2179 records identified through database searching

Medline via PubMed (n=2152)

CENTRAL (n=27)

0 additional records identified through other sources

23 Studies included in quantitative synthesis (meta-analysis)

42 Full-text articles excluded, with reasons:

・Wrong study design (n=13)

・Wrong publication type (n=7)

・Wrong population (n=8)

・Wrong reference test (n=2)

・Others (n=12)

Duplicates

n=29

2085 records excluded

**Included**

**Eligibility**

**Screening**

1. Risk of bias

Urinary antigen test Sputum Gram stain


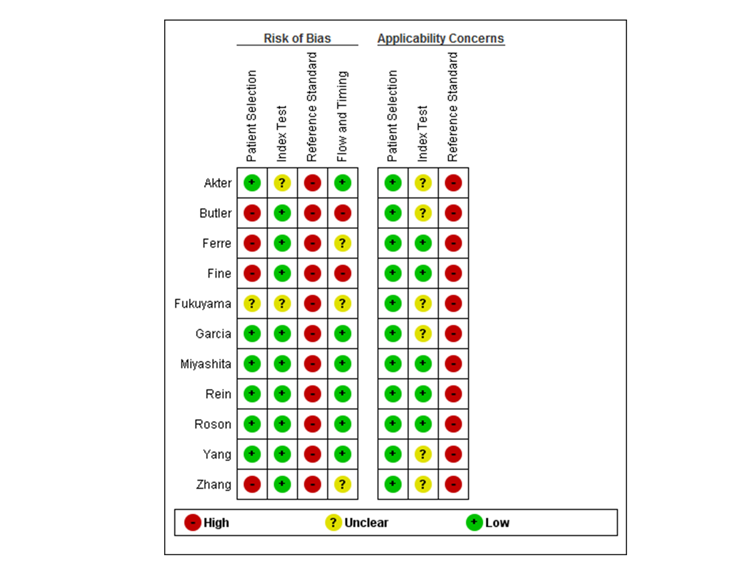


1. Forest plot

Urinary antigen test

Sputum Gram stain

See " Figure S1" in the literature below.

Ogawa H, Kitsios GD, Iwata M, Terasawa T. Sputum Gram Stain for Bacterial Pathogen Diagnosis in Community-acquired Pneumonia: A Systematic Review and Bayesian Meta-analysis of Diagnostic Accuracy and Yield. Clin Infect Dis. 2020;71(3):499-513. doi:10.1093/cid/ciz876

1. Evidence profile

Index test: Urinary antigen test

| | Sensitivity | 0.65 (95% CI: 0.61 to 0.68) | | --- | --- | | Specificity | 0.91 (95% CI: 0.85 to 0.95) | |  | | Prior probability | 5% | 10% | 20% | | --- | --- | --- | --- | |  |
| --- | --- | --- | --- | --- | --- | --- | --- | --- | --- | --- | --- |

| Outcome | No. of studies (patients) | Study design | Assessment of certainty | | | | | Prevalence in 1000 patients | | | Certainty of the evidence |
| --- | --- | --- | --- | --- | --- | --- | --- | --- | --- | --- | --- |
|  |  |  | Risk of bias | Indirectness | Inconsistency | Imprecision | Publication bias | Prior probability 5% | Prior probability 10% | Prior probability 20% |  |
| True Positive | 23 (10900) | Cross-sectional study, Cohort study | Serious ^a^ | Serious ^b^ | Serious ^c^ | Not serious ^d^ | None | 32 (30 to 34) | 65 (61 to 68) | 129 (121 to 137) | ⨁◯◯◯ Very low |
| False Negative |  |  |  |  |  |  |  | 18 (16 to 20) | 35 (32 to 39) | 71 (63 to 79) |  |
| True Negative | 23 (10900) | Cross-sectional study, Cohort study | Serious ^a^ | Serious ^b^ | Not serious ^c^ | Not serious ^e^ | None | 864 (807 to 900) | 819 (764 to 853) | 728 (679 to 758) | ⨁⨁◯◯ Low |
| False Positive |  |  |  |  |  |  |  | 86 (50 to 143) | 81 (47 to 136) | 72 (42 to 121) |  |

a. Regarding the risk of bias in the QUADAS-2 tool, the proportion of studies judged to have ahigh or unknown risk of bias was 8/23 for patient selection, 0/23 for index test, 5/23 for reference standard, and 17/23 for flow and timing. Thus, a certain proportion of studies was determined to be high or unknown risk, which was judged to be “Serious.”

b. Regarding the concerns about the applicability in the QUADAS-2 tool, the proportion of studies judged to have a high or unknown concern about the applicability was 23/23 for patient selection, 1/23 for index test, 5/23 for reference standard. Thus, the applicability was considered to be maintained because less than one category had a proportion of more than half of the study with high or unknown concerns about the applicability. However, in this systematic review, we conducted the literature search not only for patients with ARDS but also for patients with community-acquired pneumonia without respiratory failure. Owing to this gap from the ideal systematic review question that should be set, the results to the clinical situation assumed by guideline readers have limited direct applicability. Therefore, the overall applicability was judged to be "Serious.”

c. The variability in the results of each study included in the systematic review was visually assessed using forest plots.

d. The total number of patients included in the systematic review was above the threshold for optimal information content. Additionally, we examined the net benefit of the test (difference between true positives and weighted false positives) if one false positive was considered acceptable for every 0.1 true positive. When the prevalence rate was set at 5–10%, the net benefit of the test did not differ between the upper and lower limits of the confidence interval of the integrated sensitivity, and clinical judgment was not expected to change. Therefore, the overall uncertainty was judged to be “Not serious.”

e. The total number of patients included in the systematic review was above the threshold for optimal information content. Additionally, the net benefit of the test (difference between true negatives and weighted false negatives) was examined if one false positive is considered acceptable for every 0.1 true positive. When the prevalence was set at 10–20%, the net benefit of the test did not differ between the upper and lower limits of the confidence interval for the integrated specificity, and clinical judgment was not expected to change. Therefore, the overall uncertainty was judged to be “Not serious.”

Index test: Sputum Gram stain

| | Sensitivity | 0.69 (95% CI: 0.56 to 0.80) | | --- | --- | | Specificity | 0.91 (95% CI: 0.83 to 0.96) | |  | | Prior probability | 5% | 10% | 20% | | --- | --- | --- | --- | |  |
| --- | --- | --- | --- | --- | --- | --- | --- | --- | --- | --- | --- |

| Outcome | No. of studies (patients) | Study design | Assessment of certainty | | | | | Prevalence in 1000 patients | | | Certainty of the evidence |
| --- | --- | --- | --- | --- | --- | --- | --- | --- | --- | --- | --- |
|  |  |  | Risk of bias | Indirectness | Inconsistency | Imprecision | Publication bias | Prior probability 5% | Prior probability 10% | Prior probability 20% |  |
| True Positive | 11 (1794) | Cross-sectional study, Cohort study | Serious ^a^ | Very serious ^b^ | Vert serious ^c^ | Not serious ^d^ | None | 34 (28 to 40) | 69 (56 to 80) | 138 (112 to 160) | ⨁◯◯◯ Very low |
| False Negative |  |  |  |  |  |  |  | 16 (10 to 22) | 31 (20 to 44) | 62 (40 to 88) |  |
| True Negative | 11 (1794) | Cross-sectional study, Cohort study | Serious ^a^ | Very serious ^b^ | Serious ^c^ | Not serious ^e^ | None | 864 (789 to 912) | 819 (747 to 864) | 728 (664 to 768) | ⨁◯◯◯ Very low |
| False Positive |  |  |  |  |  |  |  | 86 (38 to 161) | 81 (36 to 153) | 72 (32 to 136) |  |

a. Regarding the risk of bias in the QUADAS-2 tool, the proportion of studies judged to have a high or unknown risk of bias was 4/11 for patient selection, 2/11 for index test, 11/11 for reference standard, and 5/11 for flow and timing. Thus, a certain proportion of studies had high or unknown risk, which was judged to be “Serious.”

b. Regarding the concerns about the applicability in the QUADAS-2 tool, the proportion of studies judged to have a high or unknown concern about the applicability was 0/11 for patient selection, 6/11 for index test, 11/11 for reference standard. Thus, the applicability was considered to be impaired because there were more than two categories in which the proportion of studies with high or unknown concern about the applicability was approximately more than half. Moreover, in this systematic review, we conducted the literature search not only for patients with ARDS but also for patients with community-acquired pneumonia. Because of this gap from the ideal systematic review question that should be set, the results to the clinical situation assumed by guideline readers have limited direct applicability. Therefore, we judged the overall applicability to be "Very serious.”

c. We visually assessed the variability in the results of each study included in the systematic review using forest plots.

d. The total number of patients included in the systematic review was above the threshold for optimal information content. Additionally, we examined the net benefit of the test (difference between true positives and weighted false positives) if one false positive was considered acceptable for every 0.1 true positives. When the prevalence rate was set at 5–10%, the net benefit of the test did not differ between the upper and lower limits of the confidence interval of the integrated sensitivity, and clinical judgment was not expected to change. Therefore, the overall uncertainty was judged to be “Not serious.”

e. The total number of patients included in the systematic review was above the threshold for optimal information content. Additionally, the net benefit of the test (difference between true negatives and weighted false negatives) was examined if one false positive is considered acceptable for every 0.1 true positive. When the prevalence was set at 10–20%, the net benefit of the test did not differ between the upper and lower limits of the confidence interval for the integrated specificity, and clinical judgment was not expected to change. Therefore, the overall uncertainty was judged to be “Not serious.”

1. Evidence-to-Decision table

| Question | |
| --- | --- |
| **CQ4：Should pneumococcal urinary antigen tests and sputum Gram staining be used for identifying pneumococcal pneumonia as the causative disease of ARDS?** | |
| **Population:** | Patients with ARDS or acute respiratory failure |
| **Target condition:** | Pneumococcal pneumonia |
| **Index test:** | Urinary antigen test, sputum Gram stain |
| **Purpose/role of the test:** | Differential diagnosis in ARDS management |
| **setting:** | Situation equivalent to the emergency room (ER) or intensive care unit (ICU) |
| **Main outcomes:** | Overall survival, serious adverse events from testing |
| **Medical practice based on test results:** | If positive, treatment with antimicrobial agents covering *Streptococcus pneumoniae* will be initiated. If negative, antimicrobial agents should be selected with consideration of other pathogens, and the patient should be tested and followed for other conditions that may precipitate ARDS. |
| **perspective:** | Individual |
| **background:** | Pneumococcal pneumonia can cause acute respiratory failure and lead to ARDS, and early medical intervention is important. Early diagnosis of pneumococcal pneumonia is expected to provide therapeutic intervention with appropriate antimicrobial selection and potentially contribute to improved patient prognosis. On the contrary, misdiagnosis and inappropriate selection of antimicrobial agents may worsen patient prognosis. Considering these benefits and harms, it is an important issue to examine the pros and cons of performing these tests in the management of ARDS. Therefore, we posed the question, “Should a urinary antigen test and sputum Gram stain be used to differentiate the cause of ARDS (pneumococcal pneumonia)?” |
| **conflict of interest:** | None |

# Assessment

| Problem Is the problem a priority? | | |
| --- | --- | --- |
| Judgment | Research evidence | ADDITIONAL considerations |
| ● Yes  ○ Probably yes  ○ Probably no  ○ No  ○ Varies  ○ Do not know | Pneumococcal pneumonia can cause acute respiratory failure and lead to ARDS, and early medical intervention is important. Early diagnosis of pneumococcal pneumonia is expected to provide therapeutic intervention with appropriate antimicrobial selection and potentially contribute to improved patient prognosis. On the contrary, misdiagnosis and inappropriate selection of antimicrobial agents may worsen patient prognosis. Considering these benefits and harms, it is an important issue in the management of ARDS to examine the pros and cons of performing these tests. Therefore, this clinical question was considered to be of high priority. |  |
| Test accuracy How accurate is the test? | | |
| Judgment | Research evidence | ADDITIONAL considerations |
| ○ Very accurate  ● Accurate  ○ Inaccurate  ○ Very inaccurate  ○ Varies  ○ Do not know | The results of the systematic review and meta-analysis showed the following. The accuracy of the test was judged to be “Accurate.”  **Urinary antigen test**  (23 studies, 10900 patients)  Integrated sensitivity: 0.65 (95% CI: 0.61-0.68)  Integrated specificity: 0.91 (95% CI: 0.85-0.95)   | Urinary antigen test | Prevalence in 1000 patients | | | Certainty of the evidence | | --- | --- | --- | --- | --- | | Prior probability | 5% | 10% | 20% |  | | True positive | 32 (30-34) | 65 (61-68) | 129 (121-137) | Very low | | False negative | 18 (16-20) | 35 (32-39) | 71 (63-79) | | True negative | 864 (807 -900) | 819 (764-853) | 728 (679-758) | Low | | False positive | 86 (50-143) | 81 (47-136) | 72 (42-121) |   **Sputum Gram stain**  (11 studies, 1794 patients)  Integrated sensitivity: 0.69 (95% CI: 0.56-0.80)  Integrated specificity: 0.91 (95% CI: 0.83-0.96)   | Sputum Gram stain | Prevalence in 1000 patients | | | Certainty of the evidence | | --- | --- | --- | --- | --- | | Prior probability | 5% | 10% | 20% |  | | True positive | 34 (28-40) | 69 (56-80) | 138 (112-160) | Very low | | False negative | 16 (10-22) | 31 (20-44) | 62 (40-88) | | True negative | 864 (789-912) | 819 (747-864) | 728 (664-768) | Very low | | False positive | 86 (38-161) | 81 (36-153) | 72 (32-136) | |  |
| Desirable effects How substantial are the desirable anticipated effects? | | |
| Judgment | Research evidence | ADDITIONAL considerations |
| ○ Large  ● Moderate  ○ Small  ○ Trivial  ○ Varies  ○ Do not know | The number of patients who would benefit from appropriate treatment in a sample of 1000 patients (the number of true positives). The desirable effects were judged to be “Moderate.”  **Urinary antigen test**   | Prior probability | 5% | 10% | 20% | | --- | --- | --- | --- | | Treatment based on test results | 32 | 65 | 129 | | Treatment of all patients regardless of test results | 50 | 100 | 200 | | No treatment for all patients regardless of test results | 0 | 0 | 0 |   **Sputum Gram stain**   | Prior probability | 5% | 10% | 20% | | --- | --- | --- | --- | | Treatment based on test results | 34 | 69 | 138 | | Treatment of all patients regardless of test results | 50 | 100 | 200 | | No treatment for all patients regardless of test results | 0 | 0 | 0 | | If all patients were to be treated regardless of the test results, the number of patients who would benefit from treatment was calculated as 1000 x (prior probability).  If all patients were not to be treated regardless of the test results, the number of patients who would benefit from the treatment was considered to be zero. |
| Undesirable effectsHow substantial are the undesirable anticipated effects? | | |
| Judgment | Research evidence | ADDITIONAL considerations |
| ○ Large  ○ Moderate  ○ Small  ● Trivial  ○ Varies  ○ Do not know | The relative clinical weighting of false positives to true positives was set at 0.05. The number of patients who would be harmed by unnecessary treatment in a sample of 1000 patients was calculated. The undesirable effects were judged to be “Trivial.”  **Urinary antigen test**   | Prior probability | 5% | 10% | 20% | | --- | --- | --- | --- | | Treatment based on test results | 17 | 16 | 14 | | Treatment of all patients regardless of test results | 190 | 180 | 160 | | No treatment for all patients regardless of test results | 0 | 0 | 0 |   **Sputum Gram stain**   | Prior probability | 5% | 10% | 20% | | --- | --- | --- | --- | | Treatment based on test results | 17 | 16 | 14 | | Treatment of all patients regardless of test results | 190 | 180 | 160 | | No treatment for all patients regardless of test results | 0 | 0 | 0 | | If patients were treated based on test results, the number of patients who would be harmed by unnecessary treatment was calculated as (number of false positives) x (clinical weighting).  If all patients were treated regardless of test results, the number of false positives was considered to be (1-prior probability) x 1000, and if all patients were not treated, the number was considered to be zero. The number of patients who would be harmed by unnecessary treatment was calculated by multiplying these by the clinical weighting. |
| Certainty of evidence What is the overall certainty of the evidence of test accuracy? | | |
| Judgment | Research evidence | ADDITIONAL considerations |
| ● Very low  ○ Low  ○ Moderate  ○ High  ○ No included study | The certainty of the evidence was judged to be “Very low” by adopting the certainty of the evidence with the lowest certainty. |  |
| Certainty of the evidence of test’s effects What is the overall certainty of the evidence for any critical or important direct benefits, adverse effects, or burden of the test? | | |
| Judgment | Research evidence | ADDITIONAL considerations |
| ○ Very low  ○ Low  ○ Moderate  ○ High  ● No included study  ○ Do not know | No evidence examining the direct effects of the tests. |  |
| Certainty of evidence of management’s effects What is the overall certainty of the evidence of effects of the management that is guided by the test results? | | |
| Judgment | Research evidence | ADDITIONAL considerations |
| ○ Very low  ○ Low  ○ Moderate  ○ High  ● No included study  ○ Do not know | In general, treatment of true positives with antimicrobial agents is considered “good medical practice” and is expected to improve overall survival and other outcomes. On the contrary, if patients receive unnecessary treatment due to false positives, undesirable effects (such as adverse drug events) can occur. However, no studies have included evidence on the impact of treatment on final outcomes such as overall survival. |  |
| Certainty of evidence of test result/management How certain is the link between test results and management decisions? | | |
| Judgment | Research evidence | ADDITIONAL considerations |
| ○ Very low  ○ Low  ○ Moderate  ○ High  ● No included study  ○ Do not know | Since treatment of pneumococcal pneumonia is usually initiated promptly based on the test results, it seems reasonable to assume that there is a high degree of certainty regarding the relationship between the test results and management decisions. However, no studies have been included as evidence. |  |
| Certainty of effects What is the overall certainty of the evidence of effects of the test? | | |
| Judgment | Research evidence | ADDITIONAL considerations |
| ● Very low  ○ Low  ○ Moderate  ○ High  ○ No included study  ○ Do not know | Since the certainty of the evidence of the test’s accuracy is “Very low,” the certainty of the evidence of effects of the test becomes “Very low.” |  |
| Values Is there important uncertainty about or variability in how much people value the main outcomes? | | |
| Judgment | Research evidence | ADDITIONAL considerations |
| ○ Important uncertainty or variability  ● Possibly important uncertainty or variability  ○ Probably no important uncertainty or variability  ○ No important uncertainty or variability | The value of desirable effects such as true positives and undesirable effects such as false positives and adverse events may vary according to the values and experiences of individual healthcare providers and patients.  (The relative clinical weighting of false positives to true positives may vary depending on the values and experiences of individual health care providers and patients.) |  |
| Balance of effects Does the balance between desirable and undesirable effects favor the intervention or the comparison? | | |
| Judgment | Research evidence | ADDITIONAL considerations |
| ● Favors the test  ○ Probably favors the test  ○ Does not favor either the test or the comparison  ○ Probably favors the comparison  ○ Favors the comparison  ○ Varies  ○ Do not know | The net benefit of the test was calculated, considering the benefit of appropriate treatment due to true positive diagnosis, the harm of unnecessary treatment due to false positive diagnosis, and the adverse events of the test.  **Urine antigen test**  Net benefit  (The number of patients who benefit from the test when performed on 1000 patients.)   | Prior probability | 5% | 10% | 20% | | --- | --- | --- | --- | | Treatment based on test results | 15 | 49 | 116 | | Treatment of all patients regardless of test results | -140 | -80 | 40 | | No treatment for all patients regardless of test results | 0 | 0 | 0 |   The relative clinical weight of false positives to true positives was set at 0.2, and the net benefit was calculated with a prior probability of 5-20%. Serious adverse events of the test were considered to be negligible.  **Sputum Gram stain**  Net benefit  (The number of patients who benefit from the test when performed on 1000 patients.)   | Prior probability | 5% | 10% | 20% | | --- | --- | --- | --- | | Treatment based on test results | 17 | 53 | 124 | | Treatment of all patients regardless of test results | -140 | -80 | 40 | | No treatment for all patients regardless of test results | 0 | 0 | 0 |   The relative clinical weight of false positives to true positives was set at 0.2, and the net benefit was calculated with a prior probability of 5-20%. Serious adverse events of the test were considered to be negligible.  We compared the net benefit of treating patients based on the test results, treating all patients without testing, and not treating any patients without testing. There are likely to be many clinical situations in which there would be a net benefit from testing for both tests.  Based on these results, the balance of effects was judged to be “Favors the test”.  However, in conditions where the prior probability is greater than 40%, the benefit of treating all patients regardless of the test results may outweigh the benefit of treating based on the test results. | The net benefit (net benefit) was calculated as (number of true positives receiving appropriate treatment)-(number of false positives receiving unnecessary treatment) × (clinical weighting)-(number of serious adverse events of the test) for a sample of 1000 patients.  If serious adverse events were considered negligible, they were calculated as zero. |
| Acceptability Is the intervention acceptable to key stakeholders? | | |
| Judgment | Research evidence | ADDITIONAL considerations |
| ● Yes  ○ Probably yes  ○ Probably no  ○ No  ○ Varies  ○ Do not know | It is a commonly practiced medical procedure and probably acceptable. |  |
| Feasibility Is the intervention feasible to implement? | | |
| Judgment | Research evidence | ADDITIONAL considerations |
| ○ Yes  ● Probably yes  ○ Probably no  ○ No  ○ Varies  ○ Do not know | These are commonly practiced medical procedures and are considered to be feasible. However, Gram staining needs to be performed directly by experienced medical staff. Therefore, it may be difficult to obtain results quickly, depending on the facility and time of day. |  |

# Summary of Judgment

|  | **Judgment** | | | | | | |
| --- | --- | --- | --- | --- | --- | --- | --- |
| **PROBLEM** | No | Probably no | Probably yes | Yes |  | Varies | Do not know |
| **DESIRABLE EFFECTS** | Trivial | Small | Moderate | Large |  | Varies | Do not know |
| **UNDESIRABLE EFFECTS** | Large | Moderate | Small | Trivial |  | Varies | Do not know |
| **CERTAINTY OF EVIDENCE OF TEST ACCURACY** | Very low | Low | Moderate | High |  |  | No included study |
| **CERTAINTY OF THE EVIDENCE OF TEST’S EFFECTS** | Very low | Low | Moderate | High |  |  | No included study |
| **CERTAINTY OF THE EVIDENCE OF MANAGEMENT’S EFFECTS** | Very low | Low | Moderate | High |  |  | No included study |
| **CERTAINTY OF THE EVIDENCE OF TEST RESULT/MANAGEMENT** | Very low | Low | Moderate | High |  |  | No included study |
| **CERTAINTY OF EFFECT** | Very low | Low | Moderate | High |  |  | No included study |
| **VALUES** | Important uncertainty or variability | Possibly important uncertainty or variability | Probably no important uncertainty or variability | No important uncertainty or variability |  |  |  |
| **BALANCE OF EFFECTS** | Favors the comparison | Probably favors the comparison | Does not favor either the test or the comparison | Probably favors the test | Favors the test | Varies | Do not know |
| **ACCEPTABILITY** | No | Probably no | Probably yes | Yes |  | Varies | Do not know |
| **FEASIBILITY** | No | Probably no | Probably yes | Yes |  | Varies | Do not know |

# Type of Recommendation

| Strong recommendation against the test | Conditional recommendation against the test | Conditional recommendation for either the test or the comparison | Conditional recommendation for the test | Strong recommendation for the test |
| --- | --- | --- | --- | --- |
| ○ | ○ | ○ | ● | ○ |

# Conclusions

| Recommendation |
| --- |
| **We conditionally recommend the use of a urinary antigen test and sputum Gram stain to differentiate the cause of ARDS (pneumococcal pneumonia) (Conditional recommendation/very low certainty of the evidence: GRADE: 2D).**  **Note: If the clinical situation (characteristics of the target patient, characteristics and timing of the test, prior probability, values of the patient and health care providers) changes, the balance of effects may change, and different options may be recommended.** |
|  |
| Justification |
| **Question**  Should pneumococcal urinary antigen tests and sputum Gram staining be used for identifying pneumococcal pneumonia as the causative disease of ARDS?  **Patients**  Patients with ARDS  **Index test**  Urinary antigen test, sputum Gram stain  **Purpose, role, and setting of the test**  Differential diagnosis in ARDS management in ER, ICU, or equivalent  **Medical practice based on test results**  If positive, treatment with antimicrobial agents covering *Streptococcus pneumoniae* will be initiated. If negative, antimicrobial agents should be selected with consideration of other pathogens, and the patient should be tested and followed for other conditions that may precipitate ARDS.  **Summary of evidence**：  Urinary antigen test (23 studies, 10900 patients)  Integrated sensitivity: 0.65 (95% CI: 0.61-0.68), Integrated specificity: 0.91 (95% CI: 0.85-0.95)  Sputum Gram stain (11 studies, 1794 patients)  Integrated sensitivity: 0.69 (95% CI: 0.56-0.80), Integrated specificity: 0.91 (95% CI: 0.83-0.96)  **Certainty of the evidence**：  Very low  **Values, balance of effects, acceptability, feasibility**：  The desirable effects of performing the test (prompt and appropriate treatment) are likely to be greater than the undesirable effects (adverse events, harm from false positives, and false negatives). The cost of testing is low, feasibility is probably not a problem, and it is a generally accepted medical practice.  **Panel meeting**  In the preliminary vote, the median score of “recommended text proposal” was 7, and the disagreement index was 0.164 by the modified Delphi method.  At the panel meeting, there was a discussion that implementation considerations should be described so that antimicrobial decisions and narrowing should be determined comprehensively with reference to other medical guidelines regarding infectious diseases and sepsis. An agreement was finally reached with the results of the preliminary vote.  **Additional considerations**：  For both tests, if the prior probability is above 40%, the benefit of treating all patients regardless of the test results may outweigh the benefit of treating based on the test results. In addition, the balance of effects depends on the prior probability, the clinical weighting of false positives, and the performance of the test. Therefore, if the clinical situation (characteristics of the patient, characteristics and timing of the test, prior probability, the clinical weighting of false positives, and other values held by patients and caregivers) changes, the balance of effects may change, and different options may be recommended. |

| Subgroup considerations |
| --- |
| None |
| Implementation considerations |
| Gram staining should be performed by medical staff with a certain level of skill and experience in speculum examination and determination of bacterial species. Therefore, it may be difficult to obtain results quickly depending on the facility, time of day, and other clinical conditions. In addition, when de-escalating antimicrobial agents targeting only Streptococcus pneumoniae based on test results, it is necessary to make a comprehensive decision considering the severity of the disease. For the selection of antimicrobial agents, it is advisable to refer to other guidelines, such as the Japanese Respiratory Society guidelines for the management of pneumonia in adults 2017 and The Japanese Clinical Practice Guidelines for Management of Sepsis and Septic Shock 2020. |

| Monitoring and evaluation |
| --- |
| After the publication of this medical guideline, it is necessary to collect and monitor information on the status of clinical use and problems in conducting the test using tools such as questionnaires. |
| Research priorities |
| There is a need for high-quality diagnostic accuracy studies limited to ARDS patients and, if possible, randomized controlled trials to evaluate whether testing improves important patient outcomes. |

**CQ5 Should *Legionella* urinary antigen testing be used for identifying *Legionella* pneumonia as the causative disease of ARDS?**

1.Search strategy

MEDLINE via PubMed （Search date: 2020/6/3）

| #1 | legionella [MeSH Terms] |
| --- | --- |
| #2 | legionellosis [MeSH Terms] |
| #3 | legionella [Title/Abstract] |
| #4 | legionnaire*[tiab] |
| #5 | #1 OR #2 OR #3 OR #4 |
| #6 | antigens[mh] |
| #7 | antigen[tiab] |
| #8 | #6 OR #7 |
| #9 | urine[mh] |
| #10 | urin*[tiab] |
| #11 | #9 OR #10 |
| #12 | #8 AND #11 |
| #13 | "Sputum"[Mesh] |
| #14 | "Blood Culture"[Mesh] |
| #15 | "Polymerase Chain Reaction"[Mesh] |
| #16 | # 12 OR #13 OR #14 OR #15 |
| #17 | #5 AND #16 |
| #18 | animals[mh] NOT (animals[mh] AND humans[mh]) |
| #19 | #17 NOT #18 |

CENTRAL （Search date: 2020/6/3）

| #1 | [mh legionella] |
| --- | --- |
| #2 | [mh legionellosis] |
| #3 | legionella:ti,ab |
| #4 | legionnaire*:ti,ab |
| #5 | #1 OR #2 OR #3 OR #4 |
| #6 | [mh antigens] |
| #7 | antigen*:ti,ab |
| #8 | #6 OR #7 |
| #9 | [mh Urine] |
| #10 | urin*:ti,ab |
| #11 | #9 OR #10 |
| #12 | #8 AND #11 |
| #13 | [mh Sputum] |
| #14 | [mh "Blood Culture"] |
| #15 | [mh "Polymerase Chain Reaction"] |
| #16 | #12 OR #13 OR #14 OR #15 |
| #17 | #5 AND #16 |
| #18 | [mh animals] NOT ([mh animals] AND [mh humans]) |
| #19 | #17 NOT #18 |

1. Flow diagram

**Identification**

53 Studies included in qualitative synthesis

72 Full-text articles assessed for eligibility

1326 records after duplicates removed

1334 records identified through database searching

1334 records identified through database searching

Medline via PubMed (n=1315)

CENTRAL (n=21)

0 additional records identified through other sources

21 Studies included in quantitative synthesis (meta-analysis)

19 Full-text articles excluded, with reasons:

・Wrong study design (n=11)

・Others (n=8)

Duplicates

n=8

1255 records excluded

**Included**

**Eligibility**

**Screening**

1. Risk of bias

1. Forest plot

1. Evidence profile

Index test: Urinary Legionella antigen test

| | Sensitivity | 0.79 (95% CI: 0.71 to 0.85) | | --- | --- | | Specificity | 1.00 (95% CI: 0.99 to 1.00) | |  | | Prior probability | 5% | 10% | 20% | | --- | --- | --- | --- | |  |
| --- | --- | --- | --- | --- | --- | --- | --- | --- | --- | --- | --- |

| Outcome | No. of studies (patients) | Study design | Assessment of certainty | | | | | Prevalence in 1000 patients | | | Certainty of the evidence |
| --- | --- | --- | --- | --- | --- | --- | --- | --- | --- | --- | --- |
|  |  |  | Risk of bias | Indirectness | Inconsistency | Imprecision | Publication bias | Prior probability 5% | Prior probability 10% | Prior probability 20% |  |
| True Positive | 21 (11724) | Cross-sectional study, Cohort study | Very serious ^a^ | Very serious ^b^ | Very serious ^c^ | Not serious ^d^ | None | 40 (36 to 43) | 79 (71 to 85) | 158 (142 to 170) | ⨁◯◯◯ Very low |
| False Negative |  |  |  |  |  |  |  | 10 (7 to 14) | 21 (15 to 29) | 42 (30 to 58) |  |
| True Negative | 21 (11724) | Cross-sectional study, Cohort study | Very serious ^a^ | Very serious ^b^ | Serious ^c^ | Not serious ^e^ | None | 950 (941 to 950) | 900 (891 to 900) | 800 (792 to 800) | ⨁◯◯◯ Very low |
| False Positive |  |  |  |  |  |  |  | 0 (0 to 9) | 0 (0 to 9) | 0 (0 to 8) |  |

a. Regarding risk of bias in the QUADAS-2 tool, the proportion of studies judged to have a high or unknown risk of bias was 21/21 for patient selection, 21/21 for index test, 21/21 for reference standard, and 20/21 for flow and timing. Thus, the proportion of studies with high or unknown risk in two or more categories was more than approximately 2/3, which was judged to be “Very serious.”

b. Regarding concerns about the applicability in the QUADAS-2 tool, the proportion of studies judged to have a high or unknown concern about the applicability was 17/21 for patient selection, 18/21 for index test, 0/21 for reference standard. Thus, the applicability was considered to be impaired because there were more than two categories in which the proportion of studies with high or unknown concern about the applicability was approximately more than half. Moreover, in this systematic review, we conducted the literature search not only for patients with ARDS but also for patients with suspicion of community-acquired pneumonia. Owing to this gap from the ideal systematic review question that should be set, there are limitations to the direct application of the results to the clinical situation assumed by guideline readers. Therefore, we judged the overall applicability to be "Very serious.”

c. We visually assessed the variability in the results of each study included in the systematic review using forest plots.

d. The total number of patients included in the systematic review is above the threshold for optimal information content. Additionally, we examined the net benefit of the test (difference between true positives and weighted false positives) if one false positive is considered acceptable for every 0.1 true positives. When the prevalence rate was set at 5–10%, the net benefit of the test did not differ between the upper and lower limits of the confidence interval of the integrated sensitivity, and clinical judgment was not expected to change. Therefore, the overall uncertainty was judged to be “Not serious.”

e. The total number of patients included in the systematic review was above the threshold for optimal information content. Additionally, the net benefit of the test (difference between true negatives and weighted false negatives) was examined if one false positive is considered acceptable for every 0.1 true positive. When the prevalence was set at 10–20%, the net benefit of the test did not differ between the upper and lower limits of the confidence interval for the integrated specificity, and clinical judgment was not expected to change. Therefore, the overall uncertainty was judged to be “Not serious.”

1. Evidence-to-Decision table

| Question | |
| --- | --- |
| **CQ5： Should *Legionella* urinary antigen testing be used for identifying *Legionella* pneumonia as the causative disease of ARDS?** | |
| **Population:** | Patients with ARDS or acute respiratory failure |
| **Target condition:** | Legionella pneumonia |
| **Index test:** | Urinary Legionella antigen test |
| **Purpose/role of the test:** | Differential diagnosis in ARDS management |
| **setting:** | Situation equivalent to the emergency room (ER) or intensive care unit (ICU) |
| **Main outcomes:** | Overall survival, serious adverse events from testing |
| **Medical practice based on test results:** | If positive, the patient will be diagnosed with Legionella pneumonia, and treatment with appropriate antibiotics will be initiated. If negative, unnecessary antibiotic treatment can be avoided, and additional testing and follow-up for a different diagnostic target should be performed. |
| **perspective:** | Individual |
| **background:** | Legionella pneumonia presents with acute respiratory failure and is an important cause of ARDS. If Legionella pneumonia is not treated appropriately, the outcome is likely fatal. On the contrary, appropriate antimicrobial therapy may improve the patient’s prognosis; thus, prompt diagnosis of Legionella pneumonia is important. The urine Legionella antigen test is widely used in ERs. Therefore, this issue is considered to be clinically important. Therefore, we posed the question, “Should a urinary antigen test be used to differentiate the cause of ARDS (Legionella pneumonia)?” |
| **conflict of interest:** | None |

# Assessment

| Problem Is the problem a priority? | | |
| --- | --- | --- |
| Judgment | Research evidence | ADDITIONAL considerations |
| ● Yes  ○ Probably yes  ○ Probably no  ○ No  ○ Varies  ○ Do not know | Legionella pneumonia presents with acute respiratory failure and is an important cause of ARDS. If Legionella pneumonia is not treated appropriately, the outcome is likely fatal. On the contrary, appropriate antimicrobial therapy may improve the patient’s prognosis; thus, prompt diagnosis of Legionella pneumonia is important. The urine Legionella antigen test is widely used in ERs. Therefore, this clinical question was considered to be of high priority. |  |
| Test accuracy How accurate is the test? | | |
| Judgment | Research evidence | ADDITIONAL considerations |
| ○ Very accurate  ● Accurate  ○ Inaccurate  ○ Very inaccurate  ○ Varies  ○ Do not know | The results of the systematic review and meta-analysis showed the following. The accuracy of the test was judged to be “Accurate.”  **Urinary antigen test**  (21 studies, 11724 patients)  Integrated sensitivity: 0.79 (95% CI: 0.71-0.85)  Integrated specificity: 1.00 (95% CI: 0.99-1.00)   | Urinary antigen test | Prevalence in 1000 patients | | | Certainty of the evidence | | --- | --- | --- | --- | --- | | Prior probability | 5% | 10% | 20% |  | | True positive | 40 (36-43) | 79 (71-85) | 158 (142-170) | Very low | | False negative | 10 (7-14) | 21 (15-29) | 42 (30-58) | | True negative | 950 (941-950) | 900 (891-900) | 800 (792-800) | Very low | | False positive | 0 (0-9) | 0 (0-9) | 0 (0-8) | |  |
| Desirable effects How substantial are the desirable anticipated effects? | | |
| Judgment | Research evidence | ADDITIONAL considerations |
| ○ Large  ● Moderate  ○ Small  ○ Trivial  ○ Varies  ○ Do not know | The number of patients who would benefit from appropriate treatment in a sample of 1000 patients (the number of true positives). The desirable effects were judged to be “Moderate.”  **Urinary antigen test**   | Prior probability | 5% | 10% | 20% | | --- | --- | --- | --- | | Treatment based on test results | 40 | 79 | 158 | | Treatment of all patients regardless of test results | 50 | 100 | 200 | | No treatment for all patients regardless of test results | 0 | 0 | 0 | | If all patients were to be treated regardless of the test results, the number of patients who would benefit from treatment was calculated as 1000 x (prior probability).  If all patients were not to be treated regardless of the test results, the number of patients who would benefit from the treatment was considered to be zero. |
| Undesirable effectsHow substantial are the undesirable anticipated effects? | | |
| Judgment | Research evidence | ADDITIONAL considerations |
| ○ Large  ○ Moderate  ○ Small  ● Trivial  ○ Varies  ○ Do not know | The relative clinical weighting of false positives to true positives was set at 0.1. The number of patients who would be harmed by unnecessary treatment in a sample of 1000 patients was calculated. The undesirable effects were judged to be “Trivial.”  **Urinary antigen test**   | Prior probability | 5% | 10% | 20% | | --- | --- | --- | --- | | Treatment based on test results | 0 | 0 | 0 | | Treatment of all patients regardless of test results | 95 | 90 | 80 | | No treatment for all patients regardless of test results | 0 | 0 | 0 | | The number of patients who would be harmed by unnecessary treatment was calculated as (number of false positives) x (clinical weighting).  If all patients were treated regardless of test results, the number of false positives was considered to be (1 - prior probability) x 1000.  If all patients were not treated, the number of false positives was considered to be zero. |
| Certainty of evidence What is the overall certainty of the evidence of test accuracy? | | |
| Judgment | Research evidence | ADDITIONAL considerations |
| ● Very low  ○ Low  ○ Moderate  ○ High  ○ No included study | The certainty of the evidence was judged to be “Very low” by adopting the certainty of the evidence with the lowest certainty. |  |
| Certainty of the evidence of test’s effects What is the overall certainty of the evidence for any critical or important direct benefits, adverse effects, or burden of the test? | | |
| Judgment | Research evidence | ADDITIONAL considerations |
| ○ Very low  ○ Low  ○ Moderate  ○ High  ● No included study  ○ Do not know | No evidence examining the direct effects of the tests. |  |
| Certainty of evidence of management’s effects What is the overall certainty of the evidence of effects of the management that is guided by the test results? | | |
| Judgment | Research evidence | ADDITIONAL considerations |
| ○ Very low  ○ Low  ○ Moderate  ○ High  ● No included study  ○ Do not know | In general, treatment based on the result of true positives with antimicrobial agents is considered “good medical practice” and is expected to improve overall survival and other outcomes. On the contrary, if patients receive unnecessary treatment due to false positives, undesirable effects (such as adverse drug events) can occur. However, no studies have included evidence on the impact of treatment on final outcomes such as overall survival. |  |
| Certainty of evidence of test result/management How certain is the link between test results and management decisions? | | |
| Judgment | Research evidence | ADDITIONAL considerations |
| ○ Very low  ○ Low  ○ Moderate  ○ High  ● No included study  ○ Do not know | Since treatment of Legionella pneumonia is usually initiated promptly based on the test results, it seems reasonable to assume that there is a high degree of certainty regarding the relationship between the test results and management decisions. However, no studies have been included as evidence. |  |
| Certainty of effects What is the overall certainty of the evidence of effects of the test? | | |
| Judgment | Research evidence | ADDITIONAL considerations |
| ● Very low  ○ Low  ○ Moderate  ○ High  ○ No included study  ○ Do not know | Since the certainty of the evidence of the test’s accuracy is “Very low,” the certainty of the evidence of effects of the test becomes “Very low.” |  |
| Values Is there important uncertainty about or variability in how much people value the main outcomes? | | |
| Judgment | Research evidence | ADDITIONAL considerations |
| ○ Important uncertainty or variability  ● Possibly important uncertainty or variability  ○ Probably no important uncertainty or variability  ○ No important uncertainty or variability | The value of desirable effects such as true positives and undesirable effects such as false positives and adverse events may vary according to the values and experiences of individual healthcare providers and patients.  (The relative clinical weighting of false positives to true positives may vary depending on the values and experiences of individual health care providers and patients.) |  |
| Balance of effects Does the balance between desirable and undesirable effects favor the intervention or the comparison? | | |
| Judgment | Research evidence | ADDITIONAL considerations |
| ● Favors the test  ○ Probably favors the test  ○ Does not favor either the test or the comparison  ○ Probably favors the comparison  ○ Favors the comparison  ○ Varies  ○ Do not know | The net benefit of the test was calculated, considering the benefit of appropriate treatment due to true positive diagnosis, the harm of unnecessary treatment due to false positive diagnosis, and the adverse events of the test.  **Urine antigen test**  Net benefit  (The number of patients who benefit from the test when performed on 1000 patients.)   | Prior probability | 5% | 10% | 20% | | --- | --- | --- | --- | | Treatment based on test results | 40 | 79 | 158 | | Treatment of all patients regardless of test results | -45 | 10 | 120 | | No treatment for all patients regardless of test results | 0 | 0 | 0 |   The relative clinical weight of false positives to true positives was set at 0.1, and the net benefit was calculated with a prior probability of 5-20%. Serious adverse events of the test were considered to be negligible. We compared the net benefit of treating patients based on the test results, treating all patients without testing, and not treating any patients without testing. There are likely to be many clinical situations where there would be a net benefit from testing.  Based on these results, the balance of effects was judged to be “Favors the test.” | The net benefit (net benefit) was calculated as (number of true positives receiving appropriate treatment) - (number of false positives receiving unnecessary treatment) × (clinical weighting) - (number of serious adverse events of the test) for a sample of 1000 patients.  If serious adverse events were considered negligible, they were calculated as zero. |
| Acceptability Is the intervention acceptable to key stakeholders? | | |
| Judgment | Research evidence | ADDITIONAL considerations |
| ● Yes  ○ Probably yes  ○ Probably no  ○ No  ○ Varies  ○ Do not know | It is a commonly practiced medical procedure and probably acceptable. |  |
| Feasibility Is the intervention feasible to implement? | | |
| Judgment | Research evidence | ADDITIONAL considerations |
| ● Yes  ○ Probably yes  ○ Probably no  ○ No  ○ Varies  ○ Do not know | It is a commonly practiced medical procedure and is considered to be feasible. |  |

# Summary of Judgment

|  | **Judgment** | | | | | | |
| --- | --- | --- | --- | --- | --- | --- | --- |
| **PROBLEM** | No | Probably no | Probably yes | Yes |  | Varies | Do not know |
| **DESIRABLE EFFECTS** | Trivial | Small | Moderate | Large |  | Varies | Do not know |
| **UNDESIRABLE EFFECTS** | Large | Moderate | Small | Trivial |  | Varies | Do not know |
| **CERTAINTY OF EVIDENCE OF TEST ACCURACY** | Very low | Low | Moderate | High |  |  | No included study |
| **CERTAINTY OF THE EVIDENCE OF TEST’S EFFECTS** | Very low | Low | Moderate | High |  |  | No included study |
| **CERTAINTY OF THE EVIDENCE OF MANAGEMENT’S EFFECTS** | Very low | Low | Moderate | High |  |  | No included study |
| **CERTAINTY OF THE EVIDENCE OF TEST RESULT/MANAGEMENT** | Very low | Low | Moderate | High |  |  | No included study |
| **CERTAINTY OF EFFECT** | Very low | Low | Moderate | High |  |  | No included study |
| **VALUES** | Important uncertainty or variability | Possibly important uncertainty or variability | Probably no important uncertainty or variability | No important uncertainty or variability |  |  |  |
| **BALANCE OF EFFECTS** | Favors the comparison | Probably favors the comparison | Does not favor either the test or the comparison | Probably favors the test | Favors the test | Varies | Do not know |
| **ACCEPTABILITY** | No | Probably no | Probably yes | Yes |  | Varies | Do not know |
| **FEASIBILITY** | No | Probably no | Probably yes | Yes |  | Varies | Do not know |

# Type of Recommendation

| Strong recommendation against the test | Conditional recommendation against the test | Conditional recommendation for either the test or the comparison | Conditional recommendation for the test | Strong recommendation for the test |
| --- | --- | --- | --- | --- |
| ○ | ○ | ○ | ● | ○ |

# Conclusions

| Recommendation |
| --- |
| **We conditionally recommend the use of a urinary antigen test to differentiate the cause of ARDS (Legionella pneumonia). (Conditional recommendation/very low certainty of the evidence: GRADE: 2D)**  **Note: If the clinical situation (characteristics of the target patient, characteristics and timing of the test, prior probability, values of the patient and health care providers) changes, the balance of effects may change, and different options may be recommended.** |
|  |
| Justification |
| **Question**  Should *Legionella* urinary antigen testing be used for identifying *Legionella* pneumonia as the causative disease of ARDS?  **Patients**  Patients with ARDS  **Index test**  Urinary Legionella antigen test  **Purpose, role, and setting of the test**  Differential diagnosis in ARDS management in ER, ICU, or equivalent  **Medical practice based on test results**  If positive, the patient will be diagnosed with Legionella pneumonia, and treatment with appropriate antibiotics will be initiated. If negative, unnecessary antibiotic treatment should be avoided, and additional testing and follow-up for a different diagnostic target should be performed.  **Summary of evidence**：  Urinary antigen test (21 studies, 11724 patients)  Integrated sensitivity: 0.79 (95% CI: 0.71-0.85), Integrated specificity: 1.00 (95% CI: 0.99-1.00)  **Certainty of the evidence**：  Very low  **Values, balance of effects, acceptability, feasibility**：  The desirable effect of the urine Legionella antigen test is to diagnose Legionella pneumonia and to receive appropriate treatment promptly. An undesirable effect of the test is that the patient may receive unnecessary treatment due to a false positive result. When comparing the desirable effects and harms of testing with those of deciding on a treatment plan without testing, the desirable effects of testing are considered to be greater. Feasibility is not a problem, and it is a generally accepted medical practice.  **Panel meeting**  In the preliminary vote, the median score of “recommended text proposal” was 9, and the disagreement index was 0.192 by the modified Delphi method.  At the panel meeting, the diagnostic performance of urinary antigen testing for Legionella pneumophila serotype 1 and other Legionella species was discussed and added to the implementation considerations.  **Additional considerations**：  The balance of effects depends on the prior probability, the clinical weighting of false positives, and the performance of the test. Therefore, if the clinical situation (characteristics of the patient, characteristics and timing of the test, prior probability, the clinical weighting of false positives, and other values held by patients and caregivers) changes, the balance of effects may change, and different options may be recommended. |

| Subgroup considerations |
| --- |
| None |
| Implementation considerations |
| In the studies included in this systematic review, multiple types of test kits were used: 16 results from kits for Legionella pneumophila serotype 1 only, and 20 results from kits for other serotypes and Legionella species other than Legionella pneumophila (including duplicates of multiple kits in the same study). When conducting urine antigen tests, attention should be paid to the type of test kit and the Legionella species to be tested. |

| Monitoring and evaluation |
| --- |
| After the publication of this medical guideline, it is necessary to collect and monitor information on the status of clinical use and problems in conducting the test using tools such as questionnaires. |
| Research priorities |
| Most of the studies included in the systematic review were on patients with pneumonia. Studies on the diagnostic accuracy of urinary Legionella antigen testing in patients with ARDS are needed. Research on the diagnostic accuracy of test kits, including serotypes other than type 1 and Legionella species other than Legionella pneumophila, is also required. |

**CQ6 Should antigen and PCR tests of the pharyngeal swabs and serum antibody tests be used to identify *Mycoplasma* pneumonia as the causative disease of ARDS?**

1.Search strategy

MEDLINE via PubMed （Search date: 2020/4/15）

| #1 | acute lung injury[MeSH Terms] |
| --- | --- |
| #2 | acute respiratory distress syndrome[MeSH Terms] |
| #3 | ALI[Title/Abstract] |
| #4 | ARDS[Title/Abstract] |
| #5 | acute lung injur*[Title/Abstract] |
| #6 | hypoxemic respiratory failure[Title/Abstract] |
| #7 | shock[Title/Abstract] |
| #8 | intensive care units[Title/Abstract] |
| #9 | #1 OR #2 OR #3 OR #4 OR #5 OR #6 OR #7 OR #8 |
| #10 | antigen[Title/Abstract] |
| #11 | pcr[Title/Abstract] |
| #12 | real time pcr[Title/Abstract] |
| #13 | antibody[Title/Abstract] |
| #14 | serology[Title/Abstract] |
| #15 | diagnosis[Title/Abstract] |
| #16 | #10 OR #11 OR #12 OR #13 OR #14 OR #15 |
| #17 | pneumonia, mycoplasma[MeSH Terms] |
| #18 | mycoplasma pneumon*[Title/Abstract] |
| #19 | m.pneumon*[Title/Abstract] |
| #20 | #17 OR #18 OR #19 |
| #21 | #9 AND #16 AND #20 |

1. Flow diagram

**Identification**

0 Studies included in qualitative synthesis

34 records after duplicates removed

34 records identified through database searching

34 records identified through database searching

Medline via PubMed (n=34)

0 additional records identified through other sources

0 Studies included in quantitative synthesis (meta-analysis)

34 records excluded

**Included**

**Eligibility**

**Screening**

1. Risk of bias

Not applicable

1. Forest plot

Not applicable

1. Evidence profile

Not applicable

1. Evidence-to-Decision table

| Question | |
| --- | --- |
| **CQ6： Should antigen and PCR tests of the pharyngeal swabs and serum antibody tests be used to identify *Mycoplasma* pneumonia as the causative disease of ARDS?** | |
| **Population:** | Patients with ARDS or acute respiratory failure |
| **Target condition:** | Mycoplasma pneumonia |
| **Index test:** | Antigen test (pharyngeal swab), PCR (pharyngeal swab), and serum antibody |
| **Purpose/role of the test:** | Differential diagnosis in ARDS management |
| **setting:** | Situation equivalent to the emergency room (ER) or intensive care unit (ICU) |
| **Main outcomes:** | Overall survival, serious adverse events from testing |
| **Medical practice based on test results:** | If positive (Mycoplasma pneumonia suspected), perform further diagnostic tests and initiate treatment with appropriate antimicrobial agents. If negative, avoid unnecessary antimicrobial therapy and perform additional testing and follow-up on a different diagnosis. |
| **perspective:** | Individual |
| **background:** | Mycoplasma pneumonia is a relatively common cause of community-acquired pneumonia, and although rare, it may progress to ARDS, leading to severe respiratory failure. Early diagnosis of Mycoplasma pneumonia may provide appropriate antimicrobial therapeutic intervention and potentially contribute to improved patient outcomes. On the contrary, in case of misdiagnosis, there is a possibility of harm caused by the unnecessary administration of antimicrobial agents. For the diagnosis of Mycoplasma pneumonia, an antigen test (pharyngeal swab), PCR (pharyngeal swab), and serum antibody test are commonly used. It is an important issue in the management of ARDS to examine the benefits and harms of these tests. Therefore, we posed the question, “Should an antigen test (pharyngeal swab), PCR (pharyngeal swab), and serum antibody be used to differentiate the cause of ARDS (Mycoplasma pneumonia)?” |
| **conflict of interest:** | None |

# Assessment

| Problem Is the problem a priority? | | |
| --- | --- | --- |
| Judgment | Research evidence | ADDITIONAL considerations |
| ● Yes  ○ Probably yes  ○ Probably no  ○ No  ○ Varies  ○ Do not know | Mycoplasma pneumonia is a relatively common cause of community-acquired pneumonia, and although rare, it may progress to ARDS, leading to severe respiratory failure. Early diagnosis of Mycoplasma pneumonia may provide appropriate antimicrobial therapeutic intervention and potentially contribute to improved patient outcomes. On the contrary, in case of misdiagnosis, there is a possibility of harm caused by the unnecessary administration of antimicrobial agents. For the diagnosis of Mycoplasma pneumonia, an antigen test (pharyngeal swab), PCR (pharyngeal swab), and serum antibody test are commonly used. It is an important issue in the management of ARDS to examine the benefits and harms of these tests. Therefore, this clinical question was considered to be of high priority. |  |
| Test accuracy How accurate is the test? | | |
| Judgment | Research evidence | ADDITIONAL considerations |
| ○ Very accurate  ○ Accurate  ○ Inaccurate  ○ Very inaccurate  ○ Varies  ● Do not know | A literature search did not reveal any high-quality studies reporting the diagnostic accuracy of an antigen test (pharyngeal swab), PCR (pharyngeal swab), and serum antibody test in diagnosing Mycoplasma pneumonia in patients with ARDS. Therefore, we do not know the level of accuracy of the tests.  Although not for patients with ARDS, we present the results of a diagnostic accuracy study on patients with Mycoplasma pneumonia as indirect evidence.  There are a variety of pharyngeal swab antigen test kits. Ribotest Mycoplasma (Asahi Kasei Pharma Co., Tokyo, Japan) was reported to have a sensitivity of 62.5% and a specificity of 90.9% for the diagnosis of Mycoplasma pneumonia in patients with community-acquired pneumonia when PCR testing was used as the reference standard ^1)^.  In outpatient clinics, the loop-mediated isothermal amplification (LAMP) method is often used for PCR testing of pharyngeal swabs. It has been reported that the LAMP method for pediatric Mycoplasma pneumonia has a sensitivity of 96.8% and a specificity of 100% ^2)^.  Several methods are available for testing serum mycoplasma antibodies, and the relatively widely used methods are the particulate agglutination (PA), complement binding reaction (CF), and enzyme antibody (EIA) methods. In the acute phase, immunoglobulin M (IgM) antibodies are mainly used for diagnosis. However, their sensitivity ranges from 35% to 77% and specificity from 49% to 100%, with a large variation in diagnostic accuracy depending on the time of specimen collection and the test kit ^3)^. |  |
| Desirable effects How substantial are the desirable anticipated effects? | | |
| Judgment | Research evidence | ADDITIONAL considerations |
| ○ Large  ○ Moderate  ○ Small  ○ Trivial  ○ Varies  ● Do not know | Early diagnosis of Mycoplasma pneumonia can provide appropriate antimicrobial therapeutic interventions, potentially contributing to improved patient outcomes. However, how large the desirable effect would be is not known. |  |
| Undesirable effectsHow substantial are the undesirable anticipated effects? | | |
| Judgment | Research evidence | ADDITIONAL considerations |
| ○ Large  ○ Moderate  ○ Small  ○ Trivial  ○ Varies  ● Do not know | If Mycoplasma pneumonia is misdiagnosed, there is a possibility of harm from treatment with unnecessary antimicrobials. However, how large the undesirable effect would be is not known. |  |
| Certainty of evidence What is the overall certainty of the evidence of test accuracy? | | |
| Judgment | Research evidence | ADDITIONAL considerations |
| ○ Very low  ○ Low  ○ Moderate  ○ High  ● No included study | No studies have been included as evidence of diagnostic accuracy. |  |
| Certainty of the evidence of test’s effects What is the overall certainty of the evidence for any critical or important direct benefits, adverse effects, or burden of the test? | | |
| Judgment | Research evidence | ADDITIONAL considerations |
| ○ Very low  ○ Low  ○ Moderate  ○ High  ● No included study  ○ Do not know | No evidence examining the direct effects of the tests. |  |
| Certainty of evidence of management’s effects What is the overall certainty of the evidence of effects of the management that is guided by the test results? | | |
| Judgment | Research evidence | ADDITIONAL considerations |
| ○ Very low  ○ Low  ○ Moderate  ○ High  ● No included study  ○ Do not know | In general, treatment based on the result of true positives with antimicrobial agents is considered “good medical practice” and is expected to improve overall survival and other outcomes. On the contrary, if patients receive unnecessary treatment due to false positives, undesirable effects (such as adverse drug events) can occur. However, no studies have included evidence on the impact of treatment on final outcomes such as overall survival. |  |
| Certainty of evidence of test result/management How certain is the link between test results and management decisions? | | |
| Judgment | Research evidence | ADDITIONAL considerations |
| ○ Very low  ○ Low  ○ Moderate  ○ High  ● No included study  ○ Do not know | Since treatment of Mycoplasma pneumonia is usually initiated promptly based on the test results, it seems reasonable to assume that there is a high degree of certainty regarding the relationship between the test results and management decisions. However, no studies have been included as evidence. |  |
| Certainty of effects What is the overall certainty of the evidence of effects of the test? | | |
| Judgment | Research evidence | ADDITIONAL considerations |
| ○ Very low  ○ Low  ○ Moderate  ○ High  ● No included study  ○ Do not know | No studies have been included as evidence. |  |
| Values Is there important uncertainty about or variability in how much people value the main outcomes? | | |
| Judgment | Research evidence | ADDITIONAL considerations |
| ○ Important uncertainty or variability  ● Possibly important uncertainty or variability  ○ Probably no important uncertainty or variability  ○ No important uncertainty or variability | The value of desirable effects such as true positives and undesirable effects such as false positives and adverse events may vary according to the values and experiences of individual healthcare providers and patients. |  |
| Balance of effects Does the balance between desirable and undesirable effects favor the intervention or the comparison? | | |
| Judgment | Research evidence | ADDITIONAL considerations |
| ○ Favors the test  ○ Probably favors the test  ○ Does not favor either the test or the comparison  ○ Probably favors the comparison  ○ Favors the comparison  ○ Varies  ● Do not know | The balance between the benefit of appropriate treatment due to true positive diagnosis, the harm of unnecessary treatment due to false positive diagnosis, and the adverse event of the test are not known due to the lack of evidence. |  |
| Acceptability Is the intervention acceptable to key stakeholders? | | |
| Judgment | Research evidence | ADDITIONAL considerations |
| ● Yes  ○ Probably yes  ○ Probably no  ○ No  ○ Varies  ○ Do not know | It is a commonly practiced medical procedure and probably acceptable. |  |
| Feasibility Is the intervention feasible to implement? | | |
| Judgment | Research evidence | ADDITIONAL considerations |
| ○ Yes  ● Probably yes  ○ Probably no  ○ No  ○ Varies  ○ Do not know | Some facilities may not be able to measure it in their own facilities, but it is a commonly practiced medical procedure and probably feasible. |  |

# Summary of Judgment

|  | **Judgment** | | | | | | |
| --- | --- | --- | --- | --- | --- | --- | --- |
| **PROBLEM** | No | Probably no | Probably yes | Yes |  | Varies | Do not know |
| **DESIRABLE EFFECTS** | Trivial | Small | Moderate | Large |  | Varies | Do not know |
| **UNDESIRABLE EFFECTS** | Large | Moderate | Small | Trivial |  | Varies | Do not know |
| **CERTAINTY OF EVIDENCE OF TEST ACCURACY** | Very low | Low | Moderate | High |  |  | No included study |
| **CERTAINTY OF THE EVIDENCE OF TEST’S EFFECTS** | Very low | Low | Moderate | High |  |  | No included study |
| **CERTAINTY OF THE EVIDENCE OF MANAGEMENT’S EFFECTS** | Very low | Low | Moderate | High |  |  | No included study |
| **CERTAINTY OF THE EVIDENCE OF TEST RESULT/MANAGEMENT** | Very low | Low | Moderate | High |  |  | No included study |
| **CERTAINTY OF EFFECT** | Very low | Low | Moderate | High |  |  | No included study |
| **VALUES** | Important uncertainty or variability | Possibly important uncertainty or variability | Probably no important uncertainty or variability | No important uncertainty or variability |  |  |  |
| **BALANCE OF EFFECTS** | Favors the comparison | Probably favors the comparison | Does not favor either the test or the comparison | Probably favors the test | Favors the test | Varies | Do not know |
| **ACCEPTABILITY** | No | Probably no | Probably yes | Yes |  | Varies | Do not know |
| **FEASIBILITY** | No | Probably no | Probably yes | Yes |  | Varies | Do not know |

# Type of Recommendation

| Strong recommendation against the test | Conditional recommendation against the test | Conditional recommendation for either the test or the comparison | Conditional recommendation for the test | Strong recommendation for the test |
| --- | --- | --- | --- | --- |
| ○ | ○ | ○ | ○ | ○ |

# Conclusions

| Recommendation |
| --- |
| **No specific recommendation can be made on whether an antigen test (pharyngeal swab), PCR (pharyngeal swab), and serum antibody should be used to differentiate the cause of ARDS (Mycoplasma pneumonia). These tests are currently used based on the clinician’s experience and other factors (mentioned in our practice statement).** |
|  |
| Justification |
| **Question**  Should antigen and PCR tests of the pharyngeal swabs and serum antibody tests be used to identify *Mycoplasma* pneumonia as the causative disease of ARDS?  **Patients**  Patients with ARDS  **Index test**  Antigen test (pharyngeal swab), PCR (pharyngeal swab), and serum antibody  **Purpose, role, and setting of the test**  Differential diagnosis in ARDS management in ER, ICU, or equivalent  **Explanation**  If the test result is positive, diagnose the patient with Mycoplasma pneumonia and treat with appropriate antimicrobial agents. If negative, rule out Mycoplasma pneumonia, avoid unnecessary antimicrobial agents, and perform additional testing and follow-up for alternative differential diagnoses. On the contrary, false diagnoses due to testing may lead to harm, such as the administration of unnecessary antimicrobials.  A literature search did not reveal any high-quality studies reporting the diagnostic accuracy of these tests. Although not conducted on patients with ARDS, we introduce the results of a diagnostic accuracy study on patients with Mycoplasma pneumonia as indirect evidence.  There are a variety of pharyngeal swab antigen test kits. Ribotest Mycoplasma (Asahi Kasei Pharma Co., Tokyo, Japan) was reported to have a sensitivity of 62.5% and a specificity of 90.9% for the diagnosis of Mycoplasma pneumonia in patients with community-acquired pneumonia when PCR testing was used as the reference standard ^1)^.  The LAMP method is often used in outpatient clinics for PCR testing of pharyngeal swabs. It has been reported that the LAMP method for pediatric Mycoplasma pneumonia has a sensitivity of 96.8% and a specificity of 100% ^2)^.  Several methods are available for testing serum mycoplasma antibodies, and the relatively widely used methods are the PA, complement binding reaction (CF), and enzyme antibody (EIA) methods. In the acute phase, IgM antibodies are mainly used for diagnosis, but their sensitivity ranges from 35% to 77% and specificity from 49% to 100%, with a large variation in diagnostic accuracy depending on the time of specimen collection and the test kit ^3)^.  Since there is no high-quality evidence that examines the diagnostic accuracy of these tests, it is not possible to provide a clear recommendation for this CQ. Therefore, we do not make recommendations based on evidence but only describe the current practice in this CQ.  **Summary of evidence**：  No included studies  **Certainty of the evidence**：  No included studies  **Values, balance of effects, acceptability, feasibility**：  The balance between desirable effect and harm is not known. Acceptability and feasibility will not be a problem as they are performed in daily clinical practice.  **Panel meeting**  In the preliminary vote, the median score of “recommended text proposal” was 9, and the disagreement index was 0.132 by the modified Delphi method.  At the panel meeting, there was a discussion about the Japanese wording of the recommendations, and minor revisions were made. Finally, an agreement was reached with the results of a preliminary vote.  **Additional considerations**：  None |

| Subgroup considerations |
| --- |
| None |
| Implementation considerations |
| Although no evidence was obtained for the evaluation, they are already performed in daily clinical practice and are not expected to cause any particular problems. |

| Monitoring and evaluation |
| --- |
| After the publication of this medical guideline, it is necessary to collect and monitor information on the status of clinical use and problems in conducting the test using tools such as questionnaires. |
| Research priorities |
| Diagnostic accuracy studies on this clinical question are needed. |

References

1) Miyashita N, Kawai Y, Tanaka T, Akaike H, Teranishi H, Wakabayashi T, Nakano T, Ouchi K, Okimoto N. Diagnostic sensitivity of a rapid antigen test for the detection of mycoplasma pneumoniae: comparison with real-time PCR. J Infect Chemother. 2015 Jun;21(6):473- 5. PMID: 25818195.

2) Kakuya F, Kinebuchi T, Fujiyasu H, Tanaka R, Kano H. Genetic point-of-care diagnosis of mycoplasma pneumoniae infection using LAMP assay. Pediatr Int. 2014 Aug;56(4):547-52. PMID: 24612134.

3) Beersma MF, Dirven K, van Dam AP, Templeton KE, Claas EC, Goossens H. Evaluation of 12 commercial tests and the complement fixation test for mycoplasma pneumoniae-specific immunoglobulin G (IgG) and IgM antibodies, with PCR used as the “gold standard”. J Clin Microbiol. 2005 May;43(5):2277-85. PMID: 15872256.

**CQ7 Should antigen tests of the pharyngeal/nasopharyngeal swabs and PCR tests of the bronchoalveolar lavage fluid be used for identifying influenza pneumonia as the causative disease of ARDS?**

1.Search strategy

Antigen

MEDLINE via PubMed （Search date: 2020/7/10）

| #1 | "pneumonia"[MH] |
| --- | --- |
| #2 | "influenza, human"[MH] |
| #3 | "Influenzavirus A"[MH] |
| #4 | "Influenzavirus B"[MH] |
| #5 | "Influenzavirus C"[MH] |
| #6 | #1 AND (#2 OR #3 OR #4 OR #5) |
| #7 | "antigens"[MH] OR swab*[TiAb] |
| #8 | "diagnosis"[MH] OR "diagnosis"[SH] |
| #9 | #7 AND #8 |
| #10 | #6 AND #9 |

PCR

MEDLINE via PubMed （Search date: 2020/7/10）

| #1 | "pneumonia"[MH] |
| --- | --- |
| #2 | "influenza, human"[MH] |
| #3 | "Influenzavirus A"[MH] |
| #4 | "Influenzavirus B"[MH] |
| #5 | "Influenzavirus C"[MH] |
| #6 | #1 AND (#2 OR #3 OR #4 OR #5) |
| #7 | "diagnosis"[MH] OR "diagnosis"[SH] |
| #8 | "polymerase chain reaction"[MH]) OR ("bronchoalveolar lavage fluid"[MH] |
| #9 | #7 AND #8 |
| #10 | #6 AND #9 |

1. Flow diagram

**Identification**

0 Studies included in qualitative synthesis

135 records after duplicates removed

135 records identified through database searching

135 records identified through database searching

Medline via PubMed (n=69)

CENTRAL (n=66)

0 additional records identified through other sources

0 Studies included in quantitative synthesis (meta-analysis)

135 records excluded

**Included**

**Eligibility**

**Screening**

1. Risk of bias

Not applicable

1. Forest plot

Not applicable

1. Evidence profile

Not applicable

1. Evidence-to-Decision table

| Question | |
| --- | --- |
| **CQ7： Should antigen tests of the pharyngeal/nasopharyngeal swabs and PCR tests of the bronchoalveolar lavage fluid be used for identifying influenza pneumonia as the causative disease of ARDS?** | |
| **Population:** | Patients with ARDS |
| **Target condition:** | Influenza pneumonia |
| **Index test:** | Antigen test (pharyngeal swab, nasal swab), PCR (bronchoalveolar lavage fluid) |
| **Purpose/role of the test:** | Differential diagnosis in ARDS management |
| **setting:** | Situation equivalent to the emergency room (ER) or intensive care unit (ICU) |
| **Main outcomes:** | Overall survival, serious adverse events from testing |
| **Medical practice based on test results:** | If positive (influenza pneumonia suspected), perform further diagnostic tests and initiate treatment with appropriate antiviral agents. If negative, avoid unnecessary antiviral therapy and perform additional testing and follow-up on a different diagnosis. |
| **perspective:** | Individual |
| **background:** | Although epidemiological studies are insufficient, influenza virus pneumonia has been reported to cause ARDS. Administration of neuraminidase inhibitors has been reported to reduce hospitalization, pneumonia, and mortality in influenza virus infections ^1)^. Antigen testing with rapid diagnostic kits using pharyngeal and nasal swabs is commonly used to diagnose influenza virus infections. On the contrary, there are some reports of cases of novel influenza A (H1N1) pneumonia that could not be diagnosed by antigen testing of upper airway specimens but were diagnosed by PCR testing of bronchoalveolar lavage fluid. It is important to diagnose influenza virus pneumonia in patients with ARDS using these tests and provide appropriate antiviral agents. Therefore, we posed the question, “Should an antigen test (pharyngeal swab, nasal swab) and PCR (bronchoalveolar lavage fluid) be used to differentiate the cause of ARDS (influenza pneumonia)?” |
| **conflict of interest:** | None |

# Assessment

| Problem Is the problem a priority? | | |
| --- | --- | --- |
| Judgment | Research evidence | ADDITIONAL considerations |
| ● Yes  ○ Probably yes  ○ Probably no  ○ No  ○ Varies  ○ Do not know | Although epidemiological studies are insufficient, influenza virus pneumonia has been reported to cause ARDS. The administration of neuraminidase inhibitors has been reported to reduce hospitalization, pneumonia, and mortality in influenza virus infections ^1)^. It is important to diagnose influenza virus pneumonia and provide appropriate antiviral agents. Therefore, this clinical question was considered to be of high priority. |  |
| Test accuracy How accurate is the test? | | |
| Judgment | Research evidence | ADDITIONAL considerations |
| ○ Very accurate  ○ Accurate  ○ Inaccurate  ○ Very inaccurate  ○ Varies  ● Do not know | A literature search did not reveal any high-quality studies reporting the diagnostic accuracy of an antigen test (pharyngeal swab, nasal swab), PCR (bronchoalveolar lavage fluid) in diagnosing influenza virus pneumonia in patients with ARDS. Therefore, we do not know the level of accuracy of the tests.  Although not conducted on patients with ARDS, we introduce the results of a diagnostic accuracy study on patients with influenza virus infection as indirect evidence.  A systematic review by Chartrand et al. reported a sensitivity of 62.3% (95% CI, 57.9%-66.6%) and a specificity of 98.2% (95% CI, 97.5%-98.7%) for antigen testing in the diagnosis of influenza virus infection ^2)^. Huang et al. also examined the diagnostic accuracy of various multiplex gene panel tests for influenza virus infection and reported a sensitivity of 0.940 (95% CI, 0.940-0.964) and specificity of 0.987 (95% CI, 0.979-0.992). 0.987 (95% CI, 0.979-0.992) ^3)^. |  |
| Desirable effects How substantial are the desirable anticipated effects? | | |
| Judgment | Research evidence | ADDITIONAL considerations |
| ○ Large  ○ Moderate  ○ Small  ○ Trivial  ○ Varies  ● Do not know | Early diagnosis of influenza virus pneumonia can provide appropriate antiviral therapeutic interventions, potentially contributing to improved patient outcomes. However, how large the desirable effect would be is not known. |  |
| Undesirable effectsHow substantial are the undesirable anticipated effects? | | |
| Judgment | Research evidence | ADDITIONAL considerations |
| ○ Large  ○ Moderate  ○ Small  ○ Trivial  ○ Varies  ● Do not know | If influenza virus pneumonia is misdiagnosed, there is a possibility of harm from treatment with unnecessary antiviral agents. However, how large the undesirable effect would be is not known. |  |
| Certainty of evidence What is the overall certainty of the evidence of test accuracy? | | |
| Judgment | Research evidence | ADDITIONAL considerations |
| ○ Very low  ○ Low  ○ Moderate  ○ High  ● No included study | No studies have been included as evidence of diagnostic accuracy. |  |
| Certainty of the evidence of test’s effects What is the overall certainty of the evidence for any critical or important direct benefits, adverse effects, or burden of the test? | | |
| Judgment | Research evidence | ADDITIONAL considerations |
| ○ Very low  ○ Low  ○ Moderate  ○ High  ● No included study  ○ Do not know | No evidence examining the direct effects of the tests. |  |
| Certainty of evidence of management’s effects What is the overall certainty of the evidence of effects of the management that is guided by the test results? | | |
| Judgment | Research evidence | ADDITIONAL considerations |
| ○ Very low  ○ Low  ○ Moderate  ○ High  ● No included study  ○ Do not know | In general, treatment based on the result of true positives with antiviral agents is considered “good medical practice” and is expected to improve overall survival and other outcomes. On the contrary, if patients receive unnecessary treatment due to false positives, undesirable effects (such as adverse drug events) can occur. However, no studies have included evidence on the impact of treatment on final outcomes such as overall survival. |  |
| Certainty of evidence of test result/management How certain is the link between test results and management decisions? | | |
| Judgment | Research evidence | ADDITIONAL considerations |
| ○ Very low  ○ Low  ○ Moderate  ○ High  ● No included study  ○ Do not know | Since the treatment of influenza virus pneumonia is usually initiated promptly based on the test results, it seems reasonable to assume that there is a high degree of certainty regarding the relationship between the test results and management decisions. However, no studies have been included as evidence. |  |
| Certainty of effects What is the overall certainty of the evidence of effects of the test? | | |
| Judgment | Research evidence | ADDITIONAL considerations |
| ○ Very low  ○ Low  ○ Moderate  ○ High  ● No included study  ○ Do not know | No studies have been included as evidence. |  |
| Values Is there important uncertainty about or variability in how much people value the main outcomes? | | |
| Judgment | Research evidence | ADDITIONAL considerations |
| ○ Important uncertainty or variability  ● Possibly important uncertainty or variability  ○ Probably no important uncertainty or variability  ○ No important uncertainty or variability | The value of desirable effects such as true positives and undesirable effects such as false positives and adverse events may vary according to the values and experiences of individual healthcare providers and patients. |  |
| Balance of effects Does the balance between desirable and undesirable effects favor the intervention or the comparison? | | |
| Judgment | Research evidence | ADDITIONAL considerations |
| ○ Favors the test  ○ Probably favors the test  ○ Does not favor either the test or the comparison  ○ Probably favors the comparison  ○ Favors the comparison  ○ Varies  ● Do not know | The balance between the benefit of appropriate treatment due to true positive diagnosis, the harm of unnecessary treatment due to false positive diagnosis, and the adverse event of the test are not known due to the lack of evidence. |  |
| Acceptability Is the intervention acceptable to key stakeholders? | | |
| Judgment | Research evidence | ADDITIONAL considerations |
| ● Yes  ○ Probably yes  ○ Probably no  ○ No  ○ Varies  ○ Do not know | It is a commonly practiced medical procedure and probably acceptable. |  |
| Feasibility Is the intervention feasible to implement? | | |
| Judgment | Research evidence | ADDITIONAL considerations |
| ○ Yes  ● Probably yes  ○ Probably no  ○ No  ○ Varies  ○ Do not know | Some facilities may not be able to perform bronchoalveolar lavage, but it is a commonly practiced medical procedure and probably feasible. |  |

# Summary of Judgment

|  | **Judgment** | | | | | | |
| --- | --- | --- | --- | --- | --- | --- | --- |
| **PROBLEM** | No | Probably no | Probably yes | Yes |  | Varies | Do not know |
| **DESIRABLE EFFECTS** | Trivial | Small | Moderate | Large |  | Varies | Do not know |
| **UNDESIRABLE EFFECTS** | Large | Moderate | Small | Trivial |  | Varies | Do not know |
| **CERTAINTY OF EVIDENCE OF TEST ACCURACY** | Very low | Low | Moderate | High |  |  | No included study |
| **CERTAINTY OF THE EVIDENCE OF TEST’S EFFECTS** | Very low | Low | Moderate | High |  |  | No included study |
| **CERTAINTY OF THE EVIDENCE OF MANAGEMENT’S EFFECTS** | Very low | Low | Moderate | High |  |  | No included study |
| **CERTAINTY OF THE EVIDENCE OF TEST RESULT/MANAGEMENT** | Very low | Low | Moderate | High |  |  | No included study |
| **CERTAINTY OF EFFECT** | Very low | Low | Moderate | High |  |  | No included study |
| **VALUES** | Important uncertainty or variability | Possibly important uncertainty or variability | Probably no important uncertainty or variability | No important uncertainty or variability |  |  |  |
| **BALANCE OF EFFECTS** | Favors the comparison | Probably favors the comparison | Does not favor either the test or the comparison | Probably favors the test | Favors the test | Varies | Do not know |
| **ACCEPTABILITY** | No | Probably no | Probably yes | Yes |  | Varies | Do not know |
| **FEASIBILITY** | No | Probably no | Probably yes | Yes |  | Varies | Do not know |

# Type of Recommendation

| Strong recommendation against the test | Conditional recommendation against the test | Conditional recommendation for either the test or the comparison | Conditional recommendation for the test | Strong recommendation for the test |
| --- | --- | --- | --- | --- |
| ○ | ○ | ○ | ○ | ○ |

# Conclusions

| Recommendation |
| --- |
| **No specific recommendation can be made on whether an antigen test (pharyngeal swab, nasal swab) and PCR (bronchoalveolar lavage fluid) should be used to differentiate the cause of ARDS (influenza virus pneumonia). These tests are currently used based on the clinician’s experience and other factors (mentioned in our practice statement).** |
|  |
| Justification |
| **Question**  Should antigen tests of the pharyngeal/nasopharyngeal swabs and PCR tests of the bronchoalveolar lavage fluid be used for identifying influenza pneumonia as the causative disease of ARDS?  **Patients**  Patients with ARDS  **Index test**  Antigen test (pharyngeal swab, nasal swab), PCR (bronchoalveolar lavage)  **Purpose, role, and setting of the test**  Differential diagnosis in ARDS management in ER, ICU, or equivalent  **Explanation**  If the test result is positive, diagnose the patient with influenza virus pneumonia and treat with appropriate antiviral agents. If negative, rule out influenza virus pneumonia, avoid unnecessary antiviral agents, and perform additional testing and follow-up for alternative differential diagnoses. On the contrary, false diagnoses due to testing may lead to harm, such as the administration of unnecessary antiviral agents.  We could not find any high-quality studies of the diagnostic accuracy of the above tests in patients with ARDS or influenza virus pneumonia. We present the results of diagnostic accuracy studies of common influenza virus infections as indirect evidence.  A systematic review by Chartrand et al. reported a sensitivity of 62.3% (95% CI, 57.9%-66.6%) and a specificity of 98.2% (95% CI, 97.5%-98.7%) for antigen testing in the diagnosis of influenza virus infection ^2)^. Huang et al. also examined the diagnostic accuracy of various multiplex gene panel tests for influenza virus infection and reported a sensitivity of 0.940 (95% CI, 0.940-0.964) and specificity of 0.987 (95% CI, 0.979-0.992). 0.987 (95% CI, 0.979-0.992) ^3)^.  Since there is no high-quality evidence that examines the diagnostic accuracy of these tests, it is not possible to provide a clear recommendation for this CQ. Therefore, we do not make recommendations based on evidence but only describe the current practice in this CQ.  **Summary of evidence**：  No included studies  **Certainty of the evidence**：  The certainty of the evidence cannot be assessed because there are no studies included.  **Values, balance of effects, acceptability, feasibility**：  The balance between desirable effect and harm is not known. Acceptability and feasibility will not be a problem.  **Panel meeting**  In the preliminary vote, the median score of “recommended text proposal” was 9, and the disagreement index was 0.136 by the modified Delphi method.  At the panel meeting, it was reported that no studies could be found that targeted patients with acute respiratory failure or ARDS. Finally, an agreement was reached with the results of a preliminary vote.  **Additional considerations**：  None |

| Subgroup considerations |
| --- |
| None |
| Implementation considerations |
| Although no evidence was obtained for the evaluation, they are already performed in daily clinical practice and are not expected to cause any problems. |

| Monitoring and evaluation |
| --- |
| After the publication of this medical guideline, it is necessary to collect and monitor information on the status of clinical use and problems in conducting the test using tools such as questionnaires. |
| Research priorities |
| Diagnostic accuracy studies on this clinical question are needed. |

References

1) Doll MK, Winters N, Boikos C, Kraicer-Melamed H, Gore G, Quach C. Safety and effectiveness of neuraminidase inhibitors for influenza treatment, prophylaxis, and outbreak control: a systematic review of systematic reviews and/or meta-analyses. J Antimicrob Chemother. 2017 Nov 1;72(11):2990-3007. PMID: 28961794.

2) Chartrand C, Leeflang MM, Minion J, Brewer T, Pai M. Accuracy of rapid influenza diagnostic tests: a meta-analysis. Ann Intern Med. 2012 Apr 3;156(7):500-11. PMID: 22371850.

3) Huang HS, Tsai CL, Chang J, Hsu TC, Lin S, Lee CC. Multiplex PCR system for the rapid diagnosis of respiratory virus infection: systematic review and meta-analysis. Clin Microbiol Infect. 2018 Oct;24(10):1055-1063. PMID: 29208560.

**CQ8 Should PCR tests of the bronchoalveolar lavage fluid and blood antigenemia methods be used for identifying cytomegalovirus pneumonia as the causative disease of ARDS (cytomegalovirus pneumonia)?**

In this CQ, a systematic review and meta-analysis on the diagnostic performance of the above tests and a systematic review and meta-analysis on the frequency of adverse events of bronchoalveolar lavage were conducted. Therefore, the results of multiple search formulas and systematic reviews are included.

1.Search strategy

Antigenemia

MEDLINE via PubMed　（Search date: 2020/5/26）

| #1 | Cytomegalovirus Infections/diagnosis[Mesh] |
| --- | --- |
| #2 | "Pulmonary infection"[tiab] AND "cytomegalovirus"[tiab] |
| #3 | "CMV Pneumonia"[tiab] |
| #4 | #1 OR #2 OR #3 |
| #5 | "CMV pp65"[tiab] |
| #6 | antigenaemia[tiab] OR antigenemia[tiab] |
| #7 | pp65[tiab] |
| #8 | "cytomegalovirus pp65"[tiab] |
| #9 | "pp65 antigen*"[tiab] |
| #10 | "antigenemia assay"[tiab] |
| #11 | "CMV antigenemia"[tiab] |
| #12 | "cytomegalovirus antigen*"[tiab] |
| #13 | C7HRP[tiab] |
| #14 | C10/C11[tiab] |
| #15 | #5 OR #6 OR #7 OR #8 OR #9 OR #10 OR #11 OR #12 OR #13 OR #14 |
| #16 | "polymerase chain reaction"[MeSH Terms] |
| #17 | "Cytomegalovirus/isolation and purification"[Mesh] |
| #18 | #15 OR #16 OR #17 |
| #19 | #4 AND #18 |
| #20 | animals[mh] NOT (animals[mh] AND humans[mh]) |
| #21 | #19 NOT #20 |

CENTRAL （Search date: 2020/5/26）

| #1 | [mh "Cytomegalovirus Infections"] |
| --- | --- |
| #2 | "Pulmonary infection":ti,ab AND cytomegalovirus:ti,ab |
| #3 | "CMV Pneumonia":ti,ab |
| #4 | #1 OR #2 OR #3 |
| #5 | "CMV pp65":ti,ab |
| #6 | antigenaemia:ti,ab OR antigenemia:ti,ab |
| #7 | pp65:ti,ab |
| #8 | "cytomegalovirus pp65":ti,ab |
| #9 | pp65 NEXT antigen*:ti,ab |
| #10 | "antigenemia assay":ti,ab |
| #11 | "CMV antigenemia":ti,ab |
| #12 | cytomegalovirus NEXT antigen*:ti,ab |
| #13 | C7HRP:ti,ab |
| #14 | "C10/C11":ti,ab |
| #15 | #5 OR #6 OR #7 OR #8 OR #9 OR #10 OR #11 OR #12 OR #13 OR #14 |
| #16 | [mh "polymerase chain reaction"] |
| #17 | [mh Cytomegalovirus/IP] |
| #18 | #15 OR #16 OR #17 |
| #19 | #4 AND #18 |
| #20 | [mh animals] NOT ([mh animals] AND [mh humans]) |
| #21 | #19 NOT #20 |

BAL

MEDLINE via PubMed （Search date: 2020/6/1）

| #1 | Respiratory Distress Syndrome, Adult [mh] |
| --- | --- |
| #2 | Acute lung injury [mh] |
| #3 | ALI [tiab] OR ARDS [tiab] |
| #4 | Acute [tiab] AND (lung injur* [tiab] OR respiratory distress [tiab] OR respiratory failure[tiab]) |
| #5 | (Severe [tiab] OR critical*[tiab]) AND (respiratory[tiab] OR hypox* [tiab]) |
| #6 | #1 or #2 or #3 or #4 or #5 |
| #7 | Bronchoscopy/ adverse effects [mh] OR Bronchoscopy/ complications [mh] |
| #8 | Bronchoscop* [tiab] |
| #9 | Bronchoalveolar Lavage/ adverse effects [mh] OR Bronchoalveolar Lavage/ complications [mh] |
| #10 | Bronchoalveolar Lavage [tiab] |
| #11 | BAL [tiab] OR BALF [tiab] |
| #12 | #7 or #8 or #9 or #10 or #11 |
| #13 | #6 and #12 |
| #14 | Animals [mh] NOT human [mh] |
| #15 | #13 NOT #14 |

CENTRAL （Search date: 2020/6/1）

| #1 | MeSH descriptor: [Respiratory Distress Syndrome, Adult] explode all trees |
| --- | --- |
| #2 | MeSH descriptor: [Acute Lung Injury] explode all trees |
| #3 | (ALI OR ARDS):ti,ab,kw |
| #4 | (Acute):ti,ab,kw |
| #5 | (lung NEXT injur*):ti,ab,kw |
| #6 | (“respiratory distress”):ti,ab,kw |
| #7 | (“respiratory failure”):ti,ab,kw |
| #8 | #4 AND (#5 OR #6 OR #7) |
| #9 | (Severe:ti,ab OR critical*:ti,ab) AND (respiratory:ti,ab OR hypox*:ti,ab) |
| #10 | #1 OR #2 OR #3 OR #8 OR #9 |
| #11 | MeSH descriptor: [Bronchoscopy] explode all trees |
| #12 | (Bronchoscop*):ti,ab,kw |
| #13 | MeSH descriptor: [Bronchoalveolar Lavage] explode all trees |
| #14 | (“Bronchoalveolar Lavage”):ti,ab,kw |
| #15 | (BAL OR BALF):ti,ab,kw |
| #16 | #11 OR #12 OR #13 OR #14 OR #15 |
| #17 | #10 AND #16 |
| #18 | ([mh Animals] NOT [mh human]):ti,ab,kw |
| #19 | #17 NOT #18 |

1. Flow diagram

CMV PCR and CMV antigenemia

**Identification**

8 Studies included in qualitative synthesis

135 Full-text articles assessed for eligibility

2499 records after duplicates removed

2501 records identified through database searching

2501 records identified through database searching

Medline via PubMed (n=2479)

CENTRAL (n=22)

0 additional records identified through other sources

8 Studies included in quantitative synthesis

(meta-analysis)*

*5 and 3 studies were included in the meta-analysis of CMV PCR and CMV antigenemia, respectively.

127 Full-text articles excluded, with reasons:

・Wrong language (n=15)

・Wrong study design (n=16)

・Wrong population (n=32)

・Wrong index test (n=48)

・Wrong reference test (n=10)

・Others (n=6)

Duplicates

n=2

2364 records excluded

**Included**

**Eligibility**

**Screening**

Adverse events of bronchoalveolar lavage

**Identification**

15 Studies included in qualitative synthesis

374 Full-text articles assessed for eligibility

4193 records after duplicates removed

4348 records identified through database searching

4348 records identified through database searching

Medline via PubMed (n=3929)

CENTRAL (n=419)

0 additional records identified through other sources

15 Studies included in quantitative synthesis

(meta-analysis)*

359 Full-text articles excluded, with reasons:

・Wrong language (n=18)

・Wrong study design (n=18)

・Wrong population (n=30)

・Wrong index test (n=6)

・Wrong reference test (n=285)

・Others (n=2)

Duplicates

n=155

3819 records excluded

**Included**

**Eligibility**

**Screening**

1. Risk of bias

Blood antigenemia PCR (bronchoalveolar lavage fluid)


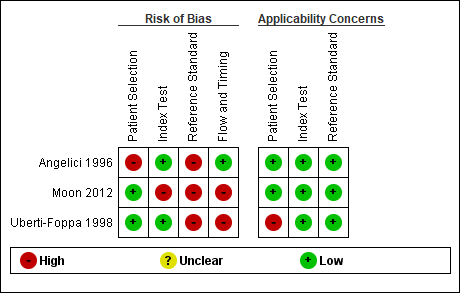

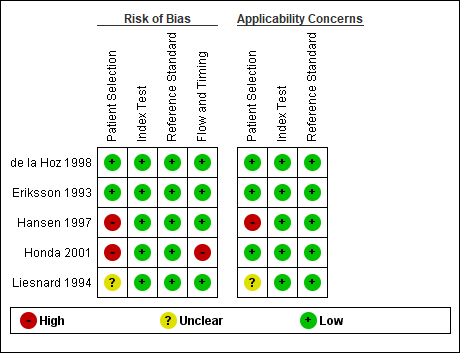


Adverse events of bronchoalveolar lavage

Mortality or serious disability


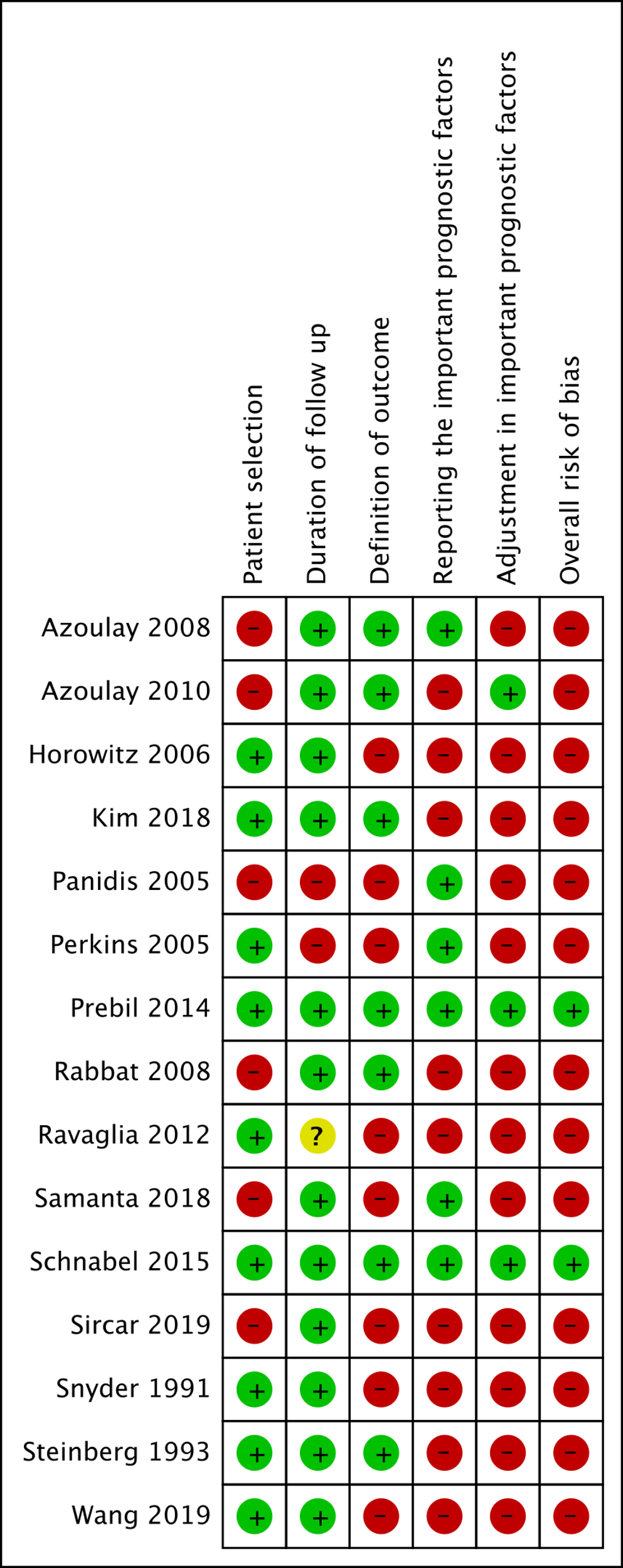


1. Forest plot

Blood antigenemia


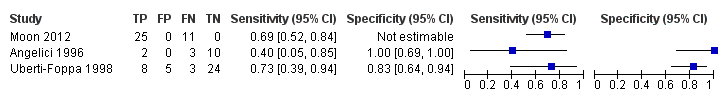


PCR (bronchoalveolar lavage fluid)


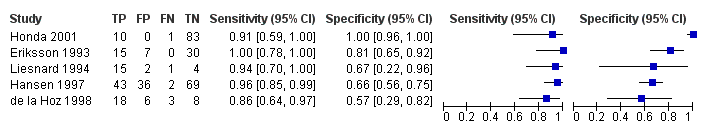


Adverse events of bronchoalveolar lavage

Mortality or serious disability


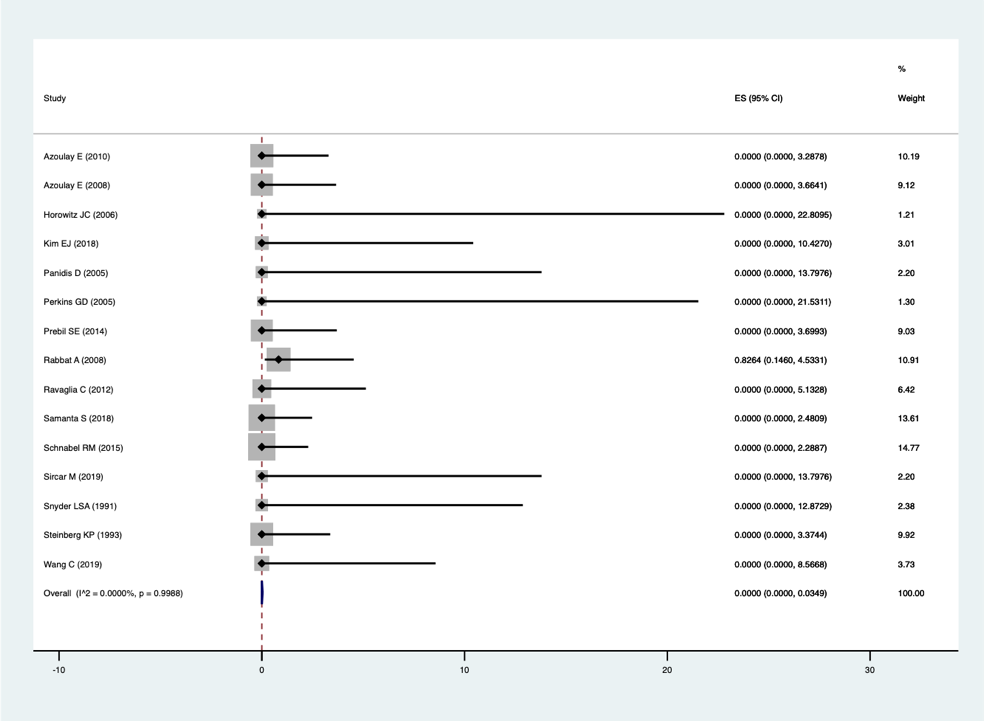


Severe complications of the respiratory system


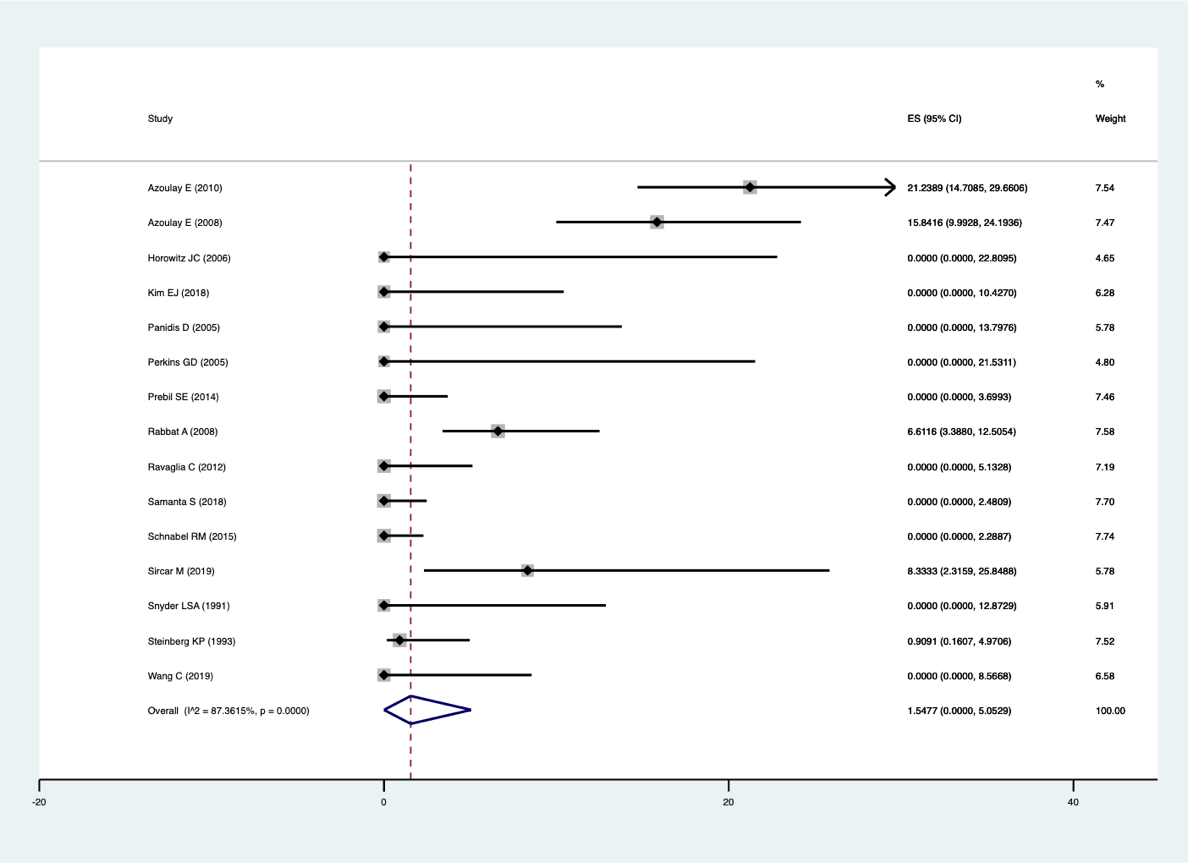


Severe complications of the cardiovascular system


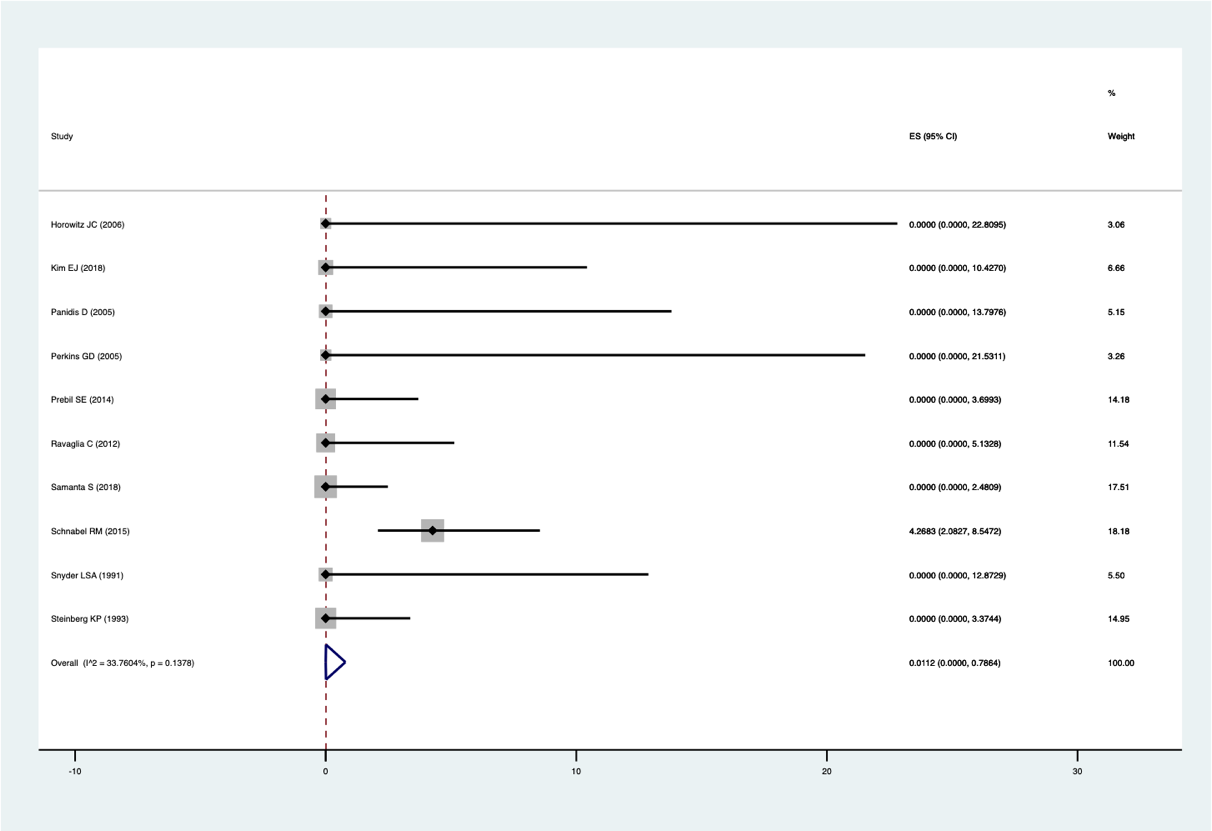


Major bleeding


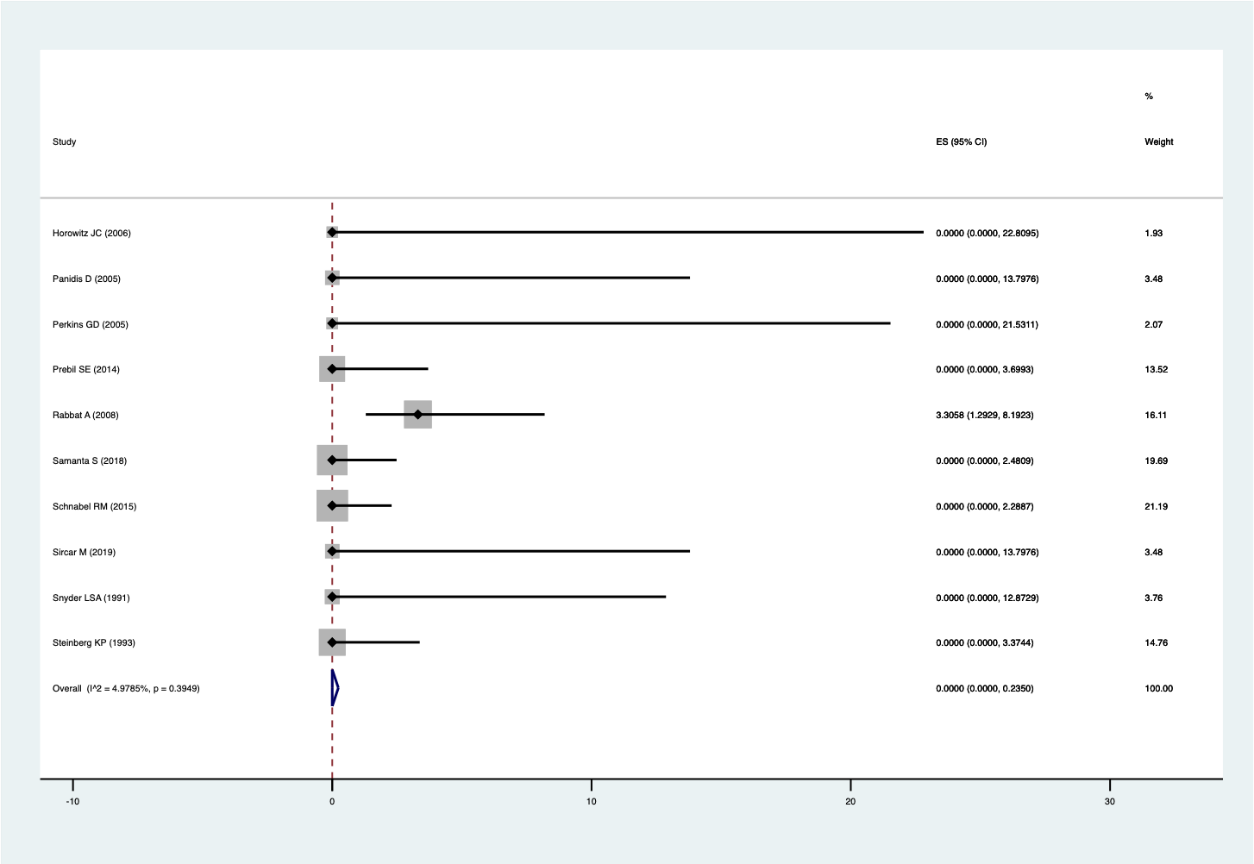


1. Evidence profile

Index test: Blood antigenemia

| | Sensitivity | 0.67 (95% CI: 0.54 to 0.79) | | --- | --- | | Specificity | 0.87 (95% CI: 0.73 to 0.95) | |  | | Prior probability | 5% | 10% | 20% | | --- | --- | --- | --- | |  |
| --- | --- | --- | --- | --- | --- | --- | --- | --- | --- | --- | --- |

| Outcome ^1^ | No. of studies (patients) | Study design | Assessment of certainty | | | | | Prevalence in 1000 patients | | | Certainty of the evidence |
| --- | --- | --- | --- | --- | --- | --- | --- | --- | --- | --- | --- |
|  |  |  | Risk of bias | Indirectness | Inconsistency | Imprecision | Publication bias | Prior probability 5% | Prior probability 10% | Prior probability 20% |  |
| True Positive | 3 (91) | Cross-sectional study, Cohort study | Very serious ^a^ | Serious ^c^ | Serious ^e^ | Serious ^f^ | None | 34 (27 to 39) | 67 (54 to 79) | 135 (107 to 157) | ⨁◯◯◯ Very low |
| False Negative |  |  |  |  |  |  |  | 16 (11 to 23) | 33 (21 to 46) | 65 (43 to 93) |  |
| True Negative | 2 (55) | Cross-sectional study, Cohort study | Serious ^b^ | Serious ^d^ | Not serious ^e^ | Very serious ^g^ | None | 828 (691 to 899) | 785 (654 to 851) | 698 (582 to 757) | ⨁◯◯◯ Very low |
| False Positive |  |  |  |  |  |  |  | 122 (51 to 259) | 115 (49 to 246) | 102 (43 to 218) |  |

1. All of the included studies defined the test result as “positive” if there were more than one positive cells per 200,000 cells.

a. In terms of risk of bias in the QUADAS-2 tool, the proportion of studies judged to have the high or unknown risk of bias was 1/3 for patient selection, 1/3 for index test, 3/3 for reference standard, and 2/3 for flow and timing. Thus, the proportion of studies with high or unknown risk in two or more categories was approximately more than 2/3, which was judged to be “Very serious.”

b. Specificity was integrated using two studies because one study had a true negative of zero. In terms of risk of bias in the QUADAS-2 tool, the proportion of studies judged to have a high or unknown risk of bias was 1/2 for patient selection, 0/2 for index test, 2/2 for reference standard, and 1/2 for flow and timing. Thus, a certain proportion of studies was determined to be a high or unknown risk, which was judged to be “Serious.”

c. Regarding the concerns about the applicability in the QUADAS-2 tool, the proportion of studies judged to have a high or unknown concern about the applicability was 1/3 for patient selection, 0/3 for index test, 0/3 for reference standard. Thus, the applicability was considered to be maintained because there was less than one category in which the proportion of studies with high or unknown concerns about applicability was approximately more than half. However, in this systematic review, we conducted the literature search not only for patients with ARDS but also for those with acute respiratory failure. Owing to this gap from the ideal systematic review question that should be set, there are limitations to the direct application of the results to the clinical situation assumed by guideline readers. Therefore, we judged the overall applicability to be "Serious.”

d. In terms of concerns about the applicability in the QUADAS-2 tool, the proportion of studies judged to have a high or unknown concern about the applicability was 1/2 for patient selection, 0/2 for index test, 0/2 for reference standard. Thus, the applicability was considered to be maintained because there was less than one category in which the proportion of studies with high or unknown concerns about applicability was more than about half. However, in this systematic review, we conducted the literature search not only for patients with ARDS but also for those with acute respiratory failure. Owing to this gap from the ideal systematic review question that should be set, the results to the clinical situation assumed by guideline readers have limited direct applicability. Therefore, we judged the overall applicability to be "Serious.”

e. We visually assessed the variability in the results of each study included in the systematic review using forest plots.

f. Results may be imprecise because the total number of patients included in the systematic review is below the optimal informative threshold. Additionally, we examined the net benefit of the test (difference between true positives and weighted false positives) if one false positive is considered acceptable for every 0.7 true positive. When the prevalence rate was set at 5–10%, the net benefit of the test did not differ between the upper and lower limits of the confidence interval of the integrated sensitivity, and clinical judgment was not expected to change. Therefore, the overall uncertainty was judged to be “Serious.”

g. Results may be imprecise because the total number of patients included in the systematic review is below the optimal informative threshold. Additionally, we examined the net benefit of the test (difference between true positives and weighted false positives) if one false positive was considered acceptable for every 0.7 true positive. When the prevalence rate was set at 10–20%, the net benefit of the test differed at the upper and lower limits of the confidence interval of the integrated specificity, which could change the clinical judgment. Consequently, the overall imprecision was judged to be “Very serious.”

Index test: PCR (bronchoalveolar lavage fluid)

| | Sensitivity | 0.94 (95% CI: 0.86 to 0.97) | | --- | --- | | Specificity | 0.84 (95% CI: 0.52 to 0.96) | |  | | Prior probability | 5% | 10% | 20% | | --- | --- | --- | --- | |  |
| --- | --- | --- | --- | --- | --- | --- | --- | --- | --- | --- | --- |

| Outcome | No. of studies (patients) | Study design | Assessment of certainty | | | | | Prevalence in 1000 patients | | | Certainty of the evidence |
| --- | --- | --- | --- | --- | --- | --- | --- | --- | --- | --- | --- |
|  |  |  | Risk of bias | Indirectness | Inconsistency | Imprecision | Publication bias | Prior probability 5% | Prior probability 10% | Prior probability 20% |  |
| True Positive | 5 (353) | Cross-sectional study, Cohort study | Serious ^a^ | Serious ^b^ | Serious ^c^ | Serious ^d^ | None | 47 (43 to 49) | 94 (86 to 97) | 187 (173 to 194) | ⨁◯◯◯ Very low |
| False Negative |  |  |  |  |  |  |  | 3 (1 to 7) | 6 (3 to 14) | 13 (6 to 27) |  |
| True Negative | 5 (353) | Cross-sectional study, Cohort study | Serious ^a^ | Serious ^b^ | Serious ^c^ | Very serious ^e^ | None | 798 (495 to 914) | 756 (469 to 866) | 672 (417 to 770) | ⨁◯◯◯ Very low |
| False Positive |  |  |  |  |  |  |  | 152 (36 to 455) | 144 (34 to 431) | 128 (30 to 383) |  |

a. In terms of risk of bias in the QUADAS-2 tool, the proportion of studies judged to a have high or unknown risk of bias was 3/5 for patient selection, 0/5 for index test, 0/5 for reference standard, and 1/5 for flow and timing. Thus, a certain proportion of studies was determined to be a high or unknown risk, which was judged to be “Serious.”

b. In terms of concerns about the applicability in the QUADAS-2 tool, the proportion of studies judged to have a high or unknown concern about the applicability was 2/5 for patient selection, 0/5 for index test, 0/5 for reference standard. Thus, the applicability was considered to be maintained because there was less than one category in which the proportion of studies with high or unknown concerns about applicability was approximately more than half. However, in this systematic review, we conducted the literature search not only for patients with ARDS but also for those with acute respiratory failure. Owing to this gap from the ideal systematic review question that should be set, the results to the clinical situation assumed by guideline readers have limited direct applicability. Therefore, we judged the overall applicability to be "Serious.”

c. We visually assessed the variability in the results of each study included in the systematic review using forest plots.

d. The results may be imprecise because the total number of patients included in the systematic review is below the optimal informative threshold. Additionally, we examined the net benefit of the test (difference between true positives and weighted false positives) if one false positive is considered acceptable for every 0.7 true positive. When the prevalence rate was set at 5–10%, the net benefit of the test did not differ between the upper and lower limits of the confidence interval of the integrated sensitivity, and clinical judgment was not expected to change. Therefore, the overall uncertainty was judged to be “Serious.”

e. The total number of patients included in the systematic review was above the threshold for optimal information content. Additionally, the net benefit of the test (difference between true negatives and weighted false negatives) was examined if one false positive is considered acceptable for every 0.7 true positive. When the prevalence was set at 10–20%, the net benefit of the test differed at the upper and lower limits of the confidence interval of the integrated specificity, which could have changed the clinical judgment. Resultantly, the overall imprecision was judged to be “Very serious.”

**Adverse events of bronchoalveolar lavage**

| Outcome | No. of studies (patients) | Study design | Summary of the result | | Assessment of certainty | | | | | Certainty of the evidence |
| --- | --- | --- | --- | --- | --- | --- | --- | --- | --- | --- |
|  |  |  | Frequency  (95%CI) | Frequency per 1000 patients (95%CI) | Risk of bias | Indirectness | Inconsistency | Imprecision | Publication bias |  |
| Death | 15 (1106) | Cohort study, Randomized controlled trial | 0.000%  (0.000-0.035) | 0 (0-0) | Serious ^c^ | Not serious ^d^ | Not serious ^e^ | Not serious ^g^ | Not assessed | ⨁⨁⨁◯ moderate |
| Severe complications of the respiratory system requiring new treatment ^a^ | 15 (1106) | Cohort study, Randomized controlled trial | 1.548% (0.000-5.053) | 15 (0-50) | Serious ^c^ | Not serious ^d^ | Not serious ^e,f^ | Not serious ^g^ | Not assessed | ⨁⨁⨁◯ moderate |
| Severe complications of the cardiovascular system requiring new treatment ^b^ | 10 (706) | Cohort study, Randomized controlled trial | 0.011% (0.000-0.788) | 0 (0-8) | Serious ^c^ | Not serious ^d^ | Not serious ^e^ | Serious ^h^ | Not assessed | ⨁⨁◯◯ low |
| Major bleeding | 10 (747) | Cohort study, Randomized controlled trial | 0.000% (0.000-0.235) | 0 (0-2) | Serious ^c^ | Not serious ^d^ | Not serious ^e^ | Serious ^h^ | Not assessed | ⨁⨁◯◯ low |

a. Initiation of mechanical ventilation or new pneumothorax

b. Initiation of vasopressor or anti-arrhythmic drugs

c. Regarding the risk of bias in the tool developed by Lorio et al. (Lorio A, et al. BMJ 2015;350:h870), the proportion of studies judged to have a high or unknown risk of bias was 6/15 for patient selection, 3/15 for duration of follow up, 8/15 for definition of the outcome, 8/15 for reporting the important prognostic factors, and 12/15 for adjustment in important prognostic factors. Thus, a certain proportion of studies was determined to have a high or unknown risk, which was judged to be “Serious”.

d. In this systematic review, we conducted a literature search using the ideal systematic review question that should be set, and thus the results were generally considered applicable to clinical situations assumed by guideline readers. Therefore, we judged the applicability to be “Not serious.”

e. The variability in the results of each study included in the systematic review was visually assessed using forest plots and based on the I^2^ statistic.

f. Despite I2 = 87%, this component was judged to be “Not serious,” because heterogeneity could be explained by subgroup analyses with separate studies that included patients, not on ventilators.

g. The total number of patients included in the systematic review was above the threshold for obtaining optimal information. Clinical judgment was not expected to change between the upper and lower ends of the confidence interval of integrated frequency. Consequently, the overall imprecision was judged to be “Not serious.”

h. The results may be imprecise because the total number of patients included in the systematic review was below the optimal informative threshold. Clinical judgment was not expected to change between the upper and lower limits of the confidence interval of integrated frequency. As a result, the overall imprecision was judged to be “Serious.”

1. Evidence-to-Decision table

| Question | |
| --- | --- |
| **CQ8： Should PCR tests of the bronchoalveolar lavage fluid and blood antigenemia methods be used for identifying cytomegalovirus pneumonia as the causative disease of ARDS (cytomegalovirus pneumonia)?** | |
| **Population:** | Patients with ARDS or acute respiratory failure |
| **Target condition:** | Cytomegalovirus (CMV) pneumonia |
| **Index test:** | PCR (bronchoalveolar lavage fluid [BALF]), blood antigenemia |
| **Purpose/role of the test:** | Differential diagnosis in ARDS management |
| **setting:** | Situation equivalent to the emergency room (ER) or intensive care unit (ICU) |
| **Main outcomes:** | Overall survival, serious adverse events from testing |
| **Medical practice based on test results:** | If positive (suspected cytomegalovirus pneumonia), initiate treatment with appropriate antiviral agents. If negative, avoid unnecessary antiviral treatment and perform additional testing and follow-up on a differential diagnosis. |
| **perspective:** | Individual |
| **background:** | ARDS is an acute respiratory failure with bilateral infiltrates without pulmonary edema, which can be caused by various diseases. CMV pneumonia is an important cause of ARDS because it is found not only in immunocompromised patients but also in patients with ARDS under immune response, and a delayed diagnosis is rarely not fatal. If CMV pneumonia is correctly diagnosed in patients with ARDS, treatment with appropriate antiviral agents may improve patient prognosis. Correct rule-out diagnosis may avoid the use of inappropriate antiviral agents, thus avoiding a worse patient prognosis. On the contrary, if the test misdiagnoses the patient, the patient may be treated with unnecessary drugs, which is harmful to the patient. The CMV blood antigenemia assay and the cytomegalovirus PCR test of BALF are used for the diagnosis of CMV infection. These tests are also used to monitor CMV infections and to determine the efficacy of antiviral agents and the timing of their discontinuation. Therefore, we posed the question, “Should PCR (BALF) and blood antigenemia be used to differentiate the cause of ARDS (CMV pneumonia)?” |
| **conflict of interest:** | None |

# Assessment

| Problem Is the problem a priority? | | |
| --- | --- | --- |
| Judgment | Research evidence | ADDITIONAL considerations |
| ● Yes  ○ Probably yes  ○ Probably no  ○ No  ○ Varies  ○ Do not know | CMV pneumonia is an important cause of ARDS because it is found not only in immunocompromised patients but also in patients with ARDS under immune response, and a delayed diagnosis is rarely not fatal. If CMV pneumonia is correctly diagnosed in patients with ARDS, treatment with appropriate antiviral agents may improve patient prognosis. Correct rule-out diagnosis may avoid the use of inappropriate antiviral agents, thus avoiding a worse patient prognosis. On the contrary, if the test misdiagnoses the patient, the patient may be treated with unnecessary drugs, which is harmful to the patient. The CMV blood antigenemia assay and the CMV-PCR test of BALF are used for the diagnosis of CMV infection. These tests are also used to monitor CMV infections and determine the efficacy of antiviral agents and the timing of their discontinuation. Therefore, this clinical question was considered to be of high priority. |  |
| Test accuracy How accurate is the test? | | |
| Judgment | Research evidence | ADDITIONAL considerations |
| ○ Very accurate  ● Accurate  ○ Inaccurate  ○ Very inaccurate  ○ Varies  ○ Do not know | The results of the systematic review and meta-analysis showed the following. The accuracy of the test was judged to be “Accurate.”  **Blood antigenemia**  (three studies, 91 patients) *  Integrated sensitivity: 0.67 (95% CI: 0.54-0.79)  Integrated specificity: 0.87 (95% CI: 0.73-0.95)  (Bivariate model)  *All the included studies defined a test result as “positive” if there were more than 1 positive cell/200,000 cells.   | Blood antigenemia | Prevalence in 1000 patients | | | Certainty of the evidence | | --- | --- | --- | --- | --- | | Prior probability | 5% | 10% | 20% |  | | True positive | 34 (27-39) | 67 (54-79) | 135 (107-157) | Very low | | False negative | 16 (11-23) | 33 (21-46) | 65 (43-93) | | True negative | 828 (691-899) | 785 (654-851) | 698 (582-757) | Very low | | False positive | 122 (51-259) | 115 (49-246) | 102 (43-218) |   **PCR (BALF)**  (five studies, 353 patients)  Integrated sensitivity: 0.94 (95% CI: 0.86-0.97)  Integrated specificity: 0.84 (95% CI: 0.52-0.96)  (Bivariate model)   | PCR (BALF) | Prevalence in 1000 patients | | | Certainty of the evidence | | --- | --- | --- | --- | --- | | Prior probability | 5% | 10% | 20% |  | | True positive | 47 (43-49) | 94 (86-97) | 187 (173-194) | Very low | | False negative | 3 (1-7) | 6 (3-14) | 13 (6-27) | | True negative | 798 (495-914) | 756 (469-866) | 672 (417-770) | Very low | | False positive | 152 (36-455) | 144 (34-431) | 128 (30-383) | |  |
| Desirable effects How substantial are the desirable anticipated effects? | | |
| Judgment | Research evidence | ADDITIONAL considerations |
| ○ Large  ● Moderate  ○ Small  ○ Trivial  ○ Varies  ○ Do not know | The number of patients who would benefit from appropriate treatment in a sample of 1000 patients (the number of true positives) was calculated. The desirable effects were judged to be “Moderate.”  **Blood antigenemia**   | Prior probability | 5% | 10% | 20% | | --- | --- | --- | --- | | Treatment based on test results | 34 | 67 | 135 | | Treatment of all patients regardless of test results | 50 | 100 | 200 | | No treatment for all patients regardless of test results | 0 | 0 | 0 |   **PCR (BALF)**   | Prior probability | 5% | 10% | 20% | | --- | --- | --- | --- | | Treatment based on test results | 21 | 55 | 124 | | Treatment of all patients regardless of test results | 50 | 100 | 200 | | No treatment for all patients regardless of test results | 0 | 0 | 0 | | If all patients were to be treated regardless of the test results, the number of patients who would benefit from treatment was calculated as 1000 x (prior probability).  If all patients were not to be treated regardless of the test results, the number of patients who would benefit from the treatment was considered to be zero. |
| Undesirable effectsHow substantial are the undesirable anticipated effects? | | |
| Judgment | Research evidence | ADDITIONAL considerations |
| ○ Large  ○ Moderate  ○ Small  ○ Trivial  ● Varies  ○ Do not know | The relative clinical weighting of false positives to true positives was set at 0.1. The number of patients who would be harmed by unnecessary treatment in a sample of 1000 patients was calculated. The undesirable effects were judged to be “Varies.”  **Blood antigenemia**   | Prior probability | 5% | 10% | 20% | | --- | --- | --- | --- | | Treatment based on test results | 12 | 12 | 10 | | Treatment of all patients regardless of test results | 95 | 90 | 80 | | No treatment for all patients regardless of test results | 0 | 0 | 0 |   **PCR (BALF)**   | Prior probability | 5% | 10% | 20% | | --- | --- | --- | --- | | Treatment based on test results | 46 | 43 | 38 | | Treatment of all patients regardless of test results | 95 | 90 | 80 | | No treatment for all patients regardless of test results | 0 | 0 | 0 |   Frequency of adverse events of bronchoalveolar lavage   |  | Summary of the result | | Certainty of the evidence | | --- | --- | --- | --- | | Outcome | Frequency  (95% CI) | Frequency per 1000 patients (95% CI) | | Death | 0.000%  (0.000-0.035) | 0 (0-0) | Moderate | | Severe complications of respiratory system requiring new treatment ^a^ | 1.548% (0.000-5.053) | 15 (0-50) | Moderate | | Severe complications of cardiovascular system requiring new treatment ^b^ | 0.011% (0.000-0.788) | 0 (0-8) | Low | | Major bleeding | 0.000% (0.000-0.235) | 0 (0-2) | Low |   **Bronchoalveolar lavage**   | Adverse events |  | | --- | --- | | Death | 0 | | Severe complications of the respiratory system requiring new treatment ^a^ | 6 | | Severe complications of the cardiovascular system requiring new treatment ^b^ | 0 | | Major bleeding | 0 |   The relative clinical weighting of death to true positives was set at 1.0, and other serious adverse events were set at 0.4. | If patients were treated based on test results, the number of patients who would be harmed by unnecessary treatment was calculated as (number of false positives) x (clinical weighting).  If all patients were treated regardless of test results, the number of false positives was calculated as (1-prior probability) x 1000.  If all patients were not treated, the number of false positives was considered to be zero.  The harm of adverse events was calculated by multiplying the frequency of adverse events expected when the test was performed on 1000 people by the clinical weighting. |
| Certainty of evidence What is the overall certainty of the evidence of test accuracy | | |
| Judgment | Research evidence | ADDITIONAL considerations |
| ● Very low  ○ Low  ○ Moderate  ○ High  ○ No included study | The certainty of the evidence was judged to be “Very low” by adopting the certainty of the evidence with the lowest certainty. |  |
| Certainty of the evidence of test’s effects What is the overall certainty of the evidence for any critical or important direct benefits, adverse effects, or burden of the test? | | |
| Judgment | Research evidence | ADDITIONAL considerations |
| ○ Very low  ● Low  ○ Moderate  ○ High  ○ No included study  ○ Do not know | No evidence examining the direct effects of the tests. The certainty of the evidence was judged to be “Low” for adverse events of bronchoalveolar lavage. |  |
| Certainty of evidence of management’s effects What is the overall certainty of the evidence of effects of the management that is guided by the test results? | | |
| Judgment | Research evidence | ADDITIONAL considerations |
| ○ Very low  ○ Low  ○ Moderate  ○ High  ● No included study  ○ Do not know | In general, treatment based on the result of true positives with antiviral agents is considered “good medical practice” and is expected to improve overall survival and other outcomes. On the contrary, if patients receive unnecessary treatment due to false positives, undesirable effects (such as adverse drug events) can occur. However, no studies have included evidence on the impact of treatment on final outcomes such as overall survival. |  |
| Certainty of evidence of test result/management How certain is the link between test results and management decisions? | | |
| Judgment | Research evidence | ADDITIONAL considerations |
| ○ Very low  ○ Low  ○ Moderate  ○ High  ● No included study  ○ Do not know | Since treatment of CMV pneumonia is usually initiated promptly based on the test results, it seems reasonable to assume that there is a high degree of certainty regarding the relationship between the test results and management decisions. However, no studies have been included as evidence. |  |
| Certainty of effects What is the overall certainty of the evidence of effects of the test? | | |
| Judgment | Research evidence | ADDITIONAL considerations |
| ● Very low  ○ Low  ○ Moderate  ○ High  ○ No included study  ○ Do not know | Since the certainty of the evidence of the test’s accuracy is “Very low,” the certainty of the evidence of effects of the test becomes “Very low.” |  |
| Values Is there important uncertainty about or variability in how much people value the main outcomes? | | |
| Judgment | Research evidence | ADDITIONAL considerations |
| ○ Important uncertainty or variability  ● Possibly important uncertainty or variability  ○ Probably no important uncertainty or variability  ○ No important uncertainty or variability | The value of desirable effects such as true positives and undesirable effects such as false positives and adverse events may vary according to the values and experiences of individual healthcare providers and patients.  (The relative clinical weighting of false positives to true positives may vary depending on the values and experiences of individual health care providers and patients.) |  |
| Balance of effects Does the balance between desirable and undesirable effects favor the intervention or the comparison? | | |
| Judgment | Research evidence | ADDITIONAL considerations |
| ● Favors the test  ○ Probably favors the test  ○ Does not favor either the test or the comparison  ○ Probably favors the comparison  ○ Favors the comparison  ○ Varies  ○ Do not know | The net benefit of the test was calculated, considering the benefit of appropriate treatment due to true positive diagnosis, the harm of unnecessary treatment due to false positive diagnosis, and the adverse events of the test.  **Blood antigenemia**  Net benefit  (The number of patients who benefit from the test when performed on 1000 patients.)   | Prior probability | 5% | 10% | 20% | | --- | --- | --- | --- | | Treatment based on test results | 22 | 56 | 124 | | Treatment of all patients regardless of test results | -45 | 10 | 120 | | No treatment for all patients regardless of test results | 0 | 0 | 0 |   The relative clinical weight of false positives to true positives was set at 0.1, and the net benefit was calculated with a prior probability of 5-20%. Serious adverse events of the test were considered to be negligible. We compared the net benefit of treating patients based on the test results, treating all patients without testing, and not treating any patients without testing. There are likely to be many clinical situations where there would be a net benefit from testing.  **PCR (BALF)**  Net benefit  (The number of patients who benefit from the test when performed on 1000 patients.)   | Prior probability | 5% | 10% | 20% | | --- | --- | --- | --- | | Treatment based on test results | 26 | 74 | 169 | | Treatment of all patients regardless of test results | -45 | 10 | 120 | | No treatment for all patients regardless of test results | 0 | 0 | 0 |   The relative clinical weight of false positives to true positives was set at 0.1, and the net benefit was calculated with a prior probability of 5-20%. Regarding serious adverse events of bronchoalveolar lavage, the relative clinical weighting of death to true positives was set at 1.0, and other serious adverse events were set at 0.4. We compared the net benefit of treating patients based on the test results, treating all patients without testing, and not treating any patients without testing. There are likely to be many clinical situations where there would be a net benefit from testing.  Based on these results, the balance of effects was judged to be “Favors the test”. | The net benefit (net benefit) was calculated as (number of true positives receiving appropriate treatment) -(number of false positives receiving unnecessary treatment) × (clinical weighting) -(number of serious adverse events of the test) for a sample of 1000 patients.  If adverse events were considered negligible, they were calculated as zero. |
| Acceptability Is the intervention acceptable to key stakeholders? | | |
| Judgment | Research evidence | ADDITIONAL considerations |
| ● Yes  ○ Probably yes  ○ Probably no  ○ No  ○ Varies  ○ Do not know | It is a commonly practiced medical procedure and probably acceptable. |  |
| Feasibility Is the intervention feasible to implement? | | |
| Judgment | Research evidence | ADDITIONAL considerations |
| ● Yes  ○ Probably yes  ○ Probably no  ○ No  ○ Varies  ○ Do not know | They are commonly practiced medical procedures and probably feasible, but some facilities may not be able to perform bronchoalveolar lavage. |  |

# Summary of Judgment

|  | **Judgment** | | | | | | |
| --- | --- | --- | --- | --- | --- | --- | --- |
| **PROBLEM** | No | Probably no | Probably yes | Yes |  | Varies | Do not know |
| **DESIRABLE EFFECTS** | Trivial | Small | Moderate | Large |  | Varies | Do not know |
| **UNDESIRABLE EFFECTS** | Large | Moderate | Small | Trivial |  | Varies | Do not know |
| **CERTAINTY OF EVIDENCE OF TEST ACCURACY** | Very low | Low | Moderate | High |  |  | No included study |
| **CERTAINTY OF THE EVIDENCE OF TEST’S EFFECTS** | Very low | Low | Moderate | High |  |  | No included study |
| **CERTAINTY OF THE EVIDENCE OF MANAGEMENT’S EFFECTS** | Very low | Low | Moderate | High |  |  | No included study |
| **CERTAINTY OF THE EVIDENCE OF TEST RESULT/MANAGEMENT** | Very low | Low | Moderate | High |  |  | No included study |
| **CERTAINTY OF EFFECT** | Very low | Low | Moderate | High |  |  | No included study |
| **VALUES** | Important uncertainty or variability | Possibly important uncertainty or variability | Probably no important uncertainty or variability | No important uncertainty or variability |  |  |  |
| **BALANCE OF EFFECTS** | Favors the comparison | Probably favors the comparison | Does not favor either the test or the comparison | Probably favors the test | Favors the test | Varies | Do not know |
| **ACCEPTABILITY** | No | Probably no | Probably yes | Yes |  | Varies | Do not know |
| **FEASIBILITY** | No | Probably no | Probably yes | Yes |  | Varies | Do not know |

# Type of Recommendation

| Strong recommendation against the test | Conditional recommendation against the test | Conditional recommendation for either the test or the comparison | Conditional recommendation for the test | Strong recommendation for the test |
| --- | --- | --- | --- | --- |
| ○ | ○ | ○ | ● | ○ |

# Conclusions

| Recommendation |
| --- |
| **We conditionally recommend the use of PCR (bronchoalveolar lavage) and blood antigenemia to differentiate the cause of ARDS (cytomegalovirus pneumonia) (Conditional recommendation/very low certainty of the evidence: GRADE: 2D).**  **Note: If the clinical situation (characteristics of the target patient, characteristics and timing of the test, prior probability, values of the patient and health care providers) changes, the balance of effects may change, and different options may be recommended.** |
|  |
| Justification |
| **Question**  Should PCR tests of the bronchoalveolar lavage fluid and blood antigenemia methods be used for identifying cytomegalovirus pneumonia as the causative disease of ARDS (cytomegalovirus pneumonia)?  **Patients**  Patients with ARDS  **Index test**  PCR (BALF), blood antigenemia  **Purpose, role, and setting of the test**  Differential diagnosis in ARDS management in ER, ICU, or equivalent  **Medical practice based on test results**  If positive, the patient will be diagnosed with CMV pneumonia and treated with appropriate antiviral agents. If negative, unnecessary antibiotic treatment will be avoided, and additional testing for different diagnostic targets or follow-up will be performed.  **Summary of evidence**：  PCR (BALF) (five studies, 353 patients) (Bivariate model)  Integrated sensitivity: 0.94 （95% CI: 0.86-0.97), Integrated specificity: 0.84 (95% CI: 0.52-0.96)  Blood antigenemia (three studies, 91 patients) (Bivariate model)  Integrated sensitivity: 0.67 （95% CI: 0.54-0.79), Integrated specificity: 0.87 (95% CI: 0.73-0.95)  <"positive" = more than 1 positive cell/200,000 cells>  Frequency of adverse events of bronchoalveolar lavage  Death: 0.000% (95% CI: 0.000 - 0.035)  **Certainty of the evidence**：  Certainty of the evidence of test accuracy was “Very low.” Certainty of the evidence of the frequency of adverse events of bronchoalveolar lavage was “Moderate” or “Low.”  **Values, balance of effects, acceptability, feasibility**：  The desirable effect of the tests is to diagnose CMV pneumonia and receive appropriate treatment promptly. An undesirable effect of the tests is that the patient may receive unnecessary treatment due to a false positive result. When comparing these effects of testing with those of deciding the treatment plan without testing, the desirable effects of testing are considered to be larger. Feasibility is not a problem, and it is a generally accepted medical practice.  **Panel meeting**  In the preliminary vote, the median score of “recommended text proposal” was 8, and the disagreement index was 0.292 by the modified Delphi method.  At the panel meeting, an agreement was achieved with the results of a preliminary vote.  **Additional considerations**：  The balance of effects depends on the prior probability, the clinical weighting of false positives, and the performance of the test. Therefore, if the clinical situation (characteristics of the patient, characteristics and timing of the test, prior probability, the clinical weighting of false positives, and other values held by patients and caregivers) changes, the balance of effects may change, and different options may be recommended. It is also important to note that the primary studies included in this systematic review included studies that limited the target to immunosuppressed patients. When the results are adapted to a group of immunocompetent patients, the balance of effects of the tests may change, and different options may be recommended. |

| Subgroup considerations |
| --- |
| Recommendations may vary depending on the patient’s immune status. |
| Implementation considerations |
| In cases where the risk of adverse events of bronchoalveolar lavage is concerned (poorly controlled bronchial asthma or interstitial pneumonia), the feasibility should be carefully considered. The systematic review included studies mainly in immunocompromised patients. In addition, in the included studies, microscopic findings of bronchoalveolar lavage fluid and lung tissue were used as reference standards. These tests may be used in the definitive diagnosis of CMV pneumonia. |

| Monitoring and evaluation |
| --- |
| After the publication of this medical guideline, it is necessary to collect and monitor information on the status of clinical use and problems in conducting the test using tools such as questionnaires. |
| Research priorities |
| The quality of the evidence was “Very low.” Further high-quality studies are warranted to evaluate the diagnostic accuracy of CMV-PCR (BALF) and CMV blood antigenemia assays in patients with ARDS. |

**CQ9 Should serum β-D-glucan be used for identifying *Pneumocystis* pneumonia as the causative disease of ARDS?**

In this CQ, the systematic review and meta-analysis on the diagnostic performance of serum β-D-glucan was partially reused from previously published systematic reviews and meta-analyses, and updated by additional searches.

White SK, Walker BS, Hanson KE, Schmidt RL. Diagnostic Accuracy of β-d-Glucan (Fungitell) Testing Among Patients With Hematologic Malignancies or Solid Organ Tumors: A Systematic Review and Meta-Analysis. Am J Clin Pathol. 2019 Feb 4;151(3):275-285.

1.Search strategy

MEDLINE via PubMed （Search date: 2020/9/18）

| #1 | BDG*.tw. |
| --- | --- |
| #2 | Fungitel*.tw. |
| #3 | Cape Cod.tw. |
| #4 | Fungitec*.tw. |
| #5 | Seikagaku.tw. |
| #6 | Wake Test.tw. |
| #7 | (Wako* or Waco*).tw. |
| #8 | #1 or #2 or #3 or #4 or #5 or #6 or #7 |
| #9 | exp beta-Glucans/ |
| #10 | Glucans/ |
| #11 | D-glucan*.tw. |
| #12 | #9 or #10 or #11 |
| #13 | exp "Sensitivity and Specificity"/ |
| #14 | (sensitivit* or specificit*).tw. |
| #15 | predictive value*.tw. |
| #16 | diagnosis.fs. |
| #17 | analysis.fs. |
| #18 | Reagent Kits, Diagnostic/ |
| #19 | #13 or #14 or #15 or #16 or #17 or #18 |
| #20 | #12 and #19 |
| #21 | exp Mycoses/ |
| #22 | exp Fungi/ |
| #23 | fungal.tw. |
| #24 | fungus.tw. |
| #25 | mycos*.tw. |
| #26 | mycot*.tw. |
| #27 | aspergill*.tw. |
| #28 | pneumocystis.tw. |
| #29 | #21 or #22 or #23 or #24 or #25 or #26 or #27 or #28 |
| #30 | #12 and #29 |
| #31 | #8 or #20 or #30 |
| #32 | exp animals/ not humans.sh. |
| #33 | #31 not #32 |
| #34 | limit #33 to yr="2017 -Current" |

EMBASE （Search date: 2020/9/16）

| S1 | TI,AB(BDG*) |
| --- | --- |
| S2 | TI,AB(Fungitel*) |
| S3 | (TI,AB(Cape Cod)) |
| S4 | TI,AB(Fungitec*) |
| S5 | TI,AB(Seikagaku) |
| S6 | (TI,AB("Wake Test")) |
| S7 | (TI,AB(Wako* OR Waco*)) |
| S8 | (S1 or S2 or S3 or S4 or S5 or S6 or S7) |
| S9 | (EMB.EXACT.EXPLODE("beta glucan")) |
| S10 | EMB.EXACT("glucan") |
| S11 | TI,AB(D-glucan*) |
| S12 | (S9 or S10 or S11) |
| S13 | (EMB.EXACT("sensitivity and specificity")) |
| S14 | (TI,AB(sensitivit* OR specificit*)) |
| S15 | (TI,AB(predictive value*)) |
| S16 | QU(DI) |
| S17 | (EMB.EXACT("diagnostic kit")) |
| S18 | (EMB.EXACT.EXPLODE("diagnostic procedure")) |
| S19 | (S13 or S14 or S15 or S16 or S17 or S18) |
| S20 | (S12 and S19) |
| S21 | EMB.EXACT.EXPLODE("mycosis") |
| S22 | EMB.EXACT.EXPLODE("fungus") |
| S23 | TI,AB(fungal) |
| S24 | TI,AB(fungus) |
| S25 | TI,AB(mycos*) |
| S26 | TI,AB(mycot*) |
| S27 | TI,AB(aspergill*) |
| S28 | TI,AB(pneumocystis) |
| S29 | (S21 or S22 or S23 or S24 or S25 or S26 or S27 or S28) |
| S30 | (S12 and #29) |
| S31 | (S8 or S20 or S30) |
| S32 | (ANIMAL(YES) NOT HUMAN(YES)) |
| S33 | (S31 not S32) |
| S34 | (S33 AND YR(>=2017)) |

1. Flow diagram

**Identification**

3 Studies covered by existing systematic reviews

3524 records identified through database searching

Medline via PubMed (n=1088)

EMBASE (ProQuest) (n=2436)

12 Studies included in quantitative synthesis

(meta-analysis)*

9 Studies invasive pulmonary aspergillosis

3 Studies pneumocystis jirovecii pneumonia

12 Studies included in qualitative synthesis

3524 records identified through database searching

2807 records after duplicates removed

71 Full-text articles assessed for eligibility

62 Full-text articles excluded, with reasons:

・Wrong language (n=2)

・Wrong study design (n=8)

・Wrong publication type (n=29)

・Wrong population (n=4)

・Wrong index test (n=3)

・Others (n=16)

2736 records excluded

Duplicates

n=717

**Screening**

**Eligibility**

**Included**

1. Risk of bias


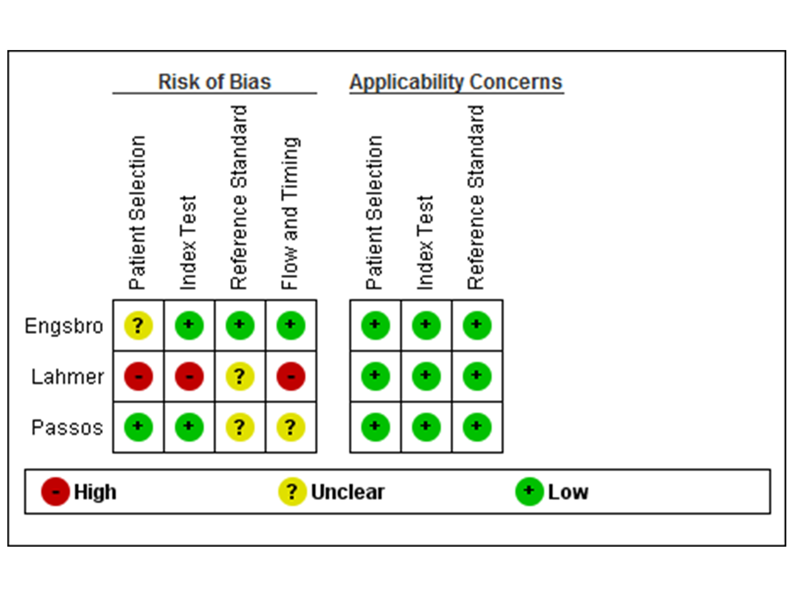


4.Forest plot

Serum β-D-glucan Sensitivity

Serum β-D-glucan Specificity

1. Evidence profile

Index test: Serum β-D-glucan

| | Sensitivity | 0.84 (95% CI: 0.66 to 0.93) | | --- | --- | | Specificity | 0.79 (95% CI: 0.69 to 0.87) | |  | | Prior probability | 5% | 10% | 20% | | --- | --- | --- | --- | |  |
| --- | --- | --- | --- | --- | --- | --- | --- | --- | --- | --- | --- |

| Outcome | No. of studies (patients) | Study design | Assessment of certainty | | | | | Prevalence in 1000 patients | | | Certainty of the evidence |
| --- | --- | --- | --- | --- | --- | --- | --- | --- | --- | --- | --- |
|  |  |  | Risk of bias | Indirectness | Inconsistency | Imprecision | Publication bias | Prior probability 5% | Prior probability 10% | Prior probability 20% |  |
| True Positive | 3 (148) | Cross-sectional study, Cohort study | Very serious ^a^ | Serious ^b^ | Serious ^c^ | Very serious ^d^ | None | 42 (33 to 47) | 84 (66 to 93) | 168 (132 to 186) | ⨁◯◯◯ Very low |
| False Negative |  |  |  |  |  |  |  | 8 (3 to 17) | 16 (7 to 34) | 32 (14 to 68) |  |
| True Negative | 3 (148) | Cross-sectional study, Cohort study | Very serious ^a^ | Serious ^b^ | Serious ^c^ | Not serious ^e^ | None | 751 (656 to 827) | 711 (621 to 783) | 632 (552 to 696) | ⨁◯◯◯ Very low |
| False Positive |  |  |  |  |  |  |  | 199 (123 to 294) | 189 (117 to 279) | 168 (104 to 248) |  |

a. Regarding the risk of bias in the QUADAS-2 tool, the proportion of studies judged to have a high or unknown risk of bias was 2/3 for patient selection, 1/3 for index test, 2/3 for reference standard, and 2/3 for flow and timing. Thus, the proportion of studies with high or unknown risk in two or more categories was approximately more than 2/3, which is judged to be “Very serious.”

b. Regarding the concerns about the applicability in the QUADAS-2 tool, the proportion of studies judged to have a high or unknown concern about the applicability was 0/3 for patient selection, 0/3 for index test, 0/3 for reference standard. Thus, the applicability was considered to be maintained because there was less than one category in which the proportion of studies with high or unknown concerns about applicability was approximately more than half. However, in this systematic review, we conducted the literature search not only for patients with ARDS but also for those with acute respiratory failure. Owing to this gap from the ideal systematic review question that should be set, the results to the clinical situation assumed by guideline readers have limited direct applicability. Therefore, we judged the overall applicability to be "Serious.”

c. We visually assessed the variability in the results of each study included in the systematic review using forest plots.

d. The results may be imprecise because the total number of patients included in the systematic review is below the optimal informative threshold. Additionally, we examined the net benefit of the test (difference between true positives and weighted false positives) if one false positive is considered acceptable for every 0.5 true positive. When the prevalence rate was set at 10%, the net benefit of the test differed at the upper and lower limits of the confidence interval of the integrated sensitivity, which could have changed the clinical judgment. Therefore, the overall uncertainty was judged to be “Very serious.”

e. The total number of patients included in the systematic review was above the threshold for optimal information content. Additionally, the net benefit of the test (difference between true negatives and weighted false negatives) was examined if one false positive is considered acceptable for every 0.5 true positive. When the prevalence was set at 10–20%, the net benefit of the test did not differ between the upper and lower limits of the confidence interval of the integrated specificity, and clinical judgment was not expected to change. Therefore, the overall imprecision was judged to be “Not serious.”

1. Evidence-to-Decision table

| Question | |
| --- | --- |
| **CQ9：** Should serum β-D-glucan be used for identifying *Pneumocystis* pneumonia as the causative disease of ARDS? | |
| **Population:** | Patients with ARDS or acute respiratory failure |
| **Target condition:** | Pneumocystis pneumonia |
| **Index test:** | Serum β-D-glucan |
| **Purpose/role of the test:** | Differential diagnosis in ARDS management |
| **setting:** | Situation equivalent to the emergency room (ER) or intensive care unit (ICU) |
| **Main outcomes:** | Overall survival, serious adverse events from testing |
| **Medical practice based on test results:** | If positive (suspected pneumocystis pneumonia), initiate treatment with appropriate agents. If negative, avoid unnecessary treatment and perform additional testing on a differential diagnosis or follow-up. |
| **perspective:** | Individual |
| **background:** | Pneumocystis pneumonia (PCP) causes acute respiratory failure and can be fatal if not promptly diagnosed and appropriately treated. Early diagnosis of PCP by serum β-D-glucan test will enable appropriate drug administration and potentially contribute to improved patient prognosis. On the contrary, if PCP is wrongly diagnosed, unnecessary medication may be administered, which may cause adverse effects. In addition, the lack of treatment for the true cause of ARDS may worsen patient prognosis. It is an important issue to examine these benefits and harms of the test in the management of ARDS. Therefore, we posed the question, “Should serum β-D-glucan be used to differentiate the cause of ARDS (pneumocystis pneumonia)?” |
| **conflict of interest:** | None |

# Assessment

| Problem Is the problem a priority? | | |
| --- | --- | --- |
| Judgment | Research evidence | ADDITIONAL considerations |
| ● Yes  ○ Probably yes  ○ Probably no  ○ No  ○ Varies  ○ Do not know | PCP causes acute respiratory failure and can be fatal if not promptly diagnosed and appropriately treated. Early diagnosis of PCP using a serum β-D-glucan test will enable appropriate drug administration and potentially contribute to improved patient prognosis. On the contrary, if PCP is wrongly diagnosed, unnecessary medication may be administered, which may cause adverse effects. In addition, the lack of treatment for the true cause of ARDS may worsen patient prognosis. It is an important issue to examine these benefits and harms of the test in the management of ARDS. Therefore, this clinical question was considered to be of high priority. |  |
| Test accuracy How accurate is the test? | | |
| Judgment | Research evidence | ADDITIONAL considerations |
| ○ Very accurate  ● Accurate  ○ Inaccurate  ○ Very inaccurate  ○ Varies  ○ Do not know | The results of the systematic review and meta-analysis showed the following. The accuracy of the test was judged to be “Accurate.”  **Serum β-D-glucan**  **<Cutoff 80 pg/mL>**  (three studies, 148 patients)  Integrated sensitivity: 0.84 (95% CI: 0.66 - 0.93)  Integrated specificity: 0.79 (95% CI: 0.69 - 0.87)   | Serum β-D-glucan | Prevalence in 1000 patients | | | Certainty of the evidence | | --- | --- | --- | --- | --- | | Prior probability | 5% | 10% | 20% |  | | True Positive | 42 (33 - 47) | 84 (66 - 93) | 168 (132 - 186) | Very low | | False Negative | 8 (3 - 17) | 16 (7 - 34) | 32 (14 - 68) | | True Negative | 751 (656 -827) | 711 (621 - 783) | 632 (552 - 696) | Very low | | False Positive | 199 (123 -294) | 189 (117 - 279) | 168 (104 - 248) | |  |
| Desirable effects How substantial are the desirable anticipated effects? | | |
| Judgment | Research evidence | ADDITIONAL considerations |
| ● Large  ○ Moderate  ○ Small  ○ Trivial  ○ Varies  ○ Do not know | The number of patients who would benefit from appropriate treatment in a sample of 1000 patients (the number of true positives) was calculated. The desirable effects were judged to be “Large.”  **Serum β-D-glucan**  **<Cutoff 80 pg/mL>**   | Prior probability | 5% | 10% | 20% | | --- | --- | --- | --- | | Treatment based on test results | 42 | 84 | 168 | | Treatment of all patients regardless of test results | 50 | 100 | 200 | | No treatment for all patients regardless of test results | 0 | 0 | 0 | | If all patients were to be treated regardless of the test results, the number of patients who would benefit from treatment was calculated as 1000 x (prior probability).  If all patients were not to be treated regardless of the test results, the number of patients who would benefit from the treatment was considered to be zero. |
| Undesirable effectsHow substantial are the undesirable anticipated effects? | | |
| Judgment | Research evidence | ADDITIONAL considerations |
| ○ Large  ○ Moderate  ● Small  ○ Trivial  ○ Varies  ○ Do not know | The relative clinical weighting of false positives to true positives was set at 0.3. The number of patients who would be harmed by unnecessary treatment in a sample of 1000 patients was calculated. The undesirable effects were judged to be “Small.”  **Serum β-D-glucan**  **<Cutoff 80 pg/mL>**   | Prior probability | 5% | 10% | 20% | | --- | --- | --- | --- | | Treatment based on test results | 60 | 57 | 50 | | Treatment of all patients regardless of test results | 285 | 270 | 240 | | No treatment for all patients regardless of test results | 0 | 0 | 0 | | If patients were treated based on test results, the number of patients who would be harmed by unnecessary treatment was calculated as (number of false positives) x (clinical weighting).  If all patients were treated regardless of test results, the number of false positives was calculated as (1 - prior probability) x 1000.  If all patients were not treated, the number of false positives was considered to be zero. |
| Certainty of evidence What is the overall certainty of the evidence of test accuracy? | | |
| Judgment | Research evidence | ADDITIONAL considerations |
| ● Very low  ○ Low  ○ Moderate  ○ High  ○ No included study | The certainty of the evidence was judged to be “Very low” by adopting the certainty of the evidence with the lowest certainty. |  |
| Certainty of the evidence of test’s effects What is the overall certainty of the evidence for any critical or important direct benefits, adverse effects, or burden of the test? | | |
| Judgment | Research evidence | ADDITIONAL considerations |
| ○ Very low  ○ Low  ○ Moderate  ○ High  ● No included study  ○ Do not know | No evidence examining the direct effects of the tests. |  |
| Certainty of evidence of management’s effects What is the overall certainty of the evidence of effects of the management that is guided by the test results? | | |
| Judgment | Research evidence | ADDITIONAL considerations |
| ○ Very low  ○ Low  ○ Moderate  ○ High  ● No included study  ○ Do not know | In general, treatment based on the result of true positives with antimicrobial agents is considered “good medical practice” and is expected to improve overall survival and other outcomes. On the contrary, if patients receive unnecessary treatment due to false positives, undesirable effects (such as adverse drug events) can be concerned. However, no studies have included evidence on the impact of treatment on final outcomes such as overall survival. |  |
| Certainty of evidence of test result/management How certain is the link between test results and management decisions? | | |
| Judgment | Research evidence | ADDITIONAL considerations |
| ○ Very low  ○ Low  ○ Moderate  ○ High  ● No included study  ○ Do not know | Since treatment of PCP is usually initiated promptly based on the test results, it seems reasonable to assume that there is a high degree of certainty regarding the relationship between the test results and management decisions. However, no studies have been included as evidence. |  |
| Certainty of effects What is the overall certainty of the evidence of effects of the test? | | |
| Judgment | Research evidence | ADDITIONAL considerations |
| ● Very low  ○ Low  ○ Moderate  ○ High  ○ No included study  ○ Do not know | Since the certainty of the evidence of the test’s accuracy is “Very low,” the certainty of the evidence of effects of the test becomes “Very low.” |  |
| Values Is there important uncertainty about or variability in how much people value the main outcomes? | | |
| Judgment | Research evidence | ADDITIONAL considerations |
| ○ Important uncertainty or variability  ● Possibly important uncertainty or variability  ○ Probably no important uncertainty or variability  ○ No important uncertainty or variability | The value of desirable effects such as true positives and undesirable effects such as false positives and adverse events may vary according to the values and experiences of individual healthcare providers and patients.  (The relative clinical weighting of false positives to true positives may vary depending on the values and experiences of individual health care providers and patients.) |  |
| Balance of effects Does the balance between desirable and undesirable effects favor the intervention or the comparison? | | |
| Judgment | Research evidence | ADDITIONAL considerations |
| ○ Favors the test  ● Probably favors the test  ○ Does not favor either the test or the comparison  ○ Probably favors the comparison  ○ Favors the comparison  ○ Varies  ○ Do not know | The net benefit of the test was calculated, considering the benefit of appropriate treatment due to true positive diagnosis, the harm of unnecessary treatment due to false positive diagnosis, and the adverse events of the test.  **Serum β-D-glucan**  **<Cutoff 80 pg/mL>**  Net benefit  (The number of patients who benefit from the test when performed on 1000 patients.)   | Prior probability | 5% | 10% | 20% | | --- | --- | --- | --- | | Treatment based on test results | -18 | 27 | 118 | | Treatment of all patients regardless of test results | -235 | -170 | -40 | | No treatment for all patients regardless of test results | 0 | 0 | 0 |   The relative clinical weight of false positives to true positives was set at 0.3, and the net benefit was calculated with a prior probability of 5-20%. Serious adverse events of the test were considered to be negligible. We compared the net benefit of treating patients based on the test results, treating all patients without testing, and not treating any patients without testing.  Assuming a prior probability of 5%, the net benefit of diagnosing by testing is estimated to be less than the net benefit of not treating everyone without testing. Assuming a prior probability of 10 or 20%, the net benefit of diagnosing by testing is estimated to be greater than the net benefit of not testing. Assuming a prior probability of 60% or more, the net benefit of diagnosing by testing is estimated to be less than the net benefit of treating everyone without testing.  Based on these results, there are likely to be many clinical situations in which there would be a net benefit from testing, and the balance of effects was judged to be “Probably favors the test.” | The net benefit (net benefit) was calculated as (number of true positives receiving appropriate treatment) - (number of false positives receiving unnecessary treatment) × (clinical weighting) - (number of serious adverse events of the test) for a sample of 1000 patients.  If adverse events were considered negligible, they were calculated as zero. |
| Acceptability Is the intervention acceptable to key stakeholders? | | |
| Judgment | Research evidence | ADDITIONAL considerations |
| ● Yes  ○ Probably yes  ○ Probably no  ○ No  ○ Varies  ○ Do not know | It is a commonly practiced medical procedure and considered acceptable. |  |
| Feasibility Is the intervention feasible to implement? | | |
| Judgment | Research evidence | ADDITIONAL considerations |
| ● Yes  ○ Probably yes  ○ Probably no  ○ No  ○ Varies  ○ Do not know | It is a commonly practiced medical procedure and considered feasible. |  |

# Summary of Judgment

|  | **Judgment** | | | | | | |
| --- | --- | --- | --- | --- | --- | --- | --- |
| **PROBLEM** | No | Probably no | Probably yes | Yes |  | Varies | Do not know |
| **DESIRABLE EFFECTS** | Trivial | Small | Moderate | Large |  | Varies | Do not know |
| **UNDESIRABLE EFFECTS** | Large | Moderate | Small | Trivial |  | Varies | Do not know |
| **CERTAINTY OF EVIDENCE OF TEST ACCURACY** | Very low | Low | Moderate | High |  |  | No included study |
| **CERTAINTY OF THE EVIDENCE OF TEST’S EFFECTS** | Very low | Low | Moderate | High |  |  | No included study |
| **CERTAINTY OF THE EVIDENCE OF MANAGEMENT’S EFFECTS** | Very low | Low | Moderate | High |  |  | No included study |
| **CERTAINTY OF THE EVIDENCE OF TEST RESULT/MANAGEMENT** | Very low | Low | Moderate | High |  |  | No included study |
| **CERTAINTY OF EFFECT** | Very low | Low | Moderate | High |  |  | No included study |
| **VALUES** | Important uncertainty or variability | Possibly important uncertainty or variability | Probably no important uncertainty or variability | No important uncertainty or variability |  |  |  |
| **BALANCE OF EFFECTS** | Favors the comparison | Probably favors the comparison | Does not favor either the test or the comparison | Probably favors the test | Favors the test | Varies | Do not know |
| **ACCEPTABILITY** | No | Probably no | Probably yes | Yes |  | Varies | Do not know |
| **FEASIBILITY** | No | Probably no | Probably yes | Yes |  | Varies | Do not know |

# Type of Recommendation

| Strong recommendation against the test | Conditional recommendation against the test | Conditional recommendation for either the test or the comparison | Conditional recommendation for the test | Strong recommendation for the test |
| --- | --- | --- | --- | --- |
| ○ | ○ | ○ | ● | ○ |

# Conclusions

| Recommendation |
| --- |
| **We conditionally recommend the use of serum β-D-glucan to differentiate the cause of ARDS (pneumocystis pneumonia) (Conditional recommendation/very low certainty of the evidence: GRADE: 2D).**  **Note: If the clinical situation (characteristics of the target patient, characteristics and timing of the test, prior probability, values of the patient and health care providers) changes, the balance of effects may change, and different options may be recommended.** |
|  |
| Justification |
| **Question**  Should serum β-D-glucan be used for identifying *Pneumocystis* pneumonia as the causative disease of ARDS?  **Patients**  Patients with ARDS  **Index test**  Serum β-D-glucan  **Purpose, role, and setting of the test**  Differential diagnosis in ARDS management in ER, ICU, or equivalent  **Medical practice based on test results**  If positive, the patient will be diagnosed with PCP and treated with appropriate agents. If negative, unnecessary treatment will be avoided, and additional testing for different diagnostic targets or follow-up will be performed.  **Summary of evidence**：  Serum β-D-glucan (three studies, 148 patients)  <Cutoff 80 pg/mL>  Integrated sensitivity: 0.84 (95% CI: 0.66-0.93), Integrated specificity: 0.79 (95% CI: 0.69-0.87)  **Certainty of the evidence**：  Certainty of the evidence of test accuracy was “Very low.”  **Values, balance of effects, acceptability, feasibility**：  When the balance of desirable and undesirable effects of testing for serum beta-D-glucan in patients with ARDS is considered, testing is generally supported. The certainty of the evidence is very low. Feasibility is not a problem, and it is a generally accepted medical practice.  **Panel meeting**  In the preliminary vote, the median score of “recommended text proposal” was 8, and the disagreement index was 0.192 by the modified Delphi method.  At the panel meeting, there was a question about the diagnostic performance of microscopic findings and polymerase chain reaction (PCR) tests of sputum and bronchoalveolar lavage fluid, but the answer was that these were used as reference standards in primary studies, and their diagnostic performance had not been examined. Finally, an agreement was achieved with the results of a preliminary vote.  **Additional considerations**：  If the prior probability is greater than 60%, the benefit of diagnosing and treating PCP regardless of the test result may outweigh the benefit of determining a treatment plan based on the test result. If the prior probability is less than 10%, the benefit of not performing the test may outweigh the benefit of performing the test. The balance of effects depends on the prior probability, the clinical weighting of false positives, and the performance of the test. Therefore, if the clinical situation (characteristics of the patient, characteristics and timing of the test, prior probability, the clinical weighting of false positives, and other values held by patients and caregivers) changes, the balance of effects may change, and different options may be recommended. |

| Subgroup considerations |
| --- |
| None |
| Implementation considerations |
| In the included studies, microscopic findings and PCR tests of sputum and bronchoalveolar lavage fluid were used as reference standards. These tests may be used in the definitive diagnosis of PCP. |

| Monitoring and evaluation |
| --- |
| After the publication of this medical guideline, it is necessary to collect and monitor information on the status of clinical use and problems in conducting the test using tools such as questionnaires. |
| Research priorities |
| The quality of the evidence was “Very low,” and higher quality diagnostic accuracy studies in ICU patients are desirable. If possible, randomized controlled trials are needed to assess whether testing improves important patient outcomes. |

**CQ10 Should serum β-D-glucan and galactomannan antigens of the blood or BAL fluid be used for identifying invasive pulmonary aspergillosis as the causative disease of ARDS?**

In this CQ, a systematic review and meta-analysis of the diagnostic performance of the above tests and a systematic review and meta-analysis of the frequency of adverse events of bronchoalveolar lavage were conducted. Therefore, the results of multiple search formulas and systematic reviews are included.

For the diagnostic performance of galactomannan antigen in bronchoalveolar lavage fluid, we reused part of a previously published systematic review and meta-analysis. (See references below)

Haydour Q, et al. Diagnosis of Fungal Infection. A Systematic Review and Meta-Analysis Suppoting American Thoracic Society Practice Guideline. Ann Am Thorac Soc. 2019; 16 (9): 1179-1188. PMID 31219341

In addition, the diagnostic performance of serum β-D glucan was updated by reusing some of the published systematic reviews and meta-analyses and conducting additional searches. (See references below)

White SK, Walker BS, Hanson KE, Schmidt RL. Diagnostic Accuracy of β-d-Glucan (Fungitell) Testing Among Patients With Hematologic Malignancies or Solid Organ Tumors: A Systematic Review and Meta-Analysis. Am J Clin Pathol. 2019 Feb 4;151(3):275-285.

1.Search strategy

Serum β-D-glucan

MEDLINE via PubMed （Search date: 2020/9/18）

| #1 | BDG*.tw. |
| --- | --- |
| #2 | Fungitel*.tw. |
| #3 | Cape Cod.tw. |
| #4 | Fungitec*.tw. |
| #5 | Seikagaku.tw. |
| #6 | Wake Test.tw. |
| #7 | (Wako* or Waco*).tw. |
| #8 | #1 or #2 or #3 or #4 or #5 or #6 or #7 |
| #9 | exp beta-Glucans/ |
| #10 | Glucans/ |
| #11 | D-glucan*.tw. |
| #12 | #9 or #10 or #11 |
| #13 | exp "Sensitivity and Specificity"/ |
| #14 | (sensitivit* or specificit*).tw. |
| #15 | predictive value*.tw. |
| #16 | diagnosis.fs. |
| #17 | analysis.fs. |
| #18 | Reagent Kits, Diagnostic/ |
| #19 | #13 or #14 or #15 or #16 or #17 or #18 |
| #20 | #12 and #19 |
| #21 | exp Mycoses/ |
| #22 | exp Fungi/ |
| #23 | fungal.tw. |
| #24 | fungus.tw. |
| #25 | mycos*.tw. |
| #26 | mycot*.tw. |
| #27 | aspergill*.tw. |
| #28 | pneumocystis.tw. |
| #29 | #21 or #22 or #23 or #24 or #25 or #26 or #27 or #28 |
| #30 | #12 and #29 |
| #31 | #8 or #20 or #30 |
| #32 | exp animals/ not humans.sh. |
| #33 | #31 not #32 |
| #34 | limit #33 to yr="2017 -Current" |

EMBASE （Search date: 2020/9/16）

| S1 | TI,AB(BDG*) |
| --- | --- |
| S2 | TI,AB(Fungitel*) |
| S3 | (TI,AB(Cape Cod)) |
| S4 | TI,AB(Fungitec*) |
| S5 | TI,AB(Seikagaku) |
| S6 | (TI,AB("Wake Test")) |
| S7 | (TI,AB(Wako* OR Waco*)) |
| S8 | (S1 or S2 or S3 or S4 or S5 or S6 or S7) |
| S9 | (EMB.EXACT.EXPLODE("beta glucan")) |
| S10 | EMB.EXACT("glucan") |
| S11 | TI,AB(D-glucan*) |
| S12 | (S9 or S10 or S11) |
| S13 | (EMB.EXACT("sensitivity and specificity")) |
| S14 | (TI,AB(sensitivit* OR specificit*)) |
| S15 | (TI,AB(predictive value*)) |
| S16 | QU(DI) |
| S17 | (EMB.EXACT("diagnostic kit")) |
| S18 | (EMB.EXACT.EXPLODE("diagnostic procedure")) |
| S19 | (S13 or S14 or S15 or S16 or S17 or S18) |
| S20 | (S12 and S19) |
| S21 | EMB.EXACT.EXPLODE("mycosis") |
| S22 | EMB.EXACT.EXPLODE("fungus") |
| S23 | TI,AB(fungal) |
| S24 | TI,AB(fungus) |
| S25 | TI,AB(mycos*) |
| S26 | TI,AB(mycot*) |
| S27 | TI,AB(aspergill*) |
| S28 | TI,AB(pneumocystis) |
| S29 | (S21 or S22 or S23 or S24 or S25 or S26 or S27 or S28) |
| S30 | (S12 and #29) |
| S31 | (S8 or S20 or S30) |
| S32 | (ANIMAL(YES) NOT HUMAN(YES)) |
| S33 | (S31 not S32) |
| S34 | (S33 AND YR(>=2017)) |

Adverse events of bronchoalveolar lavage

PubMed search strategy（Search date: 2020/6/1）

|  | 検索式 |
| --- | --- |
| #1 | Respiratory Distress Syndrome, Adult [mh] |
| #2 | Acute lung injury [mh] |
| #3 | ALI [tiab] OR ARDS [tiab] |
| #4 | Acute [tiab] AND (lung injur* [tiab] OR respiratory distress [tiab] OR respiratory failure[tiab]) |
| #5 | (Severe [tiab] OR critical*[tiab]) AND (respiratory[tiab] OR hypox* [tiab]) |
| #6 | #1 or #2 or #3 or #4 or #5 |
| #7 | Bronchoscopy/ adverse effects [mh] OR Bronchoscopy/ complications [mh] |
| #8 | Bronchoscop* [tiab] |
| #9 | Bronchoalveolar Lavage/ adverse effects [mh] OR Bronchoalveolar Lavage/ complications [mh] |
| #10 | Bronchoalveolar Lavage [tiab] |
| #11 | BAL [tiab] OR BALF [tiab] |
| #12 | #7 or #8 or #9 or #10 or #11 |
| #13 | #6 and #12 |
| #14 | Animals [mh] NOT human [mh] |
| #15 | #13 NOT #14 |

CENTRAL search strategy（Search date: 2020/6/1）

|  | 検索式 |
| --- | --- |
| #1 | MeSH descriptor: [Respiratory Distress Syndrome, Adult] explode all trees |
| #2 | MeSH descriptor: [Acute Lung Injury] explode all trees |
| #3 | (ALI OR ARDS):ti,ab,kw |
| #4 | (Acute):ti,ab,kw |
| #5 | (lung NEXT injur*):ti,ab,kw |
| #6 | (“respiratory distress”):ti,ab,kw |
| #7 | (“respiratory failure”):ti,ab,kw |
| #8 | #4 AND (#5 OR #6 OR #7) |
| #9 | (Severe:ti,ab OR critical*:ti,ab) AND (respiratory:ti,ab OR hypox*:ti,ab) |
| #10 | #1 OR #2 OR #3 OR #8 OR #9 |
| #11 | MeSH descriptor: [Bronchoscopy] explode all trees |
| #12 | (Bronchoscop*):ti,ab,kw |
| #13 | MeSH descriptor: [Bronchoalveolar Lavage] explode all trees |
| #14 | (“Bronchoalveolar Lavage”):ti,ab,kw |
| #15 | (BAL OR BALF):ti,ab,kw |
| #16 | #11 OR #12 OR #13 OR #14 OR #15 |
| #17 | #10 AND #16 |
| #18 | ([mh Animals] NOT [mh human]):ti,ab,kw |
| #19 | #17 NOT #18 |

Galactomannan antigen (blood and bronchoalveolar lavage)

See “search strategy (supplementary material)" in the literature below.

Haydour Q, et al. Diagnosis of Fungal Infection. A Systematic Review and Meta-Analysis Suppoting American Thoracic Society Practice Guideline. Ann Am Thorac Soc. 2019; 16 (9): 1179-1188. PMID 31219341

1. Flow diagram

**Identification**

- 1. Serum β-D-glucan

3 Studies covered by existing systematic reviews

3524 records identified through database searching

Medline via Ovid (n=1088)

EMBASE (ProQuest) (n=2436)

12 Studies included in quantitative synthesis

(meta-analysis)

9 Studies Invasive pulmonary aspergillosis

3 Studies Pneumocystis jirovecii pneumonia

12 Studies included in qualitative synthesis

3524 records identified through database searching

2807 records after duplicates removed

71 Full-text articles assessed for eligibility

62 Full-text articles excluded, with reasons:

・Wrong language (n=2)

・Wrong study design (n=8)

・Wrong publication type (n=29)

・Wrong population (n=4)

・Wrong index test (n=3)

・Others (n=16)

2736 records excluded

Duplicates

n=717

**Screening**

**Eligibility**

**Included**

2.2 Adverse events of bronchoalveolar lavage

**Identification**

15 Studies included in qualitative synthesis

374 Full-text articles assessed for eligibility

4193 records after duplicates removed

4348 records identified through database searching

4348 records identified through database searching

Medline via PubMed (n=3929)

CENTRAL (n=419)

0 additional records identified through other sources

15 Studies included in quantitative synthesis

(meta-analysis)*

359 Full-text articles excluded, with reasons:

・Wrong language (n=18)

・Wrong study design (n=18)

・Wrong population (n=30)

・Wrong index test (n=6)

・Wrong reference test (n=285)

・Others (n=2)

Duplicates

n=155

3819 records excluded

**Included**

**Eligibility**

**Screening**

2.3 Galactomannan antigen (blood and bronchoalveolar lavage)

See “Figure 1” in the reference below.

Haydour Q, et al. Diagnosis of Fungal Infection. A Systematic Review and Meta-Analysis Suppoting American Thoracic Society Practice Guideline. Ann Am Thorac Soc. 2019; 16 (9): 1179-1188. PMID 31219341

In addition, there were some doubts about the contents of the previous SR, and when we contacted the editorial office, we found that the contents were incorrect. The data of integrated sensitivity and integrated specificity are the corrected data provided by the author. In addition, the number of relevant papers in the text of the preceding SR is different, but the one in this guideline is the correct one.

1. Risk of bias

Serum β-D-glucan (Cutoff 80 pg/mL)


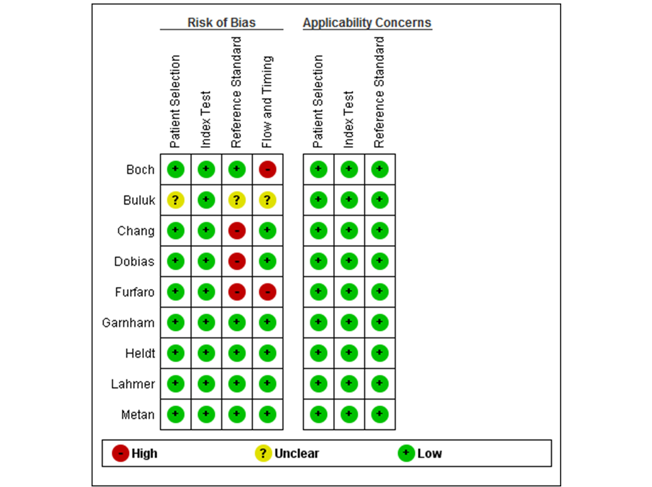


Adverse events of bronchoalveolar lavage

Mortality or serious disability


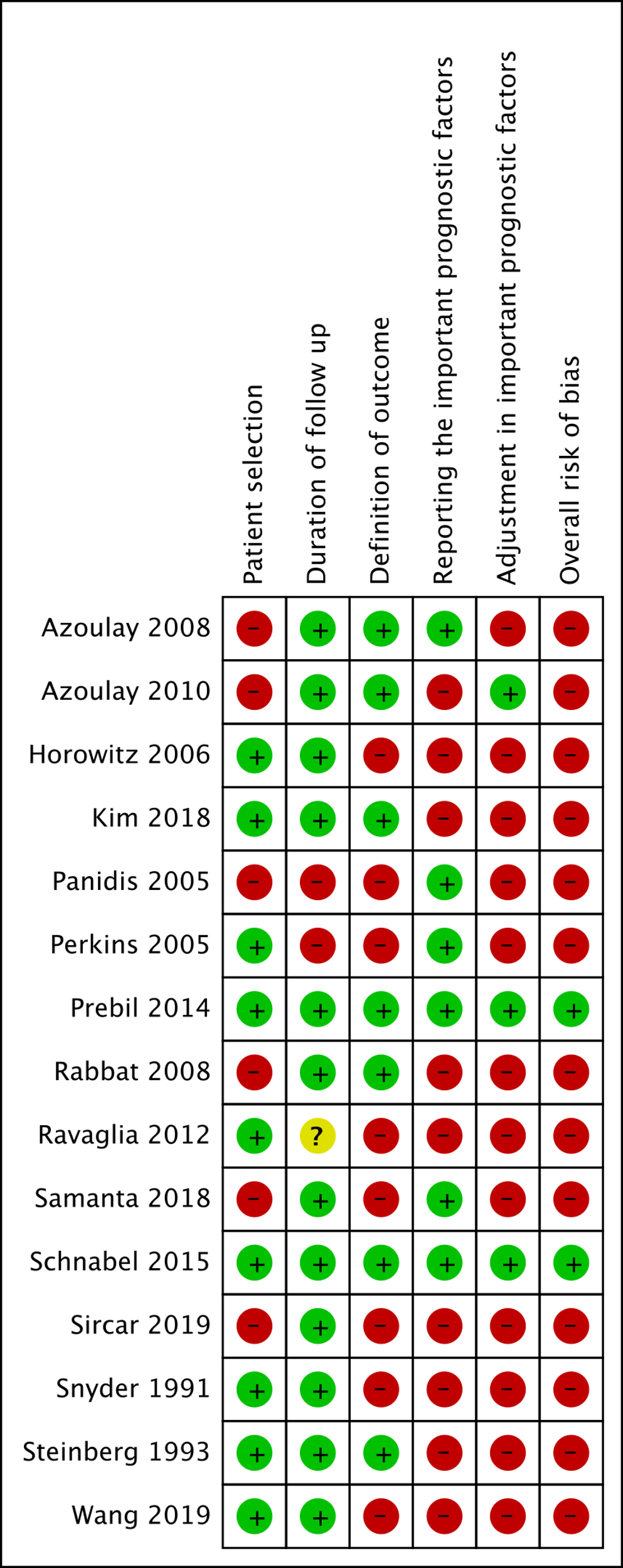


Severe complications of the respiratory system


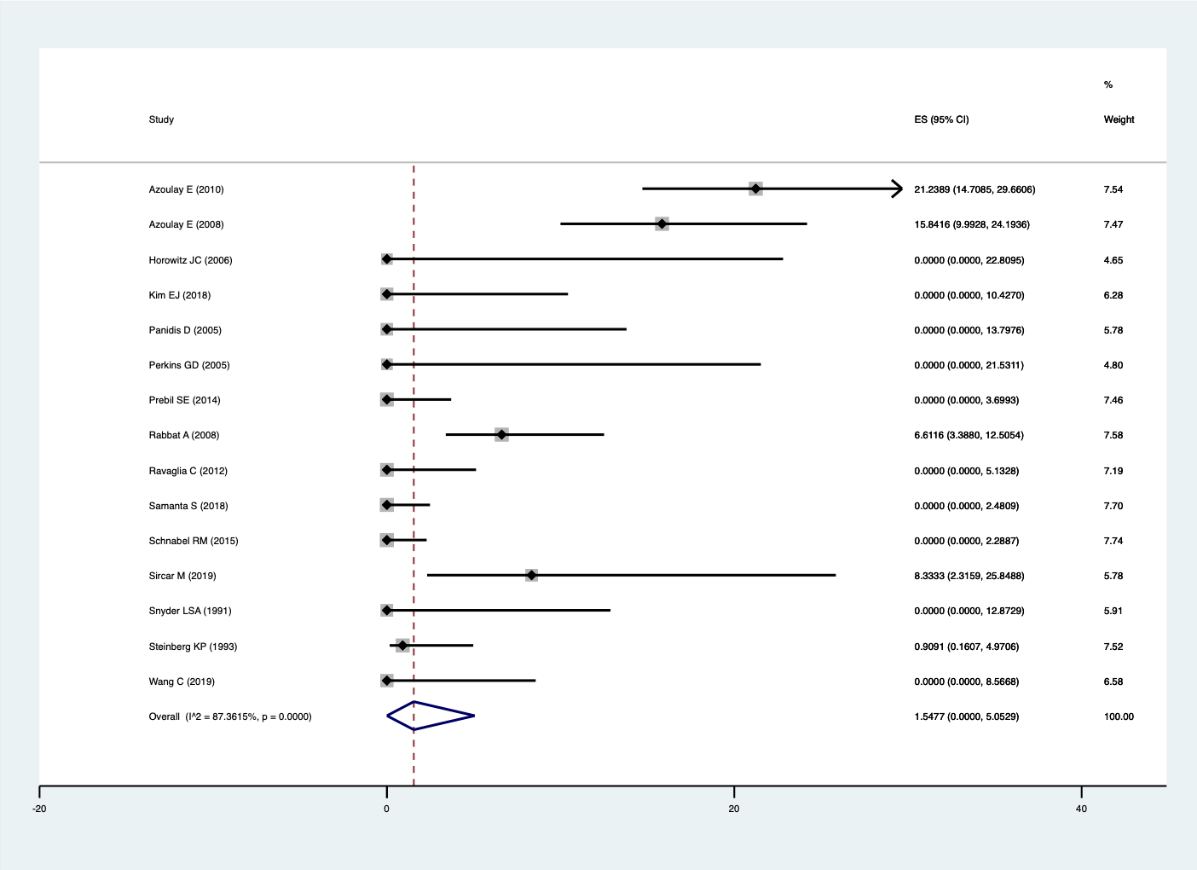


Severe complications of the cardiovascular system


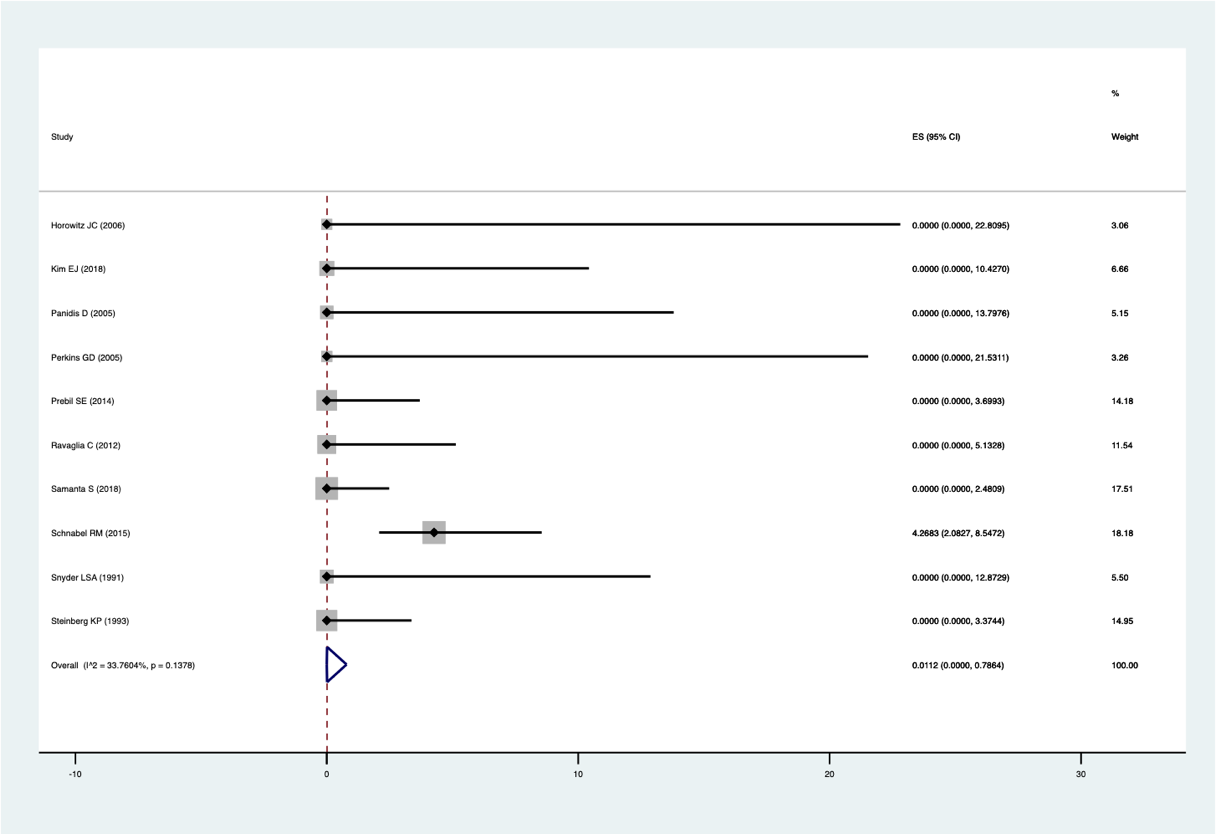


Major bleeding


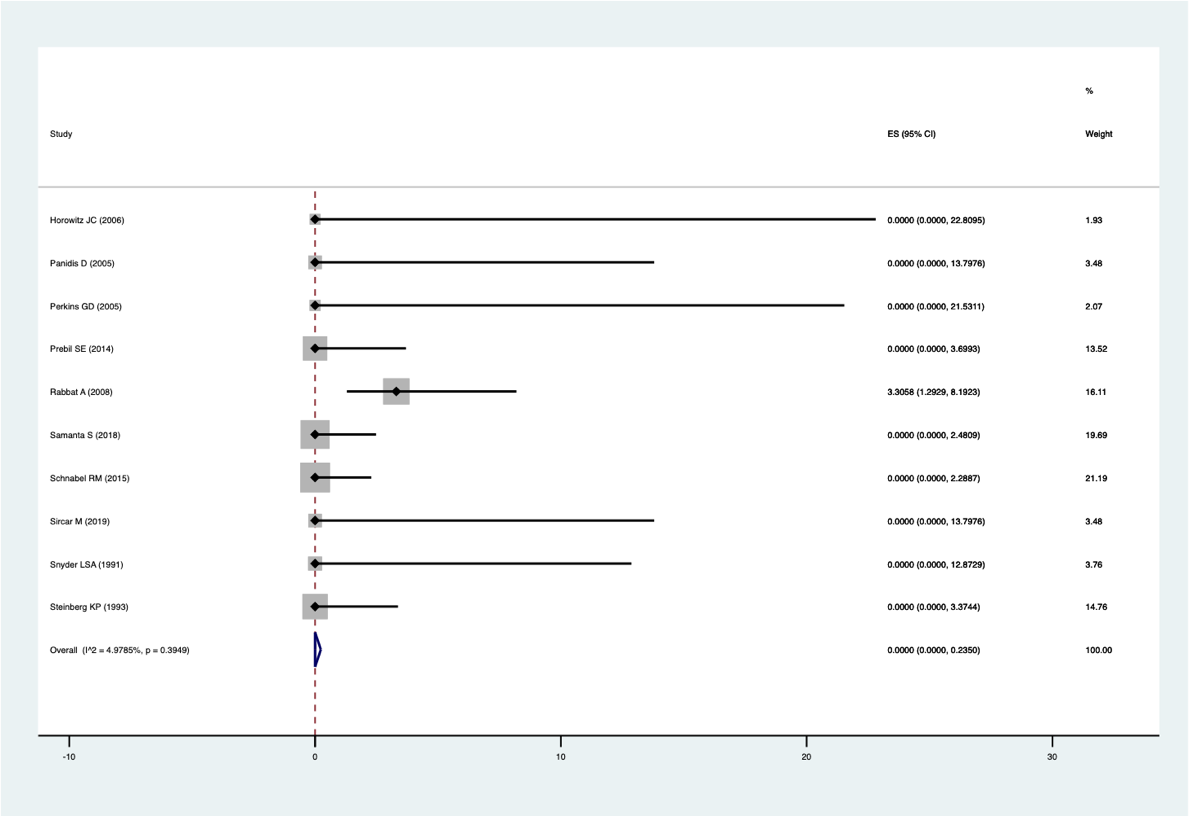


Galactomannan antigen (blood) (Cutoff 0.5 ODI)

Galactomannan antigen (blood) (Cutoff 1.0 ODI)

Galactomannan antigen (blood) (Cutoff 1.5 ODI)

Galactomannan antigen (bronchoalveolar lavage) (Cutoff 0.5 ODI)

Galactomannan antigen (bronchoalveolar lavage) (Cutoff 1.0 ODI)

See “Figure E1” in the reference below.

Haydour Q, et al. Diagnosis of Fungal Infection. A Systematic Review and Meta-Analysis Suppoting American Thoracic Society Practice Guideline. Ann Am Thorac Soc. 2019; 16 (9): 1179-1188. PMID 31219341

1. Forest plot

Serum β-D-glucan (Cutoff 80 pg/mL)


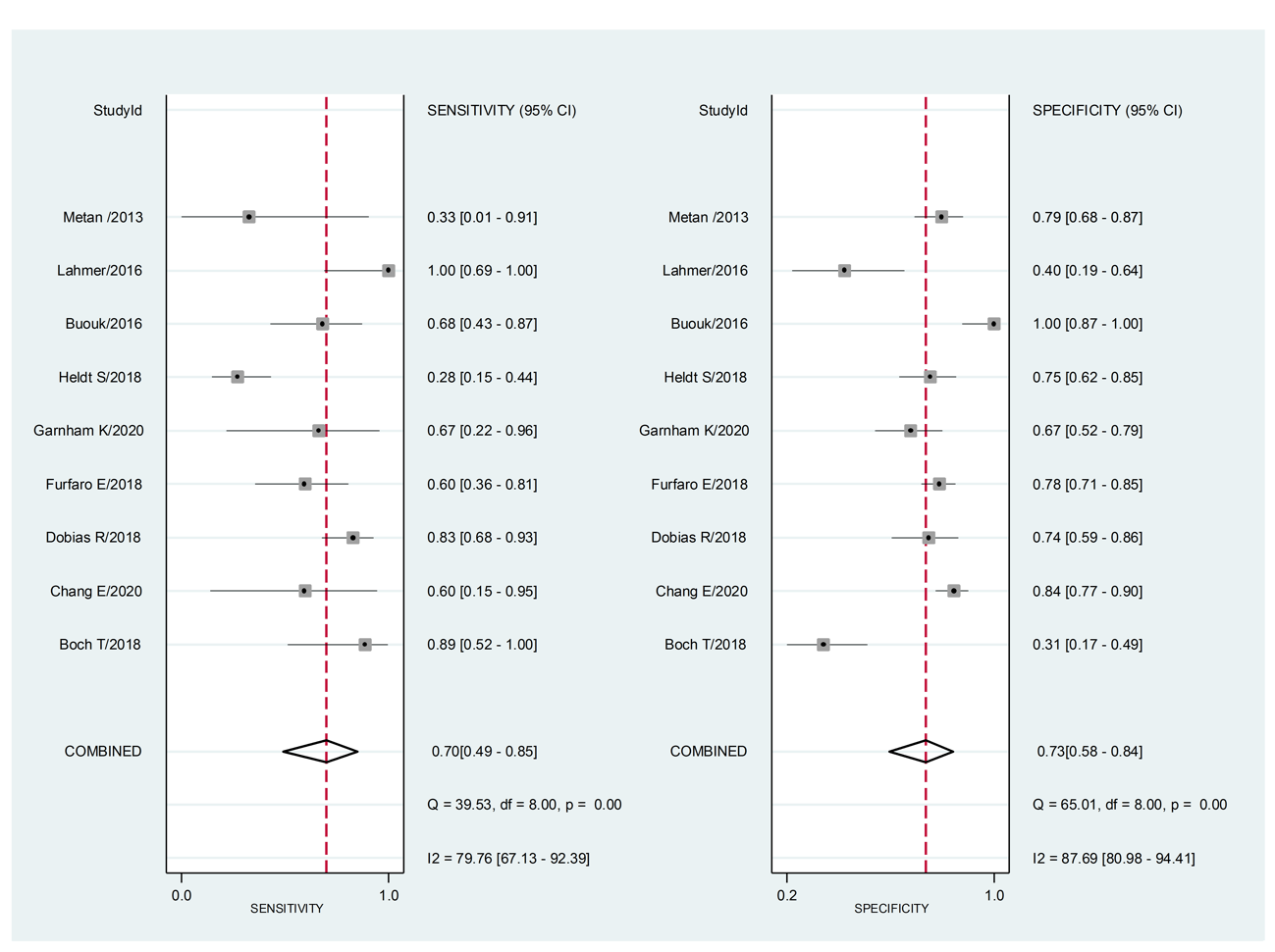


Adverse events of bronchoalveolar lavage

Mortality or serious disability


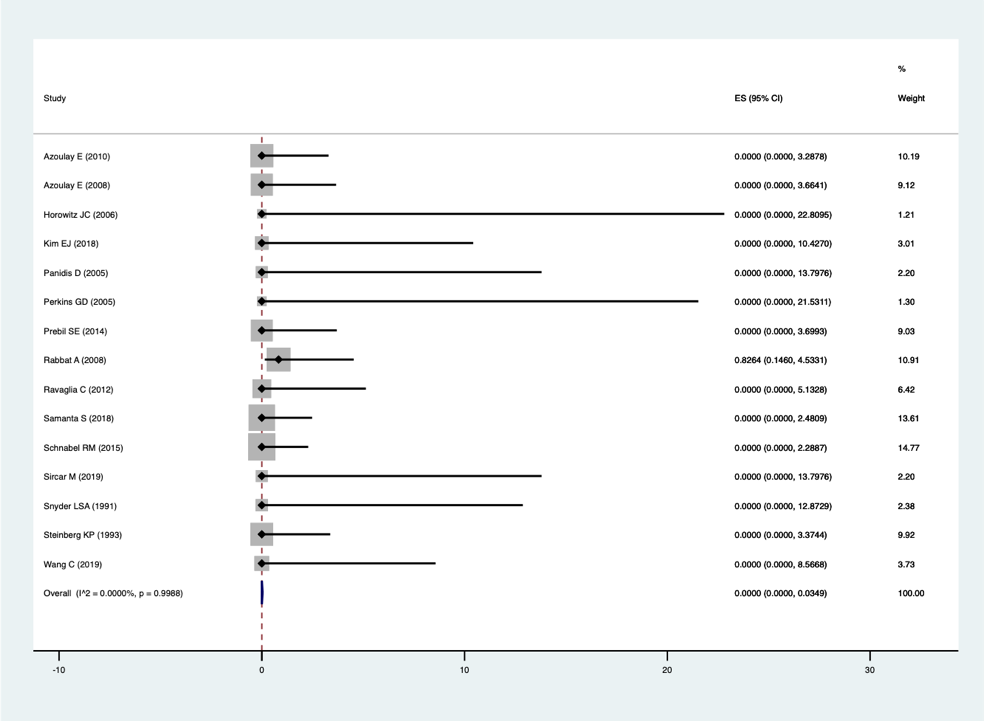


Galactomannan antigen (blood) (Cutoff 0.5 ODI)

Galactomannan antigen (blood) (Cutoff 1.0 ODI)

Galactomannan antigen (blood) (Cutoff 1.5 ODI)

Galactomannan antigen (bronchoalveolar lavage) (Cutoff 0.5 ODI)

Galactomannan antigen (bronchoalveolar lavage) (Cutoff 1.0 ODI)

See “Figure E1” in the reference below.

Haydour Q, et al. Diagnosis of Fungal Infection. A Systematic Review and Meta-Analysis Suppoting American Thoracic Society Practice Guideline. Ann Am Thorac Soc. 2019; 16 (9): 1179-1188. PMID 31219341

1. Evidence profile

Index test: Serum β-D-glucan (Cutoff 80 pg/mL)

| | Sensitivity | 0.70 (95% CI: 0.49 to 0.85) | | --- | --- | | Specificity | 0.73 (95% CI: 0.58 to 0.84) | |  | | Prior probability | 5% | 10% | 20% | | --- | --- | --- | --- | |  |
| --- | --- | --- | --- | --- | --- | --- | --- | --- | --- | --- | --- |

| Outcome | No. of studies (patients) | Study design | Assessment of certainty | | | | | Prevalence in 1000 patients | | | Certainty of the evidence |
| --- | --- | --- | --- | --- | --- | --- | --- | --- | --- | --- | --- |
|  |  |  | Risk of bias | Indirectness | Inconsistency | Imprecision | Publication bias | Prior probability 5% | Prior probability 10% | Prior probability 20% |  |
| True Positive | 9 (757) | Cross-sectional study, Cohort study | Serious ^a^ | Serious ^b^ | Very serious ^c^ | Very serious ^d^ | None | 35 (25 to 43) | 70 (49 to 85) | 140 (98 to 170) | ⨁◯◯◯ Very low |
| False Negative |  |  |  |  |  |  |  | 15 (7 to 25) | 30 (15 to 51) | 60 (30 to 102) |  |
| True Negative | 9 (757) | Cross-sectional study, Cohort study | Serious ^a^ | Serious ^b^ | Very serious ^c^ | Serious ^e^ | None | 694 (551 to 798) | 657 (522 to 756) | 584 (464 to 672) | ⨁◯◯◯ Very low |
| False Positive |  |  |  |  |  |  |  | 256 (152 to 399) | 243 (144 to 378) | 216 (128 to 336) |  |

a. Regarding the risk of bias in the QUADAS-2 tool, the proportion of studies judged to have a high or unknown risk of bias was 1/9 for patient selection, 0/9 for index test, 4/9 for reference standard, and 3/9 for flow and timing. Thus, a certain proportion of studies was determined to indicate a high or unknown risk, which was judged to be “Serious.”

b. Regarding the concerns about applicability in the QUADAS-2 tool, the proportion of studies judged to have a high or unknown concern about the applicability was 0/9 for patient selection, 0/9 for index test, 0/9 for reference standard. Thus, the applicability was considered to be maintained because in less than one category, the proportion of studies with high or unknown concerns about applicability was approximately more than half. However, in this systematic review, we conducted the literature search not only for patients with ARDS but also for those with acute respiratory failure. Owing to this gap from the ideal systematic review question that should be set, the results to the clinical situation assumed by guideline readers have limited direct applicability. Therefore, we judged the overall applicability to be "Serious.”

c. We visually assessed the variability in the results of each study included in the systematic review using forest plots.

d. The results may be imprecise because the total number of patients included in the systematic review was below the optimal informative threshold. Additionally, we examined the net benefit of the test (difference between true positives and weighted false positives) if one false positive was considered acceptable for every 0.3 true positive. When the prevalence rate was set at 10%, the net benefit of the test differed at the upper and lower limits of the confidence interval of the integrated sensitivity, which could have changed the clinical judgment. Therefore, the overall uncertainty was judged to be “Very serious.”

e. The total number of patients included in the systematic review was above the threshold for obtaining optimal information. Additionally, the net benefit of the test (difference between true negatives and weighted false negatives) was examined if one false positive is considered acceptable for every 0.3 true positive. When the prevalence rate was set at 10%, the net benefit of the test differed at the upper and lower limits of the confidence interval of the integrated specificity, which could have changed the clinical judgment. Therefore, the overall uncertainty was judged to be “Serious.”

Index test: Galactomannan antigen (blood) (Cutoff 0.5 ODI)

| | Sensitivity | 0.75 (95% CI: 0.65 to 0.83) | | --- | --- | | Specificity | 0.85 (95% CI: 0.77 to 0.90) | |  | | Prior probability | 5% | 10% | 20% | | --- | --- | --- | --- | |  |
| --- | --- | --- | --- | --- | --- | --- | --- | --- | --- | --- | --- |

| Outcome | No. of studies (patients) | Study design | Assessment of certainty | | | | | Prevalence in 1000 patients | | | Certainty of the evidence |
| --- | --- | --- | --- | --- | --- | --- | --- | --- | --- | --- | --- |
|  |  |  | Risk of bias | Indirectness | Inconsistency | Imprecision | Publication bias | Prior probability 5% | Prior probability 10% | Prior probability 20% |  |
| True Positive | 28 (518) | Cross-sectional study, Cohort study | Serious ^a^ | Serious ^b^ | Very serious ^c^ | Very serious ^d^ | None | 38 (33 to 42) | 75 (65 to 83) | 150 (130 to 166) | ⨁◯◯◯ Very low |
| False Negative |  |  |  |  |  |  |  | 12 (8 to 17) | 25 (17 to 35) | 50 (34 to 70) |  |
| True Negative | 28 (4259) | Cross-sectional study, Cohort study | Serious ^a^ | Serious ^b^ | Very serious ^c^ | Not serious ^e^ | None | 808 (731 to 855) | 765 (693 to 810) | 680 (616 to 720) | ⨁◯◯◯ Very low |
| False Positive |  |  |  |  |  |  |  | 142 (95 to 219) | 135 (90 to 207) | 120 (80 to 184) |  |

a. Regarding the risk of bias in the QUADAS-2 tool, the proportion of studies judged to have a high or unknown risk of bias was 12/28 for patient selection, 10/28 for index test, 12/28 for reference standard, and 3/28 for flow and timing. Thus, a certain proportion of studies was determined to indicate a high or unknown risk, which was judged to be “Serious.”

b. Regarding the concerns about the applicability in the QUADAS-2 tool, the proportion of studies judged to have a high or unknown concern about the applicability was 12/28 for patient selection, 4/28 for index test, 3/28 for reference standard. Thus, the applicability was considered to be maintained because there was less than one category in which the proportion of studies with high or unknown concerns about applicability was approximately more than half. However, in this systematic review, we conducted the literature search not only for patients with ARDS but also for those with invasive pulmonary aspergillosis. Owing to this gap from the ideal systematic review question that should be set, the results to the clinical situation assumed by guideline readers have limited direct applicability. Therefore, we judged the overall applicability to be “Serious.”

c. We visually assessed the variability in the results of each study included in the systematic review using forest plots.

d. Results may be imprecise because the total number of patients included in the systematic review is below the optimal informative threshold. Additionally, we examined the net benefit of the test (difference between true positives and weighted false positives) if one false positive was considered acceptable for every 0.3 true positive. When the prevalence rate was set at 5–10%, the net benefit of the test differed, which could have changed the clinical judgment. Therefore, the overall uncertainty was judged to be “Very serious.”

e. The total number of patients included in the systematic review was above the threshold for obtaining optimal information. Additionally, the net benefit of the test (difference between true negatives and weighted false negatives) was examined if one false positive is considered acceptable for every 0.3 true positive. When the prevalence was set at 10–20%, the net benefit of the test did not differ between the upper and lower limits of the confidence interval of the integrated specificity, and clinical judgment was not expected to change. Consequently, the overall imprecision was judged to be “Not serious.”

Index test: Galactomannan antigen (blood) (Cutoff 1.0 ODI)

| | Sensitivity | 0.76 (95% CI: 0.60 to 0.91) | | --- | --- | | Specificity | 0.88 (95% CI: 0.79 to 0.94) | |  | | Prior probability | 5% | 10% | 20% | | --- | --- | --- | --- | |  |
| --- | --- | --- | --- | --- | --- | --- | --- | --- | --- | --- | --- |

| Outcome | No. of studies (patients) | Study design | Assessment of certainty | | | | | Prevalence in 1000 patients | | | Certainty of the evidence |
| --- | --- | --- | --- | --- | --- | --- | --- | --- | --- | --- | --- |
|  |  |  | Risk of bias | Indirectness | Inconsistency | Imprecision | Publication bias | Prior probability 5% | Prior probability 10% | Prior probability 20% |  |
| True Positive | 8 (145) | Cross-sectional study, Cohort study | Serious ^a^ | Serious ^b^ | Very serious ^c^ | Very serious ^d^ | None | 38 (28 to 44) | 76 (57 to 88) | 152 (114 to 176) | ⨁◯◯◯ Very low |
| False Negative |  |  |  |  |  |  |  | 12 (6 to 22) | 24 (12 to 43) | 48 (24 to 86) |  |
| True Negative | 8 (1237) | Cross-sectional study, Cohort study | Serious ^a^ | Serious ^b^ | Very serious ^c^ | Not serious ^e^ | None | 836 (751 to 884) | 792 (711 to 837) | 704 (632 to 744) | ⨁◯◯◯ Very low |
| False Positive |  |  |  |  |  |  |  | 114 (66 to 199) | 108 (63 to 189) | 96 (56 to 168) |  |

a. Regarding the risk of bias in the QUADAS-2 tool, the proportion of studies judged to have a high or unknown risk of bias is 3/8 for patient selection, 2/8 for index test, 3/8 for reference standard, and 4/8 for flow and timing. Thus, a certain proportion of studies is determined to denote a high or unknown risk, which was judged to be “Serious.”

b. Regarding the concerns about the applicability in the QUADAS-2 tool, the proportion of studies judged to have a high or unknown concern about the applicability was 1/8 for patient selection, 0/8 for index test, and 3/8 for reference standard. Thus, the applicability was considered to be maintained because there was less than one category in which the proportion of studies with high or unknown concerns about applicability was approximately more than half. However, in this systematic review, we conducted the literature search not only for patients with ARDS but also for those with invasive pulmonary aspergillosis. Owing to this gap from the ideal systematic review question that should be set, the results to the clinical situation assumed by guideline readers have limited direct applicability. Therefore, we judged the overall applicability to be “Serious.”

c. We visually assessed the variability in the results of each study included in the systematic review using forest plots.

d. The results may be imprecise because the total number of patients included in the systematic review was below the optimal informative threshold. Moreover, we examined the net benefit of the test (difference between true positives and weighted false positives) if one false positive was considered acceptable for every 0.3 true positive. When the prevalence rate was set at 5%, the net benefit of the test differed at the upper and lower limits of the confidence interval of the integrated sensitivity, which can change the clinical judgment. Therefore, the overall uncertainty was judged to be “Very serious.”

e. The total number of patients included in the systematic review was above the threshold for obtaining optimal information. Additionally, the net benefit of the test (difference between true negatives and weighted false negatives) was examined if one false positive is considered acceptable for every 0.3 true positive. When the prevalence was set at 10–20%, the net benefit of the test did not differ between the upper and lower limits of the confidence interval of the integrated specificity, and clinical judgment was not expected to change. Consequently, the overall imprecision was judged to be “Not serious.”

Index test: Galactomannan antigen (blood) (Cutoff 1.5 ODI)

| | Sensitivity | 0.59 (95% CI: 0.44 to 0.72) | | --- | --- | | Specificity | 0.95 (95% CI: 0.90 to 0.97) | |  | | Prior probability | 5% | 10% | 20% | | --- | --- | --- | --- | |  |
| --- | --- | --- | --- | --- | --- | --- | --- | --- | --- | --- | --- |

| Outcome | No. of studies (patients) | Study design | Assessment of certainty | | | | | Prevalence in 1000 patients | | | Certainty of the evidence |
| --- | --- | --- | --- | --- | --- | --- | --- | --- | --- | --- | --- |
|  |  |  | Risk of bias | Indirectness | Inconsistency | Imprecision | Publication bias | Prior probability 5% | Prior probability 10% | Prior probability 20% |  |
| True Positive | 14 (272) | Cross-sectional study, Cohort study | Serious ^a^ | Serious ^b^ | Very serious ^c^ | Serious ^d^ | None | 30 (22 to 36) | 59 (44 to 72) | 118 (88 to 144) | ⨁◯◯◯ Very low |
| False Negative |  |  |  |  |  |  |  | 20 (14 to 28) | 41 (28 to 56) | 82 (56 to 112) |  |
| True Negative | 14 (2256) | Cross-sectional study, Cohort study | Serious ^a^ | Serious ^b^ | Very serious ^c^ | Not serious ^e^ | None | 903 (855 to 922) | 855 (810 to 873) | 760 (720 to 776) | ⨁◯◯◯ Very low |
| False Positive |  |  |  |  |  |  |  | 47 (28 to 95) | 45 (27 to 90) | 40 (24 to 80) |  |

a. Regarding the risk of bias in the QUADAS-2 tool, the proportion of studies judged to have a high or unknown risk of bias was 9/14 for patient selection, 8/14 for index test, 9/14 for reference standard, and 6/14 for flow and timing. Thus, a certain proportion of studies was determined to denote a high or unknown risk, which was judged to be “Serious.”

b. Regarding the concerns about the applicability in the QUADAS-2 tool, the proportion of studies judged to have a high or unknown concern about the applicability was 8/14 for patient selection, 2/14 for index test, 4/12 for reference standard. Thus, the applicability was considered to be maintained because there was less than one category in which the proportion of studies with high or unknown concerns about applicability was approximately more than half. However, in this systematic review, we conducted the literature search not only for patients with ARDS but also for those with invasive pulmonary aspergillosis. Owing to this gap from the ideal systematic review question that should be set, the results to the clinical situation assumed by guideline readers have limited applicability. Therefore, we judged the overall applicability to be “Serious.”

c. We visually assessed the variability in the results of each study included in the systematic review using forest plots.

d. The results may be imprecise because the total number of patients included in the systematic review is below the optimal informative threshold. Additionally, we examined the net benefit of the test (difference between true positives and weighted false positives) if one false positive was considered acceptable for every 0.3 true positive. When the prevalence was set at 5–10%, the net benefit of the test did not differ between the upper and lower limits of the confidence interval of the integrated sensitivity, and clinical judgment was not expected to change. Therefore, the overall imprecision was judged to be “Serious.”

e. The total number of patients included in the systematic review was above the threshold for obtaining optimal information. Additionally, the net benefit of the test (difference between true negatives and weighted false negatives) was examined if one false positive was considered acceptable for every 0.3 true positive. When the prevalence was set at 10–20%, the net benefit of the test did not differ between the upper and lower limits of the confidence interval of the integrated specificity, and clinical judgment was not expected to change. Resultantly, the overall imprecision was judged to be “Not serious.”

Index test: Galactomannan antigen (bronchoalveolar lavage) (Cutoff 0.5 ODI)

| | Sensitivity | 0.88 (95% CI: 0.75 to 1.00) | | --- | --- | | Specificity | 0.81 (95% CI: 0.71 to 0.91) | |  | | Prior probability | 5% | 10% | 20% | | --- | --- | --- | --- | |  |
| --- | --- | --- | --- | --- | --- | --- | --- | --- | --- | --- | --- |

| Outcome | No. of studies (patients) | Study design | Assessment of certainty | | | | | Prevalence in 1000 patients | | | Certainty of the evidence |
| --- | --- | --- | --- | --- | --- | --- | --- | --- | --- | --- | --- |
|  |  |  | Risk of bias | Indirectness | Inconsistency | Imprecision | Publication bias | Prior probability 5% | Prior probability 10% | Prior probability 20% |  |
| True Positive | 12 (1123) | Cross-sectional study, Cohort study | Very serious ^a^ | Serious ^b^ | Very serious ^c^ | Serious ^d^ | None | 44 (38 to 50) | 88 (75 to 100) | 176 (150 to 200) | ⨁◯◯◯ Very low |
| False Negative |  |  |  |  |  |  |  | 6 (0 to 12) | 12 (0 to 25) | 24 (0 to 50) |  |
| True Negative | 12 (1123) | Cross-sectional study, Cohort study | Very serious ^a^ | Serious ^b^ | Very serious ^c^ | Not serious ^e^ | None | 770 (675 to 864) | 729 (639 to 819) | 648 (568 to 728) | ⨁◯◯◯ Very low |
| False Positive |  |  |  |  |  |  |  | 180 (86 to 275) | 171 (81 to 261) | 152 (72 to 232) |  |

a. Regarding the risk of bias in the QUADAS-2 tool, the proportion of studies judged to have a high or unknown risk of bias was 7/12 for patient selection, 11/12 for index test, 11/12 for reference standard, and 7/12 for flow and timing. Thus, the proportion of studies with high or unknown risk in two or more categories was approximately more than 2/3, which was judged to be “Very serious.”

b. Regarding the concerns about the applicability in the QUADAS-2 tool, the proportion of studies judged to have a high or unknown concern about the applicability was 0/12 for patient selection, 0/12 for index test, and 0/12 for reference standard. Thus, the applicability was considered to be maintained because there was less than one category in which the proportion of studies with high or unknown concerns about applicability was approximately more than half. However, in this systematic review, we conducted a literature search not only for patients with ARDS but also for those with invasive pulmonary aspergillosis. Owing to this gap from the ideal systematic review question that should be set, the results to the clinical situation assumed by guideline readers have limited applicability. Therefore, we judged the overall applicability to be “Serious.”

c. We visually assessed the variability in the results of each study included in the systematic review using forest plots.

d. The total number of patients included in the systematic review was above the threshold for obtaining optimal information. Additionally, we examined the net benefit of the test (difference between true positives and weighted false positives) if one false positive is considered acceptable for every 0.3 true positive. When the prevalence rate was set at 5–10%, the net benefit of the test differed, which could have changed the clinical judgment. Therefore, the overall uncertainty was judged to be “Serious.”

e. The total number of patients included in the systematic review is above the threshold for obtaining optimal information. Additionally, the net benefit of the test (difference between true negatives and weighted false negatives) is examined if one false positive is considered acceptable for every 0.3 true positive. When the prevalence was set at 10–20%, the net benefit of the test did not differ between the upper and lower limits of the confidence interval of the integrated specificity, and clinical judgment was not expected to change. Consequently, the overall imprecision was judged to be “Not serious.”

Index test: Galactomannan antigen (bronchoalveolar lavage) (Cutoff 1.0 ODI)

| | Sensitivity | 0.78 (95% CI: 0.61 to 0.95) | | --- | --- | | Specificity | 0.93 (95% CI: 0.87 to 0.98) | |  | | Prior probability | 5% | 10% | 20% | | --- | --- | --- | --- | |  |
| --- | --- | --- | --- | --- | --- | --- | --- | --- | --- | --- | --- |

| Outcome | No. of studies (patients) | Study design | Assessment of certainty | | | | | Prevalence in 1000 patients | | | Certainty of the evidence |
| --- | --- | --- | --- | --- | --- | --- | --- | --- | --- | --- | --- |
|  |  |  | Risk of bias | Indirectness | Inconsistency | Imprecision | Publication bias | Prior probability 5% | Prior probability 10% | Prior probability 20% |  |
| True Positive | 11 (711) | Cross-sectional study, Cohort study | Very serious ^a^ | Serious ^b^ | Very serious ^c^ | Serious ^d^ | None | 39 (31 to 48) | 78 (61 to 95) | 156 (122 to 190) | ⨁◯◯◯ Very low |
| False Negative |  |  |  |  |  |  |  | 11 (2 to 19) | 22 (5 to 39) | 44 (10 to 78) |  |
| True Negative | 11 (711) | Cross-sectional study, Cohort study | Very serious ^a^ | Serious ^b^ | Very serious ^c^ | Not serious ^e^ | None | 884 (827 to 931) | 837 (783 to 882) | 744 (696 to 784) | ⨁◯◯◯ Very low |
| False Positive |  |  |  |  |  |  |  | 66 (19 to 123) | 63 (18 to 117) | 56 (16 to 104) |  |

a. Regarding the risk of bias in the QUADAS-2 tool, the proportion of studies judged to have a high or unknown risk of bias was 9/11 for patient selection, 10/11 for index test, 9/11 for reference standard, and 9/11 for flow and timing. Thus, the proportion of studies with high or unknown risk in two or more categories was approximately more than 2/3, which was judged to be “Very serious.”

b. Regarding the concerns about the applicability in the QUADAS-2 tool, the proportion of studies judged to have a high or unknown concern about the applicability was 0/11 for patient selection, 0/11 for index test, and 0/11 for reference standard. Thus, the applicability was considered to be maintained because there was less than one category in which the proportion of studies with high or unknown concerns about applicability was approximately more than half. However, in this systematic review, we conducted the literature search not only for patients with ARDS but also for those with invasive pulmonary aspergillosis. Owing to this gap from the ideal systematic review question that should be set, the results to the clinical situation assumed by guideline readers have limited applicability. Therefore, we judged the overall applicability to be "Serious.”

c. We visually assessed the variability in the results of each study included in the systematic review using forest plots.

d. The results may be imprecise because the total number of patients included in the systematic review was below the optimal informative threshold. Additionally, we examined the net benefit of the test (difference between true positives and weighted false positives), if one false positive was considered acceptable for every 0.3 true positive. When the prevalence was set at 5–10%, the net benefit of the test did not differ between the upper and lower limits of the confidence interval of the integrated sensitivity, and clinical judgment was not expected to change. Therefore, the overall imprecision was judged to be “Serious.”

e. The total number of patients included in the systematic review was above the threshold for obtainingoptimal information. Additionally, the net benefit of the test (difference between true negatives and weighted false negatives) was examined if one false positive is considered acceptable for every 0.3 true positive. When the prevalence was set at 10–20%, the net benefit of the test did not differ between the upper and lower limits of the confidence interval of the integrated specificity, and clinical judgment was not expected to change. Therefore, the overall imprecision was judged to be “Not serious.”

**Adverse events of bronchoalveolar lavage**

| Outcome | No. of studies (patients) | Study design | Summary of the result | | Assessment of certainty | | | | | Certainty of the evidence |
| --- | --- | --- | --- | --- | --- | --- | --- | --- | --- | --- |
|  |  |  | Frequency  (95%CI) | Frequency per 1000 patients (95%CI) | Risk of bias | Indirectness | Inconsistency | Imprecision | Publication bias |  |
| Death | 15 (1106) | Cohort study, Randomized controlled trial | 0.000%  (0.000-0.035) | 0 (0-0) | Serious ^c^ | Not serious ^d^ | Not serious ^e^ | Not serious ^g^ | Not assessed | ⨁⨁⨁◯ moderate |
| Severe complications of the respiratory system requiring new treatment ^a^ | 15 (1106) | Cohort study, Randomized controlled trial | 1.548% (0.000-5.053) | 15 (0-50) | Serious ^c^ | Not serious ^d^ | Not serious ^e,f^ | Not serious ^g^ | Not assessed | ⨁⨁⨁◯ moderate |
| Severe complications of the cardiovascular system requiring new treatment ^b^ | 10 (706) | Cohort study, Randomized controlled trial | 0.011% (0.000-0.788) | 0 (0-8) | Serious ^c^ | Not serious ^d^ | Not serious ^e^ | Serious ^h^ | Not assessed | ⨁⨁◯◯ low |
| Major bleeding | 10 (747) | Cohort study, Randomized controlled trial | 0.000% (0.000-0.235) | 0 (0-2) | Serious ^c^ | Not serious ^d^ | Not serious ^e^ | Serious ^h^ | Not assessed | ⨁⨁◯◯ low |

a. Initiation of mechanical ventilation or new pneumothorax

b. Initiation of vasopressor or anti-arrhythmic drugs

c. Regarding the risk of bias in the tool developed by Lorio et al. (Lorio A, et al. BMJ 2015;350:h870), the proportion of studies judged to have a high or unknown risk of bias was 6/15 for patient selection, 3/15 for duration of follow up, 8/15 for definition of the outcome, 8/15 for reporting the important prognostic factors, and 12/15 for adjustment in important prognostic factors. Thus, a certain proportion of studies was determined to have a high or unknown risk, which was judged to be “Serious”.

d. In this systematic review, we conducted a literature search using the ideal systematic review question that should be set, and thus the results were generally considered applicable to clinical situations assumed by guideline readers. Therefore, we judged the applicability to be “Not serious.”

e. The variability in the results of each study included in the systematic review was visually assessed using forest plots and based on the I^2^ statistic.

f. Despite I2 = 87%, this component was judged to be “Not serious,” because heterogeneity could be explained by subgroup analyses with separate studies that included patients, not on ventilators.

g. The total number of patients included in the systematic review was above the threshold for obtaining optimal information. Clinical judgment was not expected to change between the upper and lower ends of the confidence interval of integrated frequency. Consequently, the overall imprecision was judged to be “Not serious.”

h. The results may be imprecise because the total number of patients included in the systematic review was below the optimal informative threshold. Clinical judgment was not expected to change between the upper and lower limits of the confidence interval of integrated frequency. As a result, the overall imprecision was judged to be “Serious.”

1. Evidence-to-Decision table

| Question | |
| --- | --- |
| **CQ10： Should serum β-D-glucan and galactomannan antigens of the blood or BAL fluid be used for identifying invasive pulmonary aspergillosis as the causative disease of ARDS?** | |
| **Population:** | Patients with ARDS or acute respiratory failure |
| **Target condition:** | Invasive pulmonary aspergillosis |
| **Index test:** | Serum β-D-glucan, galactomannan antigen (blood, bronchoalveolar lavage fluid [BALF]) |
| **Purpose/role of the test:** | Differential diagnosis in ARDS management |
| **setting:** | Situation equivalent to the emergency room (ER) or intensive care unit (ICU) |
| **Main outcomes:** | Overall survival, serious adverse events from testing |
| **Medical practice based on test results:** | If positive (suspected invasive pulmonary aspergillosis), initiate treatment with appropriate agents. If negative, avoid unnecessary treatment and perform additional testing on a differential diagnosis or follow-up. |
| **perspective:** | Individual |
| **background:** | In the management of ARDS, diagnosis of the causative disease and appropriate treatment are important. Invasive pulmonary aspergillosis develops in immunocompromised patients and presents with rapidly progressive respiratory failure. Although it is a rare causative disease of ARDS, it is highly likely to have a fatal outcome if not properly treated. Therefore, it is important to diagnose invasive pulmonary aspergillosis quickly and accurately. The measurement of serum β-D-glucan and blood galactomannan antigen are widely used tests in patients suspected of aspergillosis. In recent years, the measurement of galactomannan antigen in BALF has also received attention. Therefore, we posed the question, “Should serum β-D-glucan and galactomannan antigen (blood, bronchoalveolar lavage fluid) be used to differentiate the cause of ARDS (invasive pulmonary aspergillosis)?” |
| **conflict of interest:** | None |

# Assessment

| Problem Is the problem a priority? | | |
| --- | --- | --- |
| Judgment | Research evidence | ADDITIONAL considerations |
| ● Yes  ○ Probably yes  ○ Probably no  ○ No  ○ Varies  ○ Do not know | In the management of ARDS, diagnosis of the causative disease and appropriate treatment are important. Invasive pulmonary aspergillosis develops in immunocompromised patients and presents with rapidly progressive respiratory failure. Although it is a rare causative disease of ARDS, it is highly likely to have a fatal outcome if not properly treated. Therefore, it is important to diagnose invasive pulmonary aspergillosis quickly and accurately. The measurement of serum β-D-glucan and blood galactomannan antigen are widely used tests in patients suspected of aspergillosis. In recent years, the measurement of galactomannan antigen in BALF has also received attention. Therefore, this clinical question was considered to be of high priority. |  |
| Test accuracy How accurate is the test? | | |
| Judgment | Research evidence | ADDITIONAL considerations |
| Serum β-D-glucan  ○ Very accurate  ○ Accurate  ● Inaccurate  ○ Very inaccurate  ○ Varies  ○ Do not know  Galactomannan antigen (blood, BALF)  ○ Very accurate  ○ Accurate  ○ Inaccurate  ○ Very inaccurate  ● Varies  ○ Do not know | The results of the systematic review and meta-analysis showed the following.  The accuracy of the serumβ-D-glucan was judged to be “Inaccurate.” The accuracy of the galactomannan antigen was judged to be “Varies” due to multiple samples and cutoffs.  **Serum β-D-glucan**  **<Cutoff 80 pg/mL>**  (9 studies, 757 patients)  Integrated sensitivity: 0.70 (95% CI: 0.49-0.85)  Integrated specificity: 0.73 (95% CI: 0.58-0.84)  (Bivariate model)   | Serum β-D-glucan | Prevalence in 1000 patients | | | Certainty of the evidence | | --- | --- | --- | --- | --- | | Prior probability | 5% | 10% | 20% |  | | True positive | 35 (25-43) | 70 (49-85) | 140 (98-170) | Very low | | False negative | 15 (7-25) | 30 (15-51) | 60 (30-102) | | True negative | 694 (551-798) | 657 (522-756) | 584 (464-672) | Very low | | False positive | 256 (152-399) | 243 (144-378) | 216 (128-336) |   **Galactomannan antigen (blood)**  **<Cutoff 0.5 optical density index (ODI)>**  (28 studies, 518 patients)  Integrated sensitivity: 0.75 (95% CI: 0.65-0.83)  Integrated specificity: 0.85 (95% CI: 0.77-0.90)  (Bivariate model)   | Galactomannan antigen (blood) | Prevalence in 1000 patients | | | Certainty of the evidence | | --- | --- | --- | --- | --- | | Prior probability | 5% | 10% | 20% |  | | True positive | 38 (33-42) | 75 (65-83) | 150 (130-166) | Very low | | False negative | 12 (8-17) | 25 (17-35) | 50 (34-70) | | True negative | 808 (731-855) | 765 (693-810) | 680 (616-720) | Very low | | False positive | 142 (95-219) | 135 (90-207) | 120 (80-184) |   **<Cutoff 1.0 ODI>**  (8 studies, 145 patients)  Integrated sensitivity: 0.76 (95% CI: 0.60-0.91)  Integrated specificity: 0.88 (95% CI: 0.79-0.94)  (Bivariate model)   | Galactomannan antigen (blood) | Prevalence in 1000 patients | | | Certainty of the evidence | | --- | --- | --- | --- | --- | | Prior probability | 5% | 10% | 20% |  | | True positive | 38 (28-44) | 76 (57-88) | 152 (114-176) | Very low | | False negative | 12 (6-22) | 24 (12-43) | 48 (24-86) | | True negative | 836 (751-884) | 792 (711-837) | 704 (632-744) | Very low | | False positive | 114 (66-199) | 108 (63-189) | 96 (56-168) |   **<Cutoff 1.5 ODI>**  (14 studies, 272 patients)  Integrated sensitivity: 0.59 (95% CI: 0.44-0.72)  Integrated specificity: 0.95 (95% CI: 0.90-0.97)  (Bivariate model)   | Galactomannan antigen (blood) | Prevalence in 1000 patients | | | Certainty of the evidence | | --- | --- | --- | --- | --- | | Prior probability | 5% | 10% | 20% |  | | True positive | 30 (22-36) | 59 (44-72) | 118 (88-144) | Very low | | False negative | 20 (14-28) | 41 (28-56) | 82 (56-112) | | True negative | 903 (855-922) | 855 (810-873) | 760 (720-776) | Very low | | False positive | 47 (28-95) | 45 (27-90) | 40 (24-80) |   **Galactomannan antigen (BALF)**  **<Cutoff 0.5 ODI>**  (12 studies, 1123 patients)  Integrated sensitivity: 0.88 (95% CI: 0.75-1.00)  Integrated specificity: 0.81 (95% CI: 0.71-0.91)  (Bivariate model)   | Galactomannan antigen (BALF) | Prevalence in 1000 patients | | | Certainty of the evidence | | --- | --- | --- | --- | --- | | Prior probability | 5% | 10% | 20% |  | | True positive | 44 (38-50) | 88 (75-100) | 176 (150-200) | Very low | | False negative | 6 (0-12) | 12 (0-25) | 24 (0-50) | | True negative | 770 (675-864) | 729 (639-819) | 648 (568-728) | Very low | | False positive | 180 (86-275) | 171 (81-261) | 152 (72-232) |   **Cutoff 1.0 ODI>**  (11 studies, 711 patients)  Integrated sensitivity: 0.78 (95% CI: 0.61-0.95)  Integrated specificity: 0.93 (95% CI: 0.87-0.98)  (Bivariate model)   | Galactomannan antigen (BALF) | Prevalence in 1000 patients | | | Certainty of the evidence | | --- | --- | --- | --- | --- | | Prior probability | 5% | 10% | 20% |  | | True positive | 39 (31-48) | 78 (61-95) | 156 (122-190) | Very low | | False negative | 11 (2-19) | 22 (5-39) | 44 (10-78) | | True negative | 884 (827-931) | 837 (783-882) | 744 (696-784) | Very low | | False positive | 66 (19-123) | 63 (18-117) | 56 (16-104) | |  |
| Desirable Effects How substantial are the desirable anticipated effects? | | |
| Judgment | Research evidence | ADDITIONAL considerations |
| Serum β-D-glucan  ○ Large  ● Moderate  ○ Small  ○ Trivial  ○ Varies  ○ Do not know  Galactomannan antigen (blood, BALF)  ○ Large  ○ Moderate  ○ Small  ○ Trivial  ● Varies  ○ Do not know | The number of patients who would benefit from appropriate treatment in a sample of 1000 patients (the number of true positives) was calculated.  The desirable effects of serumβ-D-glucan were judged to be “Moderate.” The desirable effects of galactomannan antigen were judged to be “Varies” due to multiple samples and cutoffs.  **Serum β-D-glucan**  **<Cutoff 80 pg/mL>**   | Prior probability | 5% | 10% | 20% | | --- | --- | --- | --- | | Treatment based on test results | 35 | 70 | 140 | | Treatment of all patients regardless of test results | 50 | 100 | 200 | | No treatment for all patients regardless of test results | 0 | 0 | 0 |   **Galactomannan antigen (blood)**  **<Cutoff 0.5 ODI>**   | Prior probability | 5% | 10% | 20% | | --- | --- | --- | --- | | Treatment based on test results | 38 | 75 | 150 | | Treatment of all patients regardless of test results | 50 | 100 | 200 | | No treatment for all patients regardless of test results | 0 | 0 | 0 |   **<Cutoff 1.0 ODI>**   | Prior probability | 5% | 10% | 20% | | --- | --- | --- | --- | | Treatment based on test results | 38 | 76 | 152 | | Treatment of all patients regardless of test results | 50 | 100 | 200 | | No treatment for all patients regardless of test results | 0 | 0 | 0 |   **<Cutoff 1.5 ODI>**   | Prior probability | 5% | 10% | 20% | | --- | --- | --- | --- | | Treatment based on test results | 30 | 59 | 118 | | Treatment of all patients regardless of test results | 50 | 100 | 200 | | No treatment for all patients regardless of test results | 0 | 0 | 0 |   **Galactomannan antigen (BALF)**  **<Cutoff 0.5 ODI>**   | Prior probability | 5% | 10% | 20% | | --- | --- | --- | --- | | Treatment based on test results | 44 | 88 | 176 | | Treatment of all patients regardless of test results | 50 | 100 | 200 | | No treatment for all patients regardless of test results | 0 | 0 | 0 |   **<Cutoff 1.0 ODI>**   | Prior probability | 5% | 10% | 20% | | --- | --- | --- | --- | | Treatment based on test results | 39 | 78 | 156 | | Treatment of all patients regardless of test results | 50 | 100 | 200 | | No treatment for all patients regardless of test results | 0 | 0 | 0 | | If all patients were to be treated regardless of the test results, the number of patients who would benefit from treatment was calculated as 1000 x (prior probability).  If all patients were not to be treated regardless of the test results, the number of patients who would benefit from the treatment was considered to be zero. |
| Undesirable EffectsHow substantial are the undesirable anticipated effects? | | |
| Judgment | Research evidence | ADDITIONAL considerations |
| Serum β-D-glucan  ○ Large  ● Moderate  ○ Small  ○ Trivial  ○ Varies  ○ Do not know  Galactomannan antigen (blood, BALF)  ○ Large  ○ Moderate  ○ Small  ○ Trivial  ● Varies  ○ Do not know | The relative clinical weighting of false positives to true positives was set at 0.3. The number of patients who would be harmed by unnecessary treatment in a sample of 1000 patients was calculated.  The undesirable effects of the serumβ-D-glucan were judged to be “Moderate.” The undesirable effects of the galactomannan antigen were judged to be “Varies” due to multiple samples and cutoffs.  **Serum β-D-glucan**  **<Cutoff 80 pg/mL>**   | Prior probability | 5% | 10% | 20% | | --- | --- | --- | --- | | Treatment based on test results | 77 | 73 | 65 | | Treatment of all patients regardless of test results | 285 | 270 | 240 | | No treatment for all patients regardless of test results | 0 | 0 | 0 |   **Galactomannan antigen (blood)**  **<Cutoff 0.5 ODI>**   | Prior probability | 5% | 10% | 20% | | --- | --- | --- | --- | | Treatment based on test results | 43 | 41 | 36 | | Treatment of all patients regardless of test results | 285 | 270 | 240 | | No treatment for all patients regardless of test results | 0 | 0 | 0 |   **<Cutoff 1.0 ODI>**   | Prior probability | 5% | 10% | 20% | | --- | --- | --- | --- | | Treatment based on test results | 43 | 41 | 36 | | Treatment of all patients regardless of test results | 285 | 270 | 240 | | No treatment for all patients regardless of test results | 0 | 0 | 0 |   **<Cutoff 1.5 ODI>**   | Prior probability | 5% | 10% | 20% | | --- | --- | --- | --- | | Treatment based on test results | 14 | 14 | 12 | | Treatment of all patients regardless of test results | 285 | 270 | 240 | | No treatment for all patients regardless of test results | 0 | 0 | 0 |   **Galactomannan antigen (BALF)**  **<Cutoff 0.5 ODI>**   | Prior probability | 5% | 10% | 20% | | --- | --- | --- | --- | | Treatment based on test results | 54 | 51 | 46 | | Treatment of all patients regardless of test results | 285 | 270 | 240 | | No treatment for all patients regardless of test results | 0 | 0 | 0 |   **<Cutoff 1.0 ODI>**   | Prior probability | 5% | 10% | 20% | | --- | --- | --- | --- | | Treatment based on test results | 20 | 19 | 17 | | Treatment of all patients regardless of test results | 285 | 270 | 240 | | No treatment for all patients regardless of test results | 0 | 0 | 0 |   Frequency of adverse events of bronchoalveolar lavage   |  | Summary of the result | | Certainty of the evidence | | --- | --- | --- | --- | | Outcome | Frequency  (95% CI) | Frequency per 1000 patients (95% CI) | | Death | 0.000%  (0.000-0.035) | 0 (0-0) | Moderate | | Severe complications of respiratory system requiring new treatment ^a^ | 1.548% (0.000-5.053) | 15 (0-50) | Moderate | | Severe complications of cardiovascular system requiring new treatment ^b^ | 0.011% (0.000-0.788) | 0 (0-8) | Low | | Major bleeding | 0.000% (0.000-0.235) | 0 (0-2) | Low |   **Bronchoalveolar lavage**   | Adverse events |  | | --- | --- | | Death | 0 | | Severe complications of the respiratory system requiring new treatment ^a^ | 6 | | Severe complications of the cardiovascular system requiring new treatment ^b^ | 0 | | Major bleeding | 0 |   The relative clinical weighting of death to true positives was set at 1.0, and that of other serious adverse events were set at 0.4. | If patients were treated based on test results, the number of patients who would be harmed by unnecessary treatment was calculated as (number of false positives) x (clinical weighting).  If all patients were treated regardless of test results, the number of false positives was calculated as (1-prior probability) x 1000.  If all patients were not treated, the number of false positives was considered to be zero.  The harm of adverse events was calculated by multiplying the frequency of adverse events expected when the test was performed on 1000 people by the clinical weighting. |
| Certainty of evidence What is the overall certainty of the evidence of test accuracy? | | |
| Judgment | Research evidence | ADDITIONAL considerations |
| ● Very low  ○ Low  ○ Moderate  ○ High  ○ No included study | The certainty of the evidence was judged to be “Very low” by adopting the certainty of the evidence with the lowest certainty. |  |
| Certainty of the evidence of test’s effects What is the overall certainty of the evidence for any critical or important direct benefits, adverse effects, or burden of the test? | | |
| Judgment | Research evidence | ADDITIONAL considerations |
| ○ Very low  ● Low  ○ Moderate  ○ High  ○ No included study  ○ Do not know | No evidence examining the direct effects of the tests. The certainty of the evidence was judged to be “Low” for adverse events of bronchoalveolar lavage. |  |
| Certainty of evidence of management’s effects What is the overall certainty of the evidence of effects of the management that is guided by the test results? | | |
| Judgment | Research evidence | ADDITIONAL considerations |
| ○ Very low  ○ Low  ○ Moderate  ○ High  ● No included study  ○ Do not know | In general, treatment based on the result of true positives with antifungal agents is considered “good medical practice” and is expected to improve overall survival and other outcomes. On the contrary, if patients receive unnecessary treatment due to false positives, undesirable effects (such as adverse drug events) can occur. However, no studies have included evidence on the impact of treatment on final outcomes such as overall survival. |  |
| Certainty of evidence of test result/management How certain is the link between test results and management decisions? | | |
| Judgment | Research evidence | ADDITIONAL considerations |
| ○ Very low  ○ Low  ○ Moderate  ○ High  ● No included study  ○ Do not know | Since treatment of invasive pulmonary aspergillosis is usually initiated promptly based on the test results, it seems reasonable to assume that there is a high degree of certainty regarding the relationship between the test results and management decisions. However, no studies have been included as evidence. |  |
| Certainty of effects What is the overall certainty of the evidence of effects of the test? | | |
| Judgment | Research evidence | ADDITIONAL considerations |
| ● Very low  ○ Low  ○ Moderate  ○ High  ○ No included study  ○ Do not know | Since the certainty of the evidence of the test’s accuracy is “Very low,” the certainty of the evidence of effects of the test becomes “Very low.” |  |
| Values Is there important uncertainty about or variability in how much people value the main outcomes? | | |
| Judgment | Research evidence | ADDITIONAL considerations |
| ○ Important uncertainty or variability  ● Possibly important uncertainty or variability  ○ Probably no important uncertainty or variability  ○ No important uncertainty or variability | The value of desirable effects such as true positives and undesirable effects such as false positives and adverse events may vary according to the values and experiences of individual healthcare providers and patients.  (The relative clinical weighting of false positives to true positives may vary depending on the values and experiences of individual health care providers and patients.) |  |
| Balance of effects Does the balance between desirable and undesirable effects favor the intervention or the comparison? | | |
| Judgment | Research evidence | ADDITIONAL considerations |
| Serum β-D-glucan  ○ Favors the test  ○ Probably favors the test  ○ Does not favor either the test or the comparison  ● Probably favors the comparison  ○ Favors the comparison  ○ Varies  ○ Do not know  Galactomannan antigen (blood, BALF)  ○ Favors the test  ● Probably favors the test  ○ Does not favor either the test or the comparison  ○ Probably favors the comparison  ○ Favors the comparison  ○ Varies  ○ Do not know | The net benefit of the test was calculated, considering the benefit of appropriate treatment due to true positive diagnosis, the harm of unnecessary treatment due to false positive diagnosis, and the adverse events of the test.  **Serum β-D-glucan**  **<Cutoff 80 pg/mL>**  Net benefit  (The number of patients who benefit from the test when performed on 1000 patients.)   | Prior probability | 5% | 10% | 20% | | --- | --- | --- | --- | | Treatment based on test results | -42 | -3 | 75 | | Treatment of all patients regardless of test results | -235 | -170 | -40 | | No treatment for all patients regardless of test results | 0 | 0 | 0 |   The relative clinical weight of false positives to true positives was set at 0.3, and the net benefit was calculated with a prior probability of 5-20%. Serious adverse events of the test were considered to be negligible. We compared the net benefit of treating patients based on the test results, treating all patients without testing, and not treating any patients without testing.  There are likely to be limited clinical situations where there would be a net benefit from testing.  **Galactomannan antigen (blood)**  **<Cutoff 0.5 ODI>**  Net benefit  (The number of patients who benefit from the test when performed on 1000 patients.)   | Prior probability | 5% | 10% | 20% | | --- | --- | --- | --- | | Treatment based on test results | -5 | 35 | 114 | | Treatment of all patients regardless of test results | -235 | -170 | -40 | | No treatment for all patients regardless of test results | 0 | 0 | 0 |   The relative clinical weight of false positives to true positives was set at 0.3, and the net benefit was calculated with a prior probability of 5-20%. Serious adverse events of the test were considered to be negligible. We compared the net benefit of treating patients based on the test results, treating all patients without testing, and not treating any patients without testing.  There are likely to be many clinical situations where there would be a net benefit from testing.  **<Cutoff 1.0 ODI>**  Net benefit  (The number of patients who benefit from the test when performed on 1000 patients.)   | Prior probability | 5% | 10% | 20% | | --- | --- | --- | --- | | Treatment based on test results | 4 | 44 | 123 | | Treatment of all patients regardless of test results | -235 | -170 | -40 | | No treatment for all patients regardless of test results | 0 | 0 | 0 |   The relative clinical weight of false positives to true positives was set at 0.3, and the net benefit was calculated with a prior probability of 5-20%. Serious adverse events of the test were considered to be negligible. We compared the net benefit of treating patients based on the test results, treating all patients without testing, and not treating any patients without testing.  There are likely to be many clinical situations where there would be a net benefit from testing.  **<Cutoff 1.5 ODI>**  Net benefit  (The number of patients who benefit from the test when performed on 1000 patients.)   | Prior probability | 5% | 10% | 20% | | --- | --- | --- | --- | | Treatment based on test results | 15 | 46 | 106 | | Treatment of all patients regardless of test results | -235 | -170 | -40 | | No treatment for all patients regardless of test results | 0 | 0 | 0 |   The relative clinical weight of false positives to true positives was set at 0.3, and the net benefit was calculated with a prior probability of 5-20%. Serious adverse events of the test were considered to be negligible. We compared the net benefit of treating patients based on the test results, treating all patients without testing, and not treating any patients without testing.  There are likely to be many clinical situations where there would be a net benefit from testing.  **Galactomannan antigen (BALF)**  **<Cutoff 0.5 ODI>**  Net benefit  (The number of patients who benefit from the test when performed on 1000 patients.)   | Prior probability | 5% | 10% | 20% | | --- | --- | --- | --- | | Treatment based on test results | -16 | 31 | 124 | | Treatment of all patients regardless of test results | -235 | -170 | -40 | | No treatment for all patients regardless of test results | 0 | 0 | 0 |   The relative clinical weight of false positives to true positives was set at 0.3, and the net benefit was calculated with a prior probability of 5-20%. Serious adverse events of the test were considered to be negligible. We compared the net benefit of treating patients based on the test results, treating all patients without testing, and not treating any patients without testing.  There are likely to be many clinical situations where there would be a net benefit from testing.  **<Cutoff 1.0 ODI>**  Net benefit  (The number of patients who benefit from the test when performed on 1000 patients.)   | Prior probability | 5% | 10% | 20% | | --- | --- | --- | --- | | Treatment based on test results | 13 | 53 | 133 | | Treatment of all patients regardless of test results | -235 | -170 | -40 | | No treatment for all patients regardless of test results | 0 | 0 | 0 |   The relative clinical weight of false positives to true positives was set at 0.3, and the net benefit was calculated with a prior probability of 5-20%. Serious adverse events of the test were considered to be negligible. We compared the net benefit of treating patients based on the test results, treating all patients without testing, and not treating any patients without testing.  There are likely to be many clinical situations where there would be a net benefit from testing. | The net benefit (net benefit) was calculated as (number of true positives receiving appropriate treatment) -(number of false positives receiving unnecessary treatment) × (clinical weighting)-(number of serious adverse events of the test) for a sample of 1000 patients.  If adverse events were considered negligible, they were calculated as zero. |
| Acceptability Is the intervention acceptable to key stakeholders? | | |
| Judgment | Research evidence | ADDITIONAL considerations |
| ● Yes  ○ Probably yes  ○ Probably no  ○ No  ○ Varies  ○ Do not know | It is a commonly practiced medical procedure and considered acceptable. |  |
| Feasibility Is the intervention feasible to implement? | | |
| Judgment | Research evidence | ADDITIONAL considerations |
| ○ Yes  ● Probably yes  ○ Probably no  ○ No  ○ Varies  ○ Do not know | They are commonly practiced medical procedures and probably feasible, but some facilities may not be able to perform bronchoalveolar lavage. |  |

# Summary of Judgment (Serum β-D-glucan)

|  | **Judgment** | | | | | | |
| --- | --- | --- | --- | --- | --- | --- | --- |
| **PROBLEM** | No | Probably no | Probably yes | Yes |  | Varies | Do not know |
| **DESIRABLE EFFECTS** | Trivial | Small | Moderate | Large |  | Varies | Do not know |
| **UNDESIRABLE EFFECTS** | Large | Moderate | Small | Trivial |  | Varies | Do not know |
| **CERTAINTY OF EVIDENCE OF TEST ACCURACY** | Very low | Low | Moderate | High |  |  | No included study |
| **CERTAINTY OF THE EVIDENCE OF TEST’S EFFECTS** | Very low | Low | Moderate | High |  |  | No included study |
| **CERTAINTY OF THE EVIDENCE OF MANAGEMENT’S EFFECTS** | Very low | Low | Moderate | High |  |  | No included study |
| **CERTAINTY OF THE EVIDENCE OF TEST RESULT/MANAGEMENT** | Very low | Low | Moderate | High |  |  | No included study |
| **CERTAINTY OF EFFECT** | Very low | Low | Moderate | High |  |  | No included study |
| **VALUES** | Important uncertainty or variability | Possibly important uncertainty or variability | Probably no important uncertainty or variability | No important uncertainty or variability |  |  |  |
| **BALANCE OF EFFECTS** | Favors the comparison | Probably favors the comparison | Does not favor either the test or the comparison | Probably favors the test | Favors the test | Varies | Do not know |
| **ACCEPTABILITY** | No | Probably no | Probably yes | Yes |  | Varies | Do not know |
| **FEASIBILITY** | No | Probably no | Probably yes | Yes |  | Varies | Do not know |

# Summary of Judgment (Galactomannan antigen [blood, BALF])

|  | **Judgment** | | | | | | |
| --- | --- | --- | --- | --- | --- | --- | --- |
| **PROBLEM** | No | Probably no | Probably yes | Yes |  | Varies | Do not know |
| **DESIRABLE EFFECTS** | Trivial | Small | Moderate | Large |  | Varies | Do not know |
| **UNDESIRABLE EFFECTS** | Large | Moderate | Small | Trivial |  | Varies | Do not know |
| **CERTAINTY OF EVIDENCE OF TEST ACCURACY** | Very low | Low | Moderate | High |  |  | No included study |
| **CERTAINTY OF THE EVIDENCE OF TEST’S EFFECTS** | Very low | Low | Moderate | High |  |  | No included study |
| **CERTAINTY OF THE EVIDENCE OF MANAGEMENT’S EFFECTS** | Very low | Low | Moderate | High |  |  | No included study |
| **CERTAINTY OF THE EVIDENCE OF TEST RESULT/MANAGEMENT** | Very low | Low | Moderate | High |  |  | No included study |
| **CERTAINTY OF EFFECT** | Very low | Low | Moderate | High |  |  | No included study |
| **VALUES** | Important uncertainty or variability | Possibly important uncertainty or variability | Probably no important uncertainty or variability | No important uncertainty or variability |  |  |  |
| **BALANCE OF EFFECTS** | Favors the comparison | Probably favors the comparison | Does not favor either the test or the comparison | Probably favors the test | Favors the test | Varies | Do not know |
| **ACCEPTABILITY** | No | Probably no | Probably yes | Yes |  | Varies | Do not know |
| **FEASIBILITY** | No | Probably no | Probably yes | Yes |  | Varies | Do not know |

# Type of Recommendation (Serum β-D-glucan)

| Strong recommendation against the test | Conditional recommendation against the test | Conditional recommendation for either the test or the comparison | Conditional recommendation for the test | Strong recommendation for the test |
| --- | --- | --- | --- | --- |
| ○ | ● | ○ | ○ | ○ |

# Type of Recommendation (Galactomannan antigen (blood, BALF))

| Strong recommendation against the test | Conditional recommendation against the test | Conditional recommendation for either the test or the comparison | Conditional recommendation for the test | Strong recommendation for the test |
| --- | --- | --- | --- | --- |
| ○ | ○ | ○ | ● | ○ |

# Conclusions

| Recommendation |
| --- |
| **We conditionally recommend that the causative disease of ARDS (invasive pulmonary aspergillosis) should not be differentiated based solely on the results of serum β-D-glucan**  **(Conditional recommendation/very low certainty of the evidence: GRADE: 2D).**  **We conditionally recommend the use of galactomannan antigen (blood, BALF) to differentiate the cause of ARDS (invasive pulmonary aspergillosis) (Conditional recommendation/very low certainty of the evidence: GRADE: 2D).**  **Note: If the clinical situation (characteristics of the target patient, characteristics and timing of the test, prior probability, values of the patient and health care providers) changes, the balance of effects may change, and different options may be recommended.** |
|  |
| Justification |
| **Question**  Should serum β-D-glucan and galactomannan antigens of the blood or BAL fluid be used for identifying invasive pulmonary aspergillosis as the causative disease of ARDS?  **Patients**  Patients with ARDS  **Index test**  Serum β-D-glucan, galactomannan antigen (blood, BALF)  **Purpose, role, and setting of the test**  Differential diagnosis in ARDS management in ER, ICU, or equivalent  **Medical practice based on test results**  If positive, the patient will be diagnosed with invasive pulmonary aspergillosis and treated with appropriate agents. If negative, unnecessary treatment will be avoided, and additional testing for different diagnostic targets or follow-up will be performed.  **Summary of evidence**：  Serum β-D-glucan  <Cutoff 80 pg/mL> (9 studies, 757 patients)  Integrated sensitivity: 0.70 (95% CI: 0.49-0.85), Integrated specificity: 0.73 (95% CI: 0.58-0.84)  Galactomannan antibody (blood)  <Cutoff 0.5 ODI> (28 studies, 518 patients)  Integrated sensitivity: 0.75 (95% CI: 0.65-0.83), Integrated specificity: 0.85 (95% CI: 0.77-0.90)  <Cutoff 1.0 ODI> (8 studies, 145 patients)  Integrated sensitivity: 0.76 (95% CI: 0.60-0.91), Integrated specificity: 0.88 (95% CI: 0.79-0.94)  <Cutoff 1.5 ODI> (14 studies, 272 patients)  Integrated sensitivity: 0.59 (95% CI: 0.44-0.72), Integrated specificity: 0.95 (95% CI: 0.90-0.97)  Galactomannan antibody (BALF)  <Cutoff 0.5 ODI> (12 studies, 1123 patients)  Integrated sensitivity: 0.88 (95% CI: 0.75-1.00), Integrated specificity: 0.81 (95% CI: 0.71-0.91)  <Cutoff 1.0 ODI> (11 studies, 711 patients)  Integrated sensitivity: 0.78 (95% CI: 0.61-0.95), Integrated specificity: 0.93 (95% CI: 0.87-0.98)  Frequency of adverse events of bronchoalveolar lavage  Death: 0.000% (95% CI: 0.000-0.035)  **Certainty of the evidence**：  Certainty of the evidence of test accuracy was “Very low.” Certainty of the evidence of the frequency of adverse events of bronchoalveolar lavage was “Moderate” or “Low.”  **Values, balance of effects, acceptability, feasibility**：  The desirable effect of the tests is to diagnose invasive pulmonary aspergillosis and receive appropriate treatment promptly. The undesirable effects of the tests are unnecessary treatment due to a false positive result and adverse effects of the tests. We compared these effects of testing with those of deciding the treatment plan without testing. Regarding serum β-D-glucan, the clinical situations that would benefit from testing were considered limited. Regarding galactomannan antigen (blood, BALF), it was considered that many clinical situations would benefit from testing. Feasibility is not a problem, and it is a generally accepted medical practice.  **Panel meeting**  In the preliminary vote, the median score of “recommended text proposal” was 9, and the disagreement index was 0.192 by the modified Delphi method.  At the panel meeting, In the panel meeting, a correction was pointed out in the way EtD is described. Finally, an agreement was achieved with the results of a preliminary vote.  **Additional considerations**：  The balance of effects depends on the prior probability, the clinical weighting of false positives, and the performance of the test. Therefore, if the clinical situation (characteristics of the patient, characteristics and timing of the test, prior probability, the clinical weighting of false positives, and other values held by patients and caregivers) changes, the balance of effects may change, and different options may be recommended. |

| Subgroup considerations |
| --- |
| None |
| Implementation considerations |
| Most of the studies included in this systematic review were targeted at immunocompromised patients. Therefore, the results of integrated sensitivity and specificity may not be applicable to patients with ARDS in general. From the perspective of human resources and adverse events, blood is likely measured more often than BALF in actual clinical practice. In addition, bronchoalveolar lavage fluid may not be available at some facilities. |

| Monitoring and evaluation |
| --- |
| After the publication of this medical guideline, it is necessary to collect and monitor information on the status of clinical use and problems in conducting the test using tools such as questionnaires. |
| Research priorities |
| Many of the studies included in the systematic review did not include patients with ARDS. There is a need for diagnostic accuracy studies in patients with ARDS. |

**CQ11 Should plain chest X-rays, chest high-resolution CT, and interferon γ release assays be used for identifying miliary tuberculosis as the causative disease of ARDS?**

1.Search strategy

MEDLINE via PubMed （Search date: 2020/7/9）

| #1 | "Tuberculosis, Miliary"/diagnosis[Mesh] |
| --- | --- |
| #2 | tuberculosi*[tiab] AND miliary[tiab] |
| #3 | disseminated tuberculosis[tiab] |
| #4 | #1 OR #2 OR #3 |
| #5 | “tomography, x-ray computed”[MeSH Terms] |
| #6 | “computed”[tiab] AND “tomography”[tiab] |
| #7 | “computed tomography”[tiab] |
| #8 | #5 OR #6 OR #7 |
| #9 | “mass chest x-ray”[MeSH Terms] |
| #10 | (“mass”[tiab] AND “chest”[tiab]) AND “x-ray”[tiab] |
| #11 | #9 OR #10 |
| #12 | "chest*"[tiab] OR "thorax"[tiab] |
| #13 | "diagnostic imaging"[tiab] |
| #14 | "radiography"[MeSH Terms] |
| #15 | "radiograph*"[tiab] |
| #16 | #13 OR #14 OR #15 |
| #17 | #12 AND #16 |
| #18 | "Thorax/diagnostic imaging"[Mesh] |
| #19 | #17 OR #18 |
| #20 | "Interferon-gamma Release Tests"[Mesh] |
| #21 | "interferon-gamma"[tiab] AND "release"[tiab] AND "tests"[tiab] |
| #22 | "Interferon-gamma/analysis"[Mesh] |
| #23 | "igra"[tiab] |
| #24 | "t-spot"[tiab] |
| #25 | quantiferon[tiab] |
| #26 | qft[tiab] |
| #27 | #20 OR #21 OR #22 OR #23 OR #24 OR #25 OR #26 |
| #28 | #8 OR #11 OR #19 OR #27 |
| #29 | #4 AND #28 |
| #30 | [mh animals] NOT ([mh animals] AND [mh humans]) |
| #31 | #29 NOT #30 |

CENTRAL （Search date: 2020/7/9）

| #1 | [mh "Tuberculosis, Miliary"] |
| --- | --- |
| #2 | tuberculosi*:ti,ab AND miliary:ti,ab |
| #3 | "disseminated tuberculosis":ti,ab |
| #4 | #1 OR #2 OR #3 |
| #5 | [mh "tomography, x-ray computed"] |
| #6 | computed:ti,ab AND tomography:ti,ab |
| #7 | “computed tomography”:ti,ab |
| #8 | #5 OR #6 OR #7 |
| #9 | [mh "mass chest x-ray"] |
| #10 | (mass:ti,ab AND chest:ti,ab) AND x-ray:ti,ab |
| #11 | #9 OR #10 |
| #12 | chest*:ti,ab OR thorax:ti,ab |
| #13 | "diagnostic imaging":ti,ab |
| #14 | [mh radiography] |
| #15 | radiograph*:ti,ab |
| #16 | #13 OR #14 OR #15 |
| #17 | #12 AND #16 |
| #18 | [mh Thorax/DG] |
| #19 | #17 OR #18 |
| #20 | [mh "Interferon-gamma Release Tests"] |
| #21 | interferon-gamma:ti,ab AND release:ti,ab AND tests:ti,ab |
| #22 | [mh Interferon-gamma] |
| #23 | igra:ti,ab |
| #24 | t-spot:ti,ab |
| #25 | quantiferon:ti,ab |
| #26 | qft:ti,ab |
| #27 | #20 OR #21 OR #22 OR #23 OR #24 OR #25 OR #26 |
| #28 | #8 OR #11 OR #19 OR #27 |
| #29 | #4 AND #28 |
| #30 | [mh animals] NOT ([mh animals] AND [mh humans]) |
| #31 | #29 NOT #30 |

1. Flow diagram

**Identification**

0 Studies included in qualitative synthesis

833 records after duplicates removed

833 records identified through database searching

833 records identified through database searching

Medline via PubMed (n=826)

CENTRAL (n=7)

0 additional records identified through other sources

0 Studies included in quantitative synthesis (meta-analysis)

833 records excluded

**Included**

**Eligibility**

**Screening**

1. Risk of bias

Not applicable

1. Forest plot

Not applicable

1. Evidence profile

Not applicable

1. Evidence-to-Decision table

| Question | |
| --- | --- |
| **CQ11： Should plain chest X-rays, chest high-resolution CT, and interferon γ release assays be used for identifying miliary tuberculosis as the causative disease of ARDS?** | |
| **Population:** | Patients with ARDS |
| **Target condition:** | Miliary tuberculosis |
| **Index test:** | Chest X-rays, chest high-resolution CT, interferon-γ release assay |
| **Purpose/role of the test:** | Differential diagnosis in ARDS management |
| **setting:** | Situation equivalent to the emergency room (ER) or intensive care unit (ICU) |
| **Main outcomes:** | Overall survival, serious adverse events from testing |
| **Medical practice based on test results:** | If positive (miliary tuberculosis suspected), perform further diagnostic tests and initiate treatment with appropriate antibacterial agents. If negative, avoid unnecessary antiviral therapy and perform additional testing and follow-up on a different diagnosis. |
| **perspective:** | Individual |
| **background:** | Although infrequent, miliary tuberculosis may be the cause of ARDS, which can be fatal if not treated with anti-tuberculosis drugs. If pulmonary tuberculosis is also present, airborne infection control measures are necessary, and thus the diagnosis is of great importance. The definitive diagnosis of tuberculosis is the detection of *Mycobacterium tuberculosis* by smear and culture tests, but preliminary screening tests include chest Xp, high-resolution computed tomography (HRCT), and interferon-γ release assay (IGRA). It is important to screen adult patients with ARDS for miliary tuberculosis with these tests, and if necessary, perform additional tests, treat with appropriate anti-tuberculosis drugs, and initiate appropriate airborne prevention measures. Therefore, we posed the question, “Should chest Xp, chest high-resolution CT, and interferon-γ release assay be used to differentiate the cause of ARDS (miliary tuberculosis)?” |
| **conflict of interest:** | None |

# Assessment

| Problem Is the problem a priority? | | |
| --- | --- | --- |
| Judgment | Research evidence | ADDITIONAL considerations |
| ● Yes  ○ Probably yes  ○ Probably no  ○ No  ○ Varies  ○ Do not know | Although infrequent, miliary tuberculosis may be the cause of ARDS, which can be fatal if not treated with anti-tuberculosis drugs. If pulmonary tuberculosis is also present, airborne infection control measures are necessary, and thus the diagnosis is of great importance. Therefore, this clinical question was considered to be of high priority. |  |
| Test accuracy How accurate is the test? | | |
| Judgment | Research evidence | ADDITIONAL considerations |
| ○ Very accurate  ○ Accurate  ○ Inaccurate  ○ Very inaccurate  ○ Varies  ● Do not know | A literature search did not reveal any high-quality studies reporting the diagnostic accuracy of chest Xp, chest HRCT, and IGRA in diagnosing miliary tuberculosis in patients with ARDS. Therefore, it was judged that there are no studies that provide direct evidence to support the recommendations in this clinical question.  Although not conducted on patients with ARDS, the results of studies examining the diagnostic accuracy of IGRAs in the diagnosis of active tuberculosis are presented for reference. T-Spot® TB has a reported sensitivity of 87.5% (95% CI, 85%-90%) with a specificity of 86.3% (95% CI, 81%-90%), and Quantiferon® TB Gold has a reported sensitivity of 81% (95% CI, 78%-83%) with a specificity of 99.2% (95% CI, 98%-100%) ^1)^. |  |
| Desirable effects How substantial are the desirable anticipated effects? | | |
| Judgment | Research evidence | ADDITIONAL considerations |
| ○ Large  ○ Moderate  ○ Small  ○ Trivial  ○ Varies  ● Do not know | If the test results are true positive and miliary tuberculosis is correctly suspected, appropriate infection control can be performed; specimens can be obtained from sputum, urine, gastric juice, bronchoscopy, bone marrow puncture, and liver biopsy. If *Mycobacterium tuberculosis* is detected, treatment with anti-tuberculosis drugs can be initiated. If the test results are true negative, miliary tuberculosis can be ruled out, unnecessary testing and treatment can be avoided, and additional testing for a different differential diagnosis or follow-up can be performed. However, how large these desirable effects would be is unknown. |  |
| Undesirable effectsHow substantial are the undesirable anticipated effects? | | |
| Judgment | Research evidence | ADDITIONAL considerations |
| ○ Large  ○ Moderate  ○ Small  ○ Trivial  ○ Varies  ● Do not know | If the test leads to a misdiagnosis (false positive), it could cause harm such as unnecessary isolation, invasive testing, and the use of anti-tuberculosis drugs. However, how large these undesirable effects would be is unknown. |  |
| Certainty of evidence What is the overall certainty of the evidence of test accuracy? | | |
| Judgment | Research evidence | ADDITIONAL considerations |
| ○ Very low  ○ Low  ○ Moderate  ○ High  ● No included study | No studies have been included evidence of the diagnostic accuracy. |  |
| Certainty of the evidence of test’s effects What is the overall certainty of the evidence for any critical or important direct benefits, adverse effects, or burden of the test? | | |
| Judgment | Research evidence | ADDITIONAL considerations |
| ○ Very low  ○ Low  ○ Moderate  ○ High  ● No included study  ○ Do not know | No evidence examining the direct effects of the tests. |  |
| Certainty of evidence of management’s effects What is the overall certainty of the evidence of effects of the management that is guided by the test results? | | |
| Judgment | Research evidence | ADDITIONAL considerations |
| ○ Very low  ○ Low  ○ Moderate  ○ High  ● No included study  ○ Do not know | Additional testing for true positives leads to a definitive diagnosis and is expected to have desirable effects such as improved overall survival with anti-tuberculosis drug therapy. On the contrary, if unnecessary tests or treatments are performed due to false positives, undesirable effects (such as adverse events of tests or side effects of drugs) can be assumed. However, no studies have included evidence on the impact of additional testing and treatment on final outcomes such as overall survival. |  |
| Certainty of evidence of test result/management How certain is the link between test results and management decisions? | | |
| Judgment | Research evidence | ADDITIONAL considerations |
| ○ Very low  ○ Low  ○ Moderate  ○ High  ● No included study  ○ Do not know | Since additional testing of miliary tuberculosis is usually initiated promptly based on the test results, it seems reasonable to assume that there is a high degree of certainty regarding the relationship between the test results and management decisions. However, no studies have been included as evidence. |  |
| Certainty of effects What is the overall certainty of the evidence of effects of the test? | | |
| Judgment | Research evidence | ADDITIONAL considerations |
| ○ Very low  ○ Low  ○ Moderate  ○ High  ● No included study  ○ Do not know | No studies have been included as evidence. |  |
| Values Is there important uncertainty about or variability in how much people value the main outcomes? | | |
| Judgment | Research evidence | ADDITIONAL considerations |
| ○ Important uncertainty or variability  ● Possibly important uncertainty or variability  ○ Probably no important uncertainty or variability  ○ No important uncertainty or variability | The value of desirable effects such as true positives and undesirable effects such as false positives and adverse events may vary according to the values and experiences of individual healthcare providers and patients. |  |
| Balance of effects Does the balance between desirable and undesirable effects favor the intervention or the comparison? | | |
| Judgment | Research evidence | ADDITIONAL considerations |
| ○ Favors the test  ○ Probably favors the test  ○ Does not favor either the test or the comparison  ○ Probably favors the comparison  ○ Favors the comparison  ○ Varies  ● Do not know | The balance between the benefit of appropriate treatment due to true positive diagnosis, the harm of unnecessary treatment due to false positive diagnosis, and the adverse event of the test are unknown due to the lack of evidence. |  |
| Acceptability Is the intervention acceptable to key stakeholders? | | |
| Judgment | Research evidence | ADDITIONAL considerations |
| ● Yes  ○ Probably yes  ○ Probably no  ○ No  ○ Varies  ○ Do not know | It is a commonly practiced medical procedure and probably acceptable. |  |
| Feasibility Is the intervention feasible to implement? | | |
| Judgment | Research evidence | ADDITIONAL considerations |
| ○ Yes  ● Probably yes  ○ Probably no  ○ No  ○ Varies  ○ Do not know | Some facilities may not be able to measure IGRA, but they are commonly practiced medical procedures and probably feasible. |  |

# Summary of Judgment

|  | **Judgment** | | | | | | |
| --- | --- | --- | --- | --- | --- | --- | --- |
| **PROBLEM** | No | Probably no | Probably yes | Yes |  | Varies | Do not know |
| **DESIRABLE EFFECTS** | Trivial | Small | Moderate | Large |  | Varies | Do not know |
| **UNDESIRABLE EFFECTS** | Large | Moderate | Small | Trivial |  | Varies | Do not know |
| **CERTAINTY OF EVIDENCE OF TEST ACCURACY** | Very low | Low | Moderate | High |  |  | No included study |
| **CERTAINTY OF THE EVIDENCE OF TEST’S EFFECTS** | Very low | Low | Moderate | High |  |  | No included study |
| **CERTAINTY OF THE EVIDENCE OF MANAGEMENT’S EFFECTS** | Very low | Low | Moderate | High |  |  | No included study |
| **CERTAINTY OF THE EVIDENCE OF TEST RESULT/MANAGEMENT** | Very low | Low | Moderate | High |  |  | No included study |
| **CERTAINTY OF EFFECT** | Very low | Low | Moderate | High |  |  | No included study |
| **VALUES** | Important uncertainty or variability | Possibly important uncertainty or variability | Probably no important uncertainty or variability | No important uncertainty or variability |  |  |  |
| **BALANCE OF EFFECTS** | Favors the comparison | Probably favors the comparison | Does not favor either the test or the comparison | Probably favors the test | Favors the test | Varies | Do not know |
| **ACCEPTABILITY** | No | Probably no | Probably yes | Yes |  | Varies | Do not know |
| **FEASIBILITY** | No | Probably no | Probably yes | Yes |  | Varies | Do not know |

# Type of Recommendation

| Strong recommendation against the test | Conditional recommendation against the test | Conditional recommendation for either the test or the comparison | Conditional recommendation for the test | Strong recommendation for the test |
| --- | --- | --- | --- | --- |
| ○ | ○ | ○ | ○ | ○ |

# Conclusions

| Recommendation |
| --- |
| **No specific recommendation can be made on whether chest Xp, chest high-resolution CT, and interferon-γ release assay should be used to differentiate the cause of ARDS (miliary tuberculosis). In the management of ARDS, a chest Xp is used in almost all cases, and high-resolution chest CT and interferon-γ release assay are currently used based on the clinician’s experience and other factors (mentioned in our practice statement).** |
|  |
| Justification |
| **Question**  Should plain chest X-rays, chest high-resolution CT, and interferon γ release assays be used for identifying miliary tuberculosis as the causative disease of ARDS?  **Patients**  Patients with ARDS  **Index test**  Chest Xp, chest HRCT, IGRA  **Purpose, role, and setting of the test**  Differential diagnosis in ARDS management in ER, ICU, or equivalent  **Explanation**  The initial screening test for miliary tuberculosis in adults with ARDS is a chest Xp. However, fine granular shadows may be missed, and chest HRCT may be performed. A random distribution of 1-3 mm nodules in both lungs on chest HRCT is a suspicious finding for miliary tuberculosis. Although IGRA is a convenient screening test that requires only a blood test, it can take several days to get the results.  If the test results are true positive and miliary tuberculosis is correctly suspected, appropriate infection control can be performed; specimens can be obtained from sputum, urine, gastric juice, bronchoscopy, bone marrow puncture, and liver biopsy. If *Mycobacterium tuberculosis* is detected, treatment with anti-tuberculosis drugs can be initiated. If the test results are true negative, miliary tuberculosis can be ruled out, unnecessary testing and treatment can be avoided, and additional testing for a different differential diagnosis or follow-up can be performed. However, if the test leads to a misdiagnosis (false positive), it could cause harm such as unnecessary isolation, invasive testing, and the use of anti-tuberculosis drugs.  A literature search did not reveal any high-quality studies that reported the diagnostic accuracy of chest Xp, chest HRCT and IGRA in diagnosing miliary tuberculosis in patients with ARDS. Although not conducted on ARDS patients, the results of studies examining the diagnostic accuracy of IGRAs in the diagnosis of active tuberculosis are presented for reference. T-Spot® TB has a reported sensitivity of 87.5% (95% CI, 85%-90%) with a specificity of 86.3% (95% CI, 81%-90%), and Quantiferon® TB Gold has a reported sensitivity of 81% (95% CI, 78%-83%) with a specificity of 99.2% (95% CI, 98%-100%) ^1)^.  Since there is no high-quality evidence that examines the diagnostic accuracy of these tests, it is not possible to provide a clear recommendation for this CQ. Therefore, we do not make recommendations based on evidence but only describe the current practice in this CQ.  **Summary of evidence**：  No included studies  **Certainty of the evidence**：  The certainty of the evidence cannot be assessed because there are no studies included.  **Values, balance of effects, acceptability, feasibility**：  The balance between the desirable effects and harm is not known. Acceptability and feasibility will not be a problem.  **Panel meeting**  In the preliminary vote, the median score of “recommended text proposal” was 9, and the disagreement index was 0.132 by the modified Delphi method.  At the panel meeting, only minor issues of description were discussed. Finally, an agreement was reached with the results of a preliminary vote.  **Additional considerations**：  None |

| Subgroup considerations |
| --- |
| In the subgroup of patients with impaired cellular immunity, the interpretation of IGRA results needs to be more cautious because of the increased likelihood of false negative results. In addition, IGRA results in elderly patients should be interpreted with caution, because a past infection may result in false positive results even in the absence of active tuberculosis, or decreased cellular immunity may result in false negative results. |
| Implementation considerations |
| These tests are already being performed in clinical practice and are not likely to cause any problems. |

| Monitoring and evaluation |
| --- |
| After the publication of this medical guideline, it is necessary to collect and monitor information on the status of clinical use and problems in conducting the test using tools such as questionnaires. |
| Research priorities |
| Diagnostic accuracy studies on this clinical question are needed. |

Reference

1) Diel R, Loddenkemper R, Nienhaus A. Evidence-based comparison of commercial interferon-gamma release assays for detecting active TB: a metaanalysis. Chest. 2010 Apr;137(4):952-68. PMID: 20022968.

**CQ12 Should anatomical indices of pathological findings of lung biopsy or chest CT imaging findings be used for predicting prognosis of ARDS patients?**

This CQ includes a systematic review and meta-analysis of the prognostic performance of the above tests and a systematic review and meta-analysis of the frequency of adverse events of in-hospital transfers to lung biopsies, CT rooms, etc. Therefore, the results of multiple search formulas and systematic reviews are included.

1.Search strategy

Frequency of adverse events of CT (intra-hospital transfer)

MEDLINE via PubMed （Search date: 2020/6/3）

| #1 | respiratory distress syndrome,adult[mh] OR shock lung OR acute respiratory distress syndrome OR adult respiratory distress syndrome |
| --- | --- |
| #2 | acute lung injury[mh] OR acute lung injury[tiab] OR acute lung injuries[tiab] |
| #3 | ARDS OR ALI |
| #4 | critical care[mh] OR critical illness[mh] |
| #5 | critical care[tiab] OR critical illness[tiab] OR critically ill[tiab] |
| #6 | tomography,x-ray computed[mh] |
| #7 | CT[tiab] OR HRCT[tiab] |
| #8 | patient transfer[mh] OR transportation of patients[mh] |
| #9 | patient transfer[tiab] OR transportation patient[tiab] OR patients transfer[tiab] OR transportation patients[tiab] OR transport patient[tiab] OR transport patients[tiab] OR intrahospital transport[tiab] |
| #10 | mortality[mh] |
| #11 | length of stay[mh] OR length of stay[tiab] |
| #12 | adverse effect*[tiab] OR adverse event*[tiab] |
| #13 | #1 OR #2 OR #3 OR #4 OR #5 |
| #14 | #6 OR #7 OR #8 OR #9 |
| #15 | #10 OR #11 OR #12 |
| #16 | animal[mh] NOT (animal[mh] AND humans[mh]) |
| #17 | #13 AND #14 AND #15 |
| #18 | #17 NOT #16 |

CENTRAL （Search date: 2020/6/3）

| #1 | MeSH descriptor: [Respiratory Distress Syndrome, Adult] explode all trees |
| --- | --- |
| #2 | MeSH descriptor: [Severe Acute Respiratory Syndrome] explode all trees |
| #3 | MeSH descriptor: [Lung Injury] explode all trees |
| #4 | respiratory distress syndrome adult |
| #5 | respiratory distress syndrome acute |
| #6 | severe acute respiratory syndrome |
| #7 | lung injury:ti,ab |
| #8 | lung injur*:ti,ab |
| #9 | MeSH descriptor: [Critical Illness] explode all trees |
| #10 | critical illness:ti,ab |
| #11 | MeSH descriptor: [Critical Care] explode all trees |
| #12 | critical care:ti,ab |
| #13 | #1 OR #2 OR #3 OR #4 OR #5 OR #6 OR #7 OR #8 OR #9 OR #10 OR #11 OR #12 |
| #14 | MeSH descriptor: [Tomography Scanners, X-Ray Computed] explode all trees |
| #15 | CT:ti,ab OR HRCT:ti,ab |
| #16 | computed tomography:ti,ab,kw |
| #17 | MeSH descriptor: [Transportation of Patients] explode all trees |
| #18 | transportation patient*:ti,ab |
| #19 | MeSH descriptor: [Patient Transfer] explode all trees |
| #20 | patient* transfer:ti,ab |
| #21 | intrahospital transport:ti,ab |
| #22 | #14 OR #15 OR #16 OR #17 OR #18 OR #19 OR #20 OR #21 |
| #23 | MeSH descriptor: [Mortality] explode all trees |
| #24 | mortality:ti,ab |
| #25 | MeSH descriptor: [Length of Stay] explode all trees |
| #26 | "length of stay":ti,ab |
| #27 | "adverse effect*":ti,ab |
| #28 | #23 OR #24 OR #25 OR #26 OR #27 |
| #29 | #13 AND #22 AND #28 |

Frequency of adverse events of lung biopsy in ARDS patients

MEDLINE via PubMed （Search date: 2020/5/22）

| #1 | Respiratory Distress Syndrome, Adult [mh] |
| --- | --- |
| #2 | Acute lung injury [mh] |
| #3 | ALI [tiab] OR ARDS [tiab] |
| #4 | Acute [tiab] AND (lung injur* [tiab] OR respiratory distress [tiab] OR respiratory failure[tiab]) |
| #5 | (Severe [tiab] OR critical*[tiab]) AND (respiratory[tiab] OR hypox* [tiab]) |
| #6 | #1 OR #2 OR #3 OR #4 OR #5 |
| #7 | biopsy[mh] AND lung[mh] |
| #8 | (cryosurger*[tiab] OR cryobiopsy[tiab] OR biopsy[tiab]) AND lung[tiab] |
| #9 | bronchoscopy[mh] |
| #10 | Thoracic Surgery, Video-Assisted[mh] |
| #11 | #7 OR #8 OR #9 OR #10 |
| #12 | #6 AND #11 |
| #13 | animals[mh] NOT human[mh] |
| #14 | #12 NOT #13 |

CENTRAL （Search date: 2020/5/22）

| #1 | [mh "Respiratory Distress Syndrome, Adult"] |
| --- | --- |
| #2 | [mh "Acute lung injury"] |
| #3 | ALI:ti,ab OR ARDS:ti,ab |
| #4 | Acute:ti,ab |
| #5 | lung NEXT injur*:ti,ab |
| #6 | "respiratory distress":ti,ab |
| #7 | "respiratory failure":ti,ab |
| #8 | #4 AND (#5 OR #6 OR #7) |
| #9 | (Severe:ti,ab OR critical*:ti,ab) AND (respiratory:ti,ab OR hypox*:ti,ab) |
| #10 | #1 OR #2 OR #3 OR #8 OR #9 |
| #11 | [mh biopsy] AND [mh lung] |
| #12 | (cryosurger*:ti,ab OR cryobiopsy:ti,ab OR biopsy:ti,ab) AND lung:ti,ab |
| #13 | [mh bronchoscopy] |
| #14 | [mh "Thoracic Surgery, Video-Assisted"] |
| #15 | #11 OR #12 OR #13 OR #14 |
| #16 | #10 AND #15 |
| #17 | [mh animals] NOT [mh human] |
| #18 | #16 NOT #17 |

Prognostic performance of CT and biopsy

MEDLINE via PubMed （Search date: 2020/6/18）

| #1 | Respiratory Distress Syndrome, Adult [mh] |
| --- | --- |
| #2 | Acute lung injury [mh] |
| #3 | ALI [tiab] OR ARDS [tiab] |
| #4 | Acute lung injur* [tiab] OR Acute respiratory distress [tiab] OR Acute respiratory failure[tiab] |
| #5 | "severe respiratory failure"[tiab] |
| #6 | #1 OR #2 OR #3 OR #4 OR #5 |
| #7 | (High Resolution Computed Tomography[tiab] OR HRCT[tiab] OR high-resolution CT[tiab] OR tomography, x-ray computed[mh]) AND lung[mh] |
| #8 | Lung/pathology[mh] OR pulmonary fibrosis/pathology[mh] |
| #9 | ("Biopsy/adverse effects"[Mesh] OR "Biopsy/methods"[Mesh])AND Lung[mh] OR "lung biopsy"[tiab] |
| #10 | #7 OR #8 OR #9 |
| #11 | #6 AND #10 |
| #12 | animals[mh] NOT humans[mh] |
| #13 | #11 NOT #12 |

CENTRAL （Search date: 2020/6/18）

| #1 | [mh "Respiratory Distress Syndrome, Adult"] |
| --- | --- |
| #2 | [mh "Acute lung injury"] |
| #3 | ALI:ti,ab OR ARDS:ti,ab |
| #4 | "Acute lung injury":ti,ab OR "Acute respiratory distress":ti,ab OR "Acute respiratory failure":ti,ab |
| #5 | "severe respiratory failure":ti,ab |
| #6 | {OR #1-#5} |
| #7 | ("High Resolution Computed Tomography":ti,ab OR HRCT:ti,ab OR "high-resolution CT":ti,ab OR [mh "tomography, x-ray computed"]) AND [mh lung] |
| #8 | [mh Lung] OR [mh "pulmonary fibrosis"] |
| #9 | ([mh "Biopsy"] AND [mh Lung]) OR "lung biopsy":ti,ab |
| #10 | {OR #7-#9} |
| #11 | #6 AND #10 |
| #12 | [mh animals] NOT [mh humans] |
| #13 | #11 NOT #12 |

1. Flow diagram

Prognostic performance of CT and biopsy

**Identification**

21 Studies included in qualitative synthesis (CT=6, biopsy=15)

79 Full-text articles assessed for eligibility

2378 records after duplicates removed

2436 records identified through database searching

2398 records identified through database searching

Medline via PubMed (n=2263)

CENTRAL (n=135)

Additional records identified through other sources

Trial registry n=38

Hand search the reference lists n=0

17 Studies included in quantitative synthesis (meta-analysis) (CT=6, biopsy=11)

58 Full-text articles excluded, with reasons:

・Wrong study design (n=31)

・Wrong population (n=8)

・Wrong index test (n=3)

・Wrong outcome (n=7)

・2x2 table unavailable (n=2)

・Patients duplicates (n=6)

Duplicates

n=58

2299 records excluded

**Included**

**Eligibility**

**Screening**

Frequency of adverse events of CT (intra-hospital transfer)

**Identification**

25 Studies included in qualitative synthesis

36 Full-text articles assessed for eligibility

1515 records after duplicates removed

1540 records identified through database searching

1540 records identified through database searching

Medline via PubMed (n=1271)

CENTRAL (n=269)

0 additional records identified through other sources

25 Studies included in quantitative synthesis (meta-analysis)

11 Full-text articles excluded, with reasons:

・Wrong study design (n=5)

・Wrong population (n=3)

・Others (n=3)

Duplicates

n=25

1479 records excluded

**Included**

**Eligibility**

**Screening**

Frequency of adverse events of lung biopsy in ARDS patients

**Identification**

13 Studies included in qualitative synthesis

69 Full-text articles assessed for eligibility

2214 records after duplicates removed

2280 records identified through database searching

2280 records identified through database searching

Medline via PubMed (n=2152)

CENTRAL (n=128)

0 additional records identified through other sources

13 Studies included in quantitative synthesis (meta-analysis)

56 Full-text articles excluded, with reasons:

・Wrong language (n=2)

・Wrong study design (n=29)

・Wrong population (n=14)

・Wrong index test (n=3)

・Others (n=8)

Duplicates

n=66

2145 records excluded

**Included**

**Eligibility**

**Screening**

1. Risk of bias

Prognostic performance of CT and biopsy Adverse events of CT

Adverse events of lung biopsy


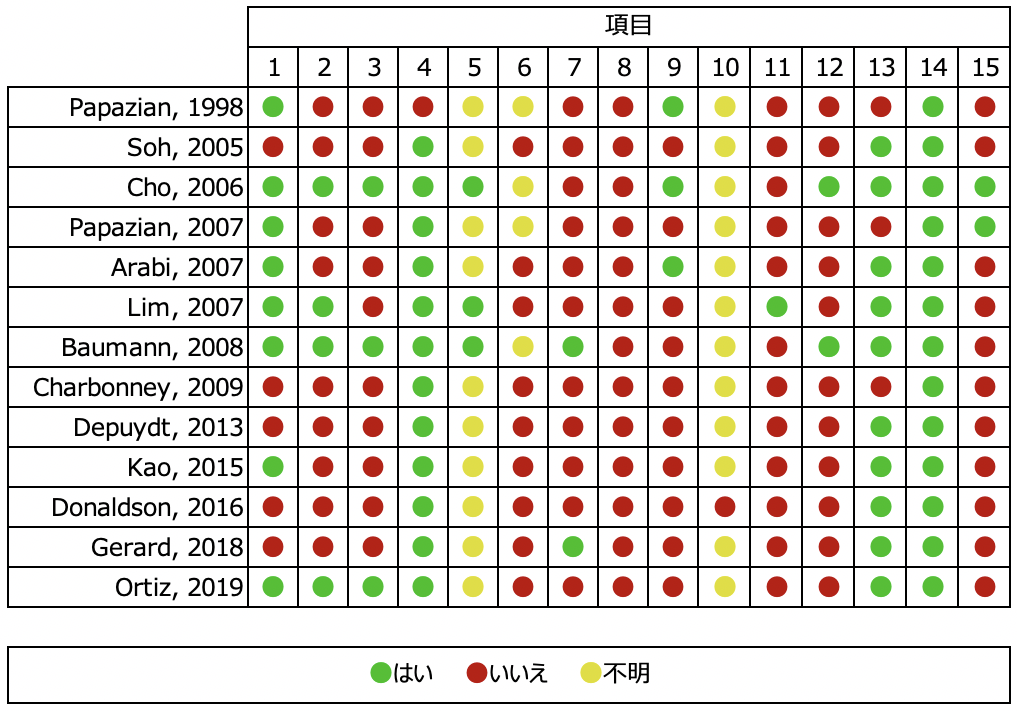


1. Forest plot

Adverse events of CT

Mortality


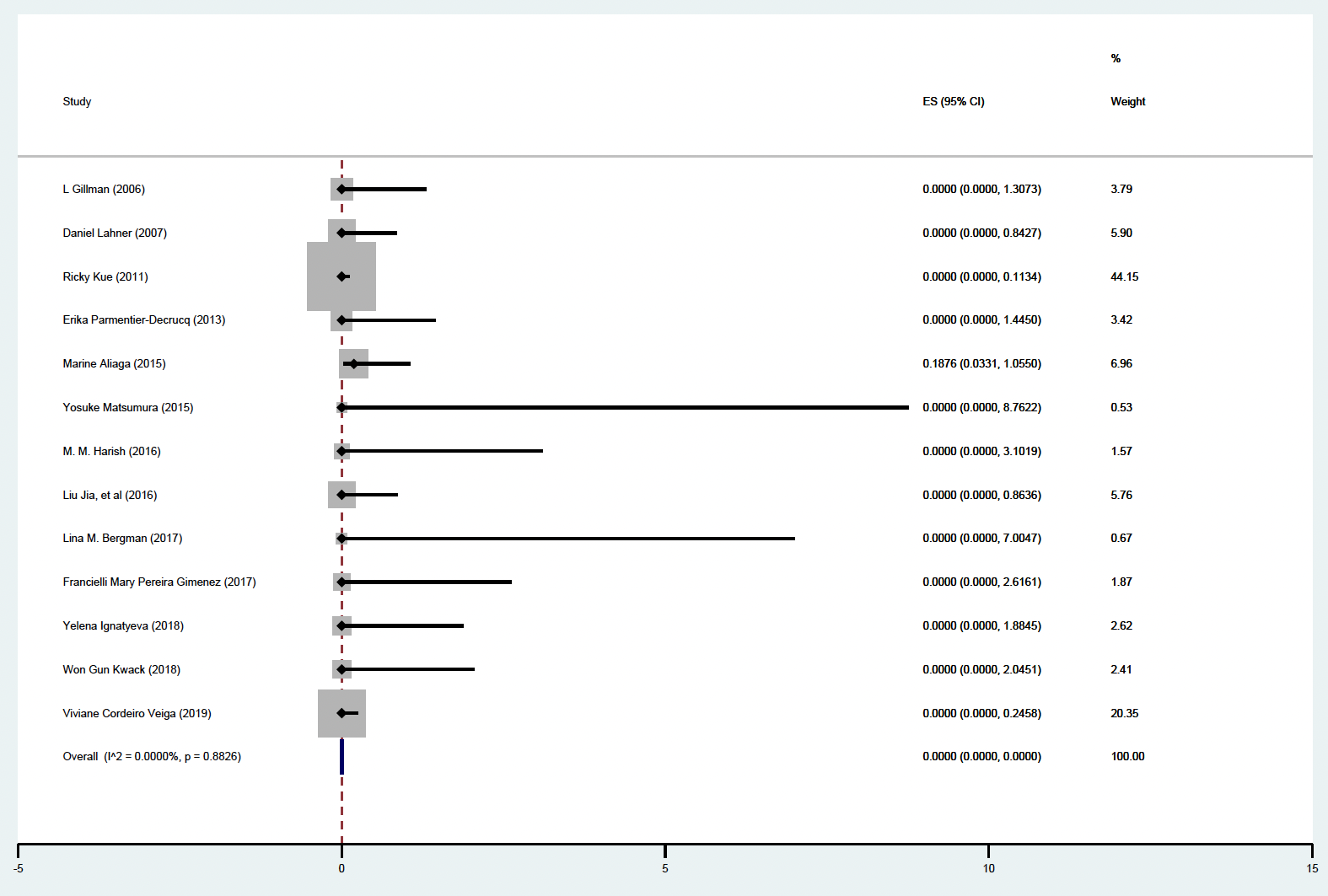


Serious sequelae


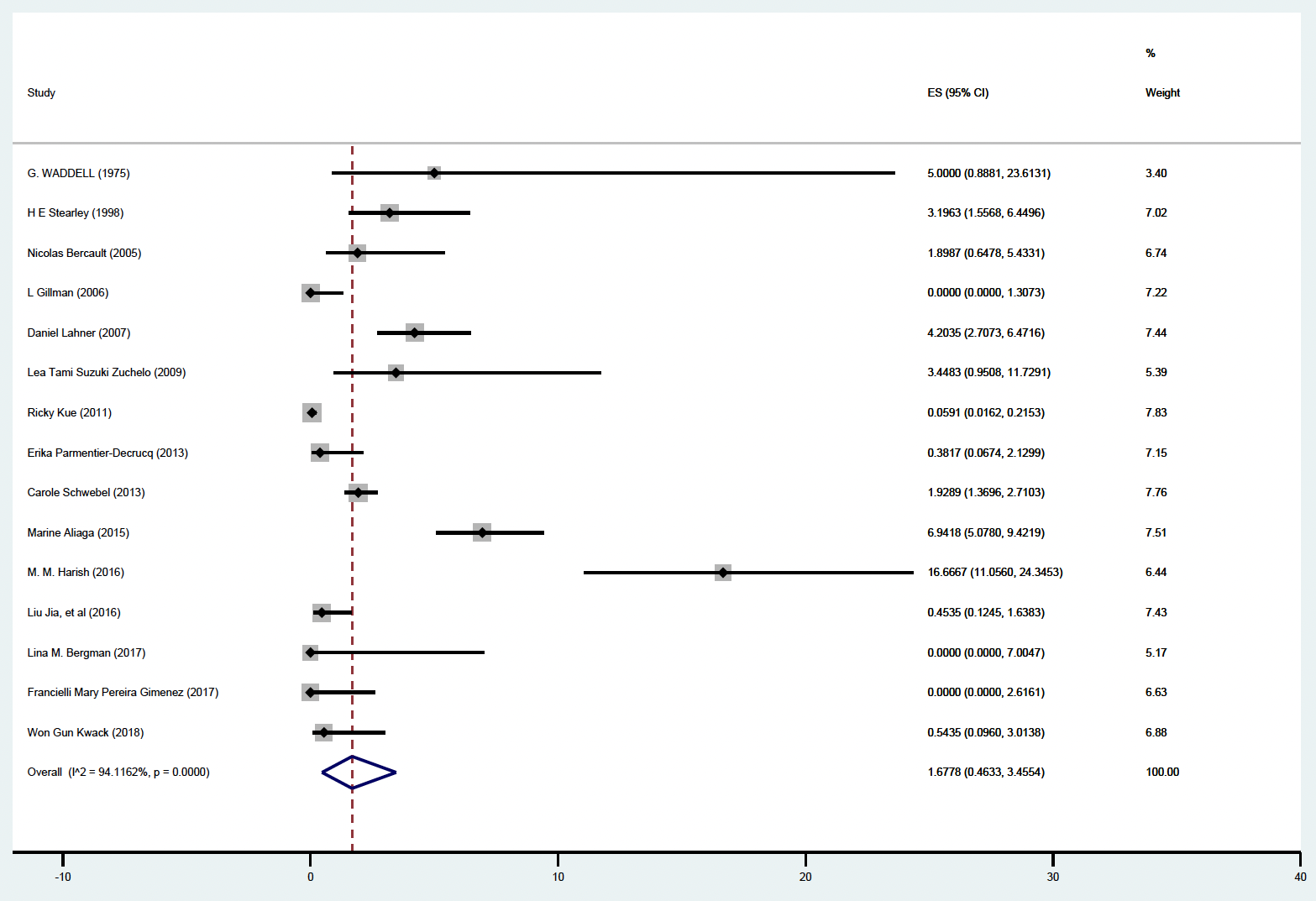


Temporal deterioration of respiratory or cardiovascular status


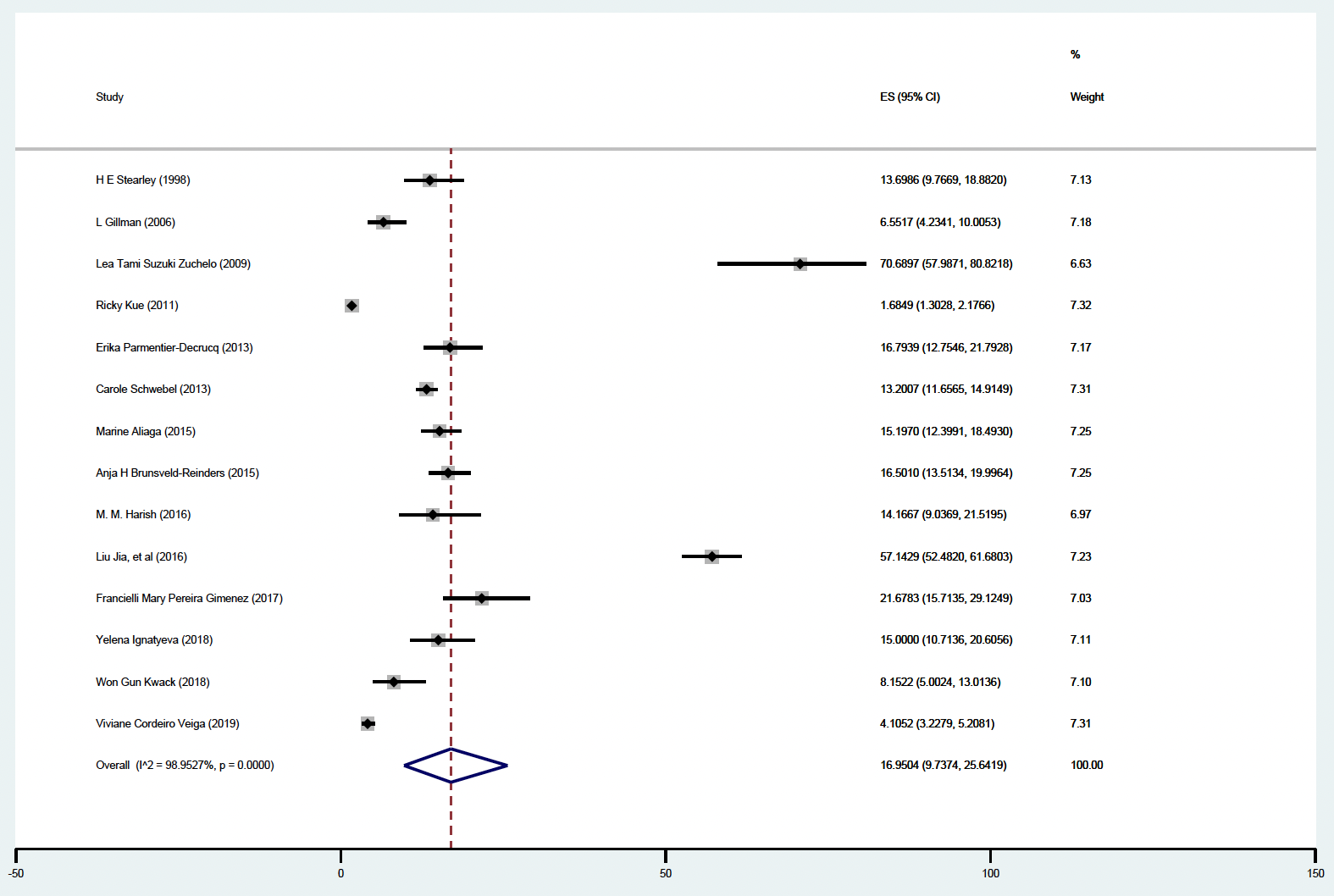


Adverse events of lung biopsy

Biopsy-related death


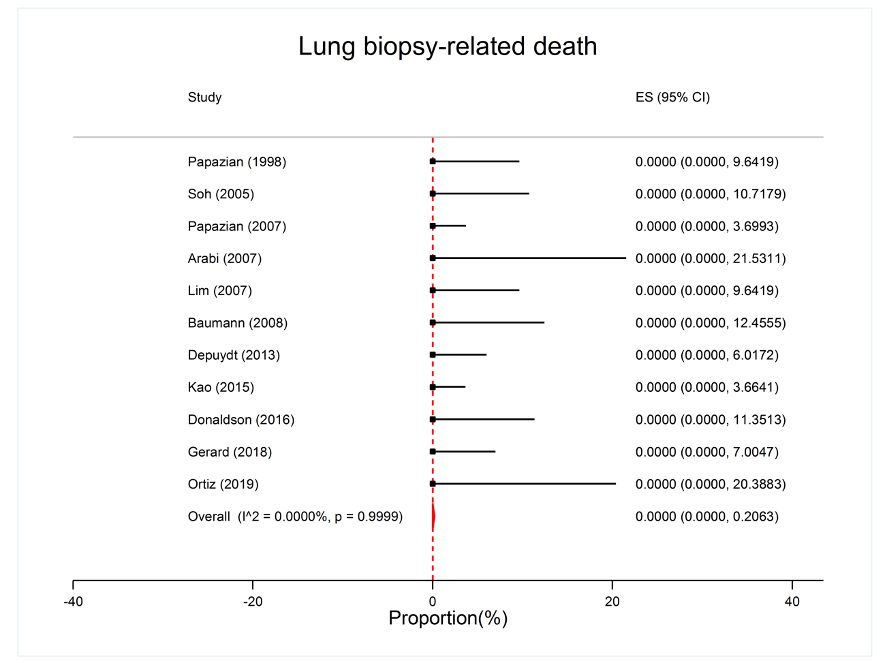


Cardiac complications


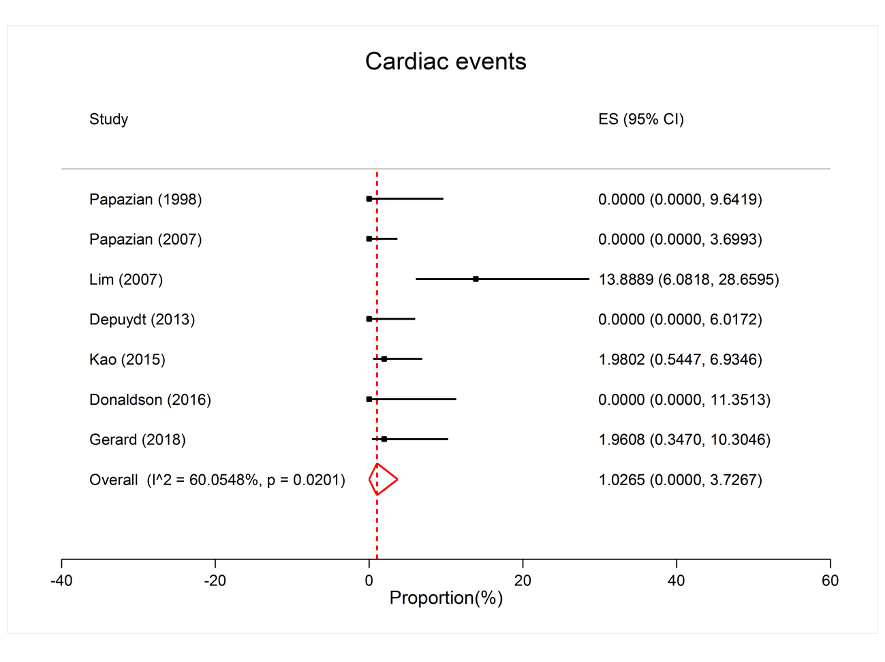


Bleeding


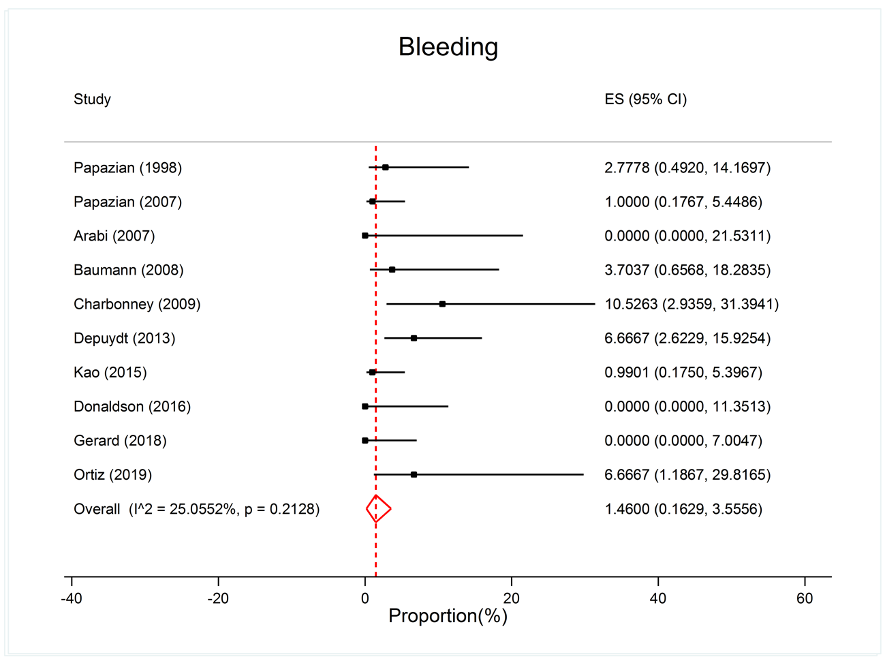


Respiratory failure


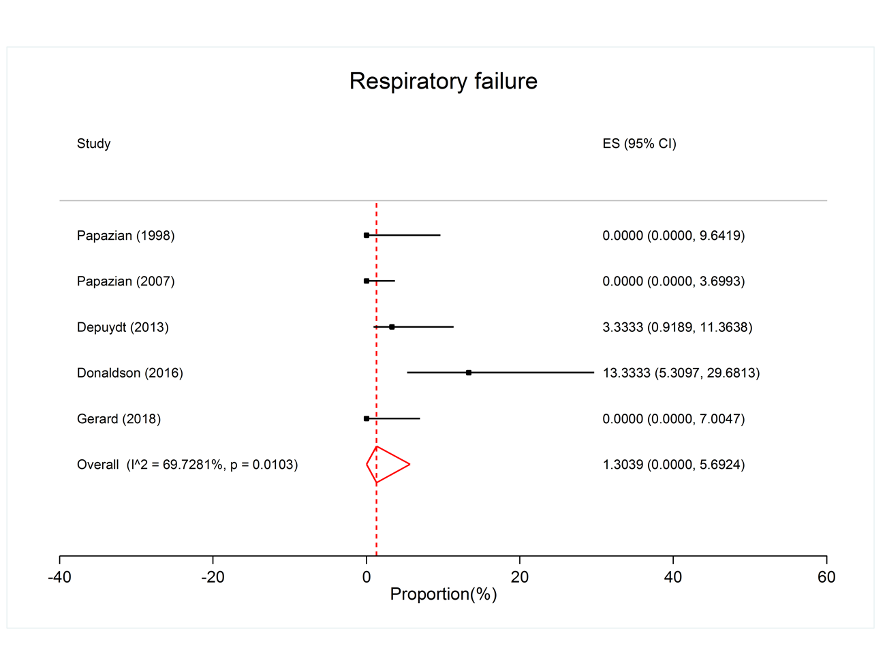


Pneumothorax


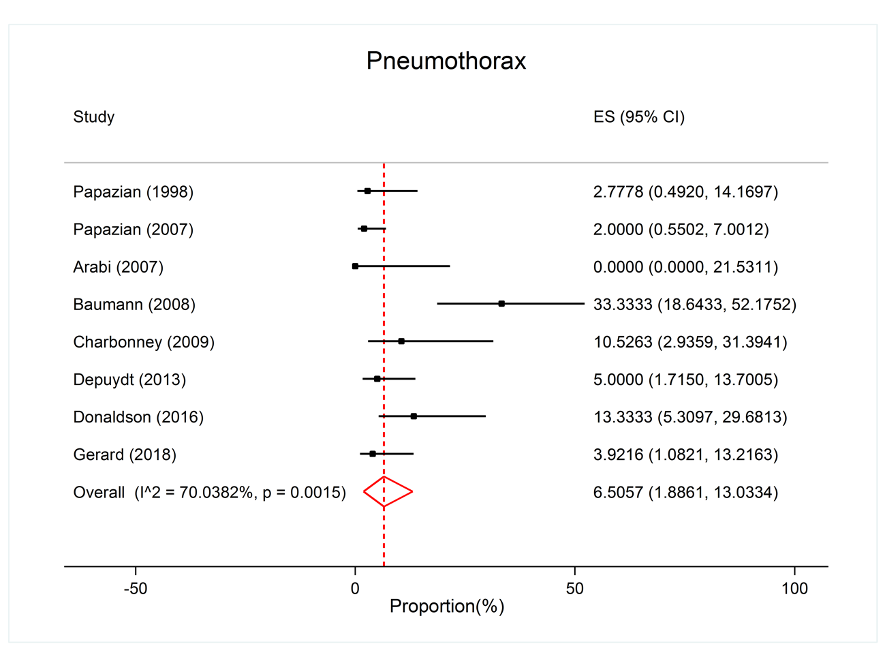


Infection


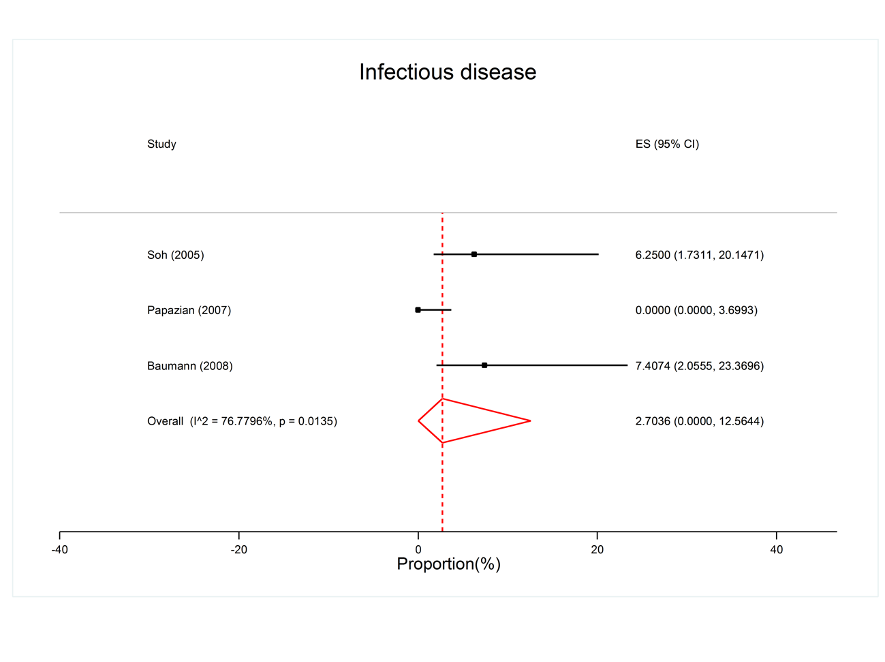


Other major complication


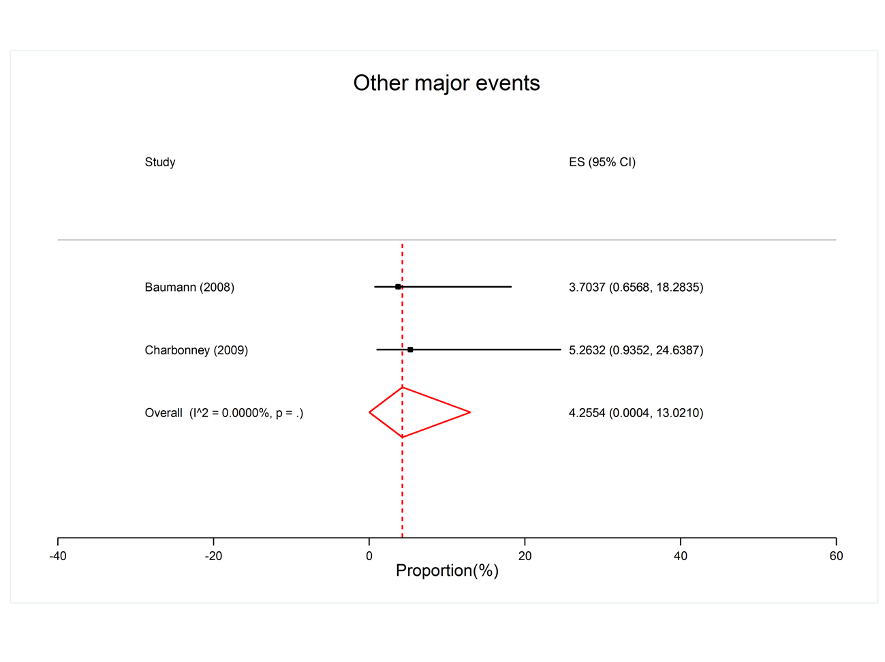


Other minor complications


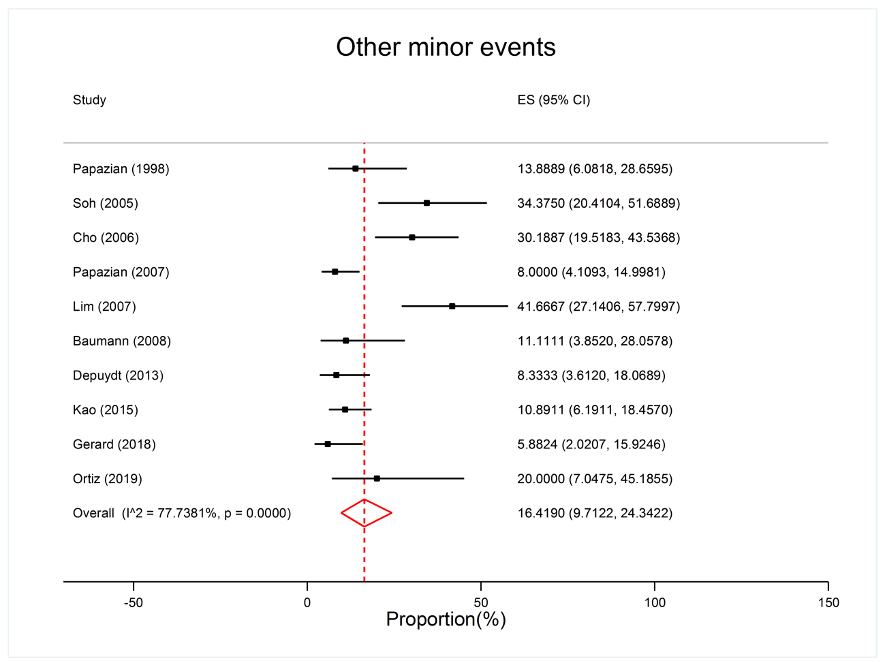


Prognostic performance of CT and biopsy

Lung biopsy (findings of DAD)


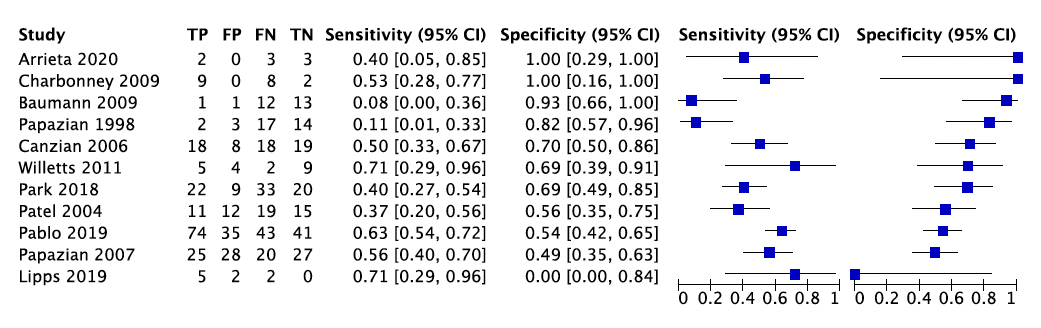


Chest CT


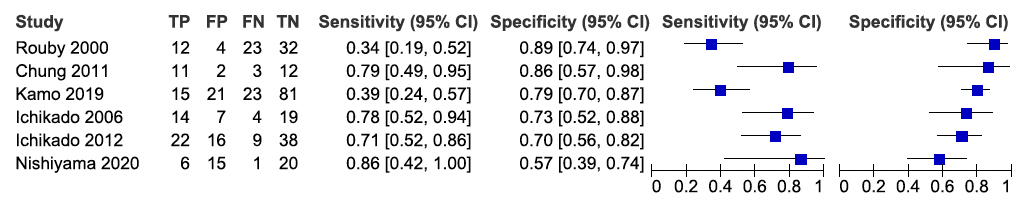


1. Evidence profile

Index test: Lung biopsy (findings of DAD)

| | Sensitivity | 0.42 (95% CI: 0.21 to 0.57) ^1^ | | --- | --- | | Specificity | 0.69 (fixed) | |  | | Prior probability | 25% | 50% | 75% | | --- | --- | --- | --- | |  |
| --- | --- | --- | --- | --- | --- | --- | --- | --- | --- | --- | --- |

| Outcome | No. of studies (patients) | Study design | Assessment of certainty | | | | | Prevalence in 1000 patients | | | Certainty of the evidence |
| --- | --- | --- | --- | --- | --- | --- | --- | --- | --- | --- | --- |
|  |  |  | Risk of bias | Indirectness | Inconsistency | Imprecision | Publication bias | Prior probability 25% | Prior probability 50% | Prior probability 75% |  |
| True Positive | 11 (616) | Cross-sectional study, Cohort study | Serious ^a^ | Not serious ^b^ | Very serious ^c^ | Not serious ^d^ | None | 105 (53 to 143) | 210 (105 to 285) | 315 (158 to 428) | ⨁◯◯◯ Very low |
| False Negative |  |  |  |  |  |  |  | 145 (107 to 197) | 290 (215 to 395) | 435 (322 to 592) |  |
| True Negative | 11 (616) | Cross-sectional study, Cohort study | Serious ^a^ | Not serious ^b^ | Very serious ^c^ | - ^e^ | None | 518 | 345 | 173 | ⨁◯◯◯ Very low |
| False Positive |  |  |  |  |  |  |  | 232 | 155 | 77 |  |

For the definition of positive or negative findings, we used “diffuse alveolar damage” as proposed by Katzenstein et al. in 1976, or “pathological findings as defined in each study.” Eleven studies that used “diffuse alveolar damage” are included. The other four studies were not included due to their clinical heterogeneity.

1. It was calculated using the hierarchical summary receiver operating characteristic (HSROC) model. For sensitivity calculation, specificity was fixed at the median of primary studies. For this reason, no confidence interval is given for the specificity.

a. In terms of risk of bias in the QUADAS-2 tool, the proportion of studies judged to have the high or unknown risk of bias was 3/11 for patient selection, 12/12 for index test, 0/11 for reference standard, and 0/11 for flow and timing. Thus, a certain proportion of studies was determined to denote a high or unknown risk, which was judged to be “Serious.”

b. In terms of concerns about the applicability in the QUADAS-2 tool, the proportion of studies judged to have a high or unknown concern about the applicability was 0/11 for patient selection, 0/11 for index test, 0/11 for reference standard. Thus, the applicability was considered to be maintained because there was less than one category in which the proportion of studies with high or unknown concerns about applicability was approximately more than half. Therefore, we judged the overall applicability to be "Not serious.”

c. We visually assessed the variability in the results of each study included in the systematic review using forest plots.

d. The results may be imprecise because the total number of patients included in the systematic review was below the optimal informative threshold. Additionally, we examined the net benefit of the test (difference between true positives and weighted false positives) if one false positive was considered acceptable for every 2 true positives. When the prevalence rate was set at 25–50%, the net benefit of the test differed at the upper and lower ends of the confidence interval of the integrated sensitivity, which could have changed the clinical judgment. Therefore, the overall uncertainty was judged to be “Very serious.”

e. The imprecision was not assessed because the specificity was fixed when estimating sensitivity using the HSROC model.

Index test: Chest CT

| | Sensitivity | 0.62 (95% CI: 0.32 to 0.91) ^1^ | | --- | --- | | Specificity | 0.76 (fixed) | |  | | Prior probability | 25% | 50% | 75% | | --- | --- | --- | --- | |  |
| --- | --- | --- | --- | --- | --- | --- | --- | --- | --- | --- | --- |

| Outcome | No. of studies (patients) | Study design | Assessment of certainty | | | | | Prevalence in 1000 patients | | | Certainty of the evidence |
| --- | --- | --- | --- | --- | --- | --- | --- | --- | --- | --- | --- |
|  |  |  | Risk of bias | Indirectness | Inconsistency | Imprecision | Publication bias | Prior probability 25% | Prior probability 50% | Prior probability 75% |  |
| True Positive | 6 (409) | Cross-sectional study, Cohort study | Serious ^a^ | Not serious ^b^ | Very serious ^c^ | Very serious ^d^ | None | 155 (80 to 228) | 310 (160 to 455) | 465 (240 to 683) | ⨁◯◯◯ Very low |
| False Negative |  |  |  |  |  |  |  | 95 (22 to 170) | 190 (45 to 340) | 285 (67 to 510) |  |
| True Negative | 6 (409) | Cross-sectional study, Cohort study | Serious ^a^ | Not serious ^b^ | Very serious ^c^ | - ^e^ | None | 570 | 380 | 190 | ⨁◯◯◯ Very low |
| False Positive |  |  |  |  |  |  |  | 180 | 120 | 60 |  |

For the definition of positive or negative findings, we used “CT imaging findings as defined in each study”. Two studies used “Diffuse attenuation,” one used “Lung volumetry,” and one used the “HRCT score.”

1. It was calculated using the hierarchical summary receiver operating characteristic (HSROC) model. For sensitivity calculation, specificity was fixed at the median of primary studies. For this reason, no confidence interval is given for the specificity.

a. In terms of risk of bias in the QUADAS-2 tool, the proportion of studies judged to have ahigh or unknown risk of bias was 3/6 for patient selection, 6/6 for index test, 0/6 for reference standard, and 1/6 for flow and timing. Thus, a certain proportion of studies was determined to denote a high or unknown risk, which was judged to be “Serious.”

b. In terms of concerns about the applicability in the QUADAS-2 tool, the proportion of studies judged to have a high or unknown concern about the applicability was 0/6 for patient selection, 0/6 for index test, 0/6 for reference standard. Thus, the applicability was considered to be maintained because there was less than one category in which the proportion of studies with high or unknown concerns about applicability was approximately more than half. Therefore, we judged the overall applicability to be "Not serious.”

c. We visually assessed the variability in the results of each study included in the systematic review using forest plots.

d. Results may be imprecise because the total number of patients included in the systematic review is below the optimal informative threshold. Additionally, we examined the net benefit of the test (difference between true positives and weighted false positives) if one false positive is considered acceptable for every 2 true positives. When the prevalence rate was set at 25%, the net benefit of the test differed at the upper and lower ends of the confidence interval of the integrated sensitivity, which could have changed the clinical judgment. Therefore, the overall uncertainty was judged to be “Very serious.”

e. The imprecision was not assessed because the specificity was fixed when estimating sensitivity using the HSROC model.

**Frequency of adverse events of lung biopsy in ARDS patients**

| Outcome | No. of studies (patients) | Study design | Summary of the result | | Assessment of certainty | | | | | Certainty of the evidence |
| --- | --- | --- | --- | --- | --- | --- | --- | --- | --- | --- |
|  |  |  | Frequency  (95%CI) | Frequency per 1000 patients (95%CI) | Risk of bias | Indirectness | Inconsistency | Imprecision | Publication bias |  |
| Biopsy-related death | 11 (502) | Cohort study | 0%  (0-0.21) | 0 (0-2) | Very serious ^a^ | Not serious ^c^ | Not serious ^d^ | Serious ^e^ | Not assessed | ⨁⨁◯◯ Low |
| Respiratory failure  (serious complication) | 5 (277) | Cohort study | 1.3%  (0-5.7) | 13 (0-57) | Very serious ^a^ | Not serious ^c^ | Very serious ^d^ | Serious ^e^ | Not assessed | ⨁◯◯◯ Very low |
| Cardiac complications  (serious complication) | 7 (414) | Cohort study | 1.0%  (0-3.7) | 10 (0-37) | Very serious ^a^ | Not serious ^c^ | Serious ^d^ | Serious ^e^ | Not assessed | ⨁◯◯◯ Very low |
| Bleeding  (requiring additional treatment) | 10 (453) | Cohort study | 1.5%  (0.22-3.6) | 15 (2-36) | Very serious ^a^ | Not serious ^c^ | Not serious ^d^ | Serious ^e^ | Not assessed | ⨁⨁◯◯ Low |
| Pneumothorax  (requiring additional treatment) | 8 (337) | Cohort study | 6.5%  (1.9-13) | 65 (19-130) | Very serious ^a^ | Not serious ^c^ | Very serious ^d^ | Very serious ^f^ | Not assessed | ⨁◯◯◯ Very low |
| Infection  (requiring additional treatment) | 3 (159) | Cohort study | 2.7%  (0-13) | 27 (0-130) | Very serious ^a^ | Not serious ^c^ | Very serious ^d^ | Very serious ^f^ | Not assessed | ⨁◯◯◯ Very low |
| Other major complications  (requiring prolonged treatment period) | 2 (46) | Cohort study | 4.3%  (0-13) | 43 (0-130) | Serious ^b^ | Not serious ^c^ | Not serious ^d^ | Very serious ^f^ | Not assessed | ⨁⨁◯◯ Low |
| Other minor complications | 10 (511) | Cohort study | 16%  (10-24) | 160 (100-240) | Very serious ^a^ | Not serious ^c^ | Very serious ^d^ | Serious ^g^ | Not assessed | ⨁◯◯◯ Very low |

#### 説明

a. In terms of the risk of bias in McMaster Quality Assessment Scale for Harms (McHarm), for more than approximately 2/3 of the categories, more than half of the studies had high or unknown risk (see Risk of bias summary), which was judged to be “Very serious.”

b. In terms of the risk of bias in the McHarm, a certain proportion of studies was determined to be a high or unknown risk, which was judged to be “Serious.”

c. In this systematic review, we conducted a literature search using the ideal systematic review question that should be set, and thus the results were generally considered applicable to the clinical situation assumed by guideline readers. Therefore, we judged the applicability to be “Not serious.”

d. The variability in the results of each study included in the systematic review was visually assessed using forest plots and based on the I^2^ statistic.

e. The results may be imprecise because the total number of patients included in the systematic review was below the optimal informative threshold. Clinical judgment was not expected to change between the upper and lower limits of the confidence interval of integrated frequency. Therefore, the overall imprecision is judged to be “Serious.”

f. Results may be imprecise because the total number of patients included in the systematic review is below the optimal informative threshold. Clinical judgment was expected to change between the upper and lower ends of the confidence interval of integrated frequency. Consequently, the overall imprecision was judged to be “Very serious.”

g. The total number of patients included in the systematic review was above the threshold for obtaining optimal information. Clinical judgment was expected to change between the upper and lower ends of the confidence interval of integrated frequency. As a result, the overall imprecision was judged to be “Serious.”

**Frequency of adverse events of CT (intra-hospital transfer)**

| Outcome ^1^ | No. of studies (patients ^2^) | Study design | Summary of the result | | Assessment of certainty | | | | | Certainty of the evidence |
| --- | --- | --- | --- | --- | --- | --- | --- | --- | --- | --- |
|  |  |  | Frequency  (95%CI) | Frequency per 1000 patients (95%CI) | Risk of bias | Indirectness | Inconsistency | Imprecision | Publication bias |  |
| Death ^3^ | 13 (7658) | Cohort study | 0%  (0-0) | 0 (0-0) | Not serious ^a^ | Serious ^b^ | Not serious ^c^ | Not serious ^d^ | Not assessed | ⨁⨁⨁◯ Moderate |
| Serious sequelae ^3^ | 14 (7738) | Cohort study | 1.45% (0.43-2.92) | 15 (4-29) | Not serious ^a^ | Serious ^b^ | Very serious ^c^ | Serious ^d^ | Not assessed | ⨁◯◯◯ Very low |
| Temporal deterioration of respiratory or cardiovascular status ^3^ | 14 (9554) | Cohort study | 17.0% (9.74-25.6) | 170 (97-256) | Not serious ^a^ | Serious ^b^ | Very serious ^c^ | Serious ^d^ | Not assessed | ⨁◯◯◯ Very low |

#### 説明

1. A meta-analysis of the outcome associated with longer treatment days could not be performed because data were not available.

2. In two studies, the number of in-hospital transfers was not available; therefore, the number of patients was used.

3. Those presumed to be directly related to in-hospital transfers were treated as deaths. Serious sequelae and temporal deterioration of respiratory or cardiovascular status were calculated based on our interpretation of adverse events as defined in each study. Serious sequelae mainly include cardiopulmonary arrest that is successfully resuscitated and changes in vital signs that are presumed to have required treatment.

a. In terms of the risk of bias in the Newcastle-Ottawa scale, the proportion of studies judged to have the high or unknown risk of bias was as follows. Death: 0/13 for patient selection, 1/13 for the outcome; serious sequelae: 1/15 for patient selection, 2/15 for the outcome; temporal deterioration of respiratory or cardiovascular status: 1/14 for patient selection, 1/14 for the outcome. Thus, for all three categories, the proportion of studies with a high or unknown risk of bias was less than 1/3, which was judged to be “Not serious.” Some studies were compared with or without in-hospital transfer; however, since the frequency of adverse events was extracted as the outcome, we did not assess the risk regarding the validity of the comparison.

b. In this systematic review, we conducted a literature search not only for patients with ARDS but also for those with acute respiratory failure or critical illness. Owing to this gap from the ideal systematic review question that should be set, the results to the clinical situation assumed by guideline readers have limited direct applicability. Therefore, we judged the overall applicability to be "Serious.”

c. The variability in the results of each study included in the systematic review was visually assessed using forest plots.

d. The total number of patients included in the systematic review was above the threshold for optimal information content. As for death, clinical judgment was not expected to change between the upper and lower ends of the confidence interval of integrated frequency; therefore, the overall imprecision was judged to be “Not serious.” As for serious sequelae and temporal deterioration of respiratory or cardiovascular status, clinical judgment was expected to change between the upper and lower limits of the confidence interval of integrated frequency; therefore, the overall imprecision was judged to be “Serious.”

1. Evidence-to-Decision table

| Question | |
| --- | --- |
| **CQ12： Should anatomical indices of pathological findings of lung biopsy or chest CT imaging findings be used for predicting prognosis of ARDS patients?** | |
| **Population:** | Adult ARDS patients |
| **Target condition:** | Short-term overall survival |
| **Index test:** | Pathological findings of lung biopsy, imaging findings of chest CT |
| **Purpose/role of the test:** | Prediction of prognosis in ARDS management |
| **setting:** | Situation equivalent to the emergency room (ER) or intensive care unit (ICU) |
| **Main outcomes:** | Overall survival, health-related quality of life, family satisfaction, adverse events from testing |
| **Medical practice based on test results:** | If positive (predicted death), change the treatment strategy according to the situation. If negative (predicted survival), continue the current treatment. |
| **perspective:** | Individual |
| **background:** | ARDS is a highly lethal condition, and its prediction of prognosis is important for treatment decision making. Although the typical pathology of ARDS is diffuse alveolar damage (DAD), the Berlin definition does not always reflect the pathology. It has been reported that DAD findings were found in only about half of the autopsy lungs of patients diagnosed with ARDS according to clinical criteria. Some studies have shown that patients with ARDS that had pathological DAD findings on lung biopsy specimens have a worse prognosis than those without DAD findings, suggesting that lung biopsy may be useful in predicting the prognosis of patients with ARDS. On the contrary, chest CT findings, especially high-resolution CT (HRCT) findings, reflect various lung pathologies.  It has also been suggested that the prognosis of patients with ARDS can be predicted from chest CT findings by estimating the presence of DAD. Therefore, the clinical question, “Should anatomical markers (pathological findings of lung biopsy, imaging findings of chest CT) be used to predict the prognosis of ARDS patients?” was considered clinically important. |
| **conflict of interest:** | None |

# Assessment

| Problem Is the problem a priority? | | |
| --- | --- | --- |
| Judgment | Research evidence | ADDITIONAL considerations |
| ● Yes  ○ Probably yes  ○ Probably no  ○ No  ○ Varies  ○ Do not know | It has been suggested that lung biopsy and chest CT may be able to predict the prognosis of patients with ARDS by estimating the presence of DAD findings and pathologic progression. On the contrary, these tests can cause false positives and adverse events. Examining such benefits and harms of these tests is an important issue in ARDS management. Therefore, this clinical question was considered to be of high priority. |  |
| Test accuracy How accurate is the test? | | |
| Judgment | Research evidence | ADDITIONAL considerations |
| ○ Very accurate  ○ Accurate  ● Inaccurate  ○ Very inaccurate  ○ Varies  ○ Do not know | The results of the systematic review and meta-analysis showed the following.  The accuracy of the tests was judged to be “Inaccurate”.  **Lung biopsy (findings of DAD)**  (11 studies, 616 patients)  Integrated sensitivity: 0.42 (95% CI: 0.21-0.57)  Integrated specificity: 0.69 (fixed)  (HSROC model)   |  | Prevalence in 1000 patients | | | Certainty of the evidence | | --- | --- | --- | --- | --- | | Prior probability | 25% | 50% | 75% |  | | True positive | 105 (53 to 143) | 210 (105 to 285) | 315 (158 to 428) | Very low | | False negative | 145 (107 to 197) | 290 (215 to 395) | 435 (322 to 592) | | True negative | 518 | 345 | 173 | Very low | | False positive | 232 | 155 | 77 |   Predicted prognosis: 30-day mortality or in-hospital mortality.  Definition of positive results: pathological findings of DAD  **Chest CT**  (6 studies, 409 patients)  Integrated sensitivity: 0.62 (95% CI: 0.32-0.91)  Integrated specificity: 0.76 (fixed)  (HSROC model)   |  | Prevalence in 1000 patients | | | Certainty of the evidence | | --- | --- | --- | --- | --- | | Prior probability | 25% | 50% | 75% |  | | True positive | 155 (80 to 228) | 310 (160 to 455) | 465 (240 to 683) | Very low | | False negative | 95 (22 to 170) | 190 (45 to 340) | 285 (67 to 510) | | True negative | 570 | 380 | 190 | Very low | | False positive | 180 | 120 | 60 |   Predicted prognosis: 30-day mortality or in-hospital mortality  Definition of positive results: CT imaging findings as defined in each study |  |
| Desirable effects How substantial are the desirable anticipated effects? | | |
| Judgment | Research evidence | ADDITIONAL considerations |
| ○ Large  ○ Moderate  ● Small  ○ Trivial  ○ Varies  ○ Do not know | The number of patients who would benefit from an appropriate change of treatment strategy in a sample of 1000 patients (the number of true positives) was calculated.  The desirable effects of the tests were judged to be “Small.”  **Lung biopsy (findings of DAD)**   | Prior probability of death | 25% | 50% | 75% | | --- | --- | --- | --- | | Decide treatment strategy based on test results | 105 | 210 | 315 | | Change treatment strategy regardless of test results | 250 | 500 | 750 | | Continue current treatment strategy regardless of test results | 0 | 0 | 0 |   **Chest CT**   | Prior probability of death | 25% | 50% | 75% | | --- | --- | --- | --- | | Decide treatment strategy based on test results | 155 | 310 | 465 | | Change treatment strategy regardless of test results | 250 | 500 | 750 | | Continue current treatment strategy regardless of test results | 0 | 0 | 0 | | If all patients were to be treated regardless of the test results, the number of patients who would benefit from treatment was calculated as 1000 x (prior probability).  If all patients were not to be treated regardless of the test results, the number of patients who would benefit from the treatment was considered to be zero. |
| Undesirable effectsHow substantial are the undesirable anticipated effects? | | |
| Judgment | Research evidence | ADDITIONAL considerations |
| ● Large  ○ Moderate  ○ Small  ○ Trivial  ○ Varies  ○ Do not know | The relative clinical weighting of false positives to true positives was set at 0.2. The number of patients who would be harmed by unnecessary change of treatment strategy in a sample of 1000 patients was calculated.  The undesirable effects of the tests were judged to be “Large.”  **Lung biopsy (findings of DAD)**   | Prior probability of death | 25% | 50% | 75% | | --- | --- | --- | --- | | Decide treatment strategy based on test results | 46 | 31 | 15 | | Change treatment strategy regardless of test results | 150 | 100 | 50 | | Continue current treatment strategy regardless of test results | 0 | 0 | 0 |   **Chest CT**   | Prior probability of death | 25% | 50% | 75% | | --- | --- | --- | --- | | Decide treatment strategy based on test results | 36 | 24 | 12 | | Change treatment strategy regardless of test results | 150 | 100 | 50 | | Continue current treatment strategy regardless of test results | 0 | 0 | 0 |   Frequency of adverse events of lung biopsy   |  | Summary of the result | | Certainty of the evidence | | --- | --- | --- | --- | | Outcome | Frequency  (95% CI) | Frequency per 1000 patients (95% CI) | | Biopsy-related death | 0% (0-0.21) | 0[0-2] | Low | | Respiratory failure | 1.3% (0-5.7) | 13[0-57] | Very low | | Cardiac complication | 1.0% (0-3.7) | 10 (0-37) | Very low |   Frequency of adverse events of CT (intra-hospital transfer)   |  | Summary of the result | | Certainty of the evidence | | --- | --- | --- | --- | | Outcome | Frequency  (95% CI) | Frequency per 1000 patients (95% CI) | | Death | 0% (0-0) | 0 [0-0] | Moderate | | Serious sequelae | 1.45% (0.43-2.92) | 15 [4-29] | Very low |   The relative clinical weighting of death to true positives was set at 1.0, and other serious adverse events were set at 0.8.  **Lung biopsy**   | Adverse events |  | | --- | --- | | Biopsy-related death | 0 | | Respiratory failure | 10 | | Cardiac complication | 8 |   **CT (intra-hospital transfer)**   | Adverse events |  | | --- | --- | | Death | 0 | | Serious sequelae | 12 | | If patients were treated based on test results, the number of patients who would be harmed by unnecessary treatment was calculated as (number of false positives) x (clinical weighting).  If all patients were treated regardless of test results, the number of false positives was calculated as (1 - prior probability) x 1000.  If all patients were not treated, the number of false positives was considered to be zero.  The harm of adverse events was calculated by multiplying the frequency of adverse events expected when the test was performed on 1000 people by the clinical weighting. |
| Certainty of evidence What is the overall certainty of the evidence of test accuracy? | | |
| Judgment | Research evidence | ADDITIONAL considerations |
| ● Very low  ○ Low  ○ Moderate  ○ High  ○ No included study | The certainty of the evidence was judged to be “Very low” by adopting the certainty of the evidence with the lowest certainty. |  |
| Certainty of the evidence of test’s effects What is the overall certainty of the evidence for any critical or important direct benefits, adverse effects, or burden of the test? | | |
| Judgment | Research evidence | ADDITIONAL considerations |
| ● Very low  ○ Low  ○ Moderate  ○ High  ○ No included study  ○ Do not know | The certainty of the evidence was judged to be “Low” for adverse events of the tests. |  |
| Certainty of evidence of management’s effects What is the overall certainty of the evidence of effects of the management that is guided by the test results? | | |
| Judgment | Research evidence | ADDITIONAL considerations |
| ○ Very low  ○ Low  ○ Moderate  ○ High  ● No included study  ○ Do not know | No studies have included evidence on the impact of change of treatment strategy on final outcomes such as overall survival. |  |
| Certainty of evidence of test result/management How certain is the link between test results and management decisions? | | |
| Judgment | Research evidence | ADDITIONAL considerations |
| ○ Very low  ○ Low  ○ Moderate  ○ High  ● No included study  ○ Do not know | No studies have been included as evidence. |  |
| Certainty of effects What is the overall certainty of the evidence of effects of the test? | | |
| Judgment | Research evidence | ADDITIONAL considerations |
| ● Very low  ○ Low  ○ Moderate  ○ High  ○ No included study  ○ Do not know | Since the certainty of the evidence of the test’s accuracy is “Very low,” the certainty of the evidence of effects of the test becomes “Very low”. |  |
| Values Is there important uncertainty about or variability in how much people value the main outcomes? | | |
| Judgment | Research evidence | ADDITIONAL considerations |
| ○ Important uncertainty or variability  ● Possibly important uncertainty or variability  ○ Probably no important uncertainty or variability  ○ No important uncertainty or variability | The relative clinical weighting of false positives to true positives may vary depending on the values and experiences of individual health care providers and patients. |  |
| Balance of effects Does the balance between desirable and undesirable effects favor the intervention or the comparison? | | |
| Judgment | Research evidence | ADDITIONAL considerations |
| ○ Favors the test  ○ Probably favors the test  ○ Does not favor either the test or the comparison  ● Probably favors the comparison  ○ Favors the comparison  ○ Varies  ○ Do not know | The net benefit of the test was calculated, considering the benefit of appropriate changes in treatment strategy due to true positive diagnosis, the harm of unnecessary changes in treatment strategy due to false positive diagnosis, and the adverse events of the test.  **Lung biopsy**  Net benefit  (The number of patients who benefit from the test when performed on 1000 patients.)   | Prior probability of death | 25% | 50% | 75% | | --- | --- | --- | --- | | Decide treatment strategy based on test results | 41 | 161 | 282 | | Change treatment strategy regardless of test results | 100 | 400 | 700 | | Continue current treatment strategy regardless of test results | 0 | 0 | 0 |   The relative clinical weight of false positives to true positives was set at 0.2, and the net benefit was calculated with a prior probability of 25-75%. Death related to lung biopsy, respiratory failure, and cardiac complications were adopted as serious adverse events of the test, and their relative clinical weighting were set at 1 and 0.8, respectively. We compared the net benefit of deciding the treatment strategy based on the test results, changing the treatment strategy without testing, and not changing the treatment strategy without testing.  There are likely to be limited clinical situations where there would be a net benefit from testing.  **Chest CT**  Net benefit  (The number of patients who benefit from the test when performed on 1000 patients.)   | Prior probability of death | 25% | 50% | 75% | | --- | --- | --- | --- | | Decide treatment strategy based on test results | 107 | 274 | 441 | | Change treatment strategy regardless of test results | 100 | 400 | 700 | | Continue current treatment strategy regardless of test results | 0 | 0 | 0 |   The relative clinical weight of false positives to true positives was set at 0.2, and the net benefit was calculated with a prior probability of 25-75%. Death and serious sequelae related to intra-hospital transfer were adopted as serious adverse events of the test, and their relative clinical weighting were set at 1 and 0.8, respectively. We compared the net benefit of deciding treatment strategy based on the test results, changing treatment strategy without testing, and not changing treatment strategy without testing.  There are likely to be limited clinical situations where there would be a net benefit from testing. | The net benefit (net benefit) was calculated as (number of true positives receiving appropriate treatment) - (number of false positives receiving unnecessary treatment) × (clinical weighting) - (number of serious adverse events of the test) for a sample of 1000 patients.  If adverse events were considered negligible, they were calculated as zero. |
| Acceptability Is the intervention acceptable to key stakeholders? | | |
| Judgment | Research evidence | ADDITIONAL considerations |
| ○ Yes  ● Probably yes  ○ Probably no  ○ No  ○ Varies  ○ Do not know | It is a commonly practiced medical procedure and considered acceptable. |  |
| Feasibility Is the intervention feasible to implement? | | |
| Judgment | Research evidence | ADDITIONAL considerations |
| ○ Yes  ○ Probably yes  ○ Probably no  ○ No  ● Varies  ○ Do not know | Chest CT can probably be performed at many facilities. The availability of lung biopsy varies by facility. Therefore, the feasibility was judged to be “Varies.” |  |

# Summary of Judgment

|  | **Judgment** | | | | | | |
| --- | --- | --- | --- | --- | --- | --- | --- |
| **PROBLEM** | No | Probably no | Probably yes | Yes |  | Varies | Do not know |
| **DESIRABLE EFFECTS** | Trivial | Small | Moderate | Large |  | Varies | Do not know |
| **UNDESIRABLE EFFECTS** | Large | Moderate | Small | Trivial |  | Varies | Do not know |
| **CERTAINTY OF EVIDENCE OF TEST ACCURACY** | Very low | Low | Moderate | High |  |  | No included study |
| **CERTAINTY OF THE EVIDENCE OF TEST’S EFFECTS** | Very low | Low | Moderate | High |  |  | No included study |
| **CERTAINTY OF THE EVIDENCE OF MANAGEMENT’S EFFECTS** | Very low | Low | Moderate | High |  |  | No included study |
| **CERTAINTY OF THE EVIDENCE OF TEST RESULT/MANAGEMENT** | Very low | Low | Moderate | High |  |  | No included study |
| **CERTAINTY OF EFFECT** | Very low | Low | Moderate | High |  |  | No included study |
| **VALUES** | Important uncertainty or variability | Possibly important uncertainty or variability | Probably no important uncertainty or variability | No important uncertainty or variability |  |  |  |
| **BALANCE OF EFFECTS** | Favors the comparison | Probably favors the comparison | Does not favor either the test or the comparison | Probably favors the test | Favors the test | Varies | Do not know |
| **ACCEPTABILITY** | No | Probably no | Probably yes | Yes |  | Varies | Do not know |
| **FEASIBILITY** | No | Probably no | Probably yes | Yes |  | Varies | Do not know |

# Type of Recommendation

| Strong recommendation against the test | Conditional recommendation against the test | Conditional recommendation for either the test or the comparison | Conditional recommendation for the test | Strong recommendation for the test |
| --- | --- | --- | --- | --- |
| ○ | ● | ○ | ○ | ○ |

# Conclusions

| Recommendation |
| --- |
| **We conditionally recommend that the prognosis of patients with ARDS should not be predicted based solely on the pathological findings of lung biopsy or imaging findings of chest CT. (Conditional recommendation/very low certainty of the evidence: GRADE: 2D)**  **Note: If the clinical situation (characteristics of the target patient, characteristics and timing of the test, prior probability, values of the patient and health care providers) changes, the balance of effects may change, and different options may be recommended.** |
|  |
| Justification |
| **Question**  Should anatomical indices of pathological findings of lung biopsy or chest CT imaging findings be used for predicting prognosis of ARDS patients?  **Patients**  Adult ARDS patients  **Index test**  Pathological findings of lung biopsy, imaging findings of chest CT  **Purpose, role, and setting of the test**  Prediction of prognosis of ARDS patients in ER, ICU, or equivalent  **Medical practice based on test results**  If positive (predicted death), change the treatment strategy according to the situation. If negative (predicted survival), continue the current treatment.  **Summary of evidence**：  Lung biopsy (11 studies, 616 patients) (HSROC model)  Integrated sensitivity: 0.42 (95% CI: 0.21-0.57), Integrated specificity: 0.69 (fixed)  Chest CT (6 studies, 409 patients) (HSROC model)  Integrated sensitivity: 0.62 (95% CI: 0.32-0.91), Integrated specificity: 0.76 (fixed)  **Certainty of the evidence**：  Certainty of the evidence was “Very low.”  **Values, balance of effects, acceptability, feasibility**：  While there are true positive benefits (appropriate changes in treatment strategy) from testing, there are also false positive harms (unnecessary changes in treatment strategy). The relative clinical weight of false positives to true positives was set at 0.2. Considering the net benefit of testing, including serious adverse events, it was thought that there were very limited clinical situations in which treatment decisions based on test results would be useful. The feasibility of the test was judged to be varied.  **Panel meeting**  In the preliminary vote, the median score of “recommended text proposal” was 9, and the disagreement index was 0.132 by the modified Delphi method.  At the panel meeting, an agreement was reached based on the results of the preliminary vote, but later, it was determined necessary to revise the description of medical management based on test results. Corrections were made to the descriptions in the relevant sections, and a final agreement was reached in a second vote.  **Additional considerations**：  The balance of effects depends on the prior probability, the clinical weighting of false positives, and the performance of the test. Therefore, if the clinical situation (characteristics of the patient, characteristics and timing of the test, prior probability, the clinical weighting of false positives, and other values held by patients and caregivers) changes, the balance of effects may change, and different options may be recommended. |

| Subgroup considerations |
| --- |
| None |
| Implementation considerations |
| The feasibility of the test may vary depending on the patient and the facility. |

| Monitoring and evaluation |
| --- |
| Continuous evaluation of adverse events of testing is needed. After the publication of this medical guideline, it is necessary to collect and monitor information on the status of clinical use and problems in conducting the test using tools such as questionnaires. |
| Research priorities |
| The problem with the previous studies is that there is variability in the timing of chest CT and lung biopsy and in the imaging and pathological findings that are considered poor prognoses. It is necessary to standardize these conditions and conduct studies to accumulate evidence. |

**CQ13 Should PaO_2_/FIO_2_ (P/F) ratio be used for predicting prognosis of patients with ARDS?**

1.Search strategy

MEDLINE via PubMed （Search date: 2020/6/19）

| #1 | Respiratory Distress Syndrome, Adult [mh] |
| --- | --- |
| #2 | Acute lung injury [mh] |
| #3 | ALI [tiab] OR ARDS [tiab] |
| #4 | Acute [tiab] AND ("lung injur*" [tiab] OR "respiratory distress" [tiab] OR "respiratory failure"[tiab] OR "distress syndrome"[tiab]) |
| #5 | "severe respiratory failure"[tiab] |
| #6 | "hypoxic respiratory failure"[tiab] |
| #7 | #1 OR #2 OR #3 OR #4 OR #5 OR #6 |
| #8 | "severity of illness index"[MeSH Terms] |
| #9 | oxygenation index[tiab] OR OI[tiab] |
| #10 | PaO2/FiO2[tiab] OR P/F[tiab] |
| #11 | #8 OR #9 OR #10 |
| #12 | #7 AND #11 |
| #13 | animals[mh] NOT humans[mh] |
| #14 | #12 NOT #13 |

CENTRAL （Search date: 2020/6/19）

| #1 | [mh "Respiratory Distress Syndrome, Adult"] |
| --- | --- |
| #2 | [mh "Acute lung injury"] |
| #3 | ALI:ti,ab OR ARDS:ti,ab |
| #4 | Acute:ti,ab AND ("lung injury":ti,ab OR "respiratory distress":ti,ab OR "respiratory failure":ti,ab OR "distress syndrome":ti,ab) |
| #5 | "severe respiratory failure":ti,ab |
| #6 | "hypoxic respiratory failure":ti,ab |
| #7 | {OR #1-#6} |
| #8 | [mh "severity of illness index"] |
| #9 | "oxygenation index":ti,ab OR OI:ti,ab |
| #10 | "PaO2/FiO2":ti,ab OR "P/F":ti,ab |
| #11 | {OR #8-#10} |
| #12 | #7 AND #11 |
| #13 | [mh animals] NOT [mh humans] |
| #14 | #12 NOT #13 |

1. Flow diagram

**Identification**

28 Studies included in qualitative synthesis

331 Full-text articles assessed for eligibility

3726 records after duplicates removed

4055 records identified through database searching

4055 records identified through database searching

Medline via PubMed (n=3599)

CENTRAL (n=456)

0 additional records identified through other sources

23 Studies included in quantitative synthesis (meta-analysis)

302 Full-text articles excluded, with reasons:

・Wrong language (n=1)

・Wrong study design or

wrong index test (n=120)

・Wrong population (n=5)

・Wrong outcome (n=23)

・Others (n=153)

Duplicates

n=329

3395 records excluded

**Included**

**Eligibility**

**Screening**

1. Risk of bias

Risk of bias Applicability Concerns


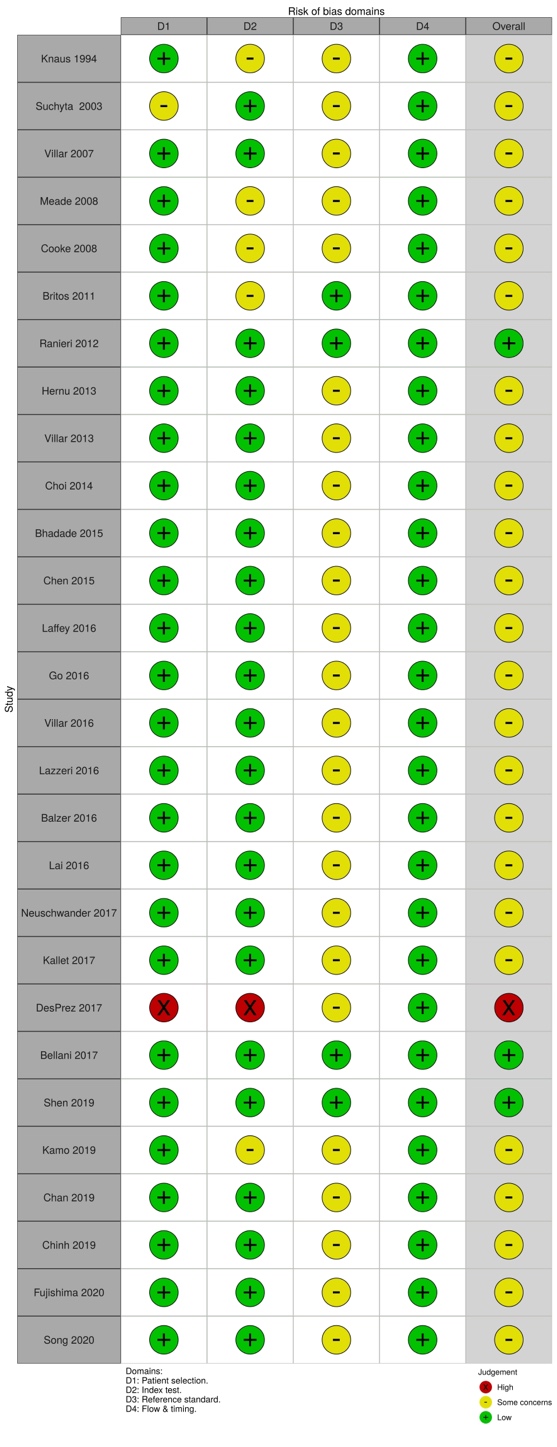

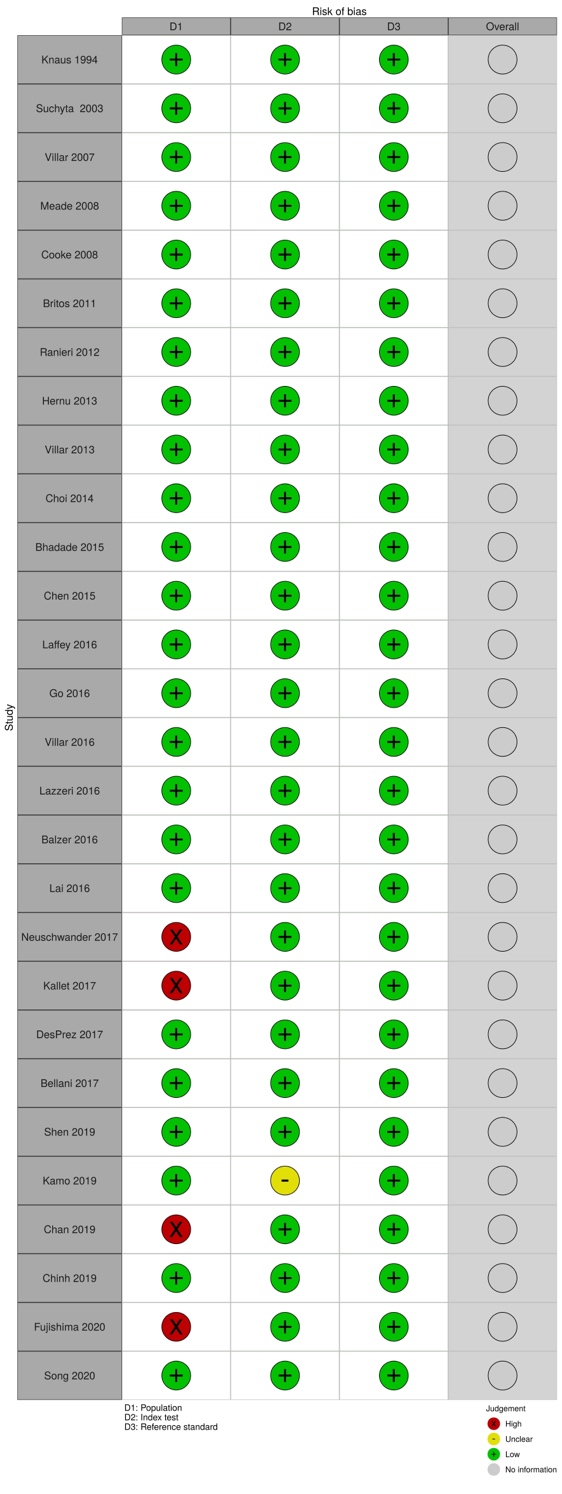


1. Forest plot

P/F ratio (cut-off 100)


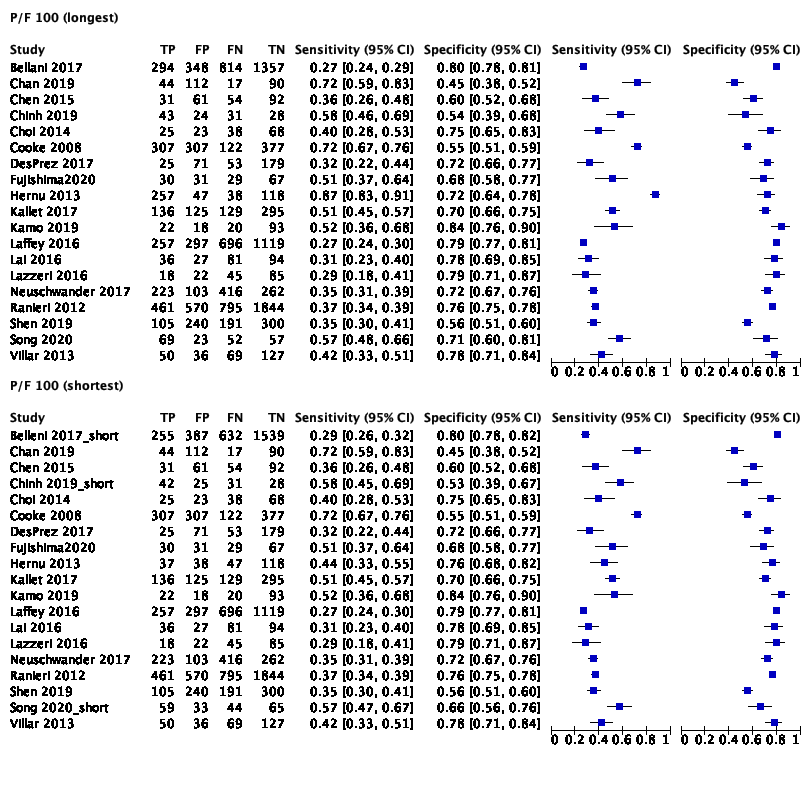


P/F ratio (cut-off 200)


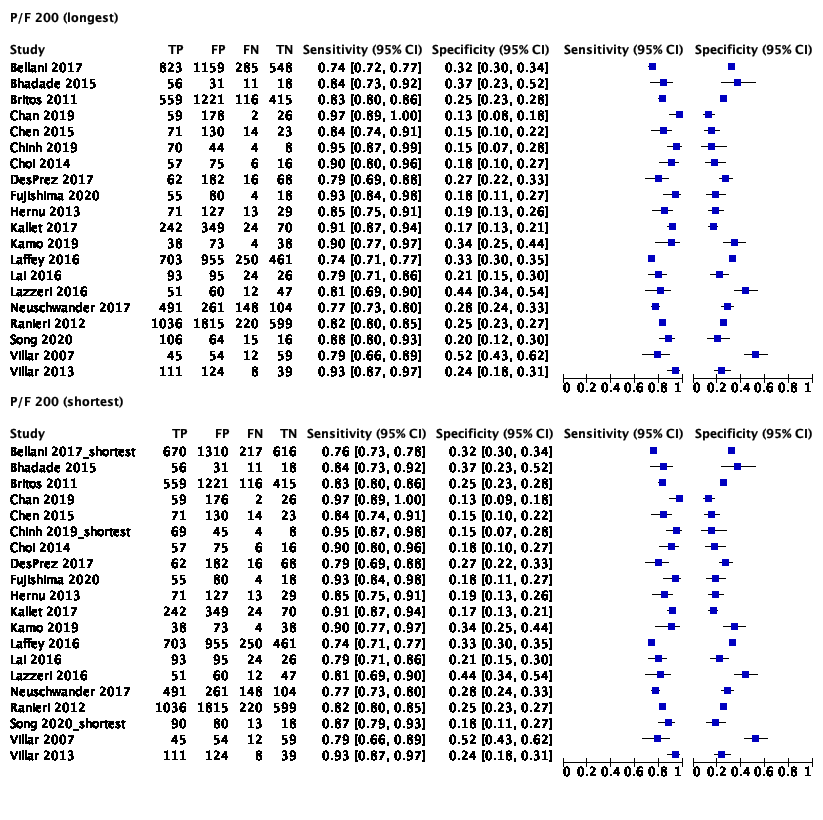


1. Evidence profile

Index test: P/F ratio (cut-off 100)

| | Sensitivity | 0.43 (95% CI: 0.37 to 0.50) | | --- | --- | | Specificity | 0.70 (95% CI: 0.66 to 0.74) | |  | | Prior probability | 25% | 50% | 75% | | --- | --- | --- | --- | |  |
| --- | --- | --- | --- | --- | --- | --- | --- | --- | --- | --- | --- |

| Outcome ^1^ | No. of studies (patients) | Study design | Assessment of certainty | | | | | Prevalence in 1000 patients | | | Certainty of the evidence |
| --- | --- | --- | --- | --- | --- | --- | --- | --- | --- | --- | --- |
|  |  |  | Risk of bias | Indirectness | Inconsistency | Imprecision | Publication bias | Prior probability 25% | Prior probability 50% | Prior probability 75% |  |
| True Positive | 19 (15040) | Cross-sectional study, Cohort study | Serious ^a^ | Not serious ^b^ | Very serious ^c^ | Serious ^d^ | None | 108 (93 to 125) | 215 (185 to 250) | 323 (277 to 375) | ⨁◯◯◯ Very low |
| False Negative |  |  |  |  |  |  |  | 142 (125 to 157) | 285 (250 to 315) | 427 (375 to 473) |  |
| True Negative | 19 (15040) | Cross-sectional study, Cohort study | Serious ^a^ | Not serious ^b^ | Serious ^c^ | Not serious ^e^ | None | 525 (495 to 555) | 350 (330 to 370) | 175 (165 to 185) | ⨁⨁◯◯ Low |
| False Positive |  |  |  |  |  |  |  | 225 (195 to 255) | 150 (130 to 170) | 75 (65 to 85) |  |

1. The deaths during the longest observation period in each study included in the systematic review were adopted as the target condition; “death” was adopted in one study, “ICU death” in two studies, “in-hospital death” in nine studies, “28-day mortality” in two studies, “30-day mortality” in two studies, “60-day mortality” in one study, “death at ICU discharge or hospital discharge or within 90 days” in one study, and “100-day mortality” in one study.

a. In terms of risk of bias in the QUADAS-2 tool, the proportion of studies judged to have high or unknown risk of bias was 1/19 for patient selection, 3/19 for index test, 16/19 for reference standard, and 0/19 for flow and timing. Thus, a certain proportion of studies was determined to indicate high or unknown risk, which was judged to be “Serious.”

b. In terms of concerns about the applicability in the QUADAS-2 tool, the proportion of studies judged to have high or unknown concern about the applicability was 4/19 for patient selection, 1/19 for index test, and 0/19 for reference standard. Thus, the applicability was considered to be maintained because there was less than one category in which the proportion of studies with high or unknown concerns about applicability was approximately more than half.

c. We visually assessed the variability in the results of each study included in the systematic review using forest plots.

d. The total number of patients included in the systematic review was above the threshold for obtaining optimal information. Additionally, we examined the net benefit of the test (difference between true positives and weighted false positives) if one false positive is considered acceptable for every 0.1 true positive. When the prevalence rate was set at 25–50%, the net benefit of the test differed, which could have changed the clinical judgment. Therefore, the overall uncertainty was judged to be “Serious.”

e. The total number of patients included in the systematic review was above the threshold for obtaining optimal information. Additionally, the net benefit of the test (difference between true negatives and weighted false negatives) was examined if one false positive was considered acceptable for every 0.1 true positive. When the prevalence was set at 50% to 75%, the net benefit of the test does not differ between the upper and lower ends of the confidence interval for the integrated specificity, and clinical judgment was not expected to change. Therefore, the overall uncertainty is judged to be “Not serious.”

Index test: P/F ratio (cut-off 200)

| | Sensitivity | 0.85 (95% CI: 0.81 to 0.88) | | --- | --- | | Specificity | 0.24 (95% CI: 0.21 to 0.29) | |  | | Prior probability | 25% | 50% | 75% | | --- | --- | --- | --- | |  |
| --- | --- | --- | --- | --- | --- | --- | --- | --- | --- | --- | --- |

| Outcome ^1^ | No. of studies (patients) | Study design | Assessment of certainty | | | | | Prevalence in 1000 patients | | | Certainty of the evidence |
| --- | --- | --- | --- | --- | --- | --- | --- | --- | --- | --- | --- |
|  |  |  | Risk of bias | Indirectness | Inconsistency | Imprecision | Publication bias | Prior probability 25% | Prior probability 50% | Prior probability 75% |  |
| True Positive | 20 (15489) | Cross-sectional study, Cohort study | Serious ^a^ | Not serious ^b^ | Very serious ^c^ | Serious ^d^ | None | 213  (203 to 220) | 425  (405 to 440) | 638  (608 to 660) | ⨁⨁◯◯ Low |
| False Negative |  |  |  |  |  |  |  | 37  (30 to 47) | 75  (60 to 95) | 112  (90 to 142) |  |
| True Negative | 20 (15489) | Cross-sectional study, Cohort study | Serious ^a^ | Not serious ^b^ | Serious ^c^ | Not serious ^e^ | None | 180  (158 to 217) | 120  (105 to 145) | 60  (53 to 73) | ⨁⨁◯◯ Low |
| False Positive |  |  |  |  |  |  |  | 570  (533 to 592) | 380  (355 to 395) | 190  (177 to 197) |  |

1. The deaths during the longest observation period in each study included in the systematic review were adopted as the target condition; “death” was adopted in one study, “ICU death” in four studies, “in-hospital death” in seven studies, “28-day mortality” in two studies, “30-day mortality” in two studies, “60-day mortality” in one study, “death at ICU discharge or hospital discharge or within 90 days” in one study, and “100-day mortality” in one study.

a. In terms of risk of bias in the QUADAS-2 tool, the proportion of studies judged to have high or unknown risk of bias was 1/20 for patient selection, 3/20 for index test, 17/20 for reference standard, and 0/20 for flow and timing. Thus, a certain proportion of studies was determined to indicate high or unknown risk, which was judged to be “Serious.”

b. In terms of concerns about the applicability in the QUADAS-2 tool, the proportion of studies judged to have high or unknown concern about the applicability was 4/20 for patient selection, 1/20 for index test, and 0/20 for reference standard. Thus, the applicability was considered to be maintained because there was less than one category in which the proportion of studies with high or unknown concerns about applicability was approximately more than half.

c. We visually assessed the variability in the results of each study included in the systematic review using forest plots.

d. The total number of patients included in the systematic review was above the threshold for obtaining optimal information. Additionally, we examined the net benefit of the test (difference between true positives and weighted false positives) if one false positive is considered acceptable for every 0.1 true positive. When the prevalence rate was set at 25–50%, the net benefit of the test differed, which could have changed the clinical judgment. Therefore, the overall uncertainty was judged to be “Serious.”

e. The total number of patients included in the systematic review was above the threshold for obtaining optimal information. Additionally, the net benefit of the test (difference between true negatives and weighted false negatives) was examined if one false positive was considered acceptable for every 0.1 true positive. When the prevalence was set at 50% to 75%, the net benefit of the test does not differ between the upper and lower ends of the confidence interval for the integrated specificity, and clinical judgment was not expected to change. Therefore, the overall uncertainty is judged to be “Not serious.”

1. Evidence-to-Decision table

| Question | |
| --- | --- |
| **CQ13： Should PaO_2_/F_I_O_2_ (P/F) ratio be used for predicting prognosis of patients with ARDS?** | |
| **Population:** | Adult ARDS patients |
| **Target condition:** | Overall survival |
| **Index test:** | P_a_O_2_/F_I_O_2_ ratio (P/F ratio) |
| **Purpose/role of the test:** | Prediction of prognosis in ARDS management |
| **setting:** | Situation equivalent to the emergency room (ER) or intensive care unit (ICU) |
| **Main outcomes:** | Overall survival, health-related quality of life, family satisfaction, adverse events from testing |
| **Medical practice based on test results:** | If positive (predicted death), change the treatment strategy according to the situation. If negative (predicted survival), continue the current treatment. |
| **perspective:** | Individual |
| **background:** | ARDS is a highly lethal condition, and its prediction of prognosis is important for treatment decision making. In the Berlin definition, the severity of ARDS is classified according to the P/F ratio (Severe: <100, Moderate: 100-200, Mild: 200-300), but its prognostic performance is unclear. Therefore, the clinical question, “Should physiological markers (P_a_O_2_/F_I_O_2_ ratio) be used to predict the prognosis of patients with ARDS?” was considered clinically important. |
| **conflict of interest:** | None |

# Assessment

| Problem Is the problem a priority? | | |
| --- | --- | --- |
| Judgment | Research evidence | ADDITIONAL considerations |
| ● Yes  ○ Probably yes  ○ Probably no  ○ No  ○ Varies  ○ Do not know | In the Berlin definition, the severity of ARDS is classified according to the P/F ratio (Severe: <100, Moderate: 100-200, Mild: 200-300), but its prognostic performance is unclear. Therefore, this clinical question was considered to be of high priority. |  |
| Test accuracy How accurate is the test? | | |
| Judgment | Research evidence | ADDITIONAL considerations |
| ○ Very accurate  ○ Accurate  ○ Inaccurate  ○ Very inaccurate  ● Varies  ○ Do not know | The results of the systematic review and meta-analysis showed the following.  The accuracy of the test was judged to be “Varies” because it depended on the cutoff value of the test.  **P/F ratio**  **<Cutoff 100>**  (19 studies, 15040 patients)  Integrated sensitivity: 0.43 (95% CI: 0.37-0.50)  Integrated specificity: 0.70 (95% CI: 0.66-0.74)  (Bivariate model)   |  | Prevalence in 1000 patients | | | Certainty of the evidence | | --- | --- | --- | --- | --- | | Prior probability | 25% | 50% | 75% |  | | True positive | 108 (93 to 125) | 215 (185 to 250) | 323 (277 to 375) | Very low | | False negative | 142 (125 to 157) | 285 (250 to 315) | 427 (375 to 473) | | True negative | 525 (495 to 555) | 350 (330 to 370) | 175 (165 to 185) | Low | | False positive | 225 (195 to 255) | 150 (130 to 170) | 75 (65 to 85) |   Predicted prognosis: ICU mortality, hospital mortality, or short-term mortality  Timing of P/F ratio measurement: at the time of ARDS diagnosis or within 3 days of ICU admission  **<Cutoff 200>**  (20 studies, 15489 patients)  Integrated sensitivity: 0.85 (95% CI: 0.81-0.88)  Integrated specificity: 0.24 (95% CI: 0.21-0.29)  (Bivariate model)   |  | Prevalence in 1000 patients | | | Certainty of the evidence | | --- | --- | --- | --- | --- | | Prior probability | 25% | 50% | 75% |  | | True positive | 213 (203 to 220) | 425 (405 to 440) | 638 (608 to 660) | Low | | False negative | 37 (30 to 47) | 75 (60 to 95) | 112 (90 to 142) | | True negative | 180 (158 to 217) | 120 (105 to 145) | 60 (53 to 73) | Low | | False positive | 570 (533 to 592) | 380 (355 to 395) | 190 (177 to 197) |   Predicted prognosis: ICU mortality, hospital mortality, or short-term mortality  Timing of P/F ratio measurement: at the time of ARDS diagnosis or within 3 days of ICU admission |  |
| Desirable effects How substantial are the desirable anticipated effects? | | |
| Judgment | Research evidence | ADDITIONAL considerations |
| ○ Large  ○ Moderate  ○ Small  ○ Trivial  ● Varies  ○ Do not know | The number of patients who would benefit from an appropriate change of treatment strategy in a sample of 1000 patients (the number of true positives) was calculated.  The desirable effects of the tests were judged to be “Varies” because it depends on the cutoff value.  **P/F ratio**  **<Cutoff 100>**   | Prior probability of death | 25% | 50% | 75% | | --- | --- | --- | --- | | Decide treatment strategy based on test results | 108 | 215 | 323 | | Change treatment strategy regardless of test results | 250 | 500 | 750 | | Continue current treatment strategy regardless of test results | 0 | 0 | 0 |   **<Cutoff 200>**   | Prior probability of death | 25% | 50% | 75% | | --- | --- | --- | --- | | Decide treatment strategy based on test results | 213 | 425 | 638 | | Change treatment strategy regardless of test results | 250 | 500 | 750 | | Continue current treatment strategy regardless of test results | 0 | 0 | 0 | | If all patients were to be treated regardless of the test results, the number of patients who would benefit from treatment was calculated as 1000 x (prior probability).  If all patients were not to be treated regardless of the test results, the number of patients who would benefit from the treatment was considered to be zero. |
| Undesirable effectsHow substantial are the undesirable anticipated effects? | | |
| Judgment | Research evidence | ADDITIONAL considerations |
| ○ Large  ○ Moderate  ○ Small  ○ Trivial  ● Varies  ○ Do not know | The relative clinical weighting of false positives to true positives was set at 0.2. The number of patients who would be harmed by unnecessary change of treatment strategy in a sample of 1000 patients was calculated.  The undesirable effects of the tests were judged to be “Varies” because it depends on the cutoff value.  **P/F ratio**  **<Cutoff 100>**   | Prior probability of death | 25% | 50% | 75% | | --- | --- | --- | --- | | Decide treatment strategy based on test results | 45 | 30 | 15 | | Change treatment strategy regardless of test results | 150 | 100 | 50 | | Continue current treatment strategy regardless of test results | 0 | 0 | 0 |   **<Cutoff 200>**   | Prior probability of death | 25% | 50% | 75% | | --- | --- | --- | --- | | Decide treatment strategy based on test results | 114 | 76 | 38 | | Change treatment strategy regardless of test results | 150 | 100 | 50 | | Continue current treatment strategy regardless of test results | 0 | 0 | 0 | | If patients were treated based on test results, the number of patients who would be harmed by unnecessary treatment was calculated as (number of false positives) x (clinical weighting).  If all patients were treated regardless of test results, the number of false positives was calculated as (1 - prior probability) x 1000.  If all patients were not treated, the number of false positives was considered to be zero.  The harm of adverse events was calculated by multiplying the frequency of adverse events expected when the test was performed on 1000 people by the clinical weighting. |
| Certainty of evidence What is the overall certainty of the evidence of test accuracy? | | |
| Judgment | Research evidence | ADDITIONAL considerations |
| ● Very low  ○ Low  ○ Moderate  ○ High  ○ No included study | The certainty of the evidence was judged to be “Very low” by adopting the certainty of the evidence with the lowest certainty. |  |
| Certainty of the evidence of test’s effects What is the overall certainty of the evidence for any critical or important direct benefits, adverse effects, or burden of the test? | | |
| Judgment | Research evidence | ADDITIONAL considerations |
| ○ Very low  ○ Low  ○ Moderate  ○ High  ● No included study  ○ Do not know | No studies have included evidence of the test’s effects. |  |
| Certainty of evidence of management’s effects What is the overall certainty of the evidence of effects of the management that is guided by the test results? | | |
| Judgment | Research evidence | ADDITIONAL considerations |
| ○ Very low  ○ Low  ○ Moderate  ○ High  ● No included study  ○ Do not know | No studies have included evidence on the impact of change of treatment strategy on final outcomes such as overall survival. |  |
| Certainty of evidence of test result/management How certain is the link between test results and management decisions? | | |
| Judgment | Research evidence | ADDITIONAL considerations |
| ○ Very low  ○ Low  ○ Moderate  ○ High  ● No included study  ○ Do not know | No studies have been included as evidence. |  |
| Certainty of effects What is the overall certainty of the evidence of effects of the test? | | |
| Judgment | Research evidence | ADDITIONAL considerations |
| ● Very low  ○ Low  ○ Moderate  ○ High  ○ No included study  ○ Do not know | Since the certainty of the evidence of the test’s accuracy is “Very low,” the certainty of the evidence of effects of the test becomes “Very low.” |  |
| Values Is there important uncertainty about or variability in how much people value the main outcomes? | | |
| Judgment | Research evidence | ADDITIONAL considerations |
| ○ Important uncertainty or variability  ● Possibly important uncertainty or variability  ○ Probably no important uncertainty or variability  ○ No important uncertainty or variability | The value of desirable effects such as true positives and undesirable effects such as false positives and adverse events may vary according to the values and experiences of individual healthcare providers and patients.  (The relative clinical weighting of false positives to true positives may vary depending on the values and experiences of individual health care providers and patients.) |  |
| Balance of effects Does the balance between desirable and undesirable effects favor the intervention or the comparison? | | |
| Judgment | Research evidence | ADDITIONAL considerations |
| ○ Favors the test  ○ Probably favors the test  ○ Does not favor either the test or the comparison  ○ Probably favors the comparison  ● Favors the comparison  ○ Varies  ○ Do not know | The net benefit of the test was calculated, considering the benefit of appropriate changes in treatment strategy due to true positive diagnosis, the harm of unnecessary changes in treatment strategy due to false positive diagnosis, and the adverse events of the test.  **P/F ratio**  **<Cutoff 100>**  Net benefit  (The number of patients who benefit from the test when performed on 1000 patients.)   | Prior probability of death | 25% | 50% | 75% | | --- | --- | --- | --- | | Decide treatment strategy based on test results | 63 | 185 | 308 | | Change treatment strategy regardless of test results | 100 | 400 | 700 | | Continue current treatment strategy regardless of test results | 0 | 0 | 0 |   The relative clinical weight of false positives to true positives was set at 0.2, and the net benefit was calculated with a prior probability of 25-75%. Serious adverse events of the test were considered to be negligible. We compared the net benefit of deciding the treatment strategy based on the test results, changing the treatment strategy without testing, and not changing the treatment strategy without testing.  There are likely to be very limited clinical situations where there would be a net benefit from testing.  **<Cutoff 200>**  Net benefit  (The number of patients who benefit from the test when performed on 1000 patients.)   | Prior probability of death | 25% | 50% | 75% | | --- | --- | --- | --- | | Decide treatment strategy based on test results | 99 | 349 | 600 | | Change treatment strategy regardless of test results | 100 | 400 | 700 | | Continue current treatment strategy regardless of test results | 0 | 0 | 0 |   The relative clinical weight of false positives to true positives was set at 0.2, and the net benefit was calculated with a prior probability of 25-75%. Serious adverse events of the test were considered to be negligible. We compared the net benefit of deciding treatment strategy based on the test results, changing treatment strategy without testing, and not changing treatment strategy without testing.  There are likely to be very limited clinical situations where there would be a net benefit from testing. | The net benefit (net benefit) was calculated as (number of true positives receiving appropriate treatment) - (number of false positives receiving unnecessary treatment) × (clinical weighting) - (number of serious adverse events of the test) for a sample of 1000 patients.  If adverse events were considered negligible, they were calculated as zero. |
| Acceptability Is the intervention acceptable to key stakeholders? | | |
| Judgment | Research evidence | ADDITIONAL considerations |
| ● Yes  ○ Probably yes  ○ Probably no  ○ No  ○ Varies  ○ Do not know | It is a commonly practiced medical procedure and considered acceptable. |  |
| Feasibility Is the intervention feasible to implement? | | |
| Judgment | Research evidence | ADDITIONAL considerations |
| ● Yes  ○ Probably yes  ○ Probably no  ○ No  ○ Varies  ○ Do not know | It is a commonly practiced medical procedure and considered feasible. |  |

# Summary of Judgment

|  | **Judgment** | | | | | | |
| --- | --- | --- | --- | --- | --- | --- | --- |
| **PROBLEM** | No | Probably no | Probably yes | Yes |  | Varies | Do not know |
| **DESIRABLE EFFECTS** | Trivial | Small | Moderate | Large |  | Varies | Do not know |
| **UNDESIRABLE EFFECTS** | Large | Moderate | Small | Trivial |  | Varies | Do not know |
| **CERTAINTY OF EVIDENCE OF TEST ACCURACY** | Very low | Low | Moderate | High |  |  | No included study |
| **CERTAINTY OF THE EVIDENCE OF TEST’S EFFECTS** | Very low | Low | Moderate | High |  |  | No included study |
| **CERTAINTY OF THE EVIDENCE OF MANAGEMENT’S EFFECTS** | Very low | Low | Moderate | High |  |  | No included study |
| **CERTAINTY OF THE EVIDENCE OF TEST RESULT/MANAGEMENT** | Very low | Low | Moderate | High |  |  | No included study |
| **CERTAINTY OF EFFECT** | Very low | Low | Moderate | High |  |  | No included study |
| **VALUES** | Important uncertainty or variability | Possibly important uncertainty or variability | Probably no important uncertainty or variability | No important uncertainty or variability |  |  |  |
| **BALANCE OF EFFECTS** | Favors the comparison | Probably favors the comparison | Does not favor either the test or the comparison | Probably favors the test | Favors the test | Varies | Do not know |
| **ACCEPTABILITY** | No | Probably no | Probably yes | Yes |  | Varies | Do not know |
| **FEASIBILITY** | No | Probably no | Probably yes | Yes |  | Varies | Do not know |

# Type of Recommendation

| Strong recommendation against the test | Conditional recommendation against the test | Conditional recommendation for either the test or the comparison | Conditional recommendation for the test | Strong recommendation for the test |
| --- | --- | --- | --- | --- |
| ○ | ● | ○ | ○ | ○ |

# Conclusions

| Recommendation |
| --- |
| **We conditionally recommend that the prognosis of patients with ARDS should not be predicted based solely on the P_a_O_2_/F_I_O_2_ ratio (cutoff value 100, 200) (Conditional recommendation/very low certainty of the evidence: GRADE: 2D).**  **Note: In clinical practice, the P/F ratio is used to evaluate the severity of the disease and decide on the treatment strategy in conjunction with other tests.** **This recommendation is not intended to deny such use of the P/F ratio.** **Decisions on treatment strategies should be made comprehensively based on a variety of clinical information, including the P/F ratio.　If the clinical situation (characteristics of the target patient, characteristics and timing of the test, prior probability, values of the patient and health care providers) changes, the balance of effects may change, and different options may be recommended.** |
|  |
| Justification |
| **Question**  Should PaO_2_/F_I_O_2_ (P/F) ratio be used for predicting prognosis of patients with ARDS?  **Patients**  Adult ARDS patients  **Index test**  P/F ratio  **Purpose, role, and setting of the test**  Prediction of prognosis of ARDS patients in ER, ICU, or equivalent  **Medical practice based on test results**  If positive (predicted death), change the treatment strategy according to the situation. If negative (predicted survival), continue the current treatment.  **Summary of evidence**：  P/F ratio  <Cutoff 100> (19 studies, 15040 patients)  Integrated sensitivity: 0.43 (95% CI: 0.37-0.50), Integrated specificity: 0.70 (95% CI: 0.66-0.74)  <Cutoff 200> (20 studies, 15489 patients)  Integrated sensitivity: 0.85 (95% CI: 0.81-0.88), Integrated specificity: 0.24 (95% CI: 0.21-0.29)  **Certainty of the evidence**：  Certainty of the evidence was “Very low.”  **Values, balance of effects, acceptability, feasibility**：  While there are true positive benefits (appropriate changes in treatment strategy) from testing, there are also false positive harms (unnecessary changes in treatment strategy). The relative clinical weight of false positives to true positives was set at 0.2. Adverse events of the test were considered to be negligible. Considering the net benefit, the clinical situations in which using test results to determine a treatment strategy was useful were considered to be very limited.  **Panel meeting**  In the preliminary vote, the median score of “recommended text proposal” was 9, and the disagreement index was 0.192 by the modified Delphi method.  At the panel meeting, an agreement was reached based on the results of the preliminary vote, but later, it was determined necessary to revise the description of medical management based on test results. Corrections were made to the descriptions in the relevant sections, and a final agreement was reached in a second vote.  **Additional considerations**：  The balance of effects depends on the prior probability, the clinical weighting of false positives, and the performance of the test. Therefore, if the clinical situation (characteristics of the patient, characteristics and timing of the test, prior probability, the clinical weighting of false positives, and other values held by patients and caregivers) changes, the balance of effects may change, and different options may be recommended. |

| Subgroup considerations |
| --- |
| None |
| Implementation considerations |
| In clinical practice, the P/F ratio is used to evaluate the severity of the disease and decide on the treatment strategy in conjunction with other tests. This recommendation is not intended to deny such use of the P/F ratio. Decisions on treatment strategies should be made comprehensively based on a variety of clinical information, including the P/F ratio. |

| Monitoring and evaluation |
| --- |
| After the publication of this medical guideline, it is necessary to collect and monitor information on the status of clinical use and problems in conducting the test using tools such as questionnaires. |
| Research priorities |
| A systematic review of predictive models for patients with ARDS, such as clinical predictive scores, is needed. |
