# Supplementary material for: ARDS Clinical Practice Guideline 2021
Source: J Intensive Care. 2022 Jul 8;10:32. doi: 10.1186/s40560-022-00615-6 (PMC9263056; doi:10.1186/s40560-022-00615-6)
Supplement: Supplementary file 2 — Additional file 2. Contains Modified Preferred Reporting items of Systematic Reviews and Meta-Analyses (PRISMA) flow-chart, risk of bias summary, forest plots, evidence profiles, and evidence to decision table for CQ14–18 (area B) according to the GRADE system [file 40560_2022_615_MOESM2_ESM.docx]

Additional file 2

Modified Preferred Reporting items of Systematic Reviews and Meta-Analyses (PRISMA) flow-chart, risk of bias summary, forest plots, evidence profiles, and evidence to decision table for CQ14-18 (area B) according to the GRADE system

Table of contents

1. CQ14
   1. Search strategy p.3
   2. Flow diagram p.3
   3. Risk of bias p.3
   4. Forest plot p.3
   5. Evidence Profile p.3
   6. Evidence-to-Decision table p.4
2. CQ15
   1. Search strategy p.7
   2. Flow diagram p.14
   3. Risk of bias p.16
   4. Forest plot p.17
   5. Evidence Profile p.18
   6. Evidence-to-Decision table p.27
3. CQ16
   1. Search strategy p.35
   2. Flow diagram p.42
   3. Risk of bias p.44
   4. Forest plot p.45
   5. Evidence Profile p.46
   6. Evidence-to-Decision table p.55
4. CQ17
   1. Search strategy p.63
   2. Flow diagram p.70
   3. Risk of bias p.72
   4. Forest plot p.73
   5. Evidence Profile p.74
   6. Evidence-to-Decision table p.83
5. CQ18
   1. Search strategy p.91
   2. Flow diagram p.98
   3. Risk of bias p.100
   4. Forest plot p.101
   5. Evidence Profile p.102
   6. Evidence-to-Decision table p.111

**CQ14 Should non-invasive respiratory support be used for patients with ARDS?**

1. Search strategy

Not applicable

1. Flow diagram

Not applicable

1. Risk of bias

Not applicable

1. Forest plot

Not applicable

1. Evidence Profile

Not applicable

1. Evidence-to-Decision table

| Question | |
| --- | --- |
| **CQ14: Should non-invasive respiratory support be used for patients with ARDS?** | |
| **Population:** | Adult patients aged ≥18 years who had acute hypoxemic respiratory failure defined by new-onset of clinical signs (e.g., tachypnea, increased work of breathing), radiologic signs (unilateral or bilateral chest radiograph opacities), and hypoxemia.  We excluded the randomized controlled trials (RCTs) that included more than half of patients with hypercapnia, congestive heart failure, chronic obstructive pulmonary disease (COPD), or asthma as the cause of respiratory failure, post-extubation respiratory failure, post-surgical, and post-trauma constituting. |
| **Intervention:** | Noninvasive positive pressure ventilation (NPPV), High flow nasal cannula (HFNC) |
| **Comparison:** | Conventional oxygen therapy (COT), Invasive mechanical ventilation (IMV) |
| **Main outcomes:** | Short-term mortality, Endotracheal intubation, Pneumonia |
| **Settings:** | Emergency room or intensive care unit |
| **Perspective:** | Individual |
| **Background:** | Recent clinical practice guidelines do not recommend NIV but HFNO for patients with ARDS (1, 2). Almost half of the patients with ARDS who received noninvasive respiratory support were intubated due to treatment failure (3). Despite the high risk of treatment failure, previous clinical practice guidelines have noted no suggestions based on evidence compared with IMV. It is an important clinical issue to clarify whether noninvasive respiratory support is more effective than COT and IMV. |
| **Conflict of interests:** | None |

# Conclusion

| Recommendation |
| --- |
| **If the patient has no contraindications for noninvasive respiratory support and no organ failure other than respiratory failure, we suggest using noninvasive respiratory support for adult patients with acute hypoxemic respiratory failure who have probable ARDS compared with COT.**  **・NPPV (Conditional recommendation, moderate certainty of evidence: GRADE 2B)**  **・HFNC (Conditional recommendation, moderate certainty of evidence: GRADE 2B)**  **If the patient has no contraindications for noninvasive respiratory support and no organ failure other than respiratory failure, we suggest using noninvasive respiratory support for adult patients with acute hypoxemic respiratory failure who have probable ARDS compared with IMV as initial respiratory management.**  **・NPPV (Conditional recommendation, moderate certainty of evidence: GRADE 2B)**  **・HFNC (Conditional recommendation, moderate certainty of evidence: GRADE 2B)**  **Supplementary condition:**  **Careful observation is needed after initiation of noninvasive respiratory support to avoid delayed intubation which may contribute to poor outcomes.**  **Contraindications for noninvasive respiratory support include the inability to protect the airway, high risk of vomiting, deterioration of consciousness, agitation, and unstable hemodynamics.** |
| **Ranking considerations** |
| The figure shows a cluster ranking plot of a surface under the cumulative ranking curve (SUCRA) which quantifies as average ranking with short-term mortality on the horizontal axis and pneumonia on the vertical axis. The SUCRA value ranges from a minimum of 0 to a maximum of 100. Considering results from the cluster ranking,  noninvasive respiratory support may be a preferable primary management approach compared with COT and IMV.  The probability of being best in reducing short-term mortality among all possible interventions was higher for HFNC (72.0), followed by NPPV (66.3), IMV (49.9), and COT (11.7). The probability of being best in reducing endotracheal intubation among all possible interventions was higher for NPPV (87.7), followed by HFNC (56.0) and COT (6.3). The probability of being best in reducing pneumonia among all possible interventions was higher for HFNC (92.3), followed by NPPV (71.9), COT (33.1), and IMV (2.7).  Figure: Cluster ranking plot  However, the ranking results may be varied by the experience of NPPV. When adapting to clinical practice, we should consider the balance of effects and values, acceptability, and feasibility. Considering the ranking analyses, among noninvasive respiratory support, NPPV was superior to HFNC in reducing endotracheal intubation but was inferior in reducing pneumonia. The effect on short-term mortality was not different among the noninvasive respiratory support techniques. |

References

1. Rochwerg B, Brochard L, Elliott MW, Hess D, Hill NS, Nava S, et al. Official ERS/ATS clinical practice guidelines: noninvasive ventilation for acute respiratory failure. Eur Respir J. 2017;50(2):1602426. PMID: 28860265

2. Rochwerg B, Einav S, Chaudhuri D, Mancebo J, Mauri T, Helviz Y, et al. The role for high flow nasal cannula as a respiratory support strategy in adults: a clinical practice guideline. Intensive Care Med. 2020;46(12):2226-37. PMID: 33201321

3. Bellani G, Laffey JG, Pham T, Madotto F, Fan E, Brochard L, et al. Noninvasive ventilation of patients with acute respiratory distress syndrome. Insights from the LUNG SAFE study. Am J Respir Crit Care Med. 2017;195(1):67-77. PMID: 27753501

**CQ15 Should NPPV be used over oxygen therapy for patients with ARDS?**

1.Search strategy

Short-term mortality/Endotracheal intubation

MEDLINE via PubMed (Search date: 2020/6/19)

| #1 | "Hypoxia"[mh] OR hypox*[tiab] OR "Respiratory Insufficiency"[mh] OR respiratory depression*[tiab] OR respiratory failure*[tiab] OR ventilatory depression*[tiab] OR respiratory insufficienc*[tiab] OR "Dyspnea"[mh] OR dyspnea*[tiab] OR "shortness of breath"[tiab] OR "Respiratory Distress Syndrome, Adult"[mh] OR acute respiratory distress[tiab] OR adult respiratory distress[tiab] OR respiratory distress syndrome*[tiab] OR RDS[tiab] OR ARDS[tiab] OR "Acute Lung Injury"[mh] OR acute lung injur*[tiab] OR ALI[tiab] |
| --- | --- |
| #2 | "Respiratory Distress Syndrome, Newborn"[mh] |
| #3 | #1 or #2 |
| #4 | "Noninvasive Ventilation"[mh] OR noninvasive ventilation*[tiab] OR non invasive ventilation*[tiab] OR NIV[tiab] OR NPPV[tiab] OR NIPPV[tiab] OR noninvasive positive pressure ventilation*[tiab] OR noninvasive mechanical ventilation*[tiab] OR noninvasive pressure support ventilation*[tiab] OR "Continuous Positive Airway Pressure"[mh] OR continuous positive airway pressure*[tiab] OR bilevel positive airway pressure*[tiab] OR biphasic positive airway pressure*[tiab] OR BIPAP[tiab] |
| #5 | "Oxygen Inhalation Therapy"[mh] OR HFNC[tiab] OR HHFNC[tiab] OR HHHFNC[tiab] OR HFNO[tiab] OR HFNT[tiab] OR HFNOT[tiab] OR HFO[tiab] OR HFOT[tiab] OR NHF[tiab] OR NHFC[tiab] OR NHFT[tiab] OR NHFO[tiab] OR NHFOT[tiab] OR high flow therap*[tiab] OR high flow oxygen[tiab] OR nasal high flow[tiab] |
| #6 | #4 or #5 |
| #7 | ("Randomized Controlled Trial"[pt] OR "Controlled Clinical Trial"[pt] OR "Clinical Trials as Topic"[mh] OR randomized[tiab] OR placebo[tiab] OR randomly[tiab] OR trial[tiab] OR groups[tiab]) NOT (Animals [mh] NOT Humans [mh]) |
| #8 | #3 and #6 and #7 |

CENTRAL (Search date: 2020/6/22)

| #1 | [mh Hypoxia] OR hypox*:ti,ab OR [mh "Respiratory Insufficiency"] OR "respiratory depression":ti,ab OR "respiratory failure":ti,ab OR "ventilatory depression":ti,ab OR "respiratory insufficiency":ti,ab OR [mh Dyspnea] OR dyspnea:ti,ab OR "shortness of breath":ti,ab OR [mh "Respiratory Distress Syndrome, Adult"] OR "acute respiratory distress":ti,ab OR "adult respiratory distress":ti,ab OR "respiratory distress syndrome":ti,ab OR RDS:ti,ab OR ARDS:ti,ab OR [mh "Acute Lung Injury"] OR "acute lung injury":ti,ab OR ALI:ti,ab |
| --- | --- |
| #2 | [mh "Respiratory Distress Syndrome, Newborn"] |
| #3 | #1 OR #2 |
| #4 | [mh "Noninvasive Ventilation"] OR "noninvasive ventilation":ti,ab OR "non invasive ventilation":ti,ab OR NIV:ti,ab OR NPPV:ti,ab OR NIPPV:ti,ab OR "noninvasive positive pressure ventilation":ti,ab OR "noninvasive mechanical ventilation":ti,ab OR "noninvasive pressure support ventilation":ti,ab OR [mh "Continuous Positive Airway Pressure"] OR "continuous positive airway pressure":ti,ab OR "bilevel positive airway pressure":ti,ab OR "biphasic positive airway pressure":ti,ab OR BIPAP:ti,ab |
| #5 | [mh "Oxygen Inhalation Therapy"] OR HFNC:ti,ab OR HHFNC:ti,ab OR HHHFNC:ti,ab OR HFNO:ti,ab OR HFNT:ti,ab OR HFNOT:ti,ab OR HFO:ti,ab OR HFOT:ti,ab OR NHF:ti,ab OR NHFC:ti,ab OR NHFT:ti,ab OR NHFO:ti,ab OR NHFOT:ti,ab OR "high flow therapy":ti,ab OR "high flow oxygen":ti,ab OR "nasal high flow":ti,ab |
| #6 | #4 OR #5 |
| #7 | #3 AND #6 |

Igaku-Chuo-Zasshi (Search date: 2020/6/19)

| #1 | 酸素欠乏/TH or 酸素欠乏/TA or anoxia/TA or Hypoxia/TA |
| --- | --- |
| #2 | 呼吸窮迫症候群-急性/TH or 急性呼吸窮迫症候群/TA or ARDS/TA |
| #3 | 急性肺損傷/TH or 急性肺損傷/TA or 急性肺障害/TA or 急性肺傷害/TA or ALI/TA |
| #4 | 呼吸不全/TH or 呼吸不全/TA |
| #5 | 呼吸困難/TH or 呼吸困難/TA |
| #6 | 呼吸窮迫症候群-新生児/TH or 新生児呼吸窮迫症候群/TA |
| #7 | #1 or #2 or #3 or #4 or #5 or #6 |
| #8 | 非侵襲的補助換気/TH or 非侵襲的補助換気/TA or NPPV/TA or NIPPV/TA |
| #9 | 持続気道陽圧/TH or 持続気道陽圧/TA or CPAP/TA |
| #10 | 非侵襲的陽圧呼吸/TH or 非侵襲的陽圧呼吸/TA or BIPAP/TA |
| #11 | 酸素吸入療法/TH or 酸素吸入/TA |
| #12 | 酸素療法/TA or ハイフロー/TA or HFNC/TA or NHF/TA or HFO/TA |
| #13 | #8 or #9 or #10 or #11 or #12 |
| #14 | #7 and #13 |
| #15 | (#14) and (PT=会議録除く) |
| #16 | ランダム化比較試験/TH or ランダム化/AL or 無作為化/AL |
| #17 | 比較試験/AL |
| #18 | 臨床試験/TH or 臨床試験/AL |
| #19 | プラセボ/TH or プラセボ/AL |
| #20 | 対照/AL |
| #21 | コントロール/AL |
| #22 | 臨床研究・疫学研究/TH or 臨床研究/AL |
| #23 | #16 or #17 or #18 or #19 or #20 or #21 or #22 |
| #24 | #15 and #23 |

Pneumonia

MEDLINE via PubMed（Search date: 2020/6/19）

|  | 検索式 |
| --- | --- |
| #1 | "Hypoxia"[mh] OR hypox*[tiab] OR "Respiratory Insufficiency"[mh] OR respiratory depression*[tiab] OR respiratory failure*[tiab] OR ventilatory depression*[tiab] OR respiratory insufficienc*[tiab] OR "Dyspnea"[mh] OR dyspnea*[tiab] OR "shortness of breath"[tiab] OR "Respiratory Distress Syndrome, Adult"[mh] OR acute respiratory distress[tiab] OR adult respiratory distress[tiab] OR respiratory distress syndrome*[tiab] OR RDS[tiab] OR ARDS[tiab] OR "Acute Lung Injury"[mh] OR acute lung injur*[tiab] OR ALI[tiab] |
| #2 | "Respiratory Distress Syndrome, Newborn"[mh] |
| #3 | #1 or #2 |
| #4 | "Noninvasive Ventilation"[mh] OR noninvasive ventilation*[tiab] OR non invasive ventilation*[tiab] OR NIV[tiab] OR NPPV[tiab] OR NIPPV[tiab] OR noninvasive positive pressure ventilation*[tiab] OR noninvasive mechanical ventilation*[tiab] OR noninvasive pressure support ventilation*[tiab] OR "Continuous Positive Airway Pressure"[mh] OR continuous positive airway pressure*[tiab] OR bilevel positive airway pressure*[tiab] OR biphasic positive airway pressure*[tiab] OR BIPAP[tiab] |
| #5 | "Oxygen Inhalation Therapy"[mh] OR HFNC[tiab] OR HHFNC[tiab] OR HHHFNC[tiab] OR HFNO[tiab] OR HFNT[tiab] OR HFNOT[tiab] OR HFO[tiab] OR HFOT[tiab] OR NHF[tiab] OR NHFC[tiab] OR NHFT[tiab] OR NHFO[tiab] OR NHFOT[tiab] OR high flow therap*[tiab] OR high flow oxygen[tiab] OR nasal high flow[tiab] |
| #6 | #4 or #5 |
| #7 | ("Randomized Controlled Trial"[pt] OR "Controlled Clinical Trial"[pt] OR "Clinical Trials as Topic"[mh] OR randomized[tiab] OR placebo[tiab] OR randomly[tiab] OR trial[tiab] OR groups[tiab]) NOT (Animals [mh] NOT Humans [mh]) |
| #8 | #3 and #6 and #7 |
| #9 | "Noninvasive Ventilation/adverse effects"[mh] OR noninvasive ventilation*[tiab] OR non invasive ventilation*[tiab] OR NIV[tiab] OR NPPV[tiab] OR NIPPV[tiab] OR noninvasive positive pressure ventilation*[tiab] OR noninvasive mechanical ventilation*[tiab] OR noninvasive pressure support ventilation*[tiab] OR "Continuous Positive Airway Pressure/adverse effects"[mh] OR continuous positive airway pressure*[tiab] OR bilevel positive airway pressure*[tiab] OR biphasic positive airway pressure*[tiab] OR BIPAP[tiab] |
| #10 | "Oxygen Inhalation Therapy/adverse effects"[mh] OR HFNC[tiab] OR HHFNC[tiab] OR HHHFNC[tiab] OR HFNO[tiab] OR HFNT[tiab] OR HFNOT[tiab] OR HFO[tiab] OR HFOT[tiab] OR NHF[tiab] OR NHFC[tiab] OR NHFT[tiab] OR NHFO[tiab] OR NHFOT[tiab] OR high flow therap*[tiab] OR high flow oxygen[tiab] OR nasal high flow[tiab] |
| #11 | #9 or #10 |
| #12 | harm*[tiab] OR side effect*[tiab] OR adverse[tiab] OR toxicity[tiab] OR infection*[tiab] OR pneumonia*[tiab] OR trauma[tiab] OR barotrauma*[tiab] OR volutrauma*[tiab] OR pneumothorax[tiab] OR ulcer*[tiab] OR discomfort*[tiab] OR breakdown[tiab] OR damage[tiab] OR intoleran*[tiab] OR "Barotrauma/complications"[mh] OR "Pneumonia/complications"[mh] OR "Pressure Ulcer/complications"[mh] OR "Pneumothorax/complications"[mh] OR "Pneumonia, Ventilator-Associated"[mh] |
| #13 | #3 and #11 and #12 |
| #14 | Animals [mh] NOT Humans [mh] |
| #15 | #13 not #14 |
| #16 | #15 not #8 |

CENTRAL（Search date: 2020/6/22）

|  | 検索式 |
| --- | --- |
| #1 | [mh Hypoxia] OR hypox*:ti,ab OR [mh "Respiratory Insufficiency"] OR "respiratory depression":ti,ab OR "respiratory failure":ti,ab OR "ventilatory depression":ti,ab OR "respiratory insufficiency":ti,ab OR [mh Dyspnea] OR dyspnea:ti,ab OR "shortness of breath":ti,ab OR [mh "Respiratory Distress Syndrome, Adult"] OR "acute respiratory distress":ti,ab OR "adult respiratory distress":ti,ab OR "respiratory distress syndrome":ti,ab OR RDS:ti,ab OR ARDS:ti,ab OR [mh "Acute Lung Injury"] OR "acute lung injury":ti,ab OR ALI:ti,ab |
| #2 | [mh "Respiratory Distress Syndrome, Newborn"] |
| #3 | #1 OR #2 |
| #4 | [mh "Noninvasive Ventilation"] OR "noninvasive ventilation":ti,ab OR "non invasive ventilation":ti,ab OR NIV:ti,ab OR NPPV:ti,ab OR NIPPV:ti,ab OR "noninvasive positive pressure ventilation":ti,ab OR "noninvasive mechanical ventilation":ti,ab OR "noninvasive pressure support ventilation":ti,ab OR [mh "Continuous Positive Airway Pressure"] OR "continuous positive airway pressure":ti,ab OR "bilevel positive airway pressure":ti,ab OR "biphasic positive airway pressure":ti,ab OR BIPAP:ti,ab |
| #5 | [mh "Oxygen Inhalation Therapy"] OR HFNC:ti,ab OR HHFNC:ti,ab OR HHHFNC:ti,ab OR HFNO:ti,ab OR HFNT:ti,ab OR HFNOT:ti,ab OR HFO:ti,ab OR HFOT:ti,ab OR NHF:ti,ab OR NHFC:ti,ab OR NHFT:ti,ab OR NHFO:ti,ab OR NHFOT:ti,ab OR "high flow therapy":ti,ab OR "high flow oxygen":ti,ab OR "nasal high flow":ti,ab |
| #6 | #4 OR #5 |
| #7 | #3 AND #6 |
| #8 | [mh "Noninvasive Ventilation"/ae] OR "noninvasive ventilation":ti,ab OR "non invasive ventilation":ti,ab OR NIV:ti,ab OR NPPV:ti,ab OR NIPPV:ti,ab OR "noninvasive positive pressure ventilation":ti,ab OR "noninvasive mechanical ventilation":ti,ab OR "noninvasive pressure support ventilation":ti,ab OR [mh "Continuous Positive Airway Pressure"/ae] OR "continuous positive airway pressure":ti,ab OR "bilevel positive airway pressure":ti,ab OR "biphasic positive airway pressure":ti,ab OR BIPAP:ti,ab |
| #9 | [mh "Oxygen Inhalation Therapy"/ae] OR HFNC:ti,ab OR HHFNC:ti,ab OR HHHFNC:ti,ab OR HFNO:ti,ab OR HFNT:ti,ab OR HFNOT:ti,ab OR HFO:ti,ab OR HFOT:ti,ab OR NHF:ti,ab OR NHFC:ti,ab OR NHFT:ti,ab OR NHFO:ti,ab OR NHFOT:ti,ab OR "high flow therapy":ti,ab OR "high flow oxygen":ti,ab OR "nasal high flow":ti,ab |
| #10 | #8 OR #9 |
| #11 | harm*:ti,ab OR "side effect":ti,ab OR adverse:ti,ab OR toxicity:ti,ab OR infection*:ti,ab OR pneumonia*:ti,ab OR trauma:ti,ab OR barotrauma*:ti,ab OR volutrauma*:ti,ab OR pneumothorax:ti,ab OR ulcer*:ti,ab OR discomfort*:ti,ab OR breakdown:ti,ab OR damage:ti,ab OR intoleran*:ti,ab OR [mh Barotrauma/co] OR [mh Pneumonia/co] OR [mh "Pressure Ulcer"/co] OR [mh Pneumothorax/co] OR [mh "Pneumonia, Ventilator-Associated"] |
| #12 | #3 AND #10 AND #11 |
| #13 | #12 NOT #7 |

Igaku-Chuo-Zasshi （Search date: 2020/6/19）

|  | 検索式 |
| --- | --- |
| #1 | 酸素欠乏/TH or 酸素欠乏/TA or anoxia/TA or Hypoxia/TA |
| #2 | 呼吸窮迫症候群-急性/TH or 急性呼吸窮迫症候群/TA or ARDS/TA |
| #3 | 急性肺損傷/TH or 急性肺損傷/TA or 急性肺障害/TA or 急性肺傷害/TA or ALI/TA |
| #4 | 呼吸不全/TH or 呼吸不全/TA |
| #5 | 呼吸困難/TH or 呼吸困難/TA |
| #6 | 呼吸窮迫症候群-新生児/TH or 新生児呼吸窮迫症候群/TA |
| #7 | #1 or #2 or #3 or #4 or #5 or #6 |
| #8 | 非侵襲的補助換気/TH or 非侵襲的補助換気/TA or NPPV/TA or NIPPV/TA |
| #9 | 持続気道陽圧/TH or 持続気道陽圧/TA or CPAP/TA |
| #10 | 非侵襲的陽圧呼吸/TH or 非侵襲的陽圧呼吸/TA or BIPAP/TA |
| #11 | 酸素吸入療法/TH or 酸素吸入/TA |
| #12 | 酸素療法/TA or ハイフロー/TA or HFNC/TA or NHF/TA or HFO/TA |
| #13 | #8 or #9 or #10 or #11 or #12 |
| #14 | #7 and #13 |
| #15 | (#14) and (PT=会議録除く) |
| #16 | ランダム化比較試験/TH or ランダム化/AL or 無作為化/AL |
| #17 | 比較試験/AL |
| #18 | 臨床試験/TH or 臨床試験/AL |
| #19 | プラセボ/TH or プラセボ/AL |
| #20 | 対照/AL |
| #21 | コントロール/AL |
| #22 | 臨床研究・疫学研究/TH or 臨床研究/AL |
| #23 | #16 or #17 or #18 or #19 or #20 or #21 or #22 |
| #24 | #15 and #23 |
| #25 | ((非侵襲的補助換気/TH) and (SH=有害作用)) or 非侵襲的補助換気/TA or NPPV/TA or NIPPV/TA |
| #26 | ((持続気道陽圧/TH) and (SH=有害作用)) or 持続気道陽圧/TA or CPAP/TA |
| #27 | ((非侵襲的陽圧呼吸/TH) and (SH=有害作用)) or 非侵襲的陽圧呼吸/TA or BIPAP/TA |
| #28 | ((酸素吸入療法/TH) and (SH=有害作用)) or 酸素吸入/TA |
| #29 | 酸素療法/TA or ハイフロー/TA or HFNC/TA or NHF/TA or HFO/TA |
| #30 | #25 or #26 or #27 or #28 or #29 |
| #31 | 害/TA or 副作用/TA or 毒性/TA or 感染/TA or 肺炎/TA or 外傷/TA or トラウマ/TA or 気胸/TA or 潰瘍/TA or 不快感/TA or 損傷/TA or 気圧障害/TA or ((気圧障害/TH) and (SH=合併症)) or ((肺炎/TH) and (SH=合併症)) or ((褥瘡性潰瘍/TH) and (SH=合併症)) or ((気胸/TH) and (SH=合併症)) or 人工呼吸器関連肺炎/TH |
| #32 | #7 and #30 and #31 |
| #33 | (#32) and (PT=会議録除く) |
| #34 | #43 not #24 |

1. Flow diagram

Short-term mortality/Endotracheal intubation

**Identification**

21 Studies included in qualitative synthesis

6482 records after duplicates removed

7771 records identified through database searching

7771 records identified through database searching

Medline via PubMed (n=3403)

Cochrane CENTRAL (n=3924)

Igaku-Chuo-Zasshi (n=444)

0 additional records identified through other sources

19 Studies included in quantitative synthesis (meta-analysis)

Duplicates

n=1289

6374 records excluded

**Included**

**Eligibility**

**Screening**

87 Full-text articles excluded, with reasons:

・Wrong language (n=4)

・Wrong study design (n=50)

・Wrong population (n=27)

・Wrong intervention (n=4)

・Duplicates (n=1)

・Difficult to obtain full text (n=1)

Etc.

108 Full-text articles assessed for eligibility

Pneumonia

**Identification**

21 Studies included in qualitative synthesis

9104 records after duplicates removed

10401 records identified through database searching

2630 records identified through database searching

Medline via PubMed (n=1442)

Cochrane CENTRAL (n=0)

Igaku-Chuo-Zasshi (n=1188)

7771 additional records identified through other sources

10 Studies included in quantitative synthesis (meta-analysis)

Duplicates

n=1297

8976 records excluded

**Included**

**Eligibility**

**Screening**

107 Full-text articles excluded, with reasons:

・Wrong language (n=4)

・Wrong study design (n=26)

・Wrong population (n=42)

・Wrong intervention (n=13)

・Duplicates (n=1)

・Conference abstract (n=20)

・Difficult to obtain full text (n=1)

Etc.

128 Full-text articles assessed for eligibility

1. Risk of bias

NPPV vs COT

Short-term mortality Endotracheal intubation

Pneumonia

1. Forest plot

NPPV vs COT

Short-term mortality

Endotracheal intubation

Pneumonia

1. Evidence profile

Details of assessments of certainty of estimates from NMA

1. Short-term mortality

| **Comparison** | **NPPV vs. COT** | **HFNC vs. COT** | **IMV vs. COT** | **NPPV vs. IMV** | **HFNC vs. IMV** | **NPPV vs. HFNC** |  |
| --- | --- | --- | --- | --- | --- | --- | --- |
| **Direct evidence** | | | | | | | |
| Risk of bias | Not serious | Not serious |  | Not serious |  | Not serious |  |
| Inconsistency | Serious^a^ | Serious^a^ |  | Serious^a^ |  | Not serious |  |
| Indirectness | Not serious | Not serious |  | Serious^b^ |  | Not serious |  |
| Publication bias | Undetected | Undetected |  | Undetected |  | Undetected |  |
| Preliminary rating direct | **Moderate** | **Moderate** |  | **Low** |  | **High** |  |
| Contributes as much as indirect | No | Yes |  | Yes |  | Yes |  |
| Need to assess indirect | Yes | Yes |  | Yes |  | Yes |  |
| Imprecision | Not serious | Serious^c^ |  | Very serious^c^ |  | Very serious^c, d^ |  |
| Final direct rating | **Moderate** | **Low** |  | **Very low** |  | **Low** |  |
| **Indirect evidence** | | | | | | | |
| Common comparator | HFNC | NPPV | NPPV |  | NPPV | COT |  |
| Intervention 1 vs. common comparator rating | **High** | **High** | **Low** |  | **High** | **Moderate** |  |
| Intervention 2 vs. common comparator rating | **Moderate** | **Moderate** | **Moderate** |  | **Low** | **Moderate** |  |
| Lowest of the two | **Moderate** | **Moderate** | **Low** |  | **Low** | **Moderate** |  |
| Intransitivity | Not serious | Not serious | Not serious |  | Not serious | Not serious |  |
| Preliminary rating indirect | **Moderate** | **Moderate** | **Low** |  | **Low** | **Moderate** |  |
| Imprecision | Very serious^c^ | Not serious | Very serious^c^ |  | Very serious^c^ | Very serious^c^ |  |
| Final indirect rating | **Very low** | **Moderate** | **Very low** |  | **Very low** | **Very low** |  |
| **Network evidence** | | | | | | | |
| Highest between direct and indirect | **Moderate** | **Moderate** | **Low** | **Low** | **Low** | **High** |  |
| Incoherence | Not serious | Serious^e^ | NA | NA | NA | Serious^e^ |  |
| Imprecision | Not serious | Serious^c^ | Very serious^c^ | Very serious^c^ | Very serious^c^ | Very serious^c, d^ |  |
| Final network rating | **Moderate** | **Very low** | **Very low** | **Very low** | **Very low** | **Very low** |  |
| Most credible estimate | **Direct** | **Network** | **Network** | **Network** | **Network** | **Network** |  |

a: Due to high I^2^.

b: In the included two RCTs, lung protective ventilation was not performed.

c: Confidence intervals contained both substantial benefit and harm.

d: Meta-analysis using only one RCT.

e: Difference between confidence intervals of direct and indirect estimates.

COT, conventional oxygen therapy; HFNC, high flow nasal cannula; IMV, invasive mechanical ventilation; NA, not applicable; NPPV, noninvasive positive pressure ventilation; RCT, randomized controlled trial.

1. Endotracheal intubation

| **Comparison** | **NPPV vs. COT** | **HFNC vs. COT** | **NPPV vs. HFNC** |
| --- | --- | --- | --- |
| **Direct evidence** | | | |
| Risk of bias | Not serious | Not serious | Not serious |
| Inconsistency | Serious^a^ | Not serious | Not serious |
| Indirectness | Not serious | Not serious | Not serious |
| Publication bias | Undetected | Undetected | Undetected |
| Preliminary rating direct | **Moderate** | **High** | **High** |
| Contributes as much as indirect | No | Yes | Yes |
| Need to assess indirect | Yes | Yes | Yes |
| Imprecision | Not serious | Very serious^b^ | Very serious^c^ |
| Final direct rating | **Moderate** | **Low** | **Low** |
| **Indirect evidence** | | | |
| Common comparator | HFNC | NPPV | COT |
| Intervention 1 vs. common comparator rating | **High** | **High** | **Moderate** |
| Intervention 2 vs. common comparator rating | **High** | **Moderate** | **High** |
| Lowest of the two | **High** | **Moderate** | **Moderate** |
| Intransitivity | Not serious | Not serious | Not serious |
| Preliminary rating indirect | **High** | **Moderate** | **Moderate** |
| Imprecision | Very serious^b^ | Not serious | Serious^c^ |
| Final indirect rating | **Low** | **Moderate** | **Low** |
| **Network evidence** | | | |
| Highest between direct and indirect | **High** | **High** | **High** |
| Incoherence | Serious^c^ | Not serious | Serious^d^ |
| Imprecision | Not serious | Very serious^b^ | Very serious^b^ |
| Final network rating | **Moderate** | **Low** | **Very low** |
| Most credible estimate | **Direct** | **Network** | **Network** |

a: Due to high I^2^.

b: Confidence intervals contained both substantial benefit and harm.

c: Confidence intervals contained substantial benefit and no benefit.

d: Difference between confidence intervals of direct and indirect estimates.

COT, conventional oxygen therapy; HFNC, high flow nasal cannula; NPPV, noninvasive positive pressure ventilation.

1. Pneumonia

| **Comparison** | **NPPV vs. COT** | **HFNC vs. COT** | **IMV vs. COT** | **NPPV vs. IMV** | **HFNC vs. IMV** | **NPPV vs. HFNC** |  |
| --- | --- | --- | --- | --- | --- | --- | --- |
| **Direct evidence** | | | | | | | |
| Risk of bias | Serious^a^ | Serious^a^ |  | Serious^a^ |  | Serious^a^ |  |
| Inconsistency | Not serious | Not serious |  | Not serious |  | Not serious |  |
| Indirectness | Not serious | Not serious |  | Not serious |  | Not serious |  |
| Publication bias | Undetected | Undetected |  | Undetected |  | Undetected |  |
| Preliminary rating direct | **Moderate** | **Moderate** |  | **Moderate** |  | **Moderate** |  |
| Contributes as much as indirect | No | Yes |  | Yes |  | Yes |  |
| Need to assess indirect | Yes | Yes |  | Yes |  | Yes |  |
| Imprecision | Not serious | Very serious^b^ |  | Not serious |  | Very serious^b^ |  |
| Final direct rating | **Moderate** | **Very low** |  | **Moderate** |  | **Very low** |  |
| **Indirect evidence** | | | | | | | |
| Common comparator |  | NPPV | NPPV |  | NPPV | COT |  |
| Intervention 1 vs. common comparator rating |  | **Moderate** | **Moderate** |  | **Moderate** | **Moderate** |  |
| Intervention 2 vs. common comparator rating |  | **Moderate** | **Moderate** |  | **Moderate** | **Moderate** |  |
| Lowest of the two |  | **Moderate** | **Moderate** |  | **Moderate** | **Moderate** |  |
| Intransitivity |  | Not serious | Not serious |  | Not serious | Not serious |  |
| Preliminary rating indirect |  | **Moderate** | **Moderate** |  | **Moderate** | **Moderate** |  |
| Imprecision |  | Very serious^b^ | Not serious |  | Not serious | Very serious^b^ |  |
| Final indirect rating |  | **Very low** | **Moderate** |  | **Moderate** | **Very low** |  |
| **Network evidence** | | | | | | | |
| Highest between direct and indirect | **Moderate** | **Moderate** | **Moderate** | **Moderate** | **Moderate** | **Moderate** |  |
| Incoherence | NA | Not serious | NA | NA | NA | Serious^c^ |  |
| Imprecision | Not serious | Not serious | Not serious | Not serious | Not serious | Very serious^b^ |  |
| Final network rating | **Moderate** | **Moderate** | **Moderate** | **Moderate** | **Moderate** | **Very low** |  |
| Most credible estimate | **Network** | **Network** | **Network** | **Network** | **Network** | **Network** |  |

a: Intervention was not blinded, and definitions of pneumonia was unclear.

b: Confidence intervals contain both substantial benefit and harm.

c: Difference between confidence intervals of direct and indirect estimates.

COT, conventional oxygen therapy; HFNC, high flow nasal cannula; IMV, invasive mechanical ventilation; NA, not applicable; NPPV, noninvasive positive pressure ventilation; RCT, randomized controlled trial.

Results and certainty assessments for the outcome

1. Short-term mortality

| Comparison | Direct estimate (RR 95% CI) |  | Indirect estimate (RR 95% CI) | Rating | Network estimate (RR 95% CI) | Rating |
| --- | --- | --- | --- | --- | --- | --- |
| NPPV vs. COT | 0.71  (0.55−0.92) | ⨁⨁⨁◯ Moderate | 3.82  (0.58−25.05) | ⨁〇〇◯ Very low | 0.71 *  (0.55−0.92) | ⨁⨁⨁◯ Moderate |
| HFNC vs. COT | 0.79  (0.47−1.32) | ⨁⨁〇◯ Low | 0.13  (0.02−0.74) | ⨁⨁⨁◯ Moderate | 0.68  (0.41−1.12) | ⨁〇〇◯ Very low |
| IMV vs. COT | NA | - | 0.79  (0.40–1.57) | ⨁〇〇◯ Very low | 0.79  (0.40−1.57) | ⨁〇〇◯ Very low |
| NPPV vs. IMV | 0.92  (0.49−1.75) | ⨁〇〇◯ Very low | NA | - | 0.92  (0.40−1.75) | ⨁〇〇◯ Very low |
| HFNC vs. IMV | NA | - | 0.86  (0.37−1.97) | ⨁〇〇◯ Very low | 0.86  (0.37−1.97) | ⨁〇〇◯ Very low |
| NPPV vs. HFNC | 2.30  (0.97−5.44) | ⨁⨁〇◯ Low | 0.67  (0.34−1.32) | ⨁⨁〇◯ Low | 1.07  (0.63−1.83) | ⨁〇〇◯ Very low |

CI, confidence interval; COT, conventional oxygen therapy; HFNC, high flow nasal cannula; IMV, invasive mechanical ventilation; NA, not applicable; NPPV, noninvasive positive pressure ventilation; RR, risk ratio

* We adopted effect estimates based on direct evidence, because direct evidence was dominant for the network estimates.

2. Endotracheal intubation

| Comparison | Direct estimate (RR 95% CI) |  | Indirect estimate (RR 95% CI) | Rating | Network estimate (RR 95% CI) | Rating |
| --- | --- | --- | --- | --- | --- | --- |
| NPPV vs. COT | 0.66  (0.52−0.82) | ⨁⨁⨁◯ Moderate | 1.42  (0.32−6.33) | ⨁⨁〇◯ Low | 0.66 *  (0.52−0.82) | ⨁⨁⨁◯ Moderate |
| HFNC vs. COT | 0.88  (0.58−1.32) | ⨁⨁〇◯ Low | 0.25  (0.07−0.84) | ⨁⨁⨁◯ Moderate | 0.77  (0.52−1.32) | ⨁⨁〇◯ Low |
| NPPV vs. HFNC | 1.33  (0.71−2.48) | ⨁⨁〇◯ Low | 0.61  (0.35 −1.07) | ⨁⨁〇◯ Low | 0.86  (0.57−1.31) | ⨁〇〇◯ Very low |

CI, confidence interval; COT, conventional oxygen therapy; HFNC, high flow nasal cannula; NPPV, noninvasive positive pressure ventilation; RR, risk ratio.

* We adopted effect estimates based on direct evidence, because direct evidence was dominant for the network estimates.

1. Pneumonia

| Comparison | Direct estimate (RR 95% CI) |  | Indirect estimate (RR 95% CI) | Rating | Network estimate (RR 95% CI) | Rating |
| --- | --- | --- | --- | --- | --- | --- |
| NPPV vs. COT | 0.61  (0.40−0.92) | ⨁⨁⨁◯ Moderate | NA | - | 0.61  (0.40−0.92) | ⨁⨁⨁◯ Moderate |
| HFNC vs. COT | 0.44  (0.14−1.43) | ⨁〇〇◯ Very low | 0.07  (0.004−1.42) | ⨁〇〇◯ Very low | 0.35  (0.12−1.03) | ⨁⨁⨁◯ Moderate |
| IMV vs. COT | NA | - | 3.28  (1.37−7.88) | ⨁⨁⨁◯ Moderate | 3.28  (1.37−7.88) | ⨁⨁⨁◯ Moderate |
| NPPV vs. IMV | 0.19  (0.09−0.40) | ⨁⨁⨁◯ Moderate | NA | - | 0.19  (0.09−0.40) | ⨁⨁⨁◯ Moderate |
| HFNC vs. IMV | NA | - | 0.11  (0.03−0.40) | ⨁⨁⨁◯ Moderate | 0.11  (0.03−0.40) | ⨁⨁⨁◯ Moderate |
| NPPV vs. HFNC | 2.17  (0.69−6.85) | ⨁〇〇◯ Very low | 0.31  (0.01−7.94) | ⨁〇〇◯ Very low | 1.75  (0.59−5.16) | ⨁〇〇◯ Very low |

CI, confidence interval; COT, conventional oxygen therapy; HFNC, high flow nasal cannula; IMV, invasive mechanical ventilation; NA, not applicable; NPPV, noninvasive positive pressure ventilation; RR, risk ratio.

1. Evidence-to-Decision table

| Question | |
| --- | --- |
| **CQ15: Should NPPV be used over oxygen therapy for patients with ARDS?** | |
| **Population:** | Adult patients aged ≥18 years who had acute hypoxemic respiratory failure defined by new-onset of clinical signs (e.g., tachypnea, increased work of breathing), radiologic signs (unilateral or bilateral chest radiograph opacities), and hypoxemia.  We excluded the randomized controlled trials (RCTs) that included more than half of patients with hypercapnia, congestive heart failure, chronic obstructive pulmonary disease (COPD), or asthma as the cause of respiratory failure, post-extubation respiratory failure, post-surgical, and post-trauma constituting. |
| **Intervention** | Noninvasive positive pressure ventilation (NPPV) |
| **Comparison:** | Conventional oxygen therapy (COT) |
| **Main outcomes:** | Short-term mortality, Endotracheal intubation, Pneumonia |
| **Settings:** | Emergency department or intensive care unit |
| **Perspective:** | Individual |
| **Background:** | NPPV is used to avoid complications from tracheal intubation in patients with acute hypoxemic respiratory failure, but delayed intubation may increase the risk of death. Although NPPV is effective for cardiogenic pulmonary edema and acute exacerbations of COPD, NPPV is not established to manage patients with ARDS. It is an important clinical issue to clarify whether NPPV is more effective than COT. |
| **Conflict of interests:** | None |

# Assessment

| ProblemIs the problem a priority? | | |
| --- | --- | --- |
| Judgment | Research evidence | Additional considerations |
| ○ No  ○ Probably no  ● Probably yes  ○ Yes  ○ Varies  ○ Do not know | The previous clinical practice guideline for the management of ARDS in Japan (1) suggested using NPPV for early respiratory management in adults with ARDS. Patients with ARDS are at high risk for NPPV failure. Treatment failure is associated with higher in-hospital mortality. Careful observation is needed, even though NPPV is performed for ARDS. NPPV is not established to manage patients with ARDS but rather cardiogenic pulmonary edema and acute exacerbations of COPD. Therefore, this issue should probably be given high priority. |  |
| Desirable effectsHow substantial are the desirable anticipated effects? | | |
| Judgment | Research evidence | Additional considerations |
| ○ Trivial  ○ Small  ● Moderate  ○ Large  ○ Varies  ○ Do not know | The search strategy identified 12,620 records, including 19 RCTs and 6 observational trials that were eligible for inclusion. Observational studies were excluded for meta-analyses due to serious concerns of a high risk of bias. We performed network meta-analyses using 19 RCTs (N=2,777).  Fourteen trials (N=1,495) were included that compared NPPV and COT. We used direct evidence for an estimated value of the effects because the direct estimate was dominant for the network estimate of short-term mortality and endotracheal intubation.  In comparing NPPV and COT, the estimated value of the effects of short-term mortality (14 RCTs: N=1,494) was 107 fewer per 1,000 (95% CI: 166 fewer to 31 fewer), and endotracheal intubation (14 RCTs: N=1,495) was 135 fewer per 1,000 (95% CI: 188 fewer to 69 fewer). The estimated value of the effects of pneumonia (8 RCTs: N=804) was a reduction of 49 per 1,000 (95% CI: 75 fewer to 10 fewer).  Based on the above, the desirable effects were considered “moderate.” |  |
| Undesirable effectsHow substantial are the undesirable anticipated effects? | | |
| Judgment | Research evidence | Additional considerations |
| ○ Large  ○ Moderate  ○ Small  ● Trivial  ○ Varies  ○ Do not know | NPPV cannot ensure the protection of the airways. We adopted pneumonia as a critical outcome of harm for this systematic review. We did not identify any significant undesirable effects using NPPV.  Based on these, the undesirable effects of NPPV were considered “trivial.” | Two RCTs reported the incidence of skin damage which was an important outcome of harm (2, 3). The incidence of skin damage was 3.2-25.5% in patients with NPPV, although it did not occur in patients with COT. However, both RCTs had small sample sizes. |
| Certainty of evidenceWhat is the overall certainty of the evidence of effects? | | |
| Judgment | Research evidence | Additional considerations |
| ○ Very low  ○ Low  ● Moderate  ○ High  ○ No included studies | **Importance and value of the main outcomes**   \| Outcome \| Importance \| Certainty of the evidence \| \| --- \| --- \| --- \| \| Short-term mortality \| Critical \| ⨁⨁⨁◯ \| \| Moderate \| \| Endotracheal intubation \| Critical \| ⨁⨁⨁◯ \| \| Moderate \| \| Pneumonia \| Critical \| ⨁⨁⨁◯ \| \| Moderate \|   **Overall certainty of the evidence**  Both the certainty of the evidence and the direction of point estimates were consistent among all outcomes. The certainty of the evidence was considered to be “moderate.” |  |
| ValuesIs there important uncertainty about or variability in how much people value the main outcomes? | | |
| Judgment | Research evidence | Additional considerations |
| ○ Important uncertainty or variability  ○ Possibly important uncertainty or variability  ● Probably no important uncertainty or variability  ○ No important uncertainty or variability | We found no data on patient and family values in this systematic review. In general, the values of main outcomes were expected to be high and consistent. |  |
| Balance of effectsDoes the balance between desirable and undesirable effects favor the intervention or the comparison? | | |
| Judgment | Research evidence | Additional considerations |
| ○ Favors the comparison  ○ Probably favors the comparison  ○ Does not favor either the intervention or the comparison  ● Probably favors the intervention  ○ Favors the intervention  ○ Varies  ○ Do not know | **Summary of evidence:**   \| Outcome \| COT (comparison)^*^ \| NPPV (intervention) ^*^ \| Risk difference (RD)  (95% CI) \| Risk ratio (RR) (95% CI) \| \| --- \| --- \| --- \| --- \| --- \| \| Short-term mortality \| 227 / 736  (30.8%) \| 177 / 758  (23.4%) \| 107 fewer/1,000 (166 fewer-31 fewer) \| 0.71 (0.55-0.92) \| \| Endotracheal intubation \| 299 / 736  (40.6%) \| 224 / 759  (29.5%) \| 135 fewer /1,000  (188 fewer-69 fewer) \| 0.66 (0.52-0.82) \| \| Pneumonia \| 52 / 392  (13.3%) \| 33 / 412  (8.0%) \| 49 fewer /1,000 (75 fewer-10 fewer) \| 0.61 (0.40-0.92) \|   * using direct comparison  Based on the above, the balance of effects of NPPV was considered to be “probably favors the intervention.” |  |
| AcceptabilityIs the intervention acceptable to key stakeholders? | | |
| Judgment | Research evidence | Additional considerations |
| ○ No  ○ Probably no  ● Probably yes  ○ Yes  ○ Varies  ○ Do not know | Acceptability was considered to be “probably yes” because NPPV has already been performed to manage patients with respiratory failure. Furthermore, undesirable effects are rare using NPPV. Although NPPV is associated with more cost than COT, the potential benefit of avoiding endotracheal intubation may reduce the total cost. |  |
| FeasibilityIs the intervention feasible to implement? | | |
| Judgment | Research evidence | Additional considerations |
| ○ No  ○ Probably no  ● Probably yes  ○ Yes  ○ Varies  ○ Do not know | Feasibility was considered to be “probably yes” because NPPV has already been performed to manage patients with respiratory failure.  NPPV ventilators are expensive. Compared with COT, a higher intensity of nursing care is needed. |  |

# Summary of Evidence

|  | **JUDGMENT** | | | | | | |
| --- | --- | --- | --- | --- | --- | --- | --- |
| **PROBLEM** | No | Probably no | **Probably yes** | Yes |  | Varies | Unknown |
| **DESIRABLE EFFECTS** | Trivial | Small | **Moderate** | Large |  | Varies | Unknown |
| **UNDESIRABLE EFFECTS** | Large | Moderate | Small | **Trivial** |  | Varies | Unknown |
| **CERTAINTY OF EVIDENCE** | Very low | Low | **Moderate** | High |  |  | No included studies |
| **VALUES** | Important uncertainty or variability | Possibly important uncertainty or variability | **Probably no important uncertainty or variability** | No important uncertainty or variability |  |  |  |
| **BALANCE OF EFFECTS** | Favors the comparison | Probably favors the comparison | Does not favor either the intervention or the comparison | **Probably favors the intervention** | Favors the intervention | Varies | Unknown |
| **ACCEPTABILITY** | No | Probably no | **Probably yes** | Yes |  | Varies | Unknown |
| **FEASIBILITY** | No | Probably no | **Probably yes** | Yes |  | Varies | Unknown |

# Type of Recommendation

| Strong recommendation against the intervention | Conditional recommendation against the intervention | Conditional recommendation for either the intervention or the comparison | Conditional recommendation for the intervention | Strong recommendation for the intervention |
| --- | --- | --- | --- | --- |
| ○ | ○ | ○ | ● | ○ |

Conclusion

| Recommendation |
| --- |
| **If the patient has no contraindications for NPPV and no organ failure other than respiratory failure, we suggest using NPPV for adult patients with acute hypoxemic respiratory failure who have probable ARDS compared with COT (Conditional recommendation, moderate certainty of evidence: GRADE 2B).**  **Supplementary condition:**  **Careful observation is needed after initiation of NPPV to avoid delayed intubation which may contribute to poor outcomes.**  **Contraindications for NPPV include the inability to protect the airway, high risk of vomiting, deterioration of consciousness, agitation, and unstable hemodynamics.** |
| Justification |
| **Question:** Should NPPV be used over oxygen therapy for patients with ARDS?  **Population:** Adult patients with acute hypoxemic respiratory failure  **Intervention:** NPPV  **Main outcomes:** Short-term mortality, Endotracheal intubation, Pneumonia  **Summary of evidence:**  We performed network meta-analyses using 19 RCTs (N=2,777). Fourteen trials (N=1,495) were included to compare NPPV and COT. We used direct evidence for the estimated value of the effects because the direct estimate was dominant for the network estimate of short-term mortality and endotracheal intubation.  In comparing NPPV and COT, the estimated value of the effects of short-term mortality (14 RCTs: N=1494) was 107 fewer per 1,000 (95% CI: 166 fewer to 31 fewer), and endotracheal intubation (14 RCTs: N=1495) was 135 fewer per 1,000 (95% CI: 188 fewer to 69 fewer). The estimated value of the effects of pneumonia (8 RCTs: N=804) was a reduction of 49 per 1,000 (95% CI: 75 fewer to 10 fewer). Therefore, the desirable effects were considered to be “moderate”. Two RCTs reported the incidence of skin damage which was an important outcome of harm. The incidence of skin damage was 3.2-25.5% in patients with NPPV, although it did not occur in patients with COT. We judged that the undesirable effects of NPPV were “trivial.”  **Certainty of evidence:**  Both the certainty of the evidence and the direction of point estimates were consistent among all outcomes. The certainty of the evidence was considered to be “moderate.”  **Values, Balance of effects, Acceptability, Feasibility:**  In general, the values of the main outcomes were expected to be high and consistent. The balance of effects of NPPV was considered to be “probably favors the intervention.” Compared with COT, a higher intensity of nursing care is needed. However, acceptability and feasibility were considered to be “probably yes” because NPPV has already been performed to manage patients with respiratory failure.  **Panel meeting:**  The modified Delphi method was used to form a consensus. Prior to the discussion conference, we sent recommendation drafts and materials prepared by the systematic reviewers to all panelists. As a result of voting, the median value of agreement was 8.0, and disagreement index was 0.13 for the use of NPPV in adult patients with ARDS compared with COT.  In the discussion at the panel meeting, it was suggested to change the target population from ARDS to acute hypoxemic respiratory failure because we cannot diagnose ARDS without positive end-expiratory pressure based on the Berlin definition.  Some panelists suggested a strong recommendation considering the balance of effects and certainty of evidence. However, there was some concern that the effects may depend on the experience of staffs in facilities.  After extensive discussion among the panelists, the current recommendation, “We suggest using NPPV for adult patients with acute hypoxemic respiratory failure who have probable ARDS compared with COT.” was approved (median value of agreement was 8.0, and disagreement index was 0.13). |
| Subgroup considerations |
| A systematic review and network meta-analysis dividing NPPV into a helmet and face mask demonstrated that non-invasive respiratory supports\, including NPPV and HFNC, were superior to COT and that helmet NPPV was the most effective in reducing the risk of short-term mortality and endotracheal intubation^(4)^. However, continuous positive airway pressure (CPAP) was used as a non-invasive ventilation mode along with helmet non-invasive ventilation in most RCTs included in this network meta-analysis. PSV may increase the tidal volume, and a larger tidal volume is a risk for NPPV failure^(5)^. We performed a sensitivity analysis to evaluate the effect of NPPV mode (CPAP and PSV).  Compared with COT, CPAP was significantly associated with a lower risk of mortality (5 RCTs: N=352; RD: 171 fewer per 1,000; 95% CI: 254 fewer to 27 fewer), endotracheal intubation (5 RCTs: N=352; RD: 198 fewer per 1,000; 95% CI: 266 fewer to 94 fewer), and pneumonia (2 RCTs: N=163; RD: 51 fewer per 1,000; 95% CI: 91 fewer to 33 fewer).  Compared with COT, PSV was significantly associated with a lower risk of mortality (9 RCTs: N=1,142; RD: 76 fewer per 1,000; 95% CI: 149 fewer to 22 more), endotracheal intubation (12 RCTs: N=1,207; RD: 99 fewer per 1,000; 95% CI: 185 fewer to 16 fewer), and pneumonia (6 RCTs: N=641; RD: 45 fewer per 1,000; 95% CI: 80 fewer to 18 more). The probability of being best in the main outcomes was higher for CPAP than for PSV. |
| Implementation considerations |
| The previous clinical practice guideline for the management of ARDS in Japan (1) suggested using NPPV for early respiratory management in adults with ARDS. The Japanese Clinical Practice Guidelines for Management of Sepsis and Septic Shock 2020 (6) suggested conducting NPPV and HFNC for early respiratory failure in adult patients with sepsis. There was no recommendation for or against using NPPV in patients with ARDS in international guidelines^(7-9)^. If NIV is used for patients with ARDS, close monitoring, including tidal volumes, was suggested.  When NPPV is performed in patients with ARDS who are at a high risk for treatment failure, we should monitor respiratory and circulatory status to avoid delayed intubation.  The heart rate, acidosis, consciousness, oxygenation, and respiratory rate (HACOR) scale, which includes vital signs and data from blood gas analysis, may be an effective way of predicting NPPV failure in hypoxemic patients. In an observational study, the HACOR scale at 1 h from initiation of NPPV showed good predictive power for NIV failure compared with later assessments^(10)^. The first assessment should be performed at least 1 h later, and repeatable assessments are needed. |
| Monitoring and evaluation |
| After the implementation of the recommendations, further evaluation is needed, especially on the clinical effects and cost-effectiveness at each institution. In addition, it is desirable to monitor the implementation situation after the publication of the guideline to prevent other clinical problems. |
| Research priorities |
| We included only one RCT that compared NPPV with HFNC, in which NPPV was performed with a face mask, PSV mode, and ventilator in an intensive care unit. Furthermore, NPPV duration was only about 8 h per day. The optimal management of NPPV in patients with ARDS, including interface, mode, setting, and management during the interval, is unknown.  In an observational study evaluating NPPV in patients with acute hypoxemic respiratory failure, although excessive tidal volume and higher HACOR score were associated with NPPV failure within 24 h, improvement in Δ esophageal pressure at 2 h was the strongest association with treatment failure^(11)^. In patients with NPPV, monitoring of spontaneous breathing, including esophageal pressure, is an important issue for future research. If spontaneous breathing is too strong, endotracheal intubation should be considered to avoid patient self-inflicted lung injury. |

References

1. Hashimoto S, Sanui M, Egi M, Ohshimo S, Shiotsuka J, Seo R, et al. The clinical practice guideline for the management of ARDS in Japan. J Intensive Care. 2017;5:50. PMID: 2877009.

2. Delclaux C, L’Her E, Alberti C, Mancebo J, Abroug F, Conti G, et al. Treatment of acute hypoxemic nonhypercapnic respiratory insufficiency with continuous positive airway pressure delivered by a face mask: A randomized controlled trial. JAMA. 2000;284(18):2352-60. PMID: 11066186

3. Ferrer M, Esquinas A, Leon M, Gonzalez G, Alarcon A, Torres A. Non-invasive ventilation in severe hypoxemic respiratory failure: a randomized clinical trial. Am J Respir Crit Care Med. 2003;168(12):1438-44. PMID: 14500259

4. Ferreyro BL, Angriman F, Munshi L, Del Sorbo L, Ferguson ND, Rochwerg B, et al. Association of noninvasive oxygenation strategies with all-cause mortality in adults with acute hypoxemic respiratory failure: a systematic review and meta-analysis. JAMA. 2020;324(1):57-67. PMID: 32496521

5. Carteaux G, Millán-Guilarte T, De Prost N, Razazi K, Abid S, Thille AW, et al. Failure of non-invasive ventilation for de novo acute hypoxemic respiratory failure: role of tidal volume. Crit Care Med. 2016;44(2):282-90. PMID: 26584191

6. Egi M, Ogura H, Yatabe T, Atagi K, Inoue S, Iba T, et al. The Japanese clinical practice guidelines for management of sepsis and septic shock 2020 (J-SSCG 2020). J Intensive Care. 2021;9(1):53. PMID: 34433491

7. Rhodes A, Evans LE, Alhazzani W, Levy MM, Antonelli M, Ferrer R, et al. Surviving sepsis campaign: international guidelines for management of sepsis and septic shock: 2016. Intensive Care Med. 2017;43(3):304-77. PMID: 28101605

8. Rhodes A, Evans LE, Alhazzani W, Levy MM, Antonelli M, Ferrer R, et al. Surviving sepsis campaign: international guidelines for management of sepsis and septic shock: 2016. Crit Care Med. 2017;45(3):486-552. PMID: 28098591

9. Rochwerg B, Brochard L, Elliott MW, Hess D, Hill NS, Nava S, et al. Official ERS/ATS clinical practice guidelines: non-invasive ventilation for acute respiratory failure. Eur Respir J. 2017;50(2):1602426. PMID: 33201321

10. Duan J, Han X, Bai L, Zhou L, Huang S. Assessment of heart rate, acidosis, consciousness, oxygenation, and respiratory rate to predict non-invasive ventilation failure in hypoxemic patients. Intensive Care Med. 2017;43(2):192-9. PMID: 27812731

11. Tonelli R, Fantini R, Tabbì L, Castaniere I, Pisani L, Pellegrino MR, et al. Early inspiratory effort assessment by esophageal manometry predicts non-invasive ventilation outcome in de novo respiratory failure. A pilot study. Am J Respir Crit Care Med. 2020;202(4):558-67. PMID: 32325004

**CQ16 Should HFNC be used over conventional oxygen therapy for patients with ARDS?**

1.Search strategy

Short-term mortality/Endotracheal intubation

MEDLINE via PubMed (Search date: 2020/6/19)

| #1 | "Hypoxia"[mh] OR hypox*[tiab] OR "Respiratory Insufficiency"[mh] OR respiratory depression*[tiab] OR respiratory failure*[tiab] OR ventilatory depression*[tiab] OR respiratory insufficienc*[tiab] OR "Dyspnea"[mh] OR dyspnea*[tiab] OR "shortness of breath"[tiab] OR "Respiratory Distress Syndrome, Adult"[mh] OR acute respiratory distress[tiab] OR adult respiratory distress[tiab] OR respiratory distress syndrome*[tiab] OR RDS[tiab] OR ARDS[tiab] OR "Acute Lung Injury"[mh] OR acute lung injur*[tiab] OR ALI[tiab] |
| --- | --- |
| #2 | "Respiratory Distress Syndrome, Newborn"[mh] |
| #3 | #1 or #2 |
| #4 | "Noninvasive Ventilation"[mh] OR noninvasive ventilation*[tiab] OR non invasive ventilation*[tiab] OR NIV[tiab] OR NPPV[tiab] OR NIPPV[tiab] OR noninvasive positive pressure ventilation*[tiab] OR noninvasive mechanical ventilation*[tiab] OR noninvasive pressure support ventilation*[tiab] OR "Continuous Positive Airway Pressure"[mh] OR continuous positive airway pressure*[tiab] OR bilevel positive airway pressure*[tiab] OR biphasic positive airway pressure*[tiab] OR BIPAP[tiab] |
| #5 | "Oxygen Inhalation Therapy"[mh] OR HFNC[tiab] OR HHFNC[tiab] OR HHHFNC[tiab] OR HFNO[tiab] OR HFNT[tiab] OR HFNOT[tiab] OR HFO[tiab] OR HFOT[tiab] OR NHF[tiab] OR NHFC[tiab] OR NHFT[tiab] OR NHFO[tiab] OR NHFOT[tiab] OR high flow therap*[tiab] OR high flow oxygen[tiab] OR nasal high flow[tiab] |
| #6 | #4 or #5 |
| #7 | ("Randomized Controlled Trial"[pt] OR "Controlled Clinical Trial"[pt] OR "Clinical Trials as Topic"[mh] OR randomized[tiab] OR placebo[tiab] OR randomly[tiab] OR trial[tiab] OR groups[tiab]) NOT (Animals [mh] NOT Humans [mh]) |
| #8 | #3 and #6 and #7 |

CENTRAL (Search date: 2020/6/22)

| #1 | [mh Hypoxia] OR hypox*:ti,ab OR [mh "Respiratory Insufficiency"] OR "respiratory depression":ti,ab OR "respiratory failure":ti,ab OR "ventilatory depression":ti,ab OR "respiratory insufficiency":ti,ab OR [mh Dyspnea] OR dyspnea:ti,ab OR "shortness of breath":ti,ab OR [mh "Respiratory Distress Syndrome, Adult"] OR "acute respiratory distress":ti,ab OR "adult respiratory distress":ti,ab OR "respiratory distress syndrome":ti,ab OR RDS:ti,ab OR ARDS:ti,ab OR [mh "Acute Lung Injury"] OR "acute lung injury":ti,ab OR ALI:ti,ab |
| --- | --- |
| #2 | [mh "Respiratory Distress Syndrome, Newborn"] |
| #3 | #1 OR #2 |
| #4 | [mh "Noninvasive Ventilation"] OR "noninvasive ventilation":ti,ab OR "non invasive ventilation":ti,ab OR NIV:ti,ab OR NPPV:ti,ab OR NIPPV:ti,ab OR "noninvasive positive pressure ventilation":ti,ab OR "noninvasive mechanical ventilation":ti,ab OR "noninvasive pressure support ventilation":ti,ab OR [mh "Continuous Positive Airway Pressure"] OR "continuous positive airway pressure":ti,ab OR "bilevel positive airway pressure":ti,ab OR "biphasic positive airway pressure":ti,ab OR BIPAP:ti,ab |
| #5 | [mh "Oxygen Inhalation Therapy"] OR HFNC:ti,ab OR HHFNC:ti,ab OR HHHFNC:ti,ab OR HFNO:ti,ab OR HFNT:ti,ab OR HFNOT:ti,ab OR HFO:ti,ab OR HFOT:ti,ab OR NHF:ti,ab OR NHFC:ti,ab OR NHFT:ti,ab OR NHFO:ti,ab OR NHFOT:ti,ab OR "high flow therapy":ti,ab OR "high flow oxygen":ti,ab OR "nasal high flow":ti,ab |
| #6 | #4 OR #5 |
| #7 | #3 AND #6 |

Igaku-Chuo-Zasshi (Search date: 2020/6/19)

| #1 | 酸素欠乏/TH or 酸素欠乏/TA or anoxia/TA or Hypoxia/TA |
| --- | --- |
| #2 | 呼吸窮迫症候群-急性/TH or 急性呼吸窮迫症候群/TA or ARDS/TA |
| #3 | 急性肺損傷/TH or 急性肺損傷/TA or 急性肺障害/TA or 急性肺傷害/TA or ALI/TA |
| #4 | 呼吸不全/TH or 呼吸不全/TA |
| #5 | 呼吸困難/TH or 呼吸困難/TA |
| #6 | 呼吸窮迫症候群-新生児/TH or 新生児呼吸窮迫症候群/TA |
| #7 | #1 or #2 or #3 or #4 or #5 or #6 |
| #8 | 非侵襲的補助換気/TH or 非侵襲的補助換気/TA or NPPV/TA or NIPPV/TA |
| #9 | 持続気道陽圧/TH or 持続気道陽圧/TA or CPAP/TA |
| #10 | 非侵襲的陽圧呼吸/TH or 非侵襲的陽圧呼吸/TA or BIPAP/TA |
| #11 | 酸素吸入療法/TH or 酸素吸入/TA |
| #12 | 酸素療法/TA or ハイフロー/TA or HFNC/TA or NHF/TA or HFO/TA |
| #13 | #8 or #9 or #10 or #11 or #12 |
| #14 | #7 and #13 |
| #15 | (#14) and (PT=会議録除く) |
| #16 | ランダム化比較試験/TH or ランダム化/AL or 無作為化/AL |
| #17 | 比較試験/AL |
| #18 | 臨床試験/TH or 臨床試験/AL |
| #19 | プラセボ/TH or プラセボ/AL |
| #20 | 対照/AL |
| #21 | コントロール/AL |
| #22 | 臨床研究・疫学研究/TH or 臨床研究/AL |
| #23 | #16 or #17 or #18 or #19 or #20 or #21 or #22 |
| #24 | #15 and #23 |

Pneumonia

MEDLINE via PubMed（Search date: 2020/6/19）

|  | 検索式 |
| --- | --- |
| #1 | "Hypoxia"[mh] OR hypox*[tiab] OR "Respiratory Insufficiency"[mh] OR respiratory depression*[tiab] OR respiratory failure*[tiab] OR ventilatory depression*[tiab] OR respiratory insufficienc*[tiab] OR "Dyspnea"[mh] OR dyspnea*[tiab] OR "shortness of breath"[tiab] OR "Respiratory Distress Syndrome, Adult"[mh] OR acute respiratory distress[tiab] OR adult respiratory distress[tiab] OR respiratory distress syndrome*[tiab] OR RDS[tiab] OR ARDS[tiab] OR "Acute Lung Injury"[mh] OR acute lung injur*[tiab] OR ALI[tiab] |
| #2 | "Respiratory Distress Syndrome, Newborn"[mh] |
| #3 | #1 or #2 |
| #4 | "Noninvasive Ventilation"[mh] OR noninvasive ventilation*[tiab] OR non invasive ventilation*[tiab] OR NIV[tiab] OR NPPV[tiab] OR NIPPV[tiab] OR noninvasive positive pressure ventilation*[tiab] OR noninvasive mechanical ventilation*[tiab] OR noninvasive pressure support ventilation*[tiab] OR "Continuous Positive Airway Pressure"[mh] OR continuous positive airway pressure*[tiab] OR bilevel positive airway pressure*[tiab] OR biphasic positive airway pressure*[tiab] OR BIPAP[tiab] |
| #5 | "Oxygen Inhalation Therapy"[mh] OR HFNC[tiab] OR HHFNC[tiab] OR HHHFNC[tiab] OR HFNO[tiab] OR HFNT[tiab] OR HFNOT[tiab] OR HFO[tiab] OR HFOT[tiab] OR NHF[tiab] OR NHFC[tiab] OR NHFT[tiab] OR NHFO[tiab] OR NHFOT[tiab] OR high flow therap*[tiab] OR high flow oxygen[tiab] OR nasal high flow[tiab] |
| #6 | #4 or #5 |
| #7 | ("Randomized Controlled Trial"[pt] OR "Controlled Clinical Trial"[pt] OR "Clinical Trials as Topic"[mh] OR randomized[tiab] OR placebo[tiab] OR randomly[tiab] OR trial[tiab] OR groups[tiab]) NOT (Animals [mh] NOT Humans [mh]) |
| #8 | #3 and #6 and #7 |
| #9 | "Noninvasive Ventilation/adverse effects"[mh] OR noninvasive ventilation*[tiab] OR non invasive ventilation*[tiab] OR NIV[tiab] OR NPPV[tiab] OR NIPPV[tiab] OR noninvasive positive pressure ventilation*[tiab] OR noninvasive mechanical ventilation*[tiab] OR noninvasive pressure support ventilation*[tiab] OR "Continuous Positive Airway Pressure/adverse effects"[mh] OR continuous positive airway pressure*[tiab] OR bilevel positive airway pressure*[tiab] OR biphasic positive airway pressure*[tiab] OR BIPAP[tiab] |
| #10 | "Oxygen Inhalation Therapy/adverse effects"[mh] OR HFNC[tiab] OR HHFNC[tiab] OR HHHFNC[tiab] OR HFNO[tiab] OR HFNT[tiab] OR HFNOT[tiab] OR HFO[tiab] OR HFOT[tiab] OR NHF[tiab] OR NHFC[tiab] OR NHFT[tiab] OR NHFO[tiab] OR NHFOT[tiab] OR high flow therap*[tiab] OR high flow oxygen[tiab] OR nasal high flow[tiab] |
| #11 | #9 or #10 |
| #12 | harm*[tiab] OR side effect*[tiab] OR adverse[tiab] OR toxicity[tiab] OR infection*[tiab] OR pneumonia*[tiab] OR trauma[tiab] OR barotrauma*[tiab] OR volutrauma*[tiab] OR pneumothorax[tiab] OR ulcer*[tiab] OR discomfort*[tiab] OR breakdown[tiab] OR damage[tiab] OR intoleran*[tiab] OR "Barotrauma/complications"[mh] OR "Pneumonia/complications"[mh] OR "Pressure Ulcer/complications"[mh] OR "Pneumothorax/complications"[mh] OR "Pneumonia, Ventilator-Associated"[mh] |
| #13 | #3 and #11 and #12 |
| #14 | Animals [mh] NOT Humans [mh] |
| #15 | #13 not #14 |
| #16 | #15 not #8 |

CENTRAL（Search date: 2020/6/22）

|  | 検索式 |
| --- | --- |
| #1 | [mh Hypoxia] OR hypox*:ti,ab OR [mh "Respiratory Insufficiency"] OR "respiratory depression":ti,ab OR "respiratory failure":ti,ab OR "ventilatory depression":ti,ab OR "respiratory insufficiency":ti,ab OR [mh Dyspnea] OR dyspnea:ti,ab OR "shortness of breath":ti,ab OR [mh "Respiratory Distress Syndrome, Adult"] OR "acute respiratory distress":ti,ab OR "adult respiratory distress":ti,ab OR "respiratory distress syndrome":ti,ab OR RDS:ti,ab OR ARDS:ti,ab OR [mh "Acute Lung Injury"] OR "acute lung injury":ti,ab OR ALI:ti,ab |
| #2 | [mh "Respiratory Distress Syndrome, Newborn"] |
| #3 | #1 OR #2 |
| #4 | [mh "Noninvasive Ventilation"] OR "noninvasive ventilation":ti,ab OR "non invasive ventilation":ti,ab OR NIV:ti,ab OR NPPV:ti,ab OR NIPPV:ti,ab OR "noninvasive positive pressure ventilation":ti,ab OR "noninvasive mechanical ventilation":ti,ab OR "noninvasive pressure support ventilation":ti,ab OR [mh "Continuous Positive Airway Pressure"] OR "continuous positive airway pressure":ti,ab OR "bilevel positive airway pressure":ti,ab OR "biphasic positive airway pressure":ti,ab OR BIPAP:ti,ab |
| #5 | [mh "Oxygen Inhalation Therapy"] OR HFNC:ti,ab OR HHFNC:ti,ab OR HHHFNC:ti,ab OR HFNO:ti,ab OR HFNT:ti,ab OR HFNOT:ti,ab OR HFO:ti,ab OR HFOT:ti,ab OR NHF:ti,ab OR NHFC:ti,ab OR NHFT:ti,ab OR NHFO:ti,ab OR NHFOT:ti,ab OR "high flow therapy":ti,ab OR "high flow oxygen":ti,ab OR "nasal high flow":ti,ab |
| #6 | #4 OR #5 |
| #7 | #3 AND #6 |
| #8 | [mh "Noninvasive Ventilation"/ae] OR "noninvasive ventilation":ti,ab OR "non invasive ventilation":ti,ab OR NIV:ti,ab OR NPPV:ti,ab OR NIPPV:ti,ab OR "noninvasive positive pressure ventilation":ti,ab OR "noninvasive mechanical ventilation":ti,ab OR "noninvasive pressure support ventilation":ti,ab OR [mh "Continuous Positive Airway Pressure"/ae] OR "continuous positive airway pressure":ti,ab OR "bilevel positive airway pressure":ti,ab OR "biphasic positive airway pressure":ti,ab OR BIPAP:ti,ab |
| #9 | [mh "Oxygen Inhalation Therapy"/ae] OR HFNC:ti,ab OR HHFNC:ti,ab OR HHHFNC:ti,ab OR HFNO:ti,ab OR HFNT:ti,ab OR HFNOT:ti,ab OR HFO:ti,ab OR HFOT:ti,ab OR NHF:ti,ab OR NHFC:ti,ab OR NHFT:ti,ab OR NHFO:ti,ab OR NHFOT:ti,ab OR "high flow therapy":ti,ab OR "high flow oxygen":ti,ab OR "nasal high flow":ti,ab |
| #10 | #8 OR #9 |
| #11 | harm*:ti,ab OR "side effect":ti,ab OR adverse:ti,ab OR toxicity:ti,ab OR infection*:ti,ab OR pneumonia*:ti,ab OR trauma:ti,ab OR barotrauma*:ti,ab OR volutrauma*:ti,ab OR pneumothorax:ti,ab OR ulcer*:ti,ab OR discomfort*:ti,ab OR breakdown:ti,ab OR damage:ti,ab OR intoleran*:ti,ab OR [mh Barotrauma/co] OR [mh Pneumonia/co] OR [mh "Pressure Ulcer"/co] OR [mh Pneumothorax/co] OR [mh "Pneumonia, Ventilator-Associated"] |
| #12 | #3 AND #10 AND #11 |
| #13 | #12 NOT #7 |

Igaku-Chuo-Zasshi （Search date: 2020/6/19）

|  | 検索式 |
| --- | --- |
| #1 | 酸素欠乏/TH or 酸素欠乏/TA or anoxia/TA or Hypoxia/TA |
| #2 | 呼吸窮迫症候群-急性/TH or 急性呼吸窮迫症候群/TA or ARDS/TA |
| #3 | 急性肺損傷/TH or 急性肺損傷/TA or 急性肺障害/TA or 急性肺傷害/TA or ALI/TA |
| #4 | 呼吸不全/TH or 呼吸不全/TA |
| #5 | 呼吸困難/TH or 呼吸困難/TA |
| #6 | 呼吸窮迫症候群-新生児/TH or 新生児呼吸窮迫症候群/TA |
| #7 | #1 or #2 or #3 or #4 or #5 or #6 |
| #8 | 非侵襲的補助換気/TH or 非侵襲的補助換気/TA or NPPV/TA or NIPPV/TA |
| #9 | 持続気道陽圧/TH or 持続気道陽圧/TA or CPAP/TA |
| #10 | 非侵襲的陽圧呼吸/TH or 非侵襲的陽圧呼吸/TA or BIPAP/TA |
| #11 | 酸素吸入療法/TH or 酸素吸入/TA |
| #12 | 酸素療法/TA or ハイフロー/TA or HFNC/TA or NHF/TA or HFO/TA |
| #13 | #8 or #9 or #10 or #11 or #12 |
| #14 | #7 and #13 |
| #15 | (#14) and (PT=会議録除く) |
| #16 | ランダム化比較試験/TH or ランダム化/AL or 無作為化/AL |
| #17 | 比較試験/AL |
| #18 | 臨床試験/TH or 臨床試験/AL |
| #19 | プラセボ/TH or プラセボ/AL |
| #20 | 対照/AL |
| #21 | コントロール/AL |
| #22 | 臨床研究・疫学研究/TH or 臨床研究/AL |
| #23 | #16 or #17 or #18 or #19 or #20 or #21 or #22 |
| #24 | #15 and #23 |
| #25 | ((非侵襲的補助換気/TH) and (SH=有害作用)) or 非侵襲的補助換気/TA or NPPV/TA or NIPPV/TA |
| #26 | ((持続気道陽圧/TH) and (SH=有害作用)) or 持続気道陽圧/TA or CPAP/TA |
| #27 | ((非侵襲的陽圧呼吸/TH) and (SH=有害作用)) or 非侵襲的陽圧呼吸/TA or BIPAP/TA |
| #28 | ((酸素吸入療法/TH) and (SH=有害作用)) or 酸素吸入/TA |
| #29 | 酸素療法/TA or ハイフロー/TA or HFNC/TA or NHF/TA or HFO/TA |
| #30 | #25 or #26 or #27 or #28 or #29 |
| #31 | 害/TA or 副作用/TA or 毒性/TA or 感染/TA or 肺炎/TA or 外傷/TA or トラウマ/TA or 気胸/TA or 潰瘍/TA or 不快感/TA or 損傷/TA or 気圧障害/TA or ((気圧障害/TH) and (SH=合併症)) or ((肺炎/TH) and (SH=合併症)) or ((褥瘡性潰瘍/TH) and (SH=合併症)) or ((気胸/TH) and (SH=合併症)) or 人工呼吸器関連肺炎/TH |
| #32 | #7 and #30 and #31 |
| #33 | (#32) and (PT=会議録除く) |
| #34 | #43 not #24 |

1. Flow diagram

Short-term mortality/Endotracheal intubation

**Identification**

21 Studies included in qualitative synthesis

6482 records after duplicates removed

7771 records identified through database searching

7771 records identified through database searching

Medline via PubMed (n=3403)

Cochrane CENTRAL (n=3924)

Igaku-Chuo-Zasshi (n=444)

0 additional records identified through other sources

19 Studies included in quantitative synthesis (meta-analysis)

Duplicates

n=1289

6374 records excluded

**Included**

**Eligibility**

**Screening**

87 Full-text articles excluded, with reasons:

・Wrong language (n=4)

・Wrong study design (n=50)

・Wrong population (n=27)

・Wrong intervention (n=4)

・Duplicates (n=1)

・Difficult to obtain full text (n=1)

Etc.

108 Full-text articles assessed for eligibility

Pneumonia

**Identification**

21 Studies included in qualitative synthesis

9104 records after duplicates removed

10401 records identified through database searching

2630 records identified through database searching

Medline via PubMed (n=1442)

Cochrane CENTRAL (n=0)

Igaku-Chuo-Zasshi (n=1188)

7771 additional records identified through other sources

10 Studies included in quantitative synthesis (meta-analysis)

Duplicates

n=1297

8976 records excluded

**Included**

**Eligibility**

**Screening**

107 Full-text articles excluded, with reasons:

・Wrong language (n=4)

・Wrong study design (n=26)

・Wrong population (n=42)

・Wrong intervention (n=13)

・Duplicates (n=1)

・Conference abstract (n=20)

・Difficult to obtain full text (n=1)

Etc.

128 Full-text articles assessed for eligibility

1. Risk of bias

HFNC vs COT

Short-term mortality Endotracheal intubation

Pneumonia

1. Forest plot

HFNC v COT

Short-term mortality

Endotracheal intubation

Pneumonia

1. Evidence profile

Details of assessments of certainty of estimates from NMA

1. Short-term mortality

| **Comparison** | **NPPV vs. COT** | **HFNC vs. COT** | **IMV vs. COT** | **NPPV vs. IMV** | **HFNC vs. IMV** | **NPPV vs. HFNC** |  |
| --- | --- | --- | --- | --- | --- | --- | --- |
| **Direct evidence** | | | | | | | |
| Risk of bias | Not serious | Not serious |  | Not serious |  | Not serious |  |
| Inconsistency | Serious^a^ | Serious^a^ |  | Serious^a^ |  | Not serious |  |
| Indirectness | Not serious | Not serious |  | Serious^b^ |  | Not serious |  |
| Publication bias | Undetected | Undetected |  | Undetected |  | Undetected |  |
| Preliminary rating direct | **Moderate** | **Moderate** |  | **Low** |  | **High** |  |
| Contributes as much as indirect | No | Yes |  | Yes |  | Yes |  |
| Need to assess indirect | Yes | Yes |  | Yes |  | Yes |  |
| Imprecision | Not serious | Serious^c^ |  | Very serious^c^ |  | Very serious^c, d^ |  |
| Final direct rating | **Moderate** | **Low** |  | **Very low** |  | **Low** |  |
| **Indirect evidence** | | | | | | | |
| Common comparator | HFNC | NPPV | NPPV |  | NPPV | COT |  |
| Intervention 1 vs. common comparator rating | **High** | **High** | **Low** |  | **High** | **Moderate** |  |
| Intervention 2 vs. common comparator rating | **Moderate** | **Moderate** | **Moderate** |  | **Low** | **Moderate** |  |
| Lowest of the two | **Moderate** | **Moderate** | **Low** |  | **Low** | **Moderate** |  |
| Intransitivity | Not serious | Not serious | Not serious |  | Not serious | Not serious |  |
| Preliminary rating indirect | **Moderate** | **Moderate** | **Low** |  | **Low** | **Moderate** |  |
| Imprecision | Very serious^c^ | Not serious | Very serious^c^ |  | Very serious^c^ | Very serious^c^ |  |
| Final indirect rating | **Very low** | **Moderate** | **Very low** |  | **Very low** | **Very low** |  |
| **Network evidence** | | | | | | | |
| Highest between direct and indirect | **Moderate** | **Moderate** | **Low** | **Low** | **Low** | **High** |  |
| Incoherence | Not serious | Serious^e^ | NA | NA | NA | Serious^e^ |  |
| Imprecision | Not serious | Serious^c^ | Very serious^c^ | Very serious^c^ | Very serious^c^ | Very serious^c, d^ |  |
| Final network rating | **Moderate** | **Very low** | **Very low** | **Very low** | **Very low** | **Very low** |  |
| Most credible estimate | **Direct** | **Network** | **Network** | **Network** | **Network** | **Network** |  |

a: Due to high I^2^.

b: In the included two RCTs, lung protective ventilation was not performed.

c: Confidence intervals contained both substantial benefit and harm.

d: Meta-analysis using only one RCT.

e: Difference between confidence intervals of direct and indirect estimates.

COT, conventional oxygen therapy; HFNC, high flow nasal cannula; IMV, invasive mechanical ventilation; NA, not applicable; NPPV, noninvasive positive pressure ventilation; RCT, randomized controlled trial.

1. Endotracheal intubation

| **Comparison** | **NPPV vs. COT** | **HFNC vs. COT** | **NPPV vs. HFNC** |
| --- | --- | --- | --- |
| **Direct evidence** | | | |
| Risk of bias | Not serious | Not serious | Not serious |
| Inconsistency | Serious^a^ | Not serious | Not serious |
| Indirectness | Not serious | Not serious | Not serious |
| Publication bias | Undetected | Undetected | Undetected |
| Preliminary rating direct | **Moderate** | **High** | **High** |
| Contributes as much as indirect | No | Yes | Yes |
| Need to assess indirect | Yes | Yes | Yes |
| Imprecision | Not serious | Very serious^b^ | Very serious^c^ |
| Final direct rating | **Moderate** | **Low** | **Low** |
| **Indirect evidence** | | | |
| Common comparator | HFNC | NPPV | COT |
| Intervention 1 vs. common comparator rating | **High** | **High** | **Moderate** |
| Intervention 2 vs. common comparator rating | **High** | **Moderate** | **High** |
| Lowest of the two | **High** | **Moderate** | **Moderate** |
| Intransitivity | Not serious | Not serious | Not serious |
| Preliminary rating indirect | **High** | **Moderate** | **Moderate** |
| Imprecision | Very serious^b^ | Not serious | Serious^c^ |
| Final indirect rating | **Low** | **Moderate** | **Low** |
| **Network evidence** | | | |
| Highest between direct and indirect | **High** | **High** | **High** |
| Incoherence | Serious^c^ | Not serious | Serious^d^ |
| Imprecision | Not serious | Very serious^b^ | Very serious^b^ |
| Final network rating | **Moderate** | **Low** | **Very low** |
| Most credible estimate | **Direct** | **Network** | **Network** |

a: Due to high I^2^.

b: Confidence intervals contained both substantial benefit and harm.

c: Confidence intervals contained substantial benefit and no benefit.

d: Difference between confidence intervals of direct and indirect estimates.

COT, conventional oxygen therapy; HFNC, high flow nasal cannula; NPPV, noninvasive positive pressure ventilation.

1. Pneumonia

| **Comparison** | **NPPV vs. COT** | **HFNC vs. COT** | **IMV vs. COT** | **NPPV vs. IMV** | **HFNC vs. IMV** | **NPPV vs. HFNC** |  |
| --- | --- | --- | --- | --- | --- | --- | --- |
| **Direct evidence** | | | | | | | |
| Risk of bias | Serious^a^ | Serious^a^ |  | Serious^a^ |  | Serious^a^ |  |
| Inconsistency | Not serious | Not serious |  | Not serious |  | Not serious |  |
| Indirectness | Not serious | Not serious |  | Not serious |  | Not serious |  |
| Publication bias | Undetected | Undetected |  | Undetected |  | Undetected |  |
| Preliminary rating direct | **Moderate** | **Moderate** |  | **Moderate** |  | **Moderate** |  |
| Contributes as much as indirect | No | Yes |  | Yes |  | Yes |  |
| Need to assess indirect | Yes | Yes |  | Yes |  | Yes |  |
| Imprecision | Not serious | Very serious^b^ |  | Not serious |  | Very serious^b^ |  |
| Final direct rating | **Moderate** | **Very low** |  | **Moderate** |  | **Very low** |  |
| **Indirect evidence** | | | | | | | |
| Common comparator |  | NPPV | NPPV |  | NPPV | COT |  |
| Intervention 1 vs. common comparator rating |  | **Moderate** | **Moderate** |  | **Moderate** | **Moderate** |  |
| Intervention 2 vs. common comparator rating |  | **Moderate** | **Moderate** |  | **Moderate** | **Moderate** |  |
| Lowest of the two |  | **Moderate** | **Moderate** |  | **Moderate** | **Moderate** |  |
| Intransitivity |  | Not serious | Not serious |  | Not serious | Not serious |  |
| Preliminary rating indirect |  | **Moderate** | **Moderate** |  | **Moderate** | **Moderate** |  |
| Imprecision |  | Very serious^b^ | Not serious |  | Not serious | Very serious^b^ |  |
| Final indirect rating |  | **Very low** | **Moderate** |  | **Moderate** | **Very low** |  |
| **Network evidence** | | | | | | | |
| Highest between direct and indirect | **Moderate** | **Moderate** | **Moderate** | **Moderate** | **Moderate** | **Moderate** |  |
| Incoherence | NA | Not serious | NA | NA | NA | Serious^c^ |  |
| Imprecision | Not serious | Not serious | Not serious | Not serious | Not serious | Very serious^b^ |  |
| Final network rating | **Moderate** | **Moderate** | **Moderate** | **Moderate** | **Moderate** | **Very low** |  |
| Most credible estimate | **Network** | **Network** | **Network** | **Network** | **Network** | **Network** |  |

a: Intervention was not blinded, and definitions of pneumonia was unclear.

b: Confidence intervals contain both substantial benefit and harm.

c: Difference between confidence intervals of direct and indirect estimates.

COT, conventional oxygen therapy; HFNC, high flow nasal cannula; IMV, invasive mechanical ventilation; NA, not applicable; NPPV, noninvasive positive pressure ventilation; RCT, randomized controlled trial.

Results and certainty assessments for the outcome

1. Short-term mortality

| Comparison | Direct estimate (RR 95% CI) |  | Indirect estimate (RR 95% CI) | Rating | Network estimate (RR 95% CI) | Rating |
| --- | --- | --- | --- | --- | --- | --- |
| NPPV vs. COT | 0.71  (0.55−0.92) | ⨁⨁⨁◯ Moderate | 3.82  (0.58−25.05) | ⨁〇〇◯ Very low | 0.71 *  (0.55−0.92) | ⨁⨁⨁◯ Moderate |
| HFNC vs. COT | 0.79  (0.47−1.32) | ⨁⨁〇◯ Low | 0.13  (0.02−0.74) | ⨁⨁⨁◯ Moderate | 0.68  (0.41−1.12) | ⨁〇〇◯ Very low |
| IMV vs. COT | NA | - | 0.79  (0.40–1.57) | ⨁〇〇◯ Very low | 0.79  (0.40−1.57) | ⨁〇〇◯ Very low |
| NPPV vs. IMV | 0.92  (0.49−1.75) | ⨁〇〇◯ Very low | NA | - | 0.92  (0.40−1.75) | ⨁〇〇◯ Very low |
| HFNC vs. IMV | NA | - | 0.86  (0.37−1.97) | ⨁〇〇◯ Very low | 0.86  (0.37−1.97) | ⨁〇〇◯ Very low |
| NPPV vs. HFNC | 2.30  (0.97−5.44) | ⨁⨁〇◯ Low | 0.67  (0.34−1.32) | ⨁⨁〇◯ Low | 1.07  (0.63−1.83) | ⨁〇〇◯ Very low |

CI, confidence interval; COT, conventional oxygen therapy; HFNC, high flow nasal cannula; IMV, invasive mechanical ventilation; NA, not applicable; NPPV, noninvasive positive pressure ventilation; RR, risk ratio

* We adopted effect estimates based on direct evidence, because direct evidence was dominant for the network estimates.

2. Endotracheal intubation

| Comparison | Direct estimate (RR 95% CI) |  | Indirect estimate (RR 95% CI) | Rating | Network estimate (RR 95% CI) | Rating |
| --- | --- | --- | --- | --- | --- | --- |
| NPPV vs. COT | 0.66  (0.52−0.82) | ⨁⨁⨁◯ Moderate | 1.42  (0.32−6.33) | ⨁⨁〇◯ Low | 0.66 *  (0.52−0.82) | ⨁⨁⨁◯ Moderate |
| HFNC vs. COT | 0.88  (0.58−1.32) | ⨁⨁〇◯ Low | 0.25  (0.07−0.84) | ⨁⨁⨁◯ Moderate | 0.77  (0.52−1.32) | ⨁⨁〇◯ Low |
| NPPV vs. HFNC | 1.33  (0.71−2.48) | ⨁⨁〇◯ Low | 0.61  (0.35 −1.07) | ⨁⨁〇◯ Low | 0.86  (0.57−1.31) | ⨁〇〇◯ Very low |

CI, confidence interval; COT, conventional oxygen therapy; HFNC, high flow nasal cannula; NPPV, noninvasive positive pressure ventilation; RR, risk ratio.

* We adopted effect estimates based on direct evidence, because direct evidence was dominant for the network estimates.

1. Pneumonia

| Comparison | Direct estimate (RR 95% CI) |  | Indirect estimate (RR 95% CI) | Rating | Network estimate (RR 95% CI) | Rating |
| --- | --- | --- | --- | --- | --- | --- |
| NPPV vs. COT | 0.61  (0.40−0.92) | ⨁⨁⨁◯ Moderate | NA | - | 0.61  (0.40−0.92) | ⨁⨁⨁◯ Moderate |
| HFNC vs. COT | 0.44  (0.14−1.43) | ⨁〇〇◯ Very low | 0.07  (0.004−1.42) | ⨁〇〇◯ Very low | 0.35  (0.12−1.03) | ⨁⨁⨁◯ Moderate |
| IMV vs. COT | NA | - | 3.28  (1.37−7.88) | ⨁⨁⨁◯ Moderate | 3.28  (1.37−7.88) | ⨁⨁⨁◯ Moderate |
| NPPV vs. IMV | 0.19  (0.09−0.40) | ⨁⨁⨁◯ Moderate | NA | - | 0.19  (0.09−0.40) | ⨁⨁⨁◯ Moderate |
| HFNC vs. IMV | NA | - | 0.11  (0.03−0.40) | ⨁⨁⨁◯ Moderate | 0.11  (0.03−0.40) | ⨁⨁⨁◯ Moderate |
| NPPV vs. HFNC | 2.17  (0.69−6.85) | ⨁〇〇◯ Very low | 0.31  (0.01−7.94) | ⨁〇〇◯ Very low | 1.75  (0.59−5.16) | ⨁〇〇◯ Very low |

CI, confidence interval; COT, conventional oxygen therapy; HFNC, high flow nasal cannula; IMV, invasive mechanical ventilation; NA, not applicable; NPPV, noninvasive positive pressure ventilation; RR, risk ratio.

6.Evidence-to-Decision table

| Question | |
| --- | --- |
| **CQ16: Should HFNC be used over conventional oxygen therapy for patients with ARDS?** | |
| **Population:** | Adult patients aged ≥18 years who had acute hypoxemic respiratory failure defined by new-onset of clinical signs (e.g., tachypnea, increased work of breathing), radiologic signs (unilateral or bilateral chest radiograph opacities), and hypoxemia.  We excluded the randomized controlled trials (RCTs) that included more than half of patients with hypercapnia, congestive heart failure, chronic obstructive pulmonary disease, or asthma as the cause of respiratory failure, post-extubation respiratory failure, post-surgical, and post-trauma constituting. |
| **Intervention** | high-flow nasal cannula (HFNC) |
| **Comparison:** | Conventional oxygen therapy (COT) |
| **Main outcomes:** | Short-term mortality, Endotracheal intubation, Pneumonia |
| **Settings:** | Emergency department or intensive care unit |
| **Perspective:** | Individual |
| **Background:** | HFNC is used to avoid complications from tracheal intubation in patients with acute hypoxemic respiratory failure, but delayed intubation may increase the risk of death.  HFNC is not established to manage patients with ARDS. It is an important clinical issue to clarify whether HFNC is more effective than COT. |
| **Conflict of interests:** | None |

# Assessment

| Problem Is the problem a priority? | | |
| --- | --- | --- |
| Judgment | Research evidence | Additional considerations |
| ○ No  ○ Probably no  ● Probably yes  ○ Yes  ○ Varies  ○ Do not know | In a multicenter RCT that examined the effectiveness of noninvasive respiratory support in patients with hypoxemic respiratory failure^(1)^, HFNC reduced the incidence of endotracheal intubation and short-term mortality compared with NPPV and COT. However, other RCTs and meta-analyses had not demonstrated the efficacy of HFNC^(2)^.  Since the effects of HFNC have not been consistent in previous studies, this clinical issue should probably be given high priority. |  |
| Desirable effects How substantial are the desirable anticipated effects? | | |
| Judgment | Research evidence | Additional considerations |
| ○ Trivial  ○ Small  ● Moderate  ○ Large  ○ Varies  ○ Do not know | The search strategy identified 12,620 records, including 19 RCTs and 6 observational trials eligible for inclusion. Observational studies were excluded for meta-analyses due to serious concern for a high risk of bias. We performed network meta-analyses using 19 RCTs (N=2,777). Four trials (N=1,176) were included in a direct comparison between HFNC and COT.  In comparing NPPV and COT, the estimated value of the effects of short-term mortality (2 RCTs: N=976) was 117 fewer per 1,000 (95% CI: 215 fewer to 44 more), and endotracheal intubation (4 RCTs: N=1,176) was 89 fewer per 1,000 (95% CI: 187 fewer to 127 more). The estimated value of the effects of pneumonia (1 RCT: N=200) was a reduction of 82 per 1,000 (95% CI: 110 fewer to 4 more).  Based on the above, the desirable effects were considered “moderate.” |  |
| Undesirable effects How substantial are the undesirable anticipated effects? | | |
| Judgment | Research evidence | Additional considerations |
| ○ Large  ○ Moderate  ○ Small  ● Trivial  ○ Varies  ○ Do not know | The risk of aspiration during HFNC is unclear. We regarded pneumonia as an outcome of harm in this systematic review. Since HFNC reduced the occurrence of pneumonia, pneumonia was not an undesirable effect of HFNC.  Considering the description in the additional considerations, the undesirable effects of HFNC were considered to be “trivial.” | In an RCT in patients with acute hypoxemic respiratory failure ^(1)^, patients who received HFNC had improved comfort compared with COT and NPPV (100 mm visual analog scale: HFNC 29±26; COT 40±29; NPPV 43±29). |
| Certainty of evidence What is the overall certainty of the evidence of effects? | | |
| Judgment | Research evidence | Additional considerations |
| ○ Very low  ○ Low  ● Moderate  ○ High  ○ No included studies | **Importance and value of the main outcomes**   \| Outcome \| Importance \| Certainty of the evidence \| \| --- \| --- \| --- \| \| Short-term mortality \| Critical \| ⨁◯◯◯ \| \| Very low \| \| Endotracheal intubation \| Critical \| ⨁⨁◯◯ \| \| Low \| \| Pneumonia \| Critical \| ⨁⨁⨁◯ \| \| Moderate \|   **Overall certainty of the evidence**  Because directions of point estimates were consistent among all outcomes, the certainty of the evidence was considered to be “moderate” based on the highest certainty of evidence. |  |
| Values Is there important uncertainty about or variability in how much people value the main outcomes? | | |
| Judgment | Research evidence | Additional considerations |
| ○ Important uncertainty or variability  ○ Possibly important uncertainty or variability  ● Probably no important uncertainty or variability  ○ No important uncertainty or variability | We found no data on patient and family values in this systematic review. In general, the values of main outcomes were expected to be high and consistent. |  |
| Balance of effects Does the balance between desirable and undesirable effects favor the intervention or the comparison? | | |
| Judgment | Research evidence | Additional considerations |
| ○ Favors the comparison  ○ Probably favors the comparison  ○ Does not favor either the intervention or the comparison  ● Probably favors the intervention  ○ Favors the intervention  ○ Varies  ○ Do not know | **Summary of the evidence:**   \| Outcome \| COT (comparison)^*^ \| HFNC (intervention) ^*^ \| Risk difference (RD)  (95% CI) \| Risk ratio (RR) (95% CI) \| \| --- \| --- \| --- \| --- \| --- \| \| Short-term mortality \| 209/482  (43.4%) \| 195/494  (39.5%) \| 117 fewer /1,000 (215 fewer～44 more) \| 0.68 (0.41〜1.12) \| \| Endotracheal intubation \| 217/582  (37.3%) \| 194/594  (32.7%) \| 89 fewer /1,000  (187 fewer～127 more) \| 0.77 (0.52〜1.32) \| \| Pneumonia \| 8/94  (8.5%) \| 4/106  (3.8%) \| 82 fewer /1,000  (110 fewer〜4 more) \| 0.35 (0.12〜1.03) \|   * using direct comparison  Based on the above, the balance of the effects of HFNC was considered to be “probably favors the intervention.” |  |
| Acceptability Is the intervention acceptable to key stakeholders? | | |
| Judgment | Research evidence | Additional considerations |
| ○ No  ○ Probably no  ● Probably yes  ○ Yes  ○ Varies  ○ Do not know | Acceptability was considered “probably yes” because HFNC has already been performed to manage patients with respiratory failure. Furthermore, undesirable effects are rare using HFNC. Although HFNC is more expensive than COT, the potential benefit of avoiding endotracheal intubation may contribute to reducing the total cost. |  |
| Feasibility Is the intervention feasible to implement? | | |
| Judgment | Research evidence | Additional considerations |
| ○ No  ○ Probably no  ● Probably yes  ○ Yes  ○ Varies  ○ Do not know | Feasibility was considered to be “probably yes” because HFNC has already been performed to manage patients with respiratory failure.  If used on many patients at the same time, we should prepare enough oxygen stored. |  |

# Summary of Evidence

|  | **JUDGEMENT** | | | | | | |
| --- | --- | --- | --- | --- | --- | --- | --- |
| **PROBLEM** | No | Probably no | **Probably yes** | Yes |  | Varies | Unknown |
| **DESIRABLE EFFECTS** | Trivial | Small | **Moderate** | Large |  | Varies | Unknown |
| **UNDESIRABLE EFFECTS** | Large | Moderate | Small | **Trivial** |  | Varies | Unknown |
| **CERTAINTY OF EVIDENCE** | Very low | Low | **Moderate** | High |  |  | No included studies |
| **VALUES** | Important uncertainty or variability | Possibly important uncertainty or variability | **Probably no important uncertainty or variability** | No important uncertainty or variability |  |  |  |
| **BALANCE OF EFFECTS** | Favors the comparison | Probably favors the comparison | Does not favor either the intervention or the comparison | **Probably favors the intervention** | Favors the intervention | Varies | Unknown |
| **ACCEPTABILITY** | No | Probably no | **Probably yes** | Yes |  | Varies | Unknown |
| **FEASIBILITY** | No | Probably no | **Probably yes** | Yes |  | Varies | Unknown |

# Type of Recommendation

| Strong recommendation against the intervention | Conditional recommendation against the intervention | Conditional recommendation for either the intervention or the comparison | Conditional recommendation for the intervention | Strong recommendation for the intervention |
| --- | --- | --- | --- | --- |
| ○ | ○ | ○ | ● | ○ |

# Conclusion

| Recommendation |
| --- |
| **If the patient has no contraindications for HFNC and no organ failure other than respiratory failure, we suggest using HFNC for adult patients with acute hypoxemic respiratory failure who have probable ARDS compared with COT (Conditional recommendation, moderate certainty of evidence: GRADE 2B).**  **Supplementary condition:**  **Careful observation is needed after initiation of HFNC to avoid delayed intubation which may contribute to poor outcomes.**  **Contraindications for HFNC include the inability to protect the airway, high risk of vomiting, deterioration of consciousness, agitation, and unstable hemodynamics.** |
| Justification |
| **Question:** Should HFNC be used over conventional oxygen therapy for patients with ARDS?  **Population:** Adult patients with acute hypoxemic respiratory failure  **Intervention:** HFNC  **Main outcomes:** Short-term mortality, Endotracheal intubation, Pneumonia  **Summary of evidence:**  We performed network meta-analyses using 19 RCTs (N=2,777). Four trials (N=1,176) were included in a direct comparison between HFNC and COT.  In comparing HFNC and COT, the estimated value of the effects of short-term mortality (2 RCTs: N=976) was 117 fewer per 1,000 (95% CI: 1215 fewer to 44 more), and endotracheal intubation (14 RCTs: N=1,176) was 89 fewer per 1,000 (95% CI: 187 fewer to 127 more). The estimated value of the effects of pneumonia (1 RCT: N=200) was a reduction of 82 per 1,000 (95% CI: 110 fewer to 4 more). Therefore, the desirable effects were considered to be “moderate.”  In an RCT in patients with acute hypoxemic respiratory failure ^(1)^, patients who received HFNC had improved comfort compared with COT and NPPV (100 mm visual analog scale: HFNC 29±26; COT 40±29; NPPV 43±29). We judged that the undesirable effects of NPPV were “trivial.”  **Certainty of evidence:**  Because the directions of the point estimates were consistent among all outcomes, the certainty of the evidence was considered to be “moderate” based on the highest certainty of evidence.  However, the approach to assess imprecision in a network meta-analysis has not been established. We thought that the current network meta-analysis did not have a large enough sample size. If we rated down for imprecision, the certainty of evidence would be “low.”  **Values, Balance of effects, Acceptability, Feasibility:**  In general, the values of main outcomes were expected to be high and consistent. Balance of effects of HFNC was considered to be “probably favors the intervention”. Acceptability and feasibility were considered to be “probably yes” because HFNC has already been performed to manage patients with respiratory failure.  **Panel meeting:**  The modified Delphi method was used to form a consensus. Prior to the discussion conference, we sent the draft recommendations and materials prepared by the systematic reviewers to all panelists. As a result of voting, the median value of agreement was 8.0, and the disagreement index was 0.13 for the use of HFNC for adult patients with ARDS compared with COT.  In the discussion at the panel meeting, it was suggested to change the target population from ARDS to acute hypoxemic respiratory failure because we cannot diagnose ARDS without positive end-expiratory pressure based on the Berlin definition. Some panelists suggested a strong recommendation considering the balance of the effects and certainty of the evidence, similar to in CQ15.  After extensive discussion among the panelists, the current recommendation, “We suggest using HFNC for adult patients with acute hypoxemic respiratory failure who have probable ARDS compared with COT.” was approved (median value of agreement was 9.0, and disagreement index was 0.13). |
| Subgroup considerations |
| We did not perform sensitivity analyses. |
| Implementation considerations |
| In the previous clinical practice guideline for the management of ARDS in Japan (3), HFNC was not discussed, but it was noted that investigations in the future should be performed.  The Japanese Clinical Practice Guidelines for Management of Sepsis and Septic Shock 2020 (4) suggested conducting NPPV and HFNC for early respiratory failure in adult patients with sepsis. In the International Guidelines for Management of Sepsis and Septic Shock^(5, 6)^, no clinical questions regarding the use of HFNC or not in patients with ARDS were found.  A clinical practice guideline for using HFNC created by a working group of the European Society of Intensive Care Medicine ^(7)^ recommends using HFNC compared to COT for patients with hypoxemic respiratory failure. Since panel members differed on whether to give a strong or conditional recommendation, a conditional recommendation was given after voting. The rationale in favor of a conditional recommendation was possibly increasing the risk of delayed intubation and inconsistent efficacy across populations, including the cause and severity of respiratory failure.  If HFNC is used for patients with hypoxemic respiratory failure, including ARDS, close monitoring is needed to avoid delayed intubation. One of the clinical prediction rules is the ROX index, defined as the ratio of oxygen saturation measured by pulse oximetry / F_I_O_2_ to the respiratory rate^(8)^. |
| Monitoring and evaluation |
| After implementing the recommendations, further evaluation is needed, especially on the clinical effects and cost-effectiveness at each institution. In addition, it is desirable to monitor the implementation situation after the publication of the guideline to prevent other clinical problems. |
| Research priorities |
| Further evaluation is needed on the optimal timing of HFNC initiation combined with prone position therapy and the weaning process. |

References

1. Frat JP, Thille AW, Mercat A, Girault C, Ragot S, Perbet S, et al. High-flow oxygen through nasal cannula in acute hypoxemic respiratory failure. N Engl J Med. 2015;372(23):2185-96. PMID: 25981908.

2. Ferreyro BL, Angriman F, Munshi L, Del Sorbo L, Ferguson ND, Rochwerg B, et al. Association of noninvasive oxygenation strategies with all-cause mortality in adults with acute hypoxemic respiratory failure: a systematic review and meta-analysis. JAMA. 2020;324(1):57-67. PMID: 32496521

3. Hashimoto S, Sanui M, Egi M, Ohshimo S, Shiotsuka J, Seo R, et al. The clinical practice guideline for the management of ARDS in Japan. J Intensive Care. 2017;5:50. PMID: 28770093

4. Egi M, Ogura H, Yatabe T, Atagi K, Inoue S, Iba T, et al. The Japanese clinical practice guidelines for management of sepsis and septic shock 2020 (J-SSCG 2020). J Intensive Care. 2021;9(1):53. PMID: 34433491

5. Rhodes A, Evans LE, Alhazzani W, Levy MM, Antonelli M, Ferrer R, et al. Surviving sepsis campaign: international guidelines for management of sepsis and septic shock: 2016. Intensive Care Med. 2017;43(3):304-77. PMID: 28101605

6. Rhodes A, Evans LE, Alhazzani W, Levy MM, Antonelli M, Ferrer R, et al. Surviving sepsis campaign: international guidelines for management of sepsis and septic shock: 2016. Crit Care Med. 2017;45(3):486-552. PMID: 28098591

7. Rochwerg B, Einav S, Chaudhuri D, Mancebo J, Mauri T, Helviz Y, et al. The role for high flow nasal cannula as a respiratory support strategy in adults: a clinical practice guideline. Intensive Care Med. 2020;46(12):2226-37. PMID: 33201321

8. Roca O, Caralt B, Messika J, Samper M, Sztrymf B, Hernández G, et al. An index combining respiratory rate and oxygenation to predict outcome of nasal high-flow therapy. Am J Respir Crit Care Med. 2019;199(11):1368-76. PMID: 30576221

**CQ17 Should NPPV be used prior to conducting tracheal intubation in patients with ARDS?**

1.Search strategy

Short-term mortality

MEDLINE via PubMed (Search date: 2020/6/19)

| #1 | "Hypoxia"[mh] OR hypox*[tiab] OR "Respiratory Insufficiency"[mh] OR respiratory depression*[tiab] OR respiratory failure*[tiab] OR ventilatory depression*[tiab] OR respiratory insufficienc*[tiab] OR "Dyspnea"[mh] OR dyspnea*[tiab] OR "shortness of breath"[tiab] OR "Respiratory Distress Syndrome, Adult"[mh] OR acute respiratory distress[tiab] OR adult respiratory distress[tiab] OR respiratory distress syndrome*[tiab] OR RDS[tiab] OR ARDS[tiab] OR "Acute Lung Injury"[mh] OR acute lung injur*[tiab] OR ALI[tiab] |
| --- | --- |
| #2 | "Respiratory Distress Syndrome, Newborn"[mh] |
| #3 | #1 or #2 |
| #4 | "Noninvasive Ventilation"[mh] OR noninvasive ventilation*[tiab] OR non invasive ventilation*[tiab] OR NIV[tiab] OR NPPV[tiab] OR NIPPV[tiab] OR noninvasive positive pressure ventilation*[tiab] OR noninvasive mechanical ventilation*[tiab] OR noninvasive pressure support ventilation*[tiab] OR "Continuous Positive Airway Pressure"[mh] OR continuous positive airway pressure*[tiab] OR bilevel positive airway pressure*[tiab] OR biphasic positive airway pressure*[tiab] OR BIPAP[tiab] |
| #5 | "Oxygen Inhalation Therapy"[mh] OR HFNC[tiab] OR HHFNC[tiab] OR HHHFNC[tiab] OR HFNO[tiab] OR HFNT[tiab] OR HFNOT[tiab] OR HFO[tiab] OR HFOT[tiab] OR NHF[tiab] OR NHFC[tiab] OR NHFT[tiab] OR NHFO[tiab] OR NHFOT[tiab] OR high flow therap*[tiab] OR high flow oxygen[tiab] OR nasal high flow[tiab] |
| #6 | #4 or #5 |
| #7 | ("Randomized Controlled Trial"[pt] OR "Controlled Clinical Trial"[pt] OR "Clinical Trials as Topic"[mh] OR randomized[tiab] OR placebo[tiab] OR randomly[tiab] OR trial[tiab] OR groups[tiab]) NOT (Animals [mh] NOT Humans [mh]) |
| #8 | #3 and #6 and #7 |

CENTRAL (Search date: 2020/6/22)

| #1 | [mh Hypoxia] OR hypox*:ti,ab OR [mh "Respiratory Insufficiency"] OR "respiratory depression":ti,ab OR "respiratory failure":ti,ab OR "ventilatory depression":ti,ab OR "respiratory insufficiency":ti,ab OR [mh Dyspnea] OR dyspnea:ti,ab OR "shortness of breath":ti,ab OR [mh "Respiratory Distress Syndrome, Adult"] OR "acute respiratory distress":ti,ab OR "adult respiratory distress":ti,ab OR "respiratory distress syndrome":ti,ab OR RDS:ti,ab OR ARDS:ti,ab OR [mh "Acute Lung Injury"] OR "acute lung injury":ti,ab OR ALI:ti,ab |
| --- | --- |
| #2 | [mh "Respiratory Distress Syndrome, Newborn"] |
| #3 | #1 OR #2 |
| #4 | [mh "Noninvasive Ventilation"] OR "noninvasive ventilation":ti,ab OR "non invasive ventilation":ti,ab OR NIV:ti,ab OR NPPV:ti,ab OR NIPPV:ti,ab OR "noninvasive positive pressure ventilation":ti,ab OR "noninvasive mechanical ventilation":ti,ab OR "noninvasive pressure support ventilation":ti,ab OR [mh "Continuous Positive Airway Pressure"] OR "continuous positive airway pressure":ti,ab OR "bilevel positive airway pressure":ti,ab OR "biphasic positive airway pressure":ti,ab OR BIPAP:ti,ab |
| #5 | [mh "Oxygen Inhalation Therapy"] OR HFNC:ti,ab OR HHFNC:ti,ab OR HHHFNC:ti,ab OR HFNO:ti,ab OR HFNT:ti,ab OR HFNOT:ti,ab OR HFO:ti,ab OR HFOT:ti,ab OR NHF:ti,ab OR NHFC:ti,ab OR NHFT:ti,ab OR NHFO:ti,ab OR NHFOT:ti,ab OR "high flow therapy":ti,ab OR "high flow oxygen":ti,ab OR "nasal high flow":ti,ab |
| #6 | #4 OR #5 |
| #7 | #3 AND #6 |

Igaku-Chuo-Zasshi (Search date: 2020/6/19)

| #1 | 酸素欠乏/TH or 酸素欠乏/TA or anoxia/TA or Hypoxia/TA |
| --- | --- |
| #2 | 呼吸窮迫症候群-急性/TH or 急性呼吸窮迫症候群/TA or ARDS/TA |
| #3 | 急性肺損傷/TH or 急性肺損傷/TA or 急性肺障害/TA or 急性肺傷害/TA or ALI/TA |
| #4 | 呼吸不全/TH or 呼吸不全/TA |
| #5 | 呼吸困難/TH or 呼吸困難/TA |
| #6 | 呼吸窮迫症候群-新生児/TH or 新生児呼吸窮迫症候群/TA |
| #7 | #1 or #2 or #3 or #4 or #5 or #6 |
| #8 | 非侵襲的補助換気/TH or 非侵襲的補助換気/TA or NPPV/TA or NIPPV/TA |
| #9 | 持続気道陽圧/TH or 持続気道陽圧/TA or CPAP/TA |
| #10 | 非侵襲的陽圧呼吸/TH or 非侵襲的陽圧呼吸/TA or BIPAP/TA |
| #11 | 酸素吸入療法/TH or 酸素吸入/TA |
| #12 | 酸素療法/TA or ハイフロー/TA or HFNC/TA or NHF/TA or HFO/TA |
| #13 | #8 or #9 or #10 or #11 or #12 |
| #14 | #7 and #13 |
| #15 | (#14) and (PT=会議録除く) |
| #16 | ランダム化比較試験/TH or ランダム化/AL or 無作為化/AL |
| #17 | 比較試験/AL |
| #18 | 臨床試験/TH or 臨床試験/AL |
| #19 | プラセボ/TH or プラセボ/AL |
| #20 | 対照/AL |
| #21 | コントロール/AL |
| #22 | 臨床研究・疫学研究/TH or 臨床研究/AL |
| #23 | #16 or #17 or #18 or #19 or #20 or #21 or #22 |
| #24 | #15 and #23 |

Pneumonia

MEDLINE via PubMed（Search date: 2020/6/19）

|  | 検索式 |
| --- | --- |
| #1 | "Hypoxia"[mh] OR hypox*[tiab] OR "Respiratory Insufficiency"[mh] OR respiratory depression*[tiab] OR respiratory failure*[tiab] OR ventilatory depression*[tiab] OR respiratory insufficienc*[tiab] OR "Dyspnea"[mh] OR dyspnea*[tiab] OR "shortness of breath"[tiab] OR "Respiratory Distress Syndrome, Adult"[mh] OR acute respiratory distress[tiab] OR adult respiratory distress[tiab] OR respiratory distress syndrome*[tiab] OR RDS[tiab] OR ARDS[tiab] OR "Acute Lung Injury"[mh] OR acute lung injur*[tiab] OR ALI[tiab] |
| #2 | "Respiratory Distress Syndrome, Newborn"[mh] |
| #3 | #1 or #2 |
| #4 | "Noninvasive Ventilation"[mh] OR noninvasive ventilation*[tiab] OR non invasive ventilation*[tiab] OR NIV[tiab] OR NPPV[tiab] OR NIPPV[tiab] OR noninvasive positive pressure ventilation*[tiab] OR noninvasive mechanical ventilation*[tiab] OR noninvasive pressure support ventilation*[tiab] OR "Continuous Positive Airway Pressure"[mh] OR continuous positive airway pressure*[tiab] OR bilevel positive airway pressure*[tiab] OR biphasic positive airway pressure*[tiab] OR BIPAP[tiab] |
| #5 | "Oxygen Inhalation Therapy"[mh] OR HFNC[tiab] OR HHFNC[tiab] OR HHHFNC[tiab] OR HFNO[tiab] OR HFNT[tiab] OR HFNOT[tiab] OR HFO[tiab] OR HFOT[tiab] OR NHF[tiab] OR NHFC[tiab] OR NHFT[tiab] OR NHFO[tiab] OR NHFOT[tiab] OR high flow therap*[tiab] OR high flow oxygen[tiab] OR nasal high flow[tiab] |
| #6 | #4 or #5 |
| #7 | ("Randomized Controlled Trial"[pt] OR "Controlled Clinical Trial"[pt] OR "Clinical Trials as Topic"[mh] OR randomized[tiab] OR placebo[tiab] OR randomly[tiab] OR trial[tiab] OR groups[tiab]) NOT (Animals [mh] NOT Humans [mh]) |
| #8 | #3 and #6 and #7 |
| #9 | "Noninvasive Ventilation/adverse effects"[mh] OR noninvasive ventilation*[tiab] OR non invasive ventilation*[tiab] OR NIV[tiab] OR NPPV[tiab] OR NIPPV[tiab] OR noninvasive positive pressure ventilation*[tiab] OR noninvasive mechanical ventilation*[tiab] OR noninvasive pressure support ventilation*[tiab] OR "Continuous Positive Airway Pressure/adverse effects"[mh] OR continuous positive airway pressure*[tiab] OR bilevel positive airway pressure*[tiab] OR biphasic positive airway pressure*[tiab] OR BIPAP[tiab] |
| #10 | "Oxygen Inhalation Therapy/adverse effects"[mh] OR HFNC[tiab] OR HHFNC[tiab] OR HHHFNC[tiab] OR HFNO[tiab] OR HFNT[tiab] OR HFNOT[tiab] OR HFO[tiab] OR HFOT[tiab] OR NHF[tiab] OR NHFC[tiab] OR NHFT[tiab] OR NHFO[tiab] OR NHFOT[tiab] OR high flow therap*[tiab] OR high flow oxygen[tiab] OR nasal high flow[tiab] |
| #11 | #9 or #10 |
| #12 | harm*[tiab] OR side effect*[tiab] OR adverse[tiab] OR toxicity[tiab] OR infection*[tiab] OR pneumonia*[tiab] OR trauma[tiab] OR barotrauma*[tiab] OR volutrauma*[tiab] OR pneumothorax[tiab] OR ulcer*[tiab] OR discomfort*[tiab] OR breakdown[tiab] OR damage[tiab] OR intoleran*[tiab] OR "Barotrauma/complications"[mh] OR "Pneumonia/complications"[mh] OR "Pressure Ulcer/complications"[mh] OR "Pneumothorax/complications"[mh] OR "Pneumonia, Ventilator-Associated"[mh] |
| #13 | #3 and #11 and #12 |
| #14 | Animals [mh] NOT Humans [mh] |
| #15 | #13 not #14 |
| #16 | #15 not #8 |

CENTRAL（Search date: 2020/6/22）

|  | 検索式 |
| --- | --- |
| #1 | [mh Hypoxia] OR hypox*:ti,ab OR [mh "Respiratory Insufficiency"] OR "respiratory depression":ti,ab OR "respiratory failure":ti,ab OR "ventilatory depression":ti,ab OR "respiratory insufficiency":ti,ab OR [mh Dyspnea] OR dyspnea:ti,ab OR "shortness of breath":ti,ab OR [mh "Respiratory Distress Syndrome, Adult"] OR "acute respiratory distress":ti,ab OR "adult respiratory distress":ti,ab OR "respiratory distress syndrome":ti,ab OR RDS:ti,ab OR ARDS:ti,ab OR [mh "Acute Lung Injury"] OR "acute lung injury":ti,ab OR ALI:ti,ab |
| #2 | [mh "Respiratory Distress Syndrome, Newborn"] |
| #3 | #1 OR #2 |
| #4 | [mh "Noninvasive Ventilation"] OR "noninvasive ventilation":ti,ab OR "non invasive ventilation":ti,ab OR NIV:ti,ab OR NPPV:ti,ab OR NIPPV:ti,ab OR "noninvasive positive pressure ventilation":ti,ab OR "noninvasive mechanical ventilation":ti,ab OR "noninvasive pressure support ventilation":ti,ab OR [mh "Continuous Positive Airway Pressure"] OR "continuous positive airway pressure":ti,ab OR "bilevel positive airway pressure":ti,ab OR "biphasic positive airway pressure":ti,ab OR BIPAP:ti,ab |
| #5 | [mh "Oxygen Inhalation Therapy"] OR HFNC:ti,ab OR HHFNC:ti,ab OR HHHFNC:ti,ab OR HFNO:ti,ab OR HFNT:ti,ab OR HFNOT:ti,ab OR HFO:ti,ab OR HFOT:ti,ab OR NHF:ti,ab OR NHFC:ti,ab OR NHFT:ti,ab OR NHFO:ti,ab OR NHFOT:ti,ab OR "high flow therapy":ti,ab OR "high flow oxygen":ti,ab OR "nasal high flow":ti,ab |
| #6 | #4 OR #5 |
| #7 | #3 AND #6 |
| #8 | [mh "Noninvasive Ventilation"/ae] OR "noninvasive ventilation":ti,ab OR "non invasive ventilation":ti,ab OR NIV:ti,ab OR NPPV:ti,ab OR NIPPV:ti,ab OR "noninvasive positive pressure ventilation":ti,ab OR "noninvasive mechanical ventilation":ti,ab OR "noninvasive pressure support ventilation":ti,ab OR [mh "Continuous Positive Airway Pressure"/ae] OR "continuous positive airway pressure":ti,ab OR "bilevel positive airway pressure":ti,ab OR "biphasic positive airway pressure":ti,ab OR BIPAP:ti,ab |
| #9 | [mh "Oxygen Inhalation Therapy"/ae] OR HFNC:ti,ab OR HHFNC:ti,ab OR HHHFNC:ti,ab OR HFNO:ti,ab OR HFNT:ti,ab OR HFNOT:ti,ab OR HFO:ti,ab OR HFOT:ti,ab OR NHF:ti,ab OR NHFC:ti,ab OR NHFT:ti,ab OR NHFO:ti,ab OR NHFOT:ti,ab OR "high flow therapy":ti,ab OR "high flow oxygen":ti,ab OR "nasal high flow":ti,ab |
| #10 | #8 OR #9 |
| #11 | harm*:ti,ab OR "side effect":ti,ab OR adverse:ti,ab OR toxicity:ti,ab OR infection*:ti,ab OR pneumonia*:ti,ab OR trauma:ti,ab OR barotrauma*:ti,ab OR volutrauma*:ti,ab OR pneumothorax:ti,ab OR ulcer*:ti,ab OR discomfort*:ti,ab OR breakdown:ti,ab OR damage:ti,ab OR intoleran*:ti,ab OR [mh Barotrauma/co] OR [mh Pneumonia/co] OR [mh "Pressure Ulcer"/co] OR [mh Pneumothorax/co] OR [mh "Pneumonia, Ventilator-Associated"] |
| #12 | #3 AND #10 AND #11 |
| #13 | #12 NOT #7 |

Igaku-Chuo-Zasshi （Search date: 2020/6/19）

|  | 検索式 |
| --- | --- |
| #1 | 酸素欠乏/TH or 酸素欠乏/TA or anoxia/TA or Hypoxia/TA |
| #2 | 呼吸窮迫症候群-急性/TH or 急性呼吸窮迫症候群/TA or ARDS/TA |
| #3 | 急性肺損傷/TH or 急性肺損傷/TA or 急性肺障害/TA or 急性肺傷害/TA or ALI/TA |
| #4 | 呼吸不全/TH or 呼吸不全/TA |
| #5 | 呼吸困難/TH or 呼吸困難/TA |
| #6 | 呼吸窮迫症候群-新生児/TH or 新生児呼吸窮迫症候群/TA |
| #7 | #1 or #2 or #3 or #4 or #5 or #6 |
| #8 | 非侵襲的補助換気/TH or 非侵襲的補助換気/TA or NPPV/TA or NIPPV/TA |
| #9 | 持続気道陽圧/TH or 持続気道陽圧/TA or CPAP/TA |
| #10 | 非侵襲的陽圧呼吸/TH or 非侵襲的陽圧呼吸/TA or BIPAP/TA |
| #11 | 酸素吸入療法/TH or 酸素吸入/TA |
| #12 | 酸素療法/TA or ハイフロー/TA or HFNC/TA or NHF/TA or HFO/TA |
| #13 | #8 or #9 or #10 or #11 or #12 |
| #14 | #7 and #13 |
| #15 | (#14) and (PT=会議録除く) |
| #16 | ランダム化比較試験/TH or ランダム化/AL or 無作為化/AL |
| #17 | 比較試験/AL |
| #18 | 臨床試験/TH or 臨床試験/AL |
| #19 | プラセボ/TH or プラセボ/AL |
| #20 | 対照/AL |
| #21 | コントロール/AL |
| #22 | 臨床研究・疫学研究/TH or 臨床研究/AL |
| #23 | #16 or #17 or #18 or #19 or #20 or #21 or #22 |
| #24 | #15 and #23 |
| #25 | ((非侵襲的補助換気/TH) and (SH=有害作用)) or 非侵襲的補助換気/TA or NPPV/TA or NIPPV/TA |
| #26 | ((持続気道陽圧/TH) and (SH=有害作用)) or 持続気道陽圧/TA or CPAP/TA |
| #27 | ((非侵襲的陽圧呼吸/TH) and (SH=有害作用)) or 非侵襲的陽圧呼吸/TA or BIPAP/TA |
| #28 | ((酸素吸入療法/TH) and (SH=有害作用)) or 酸素吸入/TA |
| #29 | 酸素療法/TA or ハイフロー/TA or HFNC/TA or NHF/TA or HFO/TA |
| #30 | #25 or #26 or #27 or #28 or #29 |
| #31 | 害/TA or 副作用/TA or 毒性/TA or 感染/TA or 肺炎/TA or 外傷/TA or トラウマ/TA or 気胸/TA or 潰瘍/TA or 不快感/TA or 損傷/TA or 気圧障害/TA or ((気圧障害/TH) and (SH=合併症)) or ((肺炎/TH) and (SH=合併症)) or ((褥瘡性潰瘍/TH) and (SH=合併症)) or ((気胸/TH) and (SH=合併症)) or 人工呼吸器関連肺炎/TH |
| #32 | #7 and #30 and #31 |
| #33 | (#32) and (PT=会議録除く) |
| #34 | #43 not #24 |

2.Flow diagram

Short-term mortality

**Identification**

21 Studies included in qualitative synthesis

6482 records after duplicates removed

7771 records identified through database searching

7771 records identified through database searching

Medline via PubMed (n=3403)

Cochrane CENTRAL (n=3924)

Igaku-Chuo-Zasshi (n=444)

0 additional records identified through other sources

19 Studies included in quantitative synthesis (meta-analysis)

Duplicates

n=1289

6374 records excluded

**Included**

**Eligibility**

**Screening**

87 Full-text articles excluded, with reasons:

・Wrong language (n=4)

・Wrong study design (n=50)

・Wrong population (n=27)

・Wrong intervention (n=4)

・Duplicates (n=1)

・Difficult to obtain full text (n=1)

Etc.

108 Full-text articles assessed for eligibility

Pneumonia

**Identification**

21 Studies included in qualitative synthesis

9104 records after duplicates removed

10401 records identified through database searching

2630 records identified through database searching

Medline via PubMed (n=1442)

Cochrane CENTRAL (n=0)

Igaku-Chuo-Zasshi (n=1188)

7771 additional records identified through other sources

10 Studies included in quantitative synthesis (meta-analysis)

Duplicates

n=1297

8976 records excluded

**Included**

**Eligibility**

**Screening**

107 Full-text articles excluded, with reasons:

・Wrong language (n=4)

・Wrong study design (n=26)

・Wrong population (n=42)

・Wrong intervention (n=13)

・Duplicates (n=1)

・Conference abstract (n=20)

・Difficult to obtain full text (n=1)

Etc.

128 Full-text articles assessed for eligibility

1. Risk of bias

NPPV vs MV

Short-term mortality Pneumonia

1. Forest plot

NPPV vs MV

Short-term mortality

Pneumonia

1. Evidence profile

Details of assessments of certainty of estimates from NMA

1. Short-term mortality

| **Comparison** | **NPPV vs. COT** | **HFNC vs. COT** | **IMV vs. COT** | **NPPV vs. IMV** | **HFNC vs. IMV** | **NPPV vs. HFNC** |  |
| --- | --- | --- | --- | --- | --- | --- | --- |
| **Direct evidence** | | | | | | | |
| Risk of bias | Not serious | Not serious |  | Not serious |  | Not serious |  |
| Inconsistency | Serious^a^ | Serious^a^ |  | Serious^a^ |  | Not serious |  |
| Indirectness | Not serious | Not serious |  | Serious^b^ |  | Not serious |  |
| Publication bias | Undetected | Undetected |  | Undetected |  | Undetected |  |
| Preliminary rating direct | **Moderate** | **Moderate** |  | **Low** |  | **High** |  |
| Contributes as much as indirect | No | Yes |  | Yes |  | Yes |  |
| Need to assess indirect | Yes | Yes |  | Yes |  | Yes |  |
| Imprecision | Not serious | Serious^c^ |  | Very serious^c^ |  | Very serious^c, d^ |  |
| Final direct rating | **Moderate** | **Low** |  | **Very low** |  | **Low** |  |
| **Indirect evidence** | | | | | | | |
| Common comparator | HFNC | NPPV | NPPV |  | NPPV | COT |  |
| Intervention 1 vs. common comparator rating | **High** | **High** | **Low** |  | **High** | **Moderate** |  |
| Intervention 2 vs. common comparator rating | **Moderate** | **Moderate** | **Moderate** |  | **Low** | **Moderate** |  |
| Lowest of the two | **Moderate** | **Moderate** | **Low** |  | **Low** | **Moderate** |  |
| Intransitivity | Not serious | Not serious | Not serious |  | Not serious | Not serious |  |
| Preliminary rating indirect | **Moderate** | **Moderate** | **Low** |  | **Low** | **Moderate** |  |
| Imprecision | Very serious^c^ | Not serious | Very serious^c^ |  | Very serious^c^ | Very serious^c^ |  |
| Final indirect rating | **Very low** | **Moderate** | **Very low** |  | **Very low** | **Very low** |  |
| **Network evidence** | | | | | | | |
| Highest between direct and indirect | **Moderate** | **Moderate** | **Low** | **Low** | **Low** | **High** |  |
| Incoherence | Not serious | Serious^e^ | NA | NA | NA | Serious^e^ |  |
| Imprecision | Not serious | Serious^c^ | Very serious^c^ | Very serious^c^ | Very serious^c^ | Very serious^c, d^ |  |
| Final network rating | **Moderate** | **Very low** | **Very low** | **Very low** | **Very low** | **Very low** |  |
| Most credible estimate | **Direct** | **Network** | **Network** | **Network** | **Network** | **Network** |  |

a: Due to high I^2^.

b: In the included two RCTs, lung protective ventilation was not performed.

c: Confidence intervals contained both substantial benefit and harm.

d: Meta-analysis using only one RCT.

e: Difference between confidence intervals of direct and indirect estimates.

COT, conventional oxygen therapy; HFNC, high flow nasal cannula; IMV, invasive mechanical ventilation; NA, not applicable; NPPV, noninvasive positive pressure ventilation; RCT, randomized controlled trial.

1. Endotracheal intubation

| **Comparison** | **NPPV vs. COT** | **HFNC vs. COT** | **NPPV vs. HFNC** |
| --- | --- | --- | --- |
| **Direct evidence** | | | |
| Risk of bias | Not serious | Not serious | Not serious |
| Inconsistency | Serious^a^ | Not serious | Not serious |
| Indirectness | Not serious | Not serious | Not serious |
| Publication bias | Undetected | Undetected | Undetected |
| Preliminary rating direct | **Moderate** | **High** | **High** |
| Contributes as much as indirect | No | Yes | Yes |
| Need to assess indirect | Yes | Yes | Yes |
| Imprecision | Not serious | Very serious^b^ | Very serious^c^ |
| Final direct rating | **Moderate** | **Low** | **Low** |
| **Indirect evidence** | | | |
| Common comparator | HFNC | NPPV | COT |
| Intervention 1 vs. common comparator rating | **High** | **High** | **Moderate** |
| Intervention 2 vs. common comparator rating | **High** | **Moderate** | **High** |
| Lowest of the two | **High** | **Moderate** | **Moderate** |
| Intransitivity | Not serious | Not serious | Not serious |
| Preliminary rating indirect | **High** | **Moderate** | **Moderate** |
| Imprecision | Very serious^b^ | Not serious | Serious^c^ |
| Final indirect rating | **Low** | **Moderate** | **Low** |
| **Network evidence** | | | |
| Highest between direct and indirect | **High** | **High** | **High** |
| Incoherence | Serious^c^ | Not serious | Serious^d^ |
| Imprecision | Not serious | Very serious^b^ | Very serious^b^ |
| Final network rating | **Moderate** | **Low** | **Very low** |
| Most credible estimate | **Direct** | **Network** | **Network** |

a: Due to high I^2^.

b: Confidence intervals contained both substantial benefit and harm.

c: Confidence intervals contained substantial benefit and no benefit.

d: Difference between confidence intervals of direct and indirect estimates.

COT, conventional oxygen therapy; HFNC, high flow nasal cannula; NPPV, noninvasive positive pressure ventilation.

1. Pneumonia

| **Comparison** | **NPPV vs. COT** | **HFNC vs. COT** | **IMV vs. COT** | **NPPV vs. IMV** | **HFNC vs. IMV** | **NPPV vs. HFNC** |  |
| --- | --- | --- | --- | --- | --- | --- | --- |
| **Direct evidence** | | | | | | | |
| Risk of bias | Serious^a^ | Serious^a^ |  | Serious^a^ |  | Serious^a^ |  |
| Inconsistency | Not serious | Not serious |  | Not serious |  | Not serious |  |
| Indirectness | Not serious | Not serious |  | Not serious |  | Not serious |  |
| Publication bias | Undetected | Undetected |  | Undetected |  | Undetected |  |
| Preliminary rating direct | **Moderate** | **Moderate** |  | **Moderate** |  | **Moderate** |  |
| Contributes as much as indirect | No | Yes |  | Yes |  | Yes |  |
| Need to assess indirect | Yes | Yes |  | Yes |  | Yes |  |
| Imprecision | Not serious | Very serious^b^ |  | Not serious |  | Very serious^b^ |  |
| Final direct rating | **Moderate** | **Very low** |  | **Moderate** |  | **Very low** |  |
| **Indirect evidence** | | | | | | | |
| Common comparator |  | NPPV | NPPV |  | NPPV | COT |  |
| Intervention 1 vs. common comparator rating |  | **Moderate** | **Moderate** |  | **Moderate** | **Moderate** |  |
| Intervention 2 vs. common comparator rating |  | **Moderate** | **Moderate** |  | **Moderate** | **Moderate** |  |
| Lowest of the two |  | **Moderate** | **Moderate** |  | **Moderate** | **Moderate** |  |
| Intransitivity |  | Not serious | Not serious |  | Not serious | Not serious |  |
| Preliminary rating indirect |  | **Moderate** | **Moderate** |  | **Moderate** | **Moderate** |  |
| Imprecision |  | Very serious^b^ | Not serious |  | Not serious | Very serious^b^ |  |
| Final indirect rating |  | **Very low** | **Moderate** |  | **Moderate** | **Very low** |  |
| **Network evidence** | | | | | | | |
| Highest between direct and indirect | **Moderate** | **Moderate** | **Moderate** | **Moderate** | **Moderate** | **Moderate** |  |
| Incoherence | NA | Not serious | NA | NA | NA | Serious^c^ |  |
| Imprecision | Not serious | Not serious | Not serious | Not serious | Not serious | Very serious^b^ |  |
| Final network rating | **Moderate** | **Moderate** | **Moderate** | **Moderate** | **Moderate** | **Very low** |  |
| Most credible estimate | **Network** | **Network** | **Network** | **Network** | **Network** | **Network** |  |

a: Intervention was not blinded, and definitions of pneumonia was unclear.

b: Confidence intervals contain both substantial benefit and harm.

c: Difference between confidence intervals of direct and indirect estimates.

COT, conventional oxygen therapy; HFNC, high flow nasal cannula; IMV, invasive mechanical ventilation; NA, not applicable; NPPV, noninvasive positive pressure ventilation; RCT, randomized controlled trial.

Results and certainty assessments for the outcome

1. Short-term mortality

| Comparison | Direct estimate (RR 95% CI) |  | Indirect estimate (RR 95% CI) | Rating | Network estimate (RR 95% CI) | Rating |
| --- | --- | --- | --- | --- | --- | --- |
| NPPV vs. COT | 0.71  (0.55−0.92) | ⨁⨁⨁◯ Moderate | 3.82  (0.58−25.05) | ⨁〇〇◯ Very low | 0.71 *  (0.55−0.92) | ⨁⨁⨁◯ Moderate |
| HFNC vs. COT | 0.79  (0.47−1.32) | ⨁⨁〇◯ Low | 0.13  (0.02−0.74) | ⨁⨁⨁◯ Moderate | 0.68  (0.41−1.12) | ⨁〇〇◯ Very low |
| IMV vs. COT | NA | - | 0.79  (0.40–1.57) | ⨁〇〇◯ Very low | 0.79  (0.40−1.57) | ⨁〇〇◯ Very low |
| NPPV vs. IMV | 0.92  (0.49−1.75) | ⨁〇〇◯ Very low | NA | - | 0.92  (0.40−1.75) | ⨁〇〇◯ Very low |
| HFNC vs. IMV | NA | - | 0.86  (0.37−1.97) | ⨁〇〇◯ Very low | 0.86  (0.37−1.97) | ⨁〇〇◯ Very low |
| NPPV vs. HFNC | 2.30  (0.97−5.44) | ⨁⨁〇◯ Low | 0.67  (0.34−1.32) | ⨁⨁〇◯ Low | 1.07  (0.63−1.83) | ⨁〇〇◯ Very low |

CI, confidence interval; COT, conventional oxygen therapy; HFNC, high flow nasal cannula; IMV, invasive mechanical ventilation; NA, not applicable; NPPV, noninvasive positive pressure ventilation; RR, risk ratio

* We adopted effect estimates based on direct evidence, because direct evidence was dominant for the network estimates.

2. Endotracheal intubation

| Comparison | Direct estimate (RR 95% CI) |  | Indirect estimate (RR 95% CI) | Rating | Network estimate (RR 95% CI) | Rating |
| --- | --- | --- | --- | --- | --- | --- |
| NPPV vs. COT | 0.66  (0.52−0.82) | ⨁⨁⨁◯ Moderate | 1.42  (0.32−6.33) | ⨁⨁〇◯ Low | 0.66 *  (0.52−0.82) | ⨁⨁⨁◯ Moderate |
| HFNC vs. COT | 0.88  (0.58−1.32) | ⨁⨁〇◯ Low | 0.25  (0.07−0.84) | ⨁⨁⨁◯ Moderate | 0.77  (0.52−1.32) | ⨁⨁〇◯ Low |
| NPPV vs. HFNC | 1.33  (0.71−2.48) | ⨁⨁〇◯ Low | 0.61  (0.35 −1.07) | ⨁⨁〇◯ Low | 0.86  (0.57−1.31) | ⨁〇〇◯ Very low |

CI, confidence interval; COT, conventional oxygen therapy; HFNC, high flow nasal cannula; NPPV, noninvasive positive pressure ventilation; RR, risk ratio.

* We adopted effect estimates based on direct evidence, because direct evidence was dominant for the network estimates.

1. Pneumonia

| Comparison | Direct estimate (RR 95% CI) |  | Indirect estimate (RR 95% CI) | Rating | Network estimate (RR 95% CI) | Rating |
| --- | --- | --- | --- | --- | --- | --- |
| NPPV vs. COT | 0.61  (0.40−0.92) | ⨁⨁⨁◯ Moderate | NA | - | 0.61  (0.40−0.92) | ⨁⨁⨁◯ Moderate |
| HFNC vs. COT | 0.44  (0.14−1.43) | ⨁〇〇◯ Very low | 0.07  (0.004−1.42) | ⨁〇〇◯ Very low | 0.35  (0.12−1.03) | ⨁⨁⨁◯ Moderate |
| IMV vs. COT | NA | - | 3.28  (1.37−7.88) | ⨁⨁⨁◯ Moderate | 3.28  (1.37−7.88) | ⨁⨁⨁◯ Moderate |
| NPPV vs. IMV | 0.19  (0.09−0.40) | ⨁⨁⨁◯ Moderate | NA | - | 0.19  (0.09−0.40) | ⨁⨁⨁◯ Moderate |
| HFNC vs. IMV | NA | - | 0.11  (0.03−0.40) | ⨁⨁⨁◯ Moderate | 0.11  (0.03−0.40) | ⨁⨁⨁◯ Moderate |
| NPPV vs. HFNC | 2.17  (0.69−6.85) | ⨁〇〇◯ Very low | 0.31  (0.01−7.94) | ⨁〇〇◯ Very low | 1.75  (0.59−5.16) | ⨁〇〇◯ Very low |

CI, confidence interval; COT, conventional oxygen therapy; HFNC, high flow nasal cannula; IMV, invasive mechanical ventilation; NA, not applicable; NPPV, noninvasive positive pressure ventilation; RR, risk ratio.

1. Evidence-to-Decision table

| Question | |
| --- | --- |
| **CQ17: Should NPPV be used prior to conducting tracheal intubation in patients with ARDS?** | |
| **Population:** | Adult patients aged ≥18 years who had acute hypoxemic respiratory failure defined by new-onset of clinical signs (e.g., tachypnea, increased work of breathing), radiologic signs (unilateral or bilateral chest radiograph opacities), and hypoxemia.  We excluded the randomized controlled trials (RCTs) that included more than half of patients with hypercapnia, congestive heart failure, chronic obstructive pulmonary disease (COPD), or asthma as the cause of respiratory failure, post-extubation respiratory failure, post-surgical, and post-trauma constituting. |
| **Intervention** | Non-invasive positive pressure ventilation (NPPV) |
| **Comparison:** | Invasive mechanical ventilation (IMV) |
| **Main outcomes:** | Short-term mortality, Pneumonia |
| **Settings:** | Emergency department or intensive care unit |
| **Perspective:** | Individual |
| **Background:** | Almost half of the patients with ARDS who received NPPV were intubated due to treatment failure. Previous clinical practice guidelines have noted no suggestions based on evidence compared with IMV. It is an important clinical issue to clarify whether NPPV is more effective than IMV. |
| **Conflict of interests:** | None |

# Assessment

| Problem Is the problem a priority? | | |
| --- | --- | --- |
| Judgment | Research evidence | Additional considerations |
| ○ No  ○ Probably no  ● Probably yes  ○ Yes  ○ Varies  ○ Do not know | The previous clinical practice guideline for the management of ARDS in Japan (1) suggested using NPPV for early respiratory management in adults with ARDS. Patients with ARDS are at a high risk for NPPV failure. Treatment failure is associated with higher in-hospital mortality. Careful observation is needed, even though NPPV is performed for ARDS. NPPV is not established to manage patients with ARDS but rather cardiogenic pulmonary edema and acute exacerbations of COPD. Therefore, this issue should probably be given high priority. |  |
| Desirable Effects How substantial are the desirable anticipated effects? | | |
| Judgment | Research evidence | Additional considerations |
| ○ Trivial  ○ Small  ● Moderate  ○ Large  ○ Varies  ○ Do not know | The search strategy identified 12,620 records, including 19 RCTs and 6 observational trials eligible for inclusion. Observational studies were excluded for meta-analyses due to serious concern for a high risk of bias. We performed network meta-analyses using 19 RCTs (N=2,777).  Two trials (N=129) were included in a direct comparison between NPPV and IMV. No loops were found to form indirect comparisons.  In comparing NPPV and IMV, the estimated value of the effects of short-term mortality (2 RCTs: N=129) was 31 fewer per 1,000 (95% CI: 203 fewer to 296 more), and pneumonia (2 RCTs: N=129) was 401 fewer per 1,000 (95% CI: 450 fewer to 295 fewer).  Based on the above, the desirable effects were considered “moderate.” |  |
| Undesirable effects How substantial are the undesirable anticipated effects? | | |
| Judgment | Research evidence | Additional considerations |
| ○ Large  ○ Moderate  ○ Small  ● Trivial  ○ Varies  ○ Do not know | NPPV cannot ensure the protection of the airways. We adopted pneumonia as a critical outcome of harm for this systematic review. We did not identify any significant undesirable effects using NPPV.  Based on these, the undesirable effects of NPPV were considered to be “trivial.” | An RCT reported the incidence of skin damage which was an important outcome of harm (2). Skin damage was more frequently observed in patients with NPPV (3/32 [9.4%] vs. 0/32 [0%]). |
| Certainty of evidence What is the overall certainty of the evidence of effects? | | |
| Judgment | Research evidence | Additional considerations |
| ○ Very low  ○ Low  ● Moderate  ○ High  ○ No included studies | **Importance and value of the main outcomes**   \| Outcome \| Importance \| Certainty of the evidence \| \| --- \| --- \| --- \| \| Short-term mortality \| Critical \| ⨁◯◯◯ \| \| Very low \| \| Pneumonia \| Critical \| ⨁⨁⨁◯ \| \| Moderate \|   **Overall certainty of the evidence**  Because directions of the point estimates were consistent among all outcomes, the certainty of the evidence was considered to be “moderate” based on the highest certainty of evidence. |  |
| Values Is there important uncertainty about or variability in how much people value the main outcomes? | | |
| Judgment | Research evidence | Additional considerations |
| ○ Important uncertainty or variability  ○ Possibly important uncertainty or variability  ● Probably no important uncertainty or variability  ○ No important uncertainty or variability | We found no data on patient and family values in this systematic review. In general, the values of the main outcomes were expected to be high and consistent. |  |
| Balance of effects Does the balance between desirable and undesirable effects favor the intervention or the comparison? | | |
| Judgment | Research evidence | Additional considerations |
| ○ Favors the comparison  ○ Probably favors the comparison  ○ Does not favor either the intervention or the comparison  ● Probably favors the intervention  ○ Favors the intervention  ○ Varies  ○ Do not know | **Summary of evidence:**   \| Outcome \| IMV (comparison)^*^ \| NPPV (intervention) ^*^ \| Risk difference (RD)  (95% CI) \| Risk ratio (RR) (95% CI) \| \| --- \| --- \| --- \| --- \| --- \| \| Short-term mortality \| 25/63  (39.7%) \| 24/66  (36.4%) \| 31 fewer /1,000 (203 fewer-296 more) \| 0.92 (0.49-1.75) \| \| Pneumonia \| 31/63  (49.2%) \| 6/66  (9.1%) \| 401 fewer /1,000 ( 450 fewer-295 fewer) \| 0.19 (0.09-0.40) \|   * using direct comparison  Based on the above, the balance of the effects of NPPV was considered to be “probably favors the intervention.” |  |
| Acceptability Is the intervention acceptable to key stakeholders? | | |
| Judgment | Research evidence | Additional considerations |
| ○ No  ○ Probably no  ● Probably yes  ○ Yes  ○ Varies  ○ Do not know | Acceptability was considered to be “probably yes” because NPPV has already been performed to manage patients with respiratory failure.  Furthermore, undesirable effects are rare using NPPV. |  |
| Feasibility Is the intervention feasible to implement? | | |
| Judgment | Research evidence | Additional considerations |
| ○ No  ○ Probably no  ● Probably yes  ○ Yes  ○ Varies  ○ Do not know | Feasibility was considered to be “probably yes” because NPPV has already been performed to manage patients with respiratory failure. |  |

# Summary of Evidence

|  | **JUDGMENT** | | | | | | |
| --- | --- | --- | --- | --- | --- | --- | --- |
| **PROBLEM** | No | Probably no | **Probably yes** | Yes |  | Varies | Unknown |
| **DESIRABLE EFFECTS** | Trivial | Small | **Moderate** | Large |  | Varies | Unknown |
| **UNDESIRABLE EFFECTS** | Large | Moderate | Small | **Trivial** |  | Varies | Unknown |
| **CERTAINTY OF EVIDENCE** | Very low | Low | **Moderate** | High |  |  | No included studies |
| **VALUES** | Important uncertainty or variability | Possibly important uncertainty or variability | **Probably no important uncertainty or variability** | No important uncertainty or variability |  |  |  |
| **BALANCE OF EFFECTS** | Favors the comparison | Probably favors the comparison | Does not favor either the intervention or the comparison | **Probably favors the intervention** | Favors the intervention | Varies | Unknown |
| **ACCEPTABILITY** | No | Probably no | **Probably yes** | Yes |  | Varies | Unknown |
| **FEASIBILITY** | No | Probably no | **Probably yes** | Yes |  | Varies | Unknown |

# Type of Recommendation

| Strong recommendation against the intervention | Conditional recommendation against the intervention | Conditional recommendation for either the intervention or the comparison | Conditional recommendation for the intervention | Strong recommendation for the intervention |
| --- | --- | --- | --- | --- |
| ○ | ○ | ○ | ● | ○ |

# Conclusion

| Recommendation |
| --- |
| **If the patient has no contraindications for NPPV and no organ failure other than respiratory failure, we suggest using NPPV for adult patients with acute hypoxemic respiratory failure who have probable ARDS compared with IMV, as initial respiratory management (Conditional recommendation, moderate certainty of evidence: GRADE 2B).**  **Supplementary condition:**  **Careful observation is needed after the initiation of NPPV to avoid delayed intubation which may contribute to poor outcomes.**  **Contraindications for NPPV include the inability to protect the airway, high risk of vomiting, deterioration of consciousness, agitation, and unstable hemodynamics.** |
| Justification |
| **Question:** Should NPPV be used prior to conducting tracheal intubation in patients with ARDS?  **Population:** Adult patients with acute hypoxemic respiratory failure  **Intervention:** NPPV  **Main outcomes:** Short-term mortality, Pneumonia  **Summary of evidence:**  We performed network meta-analyses using 19 RCTs (N=2,777). Two trials (N=129) were included to compare NPPV and IMV. No loops were found to form indirect comparisons.  In comparing NPPV and IMV, the estimated value of the effects of short-term mortality (2 RCTs: N=129) was 31 fewer per 1,000 (95% CI: 203 fewer to 296 more), and pneumonia (2 RCTs: N=129) was 401 fewer per 1,000 (95% CI: 450 fewer to 295 fewer). Therefore, the desirable effects were considered to be “moderate.”  An RCT reported the incidence of skin damage which was an important outcome of harm (2). Skin damage was more frequently observed in patients with NPPV (3/32 [9.4%] vs. 0/32 [0%]). PPV cannot ensure the protection of the airways. We adopted pneumonia as a critical outcome of harm for this systematic review. We did not identify any significant undesirable effects of the use of NPPV. We judged that the undesirable effects of NPPV were “trivial.”  **Certainty of evidence:**  Because the directions of the point estimates were consistent among all outcomes, the certainty of the evidence was considered to be “moderate” based on the highest certainty of evidence. However, the approach to assess imprecision in a network meta-analysis has not been established. We thought that the current network meta-analysis did not have a large enough sample size. If we rated down for imprecision, the certainty of evidence would be “low.”  **Values, Balance of effects, Acceptability, Feasibility:**  In general, the values of the main outcomes were expected to be high and consistent. The balance of the effects of NPPV was considered to be “probably favors the intervention.” Acceptability and feasibility were considered to be “probably yes” because NPPV has already been performed to manage patients with respiratory failure.  **Panel meeting:**  The modified Delphi method was used to form a consensus. Prior to the discussion conference, we sent draft recommendations and materials prepared by the systematic reviewers to all panelists. As a result of voting, the median value of agreement was 8.0, and the disagreement index was 0.29 for the use of NPPV for adult patients with ARDS before intubation.  In the discussion at the panel meeting, it was suggested to change the target population from ARDS to acute hypoxemic respiratory failure because we cannot diagnose ARDS without positive end-expiratory pressure based on the Berlin definition. Since some panelists raised the concern for imprecision due to the small sample size, additional explanation is noted in the section of certainty of evidence.  After extensive discussion among the panelists, the current recommendation, “We suggest using NPPV for adult patients with acute hypoxemic respiratory failure who have probable ARDS compared with IMV, as initial respiratory management.” was approved (median value of agreement was 8.0, and disagreement index was 0.22). |
| Subgroup considerations |
| A systematic review and network meta-analysis dividing NPPV into a helmet and face mask demonstrated that non-invasive respiratory support, including NPPV and HFNC, were superior to COT and that helmet NPPV was the most effective in reducing the risk of short-term mortality and endotracheal intubation^(3)^. However, continuous positive airway pressure (CPAP) was used as a non-invasive ventilation mode along with helmet non-invasive ventilation in most RCTs included in this network meta-analysis. PSV may increase the tidal volume, and a larger tidal volume is a risk for NPPV failure^(4)^. We performed a sensitivity analysis to evaluate the effect of NPPV mode (CPAP and PSV).  Compared with IMV, CPAP was significantly associated with a lower risk of mortality (no direct comparison; RD: 150 fewer per 1,000; 95% CI: 296 fewer to 206 more), and pneumonia (no direct comparison; RD: 463 fewer per 1,000; 95% CI: 487 fewer to 322 fewer).  Compared with IMV, PSV was significantly associated with a lower risk of mortality (2 RCTs: N=129; RD: 31 fewer per 1,000; 95% CI: 204 fewer to 299 more), and pneumonia (2 RCTs: N=129; RD: 382 fewer per 1,000; 95% CI: 491 fewer to 375 fewer). The probability of being the best in the main outcomes was higher for CPAP than for PSV. |
| Implementation considerations |
| The previous clinical practice guideline for the management of ARDS in Japan (1) suggested using NPPV for early respiratory management in adults with ARDS. The Japanese Clinical Practice Guidelines for Management of Sepsis and Septic Shock 2020 (5) suggested conducting NPPV and HFNC for early respiratory failure in adult patients with sepsis. There was no recommendation for or against using NPPV in patients with ARDSin international guidelines^(6-8)^. If NIV is used for patients with ARDS, close monitoring of tidal volumes is suggested.  When NPPV is performed in patients with ARDS who are at a high risk for treatment failure, we should monitor respiratory and circulatory status to avoid delayed intubation.  The Heart rate, Acidosis, Consciousness, Oxygenation, and Respiratory rate (HACOR), which includes vital signs and data from blood gas analysis, may be an effective way of predicting NPPV failure in hypoxemic patients. In an observational study, the HACOR scale at 1 h from initiation of NPPV showed good predictive power for NIV failure compared with later assessments^(9)^. The first assessment should be performed at least 1 h later, with the performance of further assessments as necessary. |
| Monitoring and evaluation |
| After the implementation of these recommendations, further evaluation is needed, especially on the clinical effects and cost-effectiveness at each institution. In addition, it is desirable to monitor the implementation situation after the publication of the guideline to prevent other clinical problems. |
| Research priorities |
| Lung protective ventilation is commonly performed in mechanically ventilated patients with ARDS. In the two RCTs included in the current systematic review (2, 10), lung protective ventilation was not performed. Further studies comparing NPPV with IMV using lung protective ventilation are needed. |

References

1. Hashimoto S, Sanui M, Egi M, Ohshimo S, Shiotsuka J, Seo R, et al. The clinical practice guideline for the management of ARDS in Japan. J Intensive Care. 2017;5:50. PMID: 2877009.

2. Antonelli M, Conti G, Rocco M, Bufi M, De Blasi RA, Vivino G, et al. A comparison of non-invasive positive-pressure ventilation and conventional mechanical ventilation in patients with acute respiratory failure. N Engl J Med. 1998;339(7):429-35. PMID: 9700176.

3. Ferreyro BL, Angriman F, Munshi L, Del Sorbo L, Ferguson ND, Rochwerg B, et al. Association of noninvasive oxygenation strategies with all-cause mortality in adults with acute hypoxemic respiratory failure: a systematic review and meta-analysis. JAMA. 2020;324(1):57-67. PMID: 32496521

4. Carteaux G, Millán-Guilarte T, De Prost N, Razazi K, Abid S, Thille AW, et al. Failure of non-invasive ventilation for de novo acute hypoxemic respiratory failure: role of tidal volume. Crit Care Med. 2016;44(2):282-90. PMID: 26584191.

5. Egi M, Ogura H, Yatabe T, Atagi K, Inoue S, Iba T, et al. The Japanese clinical practice guidelines for management of sepsis and septic shock 2020 (J-SSCG 2020). J Intensive Care. 2021;9(1):53. PMID: 34433491.

6. Rhodes A, Evans LE, Alhazzani W, Levy MM, Antonelli M, Ferrer R, et al. Surviving sepsis campaign: international guidelines for management of sepsis and septic shock: 2016. Intensive Care Med. 2017;43(3):304-77. PMID: 28101605.

7. Rhodes A, Evans LE, Alhazzani W, Levy MM, Antonelli M, Ferrer R, et al. Surviving sepsis campaign: international guidelines for management of sepsis and septic shock: 2016. Crit Care Med. 2017;45(3):486-552. PMID: 28098591.

8. Rochwerg B, Brochard L, Elliott MW, Hess D, Hill NS, Nava S, et al. Official ERS/ATS clinical practice guidelines: non-invasive ventilation for acute respiratory failure. Eur Respir J. 2017;50(2):1602426. PMID: 33201321.

9. Duan J, Han X, Bai L, Zhou L, Huang S. Assessment of heart rate, acidosis, consciousness, oxygenation, and respiratory rate to predict non-invasive ventilation failure in hypoxemic patients. Intensive Care Med. 2017;43(2):192-9. PMID: 27812731.

10. Muncharaz AB, Bort MC, Asensio DB, Campos LM, Tegedor BV, Pérez JM, et al. Non-invasive ventilation versus invasive mechanical ventilation in patients with hypoxemic acute respiratory failure in an intensive care unit. a randomized controlled study. Minerva Pneumologica. 2017;56(1):1-10.

**CQ18 Should HFNC be used prior to conducting tracheal intubation in patients with ARDS?**

1.Search strategy

Short-term mortality

MEDLINE via PubMed (Search date: 2020/6/19)

| #1 | "Hypoxia"[mh] OR hypox*[tiab] OR "Respiratory Insufficiency"[mh] OR respiratory depression*[tiab] OR respiratory failure*[tiab] OR ventilatory depression*[tiab] OR respiratory insufficienc*[tiab] OR "Dyspnea"[mh] OR dyspnea*[tiab] OR "shortness of breath"[tiab] OR "Respiratory Distress Syndrome, Adult"[mh] OR acute respiratory distress[tiab] OR adult respiratory distress[tiab] OR respiratory distress syndrome*[tiab] OR RDS[tiab] OR ARDS[tiab] OR "Acute Lung Injury"[mh] OR acute lung injur*[tiab] OR ALI[tiab] |
| --- | --- |
| #2 | "Respiratory Distress Syndrome, Newborn"[mh] |
| #3 | #1 or #2 |
| #4 | "Noninvasive Ventilation"[mh] OR noninvasive ventilation*[tiab] OR non invasive ventilation*[tiab] OR NIV[tiab] OR NPPV[tiab] OR NIPPV[tiab] OR noninvasive positive pressure ventilation*[tiab] OR noninvasive mechanical ventilation*[tiab] OR noninvasive pressure support ventilation*[tiab] OR "Continuous Positive Airway Pressure"[mh] OR continuous positive airway pressure*[tiab] OR bilevel positive airway pressure*[tiab] OR biphasic positive airway pressure*[tiab] OR BIPAP[tiab] |
| #5 | "Oxygen Inhalation Therapy"[mh] OR HFNC[tiab] OR HHFNC[tiab] OR HHHFNC[tiab] OR HFNO[tiab] OR HFNT[tiab] OR HFNOT[tiab] OR HFO[tiab] OR HFOT[tiab] OR NHF[tiab] OR NHFC[tiab] OR NHFT[tiab] OR NHFO[tiab] OR NHFOT[tiab] OR high flow therap*[tiab] OR high flow oxygen[tiab] OR nasal high flow[tiab] |
| #6 | #4 or #5 |
| #7 | ("Randomized Controlled Trial"[pt] OR "Controlled Clinical Trial"[pt] OR "Clinical Trials as Topic"[mh] OR randomized[tiab] OR placebo[tiab] OR randomly[tiab] OR trial[tiab] OR groups[tiab]) NOT (Animals [mh] NOT Humans [mh]) |
| #8 | #3 and #6 and #7 |

CENTRAL (Search date: 2020/6/22)

| #1 | [mh Hypoxia] OR hypox*:ti,ab OR [mh "Respiratory Insufficiency"] OR "respiratory depression":ti,ab OR "respiratory failure":ti,ab OR "ventilatory depression":ti,ab OR "respiratory insufficiency":ti,ab OR [mh Dyspnea] OR dyspnea:ti,ab OR "shortness of breath":ti,ab OR [mh "Respiratory Distress Syndrome, Adult"] OR "acute respiratory distress":ti,ab OR "adult respiratory distress":ti,ab OR "respiratory distress syndrome":ti,ab OR RDS:ti,ab OR ARDS:ti,ab OR [mh "Acute Lung Injury"] OR "acute lung injury":ti,ab OR ALI:ti,ab |
| --- | --- |
| #2 | [mh "Respiratory Distress Syndrome, Newborn"] |
| #3 | #1 OR #2 |
| #4 | [mh "Noninvasive Ventilation"] OR "noninvasive ventilation":ti,ab OR "non invasive ventilation":ti,ab OR NIV:ti,ab OR NPPV:ti,ab OR NIPPV:ti,ab OR "noninvasive positive pressure ventilation":ti,ab OR "noninvasive mechanical ventilation":ti,ab OR "noninvasive pressure support ventilation":ti,ab OR [mh "Continuous Positive Airway Pressure"] OR "continuous positive airway pressure":ti,ab OR "bilevel positive airway pressure":ti,ab OR "biphasic positive airway pressure":ti,ab OR BIPAP:ti,ab |
| #5 | [mh "Oxygen Inhalation Therapy"] OR HFNC:ti,ab OR HHFNC:ti,ab OR HHHFNC:ti,ab OR HFNO:ti,ab OR HFNT:ti,ab OR HFNOT:ti,ab OR HFO:ti,ab OR HFOT:ti,ab OR NHF:ti,ab OR NHFC:ti,ab OR NHFT:ti,ab OR NHFO:ti,ab OR NHFOT:ti,ab OR "high flow therapy":ti,ab OR "high flow oxygen":ti,ab OR "nasal high flow":ti,ab |
| #6 | #4 OR #5 |
| #7 | #3 AND #6 |

Igaku-Chuo-Zasshi (Search date: 2020/6/19)

| #1 | 酸素欠乏/TH or 酸素欠乏/TA or anoxia/TA or Hypoxia/TA |
| --- | --- |
| #2 | 呼吸窮迫症候群-急性/TH or 急性呼吸窮迫症候群/TA or ARDS/TA |
| #3 | 急性肺損傷/TH or 急性肺損傷/TA or 急性肺障害/TA or 急性肺傷害/TA or ALI/TA |
| #4 | 呼吸不全/TH or 呼吸不全/TA |
| #5 | 呼吸困難/TH or 呼吸困難/TA |
| #6 | 呼吸窮迫症候群-新生児/TH or 新生児呼吸窮迫症候群/TA |
| #7 | #1 or #2 or #3 or #4 or #5 or #6 |
| #8 | 非侵襲的補助換気/TH or 非侵襲的補助換気/TA or NPPV/TA or NIPPV/TA |
| #9 | 持続気道陽圧/TH or 持続気道陽圧/TA or CPAP/TA |
| #10 | 非侵襲的陽圧呼吸/TH or 非侵襲的陽圧呼吸/TA or BIPAP/TA |
| #11 | 酸素吸入療法/TH or 酸素吸入/TA |
| #12 | 酸素療法/TA or ハイフロー/TA or HFNC/TA or NHF/TA or HFO/TA |
| #13 | #8 or #9 or #10 or #11 or #12 |
| #14 | #7 and #13 |
| #15 | (#14) and (PT=会議録除く) |
| #16 | ランダム化比較試験/TH or ランダム化/AL or 無作為化/AL |
| #17 | 比較試験/AL |
| #18 | 臨床試験/TH or 臨床試験/AL |
| #19 | プラセボ/TH or プラセボ/AL |
| #20 | 対照/AL |
| #21 | コントロール/AL |
| #22 | 臨床研究・疫学研究/TH or 臨床研究/AL |
| #23 | #16 or #17 or #18 or #19 or #20 or #21 or #22 |
| #24 | #15 and #23 |

Pneumonia

MEDLINE via PubMed（Search date: 2020/6/19）

|  | 検索式 |
| --- | --- |
| #1 | "Hypoxia"[mh] OR hypox*[tiab] OR "Respiratory Insufficiency"[mh] OR respiratory depression*[tiab] OR respiratory failure*[tiab] OR ventilatory depression*[tiab] OR respiratory insufficienc*[tiab] OR "Dyspnea"[mh] OR dyspnea*[tiab] OR "shortness of breath"[tiab] OR "Respiratory Distress Syndrome, Adult"[mh] OR acute respiratory distress[tiab] OR adult respiratory distress[tiab] OR respiratory distress syndrome*[tiab] OR RDS[tiab] OR ARDS[tiab] OR "Acute Lung Injury"[mh] OR acute lung injur*[tiab] OR ALI[tiab] |
| #2 | "Respiratory Distress Syndrome, Newborn"[mh] |
| #3 | #1 or #2 |
| #4 | "Noninvasive Ventilation"[mh] OR noninvasive ventilation*[tiab] OR non invasive ventilation*[tiab] OR NIV[tiab] OR NPPV[tiab] OR NIPPV[tiab] OR noninvasive positive pressure ventilation*[tiab] OR noninvasive mechanical ventilation*[tiab] OR noninvasive pressure support ventilation*[tiab] OR "Continuous Positive Airway Pressure"[mh] OR continuous positive airway pressure*[tiab] OR bilevel positive airway pressure*[tiab] OR biphasic positive airway pressure*[tiab] OR BIPAP[tiab] |
| #5 | "Oxygen Inhalation Therapy"[mh] OR HFNC[tiab] OR HHFNC[tiab] OR HHHFNC[tiab] OR HFNO[tiab] OR HFNT[tiab] OR HFNOT[tiab] OR HFO[tiab] OR HFOT[tiab] OR NHF[tiab] OR NHFC[tiab] OR NHFT[tiab] OR NHFO[tiab] OR NHFOT[tiab] OR high flow therap*[tiab] OR high flow oxygen[tiab] OR nasal high flow[tiab] |
| #6 | #4 or #5 |
| #7 | ("Randomized Controlled Trial"[pt] OR "Controlled Clinical Trial"[pt] OR "Clinical Trials as Topic"[mh] OR randomized[tiab] OR placebo[tiab] OR randomly[tiab] OR trial[tiab] OR groups[tiab]) NOT (Animals [mh] NOT Humans [mh]) |
| #8 | #3 and #6 and #7 |
| #9 | "Noninvasive Ventilation/adverse effects"[mh] OR noninvasive ventilation*[tiab] OR non invasive ventilation*[tiab] OR NIV[tiab] OR NPPV[tiab] OR NIPPV[tiab] OR noninvasive positive pressure ventilation*[tiab] OR noninvasive mechanical ventilation*[tiab] OR noninvasive pressure support ventilation*[tiab] OR "Continuous Positive Airway Pressure/adverse effects"[mh] OR continuous positive airway pressure*[tiab] OR bilevel positive airway pressure*[tiab] OR biphasic positive airway pressure*[tiab] OR BIPAP[tiab] |
| #10 | "Oxygen Inhalation Therapy/adverse effects"[mh] OR HFNC[tiab] OR HHFNC[tiab] OR HHHFNC[tiab] OR HFNO[tiab] OR HFNT[tiab] OR HFNOT[tiab] OR HFO[tiab] OR HFOT[tiab] OR NHF[tiab] OR NHFC[tiab] OR NHFT[tiab] OR NHFO[tiab] OR NHFOT[tiab] OR high flow therap*[tiab] OR high flow oxygen[tiab] OR nasal high flow[tiab] |
| #11 | #9 or #10 |
| #12 | harm*[tiab] OR side effect*[tiab] OR adverse[tiab] OR toxicity[tiab] OR infection*[tiab] OR pneumonia*[tiab] OR trauma[tiab] OR barotrauma*[tiab] OR volutrauma*[tiab] OR pneumothorax[tiab] OR ulcer*[tiab] OR discomfort*[tiab] OR breakdown[tiab] OR damage[tiab] OR intoleran*[tiab] OR "Barotrauma/complications"[mh] OR "Pneumonia/complications"[mh] OR "Pressure Ulcer/complications"[mh] OR "Pneumothorax/complications"[mh] OR "Pneumonia, Ventilator-Associated"[mh] |
| #13 | #3 and #11 and #12 |
| #14 | Animals [mh] NOT Humans [mh] |
| #15 | #13 not #14 |
| #16 | #15 not #8 |

CENTRAL（Search date: 2020/6/22）

|  | 検索式 |
| --- | --- |
| #1 | [mh Hypoxia] OR hypox*:ti,ab OR [mh "Respiratory Insufficiency"] OR "respiratory depression":ti,ab OR "respiratory failure":ti,ab OR "ventilatory depression":ti,ab OR "respiratory insufficiency":ti,ab OR [mh Dyspnea] OR dyspnea:ti,ab OR "shortness of breath":ti,ab OR [mh "Respiratory Distress Syndrome, Adult"] OR "acute respiratory distress":ti,ab OR "adult respiratory distress":ti,ab OR "respiratory distress syndrome":ti,ab OR RDS:ti,ab OR ARDS:ti,ab OR [mh "Acute Lung Injury"] OR "acute lung injury":ti,ab OR ALI:ti,ab |
| #2 | [mh "Respiratory Distress Syndrome, Newborn"] |
| #3 | #1 OR #2 |
| #4 | [mh "Noninvasive Ventilation"] OR "noninvasive ventilation":ti,ab OR "non invasive ventilation":ti,ab OR NIV:ti,ab OR NPPV:ti,ab OR NIPPV:ti,ab OR "noninvasive positive pressure ventilation":ti,ab OR "noninvasive mechanical ventilation":ti,ab OR "noninvasive pressure support ventilation":ti,ab OR [mh "Continuous Positive Airway Pressure"] OR "continuous positive airway pressure":ti,ab OR "bilevel positive airway pressure":ti,ab OR "biphasic positive airway pressure":ti,ab OR BIPAP:ti,ab |
| #5 | [mh "Oxygen Inhalation Therapy"] OR HFNC:ti,ab OR HHFNC:ti,ab OR HHHFNC:ti,ab OR HFNO:ti,ab OR HFNT:ti,ab OR HFNOT:ti,ab OR HFO:ti,ab OR HFOT:ti,ab OR NHF:ti,ab OR NHFC:ti,ab OR NHFT:ti,ab OR NHFO:ti,ab OR NHFOT:ti,ab OR "high flow therapy":ti,ab OR "high flow oxygen":ti,ab OR "nasal high flow":ti,ab |
| #6 | #4 OR #5 |
| #7 | #3 AND #6 |
| #8 | [mh "Noninvasive Ventilation"/ae] OR "noninvasive ventilation":ti,ab OR "non invasive ventilation":ti,ab OR NIV:ti,ab OR NPPV:ti,ab OR NIPPV:ti,ab OR "noninvasive positive pressure ventilation":ti,ab OR "noninvasive mechanical ventilation":ti,ab OR "noninvasive pressure support ventilation":ti,ab OR [mh "Continuous Positive Airway Pressure"/ae] OR "continuous positive airway pressure":ti,ab OR "bilevel positive airway pressure":ti,ab OR "biphasic positive airway pressure":ti,ab OR BIPAP:ti,ab |
| #9 | [mh "Oxygen Inhalation Therapy"/ae] OR HFNC:ti,ab OR HHFNC:ti,ab OR HHHFNC:ti,ab OR HFNO:ti,ab OR HFNT:ti,ab OR HFNOT:ti,ab OR HFO:ti,ab OR HFOT:ti,ab OR NHF:ti,ab OR NHFC:ti,ab OR NHFT:ti,ab OR NHFO:ti,ab OR NHFOT:ti,ab OR "high flow therapy":ti,ab OR "high flow oxygen":ti,ab OR "nasal high flow":ti,ab |
| #10 | #8 OR #9 |
| #11 | harm*:ti,ab OR "side effect":ti,ab OR adverse:ti,ab OR toxicity:ti,ab OR infection*:ti,ab OR pneumonia*:ti,ab OR trauma:ti,ab OR barotrauma*:ti,ab OR volutrauma*:ti,ab OR pneumothorax:ti,ab OR ulcer*:ti,ab OR discomfort*:ti,ab OR breakdown:ti,ab OR damage:ti,ab OR intoleran*:ti,ab OR [mh Barotrauma/co] OR [mh Pneumonia/co] OR [mh "Pressure Ulcer"/co] OR [mh Pneumothorax/co] OR [mh "Pneumonia, Ventilator-Associated"] |
| #12 | #3 AND #10 AND #11 |
| #13 | #12 NOT #7 |

Igaku-Chuo-Zasshi （Search date: 2020/6/19）

|  | 検索式 |
| --- | --- |
| #1 | 酸素欠乏/TH or 酸素欠乏/TA or anoxia/TA or Hypoxia/TA |
| #2 | 呼吸窮迫症候群-急性/TH or 急性呼吸窮迫症候群/TA or ARDS/TA |
| #3 | 急性肺損傷/TH or 急性肺損傷/TA or 急性肺障害/TA or 急性肺傷害/TA or ALI/TA |
| #4 | 呼吸不全/TH or 呼吸不全/TA |
| #5 | 呼吸困難/TH or 呼吸困難/TA |
| #6 | 呼吸窮迫症候群-新生児/TH or 新生児呼吸窮迫症候群/TA |
| #7 | #1 or #2 or #3 or #4 or #5 or #6 |
| #8 | 非侵襲的補助換気/TH or 非侵襲的補助換気/TA or NPPV/TA or NIPPV/TA |
| #9 | 持続気道陽圧/TH or 持続気道陽圧/TA or CPAP/TA |
| #10 | 非侵襲的陽圧呼吸/TH or 非侵襲的陽圧呼吸/TA or BIPAP/TA |
| #11 | 酸素吸入療法/TH or 酸素吸入/TA |
| #12 | 酸素療法/TA or ハイフロー/TA or HFNC/TA or NHF/TA or HFO/TA |
| #13 | #8 or #9 or #10 or #11 or #12 |
| #14 | #7 and #13 |
| #15 | (#14) and (PT=会議録除く) |
| #16 | ランダム化比較試験/TH or ランダム化/AL or 無作為化/AL |
| #17 | 比較試験/AL |
| #18 | 臨床試験/TH or 臨床試験/AL |
| #19 | プラセボ/TH or プラセボ/AL |
| #20 | 対照/AL |
| #21 | コントロール/AL |
| #22 | 臨床研究・疫学研究/TH or 臨床研究/AL |
| #23 | #16 or #17 or #18 or #19 or #20 or #21 or #22 |
| #24 | #15 and #23 |
| #25 | ((非侵襲的補助換気/TH) and (SH=有害作用)) or 非侵襲的補助換気/TA or NPPV/TA or NIPPV/TA |
| #26 | ((持続気道陽圧/TH) and (SH=有害作用)) or 持続気道陽圧/TA or CPAP/TA |
| #27 | ((非侵襲的陽圧呼吸/TH) and (SH=有害作用)) or 非侵襲的陽圧呼吸/TA or BIPAP/TA |
| #28 | ((酸素吸入療法/TH) and (SH=有害作用)) or 酸素吸入/TA |
| #29 | 酸素療法/TA or ハイフロー/TA or HFNC/TA or NHF/TA or HFO/TA |
| #30 | #25 or #26 or #27 or #28 or #29 |
| #31 | 害/TA or 副作用/TA or 毒性/TA or 感染/TA or 肺炎/TA or 外傷/TA or トラウマ/TA or 気胸/TA or 潰瘍/TA or 不快感/TA or 損傷/TA or 気圧障害/TA or ((気圧障害/TH) and (SH=合併症)) or ((肺炎/TH) and (SH=合併症)) or ((褥瘡性潰瘍/TH) and (SH=合併症)) or ((気胸/TH) and (SH=合併症)) or 人工呼吸器関連肺炎/TH |
| #32 | #7 and #30 and #31 |
| #33 | (#32) and (PT=会議録除く) |
| #34 | #43 not #24 |

1. Flow diagram

Short-term mortality

**Identification**

21 Studies included in qualitative synthesis

6482 records after duplicates removed

7771 records identified through database searching

7771 records identified through database searching

Medline via PubMed (n=3403)

Cochrane CENTRAL (n=3924)

Igaku-Chuo-Zasshi (n=444)

0 additional records identified through other sources

19 Studies included in quantitative synthesis (meta-analysis)

Duplicates

n=1289

6374 records excluded

**Included**

**Eligibility**

**Screening**

87 Full-text articles excluded, with reasons:

・Wrong language (n=4)

・Wrong study design (n=50)

・Wrong population (n=27)

・Wrong intervention (n=4)

・Duplicates (n=1)

・Difficult to obtain full text (n=1)

Etc.

108 Full-text articles assessed for eligibility

Pneumonia

**Identification**

21 Studies included in qualitative synthesis

9104 records after duplicates removed

10401 records identified through database searching

2630 records identified through database searching

Medline via PubMed (n=1442)

Cochrane CENTRAL (n=0)

Igaku-Chuo-Zasshi (n=1188)

7771 additional records identified through other sources

10 Studies included in quantitative synthesis (meta-analysis)

Duplicates

n=1297

8976 records excluded

**Included**

**Eligibility**

**Screening**

1. Risk of bias

There were no RCTs that directly compared the data, so indirect evidence was used.

1. Forest plot

There were no RCTs that directly compared the data, so indirect evidence was used.

1. Evidence profile

Details of assessments of certainty of estimates from NMA

1. Short-term mortality

| **Comparison** | **NPPV vs. COT** | **HFNC vs. COT** | **IMV vs. COT** | **NPPV vs. IMV** | **HFNC vs. IMV** | **NPPV vs. HFNC** |  |
| --- | --- | --- | --- | --- | --- | --- | --- |
| **Direct evidence** | | | | | | | |
| Risk of bias | Not serious | Not serious |  | Not serious |  | Not serious |  |
| Inconsistency | Serious^a^ | Serious^a^ |  | Serious^a^ |  | Not serious |  |
| Indirectness | Not serious | Not serious |  | Serious^b^ |  | Not serious |  |
| Publication bias | Undetected | Undetected |  | Undetected |  | Undetected |  |
| Preliminary rating direct | **Moderate** | **Moderate** |  | **Low** |  | **High** |  |
| Contributes as much as indirect | No | Yes |  | Yes |  | Yes |  |
| Need to assess indirect | Yes | Yes |  | Yes |  | Yes |  |
| Imprecision | Not serious | Serious^c^ |  | Very serious^c^ |  | Very serious^c, d^ |  |
| Final direct rating | **Moderate** | **Low** |  | **Very low** |  | **Low** |  |
| **Indirect evidence** | | | | | | | |
| Common comparator | HFNC | NPPV | NPPV |  | NPPV | COT |  |
| Intervention 1 vs. common comparator rating | **High** | **High** | **Low** |  | **High** | **Moderate** |  |
| Intervention 2 vs. common comparator rating | **Moderate** | **Moderate** | **Moderate** |  | **Low** | **Moderate** |  |
| Lowest of the two | **Moderate** | **Moderate** | **Low** |  | **Low** | **Moderate** |  |
| Intransitivity | Not serious | Not serious | Not serious |  | Not serious | Not serious |  |
| Preliminary rating indirect | **Moderate** | **Moderate** | **Low** |  | **Low** | **Moderate** |  |
| Imprecision | Very serious^c^ | Not serious | Very serious^c^ |  | Very serious^c^ | Very serious^c^ |  |
| Final indirect rating | **Very low** | **Moderate** | **Very low** |  | **Very low** | **Very low** |  |
| **Network evidence** | | | | | | | |
| Highest between direct and indirect | **Moderate** | **Moderate** | **Low** | **Low** | **Low** | **High** |  |
| Incoherence | Not serious | Serious^e^ | NA | NA | NA | Serious^e^ |  |
| Imprecision | Not serious | Serious^c^ | Very serious^c^ | Very serious^c^ | Very serious^c^ | Very serious^c, d^ |  |
| Final network rating | **Moderate** | **Very low** | **Very low** | **Very low** | **Very low** | **Very low** |  |
| Most credible estimate | **Direct** | **Network** | **Network** | **Network** | **Network** | **Network** |  |

a: Due to high I^2^.

b: In the included two RCTs, lung protective ventilation was not performed.

c: Confidence intervals contained both substantial benefit and harm.

d: Meta-analysis using only one RCT.

e: Difference between confidence intervals of direct and indirect estimates.

COT, conventional oxygen therapy; HFNC, high flow nasal cannula; IMV, invasive mechanical ventilation; NA, not applicable; NPPV, noninvasive positive pressure ventilation; RCT, randomized controlled trial.

1. Endotracheal intubation

| **Comparison** | **NPPV vs. COT** | **HFNC vs. COT** | **NPPV vs. HFNC** |
| --- | --- | --- | --- |
| **Direct evidence** | | | |
| Risk of bias | Not serious | Not serious | Not serious |
| Inconsistency | Serious^a^ | Not serious | Not serious |
| Indirectness | Not serious | Not serious | Not serious |
| Publication bias | Undetected | Undetected | Undetected |
| Preliminary rating direct | **Moderate** | **High** | **High** |
| Contributes as much as indirect | No | Yes | Yes |
| Need to assess indirect | Yes | Yes | Yes |
| Imprecision | Not serious | Very serious^b^ | Very serious^c^ |
| Final direct rating | **Moderate** | **Low** | **Low** |
| **Indirect evidence** | | | |
| Common comparator | HFNC | NPPV | COT |
| Intervention 1 vs. common comparator rating | **High** | **High** | **Moderate** |
| Intervention 2 vs. common comparator rating | **High** | **Moderate** | **High** |
| Lowest of the two | **High** | **Moderate** | **Moderate** |
| Intransitivity | Not serious | Not serious | Not serious |
| Preliminary rating indirect | **High** | **Moderate** | **Moderate** |
| Imprecision | Very serious^b^ | Not serious | Serious^c^ |
| Final indirect rating | **Low** | **Moderate** | **Low** |
| **Network evidence** | | | |
| Highest between direct and indirect | **High** | **High** | **High** |
| Incoherence | Serious^c^ | Not serious | Serious^d^ |
| Imprecision | Not serious | Very serious^b^ | Very serious^b^ |
| Final network rating | **Moderate** | **Low** | **Very low** |
| Most credible estimate | **Direct** | **Network** | **Network** |

a: Due to high I^2^.

b: Confidence intervals contained both substantial benefit and harm.

c: Confidence intervals contained substantial benefit and no benefit.

d: Difference between confidence intervals of direct and indirect estimates.

COT, conventional oxygen therapy; HFNC, high flow nasal cannula; NPPV, noninvasive positive pressure ventilation.

1. Pneumonia

| **Comparison** | **NPPV vs. COT** | **HFNC vs. COT** | **IMV vs. COT** | **NPPV vs. IMV** | **HFNC vs. IMV** | **NPPV vs. HFNC** |  |
| --- | --- | --- | --- | --- | --- | --- | --- |
| **Direct evidence** | | | | | | | |
| Risk of bias | Serious^a^ | Serious^a^ |  | Serious^a^ |  | Serious^a^ |  |
| Inconsistency | Not serious | Not serious |  | Not serious |  | Not serious |  |
| Indirectness | Not serious | Not serious |  | Not serious |  | Not serious |  |
| Publication bias | Undetected | Undetected |  | Undetected |  | Undetected |  |
| Preliminary rating direct | **Moderate** | **Moderate** |  | **Moderate** |  | **Moderate** |  |
| Contributes as much as indirect | No | Yes |  | Yes |  | Yes |  |
| Need to assess indirect | Yes | Yes |  | Yes |  | Yes |  |
| Imprecision | Not serious | Very serious^b^ |  | Not serious |  | Very serious^b^ |  |
| Final direct rating | **Moderate** | **Very low** |  | **Moderate** |  | **Very low** |  |
| **Indirect evidence** | | | | | | | |
| Common comparator |  | NPPV | NPPV |  | NPPV | COT |  |
| Intervention 1 vs. common comparator rating |  | **Moderate** | **Moderate** |  | **Moderate** | **Moderate** |  |
| Intervention 2 vs. common comparator rating |  | **Moderate** | **Moderate** |  | **Moderate** | **Moderate** |  |
| Lowest of the two |  | **Moderate** | **Moderate** |  | **Moderate** | **Moderate** |  |
| Intransitivity |  | Not serious | Not serious |  | Not serious | Not serious |  |
| Preliminary rating indirect |  | **Moderate** | **Moderate** |  | **Moderate** | **Moderate** |  |
| Imprecision |  | Very serious^b^ | Not serious |  | Not serious | Very serious^b^ |  |
| Final indirect rating |  | **Very low** | **Moderate** |  | **Moderate** | **Very low** |  |
| **Network evidence** | | | | | | | |
| Highest between direct and indirect | **Moderate** | **Moderate** | **Moderate** | **Moderate** | **Moderate** | **Moderate** |  |
| Incoherence | NA | Not serious | NA | NA | NA | Serious^c^ |  |
| Imprecision | Not serious | Not serious | Not serious | Not serious | Not serious | Very serious^b^ |  |
| Final network rating | **Moderate** | **Moderate** | **Moderate** | **Moderate** | **Moderate** | **Very low** |  |
| Most credible estimate | **Network** | **Network** | **Network** | **Network** | **Network** | **Network** |  |

a: Intervention was not blinded, and definitions of pneumonia was unclear.

b: Confidence intervals contain both substantial benefit and harm.

c: Difference between confidence intervals of direct and indirect estimates.

COT, conventional oxygen therapy; HFNC, high flow nasal cannula; IMV, invasive mechanical ventilation; NA, not applicable; NPPV, noninvasive positive pressure ventilation; RCT, randomized controlled trial.

Results and certainty assessments for the outcome

1. Short-term mortality

| Comparison | Direct estimate (RR 95% CI) |  | Indirect estimate (RR 95% CI) | Rating | Network estimate (RR 95% CI) | Rating |
| --- | --- | --- | --- | --- | --- | --- |
| NPPV vs. COT | 0.71  (0.55−0.92) | ⨁⨁⨁◯ Moderate | 3.82  (0.58−25.05) | ⨁〇〇◯ Very low | 0.71 *  (0.55−0.92) | ⨁⨁⨁◯ Moderate |
| HFNC vs. COT | 0.79  (0.47−1.32) | ⨁⨁〇◯ Low | 0.13  (0.02−0.74) | ⨁⨁⨁◯ Moderate | 0.68  (0.41−1.12) | ⨁〇〇◯ Very low |
| IMV vs. COT | NA | - | 0.79  (0.40–1.57) | ⨁〇〇◯ Very low | 0.79  (0.40−1.57) | ⨁〇〇◯ Very low |
| NPPV vs. IMV | 0.92  (0.49−1.75) | ⨁〇〇◯ Very low | NA | - | 0.92  (0.40−1.75) | ⨁〇〇◯ Very low |
| HFNC vs. IMV | NA | - | 0.86  (0.37−1.97) | ⨁〇〇◯ Very low | 0.86  (0.37−1.97) | ⨁〇〇◯ Very low |
| NPPV vs. HFNC | 2.30  (0.97−5.44) | ⨁⨁〇◯ Low | 0.67  (0.34−1.32) | ⨁⨁〇◯ Low | 1.07  (0.63−1.83) | ⨁〇〇◯ Very low |

CI, confidence interval; COT, conventional oxygen therapy; HFNC, high flow nasal cannula; IMV, invasive mechanical ventilation; NA, not applicable; NPPV, noninvasive positive pressure ventilation; RR, risk ratio

* We adopted effect estimates based on direct evidence, because direct evidence was dominant for the network estimates.

2. Endotracheal intubation

| Comparison | Direct estimate (RR 95% CI) |  | Indirect estimate (RR 95% CI) | Rating | Network estimate (RR 95% CI) | Rating |
| --- | --- | --- | --- | --- | --- | --- |
| NPPV vs. COT | 0.66  (0.52−0.82) | ⨁⨁⨁◯ Moderate | 1.42  (0.32−6.33) | ⨁⨁〇◯ Low | 0.66 *  (0.52−0.82) | ⨁⨁⨁◯ Moderate |
| HFNC vs. COT | 0.88  (0.58−1.32) | ⨁⨁〇◯ Low | 0.25  (0.07−0.84) | ⨁⨁⨁◯ Moderate | 0.77  (0.52−1.32) | ⨁⨁〇◯ Low |
| NPPV vs. HFNC | 1.33  (0.71−2.48) | ⨁⨁〇◯ Low | 0.61  (0.35 −1.07) | ⨁⨁〇◯ Low | 0.86  (0.57−1.31) | ⨁〇〇◯ Very low |

CI, confidence interval; COT, conventional oxygen therapy; HFNC, high flow nasal cannula; NPPV, noninvasive positive pressure ventilation; RR, risk ratio.

* We adopted effect estimates based on direct evidence, because direct evidence was dominant for the network estimates.

1. Pneumonia

| Comparison | Direct estimate (RR 95% CI) |  | Indirect estimate (RR 95% CI) | Rating | Network estimate (RR 95% CI) | Rating |
| --- | --- | --- | --- | --- | --- | --- |
| NPPV vs. COT | 0.61  (0.40−0.92) | ⨁⨁⨁◯ Moderate | NA | - | 0.61  (0.40−0.92) | ⨁⨁⨁◯ Moderate |
| HFNC vs. COT | 0.44  (0.14−1.43) | ⨁〇〇◯ Very low | 0.07  (0.004−1.42) | ⨁〇〇◯ Very low | 0.35  (0.12−1.03) | ⨁⨁⨁◯ Moderate |
| IMV vs. COT | NA | - | 3.28  (1.37−7.88) | ⨁⨁⨁◯ Moderate | 3.28  (1.37−7.88) | ⨁⨁⨁◯ Moderate |
| NPPV vs. IMV | 0.19  (0.09−0.40) | ⨁⨁⨁◯ Moderate | NA | - | 0.19  (0.09−0.40) | ⨁⨁⨁◯ Moderate |
| HFNC vs. IMV | NA | - | 0.11  (0.03−0.40) | ⨁⨁⨁◯ Moderate | 0.11  (0.03−0.40) | ⨁⨁⨁◯ Moderate |
| NPPV vs. HFNC | 2.17  (0.69−6.85) | ⨁〇〇◯ Very low | 0.31  (0.01−7.94) | ⨁〇〇◯ Very low | 1.75  (0.59−5.16) | ⨁〇〇◯ Very low |

CI, confidence interval; COT, conventional oxygen therapy; HFNC, high flow nasal cannula; IMV, invasive mechanical ventilation; NA, not applicable; NPPV, noninvasive positive pressure ventilation; RR, risk ratio.

1. Evidence-to-Decision table

| Question | |
| --- | --- |
| **CQ18: Should HFNC be used prior to conducting tracheal intubation in patients with ARDS?** | |
| **Population:** | Adult patients aged ≥18 years who had acute hypoxemic respiratory failure defined by new-onset of clinical signs (e.g., tachypnea, increased work of breathing), radiologic signs (unilateral or bilateral chest radiograph opacities), and hypoxemia.  We excluded the randomized controlled trials (RCTs) that included more than half of patients with hypercapnia, congestive heart failure, COPD, or asthma as the cause of respiratory failure, post-extubation respiratory failure, post-surgical, and post-trauma constituting. |
| **Intervention** | High-flow nasal cannula (HFNC) |
| **Comparison:** | Invasive mechanical ventilation (IMV) |
| **Main outcomes:** | Short-term mortality, Pneumonia |
| **Settings:** | Emergency department or intensive care unit |
| **Perspective:** | Individual |
| **Background:** | HFNC is used to avoid complications from tracheal intubation in patients with acute hypoxemic respiratory failure, but delayed intubation may increase the risk of death.  HFNC is not established to manage patients with ARDS. It is an important clinical issue to clarify whether HFNC is more effective than IMV. |
| **Conflict of interests:** | None |

# Assessment

| Problem Is the problem a priority? | | |
| --- | --- | --- |
| Judgment | Research evidence | Additional considerations |
| ○ No  ○ Probably no  ● Probably yes  ○ Yes  ○ Varies  ○ Do not know | In a multicenter RCT that examined the effectiveness of noninvasive respiratory supports in patients with hypoxemic respiratory failure^(1)^, HFNC reduced the incidence of endotracheal intubation and short-term mortality compared with NPPV and COT. However, other RCTs and meta-analyses have not demonstrated the same efficacy of HFNC^(2)^.  Since the effects of HFNC have not been consistent in previous studies, this clinical issue should probably be given high priority. |  |
| Desirable effects How substantial are the desirable anticipated effects? | | |
| Judgment | Research evidence | Additional considerations |
| ○ Trivial  ○ Small  ● Moderate  ○ Large  ○ Varies  ○ Do not know | The search strategy identified 12,620 records, including 19 RCTs and 6 observational trials that were eligible for inclusion. Observational studies were excluded for meta-analyses due to serious concern for a high risk of bias. We performed network meta-analyses using 19 RCTs (N=2,777). We used indirect evidence because no trials made direct comparisons between HFNC and IMV.  In comparing HFNC and IMV, the estimated value of the effects of short-term mortality was 56 fewer per 1,000 (95% CI: 248 fewer to 386 more), and pneumonia was 440 fewer per 1,000 (95% CI: 478 fewer to 295 fewer).  Based on the above, the desirable effects were considered “moderate.” |  |
| Undesirable effects How substantial are the undesirable anticipated effects? | | |
| Judgment | Research evidence | Additional considerations |
| ○ Large  ○ Moderate  ○ Small  ● Trivial  ○ Varies  ○ Do not know | The risk of aspiration during HFNC is unclear. We regarded pneumonia as an outcome of harm in this systematic review. Since the occurrence of pneumonia was reduced by HFNC, pneumonia was not an undesirable effect of HFNC.  Considering the description in the additional considerations, the undesirable effects of HFNC were considered to be “trivial.” | The positive pressure provided by HFNC (set at 60 L/min) is approximately 4 cmH2O^(3)^, which is lower than that of IMV. The risk of barotrauma seems to be lower than IMV. |
| Certainty of evidence What is the overall certainty of the evidence of effects? | | |
| Judgment | Research evidence | Additional considerations |
| ○ Very low  ○ Low  ● Moderate  ○ High  ○ No included studies | **Importance and value of the main outcomes**   \| Outcome \| Importance \| Certainty of the evidence \| \| --- \| --- \| --- \| \| Short-term mortality \| Critical \| ⨁◯◯◯ \| \| Very low \| \| Pneumonia \| Critical \| ⨁⨁⨁◯ \| \| Moderate \|   **Overall certainty of the evidence**  Because the directions of the point estimates were consistent between both outcomes, the certainty of the evidence was considered to be “moderate” based on the highest certainty of evidence. |  |
| Values Is there important uncertainty about or variability in how much people value the main outcomes? | | |
| Judgment | Research evidence | Additional considerations |
| ○ Important uncertainty or variability  ○ Possibly important uncertainty or variability  ● Probably no important uncertainty or variability  ○ No important uncertainty or variability | We found no data on patient and family values in this systematic review. In general, the values of the main outcomes were expected to be high and consistent. |  |
| Balance of effects Does the balance between desirable and undesirable effects favor the intervention or the comparison? | | |
| Judgment | Research evidence | Additional considerations |
| ○ Favors the comparison  ○ Probably favors the comparison  ○ Does not favor either the intervention or the comparison  ● Probably favors the intervention  ○ Favors the intervention  ○ Varies  ○ Do not know | **Summary of evidence:**   \| Outcome \| IMV (comparison) \| HFNC (intervention) \| Risk difference (RD)  (95% CI) \| Risk ratio (RR) (95% CI) \| \| --- \| --- \| --- \| --- \| --- \| \| Short-term mortality \| NA \| NA \| 56 fewer /1,000 (248 fewer～386 more) \| 0.86 (0.37〜1.97) \| \| Pneumonia \| NA \| NA \| 440 fewer /1,000 (478 fewer～295 fewer) \| 0.11 (0.03〜0.40) \|   NA, not applicable (based on indirect comparison)  Based on the above, the balance of the effects of HFNC was considered to be “probably favors the intervention.” |  |
| Acceptability Is the intervention acceptable to key stakeholders? | | |
| Judgment | Research evidence | Additional considerations |
| ○ No  ○ Probably no  ● Probably yes  ○ Yes  ○ Varies  ○ Do not know | Acceptability was considered “probably yes” because HFNC has already been performed to manage patients with respiratory failure. Furthermore, undesirable effects are rare using HFNC. Although HFNC is more expensive than COT, the potential benefit of avoiding endotracheal intubation may reduce the total cost. |  |
| Feasibility Is the intervention feasible to implement? | | |
| Judgment | Research evidence | Additional considerations |
| ○ No  ○ Probably no  ● Probably yes  ○ Yes  ○ Varies  ○ Do not know | Feasibility was considered “probably yes” because HFNC has already been performed to manage patients with respiratory failure.  If used on many patients at the same time, it is important to ensure that enough oxygen has been stored. |  |

# Summary of Evidence

|  | **JUDGMENT** | | | | | | |
| --- | --- | --- | --- | --- | --- | --- | --- |
| **PROBLEM** | No | Probably no | **Probably yes** | Yes |  | Varies | Unknown |
| **DESIRABLE EFFECTS** | Trivial | Small | **Moderate** | Large |  | Varies | Unknown |
| **UNDESIRABLE EFFECTS** | Large | Moderate | Small | **Trivial** |  | Varies | Unknown |
| **CERTAINTY OF EVIDENCE** | Very low | Low | **Moderate** | High |  |  | No included studies |
| **VALUES** | Important uncertainty or variability | Possibly important uncertainty or variability | **Probably no important uncertainty or variability** | No important uncertainty or variability |  |  |  |
| **BALANCE OF EFFECTS** | Favors the comparison | Probably favors the comparison | Does not favor either the intervention or the comparison | **Probably favors the intervention** | Favors the intervention | Varies | Unknown |
| **ACCEPTABILITY** | No | Probably no | **Probably yes** | Yes |  | Varies | Unknown |
| **FEASIBILITY** | No | Probably no | **Probably yes** | Yes |  | Varies | Unknown |

# Type of Recommendation

| Strong recommendation against the intervention | Conditional recommendation against the intervention | Conditional recommendation for either the intervention or the comparison | Conditional recommendation for the intervention | Strong recommendation for the intervention |
| --- | --- | --- | --- | --- |
| ○ | ○ | ○ | ● | ○ |

# Conclusion

| Recommendation |
| --- |
| **If the patient has no contraindications for HFNC and no organ failure other than respiratory failure, we suggest using HFNC for adult patients with acute hypoxemic respiratory failure who have probable ARDS compared with IMV, as initial respiratory management (Conditional recommendation, moderate certainty of evidence: GRADE 2B).**  **Supplementary condition:**  **Careful observation is needed after the initiation of HFNC to avoid delayed intubation which may contribute to poor outcomes.**  **Contraindications for HFNC include the inability to protect the airway, high risk of vomiting, deterioration of consciousness, agitation, and unstable hemodynamics.** |
| Justification |
| **Question:** Should HFNC be used prior to conducting tracheal intubation in patients with ARDS?  **Population:** Adult patients with acute hypoxemic respiratory failure  **Intervention:** HFNC  **Main outcomes:** Short-term mortality, Pneumonia  **Summary of evidence:**  We performed network meta-analyses using 19 RCTs (N=2,777). We used indirect evidence because no trials directly compared HFNC and IMV. In comparing HFNC and IMV, the estimated value of the effects of short-term mortality was 56 fewer per 1,000 (95% CI: 248 fewer to 386 more), and pneumonia was 440 fewer per 1,000 (95% CI: 478 fewer to 295 fewer). Therefore, the desirable effects were considered to be “moderate.” The positive pressure provided by HFNC (set at 60 L/min) is approximately 4 cmH_2_O^(3)^, which is lower than that of IMV. The risk of barotrauma seems to be lower than IMV. We did not identify any significant undesirable effects of using HFNC in this systematic review. We judged that the undesirable effects of HFNC were “trivial.”  **Certainty of evidence:**  Because the directions of the point estimates were consistent among all outcomes, the certainty of the evidence was considered to be “moderate” based on the highest certainty of evidence. However, the approach to assess imprecision in a network meta-analysis has not been established. We thought that the current network meta-analysis did not have a large enough sample size. If we rated down for imprecision, the certainty of evidence would be “low.”  **Values, Balance of effects, Acceptability, Feasibility:**  In general, the values of the main outcomes were expected to be high and consistent. The balance of the effects of HFNC was considered to be “probably favors the intervention.” Acceptability and feasibility were considered to be “probably yes” because HFNC has already been performed to manage patients with respiratory failure.  **Panel meeting:**  The modified Delphi method was used to form a consensus. Prior to the discussion conference, we sent draft recommendations and materials prepared by the systematic reviewers to all panelists. As a result of voting, the median value of agreement was 8.0, and the disagreement index was 0.19 for the use of HFNC for adult patients with ARDS before intubation.  In the discussion at the panel meeting, it was suggested to change the target population from ARDS to acute hypoxemic respiratory failure because we cannot diagnose ARDS without positive end-expiratory pressure based on the Berlin definition. Since some panelists raised the concern for imprecision due to no direct evidence and the small sample size for indirect evidence, additional explanation is noted in the section of certainty of evidence.  After extensive discussion among the panelists, the current recommendation “We suggest using HFNC for adult patients with acute hypoxemic respiratory failure who have probable ARDS compared with IMV, as an initial respiratory management.” was approved (median value of agreement was 8.0, and disagreement index was 0.22). |
| Subgroup considerations |
| We did not perform sensitivity analyses. |
| Implementation considerations |
| In the previous clinical practice guideline for the management of ARDS in Japan (4), HFNC was not discussed. However, it was noted that investigations on the use of HFNC should be conducted in the future.  The Japanese Clinical Practice Guidelines for Management of Sepsis and Septic Shock 2020 (5) suggested the use of NPPV and HFNC for early respiratory failure in adult patients with sepsis. In the International Guidelines for Management of Sepsis and Septic Shock^(6, 7)^, no discussion regarding the use or not of HFNC in patients with ARDS was found.  A clinical practice guideline for using HFNC created by a working group of the European Society of Intensive Care Medicine ^(8)^ recommends the use of HFNC instead of COT for patients with hypoxemic respiratory failure. Since panel members differed on whether to give a strong or conditional recommendation, a conditional recommendation was given after voting. The rationale in favor of a conditional recommendation was that there could be an increased risk of delayed intubation and inconsistent efficacy across populations, including the cause and severity of the respiratory failure.  If HFNC is used for patients with hypoxemic respiratory failure, including ARDS, close monitoring is needed to avoid delayed intubation. One of the clinical prediction rules is the ROX index, defined as the ratio of oxygen saturation as measured by pulse oximetry / F_I_O_2_ to respiratory rate^(9)^. |
| Monitoring and evaluation |
| After the implementation of the recommendations, further evaluation is needed, especially on the clinical effects and cost-effectiveness at each institution. In addition, it is desirable to monitor the implementation situation after the publication of the guideline to prevent other clinical problems. |
| Research priorities |
| An RCT that directly compares HFNC and MV in patients with acute hypoxemic respiratory failure is needed to provide more robust evidence. |

References

1. Frat JP, Thille AW, Mercat A, Girault C, Ragot S, Perbet S, et al. High-flow oxygen through nasal cannula in acute hypoxemic respiratory failure. N Engl J Med. 2015;372(23):2185-96. PMID: 25981908.

2. Ferreyro BL, Angriman F, Munshi L, Del Sorbo L, Ferguson ND, Rochwerg B, et al. Association of noninvasive oxygenation strategies with all-cause mortality in adults with acute hypoxemic respiratory failure: a systematic review and meta-analysis. JAMA. 2020;324(1):57-67. PMID: 32496521

3. Parke RL, Eccleston ML, McGuinness SP. The effects of flow on airway pressure during nasal high-flow oxygen therapy. Respir Care. 2011;56(8):1151-5. PMID: 21496369

4. Hashimoto S, Sanui M, Egi M, Ohshimo S, Shiotsuka J, Seo R, et al. The clinical practice guideline for the management of ARDS in Japan. J Intensive Care. 2017;5:50. PMID: 28770093

5. Egi M, Ogura H, Yatabe T, Atagi K, Inoue S, Iba T, et al. The Japanese clinical practice guidelines for management of sepsis and septic shock 2020 (J-SSCG 2020). J Intensive Care. 2021;9(1):53. PMID: 34433491

6. Rhodes A, Evans LE, Alhazzani W, Levy MM, Antonelli M, Ferrer R, et al. Surviving sepsis campaign: international guidelines for management of sepsis and septic shock: 2016. Intensive Care Med. 2017;43(3):304-77. PMID: 28101605

7. Rhodes A, Evans LE, Alhazzani W, Levy MM, Antonelli M, Ferrer R, et al. Surviving sepsis campaign: international guidelines for management of sepsis and septic shock: 2016. Crit Care Med. 2017;45(3):486-552. PMID: 28098591

8. Rochwerg B, Einav S, Chaudhuri D, Mancebo J, Mauri T, Helviz Y, et al. The role for high flow nasal cannula as a respiratory support strategy in adults: a clinical practice guideline. Intensive Care Med. 2020;46(12):2226-37. PMID: 33201321

9. Roca O, Caralt B, Messika J, Samper M, Sztrymf B, Hernández G, et al. An index combining respiratory rate and oxygenation to predict outcome of nasal high-flow therapy. Am J Respir Crit Care Med. 2019;199(11):1368-76. PMID: 30576221
