# Supplementary material for: ARDS Clinical Practice Guideline 2021
Source: J Intensive Care. 2022 Jul 8;10:32. doi: 10.1186/s40560-022-00615-6 (PMC9263056; doi:10.1186/s40560-022-00615-6)
Supplement: Supplementary file 3 — Additional file 3. Contains Modified Preferred Reporting items of Systematic Reviews and Meta-Analyses (PRISMA) flow-chart, risk of bias summary, forest plots, evidence profiles, and evidence to decision table for CQ19–30 (area C) according to the GRADE system [file 40560_2022_615_MOESM3_ESM.docx]

Additional file 3

Modified Preferred Reporting items of Systematic Reviews and Meta-Analyses (PRISMA) flow-chart, risk of bias summary, forest plots, evidence profiles, and evidence to decision table for CQ19-30 (area C) according to the GRADE system

Table of contents

1. CQ19
   1. Search strategy p.4
   2. Flow diagram p.8
   3. Risk of bias p.9
   4. Forest plot p.11
   5. Evidence Profile p.13
   6. Evidence-to-Decision table p.16
2. CQ20
   1. Search strategy p.23
   2. Flow diagram p.28
   3. Risk of bias p.29
   4. Forest plot p.31
   5. Evidence Profile p.33
   6. Evidence-to-Decision table p.36
3. CQ21
   1. Search strategy p.44
   2. Flow diagram p.47
   3. Risk of bias p.48
   4. Forest plot p.49
   5. Evidence Profile p.51
   6. Evidence-to-Decision table p.54
4. CQ22
   1. Search strategy p.61
   2. Flow diagram p.63
   3. Risk of bias p.64
   4. Forest plot p.65
   5. Evidence Profile p.66
   6. Evidence-to-Decision table p.67
5. CQ23
   1. Search strategy p.74
   2. Flow diagram p.76
   3. Risk of bias p.77
   4. Forest plot p.78
   5. Evidence Profile p.79
   6. Evidence-to-Decision table p.81
6. CQ24
   1. Search strategy p.88
   2. Flow diagram p.90
   3. Risk of bias p.91
   4. Forest plot p.92
   5. Evidence Profile p.93
   6. Evidence-to-Decision table p.94
7. CQ25
   1. Search strategy p.101
   2. Flow diagram p.103
   3. Risk of bias p.104
   4. Forest plot p.104
   5. Evidence Profile p.104
   6. Evidence-to-Decision table p.105
8. CQ26
   1. Search strategy p.111
   2. Flow diagram p.113
   3. Risk of bias p.114
   4. Forest plot p.116
   5. Evidence Profile p.118
   6. Evidence-to-Decision table p.120
9. CQ27
   1. Search strategy p.127
   2. Flow diagram p.129
   3. Risk of bias p.130
   4. Forest plot p.133
   5. Evidence Profile p.135
   6. Evidence-to-Decision table p.138
10. CQ28
    1. Search strategy p.147
    2. Flow diagram p.150
    3. Risk of bias p.151
    4. Forest plot p.153
    5. Evidence Profile p.155
    6. Evidence-to-Decision table p.158
11. CQ29
    1. Search strategy p.165
    2. Flow diagram p.167
    3. Risk of bias p.168
    4. Forest plot p.168
    5. Evidence Profile p.168
    6. Evidence-to-Decision table p.169
12. CQ30
    1. Search strategy p.176
    2. Flow diagram p.179
    3. Risk of bias p.180
    4. Forest plot p.183
    5. Evidence Profile p.185
    6. Evidence-to-Decision table p.188

**CQ19 Should low tidal volume be used in mechanically ventilated adult patients with ARDS?**

1. Search strategy

**MEDLINE via PubMed (**Search date: **2020/7/6)**

| #1 | Respiratory Distress Syndrome, Adult[mh] OR ARDS[tiab] OR shock lung[tiab] |
| --- | --- |
| #2 | acute respiratory distress[tiab] OR acute respiratory failure[tiab] |
| #3 | Acute[tiab] AND ((respirat*[tiab] OR ventilat*[tiab] OR pulmon*[tiab]) AND (fail*[tiab] OR depression[tiab])) |
| #4 | Lung injury[mh] OR ALI[tiab] OR Acute lung injur*[tiab] OR Ventilator-Induced Lung Injury[tiab] |
| #5 | Respiratory insufficiency[mh] OR Respiratory insufficiency[tiab] |
| #6 | Acute chest syndrome[mh] OR Acute chest syndrome[tiab] |
| #7 | #1 OR #2 OR #3 OR #4 OR #5 OR #6 |
| #8 | "Tidal volume"[mh] OR (tidal[tiab] AND volum*[tiab]) |
| #9 | ventilation AND (strateg*[tiab] OR pressure*[tiab] OR limited[tiab] OR low[tiab] OR lower[tiab] OR less[tiab] OR differen*[tiab] OR variab*[tiab] OR varying[tiab]) |
| #10 | (lung[tiab] AND protective[tiab] AND ventilat*[tiab] ) OR LPVS[tiab] |
| #11 | Respiration,artificial[mh] OR "Artificial respiration"[tiab] OR "Artificial ventilation"[tiab] |
| #12 | Ventilators, Mechanical[mh] OR "Mechanical ventilation"[tiab] |
| #13 | #8 OR #9 OR #10 OR #11 OR #12 |
| #14 | #7 AND #13 |
| #15 | ((randomized controlled trial[pt] OR controlled clinical trial[pt] OR randomized[tiab] OR placebo[tiab] OR clinical trials as topic[mesh:noexp] OR randomly[tiab] OR trial[ti] NOT (animals[mh] NOT humans [mh]))) |
| #16 | control group*[tiab] |
| #17 | #15 OR #16 |
| #18 | #14 AND #17 |

**CENTRAL (**Search date: **2020/7/6)**

| #1 | [mh "Respiratory Distress Syndrome, Adult"] OR ARDS:ti,ab OR "shock lung":ti,ab |
| --- | --- |
| #2 | "acute respiratory distress":ti,ab OR "acute respiratory failure":ti,ab |
| #3 | Acute:ti,ab AND ((respirat*:ti,ab OR ventilat*:ti,ab OR pulmon*:ti,ab) AND (fail*:ti,ab OR depression:ti,ab)) |
| #4 | [mh "Lung injury"] OR ALI:ti,ab OR "Acute lung injury":ti,ab OR " Ventilator-Induced Lung Injury":ti,ab |
| #5 | [mh "Respiratory insufficiency"] OR "Respiratory insufficiency":ti,ab |
| #6 | [mh "Acute chest syndrome"] OR "Acute chest syndrome":ti,ab |
| #7 | {OR #1-#6} |
| #8 | [mh "Tidal volume"] OR (tidal:ti,ab AND volume:ti,ab) |
| #9 | ventilation AND (strategy:ti,ab OR pressure:ti,ab OR limited:ti,ab OR low:ti,ab OR lower:ti,ab OR less:ti,ab OR different:ti,ab OR variable:ti,ab OR varying:ti,ab) |
| #10 | (lung:ti,ab AND protective:ti,ab AND ventilatory:ti,ab) OR LPVS:ti,ab |
| #11 | [mh "Respiration, Artificial"] |
| #12 | [mh "Ventilators, Mechanical"] OR "Mechanical ventilation":ti,ab |
| #13 | {OR #8-#12} |
| #14 | #7 AND #13 |
| #15 | [mh animals] NOT [mh humans] |
| #16 | #14 NOT #15 |

Igaku-Chuo-Zasshi **(**Search date: **2020/7/12)**

| #1 | 呼吸窮迫症候群-急性/TH or 急性呼吸促迫症候群/AL or ARDS/AL or “acute respiratory distress syndrome”/AL or ショック肺/AL or “shock lung”/AL |
| --- | --- |
| #2 | 肺損傷/TH or 急性肺損傷/AL or “acute lung Injury”/AL or 人工呼吸器誘発肺損傷/AL |
| #3 | 呼吸不全/TH or 呼吸不全/AL or 呼吸機能不全/AL |
| #4 | 呼吸抑制/TH or 呼吸抑制/AL |
| #5 | #1 or #2 or #3 or #4 |
| #6 | 一回換気量/TH or 一回換気量/AL or 低容量換気/AL or “tidal volume”/AL |
| #7 | (肺/TH or 肺/AL or lung/AL) and (保護/AL or protective/AL) |
| #8 | 制限/AL or 低/AL or 保護/AL or 圧/AL |
| #9 | 戦略/AL or strategy/AL |
| #10 | #7 or #8 or #9 |
| #11 | #10 and ([換気(環境)]/TH or 換気/Al or ventilation/AL) |
| #12 | 人工呼吸/TH or 人工呼吸/AL or レスピレータ/AL or ベンチレータ/AL or 機械的換気/AL or 人工換気/AL or 調節呼吸/AL |
| #13 | LPVS/AL |
| #14 | #6 OR #11 OR #12 OR #13 |
| #15 | #5 and #14 |
| #16 | ランダム化比較試験/TH or ランダム化/AL or 無作為化/AL |
| #17 | 比較試験/AL |
| #18 | 臨床試験/TH or 臨床試験/AL |
| #19 | プラセボ/TH or プラセボ/AL |
| #20 | 対照/AL |
| #21 | コントロール/AL |
| #22 | 臨床研究・疫学研究/TH or 臨床研究/AL |
| #23 | #16 or #17 or #18 or #19 or #20 or #21 or #22 |
| #24 | #15 AND #23 |
| #25 | (#24) and (PT=会議録除く) |

**EMBASE (**Search date: **2020/6/28)**

| S1 | (EMB.EXACT("adult respiratory distress syndrome")) OR (TI,AB(ARDS OR "shock lung")) |
| --- | --- |
| S2 | (TI,AB("acute respiratory" p/0 (distress OR failure*))) |
| S3 | (TI,AB(acute n/3 (respirat* OR ventilat* OR pulmon*) n/3 (fail* OR depression))) |
| S4 | ((EMB.EXACT("acute lung injury")) OR (EMB.EXACT("hyperoxia-induced lung injury") OR EMB.EXACT("lung injury")) OR (EMB.EXACT("ventilator induced lung injury")) OR (TI,AB(ALI OR ("acute lung" p/0 injur*) OR "ventilator-Induced lung injury"))) |
| S5 | ((EMB.EXACT.EXPLODE("respiratory failure")) OR (TI,AB(respiratory p/0 insufficien*))) |
| S6 | ((EMB.EXACT("acute chest syndrome")) OR (TI,AB("acute chest syndrome"))) |
| S7 | (S1 OR S2 OR S3 OR S4 OR S5 OR S6) |
| S8 | (EMB.EXACT("tidal volume") OR TI,AB(tidal n/2 volum*)) |
| S9 | (TI,AB(ventilation AND (strateg* OR pressure* OR limited OR low OR lower OR less OR differen* OR variab* OR varying))) |
| S10 | (TI,AB(("lung protective" n/2 ventilat*) OR LPVS)) |
| S11 | ((EMB.EXACT.EXPLODE("artificial ventilation")) OR (TI,AB(artificial p/0 (respiration* OR ventilation*)) OR (TI,AB(mechanical p/0 ventilation*)))) |
| S12 | (S8 OR S9 OR S10 OR S11) |
| S13 | (S7 AND S12) |
| S14 | ((((EMB.EXACT("controlled clinical trial") OR EMB.EXACT.EXPLODE("clinical trial (topic)") OR EMB.EXACT("randomized controlled trial")) OR (TI,AB(randomized) OR TI,AB(randomly) OR TI(trial) OR TI,AB(control p/0 group*))) NOT (ANIMAL(YES) NOT HUMAN(YES)))) |
| S15 | (S13 AND S14) |
| S16 | (S15 AND UD(>=2013)) |
| S17 | (S15 AND UD(<2013)) |

**CHINAL (**Search date: **2020/7/12)**

| #1 | (MH "Respiratory Distress Syndrome, Adult") OR TI ARDS OR AB ARDS OR TI "shock lung" OR AB "shock lung" |
| --- | --- |
| #2 | TI "acute respiratory distress" OR AB "acute respiratory distress" OR TI "acute respiratory failure" OR AB "acute respiratory failure" |
| #3 | TI Acute OR AB Acute AND ((TI respirat* OR AB respirat* OR TI ventilat* OR AB ventilat* OR TI pulmon* OR AB pulmon*) AND (TI fail* OR AB fail* OR TI depression OR AB depression)) |
| #4 | (MH "Lung injury+") OR TI ALI OR AB ALI OR TI "Acute lung injur*" OR AB "Acute lung injur*" OR TI "Ventilator-Induced Lung Injury" OR AB "Ventilator-Induced Lung Injury" |
| #5 | (MH "Respiratory Failure+")  OR TI "Respiratory Failure" OR AB "Respiratory Failure" |
| #6 | (MH "Acute chest syndrome") OR TI "Acute chest syndrome" OR AB "Acute chest syndrome" |
| #7 | #1 OR #2 OR #3 OR #4 OR #5 OR #6 |
| #8 | (MH "Tidal volume") OR (TI tidal OR AB tidal AND TI volum* OR AB volum*) |
| #9 | ventilation AND (TI strateg* OR AB strateg* OR TI pressure* OR AB pressure* OR TI limited OR AB limited OR TI low OR AB low OR TI lower OR AB lower OR TI less OR AB less OR TI differen* OR AB differen* OR TI variab* OR AB variab* OR TI varying OR AB varying) |
| #10 | (TI lung OR AB lung AND TI protective OR AB protective AND TI ventilat* OR AB ventilat*) OR TI LPVS OR AB LPVS |
| #11 | (MH "Respiration, Artificial+") OR TI "Artificial respiration" OR AB "Artificial respiration" OR TI "Artificial ventilation" OR AB "Artificial ventilation" |
| #12 | (MH "Ventilators, Mechanical") OR TI “Mechanical Ventilat*” OR AB “Mechanical Ventilat*” |
| #13 | #8 OR #9 OR #10 OR #11 OR #12 |
| #14 | #7 AND #13 |

1. Flow diagram


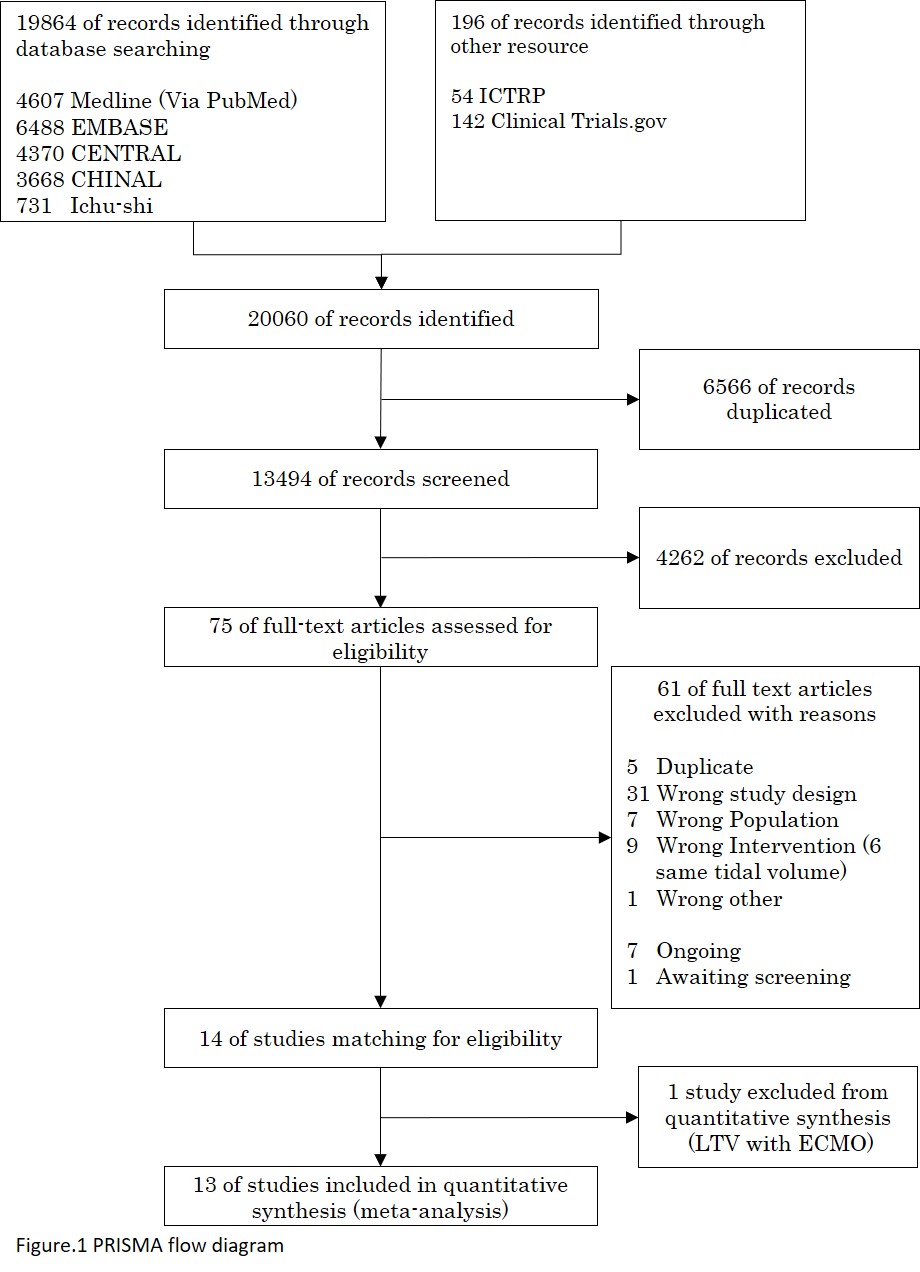


1. Risk of bias


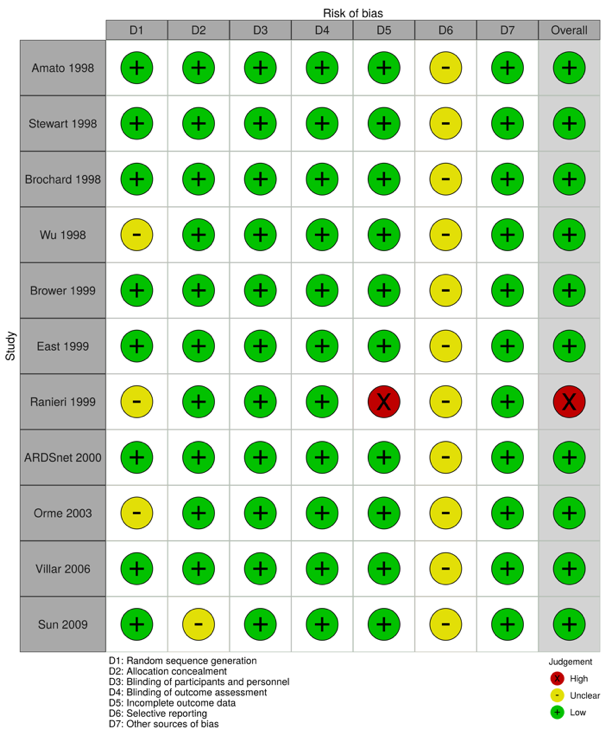
Short-term mortality Long-term mortality


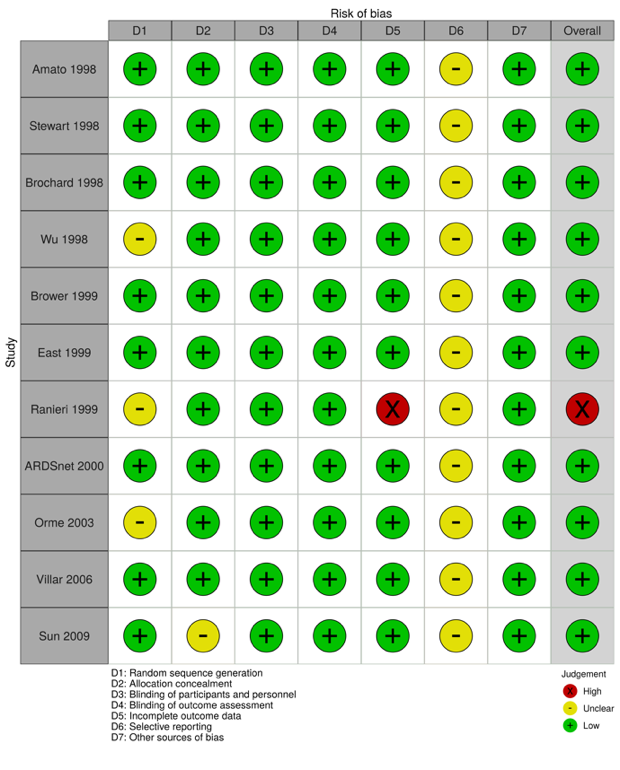


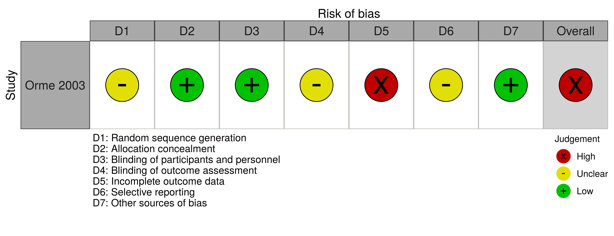
ADL/QOL P/F ratio


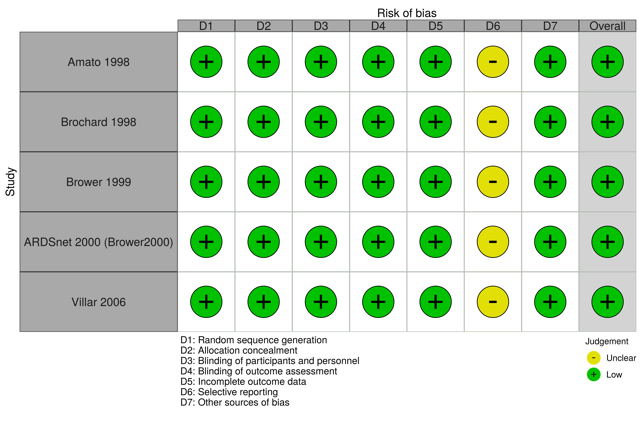


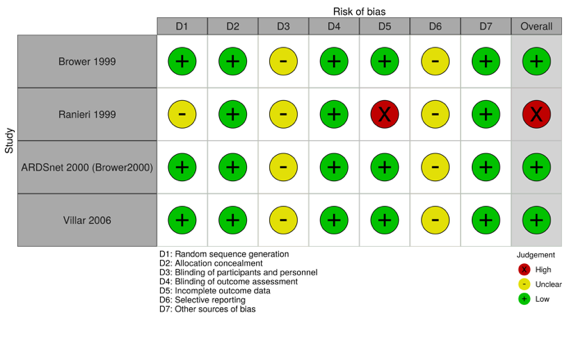
Ventilator-free days Length of hospital stay


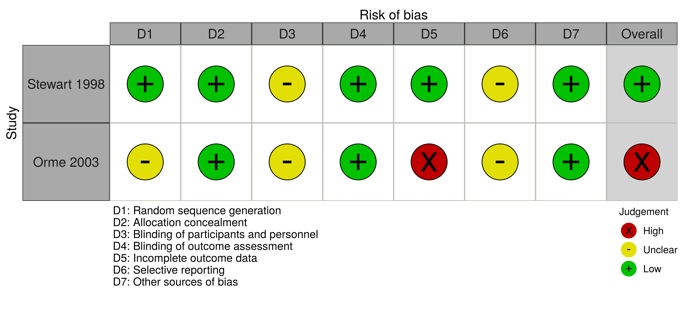


Barotrauma


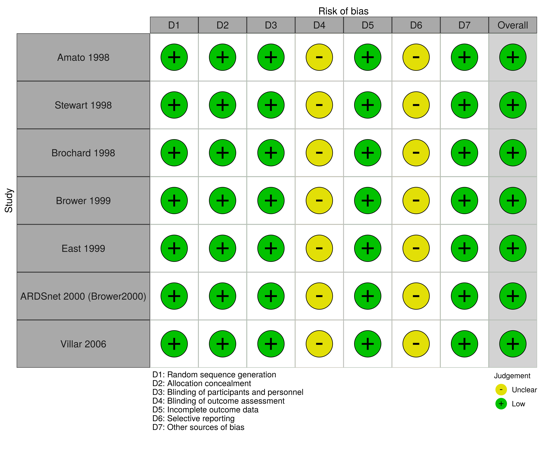


1. Forest plot

short-term mortality


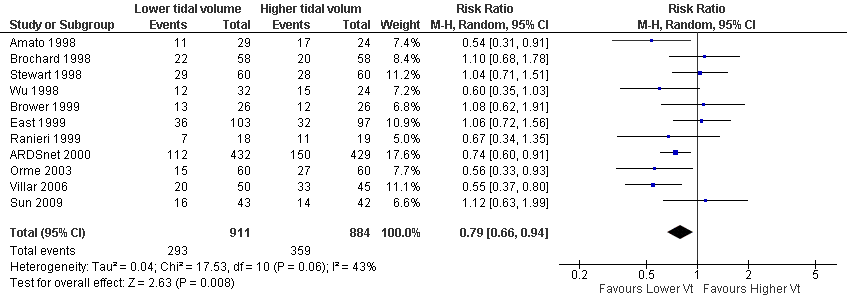


Long-term mortality


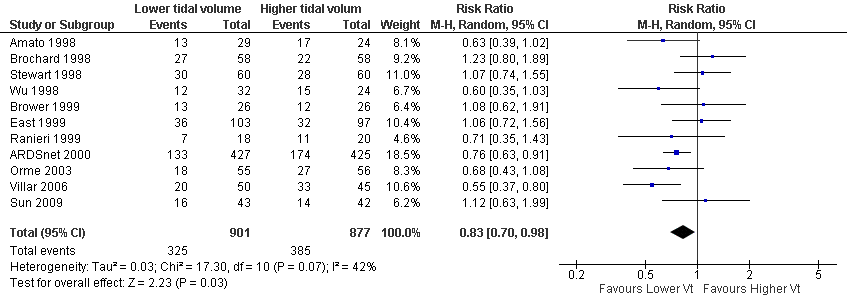


ADL/QOL


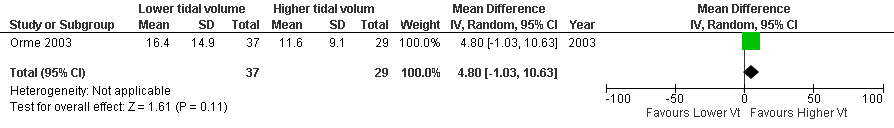


P/F ratio


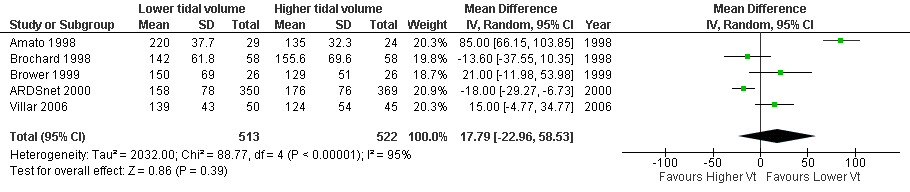


Ventilator-free days


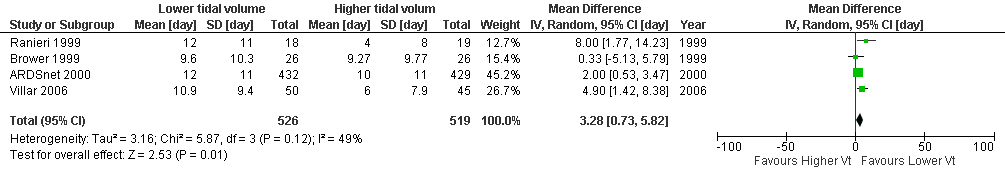


Length of hospital stay


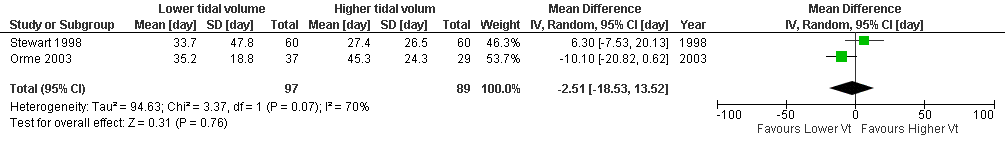


Barotrauma


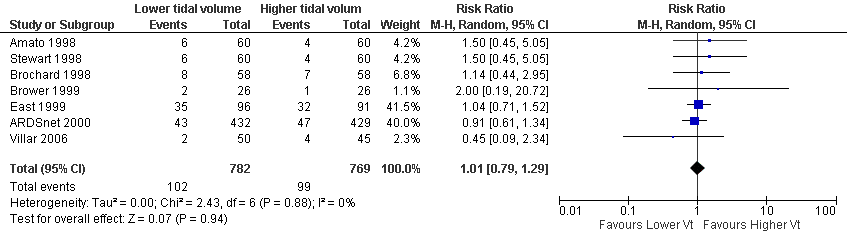


1. Evidence profile

| **Certainty assessment** | | | | | | | **№ of patients** | | **Effect** | | **Certainty** | **Importance** |
| --- | --- | --- | --- | --- | --- | --- | --- | --- | --- | --- | --- | --- |
| **№ of studies** | **Study design** | **Risk of bias** | **Inconsistency** | **Indirectness** | **Imprecision** | **Other considerations** | **Lower tidal volume (4**–**8 mL/kg)** | **higher tidal volume (>8 mL/kg)** | **Relative (95% CI)** | **Absolute (95% CI)** |  |  |
| **Short-term mortality*** | | | | | | | | | | | | |
| 11 | Randomized trials | Not serious | Serious^a^ | Not serious | Not serious | None | 293/911 (32.2%) | 359/884 (40.6%) | **RR 0.79** (0.66 to 0.94) | **85 fewer per 1,000** (from 138 fewer to 24 fewer) | ⨁⨁⨁◯ Moderate | CRITICAL |
| **Long-term mortality**** | | | | | | | | | | | | |
| 11 | Randomized trials | Not serious | Serious^a^ | Not serious | Not serious | None | 325/901 (36.1%) | 385/877 (43.9%) | **RR 0.83** (0.70 to 0.98) | **75 fewer per 1,000** (from 132 fewer to 9 fewer) | ⨁⨁⨁◯ Moderate | CRITICAL |
| **ADL/QOL** | | | | | | | | | | | | |
| 1 | Randomized trials | Serious^b^ | Not serious | Not serious | Very serious^c^ | None | 37 | 29 | - | MD 4.8 higher (1.03 lower to 10.63 higher) | ⨁◯◯◯ Very low | IMPORTANT |
| **PaO_2_/F_I_O_2_ ratio at Day 1** | | | | | | | | | | | | |
| 5 | Randomized trials | Not serious | Very serious^d,e^ | Not serious | Serious^f^ | None | 513 | 522 | - | MD 17.79 mmHg higher (22.96 lower to 58.53 higher) | ⨁◯◯◯ Very low | IMPORTANT |
| **Ventilator free day at 28** | | | | | | | | | | | | |
| 4 | Randomized trials | Not serious | Serious^g^ | Not serious | Not serious | none | 526 | 519 | - | MD 3.28 days longer (0.73 shorter to 5.82 longer) | ⨁⨁⨁◯ Moderate | CRITICAL |
| **Hospital length of stay** | | | | | | | | | | | | |
| 2 | Randomized trials | Not serious | Very serious^d,e^ | Not serious | Very serious^c^ | None | 97 | 89 | - | MD 2.51 day shorter (18.53 shorter to 13.52 longer) | ⨁◯◯◯ Very low | CRITICAL |
| **Barotrauma** | | | | | | | | | | | | |
| 7 | Randomized trials | Not serious | Serious^a^ | Not serious | Very serious^c^ | None | 102/782 (13.0%) | 99/769 (12.9%) | **RR 1.01** (0.79–1.29) | **1 more per 1,000** (from 27 fewer to 37 more) | ⨁◯◯◯ Very low | CRITICAL |

* Nearest 28-day mortality ** longest follow-up mortality **CI:** confidence interval; **MD:** mean difference; **RR:** risk ratio

#### Explanations

a. Different directions of effect in the study

b. Most of the studies had a high risk of bias

c. Wide confidence interval; the sample size did not reach the optimal information size (OIS)

d. Different direction of effect in the study (null, effective, adverse)

e. Heterogeneity (I2 statistics and significant heterogeneity test)

f. Wide confidence interval

g. Inconsistency in methods of calculation for a ventilator-free day at 28

1. Evidence-to-Decision table

| question | |
| --- | --- |
| **CQ19:** Should low tidal volume be used in mechanically ventilated adult patients with ARDS? | |
| **Group :** | Adult ARDS patients requiring ventilatory management |
| **INTERVENTIONS:** | Limited tidal volume (4-8 mL/kg) |
| **Comparison and contrast :** | Unrestricted tidal volume (>8 mL/kg) |
| **Main Outcomes:** | Long-term mortality, short-term mortality, ventilation free days (VFD), length of hospital stay, barotrauma |
| **SETTING:** | The emergency room or intensive care unit |
| **Perspectives :** | Personal |
| **Background :** | Critically ill patients with ARDS often require mechanical ventilation management. However, the mortality rate remains high in patients with ARDS. Some studies suggest that mechanical ventilation may be a cause of lung injury. Lung protective strategies are methods taken to reduce the adverse effects of mechanical ventilation. And low tidal volume ventilation is one of the lung protective strategies. Systematic reviews and meta-analyses are needed to examine the efficacy and safety of low tidal volume ventilation. |
| **Conflict of Interest:** | None |

# assessMENT

| Problem Is the problem a priority? | | |
| --- | --- | --- |
| judgment | research evidence | remarks |
| ○ No  ○ Probably no  ○ Probably yes  ● Yes  ○ Varies  ○ Do not know | Critically ill patients with ARDS often require mechanical ventilation management. However, the mortality rate remains high in patients with ARDS. Some studies suggest that mechanical ventilation may be a cause of lung injury. Lung protective strategies are methods taken to reduce the adverse effects of mechanical ventilation. And low tidal volume ventilation is one of the lung protective strategies. Systematic reviews and meta-analyses are needed to examine the efficacy and safety of low tidal volume ventilation. Therefore, this issue is of high priority. |  |
| Desirable effects How substantial are the desirable anticipated effects? | | |
| judgment | research evidence | note |
| ○Trivial  ○Small  ● Moderate  ○Large  ○Varies  ○Do not know | A systematic review found 13 randomized controlled trials (RCTs) consistent with the patient, intervention, comparison, and outcome (PICO) process (one was excluded because it included extracorporeal membrane oxygenation [ECMO]), and a meta-analysis was performed using them.  　　The effect estimate for short-term mortality (11 RCTs: N=1795) was 85 fewer deaths/1000 patients (95% CI: 138 to 24 fewer deaths) in "limited tidal volume" compared with "unrestricted tidal volume". The effect estimate for long-term mortality (11 RCTs: N=1778) was 75 fewer deaths/1000 people (95% CI: 132 fewer deaths to 9 fewer deaths), and the effect estimate for VFD (4 RCTs: N=1045) was a mean difference of 3.28 days longer (95% CI: 0.73 days longer to 5.82 days longer), the effect estimate for the length of hospital stay (2RCTs: N=186) was a mean difference of 2.51 days shorter (95% confidence interval: 18.53 days shorter to 13.52 days longer), and the effect estimate for barotrauma (7 RCTs: N=1551) was increased by 1 person/1000 people (95% confidence interval: decrease of 27 to increase of 37). Thus, the expected effect was judged to be “moderate.” |  |
| Undesirable effects How substantial are the undesirable anticipated effects? | | |
| judgment | research evidence | note |
| ○Large  ○ Moderate  ○Small  ○Trivial  ○Varies.  ● Do not know | We reviewed the outcomes of harm, but did not find any significant outcomes, including those in the studies we recruited or in other studies. Thus, the anticipated harm was judged to be “do not know.” |  |
| Certainty of evidence What is the overall certainty of the evidence of effects? | | |
| judgment | research evidence | note |
| ○Very low  ○Low  ●Moderate  ○High  ○No included studies | **Relative importance or value of the key outcomes of interest**   \| Outcome \| \| Relative importance \| \| Certainty of evidence (GRADE) \| \| \| --- \| --- \| --- \| --- \| --- \| --- \| \| Short-term mortality \| \| Serious \| \| ⨁⨁⨁◯  Moderate \| \| Long-term mortality \| \| Serious \| \| ⨁⨁⨁◯  Moderate \| \| VFD \| \| Serious \| \| ⨁⨁⨁◯  Moderate \| \| Length of hospital stay \| \| Serious \| \| ⨁◯◯◯  Very low \| \| Barotrauma \| \| Serious \| \| ⨁◯◯◯  Very low \|   **Overall evidence certainty**:  There was no agreement on the direction within the desired effect, and the certainty of evidence across the outcomes was judged to be “very low,” adopting the lowest certainty of evidence. |  |
| Values Is there important uncertainty about or variability in how much people value the main outcomes? | | |
| judgment | research evidence | note |
| ○Important uncertainty or variability  ○Possibly important uncertainty or variability  ○Probably no important uncertainty or variability  ●No important uncertainty or variability | Although there were no data on the values for outcomes in ventilatory management with limited tidal volume, the values for death are generally high and do not seem to vary. |  |
| Balance of effects Does the balance between desirable and undesirable effects favor the intervention or the comparison? | | |
| judgment | research evidence | remarks |
| ○Favors the comparison  ○Probably favors the comparison  ○Does not favor either the intervention or the comparison  ●Probably favors the intervention  ○Favors the intervention  ○Varies  ○Do not know | **Summary of results**   \| Outcome \| Unrestricted tidal volume (control) \| Limited tidal volume (intervention) \| Absolute difference  (95% CI) \| Relative effect  (95% CI) \| \| --- \| --- \| --- \| --- \| --- \| \| Short-term mortality \| 359/884 \| 293/911 \| 85 fewer people/1000  (138 people fewer - 24 people fewer) \| 0.79  (0.66-0.94) \| \| Long-term mortality \| 385/877 \| 325/901 \| 75 fewer people/1000  (132 people fewer ~ 9 people fewer) \| 0.83  (0.70-0.98) \| \| VFD \| - \| - \| MD 3.28 days longer  (0.73 days shorter to 5.82 days longer) \| - \| \| Length of hospital stay \| - \| - \| MD 2.51 days shorter  (18.53 days shorter to 13.52 days longer) \| -. \| \| Barotrauma \| 99/769 \| 102/782 \| 1 more /1000  (27 fewer ~ 37 more) \| 1.01  (0.79 - 1.29) \|   The balance of the effects and harms was judged to be “probably favors the intervention.” |  |
| Acceptability Is the intervention acceptable to key stakeholders? | | |
| judgment | research evidence | note |
| ○ No  ○ Probably no  ○ Probably yes  ● Yes  ○ Varies  ○ Do not know | Although no evidence was presented in the included studies, it has already been performed in routine clinical practice and seems feasible enough considering the cost and adverse effects. |  |
| Feasibility Is the intervention feasible to implement? | | |
| judgment | research evidence | remarks |
| ○ No  ○ Probably no  ○ Probably yes  ● Yes  ○ Varies  ○ Do not know | It is thought that limiting the tidal volume can easily reduce minute ventilation and lead to hypercapnia and respiratory acidosis. In fact, in one RCT (n=120) used in this systematic review, hypercapnia was observed in 31/60 patients (17/60 in the unrestricted tidal volume group) ^1)^. In this case, adequate monitoring is needed, although certain degree of hypercapnia is considered acceptable (permissive hypercapnia). |  |

# Summary of Judgment

|  | JUDGMENT | | | | | | |
| --- | --- | --- | --- | --- | --- | --- | --- |
| **PROBLEM** | No | Probably no | Probably yes | Yes |  | Varies | Do not know |
| **DESIRABLE EFFECTS** | Trivial | Small | Moderate | Large |  | Varies | Do not know |
| **UNDESIRABLE EFFECTS** | Large | Moderate | Small | Trivial |  | Varies | Do not know |
| **CERTAINTY OF EVIDENCE** | Very low | Low | Moderate | High |  |  | No included studies |
| **VALUES** | Important uncertainty or variability | Possibly important uncertainty or variability | Probably no important uncertainty or variability | No important uncertainty or variability |  |  |  |
| **BALANCE OF EFFECTS** | Favors the comparison | Probably favors the comparison | Does not favor either the intervention or the comparison | Probably favors the intervention | Favors the intervention | Varies | Do not know |
| **ACCEPTABILITY** | No | Probably no | Probably yes | Yes |  | Varies | Do not know |
| **FEASIBILITY** | No | Probably no | Probably yes | Yes |  | Varies | Do not know |

# Type of recommendation

| Strong recommendation against the intervention. | Conditional recommendation against the intervention. | Conditional recommendation for either the intervention or the comparison. | Conditional recommendation for the intervention. | Strong recommendation for the intervention. |
| --- | --- | --- | --- | --- |
| ○ | ○ | ○ | ○ | ● |

# Conclusion

| Recommendation |
| --- |
| **It is strongly recommended that ventilated adult patients with ARDS be restricted to a tidal volume of 4-8 mL/kg (strong recommendation/evidence of very low certainty: GRADE 1D).**  **Supplementary item**  **The recommendation is based on the results of the systematic review, which used the criterion of 4-8 mL/kg for the limited tidal volume group and >8 mL/kg for the unrestricted tidal volume group. Although the systematic review showed a very weak certainty of evidence, a re-vote at the panel meeting resulted in a strong recommendation because low tidal volume ventilation is considered to be widely and commonly practiced in daily practice.** |
|  |
| Reason/Justification |
| **Question:** Should low tidal volume be used in mechanically ventilated adult patients with ARDS?  **Patients:** Ventilated adult ARDS patients aged 16 years and older  **Intervention:** Limited tidal volume (4-8 mL/kg)  **Comparison control:** Unrestricted tidal volume (>8 mL/kg)  **Outcomes**: Short-term mortality, long-term mortality, ventilator-free days (VFD), length of hospital stay, barotrauma  **Summary of evidence**:  A systematic review found 13 RCTs consistent with PICO (one was excluded because it included ECMO), and a meta-analysis was performed using these.  Limited tidal volume reduced long-term mortality (11 RCTs: N=1778) by 75 per 1000 (95% confidence interval: 132 fewer to 9 fewer), reduced short-term mortality (11 RCTs: N=1795) by 85 per 1000 (95% confidence interval: 138 fewer to 24 fewer), prolonged VFD (4 RCTs: N=1045) by an average of 3.28 days (95% CI: 0.73 days shorter to 5.82 days more), shortened length of hospital stay (2 RCTs: N=186) by an average of 2.51 days (95% CI: 18.53 days shorter to 13.52 days longer), and increased barotrauma (7 RCTs: N=1551) by an average of 1 person per 1000 (95% confidence interval: 27 fewer patients ~ 37 more patients). Therefore, the expected desired effect was judged to be “probably large.” On the contrary, we examined the outcome of harm, but there was no significant outcome, and the expected harm was judged to be “unknown.” Therefore, the balance of effects and harms was judged to be “probably large.”  Note: Low tidal volume: defined as 4-8 mL/kg, Unrestricted tidal volume: >8 mL/kg.  **Certainty of evidence**.  The direction of the outcomes within the desired effect was not consistent, and the certainty of the evidence across the outcomes was judged to be “very low,” adopting the certainty of the least certain evidence.  **Determining values, balance of effects, acceptance, and viability**:  　The increase in patient burden and cost of performing low tidal volume ventilation is considered to be small and feasible.  **Panel Meeting:**  In the pre-vote, the modified Delphi method resulted in a median score of 8 and a disagreement index of 0.1918 for “Recommendation of low tidal volume ventilation for ventilated adult patients with ARDS (weak recommendation/very low certainty evidence: GRADE 2D).” There was some discussion at the panel meeting about the strength of the recommendation. The draft text of the recommendation was a weak recommendation, but there was a view that low tidal volume ventilation, which is already recognized worldwide as the standard of care, should be a strong recommendation. Therefore, after another round of voting and using the modified Delphi method, “Recommendation of low tidal volume ventilation for ventilated adult patients with ARDS (strong recommendation/evidence of very low certainty: GRADE 1D)” was agreed with a median score of 9 and a disagreement index of 0.1316.  **Additional Considerations**.  　This time, the overall certainty of the evidence was “very low.” However, because the barotrauma that caused the difference in the direction in the outcome of benefit also increased by only 1 per 1,000 patients, and because it is a treatment that was judged to be widely and commonly used, the panel voted to make the above recommendation, resulting in agreement as a strong recommendation.  　　For the calculation of the tidal volume, the predicted body weight calculated from the height should be used instead of the actual body weight (male: 50 + 0.91 (height (cm) - 152.4), female: 45.5 + 0.91 (height (cm) - 152.4)) is required. |

| Subgroup considerations |
| --- |
| As a sensitivity analysis, we included not only the comparison of ventilation defined above as low tidal volume ventilation: 4-8 mL/kg and unrestricted tidal volume ventilation: >8 mL/kg but also the comparison of low and high tidal volumes, as defined by the authors, regardless of the criteria. The results were similar to the main analysis. |
| Implementation considerations |
| Regarding low tidal volume ventilation for ARDS, the ARDS Clinical Practice Guideline 2016 recommended a use of tidal volume of 6-8 mL/kg (predicted body weight) (GRADE 1B). The impact of the increase in the number of adopted studies from 6 to 13 and the different methods of assessing the certainty of evidence were considered as reasons for the difference between this recommendation and the certainty of evidence. In addition, the ATS/ESICM/SCCM 2017 ARDS guidelines also strongly recommended low tidal volume ventilation of 4-8 mL/kg, suggesting that there is sufficient consensus to low tidal volume ventilation.  If spontaneous respiratory effort is high and the tidal volume does not reach the target, increased doses of muscle relaxants and sedatives should be considered in the acute phase (see also CQ31 for muscle relaxants). In addition, although hypercapnia can be tolerated to some extent when ventilatory management is performed with a low minute volume ventilation (permissive hypercapnia), the minute volume should be adjusted to avoid excessive respiratory acidosis that may affect circulatory dynamics. |

| Monitoring and evaluation |
| --- |
| To implement the recommendations, further information needs to be collected on the extent of compliance with the low tidal volume (4-8 mL/kg) as a clinical issue. It is also necessary to monitor whether there are any other clinical problems using questionnaires after the guideline is published. |
| Research priorities |
| Lung protective strategies with low tidal volume are now widely used. In the future, it may be necessary to compare this strategy with lung protective strategies that take into account driving pressure and transpulmonary pressure. |

References

1) Stewart TE, Meade MO, Cook DJ, et al. Evaluation of a ventilation strategy to prevent barotrauma in patients at high risk for acute respiratory distress syndrome. N Engl J Med. 1998;338(6):355-61. PMID: 9449728

**CQ20 Should high levels of positive end-expiratory pressure (PEEP) be used for mechanically ventilated adult patients with ARDS?**

1. Search strategy

MEDLINE via PubMed （Search date: 2020/7/8）

| #1 | Respiratory Distress Syndrome, Adult[mh] OR ARDS[tiab] OR shock lung[tiab] |
| --- | --- |
| #2 | acute respiratory distress[tiab] OR acute respiratory failure[tiab] |
| #3 | Acute[tiab] AND ((respirat*[tiab] OR ventilat*[tiab] OR pulmon*[tiab]) AND (fail*[tiab] OR depression[tiab])) |
| #4 | Lung injury[mh] OR ALI[tiab] OR Acute lung injur*[tiab] OR Ventilator-Induced Lung Injury[tiab] |
| #5 | Respiratory insufficiency[mh] OR Respiratory insufficiency[tiab] |
| #6 | Acute chest syndrome[mh] OR Acute chest syndrome[tiab] |
| #7 | #1 OR #2 OR #3 OR #4 OR #5 OR #6 |
| #8 | Positive-Pressure Respiration[mh] OR Positive Pressure Respiration[tiab] OR Positive End-Expiratory Pressure[tiab] OR PEEP[tiab] |
| #9 | Continuous Positive Airway Pressure[tiab] OR CPAP[tiab] OR NCPAP[tiab] |
| #10 | Airway Pressure Release Ventilation[tiab] OR APRV[tiab] |
| #11 | Intermittent Positive-Pressure Breathing[tiab] OR IPPB[tiab] |
| #12 | Intermittent Positive-Pressure Ventilation[tiab] OR IPPV[tiab] |
| #13 | lung protective ventilatory strateg*[tiab] OR LPVS[tiab] |
| #14 | alveolar recruit*[tiab] OR recruitment maneuve*[tiab] |
| #15 | #8 OR #9 OR #10 OR #11 OR #12 OR #13 OR #14 |
| #16 | #7 AND #15 |
| #17 | (randomized controlled trial[pt] OR controlled clinical trial[pt] OR randomized[tiab] OR placebo[tiab] OR clinical trials as topic[mesh:noexp] OR randomly[tiab] OR trial[ti]) NOT (animals[mh] NOT humans [mh]) |
| #18 | #16 AND #17 |

CENTRAL （Search date: 2020/7/8）

| #1 | [mh "Respiratory Distress Syndrome, Adult"] OR ARDS:ti,ab OR "shock lung":ti,ab |
| --- | --- |
| #2 | "acute respiratory distress":ti,ab OR "acute respiratory failure":ti,ab |
| #3 | Acute:ti,ab AND ((respirat*:ti,ab OR ventilat*:ti,ab OR pulmon*:ti,ab) AND (fail*:ti,ab OR depression:ti,ab)) |
| #4 | [mh "lung injury"] OR ALI:ti,ab OR "Acute lung injury":ti,ab OR "Ventilator-Induced Lung Injury":ti,ab |
| #5 | [mh "Respiratory insufficiency"] OR "Respiratory insufficiency":ti,ab |
| #6 | [mh "Acute chest syndrome"] OR "Acute chest syndrome":ti,ab |
| #7 | {OR #1-#6} |
| #8 | [mh "Positive Pressure Respiration"] OR "Positive Pressure Respiration":ti,ab OR "Positive End-Expiratory Pressure":ti,ab OR PEEP:ti,ab |
| #9 | "Continuous Positive Airway Pressure":ti,ab OR CPAP:ti,ab OR NCPAP:ti,ab |
| #10 | "Airway Pressure Release Ventilation":ti,ab OR APRV:ti,ab |
| #11 | "Intermittent Positive-Pressure Breathing":ti,ab OR IPPB:ti,ab |
| #12 | "Intermittent Positive-Pressure Ventilation":ti,ab OR IPPV:ti,ab |
| #13 | "lung protective ventilatory strategy":ti,ab OR LPVS:ti,ab |
| #14 | "Alveolar recruitment":ti,ab OR "recruitment maneuvers":ti,ab |
| #15 | {OR #8-#14} |
| #16 | #7 AND #15 |
| #17 | [mh animals] not [mh humans] |
| #18 | #16 not #17 |

Igaku-Chuo-Zasshi （Search date: 2020/7/12）

| #1 | 呼吸窮迫症候群-急性/TH or 急性呼吸促迫症候群/AL or ARDS/AL or "acute respiratory distress syndrome"/AL or ショック肺/AL or "shock lung"/AL |
| --- | --- |
| #2 | 肺損傷/TH or 急性肺損傷/AL or "acute lung Injury"/AL or 人工呼吸器誘発肺損傷/AL |
| #3 | 呼吸不全/TH or 呼吸不全/AL or 呼吸機能不全/AL |
| #4 | 呼吸抑制/TH or 呼吸抑制/AL |
| #5 | #1 or #2 or #3 or #4 |
| #6 | 陽圧呼吸/TH or 陽圧呼吸/AL or PEEP/AL |
| #7 | 気道圧解除換気法/AL or CPAP/AL or APRV/AL |
| #8 | 間欠的陽圧換気/AL or IPPV/AL or IPPB/AL |
| #9 | リクルートメント/AL |
| #10 | #6 or #7 or #8 or #9 |
| #11 | #5 and #10 |
| #12 | ランダム化比較試験/TH or ランダム化/AL or 無作為化/AL |
| #13 | 比較試験/AL |
| #14 | 臨床試験/TH or 臨床試験/AL |
| #15 | プラセボ/TH or プラセボ/AL |
| #16 | 対照/AL |
| #17 | コントロール/AL |
| #18 | 臨床研究・疫学研究/TH or 臨床研究/AL |
| #19 | #12 or #13 or #14 or #15 or #16 or #17 or #18 |
| #20 | #11 and #19 |
| #21 | (#20) and (PT=会議録除く) |

EMBASE (Search date: 2020/6/28)

| S1 | (EMB.EXACT("adult respiratory distress syndrome")) OR (TI,AB(ARDS OR "shock lung")) |
| --- | --- |
| S2 | (TI,AB("acute respiratory" p/0 (distress OR failure*))) |
| S3 | (TI,AB(acute n/3 (respirat* OR ventilat* OR pulmon*) n/3 (fail* OR depression))) |
| S4 | (EMB.EXACT("acute lung injury")) OR (EMB.EXACT("hyperoxia-induced lung injury") OR EMB.EXACT("lung injury")) OR (EMB.EXACT("ventilator induced lung injury")) OR (TI,AB(ALI OR ("acute lung" p/0 injur*) OR "ventilator-Induced lung injury")) |
| S5 | (EMB.EXACT.EXPLODE("respiratory failure")) OR (TI,AB(respiratory p/0 insufficien*)) |
| S6 | (EMB.EXACT("acute chest syndrome")) OR (TI,AB("acute chest syndrome")) |
| S7 | (S1 OR S2 OR S3 OR S4 OR S5 OR S6) |
| S8 | ((EMB.EXACT("positive end expiratory pressure")) OR (TI,AB("positive pressure respiration" OR "positive end-expiratory pressure" OR PEEP))) |
| S9 | (TI,AB("continuous positive airway pressure" OR CPAP OR NCPAP)) |
| S10 | (TI,AB("airway pressure release ventilation" OR APRV)) |
| S11 | (TI,AB("intermittent positive-pressure breathing" OR IPPB)) |
| S12 | (TI,AB("intermittent positive-pressure ventilation" OR IPPV)) |
| S13 | (TI,AB(("lung protective" p/0 ventilat* p/0 strateg*) OR LPVS)) |
| S14 | (TI,AB((alveolar p/0 recruit*) OR (recruitment p/0 maneuve*))) |
| S15 | (S8 OR S9 OR S10 OR S11 OR S12 OR S13 OR S14) |
| S16 | (S7 AND S15) |
| S17 | (((EMB.EXACT("controlled clinical trial") OR EMB.EXACT.EXPLODE("clinical trial (topic)") OR EMB.EXACT("randomized controlled trial")) OR (TI,AB(randomized) OR TI,AB(randomly) OR TI(trial))) NOT (ANIMAL(YES) NOT HUMAN(YES))) |
| S18 | (S16 AND S17) |

CHINAL (Search date: 2020/7/12)

| #1 | (MH "Respiratory Distress Syndrome, Adult") OR TI ARDS OR AB ARDS OR TI "shock lung" OR AB "shock lung" |
| --- | --- |
| #2 | TI "acute respiratory distress" OR AB "acute respiratory distress" OR TI "acute respiratory failure" OR AB "acute respiratory failure" |
| #3 | TI Acute OR AB Acute AND ((TI respirat* OR AB respirat* OR TI ventilat* OR AB ventilat* OR TI pulmon* OR AB pulmon*) AND (TI fail* OR AB fail* OR TI depression OR AB depression)) |
| #4 | (MH "Lung injury+") OR TI ALI OR AB ALI OR TI "Acute lung injur*" OR AB "Acute lung injur*" OR TI "Ventilator-Induced Lung Injury" OR AB "Ventilator-Induced Lung Injury" |
| #5 | (MH "Respiratory Failure+")  OR TI "Respiratory Failure" OR AB "Respiratory Failure" |
| #6 | (MH "Acute chest syndrome") OR TI "Acute chest syndrome" OR AB "Acute chest syndrome" |
| #7 | S1OR S2 OR S3 OR S4 OR S5 OR S6 |
| #8 | (MH "Positive-Pressure Respiration+") OR TI "Positive Pressure Respiration" OR AB "Positive Pressure Respiration" OR TI "Positive End-Expiratory Pressure" OR AB "Positive End-Expiratory Pressure" OR TI PEEP OR AB PEEP |
| #9 | TI "Continuous Positive Airway Pressure" OR AB "Continuous Positive Airway Pressure" OR TI CPAP OR AB CPAP OR TI NCPAP OR AB NCPAP |
| #10 | TI "Airway Pressure Release Ventilation" OR AB "Airway Pressure Release Ventilation" OR TI APRV OR AB APRV |
| #11 | TI "Intermittent Positive-Pressure Breathing" OR AB "Intermittent Positive-Pressure Breathing" OR TI IPPB OR AB IPPB |
| #12 | TI "Intermittent Positive-Pressure Ventilation" OR AB "Intermittent Positive-Pressure Ventilation" OR TI IPPV OR AB IPPV |
| #13 | TI "lung protective ventilatory strateg*" OR AB "lung protective ventilatory strateg*" OR TI LPVS OR AB LPVS |
| #14 | TI "alveolar recruit*" OR AB "alveolar recruit*" OR TI "recruitment maneuve*" OR AB "recruitment maneuve*" |
| #15 | #8 OR #9 OR #10 OR #11 OR #12 OR #13 OR #14 |
| #16 | #7 AND #15 |
| #17 | (MH "Randomized Controlled Trials") |
| #18 | TI ( random* or placebo* or blind* or double blind* ) OR AB ( random* or placebo* or blind* or double blind* ) |
| #19 | #17 OR #18 |
| #20 | #16 AND #19 |

1. Flow diagram


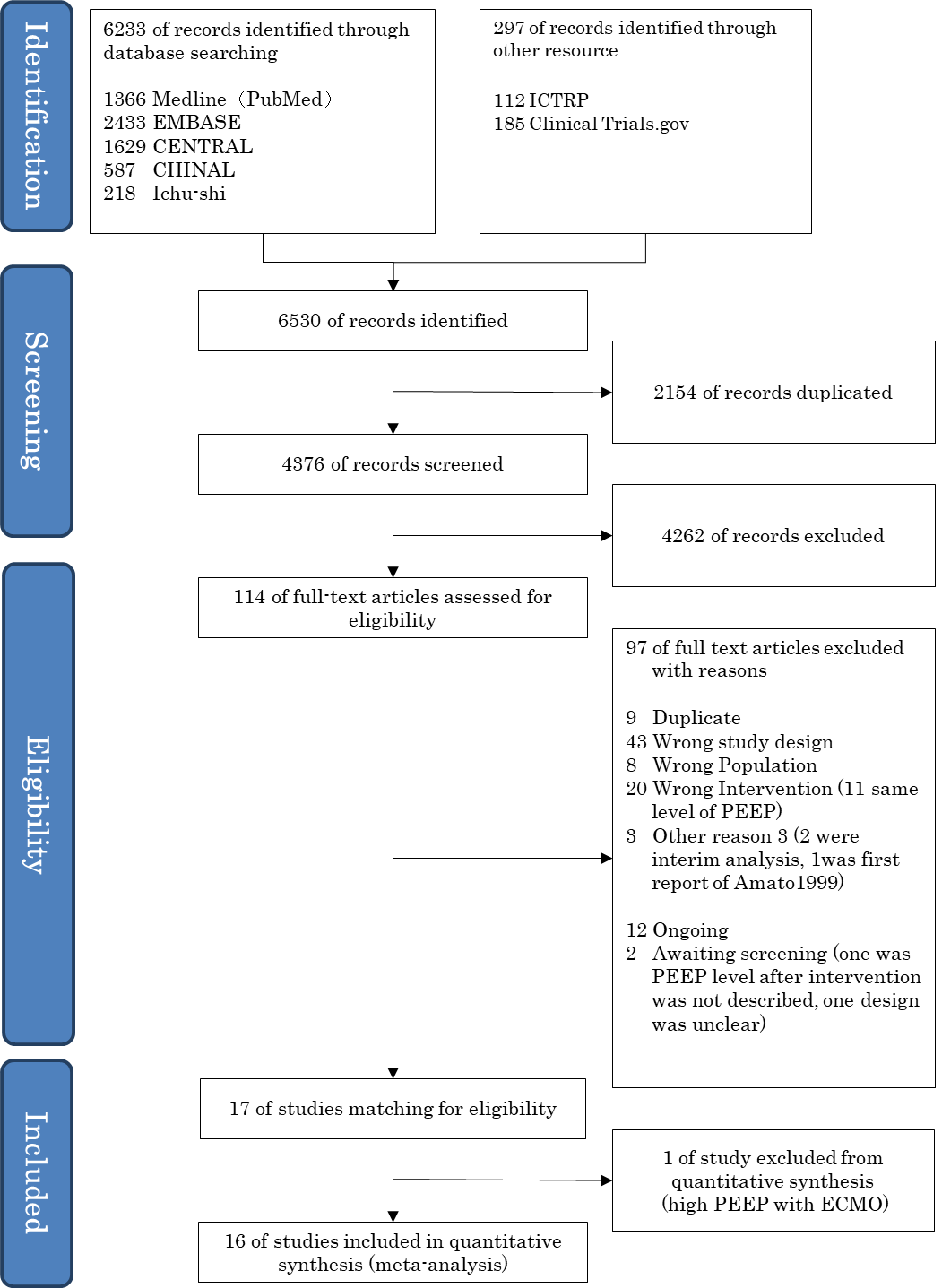


1. Risk of bias

Short-term mortality Long-term mortality


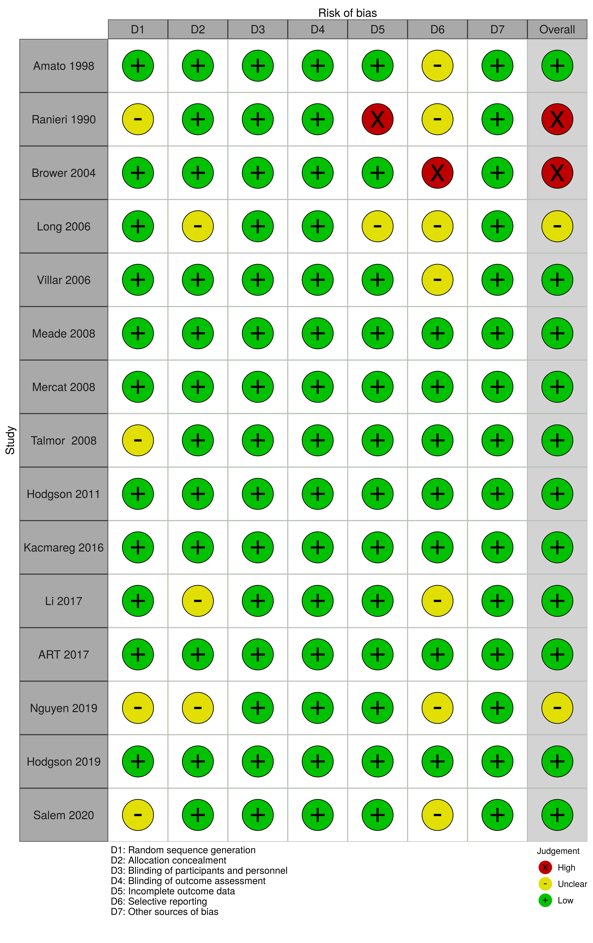

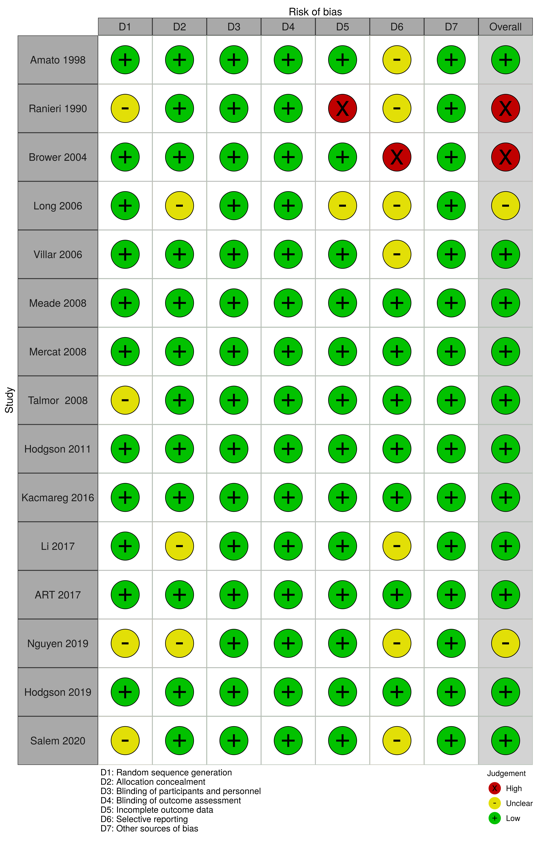


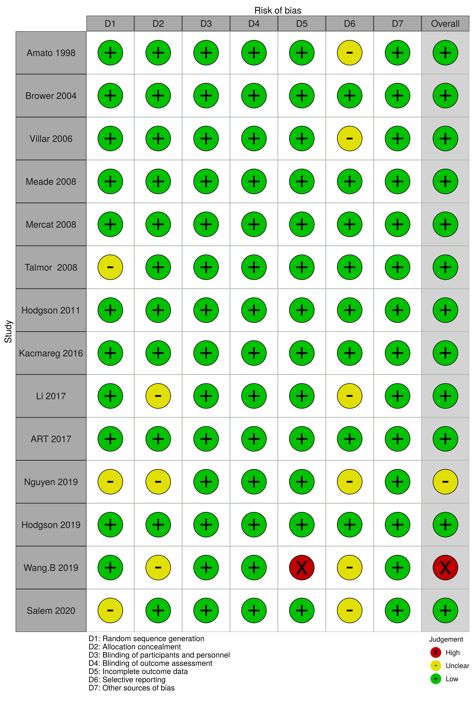
P/F ratio Vntilator-free days


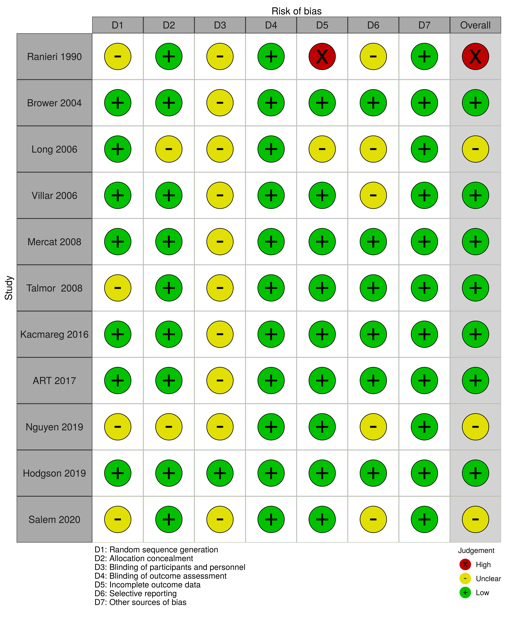


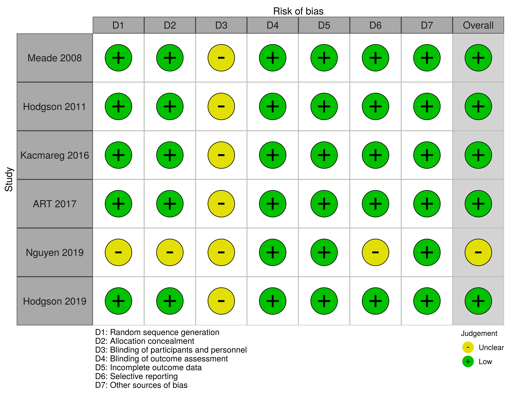
Length of hospital stay Barotrauma


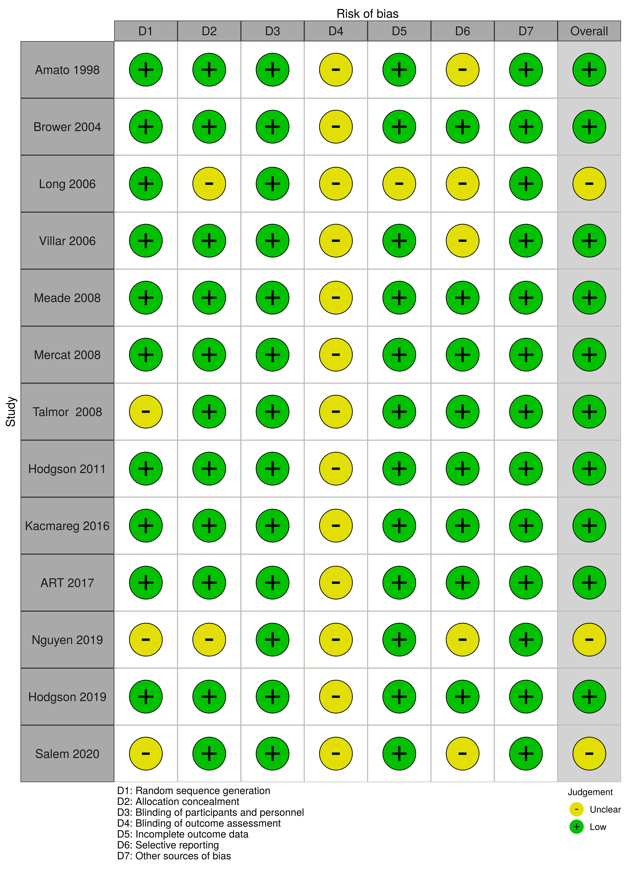


1. Forest plot

Short-term mortality


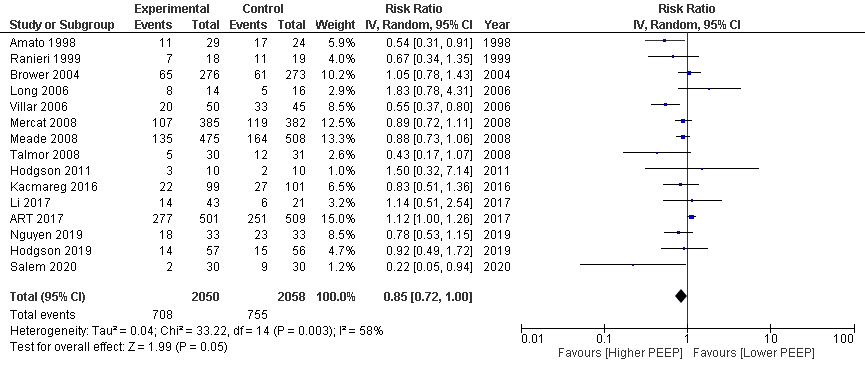


Long-term mortality


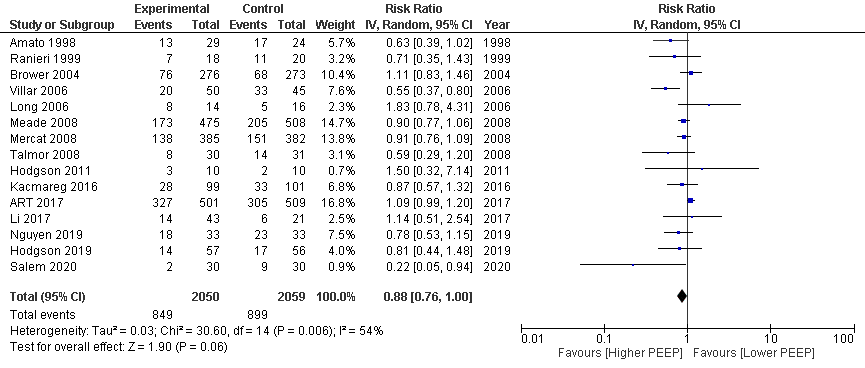


P/F ratio


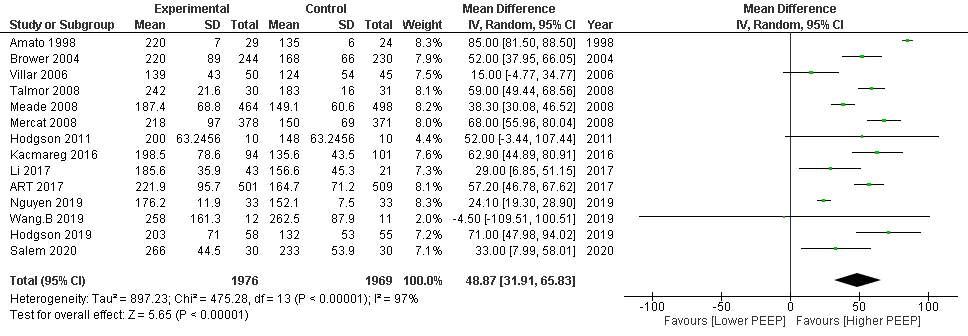


Ventilator-free days


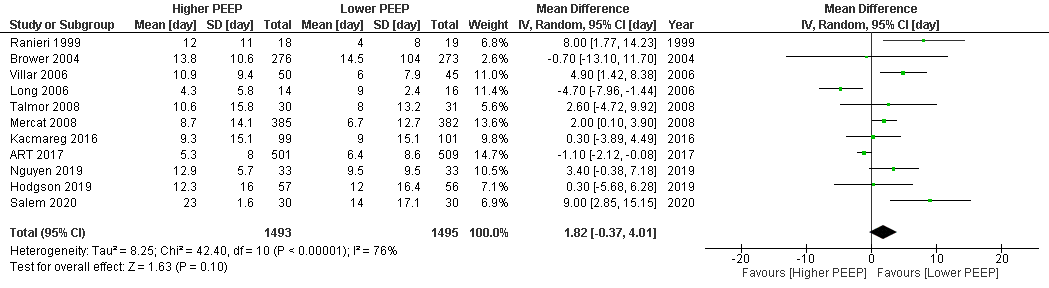


Length of hospital stay


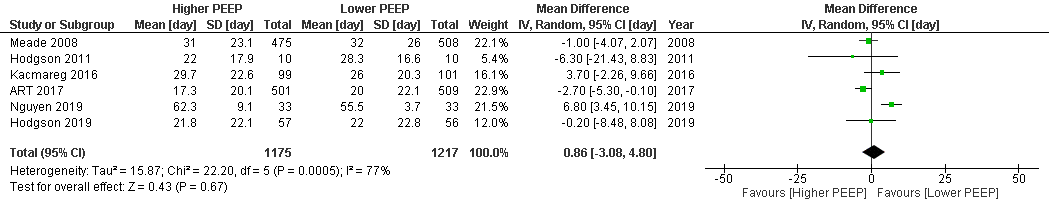


Barotrauma


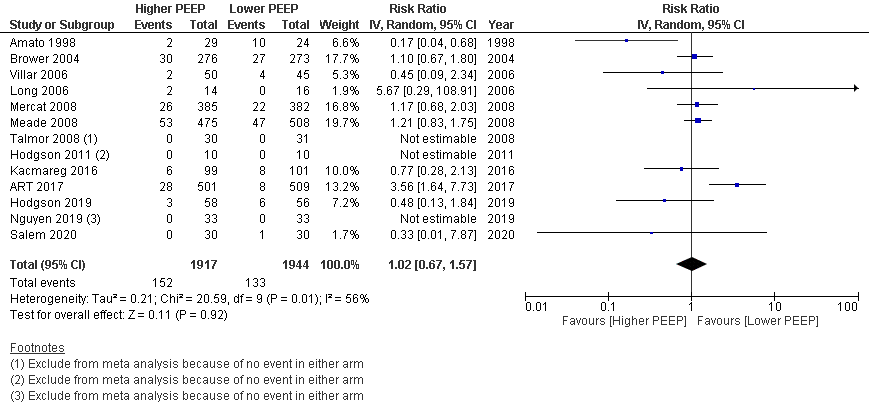


1. Evidence profile

| **Certainty assessment** | | | | | | | **№ of patients** | | **Effect** | | **Certainty** | **Importance** |
| --- | --- | --- | --- | --- | --- | --- | --- | --- | --- | --- | --- | --- |
| **№ of studies** | **Study design** | **Risk of bias** | **Inconsistency** | **Indirectness** | **Imprecision** | **Other considerations** | **Higher PEEP** | **Lower PEEP** | **Relative (95% CI)** | **Absolute (95% CI)** |  |  |
| **Short-term mortality** | | | | | | | | | | | | |
| 15 | Randomized trials | Not serious | Serious^a,b^ | Not serious | Serious^c^ | None | 708/2050 (34.5%) | 755/2058 (36.7%) | RR 0.85 (0.72 to 1.00) | **55 fewer per 1,000** (from 103 fewer to 0 fewer) | ⨁⨁◯◯ Low | CRITICAL |
| **Long-term mortality** | | | | | | | | | | | | |
| 15 | Randomized trials | Not serious | Serious^a,b^ | Not serious | Serious^c^ | None | 849/2050 (41.4%) | 899/2059 (43.7%) | RR 0.88 (0.76 to 1.00) | **52 fewer per 1,000** (from 105 fewer to 0 fewer) | ⨁⨁◯◯ Low | CRITICAL |
| **PaO_2_/F_I_O_2_ ratio at day 1** | | | | | | | | | | | | |
| 14 | Randomized trials | Not serious | Serious^a,b^ | Not serious | Not serious | None | 1976 | 1969 | - | **MD 48.87 mmHg higher** (31.91 higher to 65.83 higher) | ⨁⨁⨁◯ Moderate | IMPORTANT |
| **Ventilator free days at 28** | | | | | | | | | | | | |
| 11 | Randomized trials | Not serious | Very serious^b,d^ | Not serious | Serious^c^ | None | 1493 | 1495 | - | **MD 1.82 days longer** (0.37 shorter to 4.01 longer) | ⨁◯◯◯ Very low | CRITICAL |
| **Length of hospital stay** | | | | | | | | | | | | |
| 6 | Randomized trials | Not serious | Very serious^b,d^ | Not serious | Serious^c^ | None | 1175 | 1217 | - | **MD 0.86 days longer** (3.08 shorter to 4.8 longer) | ⨁◯◯◯ Very low | CRITICAL |
| **Barotrauma** | | | | | | | | | | | | |
| 10 | Randomized trials | Not serious | Very serious^b,d^ | Not serious | Very serious^c,e^ | None | 152/1917 (7.9%) | 133/1944 (6.8%) | RR 1.02 (0.67 to 1.57) | **1 more per 1,000** (from 23 fewer to 39 more) | ⨁◯◯◯ Very low | CRITICAL |
| **ADL/QoL - not reported** | | | | | | | | | | | | |
| 0 | - | - | - | - | - | - | - | - | - | - | - | IMPORTANT |

*Nearest 28-day mortality ** Longest follow-up mortality **CI:** confidence interval; **MD:** mean difference; **RR:** risk ratio; **QoL:** quality of life

#### Explanations

a.Different direction of effect in the study (null and effective)

b.High I2 statistics and heterogeneity test was significant

c.Wide confidence interval

d.Different directions of effect in the study (effective, null, adverse)

e.Did not attain the optimal information size (OIS)

1. Evidence-to-Decision table

| question | |
| --- | --- |
| **CQ20: Should high levels of positive end-expiratory pressure (PEEP) be used for mechanically ventilated adult patients with ARDS?** | |
| **GROUP:** | Adult ARDS patients requiring ventilatory management |
| **INTERVENTIONS:** | Ventilatory management with high PEEP (study-specific definitions) |
| **Comparison and CONTRAST:** | Ventilatory management with low PEEP (study-specific definitions) |
| **Main Outcomes:** | Short-term mortality, long-term mortality, ventilator-free days (VFD), length of hospital stay, barotrauma |
| **SETTING:** | The emergency room or intensive care unit |
| **PERSPECTIVES:** | Personal |
| **BACKGROUND:** | ARDS is an acute and severe condition that affects the structure and function of the lung due to increased permeability and damage to the capillaries surrounding the alveoli (ventilator-induced (associated) lung injury, VILI/VALI). For this reason, the treatment of these patients is based on a lung-protective ventilation strategy. The use of high levels of PEEP is one strategy that aims to reduce VILI/VALI. This review aims to update the assessment of the benefits and harms of high and low levels of PEEP in patients with ALI and ARDS. |
| **Conflict of Interest:** | None |

# assessment

| Problem Is the problem a priority? | | |
| --- | --- | --- |
| judgment | research evidence | note |
| ○ No  ○ Probably no  ○ Probably yes  ● Yes  ○ Varies  ○ Do not know | ARDS is an acute and severe condition that affects the structure and function of the lung by increasing the permeability and damage to the capillaries surrounding the alveoli. The mortality rate for ALI and ARDS is high and mechanical ventilation is often required. However, the use of ventilation can cause VILI /VALI. For this reason, the treatment of these patients is based on a lung-protective ventilation strategy. The use of high levels of PEEP is one strategy that aims to reduce VILI/VALI. This review aims to update the assessment of the benefits and harms of high and low levels of PEEP in patients with ALI and ARDS. We consider this issue to be of high priority. |  |
| Desirable effects How substantial are the desirable anticipated effects? | | |
| judgment | research evidence | remarks |
| ○Trivial  ○Small  ● Moderate  ○Large  ○Varies  ○Do not know | The systematic review found 16 randomized controlled trials (RCTs) consistent with the patient, intervention, comparison, outcomes process (one was excluded because it used extracorporeal membrane oxygenation (ECMO)), and a meta-analysis was performed using them.  The effect estimates for short-term mortality (15 RCTs: N=4108) had a risk difference of 55 fewer deaths/1000 people (95% CI: 103 fewer death to 0 deaths) for “high PEEP” compared with “low PEEP.” The effect estimate for long-term mortality (15 RCTs: N=4109) was 52 fewer patients/1000 (95% CI: 105 fewer to 0 fewer), and the effect estimate for VFD (11 RCTs: N=2988) was a mean difference of 1.82 days longer (95% CI: 0.37 shorter to 4.01 longer). The effect estimates for the length of hospital stay (6 RCTs: N=2392) was a mean difference of 0.86 days (95% CI: 3.08 days shorter to 4.8 days longer). Thus, the desired effect of the intervention was judged to be “moderate.” |  |
| Undesirable effects How substantial are the undesirable anticipated effects? | | |
| judgment | research evidence | note |
| ○Large  ○ Moderate  ○Small  ●Trivial  ○Varies.  ○ Do not know | The effect estimates for barotrauma (10 RCTs: N=2861) had a risk difference of 1 additional person/1000 people (95% CI: 23 fewer to 39 more) for “high PEEP” compared with “low PEEP.” The expected harm was judged to be “trivial.” |  |
| Certainty of evidence What is the overall certainty of the evidence of effects? | | |
| judgment | research evidence | remarks |
| ●Very low  ○Low  ○Moderate  ○High  ○No included studies | **Relative importance or value of the key outcomes of interest**   \| Outcome \| Relative importance \| Certainty of evidence (GRADE) \| \| --- \| --- \| --- \| \| Short-term mortality \| Serious \| ⨁⨁◯◯  low \| \| Long-term mortality \| Serious \| ⨁⨁◯◯  low \| \| VFD \| Serious \| ⨁◯◯◯  Very low \| \| Length of hospital stay \| Serious \| ⨁◯◯◯  Very low \| \| Barotrauma \| Serious \| ⨁◯◯◯  Very low \|   **Overall evidence certainty**:  The direction of desirable and undesirable effects was not consistent, and the certainty of the evidence across outcomes was judged to be “very low.” adopting the certainty of the least certain evidence. |  |
| Values Is there important uncertainty about or variability in how much people value the main outcomes? | | |
| judgment | research evidence | note |
| ○Important uncertainty or variability  ○Possibly important uncertainty or variability  ●Probably no important uncertainty or variability  ○No important uncertainty or variability | There are no data on values for outcomes in ventilatory management with high PEEP, but the value for death is generally high, and the variability is low. |  |
| Balance of effects Does the balance between desirable and undesirable effects favor the intervention or the comparison? | | |
| judgment | research evidence | remarks |
| ○Favors the comparison  ○Probably favors the comparison  ○Does not favor either the intervention or the comparison  ●Probably favors the intervention  ○Favors the intervention  ○Varies  ○Do not know | **Summary of results**   \| Outcome \| Low PEEP (control) \| High PEEP (Intervention) \| Absolute difference (95% CI) \| Relative effect (RR)  (95% CI) \| \| --- \| --- \| --- \| --- \| --- \| \| Short-term mortality \| 755/2058 \| 708/2050 \| 55 fewer/1000 (103 fewer~0 fewer) \| 0.85  (0.72-1.00) \| \| Long-term mortality \| 899/2059 \| 849/2050 \| 52 fewer people/1000 (105 fewer people to 0 fewer people) \| 0.88  (0.76-1.00) \| \| VFD \| - \| - \| MD 1.82 days longer (0.37 days shorter ~ 4.01 days longer) \| - \| \| Length of hospital stay \| - \| - \| MD 0.86 days longer (3.08 days shorter to 4.8 days longer) \| - \| \| Barotrauma \| 133/1944 \| 152/1917 \| 1 person more/1000 (23 people fewer to 39 people more) \| 1.02  (0.67-1.57) \|   The balance of the effects and harms of the intervention was judged to be “probably favors the intervention.” |  |
| Acceptability Is the intervention acceptable to key stakeholders? | | |
| judgment | research evidence | remarks |
| ○ No  ○ Probably no  ● Probably yes  ○ Yes  ○ Varies  ○ Do not know | Although no evidence was found in the studies reviewed, the intervention was judged to be acceptable given the balance of the cost and harms. |  |
| Feasibility Is the intervention feasible to implement? | | |
| judgment | research evidence | remarks |
| ○ No  ○ Probably no  ○ Probably yes  ● Yes  ○ Varies  ○ Do not know | No evidence was included; however, changing the ventilator settings was deemed to be feasible enough. |  |

# Summary of Judgment

|  | JUDGMENT | | | | | | |
| --- | --- | --- | --- | --- | --- | --- | --- |
| **PROBLEM** | No | Probably no | Probably yes | Yes |  | Varies | Do not know |
| **DESIRABLE EFFECTS** | Trivial | Small | Moderate | Large |  | Varies | Do not know |
| **UNDESIRABLE EFFECTS** | Large | Moderate | Small | Trivial |  | Varies | Do not know |
| **CERTAINTY OF EVIDENCE** | Very low | Low | Moderate | High |  |  | No included studies |
| **VALUES** | Important uncertainty or variability | Possibly important uncertainty or variability | Probably no important uncertainty or variability | No important uncertainty or variability |  |  |  |
| **BALANCE OF EFFECTS** | Favors the comparison | Probably favors the comparison | Does not favor either the intervention or the comparison | Probably favors the intervention | Favors the intervention | Varies | Do not know |
| **ACCEPTABILITY** | No | Probably no | Probably yes | Yes |  | Varies | Do not know |
| **FEASIBILITY** | No | Probably no | Probably yes | Yes |  | Varies | Do not know |

# Type of recommendation

| Strong recommendation against the intervention | Conditional recommendation against the intervention | Conditional recommendation for either the intervention or control | Conditional recommendation for the intervention | Strong recommendation for the intervention |
| --- | --- | --- | --- | --- |
| ○ | ○ | ○ | ● | ○ |

# Conclusion

| Recommendation |
| --- |
| **Conditional recommendation to use higher PEEP in ventilated adult patients with ARDS**  **(Conditional recommendation/evidence of very low certainty: GRADE2D).**  **Supplementary item**  **In the included studies, the PEEP values on day 1 in the intervention group ranged from about 10 to 16.3 cmH_2_O, and in the control group, the PEEP values on day 1 ranged from 6.5 to 12.0 cmH_2_O. The most common methods of setting PEEP in the intervention group were Pflex* and the ARDSnet PEEP table** (see implementation considerations). See also CQ19 for which PEEP setting method to use.**  ***Pressure at a lower inflection point in the pressure-volume curve**  ***http://www.ardsnet.org/files/ventilator_protocol_2008-07.pdf** |
|  |
| Reason/Justification |
| **Question**: Should high levels of positive end-expiratory pressure (PEEP) be used for mechanically ventilated adult patients with ARDS?  **Patient**: Ventilated adult patient with ARDS  **Intervention**: Ventilatory management with high PEEP (defined per study)  **Comparison control**: Ventilatory management with low PEEP (defined per study)  **Outcomes**: Short-term mortality, long-term mortality, VFD, length of hospital stay, barotrauma  **Summary of evidence:**  Using higher PEEP, short-term mortality (15 RCTs: N=4108) was reduced by 55 per 1000 (95% CI: 103 fewer to 0 fewer), long-term mortality (15 RCTs: N=4109) was reduced by 52 per 1000 (95% CI: 105 fewer to 0 fewer), VFD (11 RCTs: N=2988) was prolonged by a mean of 1.82 days (95% CI: 0.37 days shorter to 4.01 days longer), and length of hospital stay (6 RCTs: N=2392) was prolonged by a mean of 0.86 days (95% CI: 3.08 days shorter to 4.8 days longer). Therefore, the expected desirable effect was judged to be “moderate.” On the contrary, the number of patients with barotrauma (10 RCTs, N=2861) increased by 1 per 1000 (95% CI: 23 fewer patients to 39 more patients). Therefore, the undesirable effect was judged to be “small.” Thus, the balance of effects and harms of the intervention was judged to be “probably large.”  **Certainty of evidence**:  The direction of the desirable and undesirable effects was not consistent, and the certainty of the evidence across outcomes was judged to be “very low.” adopting the certainty of the least certain evidence.  **Determining values, balance of effects, acceptance, and viability:**  The costs were judged to be “small” compared with the benefits because the burden on patients and the increase in costs due to the high PEEP setting were considered small.  **Panel discussion**  In the pre-vote, the modified Delphi method resulted in a median score of 8.0 and a disagreement index of 0.1316 for “Propose the use of high PEEP in ventilated adult patients with ARDS (weak recommendation/very low certainty evidence: GRADE2D).” At the panel meeting, there was a discussion that the method for setting high PEEP should be specifically described so that general practitioners easily understand it. The panel meeting finally reached a consensus with the results of the pre-vote without a re-vote.  **Additional Considerations:**  The following table proposed by the ARDS network is often used to determine low and high PEEP.   \| **Low PEEP / F_I_O_2_ Table** \| \| \| \| \| \| \| \| \| \| --- \| --- \| --- \| --- \| --- \| --- \| --- \| --- \| --- \| \| F_I_O_2_ \| 0.3 \| 0.4 \| 0.5 \| 0.6 \| 0.7 \| 0.8 \| 0.9 \| 1.0 \| \| PEEP (cmH2O_)_ \| five \| 5-8 \| 8-10 \| 10 \| 10-12 \| 12-14 \| 18 \| 18-24 \|  \| **High PEEP/ F_I_O_2_Table** \| \| \| \| \| \| \| \| \| \| --- \| --- \| --- \| --- \| --- \| --- \| --- \| --- \| --- \| \| F_I_O_2_ \| 0.3 \| 0.4 \| 0.5 \| 0.6 \| 0.7 \| 0.8 \| 0.9 \| 1.0 \| \| PEEP (cmH2O_)_ \| 5-14 \| 14-16 \| 16-18 \| 18-20 \| 18-20 \| 20-22 \| 22 \| 22-24 \| |

| Subgroup considerations |
| --- |
| In the present study, we performed a subgroup analysis divided into a mild disease group (P/F ratio > 200) and a moderate to severe disease group (P/F ratio ≤ 200). Short-term mortality in the mild disease group decreased by 24 per 1000 (95% CI: 56 fewer to 12 more), long-term mortality decreased by 25 per 1000 (95% CI: 58 fewer to 15 more), and short-term mortality in the moderate to severe group decreased by 93 per 1000 (95% CI: 181 fewer to 23 more), and long-term mortality decreased by 103 per 1000 (95% CI: 195 fewer to 16 more). Although none of these differences was significant, there was a more positive trend for the high PEEP group. The point estimates also showed a more desirable direction in the moderate to severe group. Other meta-analyses^3)^, including the LOVS^1)^ and EXPRESS^2)^ studies, have also suggested the benefit of a high PEEP strategy in the moderate to severe group; thus, a high PEEP strategy may be considered in patients with a P/F ratio ≤200.  　On the contrary, several studies comparing high PEEP with low PEEP used low PEEP and high tidal volumes in the control group. Since this may have affected the results, we performed a sensitivity analysis using only those studies that used low PEEP and low tidal volumes in the control group. As a result, the point estimates were close to zero for many outcomes. As this result has been shown in other meta-analyses^4)^, we cannot exclude the possibility that the benefit of high PEEP was influenced by the harm of high tidal volume ventilation in the control group rather than by high PEEP itself.  Further verification, including the above items, is necessary. |
| Implementation considerations |
| The 2016 Japanese ARDS guideline recommendation stated, “When performing ventilation in adult patients with ARDS, we suggest that PEEP values be set within the range of plateau pressures of 30 cmH_2_O or lower and within the range that does not affect circulatory dynamics.” (GRADE 2B, Strength of recommendation: “Weak recommendation” / Confidence of evidence: “Moderate”). We also suggest that higher PEEP should be used for moderate to severe ARDS. (GRADE 2B, strength of recommendation: “Weak recommendation” / confidence in evidence: “Moderate”). This time, the certainty of the evidence may have changed because we included studies that showed a significant difference in PEEP between the two groups as a result of different intervention, 16 RCTs were recruited which was significantly more than the previous guideline, and the method of assessing the certainty of the evidence was different.  In addition, it is necessary to monitor the effects of high PEEP on circulatory dynamics. |

| Monitoring and evaluation |
| --- |
| After implementing the recommendations, information on clinical problems such as heart failure caused by high PEEP should be monitored. It is also necessary to monitor the implementation status of the guideline through questionnaires and other means after the publication of the guideline to see if any other clinical problems arise. |
| Research priorities |
| The above subgroups suggest the need for high-quality randomized controlled trials comparing high PEEP strategies with low PEEP strategies in patients with moderate to severe ARDS while maintaining low tidal volume.  The method for determining the optimal PEEP value for each patient is unclear and remains to be investigated. |

References

1) Meade MO, Cook DJ, Guyatt GH, et al. Lung open ventilation study investigators. Ventilation strategy using low tidal volumes, recruitment maneuvers, and high positive end-expiratory pressure for acute lung injury and acute respiratory distress syndrome: a randomized controlled trial. JAMA. 2008;299(6): 637-45. PMID: 23740697.

2) Mercat A, Richard JC, Vielle B, et al. Expiratory pressure (express) study group. Positive end-expiratory pressure setting in adults with acute lung injury and acute respiratory distress syndrome: a randomized controlled trial. JAMA. 2008;299(6):646-55. PMID: 18270353.

3) Briel M, Meade M, Mercat A, et al. Higher vs lower positive end-expiratory pressure in patients with acute lung injury and acute respiratory distress syndrome systematic review and Meta-analysis. JAMA. 2010;303(9):865-73. PMID: 20197533.

4) [Walkey](https://pubmed.ncbi.nlm.nih.gov/?sort=date&term=Walkey+AJ&cauthor_id=29043834) A, [Sorbo](https://pubmed.ncbi.nlm.nih.gov/?sort=date&term=Del+Sorbo+L&cauthor_id=29043834) L, [Hodgson](https://pubmed.ncbi.nlm.nih.gov/?sort=date&term=Hodgson+CL&cauthor_id=29043834) C, et al. Higher PEEP versus lower PEEP strategies for patients with acute respiratory distress syndrome: a systematic review and meta-analysis. Ann Am Thorac Soc. 2017;14(Suppl 4):S297-S303. PMID: 29043834.

**CQ21 Should plateau pressure be limited for mechanically ventilated adult patients with ARDS?**

1. Search strategy

MEDLINE via PubMed （Search date: 2020/7/23）

| #1 | Respiratory distress syndrome, adult[mh] OR adult respiratory distress syndrom*[tiab] OR acute respiratory distress syndrom*[tiab] OR shock lung[tiab] OR ARDS*[tiab] |
| --- | --- |
| #2 | lung injury[mh] OR lung injur*[tiab] OR ALI[tiab] |
| #3 | acute[tiab] AND lung[tiab] AND (fail*[tiab] OR depression[tiab]) |
| #4 | ALI[tiab] OR Acute lung injur*[tiab] OR Ventilator-Induced Lung Injury[tiab] |
| #5 | Acute [tiab] AND (respirat*[tiab] OR ventilat*[tiab] OR pulmon*[tiab]) AND (fail*[tiab] OR depression[tiab]) |
| #6 | Acute chest syndrome[mh] OR (acute[tiab] AND chest[tiab] AND syndrom*[tiab]) |
| #7 | #1 OR #2 OR #3 OR #4 OR #5 OR #6 |
| #8 | Respiration,artificial[mesh: noexp] OR Positive-Pressure Respiration[mh] OR Artificial respiration[tiab] OR Artificial ventilation[tiab] |
| #9 | Ventilators, Mechanical[mh] OR Mechanical ventilation[tiab] |
| #10 | pressure[tiab] AND (limit*[tiab] OR low[tiab] OR lower[tiab] OR less[tiab]) |
| #11 | (#8 OR #9) AND #10 |
| #12 | Tidal volume[mh] OR tidal volume*[tiab] |
| #13 | plateau pressure*[tiab] |
| #14 | LPVS [tiab] OR lung protective* [tiab] OR (protective [tiab] AND ventilat* [tiab]) |
| #15 | #11 OR #12 OR #13 OR #14 |
| #16 | #7 AND #15 |
| #17 | randomized controlled trial[pt] OR controlled clinical trial[pt] OR randomized[tiab] OR placebo[tiab] OR clinical trials as topic[mesh: noexp] OR randomly[tiab] OR trial[ti] |
| #18 | animals[mh] NOT humans[mh] |
| #19 | #17 NOT #18 |
| #20 | #16 AND #19 |

CENTRAL （Search date: 2020/7/24）

| #1 | [mh "Respiratory distress syndrome, adult"] OR "adult respiratory distress syndrome":ti,ab OR "acute respiratory distress syndrome":ti,ab OR "shock lung":ti,ab OR ARDS:ti,ab |
| --- | --- |
| #2 | [mh "lung injury"] OR "lung injury":ti,ab OR ALI:ti,ab |
| #3 | acute:ti,ab AND lung:ti,ab AND (failure:ti,ab OR depression:ti,ab) |
| #4 | ALI:ti,ab OR "Acute lung injury":ti,ab OR "Ventilator-Induced Lung Injury":ti,ab |
| #5 | Acute:ti,ab AND (respiratory:ti,ab OR ventilatory:ti,ab OR pulmonary:ti,ab) AND (failure:ti,ab OR depression:ti,ab) |
| #6 | [mh "Acute chest syndrome"] OR (acute:ti,ab AND chest:ti,ab AND syndrome:ti,ab) |
| #7 | {OR #1-#6} |
| #8 | [mh "Respiration, artificial"] OR "Artificial respiration":ti,ab OR "Artificial ventilation":ti,ab |
| #9 | [mh "Ventilators, Mechanical"] OR "Mechanical ventilation":ti,ab |
| #10 | pressure:ti,ab AND (limited:ti,ab OR low:ti,ab OR lower:ti,ab OR less:ti,ab) |
| #11 | (#8 OR #9) AND #10 |
| #12 | [mh "Tidal volume"] OR "tidal volume":ti,ab |
| #13 | "plateau pressure":ti,ab |
| #14 | LPVS:ti,ab OR "lung protective":ti,ab OR (protective:ti,ab AND ventilation:ti,ab) |
| #15 | {OR #11-#14} |
| #16 | #7 AND #15 |
| #17 | [mh animals] NOT [mh humans] |
| #18 | #16 NOT #17 |

Igaku-Chuo-Zasshi (Search date: 2020/7/16）

| #1 | 呼吸窮迫症候群-急性/TH or 成人呼吸窮迫症候群/TA or 成人型呼吸窮迫症候群/TA or 成人呼吸促迫症候群/TA or 成人型呼吸促迫症候群/TA or 急性呼吸窮迫症候群/TA or 急性呼吸促迫症候群/TA or ショック肺/TA or ARDS/TA |
| --- | --- |
| #2 | 肺損傷/TH or 肺損傷/TA or 肺傷害/TA or 肺障害/TA |
| #3 | 急性/TA and 肺/TA and (不全/TA or 低下/TA or 抑制/TA) |
| #4 | 呼吸不全/TH or 呼吸不全/TA or 呼吸機能不全/TA or 換気不全/TA or 肺機能不全/TA |
| #5 | 急性/TA and (呼吸/TA or 換気/TA or 肺機能/TA) and (不全/TA or 低下/TA or 抑制/TA) |
| #6 | 急性胸部症候群/TH or (急性/TA and 胸部/TA and 症候群/TA) |
| #7 | #1 OR #2 OR #3 OR #4 OR #5 OR #6 |
| #8 | 人工呼吸/TH or 人工呼吸/TA or 陽圧呼吸/TA or 人工換気/TA or 陽圧換気/TA or 機械的換気/TA |
| #9 | 人工呼吸器/TH or 人工呼吸器/TA or レスピレータ/TA or ベンチレータ/TA |
| #10 | 圧/TA and (制限/TA or 低/TA or 減/TA) |
| #11 | ( #8 OR #9) and #10 |
| #12 | プラトー圧/TA |
| #13 | 肺保護戦略/TA or 肺保護/TA or (保護/TA and 換気/TA) |
| #14 | #11 or #12 or #13 |
| #15 | ランダム化比較試験/TH or 準ランダム化比較試験/TH or ランダム/AL and 化/AL or 無作為化/AL or 比較試験/AL or 臨床試験/AL or プラセ/AL and ボ/AL or 対照/AL or コントロール/AL or 臨床研究/AL |
| #16 | (CK=動物) not (CK=ヒト) |
| #17 | #15 not #16 |
| #18 | #7 and #14 and #17 |
| #19 | (#18) and (PT=会議録除く) |

1. Flow diagram

**Identification**

Duplicates

n=558

26 Full-text articles assessed for eligibility (Attempt to order full text)

2264 records after duplicates removed

2822 records identified through database searching

2822 records identified through database searching

Medline via PubMed n=1362

Cochrane CENTRAL n=1335

Ichu-shi n=125

0 additional records identified through other sources

6 Studies included in quantitative synthesis (meta-analysis)

Short-term mortality:n=4　Long-term mortality:n=3

VFD:n=3 VALI:n=5

**Screening**

**Eligibility**

**Included**

17 Full-text articles excluded, with reasons:

Wrong language (n=3)

Wrong study design (n=6)

Wrong population (n=2)

Wrong intervention or comparison (n=5)

Incomplete study (n=1)

10 Studies included in qualitative synthesis

532 records excluded

1. Risk of bias

Short-term mortality Long-term mortality

Ventilator-free days VALI

1. Forest plot

Short-term mortality


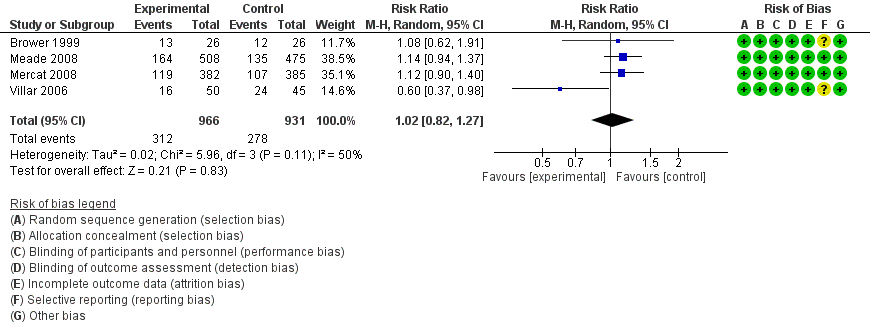


Long-term mortality


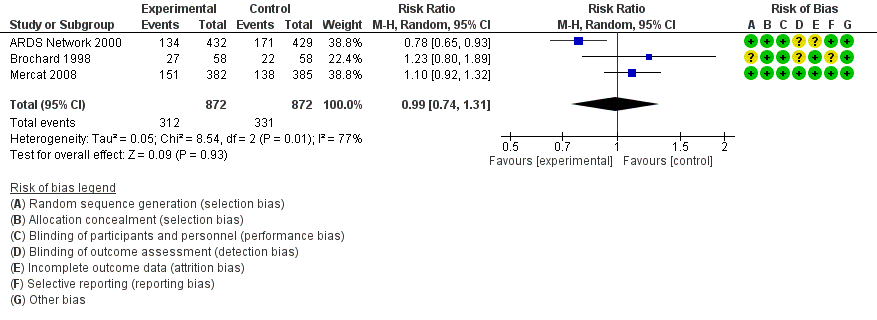


Ventilator-free days


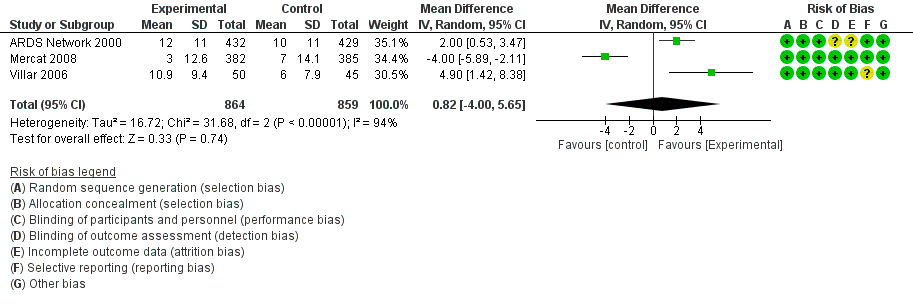


VALI


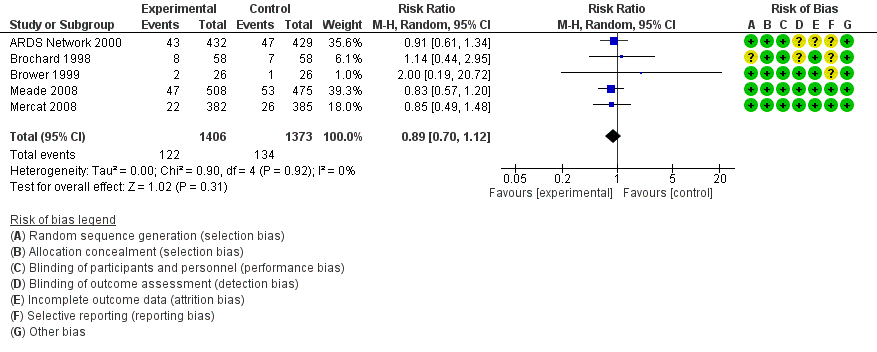


1. Evidence profile

| **Certainty assessment** | | | | | | | **№ of patients** | | **Effect** | | **Certainty** | **Importance** |
| --- | --- | --- | --- | --- | --- | --- | --- | --- | --- | --- | --- | --- |
| **№ of studies** | **Study design** | **Risk of bias** | **Inconsistency** | **Indirectness** | **Imprecision** | **Other considerations** | **Limited plateau pressure** | **High or unlimited plateau pressure** | **Relative (95% CI)** | **Absolute (95% CI)** |  |  |
| **Short-term mortality*** | | | | | | | | | | | | |
| 4 | Randomized trials | Not serious | Not serious | Not serious | Serious ^a^ | None | 312/966 (32.2%) | 278/931 (29.9%) | **RR 1.02** (0.82 to 1.27) | **6 more per 1000** (54 fewer to 81 more) | ⨁⨁⨁◯ Moderate | Critical |
| **Long-term mortality**** | | | | | | | | | | | | |
| 3 | Randomized trials | Not serious | Serious ^b^ | Not serious | Very Serious ^c^ | None | 312/872 (35.8%) | 331/872 (38.0%) | **RR 0.99** (0.74 to 1.31) | **4 fewer per 1000** (99 fewer to 118 more) | ⨁◯◯◯ Very low | Critical |
| **Ventilator free days***** | | | | | | | | | | | | |
| 3 | Randomized trials | Not serious | Very Serious ^d^ | Not serious | Serious ^e^ | None | 864 | 859 | - | **MD 0.82 days longer** (4 shorter to 5.65 longer) | ⨁⨁◯◯ Low | Critical |
| **Ventilator-associated lung injury** | | | | | | | | | | | | |
| 5 | Randomized trials | Not serious | Not serious | Not serious | Serious ^f^ | None | 122/1406 (8.7%) | 134/1373 (9.7%) | **RR 0.89** (0.70 to 1.12) | **11 fewer per 1000** (30 fewer to 12 more) | ⨁⨁⨁◯ Moderate | IMPORTANT |
| **Death due to hypercapnia** | | | | | | | | | | | | |
| 0 |  |  |  |  |  |  |  |  | Impossible to estimate | - | - | Critical |
| **Dialysis introduction rate due to hypercapnia** | | | | | | | | | | | | |
| 0 |  |  |  |  |  |  |  |  | Impossible to estimate | - | - | Important |

*28-day mortality, In-hospital mortality　**60-day mortality, 180-day mortality　***Ventilator free days in 28 days

**CI:** confidence interval; **RR:** risk ratio

#### Description

a. The total sample size of 1897 meets the optimal information size (OIS); however, clinical decisions can vary at the upper and lower limits of the 95% confidence interval due to its width. Therefore, the overall imprecision was judged as” Serious” to be one level down.

b. The Inconsistency is one level down because the statistical heterogeneity is significantly large as I^2=86%.

c. The total sample size of 1744 meets the OIS; however, clinical decisions can vary at the upper and lower limits of the 95% confidence interval due to its width. Therefore, the overall imprecision is judged as” Very Serious” to be two-level down.

d. The inconsistency is two-level down because the statistical heterogeneity was highly significant as I^2=94%, and because the clinical heterogeneity was also judged to be significant as two studies indicated obvious benefits while one reported obvious harm.

e. The total sample size of 1723 meets the OIS; however, the confidence interval straddles between -4 to +5.65 days, which was considered to exceed the minimum important difference (MID). Therefore, the valuation was one level down.

f. The number of events of 134 in the control group does not meet the OIS, and also clinical decisions can at the upper and lower limits of the 95% confidence interval due to its width. Therefore, the overall imprecision is judged as” Serious” to be one level down.

1. Evidence-to-Decision table

| Question | |
| --- | --- |
| **CQ21：Should plateau pressure be limited for mechanically ventilated adult patients with ARDS?** | |
| **GROUP:** | Adults patients with ARDS requiring mechanical ventilatory management |
| **INTERVENTIONS:** | Mechanical ventilation with plateau pressure limitation |
| **Comparison and CONTRAST:** | Mechanical ventilation without plateau pressure limitation |
| **Main Outcomes:** | Short-term mortality, long-term mortality, ventilator-free days (VFD), length of hospital stay, barotrauma, death due to hypercapnia |
| **SETTING:** | The emergency room or intensive care unit |
| **PERSPECTIVES:** | Personal |
| **BACKGROUND:** | In the mechanical ventilatory management of adult patients with ARDS, it is deeply concerning that ventilator-associated lung injury (VALI) leads to more ventilator days and an increased risk of mortality. The elevation of airway pressure is assumed to be one factor of VALI, so the limitation of plateau pressure is expected to suppress VALI. On the contrary, limitation of plateau pressure may lead to adverse events such as hypercapnia. It is necessary to clarify the effects and adverse events of plateau pressure limitation. |
| **Conflict of Interest:** | None |

# Assessment

| Problem Is the problem a priority? | | | |
| --- | --- | --- | --- |
| Judgment | Research Evidence | | Remarks |
| ○ No  ○ Probably no  ○ Probably yes  ● Yes  ○ Varies  ○ Do not know | In the mechanical ventilatory management of adult patients with ARDS, it is deeply concerning that VALI leads to more ventilator days and an increased risk of mortality. The elevation of airway pressure is assumed to be one factor of VALI, and so the limitation of plateau pressure is expected to suppress VALI. On the other hand, limitation of plateau pressure may lead to adverse events such as hypercapnia. It is necessary to clarify the effects and the adverse events of plateau pressure limitation. Therefore, this topic is clinically significant. | |  |
| Desirable effects How substantial are the desirable anticipated effects? | | | |
| Judgment | Research Evidence | | Remarks |
| ●Trivial  ○Small  ○ Moderate  ○Large  ○Varies  ○Do not know | As a result of the systematic review, 6 randomized controlled trials (RCTs) matching the patient, intervention, comparison, and outcome process were identified and meta-analyzed.  As beneficial outcomes, the estimated effects in the intervention group compared to the control group were: short-term mortality (4 RCTs: N=1897) with a risk difference of 6 more death /1000 patients (95% confidence interval: 54 fewer to 81 more), long-term mortality (4 RCTs: N=1774) with a risk difference of 4　fewer death /1000 patients (95% confidence interval: 99 fewer to 118 more), VFD (3 RCTs: N=1723) with an mean difference of 0.82 longer (95% confidence interval: 4 days shorter to 5.65 longer days), and barotrauma (5 RCTs: N=2779) with a mean difference of 11 fewer /1000 patients (95% confidence interval: 30 fewer to 12 more). Overall, the effect of the intervention was judged to be “trivial.” | |  |
| Undesirable effects How substantial are the undesirable anticipated effects? | | | |
| Judgment | Research Evidence | Remarks | |
| ○Large  ○ Moderate  ○Small  ●Trivial  ○Varies.  ○ Do not know | Death due to hypercapnia was considered undesirable, but no studies were included that reported this issue. Combined with the description of remarks, the quality of evidence was deemed to be very low, and the harm is judged to be “trivial.” | In consideration of the literature (Meade 2008) included in this systematic review, refractory acidosis (42/503 patients) and deaths due to refractory acidosis (38/508 patients) were reported as adverse events. | |
| Certainty of evidence What is the overall certainty of the evidence of effects? | | | |
| Judgment | Research Evidence | | Remarks |
| ●Very low  ○Low  ○Moderate  ○High  ○No included studies | The relative importance and values of the key outcomes of interest.   \| Outcome \| Relative importance \| Certainty of Evidence (GRADE) \| \| --- \| --- \| --- \| \| \| \| Short-term mortality \| Critical \| ⨁⨁⨁◯ \| \| Moderate \| \| Long-term mortality \| Critical \| ⨁◯◯◯ \| \| Very low \| \| VFD \| Critical \| ⨁⨁◯◯ \| \| Low \| \| Barotrauma \| Critical \| ⨁⨁⨁◯ \| \| Moderate \| \| Death due to hypercapnia \| Critical \| - \| \| - \|   There was no report of death due to hypercapnia within the included studies.  **Overall evidence certainty:**  The direction within the desired effect was not consistent; therefore, the certainty of evidence across the outcomes was judged to be “very low” adopted from the lowest certainty of evidence. | |  |
| Values Is there important uncertainty about or variability in how much people value the main outcomes? | | | |
| Judgment | Research Evidence | | Remarks |
| ○Important uncertainty or variability  ○Possibly important uncertainty or variability  ○Probably no important uncertainty or variability  ●No important uncertainty or variability | Although there is no data on the values concerning outcomes on mechanical ventilatory management with plateau pressure limitation, the value on death is generally high, and the dispersion is considered small. | |  |
| Balance of effects Does the balance between desirable and undesirable effects favor the intervention or the comparison? | | | |
| Judgment | Research Evidence | | Remarks |
| ○Favors the comparison  ○Probably favors the comparison  ○Does not favor either the intervention or the comparison  ○Probably favors the intervention  ○Favors the intervention  ○Varies  ●Do not know | **Summary of Findings**   \| Outcome \| Without plateau pressure limitation  (control) \| With plateau pressure limitation (intervention) \| Absolute difference  (95% CI) \| Relative effect  (RR) (95% CI) \| \| --- \| --- \| --- \| --- \| --- \| \| Short-term mortality \| 278/931 \| 312/966 \| 6 more /1000 (-54-81) \| 1.02 (0.82 - 1.27) \| \| Long-term mortality \| 331/872 \| 312/872 \| -4 /1000 (-99-118) \| 0.99  (0.74 - 1.31) \| \| VFD \| - \| - \| MD 0.82 days longer  （-4-5.65） \| - \| \| Barotrauma \| 134/1373 \| 122/1406 \| -11/1000  (-3-12) \| 0.89 (0.70 - 1.12) \| \| Death due to hypercapnia \| - \| - \| Impossible to estimate \| - \|   There was no report of death due to hypercapnia within the studies included.  The balance between desirable and undesirable effects was judged to be “do not know.” | |  |
| Acceptability Is the intervention acceptable to key stakeholders? | | | |
| Judgment | Research Evidence | | Remarks |
| ○ No  ○ Probably no  ○ Probably yes  ● Yes  ○ Varies  ○ Do not know | There was no evidence presented in the studies. However, plateau pressure limitation is already used in daily clinical practice. It was assumed to be fully implementable even considering the costs and the harms. Therefore, it was judged to be “Yes.” | |  |
| Feasibility Is the intervention feasible to implement? | | | |
| Judgment | Research Evidence | | Remarks |
| ○ No  ○ Probably no  ○ Probably yes  ● Yes  ○ Varies  ○ Do not know | There was no evidence presented in the studies. However, plateau pressure limitation is already used in daily clinical practice, so it was assumed that it could be implemented. | |  |

# Summary of Judgment

|  | | JUDGMENT | | | | | | |  |
| --- | --- | --- | --- | --- | --- | --- | --- | --- | --- |
| **PROBLEM** | No | | Probably no | Probably yes | Yes |  | Varies | Do not know | |
| **DESIRABLE EFFECTS** | Trivial | | Small | Moderate | Large |  | Varies | Do not know | |
| **UNDESIRABLE EFFECTS** | Large | | Moderate | Small | Trivial |  | Varies | Do not know | |
| **CERTAINTY OF EVIDENCE** | Very low | | Low | Moderate | High |  |  | No included studies | |
| **VALUES** | Important uncertainty or variability | | Possibly important uncertainty or variability | Probably no important uncertainty or variability | No important uncertainty or variability |  |  |  | |
| **BALANCE OF EFFECTS** | Favors the comparison | | Probably favors the comparison | Does not favor either the intervention or the comparison | Probably favors the intervention | Favors the intervention | Varies | Do not know | |
| **ACCEPTABILITY** | No | | Probably no | Probably yes | Yes |  | Varies | Do not know | |
| **FEASIBILITY** | No | | Probably no | Probably yes | Yes |  | Varies | Do not know | |

# type of recommendation

| Strong recommendation against the intervention | Conditional recommendation against the intervention | Conditional recommendation for either the intervention or the comparison | Conditional recommendation for the intervention | Strong recommendation for the intervention |
| --- | --- | --- | --- | --- |
| ○ | ○ | ○ | ● | ○ |

# CONCLUSION

| Recommendation |
| --- |
| **It is recommended with conditions that plateau pressure limitation is implemented in the management of mechanical ventilation for adult patients with ARDS (Conditional recommendation/ Very low evidence of certainty: GRADE 2D).**  **Supplementary items:**  **Desirable effects do not necessarily surpass the undesirable effects in this evaluation. Therefore, it is not certain that high plateau pressure is always harmful under the condition of appropriate limitation of tidal volume and transpulmonary pressure.** |
|  |
| Justification |
| **Question:** Should plateau pressure be limited for mechanically ventilated adult patients with ARDS?  **Patients:** Adult patients with ARDS that require mechanical ventilatory management  **Intervention:** Mechanical ventilation with plateau pressure limitation  **Comparison control:** Mechanical ventilation without plateau pressure limitation  **Outcome:** Short-term mortality, long-term mortality, VFD, length of hospital stay, barotrauma, death due to hypercapnia (including refractory acidosis and death).  **Summary of Evidence:**  As a result of the systematic review, 6 RCTs were identified, which compared plateau pressure limitation and non-limitation. In the mechanical ventilatory management with plateau pressure limitation, short-term mortality (4 RCTs: N=1897) with a risk difference of 6 more /1000 patients (95% confidence interval: -54-81), long-term mortality (4 RCTs: N=1774) with a risk difference of -4 /1000 patients (95% confidence interval: -99 - 118), VFD (3 RCTs: N=1723) with an mean difference of 0.82 more days (95% confidence interval: -4-5.65 days), and barotrauma (5 RCTs: N=2779) with a risk difference of -11 /1000 patients (95% confidence interval: -30-12) as beneficial outcomes. Therefore, the effect of the intervention was judged to be “only.”  On the contrary, there was no report of deaths due to hypercapnia as an undesirable effect. The RCT by Meade (2008) identified several adverse events, including refractory acidosis (42/503 patients) and deaths due to refractory acidosis (38/508 patients). Therefore, the undesirable effect of the intervention was judged to be “only.” The balance between desirable and undesirable effects was judged to be “Unknown.”  **Certainty of Evidence:**  The direction within the desired effect was inconsistent. Therefore, the certainty of evidence across all outcomes was judged to be “Very low” adopted from the lowest certainty of evidence.  **Determining the balance of effects, acceptability, feasibility:**  The intervention of plateau pressure limitation is the only setting that can be changed in mechanical ventilation and is quite common in daily clinical practice. It is assumed to be fully acceptable and feasible.  **Panel meeting:**  The pre-voting results by the modified Delphi method were a median of 8.0 points and a disagreement index of 0.2118 for “It is recommended that plateau pressure limitation is implemented in the management of mechanical ventilation for adult patients with ARDS. (Weak recommendation/ Very low evidence of certainty: GRADE 2D).”  Consequently, the panel meeting reached the final agreement of the same results of the pre-voting without voting again.  **Additional considerations:**  The research used for the meta-analysis can be divided into two groups:   1. Studies that compared low and high tidal volume causing plateau pressure differences (Brochard 1998, Brower 1999, Roy 2000 (ARDS Network 2000), and Villar 2006). 2. Studies that set the equivalent tidal volume and caused plateau pressure differences by high positive end-expiratory pressure (PEEP) (Meade 2008, Mercat 2008).   In the following subgroup analysis, subgroup 1 showed a tendency for better prognosis in the lower plateau pressure group (intervention group), while subgroup 2 showed a worse prognosis. This indicates that limitation of plateau pressure does not necessarily lead to improved prognoses. We should pay attention to the fact that plateau pressure consists of PEEP and driving pressure. |

| Subgroup considerations |
| --- |
| We divided the studies used for comparison of plateau pressure into the following two groups and performed a subgroup analysis:   1. The difference in plateau pressure was caused by low and high tidal volumes. 2. The difference in plateau pressure was caused by low and high PEEP with equivalent tidal volumes.   The results were as follows: in subgroup 1, short-term mortality -106/1000 (-284-208), long-term mortality -24/1000 (-159-178), VFD 3 more days (0.3-5.71 days), barotrauma -4/1000 (-35 - 40), in subgroup 2, short-term mortality 37 more/1000 (-6-84), long-term mortality 36 more/1000 (-29-115), VFD 3 less days (-5.89-2.11 days), barotrauma -15/1000 (-36-13).  The intervention group (low plateau pressure group) of subgroup 1 and the control group (high plateau pressure group) of subgroup 2 showed significantly longer VFD. Furthermore, the other outcomes of the point estimate values showed a tendency of superiority for the intervention group (low plateau pressure group) of subgroup 1 and the control group (high plateau pressure group) of subgroup 2. |
| Implementation considerations |
| There are no concerns about implementing plateau pressure limitation as it is frequently used in daily clinical practice. Nevertheless, there must be some situations where lowering the plateau pressure can disturb the maintenance of an adequate ventilatory volume resulting in the retention of carbon dioxide, decreased oxygenation, and increased respiratory workload, along with increased plateau pressure to some extent.   \| Monitoring and evaluation \| \| --- \| \| On implementation of this recommendation, more information about the complications of plateau pressure limitation should be collected to highlight any clinical problems. Besides, monitoring is necessary about the implementation status through questionnaires after the publication of guidelines if there are any other clinical problems. \| \| Research priorities \| \| Limitation of plateau pressure for ARDS was strongly recommended after the ARMA study^1)^ in 2000. However, in many studies that examined plateau pressures, a high tidal volume was used in control groups. As the subgroup analysis above indicated, a high tidal volume in the control group might affect the prognoses. A large-scale RCT is expected in which plateau pressures are compared (>30 cmH_2_O vs. ≦30 cmH_2_O) under the condition that driving pressure (tidal volume) is equivalently limited. \|   Moreover, adding to general monitoring (electrocardiogram, SpO_2_ monitoring), measurement of end-tidal carbon dioxide partial pressure should be monitored since carbon dioxide partial pressure in the blood tends to rise under the limitation of plateau pressure. |

References

1) [Brower R](https://pubmed.ncbi.nlm.nih.gov/?sort=date&term=Brower+RG&cauthor_id=10793162), [Matthay M](https://pubmed.ncbi.nlm.nih.gov/?sort=date&term=Matthay+MA&cauthor_id=10793162), [Morris A, et al.](https://pubmed.ncbi.nlm.nih.gov/?sort=date&term=Morris+A&cauthor_id=10793162) Ventilation with lower tidal volumes as compared with traditional tidal volumes for acute lung injury and the acute respiratory distress syndrome. N Engl J Med. 2000;342(18):1301-8. PMID: 10793162

**CQ22 Which, between pressure-control ventilation (PCV) and volume-control ventilation (VCV), is desirable for mechanical ventilation in adult patients with ARDS?**

1. Search strategy

MEDLINE via PubMed （Search date: 2020/7/8）

| #1 | respiratory distress syndrome, adult[mh] OR acute respiratory distress syndrom*[tiab] OR respiratory insufficiency[mh] OR acute lung injury[mh] OR acute lung injur*[tiab] OR acute respiratory failure*[tiab] OR ALI[tiab] OR ARDS[tiab] |
| --- | --- |
| #2 | Respiration, Artificial[mh] OR artificilal respiration*[tiab] OR Pulmonary Ventilation[mh] OR pulmonary ventilat*[tiab] OR Ventilators, Mechanical[mh] OR mechanical ventilat*[tiab] OR positive-pressure respiration*[tiab] OR positive pressure ventilat*[tiab] |
| #3 | pressure control*[tiab] OR PCV[tiab] OR volume control*[tiab] OR VCV[tiab] |
| #4 | airway pressure release ventilat*[tiab] OR APRV[tiab] |
| #5 | synchronized intermittent mandatory ventilation[tiab] OR intermittent mandatory ventilation[tiab] OR SIMV[tiab] OR IMV[tiab] |
| #6 | PSV[tiab] OR pressure support[tiab] |
| #7 | #2 OR #3 OR #4 OR #5 OR #6 |
| #8 | (randomized controlled trial [pt] OR controlled clinical trial [pt] OR randomized [tiab] OR placebo [tiab] OR clinical trials as topic [mesh: noexp] OR randomly [tiab] OR trial [ti]) NOT (animals[mh] NOT humans[mh]) |
| #9 | #1 AND #7 AND #8 |

CENTRAL （Search date: 2020/7/8）

| #1 | [mh "respiratory distress syndrome, adult"] OR "acute respiratory distress syndrom":ti,ab OR [mh "respiratory insufficiency"] OR [mh "acute lung injury"] OR "acute lung injury":ti,ab OR "acute respiratory failure":ti,ab OR ALI:ti,ab OR ARDS:ti,ab |
| --- | --- |
| #2 | [mh "Respiration, Artificial"] OR "artificilal respiration":ti,ab OR [mh "Pulmonary Ventilation"] OR "pulmonary ventilation":ti,ab OR [mh "Ventilators, Mechanical"] OR "mechanical ventilation":ti,ab OR "positive-pressure respiration":ti,ab OR "positive pressure ventilation":ti,ab |
| #3 | "pressure control":ti,ab OR PCV:ti,ab OR "volume control":ti,ab OR VCV:ti,ab |
| #4 | "airway pressure release ventilation":ti,ab OR APRV:ti,ab |
| #5 | "synchronized intermittent mandatory ventilation":ti,ab OR "intermittent mandatory ventilation":ti,ab OR SIMV:ti,ab OR IMV:ti,ab |
| #6 | PSV:ti,ab OR "pressure support":ti,ab |
| #7 | {OR #2-#6} |
| #8 | #1 AND #7 |
| #9 | [mh animals] NOT [mh humans] |
| #10 | #8 NOT #9 |

Igaku-Chuo-Zasshi （Search date: 2020/6/26）

| #1 | 呼吸窮迫症候群-急性/TH or 呼吸促迫症候群/AL or ARDS/AL or "acute respiratory distress syndrome"/AL or 急性肺損傷/TH or 急性肺損傷/AL or 急性肺障害/AL or 急性肺傷害/AL or "acute lung Injury"/AL or 呼吸不全/TH or 呼吸不全/AL or 呼吸機能不全/AL |
| --- | --- |
| #2 | 人工呼吸/TH or 人工呼吸/AL or 人工換気/AL or レスピレータ/AL or ベンチレータ/AL or 機械換気/AL or 機械的換気/AL or 人工換気/AL or 調節呼吸/AL or 調節換気/AL or 陽圧呼吸/AL or 陽圧換気/AL |
| #3 | 従圧/AL or 圧規定/AL or 従量/AL or 量規定/AL or PCV/AL or VCV/AL |
| #4 | APRV/AL or 気道内圧開放式/AL or 気道内圧解放式/AL or "Airway Pressure Release Ventilation"/AL |
| #5 | 間欠性強制換気/TH or 同期式間欠的強制/AL or 間欠的強制/AL or SIMV/AL or IMV/AL or "synchronized intermittent mandatory"/AL or "intermittent mandatory"/AL |
| #6 | プレッシャーサポート/AL or PSV/AL or "pressure support"/AL or "pressure-support"/AL |
| #7 | #2 or #3 or #4 or #5 or #6 |
| #8 | (((RD=ランダム化比較試験,準ランダム化比較試験,比較研究) or (ランダム化比較試験/TH or 準ランダム化比較試験/TH or ランダム化/AL or 無作為化/AL or 比較試験/AL or 臨床試験/AL or プラセボ/AL or 対照/AL or コントロール/AL or 臨床研究/AL)) not (CK=動物 not CK=ヒト)) and (PT=会議録除く) |
| #9 | #1 and #7 and #8 |

1. Flow diagram

**Identification**

3 Studies included in qualitative synthesis

4365 records after duplicates removed

5933 records identified through database searching

5933 records identified through database searching

Medline via PubMed (n=2824)

Cochrane CENTRAL (n=2559)

Igaku-Chuo-Zasshi (n=550)

0 additional records identified through other sources

3 Studies included in quantitative synthesis (meta-analysis)

Duplicates

n=1568

4278 records excluded

**Included**

**Eligibility**

**Screening**

84 Full-text articles excluded, with reasons:

・Wrong language (n=9)

・Wrong study design (n=30)

・Wrong population (n=11)

・Wrong intervention (n=26)

・Difficult to obtain full text (n=8)

Etc.

87 Full-text articles assessed for eligibility

1. Risk of bias

Duration of mechanical ventilation Mortality

1. Forest plot

Duration of mechanical ventilation

Mortality

1. Evidence profile

| **Certainty assessment** | | | | | | | **№ of patients** | | **Effect** | | | **Certainty** | | **Importance** |  |
| --- | --- | --- | --- | --- | --- | --- | --- | --- | --- | --- | --- | --- | --- | --- | --- |
| **№ of studies** | **Study design** | **Risk of bias** | **Inconsistency** | **Indirectness** | **Imprecision** | **Other considerations** | **PCV** | **VCV** | **Relative (95% CI)** | **Absolute (95% CI)** | |  |  |  |  |
| **Duration of mechanical ventilation** | | | | | | | | | | | | | | |  |
| 2 | Randomized trials | Serious ^a^ | Not serious | Not serious | Serious ^b^ | None | 22 | 19 | - | **MD** **4.1days shorter** (6.84 shorter to 1.37 shorter) | | ⨁⨁◯◯ Low | | Critical |  |
| **Mortality** | | | | | | | | | | | | | | |  |
| 3 | Randomized trials | Not serious | Not serious | Not serious | Serious ^b^ | None | 28/68 (41.2%) | 39/68 (57.4%) | **RR 0.74** (0.53 to 1.02) | **149 fewer per 1000**  (270 fewer to 11 more) | | ⨁⨁⨁◯ Moderate | | Critical |  |
| **Ventilator-induced lung injury** | | | | | | | | | | | | | | | |
| 0 |  |  |  |  |  |  |  |  | - | Impossible to estimate | - | | Critical | | |

**CI:** confidence interval; **RR:** risk ratio; **MD**: mean difference * There were no studies dealing with ventilator-free days (VFDs), and we used ventilator days

Ventilator-induced lung injury was not included in the evidence profile because there were no reported outcomes.

#### Explanation

a. Both the included studies were assessed as having a High Risk of Bias.

b. The included studies did not meet the optimal information size (OIS).

1. Evidence-to-Decision table

| question | |
| --- | --- |
| **CQ22: Which, between pressure-control ventilation (PCV) and volume-control ventilation (VCV), is desirable for mechanical ventilation in adult patients with ARDS?** | |
| **GROUP:** | Adult patients with respiratory failure requiring mechanical ventilation, including patients with ARDS |
| **INTERVENTIONS:** | PCV |
| **Comparison and CONTRAST:** | VCV |
| **Main Outcomes:** | Mortality, Ventilator days, Ventilator-free days (VFD), Ventilator-induced lung injury (VILI) |
| **SETTING:** | Emergency room or Intensive care unit |
| **PERSPECTIVES:** | Personal |
| **BACKGROUND:** | Ventilatory management in patients with ARDS is as important as treating the primary disease. It is still unclear what the optimal mode of ventilation should be. In particular, it is unclear which of the commonly used ventilation methods, VCV or PCV, is more effective. |
| **Conflict of Interest:** | None |

# assessment

| Problem Is the problem a priority? | | |
| --- | --- | --- |
| judgment | research evidence | note |
| ○ No  ○ Probably no  ● Probably yes  ○ Yes  ○ Varies  ○ Do not know | Ventilatory management in patients with ARDS is as important as treating the primary disease. It is still unclear what the optimal mode of ventilation should be. In particular, it is unclear which of the commonly used ventilation methods, VCV or PCV, is more effective. Therefore, the priority of this issue is judged to be “probably high.” |  |
| Desirable effects How substantial are the desirable anticipated effects? | | |
| judgment | research evidence | note |
| ○Trivial  ○Small  ● Moderate  ○Large  ○Varies  ○Do not know | As a result of a systematic review, three randomized controlled trials (RCTs) consistent with the patient, intervention, comparison, and outcome (PICO) process were identified, and a meta-analysis was performed using these studies. Since we could not find any study that addressed VFD as an outcome, we changed the outcome of VFD to “ventilator days.” The effect estimate for the ventilatior days (2 RCTs: N=41) had a mean difference of 4.1 days (95% CI: 6.84 days to 1.37 days) for PCV compared with VCV. The effect estimate for mortality (three RCTs: N=136) had a risk difference of 149 fewer deaths/1000 patients (95% CI: 270 fewer deaths to 11 more deaths). No studies were found that reported VILI. The desired effect of the intervention was judged to be “moderate.” |  |
| Undesirable effects How substantial are the undesirable anticipated effects? | | |
| judgment | research evidence | note |
| ○Large  ○ Moderate  ○Small  ●Trivial  ○Varies.  ○ Do not know | We could not find any studies that addressed outcomes such as reintubation or tracheostomy. Therefore, the undesirable effect was judged to be “only a little” when considered together with the description in the remarks section. | Chacko et al.’s meta-analysis of two RCTs found that the effect estimate for VILI (2 RCTs: N=1062) had a risk difference of 23 per 1000 (95% CI: 12 fewer to 72 more) for PCV compared with VCV. |
| Certainty of evidence What is the overall certainty of the evidence of effects? | | |
| judgment | research evidence | note |
| ●Very low  ○Low  ○Moderate  ○High  ○No included studies | **Relative importance or value of the key outcomes of interest**   \| Outcome \| \| \| Relative importance \| \| \| Certainty of evidence  (GRADE) \| \| \| \| --- \| --- \| --- \| --- \| --- \| --- \| --- \| --- \| --- \| \| Ventilator days \| Critical \| \| \| ⨁⨁◯◯  Low \| \| \| \| Mortality \| \| Critical \| \| \| ⨁⨁⨁◯  Moderate \| \| \| \| VILI \| \| Critical \| \| \| - \| \| \|   VILI was not reported in the studies included.  **Overall certainty of evidence:**  The direction of desirable and undesirable effects is not consistent.  The overall certainty of the evidence was judged to be “low” by adopting the lowest certainty of evidence. |  |
| Values Is there important uncertainty about or variability in how much people value the main outcomes? | | |
| judgment | research evidence | note |
| ○Important uncertainty or variability  ○Possibly important uncertainty or variability  ●Probably no important uncertainty or variability  ○No important uncertainty or variability | There are no data on the values for outcomes under optimal ventilation conditions.  In general, the value for death is high, and the variability is low. |  |
| Balance of effects Does the balance between desirable and undesirable effects favor the intervention or the comparison? | | |
| judgment | research evidence | note |
| ○Favors the comparison  ○Probably favors the comparison  ○Does not favor either the intervention or the comparison  ●Probably favors the intervention  ○Favors the intervention  ○Varies  ○Do not know | \| Outcome \| VCV  （Control） \| PCV  （Intervention） \| Absolute difference  (95% CI) \| Relative risk  RR (95% CI) \| \| --- \| --- \| --- \| --- \| --- \| \| Ventilator days \| - \| - \| MD 4.1days shorter(6.84days-1.37days) \| - \| \| Mortality \| 39/68 \| 28/68 \| 149 per 1000 fewer (270 fewer- 11 more) \| 0.74 (0.53 ～1.02) \| \| VILI \|  \|  \| not estimate \| - \|   VILI was not reported in the included studies.  Based on the above information, the balance between the desirable and undesirable effects was judged to “Probably favor intervention.” |  |
| Acceptability Is the intervention acceptable to key stakeholders? | | |
| judgment | research evidence | note |
| ○ No  ○ Probably no  ○ Probably yes  ● Yes  ○ Varies  ○ Do not know | PCV is already a widely used ventilation condition and is considered acceptable. |  |
| Feasibility Is the intervention feasible to implement? | | |
| judgment | research evidence | note |
| ○ No  ○ Probably no  ● Probably yes  ○ Yes  ○ Varies  ○ Do not know | PCV is already a widely used ventilation mode and is considered acceptable. However, there may be differences between facilities. |  |

**Summary of Judgment**

|  | JUDGMENT | | | | | | |
| --- | --- | --- | --- | --- | --- | --- | --- |
| **PROBLEM** | No | Probably no | Probably yes | Yes |  | Varies | Do not know |
| **DESIRABLE EFFECTS** | Trivial | Small | Moderate | Large |  | Varies | Do not know |
| **UNDESIRABLE EFFECTS** | Large | Moderate | Small | Trivial |  | Varies | Do not know |
| **CERTAINTY OF EVIDENCE** | Very low | Low | Moderate | High |  |  | No included studies |
| **VALUES** | Important uncertainty or variability | Possibly important uncertainty or variability | Probably no important uncertainty or variability | No important uncertainty or variability |  |  |  |
| **BALANCE OF EFFECTS** | Favors the comparison | Probably favors the comparison | Does not favor either the intervention or the comparison | Probably favors the intervention | Favors the intervention | Varies | Do not know |
| **ACCEPTABILITY** | No | Probably no | Probably yes | Yes |  | Varies | Do not know |
| **FEASIBILITY** | No | Probably no | Probably yes | Yes |  | Varies | Do not know |

# Type of recommendation

| Strong recommendation against the intervention | Conditional recommendation against the intervention | Conditional recommendation for either the intervention or the comparison | Conditional recommendation for the intervention | Strong recommendation for the intervention |
| --- | --- | --- | --- | --- |
| ○ | ○ | ○ | ● | ○ |

# Conclusion

| Recommendation |
| --- |
| **No recommendation can be made from the results of this systematic review as to whether PCV or VCV ventilation conditions should be used in adults with ARDS (in our practice statement).**  **Supplementary item**  **VCV has the advantage that it is easy to notice changes in compliance and airway resistance. In facilities that are proficient in the use of VCV, its use may be considered.** **Based on this systematic review alone, it cannot be judged that PCV is superior to VCV. The panel voted to leave it as “in our practice statement” without a recommendation.** |
|  |
| Justification |
| **Question**: Which, between pressure-control ventilation (PCV) and volume-control ventilation (VCV), is desirable for mechanical ventilation in adult patients with ARDS?  **Patient**: Adult patients with ARDS or critically ill ventilated patients  **Intervention**: PCV  **Comparison control**: VCV  **Outcomes**: Ventilator days, mortality  **Summary of Evidence:**  The outcome of VFD was changed to “ventilator days” because no studies were found that included VFD.  The use of PCVs reduced the ventilator day (2 RCTs: N=41) by an mean of 4.1 days (95% CI: 6.84 to 1.37 days) and mortality (3 RCTs: N=136) by 149 per 1000 (95% CI: 270 fewer to 11 more). Thus, the desired effect of the intervention was judged to be “moderate.”  The VILI was not reported in the articles included in this study. Therefore, we cited the results of Chacko et al.’s meta-analysis 1) of two RCTs, which found that VILI (2 RCTs: N=1062) increased by 23 per 1000 (95% CI: 12 fewer to 72 more) with PCV.  The undesirable effect was judged to be “slight.”  With respect to whether the effect of the intervention was greater than the harm, we judged the effect to be “probably greater.”  **Certainty of Evidence**:  There was no concordance on the direction of the desirable and undesirable effects. The certainty of the evidence was judged to be “very low,” adopting the lowest certainty of evidence.  **Determining values, balance of effects, acceptance, and viability:**  The possibility of PCV increasing the frequency of VILI is considered to be small, and the effect of PCV is considered to outweigh the harm.  In addition, since PCV, like VCV, is a ventilation condition that can be set with a regular ventilator, it does not increase the cost or burden and is judged to be feasible.  **Panel discussion:**  In the pre-vote, the panel agreed with the modified Delphi method that “PCV should be used in the ventilator management of adult patients with ARDS (very weak recommendation GRADE 2D)” with a median score of 8.0 and a disagreement index of 0.2920.  However, the panel considered that the results of this systematic review do not provide a high enough level of certainty of recommendation and evidence to recommend a change to PCV in facilities currently using VCV as the standard of care.  As a result, it was decided to re-vote. The modified Delphi method showed that “the results of this systematic review do not allow us to make a recommendation on whether PCV or VCV should be used as the ventilation condition in adult patients with ARDS (in our practice statement).” A median score of 8.0 and a disagreement index of 0.1316 were agreed upon, and the above recommendation was made.  **Additional Considerations:**  One of the three studies used in this meta-analysis (Ahmed 2016) was on patients with acute exacerbation of chronic obstructive pulmonary disease (COPD). Although the other two studies dealt with patients with ARDS, the following sensitivity analysis showed no significant difference in the duration of ventilation. It is difficult to argue the usefulness of PCV for patients with ARDS based on the results of this meta-analysis alone. |

| Subgroup considerations |
| --- |
| A meta-analysis excluding studies dealing with patients with acute exacerbation of COPD (Ahmed 2016) was performed as a sensitivity analysis (see additional considerations).  PCV resulted in a reduction of 1 day in ventilation duration (1 RCT; 7.08 days shorter to 5.08 days longer) and a 170/1000 reduction in death (2 RCTs; 312 lower patients to 27 higher).  Perhaps due to the small number of studies included, the significant difference in the reduction of ventilation duration observed in this analysis disappeared.    In addition, the use of VCV in patients with spontaneous breathing may increase lung injury due to patient-ventilator dyssynchrony and changes in airway pressure toward negative pressure. On the contrary, for patients who do not have spontaneous breathing, the difference in pulmonary effects between VCV and PCV may not be significant. Therefore, a comparison between the subgroups of patients with and without spontaneous breathing should be considered. |
| Implementation considerations |
| No statement was found in the Japanese ARDS Clinical Practice Guidelines 2016 or ATS/ESICM/SCCM 2017 regarding whether PCV or VCV is preferable. It is necessary to select a safe ventilation mode depending on the facility and healthcare providers’ familiarity and proficiency with each ventilation mode and the pathophysiology of the patient’s airway resistance. Tidal volume should be monitored when PCV is used and plateau pressure when VCV is used. |

| Monitoring and evaluation |
| --- |
| In addition to PCV and VCV, further information needs to be collected on the use of pressure-regulated volume control ventilation. After the publication of the guideline, it is necessary to survey t if there are any problems in the clinical setting. |
| Research priorities |
| Because of the dissociation between the results of the meta-analysis and the panel’s vote, we were unable to make a recommendation. The question as to whether PCV or VCV should be chosen remains important in daily practice. A large RCT comparing VCV and PCV is warranted. |

References

1. Chacko B, Peter JV, Tharyan P, et al. Pressure-controlled versus volume-controlled ventilation for acute respiratory failure due to acute lung injury (ALI) or acute respiratory distress syndrome (ARDS). Cochrane Database Syst Rev. 2015;1:CD008807. PMID: 25586462

**CQ23 Should airway pressure release ventilation (APRV) be used in the mechanical ventilation of adult patients with ARDS?**

1. Search strategy

MEDLINE via PubMed （Search date: 2020/7/8）

| #1 | respiratory distress syndrome, adult[mh] OR acute respiratory distress syndrom*[tiab] OR respiratory insufficiency[mh] OR acute lung injury[mh] OR acute lung injur*[tiab] OR acute respiratory failure*[tiab] OR ALI[tiab] OR ARDS[tiab] |
| --- | --- |
| #2 | Respiration, Artificial[mh] OR artificilal respiration*[tiab] OR Pulmonary Ventilation[mh] OR pulmonary ventilat*[tiab] OR Ventilators, Mechanical[mh] OR mechanical ventilat*[tiab] OR positive-pressure respiration*[tiab] OR positive pressure ventilat*[tiab] |
| #3 | pressure control*[tiab] OR PCV[tiab] OR volume control*[tiab] OR VCV[tiab] |
| #4 | airway pressure release ventilat*[tiab] OR APRV[tiab] |
| #5 | synchronized intermittent mandatory ventilation[tiab] OR intermittent mandatory ventilation[tiab] OR SIMV[tiab] OR IMV[tiab] |
| #6 | PSV[tiab] OR pressure support[tiab] |
| #7 | #2 OR #3 OR #4 OR #5 OR #6 |
| #8 | (randomized controlled trial [pt] OR controlled clinical trial [pt] OR randomized [tiab] OR placebo [tiab] OR clinical trials as topic [mesh: noexp] OR randomly [tiab] OR trial [ti]) NOT (animals[mh] NOT humans[mh]) |
| #9 | #1 AND #7 AND #8 |

CENTRAL （Search date: 2020/7/8）

| #1 | [mh "respiratory distress syndrome, adult"] OR "acute respiratory distress syndrom":ti,ab OR [mh "respiratory insufficiency"] OR [mh "acute lung injury"] OR "acute lung injury":ti,ab OR "acute respiratory failure":ti,ab OR ALI:ti,ab OR ARDS:ti,ab |
| --- | --- |
| #2 | [mh "Respiration, Artificial"] OR "artificilal respiration":ti,ab OR [mh "Pulmonary Ventilation"] OR "pulmonary ventilation":ti,ab OR [mh "Ventilators, Mechanical"] OR "mechanical ventilation":ti,ab OR "positive-pressure respiration":ti,ab OR "positive pressure ventilation":ti,ab |
| #3 | "pressure control":ti,ab OR PCV:ti,ab OR "volume control":ti,ab OR VCV:ti,ab |
| #4 | "airway pressure release ventilation":ti,ab OR APRV:ti,ab |
| #5 | "synchronized intermittent mandatory ventilation":ti,ab OR "intermittent mandatory ventilation":ti,ab OR SIMV:ti,ab OR IMV:ti,ab |
| #6 | PSV:ti,ab OR "pressure support":ti,ab |
| #7 | {OR #2-#6} |
| #8 | #1 AND #7 |
| #9 | [mh animals] NOT [mh humans] |
| #10 | #8 NOT #9 |

Igaku-Chuo-Zasshi （Search date: 2020/6/26）

| #1 | 呼吸窮迫症候群-急性/TH or 呼吸促迫症候群/AL or ARDS/AL or "acute respiratory distress syndrome"/AL or 急性肺損傷/TH or 急性肺損傷/AL or 急性肺障害/AL or 急性肺傷害/AL or "acute lung Injury"/AL or 呼吸不全/TH or 呼吸不全/AL or 呼吸機能不全/AL |
| --- | --- |
| #2 | 人工呼吸/TH or 人工呼吸/AL or 人工換気/AL or レスピレータ/AL or ベンチレータ/AL or 機械換気/AL or 機械的換気/AL or 人工換気/AL or 調節呼吸/AL or 調節換気/AL or 陽圧呼吸/AL or 陽圧換気/AL |
| #3 | 従圧/AL or 圧規定/AL or 従量/AL or 量規定/AL or PCV/AL or VCV/AL |
| #4 | APRV/AL or 気道内圧開放式/AL or 気道内圧解放式/AL or "Airway Pressure Release Ventilation"/AL |
| #5 | 間欠性強制換気/TH or 同期式間欠的強制/AL or 間欠的強制/AL or SIMV/AL or IMV/AL or "synchronized intermittent mandatory"/AL or "intermittent mandatory"/AL |
| #6 | プレッシャーサポート/AL or PSV/AL or "pressure support"/AL or "pressure-support"/AL |
| #7 | #2 or #3 or #4 or #5 or #6 |
| #8 | (((RD=ランダム化比較試験,準ランダム化比較試験,比較研究) or (ランダム化比較試験/TH or 準ランダム化比較試験/TH or ランダム化/AL or 無作為化/AL or 比較試験/AL or 臨床試験/AL or プラセボ/AL or 対照/AL or コントロール/AL or 臨床研究/AL)) not (CK=動物 not CK=ヒト)) and (PT=会議録除く) |
| #9 | #1 and #7 and #8 |

1. Flow diagram

**Identification**

7 Studies included in qualitative synthesis

4365 records after duplicates removed

5933 records identified through database searching

5933 records identified through database searching

Medline via PubMed (n=2824)

Cochrane CENTRAL (n=2559)

Igaku-Chuo-Zasshi (n=550)

0 additional records identified through other sources

7 Studies included in quantitative synthesis (meta-analysis)

Duplicates

n=1568

4278 records excluded

**Included**

**Eligibility**

**Screening**

80 Full-text articles excluded, with reasons:

・Wrong language (n=9)

・Wrong study design (n=30)

・Wrong population (n=11)

・Wrong intervention (n=22)

・Difficult to obtain full text (n=8)

Etc.

87 Full-text articles assessed for eligibility

1. Risk of bias

Ventilator-free days Mortality

Barotrauma

1. Forest plot

Ventilator-free days

Mortality

Barotrauma

1. Evidence profile

| **Certainty assessment** | | | | | | | | | | | | | | | **№ of patients** | | | **Effect** | | | **Certainty** | | | **Importance** | |
| --- | --- | --- | --- | --- | --- | --- | --- | --- | --- | --- | --- | --- | --- | --- | --- | --- | --- | --- | --- | --- | --- | --- | --- | --- | --- |
| **№ of studies** | **Study design** | | **Risk of bias** | | **Inconsistency** | | **Indirectness** | | **Imprecision** | | **Other considerations** | | | | **APRV** | **VCV or PCV** | | **Relative (95% CI)** | **Absolute (95% CI)** | |  |  |  |  |  |
| **Ventilator Free Days** | | | | | | | | | | | | | | | | | | | | | | | | | |
| 3 | Randomized trials | | Serious ^a^ | | Serious ^b^ | | Not serious | | Serious ^c^ | | None | | 127 | | | 121 | | - | **MD 3.64 days longer** (0.02 days shorter to 7.3 days longer) | | ⨁◯◯◯ Very low | | | Critical | |
| **Mortality** | | | | | | | | | | | | | | | | | | | | | | | | | |
| 7 | Randomized trials | | Not serious | | Not serious | | Not serious | | Serious ^d^ | | None | | 35/210 (16.7%) | | | 48/199 (24.1%) | | **RR 0.70** (0.48 to 1.02) | **72 fewer per 1000** (125 fewer to  5 more) | | ⨁⨁⨁◯ Moderate | | | Critical | |
| **Varotorauma** | | | | | | | | | | | | | | | | | | | | | | | | |  |
| 3 | | Randomized trials | | Serious ^a^ | | Not serious | | Not serious | | Very serious ^c^ | | None | | 3/128 (2.3%) | | | 6/125 (4.8%) | **RR 0.52** (0.15 to 1.87) | | **23 fewer per 1000**  (41 fewer to  42 more) | | ⨁〇〇◯ Very low | Critical | |  |

**CI:** confidence interval; **RR:** risk ratio; **MD**; mean difference

#### Explanation

a. The risk of bias was high because treatment providers and participants could not be blinded, and there were many items for which bias could not be assessed.

b. Owing to the high degree of statistical heterogeneity (I2=78%) and also clinical heterogeneity as a result of the visual judgment of the forest plot, we judged it as "serious" and downgraded it by one level

c. The total sample size of 248 does not meet the optimal information size (OIS), and the 95% confidence interval is too wide to be considered "no effect" or crossing the minimum important difference (MID), so it was judged "serious" and downgraded by one level.

d. The total sample size was 409, and the number of events was 83, which did not meet the optimal information size (OIS), and the 95% confidence interval was wide, and the confidence interval included "no effect" and "substantial benefit.”

e.The total sample size was 253, and the number of events was 9, which did not meet the OIS, and the 95% confidence interval was wide and included "considerable harm" and "considerable benefit. It was judged to be "very serious" and downgraded by two levels.

1. Evidence-to-Decision table

| question | |
| --- | --- |
| **CQ23：Should airway pressure release ventilation (APRV) be used in the mechanical ventilation of adult patients with ARDS?** | |
| **GROUP:** | Adult patients with respiratory failure requiring mechanical ventilation, including patients with ARDS |
| **INTERVENTIONS:** | APRV |
| **Comparison and CONTRAST:** | Conventional ventilator management (assist/control) |
| **Main Outcomes:** | Ventilator free days (VFD, mortality, barotrauma) |
| **SETTING:** | Emergency room or intensive care unit |
| **PERSPECTIVES:** | Personal |
| **BACKGROUND:** | Ventilatory management in patients with ARDS is as important as treating the primary disease. It is still unclear what the optimal mode of ventilation should be. APRV is often used in ARDS because of its ability to maintain high airway pressures. It is unclear whether APRV is more effective than volume-controlled ventilation (VCV) or pressure-controlled ventilation (PCV) as a commonly used ventilation mode. |
| **Conflict of Interest:** | None |

# assessment

| Problem Is the problem a priority? | | |
| --- | --- | --- |
| judgment | research evidence | note |
| ○ No  ○ Probably no  ● Probably yes  ○ Yes  ○ Varies  ○ Do not know | Ventilatory management in patients with ARDS is as important as treating the primary disease. It is still unclear what the optimal mode of ventilation should be. APRV is often used in ARDS because of its ability to maintain high airway pressures. It is unclear whether APRV is more effective than VCV or PCV as a commonly used ventilation mode. The priority of this issue is probably high. |  |
| Desirable effects How substantial are the desirable anticipated effects? | | |
| judgment | research evidence | note |
| ○Trivial  ○Small  ● Moderate  ○Large  ○Varies  ○Do not know | As a result of the systematic review, seven randomized controlled trials (RCTs) were identified that followed the patient, intervention, comparison, and outcome process, and a meta-analysis was performed using these studies. As a beneficial outcome, the effect estimate for VFD (3 RCTs: N=248) had a mean difference of 3.64 longer days (95% CI: 0.02 days decrease to 7.3 days increase) for APRV compared to VCV or PCV. The effect estimate for death (7 RCTs: N=409) had a risk difference of 72 lower deaths/1000 patients (95% CI: 125 lower deaths to five higher). Barotrauma, which was initially considered an outcome of harm, was also set as an outcome of benefit because it was in the direction of benefit. The effect estimate for barotrauma (3 RCTs: N=253) was a 23 person reduction in risk per 1000 people (95% CI: 41 person reduction to 42 person increase). Thus, the desired effect of the intervention was judged to be “moderate.” |  |
| Undesirable effects How substantial are the undesirable anticipated effects? | | |
| judgment | research evidence | note |
| ○Large  ○ Moderate  ○Small  ○Trivial  ○Varies.  ● Do not know | Barotrauma, which was initially considered an outcome of harm, was also set as an outcome of benefit because it was in the direction of benefit. We also examined other important outcomes but found no obvious harmful outcomes. Therefore, the desired effect was judged to be “unknown.” |  |
| Certainty of evidence What is the overall certainty of the evidence of effects? | | |
| judgment | research evidence | note |
| ○Very low  ○Low  ●Moderate  ○High  ○No included studies | **Relative importance or value of the key outcomes of interest**   \| Outcome \| Relative importance \| Certainty of evidence \| \| --- \| --- \| --- \| \| VFD \| Critical \| ⨁◯◯◯  Very low \| \| Mortality \| Critical \| ⨁⨁⨁◯  Moderate \| \| Barotrauma \| Critical \| ⨁◯◯◯  Very low \|   Ventilator-induced lung injury (VILI) was not reported within the included studies.  **Overall certainty of evidence：**  The direction of desirable and undesirable effects is consistent.  The certainty of the evidence was judged to be “Moderate” by adopting the highest certainty of evidence. |  |
| Values Is there important uncertainty about or variability in how much people value the main outcomes? | | |
| judgment | research evidence | note |
| ○Important uncertainty or variability  ○Possibly important uncertainty or variability  ●Probably no important uncertainty or variability  ○No important uncertainty or variability | There are no data on the values for outcomes about ventilatory management with APRV. In general, the value for death is high, and the variability is low. |  |
| Balance of effects Does the balance between desirable and undesirable effects favor the intervention or the comparison? | | |
| judgment | research evidence | note |
| ○Favors the comparison  ○Probably favors the comparison  ○Does not favor either the intervention or the comparison  ●Probably favors the intervention  ○Favors the intervention  ○Varies  ○Do not know | **結果のまとめ**   \| Outcome \| A/C(Control) \| APRV  (intervention) \| Absolute difference (95% CI) \| Relative Risk RR (95% CI) \| \| --- \| --- \| --- \| --- \| --- \| \| VFD \|  \| - \| MD 3.64 days longer (0.02 days shorter〜7.3 days longer) \| - \| \| Mortality \| 48/199 \| 35/210 \| 72 per /1000 lower  (125 fewer〜5 more) \| 0.70 (0.48-1.02) \| \| Barotrauma \| 6/125 \| 3/128 \| 23 per /1000 lower  (41 fewer〜42 more) \| 0.52 (0.15-1.87) \|   Based on the above information, the balance between desirable and undesirable effects was judged to “Probably favor intervention.” |  |
| Acceptability Is the intervention acceptable to key stakeholders? | | |
| judgment | research evidence | note |
| ○ No  ○ Probably no  ○ Probably yes  ○ Yes  ● Varies  ○ Do not know | Although there was no evidence on the acceptance of APRV reviewed, facilities with ventilators that have a dedicated mode for APRV may be able to accept it if they have staff who are skilled in its use. On the contrary, in facilities that do not have a ventilator with a dedicated APRV mode, a mode such as bi-level must be used to perform operations similar to APRV. As a result, the exhalation time may be prolonged by synchronizing with patients’ exhalation, or may not maintain sufficient auto PEEP because a short low-pressure phase time cannot be set. In this case, the use of a special ventilator would be desirable and costly. Therefore, acceptability was judged to be “ It is hard to say.” |  |
| Feasibility Is the intervention feasible to implement? | | |
| judgment | research evidence | note |
| ○ No  ○ Probably no  ○ Probably yes  ○ Yes  ● Varies  ○ Do not know | If the facility has a ventilator with an APRV mode, it should be possible to perform the procedure. However, when setting up the ventilator, the duration of the low-pressure phase should be set to a target of 50-75% of the peak expiratory flow rate (PEFR), and the medical staff should be proficient in the use of APRV. We judged the feasibility to be “ It is hard to say.” |  |

**Summary of Judgment**

|  | | **JUDGMENT** | | | | | | | |
| --- | --- | --- | --- | --- | --- | --- | --- | --- | --- |
| **PROBLEM** | | No | Probably no | Probably yes | Yes |  | Varies | Do not know | |
| **DESIRABLE EFFECTS** | | Trivial | Small | Moderate | Large |  | Varies | Do not know | |
| **UNDESIRABLE EFFECTS** | | Large | Moderate | Small | Trivial |  | Varies | Do not know | |
| **CERTAINTY OF EVIDENCE** | | Very low | Low | Moderate | High |  |  | No included studies | |
| **VALUES** | | Important uncertainty or variability | Possibly important uncertainty or variability | Probably no important uncertainty or variability | No important uncertainty or variability |  |  |  | |
| **BALANCE OF EFFECTS** | | Favors the comparison | Probably favors the comparison | Does not favor either the intervention or the comparison | Probably favors the intervention | Favors the intervention | Varies | Do not know | |
| **ACCEPTABILITY** | | No | Probably no | Probably yes | Yes |  | Varies | Do not know | |
| **FEASIBILITY** | | No | Probably no | Probably yes | Yes |  | Varies | Do not know | |

# Type of recommendation

| Strong recommendation against the intervention | Conditional recommendation against the intervention | Conditional recommendation for either the intervention or the comparison | Conditional recommendation for the intervention | Strong recommendation for the intervention |
| --- | --- | --- | --- | --- |
| ○ | ○ | ● | 〇 | ○ |

# Conclusion

| Recommendation |
| --- |
| **In adult patients with ARDS, no recommendation can be made as to which ventilatory condition should be used when spontaneous breathing remains. APRV may be considered when ventilatory management with spontaneous breathing is used (in our practice statement).**  **Supplementary item**  **This systematic review also included studies that used an inverse ratio ventilation (IRV). A meta-analysis was also performed excluding the IRV, which showed a favorable direction for VFD, mortality, and barotrauma in the APRV group. Since there was no support for presenting a recommendation at the panel meeting, it was decided to make it an “in our practice statement.”** |
|  |
| Justification |
| **Question**: Should airway pressure release ventilation (APRV) be used in the mechanical ventilation of adult patients with ARDS?  **Patient**: Adult patients with respiratory failure requiring mechanical ventilation, including patients with ARDS  **Intervention**: APRV  **Comparison control**: Conventional ventilator management (assist/control)  **Outcomes**: VFD, mortality, barotrauma  **Summary of Evidence:**  １）Overall  A systematic review was conducted, and a meta-analysis was performed for seven RCTs. The use of APRV prolonged VFD (3 RCTs: N=248) by a mean of 3.64 days (95% CI: 0.02 days shorter to 7.3 days longer), reduced mortality (7 RCTs: N=409) by 72 per 1000 (95% CI: 125 fewer to 5 more), and reduced barotrauma (3 RCTs: N=253) by (3 RCTs: N=253) by 23 per 1000 (95% CI: 41 fewer to 42 more).  Based on the above, we judged the desired effect of the intervention to be “moderate.” On the contrary, there were no obvious harmful outcomes, and thus the undesirable effect of the intervention was judged to be “unknown.” Regarding whether the effect of the intervention was greater than the harm, we judged the effect to be “probably greater.”  ２）”Typical APRV”  Four of the seven RCTs included in this study used the bi-level mode of inverse ratio ventilation, in which expiratory flow is zero by the end of the low pressure phase (Putensen 2002, Varpula 2003, Varpula 2004, Varpula 2009). It was not a commonly recognized APRV. APRV is considered to be a short-term (50-75% PEFR) low pressure phase 2). We also performed a meta-analysis of only those APRVs with non-zero expiratory flow during the low pressure phase (>0% PEFR, here referred to as “typical APRV”) (only Zhou 2017 met the 50-75% PEFR).  “Typical APRV” prolonged VFD (2 RCTs: N=190) by a mean of 5.65 days (95% CI: 2.8 days longer to 8.49 days longer), reduced mortality (3 RCTs: N=253) by 84 per 1000 (95% CI: 152 fewer to 22 more), and reduced barotrauma (3 RCTs: N=253) by 23 per 1000 (95% CI: 41 fewer to 42 more).  Thus, the desired effect of the intervention was judged to be “moderate.” On the contrary, there were no clear harmful outcomes, and thus the undesirable effects of the intervention were judged to be “unknown.” As to whether the effect of the intervention was greater than the harm, we judged the effect to be “probably greater”.  The “typical APRV” meta-analysis did not show any change in the point estimates or overall favorability direction compared to the overall analysis.  **Certainty of Evidence**:  １) Overall  The direction of the desirable and undesirable effects was consistent, and the certainty of evidence across outcomes was judged to be “moderate” by adopting the highest certainty of evidence.  2) “Typical APRV”  The direction of the desirable and undesirable effects was consistent, and the certainty of evidence across outcomes was judged to be “moderate” by adopting the highest certainty of evidence. There was no change in the certainty of the evidence.  **Determining values, balance of effects, acceptance, and viability:**  APRV has the potential to improve mortality, VFD, and barotrauma outcomes compared to conventional ventilatory management. However, it is difficult and costly to perform typical APRV with non-specific ventilators, and it requires skilled staff. Therefore, the use of APRV is considered to require a facility-specific solution.  **Panel discussion**  In the pre-vote, the modified Delphi method resulted in a median score of 7 and a disagreement index of 0.2414 for “Propose the use of APRV as a ventilatory mode in adult patients with ARDS” (weak recommendation/evidence of very low certainty: GRADE 2D). At the panel meeting, there was a discussion that it was difficult to recommend this mode of treatment with muscle relaxants. As a result, a re-vote was held, and a modified Delphi method was agreed upon with a median score of 7 and a disagreement index of 0.2188, “In our practice statement as a recommendation.”  **Additional Considerations:**  The RCTs in this "typical APRV" group included a study that defined the low-pressure phase time as 25-75% of PEFR (Maxewell2010), and a study with a short expiratory time of 0.4-0.8 seconds but no mention of PEFR (Li2016).  Habashi's review^2)^ recommended that the low-pressure phase time should meet 50-75% of PEFR, but Zhou 2017 was the only study that met this recommendation, so this may have influenced the present results.  In addition, most of the studies we included used moderate-to-severe ARDS as the inclusion criteria, and the mean P/F ratio was around 100-150 mmHg. Therefore, if APRV is to be used, it should be used in patients with moderate to severe ARDS. |

| Subgroup considerations |
| --- |
| None |
| Implementation considerations |
| No recommendations were made in the Japanese ARDS Clinical Practice Guidelines 2016 and ATS/ESICM/SCCM 2017 for the use of APRV for ARDS.  If APRV mode is set without attention to PEFR, it may not be sufficiently effective. Therefore, caution should be taken when using APRV in facilities that are not proficient in its use or do not have ventilators dedicated to APRV.  In addition, when using APRV, the possibility of increased risk of VILI in patients with poor lung recruitability and the possibility of decreased cardiac output and blood pressure due to decreased venous perfusion caused by high airway pressures should be considered. |

| Monitoring and evaluation |
| --- |
| It is necessary to collect information on how many facilities are using APRV.  In addition, it is necessary to monitor the use of APRV by conducting questionnaires after the guidelines are published. |
| Research priorities |
| In this review, the only RCT that used the PEFR setting recommended by Habashi 2) was the study by Zhou in 2017. Another large RCT is needed to confirm the effectiveness of this setting method. In addition, the effect of APRV may differ depending on the recruitability and strength of spontaneous breathing, and further studies are warranted. |

References

1. Carsetti A, Damiani E, Domizi R, et al. Airway pressure release ventilation during acute hypoxemic respiratory failure: a systematic review and meta-analysis of randomized controlled trials. Ann Intensive Care. 2019;9(1):44. PMID: 30949778

2 [Nader M Habashi](https://pubmed.ncbi.nlm.nih.gov/?sort=date&term=Habashi+NM&cauthor_id=15753733). Other approaches to open-lung ventilation: airway pressure release ventilation. Crit Care Med. 2005;33(3 Suppl):S228-40. PMID: 15753733

**CQ24 Which is preferable for ventilatory management of adult patients with ARDS: synchronized intermittent mandatory ventilation (SIMV) or assisted controlled ventilation (A/C)?**

1. Search strategy

MEDLINE via PubMed （Search date: 2020/7/8）

| #1 | respiratory distress syndrome, adult[mh] OR acute respiratory distress syndrom*[tiab] OR respiratory insufficiency[mh] OR acute lung injury[mh] OR acute lung injur*[tiab] OR acute respiratory failure*[tiab] OR ALI[tiab] OR ARDS[tiab] |
| --- | --- |
| #2 | Respiration, Artificial[mh] OR artificilal respiration*[tiab] OR Pulmonary Ventilation[mh] OR pulmonary ventilat*[tiab] OR Ventilators, Mechanical[mh] OR mechanical ventilat*[tiab] OR positive-pressure respiration*[tiab] OR positive pressure ventilat*[tiab] |
| #3 | pressure control*[tiab] OR PCV[tiab] OR volume control*[tiab] OR VCV[tiab] |
| #4 | airway pressure release ventilat*[tiab] OR APRV[tiab] |
| #5 | synchronized intermittent mandatory ventilation[tiab] OR intermittent mandatory ventilation[tiab] OR SIMV[tiab] OR IMV[tiab] |
| #6 | PSV[tiab] OR pressure support[tiab] |
| #7 | #2 OR #3 OR #4 OR #5 OR #6 |
| #8 | (randomized controlled trial [pt] OR controlled clinical trial [pt] OR randomized [tiab] OR placebo [tiab] OR clinical trials as topic [mesh: noexp] OR randomly [tiab] OR trial [ti]) NOT (animals[mh] NOT humans[mh]) |
| #9 | #1 AND #7 AND #8 |

CENTRAL （Search date: 2020/7/8）

| #1 | [mh "respiratory distress syndrome, adult"] OR "acute respiratory distress syndrom":ti,ab OR [mh "respiratory insufficiency"] OR [mh "acute lung injury"] OR "acute lung injury":ti,ab OR "acute respiratory failure":ti,ab OR ALI:ti,ab OR ARDS:ti,ab |
| --- | --- |
| #2 | [mh "Respiration, Artificial"] OR "artificilal respiration":ti,ab OR [mh "Pulmonary Ventilation"] OR "pulmonary ventilation":ti,ab OR [mh "Ventilators, Mechanical"] OR "mechanical ventilation":ti,ab OR "positive-pressure respiration":ti,ab OR "positive pressure ventilation":ti,ab |
| #3 | "pressure control":ti,ab OR PCV:ti,ab OR "volume control":ti,ab OR VCV:ti,ab |
| #4 | "airway pressure release ventilation":ti,ab OR APRV:ti,ab |
| #5 | "synchronized intermittent mandatory ventilation":ti,ab OR "intermittent mandatory ventilation":ti,ab OR SIMV:ti,ab OR IMV:ti,ab |
| #6 | PSV:ti,ab OR "pressure support":ti,ab |
| #7 | {OR #2-#6} |
| #8 | #1 AND #7 |
| #9 | [mh animals] NOT [mh humans] |
| #10 | #8 NOT #9 |

Igaku-Chuo-Zasshi （Search date: 2020/6/26）

| #1 | 呼吸窮迫症候群-急性/TH or 呼吸促迫症候群/AL or ARDS/AL or "acute respiratory distress syndrome"/AL or 急性肺損傷/TH or 急性肺損傷/AL or 急性肺障害/AL or 急性肺傷害/AL or "acute lung Injury"/AL or 呼吸不全/TH or 呼吸不全/AL or 呼吸機能不全/AL |
| --- | --- |
| #2 | 人工呼吸/TH or 人工呼吸/AL or 人工換気/AL or レスピレータ/AL or ベンチレータ/AL or 機械換気/AL or 機械的換気/AL or 人工換気/AL or 調節呼吸/AL or 調節換気/AL or 陽圧呼吸/AL or 陽圧換気/AL |
| #3 | 従圧/AL or 圧規定/AL or 従量/AL or 量規定/AL or PCV/AL or VCV/AL |
| #4 | APRV/AL or 気道内圧開放式/AL or 気道内圧解放式/AL or "Airway Pressure Release Ventilation"/AL |
| #5 | 間欠性強制換気/TH or 同期式間欠的強制/AL or 間欠的強制/AL or SIMV/AL or IMV/AL or "synchronized intermittent mandatory"/AL or "intermittent mandatory"/AL |
| #6 | プレッシャーサポート/AL or PSV/AL or "pressure support"/AL or "pressure-support"/AL |
| #7 | #2 or #3 or #4 or #5 or #6 |
| #8 | (((RD=ランダム化比較試験,準ランダム化比較試験,比較研究) or (ランダム化比較試験/TH or 準ランダム化比較試験/TH or ランダム化/AL or 無作為化/AL or 比較試験/AL or 臨床試験/AL or プラセボ/AL or 対照/AL or コントロール/AL or 臨床研究/AL)) not (CK=動物 not CK=ヒト)) and (PT=会議録除く) |
| #9 | #1 and #7 and #8 |

1. Flow diagram

**Identification**

1 Studies included in qualitative synthesis

4365 records after duplicates removed

5933 records identified through database searching

5933 records identified through database searching

Medline via PubMed (n=2824)

Cochrane CENTRAL (n=2559)

Igaku-Chuo-Zasshi (n=550)

0 additional records identified through other sources

1 Studies included in quantitative synthesis (meta-analysis)

Duplicates

n=1568

4278 records excluded

**Included**

**Eligibility**

**Screening**

86 Full-text articles excluded, with reasons:

・Wrong language (n=9)

・Wrong study design (n=30)

・Wrong population (n=11)

・Wrong intervention (n=28)

・Difficult to obtain full text (n=8)

Etc.

87 Full-text articles assessed for eligibility

1. Risk of bias

Duration of mechanical ventilation Mortality

1. Forest plot

Duration of mechanical ventilation

Mortality

1. Evidence profile

| **Certainty assessment** | | | | | | | **№ of patients** | | **Effect** | | **Certainty** | **Importance** |
| --- | --- | --- | --- | --- | --- | --- | --- | --- | --- | --- | --- | --- |
| **№ of studies** | **Study design** | **Risk of bias** | **Inconsistency** | **Indirectness** | **Imprecision** | **Other considerations** | **Lower tidal volume (4**–**8 mL/kg)** | **higher tidal volume (>8 mL/kg)** | **Relative (95% CI)** | **Absolute (95% CI)** |  |  |
| **Duration of mechanical ventilation** | | | | | | | | | | | | |
| 1 | Randomized trials | Serious ^a^ | Not serious | Not serious | Serious ^b^ | None | 20 | 20 | - | **MD 0 days shorter** (7.41 shorter to 7.41 longer) | ⨁⨁◯◯ Low | Critical |
| **Mortality** | | | | | | | | | | | | |
| 1 | Randomized trials | Not serious | Not serious | Not serious | Very Serious ^c^ | None | 6/20 (30.0%) | 8/20 (40.0%) | **RR 0.75** (0.32 to 1.77) | **100 fewer per 1000** (272 fewer to 308 more) | ⨁⨁◯◯ Low | Critical |
| **Ventilator-associated lung injury** | | | | | | | | | | | | |
| 0 |  |  |  |  |  |  |  |  | Impossible to estimate | - | - | Critical |

**CI:** confidence interval; **MD:** mean difference; **RR:** risk ratio

#### Explanation

a. The included studies had a moderate risk of bias

b. The included studies did not meet the optimal information size (OIS)

c. The included studies did not meet the OIS, and the 95% confidence interval crossed the threshold for clinical judgment.

1. Evidence-to-Decision table

| Question | |
| --- | --- |
| **CQ24: Which is preferable for ventilatory management of adult patients with ARDS: synchronized intermittent mandatory ventilation (SIMV) or assisted controlled ventilation (A/C)?** | |
| **Group:** | Adult patients with ARDS that require ventilatory management |
| **Interventions:** | Ventilatory management with SIMV |
| **Comparison and contrast:** | Ventilatory management with A/C |
| **Main outcomes:** | Ventilator days, mortality, and ventilator-induced lung injury (VILI) |
| **Setting:** | The emergency room or intensive care unit |
| **Perspectives:** | Personal |
| **Background:** | Ventilatory management in patients with ARDS is as important as treating the primary disease. In particular, it is unclear which of the two is more effective, assist control ventilation (A/C) or SIMV. In particular, although A/C and SIMV are used in routine ventilator management, it is unclear which of the two is more effective. |
| **Conflict of Interest:** | None |

# assessment

| Problem Is the problem a priority? | | |
| --- | --- | --- |
| Judgment | RESEARCH EVIDENCE | remarks |
| ○ No  ○ Probably no  ● Probably yes  ○ Yes  ○ Varies  ○ Do not know | Ventilatory management in patients with ARDS is as important as treating the primary disease. It is unclear what the optimal ventilation conditions should be and whether the A/C mode (volume control ventilation or pressure control ventilation) or SIMV is more effective. Moreover, it is unclear which is more effective: A/C mode (volume control ventilation or pressure control ventilation) or SIMV. Therefore, this issue should probably be given high priority. |  |
| Desirable Effects How substantial are the desirable anticipated effects? | | |
| JUDGMENT | RESEARCH EVIDENCE | remarks |
| ○Trivial  ●Small  ○ Moderate  ○Large  ○Varies  ○Do not know | As a result of a systematic review, only one randomized controlled trial (RCT) was found to be consistent with the patient, intervention, comparison, and outcome (PICO) process, and a meta-analysis was performed using this RCT. This study did not include ventilator-free days (VFD) as an outcome in the PICO sheet but used “duration of ventilation” as an alternative outcome. We could not find any study that reported VILI.  As a beneficial outcome, the effect estimate for the duration of ventilation (1 RCT: N=40) had a mean difference of zero days (95% CI: 7.41 days decrease to 7.41 days increase) for SIMV compared to A/C (VCV or PCV), and the estimated effect on mortality (1 RCT: N=20) was a reduced risk of 100 deaths/1000 people (95% confidence interval: 272 fewer deaths/1000 people to 308 more deaths/1000 people). Thus, we judged the desired effect of the intervention to be “small.” |  |
| Undesirable Effects How substantial are the undesirable anticipated effects? | | |
| JUDGMENT | RESEARCH EVIDENCE | remarks |
| ○Large  ○ Moderate  ○Small  ○Trivial  ○Varies.  ● Do not know | While VILI was considered an outcome of harm, no studies reported it. Therefore, the expected harm was judged to be “Unknown” when considered together with the description in the remarks section. | As for harm caused by SIMV, we referred to observational studies. An observational study by Robinson et al.1) found that a group of patients who experienced more ventilator desynchronization used SIMV significantly more frequently. 1) An observational study by Robinson et al. 1) found that SIMV was used significantly more frequently in a group of patients who experienced more ventilator dyssynchrony, and several studies 2-3) not limited to patients with ARDS also reported longer time to ventilator weaning when SIMV was used as the mode of ventilator weaning compared to other ventilator modes. |
| Certainty of evidence What is the overall certainty of the evidence of effects? | | |
| JUDGMENT | RESEARCH EVIDENCE | remarks |
| ○Very low  ●Low  ○Moderate  ○High  ○No included studies | The relative importance or value of the key outcomes of interest   \| Outcome \| Relative importance \| Certainty of evidence (GRADE) \| \| --- \| --- \| --- \| \| Ventilator days \| Critical \| ⨁⨁◯◯  Low \| \| Morality \| Critical \| ⨁⨁◯◯  Low \| \| VILI \| Critical \| - \|   VILI was not reported within the studies that were employed.  **Overall evidence certainty**:  The direction within the desired effect was consistent, and the certainty of evidence across outcomes was judged to be “low,” adopting the certainty of evidence with the highest certainty. |  |
| Values Is there important uncertainty about or variability in how much people value the main outcomes? | | |
| judgment | RESEARCH EVIDENCE | remarks |
| ○Important uncertainty or variability  ○Possibly important uncertainty or variability  ○Probably no important uncertainty or variability  ●No important uncertainty or variability | No important uncertainty or variability. |  |
| Balance of effects Does the balance between desirable and undesirable effects favor the intervention or the comparison? | | |
| judgment | research evidence | remarks |
| ○Favors the comparison  ○Probably favors the comparison  ○Does not favor either the intervention or the comparison  ●Probably favors the intervention  ○Favors the intervention  ○Varies  ○Do not know | **Summary of findings**:   \| Outcome \| Normal ventilation (control) \| Low volume ventilation (intervention) \| Absolute difference  (95% CI) \| Relative effect  (95% CI) \| \| --- \| --- \| --- \| --- \| --- \| \| Ventilator days \| - \| - \| MD zero days shorter (7.41 days shorter to 7.41 days longer) \| - \| \| Mortality \| 8/20 \| 6/20 \| 100 fewer people/1000 (272 fewer people to 308 more people) \| 0.75 (0.32～1.77) \| \| VILI \|  \|  \|  \| No report \|   VILI was not reported within the included studies.  The balance between the effects and harms of the intervention was judged to be “Probably favors the intervention.” |  |
| Acceptability Is the intervention acceptable to key stakeholders? | | |
| judgment | research evidence | remarks |
| ○ No  ○ Probably no  ● Probably yes  ○ Yes  ○ Varies  ○ Do not know | Since SIMV may have synchrony problems^1^ and several studies, not limited to patients with ARDS, have reported a longer time to ventilator weaning when SIMV was used as the mode of ventilator weaning compared to other ventilator modes^2,3^. Thus, many institutions may be cautious about using SIMV for reasons other than mortality or VILI. |  |
| Feasibility Is the intervention feasible to implement? | | |
| judgment | research evidence | remarks |
| ○ No  ○ Probably no  ● Probably yes  ○ Yes  ○ Varies  ○ Do not know | This is possible because most ventilators have a SIMV setting. |  |

# SUMMARY OF JUDGMENT

|  | | **JUDGMENT** | | | | | | | |
| --- | --- | --- | --- | --- | --- | --- | --- | --- | --- |
| **PROBLEM** | | No | Probably no | Probably yes | Yes |  | Varies | Do not know | |
| **DESIRABLE EFFECTS** | | Trivial | Small | Moderate | Large |  | Varies | Do not know | |
| **UNDESIRABLE EFFECTS** | | Large | Moderate | Small | Trivial |  | Varies | Do not know | |
| **CERTAINTY OF EVIDENCE** | | Very low | Low | Moderate | High |  |  | No included studies | |
| **VALUES** | | Important uncertainty or variability | Possibly important uncertainty or variability | Probably no important uncertainty or variability | No important uncertainty or variability |  |  |  | |
| **BALANCE OF EFFECTS** | | Favors the comparison | Probably favors the comparison | Does not favor either the intervention or the comparison | Probably favors the intervention | Favors the intervention | Varies | Do not know | |
| **ACCEPTABILITY** | | No | Probably no | Probably yes | Yes |  | Varies | Do not know | |
| **FEASIBILITY** | | No | Probably no | Probably yes | Yes |  | Varies | Do not know | |

# TYPE OF RECOMMENDATION

| Strong recommendation against the intervention | Conditional recommendation against the intervention | Conditional recommendation for either the intervention or the comparison | Conditional recommendation for the intervention | Strong recommendation for the intervention |
| --- | --- | --- | --- | --- |
| ○ | ○ | ● | ○ | ○ |

# CONCLUSION

| Recommendation |
| --- |
| **The results of this systematic review do not allow us to make a recommendation as to whether SIMV or assisted ventilation (A/C) should be used in adults with ARDS** **(in our practice statement).**  **Supplementary item**  **A/C is often used rather than SIMV due to the risks of increased asynchrony and prolonged time to ventilator weaning.** |
|  |
| Justification |
| **Question**: Which is preferable for ventilatory management of adult patients with ARDS: synchronized intermittent mandatory ventilation (SIMV) or assisted controlled ventilation (A/C)?  **Patients:** Adult ARDS patients or critically ill ventilated patients  **Intervention:** Ventilatory management with SIMV  **Comparison control**: Ventilatory management with A/C  **Outcomes:** Ventilator days, mortality, and ventilator-induced lung injury (VILI)  **Summary of Evidence**:  As a result of the systematic review, the outcome of VFD was changed to “ventilator days” because no studies were found that included VFD.  In terms of beneficial outcomes, the use of SIMV compared to A/C resulted in a decrease of zero days (95% CI: 7.41 days decrease to 7.41 days increase) in the ventilator days (1 RCT: N=40) and a decrease of 100 deaths (1 RCT: N=40) per 1000 patients (95% CI: 272 fewer to 308 more). Therefore, the expected desired effect was judged to be “probably large.” On the contrary, as an outcome of harm, an observational study by Robinson et al. showed that the group of patients who experienced more patient-ventilator asynchrony used SIMVs significantly more frequently^1)^. In addition, several studies, not limited to patients with ARDS, reported that when SIMV was used as a mode of ventilator weaning, the time to ventilator weaning was longer than with other ventilator modes^2-3)^. Therefore, the expected undesirable effects were judged to be “Unknown.” Thus, the balance of the effects and harms in the meta-analysis was considered to be “probably in favor of the intervention”.  **Certainty of Evidence**:  The only single-center RCT included in the meta-analysis had a small sample size of 40, and death and ventilator days were secondary outcomes. Although there was no significant difference due to the small sample size, there was a trend toward higher midazolam use in the A/C group, which may have contributed to delirium and asynchrony, and may have influenced ventilator days and mortality. Therefore, it is difficult to provide recommendations for the use of SIMV and A/C in this meta-analysis.  **Determining values, balance of effects, acceptance, and viability:**  Although the desired and undesired effects of the intervention are unknown, the cost of changing ventilator setting is expected to be low.  **Panel Meeting:**  　In the pre-vote, the modified Delphi method showed that “the results of this systematic review do not allow us to make a recommendation on whether SIMV or assisted ventilation (A/C) should be used in adult patients with ARDS (in our practice statement),” with a median score of 8.0 and a disagreement index of 0.1316. As a result, the panel meeting finally reached a consensus with the results of the pre-vote without a re-vote.  **Additional Considerations**：  According to the results of a questionnaire administered to 39 physicians, including non-specialists, who were not panel members during the panel meeting, 69.23% disagreed with the choice of SIMV as the mode of initial ventilator setting. Although it may be appropriate to use SIMV depending on the familiarity and practices of the facility, there are reports of increased asynchrony and prolonged time to ventilator weaning, so SIMV should be used with caution^4)^. |

| Subgroup considerations |
| --- |
| None in particular |
| Implementation considerations |
| \| Monitoring and evaluation \| \| --- \| \| To implement the recommendations, it is necessary to collect more information on the current use of SIMV in clinical practice. In addition, it is necessary to monitor whether there are any other clinical problems through the use of questionnaires after the guideline is published. \| \| Research priorities \| \| Although we were not able to make a recommendation in this study due to the high risk of bias in the studies used for the meta-analysis and the discrepancy between routine clinical practice and the results, the clinical question of whether to choose SIMV or A/C remains important in routine practice. Therefore, we hope that high-quality, large-scale RCTs that compare SIMV and A/C will continue to be performed. \|   　If SIMV is used, asynchrony between the ventilator and the patient should be monitored, and if significant asynchrony is observed, other modes should be used. It is not desirable to use SIMV as a weaning mode^2-3)^. |

References

1)Robinson BR, Blakeman TC, Toth P, et al. Patient-ventilator asynchrony in a traumatically injured population. Respir Care*.* 2013;58(11):1847-1855. PMID: 23513248.

2)Brochard L, Rauss A, Benito S, et al. Comparison of three methods of gradual withdrawal from ventilatory support during weaning from mechanical ventilation. Am J Respir Crit Care Med.1994;150(4):896-903. PMID: 7921460.

3)Esteban A, Frutos F, Tobin MJ, et al. A comparison of four methods of weaning patients from mechanical ventilation. Spanish Lung Failure Collaborative Group. N Engl J Med. 1995;332(6):345-350. PMID: 7823995.

4)Kacmarek RM, Branson RD. Should intermittent mandatory ventilation be abolished? Respir Care. 2016;61(6):854-866. PMID: 27235318.

**CQ25 When using mechanical ventilation in adult patients with ARDS with spontaneous breathing, is pressure support ventilation (PSV) or A/C preferred?**

1. Search strategy

MEDLINE via PubMed （Search date: 2020/7/8）

| #1 | respiratory distress syndrome, adult[mh] OR acute respiratory distress syndrom*[tiab] OR respiratory insufficiency[mh] OR acute lung injury[mh] OR acute lung injur*[tiab] OR acute respiratory failure*[tiab] OR ALI[tiab] OR ARDS[tiab] |
| --- | --- |
| #2 | Respiration, Artificial[mh] OR artificilal respiration*[tiab] OR Pulmonary Ventilation[mh] OR pulmonary ventilat*[tiab] OR Ventilators, Mechanical[mh] OR mechanical ventilat*[tiab] OR positive-pressure respiration*[tiab] OR positive pressure ventilat*[tiab] |
| #3 | pressure control*[tiab] OR PCV[tiab] OR volume control*[tiab] OR VCV[tiab] |
| #4 | airway pressure release ventilat*[tiab] OR APRV[tiab] |
| #5 | synchronized intermittent mandatory ventilation[tiab] OR intermittent mandatory ventilation[tiab] OR SIMV[tiab] OR IMV[tiab] |
| #6 | PSV[tiab] OR pressure support[tiab] |
| #7 | #2 OR #3 OR #4 OR #5 OR #6 |
| #8 | (randomized controlled trial [pt] OR controlled clinical trial [pt] OR randomized [tiab] OR placebo [tiab] OR clinical trials as topic [mesh: noexp] OR randomly [tiab] OR trial [ti]) NOT (animals[mh] NOT humans[mh]) |
| #9 | #1 AND #7 AND #8 |

CENTRAL （Search date: 2020/7/8）

| #1 | [mh "respiratory distress syndrome, adult"] OR "acute respiratory distress syndrom":ti,ab OR [mh "respiratory insufficiency"] OR [mh "acute lung injury"] OR "acute lung injury":ti,ab OR "acute respiratory failure":ti,ab OR ALI:ti,ab OR ARDS:ti,ab |
| --- | --- |
| #2 | [mh "Respiration, Artificial"] OR "artificilal respiration":ti,ab OR [mh "Pulmonary Ventilation"] OR "pulmonary ventilation":ti,ab OR [mh "Ventilators, Mechanical"] OR "mechanical ventilation":ti,ab OR "positive-pressure respiration":ti,ab OR "positive pressure ventilation":ti,ab |
| #3 | "pressure control":ti,ab OR PCV:ti,ab OR "volume control":ti,ab OR VCV:ti,ab |
| #4 | "airway pressure release ventilation":ti,ab OR APRV:ti,ab |
| #5 | "synchronized intermittent mandatory ventilation":ti,ab OR "intermittent mandatory ventilation":ti,ab OR SIMV:ti,ab OR IMV:ti,ab |
| #6 | PSV:ti,ab OR "pressure support":ti,ab |
| #7 | {OR #2-#6} |
| #8 | #1 AND #7 |
| #9 | [mh animals] NOT [mh humans] |
| #10 | #8 NOT #9 |

Igaku-Chuo-Zasshi （Search date: 2020/6/26）

| #1 | 呼吸窮迫症候群-急性/TH or 呼吸促迫症候群/AL or ARDS/AL or "acute respiratory distress syndrome"/AL or 急性肺損傷/TH or 急性肺損傷/AL or 急性肺障害/AL or 急性肺傷害/AL or "acute lung Injury"/AL or 呼吸不全/TH or 呼吸不全/AL or 呼吸機能不全/AL |
| --- | --- |
| #2 | 人工呼吸/TH or 人工呼吸/AL or 人工換気/AL or レスピレータ/AL or ベンチレータ/AL or 機械換気/AL or 機械的換気/AL or 人工換気/AL or 調節呼吸/AL or 調節換気/AL or 陽圧呼吸/AL or 陽圧換気/AL |
| #3 | 従圧/AL or 圧規定/AL or 従量/AL or 量規定/AL or PCV/AL or VCV/AL |
| #4 | APRV/AL or 気道内圧開放式/AL or 気道内圧解放式/AL or "Airway Pressure Release Ventilation"/AL |
| #5 | 間欠性強制換気/TH or 同期式間欠的強制/AL or 間欠的強制/AL or SIMV/AL or IMV/AL or "synchronized intermittent mandatory"/AL or "intermittent mandatory"/AL |
| #6 | プレッシャーサポート/AL or PSV/AL or "pressure support"/AL or "pressure-support"/AL |
| #7 | #2 or #3 or #4 or #5 or #6 |
| #8 | (((RD=ランダム化比較試験,準ランダム化比較試験,比較研究) or (ランダム化比較試験/TH or 準ランダム化比較試験/TH or ランダム化/AL or 無作為化/AL or 比較試験/AL or 臨床試験/AL or プラセボ/AL or 対照/AL or コントロール/AL or 臨床研究/AL)) not (CK=動物 not CK=ヒト)) and (PT=会議録除く) |
| #9 | #1 and #7 and #8 |

1. Flow diagram

**Identification**

0 Studies included in qualitative synthesis

4365 records after duplicates removed

5933 records identified through database searching

5933 records identified through database searching

Medline via PubMed (n=2824)

Cochrane CENTRAL (n=2559)

Igaku-Chuo-Zasshi (n=550)

0 additional records identified through other sources

0 Studies included in quantitative synthesis (meta-analysis)

Duplicates

n=1568

4278 records excluded

**Included**

**Eligibility**

**Screening**

メタアナリシスに組み入れた研究n=11

（CQ22：3論文, CQ23：7論文, CQ24：1論文, CQ25：なし）

1. Risk of bias

Not applicable

1. Forest plot

Not applicable

1. Evidence profile

Not applicable

1. Evidence-to-Decision table

| question | |
| --- | --- |
| **CQ25：When using mechanical ventilation in adult patients with ARDS with spontaneous breathing, is pressure support ventilation (PSV) or A/C preferred?** | |
| **Group :** | Adult patients with ARDS that require ventilatory management |
| **INTERVENTIONS:** | PSV |
| **Comparison and contrast :** | A/C (pressure-controlled ventilation [PCV] or volume-controlled ventilation [VCV]) |
| **Main Outcomes:** | Ventilator-free days, mortality, and ventilator-induced lung injury (VILI) |
| **SETTING:** | The emergency room or intensive care unit |
| **Perspectives :** | Personal |
| **Background :** | Ventilatory management in patients with ARDS is as important as treating the primary disease. It is still unclear what the optimal ventilation conditions should be and which of the commonly used ventilation conditions, PSV or assist control ventilation (A/C), is more effective, especially when spontaneous breathing is preserved. |
| **Conflict of Interest:** | None |

# assessment

| Problem Is the problem a priority? | | |
| --- | --- | --- |
| judgment | research evidence | Remarks |
| ○ No  ○ Probably no  ● Probably yes  ○ Yes  ○ Varies  ○ Do not know | Ventilatory management in patients with ARDS is as important as the treatment of the primary disease. It is still unclear what the optimal ventilation regimen should be, and which of the commonly used regimens, pressure support ventilation (PSV) or assist control ventilation (A/C), is more effective, especially when spontaneous breathing is preserved. Therefore, it is important to determine which of the two is more effective, and this issue is of high priority. |  |
| Desirable effects How substantial are the desirable anticipated effects? | | |
| judgment | research evidence | Remarks |
| ○Trivial  ○Small  ○ Moderate  ○Large  ○Varies  ●Do not know | Integrated into the certainty of the evidence. |  |
| Undesirable effects How substantial are the undesirable anticipated effects? | | |
| judgment | research evidence | Remarks |
| ○Large  ○ Moderate  ○Small  ○Trivial  ○Varies.  ● Do not know | Integrated into the certainty of the evidence. |  |
| Certainty of evidence What is the overall certainty of the evidence of effects? | | |
| judgment | research evidence | Remarks |
| ○Very low  ○Low  ○Moderate  ○High  ●No included studies | Mechanical ventilation is an important respiratory support for patients with severe respiratory failure, such as ARDS. Mechanical ventilation can improve oxygenation and reduce the patient’s respiratory effort. However, if there is asynchrony between the patient and the ventilator, mechanical ventilation can lead to lung injury, adverse hemodynamic effects, and ultimately increase the risk of patient mortality.  　PSV is a mode of ventilation that preserves the patient’s spontaneous breathing and provides support during inspiration, which may reduce the likelihood of patient-ventilator asynchrony and associated lung injury compared to the A/C mode. It is also more comfortable to breathe and may reduce the dose of sedatives. However, it is a ventilation mode that depends on spontaneous breathing, and if the patient’s spontaneous breathing effort is too strong, it may increase the volume of one breath, which may impair the lung-protective ventilatory setting.  　No trials have been conducted that compare the balance of effects and harms of these different ventilation modes, and the confidence of the evidence cannot be described. |  |
| Values Is there important uncertainty about or variability in how much people value the main outcomes? | | |
| judgment | research evidence | Remarks |
| ○Important uncertainty or variability  ○Possibly important uncertainty or variability  ●Probably no important uncertainty or variability  ○No important uncertainty or variability | Probably no important uncertainty or variability. |  |
| Balance of effects Does the balance between desirable and undesirable effects favor the intervention or the comparison? | | |
| judgment | research evidence | Remarks |
| ○Favors the comparison  ○Probably favors the comparison  ○Does not favor either the intervention or the comparison  ○Probably favors the intervention  ○Favors the intervention  ○Varies  ●Do not know | No relevant studies. |  |
| Acceptability Is the intervention acceptable to key stakeholders? | | |
| judgment | research evidence | Remarks |
| ○ No  ○ Probably no  ● Probably yes  ○ Yes  ○ Varies  ○ Do not know | Although no evidence was considered, it has already been implemented in daily clinical practice, and we believe it is feasible considering the cost and adverse effects. |  |
| Feasibility Is the intervention feasible to implement? | | |
| judgment | research evidence | Remarks |
| ○ No  ○ Probably no  ○ Probably yes  ● Yes  ○ Varies  ○ Do not know | There is no evidence used for this evaluation, but it seems feasible because it is already being used in daily clinical practice. |  |

# Summary of Judgment

|  | | JUDGMENT | | | | | | | |
| --- | --- | --- | --- | --- | --- | --- | --- | --- | --- |
| **PROBLEM** | | No | Probably no | Probably yes | Yes |  | Varies | Do not know | |
| **DESIRABLE EFFECTS** | | Trivial | Small | Moderate | Large |  | Varies | Do not know | |
| **UNDESIRABLE EFFECTS** | | Large | Moderate | Small | Trivial |  | Varies | Do not know | |
| **CERTAINTY OF EVIDENCE** | | Very low | Low | Moderate | High |  |  | No included studies | |
| **VALUES** | | Important uncertainty or variability | Possibly important uncertainty or variability | Probably no important uncertainty or variability | No important uncertainty or variability |  |  |  | |
| **BALANCE OF EFFECTS** | | Favors the comparison | Probably favors the comparison | Does not favor either the intervention or the comparison | Probably favors the intervention | Favors the intervention | Varies | Do not know | |
| **ACCEPTABILITY** | | No | Probably no | Probably yes | Yes |  | Varies | Do not know | |
| **FEASIBILITY** | | No | Probably no | Probably yes | Yes |  | Varies | Do not know | |

# Type of recommendation

| Strong recommendation against the intervention | Conditional recommendation against the intervention | Conditional recommendation for either the intervention or the comparison | Conditional recommendation for the intervention | Strong recommendation for the intervention |
| --- | --- | --- | --- | --- |
| ○ | ○ | ● | ○ | ○ |

# Conclusion

| Recommendation |
| --- |
| **No recommendation can be made for the PSV versus A/C mode for adult patients with ARDS. It is common practice to select a ventilation mode based on the individual patient’s condition and status (in our practice statement).**  **Supplementary item:**  **Based on the presence or absence of spontaneous breathing, the amount of sedatives and muscle relaxants used, and the severity of ARDS, the patient should be individualized to determine which mode should be used.** |
|  |
| Justification |
| **Question:** When using mechanical ventilation in adult patients with ARDS with spontaneous breathing, is pressure support ventilation (PSV) or A/C preferred?  **Patients:** Adult patients with ARDS that require ventilatory management  **Intervention:** PSV  **Comparison control:** A/C (PCV or VCV)  **Explanation:**  Mechanical ventilation is an important respiratory support for patients with severe respiratory failure, such as ARDS. Mechanical ventilation can improve oxygenation and reduce the patient’s respiratory effort. However, if there is an asynchrony between the patient and ventilator, mechanical ventilation can lead to lung injury, adverse hemodynamic effects, and ultimately increase the risk of patient mortality.  　PSV is a mode of ventilation that preserves the patient’s spontaneous breathing and provides support during inspiration, which may reduce the likelihood of patient-ventilator asynchrony and associated lung injury compared to the A/C mode. It is also more comfortable to breathe and may reduce the dose of sedatives. However, it is a ventilation mode that depends on spontaneous breathing, and if the patient’s spontaneous breathing effort is too strong, it may increase the volume of one breath, which may impair the lung-protective ventilatory setting.  　Because there is no high-quality evidence to compare the balance of the benefits and harms of these different ventilation modes, we cannot provide a clear recommendation for this CQ. Therefore, this CQ is not an evidence-based recommendation but only a description of the current practice.  **Summary of evidence**: No relevant studies  **Certainty of evidence**: Since there are no relevant studies, the quality of the evidence cannot be assessed.  **Determining values, balance of effects, acceptance, and viability:**  Since there are no studies on the benefits and harms, no evaluation is possible. Since the only intervention is to change the ventilation mode, and since both ventilation modes are available on standard ventilators, the burden and cost required is probably judged to be small.  **Panel Meeting**  　In a prior vote, the modified Delphi method stated, “No recommendation can be made for PSV mode versus A/C mode in adults with ARDS. It is common practice to select a ventilation mode based on the pathology and condition of the individual patient (in our practice statement),” with a median score of 8.0 and a disagreement index of 0.1918. As a result, the panel meeting finally reached a consensus with the result of the pre-vote without a re-vote being required. |

| Subgroup considerations |
| --- |
| None |
| Implementation considerations |
| No statement was found in the ARDS Clinical Practice Guidelines 2016 or ATS/ESICM/SCCM 2017 regarding whether PSV or A/C is preferred. It is also believed that both modes are often used in a single patient.  Based on the presence or absence of spontaneous breathing, the amount of sedatives and muscle relaxants used, and the severity of ARDS, the patient should be individualized to determine which mode should be used. |

| Monitoring and evaluation |
| --- |
| To implement the recommendations, it is necessary to collect more information on the actual timing of switching from A/C to PSV as a clinical problem. In addition, it is necessary to monitor whether there are any other clinical problems through the use of questionnaires after the guideline is published. |
| Research priorities |
| There are no studies in the field of this CQ that should be referred to.  In the future, randomized controlled trials that compare the PSV and A/C modes in adult patients with ARDS or severe respiratory failure are needed. |

**CQ26 Should we perform a recruitment maneuver when ventilating an adult patient with ARDS?**

1. Search strategy

MEDLINE via PubMed （Search date: 2020/6/11）

| #1 | ("respiratory insufficiency"[Mesh] OR "respiratory insufficiency"[tiab]) AND acute[tiab] |
| --- | --- |
| #2 | "acute lung injury"[Mesh] OR 　"pulmonary atelectasis"[Mesh] OR "hypoxia"[Mesh] OR "acute respiratory failure"[tiab] OR "lung collapse"[tiab] OR "alveoli |
| #3 | "Respiratory Distress Syndrome, Adult"[Mesh] OR "acute respiratory distress"[tiab] OR "acute respiratory distress syndrome"[tiab] |
| #4 | ARDS[tiab] OR ALI[tiab] OR RDS[tiab] |
| #5 | #1 OR #2 OR #3 OR #4 |
| #6 | recruitment[tiab] OR derecruitment[tiab] |
| #7 | "positive-pressure respiration"[MeSH] OR "respiration, artificial"[MeSH] OR "Pulmonary reexpansion*"[tiab] OR "open lung"[tiab] OR "incremental peep"[tiab] |
| #8 | recruit*[tiab] AND (manoe*[tiab] OR maneuv*[tiab] OR manuev*[tiab]) |
| #9 | LRM[tiab] |
| #10 | #6 OR #7 OR #8 OR #9 |
| #11 | #5 AND #10 |
| #12 | randomized controlled trial[pt] OR controlled clinical trial[pt] OR randomized[tiab] OR placebo[tiab] OR clinical trials as topic[mesh:noexp] OR |
| #13 | #11 AND #12 |

CENTRAL （Search date: 2020/6/11）

| #1 | ([mh "respiratory insufficiency"] OR "respiratory insufficiency":ti,ab,kw) AND acute:ti,ab,kw |
| --- | --- |
| #2 | [mh "acute lung injury"] OR [mh "pulmonary atelectasis"] OR [mh hypoxia] OR "acute respiratory failure":ti,ab,kw OR "lung collapse":ti,ab,kw OR "alveoli collapse":ti,ab,kw OR "respiratory depression":ti,ab,kw OR "ventilatory depression":ti,ab,kw |
| #3 | [mh "Respiratory Distress Syndrome, Adult"] OR "acute respiratory distress":ti,ab,kw OR "acute respiratory distress syndrome":ti,ab,kw |
| #4 | ARDS:ti,ab,kw OR ALI:ti,ab,kw OR RDS:ti,ab,kw |
| #5 | {OR #1-#4} |
| #6 | recruitment:ti,ab,kw OR derecruitment:ti,ab,kw |
| #7 | [mh "positive-pressure respiration"] OR [mh "respiration, artificial"] OR "Pulmonary reexpansion*":ti,ab,kw OR "open lung":ti,ab,kw OR "incremental |
| #8 | [mh "positive-pressure respiration"] OR [mh "respiration, artificial"] OR "Pulmonary reexpansion*":ti,ab,kw OR "open lung":ti,ab,kw OR "incremental |
| #9 | LRM:ti,ab,kw |
| #10 | {OR #6-#9} |
| #11 | #5 AND #10 |

Igaku-Chuo-Zasshi （Search date: 2020/6/11）

| #1 | 呼吸窮迫症候群-急性/TH or 急性呼吸窮迫症候群/AL or ARDS/AL or RDS/AL |
| --- | --- |
| #2 | 急性肺損傷/TH or 急性肺損傷/AL |
| #3 | 呼吸不全/TH or 呼吸不全/AL |
| #4 | 酸素欠乏/TH or 低酸素症/AL or hypoxia/AL |
| #5 | #1 or #2 or #3 or #4 |
| #6 | recruitment/AL or derecruitment/AL or リクルートメント/AL |
| #7 | オープンラング/AL or open/AL and (肺/TH or lung/AL) |
| #8 | 人工呼吸/TH or LRM/AL |
| #9 | 呼気/AL and 終末/AL and 陽圧/AL |
| #10 | #6 or #7 or #8 or #9 |
| #11 | #5 and #10 |
| #12 | ランダム化比較試験/TH or ランダム化/AL or 無作為化/AL |
| #13 | 比較試験/AL |
| #14 | 臨床試験/TH or 臨床試験/AL |
| #15 | プラセボ/TH or プラセボ/AL |
| #16 | 対照/AL |
| #17 | コントロール/AL |
| #18 | 臨床研究・疫学研究/TH or 臨床研究/AL |
| #19 | #12 OR #13 OR #14 OR #15 OR #16 OR #17 OR #18 |
| #20 | #11 AND #19 |
| #21 | (#20) and (PT=会議録除く) |

1. Flow diagram

**identification**

Studies matching for eligibility

(n=14)

重複研究

(n=796)

Full-text articles assessed for eligibility (n=90)

n=90

Records screened

(n=3293)

Records identified

(n=4089)

Records identified through database searching

n=4089

Medline（PubMed） 2069

Ichu-shi 416

CENTRAL 1604

医中誌 416件

Additional records identified through other sources

n=0

**^included^**

**eligibility**

**screening**

Full-text articles excluded, with reasons

(n=76)

Reasons：

Different language　 (n=11)

Different study design　(n=42)

Wrong Intervention or Control (n=23)

Records excluded

(n=3203)

Studies included in quantitative synthesis(meta-analysis) n=14

n=14

1. Risk of bias

Ventilator-free days 28-day mortality


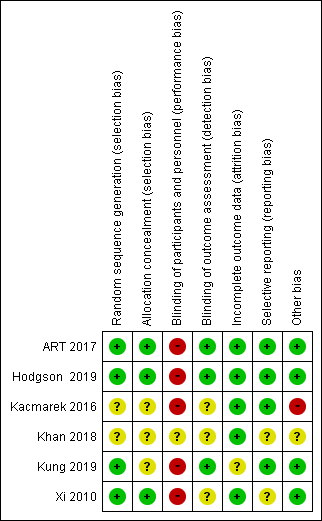

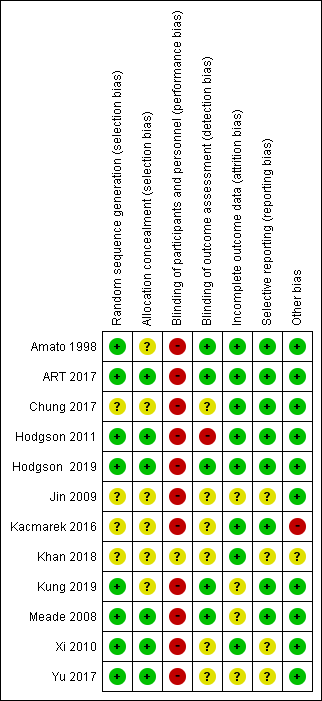


Length of ICU stay P/F ratio


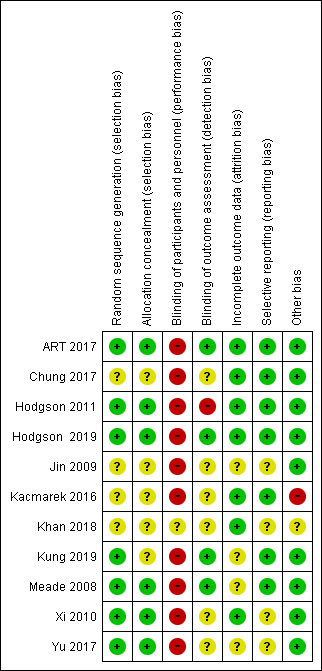

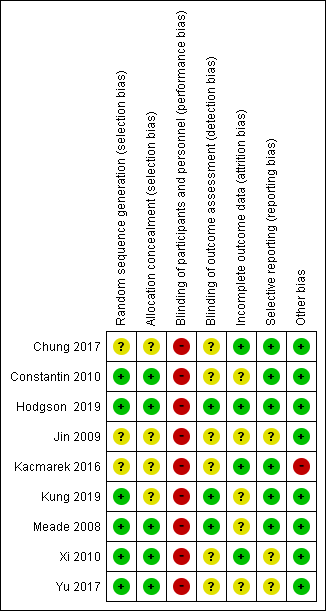


Barotrauma  **Circulatory failure**


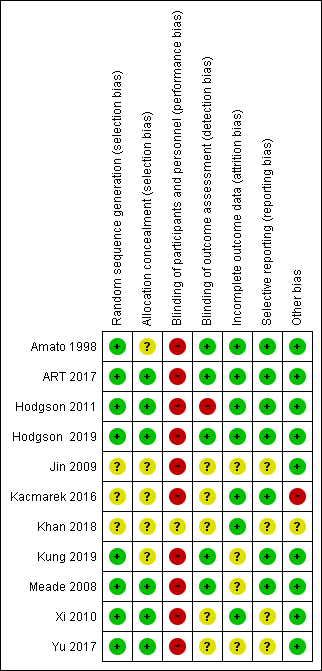

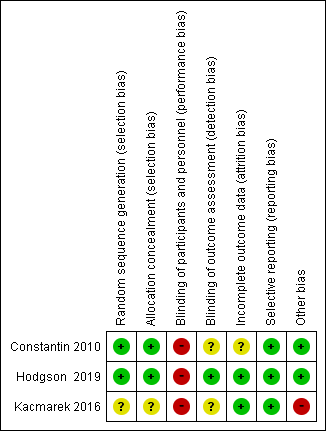


**Rescue treatment**


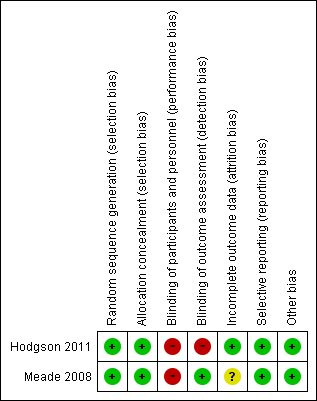


1. Forest plot

Ventilator-free days


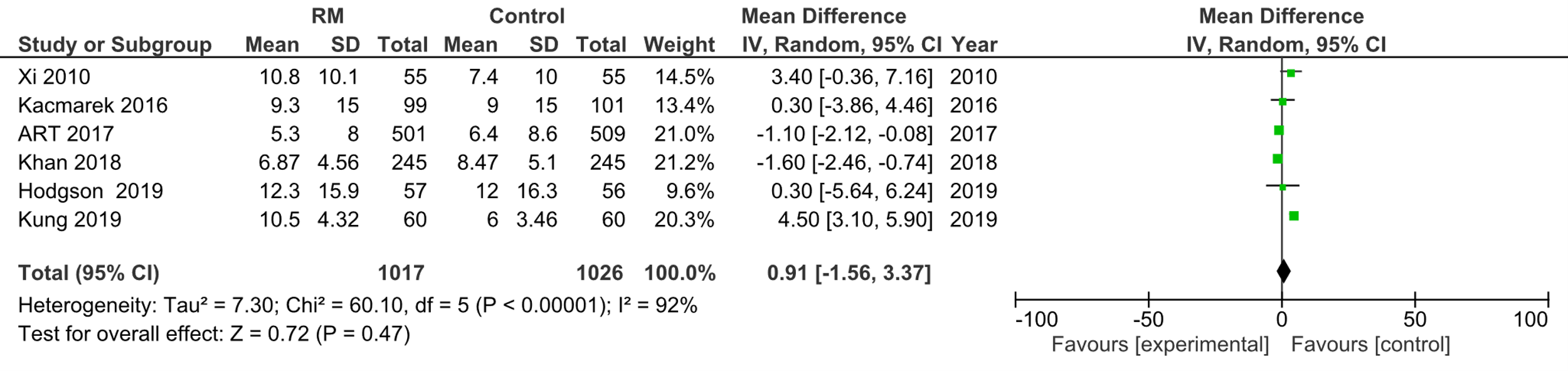


28-day mortality


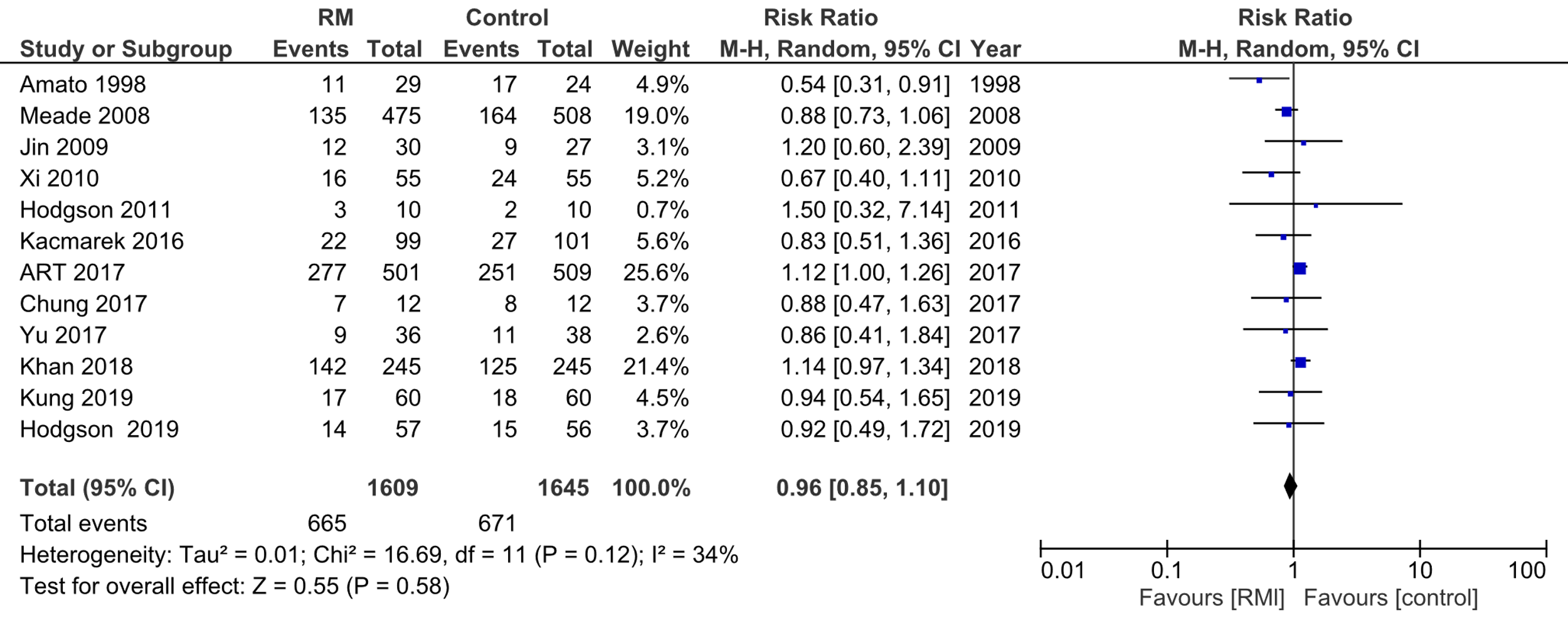


Length of ICU stay


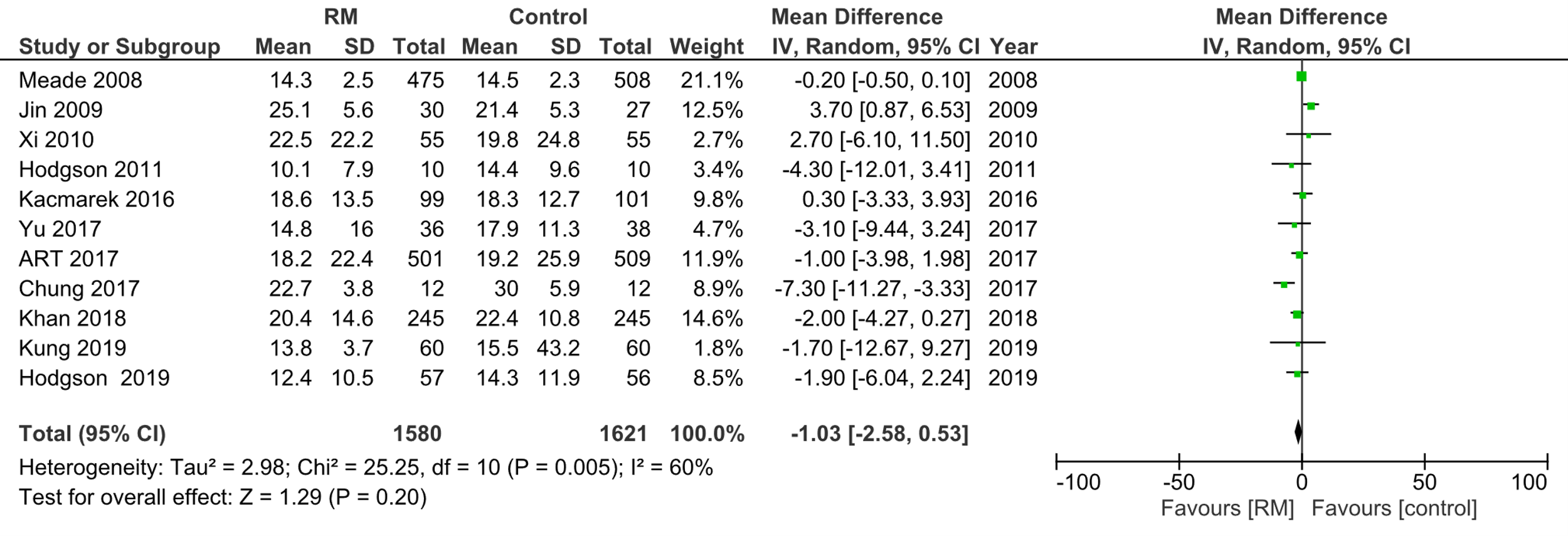


P/F ratio


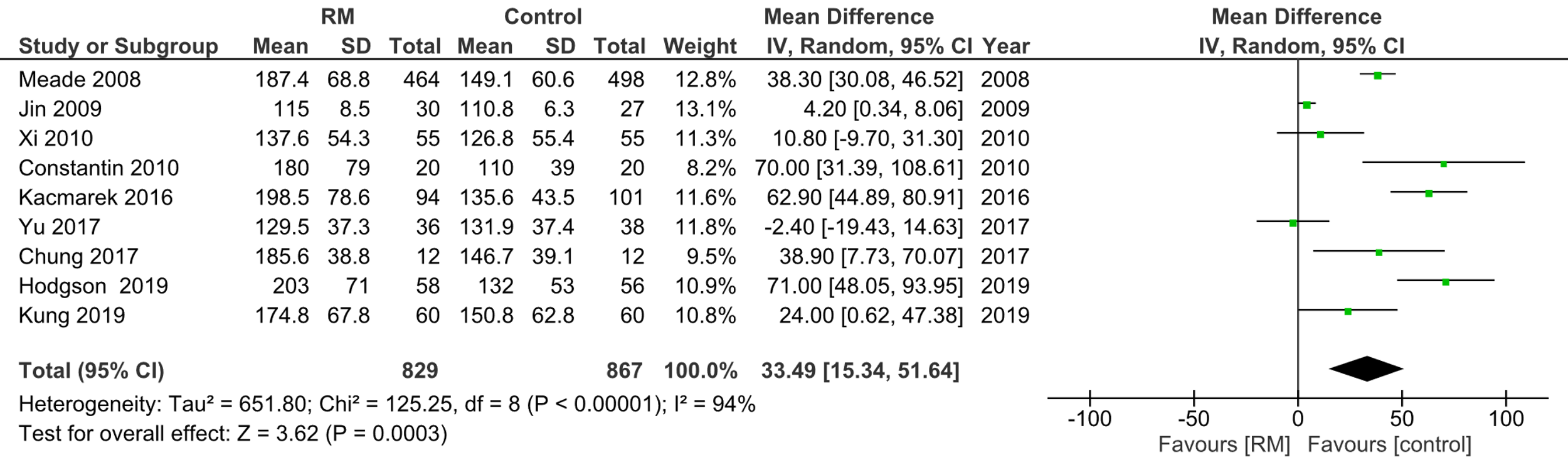


Barotrauma


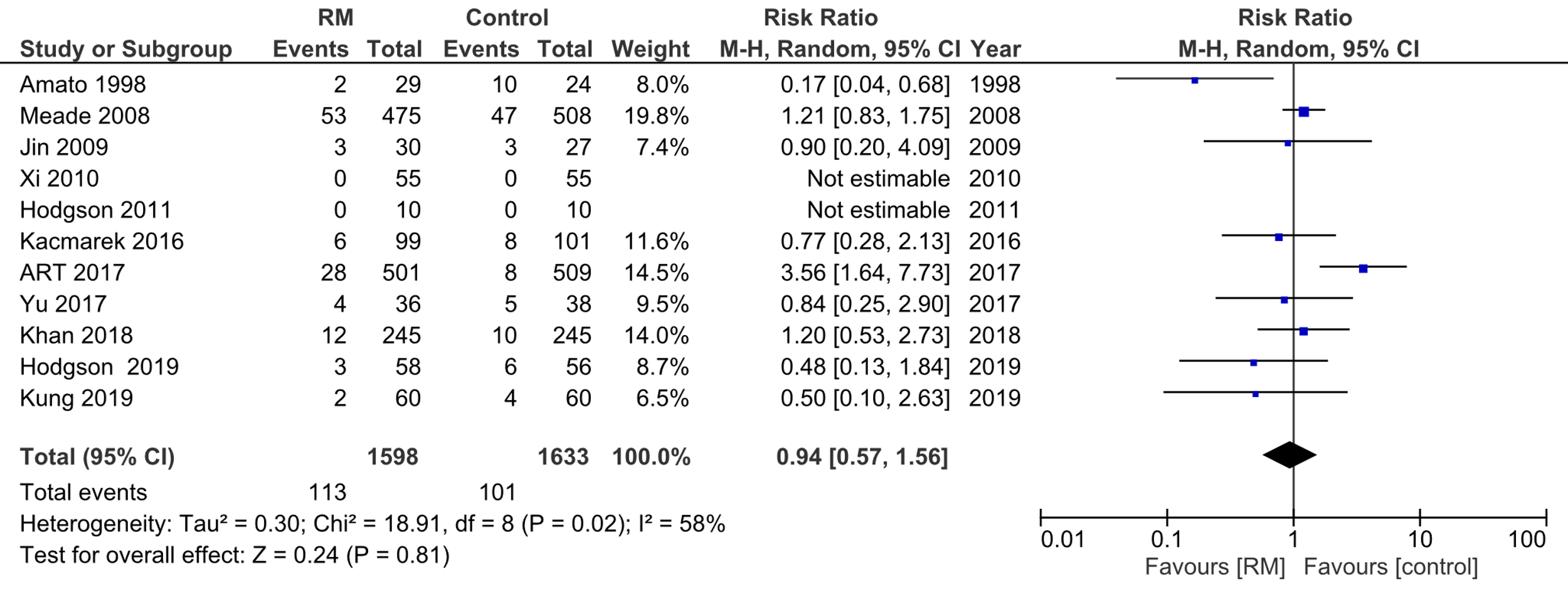


**Circulatory failure**


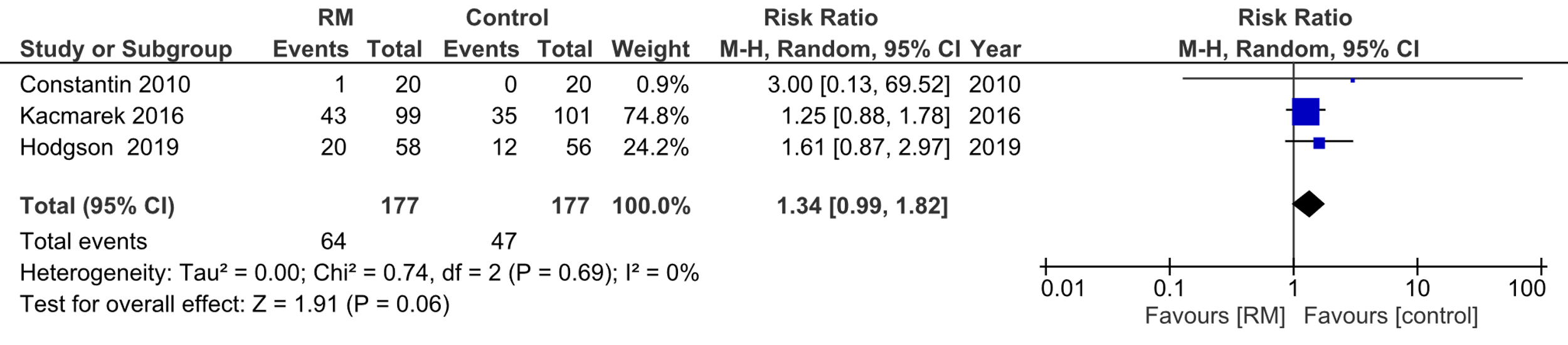


**Rescue treatment**


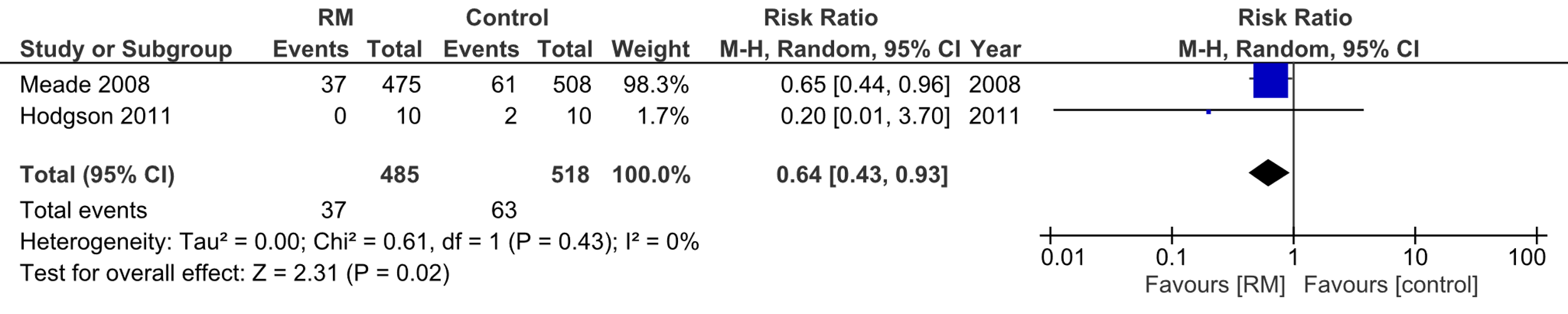


1. Evidence profile

| **Certainty assessment** | | | | | | | **№ of patients** | | **Effect** | | **Certainty** | **Importance** |
| --- | --- | --- | --- | --- | --- | --- | --- | --- | --- | --- | --- | --- |
| **№ of studies** | **Study design** | **Risk of bias** | **Inconsistency** | **Indirectness** | **Imprecision** | **Other considerations** | **With recruitment maneuver** | **Without recruitment maneuver** | **Relative (95% CI)** | **Absolute (95% CI)** |  |  |
| **Ventilator-free Days** | | | | | | | | | | | | |
| 6 | Randomized trials | Not serious | Serious ^a^ | Not serious | Not serious | None | 1017 | 1026 | - | **MD 0.91 days　longer**  (1.56 shorter to3.37 longer) | ⨁⨁⨁◯ Moderate | Critical |
| **28-day mortality** | | | | | | | | | | | | |
| 12 | Randomized trials | Not serious | Not serious | Not serious | Not serious | None | 665/1609 (41.3%) | 671/1645 (40.8%) | **RR 0.96** (0.85 to 1.10) | **16 fewer per 1000** (61 fewer to 41 more) | ⨁⨁⨁⨁ High | Critical |
| **Length of ICU stay** | | | | | | | | | | | | |
| 11 | Randomized trials | Not serious | Serious ^b^ | Not serious | Not serious | None | 1580 | 1621 | - | **MD 1.03 days shorter** (2.58 shorter to 0.53 longer) | ⨁⨁⨁◯ Moderate | Critical |
| **PaO_2_/F_I_O_2_ ratio** | | | | | | | | | | | | |
| 9 | Randomized trials | Not serious | Serious ^c^ | Not serious | Not serious | None | 829 | 867 | - | **MD 33.49 mmHg higher** (15.34 higher to 51.64 higher) | ⨁⨁⨁◯ Moderate | Important |
| **Barotrauma** | | | | | | | | | | | | |
| 11 | Randomized trials | Not serious | Serious ^d^ | Not serious | Very serious ^e^ | None | 113/1598 (7.1%) | 101/1633 (6.2%) | **RR 0.94** (0.57 to 1.56) | **4 fewer per 1000** (27 fewer to 35 more) | ⨁◯◯◯ Very low | Critical |
| **Circulatory failure** | | | | | | | | | | | | |
| 3 | Randomized trials | Not serious | Not serious | Not serious | Serious ^f^ | None | 64/177 (36.2%) | 47/177 (26.6%) | **RR 1.34** (0.99 to 1.82) | **90 more per 1000** (3 fewer to 218 more) | ⨁⨁⨁◯ Moderate | Critical |
| **Rescue treatment** | | | | | | | | | | | | |
| 2 | Randomized trials | Not serious | Not serious | Not serious | Serious ^g^ | None | 37/485 (7.6%) | 63/518 (12.2%) | **RR 0.64** (0.43 to 0.93) | **44 fewer per 1000** (69 fewer to 9 fewer) | ⨁⨁⨁◯ Moderate | Important |

**CI:** confidence interval; **RR:** risk ratio; **MD**, mean difference

#### Explanation

a. The confidence intervals partially overlapped and there was a large heterogeneity, I2=92%, P<0.00001; therefore, we decided to downgrade by one level.

b. The confidence intervals partly overlapped and there was a large heterogeneity, I2=60%, P=0.005; therefore, we decided to downgrade by one level.

c. The confidence intervals partly overlapped and there was a large heterogeneity, I2=94%, P<0.00001; therefore, we decided to downgrade by one level.

d. The confidence intervals partially overlapped and there was moderate heterogeneity (I2=58%, P=0.02); therefore, we decided to downgrade by one level.

e. Since the (95%) confidence interval was sufficiently wide to span the upper and lower thresholds, we decided to downgrade by two levels.

f. It straddles the threshold above the (95%) confidence interval.

g. The optimal information size (OIS) was not met.

1. Evidence-to-Decision table

| **CQ26：Should we perform a recruitment maneuver when ventilating an adult patient with ARDS?** | |
| --- | --- |
| **GROUP:** | Adult patients with ARDS that require ventilatory management |
| **INTERVENTIONS:** | Some kind of recruitment maneuvers performed after the onset of ARDS |
| **Comparison and CONTRAST:** | Non-implementation of recruitment maneuvers |
| **Main Outcomes:** | Ventilator-free days (VFD), 28-day mortality, length of intensive care unit (ICU) stay, barotrauma (pneumothorax, mediastinal emphysema, subcutaneous emphysema, or pneumatoceles), circulatory failure (cardiac arrest or hypotension) |
| **SETTING:** | The emergency room or ICU |
| **PERSPECTIVES:** | Personal |
| **BACKGROUND:** | In patients with ARDS, atelectasis occurs in the lungs, leading to respiratory failure due to decreased air content and the development of ventilatory induced lung injury (VILI) due to shear stress. This type of atelectasis may be improved with a positive pressure recruitment maneuver. The recruitment maneuvers are low-cost interventions that can be performed at the bedside and have been validated in multiple RCTs, suggesting that they may improve oxygenation and lung compliance and reduce the need for rescue therapies such as veno-venous extracorporeal membrane oxygenation (VV-ECMO). In contrast, high positive pressure ventilation may lead to complications such as circulatory failure and barotrauma. Considering these circumstances, a recommendation based on meta-analysis is necessary to determine whether or not to perform a recruitment maneuver for ARDS. |
| **Conflict of Interest:** | None |

# assessment

| Problem Is the problem a priority? | | |
| --- | --- | --- |
| judgment | research evidence | note |
| ○ No  ○ Probably no  ○ Probably yes  ● Yes  ○ Varies  ○ Do not know | In patients with ARDS, atelectasis occurs in the lungs, leading to respiratory failure due to decreased air content and the development of ventilatory induced lung injury (VILI) due to shear stress. This type of atelectasis may be improved with a positive pressure recruitment maneuver. The recruitment maneuvers are low-cost interventions that can be performed at the bedside and have been validated in multiple RCTs, suggesting that they may improve oxygenation and lung compliance and reduce the need for rescue therapies such as veno-venous extracorporeal membrane oxygenation (VV-ECMO). In contrast, high positive pressure ventilation may lead to complications such as circulatory failure and barotrauma. Considering these circumstances, a recommendation based on meta-analysis is necessary to determine whether or not to perform a recruitment maneuver for ARDS. Therefore, this question has a high priority. |  |
| Desirable effects How substantial are the desirable anticipated effects? | | |
| judgment | research evidence | note |
| ●Trivial  ○Small  ○ Moderate  ○Large  ○Varies  ○Do not know | As a result of the systematic review, 14 randomized controlled trials (RCTs) consistent with the patient, intervention, comparison, and outcome process were identified, and a meta-analysis was implemented with these trials.  As beneficial outcomes, the effect estimate for the duration of VFD at 28 days (6RCTs: N=2043) was 0.91 days longer (95% CI 1.56 days shorter to 3.37 days longer) for the intervention compared to the control. The effect estimate for 28-day mortality (12 RCTs: N=3254) was 16 fewer/1000 (61 fewer to 41 more), and the effect estimate for ICU length of stay (11 RCTs: N=3201) had a mean difference of 1.03 days shorter (95% CI 2.58 days shorter to 0.53 days longer). Therefore, we judged the favorable effect to be “trivial”. |  |
| Undesirable effects How substantial are the undesirable anticipated effects? | | |
| judgment | research evidence | note |
| ○Large  ○ Moderate  ●Small  ○Trivial  ○Varies.  ○ Do not know | As outcomes of harm, the effect estimate for barotrauma (pneumothorax, mediastinal emphysema, subcutaneous emphysema, or pneumatoceles) (11 RCTs: N=3231) was 4 fewer patients/1000 (95% CI: 27 fewer to 35 more), and for circulatory failure (cardiac arrest or hypotension) (3 RCTs: N=354) (3 RCT: N=354) was 90 more (95% CI: 3 fewer to 218 more). Therefore, the unfavorable effect was considered “small.” |  |
| Certainty of evidence What is the overall certainty of the evidence of effects? | | |
| judgment | research evidence | note |
| ●Very low  ○Low  ○Moderate  ○High  ○No included studies | **The relative importance or value of the key outcomes of interest**   \| Outcome \| Relative importance \| Certainty of evidence (GRADE) \| \| --- \| --- \| --- \| \| VFD \| serious \| ⨁⨁⨁◯ moderate \| \| 28-day mortality \| serious \| ⨁⨁⨁⨁  high \| \| ICU length of stay \| serious \| ⨁⨁⨁◯  moderate \| \| Barotrauma \| serious \| ⨁◯◯◯  very low \| \| Circulatory failure \| serious \| ⨁⨁⨁◯  moderate \|   Overall certainty of evidence:  Since the directions of the favorable and unfavorable effects were not consistent, the certainty of the evidence for the overall outcome was decided to be “very low” by adopting the certainty of the evidence with the lowest certainty.  . |  |
| Values Is there important uncertainty about or variability in how much people value the main outcomes? | | |
| judgment | research evidence | note |
| ○Important uncertainty or variability  ○Possibly important uncertainty or variability  ●Probably no important uncertainty or variability  ○No important uncertainty or variability | There are no data on the values for outcomes in management with the recruitment maneuvers, but the value for death is generally high, and the variability is low. |  |
| Balance of effects Does the balance between desirable and undesirable effects favor the intervention or the comparison? | | |
| judgment | research evidence | note |
| ○Favors the comparison  ●Probably favors the comparison  ○Does not favor either the intervention or the comparison  ○Probably favors the intervention  ○Favors the intervention  ○Varies  ○Do not know | Summary of the results:   \| Outcome \| Without recruitment maneuver (control) \| Recruitment maneuver (intervention) \| Absolute effect (95% CI) \| Relative effect RR (95% CI) \| \| --- \| --- \| --- \| --- \| --- \| \| VFD \| - \| - \| MD 0.91 day longer (1.56 shorter to 3.37 longer) \| - \| \| 28-day mortality \| 671/1645 \| 665/1609 \| 16 fewer per 1000 (61 fewer to 41 more) \| RR 0.96 (0.85-1.10) \| \| ICU length of stay \| - \| - \| MD 1.03 days shorter  (2.58 shorter to 0.53 longer) \| - \| \| Barotrauma \| 101/1633 \| 113/1598 \| 4 fewer per 1000 (27 fewer to 35 more) \| RR 0.94 (0.57-1.56) \| \| Circulatory failure \| 47/177 \| 64/177 \| 90 more per 1000 (3 fewer to 218 more) \| RR 1.34 (0.99-1.82) \|   Based on the above, we concluded that the balance between the benefits and harms was “probably more harmful” based on the above. |  |
| Acceptability Is the intervention acceptable to key stakeholders? | | |
| judgment | research evidence | note |
| ○ No  ○ Probably no  ○ Probably yes  ○ Yes  ○ Varies  ● Do not know | Recruitment maneuvers are feasible in facilities where medical staff are educated and trained, and where circulation monitoring is available to respond to any harm that may occur. However, its acceptance is uncertain because the balance between the harm of increased circulatory failure and the benefit of improved oxygenation and reduced rescue therapy for patients and providers is not certain. |  |
| Feasibility Is the intervention feasible to implement? | | |
| judgment | research evidence | note |
| ○ No  ○ Probably no  ● Probably yes  ○ Yes  ○ Varies  ○ Do not know | It is probably feasible in facilities with adequate education, training, and hemodynamic monitoring. |  |

**Summary of Judgment**

|  | | **JUDGMENT** | | | | | | | |
| --- | --- | --- | --- | --- | --- | --- | --- | --- | --- |
| **PROBLEM** | | No | Probably no | Probably yes | Yes |  | Varies | Do not know | |
| **DESIRABLE EFFECTS** | | Trivial | Small | Moderate | Large |  | Varies | Do not know | |
| **UNDESIRABLE EFFECTS** | | Large | Moderate | Small | Trivial |  | Varies | Do not know | |
| **CERTAINTY OF EVIDENCE** | | Very low | Low | Moderate | High |  |  | No included studies | |
| **VALUES** | | Important uncertainty or variability | Possibly important uncertainty or variability | Probably no important uncertainty or variability | No important uncertainty or variability |  |  |  | |
| **BALANCE OF EFFECTS** | | Favors the comparison | Probably favors the comparison | Does not favor either the intervention or the comparison | Probably favors the intervention | Favors the intervention | Varies | Do not know | |
| **ACCEPTABILITY** | | No | Probably no | Probably yes | Yes |  | Varies | Do not know | |
| **FEASIBILITY** | | No | Probably no | Probably yes | Yes |  | Varies | Do not know | |

# Type of recommendation

| Strong recommendation against the intervention | Conditional recommendation against the intervention | Conditional recommendation for either the intervention or the comparison | Conditional recommendation for the intervention | Strong recommendation for the intervention |
| --- | --- | --- | --- | --- |
| ○ | ● | ○ | ○ | ○ |

# Conclusion

| Recommendation |
| --- |
| **We conditionally suggest that recruitment maneuvers should not be used routinely in adult patients with ARDS.**  **(Conditional recommendation/Very low certainty of evidence: GRADE 2D)**  **Supplementary item:**  **Recruitment procedures may be feasible to improve the P/F ratio and avoid rescue therapy if the medical staff are adequately educated and trained to monitor the circulation and respond to a life-threatening situation such as cardiac arrest.** |
|  |
| Justification |
| **Clinical Question:** Should we perform a recruitment maneuver when ventilating an adult patient with ARDS?  **Patients:** Adult patients with ARDS  **Intervention:** Some kind of recruitment maneuvers performed after the onset of ARDS  **Control:** Non-implementation of recruitment maneuvers  **Outcome:** VFD, 28-day mortality, ICU length of stay, barotrauma, circulatory failure  **Summary of evidence:**  When comparing patients with and without the recruitment maneuver, VFD (6 RCTs: N=2043) increased by a mean difference of 0.91 days (95% CI 1.56 days shorter to 3.37 days longer), 28-day mortality (12 RCTs: N=3254) decreased by 16 per 1000 patients (95% CI 61 fewer to 41 more), and ICU length of stay (11 RCTs: N=3201) decreased by 1.03 days (95% CI 2.58 days shorter to 0.53 days longer). The duration of ICU stay (11 RCTs: N=3201) was reduced by 1.03 days (95% CI 2.58 days to 0.53 days longer).  We decided that the desired effect was “only” based on the above. In contrast, the harm outcomes decreased by 4 per 1000 (95% CI 27 to 35) in barotrauma (11 RCTs; n=3231) and increased by 90 per 1000 (3 to 218) in circulatory failure (3 RCTs; n=354). As a result, regarding whether the benefits of the intervention were greater than the harms, we decided that the harms probably outweighed the benefits.  Certainty of evidence:  The direction of the outcome of the favorable and unfavorable effects was not consistent, and the outcome was decided to be “very low” with the least certainty among all outcomes.  **Determining values, balance of effects, acceptance, and viability:**  The recruitment procedure’s load and cost are not considered significant, as it only requires education and training. Regarding benefits and harms, the effects for the critical outcomes of 28-day mortality, VFD, and ICU length of stay could be either slight benefit or slight harm. Furthermore, since circulatory failure, including cardiac arrest, tended to increase, the net benefit was deemed to be uncertain, and the decision for implementation should be situationally based.  **Panel meeting:**  In the pre-vote, the modified Delphi method resulted in a median score of 9.0 and a disagreement index of 0.1316 for “We suggest the recruitment technique should not be used routinely in adult patients with ARDS” (weak recommendation against/very low certainty evidence: GRADE 2D). As a result, the panel meeting finally reached a consensus with the result of the pre-vote without a re-vote being required.  **Additional recommendation:**  The recruitment techniques used in the included studies differed significantly in terms of the duration of the procedure, the target airway pressure during the procedure, and whether the procedure was followed by high positive end-expiratory pressure (PEEP) or open lung therapy. The possibility of favorable results may be expected depending on the method of the recruitment maneuvers. In addition, most of the studies included in this meta-analysis were for moderate to severe ARDS. Therefore, considering the risk of circulatory failure and other problems in this study, the procedure should be avoided in patients with mild ARDS. |

| Subgroup considerations |
| --- |
| None |
| Implementation considerations |
| There were no recommendations in the ARDS Clinical Practice Guidelines 2016 regarding the use of recruitment maneuvers for ARDS. The ATS/ESICM/SCCM2017 ARDS guidelines recommended the use of the recruitment maneuvers for adult patients with ARDS. This was the guideline before the Acute Respiratory Distress Syndrome Trial (ART) study was published and may have been influenced by several recent studies, including the ART study, which suggested that the recruitment maneurvers worsens mortality.  Recruitment maneuvers should be performed by educated and trained medical staff, with careful monitoring of circulation, and the procedure should be interrupted when there are concerns of hemodynamic deterioration.  There is no known optimal procedure, and at the least, protocols from studies with increased mortality, such as the ART study, should be avoided ( i.e., ART study method: PEEP at 25 cmH_2_O for 1 min, PEEP at 35 cmH_2_O for 1 min, PEEP at 45 cmH_2_O for 2 min).  When implementing the procedure, additional assessments (e.g., P/F ratio, rescue therapy, barotrauma, monitoring for hemodynamic compromise) may also increase confidence. |

| Monitoring and evaluation |
| --- |
| To apply the recommendations, more information needs to be accumulated regarding the methods of performing recruitment. In addition, surveillance for other clinical problems should be conducted through the use of questionnaires and other measures after the guidelines are published. |
| Research priorities |
| Studies are needed to find the optimal recruitment maneuvers that improves oxygenation, the frequency of rescue therapy, and minimizes circulatory deterioration. CT-guided and ultrasound-guided recruiting maneuvers have also been studied. Thus, a meta-analysis considering the heterogeneity of the procedures should be conducted in the future. |

References

1) Writing Group for the Alveolar Recruitment for Acute Respiratory Distress Syndrome Trial (ART) Investigators. Effect of lung recruitment and titrated positive end-expiratory pressure (PEEP) vs low PEEP on mortality in patients with acute respiratory

distress syndrome a randomized clinical trial. JAMA. 2017;318(14):1335-1345. PMID: 28973363.

**CQ27 Are ventilator weaning protocols useful in patients with mechanical ventilation?**

1. Search strategy

MEDLINE via PubMed （Search date: 2020/5/26）

| #1 | "respiration, artificial"[MeSH Terms] OR "artificilal respiration*"[Title/Abstract] OR "Pulmonary Ventilation"[MeSH Terms] OR "pulmonary ventilat*"[Title/Abstract] OR "ventilators, mechanical"[MeSH Terms] OR "mechanical ventilat*"[Title/Abstract] |
| --- | --- |
| #2 | "positive-pressure respiration"[MeSH Terms] OR "positive-pressure respiration"[Title/Abstract] |
| #3 | "Negative-Pressure Respirator"[Title/Abstract] OR "negative pressure respirators "[Title/Abstract] OR "Tank Ventilators"[Title/Abstract] OR "Tank Ventilator"[Title/Abstract] |
| #4 | "assist ventilation"[Title/Abstract] or "Adaptive support ventilation"[Title/Abstract] |
| #5 | #1 OR #2 OR #3 |
| #6 | #4 OR #5 |
| #7 | "Ventilator Weaning"[MeSH Terms] OR "Weaning Ventilator"[Title/Abstract] OR "Respirator Weaning"[Title/Abstract] OR "mechanical ventilator weaning "[Title/Abstract] |
| #8 | (((("mechanical"[Title/Abstract] AND "ventilat*"[Title/Abstract]) AND "weaning"[Title/Abstract]) AND "protocol weaning"[Title/Abstract]) OR "protocolised weaning"[Title/Abstract]) OR "protocolized weaning"[Title/Abstract] or "weaning protocol "[Title/Abstract]) |
| #9 | "systematic approach"[Title/Abstract] |
| #10 | #7 or #8 or #9 |
| #11 | ((((((("randomized controlled trial"[Publication Type] OR "controlled clinical trial"[Publication Type]) OR "randomized"[Title/Abstract]) OR "randomly"[Title/Abstract]) OR "trial"[Title/Abstract]) OR "groups"[Title/Abstract]) NOT ("animals"[MeSH Terms] NOT "humans"[MeSH Terms]) |
| #12 | #6 AND #10 AND #11 |

CENTRAL （Search date: 2020/6/3）

| #1 | [mh "respiration, artificial"] OR artificilal NEXT respiration*:ti,ab OR [mh "Pulmonary Ventilation"] OR pulmonary NEXT ventilat*:ti,ab OR [mh "ventilators, mechanical"] OR mechanical NEXT ventilat*:ti,ab |
| --- | --- |
| #2 | [mh "positive-pressure respiration"] OR "positive-pressure respiration":ti,ab |
| #3 | "Negative-Pressure Respirator":ti,ab OR "negative pressure respirators":ti,ab OR "Tank Ventilators":ti,ab OR "Tank Ventilator":ti,ab |
| #4 | "assist ventilation":ti,ab OR "Adaptive support ventilation":ti,ab |
| #5 | #1 OR #2 OR #3 OR #4 |
| #6 | [mh "Ventilator Weaning"] OR "Weaning Ventilator":ti,ab OR "Respirator Weaning":ti,ab OR "mechanical ventilator weaning":ti,ab |
| #7 | ((((mechanical:ti,ab AND ventilat*:ti,ab) AND weaning:ti,ab) AND "protocol weaning":ti,ab) OR "protocolised weaning":ti,ab) OR "protocolized weaning":ti,ab OR "weaning protocol":ti,ab |
| #8 | "systematic approach":ti,ab |
| #9 | #6 OR #7 OR #8 |
| #10 | #5 AND #9 |

1. Flow diagram

**Screening**

**Identification**

13 Studies included in qualitative synthesis

Duplicates

n=1226

48 Full-text articles assessed for

eligibility

3301 records after duplicates

removed

4574 records identified through

database searching

4574 records identified through

database searching

Medline via PubMed (n=2421)

CENTRAL (n=2056)

Igaku-Chuo-Zasshi (n=97)

0 additional record identified

through other sources

3253 records excluded

35 full-text articles

excluded, with reasons:

-Wrong study design(n=12)

-Wrong population (n=14)

-Wrong intervention (n=9)

**Included**

**Eligibility**

13 Studies included in quantitative synthesis (meta-analysis)

1. Risk of bias

In-hospital mortality ICU mortality


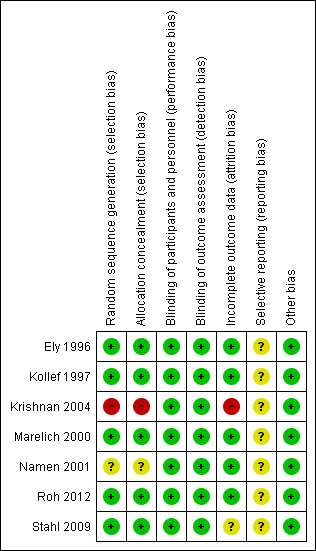

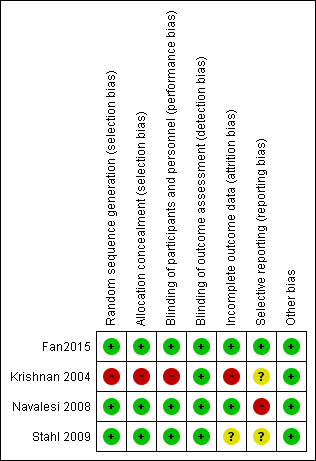


Duration of mechanical ventilator Length of hospital stay


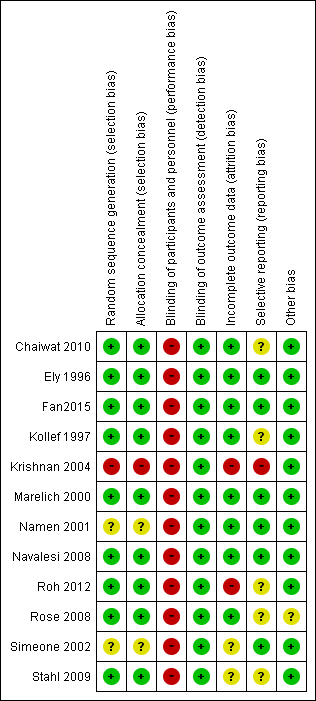

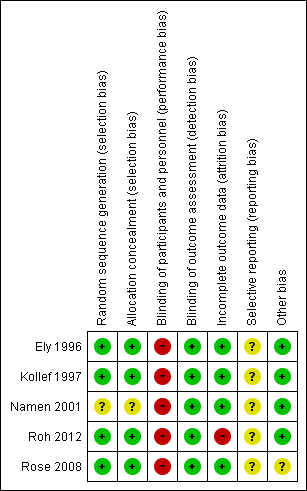


Length of ICU stay Tracheostomy


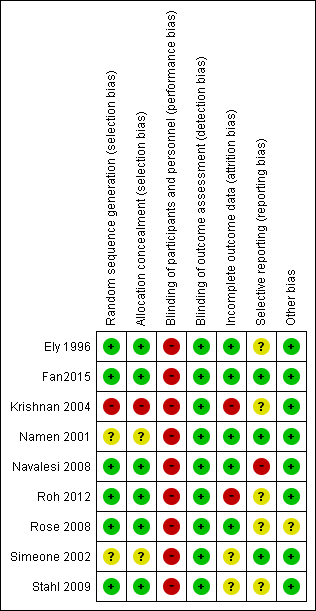

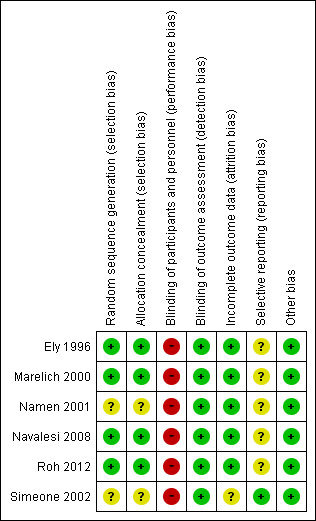


Re-intubation


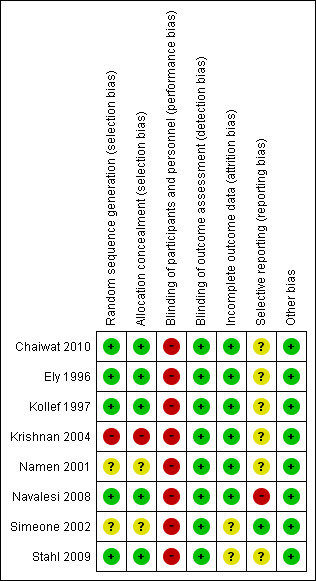


1. Forest plot

Hospital mortality


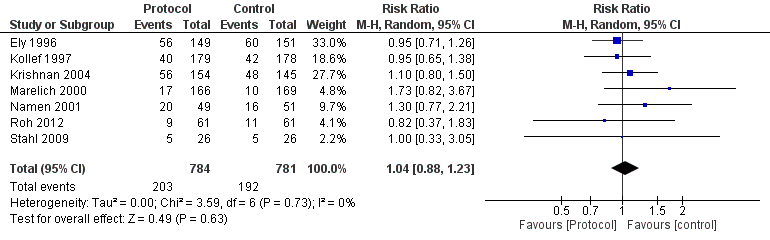


ICU mortality


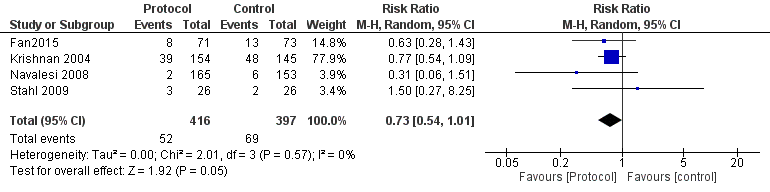


Duration of mechanical ventilation


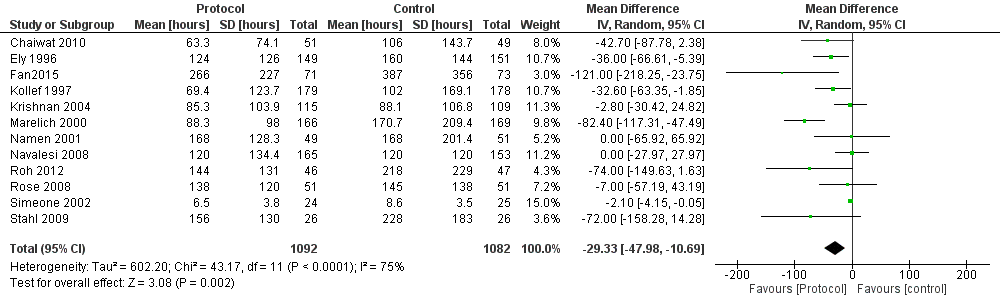


Length of hospital stay


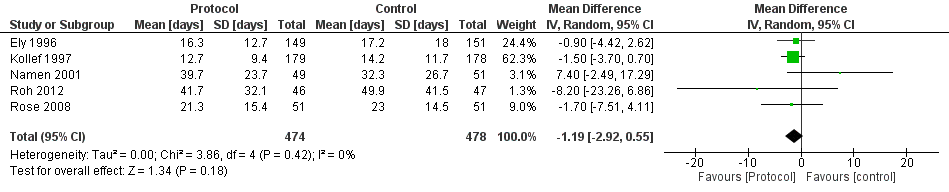


Length of ICU stay


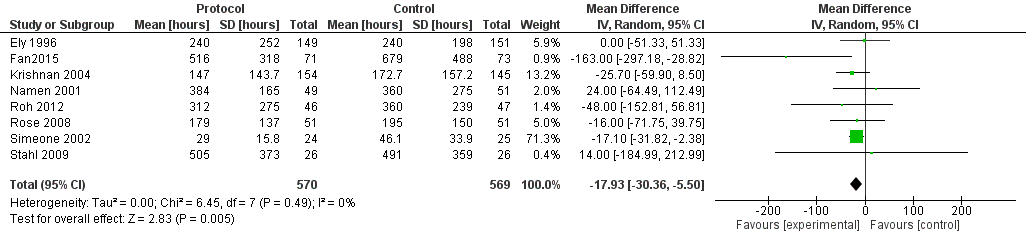


tracheostomy


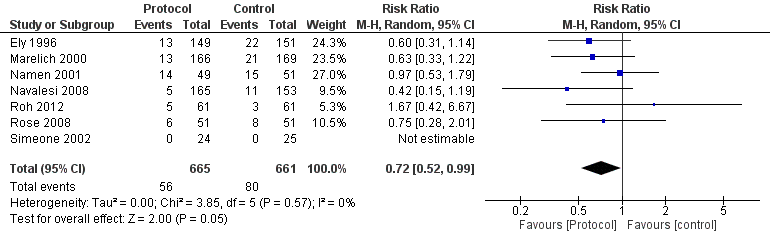


Re-intubation


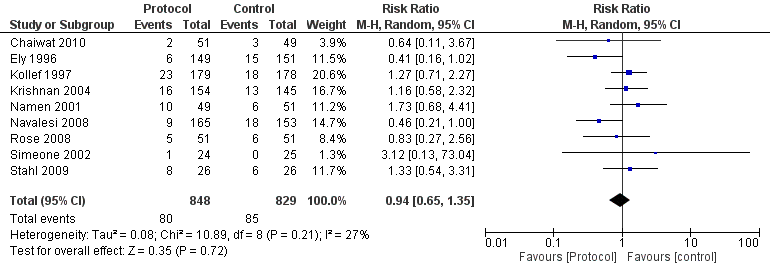


1. Evidence profile

| **Assessment of certainty** | | | | | | | **No. of patients** | | **Efficacy** | | **Certainty of the evidence** | **Importance** |
| --- | --- | --- | --- | --- | --- | --- | --- | --- | --- | --- | --- | --- |
| **No. of studies** | **Study design** | **Risk of bias** | **Inconsistency** | **Indirectness** | **Imprecision** | **Others** | **Protocolized** | **Non-protocolized** | **Relative index (95% CI)** | **Absolute index (95% CI)** |  |  |
| **In-hospital mortality** | | | | | | | | | | | | |
| 7 | Randomized trial | Not serious | Not serious | Serious^a^ | Not serious^b^ | None | 203/784 (25.9%) | 192/781 (24.6%) | **RR 1.04**  (0.88 to 1.23) | **10 more per 1000**  (30 fewer to 57 more) | ⨁⨁⨁◯  MODERATE | Critical |
| **ICU mortality** | | | | | | | | | | | | |
| 4 | Randomized trial | Serious^c^ | Not serious | Serious^a^ | Serious^d^ | None | 52/416 (12.5%) | 69/397 (17.4%) | **RR 0.73**  (0.54 to 1.01) | **47 fewer per 1000** (80 fewer to 2 more) | ⨁◯◯◯  VERY LOW | Critical |
| **Duration of mechanical ventilation** | | | | | | | | | | | | |
| 12 | Randomized trial | Serious^e^ | Serious^f^ | Serious^a^ | Not serious | None | 1092 | 1082 | - | **MD 29.33 hours shorter** (47.98 shorter to 10.69 shorter) | ⨁◯◯◯  VERY LOW | Critical |
| **Length of hospital stay** | | | | | | | | | | | | |
| 5 | Randomized trial | Serious^g^ | Not serious | Serious^a^ | Serious^h^ | None | 474 | 478 | - | **MD 1.19 days shorter** (2.92 shorter to 0.55 longer) | ⨁◯◯◯  VERY LOW | Critical |
| **Length of ICU stay** | | | | | | | | | | | | |
| 9 | Randomized trial | Serious^i^ | Not serious | Serious^a^ | Not serious | None | 735 | 722 | - | **MD 17.84 hours shorter** (29.67 shorter to 6.02 shorter) | ⨁⨁◯◯  LOW | Critical |
| **Tracheostomy** | | | | | | | | | | | | |
| 7 | Randomized trial | Serious^j^ | Not serious | Serious^a^ | Not serious^k^ | None | 56/665 (8.4%) | 80/661 (12.1%) | **RR 0.72**  (0.52 to 0.99) | **34 fewer per 1000** (58 fewer to 1 fewer) | ⨁⨁◯◯  LOW | Critical |
| **Re-intubation** | | | | | | | | | | | | |
| 9 | Randomized trial | Serious^l^ | Serious^m^ | Serious^a^ | Very serious^n^ | None | 80/848 (9.4%) | 85/829 (10.3%) | **RR 0.94**  (0.65 to 1.35) | **6 fewer per 1000** (36 fewer to 36 more) | ⨁◯◯◯  VERY LOW | Critical |

**CI**: confidence interval; **RR:** risk ratio; **MD**, mean difference; **ICU**: intensive care uni

**Description.**

1. The study population included many postoperative patients without respiratory failure, and the possibility of differences in results compared with patients with ARDS is considered, and it is decided that the certainty grade should be lowered by one level.
2. The total sample size is 1565 and the number of events is 395, which does not meet the optimal information size (OIS); however, the width of the confidence interval does not include "substantial harm" and "substantial benefit"; therefore, there is no grade down.
3. In the risk of bias category, the proportion of references judged to be high risk or unknown risk is 4/4, which is judged to be "serious" and downgraded by one level.
4. The total sample size of 813 and the number of events of 121 do not meet the OIS, and the 95% confidence intervals are wide and include "no effect" and "substantial benefit."
5. In terms of risk of bias, the proportion of references judged to be high risk or risk unknown is 12/12, which is judged to be "serious" and downgraded by one level.
6. I^2^=75% and the effect variation included in the systematic review is judged to be "serious" and downgraded by one level based on a visual forest plot.
7. In the risk of bias, the proportion of references judged to be high risk or risk unknown is 5/5, which is judged to be "serious" and downgraded by one level.
8. The total sample size of 953 does not meet the OIS and was downgraded by one level.
9. In terms of risk of bias, the proportion of references judged to be high risk or risk unknown is 9/9, which is judged to be "serious" and downgraded by one level.
10. In the risk of bias, the proportion of references judged to be high risk or risk unknown is 7/7, which is judged to be "serious" and downgraded by one level.
11. Although the total sample size of 1224 and the number of events of 122 do not meet the OIS, the confidence intervals are consistent with an effect, and no grade down is assumed.
12. In terms of risk of bias, the proportion of references judged to be high risk or risk unknown was 9/9, which is judged to be "serious" and downgraded by one level.
13. I^2^=35%; however, the point estimates of the incorporated studies differed (clinical heterogeneity) and are considered "serious" and downgraded one level.
14. The total sample size of 1575 and the number of events of 154 do not meet the OIS, and the 95% confidence interval is wide and the confidence interval includes "substantial harm" and "substantial benefit."
15. Evidence-to-Decision table

| question | |
| --- | --- |
| **CQ27: Are ventilator weaning protocols useful in patients with mechanical ventilation?** | |
| **GROUP:** | Adult patients who required ventilatory support for more than 24 hours (studies in which more than 50% of eligible patients were tracheostomized were excluded) |
| **INTERVENTIONS:** | Use of ventilator weaning protocols (daily spontaneous breathing trial (SBT), computer-driven weaning/automated weaning, and adaptive support ventilation are considered protocols) |
| **Comparison and CONTRAST:** | Non-protocolized ventilator weaning procedures (at the discretion of the medical provider) |
| **Main Outcomes:** | In-hospital mortality, intensive care unit (ICU) mortality, duration of ventilation (hours), length of ICU stay (hours), length of hospital stay (days), tracheostomy, reintubation |
| **SETTING:** | **The emergency room or ICU** |
| **PERSPECTIVES:** | **Personal** |
| **BACKGROUND:** | Prolonged ventilatory management may increase adverse events such as ventilator-associated pneumonia, and premature extubation may increase reintubation and mortality. Therefore, early and reliable determination of the timing of ventilator weaning is important. Although ventilator weaning is often at the discretion of the clinician, protocol-based weaning may be useful in shortening the duration of ventilator use. Therefore, it is necessary to validate whether ventilatory weaning using a protocol reduces mortality. |
| **Conflict of Interest:** | None |

# assessment

| Problem Is the problem a priority? | | |
| --- | --- | --- |
| judgment | research evidence | note |
| ○ No  ○ Probably no  ● Probably yes  ○ Yes  ○ Varies  ○ Do not know | Prolonged ventilatory management may increase adverse events such as ventilator-associated pneumonia, and premature extubation may increase reintubation and mortality. Therefore, early and reliable determination of the timing of ventilator weaning is important. Although ventilator weaning is often at the discretion of the clinician, protocol-based weaning may be useful in shortening the duration of ventilator use. Therefore, it is necessary to validate whether ventilatory weaning using a protocol reduces mortality. Therefore, this issue is probably of high priority. |  |
| Desirable effects How substantial are the desirable anticipated effects? | | |
| judgment | research evidence | note |
| ○Trivial  ●Small  ○ Moderate  ○Large  ○Varies  ○Do not know | The results of the systematic review did not identify any randomized controlled trials that included only patients who met the diagnostic criteria for ARDS. Therefore, in this CQ, we conducted a meta-analysis using 13 randomized controlled trials (RCTs) consistent with the patient, intervention, comparison, and outcome (PICO) process, which included patients who had been on ventilatory support for at least 24 hours. Piotto 2011, which was included in the previous guideline, was excluded because the protocol was also used in the control group, and Fan 2015 was newly included.  The effect estimate for in-hospital mortality (seven RCTs: N=1565) was higher in the “protocol group” than in the “non-protocol group”. The effect estimate for ICU deaths (4 RCTs: N=813) was a decrease of 47 patients/1000 (95% CI: decrease of 80 patients to an increase of 2 patients), and the effect estimate for the duration of ventilation (12 RCTs: N=2174) was a decrease of 29.33 hours (95% CI: 47.98 h decrease to 10.69 h decrease).  The effect estimate for the length of hospital stay (10 RCTs: N=952) had a mean difference of 1.19 days shorter (95% CI: 2.92 days shorter to 0.55 days longer), the effect estimate for ICU stay (9 RCTs: N=1457) had a mean difference of 17.84 hours shorter (95% CI: 29.67 hours shorter to 6.02 hours shorter), and the effect estimate for tracheostomy (6 RCTs: N=1224) had a risk difference of 34 fewer patients per 1000 in the protocol group (95% CI: 58 person reduction to 1 person reduction) in the protocol group, and the effect estimate for reintubation (8 RCTs: N=1575) was a risk difference of 6 fewer (95% CI: 36 fewer to 36 more). Thus, we judged the desired effect of the intervention to be “small.” |  |
| Undesirable effects How substantial are the undesirable anticipated effects? | | |
| judgment | research evidence | note |
| ○Large  ○ Moderate  ○Small  ○Trivial  ○Varies.  ● Do not know | No potentially harmful outcomes of using the protocol were identified. Therefore, the undesirable effects were judged to be “ Do not know.” |  |
| Certainty of evidence What is the overall certainty of the evidence of effects? | | |
| judgment | research evidence | note |
| ●Very low  ○Low  ○Moderate  ○High  ○No included studies | The relative importance or value of the key outcomes of interest:   \| **Outcome** \| **Relative importance** \| **Certainty of evidence**  **(GRADE)** \| \| --- \| --- \| --- \| \| In-hospital mortality \| Serious \| ⨁⨁⨁◯  Moderate \| \| ICU mortality \| Serious \| ⨁◯◯◯  Very low \| \| Length of time on ventilation \| Serious \| ⨁◯◯◯  Very low \| \| Hospital stay \| Serious \| ⨁◯◯◯  Very low \| \| Length of ICU stay \| Serious \| ⨁⨁◯◯  Low \| \| Tracheotomy \| Serious \| ⨁⨁◯◯  Low \| \| Reintubation \| Serious \| ⨁◯◯◯  Very low \|   **The certainty of the overall evidence:**  Outcome direction within the desired effect was not consistent, and the certainty of the evidence across outcomes was judged to be “very low,” adopting the certainty of the least certain evidence. |  |
| Values Is there important uncertainty about or variability in how much people value the main outcomes? | | |
| judgment | research evidence | note |
| ○Important uncertainty or variability  ○Possibly important uncertainty or variability  ●Probably no important uncertainty or variability  ○No important uncertainty or variability | There are no data on the values for outcomes in management with ventilator weaning protocols, but the value for death is generally high, and the variability is low. |  |
| Balance of effects Does the balance between desirable and undesirable effects favor the intervention or the comparison? | | |
| judgment | research evidence | note |
| ○Favors the comparison  ○Probably favors the comparison  ○Does not favor either the intervention or the comparison  ●Probably favors the intervention  ○Favors the intervention  ○Varies  ○Do not know | Summary of results:   \| Outcome \| Discretion of the person in charge  (contrast) \| Protocol use  (Intervention) \| Absolute difference (95% CI) \| Relative effect  RR  (95% CI) \| \| --- \| --- \| --- \| --- \| --- \| \| In-hospital mortality \| 192/781 \| 203/784 \| 10 persons more/1,000  (30 fewer to 57 more) \| RR 1.04  (0.88-1.23) \| \| ICU mortality \| 69/397 \| 52/416 \| 47 people fewer/1,000  (80 fewer to 2 more) \| RR 0.73  (0.54-1.01) \| \| Length of time on ventilation  (hours) \| -. \| -. \| MD 29.33 h shorter (47.98 h shorter - 10.69 h shorter) \| -. \| \| Hospital stay \| -. \| -. \| MD 1.19 days shorter  (2.92 days shorter to 0.55 days longer) \| -. \| \| Length of ICU stay (hours) \| - \| - \| MD 17.84 h shorter (29.67 h shorter - 6.02 h shorter) \| - \| \| Reintubation \| 85/829 \| 80/848 \| 6 people fewer/1,000  (36 fewer to 36 more) \| RR 0.94  (0.65-1.35) \|   As a result, the balance of the effects and harms was judged to be “probably in favor of intervention.” |  |
| Acceptability Is the intervention acceptable to key stakeholders? | | |
| judgment | research evidence | note |
| ○ No  ○ Probably no  ○ Probably yes  ○ Yes  ● Varies  ○ Do not know | It may be acceptable because of its low cost, low burden to the patient, low risk of complications, and reduced duration of ventilation and ICU stay. On the contrary, introducing and implementing special ventilation may be unacceptable because it requires additional cost and training. |  |
| Feasibility Is the intervention feasible to implement? | | |
| judgment | research evidence | note |
| ○ No  ○ Probably no  ○ Probably yes  ○ Yes  ● Varies  ○ Do not know | It may be feasible for medical personnel to establish a protocol for weaning from mechanical ventilation and implement the protocol. On the contrary, some facilities may not be able to introduce a special ventilator for automatic weaning and implement a weaning protocol. |  |

**Summary of Judgment**

|  | JUDGMENT | | | | | | |
| --- | --- | --- | --- | --- | --- | --- | --- |
| **PROBLEM** | No | Probably no | Probably yes | Yes |  | Varies | Do not know |
| **DESIRABLE EFFECTS** | Trivial | Small | Moderate | Large |  | Varies | Do not know |
| **UNDESIRABLE EFFECTS** | Large | Moderate | Small | Trivial |  | Varies | Do not know |
| **CERTAINTY OF EVIDENCE** | Very low | Low | Moderate | High |  |  | No included studies |
| **VALUES** | Important uncertainty or variability | Possibly important uncertainty or variability | Probably no important uncertainty or variability | No important uncertainty or variability |  |  |  |
| **BALANCE OF EFFECTS** | Favors the comparison | Probably favors the comparison | Does not favor either the intervention or the comparison | Probably favors the intervention | Favors the intervention | Varies | Do not know |
| **ACCEPTABILITY** | No | Probably no | Probably yes | Yes |  | Varies | Do not know |
| **FEASIBILITY** | No | Probably no | Probably yes | Yes |  | Varies | Do not know |

# Type of recommendation

| Strong recommendation against the intervention | Conditional recommendation against the intervention | Conditional recommendation for either the intervention or the comparison | Conditional recommendation for the intervention | Strong recommendation for the intervention |
| --- | --- | --- | --- | --- |
| ○ | ○ | ○ | ● | ○ |

# Conclusion

| Recommendation |
| --- |
| **Conditional recommendation to perform a protocolized ventilator weaning procedure in adult patients with ARDS**  **(Conditional recommendation/evidence of very low certainty: GRADE 2D).**  **Supplementary item:**  **When a new ventilator setting is changed by a multidisciplinary team of healthcare professionals by introducing a protocol, it is important to educate healthcare professionals and to pay close attention to patient monitoring.** |
|  |
| Justification |
| **Question:** Are ventilator weaning protocols useful in patients with mechanical ventilation?  **Patients:** Adult patients requiring ventilatory support for more than 24 h (studies in which more than 50% of the patients were tracheostomized were excluded)  **Intervention:** Ventilator weaning protocol used (Daily SBT, computer-driven weaning/automated weaning, adaptive support ventilation are considered protocols)  **Comparison and contrast:** Non-protocolized ventilator weaning methods (at the discretion of the medical provider)  **Outcomes:** In-hospital mortality, ICU mortality, duration of ventilation, length of ICU stays, length of hospital stay, tracheostomy, reintubation  **Summary of Evidence:**  The results of the systematic review did not reveal any RCTs that included only patients who met the diagnostic criteria for ARDS. Therefore, we conducted a meta-analysis using 13 RCTs that were consistent with PICO in patients who were ventilated for more than 24 h. We conducted a meta-analysis using these trials.  Using the protocol, we found that in-hospital mortality (7 RCTs: N=1565) increased by 10 per 1000 (95% confidence interval: decrease of 30 to increase of 57), ICU mortality (4 RCTs: N=813) were 47 fewer per 1000 (95% CI: 80 decrease to 2 increase), and the duration of ventilation (12 RCTs: N=2174) was on average 29.33 h shorter (95% CI: 47.98 decrease to 10.69 decrease), length of hospital stay (10 RCTs: N=952) decreased by an average of 1.19 days (95% CI: 2.92 decrease to 0.55 increase), and length of ICU stay (9 RCTs: N=1457) decreased by an average of 17.84 h (95% CI 29.67 decrease to 6.02 decrease), and tracheostomy (6 RCTs: N=1224) decreased by 34 per 1000 patients (95% CI 58 decrease to 1 increase). Intubation (8 RCTs; N=1575) was associated with a decrease of 6 per 1000 (95% confidence interval; 36 decrease to 36 increase). Therefore, we judged the expected desired effect to be “small.” There were no potentially harmful outcomes associated with the use of the protocol. Therefore, the expected undesirable effect was judged to be “unknown.” Thus, we judged the effect of the intervention to be “probably greater” than the harm. The effect was judged to be “probably large.”  **Certainty of evidence:**  The direction of the outcomes within the desired effect is not consistent, and the certainty of the evidence across the outcomes is less than the certainty of the least certain evidence.  It was adopted and judged to be “very low.”  **Determining values, balancing effects, acceptance, and viability:**  Although the association between protocol implementation and in-hospital mortality is unclear, it is expected to reduce the duration of ventilation and ICU stay and is associated with an increase in adverse events such as reintubation and tracheostomy.  No association was detected, so the benefits may outweigh the harms. While the cost of implementation is negligible, except in cases requiring special ventilation, the potential cost and time involved in developing protocols and training personnel is a consideration.  **Panel discussion:**  In the pre-vote, under the Modified Delphi Method, the “recommended text” had a median score of 8.0 and a disagreement index of 0.1168. As a result, at the Panel meeting, a re-vote was held.  In the end, an agreement was reached using the results from the preliminary vote.  **Additional considerations:**  There is clinical heterogeneity in patients and interventions in this meta-analysis. Regarding patient heterogeneity, there is clinical heterogeneity in this meta-analysis, which included surgical ICU, medical ICU, coronary ICU, and neurosurgical ICU patients. In a subgroup analysis in the Cochrane meta-analysis,1) there was an effect modification by ICU type with respect to duration of ventilation (larger effect by protocolized weaning in medical ICU, surgical ICU, and surgical and medical ICU, smaller effect in neuro ICU). Another was the heterogeneity of interventions. There is heterogeneity of interventions in withdrawal protocols: regular spontaneous breathing test protocols, gradual decrease in ventilator support protocols, human protocols and automatic ventilator weaning protocols. (see also subgroup analysis below) . A subgroup analysis in the Cochrane meta-analysis1) found no effect modification between a protocol of gradually decreasing ventilator support and a protocol of routine spontaneous breathing tests during the mechanical ventilation period (P=0.12 for interaction), and no effect modification between a human protocol and an automatic ventilator weaning protocol. No effect modification was detected in the subgroup analysis of the automatic weaning protocol with ventilator (P=0.62 for interaction).  It should also be noted that there was heterogeneity with respect to the protocol with spontaneous breathing test, the protocol with gradual decrease in assistance, and the ventilator-automated weaning protocol, with a different protocol used in each study. |

| Subgroup considerations |
| --- |
| We performed subgroup analyses of the heterogeneity of interventions described in Additional considerations. (1) a protocol of routine spontaneous breathing tests (Chaiwat2010, Ely1996, Fan2015, Krishnan2004, Namen2001, Navalesi2008), (2) a protocol of gradually decreasing ventilatory support (Kollef1997, Marelich2000 Marelich2000, Roh2008, Simeone2002), and (3) an automatic ventilator weaning protocol. In each subgroup, the effect estimates for in-hospital mortality were: 1) an increase of 18 per 1000 (95% confidence interval: decrease of 50 to increase of 96), 2) a decrease of 18 per 1000 (95% confidence interval: decrease of 78 to increase of 67), and 3) a decrease of 0 per 1000 (95% confidence interval: decrease of 129 to increase of 94). (95% CI: decrease of 129 to increase of 94), the estimated effect on ICU deaths was a decrease of 51 per 1000 (95% CI: decrease of 87 to decrease of 2) in (1), an increase of 38 per 1000 (95% CI: decrease of 56 to increase of 558) in (3), and the estimated effect on ventilator duration was a decrease of 21.15 hours in (1). The estimated effect on the length of ICU stay was a reduction of 21.15 hours in (1) (95% confidence interval: 44.1 hours reduction to 1.79 hours extension), a reduction of 42.6 hours in (2) (95% confidence interval: 85.78 hours reduction to 0.58 hours extension), and a reduction of 29.64 hours in (3) (95% confidence interval: 90.33 hours reduction to 31.06 hours extension). The estimated effect on the length of ICU stay was: (1) 18.96 hours shorter (95% CI: 48.45 hours shorter to 10.52 hours longer), (2) 17.7 hours shorter (95% CI: 32.27 hours shorter to 3.12 hours shorter), and (3) 13.82 hours shorter (95% CI: 67.5 hours shorter to 39.87 hours longer). The estimated effect on hospital stay was 1.91 days longer (95% CI: 5.79 days shorter to 9.61 days longer) for (1), 1.64 days shorter (95% CI: 3.82 days shorter to 0.54 days longer) for (2), and 1.7 days shorter (95% CI: 1.7 days shorter to 4.11 days longer) for (3). The estimated effect on tracheostomy was (1) a decrease of 41 patients per 1000 (95% CI: decrease of 74 patients to increase of 11 patients), (2) a decrease of 15 patients per 1000 (95% CI: decrease of 61 patients to increase of 94 patients), (3) a decrease of 39 patients per 1000 (95% CI: decrease of 113 patients to increase of 158 patients), and (4) a decrease of 23 patients per 1000 (95% CI: decrease of 23 patients per 1000). The estimated effect on reintubation was a decrease of 23 (95% confidence interval: decrease of 57 to increase of 37) per 1000 for (1), an increase of 27 (95% confidence interval: decrease of 23 to increase of 117) per 1000 for (2), and an increase of 17 (95% confidence interval: decrease of 70 to increase of 193) per 1000 for (3). However, the results were not consistent, and the clear superiority of the protocol method over other protocols was not clear from the results of this study. (See also additional considerations above.) |
| Implementation considerations |
| With regard to performing protocolized ventilator weaning, the ARDS Clinical Practice Guideline 2016, like this one, “Proposes to perform a protocolized ventilator weaning in adult patients with ARDS (GRADE 2D).” The ATS/ESICM/SCCM2017 did not mention it.  In creating a protocol, it is necessary to create a protocol that is appropriate for the workforce and staff knowledge at each facility. In particular, when a protocol is introduced to change the ventilator settings in a multidisciplinary team, it is important to ensure that the medical personnel are educated appropriately and that patients are closely monitored.  As an example of the protocol in Japan, the protocol for specific actions can be referred to [(https://www.mhlw.go.jp/file/06-Seisakujouhou-10800000-](http://www.mhlw.go.jp/file/06-Seisakujouhou-10800000-) Iseikyoku/0000112464.pdf ). It is common practice to extubate the patient after at least 30 minutes of observation (*) with ventilator settings of PEEP 5 cmH_2_O, PS 5 cmH_2_O.  *The healthcare provider should observe changes in circulatory status (e.g., increased pulse rate) and respiratory status (e.g., increased respiratory rate, appearance of effort-like breathing) to assess the risk of upper airway stenosis and reintubation after extubation. |

| Monitoring and evaluation |
| --- |
| It is necessary to collect more information on the implementation of additional evaluations (e.g., monitoring the duration of ventilation and ICU stay) as a clinical problem in implementing the recommendations. It is also necessary to monitor whether there are any other clinical problems through the use of questionnaires after the guideline is published. |
| Research priorities |

There are no studies in patients with ARDS, and RCTs in such patients are needed. Future meta-analyses should be conducted in populations similar to ARDS. In addition, separate meta-analyses of spontaneous breathing test protocols, protocols with a gradual decline in assistance, and protocols with automatic ventilator-assisted ventilation are desirable.

References

1)Blackwood B, Burns KE, Cardwell CR, et al. Protocolized versus non-protocolized weaning for reducing the duration of mechanical ventilation in critically ill adult patients. Cochrane Database Syst Rev. 11:CD006904. PMID: 25375085

**CQ28 Should high-frequency oscillatory ventilation (HFOV) be used for adult patients with ARDS?**

1. Search strategy

MEDLINE via PubMed （Search date: 2021/3/23）

| #1 | Respiratory Distress Syndrome, Adult [MH]  OR acute respiratory distress syndrome[tiab]  OR adult respiratory distress syndrome[tiab]  OR respiratory distress syndrome[tiab] OR ARDS[tiab] |
| --- | --- |
| #2 | Acute Lung Injury [MH] OR acute lung injury[tiab]  OR acute lung injuries[tiab] OR lung injury[tiab]  OR lung injuries[tiab] OR ALI [tiab] OR shock lung[tiab] |
| #3 | respiratory insufficiency[MH] OR respiratory insufficiency[tiab]  OR acute respiratory failure[tiab] OR respiratory depression[tiab]  OR ventilatory depression[tiab] |
| #4 | #1 OR #2 OR #3 |
| #5 | High frequency ventilation[MH]  OR High-Frequency Oscillation Ventilation[tiab]  OR High-Frequency Oscillation Ventilations[tiab]  OR High-Frequency Positive Pressure Ventilation[tiab]  OR High-Frequency Positive Pressure Ventilations[tiab]  OR HFO[tiab] OR HFOV[tiab] |
| #6 | Respiration, Artificial[MH] OR Artificial respiration[tiab]  OR Mechanical ventilation[tiab] OR Mechanical ventilations[tiab] |
| #7 | #5 AND #6 |
| #8 | Clinical trial[pt] OR trial[ti] OR randomized controlled trial[pt]  OR(controlled clinical trial[pt] OR randomized[tiab]) OR placebo[tiab]  OR clinical trials as topic[MH] OR randomly[tiab] |
| #9 | Animals[MH] NOT Humans[MH] |
| #10 | #8NOT#9 |
| #11 | #4 AND #7 AND #10 |

CENTRAL （Search date: 2021/3/23）

| #1 | MeSH descriptor: [Respiratory Distress Syndrome, Adult] explode all trees |
| --- | --- |
| #2 | ("acute respiratory distress syndrome"):ti,ab,kw |
| #3 | ("adult respiratory distress syndrome"):ti,ab,kw |
| #4 | ("respiratory distress syndrome"):ti,ab,kw |
| #5 | (ARDS):ti,ab,kw |
| #6 | {OR #1-#5} |
| #7 | MeSH descriptor: [Acute Lung Injury] explode all trees |
| #8 | ("acute lung injury"):ti,ab,kw |
| #9 | ("acute lung injuries"):ti,ab,kw |
| #10 | (ALI):ti,ab,kw |
| #11 | ("shock lung"):ti,ab,kw |
| #12 | (" lung injury "):ti,ab,kw |
| #13 | (" lung injuries "):ti,ab,kw |
| #14 | {OR #7-#13} |
| #15 | MeSH descriptor: [Respiratory Insufficiency] explode all trees |
| #16 | ("Respiratory Insufficiency"):ti,ab,kw |
| #17 | ("acute respiratory failure"):ti,ab,kw |
| #18 | ("respiratory depression"):ti,ab,kw |
| #19 | ("ventilatory depression"):ti,ab,kw |
| #20 | {OR #15-#20} |
| #21 | MeSH descriptor: [High-Frequency Ventilation] explode all trees 196 |
| #22 | ("high frequency ventilation"):ti,ab,kw |
| #23 | ("high frequency oscillatory ventilation"):ti,ab,kw |
| #24 | ("high frequency oscillation"):ti,ab,kw |
| #25 | ("high frequency positive pressure ventilation"):ti,ab,kw |
| #26 | (HFOV):ti,ab,kw |
| #27 | (HFO):ti,ab,kw |
| #28 | {OR #21-#27} |
| #29 | MeSH descriptor: [Respiration, Artificial] explode all trees |
| #30 | ("artificial respiration"):ti,ab,kw |
| #31 | ("mechanical ventilation"):ti,ab,kw |
| #32 | ("mechanical ventilatory support"):ti,ab,kw |
| #33 | ("mechanical ventilations"):ti,ab,kw |
| #34 | ({OR #29-#33} |
| #35 | #6 OR #14 OR #20 |
| #36 | #28 AND #34 |
| #37 | #35 AND #36 |

Igaku-Chuo-Zasshi （Search date: 2021/3/23）

| #1 | 呼吸窮迫症候群-急性/TH |
| --- | --- |
| #2 | 呼吸促迫症候群/AL |
| #3 | 呼吸窮迫症候群/AL |
| #4 | 急性呼吸窮迫症候群/AL |
| #5 | 成人呼吸窮迫症候群/AL |
| #6 | acute respiratory distress syndrome/AL |
| #7 | ARDS/AL |
| #8 | PT=会議録除く |
| #9 | #1 OR #2 OR #3 OR #4 OR #5 OR #6 OR #7 AND #8 |
| #10 | 急性肺損傷/TH |
| #11 | 急性肺損傷/AL |
| #12 | 急性肺障害/AL |
| #13 | acute lung injuries/AL |
| #14 | acute lung injury/AL |
| #15 | PT=会議録除く |
| #16 | #10 OR #11 OR #12 OR #13 OR #14 AND #15 |
| #17 | 高頻度換気/TH |
| #18 | 高頻度振動換気/AL |
| #19 | high frequency oscillatory ventilation |
| #20 | HFOV/AL |
| #21 | PT=会議録除く |
| #22 | #17 OR #18 OR #19 OR #20 AND #21 |
| #23 | RD=ランダム化比較試験 |
| #24 | CK=ヒト |
| #25 | CK=動物 |
| #26 | CK=成人(19～44),中年(45～64),高齢者(65～) |
| #27 | #24 AND #26 NOR #25 |
| #28 | #9 OR #16 OR #22 AND #23 AND #27 |

1. Flow diagram

**Identification**

485 records identified through database searching

485 records identified through database searching

Medline via PubMed (n=265)

CENTRAL (n=190)

Igaku-Chuo-Zasshi (n=30)

0 additional records identified through other sources

**Included**

5 Studies included in quantitative synthesis (meta-analysis)

11 Full-text articles excluded, with reasons:

・Wrong study design (n=0)

・Wrong population (n=3)

・Wrong intervention (n=8)

469 records excluded

5 Studies included in qualitative synthesis

16 Full-text articles assessed for eligibility (Attempt to order full text)

**Eligibility**

**Screening**

1. Risk of bias

Short-term mortality Inhospital mortality

Length of ICU stay Ventilator-free days

Barotrauma Refractory hypoxemia

1. Forest plot

Short-term mortality


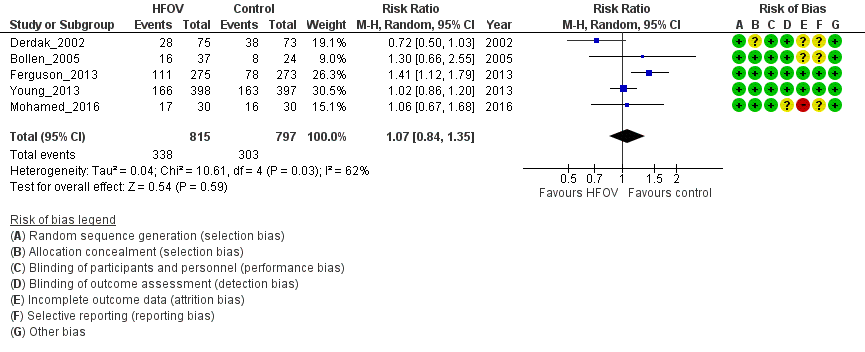


Inhospital


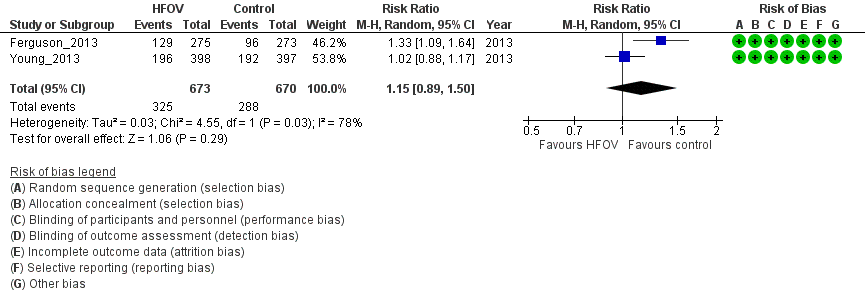


Length of ICU stay


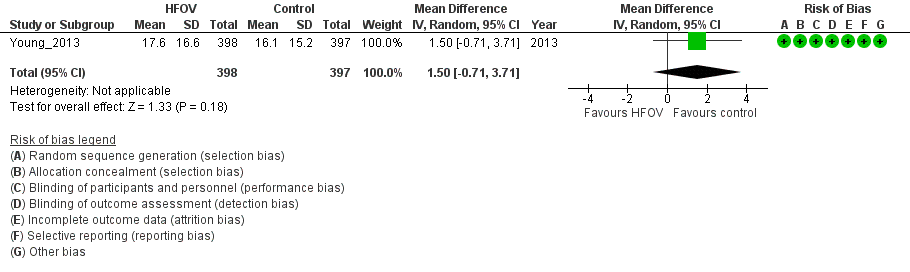


Ventilator-free days


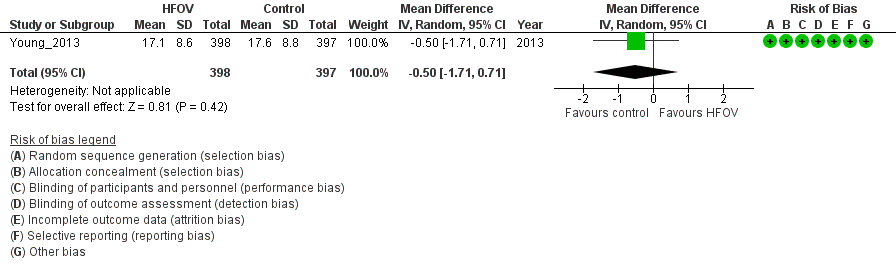


Barotrauma


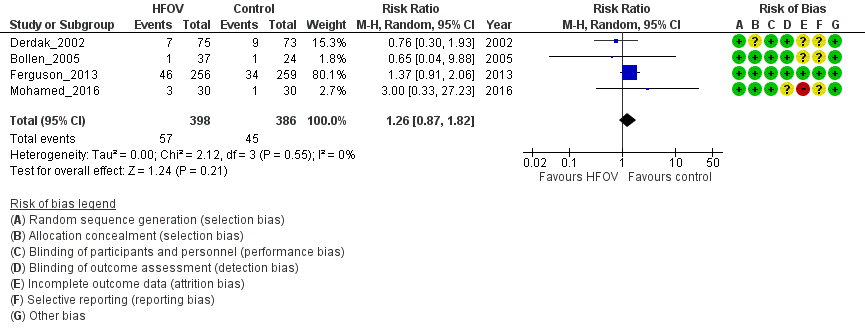


Refractory hypoxemia


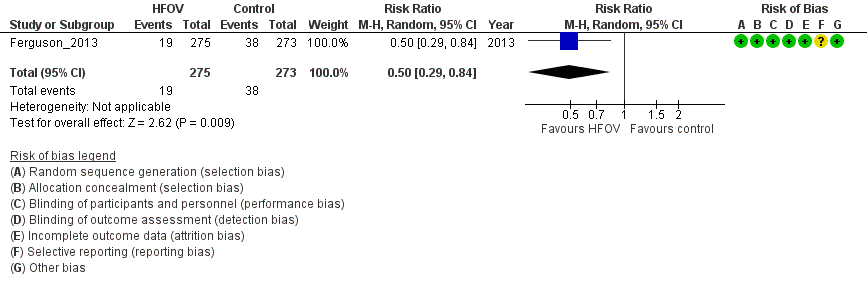


1. Evidence profile

| **Certainty assessment** | | | | | | | **№ of patients** | | **Effect** | | **Certainty** | **Importance** |
| --- | --- | --- | --- | --- | --- | --- | --- | --- | --- | --- | --- | --- |
| **№ of studies** | **Study design** | **Risk of bias** | **Inconsistency** | **Indirectness** | **Imprecision** | **Other considerations** | **HFOV** | **control** | **Relative (95% CI)** | **Absolute (95% CI)** |  |  |
| **Short-term mortality*** | | | | | | | | | | | | |
| 5 | Randomized trials | not serious | serious ^a^ | not serious | serious ^b^ | none | 338/815 (41.5%) | 303/797 (38.0%) | **RR 1.07**  (0.84 to 1.35) | **27 fewer per**  **1,000**  (61 fewer  to 133 more) | ⨁⨁◯◯  Low | CRITICAL |
| **In-hospital mortality**** | | | | | | | | | | | | |
| 2 | Randomized trials | not serious | serious | not serious | serious ^d^ | none | 325/673 (48.3%) | 288/670 (43.0%) | **RR 1.15**  (0.89 to 1.50) | **64 fewer per**  **1000**  (47 fewer  to 215 more) | ⨁⨁◯◯  Low | CRITICAL |
| **ICU length of stay** | | | | | | | | | | | | |
| 1 | Randomized trials | not serious | not serious | not serious | serious ^e^ | none | 398 | 397 | - | **MD** **1.5**  **days longer**  (0.71 shorter to  3.71 longer) | ⨁⨁⨁◯  Moderate | CRITICAL |
| **Ventilation free day (VFD)** | | | | | | | | | | | | |
| 1 | Randomized trials | not serious | not serious | not serious | not serious | none | 398 | 397 | - | **MD 0.5 days**  **shorter**  (1.71 shorter to  0.71 longer) | ⨁⨁⨁⨁  High | CRITICAL |
| **Barotrauma** | | | | | | | | | | | | |
| 4 | Randomized trials | not serious | not serious | not serious | serious ^f^ | none | 57/398 (14.3%) | 45/386 (11.7%) | **RR 1.26**  (0.87 to 1.82) | **30 more per**  **1000**  (15 fewer  to 96 more) | ⨁⨁⨁◯  Moderate | CRITICAL |
| **Refractory hypoxemia** | | | | | | | | | | | | |
| 1 | Randomized trials | not serious | not serious | not serious | serious ^g^ | none | 19/275 (6.9%) | 38/273 (13.9%) | **RR 0.50**  (0.29 to 0.84) | **70 fewer per**  **1,000**  (99 fewer  to 22 more) | ⨁⨁⨁◯  Moderate | IMPORTANT |

**CI:** confidence interval; **MD:** mean difference; **RR:** risk ratio

* Includes 28-day deaths, 30-day deaths, and ICU deaths

Explanations

a. In addition to the I2=62%, the variability of the effects included in the systematic review was visually judged by the forest plot, which is judged to be “serious” and downgraded by one level.

b. The total sample size of 1502 and the number of events of 608 met the optimal information content (OIS); however, the 95% confidence interval was wide and the confidence interval includes ^“^substantial harm” and “no effect”.

c. In addition to the I2=78%, the variability of the effects included in the systematic review was visually judged using the forest plot, which is judged to be “ serious” and downgraded by one level.

d. The total sample size was 1343 and the number of events was 613, which met the optimal information size (OIS); however, the 95% confidence interval was wide and the confidence interval included ^“^substantial harm” and ^“^no effect.”

e. The total sample size of 795 met the OIS; however, the 95% confidence interval was wide and exceeded the minimum important difference (MID), and included “substantial harm” and “no effect.” Therefore, it is downgraded by one level.

f. The total sample size was 704 and the number of events was 100, which did not meet the OIS, and the 95% confidence interval was wide enough to include ^”^substantial harm” and “no effect.”

g. The total sample size was 548 and the number of events was 57, which did not meet the OIS.

1. Evidence-to-Decision table

| question | |
| --- | --- |
| **CQ28: Should high-frequency oscillatory ventilation (HFOV) be used for adult patients with ARDS?** | |
| GROUP : | 1) Adult patients with ARDS requiring ventilator management  2) Patients with acute respiratory failure of moderate severity or greater, excluding cardiogenic pulmonary edema |
| INTERVENTIONS: | Ventilatory management by HFOV ( >12 h/day) |
| comparison and contrast: | Conventional ventilator management |
| MAIN OUTCOMES: | Short-term mortality, In-hospital mortality, intensive care unit (ICU) length of stay, ventilator-free days (VFD), barotrauma |
| SETTING: | The emergency room or ICU |
| PERSPECTIVES : | Personal |
| BACKGROUND : | Although there are various methods of ventilatory management in adult patients with ARDS, it is important to prevent ventilatory-associated lung injury, which can prolong the duration of ventilatory care and increase mortality. HFOV is a mode of ventilation that allows lung recruitment by limiting the amount of ventilation per cycle and is considered a type of lung protective ventilation. However, it is not a common method of ventilatory management for adult patients with ARDS, and its efficacy and safety need to be examined. |
| CONFLICT OF INTEREST: | None |

# assessment

| Problem Is the problem a priority? | | |
| --- | --- | --- |
| judgment | research evidence | remarks |
| ○ No  ○ Probably no  ● Probably yes  ○ Yes  ○ Varies  ○ Do not know | Previously, HFOV was considered to have the potential to reduce ventilator-induced lung injury when used for ARDS and other conditions. However, two large randomized controlled trials (RCTs) published in 2013, OSCAR^1)^ and OSCILLATE^2)^, showed no improvement in life expectancy compared to conventional lung protective ventilation, and since then, it has not been commonly used in adult patients with ARDS. However, some problems have been pointed out in the protocol of the OSCILLATE trial^3)^, and the results of this RCT may not be enough to say that HFOV should not be used. Therefore, we judged that it was necessary to confirm the safety and efficacy of HFOV again by conducting a systematic review with evidence published after the previous version of the guideline. Therefore, the priority for CQ planning is probably high. |  |
| Desirable effects How substantial are the desirable anticipated effects? | | |
| judgment | research evidence | note |
| ●Trivial  ○Small  ○ Moderate  ○Large  ○Varies  ○Do not know | The results of the systematic review showed that five RCTs consistent with the patient, intervention, comparison, and outcome process were conducted, and a meta-analysis was performed using these studies. Mortality, VFD, ICU length of stay, and barotrauma, which were initially assumed to be outcomes in the direction of benefit, were in the direction of harm. Since there were no other serious to beneficial outcomes, we considered the important outcome of refractory hypoxemia as a beneficial outcome. In the control group, 38 of 273 patients showed a reduction in hypoxemia, and in the intervention group, 19 of 275 patients showed a reduction. The desired effect of the intervention was judged to be “trivial.” |  |
| Undesirable effects How substantial are the undesirable anticipated effects? | | |
| judgment | research evidence | note |
| ○Large  ○ Moderate  ●Small  ○Trivial  ○Varies.  ○ Do not know | As outcomes of harm, the effect estimate for mortality (four RCTs: N=1612) was an increase in risk by 27 people/1000 (95% CI: 61 decrease to 133 increase), and the effect estimate for in-hospital mortality (2 RCTs: N=1343) was an increase in risk of 64 deaths/1000 (95% CI: decrease of 47 to increase of 215) and an increase in ICU length of stay (1 RCT: N=795) with an average of 1.5 days longer (95% CI: 0.71 days shorter to 3.71 days longer). The effect estimate for VFD (1 RCT: N=795) was an average of 0.5 days shorter (95% CI: 1.71 days shorter to 0.71 days longer), and the effect estimate for barotrauma (4 RCTs: N=784) was a risk difference of 30 more people/1000 (95% CI: 15 fewer people to 96 more people) for the “intervention” compared to the “control.” Based on the above, the expected undesirable effect was judged to be “small.” |  |
| Certainty of evidence What is the overall certainty of the evidence of effects? | | |
| judgment | research evidence | note |
| ○Very low  ○Low  ○Moderate  ●High  ○No included studies | **The relative importance or value of the key outcomes of interest:**   \|  \| Outcome \| Relative importance \| Certainty of evidence \| \| --- \| --- \| --- \| --- \| \|  \| Short-term mortality \| Serious \| ⨁⨁◯◯  Low \| \|  \| In-hospital mortality \| Serious \| ⨁⨁◯◯ Low \| \|  \| ICU length of stay \| Serious \| ⨁⨁⨁◯ Moderate \| \|  \| VFD \| Serious \| ⨁⨁⨁⨁ High \| \|  \| Barotrauma \| Serious \| ⨁⨁⨁◯ Moderate \|   **Overall evidence certainty**:  The direction of the effects for the benefit and harm outcomes were consistent, and the certainty of the evidence across the outcomes was judged to be “High,” adopting the highest certainty of the evidence. |  |
| Values Is there important uncertainty about or variability in how much people value the main outcomes? | | |
| judgment | research evidence | note |
| ○Important uncertainty or variability  ○Possibly important uncertainty or variability  ●Probably no important uncertainty or variability  ○No important uncertainty or variability | There are no data on the values for outcomes in HFOV-based management, but the value for death is generally high, and the variability is low. |  |
| Balance of effects Does the balance between desirable and undesirable effects favor the intervention or the comparison? | | |
| judgment | research evidence | remarks |
| ○Favors the comparison  ●Probably favors the comparison  ○Does not favor either the intervention or the comparison  ○Probably favors the intervention  ○Favors the intervention  ○Varies  ○Do not know | **Summary of results**   \| Outcome \| Normal ventilation (control) \| HFOV (intervention) \| Absolute difference  (95% CI) \| Relative effect  (95% CI) \| \| --- \| --- \| --- \| --- \| --- \| \| Short-term mortality \| 303/797 \| 338/815 \| 27 more /1000  (61 fewer to 133 more) \| 1.07  (0.84-1.35) \| \| In-hospital  mortality \| 288/670 \| 325/673 \| 64 fewer /1000  (47 fewer to 215 more) \| 1.15  (0.89-1.50) \| \| ICU length of stay \| -. \| -. \| MD 1.5 days longer  (0.71 days shorter to 3.71 days longer) \| -. \| \| VFD \| -. \| -. \| MD 0.5 days shorter  (1.71 days shorter to 0.71 days longer) \| -. \| \| Barotrauma \| 45/386 \| 57/398 \| 30 more/1000  (15 fewer to 96 more) \| 1.26  (0.87-1.82) \|  From the above, whether the desired effect of the intervention was greater than the undesired effect was judged to be “probably in favor of the control.” |  |
| Acceptability Is the intervention acceptable to key stakeholders? | | |
| judgment | research evidence | note |
| ○ No  ● Probably no  ○ Probably yes  ○ Yes  ○ Varies  ○ Do not know | Although there is no evidence that has been used to evaluate acceptability, the use of HFOV in adults is likely to require the introduction of a dedicated ventilator. This is expected to be difficult for some facilities to achieve. In addition, it will take much effort to educate medical staff that have no experience in using HFOV on how to use it properly. Therefore, we judged as “Maybe no”. |  |
| Feasibility Is the intervention feasible to implement? | | |
| judgment | research evidence | remarks |
| ○ No  ○ Probably no  ○ Probably yes  ○ Yes  ● Varies  ○ Do not know | Although there is no evidence used to evaluate feasibility, HFOV is one mode of mechanical ventilation, and its adaptation to patients is feasible. On the other hand, the feasibility of HFOV was judged to be " It is hard to say " because it requires a special ventilator and experienced staff to use it. |  |

**Summary of Judgment**

|  | **JUDGMENT** | | | | | | |
| --- | --- | --- | --- | --- | --- | --- | --- |
| **PROBLEM** | No | Probably no | Probably yes | Yes |  | Varies | Do not know |
| **DESIRABLE EFFECTS** | Trivial | Small | Moderate | Large |  | Varies | Do not know |
| **UNDESIRABLE EFFECTS** | Large | Moderate | Small | Trivial |  | Varies | Do not know |
| **CERTAINTY OF EVIDENCE** | Very low | Low | Moderate | High |  |  | No included studies |
| **VALUES** | Important uncertainty or variability | Possibly important uncertainty or variability | Probably no important uncertainty or variability | No important uncertainty or variability |  |  |  |
| **BALANCE OF EFFECTS** | Favors the comparison | Probably favors the comparison | Does not favor either the intervention or the comparison | Probably favors the intervention | Favors the intervention | Varies | Do not know |
| **ACCEPTABILITY** | No | Probably no | Probably yes | Yes |  | Varies | Do not know |
| **FEASIBILITY** | No | Probably no | Probably yes | Yes |  | Varies | Do not know |

**Type of Recommendation**

| Strong recommendation against the intervention | Conditional recommendation against the intervention | Conditional recommendation for either the intervention or the comparison | Conditional recommendation for the intervention | Strong recommendation for the intervention |
| --- | --- | --- | --- | --- |
| ○ | ● | ○ | ○ | ○ |

# Conclusion

| Recommendation |
| --- |
| **Conditionally recommend that HFOV not be used for ventilator management of adult patients with moderate to severe ARDS (conditional recommendation/evidence of high certainty: GRADE 2A).**  **Supplementary item:**  **This recommendation is for HFOV in general adult patients with moderate to severe ARDS**  **(P/F ratio <200).** |
| Justification |

Question： Should high-frequency oscillatory ventilation (HFOV) be used for adult patients with ARDS?

Patient： Adult ARDS patients over 16 years of age who require ventilatory management with HFOV

Intervention： Ventilatory management with HFOV (>12 h/day)

Control： Conventional ventilator management

Outcome： Short-term mortality, I\in-hospital mortality, ICU length of stay, VFD, barotrauma

Summary of evidence： The results of the systematic review showed that ventilatory management with HFOV reduced refractory hypoxemia, defined as an important outcome, from 38 of 273 patients to 19 of 275 patients. The desired effect was therefore judged to be a little.

On the other hand, in terms of harm outcomes, short-term mortality increased by 27 per 1000 (five RCTs: N=1612 95%CI: 61 less to 133 more), in-hospital mortality increased by 27 per 1000 (2 RCTs: N=1343 95%CI: 47 less to 215 more), ICU length of stay increased by 1.5 days (1 RCT: N=795, 95% CI 0.71 days shorter to 3.71 days longer), VFD was reduced by 0.5 days (1 RCT: N=795 95% CI: 1.71 days shorter to 0.71 days longer), barotrauma increased by 30 per 1000 (4 RCTs: N=784 95%CI: 15 less to 96 more). The undesirable effect was judged to be low.

As a result, the balance of effects was judged to be probably more harmful.

Certainty of evidence： The direction of the outcome in desirable and undesirable effects was consistent, and the highest certainty of evidence among all outcomes was adopted and judged to be high.

Values, balance of effects, acceptance, and feasibility judgments： HFOV for adults is likely to require a dedicated ventilator and skilled staff. Judging from the overall balance of equipment, human cost, and effectiveness, there is little advantage in introducing a new ventilator to a facility that is not proficient in its use.

Panel meeting：In the pre-vote, by the modified Delphi method, "Propose a conditional recommendation not to use high frequency oscillatory ventilation (HFOV) for the ventilator management in adult patients with moderate to severe ARDS (conditional weak recommendation/high certainty evidence: GRADE 2A)" had a median score of 8.0 and a disagreement index 0.2920. There was some discussion at the panel meeting about the need for a supplemental statement " It does not preclude the use in more critically ill pediatric patients or some adult ARDS patients, rescue therapy, or hemostatic use for pulmonary hemorrhage in institutions proficient in its use," but the panel concluded that this statement was not necessary. The final recommendation was decided without a re-vote and agreed with the results of the pre-vote.

Additional considerations： The inclusion criteria for the five RCTs used in this study were patients with moderate to severe ARDS with a P/F ratio <200. Although no studies were found for patients with mild ARDS, it is not recommended for patients with mild ARDS, given the potential for increased barotrauma due to the high mode of mean airway pressure.

| Subgroup considerations |
| --- |
| Among the studies recruited, Mohamed 2016 was limited to patients with ARDS associated with burns. Therefore, as a sensitivity analysis, we performed a meta-analysis excluding Mohamed 2016 (outcomes were short-term mortality and barotrauma). There were no significant changes in direction, including point estimates, for short-term mortality (increase of 26 patients/1000 [decrease of 75 to increase of 153]) and barotrauma (increase of 28 patients/1000 [decrease of 19 to increase of 96]). |
| Implementation considerations |
| With regard to the use of HFOV for ARDS, the ARDS Clinical Practice Guidelines 2016 did not recommend its use (GRADE2C), and the ARDS guidelines of the ATS/ESICM/SCCM 2017 strongly recommended that it should not be routinely used in patients with moderate to severe ARDS. Considering the above and the results of the present systematic review, the use of HFOV is basically not recommended.  However, the individual patient data meta-analysis by Meade et al. in 20164) suggested an improved survival in the P/F ratio <100 group, thus the use of HFOV for rescue purposes or in a limited number of patients should be further investigated. In HFOV, it is not possible to evaluate ventilation by minute ventilation, ETCO2, or breath sounds, and thus evaluation by transcutaneous carbon dioxide monitoring should be considered.  It may be helpful to refer to the "High Frequency Oscillatory Ventilation (HFOV) Protocol for Adult Patients" published by the Japanese Society of Respiratory Therapy in 2015. |

| Monitoring and evaluation |
| --- |
| In implementing the recommendations, it is necessary to collect more information on how often HFOV is actually used and whether it can be used as a clinical problem. In addition, it is necessary to monitor whether there are any other clinical problems through questionnaires and other means after the guidelines are published. |
| Research priorities |
| In this meta-analysis, the point estimates for all outcomes were in the direction that HFOV use was undesirable, suggesting that HFOV is unlikely to be effective for adult patients with common moderate-to-severe ARDS. On the other hand, Derdak2002 ^5)^ (a study of alternating prone positioning and supine HFOV), Mentzelopoulus2007 ^6)^ (use of HFOV during recruitment maneuver), and Samransamruajkit 2016 ^7)^ (HFOV in pediatric patients) suggested the benefit of HFOV. It may be necessary to evaluate the conditions under which HFOV may be effective (target patients, duration of use, method of use, etc.), including the meta-analysis by Meade et al.4) described in the section on implementation considerations. |

References

1) Ferguson ND, Cook DJ, Guyatt GH, et al. High-frequency oscillation in early acute respiratory distress syndrome. N Engl J Med. 2013;368:795-805. PMID: 23339639.

2) Young D, Lamb S, Shah S, et al. High-frequency oscillation for acute respiratory distress syndrome.

N Engl J Med. 2013;368:806-13. PMID:23339638.

3) Kacmarek RM, Villar J. Management of refractory hypoxemia in ARDS. Minerva Anestesiol. 2013;79:1173-9. PMID: 23857446.

4) Meade MO, Young D, Hanna S, et al. Severity of hypoxemia and effect of high-frequency oscillatory ventilation in acute respiratory distress syndrome. Am J Respir Crit Care Med. 2017;196:727–733.

PMID: 28245137.

5) [Derdak](https://pubmed.ncbi.nlm.nih.gov/?term=Derdak%2BS&cauthor_id=12231488) S, [Mehta](https://pubmed.ncbi.nlm.nih.gov/?term=Mehta%2BS&cauthor_id=12231488) S, [Stewart](https://pubmed.ncbi.nlm.nih.gov/?term=Stewart%2BTE&cauthor_id=12231488) T, et al. High-frequency oscillatory ventilation for acute respiratory distress syndrome in adults: a randomized, controlled trial. Am J Respir Crit Care Med. 2002;166(6):801-8.

PMID: 12231488.

6) [Mentzelopoulos](https://pubmed.ncbi.nlm.nih.gov/?term=Mentzelopoulos%2BSD&cauthor_id=21885390) S, [Malachias](https://pubmed.ncbi.nlm.nih.gov/?term=Malachias%2BS&cauthor_id=21885390) S, [Zintzaras](https://pubmed.ncbi.nlm.nih.gov/?term=Zintzaras%2BE&cauthor_id=21885390) E, et al. Intermittent recruitment with high-frequency oscillation/tracheal gas insufflation in acute respiratory distress syndrome. Eur Respir J. 2012;39(3):635-47. PMID: 21885390.

7) [Samransamruajkit](https://pubmed.ncbi.nlm.nih.gov/?term=Samransamruajkit%2BR&cauthor_id=27076706) R, [Rassameehirun](https://pubmed.ncbi.nlm.nih.gov/?term=Rassameehirun%2BC&cauthor_id=27076706) C, [Pongsanon](https://pubmed.ncbi.nlm.nih.gov/?term=Pongsanon%2BK&cauthor_id=27076706) K, et al. A comparison of clinical efficacy between high frequency oscillatory ventilation and conventional ventilation with lung volume recruitment in pediatric acute respiratory distress syndrome: a randomized controlled trial. Indian J Crit Care Med. 2016;20(2):72-7. PMID: 27076706

**CQ29 Should driving pressure be used as an index when implementing mechanical ventilation in adult patients with ARDS?**

1. Search strategy

MEDLINE via PubMed （Search date: 2020/7/14）

| #1 | "Respiratory Distress Syndrome, Adult"[mh] OR "Acute Lung Injury"[mh] OR "Respiratory Insufficiency"[mh] OR "Acute Chest Syndrome"[mh] OR "shock lung"[tiab] OR "respiratory distress syndrome"[tiab] OR "lung injur*"[tiab] OR ARDS[tiab] OR ALI[tiab] OR "respiratory insufficiency"[tiab] OR "respiratory failure"[tiab] OR "respiratory depression"[tiab] OR "ventilatory depression"[tiab] OR "acute chest syndrome"[tiab] |
| --- | --- |
| #2 | "driving pressure*"[tiab] OR "inspiratory airway pressure*"[tiab] OR "mechanical power"[tiab] OR "deltaP"[tiab] OR "Lung Compliance"[mh] OR "Tidal Volume"[mh] OR "lung compliance"[tiab] OR "tidal volume*"[tiab] OR "positive end-expiratory pressure*"[tiab] OR PEEP[tiab] |
| #3 | "randomized controlled trial"[pt] OR "controlled clinical trial"[pt] OR randomized[tiab] OR placebo[tiab] OR "drug therapy"[sh] OR randomly[tiab] OR trial[tiab] OR groups[tiab] |
| #4 | "Observational study"[pt] OR "Comparative study"[pt] OR "Epidemiologic studies"[mh] OR "Observational stud*"[tiab] OR "cohort stud*"[tiab] OR "concurrent study"[tiab] OR "cohort analys*"[tiab] OR "incidence stud*"[tiab] OR "epidemiological stud*"[tiab] OR "epidemiologic stud*"[tiab] |
| #5 | #3 OR #4 |
| #6 | Animals[mh] NOT Humans[mh] |
| #7 | #5 NOT #6 |
| #8 | #1 AND #2 AND #7 |

CENTRAL （Search date: 2020/7/14）

| #1 | [mh "Respiratory Distress Syndrome, Adult"] OR [mh "Acute Lung Injury"] OR [mh "Respiratory Insufficiency"] OR [mh "Acute Chest Syndrome"] OR "shock lung":ti,ab OR "respiratory distress syndrome":ti,ab OR "lung injury":ti,ab OR ARDS:ti,ab OR ALI:ti,ab OR "respiratory insufficiency":ti,ab OR "respiratory failure":ti,ab OR "respiratory depression":ti,ab OR "ventilatory depression":ti,ab OR "acute chest syndrome":ti,ab |
| --- | --- |
| #2 | "driving pressure":ti,ab OR "driving pressures":ti,ab OR "inspiratory airway pressure":ti,ab OR "mechanical power":ti,ab OR deltaP:ti,ab OR [mh "Lung Compliance"] OR [mh "Tidal Volume"] OR "lung compliance":ti,ab OR "tidal volume":ti,ab OR "positive end-expiratory pressure*":ti,ab OR PEEP:ti,ab |
| #3 | #1 AND #2 |
| #4 | [mh Animals] NOT [mh Humans] |
| #5 | #3 NOT #4 |

Igaku-Chuo-Zasshi　（Search date: 2020/7/14）

| #1 | 呼吸窮迫症候群-急性/TH or 急性呼吸窮迫症候群/AL or ARDS/AL or "acute respiratory distress syndrome"/AL or 急性肺損傷/TH or 急性肺損傷/AL or "acute lung injury"/AL or 急性胸部症候群/TH or 急性胸部症候群/AL or 呼吸障害/TH or 呼吸障害/AL |
| --- | --- |
| #2 | "driving pressure"/AL or ドライビングプレッシャー/AL or メカニカルパワー/AL or "mechanical power"/AL or deltaP/AL or デルタP/AL or 圧/AL or 陽圧呼吸/TH or PEEP/AL or 肺コンプライアンス/TH or 肺コンプライアンス/TA or 一回換気量/TH or 一回換気量/TA |
| #3 | #2 NOT (血圧/TH or 血圧/AL) |
| #4 | ランダム化比較試験/TH or 準ランダム化比較試験/TH or ランダム化/AL or 無作為化/AL or 比較試験/AL or 臨床試験/AL or プラセボ/AL or 対照/AL or コントロール/AL or 臨床研究/AL or 観察研究/TH or 観察研究/AL or 臨床研究・疫学研究/TH or 臨床研究・疫学研究/AL or コホート研究/TH or コホート研究/AL or 比較研究/AL |
| #5 | #1 AND #3 AND #4 |
| #6 | (#5) and (PT=会議録除く) |

1. Flow diagram

**Identification**

13 Studies included in qualitative synthesis

84 Full-text articles assessed for eligibility

4015 records after duplicates removed

5530 records identified through database searching

5530 records identified through database searching

Medline via PubMed (n=3147)

CENTRAL (n=1417)

Igaku-Chuo-Zasshi (n=966)

0 additional records identified through other sources

7 Full-text articles excluded, with reasons:

・couldn't get any data (n=5)

・No intended outcome (n=2)

6 Studies included in quantitative synthesis (meta-analysis)

72 Full-text articles excluded, with reasons:

・Wrong study design (n=19)

・Wrong population (n=6)

・Wrong intervention (n=44)

・duplicates (n=3)

Duplicates

n=1515

3931 records excluded

**Included**

**Eligibility**

**Screening**

1. Risk of bias

Not applicable

1. Forest plot

Not applicable

1. Evidence profile

Not applicable

1. Evidence-to-Decision table

| QUESTION | |
| --- | --- |
| **CQ29：Should driving pressure be used as an index when implementing mechanical ventilation in adult patients with ARDS?** | |
| **GROUP:** | Adult patients with ARDS that require ventilatory management |
| **INTERVENTIONS:** | Ventilatory management with limited driving pressure (driving pressure ≤15 cmH_2_O) |
| **COMPARISON AND CONTRAST:** | Ventilatory management without limiting the driving pressure (driving pressure >15 cmH_2_O) |
| **MAIN OUTCOMES:** | Short-term mortality (adopt the longest term within 90 days), ventilator-free days, barotrauma/ventilator-induced lung injury (VILI) |
| **SETTING:** | The emergency room or intensive care unit |
| **PERSPECTIVES:** | Individual |
| **BACKGROUND:** | Since the ARDS guidelines 2016, there have been reports that driving pressure was associated with survival in the ventilatory management of ARDS patients. Limiting driving pressure is expected to reduce ventilatory-associated lung injury (VALI). On the other hand, it may also increase harms such as hypercapnia and acid-base imbalance. Therefore, it is clinically important to evaluate the usefulness of driving pressures as an index for ventilation in patients with ARDS. |
| **CONFLICT OF INTEREST:** | None |

# Assessment

| Problem Is the problem a priority? | | |
| --- | --- | --- |
| Judgment | Research Evidence | Remarks |
| ○ No  ○ Probably no  ○ Probably yes  ● Yes  ○ Varies  ○ Do not know | Since the ARDS guidelines 2016, there have been reports that driving pressure was associated with survival in the ventilatory management of ARDS patients. Limiting driving pressure is expected to reduce ventilatory-associated lung injury (VALI). On the other hand, it may also increase harms such as hypercapnia and acid-base imbalance. Therefore, it is clinically important to evaluate the usefulness of driving pressures as an index for ventilation in patients with ARDS.. Hence, this issue is of high priority. |  |
| Desirable effects How substantial are the desirable anticipated effects? | | |
| Judgment | Research Evidence | Remarks |
| ○Trivial  ○Small  ○ Moderate  ○Large  ○Varies  ●Do not know | Integrated into the certainty of the evidence. |  |
| Undesirable effects How substantial are the undesirable anticipated effects? | | |
| Judgment | Research Evidence | Remarks |
| ○Large  ○ Moderate  ○Small  ○Trivial  ○Varies.  ● Do not know | Integrated into the certainty of the evidence. |  |
| Certainty of evidence What is the overall certainty of the evidence of effects? | | |
| Judgment | Research Evidence | Remarks |
| ○Very low  ○Low  ○Moderate  ○High  ●No included studies | Lung protective ventilation strategies and the open lung approach were reported to be associated with reduced ventilator-related lung injury, improved oxygenation, and improved survival. However, ventilation strategies using target tidal volume ventilation for predicted body weight (PBW) in ARDSnet do not account for differences in alveolar space unavailable for ventilation depending on the severity of ARDS.  A decrease in alveolar area available for ventilation may appear as a decrease in lung compliance. If two lungs are the same size, but one lung has low compliance and the other has high compliance, the lung with low compliance may generate more mechanical stress.  Therefore, stratifying patients using a measure of the "functional" size of the lung, which is the value of tidal volume divided by the compliance, may better predict the outcome of ARDS patients than tidal volume alone. This ratio is called the driving pressure (ΔP = VT/CRS) and can be calculated at the bedside as plateau pressure minus end-expiratory positive pressure (ΔP = Pplat - PEEP) in patients who are not spontaneously breathing.  With the above as a background, achieving a low driving pressure is expected to reduce lung overstretching/overexpansion and reduce ventilation-related lung injury (VALI). It is also expected to shorten the duration of ventilation and reduce mortality. On the other hand, low driving pressure may cause harm such as increased respiratory rate, atelectasis, hypercapnia and respiratory acidosis. Ventilation strategies using driving pressure as an index may improve the safety of ventilation strategies in patients with ARDS, but we were unable to find any relevant evidence in our search for this CQ. Therefore, the balance of benefit and harm is not yet known, and a statement of confidence in the evidence could not be made. |  |
| Values Is there important uncertainty about or variability in how much people value the main outcomes? | | |
| Judgment | Research Evidence | Remarks |
| ○Important uncertainty or variability  ○Possibly important uncertainty or variability  ●Probably no important uncertainty or variability  ○No important uncertainty or variability | Probably no important uncertainty or variability. |  |
| Balance of effects Does the balance between desirable and undesirable effects favor the intervention or the comparison? | | |
| Judgment | Research Evidence | Remarks |
| ○Favors the comparison  ○Probably favors the comparison  ○Does not favor either the intervention or the comparison  ○Probably favors the intervention  ○Favors the intervention  ○Varies  ●Do not know | No relevant studies. |  |
| Acceptability Is the intervention acceptable to key stakeholders? | | |
| Judgment | Research Evidence | Remarks |
| ○ No  ○ Probably no  ● Probably yes  ○ Yes  ○ Varies  ○ Do not know | Although there was no evidence that could be used to evaluate feasibility, it is assumed that ventilatory management using driving pressure is already being used in daily practice. However, if low driving pressure causes harm, i.e., increased respiratory rate, atelectasis, hypercapnia, respiratory acidosis or asynchrony, it is necessary to balance the benefit and harm and decide whether to use driving pressure as an index or not. |  |
| Feasibility Is the intervention feasible to implement? | | |
| Judgment | Research Evidence | Remarks |
| ○ No  ○ Probably no  ● Probably yes  ○ Yes  ○ Varies  ○ Do not know | Although there was no evidence available for review, it is likely that ventilatory management with awareness of driving pressure is already being used in daily practice. However, if low driving pressure causes harm, i.e., increased respiratory rate, atelectasis, hyper carbon dioxideemia, respiratory acidosis, or asynchrony, it is necessary to balance the benefits and decide whether to use driving pressure as an index or not. |  |

# Summary of Judgment

|  | **JUDGMENT** | | | | | | |
| --- | --- | --- | --- | --- | --- | --- | --- |
| **PROBLEM** | No | Probably no | Probably yes | Yes |  | Varies | Do not know |
| **DESIRABLE EFFECTS** | Trivial | Small | Moderate | Large |  | Varies | Do not know |
| **UNDESIRABLE EFFECTS** | Large | Moderate | Small | Trivial |  | Varies | Do not know |
| **CERTAINTY OF EVIDENCE** | Very low | Low | Moderate | High |  |  | No included studies |
| **VALUES** | Important uncertainty or variability | Possibly important uncertainty or variability | Probably no important uncertainty or variability | No important uncertainty or variability |  |  |  |
| **BALANCE OF EFFECTS** | Favors the comparison | Probably favors the comparison | Does not favor either the intervention or the comparison | Probably favors the intervention | Favors the intervention | Varies | Do not know |
| **ACCEPTABILITY** | No | Probably no | Probably yes | Yes |  | Varies | Do not know |
| **FEASIBILITY** | No | Probably no | Probably yes | Yes |  | Varies | Do not know |

# Type of Recommendation

| Strong recommendation against the intervention | Conditional recommendation against the intervention | Conditional recommendation for either the intervention or the comparison | Conditional recommendation for the intervention | Strong recommendation for the intervention |
| --- | --- | --- | --- | --- |
| ○ | ○ | ● | ○ | ○ |

# Conclusion

| Recommendation |
| --- |
| No recommendation can be made as to whether ventilatory management using driving pressure as an index should be used for adult patients with ARDS. It is becoming increasingly important to ensure standard lung protective ventilation by limiting tidal volume ventilation and plateau pressure based on ideal body weight and to limit driving pressure when possible while being careful to avoid adverse effects such as hypercarpnia and acid-base imbalance **(in our practice statement).** |
|  |
| Justification |
| **Question**: Should driving pressure be used as an index when implementing mechanical ventilation in adult patients with ARDS?  **Patients**: Patients with ARDS that are 16 years and older  **Intervention**: Ventilatory management with limited driving pressure (driving pressure ≤15 cmH_2_O)  **Comparison**: Ventilatory management without limiting the driving pressure (driving pressure ≤15 cmH_2_O)  **Description:**  MEDLINE, CENTRAL, and medical journals were searched through July 14, 2020, and 13 observational studies met the inclusion criteria. We could not find any randomized controlled trials (RCTs) that met the inclusion criteria. In addition, all included observational studies were either not adjusted for confounders or did not describe the confounders themselves, so the risk of bias was high and quantitative integration was not performed.  While low driving pressures are expected to reduce ventilator induced lung injury (VILI), there are concerns about harm in terms of increased respiratory rate, atelectasis, hypercarpnia, and respiratory acidosis. On the contrary, several observational studies have suggested that lower driving pressures were associated with lower mortality rates. One possible reason that low driving pressure was associated with a lower mortality rate was that low driving pressure may have reduced VILI due to lung overstretching/overexpansion ^1)^. Low driving pressure has been shown to be associated with decreased lung overstretching/overexpansion and VILI, as well as decreased mortality, providing evidence to support this hypothesis ^2-3)^. However, observational studies to date have not adjusted for confounding factors, and it remains to be seen whether the association between low driving pressure and mortality is correlational or causal. Therefore, although driving pressure may be a prognostic indicator, we do not know whether using driving pressure as an indicator of ventilator settings improves the prognosis of patients with ARDS.  Although not included in the systematic review of this CQ, an open-label RCT was reported that evaluated the feasibility of using driving pressure as a measure of ventilation compared with conventional lung protective ventilation in patients with ARDS whose baseline driving pressure was 13 cm H2O or higher ^4)^. When the ARDSnet protocol was used in 31 patients, with a ventilation rate of 4 to 8 mL/kg of PBW and a driving pressure of 10 cm H_2_O or less in the intervention group and a ventilation rate of 6 mL/kg PBW in the control group, the driving pressure from day 1 to day 3 was 4.6 cm H _2_O lower in the intervention group (95% CI, 6.5 to 2.8; P < 0.001). There was no statistically significant difference in the incidence of severe acidosis (pH < 7.10) within 7 days (absolute difference, -12.1; 95% CI, -41.5 to -17.3). This RCT suggested that a trial evaluating ventilation with limited driving pressure in patients with ARDS is feasible.  However, the balance between the benefits and harms of using driving pressure as a measure of management in clinical practice is unknown, and no clear recommendations can be made in response to this CQ. Therefore, this CQ is not an evidence-based recommendation but rather a description of current practice.    **Summary of evidence:** No relevant studies  **Certainty of evidence:** Since there are no relevant studies, the quality of evidence cannot be assessed  **Determine values, balance of effects, acceptance, and viability:**  We could not evaluate the benefits and harms due to the lack of high quality evidence comparing low and high driving pressures. In terms of burden and cost, we only measured the driving pressure and used it as an indicator to set the ventilator settings. As there is no cost for additional equipment, implementation would be feasible with education and appropriate staffing.  **Panel discussion:**  In a prior vote, the modified Delphi method stated, "No recommendation can be made as to whether driving pressure should be used as an index for ventilatory management in adults with ARDS. It is increasingly important to ensure standard lung protective ventilation that is based on ideal body weight and limits plateau pressures, and to limit driving pressures when possible, with attention to the harms of hypercapnia and acid-base dysbalance. (in our practice statement)," with a median score of 9.0 and a disagreement index of 0.1316. As a result, the panel meeting finally reached a consensus on the pre-vote without requiring a re-vote. |

| Subgroup considerations |
| --- |
| None |
| Implementation considerations |
| There is no mention of ventilatory management using driving pressure as an index in the ARDS Clinical Practice Guidelines 2016 and ATS/ESICM/SCCM 2017.  It should be noted that transpulmonary pressure may be high due to strong spontaneous respiratory effort, even if the apparent driving pressure is low when spontaneous breathing is present. The benefits should be balanced against the possible harms of low driving pressure, such as increased respiratory rate, atelectasis, hypercapnia, respiratory acidosis, and asynchroy. |

| Monitoring and evaluation |
| --- |
| To implement the recommendations, it is necessary to collect more information on whether or not driving pressure is being managed with awareness of clinical issues. After the guidelines are published, it is also necessary to monitor whether there are any other clinical problems through the use of questionnaires. |
| Research priorities |
| Previous observational studies have suggested an association between low driving pressure and a lower mortality rate. However, these were hypothesis-generating studies, and they only implied that driving pressure was a predictive indicator. In other words, there is currently insufficient evidence to support the routine clinical use of ventilation strategies using driving pressure as an indicator.  The pilot RCT by Romano et al. suggested that it was feasible to validate management using driving pressure as an index, and a large RCT should be conducted. Future well-designed RCTs are needed to evaluate the efficacy of ventilation strategy using driving pressure as an indicator compared to the current standard of care (i.e., lung protective ventilation). |

References

1) Tonetti T, Vasques F, Rapetti F, et al. Driving pressure and mechanical power: new targets for VILI prevention. Ann Transl Med. 2017;5(14):286. PMID: 28828361.

2) Amato MB, Meade MO, Slutsky AS, et al. Driving pressure and survival in the acute respiratory distress syndrome. N Engl J Med. 2015;372(8):747-55. PMID: 25693014.

3) Chiumello D, Carlesso E, Brioni M, et al. Airway driving pressure and lung stress in ARDS patients. Crit Care. 2016;20:276. PMID: 27545828

4) Pereira Romano ML, Maia IS, Laranjeira LN, et al. Driving pressure-limited strategy for patients with acute respiratory distress syndrome. A pilot randomized clinical trial. Ann Am Thorac Soc. 2020;17(5):596-604. PMID: 32069068.

**CQ30 Is low SpO_2_ (PaO_2_) a target for management in adult patients with ARDS?**

1. Search strategy

MEDLINE via PubMed （Search date: 2020/4/17）

| #1 | exp Oxygen Inhalation Therapy/ |
| --- | --- |
| #2 | (Oxygen adj3 (therap* OR treatment* OR intervention* OR manage*)).ti,ab,kw. |
| #3 | COT.ti,ab. |
| #4 | #1 OR #2 OR #3 |
| #5 | pulse oxygen saturation*.ti,ab,kw. |
| #6 | exp Oximetry/ |
| #7 | Oximetr*.ti,ab,kw. |
| #8 | arterial blood oxygen saturation.ti,ab,kw. |
| #9 | (arterial blood oxygen partial pressure OR arterial blood oxygen partial tension).ti,ab,kw. |
| #10 | OR /#5-#9 |
| #11 | exp Therapeutics/ |
| #12 | (therap* or treatment* or intervention* or manage*).ti,ab,kw. |
| #13 | #11 OR #12 |
| #14 | #10 AND #13 |
| #15 | #4 OR #14 |
| #16 | exp Mortality/ |
| #17 | (mortalit* or fatalit* or die or died or death or survival).ti,ab,kw. |
| #18 | #16 OR #17 |
| #19 | #15 AND #18 |
| #20 | exp Randomized Controlled Trial/ |
| #21 | randomized controlled trial.pt. |
| #22 | (random* and (study or studies or trial* or group*)).mp. |
| #23 | #20 OR #21 OR #22 |
| #24 | #19 AND #23 |

CENTRAL （Search date: 2020/4/17）

| #1 | Oxygen therap* or oxygen treatment* or oxygen intervention* or oxygen manage* |
| --- | --- |
| #2 | pulse oxygen saturation* |
| #3 | Oximetr* |
| #4 | arterial blood oxygen saturation or arterial blood oxygen partial pressure or arterial blood oxygen partial tension |
| #5 | #2 OR #3 OR #4 |
| #6 | therap* or treatment* or intervention* or manage* |
| #7 | #5 AND #6 |
| #8 | mortalit* or fatalit* or die or died or death or survival |
| #9 | #1 AND #7 AND #8 (in trials) |

EMBASE （Search date: 2020/4/17）

| S1 | exp Oxygen Therapy/ |
| --- | --- |
| S2 | (Oxygen adj3 (therap* OR treatment* OR intervention* OR manage*)).ti,ab,kw. |
| S3 | COT.ti,ab. |
| S4 | S1 OR S2 OR S3 |
| S5 | pulse oxygen saturation*.ti,ab,kw. |
| S6 | exp Oximetry/ |
| S7 | Oximetr*.ti,ab,kw. |
| S8 | exp oxygen saturation/ |
| S9 | exp arterial oxygen tension OR exp arterial oxygen saturation |
| S10 | arterial blood oxygen saturation.ti,ab,kw. |
| S11 | (arterial blood oxygen partial pressure OR arterial blood oxygen partial tension).ti,ab,kw. |
| S12 | OR/S5-S11 |
| S13 | exp Therapy |
| S14 | (therap* OR treatment* OR intervention* OR manage*).ti,ab,kw. |
| S15 | S13 OR S14 |
| S16 | S13 OR S14 |
| S17 | S4 OR S16 |
| S18 | exp Mortality/ |
| S19 | (mortalit* OR fatalit* OR die OR died OR death OR survival).ti,ab,kw. |
| S20 | S18 OR S19 |
| S21 | S17 AND S20 |
| S22 | exp Randomized Controlled Trial |
| S23 | (random* AND (study OR studies OR trial* OR group*)).mp. |
| S24 | limit S21 to randomized controlled trial |
| S25 | S22 OR S23 |
| #26 | S21 AND S25 |
| #27 | S24 OR S26 |

Web of Science （Search date: 2020/4/17）

| #1 | TS = (Oxygen Near/3 (therap* or treatment* or intervention* or manage*)) |
| --- | --- |
| #2 | TS = (COT) |
| #3 | #2 OR #1 |
| #4 | TS = (pulse oxygen saturation*) |
| #5 | TS = (Oximetr*) |
| #6 | TS = (arterial blood oxygen saturation or arterial blood oxygen partial pressure or arterial blood oxygen partial tension) |
| #7 | #6 OR #5 OR #4 |
| #8 | TS = (therap* or treatment* or intervention* or manage*) |
| #9 | #8 AND #7 |
| #10 | #9 OR #3 |
| #11 | TS = (mortalit* or fatalit* or die or died or death or survival) |
| #12 | #11 AND #10 |
| #13 | TS = (random* and (study or studies or trial* or group*)) |
| #14 | #13 AND #12 |

1. Flow diagram

**Identification**

8 Studies included in existing SR

（Xu Zhao2021^1）^）

23 Full-text articles assessed for eligibility

4948 records after duplicates removed

6499 records identified through database searching

6499 records identified through database searching

Medline via Ovid (n=1149)

EMBASE（Ovid） (n=3229)

CENTRAL (n=978)

Web of science (n=1143)

0 additional records identified through other sources

1) Xu Zhao, Huaping Xiao, Feng Dai, et.al. Classification and effectiveness of different oxygenation goals in mechanically ventilated critically ill patients: network meta-analysis of randomised controlled trials. Eur Respir J 2002928, 2021. PMID: 33632796

6 Studies included in quantitative synthesis (meta-analysis)

15 Full-text articles excluded, with reasons:

・Wrong intervention (n=5)

・Wrong population (n=4)

・Wrong outcome (n=3)

・Wrong study design (n=2)

・Result duplicated in an included study (n=1)

Duplicates

n=1551

4925 records excluded

**Included**

**Eligibility**

**Screening**

1. Risk of bias

Long-term mortality Ventilator-free days


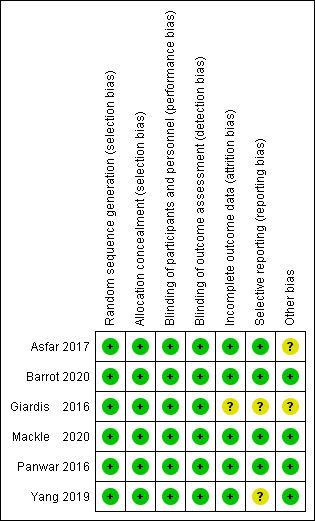

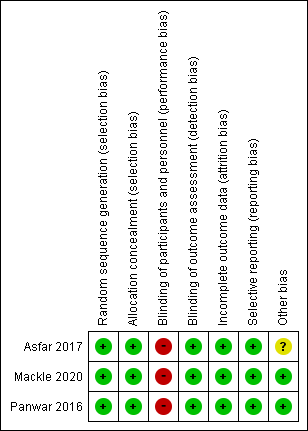


New-onset arrhythmias Bowel ischemia


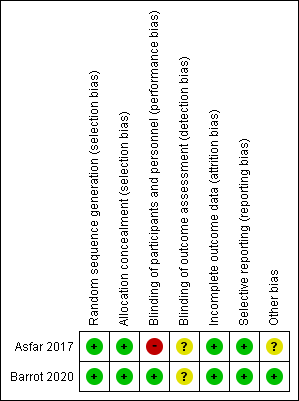

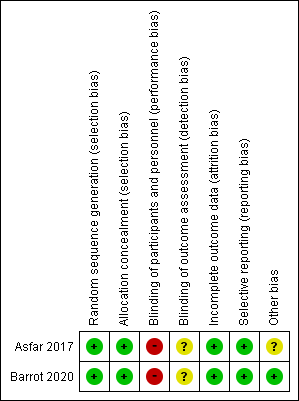


Renal replacement therapy ICU-AW


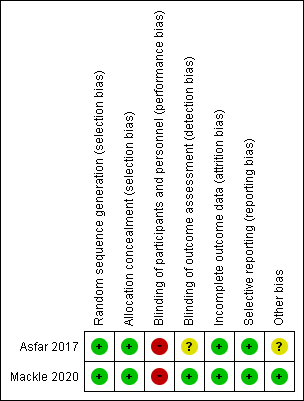

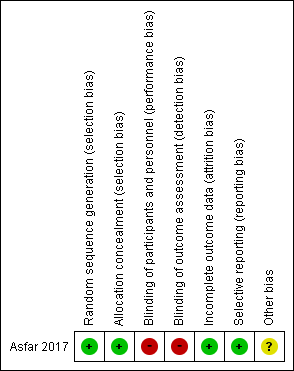


New-onset infections


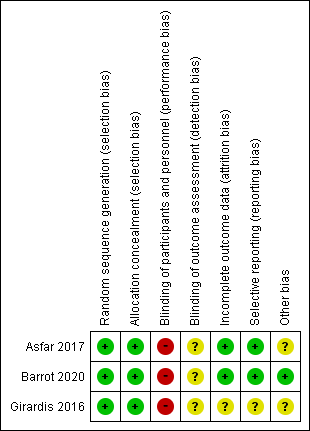


1. Forest plot

Long-term mortality


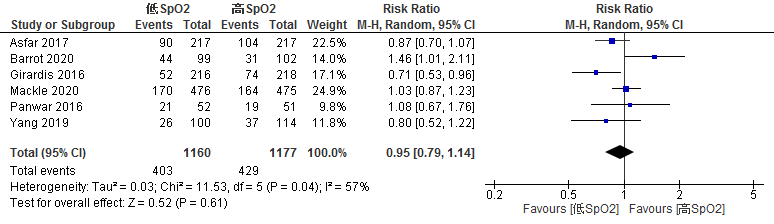


Ventilator-free days


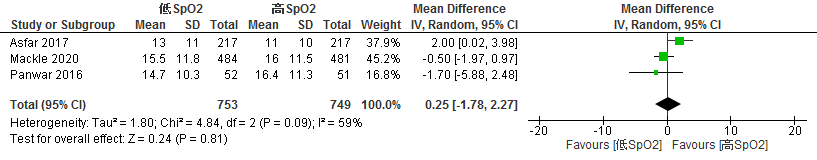


New-onset arrhythmias


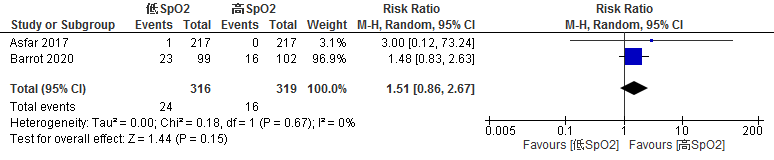


Bowel ischemia


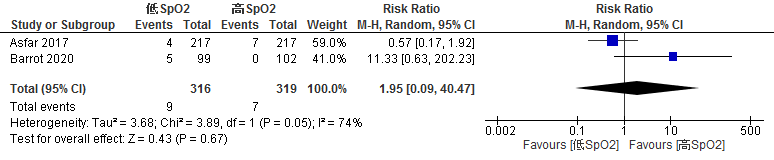


Renal replacement therapy


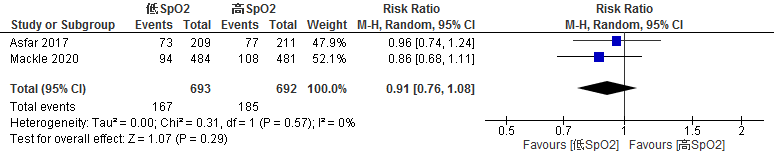


ICU-AW


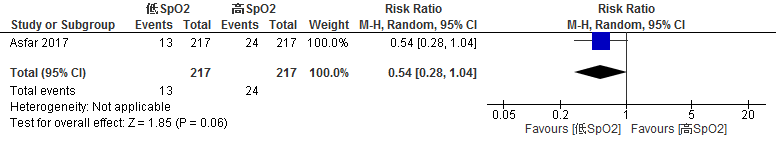


New-onset infections


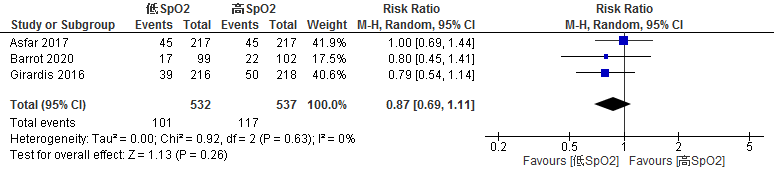


1. Evidence profile

| **Certainty assessment** | | | | | | | **№ of patients** | | **Effect** | | **Certainty** | **Importance** |
| --- | --- | --- | --- | --- | --- | --- | --- | --- | --- | --- | --- | --- |
| **№ of studies** | **Study design** | **Risk of bias** | **Inconsistency** | **Indirectness** | **Imprecision** | **Other considerations** | **LowSpO_2_** | **High SpO_2_** | **Relative (95% CI)** | **Absolute (95% CI)** |  |  |
| **Long-term mortality＊** | | | | | | | | | | | | |
| 6 | Randomized trials | Not serious | Serious ^a^ | Not serious ^b^ | Not serious ^c^ | None | 403/1160 (34.7%) | 429/1177 (36.4%) | **RR 0.95** (0.79 to 1.14) | **18 fewer per 1000**  (77 fewer to 51 more) | ⨁⨁⨁◯ Moderate | Critical |
| **VFD** | | | | | | | | | | | | |
| 3 | Randomized trials | Serious ^d^ | Serious ^e^ | Not serious ^b^ | Serious ^f^ | None | 753 | 749 | - | **MD** **0.25 days longer**  (1.78 shorter to 2.27 longer) | ⨁◯◯◯ Very low | Critical |
| **New-onset arrhythmias** | | | | | | | | | | | | |
| 2 | Randomized trials | Not serious | Not serious | Serious ^g^ | Serious ^h^ | None | 24/316 (7.6%) | 16/319 (5.0%) | **RR 1.51** (0.86 to 2.67) | **26 more per 1000** (7 fewer to 84 more) | ⨁⨁◯◯ Low | Critical |
| **Bowel ischemia** | | | | | | | | | | | | |
| 2 | Randomized trials | Serious ^i^ | Serious ^j^ | Serious ^g^ | Serious ^k^ | None | 9/316 (2.8%) | 7/319 (2.2%) | **RR 1.95** (0.09 to 40.47) | **21 more per 1000** (20 fewer to 866 more) | ⨁◯◯◯ Very low | Critical |
| **Renal replacement therapy** | | | | | | | | | | | | |
| 2 | Randomized trials | Not serious | Not serious | Not serious ^b^ | Serious ^l^ | None | 167/693 (24.1%) | 185/692 (26.7%) | **RR 0.91** (0.76 to 1.08) | **24 fewer per 1000**  (64 fewer to 21 more) | ⨁⨁⨁◯ Moderate | Important |
| **ICU-AW** | | | | | | | | | | | | |
| 1 | Randomized trials | Very serious ^m^ | Not serious | Very serious ^n^ | Serious ^o^ | None | 13/217 (6.0%) | 24/217 (11.1%) | **RR 0.54** (0.28 to 1.04) | **51 fewer per 1000**  (80 fewer to 4 more) | ⨁◯◯◯  Very low | Critical |
| **New-onset infections** | | | | | | | | | | | | |
| 3 | Randomized trials | Serious ^d^ | Not serious | Not serious ^b^ | Serious ^p^ | None | 101/532 (19.0%) | 117/537 (21.8%) | **RR 0.87** (0.69 to 1.11) | **28 fewer per 1000**  (68 fewer to 24 more) | ⨁⨁◯◯ Low | Important |

**CI:** confidence interval; **RR:** risk ratio; **MD**, mean difference; VFD: ventilator-free day; AW: acquired weakness

* We adopted the mortality outcome at the longest time point of those addressed in the study.

#### Description

a. Since there was moderate statistical heterogeneity with I2=57%, and clinical heterogeneity as a result of the visual judgment of the forest plot, it was judged as "serious" and downgraded by one level.

b. Although the study population differed from that of the guideline in some aspects, such as general ICU patients and septic patients in addition to those with ARDS, more than 50% of the studies involved ventilated patients, and the non-directivity was judged to be "not serious.”

c. The total sample size was 2337, and the number of events was 832, which met the optimal information size (OIS), and the confidence interval did not meet "substantial harm" and "substantial benefit"; therefore, no grade down was assumed.

d. The risk of bias (RoB) was high for multiple items (blinding of therapists) and the risk of bias was judged to be "severe."

e. Since there was moderate statistical heterogeneity with I2=59% and clinical heterogeneity as a result of the visual judgment of the forest plot, it was judged as "serious" and downgraded by one level.

f. The total sample size was 1502, which was considered to satisfy the OIS as a continuous variable; however, the 95% confidence interval was wide and the upper and lower confidence intervals were both considered to cross the minimum important difference (MID), which was judged to be "serious" and downgraded by one level.

g. Because the majority of the study population was septic and the design of the study was somewhat different from that targeting low SpO2, we judged the non-directivity to be "serious.

h. The total sample size of 335 and the number of events of 40 do not meet the OIS, and the 95% confidence intervals are wide and include "no effect" and "substantial harm."

i. RoB was high for multiple items (blinding of therapists) and the risk of bias was judged to be "severe."

j. Because of the moderate statistical heterogeneity of I2=74% and clinical heterogeneity based on visual assessment of the forest plot, it was judged to be "serious" and downgraded by one level.

k. The total sample size is 635 and the number of events is 16, which does not meet the OIS.

l. The total sample size is 1385 and the number of events is 352, which does not meet the OIS.

m. The RoB was high for multiple items (blinding of therapists and raters), and the risk of bias was judged to be "very serious."

n. Because the study population was limited to patients with sepsis and the study design was somewhat different from those targeting low SpO2, we judged the non-directivity to be "very serious.”

o. The total sample size was 434, and the number of events was 37, which did not meet the OIS, and the 95% confidence interval was wide, and the confidence interval includes "no effect" and "substantial benefit.”

p. The total sample size is 1069 and the number of events is 218, which do not meet the OIS, and the 95% confidence interval is wide, and the confidence interval includes "no effect" and "substantial benefit.”

1. Evidence-to-Decision table

| Question | |
| --- | --- |
| **CQ30：Is low SpO_2_ (PaO_2_) a target for management in adult patients with ARDS?** | |
| **Patient:** | Adult patients requiring a ventilator (studies will be recruited that include at least 50% of ventilated patients) |
| **Intervention:** | Targeted management of low SpO_2_ (PaO_2_) (definition per literature) |
| **CONTROL:** | Targeted management of higher SpO_2_ (PaO_2_) (definition per literature) |
| **Primary OUTCOME** | Long-term mortality, ventilator-free days (VFD), new onset arrhythmia, bowel ischemia, ICU-AW (intensive care unit-acquired weakness) |
| **SETTING:** | The emergency room or ICU |
| **POINT OF VIEW:** | Personal |
| **BACKGROUND:** | Oxygen therapy is indispensable for patients with ARDS, and SpO_2_ monitoring during treatment is essential. In recent years, randomized controlled trials have shown that management with a low SpO_2_ target improved mortality, while others suggested an increase in mortality, and the effects were not consistent. Thus, it is necessary to examine the benefits and harms of targeted management of low SpO_2_ in patients with ARDS and define a target SpO_2_ value. |
| **CONFLICT OF INCIDENTS:** | None |

# assessment

| Problem Is the problem a priority? | | |
| --- | --- | --- |
| Judgment | Research Evidence | REMARKS |
| ○ No  ○ Probably no  ○ Probably yes  ● Yes  ○ Varies  ○ Do not know | Oxygen therapy is indispensable for patients with ARDS, and SpO_2_ monitoring during treatment is essential. In recent years, randomized controlled trials have shown that management with a low SpO_2_ target improved mortality, while others suggested an increase in mortality, and the effects were not consistent. Thus, it is necessary to examine the benefits and harms of targeted management of low SpO_2_ in patients with ARDS and define a target SpO_2_ value. Thus, it is judged to be of great clinical significance. |  |
| Desirable effects How substantial are the desirable anticipated effects? | | |
| JUDGMENT | RESEARCH EVIDENCE | REMARKS |
| ○Trivial  ●Small  ● Moderate  ○Large  ○Varies  ○Do not know | A literature search using existing systematic reviews revealed six randomized controlled trials (RCTs) consistent with the patient, intervention, comparison, and outcome (PICO) process, and we performed a meta-analysis using these.  The effect estimates for long-term mortality (6 RCTs: N=2337) were 18 fewer risks/1000 (95% CI: 77 fewer to 51 more) for the intervention compared to the control, VFD (3 RCTs: N=1502) by a mean difference of 0.25 days longer (95% CI: 1.78 days shorter to 2.27 days longer), and for ICU-AW (1 RCT: N=434) by a mean difference of 51 fewer patients per 1000 (95% CI: 80 patients fewer to 4 patients more). Thus, the desired effect of the intervention was judged to be “small,” |  |
| Undesirable effects How substantial are the undesirable anticipated effects? | | |
| JUDGMENT | RESEARCH EVIDENCE | REMARKS |
| ○Large  ○ Moderate  ●Small  ○Trivial  ○Varies.  ○ Do not know | The effect estimate for new arrhythmias (2 RCTs: N=635) was an increase in the risk of 26 people/1000 (95% CI: decrease of 7 people to increase of 84 people) in the “intervention” compared to the “control.” The effect estimate for intestinal ischemia (2 RCTs: N=635) was an increase of 21/1000 (95% CI: decrease of 20 to increase of 866). Thus, the undesirable effect of the intervention was judged to be “small.” |  |
| Certainty of evidence What is the overall certainty of the evidence of effects? | | |
| JUDGMENT | RESEARCH EVIDENCE | REMARKS |
| ●Very low  ○Low  ○Moderate  ○High  ○No included studies | The relative importance and values of the key outcomes of interest:   \| Outcome \| Relative importance \| Certainty of evidence (GRADE) \| \| --- \| --- \| --- \| \| \| \| Long-term mortality* \| Critical \| ⨁⨁⨁◯ \| \| Moderate \| \| VFD \| Critical \| ⨁◯◯◯ \| \| Very low \| \| New arrhythmia \| Critical \| ⨁⨁◯◯ \| \| Low \| \| Bowel ischemia \| Critical \| ⨁◯◯◯ \| \| Very low \| \| ICU-AW \| Critical \| ⨁◯◯◯ \| \| Very low \|   * Deaths during the longest time period included in the study (including 28-day, 60-day, 90-day, 180-day, and inpatient deaths)  **Overall certainty of evidence:**  The directionality in the outcome of benefit and harm was not consistent, and the certainty of evidence across outcomes was judged to be “very low,” adopting the lowest certainty of evidence. |  |
| Values Is there important uncertainty about or variability in how much people value the main outcomes? | | |
| JUDGMENT | RESEARCH EVIDENCE | REMARKS |
| ○Important uncertainty or variability  ○Possibly important uncertainty or variability  ●Probably no important uncertainty or variability  ○No important uncertainty or variability | There are no data on values for outcomes in management with low SpO2, but the value for death is generally high, and the variability is low. |  |
| Balance of effects Does the balance between desirable and undesirable effects favor the intervention or the comparison? | | |
| JUDGMENT | RESEARCH EVIDENCE | REMARKS |
| ○Favors the comparison  ○Probably favors the comparison  ○Does not favor either the intervention or the comparison  ○Probably favors the intervention  ○Favors the intervention  ○Varies  ●Do not know | **Summary of Findings**   \| Outcome \| Management \| \| Absolute difference  (95% CI) \| Relative effect  (RR) (95% CI) \| \| --- \| --- \| --- \| --- \| --- \| \| High SpO2  (control) \| Low SpO2  (intervention) \| \| Long-term mortality \| 429/1177 \| 403/1160 \| 18 fewer /1000 (77 fewer to 51 more) \| 0.95 (0.79-1.14) \| \| VFD \| - \| - \| MD 0.25 day longer  (1.78 shorter to 2.27 longer) \| - \| \| New arrhythmia \| 16/319 \| 24/316 \| 26 more /1000 (7 fewer to 84 more) \| 1.51 (0.86-2.67) \| \| Bowel ischemia \| 7/319 \| 9/316 \| 21 more /1000 (20 fewer to 866 more) \| 1.95 (0.09-40.47) \| \| ICU-AW \| 24/217 \| 13/217 \| 51 fewer /1000 (80 fewer to 4 more) \| 0.54 (0.28-1.04) \|   The balance between the desirable and undesirable effects was judged to be “Unknown.” |  |
| Acceptability Is the intervention acceptable to key stakeholders? | | |
| JUDGMENT | RESEARCH EVIDENCE | REMARKS |
| ○ No  ○ Probably no  ● Probably yes  ○ Yes  ○ Varies  ○ Do not know | Although there is no evidence used for evaluating acceptability, it was judged to be sufficiently acceptable considering the equipment, cost, and other aspects of the ICU system. |  |
| Feasibility Is the intervention feasible to implement? | | |
| JUDGMENT | RESEARCH EVIDENCE | REMARKS |
| ○ No  ○ Probably no  ● Probably yes  ○ Yes  ○ Varies  ○ Do not know | Although there is no evidence used for evaluating feasibility, it seemed feasible for patients with ARDS because no new equipment was needed for low SpO2 management, and aiming for a relatively low SpO2 is already common in patients with chronic obstructive pulmonary disease (COPD). |  |

# SUMMARY OF JUDGMENT

|  | **JUDGMENT** | | | | | | |
| --- | --- | --- | --- | --- | --- | --- | --- |
| **PROBLEM** | No | Probably no | Probably yes | Yes |  | Varies | Do not know |
| **DESIRABLE EFFECTS** | Trivial | Small | Moderate | Large |  | Varies | Do not know |
| **UNDESIRABLE EFFECTS** | Large | Moderate | Small | Trivial |  | Varies | Do not know |
| **CERTAINTY OF EVIDENCE** | Very low | Low | Moderate | High |  |  | No included studies |
| **VALUES** | Important uncertainty or variability | Possibly important uncertainty or variability | Probably no important uncertainty or variability | No important uncertainty or variability |  |  |  |
| **BALANCE OF EFFECTS** | Favors the comparison | Probably favors the comparison | Does not favor either the intervention or the comparison | Probably favors the intervention | Favors the intervention | Varies | Do not know |
| **ACCEPTABILITY** | No | Probably no | Probably yes | Yes |  | Varies | Do not know |
| **FEASIBILITY** | No | Probably no | Probably yes | Yes |  | Varies | Do not know |

# TYPE OF RECOMMENDATION

| Strong recommendation against the intervention | Conditional recommendation against the intervention | Conditional recommendation for either the intervention or the comparison | Conditional recommendation for the intervention | Strong recommendation for the intervention |
| --- | --- | --- | --- | --- |
| ○ | ● | ○ | ○ | ○ |

# CONCLUSION

| Recommendation |
| --- |
| **It is recommended with conditions not to target excessively low SpO_2_ (PaO_2_) for management in adult patients with ARDS (conditional recommendation/evidence of very low certainty: GRADE 2D).**  **Supplementary items: The optimal SpO_2_ (PaO_2_) is unknown at this time. Management should be such that excessive hypoxia and hyperoxia are avoided.** |
|  |
| Justification |
| **Question:** Is low SpO_2_ (PaO_2_) a target for management in adult patients with ARDS?  **Patients:** Adult patients requiring a ventilator (studies will be recruited that include at least 50% of ventilated patients)  **Intervention:** Targeted management of low SpO_2_ (PaO_2_) (definition per literature)  **Comparison control:** Targeted management of higher SpO_2_ (PaO_2_) (definition per literature)  **Outcome:** Long-term mortality, VFD, new onset arrhythmia, bowel ischemia, ICU-AW  **Summary of evidence:**  From the network meta-analysis of Xu et al.1), we collected studies consistent with the PICO of this CQ and found 6 RCTs (N=2337) that compared the management of adult ventilated patients with targeted low SpO_2_ (PaO_2_) and high SpO_2_ (PaO_2_). The outcome of benefit with low SpO_2_ was long-term mortality (18 fewer patients/1000, 95% CI 77 decrease to 51 increase), VFD (0.25 days longer, 95% CI 1.76 shorter to 2.27 longer), ICU-AW (51 fewer patients/1000, 95% CI 80 decrease to 4 increase). The effect was judged to be “small.” On the contrary, as adverse outcomes, new arrhythmia (26 patients increased/1000, 95% CI 7 decrease to 84 increase) and bowel ischemia (21 patients more/1000, 95% CI 20 fewer to 866 more) were observed, which were judged as “small effect.” The balance of the effects was judged to be “unknown.”  **Certainty of evidence:**  The directions in the outcomes were not consistent, and the certainty of the evidence was judged to be “very low,” the lowest certainty of all outcomes.  **Determining the balance of effects, acceptability, feasibility:**  The intervention aims to change the SpO_2_ target value and is not considered expensive. In addition, the management of patients with COPD and others with low SpO_2_ is common and may be feasible in patients with ARDS.  **Panel meeting:**  　In the pre-vote, the modified Delphi method resulted in a median score of 8 points and a disagreement index of 0.1316 for “conditional recommendation not to manage adult patients with ARDS with excessively low SpO_2_ (PaO_2_) targets (conditional recommendation/very low certainty evidence: GRADE2D).” The panel discussed whether specific SpO_2_ values should be provided for the recommendation, but it was decided that there was not enough evidence to do so. As a result, there was no re-vote at the panel meeting, and the final consensus was reached with the result of the pre-vote.  **Additional considerations:**  　It should be noted that many of the studies included in this systematic review dealt with ventilated patients in ICUs, and the results may not necessarily apply to patients with ARDS in general (Barrot 2020, which included only patients with ARDS, found increased mortality with low SpO2 management). In addition, several of the included studies excluded patient groups for whom high oxygen concentrations may be harmful, such as patients with COPD. Therefore, these patients are not included in this recommendation.  　The network meta-analysis by Xu et al.1), also included in this systematic review, analyzed the optimal SpO_2_ for ICU patients requiring ventilator management. They identified the optimal SpO_2_ for far-conservative (PaO_2_: 55-70 mmHg, SaO2/SpO2 88-94%), conservative (PaO2: 70-90 mmHg, SaO_2_/SpO_2_ 94-97%), moderate (PaO_2_: 90-150 mmHg, SpO_2_ 97-100%), and liberal (PaO_2_>150 mmHg) groups. The prognosis tended to be better in the order of moderate, conservative, liberal, and far-conservative, although there was no significant difference.  Although it is difficult to determine the optimal SpO_2_ based on these results alone, it is highly unlikely that there will be a clear difference in prognosis within the range of normal SpO_2_ control (SpO_2_: 90-98%). In contrast, an excessively conservative or liberal approach may worsen the prognosis.  Below are the patients, sample sizes, SpO2 targets and outcomes, and mortality (RR) for the studies used in this systematic review.   \| Study \| Patient \| Sample size \| Rate of ventilation (％) \| Target SpO_2_ (Low SpO_2_ group) \| Actual SpO2 value (Low SpO_2_ group) \| Target SpO_2_  (High SpO_2_ group) \| Actual SpO2 value (High SpO_2_ group) \| Duration of target SpO_2_ \| Long-term mortality with low SpO_2_ management (RR) \| \| --- \| --- \| --- \| --- \| --- \| --- \| --- \| --- \| --- \| --- \| \| Asfar 2017 \| Septic shock \| 434 \| 100 \| 88-95％ \| Mean PaO_2_ 96 mmHg(SD 39) Median SaO_2_  97%(IQR 94-98) \| FiO2:  100% \| Mean PaO_2_ 227 mmHg(SD 124) SaO_2_: median 99% (IQR 97-100) \| 24 h \| 0.87 (0.70-1.07) \| \| Barrot 2020 \| ARDS \| 201 \| 100 \| 88-92％ \| Not Stated \| >96% \| Not stated \| 7 days \| 1.46 (1.01-2.11) \| \| Giradis 2016 \| ICU patient \| 478 \| 67 \| 94-98％ \| Median PaO_2_ 87 mmHg (IQR 79-97) \| 97-100% \| Median PaO_2_ 102 mmHg (IQR 88-116) \| During ICU \| 0.71 (0.53-0.96) \| \| Mackle 2020 \| ICU patient \| 965 \| 100 \| 91-97％ \| PaO_2_: 80-90 mmHg \| Not upper limit \| PaO_2_: 90-110 mmHg \|  \| 1.03 (0.87-1.23) \| \| Panwar 2016 \| ICU patient \| 103 \| 100 \| 88-92％ \| Mean SpO_2_: ~93% \| >96% \| Mean SpO_2_ ~97% \| >24 h \| 1.08 (0.67-1.76) \| \| Yang 2016 \| ICU patient \| 214 \| 84 \| 90-95％ \| Median PaO_2_: 84 mmHg (IQR71-99) \| 96-100% \| Median PaO_2_ 98 mmHg (IQR 79-116) \|  \| 0.80 (0.52-1.22) \| \| Olav 2021^2)^ (reference) \| ICU patient \| 2910 \| 59 \| PaO_2_: 60mmHg \| ＞50%:  PaO_2_< 70 mmHg ＞50％:  SpO_2_< 94% \| PaO2: 90mmHg \| ＞90%:  PaO_2_ 90-120 mmHg ＞50％:  SpO_2_> 97% \| 90 days \| 1.01 (0.93-1.10) \|   The only study that limited its scope to patients with ARDS was Barrot 2020, which found a significant increase in mortality with low SpO2 control. |

| Subgroup considerations |
| --- |
| In 2021, after this systematic review, a PICO-matched randomized controlled trial was published by NEJM (Olav 2012)). In total, 2910 patients were included, and considering the possibility that the inclusion of this RCT would change the recommendation, we performed a meta-analysis including this study as a sensitivity analysis. The effect estimate for long-term mortality (6 RCTs: N=5225) was a risk difference of 12 people/1000 (95% CI: 60 people decrease to 40 people increase), and the effect estimate for bowel ischemia (3 RCTs: N=3535) was a risk difference of 2 people/1000 (95% CI: 12 people decrease to 37 people increase). Although there was a reduction in the width of the CI, there was no change in the direction of the effect, and the inclusion of this study did not result in a change in the recommendation. |
| Implementation considerations |
| No mention was found in the ARDS Clinical Practice Guidelines 2016 and ATS/ESICM/SCCM 2017 regarding the management of patients with ARDS with a target of low SpO_2_.  Management using pulse oximetry is considered feasible as it is routinely performed. However, we should pay attention to conditions that may affect the accuracy and precision of SpO_2_ measurements, such as peripheral circulatory failure, high CO-Hb levels, severe anemia, and dyes, as well as conditions and underlying diseases that may worsen with hyperoxia or hypoxia, which are excluded in the above studies. |

| Monitoring and evaluation |
| --- |
| To implement the recommendations, it is necessary to collect more information on the harms of low SpO_2_ and high SpO_2_ as clinical problems. It is also necessary to monitor for other clinical problems through the use of questionnaires and other means after the guidelines are published. |
| Research priorities |
| This study mainly focused on patients undergoing ventilation in ICUs. Further studies on optimal SpO_2_ are needed. In addition, there is a need to understand whether different diseases have different targets for SpO_2_. |

References

1) Zhao X, Xiao H, Dai F, et al. Classification and effectiveness of different oxygenation goals in mechanically ventilated critically ill patients: network meta-analysis of randomized controlled trials. Eur Respir J. 2021;2002928. PMID: 33632796.

2) Schjørring OL, Klitgaard TL, Perner A, et al. Lower or higher oxygenation targets for acute hypoxemic respiratory failure. N Engl J Med. 2021;384(14):1301-1311. PMID: 33471452.
