# Supplementary material for: ARDS Clinical Practice Guideline 2021
Source: J Intensive Care. 2022 Jul 8;10:32. doi: 10.1186/s40560-022-00615-6 (PMC9263056; doi:10.1186/s40560-022-00615-6)
Supplement: Supplementary file 4 — Additional file 4. Contains Modified Preferred Reporting items of Systematic Reviews and Meta-Analyses (PRISMA) flow-chart, risk of bias summary, forest plots, evidence profiles, and evidence to decision table for CQ31–38 (area D) according to the GRADE system [file 40560_2022_615_MOESM4_ESM.docx]

Additional file 4

Modified Preferred Reporting items of Systematic Reviews and Meta-Analyses (PRISMA) flow-chart, risk of bias summary, forest plots, evidence profiles, and evidence to decision table for CQ31-38 (area D) according to the GRADE system

Table of contents

1. CQ31
   1. Search strategy p.3
   2. Flow diagram p.5
   3. Risk of bias p.6
   4. Forest plot p.8
   5. Evidence Profile p.10
   6. Evidence-to-Decision table p.13
2. CQ32
   1. Search strategy p.23
   2. Flow diagram p.25
   3. Risk of bias p.26
   4. Forest plot p.28
   5. Evidence Profile p.29
   6. Evidence-to-Decision table p.32
3. CQ33
   1. Search strategy p.40
   2. Flow diagram p.41
   3. Risk of bias p.42
   4. Forest plot p.42
   5. Evidence Profile p.42
   6. Evidence-to-Decision table p.43
4. CQ34
   1. Search strategy p.50
   2. Flow diagram p.51
   3. Risk of bias p.52
   4. Forest plot p.52
   5. Evidence Profile p.52
   6. Evidence-to-Decision table p.53
5. CQ35
   1. Search strategy p.61
   2. Flow diagram p.62
   3. Risk of bias p.63
   4. Forest plot p.65
   5. Evidence Profile p.67
   6. Evidence-to-Decision table p.71
6. CQ36
   1. Search strategy p.80
   2. Flow diagram p.83
   3. Risk of bias p.84
   4. Forest plot p.86
   5. Evidence Profile p.88
   6. Evidence-to-Decision table p.91
7. CQ37
   1. Search strategy p.99
   2. Flow diagram p.101
   3. Risk of bias p.102
   4. Forest plot p.105
   5. Evidence Profile p.107
   6. Evidence-to-Decision table p.110
8. CQ38
   1. Search strategy p.119
   2. Flow diagram p.119
   3. Risk of bias p.119
   4. Forest plot p.119
   5. Evidence Profile p.119
   6. Evidence-to-Decision table p.120

**CQ31 Should neuromuscular blockers be used at an early phase in adult patients with moderate or severe ARDS?**

1. Search strategy

MEDLINE via PubMed （Search date: 2020/6/23）

| #1 | Respiratory Distress Syndrome, Adult[mh] OR Respiratory Insufficiency[mh] OR Severe Acute Respiratory Syndrome[mh] OR respiratory distress syndrome[tiab] OR respiratory failure[tiab] OR ARDS[tiab] OR Acute Lung Injury[mh] OR acute lung injury[tiab] |
| --- | --- |
| #2 | neuromuscular blockade[mh] OR neuromuscular blocking agents[mh] OR muscle relaxants, central[mh] OR neuromuscular blocker[tiab] OR neuromuscular blockade[tiab] OR neuromuscular blocking drug*[tiab] OR neuromuscular blocking agent*[tiab] OR muscle relaxant[tiab] OR paralytics[tiab] OR respiratory paralysis[tiab] |
| #3 | vecuronium OR pancuronium OR rocuronium OR atracurium OR cisatracurium OR succinylcholine OR curare OR rapacuronium OR mivacurium OR mivacron OR tracrium OR doxacurium OR nuromax OR bex OR norcuron OR zemuron OR pavulon OR tubocurarine OR gallamine OR flaxedil OR pipecuronium OR alcuronium OR toxiferine OR suxamethonium OR raplon |
| #4 | #2 OR #3 |
| #5  #6 | #1 AND #4  animals[mh] NOT humans[mh] |
| #7 | #5 AND #6 |

CENTRAL （Search date: 2020/6/23）

| #1 | Respiratory Distress Syndrome, Adult[mh] OR Respiratory Insufficiency[mh] OR Severe Acute Respiratory Syndrome[mh] OR respiratory distress syndrome[tiab] OR respiratory failure[tiab] OR ARDS[tiab] OR Acute Lung Injury[mh] OR acute lung injury[tiab] |
| --- | --- |
| #2 | neuromuscular blockade[mh] OR neuromuscular blocking agents[mh] OR muscle relaxants, central[mh] OR neuromuscular blocker[tiab] OR neuromuscular blockade[tiab] OR neuromuscular blocking drug*[tiab] OR neuromuscular blocking agent*[tiab] OR muscle relaxant[tiab] OR paralytics[tiab] OR respiratory paralysis[tiab] |
| #3 | vecuronium OR pancuronium OR rocuronium OR atracurium OR cisatracurium OR succinylcholine OR curare OR rapacuronium OR mivacurium OR mivacron OR tracrium OR doxacurium OR nuromax OR bex OR norcuron OR zemuron OR pavulon OR tubocurarine OR gallamine OR flaxedil OR pipecuronium OR alcuronium OR toxiferine OR suxamethonium OR raplon |
| #4 | #2 OR #3 |
| #5 | #1 AND #4 |

Igaku-Chuo-Zasshi （Search date: 2020/6/23）

| #1 | 呼吸窮迫症候群-急性/TH or 急性呼吸窮迫症候群/TA or ARDS/TA |
| --- | --- |
| #2 | 急性肺損傷/TH or 急性肺損傷/TA |
| #3 | 重症急性呼吸器症候群/TH or SARS/TA |
| #4 | 呼吸不全/TH or 呼吸不全/TA |
| #5 | #1 or #2 or #3 or #4 |
| #6 | 神経筋遮断/TH or 神経筋遮断剤/TH or 中枢性筋弛緩剤/TH or 神経筋遮断/TA or 弛緩/TA |
| #7 | ("Vecuronium Bromide"/TH or vecuronium/AL) or (Pancuronium/TH or pancuronium/AL) or ("Rocuronium Bromide"/TH or rocuronium/AL) or ("Atracurium Besilate"/TH or atracurium/AL) or ("Cisatracurium Besilate"/TH or cisatracurium/AL) or (Succinylcholine/TH or succinylcholine/AL) or (Curare/TH or curare/AL) or ("Rapacuronium Bromide"/TH or rapacuronium/AL) or ("Mivacurium Chloride"/TH or mivacurium/AL) or ("Mivacurium Chloride"/TH or mivacron/AL) or ("Atracurium Besilate"/TH or tracrium/AL) or ("Doxacurium Chloride"/TH or doxacurium/AL) or ("Doxacurium Chloride"/TH or nuromax/AL) or bex/AL or ("Vecuronium Bromide"/TH or norcuron/AL) or ("Rocuronium Bromide"/TH or zemuron/AL) or (Pancuronium/TH or pavulon/AL) or (Tubocurarine/TH or tubocurarine/AL) or ("Gallamine Triethiodide"/TH or gallamine/AL) or ("Gallamine Triethiodide"/TH or flaxedil/AL) or (Pipecuronium/TH or pipecuronium/AL) or (Alcuronium/TH or alcuronium/AL) or (Toxiferine/TH or toxiferine/AL) or (Succinylcholine/TH or suxamethonium/AL) or ("Rapacuronium Bromide"/TH or raplon/AL) |
| #8 | #6 or #7 |
| #9 | #5 and #8 |
| #10 | (#9) and (PT=会議録除く) |

1. Flow diagram

**Identification**

5 Studies included in qualitative synthesis

1880 records after duplicates removed

1885 records identified through database searching

1885 records identified through database searching

Medline via PubMed (n=1337)

Cochrane CENTRAL (n=171)

Igaku-Chuo-Zasshi (n=377)

0 additional records identified through other sources

5 Studies included in quantitative synthesis (meta-analysis)

76 Full-text articles excluded, with reasons:

・Wrong language (n=3)

・Wrong study design (n=59)

・Wrong population (n=13)

・Wrong intervention (n=1)

Etc.

Duplicates

n=5

1799 records excluded

**Included**

**Eligibility**

**Screening**

81 Full-text articles assessed for eligibility

3. Risk of bias

Survival QOL

Ventilator-free days P/F ratio

VALI Pneumothorax

ICU-AW

1. Forest plot

Survival

QOL

Ventilator-free days

P/F ratio

VALI

Pneumothorax

ICU-AW

1. Evidence profile

| Assessment of certainty | | | | | | | No. of patients | | **Efficacy** | | Certainty of the evidence | **Importance** |
| --- | --- | --- | --- | --- | --- | --- | --- | --- | --- | --- | --- | --- |
| No. of studies | Study design | Risk of bias | Indirectness | Inconsistency | Imprecision | **Others** | **NBM use** | **NMB not use** | **Relative index (95% CI)** | **Absolute index (95% CI)** |  |  |
| **Survival (follow up: maximum observation period)** | | | | | | | | | | | | |
| 5 | RCT | Not Serious^a^ | Serious^b^ | Not Serious | Serious^c^ | None | 445/737 (60.4%) | 406/724(56.1%) | RR 1,15 (0.93 to 1.42) | +84 per 1000patients (-39〜+236) | ⨁⨁◯◯  low | Important |
| **QoL** | | | | | | | | | | | | |
| 1 | RCT | Serious^d^ | Not Serious | Not Serious | Serious^e^ | None | 127 | 119 |  | Average0.02 point low (-0.09〜+0.05) | ⨁⨁◯◯  low | Important |
| **Ventilator Free Days (28 days)** | | | | | | | | | | | | |
| 5 | RCT | Serious^f^ | Not Serious | Not Serious | Serious^g^ | None | 737 | 724 |  | Average0.72 days long(-0.52〜+1.95) | ⨁⨁◯◯ | Important |
| **P/F ratio (after 24 h)** | | | | | | | | | | | | |
| 3 |  | RCT | Serious^h^ | Not Serious | Not Serious | Serious^g^ | 636 | 595 |  | Average 5.68mmHg high (-3.56〜+14.91) | ⨁⨁◯◯  low | Important |
| **Ventilator-associated lung injury** | | | | | | | | | | | | |
| 4 | RCT | Serious^i^ | Not Serious | Not Serious | Serious^j^ | None | 29/724(4.0%) | 51/713(7.2%) | RR 0.56 (0.36 to 0.87) | -31 per 1000patients (-47〜-9) | ⨁⨁◯◯  low | Important |
| **Pneumothorax** | | | | | | | | | | | | |
| 3 | RCT | Serious^k^ | Not Serious | Not Serious | Serious^j^ | None | 21/706(3.0%) | 45/695(6.5%) | RR0.46(0.28 to 0.77) | -35 per 1000 patients(-47〜-15) | ⨁⨁◯◯  low | Important |
| **ICU acquired muscle weakness** | | | | | | | | | | | | |
| 4 | RCT | Serious^j^ | Serious^b^ | Not Serious | Serious^c^ | None | 180/384(46.9%) | 151/363(41.6%) | RR 1.06 (0.86 to 1.30) | +25 per 1000 patients(-58〜+125) | ⨁◯◯◯  Very low | Important |

ICU: intensive care unit; RCT: randomized controlled trial; CI: confidence interval; NMB: neuromuscular blockers; RR: relative risk

1. Inadequate blinding in four trials may have affected results; however, not sufficient to lower the grade.
2. Ranked down by 1 owing to the low overlap of confidence intervals and high heterogeneity.
3. Because the 95% confidence interval crosses the threshold for clinical judgment, it is downgraded by one rank.

d. Ranked down 1 as it is not blinded and the outcome is subjective.

e. Downgraded by 1 because the 95% confidence interval crossed the threshold for clinical judgment, the

proportion of outcomes reported is low, and the optimal information size (OIS) is not met.

f. Four trials are downgraded by one rank owing to inadequate blinding, which may have affected the results, and because of outcomes due to medical diagnosis.

g. One rank down because the 95% confidence interval crossed the threshold for clinical judgment.

h. Two trials have inadequate blinding, which may have affected the results, and one trial has a low proportion of outcomes reported, but not enough to lower the grade.

i. Three trials have inadequate blinding, which can affect the results, and one trial is downgraded by one grade owing to a medically diagnosed outcome.

j. One rank down due to failure to meet OIS.

k. Downgraded by one rank due to inadequate blinding in two studies, which may affect the results, and due to the outcome of medical diagnosis.

1. Evidence-to-Decision table

| **Question** | |
| --- | --- |
| **CQ31** Should neuromuscular blockers be used at an early phase in adult patients with moderate or severe ARDS? | |
| Population: | Adult patients with ARDS with moderate or severe disease (PaO2/FIO2 ratio ≤ 200 mmHg) |
| **Intervention:** | Use of any agent of neuromuscular blockade within 48 h of onset |
| **Comparison:** | No use of neuromuscular blockade |
| **Primary outcome:** | Survival (longest observation period), quality of life (QoL; EQ-5D), ventilator-free days (VFD: 28 days), barotrauma, intensive care unit-acquired weakness (ICU-AW) |
| **Setting:** | Situation equivalent to the emergency room or ICU |
| **Perspective:** | Individual |
| **Background:** | Preservation of spontaneous breathing during ventilation promotes lung recruitment during low airway pressure and contributes, preventing atelectasis and improving oxygenation. In contrast, respiratory effort is increased in patients with ARDS, and excessive spontaneous respiratory effort may cause patient self-inflicted lung injury (P-SILI), in which excessive stress on the alveolar space causes further lung injury. The use of muscle relaxants in adult patients with moderate to severe ARDS has been reported to improve patient-ventilator synchrony, decrease ventilator-induced lung injury (VILI), avoid excessive stress on alveolar spaces, decrease barotrauma, improve oxygenation, and improve the prognosis of patients with ARDS. However, there are concerns that unnecessary administration of muscle relaxants may delay early weaning, ICU-AW, and decrease QOL. Patients with ARDS may benefit from these outcomes by reducing patient-ventilator dyssynchrony, work of breathing, and alveolar fluid accumulation. However, prolonged administration of neuromuscular blocking agents leads to subsequent neuromuscular weakness. Many complications from treatment with reduced or eliminated spontaneous breathing have also been reported. In addition, muscle relaxants require deep sedation, which may be harmful. It may be an important clinical issue to clarify whether ventilatory management with muscle relaxants to reduce or eliminate spontaneous breathing in patients with moderate or severe ARDS is beneficial or harmful. Therefore, the priority of this issue is probably high. |
| **Conflict of interests:** | None |

**Assessment**

| **Problem**  Is the problem a priority? | | |
| --- | --- | --- |
| **Judgment** | **Research Evidence** | **ADDITIONAL CONSIDERATIONS** |
| ○ No  ○ Probably no  ○ Probably yes  ● Yes  ○ Varies  ○ Do not know | Preservation of spontaneous breathing during ventilation promotes lung recruitment due to low airway pressure and contributes to the prevention of atelectasis and improvement of oxygenation. On the other hand, respiratory effort is increased in patients with ARDS, and excessive spontaneous respiratory effort may cause patient self-inflicted lung injury (P-SILI), in which excessive stress on the alveolar space causes further lung injury 1). The use of muscle relaxants in adult patients with moderate to severe ARDS has been reported to improve patient-ventilator synchrony, decrease VILI, and improve oxygenation 2), suggesting that it may improve the prognosis of patients with ARDS 3). However, there are concerns that unnecessary administration of muscle relaxants may delay early weaning, ICU-AW, and decrease QOL. In addition, muscle relaxants require deep sedation, and deep sedation itself may be harmful. Clarifying the benefits and harms of ventilatory management with muscle relaxants to reduce or eliminate spontaneous breathing in patients with moderate or severe ARDS is likely to be an important clinical issue. Therefore, this issue is probably of high priority. |  |
| **Desirable effects**  How substantial are the desirable anticipated effects? | | |
| **Judgment** | **Research Evidence** | ADDITIONAL CONSIDERATIONS |
| ○ Trivial  ○ Small  ● Moderate  ○ Large  ○ Varies  ○ Do not Know | A systematic review identified five randomized controlled trials (RCTs) consistent with the patient, intervention, comparison, and outcome (PICO) process, and a meta-analysis was performed using these studies.  The effect estimates for survival (5 RCTs, N=1461) and QoL (EQ-5D, 1 RCT, N=246) showed and a mean difference of 0.02 points (95%CI: 0.09 lower to 0.05 higher), respectively. VFD (5 RCTs, N=1461) increased by 0.72 days (95% CI: 0.52 days decrease to 1.95 days increase), and barotrauma (4 RCTs, N=1437) decreased by 31 persons/1000 people ( 95%CI: 46 fewer to 9 fewer). Thus, the expected effect was judged to be “moderate.” |  |
| **Undesirable effects**  How substantial are the undesirable anticipated effects? | | |
| **Judgment** | **Research Evidence** | ADDITIONAL CONSIDERATIONS |
| ○ Large  ● Small  ○ Moderate  ○ Trivial  ○ Vary  ○ Do not know | As an outcome of harm, the effect estimates for ICU-AW (4 RCTs: N=747) was a risk difference of 25 more people/1000 (95% CI: 58 fewer people to 125 more people) with the use of muscle relaxants compared to no muscle relaxants. Thus, the expected harm was judged to be “small.” |  |
| **Certainty of evidence**  What is the overall certainty of the evidence of effects? | | |
| **Judgment** | **Research Evidence** | ADDITIONAL CONSIDERATIONS |
| ● Very low  ○ Low  ○ Moderate  ○ High  ○ No included studies | \| Outcome \| Importance \| **Certainty of the evidence** \| \| --- \| --- \| --- \| \| Survival (longest observation period) \| Critical \| ⨁⨁◯◯  Low \| \| QoL (EQ-5D) \| Critical \| ⨁⨁◯◯  low \| \| VFD (follow up: 28 days) \| Critical \| ⨁⨁◯◯  low \| \| Barotrauma \| Critical \| ⨁⨁◯◯  low \| \| ICU-AW \| Critical \| ⨁◯◯◯  Very low \|   **Overall certainty of the evidence:**  The direction within the desired effect was not consistent, and the certainty of evidence across outcomes was judged to be “very low,” adopting the certainty of the least certain evidence. |  |
| **Values**  Is there important uncertainty about or variability in how much people value the main outcomes? | | |
| **Judgment** | **Research Evidence** | ADDITIONAL CONSIDERATIONS |
| ○Important uncertainty or variability  ○ Possibly important uncertainty or variability  ● Probably no important uncertainty or variability   ○ No important uncertainty or variability | Maybe no significant uncertainty or diversity. |  |
| **Balance of effects**  Does the balance between desirable and undesirable effects favor the intervention or the comparison? | | |
| **Judgment** | **Research Evidence** | ADDITIONAL CONSIDERATIONS |
| ○ Favors the comparison  ○Probably favors the comparison  ○ Does not favor either the intervention or the comparison  ● Probably favors the intervention  ○Favors the intervention  ○ Varies  ○ Do not know | \| Outcome \| Comparison \| Intervention \| Absolute difference \| Relative effect (95% CI) \| \| --- \| --- \| --- \| --- \| --- \| \| Survival (longest observation period) \| 406/724 (56.1%) \| 445/737 (60.4%) \| +84 per 1000 patients (-39～+236) \| 1.15 (0.93〜1.42) \| \| QoL (EQ-5D) \| - \| - \| Average -0.02 point (-0.09～+0.05) \|  \| \| VFD (28 days) \| - \| - \| Average +0.72 days (-0.52～+1.95) \|  \| \| Barotrauma \| 51/713 (7.2%) \| 29/724 (4.0%) \|  \| 0.56 (0.36〜0.87) \| \| ICU-AW \| 151/363 (41.6%) \| 180/384 (46.9%) \|  \| 1.06 (0.86〜 1.30) \|   As a result, we judged that the effect of the intervention would probably be greater than the harm. |  |
| **Acceptability**  Is the intervention acceptable to key stakeholders? | | |
| **Judgment** | **Research Evidence** | ADDITIONAL CONSIDERATIONS |
| ○ No  ○ Probably no  ● Probably yes  ○ Yes  ○ Varies  ○ Do not know | There is no evidence used for the study, but it is already being done in regular practice and seems feasible considering the cost and other factors. |  |
| **Feasibility**  Is the intervention feasible to implement? | | |
| **Judgment** | **Research Evidence** | ADDITIONAL CONSIDERATIONS |
| ○ No  ○ Probably no  ○ Probably yes  ● Yes  ○ Varies  ○ Do not know | There is no evidence used for the study, but it has already been implemented in regular practice and seems feasible. |  |

**Summary of Judgment**

|  | Judgment | | | | | | |
| --- | --- | --- | --- | --- | --- | --- | --- |
| **PROBLEM** | No | Probably no | Probably yes | Yes |  | Varies | Do not know |
| **DESIRABLE EFFECTS** | Trivial | Low | Moderate | Large |  | Varies | Do not know |
| **UNDESIRABLE EFFECTS** | Large | Moderate | Small | Trivial |  | Varies | Do not know |
| **CERTAINTY OF EVIDENCE** | Very low | Low | Middle | High |  |  | No studies |
| **VALUES** | Important uncertainty or variability | Possibly important uncertainty or variability | Probably no important uncertainty or variability | No Important uncertainty or variability |  |  |  |
| **BALANCE OF EFFECTS** | Favors the comparison | Probably favors the comparison | Does not favor either the intervention or the comparison | Probably favors the intervention | Favors the intervention | Varies | Do not know |
| **ACCEPTABILITY** | No | Probably No | Probably yes | Yes |  | Varies | Do not know |
| **FEASIBILITY** | No | Probably no | Probably yes | Yes |  | Varies | Do not know |

**Type of Recommendation**

| Strong recommendation against the intervention | Conditional recommendation against the intervention | Conditional recommendation for either the intervention or the comparison | Conditional recommendation for the intervention | Strong recommendation for the intervention |
| --- | --- | --- | --- | --- |
| ○ | ○ | ○ | ● | ○ |

**Conclusions**

| **Recommendation** |
| --- |
| **Conditional recommendation for the early administration of muscle relaxants in adult patients with moderate or severe ARDS (weak recommendation/very low certainty evidence: GRADE2D).**    **Note：**  **They should be used in patients with moderate-to-severe ARDS, early in the course of the disease, and for a limited duration of administration (48 h or less). Non-depolarizing muscle relaxants are classified into aminosteroids (rocuronium, vecuronium, pancuronium) and benzylisoquinolines (atracurium, cis-atracurium, mivacurium). It should be noted that the drug used in the overseas RCT (cis-atracurium) is not marketed in Japan. The alternatives to cis-atracurium in Japan include rocuronium and vecuronium, which are aminosteroid muscle relaxants. Caution should be exercised in the use of aminosteroids, as their metabolism is prolonged in hepatic and renal disorders, and muscle atrophy may increase the risk of ICU-related muscle weakness. Particular attention should be paid to the concomitant use of steroids.** |
|  |
| **Justification** |
| **Question：**Should neuromuscular blockers be used at an early phase in adult patients with moderate or severe ARDS?  **Patient：** Adult patients with ARDS (moderate or severe)  **Intervention：** Use of muscle relaxants (any drug) within 48 h of onset  **Comparison：** No use of muscle relaxants  **Summary of evidence：**  A systematic review resulted in five RCTs that met PICO, and a meta-analysis was performed using these studies. Effect estimates for survival (five RCTs: N=1461) showed a risk difference of 84 more people/1000 (95% CI: 39 fewer people to 236 more people) with muscle relaxants compared to no muscle relaxants, and a mean difference of 0.02 points for QoL (EQ-5D;1 RCT: N=246; CI: decrease of 0.09 points to increase of 0.05 points), ventilator-free days (28 days; 5 RCTs: N=1461) increased by a mean difference of 0.72 days (95% CI: decrease of 0.52 days to increase of 1.95 days), and barotrauma (4 RCTs: N=1437) decreased by a risk difference of 31 persons/1000 people (95% CI: decrease of 46 persons to decrease of 9 persons). Pneumothorax was considered an important outcome. For pneumothorax (3 RCTs: N=1401), the risk difference was 35 fewer patients/1000 (95% CI: 47 to 15 fewer patients). Thus, the desired effect of the intervention was judged to be “moderate.” As an outcome of harm, the effect estimates for ICU-AW (4 RCTs: N=747) was a risk difference of 25 more people/1000 people (95% CI: 58 fewer people to 125 more people) with the use of muscle relaxants compared to no muscle relaxants (95% CI: 58 fewer to 125 more). The expected harm was judged to be “small.”  Thus, whether the effect of the intervention was greater than the harm was judged to be “probably in favor of the intervention.”  **Certainty of the evidence：**  　The direction within the desired effect was not consistent, and the certainty of evidence across outcomes was judged to be “very low,” adopting the certainty of the least certain evidence.  **Values, balance of effect, acceptability, feasibility：**  The typical non-depolarizing muscle relaxant in Japan is rocuronium, which costs less than several thousand yen per day for continuous administration. Muscle relaxants are easy to obtain and use, and there is no need to purchase additional equipment or secure personnel, so costs are likely to be minimal. It should be noted that the drug mainly used in the overseas RCT (cis-atracurium) is not available in Japan. With regard to resource utilization, when muscle relaxants are administered, the cost is acceptable, and the benefit outweighs the harm, which is of high importance because it is minimal.  Concerning the equity of medical resources, there is no evidence used in the study, but there is no concern about the distribution of medical resources, and the medical treatment is within the scope of usual care.  As for feasibility, there is no evidence used in the study, but it has already been implemented in regular practice, and it is considered feasible in consideration of cost and other factors.  **Panel meeting：**  In the pre-vote, the modified Delphi method resulted in a median score of 8.0, with a disagreement index of 0.292, for “Conditionally recommend early use of muscle relaxants in adults with moderate or severe ARDS.”  The panel discussed the usefulness of monitoring muscle relaxation during the use of muscle relaxants in patients with ARDS, but there were no specific objections to the draft recommendation. As a result, the panel meeting finally reached a consensus with the results of the pre-vote without a re-vote being required.    **Note：**  In the landmark trials, Papazian 2010 (ACURASYS Trial) 3) and The National Heart, Lung and Blood Institute PETAL Clinical Trials Network 2019 (ROSE Trial) 4) continuous muscle relaxant (cis-atracurium) administration limited to 48 h in patients with ARDS with a PaO_2_/FIO2 (P/F ratio) of 150 or less was used. Therefore, it was decided that the duration of administration should be limited to 48 h. |

| **Subgroup considerations** |
| --- |
| As a subgroup, it is necessary to examine the severity of oxygenation impairment (mild, moderate, and severe) in the Berlin definition of ARDS and the level of sedation (light sedation vs. deep sedation) in the control group. The difference in results between the ACURASYS Trial 3) and the ROSE Trial 4) may be due to the difference in sedation level in the control group. |
| **Implementation considerations** |
| It is important to note that most studies reviewed in this systematic review used a PF ratio of <150 mmHg as an inclusion criterion, which is different from the AECC criteria and the Berlin definition of impaired oxygenation in ARDS.  Non-depolarizing muscle relaxants are classified into aminosteroids (rocuronium, vecuronium, pancuronium) and benzylisoquinolines (atracurium, cis-atracurium, mivacurium). It should be noted that the drug mainly used in the overseas RCT (cis-atracurium) is not available in Japan. Alternatives to cis-atracurium in Japan include rocuronium and vecuronium, which are aminosteroid muscle relaxants. Caution should be exercised in the use of aminosteroids because of their prolonged metabolism in hepatic and renal impairment and their muscle atrophy effects.  The 2016 Japanese ARDS guideline recommendation 6) was to “suggest the limited use of muscle relaxants when performing ventilation in adult patients with ARDS (GRADE 2B, weak recommendation/moderate certainty evidence).” Subsequently, the ROSE trial 4) published in 2019 enrolled 1,006 patients with moderate or severe ARDS (Berlin definition), the largest to date, and examined the efficacy of continuous muscle relaxant (cis-atracurium) administration. The results showed no improvement in mortality or other patient-important outcomes. In this review, the 2016 guidelines were UPDATED based on the results of the ROSE study. The Surviving Sepsis Campaign 2016 guideline 7) recommends “the use of muscle relaxants for 48 hours or less is recommended for adult patients with sepsis and ARDS and a PaO2/FIO2 ratio of <150 mmHg (weak recommendation/moderate certainty evidence).” The 2020 Rapid Practice Guideline for the use of muscle relaxants in patients with ARDS from the European Society of Intensive Care Medicine 8) found discrepancies between subgroup analyses at the sedation level, and based on these results, it is recommended that “adults with moderate or severe In adults with ARDS who require deep sedation and muscle relaxant administration to promote lung protective ventilation, it is recommended that muscle relaxants be used for up to 48 hours (weak recommendation/confidence of low evidence.” |

| **Monitoring and evaluation** |
| --- |
| When administering muscle relaxants to high-risk adult patients with ARDS, it is necessary to monitor the degree of muscle relaxation by providing adequate sedation to the patient (sedation monitoring) using sedation monitors such as the bispectral index while monitoring respiration and circulation. To measure the degree of muscle relaxation, a muscle relaxation monitor (train of four stimulation) can be used 8). In two large overseas RCTs 3-4), muscle relaxants (cis-atracurium) were administered continuously at fixed doses without the use of muscle relaxant monitors. Informed consent from the patient’s family is necessary for the administration of muscle relaxants.  Further information on adverse events of muscle relaxants needs to be collected before recommendations can be made. In addition, it is necessary to monitor the implementation status of the guideline through questionnaires, and other means after the guideline is published to see if there are any other clinical problems. |
| **Research priorities** |
| The effects of muscle relaxants on long-term outcomes in physical and cognitive function, the effects of other muscle relaxants, and the efficacy and safety of administration methods (partial muscle relaxation 9),10), intermittent administration, and continuous administration) need to be examined. |

References

1) Brochard L, Slutsky A, Pesenti A. Mechanical ventilation to minimize progression of lung injury in acute respiratory failure. Am J Respir Crit Care Med 2017;195:438-442. PMID 27626833.

2) Slutsky AS. Neuromuscular blocking agents in ARDS. N Engl J Med 2010;363:1176-80. PMID 20843254.

3) Papazian L, Forel J-M, Gacouin A, et al. Neuromuscular blockers in early acute respiratory distress syndrome. N Engl J Med 2010;363:1107-16. PMID 20843245.

4) National Heart, Lung and BIPCTN, Moss M, Huang DT, et al. Early neuromuscular blockade in the acute respiratory distress syndrome. N Engl J Med 2019;380:1997-2008. PMID 31112383.

5) Slutsky AS, Villar J. Early paralytic agents for ARDS? Yes, no, and sometimes. N Engl J Med 2019;380:2061-2063. PMID 31112382.

6) Hashimoto S, Sanui M, Egi M, et al. The clinical practice guideline for the management of ARDS in Japan. J intensive care 2017;5:50. PMID 28770093.

7) Rhodes A, Evans LE, Alhazzani W, et al. Surviving sepsis campaign: international guidelines for management of sepsis and septic shock: 2016. Intensive Care Med 2017;43:304-377. PMID 28101605.

8) Alhazzani W, Belley-Cote E, Møller MH, et al. Neuromuscular blockade in patients with ARDS: a rapid practice guideline. Intensive Care Med 2020;46:1977-1986. PMID 33104824.

9) Hraiech S, Yoshida T, Annane D, et al. Myorelaxants in ARDS patients. Intensive Care Med 2020;46:2357-2372. PMID 33159530.

10) Doorduin J, Nollet JL, Roesthuis LH, et al. Partial neuromuscular blockade during partial ventilatory support in sedated patients with high tidal volumes. Am J Respir Crit Care Med 2017;195:1033-1042. PMID 27748627.

**CQ32 Should transpulmonary pressure be used when setting PEEP in patients with ARDS?**

1. Search strategy

MEDLINE via PubMed （Search date: 2020/6/4）

| #1 | Respiratory Distress Syndrome, Adult [mh] |
| --- | --- |
| #2 | Acute lung injury [mh] |
| #3 | ALI [tiab] OR ARDS [tiab] |
| #4 | Acute lung injur* [tiab] OR acute respiratory distress [tiab] OR acute respiratory failure[tiab] |
| #5 | (Severe[tiab] OR critical*[tiab]) AND (respiratory[tiab] OR hypox* [tiab]) |
| #6 | “shock lung”[tiab] |
| #7 | #1 OR #2 OR #3 OR #4 OR #5 OR #6 |
| #8 | “omega 3 fatty acids”[tiab] |
| #9 | “n 3 fatty acids” [tiab] |
| #10 | "fatty acids, unsaturated"[mesh] |
| #11 | "unsaturated fatty acids"[tiab] |
| #12 | “linolenic acid”[tiab] |
| #13 | “Fish Oils”[mesh] |
| #14 | “fish oil”[tiab] |
| #15 | #8 OR #9 OR #10 OR #11 OR #12 OR #13 OR #14 |
| #16 | #7 AND #15 |
| #17 | Controlled clinical trial[Publication Type] |
| #18 | randomized[Title/Abstract] |
| #19 | placebo[Title/Abstract] |
| #20 | randomly [tiab] |
| #21 | trial[tiab] |
| #22 | groups [tiab] |
| #23 | "systematic review"[Publication Type] |
| #24 | "meta-analysis"[Publication Type] |
| #25 | "review"[Publication Type] |
| #26 | #17 OR #18 OR #19 OR #20 OR #21 OR #22 OR #23 OR #24 OR #25 |
| #27 | #16 AND #26 |
| #28 | animals [mh] NOT humans [mh] |
| #29 | #27 NOT #28 |

CENTRAL（Search date: 2020/6/4）

| #1 | [mh "Respiratory Distress Syndrome, Adult"] |
| --- | --- |
| #2 | [mh "Acute lung injury"] |
| #3 | ALI:ti,ab OR ARDS:ti,ab |
| #4 | (Acute NEXT lung NEXT injur*:ti,ab) OR "acute respiratory distress":ti,ab OR "acute respiratory failure":ti,ab |
| #5 | (Severe:ti,ab OR critical*:ti,ab) AND (respiratory:ti,ab OR hypox*:ti,ab) |
| #6 | "shock lung":ti,ab |
| #7 | {OR #1-#6} |
| #8 | "omega 3 fatty acids":ti,ab |
| #9 | "n 3 fatty acids":ti,ab |
| #10 | [mh "fatty acids, unsaturated"] |
| #11 | "unsaturated fatty acids":ti,ab |
| #12 | "linolenic acid":ti,ab |
| #13 | [mh "Fish Oils"] |
| #14 | “fish oil”:ti,ab |
| #15 | {OR #8-#14} |
| #16 | #7 AND #15 |
| #17 | [mh animals] NOT [mh humans] |
| #18 | #16 NOT #17 |

1. Flow diagram

**Identification**

10 Studies included in qualitative synthesis

30 Full-text articles assessed for eligibility

1185 records after duplicates removed

1293 records identified through database searching

1293 records identified through database searching

Medline via PubMed (n=774)

CENTRAL (n=431)

Igaku-Chuo-Zasshi (n=88)

0 additional records identified through other sources

10 Studies included in quantitative synthesis (meta-analysis)

20 Full-text articles excluded, with reasons:

・Wrong language (n=4)

・Wrong publication type (n=7)

・Wrong population (n=2)

・Wrong intervention (n=7)

Etc.

Duplicates

n=106

1155 records excluded

**Included**

**Eligibility**

**Screening**

1. Risk of bias

Short-term mortality Long-term mortality


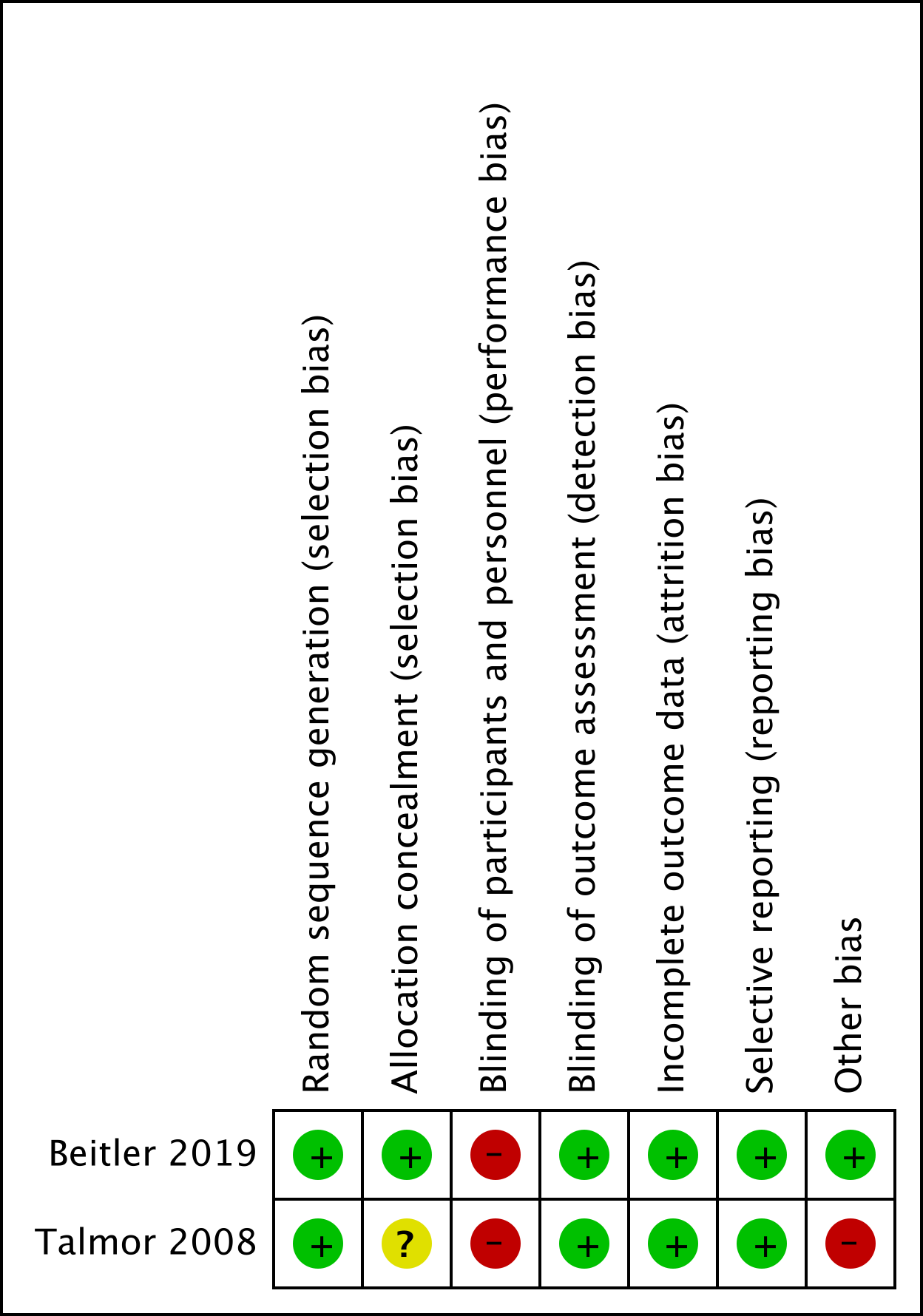

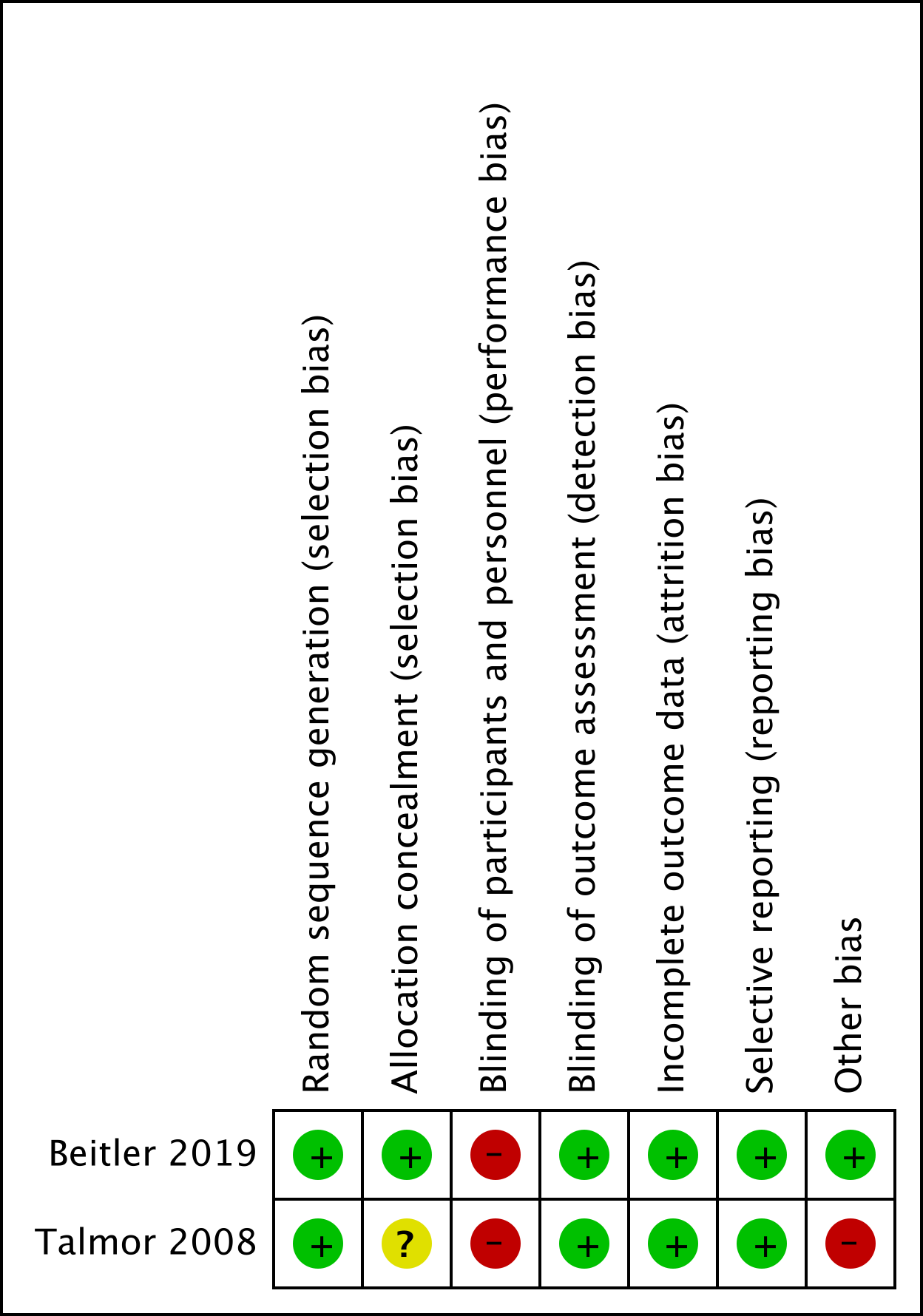


Ventilator-free days P/F ratio


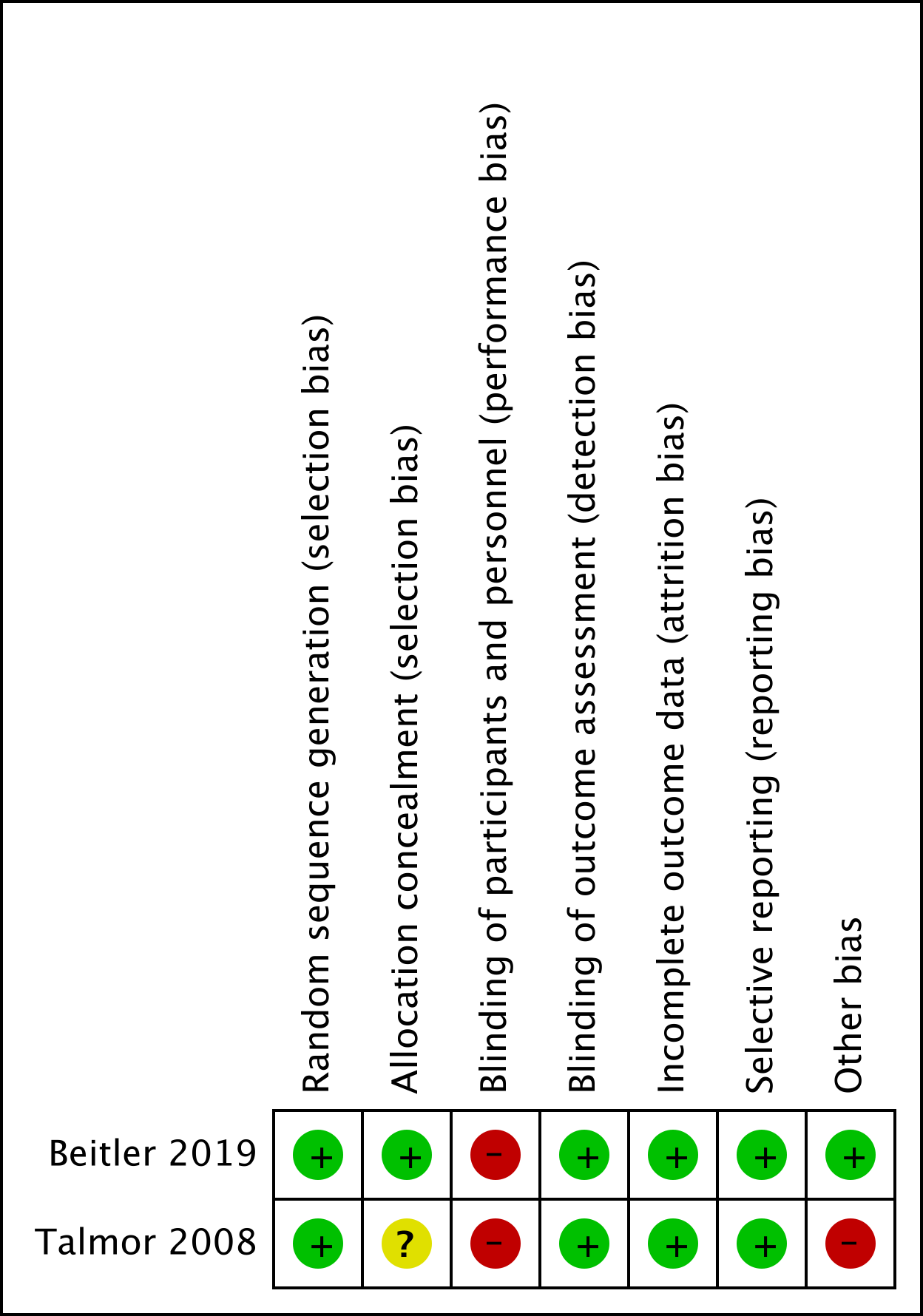

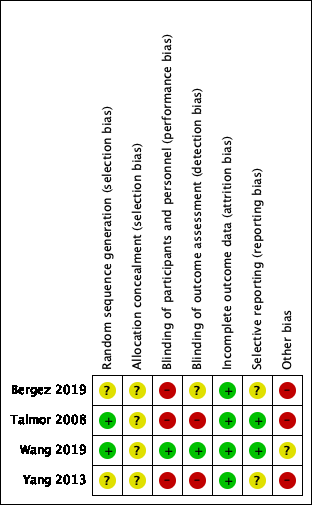


Lung compliance


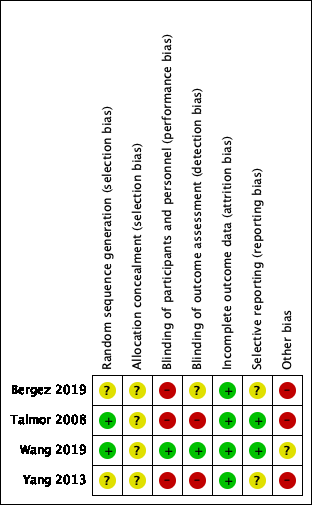


1. Forest plot

Short-term mortality


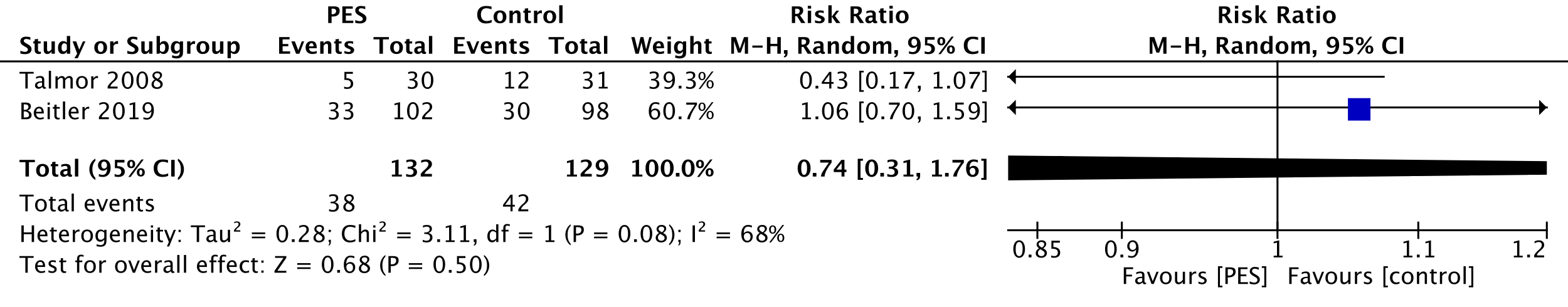


Long-term mortality


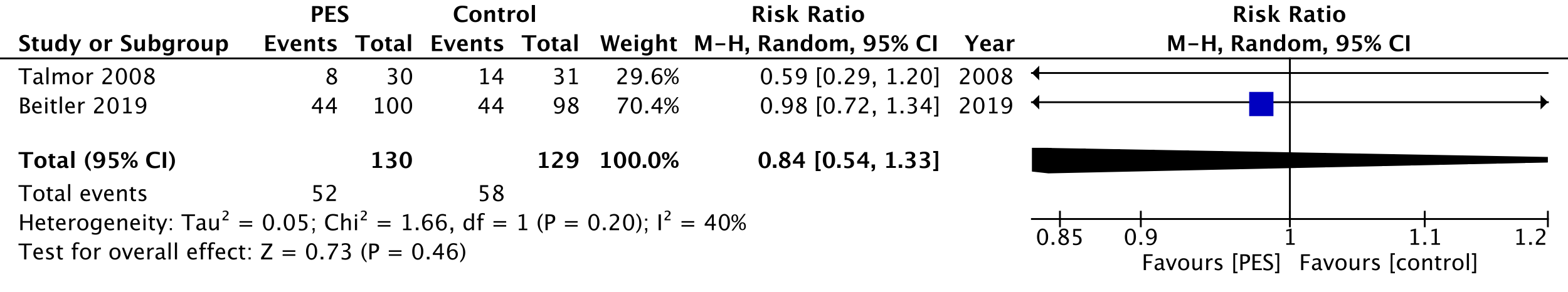


Ventilator-free days


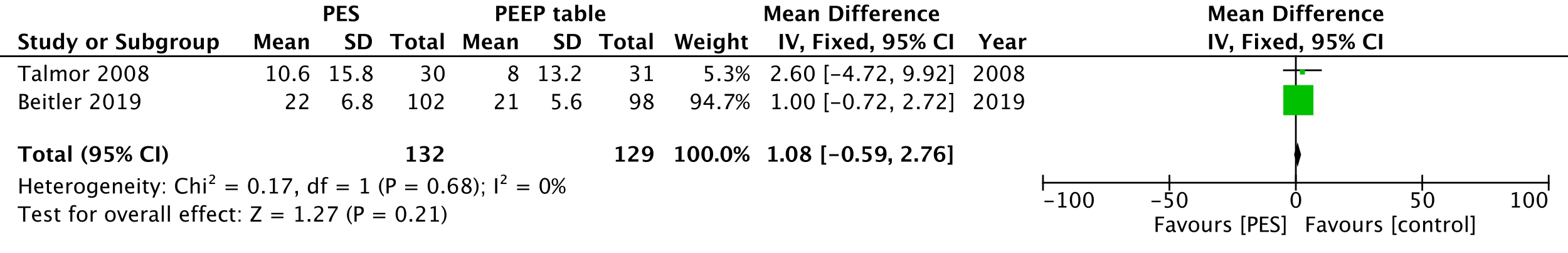


P/F ratio


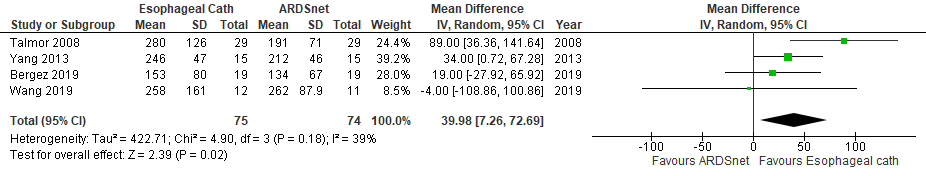


Lung compliance


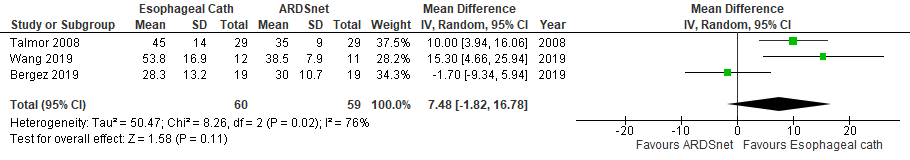


1. Evidence profile

| Assessment of certainty | | | | | | | No. of patients | | **Efficacy** | | Certainty of the evidence | **Importance** |
| --- | --- | --- | --- | --- | --- | --- | --- | --- | --- | --- | --- | --- |
| No. of studies | Study design | Risk of bias | Indirectness | Inconsistency | Imprecision | **Others** | **PES** | **Placebo** | **Relative index (95% CI)** | **Absolute index (95% CI)** |  |  |
| Short-term mortality | | | | | | | | | | | | |
| 2 | RCT | Not Serious | Serious^a^ | Not Serious | Serious^b^ | None | 38/132 (28.8%) | 42/129 (32.6%) | RR 0.74 (0.31〜1.76) | -85 per 1000 patients (-225〜+247) | ⨁⨁◯◯  low | Critical |
| Long-term mortality | | | | | | | | | | | | |
| 2 | RCT | Not Serious | Not Serious | Not Serious | Serious^b^ | None | 52/130 (40.0%) | 58/129 (45.0%) | RR (0.54〜1.33) | -72 per 1000 patients (-207〜+148) | ⨁⨁⨁◯ moderate | Critical |
| **Ventilator-Free-Days (at 28 days)** | | | | | | | | | | | | |
| 2 | RCT | Serious^c^ | Not Serious | Not Serious | Serious^b^ | None | 132 | 129 | - | Average 1.08 long (-0.59〜+2.76) | ⨁⨁◯◯  low | Important |
| **P/F ratio** | | | | | | | | | | | | |
| 4 | RCT | Serious^d^ | Serious^e^ | Serious^f^ | Serious^g^ | Strong correlation | 75 | 74 | - | Average 39.98 high (+7.26〜+72.69) | ⨁◯◯◯  Very low | Important |
| **Lung Compliance** | | | | | | | | | | | | |
| 3 | RCT | Serious^d^ | Serious^e^ | Serious^f^ | Serious^g^ | Strong correlation | 60 | 59 | - | Average 7.48 high (+1.82〜+16.78) | ⨁◯◯◯  Very low | Important |
| **ICU days** | | | | | | | | | | | | |
| - | - | - | - | - | ^-^ | - | - | - | Impossible to estimate | - | - | Important |
| **Hospital days** | | | | | | | | | | | | |
| - | - | - | - | - | ^--^ | - |  | - | Impossible to estimate | - | - | Important |

PES: esophageal pressure; RCT: Randomized controlled trial; ICU: Intensive care unit; CI: Confidence interval; RR: Relative risk

ICU duration and hospitalization duration are not included in the evidence profile because the outcomes are not reported.

a. I^2^ value ≒71%, which is a large heterogeneity and one grade down.

b. Because practice behavior is likely to differ when the lower vs. upper limits of the 95% confidence interval are true

c. Risk of bias because two studies were poorly blinded and one study was block randomized

d. Unclear whether the raters were blinded

e. Not completely consistent

f. One study included patients with elevated intra-abdominal pressure

g. Small sample size

h. An investigator can be changed

1. Evidence-to-Decision table

| **Question** | |
| --- | --- |
| **CQ32** Should transpulmonary pressure be used when setting PEEP in patients with ARDS? | |
| Population: | Adult patients suffering from hypoxemia (PaO_2_/F_I_O_2_ ratio ≤ 300 mmHg or equivalent) |
| **Intervention:** | PEEP determination using transpulmonary pressure |
| **Comparison:** | PEEP determination according to the ARDSnet table, PEEP determined by a physician |
| **Primary Outcome:** | Short term mortality, long term mortality |
| **Setting:** | Situation equivalent to the emergency room or intensive care unit |
| **Perspective:** | Individual |
| **Background:** | Excessive PEEP enhances ventilator-induced lung injury (VILI) in patients with ARDS and adversely affects circulatory dynamics. Insufficient PEEP increases collapsed lung, which has also been reported to affect VILI and worsen oxygenation. On the contrary, there is no established method for appropriate PEEP settings.  Clarification of useful methods for PEEP setting is an important clinical issue. |
| **Conflict of interests:** | None |

**Assessment**

| **Problem**  Is the problem a priority? | | |
| --- | --- | --- |
| **Judgment** | **Research Evidence** | **ADDITIONAL CONSIDERATIONS** |
| ○ No  ○ Probably no  ○ Probably yes  ● Yes  ○ Varies  ○ Do not know | In patients with ARDS, excessive PEEP increases the risk of VALI and has a negative impact on circulatory dynamics. Too low PEEP has also been reported to increase collapsed lung, affect VILI, and worsen oxygenation. In contrast, there is no established method for determining appropriate PEEP, and identifying a useful method for setting PEEP is an important clinical issue. Therefore, the priority of this issue is probably high. |  |
| **Desirable effects**  How substantial are the desirable anticipated effects? | | |
| **Judgment** | **Research Evidence** | ADDITIONAL CONSIDERATIONS |
| ○ Trivial  ● Small  ○ Moderate  ○ Large  ○ Varies  ○ Do not Know | A systematic review identified two RCTs consistent with the patient, intervention, comparison, and outcome process, and a meta-analysis was performed using these trials. The effect estimates for short term mortality (2 RCTs: N=261) was 85 fewer deaths/1000 people (95% CI: 225 fewer deaths to 247 more deaths) for the intervention compared to the control. 〜The estimated effect on long term mortality (2 RCTs: N=259) was a reduction of 72/1000 (95% CI: 207 to 148). Thus, we judged the desired effect of the intervention to be “small.” |  |
| **Undesirable effects**  How substantial are the undesirable anticipated effects? | | |
| **Judgment** | **Research Evidence** | ADDITIONAL CONSIDERATIONS |
| ○ Large  ○ Small  ● Moderate  ○ Trivial  ○ Varies  ○ Do not know | Harm has only been examined in one RCT (EPVent 2 study) and was not examined in the meta-analysis. Therefore, the expected harm was judged to be “small” when considered in conjunction with the description in the remarks section. | In the EPVent 2 study, there was no increase in the frequency of pneumothorax or pressure trauma in the “intervention” group compared to the “control” group (pneumothorax 2/98 vs. 3/102, pressure trauma 5/98 vs. 6/102). In addition, there were no complications associated with esophageal balloon placement in the intervention group. |
| **Certainty of evidence**  What is the overall certainty of the evidence of effects? | | |
| **Judgment** | **Research Evidence** | ADDITIONAL CONSIDERATIONS |
| ○ Very low  ○ Low  ● Moderate  ○ High  ○ No included studies | \| Outcome \| Importance \| **Certainty of the evidence** \| \| --- \| --- \| --- \| \| Short-term mortality \| Critical \| ⨁⨁◯◯  Low \| \| Long-term mortality \| Critical \| ⨁⨁⨁◯  moderate \|   **Overall certainty of the evidence:**  The direction of the outcome was consistent, and the certainty of the evidence across the outcome was judged to be “medium,” using the lowest certainty of the evidence. |  |
| **Values**  Is there important uncertainty about or variability in how much people value the main outcomes? | | |
| **Judgment** | **Research Evidence** | ADDITIONAL CONSIDERATIONS |
| ○Important uncertainty or variability  ○ Possibly important uncertainty or variability  ● Probably no important uncertainty or variability   ○ No important uncertainty or variability | Maybe no significant uncertainty or diversity. |  |
| **Balance of effects**  Does the balance between desirable and undesirable effects favor the intervention or the comparison? | | |
| **Judgment** | **Research Evidence** | ADDITIONAL CONSIDERATIONS |
| ○ Favors the comparison  ○Probably favors the comparison  ○ Does not favor either the intervention or the comparison  ○ Probably favors the intervention  ○Favors the intervention  ○ Varies  ● Do not know | \| Outcome \| Comparison \| Intervention \| Absolute difference \| Relative effect (95% CI) \| \| --- \| --- \| --- \| --- \| --- \| \| Short-term mortality \| 42/129 (32.6%) \| 38/132 (28.8%) \| +85 per 1000 patients (-225～+247) \| 0.74 (0.31〜1.76) \| \| Long-term mortality \| 58/129 (45.0%) \| 52/130 (40.0%) \| -72 per 1000 patients (-207～+148) \| 0.84 (0.54〜1.33) \|   Usual methods*: PEEP determined according to ARDSnet tables, PEEP determined by a physician  Thus, we judged that we do not know if the effect of the intervention is greater than the harm. |  |
| **Acceptability**  Is the intervention acceptable to key stakeholders? | | |
| **Judgment** | **Research Evidence** | ADDITIONAL CONSIDERATIONS |
| ○ No  ○ Probably no  ○ Probably yes  ○ Yes  ○ Varies  ● Do not know | There are concerns about the acceptance of the project by key stakeholders because of the special medical equipment required. |  |
| **Feasibility**  Is the intervention feasible to implement? | | |
| **Judgment** | **Research Evidence** | ADDITIONAL CONSIDERATIONS |
| ○ No  ● Probably no  ○ Probably yes  ○ Yes  ○ Varies  ○ Do not know | Since it requires special medical equipment and materials, it may be practically difficult to perform in some facilities. |  |

**Summary of Judgment**

|  | Judgment | | | | | | |
| --- | --- | --- | --- | --- | --- | --- | --- |
| **PROBLEM** | No | Probably no | Probably yes | Yes |  | Varies | Do not know |
| **DESIRABLE EFFECTS** | Trivial | Low | moderate | Large |  | Varies | Do not know |
| **UNDESIRABLE EFFECTS** | Large | Moderate | Low | Trivial |  | Varies | Do not know |
| **CERTAINTY OF EVIDENCE** | Very low | Low | Middle | High |  |  | No studies |
| **VALUES** | Important uncertainty or variability | Possibly important uncertainty or variability | Probably no important uncertainty or variability | No Important uncertainty or variability |  |  |  |
| **BALANCE OF EFFECTS** | Favors the comparison | Probably favors the comparison | Does not favor either the intervention or the comparison | Probably favors the intervention | Favors the intervention | Varies | Do not know |
| **ACCEPTABILITY** | No | Probably No | Probably yes | Yes |  | Varies | Do not know |
| **FEASIBILITY** | No | Probably no | Probably yes | Yes |  | Varies | Do not know |

**Type of Recommendation**

| Strong recommendation against the intervention | Conditional recommendation against the intervention | Conditional recommendation for either the intervention or the comparison | Conditional recommendation for the intervention | Strong recommendation for the intervention |
| --- | --- | --- | --- | --- |
| ○ | ● | ○ | ○ | ○ |

**Conclusions**

| **Recommendation** |
| --- |
| **Conditional recommendation not to use transpulmonary pressure routinely for setting PEEP in patients with ARDS (conditional recommendation/moderate certainty evidence: GRADE 2B).**    **Note：**  The results of the systematic review showed no significant difference, but there was a trend toward a reduction in 28-day mortality and long-term mortality. However, since the esophageal pressure balloon kit for measurement is currently not available in Japan unless the patient has a specific ventilator, it is difficult to recommend routine measurement. This does not preclude the use of esophageal pressure balloon kits in situations where they are available. |
|  |
| **Justification** |
| **Question：** Should transpulmonary pressure be used when setting PEEP in patients with ARDS?  **Patient：** Patients over 18 years of age with hypoxic respiratory failure requiring a ventilator  **Intervention：** Method for determining PEEP using transpulmonary pressure  **Comparison：** PEEP determination according to the ARDSnet table, PEEP determined by a physician  **Outcome:** 28-day mortality, long-term mortality  **Summary of evidence：**  A systematic review resulted in a meta-analysis of two RCTs (EPVent 1 study^1)^ and EPVent 2 study^2)^ comparing PEEP setting by transpulmonary pressure versus the traditional ARDSnet table PEEP determination method in patients with ARDS. We conducted a meta-analysis using these studies. The estimated effect on short-term mortality (two RCTs: N=261) was a reduction in risk of 85 deaths per 1000 people (95% CI: 225 to 247) for the intervention compared with the control. 〜The estimated effect on long term mortality (2 RCTs: N=259) was a reduction of 72 patients/1000 (95% CI: 207 to 148). Thus, we judged the desired effect of the intervention to be “small.” The harm was only examined in one RCT (EPVent 2 study) and was not examined in the meta-analysis. In the EPVent 2 study, the frequency of pneumothorax and pressure trauma did not increase in the “intervention” compared to the “control” (pneumothorax 2/98 vs. 3/102 absolute difference 0.9% (-3.4% to 5.2%), pressure 5/98 vs. 6/102 absolute difference 0.8% (-5.5% to 7.1%)), and there were no complications associated with esophageal balloon implantation in the intervention group. Therefore, the undesirable effect of the intervention was judged to be “small.” Therefore, it was judged that the undesirable effect of the intervention was “small” and the effect of the intervention was “unknown.”  **Certainty of the evidence：**  The directionality of the outcomes was consistent, and the certainty of the evidence across the outcomes was judged to be “medium,” adopting the certainty of the evidence with the lowest certainty.  **Values, balance of effect, acceptability, feasibility：**  An esophageal pressure balloon kit is necessary to measure transpulmonary pressure, which is currently not available in Japan unless the patient has a specific ventilator and is difficult to perform in some facilities due to cost concerns.  **Panel meeting：**  According to the modified Delphi method in the pre-vote, the recommendation that “transpulmonary pressure not be used routinely for setting PEEP in patients with ARDS” had a median score of 8.0 and a disagreement index of 0.1316.  At the panel meeting, there was a discussion on whether to recommend or deprecate the use of transpulmonary pressure, as the SR results were only in the beneficial direction.  As a result, a re-vote was held at the panel meeting, resulting in a median score of 8.0 and a disagreement index of 0.2920, and it was finally agreed to remain deprecated as in the pre-vote results. |

| **Subgroup considerations** |
| --- |
| The transpulmonary pressure is calculated as the difference between the intrapulmonary airway pressure measured by the ventilator and the intrathoracic pressure (approximated from the esophageal pressure measured by the esophageal pressure balloon kit). Normally, PEEP is set not to exceed the upper limit of airway pressure (end-inspiratory plateau pressure less than 30 cmH2O). However, in patients with high intrathoracic pressure, even when the upper limit of airway pressure is reached (highly obese, abdominal compartment syndrome), transpulmonary pressure during inspiration is not expected to be so high. Using transpulmonary pressure monitoring makes it possible to set a higher PEEP in patients with high intrathoracic pressure without being limited by airway pressure. A subgroup analysis could not be performed in this study but will be considered in the future. |
| **Implementation considerations** |
| The esophageal manometry balloon kit must be used correctly to accurately measure transpulmonary pressure. In particular, the position of the esophageal pressure balloon and the amount of air injected into the balloon may affect the measured values of esophageal pressure, requiring knowledge and skills for correct use. In addition, since intrathoracic pressure is not uniform in the thoracic cavity, it is necessary to consider whether the intrathoracic pressure approximated by the measured esophageal pressure reflects the pressure in any part of the thoracic cavity. Yoshida et al. 3) reported that the transpulmonary pressure calculated by esophageal pressure reflects the transpulmonary pressure in the middle to slightly dorsal part of the thoracic cavity. |

| **Monitoring and evaluation** |
| --- |
| Because of the special medical equipment required, the number of facilities where it can be performed is limited. It is necessary to consider the cost and the fact that it may be practically difficult to perform the procedure in some facilities, and further information on adverse events and cost-effectiveness should be collected before implementing the recommendations. The implementation status needs to be monitored through the use of questionnaires after the guidelines are published to ensure that there are no other clinical problems. |
| **Research priorities** |
| As mentioned above, studies are expected to investigate the usefulness of transpulmonary pressure monitoring in patients with high intrathoracic pressure. In addition to the PEEP setting, esophageal pressure and transpulmonary pressure monitoring can be used to observe the intensity of inspiratory effort under spontaneous breathing. Future studies are also expected to investigate the usefulness of this monitoring. |

References

1) [Talmor](https://pubmed.ncbi.nlm.nih.gov/?term=Talmor+D&cauthor_id=19001507) D, [Sarge](https://pubmed.ncbi.nlm.nih.gov/?term=Sarge+T&cauthor_id=19001507) T, [Malhotra](https://pubmed.ncbi.nlm.nih.gov/?term=Malhotra+A&cauthor_id=19001507) A, et al. Mechanical ventilation guided by esophageal pressure in acute lung injury. N Engl J Med. 2008;359(20):2095-104.

2) [Beitler](https://pubmed.ncbi.nlm.nih.gov/?term=Beitler+JR&cauthor_id=30776290) JR, [Sarge](https://pubmed.ncbi.nlm.nih.gov/?term=Sarge+T&cauthor_id=30776290) T, B[anner-Goodspeed](https://pubmed.ncbi.nlm.nih.gov/?term=Banner-Goodspeed+VM&cauthor_id=30776290) VM, et al. Effect of titrating positive end-expiratory pressure (PEEP) with an esophageal pressure-guided strategy vs an empirical high PEEP-FIO2 strategy on death and days free from mechanical ventilation among patients with acute respiratory distress syndrome: a randomized clinical trial. JAMA. 2019;321(9):846-857.

3)[Yoshida](https://pubmed.ncbi.nlm.nih.gov/?term=Yoshida+T&cauthor_id=29323931) T,  [Amato](https://pubmed.ncbi.nlm.nih.gov/?term=Amato+MBP&cauthor_id=29323931) MBP,  [Grieco](https://pubmed.ncbi.nlm.nih.gov/?term=Grieco+DL&cauthor_id=29323931) DL, et al. Esophageal manometry and regional transpulmonary pressure in lung injury. Am J Respir Crit Care Med. 2018;197(8):1018-1026.

**CQ33 Should electrical impedance tomography (EIT) be used in PEEP settings for patients with ARDS?**

1. Search strategy

MEDLINE via PubMed （Search date: 2020/3/19）

| #1 | (((((((Respiratory Distress Syndrome, Adult[MeSH Terms]) OR Lung Injury[MeSH Terms]) OR Respiration, Artificial[MeSH Terms]) OR ARDS[Title/Abstract]) OR acute respiratory distress syndrome[Title/Abstract]) OR ALI[Title/Abstract]) OR acute lung injury[Title/Abstract]) OR ventilat*[Title/Abstract] |
| --- | --- |
| #2 | ((Electric Impedance[MeSH Terms]) OR electric impedance tomography[Title/Abstract]) OR EIT[Title/Abstract] |
| #3 | #1 AND #2 |

1. Flow diagram

**Identification**

0 Studies included in qualitative synthesis

5 Full-text articles assessed for eligibility

874 records after duplicates removed

922 records identified through database searching

922 records identified through database searching

Medline via PubMed (n=669)

CENTRAL (n=179)

Igaku-Chuo-Zasshi (n=74)

0 additional records identified through other sources

0 Studies included in quantitative synthesis (meta-analysis)

Duplicates

n=48

869 records excluded

**Included**

**Eligibility**

**Screening**

5 Full-text articles excluded, with reasons:

・Wrong study design (n=5)

Etc.

1. Risk of bias

Not applicable

1. Forest plot

Not applicable

1. Evidence profile

Not applicable

1. Evidence-to-Decision table

| **Question** | |
| --- | --- |
| **CQ33** Should electrical impedance tomography (EIT) be used in PEEP settings for patients with ARDS? | |
| Population: | Adult patients with ARDS |
| **Intervention:** | PEEP determination using EIT |
| **Comparison:** | PEEP determination according to the ARDSnet table, PEEP determined by a physician |
| **Primary Outcome:** | Mortality rate, VALI |
| **Setting:** | Situation equivalent to the emergency room or intensive care unit |
| **Perspective:** | Individual |
| **Background:** | In patients with ARDS, excessive PEEP enhances VILI and adversely affects circulatory dynamics. Insufficient PEEP increases the risk of collapsed lung, which has also been reported to affect VILI and worsen oxygenation. On the contrary, there is no established method for establishing an appropriate PEEP setting.  Clarification of useful methods for establishing PEEP settings is an important clinical issue. |
| **Conflict of interests:** | None |

**Assessment**

| **Problem**  Is the problem a priority? | | |
| --- | --- | --- |
| **Judgment** | **Research Evidence** | **ADDITIONAL CONSIDERATIONS** |
| ○ No  ○ Probably no  ● Probably yes  ○ Yes  ○ Varies  ○ Do not know | In patients with ARDS, excessive PEEP causes VILI by hyperinflating the lungs, which also adversely affects circulatory dynamics. Too low PEEP has also been reported to increase the risk of a collapsed lung, affect VILI, and worsen oxygenation. On the contrary, there is no established method for determining appropriate PEEP, and identifying useful methods for setting PEEP is an important clinical issue. Therefore, the priority of this issue is probably high. |  |
| **Desirable effects**  How substantial are the desirable anticipated effects? | | |
| **Judgment** | **Research Evidence** | ADDITIONAL CONSIDERATIONS |
| ○ Trivial  ○ Small  ○ Moderate  ○ Large  ○ Varies  ● Do not Know | Integrated into the certainty of the evidence. |  |
| **Undesirable effects**  How substantial are the undesirable anticipated effects? | | |
| **Judgment** | **Research Evidence** | ADDITIONAL CONSIDERATIONS |
| ○ Large  ○ Small  ○ Moderate  ○ Trivial  ○ Varies  ● Do not know | Integrated into the certainty of the evidence. |  |
| **Certainty of evidence**  What is the overall certainty of the evidence of effects? | | |
| **Judgment** | **Research Evidence** | ADDITIONAL CONSIDERATIONS |
| ○ Very low  ○ Low  ○ Moderate  ○ High  ● No included studies | EIT is a device used for bedside imaging of gas distribution in the lungs by measuring impedance changes in the body. It is a non-invasive and continuous method to visualize the ventilation distribution in real-time, and it is possible to depict the ventilation status of each lung region in a cross-sectional image. In this systematic literature search, we found no studies that compared PEEP determination by EIT with other methods for critical outcomes.  In a prospective observational study by Zhao et al ^1)^, the method of PEEP determination by EIT was investigated in 24 patients with severe ARDS. We used EIT to find the PEEP with the most uniformly inflated lungs, where both the ventral lung hyperinflation area and dorsal collapse area were low, and set that PEEP as the PEEP set by EIT. The PEEP set by this technique was relatively high, averaging 17.6 cmH_2_O (±3.6), but no obvious pressure trauma occurred. Compared to previous severe ARDS cases at the same institution, higher PEEP and lower driving pressure were achieved.  From this study, it appears possible that EIT can depict the ventilation status of each lung region, and appropriate PEEP can be set by looking for PEEP with uniformly inflated lungs. However, PEEP determination by EIT has no established superior evidence over conventional PEEP determination. Therefore, it is not possible to evaluate the benefits and harms of this method. |  |
| **Values**  Is there important uncertainty about or variability in how much people value the main outcomes? | | |
| **Judgment** | **Research Evidence** | ADDITIONAL CONSIDERATIONS |
| ○Important uncertainty or variability  ○ Possibly important uncertainty or variability  ● Probably no important uncertainty or variability   ○ No important uncertainty or variability | Maybe no significant uncertainty or diversity. |  |
| **Balance of effects**  Does the balance between desirable and undesirable effects favor the intervention or the comparison? | | |
| **Judgment** | **Research Evidence** | ADDITIONAL CONSIDERATIONS |
| ○ Favors the comparison  ○Probably favors the comparison  ○ Does not favor either the intervention or the comparison  ○ Probably favors the intervention  ○Favors the intervention  ○ Varies  ● Do not know | No relevant studies. |  |
|  |  |  |
| **Acceptability**  Is the intervention acceptable to key stakeholders? | | |
| **Judgment** | **Research Evidence** | ADDITIONAL CONSIDERATIONS |
| ● No  ○ Probably no  ○ Probably yes  ○ Yes  ○ Varies  ○ Do not know | There is no evidence used for the study, but it is an experimental method and would not be feasible in daily clinical practice. |  |
| **Feasibility**  Is the intervention feasible to implement? | | |
| **Judgment** | **Research Evidence** | ADDITIONAL CONSIDERATIONS |
| ● No  ○ Probably no  ○ Probably yes  ○ Yes  ○ Varies  ○ Do not know | As of February 2021, EIT has not been released in Japan and is an experimental device. In addition, although EIT itself is non-invasive, there is no evidence to compare it with conventional PEEP determination methods, and PEEP determination by EIT may lead to the setting of excessive PEEP. Therefore, the feasibility of EIT is considered to be difficult. |  |

**Summary of Judgment**

|  | Judgment | | | | | | |
| --- | --- | --- | --- | --- | --- | --- | --- |
| **PROBLEM** | No | Probably no | Probably yes | Yes |  | Varies | Do not know |
| **DESIRABLE EFFECTS** | Trivial | Low | Moderate | Large |  | Varies | Do not know |
| **UNDESIRABLE EFFECTS** | Large | Moderate | Low | Trivial |  | Varies | Do not know |
| **CERTAINTY OF EVIDENCE** | Very low | Low | Middle | high |  |  | No studies |
| **VALUES** | Important uncertainty or variability | Possibly important uncertainty or variability | Probably no important uncertainty or variability | No Important uncertainty or variability |  |  |  |
| **BALANCE OF EFFECTS** | Favors the comparison | Probably favors the comparison | Does not favor either the intervention or the comparison | Probably favors the intervention | Favors the intervention | Varies | Do not know |
| **ACCEPTABILITY** | No | Probably No | Probably yes | Yes |  | Varies | Do not know |
| **FEASIBILITY** | No | Probably no | Probably yes | Yes |  | Varies | Do not know |

**Type of Recommendation**

| Strong recommendation against the intervention | Conditional recommendation against the intervention | Conditional recommendation for either the intervention or the comparison | Conditional recommendation for the intervention | Strong recommendation for the intervention |
| --- | --- | --- | --- | --- |
| ○ | ○ | ○ | ○ | ○ |

**Conclusions**

| **Recommendation** |
| --- |
| EIT cannot be routinely used for determining PEEP settings in patients with ARDS (in our practice statement).  **Note：**  EIT is not available in Japan as of February 2021. Although there is no evidence to compare EIT with conventional methods of PEEP determination, EIT may help establish an appropriate PEEP for uniform lung ventilation. |
|  |
| **Justification** |
| **Question：** Should electrical impedance tomography (EIT) be used in PEEP settings for patients with ARDS?  **Patient：** Adult patients with ARDS.  **Intervention：** PEEP determination using EIT  **Comparison：** PEEP determination according to ARDSnet's FIO2/PEEP table  **Outcome:** Mortality, VILI  **Summary of evidence：**  EIT is a bedside device that visualizes gas distribution in the lungs by measuring impedance changes in the body. EIT is a non-invasive, continuous, real-time imaging system that can be used to visualize ventilation in different parts of the lung in cross-sectional images. In this systematic literature search, we found no studies that compared EIT PEEP determination with other methods for critical outcomes.  In a prospective observational study by Zhao et al.1), the method of PEEP determination by EIT was investigated in 24 patients with severe ARDS, using EIT to find the PEEP with the most uniformly inflated lungs, with both ventral lung hyperinflation and dorsal collapse regions low, while gradually decreasing PEEP from high pressure. This PEEP was used as the set PEEP by EIT. The PEEP set by this method was relatively high, averaging 17.6 cmH_2_O (±3.6), but no obvious pressure trauma occurred. This study shows that EIT can be used to depict the ventilation status of each lung region, and it may be possible to find PEEPs with uniformly inflated lungs to set appropriate PEEPs. However, few studies have evaluated PEEP determination by EIT, and there are no studies that directly compare PEEP determination by EIT with conventional PEEP determination methods. Therefore, it is not possible to provide a clear recommendation for this CQ. Therefore, this CQ is not an evidence-based recommendation but rather a description of current practice.  **Summary of the evidence:** No relevant studies  **Certainty of the evidence：**  Since there are no relevant studies, the quality of evidence cannot be assessed.  **Values, balance of effect, acceptability, feasibility：**  As of February 2021, EIT has not been released in Japan and is an experimental device. Even if it is released in the future, the cost is expected to be excessive.  **Panel meeting：**  In the pre-vote, according to the modified Delphi method, "EIT cannot be routinely used for setting PEEP in patients with ARDS" had a median score of 9.0 and a disagreement index of 0.049. As a result, the panel meeting finally agreed as "in our practice statement" with the result of the pre-vote without requiring a re-vote. |

| **Subgroup considerations** |
| --- |
| None |
| **Implementation considerations** |
| As of February 2021, EIT has not been released in Japan and is an experimental device. In addition, although EIT itself is non-invasive, there is no evidence to compare it with conventional PEEP determination methods, and PEEP determination by EIT may lead to excessive PEEP setting. Therefore, it is difficult to implement EIT in general. |

| **Monitoring and evaluation** |
| --- |
| To implement the recommendations, further information on the cost-effectiveness of EIT use as a clinical issue needs to be collected. In addition, it is necessary to monitor the implementation status of the guideline through the use of questionnaires and other means after the guideline is published to see if there are any other clinical problems. |
| **Research priorities** |
| In the future, a randomized controlled trial should be performed to compare PEEP determination by EIT with the routine method of determining PEEP according to the ARDSnet FIO2/PEEP table. |

References

1) [Zhao](https://pubmed.ncbi.nlm.nih.gov/?term=Zhao+Z&cauthor_id=30656479) Z, [Chang](https://pubmed.ncbi.nlm.nih.gov/?term=Chang+MY&cauthor_id=30656479) M-Y, [Chang](https://pubmed.ncbi.nlm.nih.gov/?term=Chang+MY&cauthor_id=30656479) M-Y, et al. Positive end-expiratory pressure titration with electrical impedance tomography and pressure-volume curve in severe acute respiratory distress syndrome. Ann Intensive Care. 2019;9(1):7.

**CQ34 Should pulmonary ultrasound be used for PEEP settings in patients with ARDS?**

1. Search strategy

MEDLINE via PubMed （Search date: 2020/ 3/ 21）

| #1 | “Respiratory Distress Syndrome, Adult”[MeSH Terms] OR “Lung Injury”[MeSH Terms] OR ARDS[Title/Abstract] OR “acute respiratory distress syndrome”[Title/Abstract] OR ALI[Title/Abstract] OR “acute lung injury”[Title/Abstract] |
| --- | --- |
| #2 | Ultrasonography[MeSH Terms] OR Echocardiography[MeSH Terms] OR ultraso*[Title/Abstract]) OR echo*[Title/Abstract] |
| #3 | #1 AND #2 |

1. Flow diagram

**Identification**

0 Studies included in qualitative synthesis

8 Full-text articles assessed for eligibility

3067 records after duplicates removed

3281 records identified through database searching

3281 records identified through database searching

Medline via PubMed (n=2420)

CENTRAL (n=391)

Igaku-Chuo-Zasshi (n=470)

0 additional records identified through other sources

0 Studies included in quantitative synthesis (meta-analysis)

8 Full-text articles excluded, with reasons:

・Wrong study design (n=8)

Etc.

Duplicates

n=214

3059 records excluded

**Included**

**Eligibility**

**Screening**

1. Risk of bias

Not applicable

1. Forest plot

Not applicable

1. Evidence profile

Not applicable

1. Evidence-to-Decision table

| **Question** | |
| --- | --- |
| **CQ34** Should pulmonary ultrasound be used for PEEP settings in patients with ARDS? | |
| **Population:** | Patients with ARDS that are over 18 years old |
| **Intervention:** | PEEP determination using lung echo |
| **Comparison:** | PEEP determination according to the ARDSnet table, PEEP determined by a physician |
| **Primary Outcome:** | Non-ventilated area, VALI, mortality |
| **Setting:** | Situation equivalent to the emergency room or intensive care unit |
| **Perspective:** | Individual |
| **Background:** | In patients with ARDS, excessive PEEP enhances VILI and adversely affects circulatory dynamics. Insufficient PEEP increases the risk of a collapsed lung, which has also been reported to affect VILI and worsen oxygenation. On the other hand, there is no established method for establishing an appropriate PEEP setting.  Clarification of useful methods for PEEP setting is an important clinical issue. |
| **Conflict of interests:** | None |

**Assessment**

| **Problem**  Is the problem a priority? | | |
| --- | --- | --- |
| **Judgment** | **Research Evidence** | **ADDITIONAL CONSIDERATIONS** |
| ○ No  ○ Probably no  ● Probably yes  ○ Yes  ○ Varies  ○ Do not know | In patients with ARDS, excessive PEEP causes VILI by hyperinflating the lungs, which also adversely affects circulatory dynamics. Too low PEEP has also been reported to increase collapsed lung, affect VILI, and worsen oxygenation. On the contrary, there is no established method for determining appropriate PEEP, and identifying useful methods for setting PEEP is an important clinical issue. Therefore, the priority of this issue is probably high. |  |
| **Desirable effects**  How substantial are the desirable anticipated effects? | | |
| **Judgment** | **Research Evidence** | ADDITIONAL CONSIDERATIONS |
| ○ Trivial  ○ Small  ○ Moderate  ○ Large  ○ Varies  ● Do not Know | Integrated into the certainty of the evidence. |  |
| **Undesirable effects**  How substantial are the undesirable anticipated effects? | | |
| **Judgment** | **Research Evidence** | ADDITIONAL CONSIDERATIONS |
| ○ Large  ○ Small  ○ Moderate  ○ Trivial  ○ Varies  ● Do not know | Integrated into the certainty of the evidence. |  |
| **Certainty of evidence**  What is the overall certainty of the evidence of effects? | | |
| **Judgment** | **Research Evidence** | ADDITIONAL CONSIDERATIONS |
| ○ Very low  ○ Low  ○ Moderate  ○ High  ● No included studies | When PEEP is increased, the area of the unventilated lung can be observed, and if the area decreases, PEEP has improved the lung collapse or recruits the lung. We conducted a systematic literature search and found no studies that compared PEEP determination by echocardiography with other methods for critical outcomes. The main evidence evaluating PEEP determination by echocardiography is presented below.  　In a prospective observational study by Stefanidis et al^1)^, they investigated whether lung echocardiography could confirm the effect of recruitment by increasing PEEP in 10 patients with ARDS. As PEEP was increased from 5 to 10 to 15 cmH2O, the decrease in the non-ventilated area was quantifiable by lung echocardiography, and an improvement in oxygenation was also observed.  　In a prospective observational study by Bouhemad et al.2), we investigated whether lung echocardiography could confirm the effect of recruitment by increasing PEEP in 40 patients with ARDS. The lung volumes were measured by PV curves when PEEP was increased from 0 to 15 cmH2O, and the ultrasound aeration scores measured by lung echography were compared with the PV curves. The results showed that the increase in lung volume measured by the PV curve correlated significantly with the ultrasound aeration score.  　A prospective observational study by Chiumello et al.3) compared the lung ultrasound score (LUS score) by echocardiography (evaluation of lung air content at six sites bilaterally, each with a score of 0-3 points) and lung air content by computed tomography (CT) in 20 patients with ARDS. When PEEP was performed at 5 or 15 cmH2O, the LUS score and CT lung air content were significantly correlated in both cases.  　These results suggest that lung echocardiography may be able to evaluate the recruits of a collapsed lung due to elevated PEEP. However, no studies compare PEEP determination by echocardiography with the ARDSnet method for critical outcomes, so the benefits and harms of this method cannot be assessed. |  |
| **Values**  Is there important uncertainty about or variability in how much people value the main outcomes? | | |
| **Judgment** | **Research Evidence** | ADDITIONAL CONSIDERATIONS |
| ○Important uncertainty or variability  ○ Possibly important uncertainty or variability  ● Probably no important uncertainty or variability   ○ No important uncertainty or variability | Maybe no significant uncertainty or diversity. |  |
| **Balance of effects**  Does the balance between desirable and undesirable effects favor the intervention or the comparison? | | |
| **Judgment** | **Research Evidence** | ADDITIONAL CONSIDERATIONS |
| ○ Favors the comparison  ○Probably favors the comparison  ○ Does not favor either the intervention or the comparison  ○ Probably favors the intervention  ○Favors the intervention  ○ Varies  ● Do not know | No relevant studies. |  |
|  |  |  |
| **Acceptability**  Is the intervention acceptable to key stakeholders? | | |
| **Judgment** | **Research Evidence** | ADDITIONAL CONSIDERATIONS |
| ○ No  ○ Probably no  ● Probably yes  ○ Yes  ○ Varies  ○ Do not know | Although there is no evidence used for the study, it has already been implemented in daily clinical practice and seems feasible considering the cost and adverse effects. |  |
| **Feasibility**  Is the intervention feasible to implement? | | |
| **Judgment** | **Research Evidence** | ADDITIONAL CONSIDERATIONS |
| ○ No  ○ Probably no  ○ Probably yes  ○ Yes  ● Varies  ○ Do not know | Lung echocardiography is easy to implement because it is non-invasive and does not require additional cost, but it requires skill and practice to evaluate lung air content by lung echocardiography. In addition, since there is no evidence to compare this method with the conventional PEEP determination method, PEEP determination by lung echocardiography may lead to an excessive PEEP setting. Therefore, it cannot be said to be feasible in general. |  |

**Summary of Judgment**

|  | Judgment | | | | | | |
| --- | --- | --- | --- | --- | --- | --- | --- |
| **PROBLEM** | No | Probably no | Probably yes | Yes |  | Varies | Do not know |
| **DESIRABLE EFFECTS** | Trivial | Low | Moderate | Large |  | Varies | Do not know |
| **UNDESIRABLE EFFECTS** | Large | Moderate | Low | Trivial |  | Varies | Do not know |
| **CERTAINTY OF EVIDENCE** | Very low | Low | Middle | high |  |  | No studies |
| **VALUES** | Important uncertainty or variability | Possibly important uncertainty or variability | Probably no important uncertainty or variability | No Important uncertainty or variability |  |  |  |
| **BALANCE OF EFFECTS** | Favors the comparison | Probably favors the comparison | Does not favor either the intervention or the comparison | Probably favors the intervention | Favors the intervention | Varies | Do not know |
| **ACCEPTABILITY** | No | Probably No | Probably yes | Yes |  | Varies | Do not know |
| **FEASIBILITY** | No | Probably no | Probably yes | Yes |  | Varies | Do not know |

**Type of Recommendation**

| Strong recommendation against the intervention | Conditional recommendation against the intervention | Conditional recommendation for either the intervention or the comparison | Conditional recommendation for the intervention | Strong recommendation for the intervention |
| --- | --- | --- | --- | --- |
| ○ | ○ | ○ | ○ | ○ |

**Conclusions**

| **Recommendation** |
| --- |
| It is not common to use lung echocardiography for setting PEEP in patients with ARDS (in our practice statement).  **Note：**  The assessment of non-ventilated areas of the lung by echocardiography requires skill and practice, and there is no evidence to compare it with conventional methods of PEEP determination. In addition, PEEP determination by echocardiography may lead to an excessive PEEP setting. On the contrary, lung echocardiography can assess the recruitment of collapsed lung due to elevated PEEP and may help in PEEP determination because it is non-invasive and has no additional cost. |
|  |
| **Justification** |
| **Question：** Should pulmonary ultrasound be used for PEEP settings in patients with ARDS?  **Patient：** Adult patients with ARDS.  **Intervention：** PEEP determination using lung echo  **Comparison：** PEEP determination according to ARDSnet's FIO2/PEEP table  **Outcome:** Non-ventilated area, VILI, mortality  **Summary of evidence：**  When PEEP is increased, the area of the unventilated lung can be observed, and if the area decreases, PEEP has improved the lung collapse or recruits the lung. We conducted a systematic literature search and found no studies that compared PEEP determination by echocardiography with other methods for critical outcomes. The main evidence evaluating PEEP determination by echocardiography is presented below.  A prospective observational study by Stefanidis et al.1) investigated whether echocardiography could confirm the effect of recruitment by PEEP elevation in 10 patients with ARDS. Echocardiography was able to quantify the reduction in the area of non-ventilated areas when PEEP was increased from 5 to 10 to 15 cmH2O. The decrease in the non-ventilated area was quantifiable by lung echocardiography, and the improvement in oxygenation was also observed.  A prospective observational study by Bouhemad et al.2) investigated whether lung echocardiography could confirm the effect of recruitment by increasing PEEP in 40 patients with ARDS. The lung volumes were measured by PV curves when PEEP was increased from 0 to 15 cmH2O, and the ultrasound aeration scores measured by lung echography were compared with the PV curves. The results showed that the increase in lung volume measured by the PV curve correlated significantly with the ultrasound aeration score.  A prospective observational study by Chiumello et al^3)^ compared the LUS score by echocardiographic assessment (six sites bilaterally, each with a score of 0-3 points to assess lung air content) and CT lung air content assessment in 20 patients with ARDS. Lung air content assessment by CT was compared. As a result, when PEEP was examined at 5 or 15 cmH2O, the LUS score and CT lung air content assessment were significantly correlated in both cases.  These results suggest that lung echocardiography may be able to evaluate the recruits of a collapsed lung due to elevated PEEP. However, no studies compare PEEP determination by echocardiography with the ARDSnet method for critical outcomes, and no clear recommendation can be made for this CQ. Therefore, this CQ is not an evidence-based recommendation but only a description of the current practice.  **Summary of the evidence:** No relevant studies  **Certainty of the evidence：**  Since there are no relevant studies, the quality of evidence cannot be assessed.  **Values, balance of effect, acceptability, feasibility：**  It is assumed that each facility treating ARDS has its own echocardiogram, and no additional cost or resources are required.  **Panel meeting：**  In the pre-vote, "It is not common to use lung echocardiography for setting PEEP in patients with ARDS" was selected by the modified Delphi method with a median score of 9.0 and a disagreement index of 0.2920. As a result, there was no re-vote in the panel meeting, and the final consensus was reached as "in our practice statement" with the result of the pre-vote. |

| **Subgroup considerations** |
| --- |
| None |
| **Implementation considerations** |
| Lung echocardiography is easy to implement because it is non-invasive and does not require additional cost. However, it requires skill and practice to evaluate lung air content by lung echocardiography. In addition, since there is no evidence to compare this method with the conventional method of PEEP determination, PEEP determination by lung echocardiography may lead to an excessive PEEP setting, and thus it cannot be said to be feasible in general. See the reason justification section. |

| **Monitoring and evaluation** |
| --- |
| To implement the recommendations, it is necessary to collect more information on clinical issues, such as the rate of agreement in evaluation among different examiners and the effectiveness of the test time. In addition, it is necessary to monitor the implementation status of the guideline through questionnaires, and other means after the guideline is published to see if there are any other clinical problems. |
| **Research priorities** |
| In the future, a high-quality randomized controlled trial comparing PEEP determination by echocardiography with the routine method of determining PEEP according to the ARDSnet FIO2/PEEP table should be performed. |

References

1. [Stefanidis](https://pubmed.ncbi.nlm.nih.gov/?term=Stefanidis+K&cauthor_id=21816054) K, [Dimopoulos](https://pubmed.ncbi.nlm.nih.gov/?term=Dimopoulos+S&cauthor_id=21816054) S, [Tripodaki](https://pubmed.ncbi.nlm.nih.gov/?term=Tripodaki+ES&cauthor_id=21816054) E-S, et al. Lung sonography and recruitment in patients with early acute respiratory distress syndrome: a pilot study. Crit Care. 2011;15(4):R185.

2. [Bouhemad](https://pubmed.ncbi.nlm.nih.gov/?term=Bouhemad+B&cauthor_id=20851923) B, [Brisson](https://pubmed.ncbi.nlm.nih.gov/?term=Brisson+H&cauthor_id=20851923) H, [Le-Guen](https://pubmed.ncbi.nlm.nih.gov/?term=Le-Guen+M&cauthor_id=20851923) M, et al. Bedside ultrasound assessment of positive end-expiratory pressure-induced lung recruitment. Am J Respir Crit Care Med. 2011;183(3):341-7.

3. [Chiumello](https://pubmed.ncbi.nlm.nih.gov/?term=Chiumello+D&cauthor_id=30048331) D, [Mongodi](https://pubmed.ncbi.nlm.nih.gov/?term=Mongodi+S&cauthor_id=30048331) S, [Algieri](https://pubmed.ncbi.nlm.nih.gov/?term=Algieri+I&cauthor_id=30048331) I, et al. Assessment of lung aeration and recruitment by CT scan and ultrasound in acute respiratory distress syndrome patients. Crit Care Med. 2018;46(11):1761-1768.

**CQ35 Should prone positioning be used in adult patients with moderate or severe ARDS?**

1. Search strategy

MEDLINE via PubMed （Search date: 2020/6/28）

| #1 | "Respiratory Distress Syndrome, Adult"[Mesh] OR "Acute Respiratory Distress"[Title/Abstract] OR ARDS[Title/Abstract] OR "Acute Lung Injury"[Mesh] OR Acute Lung Injur*[Title/Abstract] |
| --- | --- |
| #2 | "Respiratory Distress Syndrome, Newborn"[Mesh] |
| #3 | #1 OR #2 |
| #4 | "Prone Position"[Mesh] OR Prone Position*[Title/Abstract] |
| #5 | #3 AND #4 |

CENTRAL （Search date: 2020/7/5）

| #1 | [mh "Respiratory Distress Syndrome, Adult"] OR "Acute Respiratory Distress":ti,ab OR ARDS:ti,ab OR [mh "Acute Lung Injury"] OR "Acute Lung Injury":ti,ab |
| --- | --- |
| #2 | [mh "Respiratory Distress Syndrome, Newborn"] |
| #3 | #1 OR #2 |
| #4 | [mh "Prone Position"] OR "Prone Position":ti,ab |
| #5 | #3 AND #4 |

Igaku-Chuo-Zasshi（Search date: 2020/6/28）

| #1 | 呼吸窮迫症候群-急性/TH or 急性呼吸窮迫症候群/TA or ARDS/TA |
| --- | --- |
| #2 | 急性肺損傷/TH or 急性肺損傷/TA |
| #3 | #1 or #2 |
| #4 | 腹臥位/TH or 腹臥位/TA |
| #5 | #3 and #4 |
| #6 | (#5) and (PT=会議録除く) |

1. Flow diagram

**Identification**

17 Studies included in qualitative synthesis

196 Full-text articles assessed for eligibility

1330 records after duplicates removed

1362 records identified through database searching

1362 records identified through database searching

Medline via PubMed (n=1008)

CENTRAL (n=118)

Igaku-Chuo-Zasshi (n=236)

0 additional records identified through other sources

14 Studies included in quantitative synthesis (meta-analysis)

179 Full-text articles excluded, with reasons:

・Wrong language (n=1)

・Wrong study design (n=169)

・Wrong population (n=8)

・Wrong intervention (n=1)

Etc.

Duplicates

n=32

1134 records excluded

**Included**

**Eligibility**

**Screening**

1. Risk of bias

Mortality Duration of mechanical ventilation


Length of ICU stay Catheter troubles

Tracheak tube obstruction Decubitus ulcer

VAP

1. Forest plot

Mortality

Duration of mechanical ventilation

Length of ICU stay

Catheter troubles

Tracheal tube obstruction

Decubitus ulcer

VAP

1. Evidence profile

| Assessment of certainty | | | | | | | No. of patients | | **Efficacy** | | Certainty of the evidence | **Importance** |
| --- | --- | --- | --- | --- | --- | --- | --- | --- | --- | --- | --- | --- |
| No. of studies | Study design | Risk of bias | Indirectness | Inconsistency | Imprecision | **Others** | **Prone position** | **Control** | **Relative index (95% CI)** | **Absolute index (95% CI)** |  |  |
| **Mortality*** | | | | | | | | | | | | |
| 7 | RCT | Serious^a^ | Not Serious^b^ | Not Serious | Serious^c^ | None | 418/1088 (38.4%) | 447/1030 (43.4%) | **RR 0.84** (0.69 to 1.03) | -69 per 1000 patients (-135〜+13) | ⨁⨁◯◯  low | Critical |
| **Duration of mechanical ventilation** | | | | | | | | | | | | |
| 3 | RCT | Very Serious^d^ | Not Serious | Not Serious | Serious^e^ | None | 455 | 416 | - | Average 0.48days short (-1.61〜+0.65) | ⨁◯◯◯  Very low | Critical |
| **Length of ICU stay** | | | | | | | | | | | | |
| 3 | RCT | Serious^f^ | Not Serious | Not Serious | Very Serious^g^ | None | 265 | 253 | - | Average 1.03days long (-1.7〜+3.75) | ⨁◯◯◯  Very low | Critical |
| **Catheter trouble**** | | | | | | | | | | | | |
| 3 | RCT | Serious^h^ | Very Serious^i^ | Not Serious | Very Serious^j^ | None | 35/341(10.3%) | 21/345(6.1%) | **RR 1.52** (0.22 to 10.26) | +32 per 1000 patients (-47〜+564) | ⨁◯◯◯  Very low | Important |
| **Tracheal tube obstruction** | | | | | | | | | | | | |
| 2 | RCT | Serious^k^ | Not Serious | Not Serious | Not Serious | None | 96/405(23.7%) | 64/403(15.9%) | **RR 1.52** (1.19 to 1.95) | +83per 1000 patients (+30〜+151) | ⨁⨁⨁◯ moderate | Critical |
| **Decubitus ulcer** | | | | | | | | | | | | |
| 2 | RCT | Serious^i^ | Not Serious | Not Serious | Not Serious | None | 74/173(42.8%) | 54/171(31.6%) | **RR 1.36** (1.06 to 1.75) | +114 per 1000 patients (+19〜+237) | ⨁⨁⨁◯ moderate | Critical |
| **Ventilator associated pneumonia** | | | | | | | | | | | | |
| 3 | RCT | Very Serious^m^ | Not Serious | Not Serious | Serious^n^ | None | 102/510(20.0%) | 101/457(22.1%) | **RR 0.90** (0.70 to 1.15) | +22 per 1000 patients (-66〜+33) | ⨁◯◯◯  Very low | Important |

*Include 28 days, 30 days, 90 days, ICU mortality, and hospital death

**Include unplanned extubation, obstruction, and tube displacement

a. All studies include at least one high risk of bias (RoB) item

b. Considerably large I^2 values (50–90%: 65%),; however, inconsistencies are explained by subgroup differences

c. Does not meet the optimal information size (OIS) criterion with α = 0.05 and β = 0.20; however, the sample size is not large enough to lower the grade. The 95% CI includes no effect and the lower confidence limit crosses the threshold.

d. Studies with high contribution rates include many high RoBs

e. The 95% CI includes no effect and upper confidence limit crosses the threshold

f. All studies contain at least one high RoB

g. Small sample size, 95% CI contains no effect, and lower confidence limit crosses the threshold

h. All studies contain at least one high RoB

i. Very large I^2 values (75–100%: 91%)

j. Does not meet the OIS criterion with α = 0.05 and β = 0.20, wide 95% CI, upper and lower confidence limits crossing the threshold

k. All studies contain at least one high RoB item.

l. All studies contain at least one high RoB

m. Studies with high contribution rate contain more high RoB

n. Does not meet the OIS criterion when α = 0.05 and β = 0.20

o. Only one cohort study with one high RoB (blinded to outcome assessment)

p. Does not meet the OIS criterion with α=0.05 and β=0.20

1. Evidence-to-Decision table

| **Question** | |
| --- | --- |
| **CQ35** Should prone positioning be used in adult patients with moderate or severe ARDS? | |
| **Population:** | Adult patients with ARDS |
| **Intervention:** | Prone position |
| **Comparison:** | No prone position |
| **Primary outcome:** | Mortality (28 days, 30 days, 60 days, 90 days, intensive care unit (ICU) deaths, in-hospital mortality), ventilatory days, ICU stays, tracheal tube problems (unplanned removal, blockage, malposition), skin damage due to pressure, bedsores |
| **Setting:** | Situation equivalent to the emergency room or ICU |
| **Perspective:** | Individual |
| **Background:** | In patients with ARDS, changing position from supine to prone can cause more uniform distribution of stress and strain in the lungs. In addition, oxygenation is improved due to improved ventilation imbalance. Because of this, the prone position is considered one of the remedies for severe hypoxemia because it improves oxygenation in patients with ARDS. It has also been suggested to prevent the occurrence and progression of ventilator-induced lung injury (VILI), and prone positioning early in the course of ARDS may improve the patient’s prognosis. Although the prone position itself does not require any special equipment, complications can occur with repositioning and prolonged management. In this context, clarifying the benefits and harms of the prone position for patients with ARDS is an important clinical issue. Therefore, this issue is probably of high priority. |
| **Conflict of interests:** | None |

**Assessment**

| **Problem**  Is the problem a priority? | | |
| --- | --- | --- |
| **Judgment** | **Research Evidence** | **ADDITIONAL CONSIDERATIONS** |
| ○ No  ○ Probably no  ○ Probably yes  ● Yes  ○ Varies  ○ Do not know | In patients with ARDS, changing position from supine to prone can helps distribute lung stress and strain more evenly. Furthermore, oxygenation is improved due to the improvement of ventilation-perfusion imbalance ^1)^. The prone position is one of the remedies for severe hypoxemia because it improves oxygenation in patients with ARDS. It has also been suggested to prevent the occurrence and progression of VILI 1), and prone positioning early in the course of ARDS may improve patient prognosis 1). Prone positioning itself does not require any special equipment, but complications can occur with repositioning and prolonged management. In this context, clarifying the benefits and harms of the prone position for patients with ARDS is an important clinical issue. Therefore, this issue is probably of high priority. |  |
| **Desirable effects**  How substantial are the desirable anticipated effects? | | |
| **Judgment** | **Research Evidence** | ADDITIONAL CONSIDERATIONS |
| ○ Trivial  ○ Small  ● Moderate  ○ Large  ○ Varies  ○ Do not Know | A systematic review showed that seven randomized controlled trials (RCTs) consistent with the patient, intervention, comparison, and outcome (PICO) process had been performed, and a meta-analysis was performed using these studies. Since only one RCT examined the outcome of the non-ventilated period (VFD), we examined the ventilated period as an alternative. Since unplanned removal, obstruction, and malposition of the tracheal tube were reported as separate outcomes, we judged that integration was not possible and considered the outcome of tracheal tube trouble (obstruction, airway obstruction) important.  The estimated effect on short-term mortality (7 RCTs, N=2118) was a reduction of 69 patients per 1000 (95% CI: reduction of 135 to increase of 13), and the mean difference in ventilator days (3 RCTs, N=871) was a reduction of 0.48 days (95% CI: 1.61 days shorter to 0.65 longer). The mean difference in length of ICU stay (3 RCTs: N=518) was an increase of 1.03 days (95% CI: 1.70 days shorter to 3.75 days longer). Therefore, we judged the desired effect of the intervention to be “moderate.” |  |
| **Undesirable effects**  How substantial are the undesirable anticipated effects? | | |
| **Judgment** | **Research Evidence** | ADDITIONAL CONSIDERATIONS |
| ○ Large  ● Small  ○ Moderate  ○ Trivial  ○ Varies  ○ Do not know | In terms of adverse outcomes, the effect estimate for tracheal tube problems (2 RCTs: N=808) was a risk difference of 83 more people/1000 (95% CI: 30 more people　-　151 more people) in the prone position compared to no prone position, and a risk difference of 114 more people/1000 (95% CI: 19 more people-237 more people) for pressure-induced skin damage and pressure ulcers (2 RCTs: N=344). Therefore, the desired effect of the intervention was judged to be “small.” |  |
| **Certainty of evidence**  What is the overall certainty of the evidence of effects? | | |
| **Judgment** | **Research Evidence** | ADDITIONAL CONSIDERATIONS |
| ● Very low  ○ Low  ○ Moderate  ○ High  ○ No included studies | \| Outcome \| Importance \| **Certainty of the evidence** \| \| --- \| --- \| --- \| \| Mortality \| Critical \| ⨁⨁◯◯  Low \| \| Ventilator days \| Critical \| ⨁◯◯◯  Very low \| \| Length of ICU stay \| Critical \| ⨁◯◯◯  Very low \| \| Tracheal tube obstruction \| Critical \| ⨁⨁⨁◯  Moderate \| \| Decubitus ulcer \| Critical \| ⨁⨁⨁◯  Moderate \|   **Overall certainty of the evidence:**  The direction of desirable and undesirable effects was not consistent, and the certainty of evidence across outcomes was judged to be “very low” by adopting the certainty of the least certain evidence. |  |
| **Values**  Is there important uncertainty about or variability in how much people value the main outcomes? | | |
| **Judgment** | **Research Evidence** | ADDITIONAL CONSIDERATIONS |
| ○Important uncertainty or variability  ○ Possibly important uncertainty or variability  ● Probably no important uncertainty or variability   ○ No important uncertainty or variability | Maybe no significant uncertainty or diversity. |  |
| **Balance of effects**  Does the balance between desirable and undesirable effects favor the intervention or the comparison? | | |
| **Judgment** | **Research Evidence** | ADDITIONAL CONSIDERATIONS |
| ○ Favors the comparison  ○Probably favors the comparison  ○ Does not favor either the intervention or the comparison  ● Probably favors the intervention  ○Favors the intervention  ○ Varies  ○ Do not know | \| Outcome \| Comparison \| Intervention \| Absolute difference \| Relative effect (95% CI) \| \| --- \| --- \| --- \| --- \| --- \| \| Mortality \| 447/1030 (43.4%) \| 418/1088 (38.4%) \| -69 per 1000 patients (-135～+13) \| 0.84 (0.69〜1.03) \| \| Ventilator days \| - \| - \| Average -0.48 days (-1.61～+0.65) \|  \| \| ICU days \| - \| - \| Average +1.03 days (-1.7～+3.75) \|  \| \| Tube trouble  (obstruction, tracheal obstruction) \| 64/403 (15.9%) \| 96/405  (23.7%) \| +83 per1000 patients (+30～+151) \| 1.52 (1.19〜1.95) \| \| Decubitus ulcer \| 54/171 (31.6%) \| 74/173 (42.8%) \| +114 per1000 patients (+19～+237) \| 1.36 (1.06〜1.75) \|   Determined whether the effects of the intervention outweighed the harms, “probably in favor of the intervention”. |  |
| **Acceptability**  Is the intervention acceptable to key stakeholders? | | |
| **Judgment** | **Research Evidence** | ADDITIONAL CONSIDERATIONS |
| ○ No  ○ Probably no  ● Probably yes  ○ Yes  ○ Varies  ○ Do not know | There is no evidence used for the study, but it is already being done in regular practice and seems feasible enough considering the cost and other factors. |  |
| **Feasibility**  Is the intervention feasible to implement? | | |
| **Judgment** | **Research Evidence** | ADDITIONAL CONSIDERATIONS |
| ○ No  ○ Probably no  ● Probably yes  ○ Yes  ○ Varies  ○ Do not know | There is no evidence used for the study, but it has already been implemented in regular practice and seems feasible. |  |

**Summary of Judgment**

|  | Judgment | | | | | | |
| --- | --- | --- | --- | --- | --- | --- | --- |
| **PROBLEM** | No | Probably no | Probably yes | Yes |  | Varies | Do not know |
| **DESIRABLE EFFECTS** | Trivial | Low | Moderate | Large |  | Varies | Do not know |
| **UNDESIRABLE EFFECTS** | Large | Moderate | Small | Trivial |  | Varies | Do not know |
| **CERTAINTY OF EVIDENCE** | Very low | Low | Middle | High |  |  | No studies |
| **VALUES** | Important uncertainty or variability | Possibly important uncertainty or variability | Probably no important uncertainty or variability | No Important uncertainty or variability |  |  |  |
| **BALANCE OF EFFECTS** | Favors the comparison | Probably favors the comparison | Does not favor either the intervention or the comparison | Probably favors the intervention | Favors the intervention | Varies | Do not know |
| **ACCEPTABILITY** | No | Probably No | Probably yes | Yes |  | Varies | Do not know |
| **FEASIBILITY** | No | Probably no | Probably yes | Yes |  | Varies | Do not know |

**Type of Recommendation**

| Strong recommendation against the intervention | Conditional recommendation against the intervention | Conditional recommendation for either the intervention or the comparison | Conditional recommendation for the intervention | Strong recommendation for the intervention |
| --- | --- | --- | --- | --- |
| ○ | ○ | ○ | ● | ○ |

**Conclusions**

| **Recommendation** |
| --- |
| **Conditional recommendation for prolonged prone positioning in adult patients with moderate and severe ARDS (conditional recommendation/evidence of very low certainty: GRADE2D).**    **Note：**  **It should be performed in medical institutions familiar with the prone position. When the prone position is used, a long period of time (12 h or more) should be considered.** |
|  |
| **Justification** |
| **Question：**Should prone positioning be used in adult patients with moderate or severe ARDS?  **Patient：** Adult patients with ARDS  **Intervention：** Prone position  **Comparison：** No prone position  **Outcome:** Deaths (28 days, 30 days, 60 days, 90 days, ICU mortalities, in-hospital mortalities), ventilated days, ICU stays, tracheal tube problems (obstruction, airway obstruction), and decubitus ulcer  **Summary of evidence：**  As a result of a systematic review, seven RCTs consistent with PICO were identified, and a meta-analysis using these was performed. Since only one RCT included the outcome of VFD, the duration of ventilation was considered as an alternative.  The estimated effect on short-term mortality (7 RCTs, N=2118) was a reduction of 69 patients per 1000 (95% CI: reduction of 135 to increase of 13), and the mean difference in duration of ventilation (3 RCTs, N=871) was a reduction of 0.48 days (95% CI: 1.61 days shorter to 0.65 days higher). The mean difference in duration of ICU stay (3 RCTs: N=518) was an increase of 1.03 days (95% CI: 1.7 days shorter to 3.75 days longer), and the desired effect of the intervention was judged to be “moderate.” On the contrary, as outcomes of harm, the effect estimates for tracheal tube problems (2 RCTs: N=808) were 83 more patients/1000 at risk in the prone position compared to no prone position (95% CI: 30-151 more patients). For pressure sores and skin disorders (2 RCTs: N=344), the difference in risk was 114 per 1000 patients increase (95% CI: 19 higher to 237 higher), and the expected harm was judged to be “small.” Thus, whether the effect of the intervention was greater than the harm was judged to be “probably in favor of the intervention.”  **Certainty of the evidence：**  　Desirable effect results showed that the intervention reduced death, shortened ventilator duration, and increased ICU length of stay. On the contrary, the undesirable effect results showed that the intervention increased airway and skin problems. Therefore, the direction of the desirable and undesirable effects were not consistent, and the certainty of the evidence across the outcomes was judged to be “very low,” adopting the certainty of the evidence with the lowest certainty.  **Values, balance of effect, acceptability, feasibility：**  Although repositioning from the supine to the prone position requires more human resources than usual, the cost is likely to be minimal because no additional expensive equipment needs to be purchased. On the other hand, there is a concern that the prone position may cause airway problems and skin damage. However, the harm is likely to be minimal. In terms of fairness, prone treatment is considered to be a medical procedure within the scope of normal ARDS care and can be performed in facilities that are proficient in ARDS management. In contrast, safety may be inequitable depending on the facility’s level of proficiency and workforce. Concerning feasibility, in an international epidemiological study, prone therapy was used only in 33% of patients with severe ARDS 2). Although there is no evidence in Japan, it is already practiced in regular practice, and it seems feasible even if costs are taken into consideration.  **Panel meeting：**  In the pre-vote, the modified Delphi method gave a median score of nine and a disagreement index of 0.132 for “conditionally recommend prolonged prone positioning in moderately and severely ill adult patients with ARDS.”  At the panel meeting, there was a discussion regarding the fact that muscle relaxants were used in combination in both the prone and control groups in the landmark PROSEVA study 3). As a result, the panel meeting finally reached a consensus with the results of the pre-vote without a re-vote being required.    **Note：**  It should be performed in a medical institution with some familiarity with the prone position. The use of the prone position should be limited to patients with moderate to severe ARDS, and the duration of prone positioning should be extended (≧12 h). In the landmark PROSEVA study, muscle relaxants were used in both the prone and control groups (91% in the prone group and 82.3% in the control group). |

| **Subgroup considerations** |
| --- |
| Subgroups were analyzed for prone time (≧12 h), concomitant lung protection ventilation (≤8 mL/PBW), and impaired oxygenation by the Berlin definition (moderate and severe only).  For prone time (≧12 h; 4 RCTs: N=984), the effect estimate for short-term mortality was a risk difference of 119 fewer deaths/1000 patients (95% CI: 185 fewer to 35 fewer deaths) with prone compared to no prone. With lung-protective ventilation (≤8 mL/PBW; 4 RCTs: N=888), there was a risk difference of 118 fewer patients/1000 (95% CI: 200 fewer to 4 fewer). In the Berlin definition of impaired oxygenation (moderate/severe only) (4 RCTs: N=984), the risk difference was 119 fewer patients/1000 (95% CI: 185 fewer to 35 fewer patients).  These results suggest that prone positioning may improve mortality in all subgroups (prolonged prone, concomitant lung-protective ventilation, moderate and severe hypoxemia).  Future studies with sufficient power to determine subgroup benefits are needed. In the meantime, prone positioning should be limited to patients with moderate to severe ARDS, and prolonged prone positioning (≧12 h) should be considered. |
| **Implementation considerations** |
| The prone position should be performed in a facility with some familiarity with ARDS management and the prone position. It requires multiple staff members and may not be feasible in some facilities or at certain times of the day. Most studies reviewed in this systematic review used a PaO2/FIO2 ratio of <150 mmHg as an inclusion criterion, which is different from the AECC criteria and the Berlin definition of impaired oxygenation in ARDS.  The 2016 Japanese ARDS guideline recommendation 4) was “We suggest that prone management be implemented in adult patients with ARDS (especially moderate and severe cases) (GRADE 2C, weak recommendation/low certainty evidence).”  The Surviving Sepsis Campaign 2016 guideline 5) recommended: “the use of the prone position rather than the prone position is recommended for adult patients with sepsis and ARDS and a Pao2/FIO2 ratio of less than 150 mmHg (strong recommendation/moderate certainty of (strong recommendation/moderate certainty evidence).”. |

| **Monitoring and evaluation** |
| --- |
| When prone positioning is performed in high-risk adult patients with ARDS, respiration and circulation should be monitored. During prone positioning, care should be taken to avoid obstruction or malposition of the tracheal tube, obstruction of the intravenous line, pressure on the eyes or other parts of the body, and possible neurological damage due to the position. Informed consent from the patient’s family is necessary for prone positioning. Further information on the adverse effects of the prone position as a clinical issue needs to be collected before recommendations can be implemented. In addition, it is necessary to monitor the implementation status of the guideline through the use of questionnaires and other means after the guideline is published to ensure that there are no other clinical problems. |
| **Research priorities** |
| Long-term prognosis, effectiveness in combination with other therapies (ECMO), and optimal duration of the prone position need to be verified. |

References

1) Guérin C, Albert RK, Beitler J, et al. Prone position in ARDS patients: why, when, how and for whom. Intensive Care Med. 2020;46:2385-2396. PMID 33169218.

2) Guérin C, Beuret P, Constantin JM, et al. A prospective international observational prevalence study on prone positioning of ARDS patients: the APRONET (ARDS Prone Position Network) study. Intensive Care Med. 2018;44:22-37. PMID 29218379.

3) Guérin C, Reignier J, Richard J-C, et al. Prone positioning in severe acute respiratory distress syndrome. N Engl J Med. 2013;368:2159-68. PMID 23688302.

4) Hashimoto S, Sanui M, Egi M, et al. The clinical practice guideline for the management of ARDS in Japan. J Intens Care. 2017;5:50. PMID 28770093.

5) Rhodes A, Evans LE, Alhazzani W, et al. Surviving sepsis campaign: international guidelines for management of sepsis and septic shock: 2016. Intensive Care Med. 2017;43:304-377. PMID 28101605.

**CQ36 Should ECMO be conducted in adult patients with severe ARDS?**

1. Search strategy

MEDLINE via PubMed （Search date: 2020/5/26）

| #1 | Respiratory Distress Syndrome, Adult [mh] |
| --- | --- |
| #2 | Acute lung injury [mh] |
| #3 | ALI [tiab] OR ARDS [tiab] |
| #4 | acute lung injur* [tiab] OR acute respiratory distress [tiab] OR acute respiratory failure[tiab] |
| #5 | #1 OR #2 OR #3 OR #4 |
| #6 | Extracorporeal Membrane Oxygenation[mh] |
| #7 | Oxygenators[mh] |
| #8 | Extracorporeal Membrane Oxygenation[tiab] |
| #9 | Oxygenator*[tiab] |
| #10 | extracorporeal lung assist[tiab] |
| #11 | extracorporeal life support[tiab] |
| #12 | ECMO[tiab] OR ECLA[tiab] OR ECLS[tiab] |
| #13 | #6 OR #7 OR #8 OR #9 OR #10 OR #11 OR #12 |
| #14 | #5 AND #13 |
| #15 | Controlled clinical trial[pt] |
| #16 | randomized[tiab] |
| #17 | placebo[tiab] |
| #18 | randomly [tiab] |
| #19 | trial[tiab] |
| #20 | groups [tiab] |
| #21 | systematic review[pt] |
| #22 | meta-analysis[pt] |
| #23 | review[pt] |
| #24 | #15 OR #16 OR #17 OR #18 OR #19 OR #20 OR #21 OR #22 OR #23 |
| #25 | #14 AND #24 |
| #26 | animals[mh] NOT humans[mh] |
| #27 | #25 NOT #26 |

CENTRAL （Search date: 2020/5/26）

| #1 | Respiratory Distress Syndrome, Adult [mh] |
| --- | --- |
| #2 | Acute lung injury [mh] |
| #3 | ALI [tiab] OR ARDS [tiab] |
| #4 | acute lung injur* [tiab] OR acute respiratory distress [tiab] OR acute respiratory failure[tiab] |
| #5 | #1 OR #2 OR #3 OR #4 |
| #6 | Extracorporeal Membrane Oxygenation[mh] |
| #7 | Oxygenators[mh] |
| #8 | Extracorporeal Membrane Oxygenation[tiab] |
| #9 | Oxygenator*[tiab] |
| #10 | extracorporeal lung assist[tiab] |
| #11 | extracorporeal life support[tiab] |
| #12 | ECMO[tiab] OR ECLA[tiab] OR ECLS[tiab] |
| #13 | #6 OR #7 OR #8 OR #9 OR #10 OR #11 OR #12 |
| #14 | #5 AND #13 |
| #15 | Controlled clinical trial[pt] |
| #16 | randomized[tiab] |
| #17 | placebo[tiab] |
| #18 | randomly [tiab] |
| #19 | trial[tiab] |
| #20 | groups [tiab] |
| #21 | systematic review[pt] |
| #22 | meta-analysis[pt] |
| #23 | review[pt] |
| #24 | #15 OR #16 OR #17 OR #18 OR #19 OR #20 OR #21 OR #22 OR #23 |
| #25 | #14 AND #24 |
| #26 | animals[mh] NOT humans[mh] |
| #27 | #25 NOT #26 |

Igaku-Chuo-Zasshi（Search date: 2020/ 5/ 26）

| #1 | 呼吸窮迫症候群-急性/TH or 急性呼吸窮迫症候群/TA or ARDS/TA |
| --- | --- |
| #2 | 呼吸不全/TH or 呼吸不全/TA |
| #3 | 急性肺損傷/TH or 急性肺損傷/TA |
| #4 | #1 or #2 or #3 |
| #5 | ECMO/TH or ECMO/TA or 膜型体外循環人工肺/TA |

1. Flow diagram

**Identification**

2 Studies included in qualitative synthesis

17 Full-text articles assessed for eligibility

1630 records after duplicates removed

1649 records identified through database searching

1649 records identified through database searching

Medline via PubMed (n=734)

CENTRAL (n=100)

Igaku-Chuo-Zasshi (n=815)

0 additional records identified through other sources

2 Studies included in quantitative synthesis (meta-analysis)

Duplicates

n=19

1613 records excluded

**Included**

**Eligibility**

**Screening**

15 Full-text articles excluded, with reasons:

・Wrong language (n=2)

・Wrong study design (n=7)

・Wrong publication type (n=2)

・Wrong population (n=1)

・Wrong intervention (n=3)

1. Risk of bias

60-day mortality 90-day mortality

Length of hospital stay Ventilator-free days

Stroke SF-36

Bleeding complications

1. Forest plot

60-day mortality

90-day mortality

Length of hospital stay

Ventilator-free days

Stroke

SF-36

Bleeding complications

1. Evidence profile

| Assessment of certainty | | | | | | | No. of patients | | **Efficacy** | | Certainty of the evidence | **Importance** |
| --- | --- | --- | --- | --- | --- | --- | --- | --- | --- | --- | --- | --- |
| No. of studies | Study design | Risk of bias | Indirectness | Inconsistency | Imprecision | **Others** | **ECMO** | **CMV** | **Relative index (95% CI)** | **Absolute index (95% CI)** |  |  |
| **60-day mortality** | | | | | | | | | | | | |
| 2 | RCT | Serious^a^ | Not Serious | Not Serious | Not Serious | None | 73/214 (34.1%) | 102/215 (47.4%) | **RR 0.72** (0.57 to 0.91) | -133 per 1000 patients (-204〜-43) | ⨁⨁⨁◯  moderate | Critical |
| **90-day mortality** | | | | | | | | | | | | |
| 2 | RCT | Serious^a^ | Not Serious | Not Serious | Serious^c^ | None | 77/214(36.0%) | 105/215 (48.8%) | **RR 0.74** (0.59 to 0.92) | -127 per 1000 patients (-209〜-39) | ⨁⨁⨁◯  moderate | Critical |
| **Length of hospital stay** | | | | | | | | | | | | |
| 2 | RCT | Serious^a^ | Not Serious | Not Serious | Serious^c^ | None | 213 | 215 | - | Average 14.1 days long (+8.22〜+19.98) | ⨁⨁◯◯  low | Important |
| **Duration of mechanical ventilaton** | | | | | | | | | | | | |
| 1 | RCT | Serious^a^ | Not Serious | Not Serious | Serious^c^ | None | 124 | 125 | - | Average 8 days long (+0.91〜+15.09) | ⨁⨁◯◯  low | Important |
| **Stroke** | | | | | | | | | | | | |
| 1 | RCT | Serious^a^ | Not Serious | Not Serious | Serious^c^ | None | 3/124 (2.4%) | 8/125 (6.4%) | **RR 0.38** (0.10 to 1.39) | -40 per 1000 patients (-58〜-25) | ⨁⨁◯◯  low | Critical |
| **SF-36** | | | | | | | | | | | | |
| 1 | RCT | Serious^a^ | Not Serious | Not Serious | Serious^c^ | None | 90 | 90 | - | Average 4.5 points high (+3〜+6) | ⨁⨁◯◯  low | Important |
| **Bleeding complications** | | | | | | | | | | | | |
| 1 | RCT | Serious^a^ | Not Serious | Not Serious | Serious^c^ | None | 57/124 (46%) | 35/125 (28.0%) | **RR 1.64** (1.17 to 2.31) | -179 per 1000patients (+48〜+367) | ⨁⨁◯◯  low | Important |

CI: confidence interval; RR: risk ratio

a. One of the two RCTs recruited because 35/128 patients were crossed-over in the middle of treatment and the remaining because 22/89 patients in the ECMO intervention group did not have ECMO induction.

b. I2=88%.

c. Small sample size

1. Evidence-to-Decision table

| **Question** | |
| --- | --- |
| **CQ36 Should ECMO be conducted in adult patients with severe ARDS?** | |
| Population: | Adult patients with ARDS who have hypoxemia or hypercapnia refractory to conventional ventilatory management |
| **Intervention:** | ECMO: Treatment by the introduction of ECMO (VA-ECMO and VV-ECMO) *ECCO2R is not included. |
| **Comparison:** | Treatment with conventional ventilatory management only |
| **Primary outcome:** | 60-day mortality, 90-day mortality, stroke |
| **Setting:** | Situation equivalent to the emergency room or intensive care unit |
| **Perspective:** | Individual |
| **Background:** | The introduction of ECMO in patients with severe ARDS is expected to have a rescue effect on severe respiratory failure that is difficult to maintain with conventional ventilatory management and reduce mortality by minimizing lung injury. Clarification of the relationship between ECMO and mortality and adverse events is an important clinical issue. |
| **Conflict of interests:** | None |

**Assessment**

| **Problem**  Is the problem a priority? | | |
| --- | --- | --- |
| **Judgment** | **Research Evidence** | **ADDITIONAL CONSIDERATIONS** |
| ○ No  ○ Probably no  ○ Probably yes  ● Yes  ○ Varies  ○ Do not know | The introduction of ECMO in patients with severe ARDS is expected to have a rescue effect on severe respiratory failure that is difficult to maintain with conventional ventilatory management and reduce mortality by minimizing lung injury. However, the efficacy of ECMO has not been established due to the difficulty in determining indications and timing and the many complications. The relationship between ECMO and mortality and adverse events is an important clinical issue and, therefore, a high priority. |  |
| **Desirable effects**  How substantial are the desirable anticipated effects? | | |
| **Judgment** | **Research Evidence** | ADDITIONAL CONSIDERATIONS |
| ○ Trivial  ○ Small  ● Moderate  ○ Large  ○ Varies  ○ Do not Know | A systematic review identified that two RCTs consistent with PICO had been conducted, and a meta-analysis was performed using these trials.  The effect estimates for 60-day mortality (2 RCTs, N=429) was 133 fewer patients/1000 (95% CI: 204 to 43 fewer) in the intervention compared with the control. The effect estimates for 90-day mortality (2 RCTs: N=429) was a risk difference of 127/1000 (95% CI: 200 to 39) for the “intervention” compared to the “control” (95% CI: reduction of 200 to 39 persons). Thus, the desired effect of the intervention was judged to be “moderate.” |  |
| **Undesirable effects**  How substantial are the undesirable anticipated effects? | | |
| **Judgment** | **Research Evidence** | ADDITIONAL CONSIDERATIONS |
| ○ Large  ○ Moderate  ● Small  ○ Trivial  ○ Varies  ○ Do not know | Stroke was assessed as a major outcome of harm. The results showed that ECMO resulted in 40 fewer strokes (1 RCT: N=249) per 1000 patients (95% CI: 58 fewer strokes to 25 more strokes). However, the result was judged to be “small” because the number of strokes could have increased by 25 with the intervention. |  |
| **Certainty of evidence**  What is the overall certainty of the evidence of effects? | | |
| **Judgment** | **Research Evidence** | ADDITIONAL CONSIDERATIONS |
| ○ Very low  ○ Low  ● Moderate  ○ High  ○ No included studies | **Overall certainty of the evidence:**   \| Outcome \| Importance \| **Certainty of the evidence** \| \| --- \| --- \| --- \| \| 60-day mortality \| Critical \| ⨁⨁⨁◯  Moderate \| \| 90-day mortality \| Critical \| ⨁⨁⨁◯  Moderate \| \| Stroke \| Critical \| ⨁⨁◯◯  Low \| |  |
| **Values**  Is there important uncertainty about or variability in how much people value the main outcomes? | | |
| **Judgment** | **Research Evidence** | ADDITIONAL CONSIDERATIONS |
| ○Important uncertainty or variability  ○ Possibly important uncertainty or variability  ○ Probably no important uncertainty or variability  ● No important uncertainty or variability | No significant uncertainty or diversity. |  |
| **Balance of effects**  Does the balance between desirable and undesirable effects favor the intervention or the comparison? | | |
| **Judgment** | **Research Evidence** | ADDITIONAL CONSIDERATIONS |
| ○ Favors the comparison  ○Probably favors the comparison  ○ Does not favor either the intervention or the comparison  ● Probably favors the intervention  ○Favors the intervention  ○ Varies  ○ Do not know | \| Outcome \| Comparison \| Intervention \| Absolute difference \| Relative effect (95% CI) \| \| --- \| --- \| --- \| --- \| --- \| \| 60-day mortality \| 102/215 (47.4%) \| 73/214 (34.1%) \| -133 per 1000 patients (-204～-43) \| 0.72 (0.57〜0.91) \| \| 90-day mortality \| 105/215 (48.8%) \| 77/214 (36.0%) \| -127 per 1000 patients (-200～-39) \| 0.74 (0.59〜0.92) \| \| Stroke \| 8/125 (6.4%) \| 3/124 (2.4%) \| -40 per 1000 patients (-58～+25) \| 0.38 (0.10〜1.39) \|   The effects of the intervention outweighed the harms; thus, we determined the balance of the effects to be “probably in favor of the intervention.” |  |
| **Acceptability**  Is the intervention acceptable to key stakeholders? | | |
| **Judgment** | **Research Evidence** | ADDITIONAL CONSIDERATIONS |
| ○ No  ○ Probably no  ● Probably yes  ○ Yes  ○ Varies  ○ Do not know | Although it requires special medical equipment, it is often required in facilities that treat severe ARDS in Japan and is probably accepted as an option by major stakeholders. |  |
| **Feasibility**  Is the intervention feasible to implement? | | |
| **Judgment** | **Research Evidence** | ADDITIONAL CONSIDERATIONS |
| ○ No  ○ Probably no  ● Probably yes  ○ Yes  ○ Varies  ○ Do not know | Because it requires special medical equipment and materials, it may be practically difficult to perform ECMO in some facilities. On the contrary, in Europe and the United States, ECMO treatment has been centralized so that patients can be transported to an ECMO center for ECMO treatment when an eligible patient arises in a facility where ECMO cannot be performed. An ECMO project has been started in Japan, and the centralization of ECMO treatment is being attempted. |  |

**Summary of Judgment**

|  | Judgment | | | | | | |
| --- | --- | --- | --- | --- | --- | --- | --- |
| **PROBLEM** | No | Probably no | Probably yes | Yes |  | Varies | Do not know |
| **DESIRABLE EFFECTS** | Trivial | Low | Moderate | Large |  | Varies | Do not know |
| **UNDESIRABLE EFFECTS** | Large | Moderate | Small | Trivial |  | Varies | Do not know |
| **CERTAINTY OF EVIDENCE** | Very low | Low | Middle | High |  |  | No studies |
| **VALUES** | Important uncertainty or variability | Possibly important uncertainty or variability | Probably no important uncertainty or variability | No Important uncertainty or variability |  |  |  |
| **BALANCE OF EFFECTS** | Favors the comparison | Probably favors the comparison | Does not favor either the intervention or the comparison | Probably favors the intervention | Favors the intervention | Varies | Do not know |
| **ACCEPTABILITY** | No | Probably No | Probably yes | Yes |  | Varies | Do not know |
| **FEASIBILITY** | No | Probably no | Probably yes | Yes |  | Varies | Do not know |

**Type of Recommendation**

| Strong recommendation against the intervention | Conditional recommendation against the intervention | Conditional recommendation for either the intervention or the comparison | Conditional recommendation for the intervention | Strong recommendation for the intervention |
| --- | --- | --- | --- | --- |
| ○ | ○ | ○ | ● | ○ |

**Conclusions**

| **Recommendation** |
| --- |
| **Conditional recommendation for the use of ECMO in adults with severe ARDS (conditional recommendation/evidence of moderate certainty: GRADE 2B).**    **Note：**  **In the presence of severe hypoxia or hypercapnia that is refractory to standard lung-protective ventilation and adjunctive therapies such as muscle relaxants and prone positioning, it is advisable to seek the advice of an experienced institution or specialist regarding the indications for ECMO and patient transport.** |
|  |
| **Justification** |
| **Question：**Should ECMO be conducted in adult patients with severe ARDS?  **Patient：** Adult patients with ARDS, with hypoxemia and hypercapnia refractory to conventional ventilatory management  **Intervention：** Treatment by the introduction of ECMO (VA-ECMO and VV-ECMO) (not including ECCO2R)  **Comparison：** Treatment with conventional ventilatory management only  **Outcome:** 60-day mortality, 90-day mortality, stroke  **Summary of evidence：**  A systematic review identified two RCTs that compared the use of ECMO versus conventional ventilatory management alone in patients with severe ARDS (CESAR study 1) and EOLIA study 2). We conducted a meta-analysis of the results of these two RCTs.  The effect estimates for 60-day mortality (2 RCTs, N=429) were 133 fewer patients/1000 (95% CI: 204). The effect estimates for 90-day mortality (2 RCTs: N=429) was a reduction in risk of 127 deaths/1000 persons (95% CI: 200 to 39) for the “intervention” compared with the “control (95% CI: 200 reduction to 39 reduction). Thus, the desired effect of the intervention was judged to be “moderate.”  On the contrary, we evaluated stroke as an outcome of harm. The results showed that ECMO reduced the number of strokes (1 RCT: N=249) by 40 per 1000 patients (95% CI: 58 reduction to 25 increase). However, we judged the outcome to be “small” because the intervention could have resulted in 25 more. We also evaluated bleeding-related complications as an “important” outcome and found that the number of bleeding-related complications (1 RCT: N=249) increased by 179/1000 (95% CI: 48 increase to 367 increase) with ECMO.  Thus, we judged that the intervention was probably advantageous in terms of whether the effect was greater than the harm.  **Certainty of the evidence：**  　The direction of desirable and undesirable effects was consistent, and the certainty of the evidence across outcomes was judged to be “medium” using the highest certainty of the evidence.  **Values, balance of effect, acceptability, feasibility：**  To perform ECMO, a circuit including a cardiopulmonary machine and an artificial lung is required, and the cost is a concern. The CESAR study 1) conducted in the United Kingdom to evaluate the usefulness of ECMO for patients with ARDS estimated the cost of ECMO to be 19,252 pounds per quality-adjusted life-year (QALY; approximately 2.7 million Japanese yen per QALY). Similar data have not been presented in Japan. In addition, the introduction and management of ECMO requires a multitude of staff with specialized knowledge and skills, and the workforce of each facility should be considered.  Because of the need for special medical equipment and materials, ECMO may be practically difficult to implement in some facilities. Especially in Europe and the United States, ECMO treatment is centralized, and when a patient is eligible for ECMO at a facility where ECMO cannot be performed, the patient is transported to an ECMO center for ECMO. An ECMO project has been started in Japan, and the centralization of ECMO treatment is being attempted.  **Panel meeting：**  In the pre-vote, the modified Delphi method resulted in a median score of 8.0 and a disagreement index of 0.1316 for “conditionally recommend ECMO in adults with severe ARDS.” During the panel meeting, there were concerns about bleeding-related complications and discussions about training and patient selection for ECMO. As a result, the panel meeting finally reached a consensus with the results of the pre-vote without a re-vote being required. |

| **Subgroup considerations** |
| --- |
| Although there are no subgroups to consider, there is no specific definition of severe hypoxemia or hypercapnia that is resistant to ECMO. The CESAR study included patients with uncompensated hypercapnia with a Murray score of < 3 or pH < 7.2. In the EOLIA study, the criteria for the introduction of ECMO were a P/F ratio of < 50 for more than 3 h, P/F ratio of < 80 for more than 6 h, pH of < 7.25, and PaCO2 ≥ 60 mmHg for more than 6 h, even with conventional ventilatory management and adjunctive therapy such as muscle relaxants and prone positioning. Indications for ECMO should be determined at each institution with reference to these criteria. |
| **Implementation considerations** |
| An artificial cardiopulmonary machine and a circuit including an artificial lung are required to perform ECMO, and the cost is a concern. The CESAR study 1) conducted in the United Kingdom to evaluate the efficacy of ECMO in patients with ARDS estimated the cost of ECMO to be 19,252 pounds per QALY (approximately 2.7 million Japanese yen per QALY). Similar data have not been presented in Japan and should be considered in the future. In addition, the introduction and management of ECMO requires a multitude of staff with specialized knowledge and skills, and the workforce at each facility should be considered. To maintain a skilled workforce, exposure to a certain number of cases and simulation training for troubleshooting is necessary. As for the feasibility of ECMO at each facility, it is expected to be centralized at an ECMO center rather than implemented at all facilities.  The purpose of ECMO is to maintain lung-protective ventilation in patients with severe hypoxemia or hypercapnia during self-lung ventilation with the help of oxygenation and ventilation by artificial lungs. Although there is no definite ventilator setting during ECMO, it should be set to achieve at least normal lung-protective ventilation. In addition, anticoagulation is mandatory during ECMO to prevent intracircuit coagulation, and appropriate coagulation monitoring should be performed to avoid bleeding complications. We must also be aware of infectious complications due to the insertion of foreign bodies. Furthermore, it is necessary to be familiar with mechanical complications such as sudden ECMO shutdown due to intracircuit coagulation or deterioration of artificial lung or pump function, and simulation training for troubleshooting is necessary. |

| **Monitoring and evaluation** |
| --- |
| To implement the recommendations, it is necessary to collect more information on clinical issues such as adverse events and the cost-effectiveness of ECMO use. In addition, it is necessary to monitor the implementation status of the guideline through questionnaires, and other means after the guideline is published to see if there are any other clinical problems. |
| **Research priorities** |
| Further research is expected on appropriate management methods during ECMO, including ventilator settings and coagulation monitoring. Further studies are needed to determine the cost-benefit ratio in Japan. |

References

1) Guérin C, Albert RK, Beitler J, et al. Prone position in ARDS patients: why, when, how and for whom. Intensive Care Med. 2020;46:2385-2396. PMID 33169218.

2) Guérin C, Beuret P, Constantin JM, et al. A prospective international observational prevalence study on prone positioning of ARDS patients: the APRONET (ARDS Prone Position Network) study. Intensive Care Med. 2018;44:22-37. PMID 29218379.

3) Guérin C, Reignier J, Richard J-C, et al. Prone positioning in severe acute respiratory distress syndrome. N Engl J Med. 2013;368:2159-68. PMID 23688302.

4) Hashimoto S, Sanui M, Egi M, et al. The clinical practice guideline for the management of ARDS in Japan. J Intens Care. 2017;5:50. PMID 28770093.

5) Rhodes A, Evans LE, Alhazzani W, et al. Surviving sepsis campaign: international guidelines for management of sepsis and septic shock: 2016. Intensive Care Med. 2017;43:304-377. PMID 28101605.

**CQ37 Should early tracheostomy be performed in adult patients with ARDS?**

1. Search strategy

MEDLINE via PubMed （Search date: 2020/5/26）

| #1 | Respiration, Artificial[mh] |
| --- | --- |
| #2 | Mechanical Ventilation* [tiab] |
| #3 | #1 OR #2 |
| #4 | Tracheostomy[mh] |
| #5 | Tracheotomy[mh] |
| #6 | Tracheostom* [tiab] |
| #7 | Tracheotom[tiab] |
| #8 | Prone Position[mh] OR prone position*[tiab] |
| #9 | #4 OR #5 OR #6 OR #7 OR #8 |
| #10 | #3 AND #9 |
| #11 | Controlled clinical trial[pt] |
| #12 | Randomized[tiab] |
| #13 | Placebo[tiab] |
| #14 | Randomly[tiab] |
| #15 | Trial[tiab] |
| #16 | Groups [tiab] |
| #17 | systematic review [pt] |
| #18 | meta-analysis [pt] |
| #19 | Review [pt] |
| #20 | #11 OR #12 OR #13 OR #14 OR #15 OR #16 OR #19 |
| #21 | #10 AND #20 |
| #22 | Animals[mh] NOT humans[mh] |
| #23 | #21 NOR #22 |

CENTRAL （Search date: 2020/4/6）

| #1 | [mh”Respiration, Artifical”] |
| --- | --- |
| #2 | Mechanical NEXT Ventilation*:ti,ab |
| #3 | #1 OR #2 |
| #4 | [mh Tracheostomy] |
| #5 | #[mh Tracheotomy] |
| #6 | Tracheostom*:ti,ab |
| #7 | Tracheotom*:ti,ab |
| #8 | [mh”Prone Position”] OR prone NEXT position*:ti,ab |
| #9 | {OR #4- #8] |
| #10 | #3 AND #9 |
| #11 | [mh animals] NOT [mh humans] |
| #12 | #10 NOT #11 |

Igaku-Chuo-Zasshi （Search date: 2020/ 6/1）

| #1 | 人工呼吸/TH or 人工呼吸/TA or 機械的換気/TA |
| --- | --- |
| #2 | 気管開口術/TH or 気管開口/TA |
| #3 | 気管切開術/TH or 気管切開/TA |
| #4 | #2 or #3 |
| #5 | #1 and #4 |
| #6 | (#5) and (PT=会議録除く) |
| #7 | ランダム化比較試験/TH or ランダム化/AL or無作為化/AL |
| #8 | 比較試験/AL |
| #9 | 臨床試験/TH or 臨床試験/AL |
| #10 | プラセボ/TH or プラセボ/AL |
| #11 | 対照/AL |
| #12 | コントロール/AL |
| #13 | 臨床研究・疫学研究/TH or 臨床研究/AL |
| #14 | #7 or #8 or #9 or #10 or #11 or #12 or #13 |
| #15 | #6 and #14 |

1. Flow diagram

**Identification**

64 Studies included in qualitative synthesis

135 Full-text articles assessed for eligibility

2140 records after duplicates removed

2408 records identified through database searching

2408 records identified through database searching

Medline via PubMed (n=1718)

CENTRAL (n=557)

Igaku-Chuo-Zasshi (n=133)

0 additional records identified through other sources

14 Studies included in quantitative synthesis (meta-analysis)

71 Full-text articles excluded, with reasons:

・Wrong language (n=5)

・Wrong study design (n=56)

・Wrong population (n=4)

・Wrong intervention (n=2)

・Duplication (n=4)

Etc.

Duplicates

n=268

2005 records excluded

**Included**

**Eligibility**

**Screening**

1. Risk of bias

Mortality VAP

Ventilator-free days Days nurses rated as comfortable

Bleeding at tracheostomy Infection at tracheostomy site

1. Forest plot

Mortality

VAP

Ventilator-free days

Days nurses rated as comfortable

Bleeding at tracheostomy

Infection at tracheostomy site

1. Evidence profile

| Assessment of certainty | | | | | | | No. of patients | | **Efficacy** | | Certainty of the evidence | **Importance** |
| --- | --- | --- | --- | --- | --- | --- | --- | --- | --- | --- | --- | --- |
| No. of studies | Study design | Risk of bias | Indirectness | Inconsistency | Imprecision | **Others** | **Early tracheostomy** | **No Early tracheostomy** | **Relative index (95% CI)** | **Absolute index (95% CI)** |  |  |
| **Mortality*** | | | | | | | | | | | | |
| 14 | RCT | Not Serious | Not Serious | Serious^a^ | Not Serious | None | 440/1462 (30.1%) | 476/1425 (33.4%) | **RR 0.89** (0.79 to 1.00) | -37 per 1000 patients (-70〜0) | ⨁⨁⨁◯  moderate | Critical |
| **Ventilator Associated Pneumonia (VAP)** | | | | | | | | | | | | |
| 8 | RCT | Serious^b^ | Serious^c^ | Serious^a^ | Serious^d^ | None | 163/766 (21.3%) | 203/761 (26.7%) | **RR 0.75** (0.55 to 1.02) | -67 per 1000 patients (-120〜+5) | ⨁◯◯◯  Very low | Critical |
| **Ventilator Free Days (28 days)** | | | | | | | | | | | | |
| 4 | RCT | Serious^b^ | Not Serious | Serious^a^ | Not Serious | None | 621 | 622 | - | Average 1.2 days longer (+0.57〜+1.82) | ⨁⨁◯◯  low | Critical |
| **Average number of days nurses rated as comfortable (1-15 days)** | | | | | | | | | | | | |
| 1 | RCT | Serious^b^ | Not Serious | Serious^a^ | Serious^e^ | None | 109 | 107 | - | Average 1.4 days longer (+0.3〜+2.5) | ⨁◯◯◯  Very low | Important |
| **Bleeding at tracheostomy site** | | | | | | | | | | | | |
| 5 | RCT | Serious^b^ | Not Serious | Serious^a^ | Very Serious^f^ | None | 22/858 (2.6%) | 15/857 (1.8%) | **RR 1.42** (0.74 to 2.71) | +7 per 1000 patients(-5〜+30) | ⨁◯◯◯  Very low | Critical |
| **Infection at tracheostomy site** | | | | | | | | | | | | |
| 4 | RCT | Serious^b^ | Not Serious | Serious^a^ | Very Serious^f^ | None | 16/407 (3.9%) | 8/409 (2.0%) | **RR 1.62** (0.70 to 3.74) | +12 per 1000 patients (-6〜+54) | ⨁◯◯◯  Very low | Critical |
| **Workload of Respiration** | | | | | | | | | | | | |
| 0 | RCT | - | - | - | ^-^ |  |  |  | Impossible to estimate | - | - | Important |

*Include 28-day mortality, ICU mortality and Hospital Death

CI: Confidence Interval; RR: risk ratio

a. Downgraded by one level due to differences in populations (including populations other than ARDS) and differences in interventions and comparisons (definition of early tracheostomy, setting of control group).

b. Downgraded by one level due to risk of bias (difficulty in blinding patient care providers and insufficient blinding of outcome assessors).

c. Downgraded by one level due to high heterogeneity (I2=63%).

d. The width of the 95% confidence interval was wide, and clinical judgments were expected to change at the lower end of the confidence interval. Therefore, the overall imprecision was judged to be "serious" and downgraded by one level.

e. The total sample size is 216, which does not meet the optimal information size (OIS). The total sample size is 216, which does not meet the OIS. Therefore, it is considered to lack sufficient power and was downgraded by one level.

f. The width of the 95% confidence interval was wide, and the clinical judgment was considered to change at the upper and lower ends of the confidence interval. Therefore, we judged the overall imprecision to be "very serious" and downgraded it by two levels.

1. Evidence-to-Decision table

| **Question** | |
| --- | --- |
| **CQ37** Should early tracheostomy be performed in adult patients with ARDS? | |
| **Population:** | Adult ARDS patients |
| **Intervention:** | Early tracheostomy (surgical or percutaneous) |
| **Comparison:** | No early tracheostomy (surgical or percutaneous) |
| **Primary outcome:** | Mortality, ventilator-associated pneumonia (VAP), ventilator-free days (VFD), tracheostomy bleeding, tracheostomy infection |
| **Setting:** | Situation equivalent to the emergency room or intensive care unit |
| **Perspective:** | Individual |
| **Background:** | Compared to tracheal intubation, tracheostomy has the advantage of reducing sedation analgesics and avoiding vocal cord injury. However, tracheostomy is invasive, and there is a possibility of difficulty in extubation due to bleeding or airway narrowing. Although tracheostomy is generally used for tracheal intubation for more than 14 days, it has been reported that earlier tracheostomy may increase the benefits of tracheostomy and improve the prognosis by reducing the duration of ventilation and VAP. Therefore, it is an important clinical issue to determine whether early tracheostomy improves outcomes in patients with ARDS who are likely to have prolonged ventilatory periods. At present, no studies have investigated early tracheostomy in pediatric patients with ARDS, so only adult patients with ARDS were included in this study. |
| **Conflict of interests:** | None |

**Assessment**

| **Problem**  Is the problem a priority? | | |
| --- | --- | --- |
| **Judgment** | **Research Evidence** | **ADDITIONAL CONSIDERATIONS** |
| ○ No  ○ Probably no  ○ Probably yes  ● Yes  ○ Varies  ○ Do not know | Compared to tracheal intubation, tracheostomy has the advantage of reducing the amount of sedative analgesics and avoiding vocal cord damage 1-2). However, tracheostomy is invasive, and there is a possibility of difficulty in extubation due to bleeding or airway narrowing. Although tracheostomy is generally used for tracheal intubation for more than 14 days, it has been reported that earlier tracheostomy may increase the benefits of tracheostomy and improve the prognosis by reducing the duration of ventilation and VAP3). 3) Therefore, it is an important clinical issue to clarify whether early tracheostomy improves the prognosis of patients with ARDS who are likely to have prolonged ventilatory periods. At this time, no studies have examined early tracheostomy in pediatric ARDS patients, so only adult ARDS was included. Therefore, the priority of this issue is probably high. |  |
| **Desirable effects**  How substantial are the desirable anticipated effects? | | |
| **Judgment** | **Research Evidence** | ADDITIONAL CONSIDERATIONS |
| ○ Trivial  ● Small  ○ Moderate  ○ Large  ○ Varies  ○ Do not Know | A systematic review identified 14 RCTs consistent with process, and a meta-analysis was performed using these studies.  The effect estimates for short-term mortality (14 RCTs: N=2887) were 37 fewer per 1000 (95% CI: 70 fewer to 0 fewer), with early tracheostomy compared to no tracheostomy. VAP (8 RCTs; N=1527) had a mean difference of 67 fewer patients/1000 (95% CI: 120 fewer to 5 more), and VFD (4 RCTs; N=1243) had a mean difference of 1.2 days longer (95% CI: 0.57 days longer to 1.82 days longer). Based on the above, we judged the desired effect of the intervention to be “small.” |  |
| **Undesirable effects**  How substantial are the undesirable anticipated effects? | | |
| **Judgment** | **Research Evidence** | ADDITIONAL CONSIDERATIONS |
| ○ Large  ○ Moderate  ○ Small  ● Trivial  ○ Varies  ○ Do not know | Regarding harm outcomes, the effect estimates for tracheostomy bleeding (5 RCTs N=1715) showed a risk difference of 7 more people/1000 (95% CI: 5 fewer people to 30 more people) with early tracheostomy compared to no early tracheostomy. Tracheostomy infection (4 RCTs: N=816) showed a risk difference of 12 people increase/1000 people (95% CI: 6 people decrease to 54 people increase), and the expected harm was judged to be “slight.” |  |
| **Certainty of evidence**  What is the overall certainty of the evidence of effects? | | |
| **Judgment** | **Research Evidence** | ADDITIONAL CONSIDERATIONS |
| ● Very low  ○ Low  ○ Moderate  ○ High  ○ No included studies | \| Outcome \| Importance \| **Certainty of the evidence** \| \| --- \| --- \| --- \| \| Mortality \| Critical \| ⨁⨁⨁◯  Moderate \| \| VAP \| Critical \| ⨁◯◯◯  Very low \| \| VFD (28 days) \| Critical \| ⨁⨁◯◯  Low \| \| Bleeding at tracheostomy site \| Critical \| ⨁◯◯◯  Very low \| \| Infection at tracheostomy site \| Critical \| ⨁◯◯◯  Very low \|   **Overall certainty of the evidence:**  The direction of desirable and undesirable effects was not consistent, and the certainty of evidence across outcomes was judged to be “very low” by adopting the certainty of the least certain evidence. |  |
| **Values**  Is there important uncertainty about or variability in how much people value the main outcomes? | | |
| **Judgment** | **Research Evidence** | ADDITIONAL CONSIDERATIONS |
| ○Important uncertainty or variability  ○ Possibly important uncertainty or variability  ○ Probably no important uncertainty or variability  ● No important uncertainty or variability | No evidence was used in the study, but variability seems to be small. |  |
| **Balance of effects**  Does the balance between desirable and undesirable effects favor the intervention or the comparison? | | |
| **Judgment** | **Research Evidence** | ADDITIONAL CONSIDERATIONS |
| ○ Favors the comparison  ○Probably favors the comparison  ○ Does not favor either the intervention or the comparison  ● Probably favors the intervention  ○Favors the intervention  ○ Varies  ○ Do not know | \| Outcome \| Comparison \| Intervention \| Absolute difference \| Relative effect (95% CI) \| \| --- \| --- \| --- \| --- \| --- \| \| Mortality \| 476/1425 (33.4%) \| 440/1462 (30.1%) \| -37 per 1000 patients (-70～0) \| 0.89 (0.79〜1.00) \| \| VAP \| 203/761 (26.7%) \| 163/766 (21.3%) \| -67 per 1000 patients (-120～+5) \| 0.75 (0.55〜1.02) \| \| VFD (28 days) \| - \| - \| Average +1.2 days longer (+0.57～+1.82) \|  \| \| Tracheostomy bleeding \| 15/857 (1.8%) \| 22/858 (2.6%) \| +7 per 1000 patients (-5～+30) \| 1.42 (0.74〜2.71 \| \| Tracheostomy infection \| 8/409 (2.0%) \| 16/407 (3.9%) \| +12 per 1000 patients (-6～+54) \| 1.62 (0.70〜3.74) \|   Mortality: 28-days, ICU mortality, hospital death  We determined the balance between the effects to be “probably in favor of the intervention.” |  |
| **Acceptability**  Is the intervention acceptable to key stakeholders? | | |
| **Judgment** | **Research Evidence** | ADDITIONAL CONSIDERATIONS |
| ○ No  ○ Probably no  ● Probably yes  ○ Yes  ○ Varies  ○ Do not know | There is no evidence used for the study, but it is already being done in regular practice and seems feasible enough considering the cost and other factors. |  |
| **Feasibility**  Is the intervention feasible to implement? | | |
| **Judgment** | **Research Evidence** | ADDITIONAL CONSIDERATIONS |
| ○ No  ○ Probably no  ○ Probably yes  ● Yes  ○ Varies  ○ Do not know | There is no evidence used for the study, but it has already been implemented in regular practice and seems feasible. |  |

**Summary of Judgment**

|  | Judgment | | | | | | |
| --- | --- | --- | --- | --- | --- | --- | --- |
| **PROBLEM** | No | Probably no | Probably yes | Yes |  | Varies | Do not know |
| **DESIRABLE EFFECTS** | Trivial | Low | Moderate | Large |  | Varies | Do not know |
| **UNDESIRABLE EFFECTS** | Large | Moderate | Small | Trivial |  | Varies | Do not know |
| **CERTAINTY OF EVIDENCE** | Very low | Low | Middle | High |  |  | No studies |
| **VALUES** | Important uncertainty or variability | Possibly important uncertainty or variability | Probably no important uncertainty or variability | No Important uncertainty or variability |  |  |  |
| **BALANCE OF EFFECTS** | Favors the comparison | Probably favors the comparison | Does not favor either the intervention or the comparison | Probably favors the intervention | Favors the intervention | Varies | Do not know |
| **ACCEPTABILITY** | No | Probably No | Probably yes | Yes |  | Varies | Do not know |
| **FEASIBILITY** | No | Probably no | Probably yes | Yes |  | Varies | Do not know |

**Type of Recommendation**

| Strong recommendation against the intervention | Conditional recommendation against the intervention | Conditional recommendation for either the intervention or the comparison | Conditional recommendation for the intervention | Strong recommendation for the intervention |
| --- | --- | --- | --- | --- |
| ○ | ○ | ○ | ● | ○ |

**Conclusions**

| **Recommendation** |
| --- |
| **Conditional recommendation to perform early tracheostomy in adults with ARDS (conditional recommendation/evidence of very low certainty: GRADE2D).**    **Note：**  **There is no clear definition of early tracheostomy, but many studies have defined it as within 48 h to 10 days after the start of ventilation. Early tracheostomy can be considered once the patient’s general condition is stable. On the other hand, there is a concern that early tracheostomy may be performed on patients who do not need a tracheostomy. In addition, patients with ARDS requiring high oxygen concentrations, high airway pressures, or high positive end-expiratory pressure (PEEP) may be more at risk from tracheostomy itself than the patients in the studies included in this systematic review.** |
|  |
| **Justification** |
| **Question：**Should early tracheostomy be performed in adult patients with ARDS?  **Patient：** Adult patients with ARDS that require ventilatory management  **Intervention：** Early tracheostomy (surgical or percutaneous)  **Comparison：** No early tracheostomy (surgical or percutaneous)  **Outcome:** mortality (short-term), VAP, VFD, tracheostomy bleeding, tracheostomy infection  **Summary of evidence：**  A systematic review showed that 14 RCTs consistent with the PICO process were conducted, and a meta-analysis was performed using these studies.  　The effect estimates for short-term mortality (14 RCTs: N=2887) were 37 fewer per 1000 (95% CI: 70 fewer to 0 fewer), respectively, with early tracheostomy compared to no tracheostomy. VAP (8 RCTs, N=1527) had a mean difference of 67 fewer people/1000 (95% CI: 120 fewer people to 5 more people), and VFD (4 RCTs, N=1243) had a mean difference of 1.2 days longer (95% CI: 0.57 days longer to 1.82 days longer). Thus, we judged the desired effect of the intervention to be “probably small.” In contrast, the effect estimates for bleeding at the tracheostomy site (5 RCTs, N=1715) and infection at the tracheostomy site (4 RCTs, N=816) were not statistically significant, with a risk difference of 7 per 1000 patients (95% CI: 5 fewer to 30 higher) for early tracheostomy compared to no early tracheostomy. The expected harm was judged to be “slight,” with a difference of 12 higher per 1000 (95% CI: 6 patients fewer to 54 patients higher). Thus, whether the effect of the intervention was greater than the harm was judged to be “probably in favor of the intervention.”  **Certainty of the evidence：**  　The direction of desirable and undesirable effects was not consistent, and the certainty of evidence across outcomes was judged to be “very low” by adopting the certainty of the least certain evidence.  **Values, balance of effect, acceptability, feasibility：**  The medical fee point for tracheostomy in Japan is 2,570 points (25,700 yen). Since tracheostomy is a medical procedure within the scope of regular medical care, and there is no need to purchase additional equipment or secure personnel, the adverse effects of the intervention are considered to be minimal. On the contrary, there is a possibility that tracheostomy may be performed unnecessarily due to early tracheostomy. Although there is no evidence used in the study regarding the fairness of medical resources, there is no concern about the distribution of medical resources, and the medical treatment is within the scope of normal practice. As for the feasibility, there is no evidence used for the study, but it has already been implemented in regular practice, and it seems feasible enough considering the cost and other factors.  **Panel meeting：**  In the pre-vote, the modified Delphi method showed that “It is recommended that early tracheostomy be performed in adult patients with ARDS,” with a median score of 8.0 and a disagreement index of 0.292.  During the panel meeting, there was a discussion on the definition of early tracheostomy and the direction of the recommendation. The results of this systematic review and meta-analysis showed that early tracheostomy may improve prognosis. On the contrary, the certainty of the evidence is very low, and there were concerns about the negative effects (unnecessary tracheostomy, increased associated costs) of increased routine early tracheostomy following this recommendation. As a result, the panel re-voted on the “recommendation to perform early tracheostomy in adult patients with ARDS” and reached a final consensus with a median score of 8.0 and a disagreement index of 0.000. |

| **Subgroup considerations** |
| --- |
| It should be noted that patients with ARDS generally have a higher severity of illness and a poorer prognosis than the patients studied here who are expected to receive long-term ventilatory support (e.g., head injury). There is no clear definition of early tracheostomy, and it ranges from less than 48 h to less than 10 days between studies. As a subgroup, the timing of tracheostomy needs to be examined. |
| **Implementation considerations** |
| Patients with severe ARDS require high oxygen concentration, high airway pressure, or high PEEP. Adequate explanation and consent from the patient’s family are necessary.  The 2016 Japanese ARDS guideline recommendation 4) states, “We suggest that early tracheostomy should not be performed in adult patients with ARDS (GRADE 2C, weak recommendation/low certainty evidence).” However, early tracheostomy is not recommended in all cases. In the previous guideline,4) the only outcomes considered were death and VAP. Nevertheless, in this guideline, short-term mortality, VAP, ventilatory free days, and bleeding and infection of the tracheostomy hole are considered, and the benefits and harms of early tracheostomy are examined from a more multifaceted perspective. On the contrary, as with the 2015 Cochrane Database systematic review 5), the quality of evidence was very low, and we struggled to determine the direction of our recommendations. We believed that this was due to clinical and methodological heterogeneity among studies, which reduced the quality of evidence. Therefore, adapting this recommendation to clinical practice requires careful consideration on a case-by-case basis. Specifically, in critically ill patients, tracheostomy should be performed when there is a clear clinical indication and after multidisciplinary evaluation, taking into account the patient’s prognosis and QOL). |

| **Monitoring and evaluation** |
| --- |
| To implement the recommendations, it is necessary to collect more information on adverse events associated with early tracheostomy as a clinical problem. In addition, it is necessary to monitor the implementation status of the guideline through the use of questionnaires and other means after its publication to see if there are any other clinical problems. |
| **Research priorities** |
| Studies are needed to determine the appropriate timing of intervention in patients with ARDS.  Moreover, there is a need for studies to examine the technique of tracheostomy (surgical versus percutaneous tracheostomy).^5)^ |

References

1) Guérin C, Albert RK, Beitler J, et al. Prone position in ARDS patients: why, when, how and for whom. Intensive Care Med. 2020;46:2385-2396. PMID 33169218.

2) Guérin C, Beuret P, Constantin JM, et al. A prospective international observational prevalence study on prone positioning of ARDS patients: the APRONET (ARDS prone position network) study. Intensive Care Med. 2018;44:22-37. PMID 29218379.

3) Guérin C, Reignier J, Richard J-C, et al. Prone positioning in severe acute respiratory distress syndrome. N Engl J Med. 2013;368:2159-68. PMID 23688302.

4) Hashimoto S, Sanui M, Egi M, et al. The clinical practice guideline for the management of ARDS in Japan. J Intens Care. 2017;5:50. PMID 28770093.

5) Rhodes A, Evans LE, Alhazzani W, et al. Surviving sepsis campaign: international guidelines for management of sepsis and septic shock: 2016. Intensive Care Med. 2017;43:304-377. PMID 28101605.

**CQ38 Should a VAP prevention bundle be routinely conducted for adult patients with ARDS?**

1. Search strategy

Not applicable

1. Flow diagram

Not applicable

1. Risk of bias

Not applicable

1. Forest plot

Not applicable

1. Evidence profile

Not applicable

1. Evidence-to-Decision table

| **Question** | |
| --- | --- |
| **CQ38 Should a VAP prevention bundle be routinely conducted for adult patients with ARDS?** | |
| **Population:** | Adult patients with ARDS requiring ventilatory support |
| **Intervention:** | Routine ventilator-associated pneumonia (VAP) prophylaxis bundle |
| **Comparison:** | Routine VAP prophylaxis bundle not implemented |
| **Primary outcome:** | ICU mortality, in-hospital mortality, post-intensive care syndrome (PICS), VAP, ventilator-free days, ICU stay, length of hospital stay |
| **Setting:** | Situation equivalent to the emergency room or ICU |
| **Perspective:** | Individual |
| **Background:** | VAP is a bacterial pneumonia that newly occurs after 48 h from the start of artificial respiration, and VAP includes pneumonia that occurs within 48 h of ventilator withdrawal. According to a 2019 report by the Japan Nosocomial Surveillance (JANIS) of the Ministry of Health, Labour and Welfare in Japan, the incidence of VAP was 1.3/1,000 patients/day. VAP occurs in 10-20% of ventilated patients and 3-4% of ICU patients, and the incidence of VAP in ARDS patients is 20-40%. The standardized mortality ratio for VAP was 1.3, and the severity-adjusted length of hospital stay was 8-11 days. VAP is one of the most important complications in ventilated patients, and it is important to implement appropriate preventive measures. A VAP prevention bundle, which is a set of care, has been proposed as a method of VAP prevention, but its effectiveness and certainty of evidence are unknown. |
| **Conflict of interests:** | None |

**Seven criteria for judging a good practice statement**

| Question | Answer |
| --- | --- |
| (1) Is the description clear and actionable? | Yes  Rationale: VAP prevention bundles are inexpensive, have few complications, and are easily implemented. |
| (2) Is the message truly necessary? | Yes  Rationale: Implementing a VAP prevention bundle is harmless, widely accepted in daily clinical practice, and a necessary message. |
| (3) Is the net benefit significant and unquestionable? | Yes  Reason: The benefits of VAP prevention bundles are clearly greater than the harms. |
| (4) Is the evidence for the clinical question difficult to collect and summarize? | Yes  Rationale: Many intervention studies have evaluated each of the components of the VAP prevention bundle. However, although clinical studies have compared the implementation of VAP prevention bundles, most of them are observational studies, and the contents of the bundles differ, making it difficult to summarize. |
| (5) Are there specific issues (e.g., fairness) to consider? | No  Reason: There is no issue of fairness regarding the implementation of the VAP prevention bundle. |
| (6) Is the rationale clearly articulated? | Yes  Rationale: The implementation of VAP prevention bundles is important for medical safety, and there is a possibility that patient outcomes will be improved by the implementation of VAP prevention bundles. |
| (7) Should the evaluation be based on a formal GRADE? | No reason  Rationale: It is difficult to summarize the evidence on VAP care bundles, and there seems to be no need to consider the balance of benefits and harms, fairness, or cost. |

**Assessment**

| **Problem**  Is the problem a priority? | | |
| --- | --- | --- |
| **Judgment** | **Research Evidence** | **ADDITIONAL CONSIDERATIONS** |
| ○ No  ○ Probably no  ○ Probably yes  ● Yes  ○ Varies  ○ Do not know | VAP is a bacterial pneumonia that newly occurs after 48 h from the start of artificial respiration, and VAP includes pneumonia that occurs within 48 h of ventilator withdrawal. According to the JANIS of the Ministry of Health, Labour and Welfare in Japan, the incidence of VAP was 1.3/1,000 patients/day in 2019. VAP occurs in 10-20% of ventilated patients and 3-4% of ICU patients, and the incidence of VAP in ARDS patients is 20-40%. The standardized mortality ratio for VAP was 1.3, and the severity-adjusted length of hospital stay was 8-11 days. VAP is one of the most important complications in ventilated patients, and it is important to implement appropriate preventive measures. A VAP prevention bundle, which is a set of care, has been proposed as a method to prevent VAP, but its effectiveness and certainty of evidence are unknown. Therefore, the priority of this issue is high. |  |
| **Desirable effects**  How substantial are the desirable anticipated effects? | | |
| **Judgment** | **Research Evidence** | ADDITIONAL CONSIDERATIONS |
| ○ Trivial  ○ Small  ● Moderate  ○ Large  ○ Varies  ○ Do not Know | The outcomes of benefit from the introduction of the VAP prevention bundle were ICU mortality, in-hospital mortality, PICS, VAP, ventilator-free days, ICU length of stay, and length of hospital stay. However, no RCTs were found, and observational studies evaluating the association between VAP prevention bundles and the incidence of VAP and in-hospital mortality were found.  In a prospective, before-and-after study of 3,665 ventilated patients, the introduction of the VAP prevention bundle reduced VAP incidence from 8.6/1,000 patient-days to 2.0/1,000 patient-days (p < 0.0001). However, in this study, the VAP prevention bundle was not significantly associated with in-hospital mortality (adjusted odds ratio 1.13, 95% CI 0.98-1.31) ^1)^.  In a before-and-after study of 1,068 ventilated patients, the implementation of an active VAP prevention bundle significantly reduced the incidence of VAP from 19.2/1,000 patient-days to 7.5/1,000 patient-days (incidence difference 11.6/1,000 patient-days, 95% CI 2.3-21.0/ 1,000 patients/day). However, again in this study, the VAP prevention bundle was not significantly associated with in-hospital mortality (30% vs. 23%, p = 0.06)^2)^.  Although the VAP prevention bundle is expected to reduce VAP incidence, its effect on in-hospital mortality is unknown. Therefore, we judged the expected effect to be “moderate.” |  |
| **Undesirable effects**  How substantial are the undesirable anticipated effects? | | |
| **Judgment** | **Research Evidence** | ADDITIONAL CONSIDERATIONS |
| ○ Large  ○ Moderate  ○ Small  ● Trivial  ○ Varies  ○ Do not know | As outcomes of harm due to the introduction of the VAP prevention bundle, we set abnormal tracheal tube position and obstruction, as well as blood pressure changes. However, we could not find any studies that examined these items, so we judged the predicted harm to be “slight.” |  |
| **Certainty of evidence**  What is the overall certainty of the evidence of effects? | | |
| **Judgment** | **Research Evidence** | ADDITIONAL CONSIDERATIONS |
| ○ Very low  ○ Low  ○ Moderate  ● High  ○ No included studies | We could not find any RCTs that directly compared the use of VAP prevention bundles in adults with ARDS requiring ventilatory support.  In addition, the components of the VAP prevention bundle vary among countries and sites.  The United States Institute for Healthcare Improvement (IHI) developed a VAP prevention bundle in 2005 that includes the following four items ^3)^.  1. Elevation of the head of the bed  2. Daily discontinuation of sedation and assessment of weaning from the ventilator  3. Prevention of peptic ulcer  4. Prevention of deep vein thrombosis  In a multicenter observational study, implementation of this bundle resulted in a 44.5% reduction in the incidence of VAP ^3)^. In addition, a small observational study using this bundle reported a reduction in the duration of ventilator use and ICU stay ^4)^. In 2010, daily oral care with chlorhexidine was added to the IHI VAP prevention bundle, bringing the total to five items.  On the contrary, in Europe, a bundle has been developed with the following five items, which differ from the IHI ^5)^.  1. Do not change the respiratory circuit  2. Reliable hand hygiene with alcohol  3. Appropriate education and training for staff  4. Protocols for sedation and respiratory weening  5. Oral care with chlorhexidine  In Japan, the Japanese Society of Intensive Care Medicine’s Ventilation-Associated Pneumonia Prevention Bundle 2010 Revision is well known and consists of the following five items ^6)^:  1. Hand hygiene should be practiced without fail  2. Do not change the ventilator circuit frequently  3. Ensure appropriate sedation and analgesia, especially avoiding over-sedation  4. Assess daily for ability to wean from the ventilator  5. Do not manage patients in the supine position while on a ventilator  In addition to the above, some before-and-after studies included subglottic cuff suctioning and closed suction systems in the care bundle ^2)^.  To understand the current status of the implementation rate of each bundle item in Japan, we conducted a web-based questionnaire survey of the members of the three societies: the Japanese Respiratory Society, the Japanese Society of Respiratory Therapy, and the Japanese Society of Intensive Care Medicine (survey period: August 7 to August 31, 2020; the number of valid responses: 658). The results were as follows.  No periodic replacement of ventilator circuits: 55.4%.  Not managing patients in the prone position: 68%.  Avoiding deep sedation: 56.6% (when the patient is unstable) and 76% (when the patient is stable).  Evaluate daily whether the patient can be weaned from the ventilator: 67%.  Oral care at least once a day: 95%.  Use of endotracheal tube with suction port above cuff: 69%.  Use of closed suction catheter kit: 87%.  The implementation rate of each item varied from 55.5% to 95%, suggesting that the contents of VAP prevention implemented in each facility differed.  The recommended care bundle differs in each region and facility as described above. |  |
| **Values**  Is there important uncertainty about or variability in how much people value the main outcomes? | | |
| **Judgment** | **Research Evidence** | ADDITIONAL CONSIDERATIONS |
| ○Important uncertainty or variability  ○ Possibly important uncertainty or variability  ○ Probably no important uncertainty or variability  ● No important uncertainty or variability | The primary outcomes were ICU mortalities, in-hospital mortalities, and the occurrence of PICS and VAP, all of which were judged to have “no” variation in magnitude of value. |  |
| **Balance of effects**  Does the balance between desirable and undesirable effects favor the intervention or the comparison? | | |
| **Judgment** | **Research Evidence** | ADDITIONAL CONSIDERATIONS |
| ○ Favors the comparison  ○Probably favors the comparison  ○ Does not favor either the intervention or the comparison  ● Probably favors the intervention  ○Favors the intervention  ○ Varies  ○ Do not know | Although we established abnormal tracheal tube position and obstruction and blood pressure fluctuations as predicted harms, we judged that the predicted harms were “small” because we could not find studies that examined these items. Therefore, we judged that the balance between desirable and undesirable effects was “probably in favor of the intervention.” |  |
| **Acceptability**  Is the intervention acceptable to key stakeholders? | | |
| **Judgment** | **Research Evidence** | ADDITIONAL CONSIDERATIONS |
| ○ No  ○ Probably no  ○ Probably yes  ● Yes  ○ Varies  ○ Do not know | The VAP prevention bundle was judged to be “acceptable” to key stakeholders because it is widespread nationwide and addressed in Japan’s National Joint Action on Medical Safety ^7)^. |  |
| **Feasibility**  Is the intervention feasible to implement? | | |
| **Judgment** | **Research Evidence** | ADDITIONAL CONSIDERATIONS |
| ○ No  ○ Probably no  ○ Probably yes  ● Yes  ○ Varies  ○ Do not know | Many facilities are already implementing this in their daily clinical practice, so it seems feasible. However, the VAP prevention bundle content needs to be formulated after considering what can be implemented at each facility.  For example, oral care using chlorhexidine, which is used overseas, is a 2% chlorhexidine formulation, and its use is not approved in Japan due to concerns about anaphylactic shock.  It has also been pointed out that there is insufficient consideration of bundle compliance, and appropriate education for medical staff is necessary ^8)^. |  |

**Summary of Judgment**

|  | Judgment | | | | | | |
| --- | --- | --- | --- | --- | --- | --- | --- |
| **PROBLEM** | No | Probably no | Probably yes | Yes |  | Varies | Do not know |
| **DESIRABLE EFFECTS** | Trivial | Low | Moderate | Large |  | Varies | Do not know |
| **UNDESIRABLE EFFECTS** | Large | Moderate | Small | Trivial |  | Varies | Do not know |
| **CERTAINTY OF EVIDENCE** | Very low | Low | Middle | high |  |  | No studies |
| **VALUES** | Important uncertainty or variability | Possibly important uncertainty or variability | Probably no important uncertainty or variability | No Important uncertainty or variability |  |  |  |
| **BALANCE OF EFFECTS** | Favors the comparison | Probably favors the comparison | Does not favor either the intervention or the comparison | Probably favors the intervention | Favors the intervention | Varies | Do not know |
| **ACCEPTABILITY** | No | Probably No | Probably yes | Yes |  | Varies | Do not know |
| **FEASIBILITY** | No | Probably no | Probably yes | Yes |  | Varies | Do not know |

**Type of Recommendation**

| Strong recommendation against the intervention | Conditional recommendation against the intervention | Conditional recommendation for either the intervention or the comparison | Conditional recommendation for the intervention | Strong recommendation for the intervention |
| --- | --- | --- | --- | --- |
| ○ | ○ | ○ | ○ | ● |

**Conclusions**

| **Recommendation** |
| --- |
| **It is recommended that routine VAP prophylaxis bundles be implemented in adult patients with ARDS on ventilators.**  **Note：**  **The content of the bundle should be discussed at each institution (see “summary of evidence”).** |
|  |
| **Justification** |
| **Question：**Should a VAP prevention bundle be routinely conducted for adult patients with ARDS?  **Patient：** Adult ARDS patients requiring ventilatory management  **Intervention：** Routine VAP prophylaxis bundle  **Comparison：** Routine VAP prophylaxis bundle not implemented  **Outcome:** ICU mortality, in-hospital mortality, PICS, VAP, ventilator-free days, ICU stay, hospital stay  **Summary of evidence：**  VAP is a bacterial pneumonia that occurs after 48 h of ventilation, and VAP includes pneumonia that occurs within 48 h of ventilator withdrawal. VAP occurs in 10-20% of ventilated patients and 3-4% of patients admitted to the ICU ^9-11)^, and in 20-40% of patients with ARDS ^12-13)^. In Japan, the incidence of VAP in the ICU is 1.3/1,000 patients/day ^14)^. VAP is the most frequent nosocomial infection in the ICU and one of the most important complications in ventilated patients, so it is important to implement appropriate preventive measures. The VAP prevention bundle has been proposed as a method to prevent VAP. However, there are no RCTs of VAP prevention bundles in patients with ARDS, and the efficacy is unknown. Although this CQ is a high priority, it is difficult to present good quality evidence for VAP prevention bundles in patients with ARDS.  The desired effect expected from the implementation of the VAP prevention bundle was judged to be moderate. We could not locate any randomized controlled trials that directly compared the use of VAP prevention bundles in adults with ARDS requiring ventilatory management. In a prospective, before-and-after study of 3,665 ventilated patients, the VAP prevention bundle reduced the incidence of VAP from 8.6/1,000 patient-days to 2.0/1,000 patient-days. In a prospective pre/post-study, the VAP prevention bundle reduced the incidence of VAP from 8.6/1,000 patient-days to 2.0/1,000 patient-days (p < 0.0001). However, the VAP prophylaxis bundle in this study was not significantly associated with in-hospital mortality (adjusted odds ratio 1.13, 95% CI 0.98-1.31) ^1)^. In a before-and-after study of 1,068 ventilated patients, the implementation of an active VAP prevention bundle significantly reduced the incidence of VAP from 19.2/1,000 patient-days to 7.5/1,000 patient-days (difference in incidence 11.6/1,000 patient-days, 95% CI 2.3-21.0). However, again in this study, the VAP prevention bundle was not significantly associated with in-hospital mortality (30% vs. 23%, p = 0.06) ^2)^. Based on the above, we believe that the VAP prevention bundle will reduce the incidence of VAP, but the effects on in-hospital mortality, ICU mortality, occurrence of PICS, duration of non-ventilation, ICU stay, and length of hospital stay are unknown. Therefore, the expected desired effect was judged to be “moderate.” In addition, we established abnormal tracheal tube position and obstruction, and blood pressure fluctuations as expected harms from the introduction of the VAP prevention bundle, but we could not find any studies that examined these items, so we judged the expected harms to be slight, and the balance of desirable and undesirable effects to be “probably in favor of intervention.” A systematic review showed that 14 RCTs consistent with the patient, intervention, comparison, and outcome process were conducted, and a meta-analysis was performed using these studies.  　The effect estimates for short-term mortality (14 RCTs: N=2887) and VAP (8 RCTs: N=1527) were 37 (95% CI: 70 to 0) and 67 (95% CI: 120 to 1,000) fewer patients, respectively, with early tracheostomy compared to no tracheostomy. VAP (8 RCTs, N=1527) had a mean difference of 67 fewer people/1000 (95% CI: 120 fewer people to 5 more people), and VFD (4 RCTs, N=1243) had a mean difference of 1.2 days longer (95% CI: 0.57 days longer to 1.82 days longer). Thus, we judged the desired effect of the intervention to be “probably small.” In contrast, the effect estimates for bleeding at the tracheostomy site (5 RCTs, N=1715) and infection at the tracheostomy site (4 RCTs, N=816) were not statistically significant, with a risk difference of 7 per 1000 patients (95% CI: 5 to 30) for early tracheostomy compared with no early tracheostomy. The expected harm was judged to be “slight,” with a difference of 12 people/1000 (95% CI: 6 people decrease to 54 people increase). Thus, whether the effect of the intervention was greater than the harm was judged to be “probably in favor of the intervention.”  In addition, the composition of the VAP prevention bundle has adopted different items depending on each country and each facility.  The United States IHI developed a VAP prevention bundle in 2005 that includes the following four items 8):  1. Elevation of the head of the bed  2. Daily discontinuation of sedation and assessment of weaning from the ventilator  3. Prevention of peptic ulcer  4. Prevention of deep vein thrombosis  In a multicenter observational study, implementation of this bundle resulted in a 44.5% reduction in the incidence of VAP ^3)^. In addition, a small observational study using this bundle reported a reduction in the duration of ventilator use and ICU stay ^4)^. In 2010, daily oral care with chlorhexidine was added to the IHI VAP prevention bundle, making it a five-item bundle.  In Europe, a bundle has been developed with a combination of the following five items that differ from the IHI 10):  1. Do not change the respiratory circuit  2. Reliable hand hygiene with alcohol  3. Appropriate education and training for staff  4. Protocols for sedation and respiratory weening  5. Oral care with chlorhexidine  On the contrary, in Japan, the 2010 revision of the Japanese Society of Intensive Care Medicine’s bundle for the prevention of VAP is well known and consists of the following five items ^6)^:  1. Hand hygiene should be practiced without fail  2. Do not change the ventilator circuit frequently  3. Ensure appropriate sedation and analgesia, especially avoiding over-sedation  4. Assess daily for ability to wean from the ventilator  5. Do not manage patients in the supine position while on the ventilator.  In addition to the above, some before-and-after studies include subglottic cuff suctioning and closed suction systems in the care bundle ^2)^.  However, as mentioned above, the content of the VAP prevention bundle differs from country to country and facility to facility, and it is necessary to consider and formulate what can be implemented at each facility.  **Certainty of the evidence：**  　Satisfies seven items of the good practice statement.  **Values, balance of effect, acceptability, feasibility：**  In this CQ, the primary outcomes were ICU motality, in-hospital mortality, and occurrence of PICS and VAP, all of which were judged to have no variation in magnitude of value. The predicted harms included tracheal tube malposition and obstruction and blood pressure variability, but we could not find any studies that examined these items. Based on the above, we concluded that the balance between desirable and undesirable effects probably favors the intervention.  In addition, the VAP prevention bundle has been taken up in the National Joint Action for Medical Safety in Japan, and it is considered to be accepted by major stakeholders because it is widespread nationwide. The feasibility of the VAP prevention bundle was judged to be feasible, as many facilities are already implementing it in their daily clinical practice.  **Panel meeting：**  A recommendation was proposed for this CQ: “It has been shown that the desirable consequences of implementing a VAP prevention bundle far outweigh the undesirable consequences of the intervention.” The recommendation was based on the following questions: (1) is the statement clear and actionable, (2) is the message truly necessary, (3) is the net benefit significant and unquestionable?, (4) is the evidence for the clinical question difficult to collect and summarize, (5) are there specific issues to consider (e.g., fairness), (6) is the rationale clearly presented, and (7) would a formal GRADE-based evaluation be warranted? The panel unanimously agreed on the “good practice statement.”  In the panel meeting, there was a discussion on whether specific items for the VAP prevention bundle should be presented. However, since the implementation of the VAP prevention bundle itself is widely accepted and it is difficult to present high quality evidence to change the practices of facilities that are already implementing the bundle, it was decided not to present specific details and to add a supplementary note that the contents of the bundle should be considered by each facility.  **Note:**   The contents of the items comprising the VAP prevention bundle should be formulated by considering what can be implemented at each facility.  For example, oral care using chlorhexidine, which is a 2% chlorhexidine preparation used overseas, is not approved for use in Japan due to concerns about anaphylactic shock. |

| **Subgroup considerations** |
| --- |
| Since the risk of death due to VAP complications is likely to be high in the subgroup of severe ARDS, the benefits of active use of the bundle may be high ^12)^. |
| **Implementation considerations** |
| The previous ARDS guidelines 2016 described the epidemiology, diagnosis, pathophysiology, and diagnosis of VAP but did not set a CQ for VAP prevention bundles. The ARDS guidelines in each country also did not discuss VAP prevention bundles.  The following is a list of recommended VAP prevention bundles in each country. The United States IHI recommends the following five items ^3)^:  1. Elevation of the head of the bed  2. Daily discontinuation of sedation and assessment of weaning from the ventilator  3. Prevention of peptic ulcer  4. Prevention of deep vein thrombosis  5. Daily oral care with chlorhexidine  On the contrary, Europe recommends a combined bundle of the following five items that differs from the IHI ^5)^:  1. Do not replace the respiratory circuit  2. Reliable hand hygiene with alcohol  3. Appropriate education and training for staff  4. Protocols for sedation and respiratory weening  5. Oral care with chlorhexidine  In Japan, the Japanese Society of Intensive Care Medicine’s Ventilation-Associated Pneumonia Prevention Bundle 2010 Revision is well known and consists of the following five items ^6)^:  1. Hand hygiene should be performed without fail  2. Do not change the ventilator circuit frequently  3. Ensure appropriate sedation and analgesia, especially avoid over-sedation  4. Assess daily for ability to wean from the ventilator  5. Do not manage patients in the supine position while on the ventilator  As described above, the content of the VAP prevention bundle recommended in each country is different, and it is possible that the VAP prevention bundle implemented in each facility in Japan is also different. Therefore, to understand the current implementation rate of each item of the bundle in Japan, we conducted a web-based questionnaire survey of members of the three societies: the Japanese Respiratory Society, the Japanese Society of Respiratory Therapy, and the Japanese Society of Intensive Care Medicine (questionnaire period: August 7 to August 31, 2020; valid responses: 658). The results were as follows:  No periodic replacement of ventilator circuits: 55.4%  Not managing patients in the prone position: 68%  Avoiding deep sedation: 56.6% (when the patient is unstable) and 76% (when the patient is stable)  Evaluate daily whether the patient can be weaned from the ventilator: 67%  Oral care at least once a day: 95%  Use of endotracheal tube with suction port above cuff: 69%.  Use of closed suction catheter kit: 87%  The implementation rate of each item varied from 55.5% to 95%, suggesting that the content of VAP prevention implemented in each facility is different.  Although the items in the VAP prevention bundle are already commonly used or have been shown to have high quality evidence, the contents of the VAP prevention bundle differ among countries and facilities as described above, and it is necessary to consider what can be implemented at each facility. For example, the oral care using chlorhexidine used overseas is a 2% chlorhexidine formulation, which is not approved for use in Japan due to concerns about anaphylactic shock. It has also been pointed out that there is insufficient consideration of bundle compliance, and appropriate education for medical staff is necessary ^8)^. |

| **Monitoring and evaluation** |
| --- |
| Further information on the adverse events and cost-effectiveness of the VAP care bundle needs to be collected to implement the recommendations. In addition, it is necessary to monitor the implementation status of the guideline through the use of questionnaires and other means after the guideline is published to see if there are any other clinical problems. |
| **Research priorities** |
| It has been reported in before-and-after studies that VAP prevention bundles reduce the incidence of VAP ^1-4)^. However, the content of the VAP prevention bundle in previous studies differs from study to study, and the content of the VAP prevention bundle recommended in each country also differs ^3,5,6)^. Furthermore, no interventional studies have compared VAP prevention bundles in patients with ARDS. Future clinical studies are needed to verify the effectiveness of VAP prevention bundles in patients with ARDS. |

References

1) Khan R, Al-Dorzi HM, Al-Attas K, et al. The impact of implementing multifaceted interventions on the prevention of ventilator-associated pneumonia. Am J Infect Control. 2016;44:320-326.

2) Hawe CS, Ellis KS, Cairns CJS, Longmate A. Reduction of ventilator-associated pneumonia: active versus passive guideline implementation. Intens Care Med. 2009;35:1180-1186.

3) Resar R, Pronovost P, Haraden C, Simmonds T, Rainey T, Nolan T. Using a bundle approach to improve ventilator care processes and reduce ventilator-associated pneumonia. Jt Comm J Qual Patient Saf. 2005;31:243-248.

4) Crunden E, Boyce C, Woodman H, Bray B. An evaluation of the impact of the ventilator care bundle. Nurs Crit Care. 2005;10:242-246.

5) Contributors TVCB, Rello J, Lode H, Cornaglia G, Masterton R. A European care bundle for prevention of ventilator-associated pneumonia. Intens Care Med. 2010;36:773-780.

6) Japanese Society of Intensive Care Medicine Committee on ICU Function Assessment. Ventilation-associated pneumonia prevention bundle 2010 revised edition. [Internet]. [cited 2021 Feb 2]. Available from: <https://www.jsicm.org/pdf/2010VAP.pdf>

7) National Joint Action on Medical Safety. Action Goal 5: Safe operation and management of medical devices [Internet]. [cited 2021 Feb 2]. Available from: http://kyodokodo.jp/koudoumokuhyou/gaiyou/mokuhyou5/

8) Zilberberg MD, Shorr AF, Kollef MH. Implementing quality improvements in the intensive care unit: ventilator bundle as an example. Crit Care Med. 2009;37:305-309.

9) American Thoracic Society, Infectious Diseases Society of America. Guidelines for the management of adults with hospital-acquired, ventilator-associated, and healthcare-associated pneumonia. Am J Resp Crit Care. 2012;171:388-416.

10) Reignier J, Mercier E, Gouge AL, et al. Effect of not monitoring residual gastric volume on risk of ventilator-associated pneumonia in adults receiving mechanical ventilation and early enteral feeding: a randomized controlled trial. JAMA. 2013;309:249-256.

11) Safdar N, Dezfulian C, Collard HR, Saint S. Clinical and economic consequences of ventilator-associated pneumonia&colon; a systematic review. Crit Care Med. 2005;33:2184-2193.

12) Forel J-M, Voillet F, Pulina D, et al. Ventilator-associated pneumonia and ICU mortality in severe ARDS patients ventilated according to a lung-protective strategy. Crit Care. 2012;16:R65.

13) Ayzac L, Girard R, Baboi L, et al. Ventilator-associated pneumonia in ARDS patients: the impact of prone positioning. A secondary analysis of the PROSEVA trial. Intens Care Med. 2016;42:871-878.

14) Ministry of Health, Labour and Welfare Surveillance of Nosocomial Infection Control. Intensive Care Unit Section 2019 Annual Report [Internet]. Available from: https://janis.mhlw.go.jp/report/open_report/2019/3/3/ICU_Open_Report_201900.pdf
