# Supplementary material for: ARDS Clinical Practice Guideline 2021
Source: J Intensive Care. 2022 Jul 8;10:32. doi: 10.1186/s40560-022-00615-6 (PMC9263056; doi:10.1186/s40560-022-00615-6)
Supplement: Supplementary file 5 — Additional file 5. Contains Modified Preferred Reporting items of Systematic Reviews and Meta-Analyses (PRISMA) flow-chart, risk of bias summary, forest plots, evidence profiles, and evidence to decision table for CQ39–48 (area E) according to the GRADE system [file 40560_2022_615_MOESM5_ESM.docx]

Additional file 5

Modified Preferred Reporting items of Systematic Reviews and Meta-Analyses (PRISMA) flow-chart, risk of bias summary, forest plots, evidence profiles, and evidence to decision table for CQ39-48 (area E) according to the GRADE system

Table of contents

1. CQ39
   1. Search strategy p.3
   2. Flow diagram p.5
   3. Risk of bias p.6
   4. Forest plot p.6
   5. Evidence Profile p.6
   6. Evidence-to-Decision table p.7
2. CQ40
   1. Search strategy p.14
   2. Flow diagram p.15
   3. Risk of bias p.16
   4. Forest plot p.17
   5. Evidence Profile p.19
   6. Evidence-to-Decision table p.21
3. CQ41
   1. Search strategy p.28
   2. Flow diagram p.30
   3. Risk of bias p.31
   4. Forest plot p.32
   5. Evidence Profile p.33
   6. Evidence-to-Decision table p.35
4. CQ42
   1. Search strategy p.43
   2. Flow diagram p.44
   3. Risk of bias p.45
   4. Forest plot p.47
   5. Evidence Profile p.49
   6. Evidence-to-Decision table p.54
5. CQ43
   1. Search strategy p.64
   2. Flow diagram p.66
   3. Risk of bias p.67
   4. Forest plot p.69
   5. Evidence Profile p.71
   6. Evidence-to-Decision table p.74
6. CQ44
   1. Search strategy p.82
   2. Flow diagram p.83
   3. Risk of bias p.84
   4. Forest plot p.86
   5. Evidence Profile p.89
   6. Evidence-to-Decision table p.92
7. CQ45
   1. Search strategy p.101
   2. Flow diagram p.104
   3. Risk of bias p.105
   4. Forest plot p.107
   5. Evidence Profile p.109
   6. Evidence-to-Decision table p.112
8. CQ46
   1. Search strategy p.119
   2. Flow diagram p.121
   3. Risk of bias p.122
   4. Forest plot p.124
   5. Evidence Profile p.126
   6. Evidence-to-Decision table p.129

**CQ39 Should thrombomodulin be used in patients with ARDS?**

1. Search strategy

MEDLINE via PubMed （Search date: 2020/7/4）

| #1 | "Respiratory Distress Syndrome, Adult"[Mesh] OR "shock lung"[tiab] OR "acute respiratory distress syndrome"[tiab] OR "adult respiratory distress syndrome"[tiab] |
| --- | --- |
| #2 | Respiratory Distress Syndrome, Newborn[mh] |
| #3 | ARDS[tiab] OR ALI[tiab] |
| #4 | "Respiratory Insufficiency"[Mesh] OR "Respiratory Failure"[tiab] OR "Respiratory Depression"[tiab] OR "Ventilatory Depression"[tiab] OR "acute respiratory failure"[tiab] |
| #5 | "Acute Lung Injury"[Mesh] OR "lung injur*"[tiab] |
| #6 | "Severe Acute Respiratory Syndrome"[Mesh] OR "severe acute respiratory syndrome"[tiab] |
| #7 | #1 OR #2 OR #3 OR #4 OR #5 OR #6 |
| #8 | "thrombomodulin"[mh] |
| #9 | thrombomodulin[tiab] |
| #10 | rTM[tiab] |
| #11 | ART123 [Supplementary Concept] |
| #12 | ART123[tiab] |
| #13 | Recomodulin[tiab] |
| #14 | #8 OR #9 OR #10 OR #11 OR #12 OR #13 |
| #15 | #7 AND #14 |
| #16 | Controlled clinical trial[Publication Type] |
| #17 | randomized[Title/Abstract] |
| #18 | placebo[Title/Abstract] |
| #19 | drug therapy[SH] |
| #20 | randomly [tiab] |
| #21 | trial[tiab] |
| #22 | groups [tiab] |
| #23 | "systematic review"[Publication Type] |
| #24 | "meta-analysis"[Publication Type] |
| #25 | "review"[Publication Type] |
| #26 | #16 OR #17 OR #18 OR #19 OR #20 OR #21 OR #22 OR #23 OR #24 OR #25 |
| #27 | #15 AND #26 |
| #28 | animals [mh] NOT humans [mh] |
| #29 | #27 NOT #28 |

CENTRAL（Search date: 2020/7/4）

| #1 | [mh "Respiratory Distress Syndrome, Adult"] OR "shock lung":ti,ab OR "acute respiratory distress syndrome":ti,ab OR "adult respiratory distress syndrome":ti,ab |
| --- | --- |
| #2 | [mh "Respiratory Distress Syndrome, Newborn"] |
| #3 | ARDS OR ALI |
| #4 | [mh "Respiratory Insufficiency"] OR "Respiratory Failure":ti,ab OR "Respiratory Depression":ti,ab OR "Ventilatory Depression":ti,ab OR "acute respiratory failure":ti,ab |
| #5 | [mh "Acute Lung Injury"] OR "lung injury":ti,ab |
| #6 | [mh "Severe Acute Respiratory Syndrome"] OR "severe acute respiratory syndrome":ti,ab |
| #7 | {OR #1-#6} |
| #8 | [mh thrombomodulin] |
| #9 | thrombomodulin:ti,ab |
| #10 | rTM:ti,ab |
| #11 | ART123:ti,ab |
| #12 | Recomodulin:ti,ab |
| #13 | {OR #8-#12} |
| #14 | #7 AND #13 |

1. Flow diagram

**Identification**

0 Studies included in qualitative synthesis

0 Full-text articles assessed for eligibility

71 records after duplicates removed

80 records identified through database searching

80 records identified through database searching

Medline via PubMed (n=49)

CENTRAL (n=21)

Igaku-Chuo-Zasshi (n=10)

0 additional records identified through other sources

0 Studies included in quantitative synthesis (meta-analysis)

0 Full-text articles excluded, with reasons:

・Wrong language (n=0)

・Wrong study design (n=0)

・Wrong population (n=0)

・Wrong intervention (n=0)

Duplicates

n=9

71 records excluded

**Included**

**Eligibility**

**Screening**

1. Risk of bias

Not applicable

1. Forest plot

Not applicable

1. Evidence profile

Not applicable

1. Evidence-to-Decision table

| Question | |
| --- | --- |
| CQ39：Should thrombomodulin be used in patients with ARDS? | |
| **Population:** | Adult patients with hypoxic respiratory failure |
| **Intervention:** | Thrombomodulin |
| **Comparison:** | No thrombomodulin |
| **Main outcomes:** | Mortality, duration of mechanical ventilation, serious bleeding |
| **Setting:** | Emergency room (ER) or intensive care unit (ICU) |
| **Perspective:** | Individual |
| **Background:** | ARDS is a condition caused by inflammation that spreads to the lungs due to various causes ^1)^. Thrombomodulin, which is indicated for the treatment of disseminated intravascular coagulation (DIC), has been reported to have anticoagulant and anti-inflammatory effects. Therefore, thrombomodulin may be effective in suppressing inflammation in ARDS. However, no clinical studies have been reported on thrombomodulin in ARDS alone. Therefore, a systematic review of thrombomodulin would be an important clinical issue but is not possible at this time. |
| **Conflict of interests:** | None |

# Assessment

| Problem Is the problem a priority? | | |
| --- | --- | --- |
| Judgment | research evidence | ADDITIONAL CONSIDERATIONS |
| ○ No  ○ Probably no  ○Probably yes  ● Yes  ○ Varies  ○ Do not know | ARDS is a condition caused by inflammation that spreads to the lungs due to various causes ^1)^. Thrombomodulin, which is indicated for the treatment of DIC, has been reported to have anticoagulant and anti-inflammatory effects. Therefore, thrombomodulin may be effective in reducing inflammation in ARDS. It was judged that addressing the CQ on thrombomodulin is an important clinical issue with high priority |  |
| Desirable effects How substantial are the desirable anticipated effects? | | |
| Judgment | research evidence | ADDITIONAL CONSIDERATIONS |
| ○ Trivial  ○Small  ○ Moderate  ○ Large  ○ Varies  ●Do not know | Integrated into the certainty of the evidence. |  |
| Undesirable effects How substantial are the undesirable anticipated effects? | | |
| Judgment | research evidence | ADDITIONAL CONSIDERATIONS |
| ○ Large  ○ Moderate  ○ Small  ○ Trivial  ○ Varies  ● Do not know | Integrated into the certainty of the evidence. |  |
| Certainty of evidence What is the overall certainty of the evidence of effects? | | |
| Judgment | research evidence | ADDITIONAL CONSIDERATIONS |
| ○ Very low  ○ Low  ○ Moderate  ○ High  ● No included studies | A systematic review was conducted, but no randomized controlled trials (RCTs) were consistent with the patient, intervention, comparison, and outcome (PICO) process. In a mouse ARDS model, thrombomodulin has been reported to inhibit survival and ARDS progression by controlling inflammation ^2)^. In another LPS-induced mouse ARDS model, thrombomodulin was also reported to regulate inflammation ^3)^. In clinical studies, treatment with thrombomodulin and corticosteroids in acute exacerbations of idiopathic interstitial pneumonia has been reported to improve survival and respiratory function ^4)^. As for septic DIC, the Japanese Guideline for the Treatment of Sepsis 2020 weakly recommends the administration of thrombomodulin to patients with septic DIC because the benefits of death and DIC withdrawal outweigh the harms of hemorrhagic complications. |  |
| Values Is there important uncertainty about or variability in how much people value the main outcomes? | | |
| Judgment | research evidence | ADDITIONAL CONSIDERATIONS |
| ○ Important uncertainty or variability  ○ Possibly important uncertainty or variability  ● Probably no important uncertainty or variability  ○ No important uncertainty or variability | Probably no important uncertainty or variability. |  |
| Balance of effects Does the balance between desirable and undesirable effects favor the intervention or the comparison? | | |
| Judgment | research evidence | ADDITIONAL CONSIDERATIONS |
| ○ Favors the comparison  ○Probably favors the comparison  ○ Does not favor either the intervention or the comparison  ○ Probably favors the intervention  ○Favors the intervention  ○ Varies  ●Do not know | No studies. |  |
| Acceptability Is the intervention acceptable to key stakeholders? | | |
| Judgment | research evidence | ADDITIONAL CONSIDERATIONS |
| ○ No  ● Probably no  ○ Probably yes  ○ Yes  ○ Varies  ○ Do not know | Considering the lack of evidence and the off-label use, it is probably unacceptable. |  |
| Feasibility Is the intervention feasible to implement? | | |
| Judgment | research evidence | ADDITIONAL CONSIDERATIONS |
| ○ No  ○Probably no  ○ Probably yes  ○ Yes  ● Varies  ○ Do not know | As mentioned above, its administration for ARDS is not covered by insurance and requires approval from the ethics committee. Therefore, it is difficult to make a general statement. |  |

# Summary of judgment

|  | **Judgment** | | | | | | |
| --- | --- | --- | --- | --- | --- | --- | --- |
| **PROBLEM** | No | Probably no | Probably yes | Yes |  | Varies | Do not know |
| **DESIRABLE EFFECTS** | Trivial | Small | Moderate | Large |  | Varies | Do not know |
| **UNDESIRABLE EFFECTS** | Large | Moderate | Small | Trivial |  | Varies | Do not know |
| **CERTAINTY OF EVIDENCE** | Very low | Low | Moderate | High |  |  | No included studies |
| **VALUES** | Important uncertainty or variability | Possibly important uncertainty or variability | Probably no important uncertainty or variability | No important uncertainty or variability |  |  |  |
| **BALANCE OF EFFECTS** | Favors the comparison | Probably favors the comparison | Does not favor either the intervention or the comparison | Probably favors the intervention | Favors the intervention | Varies | Do not know |
| **ACCEPTABILITY** | No | Probably no | Probably yes | Yes |  | Varies | Do not know |
| **FEASIBILITY** | No | Probably no | Probably yes | Yes |  | Varies | Do not know |

# Type of Recommendation

| Strong recommendation against the intervention | Conditional recommendation against the intervention | Conditional recommendation for either the intervention or the comparison | Conditional recommendation for the intervention | Strong recommendation for the intervention |
| --- | --- | --- | --- | --- |
| ○ | ○ | ○ | ○ | ○ |

# Conclusions

| Recommendation |
| --- |
| **We could not provide a recommendation for the administration of thrombomodulin in patients with ARDS (in our practice statement).** |
|  |
| Justification |
| **Question**: Should thrombomodulin be used in patients with ARDS?  **Patient:** Adult patients with hypoxic respiratory failure.  **Intervention:** Thrombomodulin  **Comparison:** No thrombomodulin  **Outcome：** Mortality, duration of mechanical ventilation, serious bleeding  **Explanation:**  In a bovine ARDS model, it has been reported that thrombomodulin suppresses survival and ARDS progression by controlling inflammation ^2)^. In another LPS-induced murine ARDS model, thrombomodulin was also reported to regulate inflammation ^3)^. In clinical studies, treatment with thrombomodulin and corticosteroids in acute exacerbations of idiopathic interstitial pneumonia has been reported to improve survival and respiratory function ^4).^ Regarding septic DIC, the Japanese Guideline for the Treatment of Sepsis 2020 weakly recommends the administration of thrombomodulin to patients with septic DIC because the benefits of death and DIC withdrawal outweigh the harms of hemorrhagic complications. However, a systematic review was conducted, and no RCTs were found to be consistent with the PICO process. Therefore, we cannot provide a clear recommendation for this CQ. Therefore, this CQ is not an evidence-based recommendation but only a description of current practice.  **Summary of evidence**: No studies  **Certainty of the evidence**: Since there are no relevant studies, the quality of evidence cannot be assessed.  **Values, balance of effects, acceptability, feasibility:**  We do not know about the balance of effects because there are no relevant studies. Acceptance is probably unacceptable considering the lack of evidence and off-label use. In addition, the administration of the drug for ARDS is not covered by insurance and requires the approval of an ethics committee. Therefore, it is difficult to say whether it is feasible. Even in the case of DIC, the burden on patients and payment institutions may increase depending on the coding used.  **Panel meeting:**  In the pre-vote, the modified Delphi method resulted in a median of 9.0 and a disagreement index of 0.1316 for “Cannot provide a recommendation for the use of thrombomodulin in patients with ARDS.”  At the panel meeting, it was suggested that "no insurance coverage" included in the recommendation be added as an additional item. It was also suggested that the word "acceptance" be changed to "probably unacceptable." |

| Subgroup considerations |
| --- |
| None |
| Implementation considerations |
| Since the procedure is not covered by insurance for ARDS, it must be performed after appropriate preparations, such as obtaining approval from the hospital's ethics committee. |

| Monitoring and evaluation |
| --- |
| It is necessary to monitor the extent to which thrombomodulin is used for ARDS in clinical practice in Japan through the use of questionnaires and other means after the guidelines are published. |
| Research priorities |
| Clinical trials and RCTs on the effects of thrombomodulin on ARDS are needed. |

References

1．Pelosi P, D'Onofrio D, Chiumello D, Paolo S, Chiara G, Capelozzi VL, et al. Pulmonary and extrapulmonary acute respiratory distress syndrome are different. Eur Respir J. 2003;42:48s-56s. PMID: 12946001.

2. Kudo D, Toyama M, Aoyagi T, Akahori Y, Yamamoto H, Ishii K, et al. Involvement of high mobility group box 1 and the therapeutic effect of recombinant thrombomodulin in a mouse model of severe acute respiratory distress syndrome. Clin Exp Immunol. 2013;173(2): 276-287. PMID: 23607598.

3. Suzuki K, Okada H, Takemura G, Takada C, Tomita H, Yano H, et al. Recombinant thrombomodulin protects against LPS-induced acute respiratory distress syndrome via preservation of pulmonary endothelial glycocalyx. Br J Pharmacol. 2020;177(17): 4021-4033. PMID: 32497259.

4. Hayakawa S, Matsuzawa Y, Irie T, Rikitake H, Okada N, Suzuki Y. Efficacy of recombinant human soluble thrombomodulin for the treatment of acute exacerbation of idiopathic pulmonary fibrosis: a single arm, non-randomized prospective clinical trial. Multidiscip Respir Med. 2016;11:38. PMID: 27826444.

**CQ40 Should nitric oxide inhalation be used for patients with ARDS?**

1. Search strategy

MEDLINE via PubMed（Search date: 2020/7/4）

| #1 | Respiratory Distress Syndrome, Adult[mh] OR Respiratory Distress Syndrome, Newborn[mh] OR ARDS[tiab] OR Acute Lung Injury[tiab] OR Acute Hypoxemic Respiratory Failure[tiab] |
| --- | --- |
| #2 | Nitric oxide[mh] OR Nitric oxide[tiab] |
| #3 | #1 AND #2 |
| #4 | (randomized controlled trial [pt] OR controlled clinical trial [pt] OR randomized [tiab] OR placebo [tiab] OR drug therapy [sh] OR randomly [tiab] OR trial [tiab] OR groups [tiab]) NOT (animals[mh] NOT humans[mh]) |
| #5 | #1 AND #2 AND #4 |

CENTRAL（Search date: 2020/7/4）

| #1 | MeSH descriptor: [Respiratory Distress Syndrome, Adult] explode all trees |
| --- | --- |
| #2 | MeSH descriptor: [Respiratory Distress Syndrome, Newborn] explode all trees |
| #3 | (ARDS):ti,ab,kw |
| #4 | ("Acute Lung Injury"):ti,ab,kw |
| #5 | ("Acute Hypoxemic Respiratory Failure"):ti,ab,kw |
| #6 | ("acute respiratory distress syndrome"):ti,ab,kw |
| #7 | {OR #1-#6} |
| #8 | MeSH descriptor: [Nitric Oxide] explode all trees |
| #9 | ("nitric oxide"):ti,ab,kw |
| #10 | #8 OR #9 |
| #11 | #7 AND #10 |

1. Flow diagram

**Identification**

9 Studies included in qualitative synthesis

45 Full-text articles assessed for eligibility

720 records after duplicates removed

828 records identified through database searching

828 records identified through database searching

Medline via PubMed (n=643)

CENTRAL (n=170)

Igaku-Chuo-Zasshi (n=15)

0 additional records identified through other sources

7 Studies included in quantitative synthesis (meta-analysis)

36 Full-text articles excluded, with reasons:

・Wrong language (n=2)

・Wrong study design (n=25)

・Wrong population (n=3)

・Wrong intervention (n=6)

Duplicates

n=108

675 records excluded

**Included**

**Eligibility**

**Screening**

1. Risk of bias

Short-term mortality Duration of mechanical ventilation


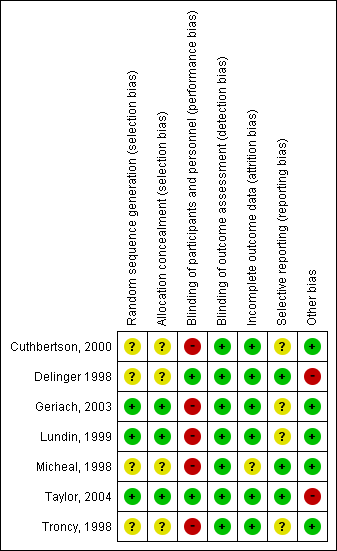

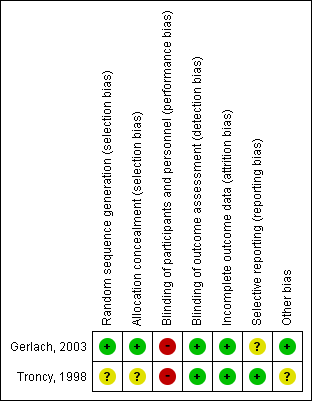


Length of ICU stay Kidney injury


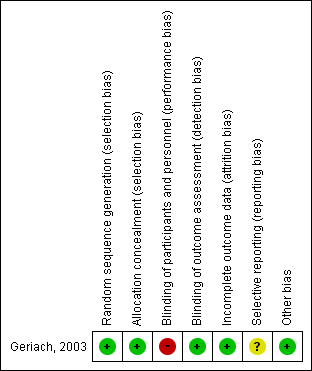

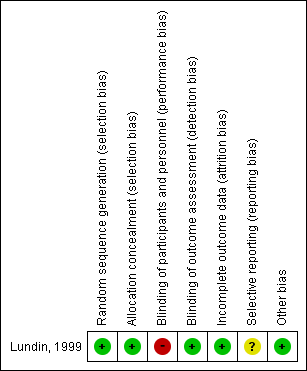


1. Forest plot

Short-term mortality


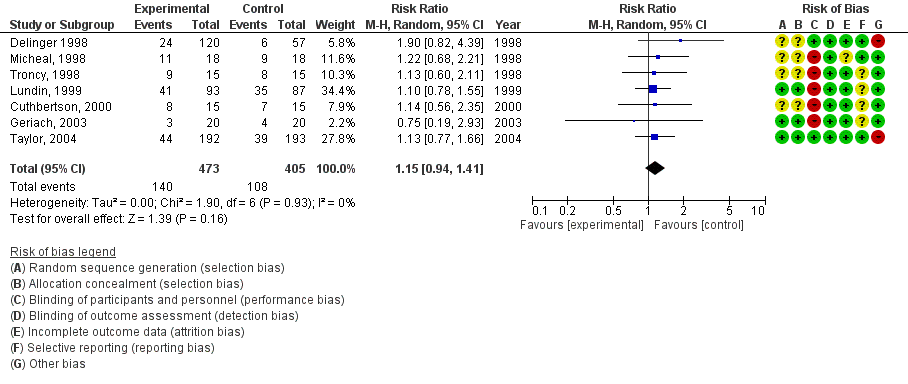


Duration of mechanical ventilation


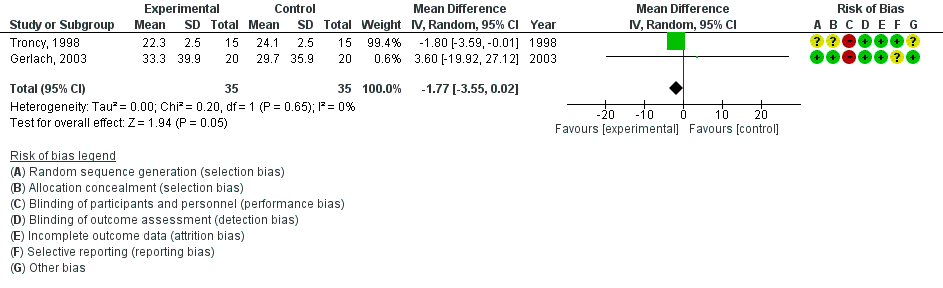


Length of ICU stay


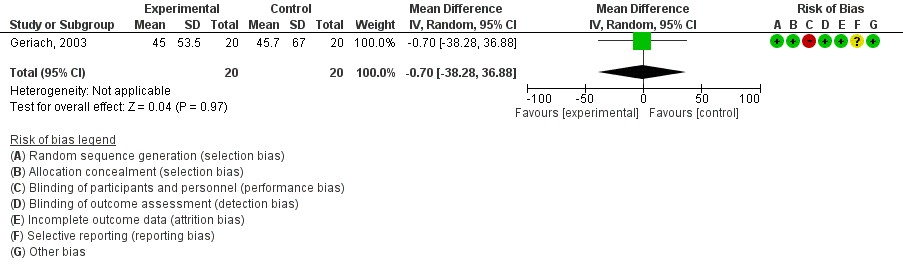


Kidney injury


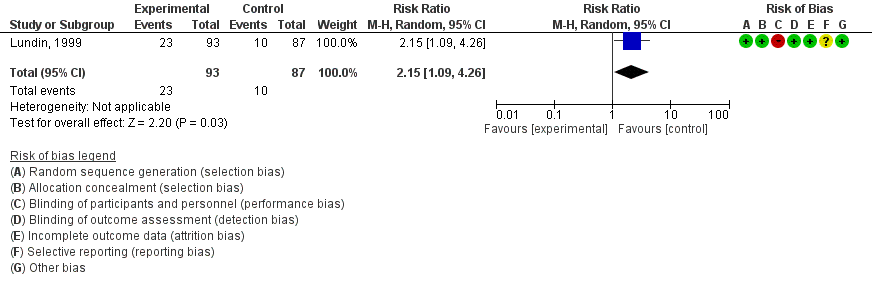


1. Evidence profile

| **Assessment of certainty** | | | | | | | **№ of patients** | | **Effect** | | | **Certainty** | **Importance** |
| --- | --- | --- | --- | --- | --- | --- | --- | --- | --- | --- | --- | --- | --- |
| **№ of studies** | **Study design** | **Risk of bias** | **Inconsistency** | **Indirectness** | **Imprecision** | **Others** | **Use NO** | **Not use NO** | **Relative index (95% CI)** | **Absolute index (95% CI)** | |  |  |
| **Short-term mortality** | | | | | | | | | | | | | |
| 7 | RCT | Serious ^a^ | Not serious | Not serious | Serious ^b^ | None | 140/473 (29.6%) | 108/405 (26.7%) | RR 1.15 (0.94 to 1.41) | **40 more per 1000**  (16 fewer ～ 109 more) | | ⨁⨁◯◯ Low | Critical |
| **Duration of mechanical ventilation** | | | | | | | | | | | | | |
| 2 | RCT | Serious ^c^ | Not serious | Not serious | Very serious ^d^ | None | 35 | 35 | - | MD 1.77 days shorter (3.55 shorter ～ 0.02 longer) | | ⨁◯◯◯ Very low | Critical |
| **Length of ICU stay** | | | | | | | | | | | | | |
| 1 | RCT | Serious ^e^ | Not serious | Not serious | Very serious ^d^ | None | 20 | 20 | - | MD 0.7days shorter (38.28 shorter ～ 36.88 longer) | | ⨁◯◯◯ Very low | Critical |
| **Kidney injury** | | | | | | | | | | | | | |
| 1 | RCT | Serious ^e^ | Not serious | Not serious | Serious ^f^ | None | 23/93 (24.7%) | 10/87 (11.5%) | RR 2.15 (1.09 to 4.26) | **132 more per 1000** (10 more ～ 375 more) | | ⨁⨁◯◯ Low | Critical |
| **Length of hospital stay** | | | | | | | | | | | | | |
| 0 | - | - | - | - | - | - | - | - | Not estimate | | **-** | - | Critical |
| **Methemoglobinemia** | | | | | | | | | | | | | |
| 0 | - | - | - | - | - | - | - | - | Not estimate | | **-** | - | Critical |

**CI:** confidence interval; **RR:** risk ratio; **MD:** mean difference; RCT: randomized controlled trial

Length of hospital stay, methemoglobinemia were not included in the evidence profile because the outcomes are not reported.

#### Explanation

a. The risk of bias was downgraded by one level to "serious" due to moderate risk.

b. The 95% confidence interval straddles the threshold for clinical judgment; therefore, it was downgraded by one level.

c. In the risk of bias, the risk was downgraded by one level because blinded in all studies is a high risk and the risk of generating random allocation order and concealing allocation in the study by Troncy et al. is unknown.

d. Two steps down because the optimal information size (OIS) was not met and the 95% confidence interval straddled the threshold for clinical judgment.

e. In the risk of bias, blinding of study participants and treatment providers was a high risk and was downgraded by one level.

f. It was downgraded by one level because the OIS was not met.

1. Evidence-to-Decision table

| Question | |
| --- | --- |
| CQ40： Should nitric oxide inhalation be used for patients with ARDS? | |
| **Population:** | Adult patients with ARDS |
| **Intervention:** | Use NO |
| **Comparison:** | Not use NO |
| **Main outcomes:** | Mortality, duration of mechanical ventilation, length of intensive care unit (ICU) stay, length of hospital stay, kidney injury, methemoglobinemia |
| **Setting:** | Emergency room (ER) or ICU |
| **Perspective:** | Individual |
| **Background:** | ARDS is a condition caused by inflammation spreading to the lungs due to various causes ^1)^. The pathogenesis involves various factors such as alveolar epithelial damage, increased pulmonary vascular resistance due to hypoxic pulmonary vasoconstriction, imbalance in ventilation/blood flow ratio, and alveolar surfactant dysfunction. Therefore, inhalation of NO, which has a pulmonary vasodilator effect, may be effective, and there are reports of improved oxygenation with NO inhalation ^2)^. There are reports of improved oxygenation with NO inhalation ^2)^. NO is sometimes used as a rescue therapy when oxygenation does not improve with normal ventilation settings, but it is not indicated in Japan. On the contrary, there are reports that NO inhalation does not improve the duration of ventilation or mortality^3)^. Therefore, conducting a systematic review of NO inhalation is an important clinical issue. |
| **Conflict of interests:** | None |

# Assessment

| Problem Is the problem a priority? | | |
| --- | --- | --- |
| Judgment | research evidence | ADDITIONAL CONSIDERATIONS |
| ○ No  ○ Probably no  ● Probably yes  ○ Yes  ○ Varies  ○ Do not know | ARDS is a condition caused by inflammation that has spread to the lungs from various sources. The pathogenesis involves various factors such as alveolar epithelial damage, increased pulmonary vascular resistance due to hypoxic pulmonary vasoconstriction, ventilation/blood flow ratio imbalance, and alveolar surfactant dysfunction. Therefore, inhalation of NO, which has a pulmonary vasodilator effect, may be effective, and there are reports of improved oxygenation by NO inhalation. On the contrary, there are reports that NO inhalation does not improve the duration of ventilation or mortality. Therefore, clarifying the balance of benefits and harms associated with NO inhalation is an important clinical issue. |  |
| Desirable effects How substantial are the desirable anticipated effects? | | |
| Judgment | research evidence | ADDITIONAL CONSIDERATIONS |
| ○ Trivial  ● Small  ○ Moderate  ○ Large  ○ Varies  ○ Do not know | A meta-analysis was performed using seven randomized controlled trials (RCTs). The estimated values of desirable anticipated effects were as follows: duration of mechanical ventilation yielded an MD of 1.77 days shorter (2 RCTs: N=77) (3.55 shorter to 0.02 longer); length of ICU stay yielded an MD of 0.7 days shorter (1 RCT: N=40) (38.28 shorter to 36.88 longer). From the above, it was judged that the desirable anticipated effect was “small.” |  |
| Undesirable effects How substantial are the undesirable anticipated effects? | | |
| Judgment | research evidence | ADDITIONAL CONSIDERATIONS |
| ○ Large  ● Moderate  ○ Small  ○ Trivial  ○ Varies  ○ Do not know | The estimated values of the undesirable anticipated effects were as follows: mortality yielded an RD of 40 more per 1000 (7 RCTs: N=878) (95% CI: 16 fewer to 109 more); kidney injury yielded an RD of 132 more per 1000 (1 RCT: N=180) (10 more to 375 more). From the above, it was judged that the undesirable anticipated effect was “moderate.” |  |
| Certainty of evidence What is the overall certainty of the evidence of effects? | | |
| Judgment | research evidence | ADDITIONAL CONSIDERATIONS |
| ● Very low  ○ Low  ○ Moderate  ○ High  ○ No included studies | \| **Outcome** \| **Importance** \| **Certainty of the evidence** \| \| --- \| --- \| --- \| \| \| \| Mortality \| Critical \| ⨁⨁◯◯ \| \| Low \| \| Duration of mechanical ventilation \| Critical \| ⨁◯◯◯ \| \| Very low \| \| Length of ICU stay \| Critical \| ⨁◯◯◯ \| \| Very low \| \| Kidney injury \| Critical \| ⨁⨁◯◯ \| \| Low \| \| Length of hospital stay \| Critical \| - \| \| - \| \| Methemoglobinemia \| Critical \| - \| \| - \|   No RCTs reported the length of hospital stay and methemoglobinemia as outcomes.  **Overall certainty of the evidence:**  The direction of the desirable and undesirable effects was not consistent. Thus, the overall certainty of the evidence was judged to be “very low.” |  |
| Values Is there important uncertainty about or variability in how much people value the main outcomes? | | |
| Judgment | research evidence | ADDITIONAL CONSIDERATIONS |
| ○ Important uncertainty or variability  ○ Possibly important uncertainty or variability  ○ Probably no important uncertainty or variability  ●  No important uncertainty or variability | No important uncertainty or variability. |  |
| Balance of effects Does the balance between desirable and undesirable effects favor the intervention or the comparison? | | |
| Judgment | research evidence | ADDITIONAL CONSIDERATIONS |
| ○ Favors the comparison  ●Probably favors the comparison  ○ Does not favor either the intervention or the comparison  ○ Probably favors the intervention  ○Favors the intervention  ○ Varies  ○ Do not know | \| Outcome \| Comparison \| Intervention  (No) \| Absolute difference \| Relative effect RR (95% CI) \| \| --- \| --- \| --- \| --- \| --- \| \| Mortality \| 108/405  (26.7%) \| 140 /473 (29.6%) \| 40 more/ 1,000  (16 fewer～109 more) \| 1.15  (0.94～1.41) \| \| Duration of mechanical ventilation \| － \| － \| MD 1.77days shorter  (3.55 shorter ～0.02 longer) \| － \| \| Length of ICU stay \| － \| － \| MD 0.7 days shorter  (38.28 shorter～36.88 longer) \| － \| \| Kidney injury \| 10/87  (11.5%) \| 23/93 (24.7%) \| 132 more/1,000  (10 more～375 more) \| 2.15  (1.09～4.26) \| \| Length of hospital stay \| － \| － \| No estimate \| － \| \| Methemoglobinemia \| － \| － \| No estimate \| － \|   From the above, we concluded that the balance between benefits and harms is “Probably favors the comparison.” |  |
| Acceptability Is the intervention acceptable to key stakeholders? | | |
| Judgment | research evidence | ADDITIONAL CONSIDERATIONS |
| ● No  ○ Probably no  ○ Probably yes  ○ Yes  ○ Varies  ○ Do not know | The harm from the intervention outweighed the benefit and was deemed unacceptable due to off-label use, increased costs, and the need for specialized equipment. |  |
| Feasibility Is the intervention feasible to implement? | | |
| Judgment | research evidence | ADDITIONAL CONSIDERATIONS |
| ○ No  ● Probably no  ○ Probably yes  ○ Yes  ○ Varies  ○ Do not know | Since this is an off-label use, it requires the approval of the ethics committee at the institution. In addition, the number of medical institutions that can perform the procedure is expected to be limited due to the need to prepare special equipment. |  |

# Summary of judgment

|  | **Judgment** | | | | | | |
| --- | --- | --- | --- | --- | --- | --- | --- |
| **PROBLEM** | No | Probably no | Probably yes | Yes |  | Varies | Do not know |
| **DESIRABLE EFFECTS** | Trivial | Small | Moderate | Large |  | Varies | Do not know |
| **UNDESIRABLE EFFECTS** | Large | Moderate | Small | Trivial |  | Varies | Do not know |
| **CERTAINTY OF EVIDENCE** | Very low | Low | Moderate | High |  |  | No included studies |
| **VALUES** | Important uncertainty or variability | Possibly important uncertainty or variability | Probably no important uncertainty or variability | No important uncertainty or variability |  |  |  |
| **BALANCE OF EFFECTS** | Favors the comparison | Probably favors the comparison | Does not favor either the intervention or the comparison | Probably favors the intervention | Favors the intervention | Varies | Do not know |
| **ACCEPTABILITY** | No | Probably no | Probably yes | Yes |  | Varies | Do not know |
| **FEASIBILITY** | No | Probably no | Probably yes | Yes |  | Varies | Do not know |

# Type of Recommendation

| Strong recommendation against the intervention | Conditional recommendation against the intervention | Conditional recommendation for either the intervention or the comparison | Conditional recommendation for the intervention | Strong recommendation for the intervention |
| --- | --- | --- | --- | --- |
| ○ | ● | ○ | ○ | ○ |

# Conclusions

| Recommendation |
| --- |
| **We suggest not to administer NO to patients with ARDS**  **(conditional recommendation/Very low: GRADE 2D).**  **Note: This does not preclude the use of NO as a rescue therapy in facilities already using it.** |
|  |
| Justification |
| **Question:** Should nitric oxide inhalation be used for patients with ARDS?  **Patients:**Adult patients with hypoxic respiratory failure  **Intervention:**Use NO  **Comparison:** Not use NO  **Outcome:**Mortality, duration of mechanical ventilation, length of ICU stay, kidney injury  **Summary of evidence**: The estimated values of desirable anticipated effects were as follows: duration of mechanical ventilation yielded an MD of 1.77 days shorter (2 RCTs: N=77) (3.55 shorter to 0.02 longer); length of ICU stay yielded an MD of 0.7 days shorter (1 RCT: N=40) (38.28 shorter to 36.88 longer). From the above, it was judged that the desirable anticipated effect was “small.” On the contrary, the estimated values of the undesirable anticipated effects were as follows: mortality yielded an RD of 40 more per 1000 (7 RCTs: N=878) (95% CI: 16 fewer to 109 more); kidney injury yielded an RD of 132 more per 1000 (1 RCT: N=180) (10 more to 375 more). From the above, it was judged that the undesirable anticipated effect was moderate.  **Certainty of the evidence**: The direction of desirable and undesirable effects was not consistent. Thus, the overall certainty of the evidence was judged to be “very low.”  **Values, balance of effects, acceptability, feasibility**：　Regarding values, we found that, in general, the value for death was high, with little variability. Regarding the balance of effects, we judged that the harms outweighed the benefits of this intervention even when considering the point estimates, upper and lower confidence intervals. Regarding acceptance, we judged that the harms of the intervention outweighed the benefits and that the intervention was unacceptable due to its off-label use, increased costs, and the need for dedicated equipment. As for feasibility, it is expected that the number of medical institutions that can implement the intervention will be limited because it will be used off-label and will require approval from the ethics committee at the institution and dedicated facilities.  **Panel meeting：**  In the pre-vote, the “recommended text proposal” had a median score of 9.0 and a disagreement index of 0.0497, according to the modified Delphi method.  The panel meeting was reminded that the previous guideline recommended not to use NO (GRADE 1C), but this time it would be changed to suggest not to use NO (GRADE 2D) due to the very low certainty of the evidence and that clinical trials of NO in patients with COVID-19 are ongoing.  As a result, the panel meeting finally reached a consensus with the result of the pre-vote without a re-vote being required. |

| Subgroup considerations |
| --- |
| None |
| Implementation considerations |
| In the previous ARDS guideline 2016, it was judged to be strongly discouraged (GRADE 1C), but in this guideline, it has been changed to suggest that it should not be used (GRADE 2D) due to the very low certainty of the evidence.  Since this is an off-label use, approval by the ethics committee at the institution is required. |

| Monitoring and evaluation |
| --- |
| It is necessary to collect further information on the adverse events and cost-effectiveness as clinical problems in implementing the recommendations. In addition, it is necessary to monitor the implementation status of the guideline with questionnaires and other means after the guideline is published to see if there are any other clinical problems. |
| Research priorities |
| The RCTs employed in this meta-analysis were conducted between 1998 and 2004. If the use of NO is to be reconsidered in patients with ARDS in the future, it is hoped that high quality RCTs will be conducted. We must also wait for the results of RCTs to determine the usefulness of inhaled NO therapy (https://clinicaltrials.gov/ct2/show/NCT04306393) in ARDS patients with COVID-19. |

References

1. Pelosi P, D'Onofrio D, Chiumello D, Paolo S, Chiara G, Capelozzi VL, et al. Pulmonary and extrapulmonary acute respiratory distress syndrome are different. Eur Respir J. 2003;42:48s-56s. PMID: 12946001.

2. Park KJ, Lee YJ, Oh YJ, Lee KS, Sheen SS, Hwang SC. Combined effects of inhaled nitric oxide and a recruitment maneuver in patients with acute respiratory distress syndrome. Yonsei Med J. 2003;44(2):219-226. PMID: 12728461.

3. Taylor RW, Zimmerman JL, Dellinger RP, Straube RC, Criner GJ, Davis K Jr, et al. Low-dose inhaled nitric oxide in patients with acute lung injury: a randomized controlled trial. JAMA. 2004;291(13):1603-1609. PMID: 15069048.

**CQ41 Should sivelestat be used for patients with ARDS?**

1. Search strategy

MEDLINE via PubMed （Search date: 2020/6/3）

| #1 | Respiratory Distress Syndrome, Adult [mh] |
| --- | --- |
| #2 | Respiratory Distress Syndrome, Newborn[mh] |
| #3 | Acute lung injury [mh] |
| #4 | ALI [tiab] OR ARDS [tiab] |
| #5 | Acute lung injur* [tiab] OR acute respiratory distress [tiab] OR acute respiratory failure[tiab] |
| #6 | (Severe[tiab] OR critical*[tiab]) AND (respiratory[tiab] OR hypox* [tiab]) |
| #7 | #1 OR #2 OR #3 OR #4 OR #5 OR #6 |
| #8 | Leukocyte elastase[mh] |
| #9 | "leukocyte elastase"[tiab] |
| #10 | sivelestat [Supplementary Concept] |
| #11 | sivelestat [tiab] |
| #12 | "Neutrophil elastase"[tiab] |
| #13 | #8 OR #9 OR #10 OR #11 OR #12 |
| #14 | #7 AND #13 |
| #15 | animals [mh] NOT humans [mh] |
| #16 | #14 NOT #15 |
| #17 | Controlled clinical trial[Publication Type] |
| #18 | randomized[Title/Abstract] |
| #19 | placebo[Title/Abstract] |
| #20 | randomly [tiab] |
| #21 | trial[tiab] |
| #22 | groups [tiab] |
| #23 | ”Follow-Up Studies”[Mesh] |
| #24 | "systematic review"[Publication Type] |
| #25 | "meta-analysis"[Publication Type] |
| #26 | "review"[Publication Type] |
| #27 | #17 OR #18 OR #19 OR #20 OR #21 OR #22 OR #23 OR #24 OR #25 OR #26 |
| #28 | #16 AND #27 |

CENTRAL （Search date: 2020/6/4）

| #1 | [mh "Respiratory Distress Syndrome, Adult"] |
| --- | --- |
| #2 | [mh "Respiratory Distress Syndrome, Newborn"] |
| #3 | [mh "Acute lung injury"] |
| #4 | ALI:ti,ab OR ARDS:ti,ab |
| #5 | (Acute NEXT lung NEXT injur*:ti,ab) OR "acute respiratory distress":ti,ab OR "acute respiratory failure":ti,ab |
| #6 | (Severe:ti,ab OR critical*:ti,ab) AND (respiratory:ti,ab OR hypox*:ti,ab) |
| #7 | {OR #1-#6} |
| #8 | [mh "Leukocyte elastase"] |
| #9 | "leukocyte elastase":ti,ab |
| #10 | sivelestat:ti,ab |
| #11 | "Neutrophil elastase":ti,ab |
| #12 | {OR #8-#11} |
| #13 | #7 AND #12 |
| #14 | [mh animals] NOT [mh humans] |
| #15 | #13 NOT #14 |

1. Flow diagram

**Identification**

9 Studies included in qualitative synthesis

14 Full-text articles assessed for eligibility

245 records after duplicates removed

285 records identified through database searching

285 records identified through database searching

Medline via PubMed (n=158)

CENTRAL (n=74)

Igaku-Chuo-Zasshi (n=53)

0 additional records identified through other sources

5 Studies included in quantitative synthesis (meta-analysis)

5 Full-text articles excluded, with reasons:

・Wrong study design (n=3)

・Wrong population/intervention (n=2)

Duplicates

n=40

231 records excluded

**Included**

**Eligibility**

**Screening**

1. Risk of bias


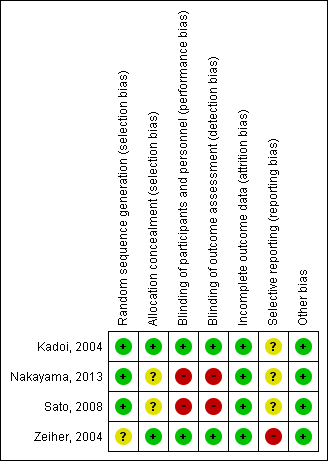
Mortality Duration of mechanical ventilation


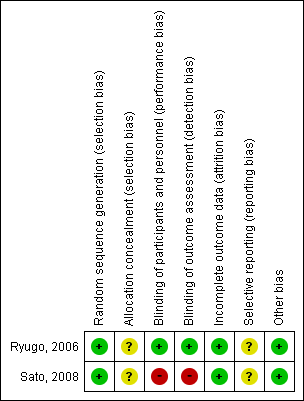


Length of ICU stay


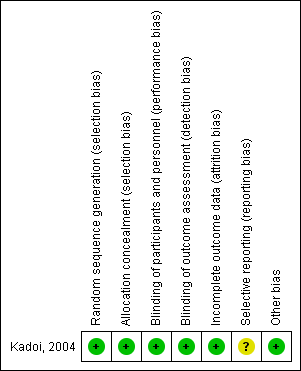


1. Forest plot

Mortality


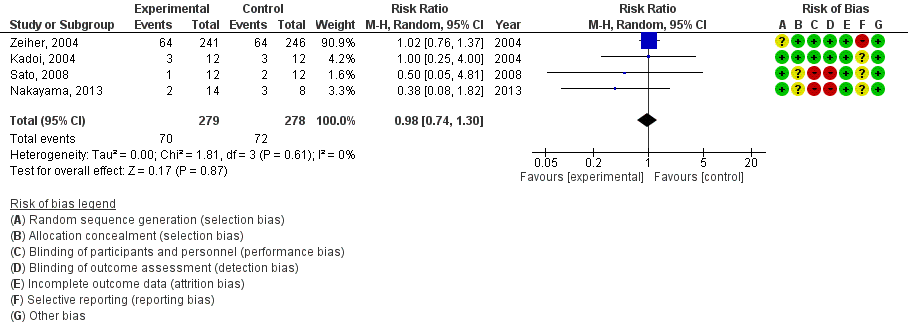


Duration of mechanical ventilation


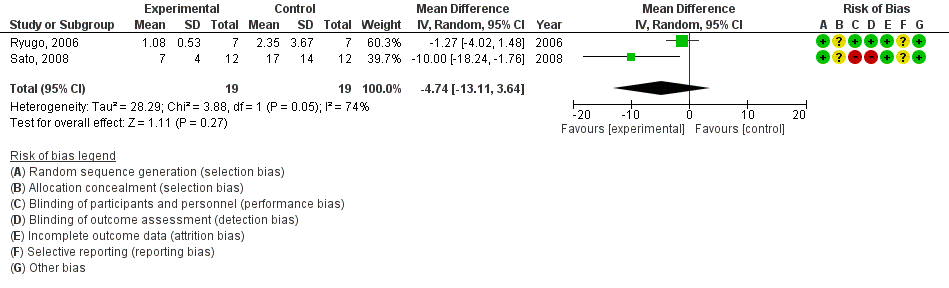


Length of ICU stay


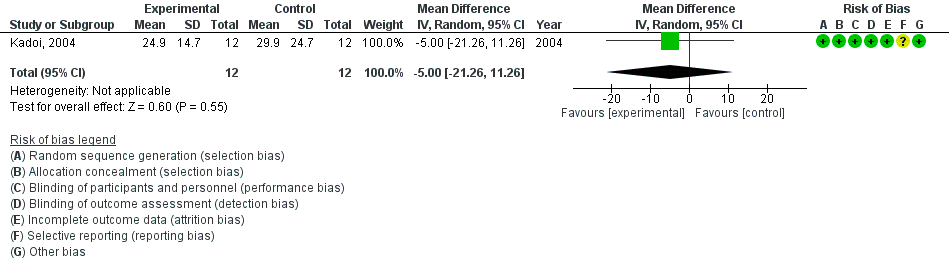


1. Evidence profile

| **Assessment of certainty** | | | | | | | | | | | | | **№ of patients** | | **Effect** | | | | | | **Certainty** | | | **Importance** |  |  |  |
| --- | --- | --- | --- | --- | --- | --- | --- | --- | --- | --- | --- | --- | --- | --- | --- | --- | --- | --- | --- | --- | --- | --- | --- | --- | --- | --- | --- |
| **№ of studies** | | **Study design** | | | **Risk of bias** | | | **Inconsistency** | | **Indirectness** | **Imprecision** | **Others** | **Sivelestat** | **No sivelestat** | **Relative index (95% CI)** | | | **Absolute index (95% CI)** | | |  |  |  |  |  |  |  |
| **Mortality** | | | | | | | | | | | | | | | | | | | | | | | | |  |  |  |
| 4 | | RCT | | | Serious ^a^ | | | Not serious | | Not serious | Very serious ^b^ | None | 70/279 (25.1%) | 72/278 (25.9%) | **RR 0.98** (0.74 to 1.30) | | | **5 fewer per 1000**  (67 fewer ～78 more) | | | ⨁◯◯◯ Very low | | | Critical |  |  |  |
| **Duration of mechanical ventilation** | | | | | | | | | | | | | | | | | | | | | | | | |  |  |  |
| 2 | | RCT | | | Serious ^c^ | | | Not serious | | Not serious | Very serious ^d^ | None | 19 | 19 | - | | | MD4.74 days shorter  (13.11 shorter ～ 3.64 longer) | | | ⨁◯◯◯ Very low | | | Critical |  |  |  |
| **Length of ICU stay** | | | | | | | | | | | | | | | | | | | | | | | | |  |  |  |
| 1 | | RCT | | | Not serious | | | Not serious | | Not serious | Very serious ^d^ | None | 12 | 12 | - | | | MD **5 days shorter** (21.26 shorter ～ 11.26 longer) | | | ⨁⨁◯◯ Low | | | Critical |  |  |  |
| **Length of hospital stay** | | | | | | | | | | | | | | | | | | | | | | | | | |  |  |
| 0 | | - | | | - | | | - | | |  | - | - | - | - | Not estimated | | - | | - | | | Critical | | | |  |
| **Liver dysfunction** | | | | | | | | | | | | | | | | | | | | | | | | | | |  |
| 0 | | | - | | - | | | - | | |  | | - | - | - | - | | Not estimated | | - | | - | | | Critical | | |
| **Kidney injury** | | | | | | | | | | | | | | | | | | | | | | | | | | |  |
| 0 | | | - | | | - | | | - | | |  | - | - | - | - | | Not estimated | | - | | - | | | Critical | | |
| **Allergy** | | | | | | | | | | | | | | | | | | | | | | | | | | |  |
| 0 | | | - | | | - | | | - | | |  | - | - | - | - | Not estimated | | - | | - | | | Critical | | | |

**CI:** confidence interval; **RR:** risk ratio; **MD:** mean difference; **RCT**: randomized controlled trial

Length of hospital stay, liver dysfunction, kidney injury, allergy were not included in the evidence profile because the outcomes are not reported.

#### Explanation

a. Because there are many studies with moderate and high risk of bias

b. Because the optimal information size (OIS) was not met and the 95% confidence interval crossed the threshold for clinical judgment.

c. Because most of the studies in weight are high risk of bias studies.

d. Because the OIS was not met and the 95% confidence interval crossed the threshold for clinical judgment.

1. Evidence-to-Decision table

| question | |
| --- | --- |
| CQ41： Should sivelestat be used for patients with ARDS? | |
| **Population:** | Adult patients with ARDS |
| **Intervention:** | Sivelestat |
| **Comparison:** | No sivelestat |
| **Main outcomes:** | Mortality, duration of mechanical ventilation, length of intensive care unit (ICU) stay, length of hospital stay, liver dysfunction, kidney injury, allergy |
| **Setting:** | Emergency room (ER) or ICU |
| **Perspective:** | Individual |
| **Background:** | The main body of ARDS is hyper-permeabilized pulmonary edema caused by non-specific inflammation in the alveolar space, and neutrophil elastase is considered to be one of the important mediators in the pathogenesis of ARDS ^1,2)^. In Japan, sivelestat, a neutrophil elastase inhibitor, is clinically available and has been studied for its prognostic value. While there are reports of no improvement in mortality ^3)^, there are also reports of promising efficacy ^4)^. Therefore, a systematic review of this clinical question is an important clinical issue to be addressed. |
| **Conflict of interests:** | None |

# Assessment

| Problem Is the problem a priority? | | |
| --- | --- | --- |
| Judgment | research evidence | ADDITIONAL CONSIDERATIONS |
| ○ No  ○ Probably no  ● Probably yes  ○ Yes  ○ Varies  ○ Do not know | The main pathogenesis of ARDS is hyper-permeabilized pulmonary edema due to non-specific inflammation in the alveolar space, and neutrophil elastase is considered to be one of the important mediators in its pathogenesis. Sivelestat, a neutrophil elastase inhibitor, is available for clinical use in Japan and has been studied for its prognostic value. While some reports suggest that it does not improve mortality, others suggest that it may be effective. Therefore, clarifying the balance of benefits and harms of sivelestat is an important clinical issue. |  |
| Desirable effects How substantial are the desirable anticipated effects? | | |
| Judgment | research evidence | ADDITIONAL CONSIDERATIONS |
| ● Trivial  ○ Small  ○ Moderate  ○ Large  ○ Varies  ○ Do not know | A meta-analysis was performed using four randomized controlled trials (RCTs). The estimated values of the desirable anticipated effects were as follows: mortality yielded an RD of 5 fewer per 1000 (4 RCTs: N=557) (95% CI: 67 fewer to 78 more); duration of mechanical ventilation yielded an MD of 4.7 days shorter (2 RCTs: N=38) (13.11 shorter to 3.64 shorter); length of ICU stay yielded an MD of 5 days shorter (1 RCT: N=24) (21.26 shorter to 11.26 longer). From the above, it was judged that the desirable anticipated effect was “trivial.” |  |
| Undesirable effects How substantial are the undesirable anticipated effects? | | |
| Judgment | research evidence | ADDITIONAL CONSIDERATIONS |
| ○ Large  ○ Moderate  ○ Small  ● Trivial  ○ Varies  ○ Do not know | The anticipated harm of sivelestat was not reported in the paper adopted for this study. Therefore, when considered together with the statements in the additional considerations, the anticipated undesirable effect was judged to be “trivial.” | Zeiher et al. reported no significant difference in the percentage of patients who discontinued the drug due to adverse events between the sivelestat and control groups (4.6 vs. 3.7%). Kadoi et al. also reported no difference in laboratory values before and after treatment between the two groups. In the package insert of the original product, Elaspor, liver dysfunction was reported as an adverse effect in less than 1-10% of patients. |
| Certainty of evidence What is the overall certainty of the evidence of effects? | | |
| Judgment | research evidence | ADDITIONAL CONSIDERATIONS |
| ● Very low  ○ Low  ○ Moderate  ○ High  ○ No included studies | \| **Outcome** \| **Importance** \| **Certainty of the evidence** \| \| --- \| --- \| --- \| \| \| \| Mortality \| Critical \| ⨁◯◯◯ \| \| Very low \| \| Duration of mechanical ventilation \| Critical \| ⨁◯◯◯ \| \| Very low \| \| Length of ICU stay \| Critical \| ⨁⨁◯◯ \| \| Low \| \| Length of hospital stay \| Critical \| - \| \| - \| \| Liver dysfunction \| Critical \| - \| \| - \| \| Kidney injury \| Critical \| - \| \| - \| \| Allergy \| Critical \| - \| \| - \|   No RCTs reported the length of hospital stay, liver dysfunction, kidney injury, and allergy as outcomes.  **Overall certainty of the evidence:**  The direction of the desirable and undesirable effects was not consistent. Thus, the overall certainty of the evidence was judged to be “very low.” |  |
| Values Is there important uncertainty about or variability in how much people value the main outcomes? | | |
| Judgment | research evidence | ADDITIONAL CONSIDERATIONS |
| ○ Important uncertainty or variability  ○ Possibly important uncertainty or variability  ○ Probably no important uncertainty or variability  ● No important uncertainty or variability | No important uncertainty or variability. |  |
| Balance of effects Does the balance between desirable and undesirable effects favor the intervention or the comparison? | | |
| Judgment | research evidence | ADDITIONAL CONSIDERATIONS |
| ○ Favors the comparison  ○Probably favors the comparison  ○ Does not favor either the intervention or the comparison  ○ Probably favors the intervention  ○Favors the intervention  ○ Varies  ● Do not know | \| Outcome \| Comparison \| Intervention  (Sivelestat） \| Absolute difference \| Relative effect RR (95% CI) \| \| --- \| --- \| --- \| --- \| --- \| \| Mortality \| 72/278  (25.9%) \| 70//279  (25.1%) \| 5 fewer/1,000  (67 fewer～78 fewer) \| 0.98  (0.74～ 1.30) \| \| Duration of mechanical ventilation \| － \| － \| MD 4.74 days shorter  (13.11 shorter～3.64 shorter) \| － \| \| Length of ICU stay \| － \| － \| MD 5 days shorter  (21.26 shorter～11.26 shorter) \| － \| \| Length of hospital stay \| － \| － \| No estimate \| － \| \| Liver dysfunction \| － \| － \| No estimate \| － \| \| Kidney injury \| － \| － \| No estimate \| － \| \| Allergy \| － \| － \| No estimate \| － \|   From the above data, we concluded that the balance between benefits and harms is uncertain. |  |
| Acceptability Is the intervention acceptable to key stakeholders? | | |
| Judgment | research evidence | ADDITIONAL CONSIDERATIONS |
| ○ No  ● Probably no  ○ Probably yes  ○ Yes  ○ Varied  ○ Do not know | The benefits of the intervention are not clear and may increase mortality. In addition, the possibility of some increase in costs made it probably unacceptable. |  |
| Feasibility Is the intervention feasible to implement? | | |
| Judgment | research evidence | ADDITIONAL CONSIDERATIONS |
| ○ No  ○ Probably no  ○ Probably yes  ● Yes  ○ Varies  ○ Do not know | The availability of sivelestat is easy, and it is highly feasible in any hospital. |  |

# Summary of judgment

|  | **Judgment** | | | | | | |
| --- | --- | --- | --- | --- | --- | --- | --- |
| **PROBLEM** | No | Probably no | Probably yes | Yes |  | Varies | Do not know |
| **DESIRABLE EFFECTS** | Trivial | Small | Moderate | Large |  | Varies | Do not know |
| **UNDESIRABLE EFFECTS** | Large | Moderate | Small | Trivial |  | Varies | Do not know |
| **CERTAINTY OF EVIDENCE** | Very low | Low | Moderate | High |  |  | No included studies |
| **VALUES** | Important uncertainty or variability | Possibly important uncertainty or variability | Probably no important uncertainty or variability | No important uncertainty or variability |  |  |  |
| **BALANCE OF EFFECTS** | Favors the comparison | Probably favors the comparison | Does not favor either the intervention or the comparison | Probably favors the intervention | Favors the intervention | Varies | Do not know |
| **ACCEPTABILITY** | No | Probably no | Probably yes | Yes |  | Varies | Do not know |
| **FEASIBILITY** | No | Probably no | Probably yes | Yes |  | Varies | Do not know |

# Type of Recommendation

| Strong recommendation against the intervention | Conditional recommendation against the intervention | Conditional recommendation for either the intervention or the comparison | Conditional recommendation for the intervention | Strong recommendation for the intervention |
| --- | --- | --- | --- | --- |
| ○ | ● | ○ | ○ | ○ |

# Conclusions

| Recommendation |
| --- |
| **We suggest against administering sivelestat to patients with ARDS**  (**Conditional recommendation**/**Very low: GRADE 2D).** |
|  |
| Justification |
| **Question:** Should sivelestat be used for patients with ARDS?  **Patient:**Adult patients with ARDS  **Intervention:**Sivelestat  **Comparison:**　No sivelestat  **Outcome:**Mortality, Duration of ventilation, length of ICU stay  **Summary of evidence**:  The estimated values of the desirable anticipated effects were as follows: mortality yielded an RD of 5 fewer per 1000 (4 RCTs: N=557) (95% CI: 67 fewer to 78 more); duration of mechanical ventilation yielded an MD of 4.7 days shorter (2 RCTs: N=38) (13.11 shorter to 3.64 shorter); length of ICU stay yielded an MD of 5 days shorter (1 RCT: N=24) (21.26 shorter to 11.26 longer). From the above data, it was judged that the desirable anticipated effect was “trivial.” On the contrary, the anticipated harm of sivelestat was not reported in the paper adopted for this study. However, Zeiher et al. reported no significant difference in the percentage of patients who discontinued the drug due to adverse events between the sivelestat and control groups (4.6 vs. 3.7%). Kadoi et al. also reported no difference in the laboratory values before and after treatment between the two groups. In the package insert of the original product, Elaspor, liver dysfunction was reported as an adverse effect in less than 1-10% of patients. Therefore, when considered together with the statements in the additional considerations, the anticipated undesirable effect was judged to be “trivial.”  **Certainty of the evidence**: The direction of desirable and undesirable effects was not consistent. Thus, the overall certainty of the evidence was judged to be “very low.”  **Values, balance of effects, acceptability, feasibility**：  With regard to values, the value for death is generally high, and its variability is expected to be small. Although the predicted undesirable effect of this intervention is small, the desirable effect may change direction when the upper and lower limits of the confidence interval are considered. That is, mortality may increase by 78 per 1000. Therefore, the balance of effects was judged to be “not known.” As for acceptance, the benefit of the intervention is unclear and may increase deaths. In addition, costs may increase to some extent, so we judged that it is probably unacceptable. As for feasibility, sivelestat is readily available and feasible in any hospital.  **Panel meeting：**  In the pre-vote, the modified Delphi method resulted in a median score of 8 points, with a disagreement index of 0.1316, for a “conditional recommendation to not use sivelestat in patients with ARDS”.  The panel discussed the direction of the recommendation based on the balance of harm and benefit and cost. The panel also discussed the possibility of limiting the route of administration of the drug because it is often combined with other drugs. As a result, the panel reached a consensus based on the results of the preliminary vote without a second vote being required. |

| Subgroup considerations |
| --- |
| None |
| Implementation considerations |
| There are no changes in recommendations from the ARDS Guidelines 2016. |

| Monitoring and evaluation |
| --- |
| Further collection of information on the adverse events and cost-effectiveness is necessary because of clinical problems in implementing the recommendations. In addition, it is necessary to monitor the implementation status of the guideline through the use of questionnaires and other means after the guideline is published to check if there are any other clinical problems. |
| Research priorities |
| Three of the four RCTs used in this meta-analysis had small sample sizes (22-24 patients), and one large study was conducted in 2004. It is hoped that larger RCTs will be conducted on patients with ARDS in the future. |

References

1. Donnelly SC, MacGregor I, Zamani A, Gordon MW, Robertson CE, Steedman DJ, et al. Plasma elastase levels and the development of the adult respiratory distress syndrome. Am J Respir Crit Care Med. 1995;151(5):1428-1433. PMID: 7735596.

2. Moraes TJ, Chow CW, Downey GP. Proteases and lung injury. Crit Care Med. 2003;31(4): S189-194. PMID: 12682439.

3. Iwata K, Doi A, Ohji G, Oka H, Oba Y, Takimoto K, et al. Effect of neutrophil elastase inhibitor (sivelestat sodium) in the treatment of acute lung injury (ALI) and acute respiratory distress syndrome (ARDS): a systematic review and meta-analysis. Intern Med. 2010;49(22):2423-2432. PMID: 21088343.

4. Aikawa N, Kawasaki Y. Clinical utility of the neutrophil elastase inhibitor sivelestat for the treatment of acute respiratory distress syndrome. Ther Clin Risk Manag. 2014;10:621-629. PMID: 25120368.

**CQ42 Should corticosteroids be used for adult patients with ARDS?**

1. Search strategy

MEDLINE via PubMed (Search date: 2020/6/26）

| #1 | "respiratory distress syndrome, adult"[MeSH Terms] OR "ARDS"[Title/Abstract] OR "respiratory distress syndrome"[Title/Abstract] OR "acute lung injury"[MeSH Terms] OR "acute lung injur*"[Title/Abstract] OR "ALI"[Title/Abstract] |
| --- | --- |
| #2 | "respiratory distress syndrome, newborn"[MeSH Terms] |
| #3 | #1 OR #2 |
| #4 | "steroids"[MeSH Terms] OR "adrenal cortex hormones"[MeSH Terms] |
| #5 | "methylprednisolone"[Title/Abstract] OR "hydrocortisone"[Title/Abstract] OR "glucocorticoid*"[Title/Abstract] OR "dexamethasone"[Title/Abstract] OR "corticosteroid*"[Title/Abstract] OR "cortiso*"[Title/Abstract] |
| #6 | #4 OR #5 |
| #7 | #3 AND #6 |
| #8 | ((((((("randomized controlled trial"[Publication Type] OR "controlled clinical trial"[Publication Type]) OR "randomized"[Title/Abstract]) OR "placebo"[Title/Abstract]) OR "drug therapy"[MeSH Subheading]) OR "randomly"[Title/Abstract]) OR "trial"[Title/Abstract]) OR "groups"[Title/Abstract]) NOT ("animals"[MeSH Terms] NOT "humans"[MeSH Terms]) |
| #9 | #7 AND #8 |

CENTRAL（Search date: 2020/6/26）

| #1 | [mh "Respiratory Distress Syndrome, Adult"] OR ARDS:ti,ab OR "respiratory distress syndrome":ti,ab OR [mh "Acute Lung Injury"] OR "acute lung injury":ti,ab OR ALI:ti,ab |
| --- | --- |
| #2 | [mh "Respiratory Distress Syndrome, Newborn"] |
| #3 | #1 OR #2 |
| #4 | [mh Steroids] OR [mh "adrenal cortex hormones"] |
| #5 | methyl prednisolone:ti,ab OR hydrocortisone:ti,ab OR glucocorticoid*:ti,ab OR dexamethasone:ti,ab OR corticosteroid*:ti,ab OR cortiso*:ti,ab |
| #6 | #4 OR #5 |
| #7 | #3 AND #6 |

1. Flow diagram

**Identification**

8 Studies included in qualitative synthesis

98 Full-text articles assessed for eligibility

2161 records after duplicates removed

2443 records identified through database searching

2443 records identified through database searching

Medline via PubMed (n=1827)

CENTRAL (n=519)

Igaku-Chuo-Zasshi (n=97)

0 additional records identified through other sources

7 Studies included in quantitative synthesis (meta-analysis)

90 Full-text articles excluded, with reasons:

・Wrong language (n=7)

・Wrong study design (n=28)

・Wrong population (n=53)

・Protocol (n=2)

Duplicates

n=282

2063 records excluded

**Included**

**Eligibility**

**Screening**

1. Risk of bias

High dose corticosteroid


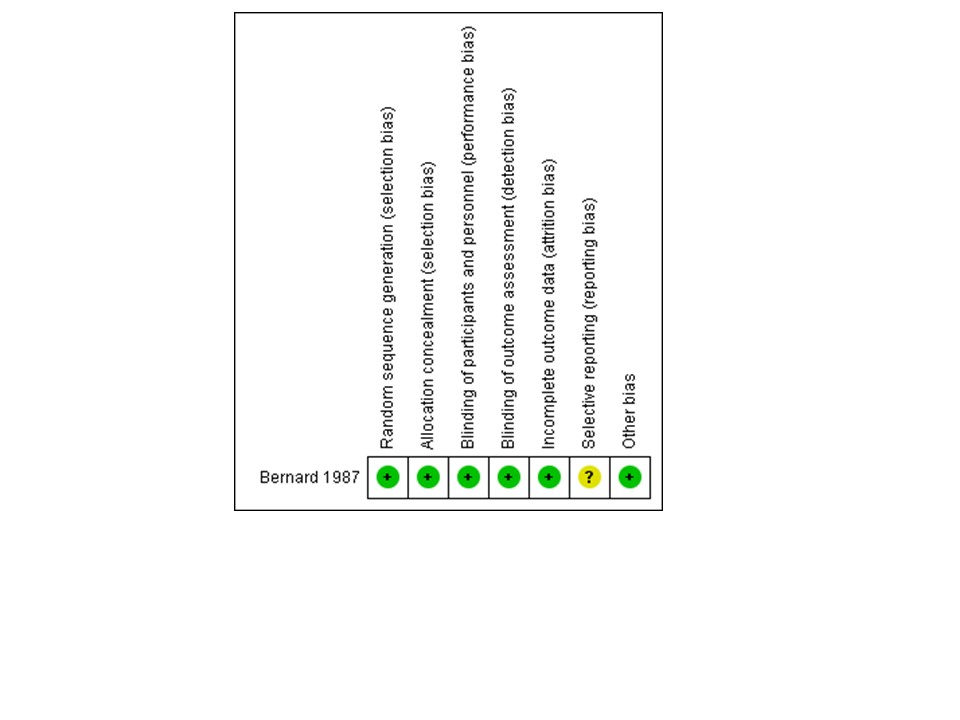
Long-term mortality Infection


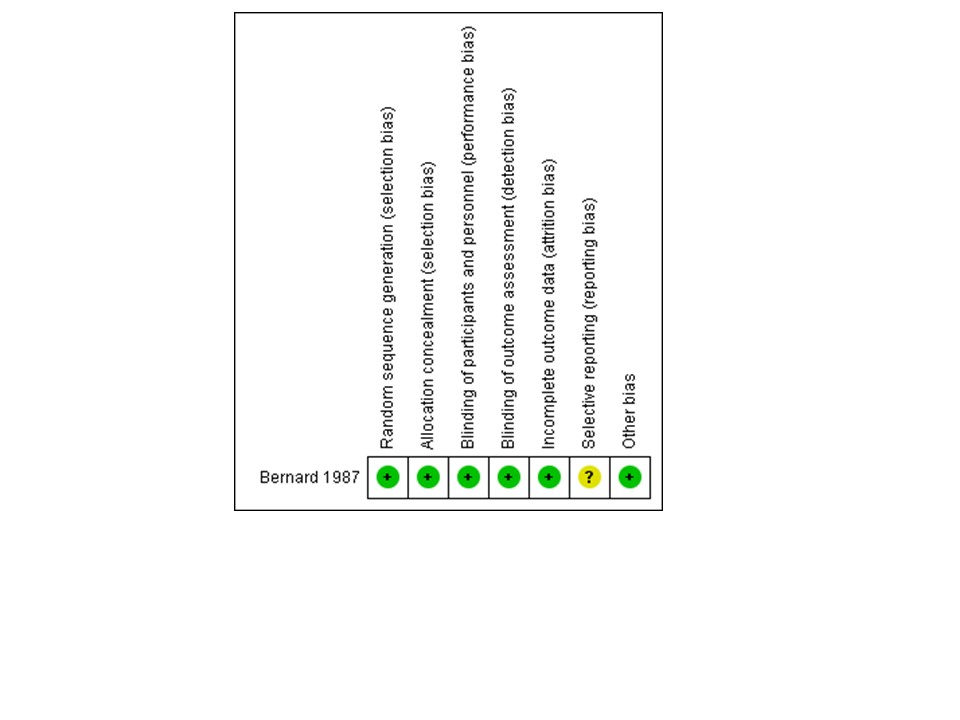


Low dose corticosteroid

Long-term mortality Ventilator-free days


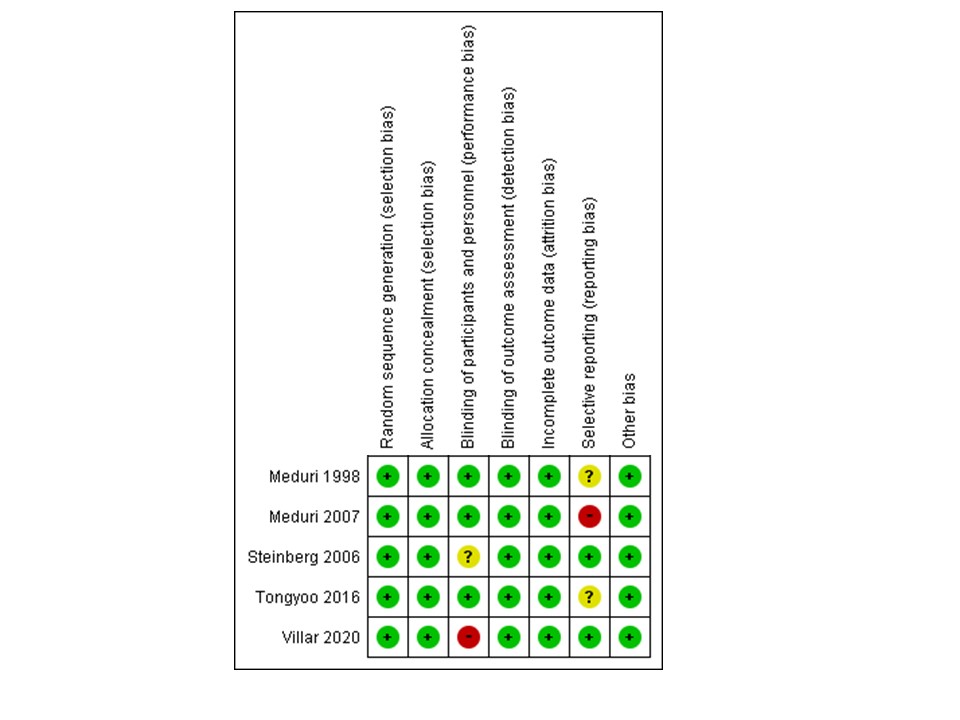

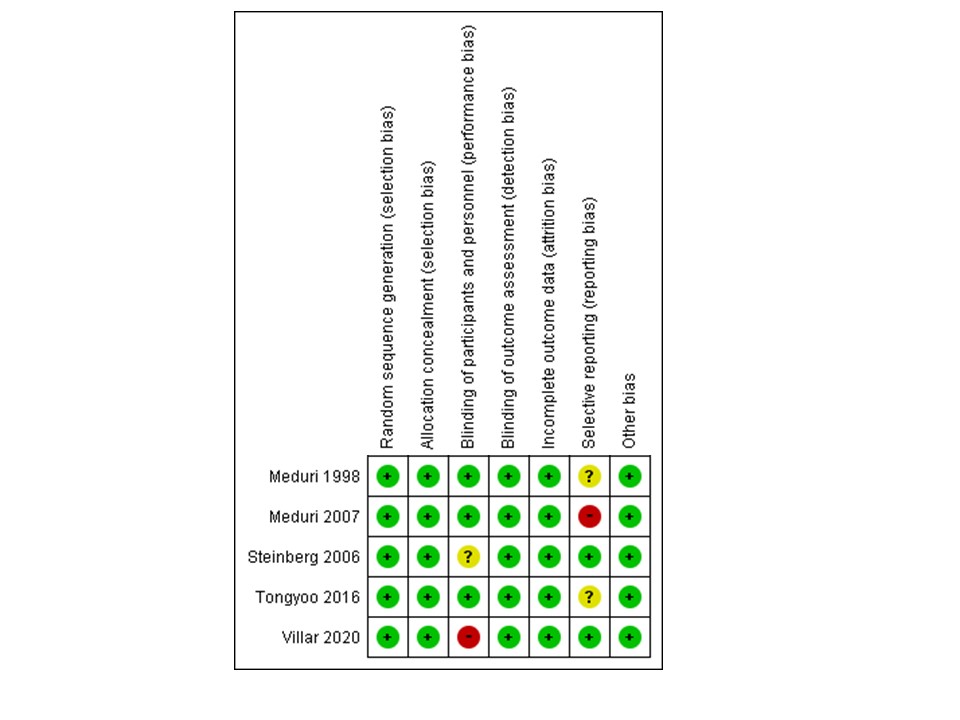


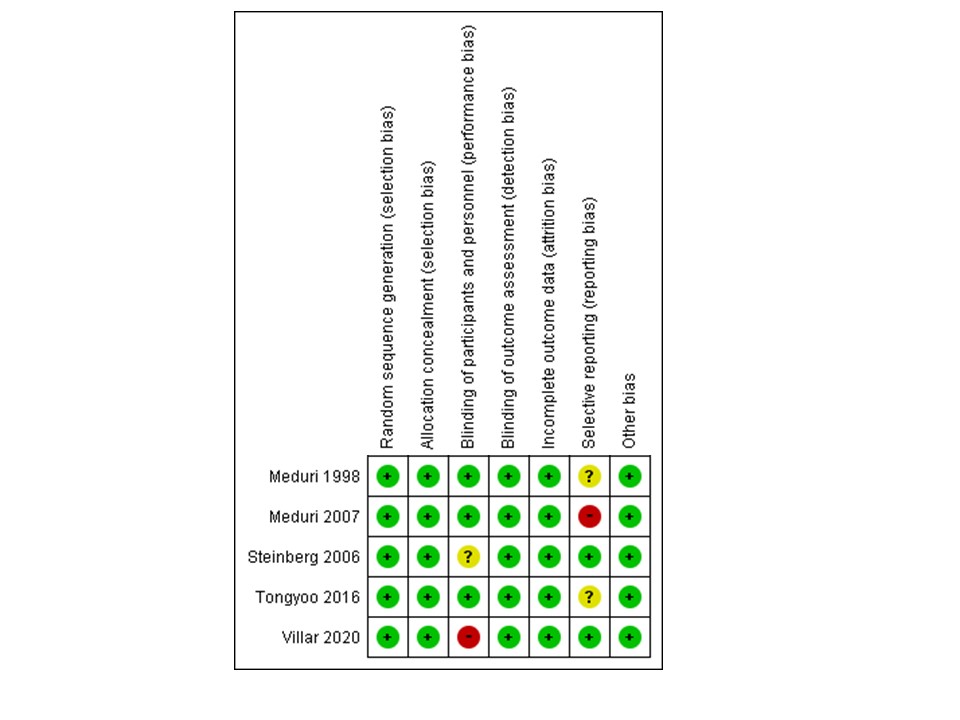
Infection Length of ICU stay


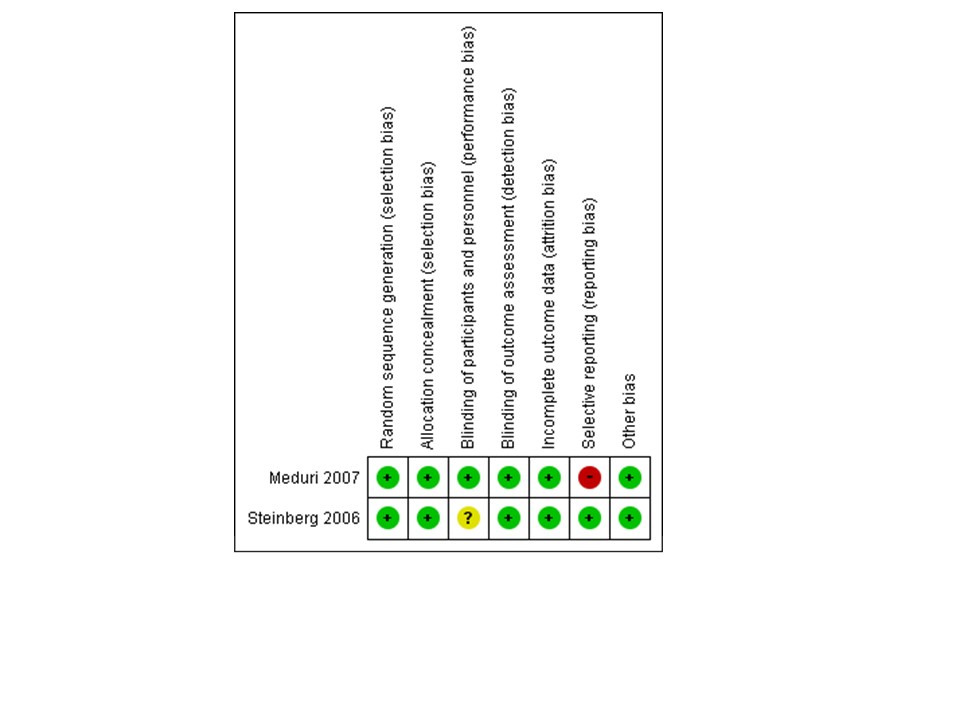


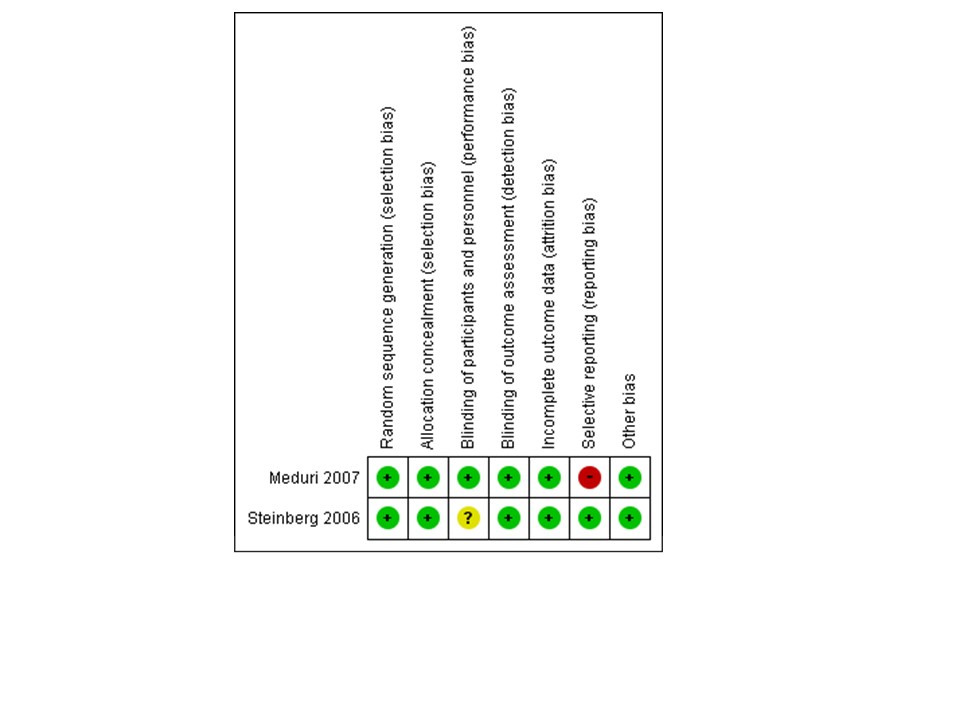
Length of hospital stay P/F ratio


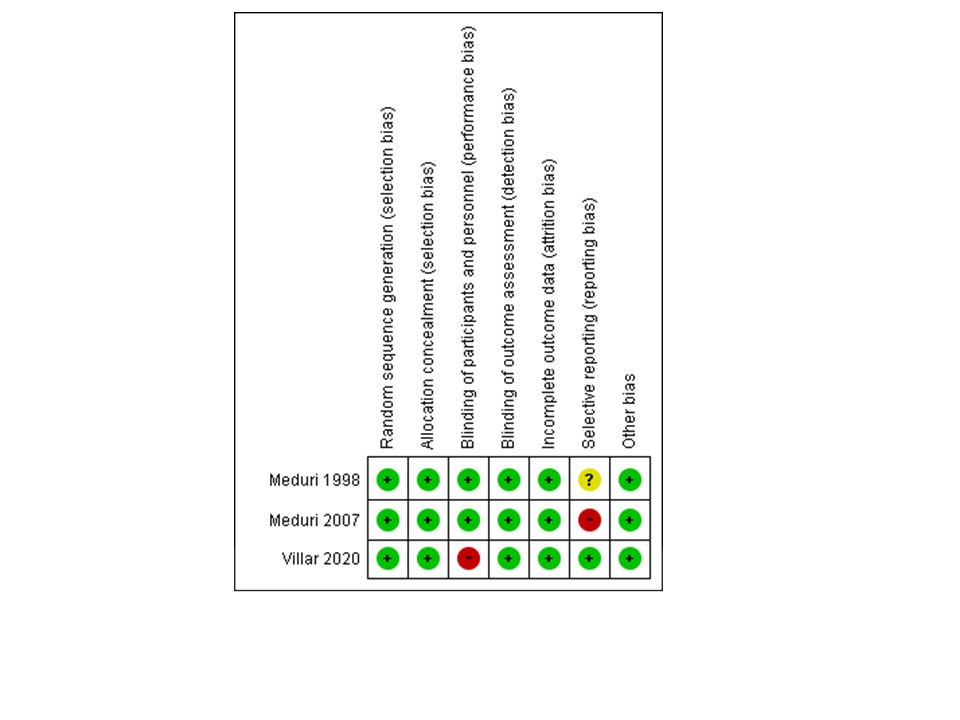


1. Forest plot

High dose corticosteroid

Long-term mortality


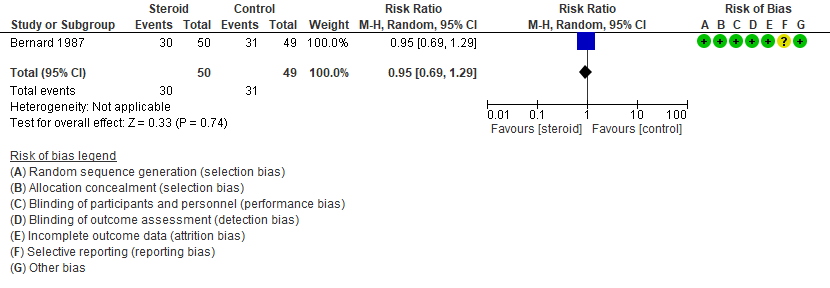


Infection


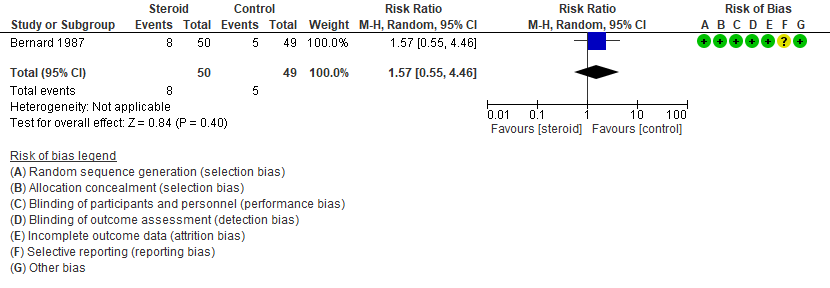


low dose corticosteroid

Long-term mortality


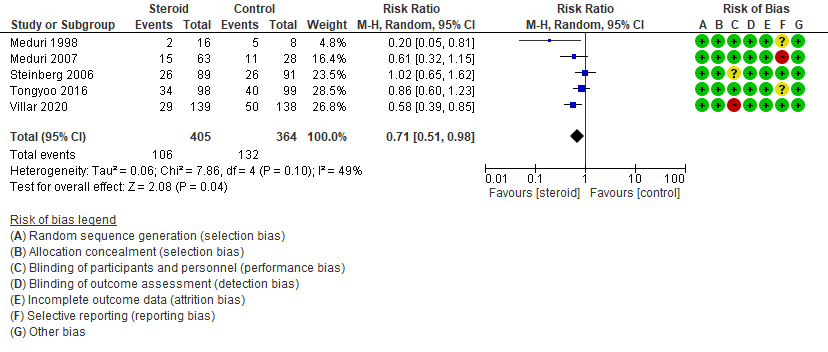


Ventilator-free days


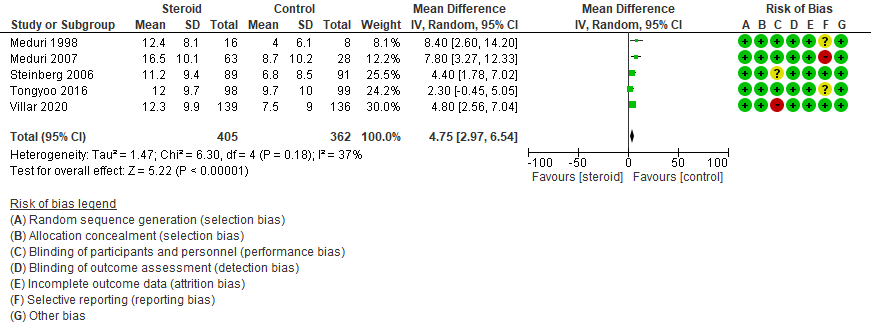


Infection


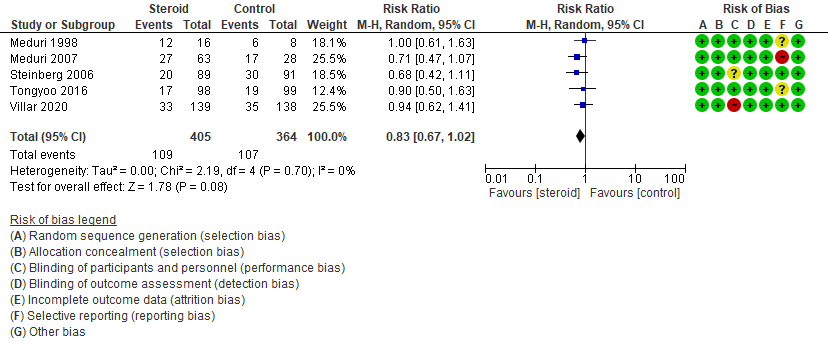


Length of ICU stay


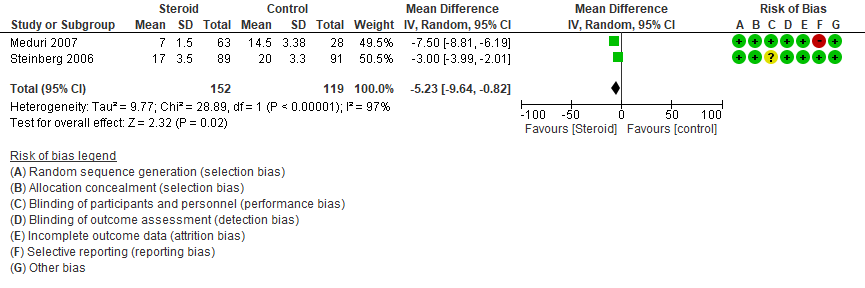


Length of hospital stay


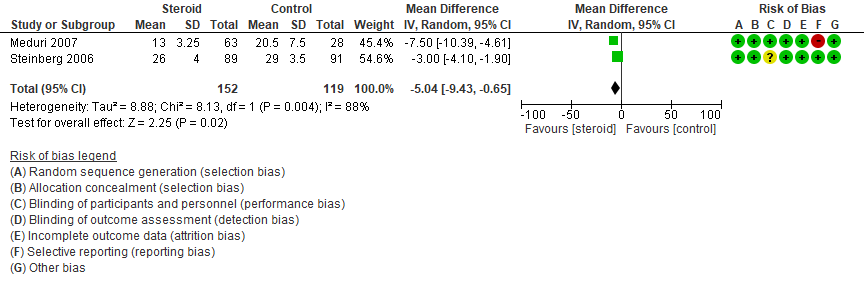


P/F ratio


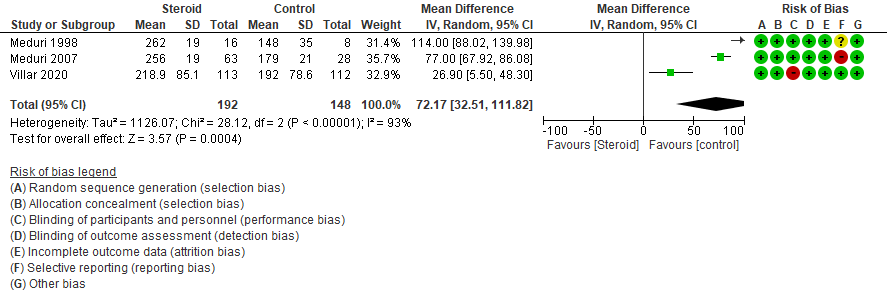


1. Evidence profile

High dose corticosteroids

| **Assessment of certainty** | | | | | | | **№ of patients** | | **Effect** | | **Certainty** | **Importance** |
| --- | --- | --- | --- | --- | --- | --- | --- | --- | --- | --- | --- | --- |
| **№ of studies** | **Study design** | **Risk of bias** | **Inconsistency** | **Indirectness** | **Imprecision** | **Others** | **Corticosteroid** | **No corticosteroid** | **Relative index (95% CI)** | **Absolute index (95% CI)** |  |  |
| Long term mortality | | | | | | | | | | | | |
| 1 | RCT | Not serious | Not serious | Not serious | very serious ^a^ | None | 30/50 (60.0%) | 31/49 (63.3%) | RR 0.95 (0.69 to 1.29) | **32 fewer per 1000** (196 fewer ～ 183 more) | ⨁⨁◯◯ Low | Critical |
| Infection | | | | | | | | | | | | |
| 1 | RCT | Not serious | Not serious | Not serious | very serious ^a^ | None | 8/50 (16.0%) | 5/49 (10.2%) | RR 1.57 (0.55 to 4.46) | **58 more per 1000** (46 fewer ～ 353 more) | ⨁⨁⨁◯ Moderate | Critical |
| Ventilator free days | | | | | | | | | | | | |
| 0 | - | - | - | - | - | - | - | - | - | **Not estimated** | - | Critical |
| Length of ICU stay | | | | | | | | | | | | |
| 0 | - | - | - | - | - | - | - | - | - | **Not estimated** | - | Critical |
| Length of hospital stay | | | | | | | | | | | | |
| 0 | - | - | - | - | - | - | - | - | - | **Not estimated** | - | Critical |
| P/F ratio | | | | | | | | | | | | |
| 0 | - | - | - | - | - | - | - | - | - | **Not estimated** | - | Important |

**CI:** confidence interval; **RR:** risk ratio; **MD:** mean difference; RCT: randomized controlled trial

Ventilator free days, length of ICU stay, length of hospital stay, and P/F ratio are not included in the Evidence profile because the outcomes are were not reported.

#### Explanation

#### a. The optimal information size (OIS) criteria are not met. In addition, the 95% confidence interval straddles the threshold for clinical judgment.

Low dose corticosteroids

| **Assessment of certainty** | | | | | | | **№ of patients** | | **Effect** | | **Certainty** | **Importance** |
| --- | --- | --- | --- | --- | --- | --- | --- | --- | --- | --- | --- | --- |
| **№ of studies** | **Study design** | **Risk of bias** | **Inconsistency** | **Indirectness** | **Imprecision** | **Others** | **Corticosteroid** | **No corticosteroid** | **Relative index (95% CI)** | **Absolute index (95% CI)** |  |  |
| Long term mortality | | | | | | | | | | | | |
| 5 | RCT | Serious ^a^ | Serious ^b^ | Not serious | Not serious | None | 106/405 (26.2%) | 132/364 (36.3%) | RR 0.71 (0.51 to 0.98) | **105 fewer per 1000** (178 fewer ～ 7 fewer) | ⨁⨁◯◯ Low | Critical |
| Ventilator free days | | | | | | | | | | | | |
| 5 | RCT | serious ^a^ | Not serious | Not serious | Not serious | None | 405 | 362 | - | MD **4.75 days longer** (2.97 longer ～ 6.54 longer) | ⨁⨁⨁◯ Moderate | Critical |
| Infection | | | | | | | | | | | | |
| 5 | RCT | serious ^a^ | Not serious | Not serious | serious ^c^ | None | 109/405 (26.9%) | 107/364 (29.4%) | RR 0.83 (0.67 to 1.02) | **50 fewer per 1000** (97 fewer ～ 6 more) | ⨁⨁◯◯ Low | Critical |
| Length of ICU stay | | | | | | | | | | | | |
| 2 | RCT | serious ^a^ | Not serious | Not serious | serious ^d^ | None | 152 | 119 | - | MD **5.23 days shorter** (9.64 shorter ～ 0.82 shorter) | ⨁⨁◯◯ Low | Critical |
| Length of hospital stay | | | | | | | | | | | | |
| 2 | RCT | serious ^a^ | Not serious | Not serious | serious ^d^ | None | 152 | 119 | - | MD **5.04 days shorter** (9.43 shorter ～ 0.65 shorter) | ⨁⨁◯◯ Low | Critical |
| P/F ratio | | | | | | | | | | | | |
| 3 | RCT | serious ^a^ | Not serious | Not serious | serious ^d^ | None | 192 | 148 | - | MD **72.17 mmHg higher** (32.51 mmHg higher ～ 111.82 mmHg higher) | ⨁⨁◯◯ Low | Important |

**CI:** confidence interval; **RR:** risk ratio; **MD:** mean difference; RCT: randomized controlled trial

Short-term mortality iswas excluded from the outcome because of overlap in outcome reporting with long-term mortality.

#### Explanation

a. The risk of bias (RoB) of all included studies are low or moderate.

b. Due to moderate heterogeneity.

c. The 95% confidence interval straddles the threshold for clinical judgment.

d. The optimal information size (OIS) criteria are not met.

1. Evidence-to-Decision table

| Question | |
| --- | --- |
| CQ42：Should corticosteroids be used for adult patients with ARDS? | |
| **Population:** | Adult patients with ARDS |
| **Intervention:** | Corticosteroids (any corticosteroid type, dose, duration, or method of administration) |
| **Comparison:** | No corticosteroids |
| **Main outcomes:** | Mortality, length of hospital stay, length of intensive care unit (ICU) stay (ICU-free days), duration of ventilation (ventilator-free days), infection (pneumonia, sepsis, others) |
| **Setting:** | Emergency room or ICU |
| **Perspective:** | Individual |
| **Background:** | ARDS is a condition caused by inflammation that has spread to the lungs from various causes ^1)^. Corticosteroids are thought to be effective in inactivating the inflammatory mediators involved in the inflammation of ARDS. Corticosteroids are thought to be effective because they inactivate inflammatory mediators involved in the inflammation of ARDS. However, corticosteroids may reduce immune function and cause infection. Whether it improves mortality and other clinical outcomes is still controversial, as conflicting results have been published in previous systematic reviews ^2-4)^. Therefore, a systematic review of this clinical question is an important issue. |
| **Conflict of interests:** | None |

# Assessment

| Problem Is the problem a priority? | | |
| --- | --- | --- |
| Judgment | research evidence | ADDITIONAL CONSIDERATIONS |
| ○ No  ○ Probably no  ● Probably yes  ○ Yes  ○ Varies  ○ Do not know | ARDS is a condition caused by inflammation that has spread to the lungs from various sources. Corticosteroids are thought to be effective because they inactivate the inflammatory mediators involved in the inflammation of ARDS. However, corticosteroids have the potential to reduce immune function and cause infection. Whether it improves mortality and other clinical outcomes is still controversial, as previous systematic reviews have published conflicting results. Therefore, the priority of this CQ is relatively high. |  |
| Desirable effects How substantial are the desirable anticipated effects? | | |
| Judgment | research evidence | ADDITIONAL CONSIDERATIONS |
| ● Trivial (High dose)  ○ Small  ● Moderate (Low dose)  ○ Large  ○ Varies  ○ Do not know | We conducted a meta-analysis of seven randomized controlled trials (RCTs) comparing the use of corticosteroids and no corticosteroids in ventilated adult patients with ARDS (P/F ratio of less than 200). Of these, the study by Bernard et al. had a high dose of methylprednisolone (30 mg/kg). Thus, it was separated from the other low dose studies.  <High dose>  The estimated values of the desirable anticipated effects were as follows: mortality yielded an RD of 32 fewer per 1000 (1 RCT: N=99) (95% CI: 196 fewer to 183 more). From the above data, it was judged that the desirable anticipated effect was “trivial.”  <Low dose>  The estimated values of the desirable anticipated effects were as follows: mortality yielded an RD of 105 fewer per 1000 (5 RCTs: N=769) (95% CI: 178 fewer to 7 fewer); infection yielded an RD of 50 fewer per 1000 (5 RCTs: N=769) (95% CI: 97 fewer to 60 more); ventilator-free days yielded an MD of 4.75 days longer (5 RCTs: N=769) (95% CI: 2.97 longer to 6.54 longer); and length of hospital stay yielded an MD of 5.04 days shorter (2 RCTs: N=271) (95% CI: 9.43 shorter to 0.82 shorter). From the above data, it was judged that the desirable anticipated effect was “moderate.” |  |
| Undesirable effects How substantial are the undesirable anticipated effects? | | |
| Judgment | research evidence | ADDITIONAL CONSIDERATIONS |
| ○ Large  ○ Moderate  ● Small (High dose)  ● Trivial (Low dose)  ○ Varies  ○ Do not know | <High dose>  The estimated values of the undesirable anticipated effects were as follows: infection yielded an RD of 58 more per 1000 (1 RCT: N=99) (95% CI: 46 fewer to 353 more). From the above data, it was judged that the undesirable anticipated effect was “small.”  <Low dose>  No undesirable effects were observed at low doses. Therefore, when considered together with the description in the additional considerations, it was judged to be “trivial”. | Infection is a concern at high doses, and in a meta-analysis of corticosteroid therapy in patients with septic shock in the Japanese Guidelines for the Treatment of Sepsis 2020, there were 8 more infections per 1000 patients (12 fewer to 31 more) and 6 more gastrointestinal bleeding per 1000 patients (13 fewer to 32 more). |
| Certainty of evidence What is the overall certainty of the evidence of effects? | | |
| Judgment | research evidence | ADDITIONAL CONSIDERATIONS |
| ○ Very low  ● Low (High dose)  ● Moderate (Low dose)  ○ High  ○ No included studies | \| **Outcome** \| **Importance** \| **Certainty of the evidence**  **(High dose)** \| **Certainty of the evidence**  **(Low dose)** \| \| --- \| --- \| --- \| --- \| \| Mortality \| Serious \| ⨁⨁◯◯ \| ⨁⨁◯◯ \| \| Low \| Low \| \| Ventilator-free days \| Serious \| ― \| ⨁⨁⨁◯ \| \| ― \| Moderate \| \| Length of hospital stay \| Serious \| ― \| ⨁⨁◯◯ \| \| ― \| Low \| \| Length of ICU stay \| Serious \| ― \| ⨁⨁◯◯ \| \| ― \| Low \| \| Infection \| Serious \| ⨁⨁◯◯ \| ⨁⨁◯◯ \| \| Low \| Low \|   **Overall certainty of the evidence**：  <High dose>  The direction of the desirable and undesirable effects was not consistent, and the certainty of evidence across outcomes was judged to be “very low” by adopting the lowest certainty of evidence.  <Low dose>  The direction of the desirable and undesirable effects was consistent, and the certainty of the evidence across the outcomes was judged to be “moderate,” adopting the certainty of the evidence with the highest certainty. | In preparing this table, we added infection to the critical outcomes because there were no harm outcomes included in the originally defined critical outcomes. |
| Values Is there important uncertainty about or variability in how much people value the main outcomes? | | |
| Judgment | research evidence | ADDITIONAL CONSIDERATIONS |
| ○ Important uncertainty or variability  ○ Possibly important uncertainty or variability  ○ Probably no important uncertainty or variability  ●  No important uncertainty or variability | No important uncertainty or variability. |  |
| Balance of effects Does the balance between desirable and undesirable effects favor the intervention or the comparison? | | |
| Judgment | research evidence | ADDITIONAL CONSIDERATIONS |
| ○ Favors the comparison  ●Probably favors the comparison (High dose)  ○ Does not favor either the intervention or the comparison  ● Probably favors the intervention (Low dose)  ○Favors the intervention  ○ Varies  ○Do not know | ＜High dose＞   \| Outcome \| Comparison \| Intervention  (Corticosteroid) \| Absolute difference \| Relative effect RR (95% CI) \| \| --- \| --- \| --- \| --- \| --- \| \| Mortality \| 31/49  (63.3%) \| 30/50 (60.0%) \| 32 fewer/1000 (196 fewer〜183 more) \| 0.95 (0.69〜1.29) \| \| Infection \| 5/49  (10.2%) \| 8/50  (16.0%) \| 58 fewer/1000 (46 fewer〜353 more) \| 1.57 (0.55〜4.46) \|   Based on the above, we concluded that the balance between the effects and harms of intervention was “probably favors the comparison.”  ＜Low dose＞   \| Outcome \| Comparison \| Intervention  (Corticosteroid) \| Absolute difference \| Relative effect RR (95% CI) \| \| --- \| --- \| --- \| --- \| --- \| \| Mortality \| 132/364  (36.3%) \| 106/405 (26.2%) \| 105 fewer/1000 (178 fewer〜7 fewer) \| 0.71 (0.51〜0.98) \| \| Ventilator-free days \| - \| - \| MD 4.75 days longer (2.97 longer〜 6.54 longer ) \| - \| \| Length of hospital stay \| - \| - \| MD 5.04 days shorter (9.43 shorter〜 0.65 shorter) \| - \| \| Length of ICU stay \| - \| - \| MD 5.23 days shorter (9.64 shorter〜0.82 shorter) \| - \| \| Infection \| 107/364  (29.4%) \| 109/405  (26.9%) \| 50 fewer/1000 (97 fewer〜6 more) \| 0.83 (0.67〜1.02) \|   Based on the above, we concluded that the balance between the effects and harms of intervention was “probably favors the intervention.” |  |
| Acceptability Is the intervention acceptable to key stakeholders? | | |
| Judgment | research evidence | ADDITIONAL CONSIDERATIONS |
| ○ No  ● Probably no (High dose)  ● Probably yes (Low dose)  ○ Yes  ○ Varies  ○ Do not know | <High dose>  Intervention may reduce mortality but may increase infections. Intervention may cause more harm and may be unacceptable.  <Low dose>  Interventions may reduce mortality, increase VFDs, and reduce ICU and hospital stays, which may result in economic relief and may be acceptable. |  |
| Feasibility Is the intervention feasible to implement? | | |
| Judgment | research evidence | ADDITIONAL CONSIDERATIONS |
| ○ No  ○ Probably no  ● Probably yes  ○ Yes  ○ Varies  ○ Do not know | Corticosteroids are not covered by insurance for ARDS. However, the causes of ARDS are mostly sepsis and pneumonia. Since dexamethasone is indicated by insurance for severe infections, and methylprednisolone and hydrocortisone are indicated for infectious shock, they may be used in many patients. However, they may not be used in extrapulmonary ARDS for reasons of insurance coverage. |  |

# Summary of judgment

|  | **Judgment** | | | | | | |
| --- | --- | --- | --- | --- | --- | --- | --- |
| **PROBLEM** | No | Probably no | Probably yes | Yes |  | Varies | Do not know |
| **DESIRABLE EFFECTS** | Trivial  （High dose） | Small | Moderate  （Low dose） | Large |  | Varies | Do not know |
| **UNDESIRABLE EFFECTS** | Large | Moderate | Small  （High dose） | Trivial  （Low dose） |  | Varies | Do not know |
| **CERTAINTY OF EVIDENCE** | Very low | Low  （High dose） | Moderate  （Low dose） | High |  |  | No included studies |
| **VALUES** | Important uncertainty or variability | Possibly important uncertainty or variability | Probably no important uncertainty or variability | No important uncertainty or variability |  |  |  |
| **BALANCE OF EFFECTS** | Favors the comparison | Probably favors the comparison  (High dose) | Does not favor either the intervention or the comparison | Probably favors the intervention  (Low dose) | Favors the intervention | Varies | Do not know |
| **ACCEPTABILITY** | No | Probably no  (High dose) | Probably yes  (Low dose) | Yes |  | Varies | Do not know |
| **FEASIBILITY** | No | Probably no | Probably yes | Yes |  | Varies | Do not know |

# Type of Recommendation

| Strong recommendation against the intervention | Conditional recommendation against the intervention | Conditional recommendation for either the intervention or the comparison | Conditional recommendation for the intervention | Strong recommendation for the intervention |
| --- | --- | --- | --- | --- |
| ○ | ●  （High dose） | ○ | ○ | ●  （Low dose） |

# Conclusions

| Recommendation |
| --- |
| **We suggest against administering high-dose corticosteroids to patients with ARDS**  **(conditional recommendation/low: GRADE 2C).**  **We recommend administering low dose corticosteroids to patients with ARDS**  **(strong recommendation/moderate: GRADE 1B).**  **Note: The type of corticosteroid and method of administration varies from study to study. High doses include studies of methylprednisolone 30 mg/kg, and low doses include studies of methylprednisolone 1-2 mg/kg. The subgroup analysis of this committee and previous meta-analysis papers suggest the usefulness of starting low dose corticosteroids early and continuing them for more than 7 days.** |
|  |
| Justification |
| **Question**: Should corticosteroids be used for adult patients with ARDS?  **Patients**: Adult patients with ARDS receiving mechanical ventilation (P/F ratio less than 200)  **Intervention:** Corticosteroids  **Comparison:** No corticosteroids  **Outcome**: Mortality, length of hospital stay, length of ICU stay (ICU-free days), duration of ventilation (ventilator-free days), infection (pneumonia, sepsis, others)  **Summary of evidence**:  We conducted a meta-analysis of seven RCTs that compared the use of corticosteroids and no corticosteroids in ventilated adult patients with ARDS (P/F ratio of less than 200). Of these, the study by Bernard et al. had a high dose of methylprednisolone (30 mg/kg). Thus, it was separated from the other low dose studies.  <High dose>  The estimated values of desirable anticipated effects were as follows: mortality yielded an RD of 32 fewer per 1000 (1 RCT: N=99) (95% CI: 196 fewer to 183 more). From the above, it was judged that the desirable anticipated effect was “trivial”.  <Low dose>  The estimated values of desirable anticipated effects were as follows: mortality yielded an RD of 105 fewer per 1000 (5 RCTs: N=769) (95% CI: 178 fewer to 7 fewer); infection yielded an RD of 50 fewer per 1000 (5 RCTs: N=769) (95% CI: 97 fewer to 60 more); ventilator-free days yielded an MD of 4.75 days longer (5 RCTs: N=769) (95% CI: 2.97 longer to 6.54 longer); and length of hospital stay yielded an MD of 5.04 days shorter (2 RCTs: N=271) (95% CI: 9.43 shorter to 0.82 shorter). From the above, it was judged that the desirable anticipated effect was “moderate.”  On the contrary, the estimated values of undesirable anticipated effects were as follows:  <High dose>  The estimated values of the undesirable anticipated effects were as follows: infection yielded an RD of 58 more per 1000 (1 RCT: N=99) (95% CI: 46 fewer to 353 more). From the above, it was judged that the undesirable anticipated effect was “small”.  <Low dose>  No undesirable effects were observed at low doses. Therefore, when considered together with the description in the additional considerations, it was judged to be “trivial.”  **Certainty of the evidence:**  <High dose>  The direction of the desirable and undesirable effects was not consistent, and the certainty of evidence across outcomes was judged to be “very low” by adopting the lowest certainty of evidence.  <Low dose>  The direction of the desirable and undesirable effects was consistent, and the certainty of the evidence across the outcomes was judged to be “moderate,” adopting the certainty of the evidence with the highest certainty.  **Values, balance of effects, acceptability, feasibility**：  There is little variation in terms of value for both high and low doses. As for feasibility, corticosteroids do not have an insurance indication for ARDS. However, ARDS is mostly caused by sepsis and pneumonia. Since dexamethasone is indicated by insurance for severe infections, and methylprednisolone and hydrocortisone are indicated for infectious shock, we decided “probably yes” because we believe they can be used in many patients.  As described below, the balance of effects and acceptance differs between high and low doses.  <High dose>  When considering the point estimate of the outcome, per 1,000 people, long-term mortality will decrease by 32, but infection will increase by 58. When confidence intervals are considered, there is a potential increase of 183 deaths and 353 infections. This suggests that the harms may outweigh the benefits of the intervention. The intervention may reduce deaths but increase infections. The intervention is likely to cause significant harm and is not acceptable.  <Low dose>  Considering point estimates of outcomes, per 1000 people, long-term mortality is reduced by 105, and infections are reduced by 50. Although the costs have not been statistically examined, the benefits of the intervention may outweigh the harms, as in addition to reducing deaths, increasing VFDs and reducing ICU and hospital stays may mitigate the costs of administering corticosteroids, which would offset the increased costs. In addition to reducing deaths, interventions may also increase VFDs and reduce ICU and hospital stays, which may result in economic savings that may be acceptable.  **Panel meeting:**  In the pre-vote, the modified Delphi method showed that “recommend the use of corticosteroids in adult patients with ARDS” had a median score of 9.0 and a disagreement index of 0.1316.  **Note**:  The type of corticosteroid and method of administration varies from study to study. Sub-analyses have shown that the net benefit is superior with low dose long-term administration. In addition, previous meta-analyses have suggested that early initiation (within 72 h of onset^5)^, 14 days^6,7)^), and long-term administration (more than 7 days^5,6,7)^) may be beneficial in reducing mortality. |

| Subgroup considerations |
| --- |
| The method of corticosteroid administration varied among studies. Bernard 1986 administered methylprednisolone 30 mg/kg four times daily (high dose methylprednisolone). Meduri 1998, Meduri 2007, and Steinberg 2016 administered methylprednisolone at an initial dose of 1-2 mg/kg/day for 1-14 days, 1-2 mg/kg/day for 1-14 days, and 0.5-1.0 mg/kg/day for 15-21 days, tapering to a maximum dose of 32 days (low dose methylprednisolone). In the Villar 2020 study, corticosteroids were discontinued or tapered when extubated prior to the regimen.  Point estimates sub-analyzed by glycocorticoid titer and the duration of administration show that point estimates indicate increased benefit with lower (Meduri 1998, Meduri 2007, Steinberg 2016, Villar 2020) and longer (Meduri 1998, Meduri 2007, Steinberg 2016, Villar 2020, Tongyoo 2016) doses.   \|  \| Risk deference with corticosteroid vs. control \| \| \| \| --- \| --- \| --- \| --- \| \| Point Estimation \| Upper limit \| Lower limit \| \| Mortality (overall) \| -98 \| -169 \| 0 \| \| Less than 7 days \| -20 \| -122 \| 114 \| \| 7-14 days \| -114 \| -204 \| 20 \| \| More than 15 days \| -145 \| -270 \| 110 \| |
| Implementation considerations |
| Corticosteroids are not covered by insurance for ARDS. However, the causes of ARDS are mostly sepsis and pneumonia. Since dexamethasone is indicated by insurance for severe infections, and methylprednisolone and hydrocortisone are indicated for infectious shock, they may be used in many patients. However, they may not be used in extrapulmonary ARDS for insurance coverage reasons.  The ARDS Guidelines 2016 suggest the use of corticosteroids (methylprednisolone 1 to 2 mg/kg/day equivalent) in adult patients with ARDS (GRADE 2B, strength of recommendation “weak recommendation”/confidence of evidence “medium”). In this CQ, recommendations are presented separately for low and high doses, with the addition of articles published after 2016. |

| Monitoring and evaluation |
| --- |
| Collection of more information on the adverse events and cost-effectiveness is necessary because of clinical problems in implementing the recommendations. In addition, it is necessary to monitor the implementation status of the guideline through the use of questionnaires and other means after the guideline is published to see if there are any other clinical problems. |
| Research priorities |
| We performed a sensitivity analysis in this study of management by restrictive ventilation and confirmed that the direction of the point estimate did not change. However, in recent years, other methods of managing ARDS that improve clinical outcomes have been reported in addition to restrictive ventilation, including supine therapy. Furthermore, the efficacy of corticosteroid administration for ARDS may differ depending on the timing of initiation, duration of administration, and method of tapering. There is a need to unify these factors and study the effects of corticosteroids in the future. |

References

1. Pelosi P, D'Onofrio D, Chiumello D, Paolo S, Chiara G, Capelozzi VL, et al. Pulmonary and extrapulmonary acute respiratory distress syndrome are different. Eur Respir J. 2003;42: 48s-56s. PMID: 12946001.

2. Meduri GU, Siemieniuk RAC, Ness RA, Seyler SJ. Prolonged low-dose methylprednisolone treatment is highly effective in reducing duration of mechanical ventilation and mortality in patients with ARDS. J Intensive Care. 2018;6:53. PMID: 30155260.

3. Ruan SY, Lin HH, Huang CT, Kuo PH, Wu HD, Yu CJ. Exploring the heterogeneity of effects of corticosteroids on acute respiratory distress syndrome: a systematic review and meta-analysis. Crit Care. 2014;18(2):R63. PMID: 24708846.

4. Lewis SR, Pritchard MW, Thomas CM, Smith AF. Pharmacological agents for adults with acute respiratory distress syndrome. Cochrane Database Syst Rev. 2019;7(7):CD004477. PMID: 31334568.

5. Hirano Y, Madokoro S, Kondo Y, Okamoto K, Tanaka H. Corticosteroid treatment for early acute respiratory distress syndrome: a systematic review and meta-analysis of randomized trials. J Intensive Care. 2020;8(1):91. PMID: 33722302.

6. Meduri GU, Marik PE, Chrousos GP, Pastores SM, Arlt W, Beishuizen A, et al. Steroid treatment in ARDS: a critical appraisal of the ARDS network trial and the recent literature. Intensive Care Med. 2008;34(1):61-69. PMID: 18000649.

7. Meduri GU, Bridges L, Shih MC, Marik PE, Siemieniuk RAC, Kocak M. Prolonged glucocorticoid treatment is associated with improved ARDS outcomes: analysis of individual patients’ data from four randomized trials and trial-level meta-analysis of the updated literature. Intensive Care Med. 2016;42(5):829-840. PMID: 26508525.

**CQ43 Should early rehabilitation intervention be conducted for adult patients with ARDS?**

1. Search strategy

MEDLINE via PubMed （Search date: 2020/7/6）

| #1 | "Respiration, Artificial"[mh] OR "mechanical ventilation"[tiab] OR "Noninvasive Ventilation"[mh] OR "non invasive ventilation"[tiab] OR "intermittent mandatory ventilation"[tiab] OR "invasive ventilation"[tiab] OR "mechanically ventilated"[tiab] OR "Tracheostomy"[mh] OR "tracheostomy"[tiab] OR NPPV[tiab] OR IPV[tiab] OR TPPV[tiab] OR NIPPV[tiab] OR NIV[tiab] OR IMV[tiab] OR "Intermittent Positive-Pressure Ventilation"[mh] |
| --- | --- |
| #2 | "Early Ambulation"[mh] OR "mobilization"[tiab] OR "Rehabilitation"[mh] OR "rehabilitation"[tiab] OR "exercise therapy"[tiab] OR "physical therapy"[tiab] OR "occupational therapy"[tiab] OR mobilizat*[tiab] OR mobilisat*[tiab] OR mobility[tiab] OR exercis*[tiab] OR training[tiab] OR pregait[tiab] OR pre-gait[tiab] OR walk*[tiab] OR adl[tiab] OR physiotherap*[tiab] OR ambulation[tiab] |
| #3 | ergomet*[tiab] OR Ergometry[mh] OR cycl*[tiab] OR "lower extremit*"[tiab] OR "upper extremit*"[tiab] OR "lower limb"[tiab] OR "upper limb"[tiab]OR "muscle strength*"[tiab] OR endurance[tiab] OR "Range of Motion, Articular"[mh] |
| #4 | "Electric Stimulation Therapy"[mh] OR ((muscle* OR muscular OR neuromuscular OR neuro-muscular) AND electric* AND stimulat*[tiab]) OR NMES[tiab] |
| #5 | #2 OR #3 OR #4 |
| #6 | #1 AND #5 |
| #7 | "randomized controlled trial"[pt] OR "controlled clinical trial"[pt] OR randomized[tiab] OR placebo[tiab] OR randomly[tiab] OR trial[tiab] OR groups[tiab] |
| #8 | "systematic review"[pt] OR meta-analysis[pt] |
| #9 | #7 OR #8 |
| #10 | Animals[mh] NOT Humans[mh] |
| #11 | #9 NOT #10 |
| #12 | #6 AND #11 |

CENTRAL （Search date: 2020/7/6）

| #1 | [mh "Respiration, Artificial"] OR [mh "Noninvasive Ventilation"] OR "intermittent mandatory ventilation":ti,ab OR "mechanical ventilation":ti,ab OR "non invasive ventilation":ti,ab OR "invasive ventilation":ti,ab OR "mechanically ventilated":ti,ab OR [mh Tracheostomy] OR tracheostomy:ti,ab OR NPPV:ti,ab OR NIPPV:ti,ab OR NIV:ti,ab OR IMV:ti,ab OR IPV:ti,ab OR TPPV:ti,ab OR [mh "Positive-Pressure Respiration"] OR [mh "Intermittent Positive-Pressure Ventilation"] |
| --- | --- |
| #2 | [mh "Early Ambulation"] OR [mh Rehabilitation] OR ambulation:ti,ab OR mobilization:ti,ab OR rehabilitation:ti,ab OR "Exercise Therapy":ti,ab OR "Physical Therapy":ti,ab OR "Occupational Therapy":ti,ab OR mobilization:ti,ab OR mobilisation:ti,ab OR mobility:ti,ab OR exercise:ti,ab OR training:ti,ab OR pregait:ti,ab OR pre-gait:ti,ab OR walk:ti,ab OR adl:ti,ab OR physiotherapy:ti,ab OR ambulation:ti,ab |
| #3 | [mh Ergometry] OR ergometry:ti,ab OR cycle:ti,ab OR "lower extremities":ti,ab OR "upper extremities":ti,ab OR [mh "Range of Motion, Articular"] OR "lower limb":ti,ab OR "upper limb":ti,ab OR "muscle strength":ti,ab OR endurance:ti,ab |
| #4 | [mh "Electric Stimulation Therapy"] OR NMES:ti,ab |
| #5 | muscle:ti,ab OR muscular:ti,ab OR neuromuscular:ti,ab OR neuro-muscular:ti,ab |
| #6 | {OR #2-#5} |
| #7 | #1 AND #6 |
| #8 | [mh Animals] NOT [mh Humans] |
| #9 | #7 NOT #8 |

1. Flow diagram

**Identification**

8 Studies included in qualitative synthesis

97 Full-text articles assessed for eligibility

5398 records after duplicates removed

5402 records identified through database searching

5402 records identified through database searching

Medline via PubMed (n=2234)

CENTRAL (n=2395)

Igaku-Chuo-Zasshi (n=773)

0 additional records identified through other sources

8 Studies included in quantitative synthesis (meta-analysis)

89 Full-text articles excluded, with reasons:

・Wrong language (n=3)

・Wrong study design (n=28)

・Wrong intervention (n=7)

・Duplication (n=19)

・Awaiting for classification (n=32)

Duplicates

n=4

5301 records excluded

**Included**

**Eligibility**

**Screening**

1. Risk of bias

Mortality ICU-AW

**
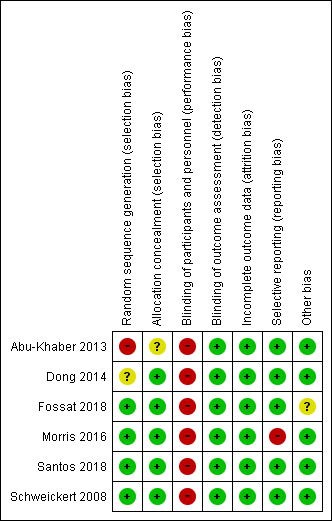

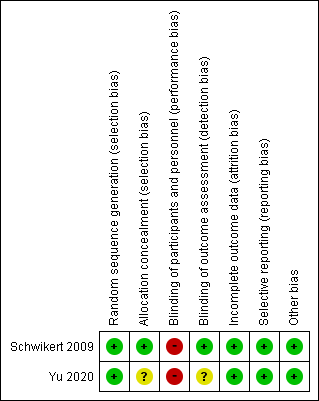
**

Duration of mechanical ventilation Length of ICU stay

**
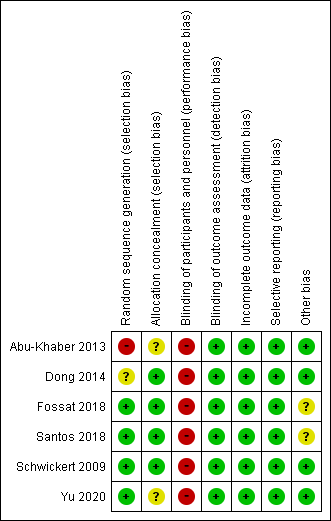

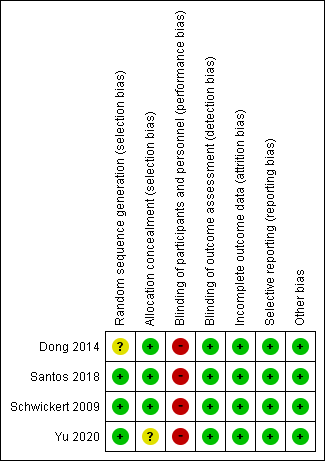
**

**
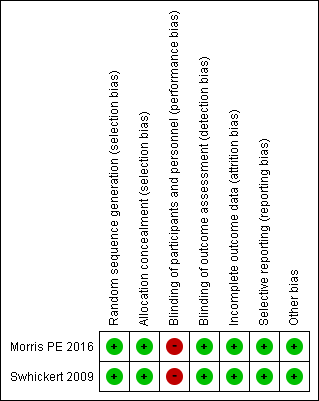
**Length of hospital stay Adverse events

**
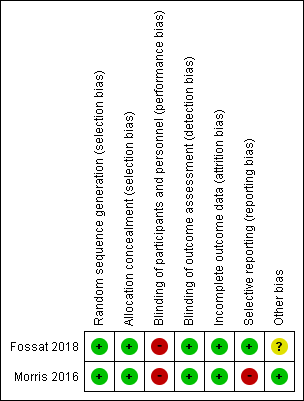
**

1. Forest plot

Mortality

**
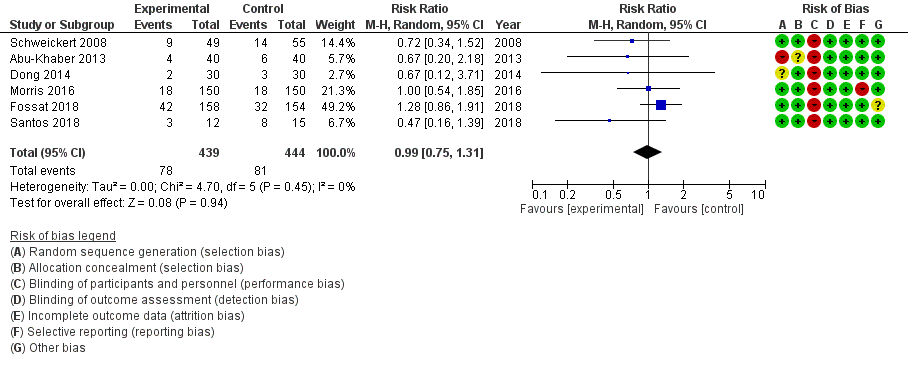
**

ICU-AW

**
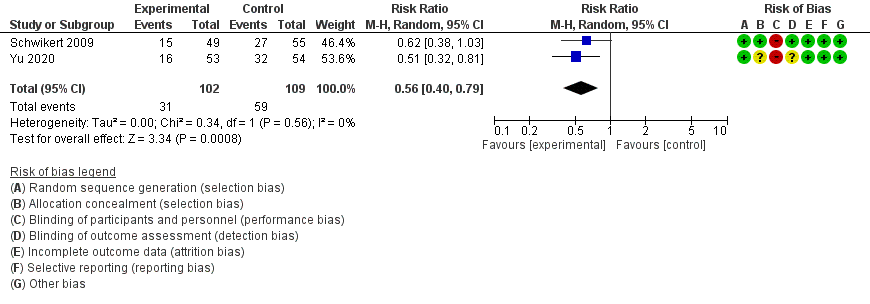
**

Duration of mechanical ventilation

**
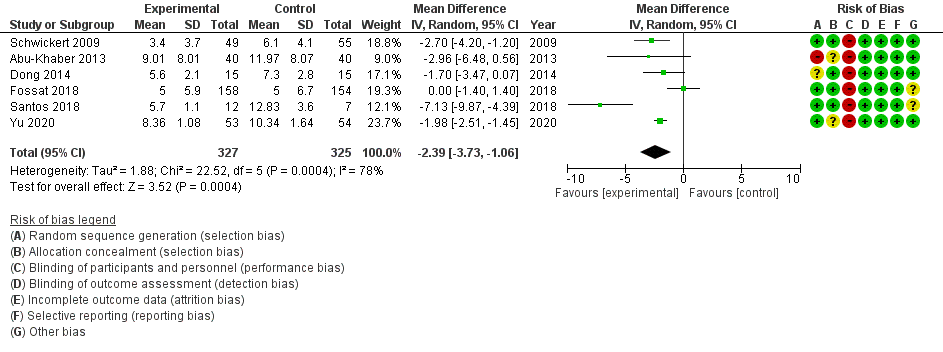
**

Length of ICU stay

**
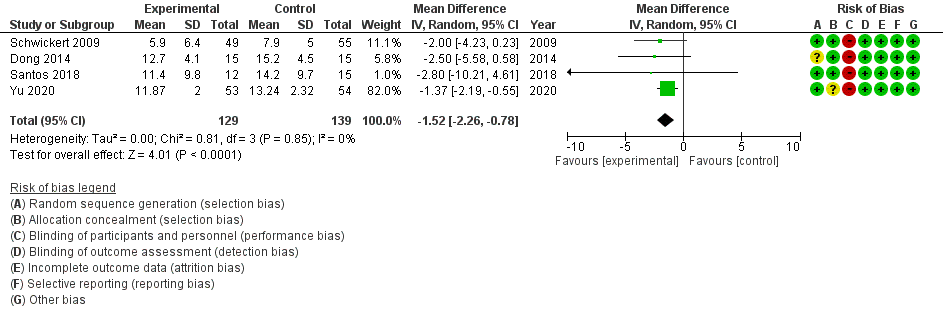
**

Length of hospital stay

**
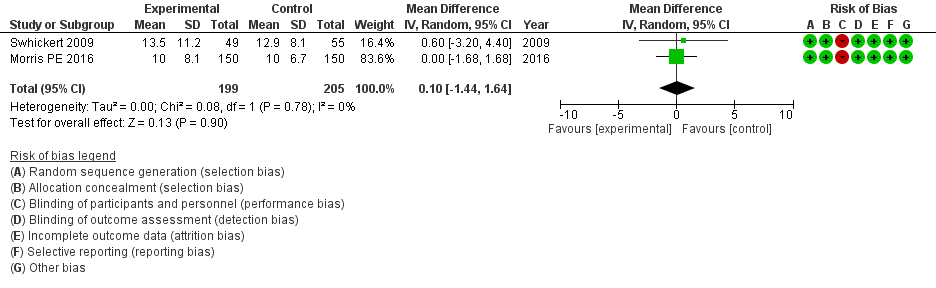
**

Adverse events

**
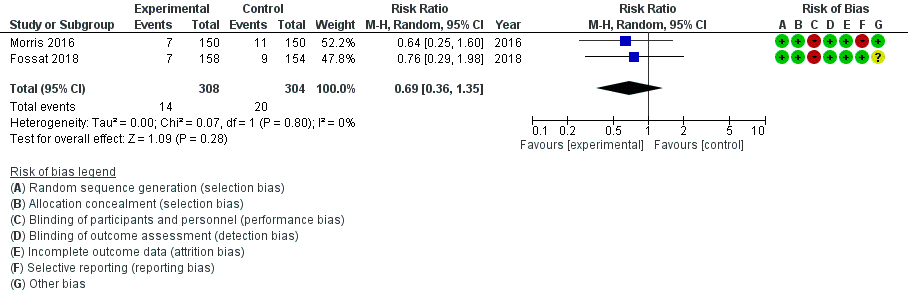
**

1. Evidence profile

| **Assessment of certainty** | | | | | | | | | | | | | **№ of patients** | | | | **Effect** | | | **Certainty** | | | **Importance** |  |  |
| --- | --- | --- | --- | --- | --- | --- | --- | --- | --- | --- | --- | --- | --- | --- | --- | --- | --- | --- | --- | --- | --- | --- | --- | --- | --- |
| **№ of studies** | | **Study design** | | **Risk of bias** | | **Inconsistency** | | **Indirectness** | | **Imprecision** | **Others** | | **Early rehabilitation** | | **No early rehabilitation** | | **Relative index (95% CI)** | | **Absolute index (95% CI)** |  |  |  |  |  |  |
| **Mortality** | | | | | | | | | | | | | | | | | | | | | | | |  |  |
| 6 | | RCT | | Not serious | | Not serious | | Serious ^a^ | | Serious ^b^ | None | | 78/439 (17.8%) | | 81/444 (18.2%) | | RR 0.99 (0.75 to 1.31) | | **2 fewer per 1000**  (46 fewer ～ 57more) | ⨁⨁◯◯ Low | | | Critical |  |  |
| PICS_ICU-AW | | | | | | | | | | | | | | | | | | | | | | | |  |  |
| 2 | | RCT | | Not serious | | Not serious | | Serious ^a^ | | Serious ^c^ | None | | 31/102 (30.4%) | | 59/109 (54.1%) | | RR 0.56 (0.40 to 0.79) | | **238 fewer per 1000** (325 fewer ～ 114 fewer) | ⨁⨁◯◯ Low | | | Critical |  |  |
| Duration of mechanical ventilation | | | | | | | | | | | | | | | | | | | | | | | |  |  |
| 6 | | RCT | | Not serious | | Serious ^d^ | | Serious ^a^ | | Not serious | None | | 327 | | 325 | | - | | MD **2.39 days shorter** (3.73 shorter ～ 1.06 shorter) | ⨁⨁◯◯ Low | | | Critical |  |  |
| Length of ICU stay | | | | | | | | | | | | | | | | | | | | | | | |  |  |
| 4 | | RCT | | Not serious | | Not serious | | Serious ^a^ | | Serious ^c^ | None | | 129 | | 139 | | - | | MD **1.52 days shorter** (2.26 shorter ～ 0.78 shorter) | ⨁⨁◯◯ Low | | | Critical |  |  |
| Length of hospital stay | | | | | | | | | | | | | | | | | | | | | | | |  |  |
| 2 | | RCT | | Not serious | | Not serious | | Serious ^a^ | | Serious ^b^ | None | | 199 | | 205 | | - | | MD **0.1 days longer**  (1.44 shorter ～ 1.64 longer) | ⨁⨁◯◯ Low | | | Critical |  |  |
| Adverse events | | | | | | | | | | | | | | | | | | | | | | | |  |  |
| 2 | | RCT | | Not serious | | Not serious | | Serious ^a^ | | Serious ^b^ | None | | 14/308 (4.5%) | | 20/304 (6.6%) | | RR 0.69 (0.36 to 1.35) | | **20 fewer per 1000** (42 fewer ～ 23 more) | ⨁⨁◯◯ Low | | | Critical |  |  |
| **Return to home** | | | | | | | | | | | | | | | | | | | | | | | | | |
| 0 | | - | | - | | - ^a^ | | -- | | - | | | - | | - | | - | Not estimated | - | | | - | Critical | | |

**CI:** confidence interval; **RR:** risk ratio; **MD:** mean difference; RCT: randomized controlled trial

Return to home is not included in the evidence profile because the outcomes are not reported.

#### Explanation

a. In patients with respiratory failure requiring artificial respiration

b. Because the 95% confidence interval crosses the threshold for clinical judgment

c. The Optimal information size (OIS) was not met

d. I2=78%.

1. Evidence-to-Decision table

| Question | |
| --- | --- |
| CQ43： Should early rehabilitation intervention be conducted for adult patients with ARDS? | |
| **Population:** | Patients with respiratory failure that require mechanical ventilation |
| **Intervention:** | Early rehabilitation (initiation within 72 hours of intensive care unit (ICU) admission. Includes early mobilization) |
| **Comparison:** | Standard care |
| **Main outcomes:** | Mortality, post-intensive care syndrome (PICS), return to home, duration of mechanical ventilation or ventilator-free days, length of ICU stay, length of hospital stay, adverse events |
| **Setting:** | Emergency room (ER) or ICU |
| **Perspective:** | Individual |
| **Background:** | Patients with ARDS show a long-term decline in physical and mental functioning even after surviving ICU discharge ^1)^. Since PICS is a common problem in critically ill patients, including ARDS, early rehabilitation is provided during ICU stays to prevent PICS. It has been reported that early rehabilitation improves physical function in critically ill and ventilated patients ^2,3)^. There is no systematic review of early rehabilitation only for respiratory failure patients, including ARDS patients, to date, and the effectiveness and certainty of the evidence are unknown. |
| **Conflict of interests:** | None |

# Assessment

| Problem Is the problem a priority? | | |
| --- | --- | --- |
| Judgment | research evidence | ADDITIONAL CONSIDERATIONS |
| ○ No  ○ Probably no  ● Probably yes  ○ Yes  ○ Varies  ○ Do not know | Patients with ARDS show a long-term decline in physical and mental functioning even after surviving ICU discharge. Since PICS is a common problem in critically ill patients, including ARDS, early rehabilitation is provided during ICU stay to prevent PICS. It has been reported that early rehabilitation improves physical function in critically ill and ventilated patients. On the contrary, the effect of early rehabilitation only for respiratory failure patients, including ARDS patients, is unknown, and it is an important clinical issue to clarify the effectiveness of early rehabilitation. |  |
| Desirable effects How substantial are the desirable anticipated effects? | | |
| Judgment | research evidence | ADDITIONAL CONSIDERATIONS |
| ○ Trivial  ○ Small  ● Moderate  ○ Large  ○ Varies  ○ Do not know | A meta-analysis was performed using eight randomized controlled trials (RCTs). The estimated values of desirable anticipated effects were as follows: mortality yielded an RD of 2 fewer per 1000 (6 RCTs: N=883) (95% CI: 46 fewer to 57 more); ICU-AW yielded an RD of 238 fewer per 1000 (2 RCTs: N=211) (325 fewer to 114 fewer); duration of mechanical ventilation yielded an MD of 2.39 days shorter (6 RCTs: N=652) (3.73 shorter to 1.06 shorter); length of ICU stay yielded an MD of 1.25 days shorter (4 RCTs: N=268) (2.26 shorter to 0.78 shorter); length of hospital stay yielded an MD of 0.1 days longer (2 RCTs: N=404) (1.44 shorter to 1.64 longer). From the above, it was judged that the desirable anticipated effect was “moderate.” |  |
| Undesirable effects How substantial are the undesirable anticipated effects? | | |
| Judgment | research evidence | ADDITIONAL CONSIDERATIONS |
| ○ Large  ○ Moderate  ○ Small  ● Trivial  ○ Varies  ○ Do not know | The estimated values of the undesirable anticipated effects were as follows: adverse events yielded an RD of 20 fewer per 1000 (2 RCTs: N=612) (95% CI: 42 fewer to 23 more). Therefore, when considered together with additional considerations, it was judged to be “trivial.” | Other RCTs of the harms of early rehabilitation reported unplanned extubation of tracheal tubes, accidental extubation of arterial catheters, and allergy to electrical stimulation pads in the intervention group, albeit in one case each. |
| Certainty of evidence What is the overall certainty of the evidence of effects? | | |
| Judgment | research evidence | ADDITIONAL CONSIDERATIONS |
| ○ Very low  ● Low  ○ Moderate  ○ High  ○ No included studies | \| **Outcome** \| **Importance** \| **Certainty of the evidence** \| \| --- \| --- \| --- \| \| \| \| Mortality \| Critical \| ⨁⨁◯◯ \| \| Low \| \| PICS (ICU-AW) \| Critical \| ⨁⨁◯◯ \| \| Low \| \| Duration of mechanical ventilation \| Critical \| ⨁⨁◯◯ \| \| Low \| \| Length of ICU stay \| Critical \| ⨁⨁◯◯ \| \| Low \| \| Length of hospital stay \| Critical \| ⨁⨁◯◯ \| \| Low \| \| Adverse events \| Critical \| ⨁⨁◯◯ \| \| Low \| \| Return to home \| Critical \| - \| \| - \|   No RCTs reported a return to home as an outcome.  **Overall certainty of the evidence**：  The certainty of all outcomes was “low.” |  |
| Values Is there important uncertainty about or variability in how much people value the main outcomes? | | |
| Judgment | research evidence | ADDITIONAL CONSIDERATIONS |
| ○ Important uncertainty or variability  ○ Possibly important uncertainty or variability  ○ Probably no important uncertainty or variability  ● No important uncertainty or variability | No important uncertainty or variability. |  |
| Balance of effects Does the balance between desirable and undesirable effects favor the intervention or the comparison? | | |
| Judgment | research evidence | ADDITIONAL CONSIDERATIONS |
| ○ Favors the comparison  ○Probably favors the comparison  ○ Does not favor either the intervention or the comparison  ● Probably favors the intervention  ○Favors the intervention  ○ Varies  ○Do not know | \| Outcome \| Comparison \| Intervention  (Early rehabilitation) \| Absolute difference \| Relative effect RR (95% CI) \| \| --- \| --- \| --- \| --- \| --- \| \| Mortality \| 81 / 444  (18.2%) \| 78 / 439  (17.8%) \| 2 fewer/ 1,000 (46 fewer～57 more) \| RR 0.99 (0.75～1.31) \| \| PICS (ICU-AW) \| 59 / 109  (54.1%) \| 32 / 102  (30.4%) \| 238 fewer/1,000 (325 fewer～114 fewer) \| RR 0.56 (0.40～0.79) \| \| Duration of ventilation \| - \| - \| MD 2.39 days shorter (3.73 shorter～1.06 shorter) \| - \| \| Length of ICU stay \| - \| - \| MD 1.52 days shorter (2.26 shorter～0.78 shorter) \| - \| \| Length of hospital stay \| - \| - \| MD 0.1 days longer  (1.44 shorter～1.64 longer) \| - \| \| Adverse events \| 20 / 304  (6.8%) \| 14 / 308  (4.5%) \| 20 fewer / 1,000 (42 fewer ～23 more) \| RR 0.69 (0.36～1.35) \| \| Return to home \| - \| - \| No estimate \| - \|     Based on the above, we concluded that the balance between the effects and harms of intervention was “probably favors the intervention.” |  |
| Acceptability Is the intervention acceptable to key stakeholders? | | |
| Judgment | research evidence | ADDITIONAL CONSIDERATIONS |
| ○ No  ○ Probably no  ● Probably yes  ○ Yes  ○ Varies  ○ Do not know○ | It was decided that the possibility of reducing the costs would probably outweigh the benefits of the intervention, so it was probably acceptable. |  |
| Feasibility Is the intervention feasible to implement? | | |
| Judgment | research evidence | ADDITIONAL CONSIDERATIONS |
| ○ No  ○ Probably no  ● Probably yes  ○ Yes  ○ Varies  ○ Do not know | If the facility is accustomed to conducting early rehabilitation, has physical therapists, occupational therapists, and speech therapists assigned to intensive care, or has appropriate staffing such as an ICU with 2:1 nursing care, it is highly feasible. |  |

# Summary of judgment

|  | **Judgment** | | | | | | |
| --- | --- | --- | --- | --- | --- | --- | --- |
| **PROBLEM** | No | Probably no | Probably yes | Yes |  | Varies | Do not know |
| **DESIRABLE EFFECTS** | Trivial | Small | Moderate | Large |  | Varies | Do not know |
| **UNDESIRABLE EFFECTS** | Large | Moderate | Small | Trivial |  | Varies | Do not know |
| **CERTAINTY OF EVIDENCE** | Very low | Low | Moderate | High |  |  | No included studies |
| **VALUES** | Important uncertainty or variability | Possibly important uncertainty or variability | Probably no important uncertainty or variability | No important uncertainty or variability |  |  |  |
| **BALANCE OF EFFECTS** | Favors the comparison | Probably favors the comparison | Does not favor either the intervention or the comparison | Probably favors the intervention | Favors the intervention | Varies | Do not know |
| **ACCEPTABILITY** | No | Probably no | Probably yes | Yes |  | Varies | Do not know |
| **FEASIBILITY** | No | Probably no | Probably yes | Yes |  | Varies | Do not know |

# Type of Recommendation

| Strong recommendation against the intervention | Conditional recommendation against the intervention | Conditional recommendation for either the intervention or the comparison | Conditional recommendation for the intervention | Strong recommendation for the intervention |
| --- | --- | --- | --- | --- |
| ○ | ○ | ○ | ● | ○ |

# Conclusions

| Recommendation |
| --- |
| **We suggest conducting early rehabilitation (within 72 hours) in patients with ARDS**  **(conditional recommendation/low: GRADE 2C).** |
|  |
| Justification |
| **Question:** Should early rehabilitation intervention be conducted for adult patients with ARDS?  **Patients:** Respiratory failure patients requiring mechanical ventilation  **Intervention:**Early rehabilitation (Initiation within 72 hours of ICU admission. Includes early mobilization)  **Comparison:**Standard care  **Outcome:**Mortality, PICS, return to home, duration of mechanical ventilation or ventilator-free days, length of ICU stay, length of hospital stay, adverse events  **Summary of evidence**:　The estimated values of desirable anticipated effects were as follows: mortality yielded an RD of 2 fewer per 1000 (6 RCTs: N=883) (95% CI: 46 fewer to 57 more); ICU-AW yielded an RD of 238 fewer per 1000 (2 RCTs: N=211) (325 fewer to 114 fewer); duration of mechanical ventilation yielded an MD of 2.39 days shorter (6 RCTs: N=652) (3.73 shorter to 1.06 shorter); length of ICU stay yielded an MD of 1.25 days shorter (4 RCTs: N=268) (2.26 shorter to 0.78 shorter); length of hospital stay yielded an MD 0.1 days longer (2 RCTs: N=404) (1.44 shorter to 1.64 longer). From the above, it was judged that the desirable anticipated effect was “moderate.” On the contrary, the estimated values of the undesirable anticipated effects were as follows: adverse events yielded an RD of 20 fewer per 1000 (2 RCTs: N=612) (95% CI: 42 fewer to 23 more). Therefore, when considered together with the additional considerations, it was judged to be “trivial.”  **Certainty of the evidence**: The certainty of all outcomes was “low.”  **Values, balance of effects, acceptability, feasibility**: There is no significant uncertainty or diversity in values. The predicted desirable effects of this intervention are probably superior to the predicted undesirable effects when considered as point estimates. Regarding acceptance, it was judged that the benefits of the intervention probably outweighed the costs and could be reduced. As for feasibility, it is highly feasible if the facility is accustomed to early rehabilitation, has physical therapists, occupational therapists, and speech therapists in charge of intensive care, and has appropriate staffing such as an ICU with 2:1 nursing care.  **Panel meeting:** The pre-vote was “conditionally recommend early (within 72 h) rehabilitation for adult patients with ARDS” by the modified Delphi method, with a median score of 9.0 and a disagreement index of 0.1316. At the panel meeting, there was a discussion about the low outcome of harm in the early rehabilitation intervention group. It was suggested that standardized protocols and facility factors such as workforce availability should be added. As a result, the panel meeting finally reached a consensus on the pre-vote without requiring a re-vote. |

| Subgroup considerations |
| --- |
| Of the RCTs included in this meta-analysis, two examined the effects of neuromuscular electrical stimulation, and one examined the effects of bicycle ergometer exercise alone. The results of the sub-analyses for each are as follows:  Neuromuscular electrical stimulation: Mortality, 110 more per 1,000; Duration of ventilation, 5.2 days shorter; Length of ICU stay, 2.8 days shorter.  Bicycle ergometer: PICS (ICU-AW), 290 fewer per 1000; Duration of ventilation, 1.98 days shorter; Length of ICU stay, 1.37 days shorter. |
| Implementation considerations |
| The types of early rehabilitation interventions include early release, neuromuscular electrical stimulation, and bicycle ergometer, and which interventions to use and to what extent may be influenced by the patient’s condition and the skill level of the healthcare provider. In addition, it should be noted that none of the eight RCTs included in this study included only patients with ARDS. In patients with moderate to severe ARDS, lung-protective ventilation with muscle relaxants and supine management under deep sedation may be used. Therefore, what can be implemented as early rehabilitation may be affected by other interventions. |

| Monitoring and evaluation |
| --- |
| In order to implement the recommendations, it is necessary to collect further information on the extent to which early rehabilitation is adhered to as a clinical problem. In addition, it is necessary to monitor whether there are any other clinical problems through questionnaires after the guidelines are published. |
| Research priorities |
| Not all RCTs employed in this meta-analysis included only patients with ARDS. The evidence is insufficient, and it is hoped that future large RCTs will be conducted in patients with ARDS according to the type of early rehabilitation intervention. |

References

1. Herridge MS, Tansey CM, Matté A, Tomlinson G, Diaz-Granados N, Cooper A, et al. Functional disability 5 years after acute respiratory distress syndrome. N Engl J Med. 2011;364(14):1293-1304. PMID: 21470008.

2. Tipping CJ, Harrold M, Holland A, Romero L, Nisbet T, Hodgson CL. The effects of active mobilisation and rehabilitation in ICU on mortality and function: a systematic review. Intensive Care Med. 2017;43(2):171-183. PMID: 27864615.

3. Doiron KA, Hoffmann TC, Beller EM. Early intervention (mobilization or active exercise) for critically ill adults in the intensive care unit. Cochrane Database Syst Rev. 2018;3(3):CD010754. PMID: 29582429.

**CQ44 Should non-sedative or light-sedative management be conducted for adult patients with ARDS?**

1. Search strategy

MEDLINE via PubMed（Search date: 2020/6/18）

| #1 | Respiratory Distress Syndrome, Adult[mh] OR ARDS[tiab] OR respiratory distress syndrome[tiab] OR Acute Lung Injury[mh] OR acute lung injury[tiab] OR ALI[tiab] |
| --- | --- |
| #2 | Critical Illness[mh] OR Critical Care[mh] OR Intensive Care Units[mh] OR critically ill[tiab] OR critical care[tiab] OR intensive care[tiab] |
| #3 | #1 OR #2 |
| #4 | Respiration, Artificial[mh] OR ventilators, mechanical[mh] OR ventilator[tiab] OR Ventilators[tiab] OR ventilation[tiab] OR ventilations[tiab] |
| #5 | sedation*[tiab] OR deep sedation[mh] OR Conscious Sedation[mh] OR Hypnotics and Sedatives[mh] |
| #6 | #3 AND #4 AND #5 |
| #7 | (randomized controlled trial[pt] OR controlled clinical trial[pt] OR randomized[tiab] OR placebo[tiab] OR clinical trials as topic[mesh:noexp] OR randomly[tiab] OR trial[ti]) NOT (animals[mh] NOT humans[mh]) |
| #8 | #6 AND #7 |

CENTRAL（Search date: 2020/6/18）

| #1 | [mh "Respiratory Distress Syndrome, Adult"] OR ARDS:ti,ab OR "respiratory distress syndrome":ti,ab OR [mh "Acute Lung Injury"] OR "acute lung injury":ti,ab |
| --- | --- |
| #2 | [mh "Critical Illness"] OR [mh "Critical Care"] OR [mh "Intensive Care Units"] OR "critically ill":ti,ab OR "critical care":ti,ab OR "intensive care":ti,ab |
| #3 | #1 OR #2 |
| #4 | [mh "Respiration, Artificial"] OR [mh "ventilators, mechanical"] OR ventilator:ti,ab OR Ventilators:ti,ab OR ventilation:ti,ab OR ventilations:ti,ab |
| #5 | sedation*:ti,ab OR [mh "deep sedation"] OR [mh "Conscious Sedation"] OR [mh "Hypnotics and Sedatives"] |
| #6 | #3 AND #4 AND #5 |

1. Flow diagram

**Identification**

6 Studies included in qualitative synthesis

33 Full-text articles assessed for eligibility

1138 records after duplicates removed

1597 records identified through database searching

1597 records identified through database searching

Medline via PubMed (n=628)

CENTRAL (n=926)

Igaku-Chuo-Zasshi (n=43)

0 additional records identified through other sources

6 Studies included in quantitative synthesis (meta-analysis)

27 Full-text articles excluded, with reasons:

・Wrong language (n=3)

・Wrong study design (n=3)

・Wrong intervention (n=21)

Duplicates

n=459

1105 records excluded

**Included**

**Eligibility**

**Screening**

1. Risk of bias

Survival discharge Length of ICU stay

**
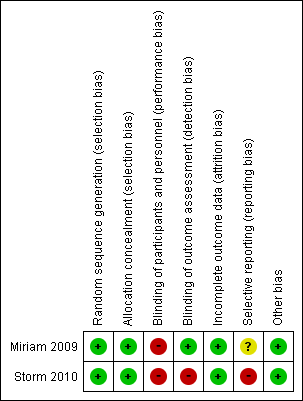

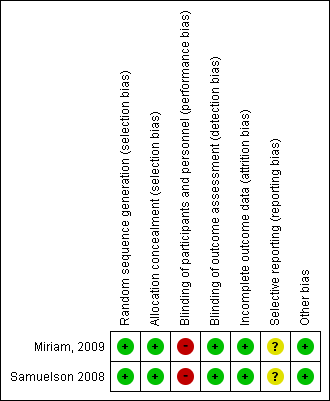
**

Ventilator-free days Tracheostomy

**
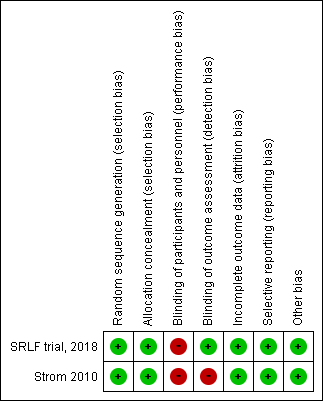

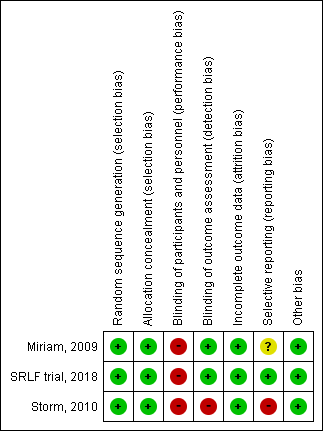
**

Accidental extubation VAP

**
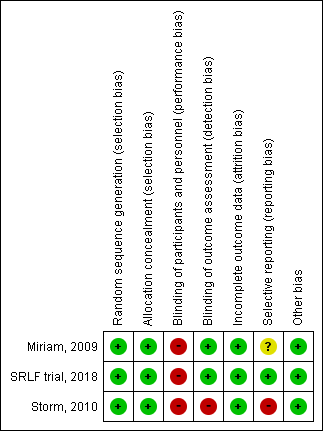

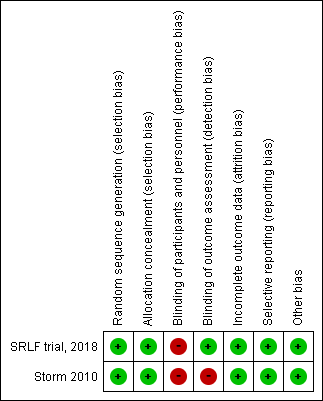
**

Number and duration of patients requiring physical restraint


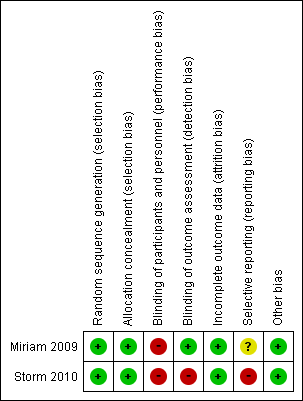


1. Forest plot

Survival discharge

**
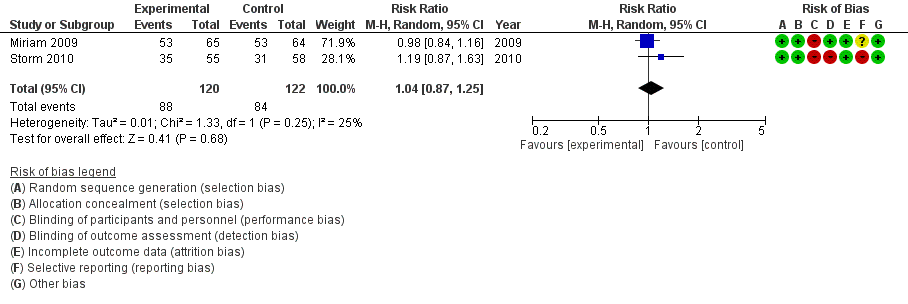
**

Length of ICU stay

**
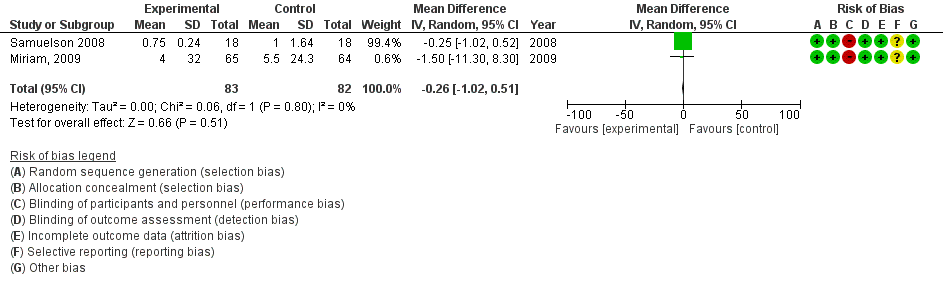
**

Ventilator-free days

**
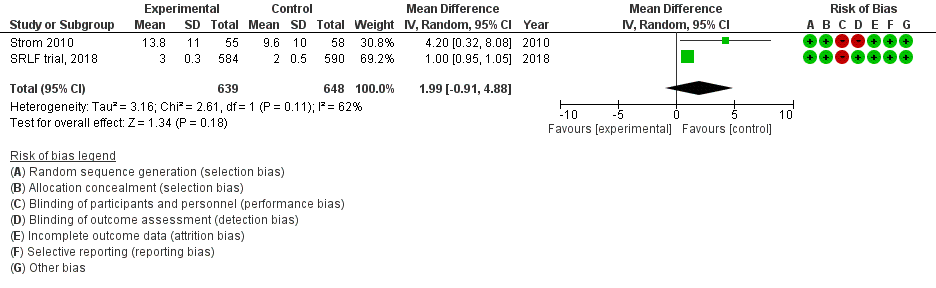
**

Tracheostomy

**
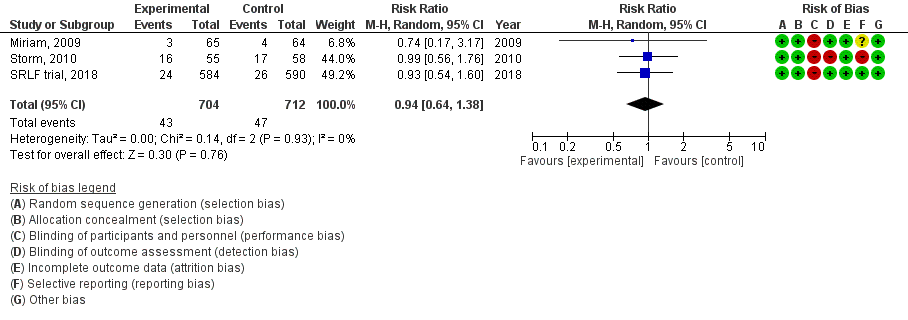
**

Accidental extubation

**
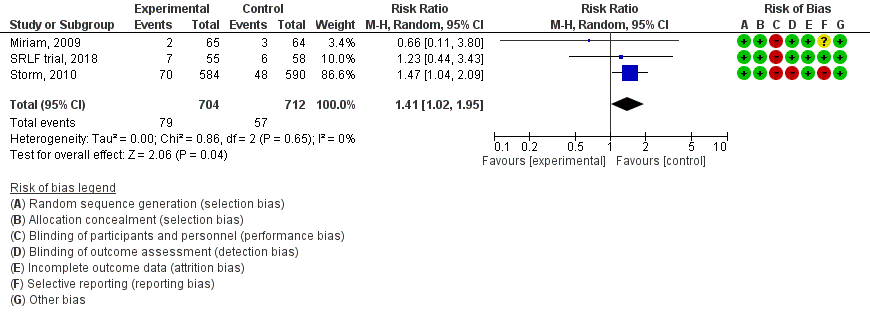
**

VAP

**
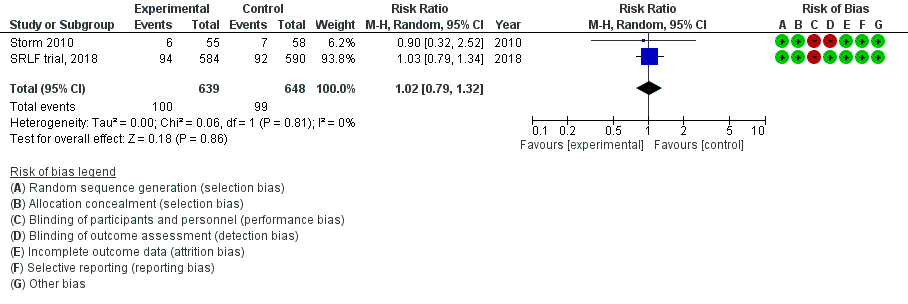
**

Number and duration of patients requiring physical restraint

**
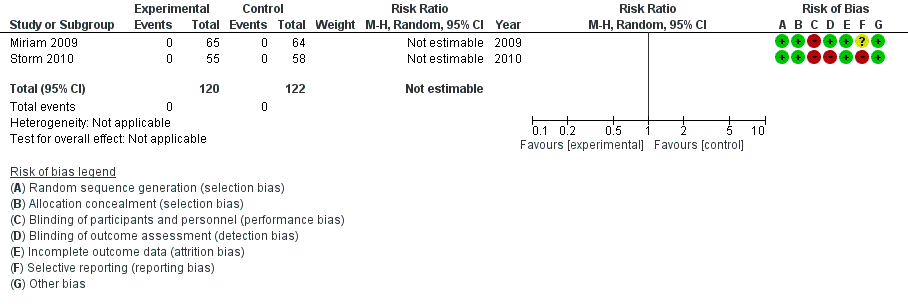
**

1. Evidence profile

| **Assessment of certainty** | | | | | | | | | | | | | **№ of patients** | | | | **Effect** | | | **Certainty** | | **Importance** | | |  |  |  |  |
| --- | --- | --- | --- | --- | --- | --- | --- | --- | --- | --- | --- | --- | --- | --- | --- | --- | --- | --- | --- | --- | --- | --- | --- | --- | --- | --- | --- | --- |
| **№ of studies** | | **Study design** | | **Risk of bias** | | **Inconsistency** | | **Indirectness** | **Imprecision** | | **Others** | | **No sedation/ light sedation** | | **Others** | | **Relative index (95% CI)** | | **Absolute index (95% CI)** |  |  |  |  |  |  |  |  |  |
| **Survival discharge** | | | | | | | | | | | | | | | | | | | | | | | | |  |  |  |  |
| 2 | | RCT | | Not serious | | Not serious | | Serious ^a^ | Serious ^b^ | | None | | 88/120 (73.3%) | | 84/122 (68.9%) | | RR 1.04 (0.87 to 1.25) | | **28 more per 1000**  (90 fewer ～ 172 more) | ⨁⨁◯◯ Low | | Critical | | |  |  |  |  |
| Length of ICU stay | | | | | | | | | | | | | | | | | | | | | | | | |  |  |  |  |
| 2 | | RCT | | Not serious | | Not serious | | Serious ^a^ | Serious ^b^ | | None | | 83 | | 82 | | - | | MD **0.26 days shorter**  (1.02 shorter ～ 0.51 longer) | ⨁⨁◯◯ Low | | Critical | | |  |  |  |  |
| Ventilator Free Days | | | | | | | | | | | | | | | | | | | | | | | | |  |  |  |  |
| 2 | | RCT | | Not serious | | Not serious | | Serious ^a^ | Serious ^b^ | | None | | 639 | | 648 | | - | | MD **1.99 days longer** (0.91 shorter ～ 4.88 longer) | ⨁⨁◯◯ Low | | Critical | | |  |  |  |  |
| Tracheotomy | | | | | | | | | | | | | | | | | | | | | | | | |  |  |  |  |
| 3 | | RCT | | Not serious | | Not serious | | Serious ^a^ | Very serious ^c^ | | None | | 43/704 (6.1%) | | 47/712 (6.6%) | | RR 0.94 (0.64 to 1.38) | | **4 fewer per 1000**  (24 fewer ～ 25 more) | ⨁◯◯◯ Very low | | Critical | | |  |  |  |  |
| Accidental extubation | | | | | | | | | | | | | | | | | | | | | | | | |  |  |  |  |
| 3 | | RCT | | Not serious | | Not serious | | Serious ^a^ | Serious ^b^ | | None | | 79/704 (11.2%) | | 57/712 (8.0%) | | RR 1.41 (1.02 to 1.95) | | **33 more per 1000**  (2 more ～ 76 more) | ⨁⨁◯◯ Low | | Critical | | |  |  |  |  |
| VAP | | | | | | | | | | | | | | | | | | | | | | | | |  |  |  |  |
| 2 | | RCT | | Not serious | | Not serious | | Serious ^a^ | Serious ^b^ | | None | | 100/639 (15.6%) | | 99/648 (15.3%) | | RR 1.02 (0.79 to 1.32) | | **3 more per 1000**  (32 fewer ～ 49 more) | ⨁⨁◯◯ Low | | Critical | | |  |  |  |  |
| Number and duration of patients requiring physical restraint | | | | | | | | | | | | | | | | | | | | | | | | |  |  |  |  |
| 2 | | RCT | | Serious ^d^ | | Not serious | | Serious ^a^ | Serious ^b^ | | None | | 0/120 (0.0%) | | 0/122 (0.0%) | | Disintegration | | Disintegration | ⨁◯◯◯ Very low | | Important | | |  |  |  |  |
| **Survival at 3 months or 6 months after discharge from hospital** | | | | | | | | | | | | | | | | | | | | | | | | | | |  |  |
| 0 | | - | | - | | - ^a^ | | -- | | | - | | - | | - | | - | Not estimated | - | | | **-** | | | Important | | |  |
| **Physical function (6min walk、QoL score)** | | | | | | | | | | | | | | | | | | | | | | | | | | |  |  |
| 0 | | - | | - | | - ^a^ | | -- | | | - | | - | | - | | - | Not estimated | - | | | **-** | | Important | | | |  |
| **Delirium free days** | | | | | | | | | | | | | | | | | | | | | | | | | | |  |  |
| 0 | | - | | - | | - ^a^ | | -- | | | - | | - | | - | | - | Not estimated | - | | | **-** | | Important | | | |  |
| **Mental status (anxiety, depression, PTSD score)** | | | | | | | | | | | | | | | | | | | | | | | | | | | | |
| 0 | | | - | | - | | - ^a^ | | -- | | | - | | - | | - | | - | Not estimated | - | | | **-** | | | Important | | |

**CI:** confidence interval; **RR:** risk ratio; **MD:** mean difference; QoL: quality of life; ICU: intensive care unit; RCT: randomized controlled trial; VAP: ventilator-associated pneumonia; PTSD: posttraumatic stress disorder

Mental status, survival at 3 months or 6 months after discharge, physical function, and delirium free days aree not included in the evidence profile because the outcomes are not reported.

#### Explanation

a. Incorporate studies with ventilated patients

b. Because it does not meet the optimal information size (OIS)

c. Because the 95% CI straddles the upper and lower thresholds for clinical decision.

d. Because the reporting of the blinded and selected outcomes denotes a high risk or unclear risk

1. Evidence-to-Decision table

| Question | |
| --- | --- |
| CQ44： Should non-sedative or light-sedative management be conducted for adult patients with ARDS? | |
| **Population:** | Adult patients with ARDS or patients that have received more than 12 hours of mechanical ventilation |
| **Intervention:** | No sedation or light sedation |
| **Comparison:** | Traditional sedation |
| **Main outcomes:** | Survival discharge, survival at 3 or 6 months after hospital discharge, physical function (6 munites walk, QOL score), Mental status (anxiety, depression, PTSD score), length of intensive care unit (ICU) stay, ventilator-free days, tracheotomy, accidental extubation, VAP |
| **Setting:** | Emergency room or ICU |
| **Perspective:** | Individual |
| **Background:** | Although adequate sedation is sometimes required for ventilatory management, high doses of sedatives may prolong the duration of ventilatory use and increase the risk of delirium, so it is expected that no sedation/light sedation will be effective and avoid complications. Sedation is not an established treatment for the ventilatory management of patients with ARDS, and clarification of its efficacy is an important clinical issue. |
| **Conflict of interests:** | None |

# Assessment

| Problem Is the problem a priority? | | |
| --- | --- | --- |
| Judgment | research evidence | ADDITIONAL CONSIDERATIONS |
| ○ No  ○ Probably no  ● Probably yes  ○ Yes  ○ Varies  ○ Do not know | Although adequate sedation is sometimes required for ventilatory management, high doses of sedatives may prolong the duration of ventilatory use and increase the risk of delirium, so it is expected that no sedation/light sedation will be effective and avoid complications. No sedation/light sedation is not an established therapy for ventilatory management of patients with ARDS, and clarifying its efficacy is an important clinical issue. |  |
| Desirable effects How substantial are the desirable anticipated effects? | | |
| Judgment | research evidence | ADDITIONAL CONSIDERATIONS |
| ○ Trivial  ● Small  ○ Moderate  ○ Large  ○ Varies  ○ Do not know | A meta-analysis was performed using four randomized controlled trials (RCTs). The estimated values of desirable anticipated effects were as follows: survival discharge yielded an RD of 28 more per 1000 (2 RCTs: N=242) (95% CI: 90 fewer to 172 more); length of ICU stay yielded an MD of 0.26 days shorter (2 RCTs: N=165) (1.02 shorter to 0.51 longer); ventilator-free days yielded an MD of 2 days longer (2 RCTs: N=1287) (0.91 shorter to 4.88 longer). From the above, it was judged that the desirable anticipated effect was “small.” |  |
| Undesirable effects How substantial are the undesirable anticipated effects? | | |
| Judgment | research evidence | ADDITIONAL CONSIDERATIONS |
| ○ Large  ○ Moderate  ● Small  ○ Trivial  ○ Varies  ○ Do not know | The estimated values of undesirable anticipated effects were as follows: tracheotomy yielded an RD of 4 fewer per 1000 (3 RCTs: N=1416) (95% CI: 24 fewer to 25 more); accidental extubation yielded an RD of 33 more per 1000 (3 RCTs: N=1416) (2 more to 76 more); VAP yielded an RD of 3 more per 1000 (2 RCTs: N=1287) (32 fewer to 49 more). From the above, it was judged that the undesirable anticipated effect was small. |  |
| Certainty of evidence What is the overall certainty of the evidence of effects? | | |
| Judgment | research evidence | ADDITIONAL CONSIDERATIONS |
| ● Very low  ○ Low  ○ Moderate  ○ High  ○ No included studies | \| **Outcome** \| **Importance** \| **Certainty of the evidence** \| \| --- \| --- \| --- \| \| \| \| Survival discharge \| Critical \| ⨁⨁◯◯ \| \| Low \| \| Length of ICU stay \| Critical \| ⨁⨁◯◯ \| \| Low \|  \| Ventilator-free days \| Critical \| ⨁⨁◯◯ \| \| --- \| --- \| --- \| \| Low \| \| Tracheotomy \| Critical \| ⨁◯◯◯ \| \| Very low \| \| Accidental extubation \| Critical \| ⨁⨁◯◯ \| \| Low \| \| VAP \| Critical \| ⨁⨁◯◯ \| \| Low \| \| Mental status (anxiety, depression, PTSD score) \| Critical \| - \| \| - \| \| Survival at 3 months or 6 months after hospital discharge \| Critical \| - \| \| - \| \| Physical function (6 min walk, QOL score) \| Critical \| - \| \| - \| \| Delirium free days \| Critical \| - \| \| - \|   No RCTs reported mental status, survival at 3 or 6 months after discharge, physical function, delirium free days as outcomes.  **Overall certainty of the evidence:**  The direction of the desirable and undesirable effects was not consistent. Thus, the overall certainty of the evidence was judged to be “very low.” |  |
| Values Is there important uncertainty about or variability in how much people value the main outcomes? | | |
| Judgment | research evidence | ADDITIONAL CONSIDERATIONS |
| ○ Important uncertainty or variability  ○ Possibly important uncertainty or variability  ○ Probably no important uncertainty or variability  ● No important uncertainty or variability | No important uncertainty or variability. |  |
| Balance of effects Does the balance between desirable and undesirable effects favor the intervention or the comparison? | | |
| Judgment | research evidence | ADDITIONAL CONSIDERATIONS |
| ○ Favors the comparison  ○Probably favors the comparison  ○ Does not favor either the intervention or the comparison  ○ Probably favors the intervention  ○Favors the intervention  ○ Varies  ● Do not know | \| Outcome \| Comparison \| Intervention  (No sedation/light sedation) \| Absolute difference \| Relative effect RR (95% CI) \| \| --- \| --- \| --- \| --- \| --- \| \| Survival discharge \| 84/122  (68.9%) \| 88/120 (73.3%) \| 28 more/ 1,000  (90 fewer to 172 more) \| 1.04  (0.87 to 1.25) \| \| Length of ICU stay \| － \| － \| MD 0.26 days shorter  (1.02 shorter to 0.51 longer) \| － \| \| VFD \| － \| － \| MD 1.99 days longer  (0.91 shorter to 4.88 longer) \| － \| \| Tracheotomy \| 47/712  (6.6%) \| 43/704 (6.1%) \| 4 fewer/ 1,000  (24 fewer to 25 more) \| 0.94  (0.64 to 1.38) \| \| Accidental extubation \| 57/712  (8.0%) \| 79/704 (11.2%) \| 33 more / 1,000  (2 more to 76 more) \| 1.41  (1.02 to 1.95) \| \| VAP \| 99/648  (15.3%) \| 100/639 (15.3%) \| 3 more /1,000  (32 fewer to 49 more) \| 1.02  (0.79 to 1.32) \| \| Survival at 3 or 6 months \| － \| － \| No estimate \| － \| \| Physical function \| － \| － \| No estimate \| － \| \| Mental status \| － \| － \| No estimate \| － \| \| Delirium free days \| － \| － \| No estimate \| － \|   No RCTs reported mental status, survival at three or six months after discharge, physical function, delirium free days as outcomes.  The predicted desired effect of this intervention is almost the same when considered as a point estimate, and the direction of the effect changes when the upper and lower limits of the confidence interval are considered. From the above, we concluded that the balance between benefits and harms is uncertain. |  |
| Acceptability Is the intervention acceptable to key stakeholders? | | |
| Judgment | research evidence | ADDITIONAL CONSIDERATIONS |
| ○ No  ○ Probably no  ● Probably yes  ○ Yes  ○ Varies  ○ Do not know | It is possible that the balance of benefits and harms from the intervention is equivalent to that of the control. The potential increase in accidental extubation may also raise concerns about the increased nursing burden. However, we judged that it was probably acceptable because it would increase the number of survival discharge by up to 172 per 1,000 patients and could potentially reduce costs. |  |
| Feasibility Is the intervention feasible to implement? | | |
| Judgment | research evidence | ADDITIONAL CONSIDERATIONS |
| ○ No  ○ Probably no  ● Probably yes  ○ Yes  ○ Varies  ○ Do not know | If you are in a facility that is used to sedation management or in an environment with adequate staffing, such as an ICU with 2:1 nursing care, it is highly feasible. |  |

# Summary of judgment

|  | **Judgment** | | | | | | |
| --- | --- | --- | --- | --- | --- | --- | --- |
| **PROBLEM** | No | Probably no | Probably yes | Yes |  | Varied | Do not know |
| **DESIRABLE EFFECTS** | Trivial | Small | Moderate | Large |  | Varied | Do not know |
| **UNDESIRABLE EFFECTS** | Large | Moderate | Small | Trivial |  | Varied | Do not know |
| **CERTAINTY OF EVIDENCE** | Very low | Low | Moderate | High |  |  | No included study |
| **VALUES** | Important uncertainty or variability | Possibly important uncertainty or variability | Probably no important uncertainty or variability | No important uncertainty or variability |  |  |  |
| **BALANCE OF EFFECTS** | Favors the comparison | Probably favors the comparison | Does not favor either the intervention or the comparison | Probably favors the intervention | Favors the intervention | Varied | Do not know |
| **ACCEPTABILITY** | No | Probably no | Probably yes | Yes |  | Varied | Do not know |
| **FEASIBILITY** | No | Probably no | Probably yes | Yes |  | Varied | Do not know |

# Type of Recommendation

| Strong recommendation against the intervention | Conditional recommendation against the intervention | Conditional recommendation for either the intervention or the comparison | Conditional recommendation for the intervention | Strong recommendation for the intervention |
| --- | --- | --- | --- | --- |
| ○ | ○ | ○ | ● | ○ |

# Conclusions

| Recommendation |
| --- |
| **We suggest using no sedation or light sedation as supportive care in adult patients with ARDS**  **(conditional recommendation/Very low: GRADE 2D).**  **Note: Light sedation refers to sedation that can be awakened by stimulation or calling instead of deep sedation that is unresponsive to stimulation. This recommendation results from a study that excluded patients who require deep sedation and should be considered when implementing this recommendation. There are no studies limited to patients with ARDS, so caution should be exercised when applying this recommendation.** |
|  |
| Justification |
| **Question:** Should non-sedative or light-sedative management be conducted for adult patients with ARDS?  **Patient:**Adult patients with ARDS or patients who received more than 12 hours mechanical ventilation  **Intervention:**No sedation or light sedation  **Comparison:**　Traditional sedation  **Outcome**Survival discharge, length of ICU stay, ventilator-free days, tracheotomy, accidental extubation, VAP  **Summary of evidence**: The estimated values of desirable anticipated effects were as follows: survival discharge yielded an RD of 28 more per 1000 (2 RCTs: N=242) (95% CI: 90 fewer to 172 more); length of ICU stay yielded an MD of 0.26 days shorter (2 RCTs: N=165) (1.02 shorter to 0.51 longer); ventilator-free days yielded an MD of 2 days longer (2 RCTs: N=1287) (0.91 shorter to 4.88 longer). From the above, it was judged that the desirable anticipated effect was small. On the contrary, the estimated values of undesirable anticipated effects were as follows: tracheotomy yielded an RD of 4 fewer per 1000 (3 RCTs: N=1416) (95% CI: 24 fewer to 25 more); accidental extubation yielded an RD of 33 more per 1000 (3 RCTs: N=1416) (2 more to 76 more); VAP yielded an RD of 3 more per 1000 (2 RCTs: N=1287) (32 fewer to 49 more). From the above, it was judged that the undesirable anticipated effect was “small.”  **Certainty of the evidence:** The direction of desirable and undesirable effects was not consistent. Thus, the overall certainty of the evidence was judged to be “very low.”  **Values, balance of effects, acceptability, feasibility**: In terms of values, there is no significant uncertainty or diversity. The predicted desired effects of the intervention are almost the same when considered as point estimates, and the direction of the effect changes when the upper and lower confidence intervals are considered. From the above, we conclude that the balance between benefit and harm is uncertain. However, there is the possibility of up to 172 more surviving discharges per 1,000 patients and reduced costs. Thus, we concluded that the benefits of the intervention probably outweigh the harms. Regarding acceptance, the possibility of increased accidental extubation may increase the nursing burden, but the maximum number of survival discharges would increase by 172 per 1,000, and there is a possibility of reducing costs, so it was judged to be acceptable. As for feasibility, we judged that it would be feasible in an environment with adequate staffing, such as a facility accustomed to sedation management and an ICU with 2:1 nursing care.  **Panel meeting：** In the pre-vote, the median score was 8.0, with a disagreement index of 0.1168, for the “recommended text” according to the modified Delphi method. |

| Subgroup considerations |
| --- |
| Three RCTs examined the effect of light sedation, and one RCT examined the effect of no sedation. The results of the subgroup analysis for each are as follows  Light sedation: Survival discharge, 17 fewer per 1,000; VFD, 1 day longer; tracheostomy, 4 fewer per 1000; accidental extubation, 34 more per 1000; VAP, 5 more per 1000.  No sedation: Survival discharge, 102 more per 1,000; VFD, 4.2 days longer; tracheostomy, 3 fewer per 1000; accidental extubation, 24 more per 1000; VAP, 12 fewer per 1000. |
| Implementation considerations |
| Even in RCTs with no sedation as the intervention, morphine analgesia was given when needed. It is important to note that analgesia is a prerequisite. It is also important to note that none of the four RCTs included in this study focused on only patients with ARDS.  In patients with moderate to severe ARDS, lung-protective ventilation with muscle relaxants and supine management under deep sedation may be used. Therefore, management with no sedation or light sedation may not be possible. In the description of other relevant practice guidelines, Clinical Practice Guidelines for the Prevention and Management of Pain, Agitation/Sedation, Delirium, Immobility, and Sleep Disruption in Adult Patients in the ICU suggests the use of shallow sedation management for critically ill ventilated patients. |

| Monitoring and evaluation |
| --- |
| Collection of further information on the adverse events and cost-effectiveness is necessary because of clinical problems in implementing the recommendations. In addition, it is necessary to monitor the implementation status of the guideline through the use of questionnaires and other means after the guideline is published to see if there are any other clinical problems. |
| Research priorities |
| Not all RCTs employed in this meta-analysis included only patients with ARDS. The evidence is insufficient, and it is hoped that large RCTs will be conducted in patients with ARDS in the future. |

**CQ45 Should restrictive fluid management strategies be implemented for adult patients with ARDS?**

1. Search strategy

MEDLINE via PubMed （Search date: 2020/6/27）

| #1 | "Respiratory Distress Syndrome, Adult"[Mesh] |
| --- | --- |
| #2 | Acute lung injury [mh] |
| #3 | ALI [tiab] OR ARDS [tiab] |
| #4 | "Acute lung injur*" [tiab] OR "acute respiratory distress" [tiab] OR "acute respiratory failure"[tiab] |
| #5 | #1 OR #2 OR #3 OR #4 |
| #6 | “Body Weight” [Mesh] |
| #7 | “Body Weight” [tiab] |
| #8 | “Central Venous Pressure” [Mesh] |
| #9 | “Central Venous Pressure*” [tiab] |
| #10 | “Cardiac Output” [Mesh] |
| #11 | “Cardiac Output” [tiab] |
| #12 | “Stroke Volume” [Mesh] |
| #13 | “Stroke Volume*” [tiab] |
| #14 | “Catheterization, Swan-Ganz” [Mesh] |
| #15 | “Swan Ganz” [tiab] |
| #16 | “Pulmonary Wedge Pressure"[Mesh] |
| #17 | “Pulmonary Wedge Pressure*”[tiab] |
| #18 | “Pulmonary Artery Pressure*” [tiab] |
| #19 | “Hemodynamics” [Mesh] |
| #20 | “hemodynamic*” [tiab] |
| #21 | “Hemodynamic Monitoring” [Mesh] |
| #22 | “Ultrasonography” [Mesh] |
| #23 | “ultrasono*” [tiab] |
| #24 | “ultrasound*” [tiab] |
| #25 | "Echocardiography"[Mesh] |
| #26 | “Echocardiography” [tiab] |
| #27 | “Fluid Therapy” [Mesh] |
| #28 | “Fluid Therap*” [tiab] |
| #29 | “fluid management” [tiab] |
| #30 | “Diuretics” [Mesh] |
| #31 | “Diuretics” [tiab] |
| #32 | “Furosemide” [Mesh] |
| #33 | “Furosemid*” [tiab] |
| #34 | “Acetazolamide” [Mesh] |
| #35 | “Acetazolamid*” [tiab] |
| #36 | “Water-Electrolyte Balance” [Mesh] |
| #37 | “Water-Electrolyte Balance” [tiab] |
| #38 | #6 OR #7 OR #8 OR #9 OR #10 OR #11 OR #12 OR #13 OR #14 OR #15 OR #16 OR #17 OR #18 OR #19 OR #20 OR #21 OR #22 OR #23 OR #24 OR #25 OR #26 OR #27 OR #28 OR #29 OR #30 OR #31 OR #32 OR #33 OR #34 OR #35 OR #36 OR #37 |
| #39 | #5 AND #38 |
| #40 | randomized controlled trial [pt] |
| #41 | controlled clinical trial [pt] |
| #42 | randomized [tiab] |
| #43 | placebo [tiab] |
| #44 | drug therapy [sh] |
| #45 | randomly [tiab] |
| #46 | trial [tiab] |
| #47 | groups [tiab] |
| #48 | #40 OR #41 OR #42 OR #43 OR #44 OR #45 OR #46 OR #47 |
| #49 | animals [mh] NOT humans [mh] |
| #50 | #48 NOT #49 |
| #51 | #39 AND #50 |

CENTRAL（Search date: 2020/6/26）

| #1 | MeSH descriptor: [Respiratory Distress Syndrome, Adult] explode all trees |
| --- | --- |
| #2 | MeSH descriptor: [Acute Lung Injury] explode all trees |
| #3 | ALI:ti,ab OR ARDS:ti,ab |
| #4 | Acute NEXT lung NEXT injur*:ti,ab OR "acute respiratory distress":ti,ab OR "acute respiratory failure":ti,ab |
| #5 | (Severe:ti,ab OR critical*:ti,ab) AND (respiratory:ti,ab OR hypox*:ti,ab) |
| #6 | {OR #1-#5} |
| #7 | [mh "Body Weight"] |
| #8 | "Body Weight":ti,ab |
| #9 | [mh "Central Venous Pressure"] |
| #10 | "Central Venous Pressure":ti,ab |
| #11 | [mh "Cardiac Output"] |
| #12 | "Cardiac Output":ti,ab |
| #13 | [mh "Stroke Volume"] |
| #14 | "Stroke Volume":ti,ab |
| #15 | [mh "Catheterization, Swan-Ganz"] |
| #16 | Swan?Ganz:ti,ab |
| #17 | "Swan Ganz":ti,ab |
| #18 | [mh "pulmonary Wedge Pressure"] |
| #19 | "Pulmonary Wedge Pressure":ti,ab |
| #20 | "Pulmonary Artery Pressure":ti,ab |
| #21 | [mh Hemodynamics] |
| #22 | [mh "Hemodynamic Monitoring"] |
| #23 | hemodynamic*:ti,ab |
| #24 | [mh Ultrasonography] |
| #25 | ultrasound*:ti,ab |
| #26 | ultrasono*:ti,ab |
| #27 | [mh Echocardiography] |
| #28 | Echocardiography:ti,ab |
| #29 | [mh "Fluid Therapy"] |
| #30 | "Fluid Therapy":ti,ab |
| #31 | "fluid management":ti,ab |
| #32 | [mh Diuretics] |
| #33 | Diuretics:ti,ab |
| #34 | [mh Furosemide] |
| #35 | Furosemid*:ti,ab |
| #36 | [mh Acetazolamide] |
| #37 | Acetazolamid*:ti,ab |
| #38 | [mh "Water-Electrolyte Balance"] |
| #39 | "Water-Electrolyte Balance":ti,ab |
| #40 | {OR #7-#39} |
| #41 | #6 AND #40 |

1. Flow diagram

**Identification**

10 Studies included in qualitative synthesis

29 Full-text articles assessed for eligibility

3563 records after duplicates removed

4012 records identified through database searching

4012 records identified through database searching

Medline via PubMed (n=1534)

CENTRAL (n=2284)

Igaku-Chuo-Zasshi (n=194)

0 additional records identified through other sources

9 Studies included in quantitative synthesis (meta-analysis)

19 Full-text articles excluded, with reasons:

・Wrong language (n=5)

・Wrong study design (n=5)

・Wrong population (n=3)

・Wrong intervention (n=6)

Duplicates

n=449

3534 records excluded

**Included**

**Eligibility**

**Screening**

1. Risk of bias

Mortality Ventilator-free days


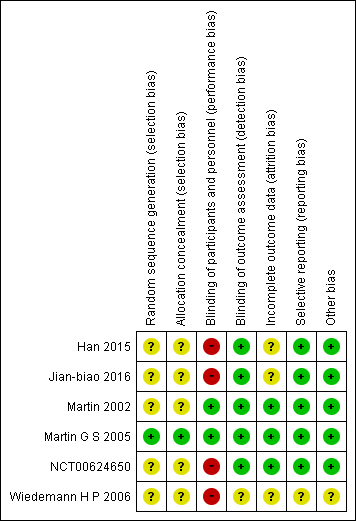

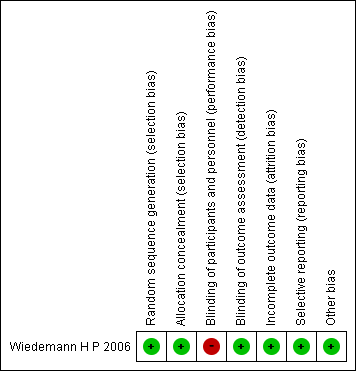


ICU-free days Hemodynamic instability


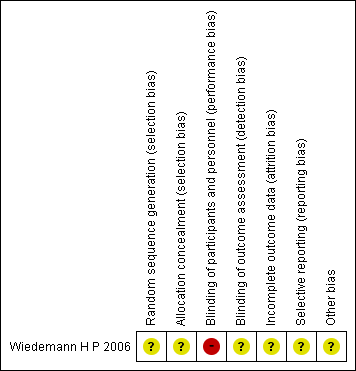
**
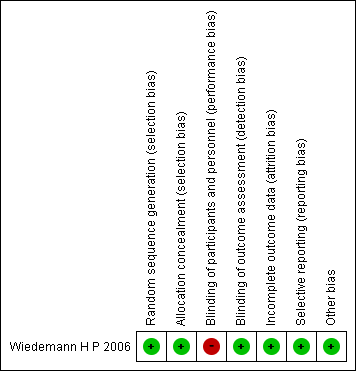
**

Renal replacement therapy Late onset of higher brain dysfunction

**
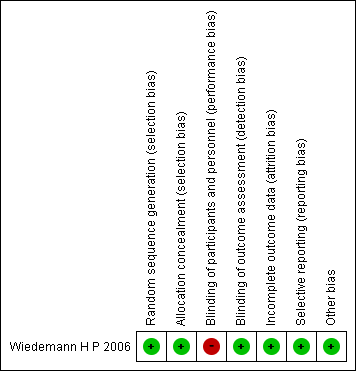

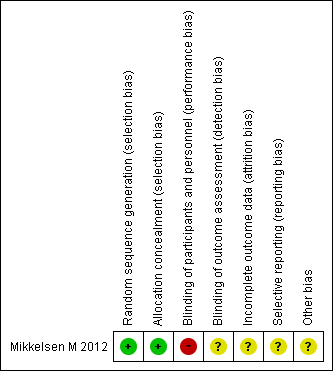
**

Early onset of higher brain dysfunction


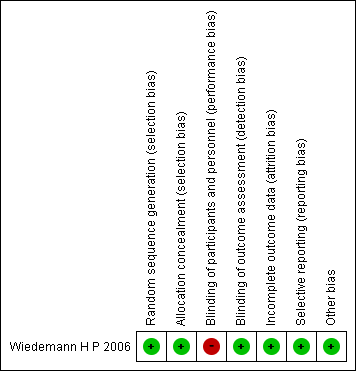


1. Forest plot

Mortality


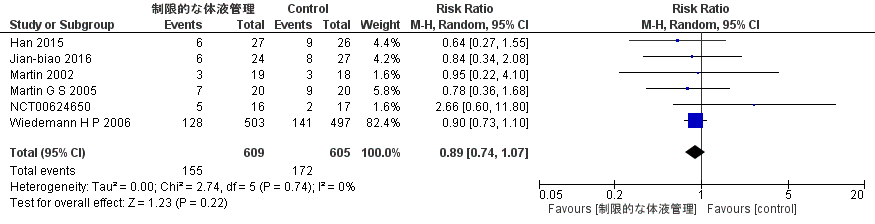


Ventilator-free days


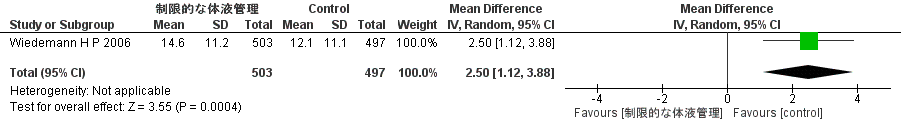


ICU-free days


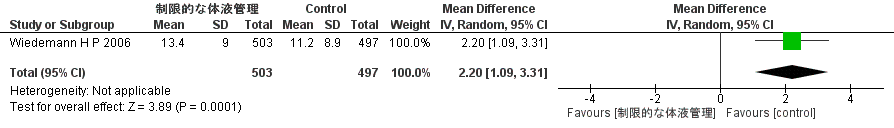


Hemodynamic instability

**
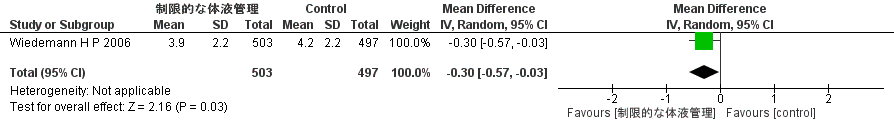
**

Renal replacement therapy

**
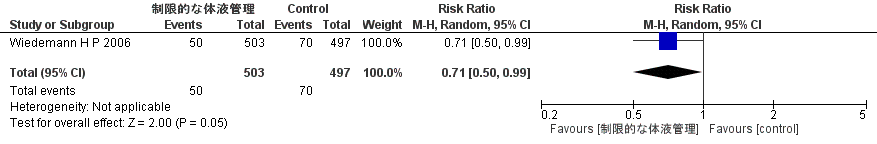
**

Late onset of higher brain dysfunction

**
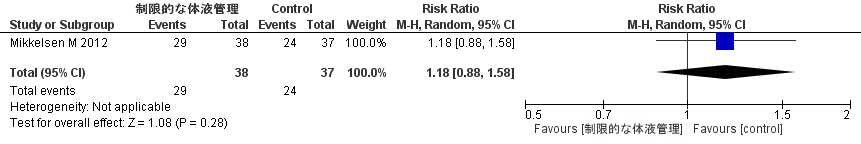
**

Early onset of higher brain dysfunction

**
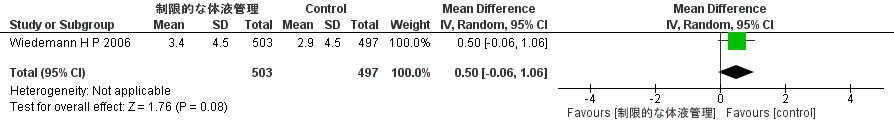
**

1. Evidence profile

| **Assessment of certainty** | | | | | | | | | | | | | **№ of patients** | | | | | **効果** | | | | **Certainty** | | **Importance** |  |  |  |
| --- | --- | --- | --- | --- | --- | --- | --- | --- | --- | --- | --- | --- | --- | --- | --- | --- | --- | --- | --- | --- | --- | --- | --- | --- | --- | --- | --- |
| **№ of studies** | | **Study design** | | **Risk of bias** | | **Inconsistency** | | **Indirectness** | **Imprecision** | | **Others** | | **Restricted fluids management** | | **Others** | | | **Relative index (95% CI)** | | **Absolute index (95% CI)** | |  |  |  |  |  |  |
| Mortality | | | | | | | | | | | | | | | | | | | | | | | | |  |  |  |
| 6 | | RCT | | Not serious | | Not serious | | Not serious | Serious ^a^ | | None | | 155/609 (25.5%) | | 172/605 (28.4%) | | | RR 0.90 (0.74 to 1.08) | | **2**8 fewer per 1000 (74 fewer ～ 23 more) | | ⨁⨁⨁◯ Moderate | | Critical |  |  |  |
| Ventilator-free days | | | | | | | | | | | | | | | | | | | | | | | | |  |  |  |
| 1 | | RCT | | Not serious | | Not serious | | Not serious | Serious ^b^ | | None | | 503 | | 497 | | | - | | MD **2.5 days longer** (1.12 longer ～ 3.88 longer) | | ⨁⨁⨁◯ Moderate | | Critical |  |  |  |
| ICU-free days | | | | | | | | | | | | | | | | | | | | | | | | |  |  |  |
| 1 | | RCT | | Not serious | | Not serious | | Serious ^b^ | Not serious | | None | | 503 | | 497 | | | - | | **MD 2.2days longer** (1.09 longer ～ 3.31 longer) | | ⨁⨁⨁◯ Moderate | | Important |  |  |  |
| Hemodynamic instability (cardiovascular event free days) | | | | | | | | | | | | | | | | | | | | | | | | |  |  |  |
| 1 | | RCT | | Not serious | | Not serious | | Not serious | Serious ^a^ | | None | | 503 | | 497 | | | - | | MD **0.3 days shorter** (0.57 shorter ～ 0.03 shorter) | | ⨁⨁⨁◯ Moderate | | Critical |  |  |  |
| Renal replacement therapy | | | | | | | | | | | | | | | | | | | | | | | | |  |  |  |
| 1 | | RCT | | Not serious | | Not serious | | Not serious | Serious ^b^ | | None | | 50/503 (9.9%) | | 70/497 (14.1%) | | | RR 0.71 (0.50 to 0.99) | | **41 fewer per 1000** (70 fewer ～ 1 fewer) | | ⨁⨁⨁◯ Moderate | | Critical |  |  |  |
| Late onset of higher brain dysfunction | | | | | | | | | | | | | | | | | | | | | | | | |  |  |  |
| 1 | | RCT | | Serious | | Not serious | | Not serious | Serious ^b^ | | None | | 29/38 (76.3%) | | 24/37 (64.9%) | | | RR 1.18 (0.88 to 1.58) | | **117 more per 1000** (78 fewer ～ 376 more) | | ⨁⨁◯◯ Low | | Important |  |  |  |
| Early onset of higher brain dysfunction | | | | | | | | | | | | | | | | | | | | | | | | |  |  |  |
| 1 | | RCT | | Not serious | | Not serious | | Not serious | Serious ^b^ | | None | | 503 | | 497 | | | - | | MD **0.5 days longer** (0.06 shorter ～ 1.06 longer) | | ⨁⨁⨁◯ Moderate | | Important |  |  |  |
| **L**ength of hospital stay | | | | | | | | | | | | | | | | | | | | | | | | | | |  |
| 0 | | - | | - | | - ^a^ | | -- | | | - | | - | | - | | - | Not estimated | | - | | - | | Important | | | |

**CI:** confidence interval; **RR:** risk ratio; **MD:** mean difference; **RCT**: randomized controlled trial

#### Length of hospital stay was not included in the evidence profile because the outcomes are not reported.

#### Explanation

#### a. The 95% confidence interval straddles the threshold for clinical judgment.

#### b. Because the optimal information size (OIS) was not met.

For mortality, the shortest time period reported was used; Jolley SE, 2017 reported death within 1 year; however, this study is post hoc to Wiedemann H P, 2006, and death within 60 days was reported in Wiedemann H P, 2006 and included in this evidence profile. However, this study is post hoc of Wiedemann H P, 2006 and excludes Jolley SE, 2017 because deaths within 60 days were reported in Wiedemann H P, 2006 and included in this evidence profile.

1. Evidence-to-Decision table

| Question | |
| --- | --- |
| CQ45：Should restrictive fluid management strategies be implemented for adult patients with ARDS**?** | |
| **Population:** | Adult patients with ARDS |
| **Intervention:** | Restricted fluids management |
| **Comparison:** | Do not use restricted fluids management |
| **Main outcomes:** | Mortality, ventilator-free days, hemodynamic instability, kidney injury/renal replacement therapy |
| **Setting:** | Emergency room or ICU |
| **Perspective:** | Individual |
| **Background:** | In ARDS, pulmonary edema caused by vascular endothelial damage and increased vascular permeability is a problem, while the direct cause of death in ARDS is not hypoxemia but organ failure other than lung. In other words, it is controversial whether fluid management in ARDS should be directed toward improving pulmonary oxygenation through restrictive fluid management that may lead to impaired organ perfusion (PMID:31060088). Therefore, the clinical question of whether to use restrictive fluid management as supportive care in adult patients with ARDS is of high priority and deserves a systematic review in the development of this guideline. |
| **Conflict of interests:** | None |

# Assessment

| Problem Is the problem a priority? | | |
| --- | --- | --- |
| Judgment | research evidence | ADDITIONAL CONSIDERATIONS |
| ○ No  ○ Probably no  ● Probably yes  ○ Yes  ○ Varies  ○ Do not know | In ARDS, pulmonary edema caused by vascular endothelial damage and increased vascular permeability is a problem, but on the other hand, the direct cause of death in ARDS is not hypoxemia but organ failure other than the lung. In other words, it is controversial whether fluid management in ARDS should be aimed at improving pulmonary oxygenation through restrictive fluid management that may lead to organ perfusion impairment. Therefore, the clinical question of whether to use restrictive fluid management as supportive care in adult patients with ARDS is a high priority and an important clinical issue in this guideline. |  |
| Desirable effects How substantial are the desirable anticipated effects? | | |
| Judgment | research evidence | ADDITIONAL CONSIDERATIONS |
| ○ Trivial  ● Small  ○ Moderate  ○ Large  ○ Varies  ○ Do not know | A meta-analysis was performed using eight randomized controlled trials (RCTs). The estimated values of desirable anticipated effects were as follows: mortality yielded an RD of 28 fewer per 1000 (6 RCTs: N=1214) (95% CI: 74 fewer to 23 more); ventilator-free days yielded an MD of 2.5 days longer (1 RCT: N=1000) (95% CI: 1.12 longer to 3.88 longer). From the above, it was judged that the desirable anticipated effect was “small.” |  |
| Undesirable effects How substantial are the undesirable anticipated effects? | | |
| Judgment | research evidence | ADDITIONAL CONSIDERATIONS |
| ○ Large  ○ Moderate  ○ Small  ● Trivial  ○ Varies  ○ Do not know | The estimated values of the undesirable anticipated effects were as follows: cardiovascular event free days yielded an MD of 0.3 days shorter (1 RCT: N=1000) (95% CI: 0.57 shorter to 0.03 shorter); renal replacement therapy yielded an RD of 41 fewer per 1000 (1 RCT: N=1000) (95% CI: 70 fewer to 1 fewer); late onset brain dysfunction as an important outcome yielded an RD of 117 more per 1000 (1 RCT, certainty of evidence: low). From the above, it was judged that the undesirable anticipated effect was “trivial.” |  |
| Certainty of evidence What is the overall certainty of the evidence of effects? | | |
| Judgment | research evidence | ADDITIONAL CONSIDERATIONS |
| ○ Very low  ○ Low  ● Moderate  ○ High  ○ No included studies | \| **Outcome** \| **Importance** \| **Certainty of the evidence** \| \| --- \| --- \| --- \| \| \| \| Mortality \| Critical \| ⨁⨁⨁◯ \| \| Moderate \| \| Ventilator-free days \| Critical \| ⨁⨁⨁◯ \| \| Moderate \| \| Hemodynamic instability (cardiovascular event free days) \| Critical \| ⨁⨁⨁◯ \| \| Moderate \| \| Renal replacement therapy \| Critical \| ⨁⨁⨁◯ \| \| Moderate \|   **Overall certainty of the evidence:**  The certainty of all outcomes was the same. Thus, we judged it to be “moderate.” |  |
| Values Is there important uncertainty about or variability in how much people value the main outcomes? | | |
| Judgment | research evidence | ADDITIONAL CONSIDERATIONS |
| ○ Important uncertainty or variability  ○ Possibly important uncertainty or variability  ○ Probably no important uncertainty or variability  ● No important uncertainty or variability | There are no data on the values of outcomes in fluid therapy. However, in general, the values for death are high, and the variability is low. |  |
| Balance of effects Does the balance between desirable and undesirable effects favor the intervention or the comparison? | | |
| Judgment | research evidence | ADDITIONAL CONSIDERATIONS |
| ○ Favors the comparison  ○Probably favors the comparison  ○ Does not favor either the intervention or the comparison  ● Probably favors the intervention  ○Favors the intervention  ○ Varies  ○ Do not know | \| Outcome \| Comparison \| Intervention  (restricted fluids management) \| Absolute difference \| Relative effect RR (95% CI) \| \| --- \| --- \| --- \| --- \| --- \| \| Mortality \| 172/605  (28.4%) \| 155/609 (25.5%) \| 28 fewer/1,000  (74 fewer to 23 more) \| 0.90  (0.74 to 1.08) \| \| Ventilator-free days \| － \| － \| MD 2.5 days longer  (1.12 longer to 3.88 longer) \| － \| \| Hemodynamic instability (cardiovascular event free days) \| － \| － \| MD 0.3 days shorter  (0.57 shorter to 0.03 shorter) \| － \| \| Renal replacement therapy \| 70/497  (14.1%) \| 50/503  (9.9%) \| 41 fewer/ 1,000  (70 fewer to 1 fewer) \| 0.71  (0.50 to 0.99) \|   Based on the above, we concluded that the balance between the desirable and undesirable effects of intervention was “probably favors the intervention.” |  |
| Acceptability Is the intervention acceptable to key stakeholders? | | |
| Judgment | research evidence | ADDITIONAL CONSIDERATIONS |
| ○ No  ○ Probably no  ● Probably yes  ○ Yes  ○ Varies  ○ Do not know | The balance of benefits and harms from interventions may possibly outweigh the benefits. However, we cannot deny the possibility that the decision may be divided depending on the method of restricted fluid management. Based on the above, it was judged to be “probably acceptable.” |  |
| Feasibility Is the intervention feasible to implement? | | |
| Judgment | research evidence | ADDITIONAL CONSIDERATIONS |
| ○ No  ○ Probably no  ● Probably yes  ○ Yes  ○ Varies  ○ Do not know | Restrictive fluid management, mainly furosemide and IV fluid restriction, can be implemented by any hospital. However, if albumin or renal replacement therapy is chosen, it may be difficult to implement in some hospitals. |  |

# Summary of judgment

|  | **judgMENT** | | | | | | |
| --- | --- | --- | --- | --- | --- | --- | --- |
| **Problem** | No | Probably no | Probably yes | Yes |  | Varied | Do not know |
| **Desirable Effects** | Trivial | Small | Moderate | Large |  | Varied | Do not know |
| **Undesirable Effects** | Large | Moderate | Small | Trivial |  | Varied | Do not know |
| **Certainty of evidence** | Very low | Low | Moderate | High高 |  |  | No included study |
| **Values** | Important uncertainty or variability | Possibly important uncertainty or variability | Probably no important uncertainty or variability | No important uncertainty or variability |  |  |  |
| **Balance of effects** | Favors the comparison | Probably favors the comparison | Does not favor either the intervention or the comparison | Probably favors the intervention | Favors the intervention | Varied | Do not know |
| **Acceptability** | No | Probably no | Probably yes | Yes |  | Varied | Do not know |
| **Feasibility** | No | Probably no | Probably yes | Yes |  | Varied | Do not know |

# Type of Recommendation

| Strong recommendation against the intervention | Conditional recommendation against the intervention | Conditional recommendation for either the intervention or the comparison | Conditional recommendation for the intervention | Strong recommendation for the intervention |
| --- | --- | --- | --- | --- |
| ○ | ○ | ○ | ● | ○ |

# Conclusions

| Recommendation |
| --- |
| **We suggest using restricted fluids management as supportive care in adult patients with ARDS**  **(weak recommendation/moderate: GRADE 2B).**  **Note: There are multiple methods of restricted fluid management, such as restricted infusion and diuretics. Appropriate choices should be made according to the patient’s condition.** |
|  |
| Justification |
| **Question:** Should restrictive fluid management strategies be implemented for adult patients with ARDS**?**  **Patients:**Adult patients with ARDS  **Intervention:**Restricted fluid management  **Comparison:**　Do not use restricted fluid management  **Outcome**Mortality, ventilator-free days, hemodynamic instability, kidney injury/renal replacement therapy  **Summary of evidence**: The estimated values of desirable anticipated effects were as follows: mortality yielded an RD of 28 fewer per 1000 (6 RCTs: N=1214) (95% CI: 74 fewer to 23 more); ventilator-free days yielded an MD of 2.5 days longer (1 RCT: N=1000) (95% CI: 1.12 longer to 3.88 longer). From the above, it was judged that the desirable anticipated effect was “small”. On the other hand, the estimated values of the undesirable anticipated effects were as follows: cardiovascular event free days yielded an MD of 0.3 days shorter (1 RCT: N=1000) (95% CI: 0.57 shorter to 0.03 shorter); renal replacement therapy yielded an RD of 41 fewer per 1000 (1 RCT: N=1000) (95% CI: 70 fewer to 1 fewer); late onset of brain dysfunction as important outcome yielded an RD of 117 more per 1000 (1 RCT, certainty of evidence: low). From the above, it was judged that the undesirable anticipated effect was “trivial.”  **Certainty of the evidence**: The certainty of all outcomes was the same. Thus, we judged it to be “moderate.”  **Values, balance of effects, acceptability, feasibility**：　In terms of values, the value for death was generally considered to be high, with little variability. We judged that the predicted desired effects of this intervention outweighed the harms. Regarding acceptance, we judged that it would probably be acceptable, as we cannot deny the possibility that judgments may differ depending on the method of restrictive fluid management. The feasibility depends on the method of restrictive fluid management, but restrictive fluid management centered on furosemide and IV fluid restriction can be implemented in any medical institution.  **Panel meeting：**  In a preliminary vote, the modified Delphi method resulted in a median score of 9.0 and a disagreement index of 0.1316 for the “recommended text.”  The panel discussed the certainty of the evidence and the modification of the phrase “fluid management with fluid restriction.”  As a result, the panel finally agreed on the results of the pre-vote without a re-vote being required. |

| Subgroup considerations |
| --- |
| Two RCTs included in this meta-analysis selected renal replacement therapy as the method of choice for restrictive fluid management. These two studies were included only for the outcome of death. Therefore, even if we exclude these two RCTs, deaths will be 25 fewer per 1000. |
| Implementation considerations |
| The choice of fluid management with fluid restriction should be based on the patient’s condition.  For other relevant practice guidelines, the ARDS Guidelines 2016 “suggest fluid-restricted management in adult patients with ARDS” (GRADE 2B). |

| Monitoring and evaluation |
| --- |
| The hemodynamics should be monitored closely. |
| Research priorities |
| There are differences between each RCT that employed a restrictive fluid management approach. In addition, some studies have suggested an impact on late onset higher brain dysfunction. Thus, it is hoped that large RCTs will be conducted in patients with ARDS in the future. |

**CQ46 Should enteral nutrition with high ω3 fatty acid content be given to patients with ARDS?**

1. Search strategy

MEDLINE via PubMed （Search date: 2020/6/4）

| #1 | Respiratory Distress Syndrome, Adult [mh] |
| --- | --- |
| #2 | Acute lung injury [mh] |
| #3 | ALI [tiab] OR ARDS [tiab] |
| #4 | Acute lung injur* [tiab] OR acute respiratory distress [tiab] OR acute respiratory failure[tiab] |
| #5 | (Severe[tiab] OR critical*[tiab]) AND (respiratory[tiab] OR hypox* [tiab]) |
| #6 | “shock lung”[tiab] |
| #7 | #1 OR #2 OR #3 OR #4 OR #5 OR #6 |
| #8 | “omega 3 fatty acids”[tiab] |
| #9 | “n 3 fatty acids” [tiab] |
| #10 | "fatty acids, unsaturated"[mesh] |
| #11 | "unsaturated fatty acids"[tiab] |
| #12 | “linolenic acid”[tiab] |
| #13 | “Fish Oils”[mesh] |
| #14 | “fish oil”[tiab] |
| #15 | #8 OR #9 OR #10 OR #11 OR #12 OR #13 OR #14 |
| #16 | #7 AND #15 |
| #17 | Controlled clinical trial[Publication Type] |
| #18 | randomized[Title/Abstract] |
| #19 | placebo[Title/Abstract] |
| #20 | randomly [tiab] |
| #21 | trial[tiab] |
| #22 | groups [tiab] |
| #23 | "systematic review"[Publication Type] |
| #24 | "meta-analysis"[Publication Type] |
| #25 | "review"[Publication Type] |
| #26 | #17 OR #18 OR #19 OR #20 OR #21 OR #22 OR #23 OR #24 OR #25 |
| #27 | #16 AND #26 |
| #28 | animals [mh] NOT humans [mh] |
| #29 | #27 NOT #28 |

CENTRAL（Search date: 2020/6/4）

| #1 | [mh "Respiratory Distress Syndrome, Adult"] |
| --- | --- |
| #2 | [mh "Acute lung injury"] |
| #3 | ALI:ti,ab OR ARDS:ti,ab |
| #4 | (Acute NEXT lung NEXT injur*:ti,ab) OR "acute respiratory distress":ti,ab OR "acute respiratory failure":ti,ab |
| #5 | (Severe:ti,ab OR critical*:ti,ab) AND (respiratory:ti,ab OR hypox*:ti,ab) |
| #6 | "shock lung":ti,ab |
| #7 | {OR #1-#6} |
| #8 | "omega 3 fatty acids":ti,ab |
| #9 | "n 3 fatty acids":ti,ab |
| #10 | [mh "fatty acids, unsaturated"] |
| #11 | "unsaturated fatty acids":ti,ab |
| #12 | "linolenic acid":ti,ab |
| #13 | [mh "Fish Oils"] |
| #14 | “fish oil”:ti,ab |
| #15 | {OR #8-#14} |
| #16 | #7 AND #15 |
| #17 | [mh animals] NOT [mh humans] |
| #18 | #16 NOT #17 |

1. Flow diagram

**Identification**

11 Studies included in qualitative synthesis

19 Full-text articles assessed for eligibility

520 records after duplicates removed

592 records identified through database searching

592 records identified through database searching

Medline via PubMed (n=410)

CENTRAL (n=138)

Igaku-Chuo-Zasshi (n=44)

0 additional records identified through other sources

9 Studies included in quantitative synthesis (meta-analysis)

8 Full-text articles excluded, with reasons:

・Wrong study design (n=4)

・Wrong population (n=3)

・Wrong intervention (n=1)

Duplicates

n=72

501 records excluded

**Included**

**Eligibility**

**Screening**

1. Risk of bias


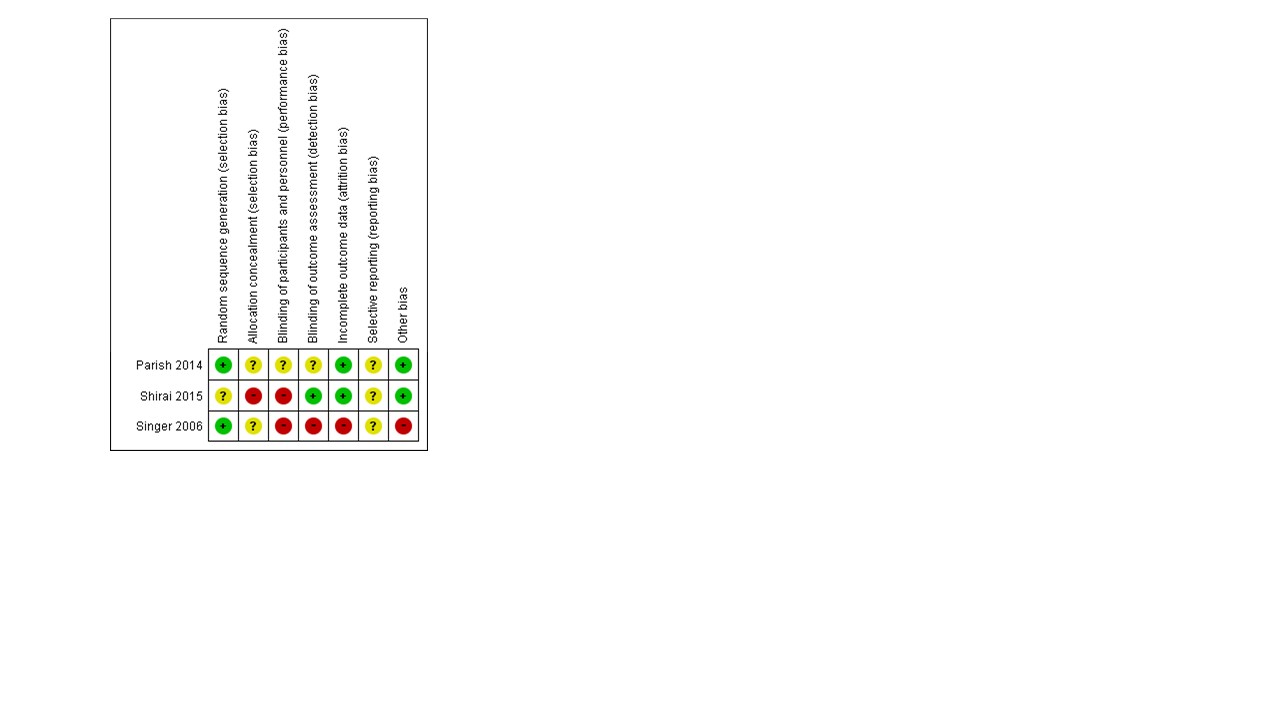

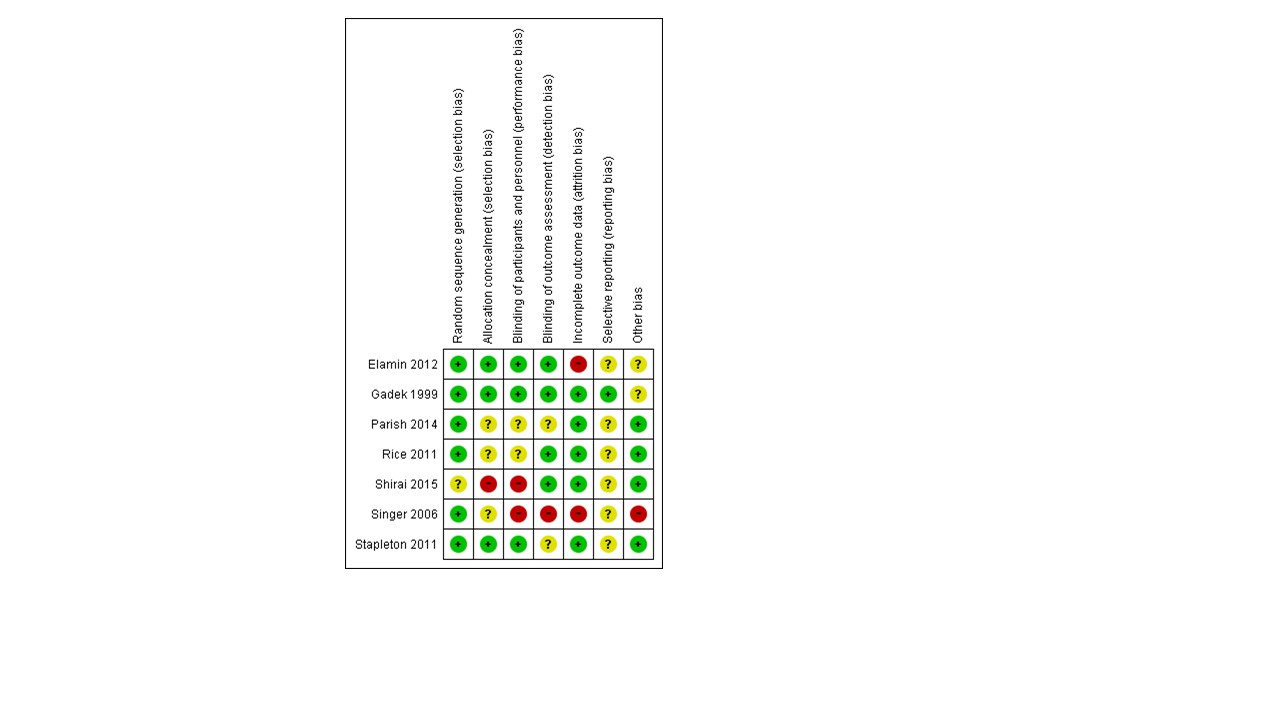
Mortality P/F ratio


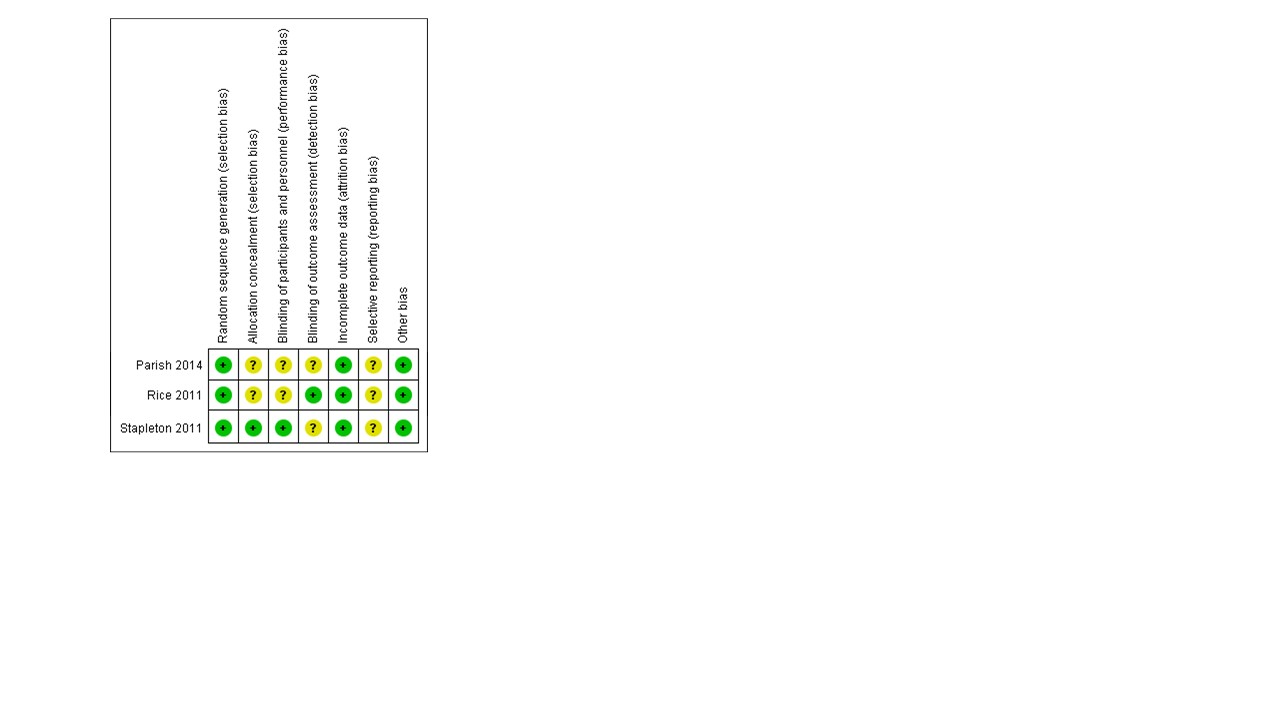

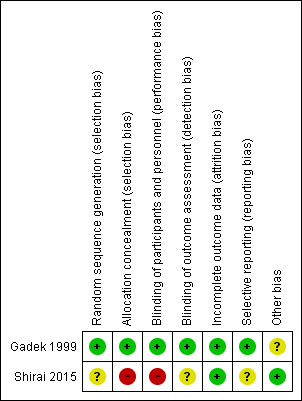
Ventilator-free days Length of ICU stay

Gastrointestinal intolerance Infection


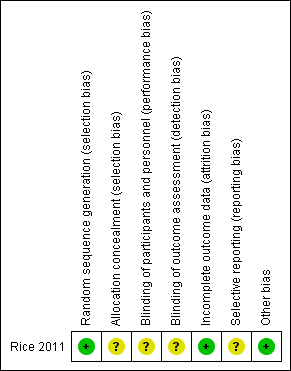

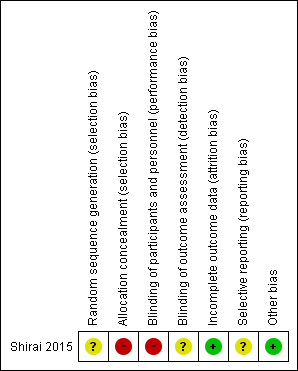


1. Forest plot

Mortality


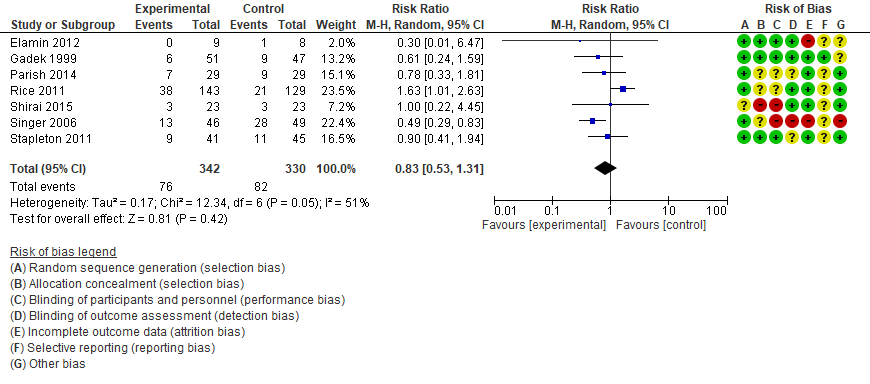


P/F ratio


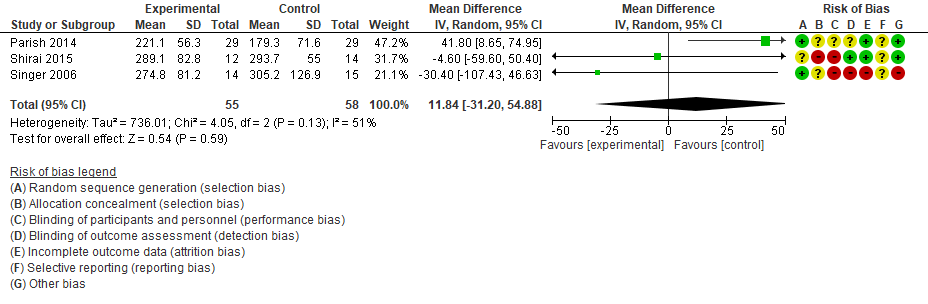


Ventilator-free days


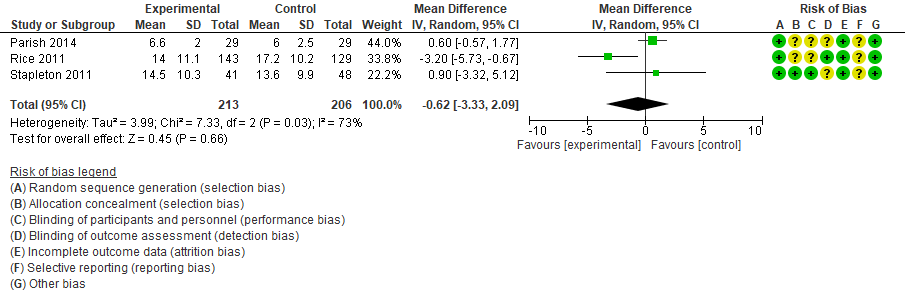


Length of ICU stay


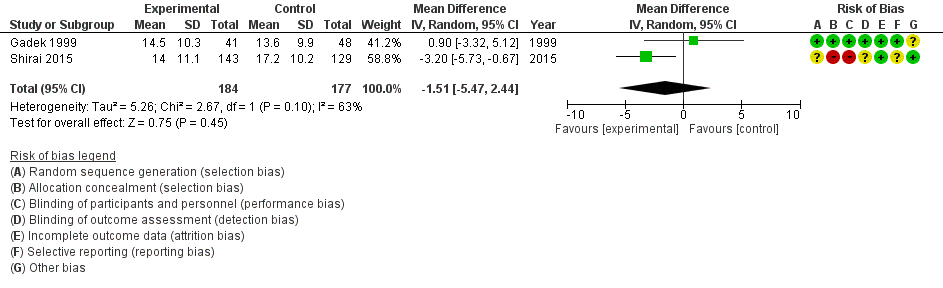


Gastrointestinal intolerance


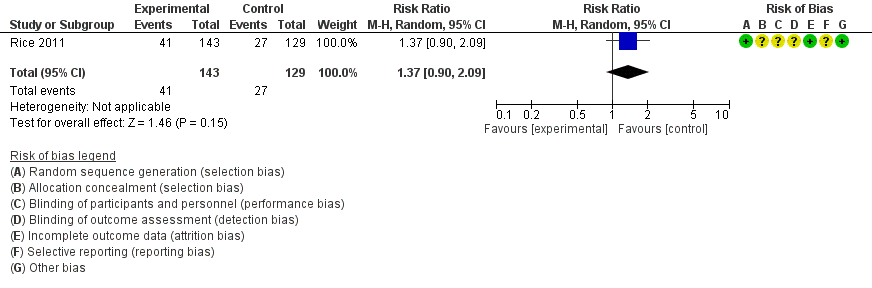


Infection


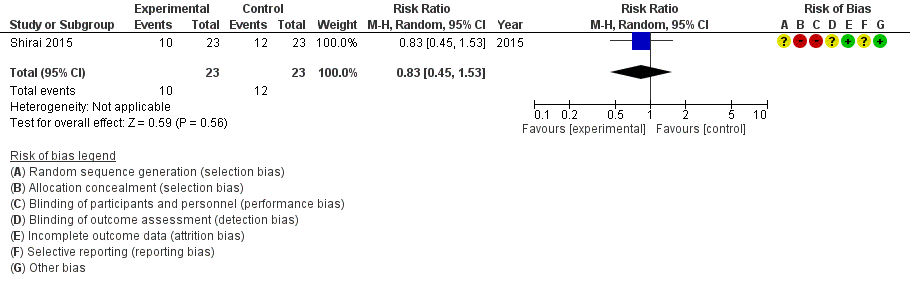


1. Evidence profile

| **Assessment of certainty** | | | | | | | **№ of patients** | | **効果** | | | **Certainty** | | **Importance** |
| --- | --- | --- | --- | --- | --- | --- | --- | --- | --- | --- | --- | --- | --- | --- |
| **№ of studies** | **Study design** | **Risk of bias** | **Inconsistency** | **Indirectness** | **Imprecision** | **Others** | **ω-3 fatty acids** | **placebo** | **Relative index (95% CI)** | **Absolute index (95% CI)** | |  |  |  |
| **Mortality** | | | | | | | | | | | | | | |
| 8 | RCT | Serious ^a^ | Not serious | Serious ^b^ | Serious ^c^ | None | 85/382 (22.3%) | 93/375 (24.8%) | **RR 0.85** (0.58 to 1.24) | **37 fewer per 1,000** (104 fewer to 60 more) | | ⨁◯◯◯ Very low | | Critical |
| **P/F ratio** | | | | | | | | | | | | | | |
| 3 | RCT | Serious ^d^ | Not serious | Serious ^b^ | Serious ^e^ | None | 55 | 58 | - | MD **11.84 higher** (31.2 lower to 54.88 higher) | | ⨁◯◯◯ Very low | | Critical |
| **Ventilator free days** | | | | | | | | | | | | | | |
| 3 | RCT | Serious ^f^ | Not serious | Serious ^b^ | Serious ^e^ | None | 213 | 206 | - | MD **0.62 days shorter** (3.33 shorter to 2.09 longer) | | ⨁◯◯◯ Very low | | Critical |
| **Length of ICU stay** | | | | | | | | | | | | | | |
| 2 | RCT | Not serious | Not serious | Serious ^b^ | Serious ^c^ | None | 74 | 70 | - | MD **5.12 days shorter**  (7.36 shorter to 2.88 shorter) | | ⨁⨁◯◯  Low | | Critical |
| **G**astrointestinal intolerance | | | | | | | | | | | | | | |
| 1 | RCT | Serious ^f^ | Not serious | Serious ^b^ | Serious ^g^ | None | 41/143 (28.7%) | 27/129 (20.9%) | **RR 1.37** (0.90 to 2.09) | **77 more per 1,000** (21 fewer to 228 more) | | ⨁◯◯◯ Very low | | Critical |
| **I**nfection | | | | | | | | | | | | | | |
| 1 | RCT | Serious ^h^ | Not serious | Serious ^b^ | Serious ^g^ | None | 10/23 (43.5%) | 12/23 (52.2%) | **RR 0.83** (0.45 to 1.53) | **89 fewer per 1,000** (287 fewer to 277 more) | | ⨁◯◯◯ Very low | | Critical |
| MODS score | | | | | | | | | | | | | | |
| 0 | - | - | - | - ^a^ | -- | - | - | - | Not estimated | | **-** | | - | Important |
| Heart disease | | | | | | | | | | | | | | |
| 0 | - | - | - | - ^a^ | -- | - | - | - | Not estimated | | **-** | - | | Critical |

**CI:** confidence interval; **RR:** risk ratio; **MD:** mean difference; RCT: randomized controlled trial

#### Multiple organ dysfunction score (MODS) and cardiac disease were not included in the evidence profile because the outcomes were not reported.

#### Explanation

a. Singer et al.'s study with high weight and Elamin et al.'s study with low weight were high risk of bias studies, and many other studies had unclear risk items.

b. We included studies of critically ill patients rather than only patients with acute respiratory distress syndrome.

c. Because the optimal information size (OIS) was not met.

d. Too many blinded and selected outcome reports with high risk and unclear risk ratings.

e. Because the 95% confidence interval crosses the threshold for clinical judgment.

f. The number of unclear risk items.

g. Because the 95% confidence interval crosses the threshold for clinical judgment.

h. Because there are many items for high risk and unclear risk.

1. Evidence-to-Decision table

| Question | |
| --- | --- |
| CQ46： Should enteral nutrition with high ω3 fatty acid content be given to patients with ARDS**?** | |
| **Population:** | Adult patients with ARDS |
| **Intervention:** | Enteral nutrition enriched with omega-3 fatty acids |
| **Comparison:** | Other enteral nutrition |
| **Main outcomes:** | Mortality, P/F ratio, ventilator-free days, length of intensive care unit (ICU) stay, gastrointestinal intolerance, infection, heart disease |
| **Setting:** | Emergency room or ICU |
| **Perspective:** | Individual |
| **Background:** | Fatty acids are broadly classified into unsaturated fatty acids, which have double bonds in their carbon chains, and saturated fatty acids, which do not. Unsaturated fatty acids are further divided into ω3 fatty acids and ω6 fatty acids according to the location of the first double bond. Experiments in animal models of sepsis and septic acute lung injury (ALI) have shown that the administration of fats with a high content of ω3 fatty acids suppresses pulmonary vascular permeability, pulmonary edema, and pulmonary hypertension. It is an important clinical issue to clarify the efficacy of enteral nutrition with high ω3 fatty acid content, such as fish oil, for patients with ARDS by this mechanism. |
| **Conflict of interests:** | None |

# Assessment

| Problem Is the problem a priority? | | |
| --- | --- | --- |
| Judgment | research evidence | ADDITIONAL CONSIDERATIONS |
| ○ No  ○ Probably no  ● Probably yes  ○ Yes  ○ Varies  ○ Do not know | Fatty acids are broadly classified into unsaturated fatty acids, which have double bonds in their carbon chains, and saturated fatty acids, which do not. Unsaturated fatty acids are further divided into ω3 fatty acids and ω6 fatty acids according to the location of the first double bond. Experiments in animal models of sepsis and septic ALI have shown that the administration of fats with a high content of ω3 fatty acids suppresses pulmonary vascular permeability, pulmonary edema, and pulmonary hypertension. It is an important clinical issue to clarify the efficacy of enteral nutrition with high ω3 fatty acid content, such as fish oil, for patients with ARDS by this mechanism. |  |
| Desirable effects How substantial are the desirable anticipated effects? | | |
| Judgment | research evidence | ADDITIONAL CONSIDERATIONS |
| ○ Trivial  ○ Small  ● Moderate  ○ Large  ○ Varies  ○ Do not Know | A meta-analysis was performed using eight randomized controlled trials (RCTs). The estimated values of desirable anticipated effects were as follows: mortality yielded an RD of 37 fewer per 1000 (8 RCTs: N=757) (95% CI: 104 fewer to 60 more); ventilator-free days yielded an MD of 0.62 days shorter (3 RCTs: N=419) (95% CI: 3.33 shorter to 2.09 longer); length of ICU stay yielded an MD of 5.12 days shorter (2 RCTs: N=144) (95% CI: 7.36 shorter to 2.88 shorter); P/F ratio yielded an MD of 11.84 higher (95%CI: 31.2 shorter to 54.88 longer). From the above, it was judged that the desirable anticipated effect was “moderate.” No RCTs reported the MODS score as an outcome. |  |
| Undesirable effects How substantial are the undesirable anticipated effects? | | |
| Judgment | research evidence | ADDITIONAL CONSIDERATIONS |
| ○ Large  ○ Moderate  ● Small  ○ Trivial  ○ Varies  ○ Do not know | The estimated values of undesirable anticipated effects were as follows: gastrointestinal intolerance yielded an RD of 77 more per 1000 (95%CI: 21 fewer to 228 more); infection yielded an RD of 89 fewer per 1000 (95%CI: 287 fewer to 277 more). From the above, it was judged that the undesirable anticipated effect was “small.” No RCTs reported heart disease as an outcome. |  |
| Certainty of evidence What is the overall certainty of the evidence of effects? | | |
| Judgment | research evidence | ADDITIONAL CONSIDERATIONS |
| ● Very low  ○ Low  ○ Moderate  ○ High  ○ No included studies | \| **Outcome** \| **Importance** \| **Certainty of the evidence** \| \| --- \| --- \| --- \| \| \| \| Mortality \| Critical \| ⨁◯◯◯ \| \| Very low \| \| Ventilator-free days \| Critical \| ⨁◯◯◯ \| \| Very low \| \| Length of ICU stay \| Critical \| ⨁⨁◯◯ \| \| Low \| \| P/F ratio \| Critical \| ⨁◯◯◯ \| \| Very low \| \| Gastrointestinal intolerance \| Critical \| ⨁◯◯◯ \| \| Very low \| \| Infection \| Critical \| ⨁◯◯◯ \| \| Very low \| \| Heart disease \| Critical \| - \| \| - \|   No RCTs reported heart disease as an outcome.  **Overall certainty of the evidence**:  The direction of desirable and undesirable effects was not consistent. Thus, the overall certainty of the evidence was judged to be “very low.” |  |
| Values Is there important uncertainty about or variability in how much people value the main outcomes? | | |
| Judgment | research evidence | ADDITIONAL CONSIDERATIONS |
| ○ Important uncertainty or variability  ○ Possibly important uncertainty or variability  ○ Probably no important uncertainty or variability  ● No important uncertainty or variability | There are no data on the values of outcomes in nutrition therapy. However, in general, the values for death are high, and the variability is low. |  |
| Balance of effects Does the balance between desirable and undesirable effects favor the intervention or the comparison? | | |
| Judgment | research evidence | ADDITIONAL CONSIDERATIONS |
| ○ Favors the comparison  ○Probably favors the comparison  ○ Does not favor either the intervention or the comparison  ● Probably favors the intervention  ○Favors the intervention  ○ Varies  ○ Do not know | \| Outcome \| Comparison \| Intervention \| Absolute difference \| Relative effect RR (95% CI) \| \| --- \| --- \| --- \| --- \| --- \| \| Mortality \| 93/375（24.8%) \| 85/382  (22.3%) \| 37 fewer/ 1,000  (104 fewer～60 more) \| 0.85  (0.58～1.24) \| \| Ventilator-free days \| － \| － \| MD 0.62 days shorter  (3.33 shorter～2.09 longer ) \| － \| \| Length of ICU stay \| － \| － \| MD 5.12 days shorter  (7.36 shorter～2.88 shorter) \| － \| \| P/F ratio \| － \| － \| MD11.84 higher  (31.2 lower～54.88 higher) \| － \| \| Gastrointestinal intolerance \| 27/129  (20.9%) \| 41/143  (28.7%) \| 77 more/ 1,000  (21 fewer～228 more) \| 1.37  (0.90～2.09) \| \| Infection \| 12/23  (52.2%) \| 10/23 (43.5%) \| 89 fewer/ 1,000  (287 fewer～277 more) \| 0.83  (0.45～1.53) \| \| Heart disease \| － \| － \| No estimate \| － \|   No RCTs reported heart disease as an outcome.  Based on the above, we concluded that the balance between the desirable and undesirable effects of intervention was “probably favors the intervention.” |  |
| Acceptability Is the intervention acceptable to key stakeholders? | | |
| Judgment | research evidence | ADDITIONAL CONSIDERATIONS |
| ○ No  ○ Probably no  ○ Probably yes  ○ Yes  ● Varies  ○ Do not know | Although the benefits of the intervention may outweigh the costs, the additional administration of ω3 fatty acid preparations with water flush of the tube every 4 h increases the workload of the stakeholders involved in the actual management. Therefore, it was decided that acceptance was not a general rule. |  |
| Feasibility Is the intervention feasible to implement? | | |
| Judgment | research evidence | ADDITIONAL CONSIDERATIONS |
| ○ No  ○ Probably no  ○ Probably yes  ○ Yes  ● Varies  ○ Do not know | Enteral nutrition itself is a common treatment. General enteral nutrition products are readily available, but hospitals that do not use ω3 fatty acid products must adopt new ones. In addition, the additional administration of ω3 fatty acid preparations requires multiple doses per day, and considering the time and effort required, the feasibility of this approach was not entirely clear. |  |

# Summary of Judgment

|  | **Judgment** | | | | | | |
| --- | --- | --- | --- | --- | --- | --- | --- |
| **PROBLEM** | No | Probably no | Probably yes | Yes |  | Varies | Do not know |
| **DESIRABLE EFFECTS** | Trivial | Small | Moderate | Large |  | Varies | Do not know |
| **UNDESIRABLE EFFECTS** | Large | Moderate | Small | Trivial |  | Varies | Do not know |
| **CERTAINTY OF EVIDENCE** | Very low | Low | Moderate | High |  |  | No included study |
| **VALUES** | Important uncertainty or variability | Possibly important uncertainty or variability | Probably no important uncertainty or variability | No important uncertainty or variability |  |  |  |
| **BALANCE OF EFFECTS** | Favors the comparison | Probably favors the comparison | Does not favor either the intervention or the comparison | Probably favors the intervention | Favors the intervention | Varies | Do not know |
| **ACCEPTABILITY** | No | Probably no | Probably yes | Yes |  | Varies | Do not know |
| **FEASIBILITY** | No | Probably no | Probably yes | Yes |  | Varies | Do not know |

# Type of Recommendation

| Strong recommendation against the intervention | Conditional recommendation against the intervention | Conditional recommendation for either the intervention or the comparison | Conditional recommendation for the intervention | Strong recommendation for the intervention |
| --- | --- | --- | --- | --- |
| ○ | ○ | ○ | ● | ○ |

# Conclusions

| Recommendation |
| --- |
| **We suggest administering enteral nutrition enriched with omega-3 fatty acids to patients with ARDS (conditional recommendation for the intervention/very low**: **GRADE 2D).**  **Note: Currently, enteral nutrition products with a high content of ω3 fatty acids are no longer being produced, and to provide enteral nutrition with an equivalent high content of ω3 fatty acids, it is necessary to add ω3 fatty acid products to regular enteral nutrition.** |
|  |
| Justification |
| **Question** Should enteral nutrition with high ω3 fatty acid content be given to patients with ARDS**?**  **Patients**Adults patients with ARDS  **Intervention**enteral nutrition enriched with omega-3 fatty acids  **Comparison**　Other enteral nutrition  **Outcome**Mortality, P/F ratio, ventilator-free days, length of ICU stay, gastrointestinal intolerance, infection, heart disease  **Summary of evidence**：　The estimated values of desirable anticipated effects were as follows: mortality yielded an RD of 37 fewer per 1000 (8 RCTs: N=757) (95% CI: 104 fewer to 60 more); ventilator-free days yielded an MD of 0.62 days shorter (3 RCTs: N=419) (95% CI: 3.33 shorter to 2.09 longer); length of ICU stay yielded an MD of 5.12 days shorter (2 RCTs: N=144) (95% CI: 7.36 shorter to 2.88 shorter); P/F ratio yielded an MD of 11.84 higher (95%CI: 31.2 shorter to 54.88 longer). From the above, it was judged that the desirable anticipated effect was “moderate.” On the contrary, the estimated values of the undesirable anticipated effects were as follows: gastrointestinal intolerance yielded an RD of 77 more per 1000 (95%CI: 21 fewer to 228 more); infection yielded an RD of 89 fewer per 1000 (95%CI: 287 fewer to 277 more). From the above, it was judged that the undesirable anticipated effect was “small.”  **Certainty of the evidence**: The direction of the desirable and undesirable effects was not consistent. Thus, the overall certainty of the evidence was judged to be “very low.”  **Values, balance of effects, acceptability, feasibility**：　In general, the values for death are high, and the variability is low. The predicted desirable effects of this intervention are probably significant relative to the predicted undesirable effects, not only when considering the point estimates, but also when considering the upper and lower confidence intervals. Regarding acceptability, it was judged that the benefits of the intervention outweighed the potential to reduce costs, but this could not be generalized because of the increased effort of the intervention to perform the procedure. Regarding feasibility, enteral nutrition itself is a common treatment, and common enteral nutrition products are readily available. However, we judged that feasibility could not be generally stated because of the limited number of hospitals adopting omega-3 fatty acid products.  **Panel meeting：**  In the pre-vote, the median score was 8.0, with a disagreement index of 0.1316, for the “recommended text proposal” according to the modified Delphi method.  At the panel meeting, there was a discussion on the composition of nutritional supplements and the risk of developing gastrointestinal intolerance.  As a result, the panel meeting finally reached a consensus with the result of the pre-vote without a re-vote being required.  However, the panel meeting was held again in response to public comments pointing out that enteral nutrition products with a high content of omega-3 fatty acids should not be sold.  Although the feasibility and acceptance of the proposal needed to be changed to “not generally accepted,” it was judged that it would be possible to provide enteral nutrition with the same content rate by administering additional ω3 fatty acid preparations available on the market. No change was made. |

| Subgroup considerations |
| --- |
| None |
| Implementation considerations |
| The heterogeneity of the studies employed in this guideline is high, and the formulations used in the control groups vary. In addition, a higher content of ω3 fatty acids may increase gastrointestinal intolerance. The Japanese guidelines for nutritional therapy of critically ill patients weakly recommend the use of enteral nutrition fortified with ω3 (n-3) fatty acids, γ-linolenic acid, and antioxidants for patients with ARDS. |

| Monitoring and evaluation |
| --- |
| Collection of more information on the adverse events and cost-effectiveness is necessary because of clinical problems in implementing the recommendations. In addition, it is necessary to monitor the implementation status of the guideline through the use of questionnaires and other means after the guideline is published to see if there are any other clinical problems. |
| Research priorities |
| There were eight RCTs in this meta-analysis, and the total number of patients was 757. It is hoped that larger RCTs will be conducted on patients with ARDS in the future. |
