# Supplementary material for: ARDS Clinical Practice Guideline 2021
Source: J Intensive Care. 2022 Jul 8;10:32. doi: 10.1186/s40560-022-00615-6 (PMC9263056; doi:10.1186/s40560-022-00615-6)
Supplement: Supplementary file 6 — Additional file 6. Contains Modified Preferred Reporting items of Systematic Reviews and Meta-Analyses (PRISMA) flow-chart, risk of bias summary, forest plots, evidence profiles, and evidence to decision table for PCQ1–15 (children) according to the GRADE system [file 40560_2022_615_MOESM6_ESM.docx]

Additional file 6

Modified Preferred Reporting items of Systematic Reviews and Meta-Analyses (PRISMA) flow-chart, risk of bias summary, forest plots, evidence profiles, and evidence to decision table for PCQ1-15 (children) according to the GRADE system

Table of contents

1. PCQ1
   1. Search strategy p.5
   2. Flow diagram p.8
   3. Risk of bias p.9
   4. Forest plot p.11
   5. Evidence Profile p.13
   6. Evidence-to-Decision table p.16
2. PCQ2
   1. Search strategy p.26
   2. Flow diagram p.31
   3. Risk of bias p.32
   4. Forest plot p.32
   5. Evidence Profile p.32
   6. Evidence-to-Decision table p.33
3. PCQ3
   1. Search strategy p.40
   2. Flow diagram p.46
   3. Risk of bias p.47
   4. Forest plot p.47
   5. Evidence Profile p.47
   6. Evidence-to-Decision table p.48
4. PCQ4
   1. Search strategy p.55
   2. Flow diagram p.58
   3. Risk of bias p.59
   4. Forest plot p.59
   5. Evidence Profile p.59
   6. Evidence-to-Decision table p.60
5. PCQ5
   1. Search strategy p.68
   2. Flow diagram p.70
   3. Risk of bias p.71
   4. Forest plot p.72
   5. Evidence Profile p.73
   6. Evidence-to-Decision table p.75
6. PCQ6
   1. Search strategy p.84
   2. Flow diagram p.87
   3. Risk of bias p.88
   4. Forest plot p.90
   5. Evidence Profile p.92
   6. Evidence-to-Decision table p.96
7. PCQ7
   1. Search strategy p.104
   2. Flow diagram p.106
   3. Risk of bias p.107
   4. Forest plot p.109
   5. Evidence Profile p.111
   6. Evidence-to-Decision table p.113
8. PCQ8
   1. Search strategy p.121
   2. Flow diagram p.123
   3. Risk of bias p.124
   4. Forest plot p.124
   5. Evidence Profile p.124
   6. Evidence-to-Decision table p.125
9. PCQ9
   1. Search strategy p.133
   2. Flow diagram p.135
   3. Risk of bias p.136
   4. Forest plot p.136
   5. Evidence Profile p.136
   6. Evidence-to-Decision table p.137
10. PCQ10
    1. Search strategy p.145
    2. Flow diagram p.146
    3. Risk of bias p.147
    4. Forest plot p.149
    5. Evidence Profile p.151
    6. Evidence-to-Decision table p.153
11. PCQ11
    1. Search strategy p.161
    2. Flow diagram p.163
    3. Risk of bias p.164
    4. Forest plot p.165
    5. Evidence Profile p.166
    6. Evidence-to-Decision table p.169
12. PCQ12
    1. Search strategy p.177
    2. Flow diagram p.180
    3. Risk of bias p.181
    4. Forest plot p.183
    5. Evidence Profile p.184
    6. Evidence-to-Decision table p.187
13. PCQ13
    1. Search strategy p.196
    2. Flow diagram p.198
    3. Risk of bias p.199
    4. Forest plot p.201
    5. Evidence Profile p.202
    6. Evidence-to-Decision table p.205
14. PCQ14
    1. Search strategy p.214
    2. Flow diagram p.216
    3. Risk of bias p.217
    4. Forest plot p.219
    5. Evidence Profile p.220
    6. Evidence-to-Decision table p.222
15. PCQ15
    1. Search strategy p.231
    2. Flow diagram p.233
    3. Risk of bias p.234
    4. Forest plot p.236
    5. Evidence Profile p.237
    6. Evidence-to-Decision table p.240

**PCQ1 Should non-invasive respiratory support (NPPV/HFNC) be used for pediatric patients with ARDS?**

1. Search strategy

MEDLINE via PubMed (Search date: 2020/6/15)

| #1 | "Hypoxia"[mh] OR hypox*[tiab] OR "Respiratory Insufficiency"[mh] OR respiratory depression*[tiab] OR respiratory failure*[tiab] OR ventilatory depression*[tiab] OR respiratory insufficienc*[tiab] OR "Dyspnea"[mh] OR dyspnea*[tiab] OR "shortness of breath"[tiab] OR "Respiratory Distress Syndrome, Adult"[mh] OR acute respiratory distress[tiab] OR adult respiratory distress[tiab] OR respiratory distress syndrome*[tiab] OR RDS[tiab] OR ARDS[tiab] OR "Acute Lung Injury"[mh] OR acute lung injur*[tiab] OR ALI[tiab] |
| --- | --- |
| #2 | "Respiratory Distress Syndrome, Newborn"[mh] |
| #3 | #1 or #2 |
| #4 | "Noninvasive Ventilation"[mh] OR noninvasive ventilation*[tiab] OR non invasive ventilation*[tiab] OR NIV[tiab] OR NPPV[tiab] OR NIPPV[tiab] OR noninvasive positive pressure ventilation*[tiab] OR noninvasive mechanical ventilation*[tiab] OR noninvasive pressure support ventilation*[tiab] OR "Continuous Positive Airway Pressure"[mh] OR continuous positive airway pressure*[tiab] OR bilevel positive airway pressure*[tiab] OR biphasic positive airway pressure*[tiab] OR BIPAP[tiab] |
| #5 | "Oxygen Inhalation Therapy"[mh] OR HFNC[tiab] OR HHFNC[tiab] OR HHHFNC[tiab] OR HFNO[tiab] OR HFNT[tiab] OR HFNOT[tiab] OR HFO[tiab] OR HFOT[tiab] OR NHF[tiab] OR NHFC[tiab] OR NHFT[tiab] OR NHFO[tiab] OR NHFOT[tiab] OR high flow therap*[tiab] OR high flow oxygen[tiab] OR nasal high flow[tiab] |
| #6 | #4 or #5 |
| #7 | ("Randomized Controlled Trial"[pt] OR "Controlled Clinical Trial"[pt] OR "Clinical Trials as Topic"[mh] OR randomized[tiab] OR placebo[tiab] OR randomly[tiab] OR trial[tiab] OR groups[tiab]) NOT (Animals [mh] NOT Humans [mh]) |
| #8 | #3 and #6 and #7 |

CENTRAL (Search date: 2020/6/15)

| #1 | [mh Hypoxia] OR hypox*:ti,ab OR [mh "Respiratory Insufficiency"] OR "respiratory depression":ti,ab OR "respiratory failure":ti,ab OR "ventilatory depression":ti,ab OR "respiratory insufficiency":ti,ab OR [mh Dyspnea] OR dyspnea:ti,ab OR "shortness of breath":ti,ab OR [mh "Respiratory Distress Syndrome, Adult"] OR "acute respiratory distress":ti,ab OR "adult respiratory distress":ti,ab OR "respiratory distress syndrome":ti,ab OR RDS:ti,ab OR ARDS:ti,ab OR [mh "Acute Lung Injury"] OR "acute lung injury":ti,ab OR ALI:ti,ab |
| --- | --- |
| #2 | [mh "Respiratory Distress Syndrome, Newborn"] |
| #3 | #1 OR #2 |
| #4 | [mh "Noninvasive Ventilation"] OR "noninvasive ventilation":ti,ab OR "non invasive ventilation":ti,ab OR NIV:ti,ab OR NPPV:ti,ab OR NIPPV:ti,ab OR "noninvasive positive pressure ventilation":ti,ab OR "noninvasive mechanical ventilation":ti,ab OR "noninvasive pressure support ventilation":ti,ab OR [mh "Continuous Positive Airway Pressure"] OR "continuous positive airway pressure":ti,ab OR "bilevel positive airway pressure":ti,ab OR "biphasic positive airway pressure":ti,ab OR BIPAP:ti,ab |
| #5 | [mh "Oxygen Inhalation Therapy"] OR HFNC:ti,ab OR HHFNC:ti,ab OR HHHFNC:ti,ab OR HFNO:ti,ab OR HFNT:ti,ab OR HFNOT:ti,ab OR HFO:ti,ab OR HFOT:ti,ab OR NHF:ti,ab OR NHFC:ti,ab OR NHFT:ti,ab OR NHFO:ti,ab OR NHFOT:ti,ab OR "high flow therapy":ti,ab OR "high flow oxygen":ti,ab OR "nasal high flow":ti,ab |
| #6 | #4 OR #5 |
| #7 | #3 AND #6 |
| #8 | [mh animals] NOT [mh humans] |
| #9 | #7 NOT #8 |

Igaku-Chuo-Zasshi (Search date: 2020/6/15)

| #1 | 酸素欠乏/TH or 酸素欠乏/TA or anoxia/TA or Hypoxia/TA |
| --- | --- |
| #2 | 呼吸窮迫症候群-急性/TH or 急性呼吸窮迫症候群/TA or ARDS/TA |
| #3 | 急性肺損傷/TH or 急性肺損傷/TA or 急性肺障害/TA or 急性肺傷害/TA or ALI/TA |
| #4 | 呼吸不全/TH or 呼吸不全/TA |
| #5 | 呼吸困難/TH or 呼吸困難/TA |
| #6 | 呼吸窮迫症候群-新生児/TH or 新生児呼吸窮迫症候群/TA |
| #7 | #1 or #2 or #3 or #4 or #5 or #6 |
| #8 | 非侵襲的補助換気/TH or 非侵襲的補助換気/TA or NPPV/TA or NIPPV/TA |
| #9 | 持続気道陽圧/TH or 持続気道陽圧/TA or CPAP/TA |
| #10 | 非侵襲的陽圧呼吸/TH or 非侵襲的陽圧呼吸/TA or BIPAP/TA |
| #11 | 酸素吸入療法/TH or 酸素吸入/TA |
| #12 | 酸素療法/TA or ハイフロー/TA or HFNC/TA or NHF/TA or HFO/TA |
| #13 | #8 or #9 or #10 or #11 or #12 |
| #14 | #7 and #13 |
| #15 | (#14) and (PT=会議録除く) |
| #16 | ランダム化比較試験/TH or ランダム化/AL or 無作為化/AL |
| #17 | 比較試験/AL |
| #18 | 臨床試験/TH or 臨床試験/AL |
| #19 | プラセボ/TH or プラセボ/AL |
| #20 | 対照/AL |
| #21 | コントロール/AL |
| #22 | 臨床研究・疫学研究/TH or 臨床研究/AL |
| #23 | #16 or #17 or #18 or #19 or #20 or #21 or #22 |
| #24 | #15 and #23 |

1. Flow diagram

**Identification**

6 Studies included in qualitative synthesis

64 Full-text articles assessed for eligibility

6,482 records after duplicates removed

7,771 records identified through database searching

7,771 records identified through database searching

Medline via PubMed (n=3,403)

CENTRAL (n=3,924)

Igaku-Chuo-Zasshi (n=444)

0 additional records identified through other sources

5 Studies included in quantitative synthesis (meta-analysis)

1 Full-text articles excluded, with reasons:

・Wrong outcome (n=1)

58 Full-text articles excluded, with reasons:

・Wrong language (n=2)

・Wrong study design (n=26)

・Wrong population (n=27)

・Wrong intervention (n=1)

・Wrong outcome (n=2)

Duplicates

n=1,289

6,418 records excluded

**Included**

**Eligibility**

**Screening**

1. Risk of bias

Mortality Ventilator-free days (VFD)

Length of hospital stay Tracheal intubation rate

Pressure ulcer

1. Forest plot

Mortality

Ventilator-free days (VFD)

Length of hospital stay

Tracheal intubation rate

Pressure ulcer

1. Evidence profile

| **Assessment of certainty** | | | | | | | **No. of patients** | | **Efficacy** | | **Certainty of the Evidence** | **Importance** |
| --- | --- | --- | --- | --- | --- | --- | --- | --- | --- | --- | --- | --- |
| **No. of studies** | **Study design** | **Risk of bias** | **Inconsistency** | **Indirectness** | **Imprecision** | **Others** | **NPPV** | **Placebo** | **Relative index** **(95% CI)** | **Absolute index** **(95% CI)** |  |  |
| **Mortality ^a^** | | | | | | | | | | | | |
| 4 | RCT | Serious ^b^ | Serious ^c^ | Serious ^d^ | Very serious ^e^ | None | 67/439 (15.3%) | 46/431 (10.7%) | **RR 1.61** (0.43 to 6.11) | **+65 per 1000 patients** (-61 to +545) | ⨁◯◯◯ Very low | Critical |
| **Developmental prognosis** | | | | | | | | | | | | |
| 0 |  |  |  |  |  |  |  |  | Impossible to estimate |  | - | Critical |
| **Ventilator-free days (VFD)** | | | | | | | | | | | | |
| 1 | RCT | Very serious ^f^ | Not serious | Serious ^d^ | Very serious ^g^ | None | 21 | 21 | - | **MD 6 day shorter** (-13.37 to +1.37) | ⨁◯◯◯ Very low | Critical |
| **Length of hospital stay** | | | | | | | | | | | | |
| 2 | RCT | Very serious ^f^ | Not serious | Serious ^d^ | Very serious ^g^ | None | 46 | 46 | - | **MD** **0.25 day shorter** (-3.86 to +3.37) | ⨁◯◯◯ Very low | Critical |
| **Tracheal intubation rate** | | | | | | | | | | | | |
| 4 | RCT | Very serious ^f^ | Not serious | Serious ^d^ | Very serious ^e^ | None | 21/143 (14.7%) | 29/133 (21.8%) | **RR 0.79** (0.31 to 2.04) | **-46 per 1000 patients** (-150 to +227) | ⨁◯◯◯ Very low | Critical |
| **Adverse events due to delay in invasive respiratory management** | | | | | | | | | | | | |
| 0 |  |  |  |  |  |  |  |  | Impossible to estimate |  |  | Critical |
| **Pressure ulcer** | | | | | | | | | | | | |
| 2 | RCT | Serious ^h^ | Not serious | Serious ^d^ | Very serious ^i^ | None | 5/43 (11.6%) | 0/44 (0.0%) | **RR 11.00** (0.64 to 188.95) | **+112 per 1000 patients** (-4 to +1000) | ⨁◯◯◯ Very low | Important |

**CI:** confidence interval; **RR:** risk ratio; **MD:** mean difference; RCT: randomized controlled trial

#### Explanations

a. Mortality rates are a composite of 30-day deaths, in-hospital deaths, and those with no stated duration.

b. Rated as Serious for multiple Risk of bias (RoB) items (blinded study participants and treatment providers, other biases).

c. Rated as Serious because the direction of the effect is not consistent across the literature and the 95% confidence intervals overlap only slightly.

d. Since the target population included patients who were not ARDS patients but were at a high risk of developing ARDS, the grade of certainty was lowered by one level to account for the possibility of differences in results compared to ARDS patients.

e. The optimal information size (OIS) is not met and the 95% confidence interval includes both clinically meaningful thresholds for benefit and harm.

f. Rated as Very serious for multiple RoB items (blinded study participants and treatment providers, blinded outcome assessors, and other biases).

g. Not meeting OIS, with upper or lower confidence limits exceeding 0.5 standard deviation (SD), rated as “Very serious.”

h. Rated as serious for multiple RoB items (blinded study participants and treatment providers, blinded outcome assessors (Yanes 2008), and other biases (Cam 2002)).

i. OIS is not met and the 95% confidence interval includes both clinically meaningful thresholds for benefit and harm. Additionally, no event has occurred in the target group.

Note: No studies reported outcomes of developmental prognosis or adverse events due to delay in invasive respiratory management.

1. Evidence-to-Decision table

| **QUESTION** | |
| --- | --- |
| **PCQ1：** Should non-invasive respiratory support (NPPV/HFNC) be used for pediatric patients with ARDS? | |
| **POPULATION:** | Pediatric patients (as defined in the article, 20 years old or younger if not specified) with hypoxemia (P/F ≤ 300 or equivalent) |
| **INTERVENTION:** | Noninvasive respiratory support (Noninvasive positive pressure ventilation: NPPV, High flow nasal cannula: HFNC） |
| **COMPARISON:** | Conventional oxygen therapy (COT) |
| **MAIN OUTCOMES:** | Mortality, Developmental prognosis, Ventilator-free days (VFD), Length of hospital stay, Tracheal intubation rate, Adverse events due to delay in invasive respiratory management |
| **SETTINGS:** | Emergency department or intensive care unit (ICU) |
| **PERSPECTIVE:** | Individual |
| **BACKGROUND:** | Noninvasive positive pressure ventilation (NPPV) and high flow nasal cannula (HFNC) are expected to avoid complications from tracheal intubation, although delayed intubation has been reported to increase mortality. NPPV and HFNC may also increase the mortality rate in patients with severe acute respiratory distress syndrome (ARDS). Noninvasive respiratory support is not an established therapy in the respiratory management of pediatric ARDS patients, and clarifying its efficacy is an important clinical issue.  In this CQ, the target population is not limited to patients with ARDS. The rationale is that although the clinical application of noninvasive respiratory support in pediatric patients with ARDS has increased in recent years, the evidence is limited. |
| **CONFLICT OF INTERESTS:** | None |

**ASSESSMENT**

| **Problem**  Is the problem a priority? | | |
| --- | --- | --- |
| **Judgement** | **Research evidence** | **Additional considerations** |
| ○ No  ○ Probably no  ○ Probably yes  ● Yes  ○ Varies  ○ Do not know | NPPV and HFNC are expected to avoid complications from tracheal intubation, although delayed intubation has been reported to increase mortality. NPPV and HFNC may also increase the mortality rate in patients with severe ARDS. In addition, due to tolerance issues in children, noninvasive respiratory support is not an established treatment for respiratory management in pediatric ARDS patients. Therefore, clarifying its efficacy is an important clinical issue with high priority. |  |
| **Desirable Effects**  How substantial are the desirable anticipated effects? | | |
| **Judgement** | **Research evidence** | **Additional considerations** |
| ● Trivial  ○ Small  ○ Moderate  ○ Large  ○ Varies  ○ Do not know | Five randomized controlled trials (RCTs)^1-5^ consistent with PICO were included in the systematic review and used in the meta-analysis. Two of the seven planned outcomes (developmental prognosis and adverse events due to delay in invasive respiratory management) were not reported, and the meta-analysis was conducted for the following five outcomes.  As a beneficial outcome, the estimate of the effect of tracheal intubation (4 RCTs, N=276) was 46 fewer per 1,000 (95% confidence interval [CI]: 150 fewer to 227 more) in the intervention group than that in the control group. The estimate of effect for length of hospital stays (two RCTs, N=92) was the mean difference of 0.25 days shorter (95% CI: 3.86 days shorter to 3.37 longer). Therefore, the predicted desirable effect was deemed "trivial." |  |
| **Undesirable Effects**  How substantial are the undesirable anticipated effects? | | |
| **Judgement** | **Research evidence** | **Additional considerations** |
| ○ Large  ○ Moderate  ● Small  ○ Trivial  ○ Varies  ○ Do not know | As a harmful outcome, the estimate of effect for mortality (4 RCTs, N=870) was 65 more per 1,000 (95% CI: 61–545 more) in the intervention group than that in the control group. The estimate of effect for VFD (1 RCT, N=42) was the mean difference of 6 days shorter (95% CI: 13.37 days shorter to 1.37 days longer). Pressure ulcers occurred in 11.6% of the intervention group and none in the control group. Therefore, the predicted undesirable effect was judged to be "small.” |  |
| **Certainty of evidence**  What is the overall certainty of the evidence of effects? | | |
| **Judgement** | **Research evidence** | **Additional considerations** |
| ● Very low  ○ Low  ○ Moderate  ○ High  ○ No included studies | **The relative importance or values of the main outcomes of interest:**   \| **Outcome** \| **Importance** \| **Certainty of the Evidence**  **(GRADE)** \| \| --- \| --- \| --- \| \| Mortality* \| Critical \| ⨁◯◯◯ \| \| Very low \| \| Developmental prognosis** \| Critical \| - \| \| VFD \| Critical \| ⨁◯◯◯ \| \| Very low \| \| Length of hospital stay \| Critical \| ⨁◯◯◯ \| \| Very low \| \| Tracheal intubation \| Critical \| ⨁◯◯◯ \| \| Very low \| \| Adverse events due to delay in invasive respiratory management** \| Critical \| - \|   *Mortality is a combination of 30-day mortality, in-hospital mortality, and mortality for which the time period is not stated.  **There were no outcome reports on developmental prognosis and adverse events due to delay in invasive respiratory management in the accepted literature.  **Overall certainty of evidence**  This intervention has the desirable effect of decreasing the tracheal intubation rate. However, there are undesirable effects of increasing mortality and shortening VFD. There were no clinically significant differences in terms of the length of hospital stay. Because of the inconsistent direction of the desirable and undesirable effects, the certainty of the evidence was considered "very low.” |  |
| **Values**  Is there important uncertainty about or variability in how much people value the main outcomes? | | |
| **Judgement** | **Research evidence** | **Additional considerations** |
| ○ Important uncertainty or variability  ○ Possibly important uncertainty or variability  ○ Probably no important uncertainty or variability  ● No important uncertainty or variability | “Mortality” is generally a critical outcome, and there is no great uncertainty or diversity of values about this. |  |
| **Balance of effects**  Does the balance between desirable and undesirable effects favor the intervention or the comparison? | | |
| **Judgement** | **Research evidence** | **Additional considerations** |
| ○ Favors the comparison  ● Probably favors the comparison  ○ Does not favor either the intervention or the comparison  ○ Probably favors the intervention  ○ Favors the intervention  ○ Varies  ○ Do not know | **Summary of evidence:**   \| **Outcome** \| **NPPV** \| **COT** \| **Absolute difference**  **(95% CI)** \| **Risk ratio (RR)**  **(95% CI)** \| \| --- \| --- \| --- \| --- \| --- \| \| Mortality \| 67/439  (15.3%) \| 46/431  (10.7%) \| 65 more per 1,000  （61 fewer to 545 more） \| RR 1.61  (0.43 to 6.11) \| \| Developmental prognosis \| - \| - \| - \| - \| \| VFD \| - \| - \| MD 6 day shorter  （13.37 shorter to 1.37 longer） \| - \| \| Length of hospital stay \| - \| - \| MD 0.25 day shorter  （3.86 shorter to 3.37 longer） \| - \| \| Tracheal intubation \| 21/143  (14.7%) \| 29/133  (21.8%) \| 46 fewer per 1,000  （150 fewer to 227 more） \| RR 0.79  (0.31 to 2.04) \| \| Adverse events due to delay in invasive respiratory management \| - \| - \| - \| - \|   As a beneficial outcome, the estimate of the effect of tracheal intubation (4 RCTs, N=276) was 46 fewer per 1,000 (95% CI: 150 fewer to 227 more), and the mean difference in length of hospital stay (2 RCTs, N=92) was 0.25 days shorter (95% CI: 3.86 days shorter to 3.37 longer). Therefore, the predicted desirable effect was deemed "trivial." Conversely, as harmful outcomes, the estimate of effect of mortality (4 RCTs, N=870) was 65 more per 1,000 (95% CI: 61 less to 545 more), and the mean difference of VFD (1 RCT, N=42) was 6 days shorter (95% CI: 13.37 days shorter to 1.37 days longer). Pressure ulcers occurred in 11.6% of the intervention group and none in the control group. Therefore, the predicted undesirable effect was considered “small.”  Given the clinical importance of tracheal intubation rates and mortality, the balance of effects of NPPV was considered to be "probably favors the comparison.” | Chisti et al. compared Bubble continuous positive airway pressure (CPAP), HFNC, and oxygen therapy in an unmonitored setting.^1^ Cam et al. compared oxygen therapy with nasal CPAP in patients with respiratory failure due to dengue fever.^2^ Mark et al. compared oxygen therapy with nasal CPAP during acute respiratory failure or acute exacerbation of chronic respiratory failure in immunocompromised patients.^3^ McCollum et al. compared oxygen therapy with CPAP for pneumonia in high-risk patients in developing countries.^4^ |
| **Acceptability**  Is the intervention acceptable to key stakeholders? | | |
| **Judgement** | **Research evidence** | **Additional considerations** |
| ○ No  ○ Probably no  ○ Probably yes  ○ Yes  ● Varies  ○ Do not know | Since pediatric patients may not be able to accept the interface or positive pressure, acceptability was set as “varies”. |  |
| **Feasibility**  Is the intervention feasible to implement? | | |
| **Judgement** | **Research evidence** | **Additional considerations** |
| ○ No  ○ Probably no  ○ Probably yes  ○ Yes  ● Varies  ○ Do not know | In pediatric intensive care units (ICUs), NPPV is the standard of care and is highly feasible. On the other hand, pediatric interfaces may not be fully available in some ICUs that accommodate both adult and pediatric patients. In addition, the staff needs to be educated on how to apply the interface and how to interpret the graphic monitor to see if the appropriate positive pressure is being applied. Therefore, NPPV is not always feasible. |  |

**Summary of Judgement**

|  | **JUDGMENT** | | | | | | |
| --- | --- | --- | --- | --- | --- | --- | --- |
| **PROBLEM** | No | Probably no | Probably yes | **Yes** |  | Varies | Do not know |
| **DESIRABLE EFFECTS** | **Trivial** | Small | Moderate | Large |  | Varies | Do not know |
| **UNDESIRABLE EFFECTS** | Large | Moderate | **Small** | Trivial |  | Varies | Do not know |
| **CERTAINTY OF EVIDENCE** | **Very low** | Low | Moderate | High |  |  | No included studies |
| **VALUES** | Important uncertainty or variability | Possibly important uncertainty or variability | Probably no important uncertainty of variability | **No important uncertainty of variability** |  |  |  |
| **BALANCE OF EFFECTS** | Favors the comparison | **Probably favors the comparison** | Does not favor either the intervention or the comparison | Probably favors the intervention | Favors the intervention | Varies | Do not know |
| **ACCEPTABILITY** | No | Probably no | Probably yes | Yes |  | **Varies** | Do not know |
| **FEASIBILITY** | No | Probably no | Probably yes | Yes |  | **Varies** | Do not know |

**Type of Recommendation**

| Strong recommendation against the intervention | Conditional recommendation against the intervention | Conditional recommendation for either the intervention or the comparison | Conditional recommendation for the intervention | Strong recommendation for the intervention |
| --- | --- | --- | --- | --- |
| ○ | ● | ○ | ○ | ○ |

**CONCLUSION**

| **Recommendation** |
| --- |
| We suggest against using non-invasive respiratory support (NPPV/HFNC) in pediatric patients with ARDS. (weak recommendation / very low certainty of evidence：GRADE 2D）  Supplementary item:  This recommendation does not reject the use of non-invasive respiratory support in the early stage of pediatric acute respiratory failure in cases other than ARDS. |
|  |
| **Justification** |
| **Question：**　Should non-invasive respiratory support (NPPV/HFNC) be used for pediatric patients with ARDS?  **Population：**　Pediatric patients with hypoxemia (P/F ≤300 or equivalent) (according to the definition in the article, if not stated, under 20 years old)  **Intervention：**　NPPV, HFNC  **Comparison：**　Conventional oxygen therapy  **Outcome：**　Mortality, ventilator-free days (VFD), length of hospital stay, tracheal intubation  **Summary of evidence**：  Five randomized controlled trials (RCTs)^1-5^ consistent with PICO were included in the systematic review and used in the meta-analysis. Two of the seven planned outcomes (developmental prognosis and adverse events due to delay in invasive respiratory management) were not reported, and the meta-analysis was conducted for the following five outcomes.  As a beneficial outcome, the estimate of the effect of tracheal intubation (4 RCTs, N=276) was 46 fewer per 1,000 (95% confidence interval [CI]: 150 fewer to 227 more) in the intervention group than that in the control group. The estimate of effect for length of hospital stays (two RCTs, N=92) was the mean difference of 0.25 days shorter (95% CI: 3.86 days shorter to 3.37 longer). Therefore, the predicted desirable effect was deemed "trivial."  As a harmful outcome, the estimate of effect for mortality (4 RCTs, N=870) was 65 more per 1,000 (95% CI: 61–545 more) in the intervention group than that in the control group. The estimate of effect for VFD (1 RCT, N=42) was the mean difference of 6 days shorter (95% CI: 13.37 days shorter to 1.37 days longer). Pressure ulcers occurred in 11.6% of the intervention group and none in the control group. Therefore, the predicted undesirable effect was judged to be "small.”  **Quality of evidence**：  This intervention has the desirable effect of decreasing the tracheal intubation rate. However, there are undesirable effects of increasing mortality and shortening VFD. There were no clinically significant differences in terms of the length of hospital stay. Because of the inconsistent direction of the desirable and undesirable effects, the certainty of the evidence was considered "very low.”  **Balance of effects, Acceptability, Feasibility：**  A meta-analysis found that NPPV may have desirable effects on hospital length of stay and tracheal intubation rates, but may shorten ventilator-free days (VFD), increase mortality, and may cause pressure ulcers. Given the clinical importance of tracheal intubation rates and mortality, the balance of effects of NPPV was considered to "probably favors the comparison.”　Since pediatric patients may not be able to accept the interface or positive pressure, acceptability was set as “varies”. In pediatric intensive care units (ICUs), NPPV is the standard of care and is highly feasible. On the other hand, pediatric interfaces may not be fully available in some ICUs that accommodate both adult and pediatric patients. In addition, the staff needs to be educated on how to apply the interface and how to interpret the graphic monitor to see if the appropriate positive pressure is being applied. Therefore, NPPV is not always feasible.  **Panel meeting：**  In the pre-vote, the modified Delphi method recommended that noninvasive respiratory support (NPPV/HFNC) should not be used in pediatric patients with ARDS (grade 2D, weak recommendation/certainty of evidence: “very low”) and the supplementary item "There are no studies of noninvasive respiratory support (NPPV/HFNC) that focus only on mild pediatric ARDS. Therefore, it is unclear whether noninvasive respiratory support (NPPV/HFNC) should be used when focusing only on the mild disease group." was agreed upon with a median score of 8.0 and a disagreement index of 0.1316.  At the panel meeting, it was suggested that in the ancillary information, it would be better to mention the group of pediatric patients who could use noninvasive respiratory support. Therefore, the incidental matter was revised to "There are no studies of noninvasive respiratory support (NPPV/HFNC) targeted only to mild pediatric ARDS. This does not preclude non invasive assisted ventilation for patients with acute respiratory failure who are at risk for pediatric ARDS." The second vote was taken as follows: Using the modified Delphi method, a consensus was reached with a median score of 9.0 and a disagreement index of 0.0000.  After the panel meeting, the Clinical practice guideline creation governing committee emphasized that the phrase "patients with acute respiratory failure who are at risk for pediatric ARDS" in the supplementary material was not clear and could be misunderstood as referring to pediatric patients with acute respiratory failure in general, and the supplementary material was revised.  On a third ballot, the modified Delphi method resulted in the following recommendation: "We suggest against using non-invasive respiratory support (NPPV/HFNC) in pediatric patients with ARDS. (weak recommendation / very low certainty of evidence：GRADE 2D） and the Supplementary item “This recommendation does not reject the use of non-invasive respiratory support in the early stage of pediatric acute respiratory failure in cases other than ARDS." were agreed upon with a median score of 8.0 and a disagreement index of 0.1316.  **Additional considerations**：  In recent years, the use of noninvasive respiratory management, especially HFNC, has increased in the management of pediatric respiratory failure, and it is true that there are cases in which invasive respiratory management can be avoided. Therefore, noninvasive respiratory support in the early stages of pediatric acute respiratory failure should not be ruled out. In pediatric patients with ARDS, there is concern that noninvasive respiratory management may delay invasive respiratory management, resulting in increased mortality. |

| **Subgroup considerations** |
| --- |
| None |
| **Implementation considerations** |
| Delayed invasive respiratory management with NPPV in pediatric ARDS patients may worsen their prognosis. If the facility is not proficient in invasive respiratory management, transfer to a facility that is proficient in invasive respiratory management such as PICU or emergency medical service center should be considered. |

| **Monitoring and evaluation** |
| --- |
| In order to implement the recommendations, more information needs to be collected on clinical issues, such as whether the evaluation after the initiation of NPPV has been done appropriately and whether the use of the interface has increased costs. As for HFNC, it needs to be monitored to determine if there is any impact on the hospital's remaining oxygen levels. In addition, the implementation of the HFNC needs to be monitored through questionnaires and other means after the guidelines are published to see if there are any other clinical issues. |
| **Research priorities** |
| The Pediatric Acute Lung Injury Consensus Conference (PALICC) recommends that NPPV be considered for patients at risk for pediatric ARDS, although not for use in severe pediatric ARDS. Therefore, it is not feasible to study the use of NPPV in pediatric patients with moderate-to-severe ARDS. This is also related to the fact that the patients included in this systematic review were immunocompromised, dengue fever patients, and not pediatric ARDS as defined by PALICC. Regarding intervention methods, four studies used Bubble CPAP specifically for pediatric patients, one study included HFNC, and few studies used NPPV-dedicated machines or HFNC. Future studies are needed to evaluate the efficacy of HFNC and NPPV as initial management strategies, especially for mild ARDS. It is also necessary to clarify which is more effective, CPAP or biphasic positive airway pressure as a ventilation method when NPPV is used. In addition, NPPV is used as part of a strategy for early weaning from the ventilator. Therefore, studies on the usefulness of NPPV and HFNC after extubation are warranted to address airway narrowing, which is more likely to occur in children, and assist with ventilation and oxygenation.  In Europe and the United States, the term non-invasive ventilation (NIV) and NPPV are widely used to describe noninvasive respiratory support, including negative pressure ventilation. In this guideline, we use the term NPPV, which is widely used in Japan, although the terminology needs to be standardized in the future. |

Reference

1. Chisti MJ, Salam MA, Smith JH, et al. Bubble continuous positive airway pressure for children with severe pneumonia and hypoxaemia in Bangladesh: an open, randomised controlled trial. Lancet 2015;386:1057-65.
2. Cam BV, Tuan DT, Fonsmark L, et al. Randomized comparison of oxygen mask treatment vs. nasal continuous positive airway pressure in dengue shock syndrome with acute respiratory failure. J Trop Pediatr 2002;48:335-9.
3. Peters MJ, Agbeko R, Davis P, et al. Randomized Study of Early Continuous Positive Airways Pressure in Acute Respiratory Failure in Children With Impaired Immunity (SCARF) ISRCTN82853500. Pediatr Crit Care Med 2018;19:939-48.
4. McCollum ED, Mvalo T, Eckerle M, et al. Bubble continuous positive airway pressure for children with high-risk conditions and severe pneumonia in Malawi: an open label, randomised, controlled trial. Lancet Respir Med 2019;7:964-74.
5. Yanez LJ, Yunge M, Emilfork M, et al. A prospective, randomized, controlled trial of noninvasive ventilation in pediatric acute respiratory failure. Pediatr Crit Care Med 2008; 9: 484-9.

**PCQ2 Should tidal volume be restricted in pediatric patients with ARDS?**

1.Search strategy

MEDLINE via Pubmed (Search date: 2020/7/6)

| #1 | Respiratory Distress Syndrome, Adult[mh] OR ARDS[tiab] OR shock lung[tiab] |
| --- | --- |
| #2 | acute respiratory distress[tiab] OR acute respiratory failure[tiab] |
| #3 | Acute[tiab] AND ((respirat*[tiab] OR ventilat*[tiab] OR pulmon*[tiab]) AND (fail*[tiab] OR depression[tiab])) |
| #4 | Lung injury[mh] OR ALI[tiab] OR Acute lung injur*[tiab] OR Ventilator-Induced Lung Injury[tiab] |
| #5 | Respiratory insufficiency[mh] OR Respiratory insufficiency[tiab] |
| #6 | Acute chest syndrome[mh] OR Acute chest syndrome[tiab] |
| #7 | #1 OR #2 OR #3 OR #4 OR #5 OR #6 |
| #8 | "Tidal volume"[mh] OR (tidal[tiab] AND volum*[tiab]) |
| #9 | ventilation AND (strateg*[tiab] OR pressure*[tiab] OR limited[tiab] OR low[tiab] OR lower[tiab] OR less[tiab] OR differen*[tiab] OR variab*[tiab] OR varying[tiab]) |
| #10 | (lung[tiab] AND protective[tiab] AND ventilat*[tiab] ) OR LPVS[tiab] |
| #11 | Respiration,artificial[mh] OR "Artificial respiration"[tiab] OR "Artificial ventilation"[tiab] |
| #12 | Ventilators, Mechanical[mh] OR "Mechanical ventilation"[tiab] |
| #13 | #8 OR #9 OR #10 OR #11 OR #12 |
| #14 | #7 AND #13 |
| #15 | ((randomized controlled trial[pt] OR controlled clinical trial[pt] OR randomized[tiab] OR placebo[tiab] OR clinical trials as topic[mesh:noexp] OR randomly[tiab] OR trial[ti] NOT (animals[mh] NOT humans [mh]))) |
| #16 | control group*[tiab] |
| #17 | #15 OR #16 |
| #18 | #14 AND #17 |

CENTRAL (Search date: 2020/7/6)

| #1 | [mh "Respiratory Distress Syndrome, Adult"] OR ARDS:ti,ab OR "shock lung":ti,ab |
| --- | --- |
| #2 | "acute respiratory distress":ti,ab OR "acute respiratory failure":ti,ab |
| #3 | Acute:ti,ab AND ((respirat*:ti,ab OR ventilat*:ti,ab OR pulmon*:ti,ab) AND (fail*:ti,ab OR depression:ti,ab)) |
| #4 | [mh "Lung injury"] OR ALI:ti,ab OR "Acute lung injury":ti,ab OR " Ventilator-Induced Lung Injury":ti,ab |
| #5 | [mh "Respiratory insufficiency"] OR "Respiratory insufficiency":ti,ab |
| #6 | [mh "Acute chest syndrome"] OR "Acute chest syndrome":ti,ab |
| #7 | {OR #1-#6} |
| #8 | [mh "Tidal volume"] OR (tidal:ti,ab AND volume:ti,ab) |
| #9 | ventilation AND (strategy:ti,ab OR pressure:ti,ab OR limited:ti,ab OR low:ti,ab OR lower:ti,ab OR less:ti,ab OR different:ti,ab OR variable:ti,ab OR varying:ti,ab) |
| #10 | (lung:ti,ab AND protective:ti,ab AND ventilatory:ti,ab) OR LPVS:ti,ab |
| #11 | [mh "Respiration, Artificial"] |
| #12 | [mh "Ventilators, Mechanical"] OR "Mechanical ventilation":ti,ab |
| #13 | {OR #8-#12} |
| #14 | #7 AND #13 |
| #15 | [mh animals] NOT [mh humans] |
| #16 | #14 NOT #15 |

EMBASE (Search date: 2020/6/28)

| S1 | (EMB.EXACT("adult respiratory distress syndrome")) OR (TI,AB(ARDS OR "shock lung")) |
| --- | --- |
| S2 | (TI,AB("acute respiratory" p/0 (distress OR failure*))) |
| S3 | (TI,AB(acute n/3 (respirat* OR ventilat* OR pulmon*) n/3 (fail* OR depression))) |
| S4 | ((EMB.EXACT("acute lung injury")) OR (EMB.EXACT("hyperoxia-induced lung injury") OR EMB.EXACT("lung injury")) OR (EMB.EXACT("ventilator induced lung injury")) OR (TI,AB(ALI OR ("acute lung" p/0 injur*) OR "ventilator-Induced lung injury"))) |
| S5 | ((EMB.EXACT.EXPLODE("respiratory failure")) OR (TI,AB(respiratory p/0 insufficien*))) |
| S6 | ((EMB.EXACT("acute chest syndrome")) OR (TI,AB("acute chest syndrome"))) |
| S7 | (S1 OR S2 OR S3 OR S4 OR S5 OR S6) |
| S8 | (EMB.EXACT("tidal volume") OR TI,AB(tidal n/2 volum*)) |
| S9 | (TI,AB(ventilation AND (strateg* OR pressure* OR limited OR low OR lower OR less OR differen* OR variab* OR varying))) |
| S10 | (TI,AB(("lung protective" n/2 ventilat*) OR LPVS)) |
| S11 | ((EMB.EXACT.EXPLODE("artificial ventilation")) OR (TI,AB(artificial p/0 (respiration* OR ventilation*)) OR (TI,AB(mechanical p/0 ventilation*)))) |
| S12 | (S8 OR S9 OR S10 OR S11) |
| S13 | (S7 AND S12) |
| S14 | ((((EMB.EXACT("controlled clinical trial") OR EMB.EXACT.EXPLODE("clinical trial (topic)") OR EMB.EXACT("randomized controlled trial")) OR (TI,AB(randomized) OR TI,AB(randomly) OR TI(trial) OR TI,AB(control p/0 group*))) NOT (ANIMAL(YES) NOT HUMAN(YES)))) |
| S15 | (S13 AND S14) |
| S16 | (S15 AND UD(>=2013)) |
| S17 | (S15 AND UD(<2013)) |

CHINAL (Search date: 2020/7/12)

| #1 | (MH "Respiratory Distress Syndrome, Adult") OR TI ARDS OR AB ARDS OR TI "shock lung" OR AB "shock lung" |
| --- | --- |
| #2 | TI "acute respiratory distress" OR AB "acute respiratory distress" OR TI "acute respiratory failure" OR AB "acute respiratory failure" |
| #3 | TI Acute OR AB Acute AND ((TI respirat* OR AB respirat* OR TI ventilat* OR AB ventilat* OR TI pulmon* OR AB pulmon*) AND (TI fail* OR AB fail* OR TI depression OR AB depression)) |
| #4 | (MH "Lung injury+") OR TI ALI OR AB ALI OR TI "Acute lung injur*" OR AB "Acute lung injur*" OR TI "Ventilator-Induced Lung Injury" OR AB "Ventilator-Induced Lung Injury" |
| #5 | (MH "Respiratory Failure+")  OR TI "Respiratory Failure" OR AB "Respiratory Failure" |
| #6 | (MH "Acute chest syndrome") OR TI "Acute chest syndrome" OR AB "Acute chest syndrome" |
| #7 | S1OR S2 OR S3 OR S4 OR S5 OR S6 |
| #8 | (MH "Tidal volume") OR (TI tidal OR AB tidal AND TI volum* OR AB volum*) |
| #9 | ventilation AND (TI strateg* OR AB strateg* OR TI pressure* OR AB pressure* OR TI limited OR AB limited OR TI low OR AB low OR TI lower OR AB lower OR TI less OR AB less OR TI differen* OR AB differen* OR TI variab* OR AB variab* OR TI varying OR AB varying) |
| #10 | (TI lung OR AB lung AND TI protective OR AB protective AND TI ventilat* OR AB ventilat*) OR TI LPVS OR AB LPVS |
| #11 | (MH "Respiration, Artificial+") OR TI "Artificial respiration" OR AB "Artificial respiration" OR TI "Artificial ventilation" OR AB "Artificial ventilation" |
| #12 | (MH "Ventilators, Mechanical") OR TI “Mechanical Ventilat*” OR AB “Mechanical Ventilat*” |
| #13 | #8 OR #9 OR #10 OR #11 OR #12 |
| #14 | #7 AND #13 |

Igaku-Chuo-Zasshi (Search date: 2020/7/12)

| #1 | 呼吸窮迫症候群-急性/TH or 急性呼吸促迫症候群/AL or ARDS/AL or “acute respiratory distress syndrome”/AL or ショック肺/AL or “shock lung”/AL |
| --- | --- |
| #2 | 肺損傷/TH or 急性肺損傷/AL or “acute lung Injury”/AL or 人工呼吸器誘発肺損傷/AL |
| #3 | 呼吸不全/TH or 呼吸不全/AL or 呼吸機能不全/AL |
| #4 | 呼吸抑制/TH or 呼吸抑制/AL |
| #5 | #1 or #2 or #3 or #4 |
| #6 | 一回換気量/TH or 一回換気量/AL or 低容量換気/AL or “tidal volume”/AL |
| #7 | (肺/TH or 肺/AL or lung/AL) and (保護/AL or protective/AL) |
| #8 | 制限/AL or 低/AL or 保護/AL or 圧/AL |
| #9 | 戦略/AL or strategy/AL |
| #10 | #7 or #8 or #9 |
| #11 | #10 and ([換気(環境)]/TH or 換気/Al or ventilation/AL) |
| #12 | 人工呼吸/TH or 人工呼吸/AL or レスピレータ/AL or ベンチレータ/AL or 機械的換気/AL or 人工換気/AL or 調節呼吸/AL |
| #13 | LPVS/AL |
| #14 | #6 OR #11 OR #12 OR #13 |
| #15 | #5 and #14 |
| #16 | ランダム化比較試験/TH or ランダム化/AL or 無作為化/AL |
| #17 | 比較試験/AL |
| #18 | 臨床試験/TH or 臨床試験/AL |
| #19 | プラセボ/TH or プラセボ/AL |
| #20 | 対照/AL |
| #21 | コントロール/AL |
| #22 | 臨床研究・疫学研究/TH or 臨床研究/AL |
| #23 | #16 or #17 or #18 or #19 or #20 or #21 or #22 |
| #24 | #15 AND #23 |
| #25 | (#24) and (PT=会議録除く) |

1. Flow diagram

196 additional records identified through other sources

ICTRP (n=54)

Clinical Trials gov (n=142)

**Identification**

19,864 records identified through database searching

Medline via PubMed (n=4,607)

CENTRAL (n=4,370)

EMBASE (n=6,488)

CHINAL (n=3,668)

Igaku-Chuo-Zasshi (n=731)

0 Studies included in qualitative synthesis

59 Full-text articles assessed for eligibility

13,494 records after duplicates removed

20,060 records identified through database searching

0 Studies included in quantitative synthesis (meta-analysis)

Duplicates

n=6,566

13,435 records excluded

**Included**

**Eligibility**

**Screening**

59 Full-text articles excluded, with reasons:

・Wrong language (n=4)

・Wrong study design (n=11)

・Wrong publication type (n=8)

・Wrong population (n=36)

1. Risk of bias

Not applicable

1. Forest plot

Not applicable

1. Evidence profile

Not applicable

1. Evidence-to-Decision table

| QUESTION | |
| --- | --- |
| **PCQ2：** Should tidal volume be restricted in pediatric patients with ARDS? | |
| **POPULATION:** | Pediatric patients (as defined in the article, 20 years old or younger if not specified) on ventilators for ARDS (as defined in the article) |
| **INTERVENTION:** | Restrict tidal volume (as defined in the article) |
| **COMPARISON:** | Do not restrict tidal volume (as defined in the article) |
| **MAIN OUTCOMES:** | Mortality, Developmental prognosis, Long-term respiratory function, Ventilator-free days (VFD), Length of hospital stay, Hemodynamic deterioration due to respiratory acidosis |
| **SETTINGS:** | Emergency department or intensive care unit (ICU) |
| **PERSPECTIVE:** | Individual |
| **BACKGROUND:** | For patients with severe ARDS, ventilator management can buy time for recovery, although mortality is still high. Some studies have suggested that mechanical ventilation itself may cause lung injury. In this context, one of the lung protective strategies to reduce adverse events caused by mechanical ventilation is to restrict the amount of tidal volume. However, its effectiveness has been inconsistent in large randomized controlled trials (RCTs) in adults. Systematic reviews and meta-analyses are needed to examine the efficacy and safety of tidal volume restrictions in pediatric patients with ARDS. |
| **CONFLICT OF INTERESTS:** | None |

# ASSESSMENT

| Problem Is the problem a priority? | | |
| --- | --- | --- |
| Judgement | Research evidence | Additional considerations |
| ○ No  ○ Probably no  ○ Probably yes  ● Yes  ○ Varies  ○ Do not know | For patients with severe ARDS, ventilator management can buy time for recovery, although mortality is still high. Some studies have suggested that mechanical ventilation itself may cause lung injury. In this context, one of the lung protective strategies to reduce adverse events caused by mechanical ventilation is to restrict the amount of tidal volume. However, its effectiveness has been inconsistent in large RCTs in adults. Systematic reviews and meta-analyses are needed to examine the efficacy and safety of tidal volume restrictions in pediatric patients with ARDS. Therefore, this issue is of high priority. |  |
| Desirable Effects How substantial are the desirable anticipated effects? | | |
| Judgement | Research evidence | Additional considerations |
| ○ Trivial  ○ Small  ○ Moderate  ○ Large  ○ Varies  ● Do not know | Integrated into the “certainty of evidence”. |  |
| Undesirable Effects How substantial are the undesirable anticipated effects? | | |
| Judgement | Research evidence | Additional considerations |
| ○ Large  ○ Moderate  ○ Small  ○ Trivial  ○ Varies  ● Do not know | Integrated into the “certainty of evidence”. |  |
| Certainty of evidence What is the overall certainty of the evidence of effects? | | |
| Judgement | Research evidence | Additional considerations |
| ○ Very low  ○ Low  ○ Moderate  ○ High  ● No included studies | Strain is an important factor in ventilator-associated lung injury. Restricting tidal volume to prevent hyperextension of alveoli may relieve strain and reduce lung injury. Conversely, restricting tidal volume may have adverse effects, such as hypercapnia. However, since there have been no applicable RCTs in pediatric ARDS patients undergoing ventilatory management, the certainty of the evidence cannot be described.  Strain: Defined by how much the lung is structurally deformed as a result of an external force applied from the resting position. |  |
| Values Is there important uncertainty about or variability in how much people value the main outcomes? | | |
| Judgement | Research evidence | Additional considerations |
| ○ Important uncertainty or variability  ○ Possibly important uncertainty or variability  ○ Probably no important uncertainty or variability  ● No important uncertainty or variability | “Mortality” is generally a critical outcome, and there is no great uncertainty or diversity of values about this. |  |
| Balance of effects Does the balance between desirable and undesirable effects favor the intervention or the comparison? | | |
| Judgement | Research evidence | Additional considerations |
| ○ Favors the comparison  ○ Probably favors the comparison  ○ Does not favor either the intervention or the comparison  ○ Probably favors the intervention  ○ Favors the intervention  ○ Varies  ● Do not know | No relevant studies. |  |
| Acceptability Is the intervention acceptable to key stakeholders? | | |
| Judgement | Research evidence | Additional considerations |
| ○ No  ○ Probably no  ● Probably yes  ○ Yes  ○ Varies  ○ Do not know | All that needs to be done is to change the ventilator settings, which will probably be acceptable to the patient and family, even considering the disadvantages. |  |
| Feasibility Is the intervention feasible to implement? | | |
| Judgement | Research evidence | Additional considerations |
| ○ No  ○ Probably no  ○ Probably yes  ● Yes  ○ Varies  ○ Do not know | All that needs to be done is to change the ventilator settings, which is considered feasible. |  |

# Summary of Judgement

|  | **JUDGEMENT** | | | | | | |
| --- | --- | --- | --- | --- | --- | --- | --- |
| **PROBLEM** | No | Probably no | Probably yes | **Yes** |  | Varies | Do not know |
| **DESIRABLE EFFECTS** | Trivial | Small | Moderate | Large |  | Varies | **Do not know** |
| **UNDESIRABLE EFFECTS** | Large | Moderate | Small | Trivial |  | Varies | **Do not know** |
| **CERTAINTY OF EVIDENCE** | Very low | Low | Moderate | High |  |  | **No included studies** |
| **VALUES** | Important uncertainty of variability | Possibly important uncertainty of variability | Probably No important uncertainty of variability | **No important uncertainty of variability** |  |  |  |
| **BALANCE OF EFFECTS** | Favors the comparison | Probably favors the comparison | Does not favor either the intervention or the comparison | Probably favors the intervention | Favors the intervention | Varies | **Do not know** |
| **ACCEPTABILITY** | No | Probably no | **Probably yes** | Yes |  | Varies | Do not know |
| **FEASIBILITY** | No | Probably no | Probably yes | **Yes** |  | Varies | Do not know |

# Type of Recommendation

| Strong recommendation against the intervention | Conditional recommendation against the intervention | Conditional recommendation for either the intervention or the comparison | Conditional recommendation for the intervention | Strong recommendation for the intervention |
| --- | --- | --- | --- | --- |
| ○ | ○ | ○ | ○ | ○ |

# CONCLUSION

| Recommendation |
| --- |
| We cannot provide a recommendation regarding tidal volume restrictions for pediatric patients with ARDS. It is generally accepted not to exceed tidal volume in accordance with treatment strategy of adult patients with ARDS (in our practice statement).  Supplementary item:  Currently, it is unclear whether to use standard weight or actual weight for the tidal volume per body weight. There is a reference physiological tidal volume value of less than 8 mL/kg. |
|  |
| Justification |
| **Question：**　Should tidal volume be restricted in pediatric patients with ARDS?  **Population：**　Pediatric patients (as defined in the article, 20 years old or younger if not specified) on ventilators for ARDS (as defined in the article)  **Intervention：** Restrict tidal volume (as defined in the article)  **Comparison：** Do not restrict tidal volume (as defined in the article)  **Explanation：**  The restriction of tidal volume in ARDS has become a lung protective strategy. In this guideline for adults, a meta-analysis of 7 RCTs found similar levels of pressure injury between the intervention and control groups, 11 RCTs showed significant reductions in short- and long-term mortality, and four RCTs showed a trend toward increased VFD in the intervention group. Therefore, tidal volume restrictions are recommended in adults (GRADE 1D, strong recommendation/certainty of evidence "very low"). It is also weakly recommended in the lung protective strategy of the Japanese version of the "Surviving Sepsis Campaign Guideline 2020" (J-SSCG 2020) (GRADE 2B, certainty of evidence "moderate"). Similarly, the PALICC (2015) weakly recommends physiological tidal volume (5-8 mL/kg) at the standard weight for age, and lower tidal volume (3-6 mL/kg) for severe ARDS. In addition, the PEMVECC (2017) strongly recommends a tidal volume of less than 10 mL/kg, not exceeding the physiologic tidal volume.  Although all of these are based on the "baby lung concept” of avoiding over-expansion of healthy lungs in adults, some clinicians are cautious about applying the same concepts to children. This is because the anatomy and physiology of a developing child is often different from that of an adult, and some observational studies have reported that high tidal volume does not correlate with patient outcomes in children, although the results may be affected by the severity of the disease.  　Due to the lack of evidence, it is not possible to make a clear recommendation for this CQ in children. Therefore, this CQ is not an evidence-based recommendation, but rather a description of the current practice.  **Summary of evidence**：  No relevant studies.  **Certainly of evidence**：  There have been no applicable RCTs; the certainty of the evidence cannot be described.  **Balance of evidence, Acceptability, Feasibility：**  There have been no applicable RCTs; the balance of effect cannot be described. However, with regard to the restriction of tidal volume, all that needs to be done is to change the ventilator settings, and there is no increase in burden or cost.  **Panel meeting：**  In the pre-vote, the modified Delphi method resulted in the recommendation statement, "We can not provide a recommendation regarding  tidal volume restrictions for pediatric patients with ARDS. It is generally accepted not to exceed tidal volume in accordance with treatment strategy of adult patients with ARDS  (in our practice statement)." and the supplementary items "There is no standard weight for children, but it may be better to calculate it from the height of pediatric ARDS patients using a standard growth curve, since lung capacity is expected to increase following alveolar formation, which continues until approximately 2 years of age, until the growth of the (long-stem) bones ceases." was agreed upon in our practice statement with a median score of 8.0 and a disagreement index of 0.1316.  At the panel meeting, it was suggested that the supplementary items should include a reference value for the physiological tidal volume. Therefore, the supplementary items were changed to "Currently, it is unclear whether to use standard weight or actual weight for the tidal volume per body weight. There is a reference physiological tidal volume value of less than 8 mL/kg." and revoting was conducted. The modified Delphi method resulted in a median score of 8.0, and a disagreement index of 0.1316, which was agreed upon, as in our practice statement.  **Additional considerations:**  It is not clear at this stage whether standard or actual weight should be used for calculating the physiological tidal volume. There is no standard weight for children, but it may be better to calculate it from the height of pediatric ARDS patients using a standard growth curve, since lung capacity is expected to increase following alveolar formation, which continues until approximately 2 years of age, until the growth of the (long-stem) bones ceases. In the US study by Imber et al, the Traub-Johnson formula and the WHO standard developmental chart for children were used, A reference value for physiological tidal volume is 8 mL/kg or less. |

| Subgroup considerations |
| --- |
| There are no RCTs on tidal volume restriction in pediatric patients with ARDS, so subgroup analysis is not possible. |
| Implementation considerations |
| As for the restriction of tidal volume, the most important thing is to change the setting of the ventilator, and there is no problem in introducing the strategy considering its cost, disadvantage, and acceptability.  It is also unclear whether standard or actual weight should be used to calculate the tidal volume per body weight.  The standard value for physiological tidal volume is 8 mL/kg or less. |

| Monitoring and evaluation |
| --- |
| Further information on the benefits and harms, such as mortality and VFD, needs to be collected. In addition, even after the publication of this guideline, it will be necessary to monitor the implementation status through questionnaires and other means to confirm whether there are any other clinical problems. |
| Research priorities |
| Lung protective strategies are ventilation methods used to reduce the adverse effects of mechanical ventilation, and tidal volume restriction is one of them. It is expected to reduce VILI/VALI, but it is unclear whether it leads to improved mortality. In addition, no studies have established specific numerical targets for tidal volume restrictions. In the future, clinical trials and RCTs on the feasibility of tidal volume restriction and its numerical targets in pediatric patients with ARDS are needed. |

Reference

1. Khemani RG, Newth CJ. The design of future pediatric mechanical ventilation trials for acute lung injury. Am J Respir Crit Care Med. 2010;182(12):1465-74.
2. Kneyber MC, Zhang H, Slutsky AS. Ventilator-induced lung injury. Similarity and differences between children and adults. Am J Respir Crit Care Med. 2014;190(3):258-65.
3. Imber DA, Thomas NJ, Yehya N. Association Between Tidal Volumes Adjusted for Ideal Body Weight and Outcomes in Pediatric Acute Respiratory Distress Syndrome. Pediatr Crit Care Med. 2019;20(3):e145-e53.
4. de Jager P, Burgerhof JG, van Heerde M, Albers MJ, Markhorst DG, Kneyber MC. Tidal volume and mortality in mechanically ventilated children: a systematic review and meta-analysis of observational studies*. Crit Care Med. 2014;42(12):2461-72.
5. Traub SL, Johnson CE. Comparison of methods of estimating creatinine clearance in children. Am J Hosp Pharm. 1980;37(2):195-201.

**PCQ3 Should high PEEP be used in pediatric patients with moderate to severe ARDS?**

1.Search strategy

MEDLINE via PubMed （Search date: 2020/7/8）

| #1 | Respiratory Distress Syndrome, Adult[mh] OR ARDS[tiab] OR shock lung[tiab] |
| --- | --- |
| #2 | acute respiratory distress[tiab] OR acute respiratory failure[tiab] |
| #3 | Acute[tiab] AND ((respirat*[tiab] OR ventilat*[tiab] OR pulmon*[tiab]) AND (fail*[tiab] OR depression[tiab])) |
| #4 | Lung injury[mh] OR ALI[tiab] OR Acute lung injur*[tiab] OR Ventilator-Induced Lung Injury[tiab] |
| #5 | Respiratory insufficiency[mh] OR Respiratory insufficiency[tiab] |
| #6 | Acute chest syndrome[mh] OR Acute chest syndrome[tiab] |
| #7 | #1 OR #2 OR #3 OR #4 OR #5 OR #6 |
| #8 | Positive-Pressure Respiration[mh] OR Positive Pressure Respiration[tiab] OR Positive End-Expiratory Pressure[tiab] OR PEEP[tiab] |
| #9 | Continuous Positive Airway Pressure[tiab] OR CPAP[tiab] OR NCPAP[tiab] |
| #10 | Airway Pressure Release Ventilation[tiab] OR APRV[tiab] |
| #11 | Intermittent Positive-Pressure Breathing[tiab] OR IPPB[tiab] |
| #12 | Intermittent Positive-Pressure Ventilation[tiab] OR IPPV[tiab] |
| #13 | lung protective ventilatory strateg*[tiab] OR LPVS[tiab] |
| #14 | alveolar recruit*[tiab] OR recruitment maneuve*[tiab] |
| #15 | #8 OR #9 OR #10 OR #11 OR #12 OR #13 OR #14 |
| #16 | #7 AND #15 |
| #17 | (randomized controlled trial[pt] OR controlled clinical trial[pt] OR randomized[tiab] OR placebo[tiab] OR clinical trials as topic[mesh:noexp] OR randomly[tiab] OR trial[ti]) NOT (animals[mh] NOT humans [mh]) |
| #18 | #16 AND #17 |

CENTRAL （Search date: 2020/7/8）

| #1 | [mh "Respiratory Distress Syndrome, Adult"] OR ARDS:ti,ab OR "shock lung":ti,ab |
| --- | --- |
| #2 | "acute respiratory distress":ti,ab OR "acute respiratory failure":ti,ab |
| #3 | Acute:ti,ab AND ((respirat*:ti,ab OR ventilat*:ti,ab OR pulmon*:ti,ab) AND (fail*:ti,ab OR depression:ti,ab)) |
| #4 | [mh "lung injury"] OR ALI:ti,ab OR "Acute lung injury":ti,ab OR "Ventilator-Induced Lung Injury":ti,ab |
| #5 | [mh "Respiratory insufficiency"] OR "Respiratory insufficiency":ti,ab |
| #6 | [mh "Acute chest syndrome"] OR "Acute chest syndrome":ti,ab |
| #7 | {OR #1-#6} |
| #8 | [mh "Positive Pressure Respiration"] OR "Positive Pressure Respiration":ti,ab OR "Positive End-Expiratory Pressure":ti,ab OR PEEP:ti,ab |
| #9 | "Continuous Positive Airway Pressure":ti,ab OR CPAP:ti,ab OR NCPAP:ti,ab |
| #10 | "Airway Pressure Release Ventilation":ti,ab OR APRV:ti,ab |
| #11 | "Intermittent Positive-Pressure Breathing":ti,ab OR IPPB:ti,ab |
| #12 | "Intermittent Positive-Pressure Ventilation":ti,ab OR IPPV:ti,ab |
| #13 | "lung protective ventilatory strategy":ti,ab OR LPVS:ti,ab |
| #14 | "Alveolar recruitment":ti,ab OR "recruitment maneuvers":ti,ab |
| #15 | {OR #8-#14} |
| #16 | #7 AND #15 |
| #17 | [mh animals] not [mh humans] |
| #18 | #16 not #17 |

Igaku-Chuo-Zasshi （Search date: 2020/7/12）

| #1 | 呼吸窮迫症候群-急性/TH or 急性呼吸促迫症候群/AL or ARDS/AL or "acute respiratory distress syndrome"/AL or ショック肺/AL or "shock lung"/AL |
| --- | --- |
| #2 | 肺損傷/TH or 急性肺損傷/AL or "acute lung Injury"/AL or 人工呼吸器誘発肺損傷/AL |
| #3 | 呼吸不全/TH or 呼吸不全/AL or 呼吸機能不全/AL |
| #4 | 呼吸抑制/TH or 呼吸抑制/AL |
| #5 | #1 or #2 or #3 or #4 |
| #6 | 陽圧呼吸/TH or 陽圧呼吸/AL or PEEP/AL |
| #7 | 気道圧解除換気法/AL or CPAP/AL or APRV/AL |
| #8 | 間欠的陽圧換気/AL or IPPV/AL or IPPB/AL |
| #9 | リクルートメント/AL |
| #10 | #6 or #7 or #8 or #9 |
| #11 | #5 and #10 |
| #12 | ランダム化比較試験/TH or ランダム化/AL or 無作為化/AL |
| #13 | 比較試験/AL |
| #14 | 臨床試験/TH or 臨床試験/AL |
| #15 | プラセボ/TH or プラセボ/AL |
| #16 | 対照/AL |
| #17 | コントロール/AL |
| #18 | 臨床研究・疫学研究/TH or 臨床研究/AL |
| #19 | #12 or #13 or #14 or #15 or #16 or #17 or #18 |
| #20 | #11 and #19 |
| #21 | (#20) and (PT=会議録除く) |

EMBASE (Search date: 2020/6/28)

| S1 | (EMB.EXACT("adult respiratory distress syndrome")) OR (TI,AB(ARDS OR "shock lung")) |
| --- | --- |
| S2 | (TI,AB("acute respiratory" p/0 (distress OR failure*))) |
| S3 | (TI,AB(acute n/3 (respirat* OR ventilat* OR pulmon*) n/3 (fail* OR depression))) |
| S4 | (EMB.EXACT("acute lung injury")) OR (EMB.EXACT("hyperoxia-induced lung injury") OR EMB.EXACT("lung injury")) OR (EMB.EXACT("ventilator induced lung injury")) OR (TI,AB(ALI OR ("acute lung" p/0 injur*) OR "ventilator-Induced lung injury")) |
| S5 | (EMB.EXACT.EXPLODE("respiratory failure")) OR (TI,AB(respiratory p/0 insufficien*)) |
| S6 | (EMB.EXACT("acute chest syndrome")) OR (TI,AB("acute chest syndrome")) |
| S7 | (S1 OR S2 OR S3 OR S4 OR S5 OR S6) |
| S8 | ((EMB.EXACT("positive end expiratory pressure")) OR (TI,AB("positive pressure respiration" OR "positive end-expiratory pressure" OR PEEP))) |
| S9 | (TI,AB("continuous positive airway pressure" OR CPAP OR NCPAP)) |
| S10 | (TI,AB("airway pressure release ventilation" OR APRV)) |
| S11 | (TI,AB("intermittent positive-pressure breathing" OR IPPB)) |
| S12 | (TI,AB("intermittent positive-pressure ventilation" OR IPPV)) |
| S13 | (TI,AB(("lung protective" p/0 ventilat* p/0 strateg*) OR LPVS)) |
| S14 | (TI,AB((alveolar p/0 recruit*) OR (recruitment p/0 maneuve*))) |
| S15 | (S8 OR S9 OR S10 OR S11 OR S12 OR S13 OR S14) |
| S16 | (S7 AND S15) |
| S17 | (((EMB.EXACT("controlled clinical trial") OR EMB.EXACT.EXPLODE("clinical trial (topic)") OR EMB.EXACT("randomized controlled trial")) OR (TI,AB(randomized) OR TI,AB(randomly) OR TI(trial))) NOT (ANIMAL(YES) NOT HUMAN(YES))) |
| S18 | (S16 AND S17) |

CHINAL (Search date: 2020/7/12)

| #1 | (MH "Respiratory Distress Syndrome, Adult") OR TI ARDS OR AB ARDS OR TI "shock lung" OR AB "shock lung" |
| --- | --- |
| #2 | TI "acute respiratory distress" OR AB "acute respiratory distress" OR TI "acute respiratory failure" OR AB "acute respiratory failure" |
| #3 | TI Acute OR AB Acute AND ((TI respirat* OR AB respirat* OR TI ventilat* OR AB ventilat* OR TI pulmon* OR AB pulmon*) AND (TI fail* OR AB fail* OR TI depression OR AB depression)) |
| #4 | (MH "Lung injury+") OR TI ALI OR AB ALI OR TI "Acute lung injur*" OR AB "Acute lung injur*" OR TI "Ventilator-Induced Lung Injury" OR AB "Ventilator-Induced Lung Injury" |
| #5 | (MH "Respiratory Failure+")  OR TI "Respiratory Failure" OR AB "Respiratory Failure" |
| #6 | (MH "Acute chest syndrome") OR TI "Acute chest syndrome" OR AB "Acute chest syndrome" |
| #7 | S1OR S2 OR S3 OR S4 OR S5 OR S6 |
| #8 | (MH "Positive-Pressure Respiration+") OR TI "Positive Pressure Respiration" OR AB "Positive Pressure Respiration" OR TI "Positive End-Expiratory Pressure" OR AB "Positive End-Expiratory Pressure" OR TI PEEP OR AB PEEP |
| #9 | TI "Continuous Positive Airway Pressure" OR AB "Continuous Positive Airway Pressure" OR TI CPAP OR AB CPAP OR TI NCPAP OR AB NCPAP |
| #10 | TI "Airway Pressure Release Ventilation" OR AB "Airway Pressure Release Ventilation" OR TI APRV OR AB APRV |
| #11 | TI "Intermittent Positive-Pressure Breathing" OR AB "Intermittent Positive-Pressure Breathing" OR TI IPPB OR AB IPPB |
| #12 | TI "Intermittent Positive-Pressure Ventilation" OR AB "Intermittent Positive-Pressure Ventilation" OR TI IPPV OR AB IPPV |
| #13 | TI "lung protective ventilatory strateg*" OR AB "lung protective ventilatory strateg*" OR TI LPVS OR AB LPVS |
| #14 | TI "alveolar recruit*" OR AB "alveolar recruit*" OR TI "recruitment maneuve*" OR AB "recruitment maneuve*" |
| #15 | #8 OR #9 OR #10 OR #11 OR #12 OR #13 OR #14 |
| #16 | #7 AND #15 |
| #17 | (MH "Randomized Controlled Trials") |
| #18 | TI ( random* or placebo* or blind* or double blind* ) OR AB ( random* or placebo* or blind* or double blind* ) |
| #19 | #17 OR #18 |
| #20 | #16 AND #19 |

1. Flow diagram

297 additional records identified through other sources

ICTRP (n=112)

Clinical Trials.gov (n=185)

**Identification**

6,233 records identified through database searching

Medline via PubMed (n=1,366)

CENTRAL (n=1,629)

EMBASE (n=2,433)

CHINAL (n=587)

Igaku-Chuo-Zasshi (n=218)

0 Studies included in qualitative synthesis

101 Full-text articles assessed for eligibility

4,376 records after duplicates removed

6,530 records identified through database searching

0 Studies included in quantitative synthesis (meta-analysis)

101 Full-text articles excluded, with reasons:

・Wrong language (n=5)

・Wrong study design (n=4)

・Wrong publication type (n=45)

・Wrong population (n=47)

Duplicates

n=2,154

4,275 records excluded

**Included**

**Eligibility**

**Screening**

1. Risk of bias

Not applicable

1. Forest plot

Not applicable

1. Evidence profile

Not applicable

1. Evidence-to-Decision table

| **QUESTION** | |
| --- | --- |
| **PCQ3：** Should high PEEP be used in pediatric patients with moderate to severe ARDS? | |
| **POPULATION:** | Pediatric patients (as defined in the article, 20 years old or younger if not specified) on ventilators for moderate to severe ARDS (as defined in the article) |
| **INTERVENTION:** | High Positive end-expiratory pressure (PEEP) (as defined in the article) |
| **COMPARISON:** | Low PEEP (as defined in the article) |
| **MAIN OUTCOMES:** | Mortality, Developmental prognosis, Ventilator-free days (VFD), Length of hospital stay, Hemodynamic deterioration |
| **SETTINGS:** | Emergency department or intensive care unit (ICU) |
| **PERSPECTIVE:** | Individual |
| **BACKGROUND:** | Acute respiratory distress syndrome (ARDS) is an acute and severe condition that affects the structure and function of the lungs due to increased permeability and damage to the capillaries surrounding the alveoli. ARDS has a high mortality rate and often requires mechanical ventilation. However, the use of ventilators can cause VILI/VALI. Therefore, treatment of these patients is based on a lung-protective ventilation strategy. The use of high levels of positive end-expiratory pressure (PEEP) is a strategy aimed at reducing VILI/VALI. Whether children can expect the same benefits from PEEP as adults, the benefits and harms of PEEP in children need to be examined. |
| **CONFLICT OF INTERESTS:** | None |

**ASSESSMENT**

| **Problem**  Is the problem a priority? | | |
| --- | --- | --- |
| **Judgement** | **Research evidence** | **Additional considerations** |
| ○ No  ○ Probably no  ○ Probably yes  ● Yes  ○ Varies  ○ Do not know | The use of high levels of PEEP is one of the strategies aimed at reducing VILI/VALI. Although the use of high PEEP may also reduce ventilator-associated lung injury in pediatric patients with ARDS, its efficacy and safety have not been established. Therefore, this issue is of high priority. |  |
| **Desirable Effects**  How substantial are the desirable anticipated effects? | | |
| **Judgement** | **Research evidence** | **Additional considerations** |
| ○ Trivial  ○ Small  ○ Moderate  ○ Large  ○ Varies  ● Do not know | Integrated into the “certainty of evidence”. |  |
| **Undesirable Effects**  How substantial are the undesirable anticipated effects? | | |
| **Judgement** | **Research evidence** | **Additional considerations** |
| ○ Large  ○ Moderate  ○ Small  ○ Trivial  ○ Varies  ● Do not know | Integrated into the “certainty of evidence”. |  |
| **Certainty of evidence**  What is the overall certainty of the evidence of effects? | | |
| **Judgement** | **Research evidence** | **Additional considerations** |
| ○ Very low  ○ Low  ○ Moderate  ○ High  ● No included studies | The use of high PEEP in patients with ARDS is thought to reduce VILI/VALI by preventing end-expiratory alveolar collapse, improving oxygenation by increasing end-expiratory lung capacity, and reducing tidal recruitment by improving respiratory system compliance. On the other hand, the use of high PEEP may have negative influences, such as adverse effects on the cardiovascular system due to increased intrathoracic pressure. A comparison of the balance of these benefits and harms in pediatric ARDS patients on ventilatory management has not been performed, and the certainty of the evidence cannot be described. |  |
| **Values**  Is there important uncertainty about or variability in how much people value the main outcomes? | | |
| **Judgement** | **Research evidence** | **Additional considerations** |
| ○ Important uncertainty or variability  ○ Possibly important uncertainty or variability  ○ Probably no important uncertainty or variability  ● No important uncertainty or variability | “Mortality” is generally a critical outcome, and there is no great uncertainty or diversity of values about this. |  |
| **Balance of effects**  Does the balance between desirable and undesirable effects favor the intervention or the comparison? | | |
| **Judgement** | **Research evidence** | **Additional considerations** |
| ○ Favors the comparison  ○ Probably favors the comparison  ○ Does not favor either the intervention or the comparison  ○ Probably favors the intervention  ○ Favors the intervention  ○ Varies  ● Do not know | No relevant studies. |  |
| **Acceptability**  Is the intervention acceptable to key stakeholders? | | |
| **Judgement** | **Research evidence** | **Additional considerations** |
| ○ No  ○ Probably no  ● Probably yes  ○ Yes  ○ Varies  ○ Do not know | All that needs to be done is to change the ventilator settings, which will probably be acceptable to the patient and family. |  |
| **Feasibility**  Is the intervention feasible to implement? | | |
| **Judgement** | **Research evidence** | **Additional considerations** |
| ○ No  ○ Probably no  ○ Probably yes  ● Yes  ○ Varies  ○ Do not know | All that needs to be done is to change the ventilator settings, which is considered feasible. |  |

**Summary of Judgement**

|  | **JUDGEMENT** | | | | | | |
| --- | --- | --- | --- | --- | --- | --- | --- |
| **PROBLEM** | No | Probably no | Probably yes | **Yes** |  | Varies | Do not know |
| **DESIRABLE EFFECTS** | Trivial | Small | Moderate | Large |  | Varies | **Do not know** |
| **UNDESIRABLE EFFECTS** | Large | Moderate | Small | Trivial |  | Varies | **Do not know** |
| **CERTAINTY OF EVIDENCE** | Very low | Low | Moderate | High |  |  | **No included studies** |
| **VALUES** | Important uncertainty or variability | Possibly important uncertainty or   variability | Probably no important uncertainty or variability | **No important uncertainty or variability** |  |  |  |
| **BALANCE OF EFFECTS** | Favors the comparison | Probably favors the comparison | Does not favor either the intervention or the comparison | Probably favors the intervention | Favors the intervention | Varies | **Do not know** |
| **ACCEPTABILITY** | No | Probably no | **Probably yes** | Yes |  | Varies | Do not know |
| **FEASIBILITY** | No | Probably no | Probably yes | **Yes** |  | Varies | Do not know |

**Type of Recommendation**

| Strong recommendation against the intervention | Conditional recommendation against the intervention | Conditional recommendation for either the intervention or the comparison | Conditional recommendation for the intervention | Strong recommendation for the intervention |
| --- | --- | --- | --- | --- |
| ○ | ○ | ○ | ○ | ○ |

**CONCLUSION**

| **Recommendation** |
| --- |
| We could not provide a recommendation on whether to use high PEEP in pediatric patients with moderate to severe ARDS, but high PEEP is commonly used based on clinician’s experience and findings from adult patients with ARDS (in our practice statement).  Supplementary item:  Care should be taken to avoid an excessive plateau pressure when using high PEEP (especially >10 cmH_2_O). Additionally, careful monitoring of adverse events such as hemodynamic instability is needed. The effects of PEEP differ according to lung pathophysiology, and high PEEP does not necessarily improve oxygenation or lung injury. |
|  |
| **Justification** |
| **Question:** Should high PEEP be used in pediatric patients with moderate to severe ARDS?  **Population:** Pediatric patients (as defined in the article, 20 years old or younger if not specified) on ventilators for moderate to severe ARDS (as defined in the article)  **Intervention:** High PEEP (as defined in the article)  **Comparison:** Low PEEP (as defined in the article)  **Explanation:** In adult patients with ARDS, a meta-analysis of 16 RCTs in this guideline found a trend toward lower short- and long-term mortality and increased VFD in the high PEEP group and a similar level of pressure injuries between the high and low PEEP groups. It is suggested that high PEEP should be used for ventilation in adults with ARDS (grade 2D, weak recommendation/certainty of evidence "very low").  On the other hand, there are no RCTs in pediatric ARDS patients, which may not lead to a recommendation in this guideline. In terms of PEEP settings for pediatric patients with ARDS, the PALICC^1^ recommends a PEEP of 10–15 cmH_2_O with careful attention to oxygenation and hemodynamics in children and also notes plateau pressure and mention the possibility that more than 15 cmH_2_O may be needed, based on the results of studies in adults. Surviving Sepsis Campaign International Guidelines for the Management of Septic Shock and Sepsis-Associated Organ Dysfunction in Children^3^ recommends the use of high PEEP for pediatric patients with ARDS due to sepsis (weak recommendation/certainty of evidence "very low"), although all the studies used as evidence are observational ones. The Pediatric Mechanical Ventilation Consensus Conference^2^ did not mention specific PEEP values, but noted that in more severe pediatric ARDS patients, a higher PEEP may be needed to improve end-expiratory lung volumes and respiratory compliance, it should be noted that higher PEEP may be required in more severe pediatric ARDS patients to improve end-expiratory lung volumes and respiratory compliance. The use of PEEP equivalent to or higher than the ARDSNet low PEEP/FIO2 table in pediatric ARDS patients has been reported to improve mortality adjusted for multivariate analysis and propensity score methods.^4^ However, these results have not been validated in RCTs, and the validity of using the PEEP/FIO2 table used in adults in pediatric patients has not been established. It is also not clear whether the appropriate PEEP should be determined uniformly or physiologically on an individual basis.  PEEP settings in pediatric ARDS patients should be individualized according to the findings of adult RCTs, institutional management policies, and clinician experience, with attention to the degree of oxygenation, underlying pathology, and hemodynamics. In recent observational studies, approximately 10 cmH2O has been used.^5,6^  　Due to the lack of good evidence on the use of high PEEP in high-risk pediatric ARDS patients, no clear recommendation can be made for this CQ. Therefore, this CQ is not an evidence-based recommendation, but only a description of the current practice.  **Summary of evidence:** no relevant studies.  **Certainty of evidence:** Since there are no relevant studies, the certainty of evidence cannot be assessed.  **Balance of effects, Acceptability, Feasibility:** It is impossible to assess the balance of effects as there are no relevant studies. PEEP can be changed only by changing the ventilator settings, and there is no problem with acceptance or feasibility.  **Panel meeting:** In the pre ballot, the modified Delphi method resulted in the following recommendation: "We could not provide a recommendation whether to use high PEEP in pediatric patients with moderate to severe ARDS, but high PEEP is commonly used based on clinician’s experience and findings from adult patients with ARDS (in our practice statement)." and the supplementary items “When using a high PEEP (>10 cmH2O), care should be taken to avoid excessive plateau pressure (maximal inspiratory pressure).” In addition, careful monitoring for adverse events such as hemodynamic compromise is required." was agreed upon in our practice statement with a median score of 8 and a disagreement index of 0.1316.  At the panel meeting, it was suggested that the supplementary items should include the fact that lung recruitment varies from patient to patient. Therefore, the supplementary conditions were changed into "Care should be taken to avoid an excessive plateau pressure when using high PEEP (specially more than 10 cmH_2_O). Additionally, careful monitoring is needed to monitor adverse events such as hemodynamic instability. The effects of PEEP differ according to lung pathophysiology, and high PEEP does not necessarily improve oxygenation or lung injury." The change was in agreement with a median score of 9 and a disagreement index of 0.1316, as in our practice statement, using the modified Delphi method.  **Additional considerations:** When using high PEEP (>10 cmH2O), care should be taken to avoid excessive plateau pressure (peak inspiratory pressure) and careful monitoring for adverse events such as hemodynamic compromise is needed. The effect of PEEP on oxygenation varies among individuals, and high PEEP does not always improve oxygenation. |

| **Subgroup considerations** |
| --- |
| Patients in shock, congenital heart disease (especially single ventricle, right heart bypass), and those with elevated intracranial pressure may require more careful monitoring when using high PEEP. |
| **Implementation considerations** |
| High PEEP can have adverse cardiovascular effects, such as hypotension. In addition, the response to high PEEP varies from patient to patient, depending on the recruitability of the lungs. Once a high PEEP is applied, its efficacy should be assessed and its effects on circulatory dynamics should be continuously monitored. |

| **Monitoring and evaluation** |
| --- |
| After implementing this recommendation, it is necessary to monitor the status of implementation and adverse effects on the cardiovascular system or central nervous system through questionnaires and other means. |
| **Research priorities** |
| There is no clear data on the level of PEEP actually used in the treatment of pediatric ARDS patients in Japan, and further studies are needed. In this CQ, there are no RCTs, and no ongoing studies have been registered with Clinical Trial.gov or UMIN. RCTs are needed to assess the necessity of higher PEEP in high-risk pediatric patients with ARDS. On that occasion, because the optimal method for setting PEEP has not been established, and we need to consider whether to set a uniform PEEP or a more individualized PEEP based on physiological assessment needs to be investigated. |

Reference

1. Rimensberger, P.C., I.M. Cheifetz, and G. Pediatric Acute Lung Injury Consensus Conference, Ventilatory support in children with pediatric acute respiratory distress syndrome: proceedings from the Pediatric Acute Lung Injury Consensus Conference. Pediatr Crit Care Med, 2015. 16(5 Suppl 1): p. S51-60.
2. Kneyber, M.C.J., et al., Recommendations for mechanical ventilation of critically ill children from the Paediatric Mechanical Ventilation Consensus Conference (PEMVECC). Intensive Care Med, 2017. 43(12): p. 1764-1780.
3. Weiss, S.L., et al., Surviving Sepsis Campaign International Guidelines for the Management of Septic Shock and Sepsis-Associated Organ Dysfunction in Children. Pediatr Crit Care Med, 2020. 21(2): p. e52-e106.
4. Khemani, R.G., PEEP lower than the ARDS Network protocol is associated with higher pediatric ARDS mortality. American Journal of Respiratory and Critical Care Medicine, 2018.
5. Yehya, N., G. Keim, and N.J. Thomas, Subtypes of pediatric acute respiratory distress syndrome have different predictors of mortality. Intensive Care Med, 2018.
6. Wong, J.J.M., et al., Lung-Protective Mechanical Ventilation Strategies in Pediatric Acute Respiratory Distress Syndrome. Pediatr Crit Care Med, 2020. 21(8): p. 720-728.

**PCQ4 Should plateau pressure be restricted in pediatric patients with ARDS?**

1.Search strategy

MEDLINE via Pubmed (Search date: 2020/7/23)

| #1 | Respiratory distress syndrome, adult[mh] OR adult respiratory distress syndrom*[tiab] OR acute respiratory distress syndrom*[tiab] OR shock lung[tiab] OR ARDS*[tiab] |
| --- | --- |
| #2 | lung injury[mh] OR lung injur*[tiab] OR ALI[tiab] |
| #3 | acute[tiab] AND lung[tiab] AND (fail*[tiab] OR depression[tiab]) |
| #4 | ALI[tiab] OR Acute lung injur*[tiab] OR Ventilator-Induced Lung Injury[tiab] |
| #5 | Acute [tiab] AND (respirat*[tiab] OR ventilat*[tiab] OR pulmon*[tiab]) AND (fail*[tiab] OR depression[tiab]) |
| #6 | Acute chest syndrome[mh] OR (acute[tiab] AND chest[tiab] AND syndrom*[tiab]) |
| #7 | #1 OR #2 OR #3 OR #4 OR #5 OR #6 |
| #8 | Respiration,artificial[mesh: noexp] OR Positive-Pressure Respiration[mh] OR Artificial respiration[tiab] OR Artificial ventilation[tiab] |
| #9 | Ventilators, Mechanical[mh] OR Mechanical ventilation[tiab] |
| #10 | pressure[tiab] AND (limit*[tiab] OR low[tiab] OR lower[tiab] OR less[tiab]) |
| #11 | (#8 OR #9) AND #10 |
| #12 | Tidal volume[mh] OR tidal volume*[tiab] |
| #13 | plateau pressure*[tiab] |
| #14 | LPVS [tiab] OR lung protective* [tiab] OR (protective [tiab] AND ventilat* [tiab]) |
| #15 | #11 OR #12 OR #13 OR #14 |
| #16 | #7 AND #15 |
| #17 | randomized controlled trial[pt] OR controlled clinical trial[pt] OR randomized[tiab] OR placebo[tiab] OR clinical trials as topic[mesh: noexp] OR randomly[tiab] OR trial[ti] |
| #18 | animals[mh] NOT humans[mh] |
| #19 | #17 NOT #18 |
| #20 | #16 AND #19 |

CENTRAL (Search date: 2020/7/24)

| #1 | [mh "Respiratory distress syndrome, adult"] OR "adult respiratory distress syndrome":ti,ab OR "acute respiratory distress syndrome":ti,ab OR "shock lung":ti,ab OR ARDS:ti,ab |
| --- | --- |
| #2 | [mh "lung injury"] OR "lung injury":ti,ab OR ALI:ti,ab |
| #3 | acute:ti,ab AND lung:ti,ab AND (failure:ti,ab OR depression:ti,ab) |
| #4 | ALI:ti,ab OR "Acute lung injury":ti,ab OR "Ventilator-Induced Lung Injury":ti,ab |
| #5 | Acute:ti,ab AND (respiratory:ti,ab OR ventilatory:ti,ab OR pulmonary:ti,ab) AND (failure:ti,ab OR depression:ti,ab) |
| #6 | [mh "Acute chest syndrome"] OR (acute:ti,ab AND chest:ti,ab AND syndrome:ti,ab) |
| #7 | {OR #1-#6} |
| #8 | [mh "Respiration, artificial"] OR "Artificial respiration":ti,ab OR "Artificial ventilation":ti,ab |
| #9 | [mh "Ventilators, Mechanical"] OR "Mechanical ventilation":ti,ab |
| #10 | pressure:ti,ab AND (limited:ti,ab OR low:ti,ab OR lower:ti,ab OR less:ti,ab) |
| #11 | (#8 OR #9) AND #10 |
| #12 | [mh "Tidal volume"] OR "tidal volume":ti,ab |
| #13 | "plateau pressure":ti,ab |
| #14 | LPVS:ti,ab OR "lung protective":ti,ab OR (protective:ti,ab AND ventilation:ti,ab) |
| #15 | {OR #11-#14} |
| #16 | #7 AND #15 |
| #17 | [mh animals] NOT [mh humans] |
| #18 | #16 NOT #17 |

Igaku-Chuo-Zasshi (Search date: 2020/7/16)

| #1 | 呼吸窮迫症候群-急性/TH or 成人呼吸窮迫症候群/TA or 成人型呼吸窮迫症候群/TA or 成人呼吸促迫症候群/TA or 成人型呼吸促迫症候群/TA or 急性呼吸窮迫症候群/TA or 急性呼吸促迫症候群/TA or ショック肺/TA or ARDS/TA |
| --- | --- |
| #2 | 肺損傷/TH or 肺損傷/TA or 肺傷害/TA or 肺障害/TA |
| #3 | 急性/TA and 肺/TA and (不全/TA or 低下/TA or 抑制/TA) |
| #4 | 呼吸不全/TH or 呼吸不全/TA or 呼吸機能不全/TA or 換気不全/TA or 肺機能不全/TA |
| #5 | 急性/TA and (呼吸/TA or 換気/TA or 肺機能/TA) and (不全/TA or 低下/TA or 抑制/TA) |
| #6 | 急性胸部症候群/TH or (急性/TA and 胸部/TA and 症候群/TA) |
| #7 | #1 or #2 or #3 or #4 or #5 or #6 |
| #8 | 人工呼吸/TH or 人工呼吸/TA or 陽圧呼吸/TA or 人工換気/TA or 陽圧換気/TA or 機械的換気/TA |
| #9 | 人工呼吸器/TH or 人工呼吸器/TA or レスピレータ/TA or ベンチレータ/TA |
| #10 | 圧/TA and (制限/TA or 低/TA or 減/TA) |
| #11 | ( #8 OR #9) and #10 |
| #12 | プラトー圧/TA |
| #13 | 肺保護戦略/TA or 肺保護/TA or (保護/TA and 換気/TA) |
| #14 | #11 or #12 or #13 |
| #15 | ランダム化比較試験/TH or 準ランダム化比較試験/TH or ランダム/AL and 化/AL or 無作為化/AL or 比較試験/AL or 臨床試験/AL or プラセ/AL and ボ/AL or 対照/AL or コントロール/AL or 臨床研究/AL |
| #16 | (CK=動物) not (CK=ヒト) |
| #17 | #15 not #16 |
| #18 | #7 and #14 and #17 |
| #19 | (#18) and (PT=会議録除く) |

1. Flow diagram

**Identification**

0 Studies included in qualitative synthesis

9 Full-text articles assessed for eligibility

2,264 records after duplicates removed

2,822 records identified through database searching

2,822 records identified through database searching

Medline via PubMed (n=1,362)

CENTRAL (n=1,335)

Igaku-Chuo-Zasshi (n=125)

0 additional records identified through other sources

0 Studies included in quantitative synthesis (meta-analysis)

Duplicates

n=558

2,255 records excluded

**Included**

**Eligibility**

**Screening**

9 Full-text articles excluded, with reasons:

・Wrong population (n=9)

1. Risk of bias

Not applicable

1. Forest plot

Not applicable

1. Evidence profile

Not applicable

1. Evidence-to-Decision table

| **QUESTION** | |
| --- | --- |
| **PCQ4：** Should plateau pressure be restricted in pediatric patients with ARDS? | |
| **POPULATION:** | Pediatric patients (as defined in the article, 20 years old or younger if not specified) on ventilators for ARDS (as defined in the article) |
| **INTERVENTION:** | Restrict plateau pressure |
| **COMPARISON:** | Do not restrict plateau pressure |
| **MAIN OUTCOMES:** | Mortality, Developmental prognosis, Ventilator-free days (VFD), Length of hospital stay, Hemodynamic deterioration |
| **SETTINGS:** | Emergency department or intensive care unit (ICU) |
| **PERSPECTIVE:** | Individual |
| **BACKGROUND:** | In the ventilatory management of adult patients with ARDS, VILI/VALI may not only lead to prolonged duration of mechanical ventilation, but also to increased mortality. As one of the factors causing VILI/VALI is an increase in airway pressure, restricting plateau pressure is expected to suppress VILI/VALI. On the other hand, restricting plateau pressure is not only beneficial but may also cause adverse events such as hypercapnia. The effects and adverse events of plateau pressure restriction in pediatric patients with ARDS need to be clarified. |
| **CONFLICT OF INTERESTS:** | None |

**ASSESSMENT**

| **Problem**  Is the problem a priority? | | |
| --- | --- | --- |
| **Judgement** | **Research evidence** | **Additional considerations** |
| ○ No  ○ Probably no  ○ Probably yes  ● Yes  ○ Varies  ○ Do not know | Since VILI/VALI is thought to be caused by increased ventilation and airway pressure during ventilatory management, restricting plateau pressure is expected to reduce VILI/VALI. On the other hand, restricting plateau pressure is not only beneficial but may also cause adverse events such as hypercapnia. Validation to clarify the effects and adverse events of plateau pressure restriction in pediatric patients with ARDS is needed, and this issue is of high priority. |  |
| **Desirable Effects**  How substantial are the desirable anticipated effects? | | |
| **Judgement** | **Research evidence** | **Additional considerations** |
| ○ Trivial  ○ Small  ○ Moderate  ○ Large  ○ Varies  ● Do not know | Integrated into the “Certainty of evidence”. |  |
| **Undesirable Effects**  How substantial are the undesirable anticipated effects? | | |
| **Judgement** | **Research evidence** | **Additional considerations** |
| ○ Large  ○ Moderate  ○ Small  ○ Trivial  ○ Varies  ● Do not know | Integrated into the “Certainty of evidence”. |  |
| **Certainty of evidence**  What is the overall certainty of the evidence of effects? | | |
| **Judgement** | **Research evidence** | **Additional considerations** |
| ○ Very low  ○ Low  ○ Moderate  ○ High  ● No included studies | While restricting plateau pressure in ARDS patients may reduce VILI/VALI, there are concerns about adverse effects on hemodynamics and tissue metabolism due to hypercapnia and altered blood acid-base balance. There are no applicable RCTs in pediatric patients with ARDS undergoing ventilatory management, and the certainty of the evidence cannot be described. |  |
| **Values**  Is there important uncertainty about or variability in how much people value the main outcomes? | | |
| **Judgement** | **Research evidence** | **Additional considerations** |
| ○ Important uncertainty or variability  ○ Possibly important uncertainty or variability  ○ Probably no important uncertainty or variability  ● No important uncertainty or variability | “Mortality” is generally a critical outcome, and there is no great uncertainty or diversity of values about this. |  |
| **Balance of effects**  Does the balance between desirable and undesirable effects favor the intervention or the comparison? | | |
| **Judgement** | **Research evidence** | **Additional considerations** |
| ○ Favors the comparison  ○ Probably favors the comparison  ○ Does not favor either the intervention or the comparison  ○ Probably favors the intervention  ○ Favors the intervention  ○ Varies  ● Do not know | No relevant studies. |  |
| **Acceptability**  Is the intervention acceptable to key stakeholders? | | |
| **Judgement** | **Research evidence** | **Additional considerations** |
| ○ No  ○ Probably no  ● Probably yes  ○ Yes  ○ Varies  ○ Do not know | Even considering the harm, what is required is to change the ventilator settings, which will probably be acceptable to the patient and family. |  |
| **Feasibility**  Is the intervention feasible to implement? | | |
| **Judgement** | **Research evidence** | **Additional considerations** |
| ○ No  ○ Probably no  ○ Probably yes  ● Yes  ○ Varies  ○ Do not know | All that needs to be done is to change the ventilator settings, which is considered feasible. |  |

**Summary of Judgement**

|  | **JUDGEMENT** | | | | | | |
| --- | --- | --- | --- | --- | --- | --- | --- |
| **PROBLEM** | No | Probably no | Probably yes | **Yes** |  | Varies | Do not know |
| **DESIRABLE EFFECTS** | Trivial | Small | Moderate | Large |  | Varies | **Do not know** |
| **UNDESIRABLE EFFECTS** | Large | Moderate | Small | Trivial |  | Varies | **Do not know** |
| **CERTAINTY OF EVIDENCE** | Very low | Low | Moderate | High |  |  | **No included studies** |
| **VALUES** | Important uncertainty or variability | Possibly important uncertainty or   variability | Probably no important uncertainty or variability | **No important uncertainty or variability** |  |  |  |
| **BALANCE OF EFFECTS** | Favors the comparison | Probably favors the comparison | Does not favor either the intervention or the comparison | Probably favors the intervention | Favors the intervention | Varies | **Do not know** |
| **ACCEPTABILITY** | No | Probably no | **Probably yes** | Yes |  | Varies | Do not know |
| **FEASIBILITY** | No | Probably no | Probably yes | **Yes** |  | Varies | Do not know |

**Type of Recommendation**

| Strong recommendation against the intervention | Conditional recommendation against the intervention | Conditional recommendation for either the intervention or the comparison | Conditional recommendation for the intervention | Strong recommendation for the intervention |
| --- | --- | --- | --- | --- |
| ○ | ○ | ○ | ○ | ○ |

**CONCLUSION**

| **Recommendation** |
| --- |
| We cannot provide a recommendation regarding plateau pressure restrictions for pediatric patients with ARDS, but respiratory management with restrictions on plateau pressure is implemented in accordance with the treatment strategy of adult patients with ARDS (in our practice statement).  Supplementary item:  The standard has been less than 28cmH_2_O (more pressure may be required in conditions with decreased chest wall compliance). It should be noted that plateau pressure alone is not sufficient in controlling alveolar hyperextension if there is strong spontaneous breathing. It should also be noted that the plateau pressure and maximum airway pressure are different even in the case of pressure-control ventilation. |
|  |
| **Justification** |
| **Question：**　Should plateau pressure be restricted in pediatric patients with ARDS?  **Population：**　Pediatric patients (as defined in the article, 20 years old or younger if not specified) on ventilators for ARDS (as defined in the article)  **Intervention：**　Restrict plateau pressure  **Comparison：**　Do not restrict plateau pressure  **Explanation：**  　In general, increased tidal volume and increased airway pressure are the main factors in the development of VILI/VALI in ventilated patients. Restricting plateau pressure is expected to suppress both of these factors, but due to the lack of high-quality evidence in pediatric patients with ARDS, we cannot provide clear recommendations in this CQ.  In adult patients with ARDS, a meta-analysis of six RCTs in this guideline suggested restricting plateau pressure (Grade 2D, strength of recommendation "weak recommendation"/certainty of evidence "very low"). The Surviving Sepsis Campaign: International Guidelines for the Management of Sepsis and Septic Shock 2016^1^ also recommends restricting the plateau pressure to less than 30 cmH_2_O (strong recommendation, moderate quality of evidence).  Regarding plateau pressure restricts for pediatric patients with ARDS, the PALICC (2015)^2^ and the PEMVECC (2017)^3^ recommended a plateau pressure of 28 cmH_2_O or less (29–32 cmH_2_O or less with reduced thoracic compliance) based on the results of studies in adults and observational studies in children^4-7^ (weak agreement). However, the pediatric studies on which this recommendation was based were all observational studies and examined the relationship between ⊿P (maximal inspiratory pressure - PEEP) and mortality, not plateau pressure. In addition, an observational study^8^ found no association between ⊿P and mortality in pediatric patients with ARDS.  Although there is insufficient evidence that plateau pressure restriction (or ventilatory pressure restriction) is beneficial in pediatric patients with ARDS, considering the results of studies in adult ARDS patients and its theoretical potential to reduce VILI/VALI, and thus its impact on long-term lung function, plateau pressure restriction seems to be a useful strategy in pediatric ARDS patients.    **Summary of evidence**：  No relevant studies.  **Certainty of evidence**：  There have been no applicable RCTs, the certainty of the evidence cannot be described.  **Balance of evidence, Acceptability, Feasibility：**  There have been no applicable RCTs, the balance of effect cannot be described. However, with regard to the restriction of plateau pressure, all that needs to be done is change the ventilator settings, and there Is no problem in acceptability and feasibility.  **Panel meeting：**  In the pre-vote, the recommendation that "We cannot provide a recommendation regarding plateau pressure restrictions for pediatric patients with ARDS, but respiratory management with restrictions on plateau pressure is implemented in accordance with the treatment strategy of adult patients with ARDS (in our practice statement).” and the supplementary items “However a plateau pressure of 28 cmH_2_O or less (29–32 cmH_2_O or less with increased chest wall compliance) is guided, it should be noted that the plateau pressure restrict alone is not sufficient to control transpulmonary pressure in the presence of strong spontaneous breathing” was agreed as in our practice statement with a median score of 9 and disagreement index of 0.1316, using the modified Delphi method.  At the panel meeting, concerns about the supplementary items, especially the strength of the rationale for the data of 29–32 cmH_2_O and the recognition of the expression "transpulmonary pressure" in Japan, were expressed. Therefore, the supplementary items were changed to “The standard has been less than 28cm H_2_O (more pressure may be required in conditions with decreased chest wall compliance). It should be noted that plateau pressure alone is not sufficient in controlling alveolar hyperextension if there is strong spontaneous breathing. It should also be noted that the plateau pressure and maximum airway pressure are different even in the case of pressure-control ventilation.” and revoting was conducted. The modified Delphi method resulted in a median score of 9.0, and a disagreement index of 0.1316, which was agreed upon, as in our practice statement.  **Additional considerations：**  However, a plateau pressure of 28 cmH_2_O or less is common, higher pressures may be required with reduced thoracic compliance. It should be noted that the plateau pressure restriction alone is not sufficient to control barotrauma (alveolar hyperextension) in the presence of spontaneous breathing.  Thoracic compliance refers to the ease in expanding of thoracic. Low compliance means that thoracic is not easy to expand.  The pressure disparity between the inside and outside of the alveoli is called transpulmonary pressure, and it is the pressure that actually causes the alveoli to expand. Transpulmonary pressure is calculated by the formula that airway pressure minus intrathoracic pressure, and transpulmonary pressure can be calculated by monitoring the esophageal pressure. |

| **Subgroup considerations** |
| --- |
| There are no RCTs on tidal volume restriction in pediatric ARDS patients, so subgroup analysis is not possible. |
| **Implementation considerations** |
| Restricting the plateau pressure can be achieved by simply changing the ventilator settings, and there are no implementation issues such as cost, disadvantage, or acceptability. The plateau pressure displayed on the ventilator was influenced by the compliance of the lungs and the thorax. The purpose of plateau pressure restriction is to prevent lung pressure injury by restricting transpulmonary pressure. Transpulmonary pressure is defined by the intrapulmonary pressure generated by the ventilator and the intrathoracic negative pressure generated by spontaneous breathing. Therefore, in the presence of strong spontaneous breathing, the plateau pressure restriction is not sufficient to limit the transpulmonary pressure. On the other hand, if thoracic compliance is low, a much higher plateau pressure may be required. In addition, in pressure control ventilation, the maximum inspiratory pressure and plateau pressure do not always coincide if the airflow is not zero at the end of the inspiratory phase. |

| **Monitoring and evaluation** |
| --- |
| As for clinical issues in the implementation of this recommendation, further information on the benefits and harms, such as death and VFD, needs to be collected. In addition, even after the publication of this guideline, it will be necessary to monitor the implementation status through questionnaires and other means to confirm whether there are any other clinical problems. |
| **Research priorities** |
| Restricting plateau pressure is expected to reduce tidal volume during ventilatory management and reduce the increase in airway pressure; however, it is unclear whether this will lead to improved mortality. No studies that have defined an upper limit for the plateau pressure. Future clinical trials and RCTs on the plateau pressure restriction in pediatric patients with ARDS are needed. |

Reference

1. Rhodes A, Evans LE, Alhazzani W, et al. Surviving sepsis campaign: International guidelines for management of sepsis and septic shock: 2016. Intensive Care Med. 2017; 43 (3): 304-77. PMID: 28101605
2. Rimensberger PC, Cheifetz IM, Pediatric Acute Lung Injury Consensus Group. Ventilatory support in children with pediatric acute respiratory distress syndrome: proceedings from the pediatric acute lung injury consensus conference. 2015; 16 (5 Suppl 1): S51-60. PMID: 26035364
3. Kneyber MC, de Luca DD, Calderini, et al. Recommendations for mechanical ventilation of critically ill children from the paediatric mechanical ventilation concensus conference (PEMVECC). Intensive Care Med. 2017; 43(12): 1764-80. PMID: 28936698
4. Flori HR, Gidden DV, Rutherford GW, Matthay MA. Pediatric acute lung injury: prospective evaluation of risk factors associated with mortality. Am J Respr Crit Care Med. 2005; 171(9): 995-1001. PMID:15618461
5. Erickson S, Schibler A, Numa A, et al. Acute lung injury in pediatric intensive care in Australia and New Zealand: a prospective multicenter, observational study. Pediatr Crit Care Med. 2007; 8(4): 317-23. PMID:17545931
6. Khemani RG, Conti D, Alonzo TA, et al. Effect of tidal volume in children with acute hypoxemic respiratory failure. Intensive Care Med. 2009; 35(8): 1428-37. PMID:19533092
7. Panico FF, Troster EJ, Oliveria CS, et al. Risk factors for mortality and outcomes in pediatric acute lung injury/acute respiratory distress syndrome. Pediatr Crit Care Med. 2015; 16(7): e194-200. PMID:26181296
8. Yehya N, Thomas NJ. Disassociating lung mechanics and oxygenation in pediatric acute respiratory distress syndrome. Crit Care Med. 2017; 45(7): 1232-39. PMID: 28350644

**PCQ5 Should a protocol be used when liberating pediatric patients with acute respiratory failure from mechanical ventilator?**

1.Search strategy

MEDLINE via Pubmed (Search date: 2020/7/2)

| 1 | Ventilator Weaning[mh] OR artificial respiration*[tiab] OR Positive-Pressure Respiration[mh] OR positive-pressure respiration[tiab] |
| --- | --- |
| 2 | Pulmonary Ventilation[mh] OR pulmonary ventilat*[tiab] |
| 3 | Ventilators, Mechanical[mh] OR mechanical ventilat*[tiab] OR Negative-Pressure Respirator[tiab] OR negative pressure respirators[tiab] |
| 4 | #1 OR #2 OR #3 |
| 5 | Clinical Protocols[mh] or protocol*[tiab] |
| 6 | assist ventilation[tiab] OR adaptive support ventilation[tiab] OR daily screening[tiab] |
| 7 | (auto*[tiab] OR system*[tiab]) AND weaning*[tiab] |
| 8 | #5 OR #6 OR #7 |
| 9 | (randomized controlled trial [pt] OR controlled clinical trial [pt] OR randomized [tiab] OR placebo [tiab] OR drug therapy [sh] OR randomly [tiab] OR trial [tiab] OR groups [tiab]) NOT (Animals[mh] NOT Humans[mh]) |
| 10 | #4 AND #8 AND #9 |

CENTRAL (Search date: 2020/7/2)

| #1 | [mh "Ventilator Weaning"] OR "artificial respiration":ti,ab OR [mh "Positive-Pressure Respiration"] OR "positive-pressure respiration":ti,ab |
| --- | --- |
| #2 | [mh "Pulmonary Ventilation"] OR "pulmonary ventilation":ti,ab |
| #3 | [mh "Ventilators, Mechanical"] OR "mechanical ventilation":ti,ab OR "Negative-Pressure Respirator":ti,ab OR "negative pressure respirators":ti,ab |
| #4 | {OR #1-#3} |
| #5 | [mh "Clinical Protocols"] OR protocol*:ti,ab |
| #6 | "assist ventilation":ti,ab OR "adaptive support ventilation":ti,ab OR "daily screening":ti,ab |
| #7 | (auto*:ti,ab OR system*:ti,ab) AND weaning*:ti,ab |
| #8 | {OR #5-#7} |
| #9 | #4 AND #8 |
| #10 | [mh Animals] NOT [mh Humans] |
| #11 | #9 NOT #10 |

Igaku-Chuo-Zasshi (Search date: 2020/6/26)

| #1 | 人工呼吸器取りはずし/TH or 人工呼吸/AL or 陽圧呼吸/TH or 陽圧呼吸/TA |
| --- | --- |
| #2 | 肺換気/TH or 肺換気/AL |
| #3 | 人工呼吸器/TH or 人工呼吸器/AL |
| #4 | #1 or #2 or #3 |
| #5 | 臨床プロトコール/TH or プロトコル/AL or プロトコール/AL |
| #6 | インタラクティブ換気補助/TH or 換気補助/AL |
| #7 | #5 or #6 |
| #8 | (((RD=ランダム化比較試験,準ランダム化比較試験,比較研究) or (ランダム化比較試験/TH or 準ランダム化比較試験/TH or ランダム化/AL or 無作為化/AL or 比較試験/AL or 臨床試験/AL or プラセボ/AL or 対照/AL or コントロール/AL or 臨床研究/AL)) not (CK=動物 not CK=ヒト)) and (PT=会議録除く) |
| #9 | #4 and #7 and #8 |

1. Flow diagram

**Identification**

2 Studies included in qualitative synthesis

Duplicates

n=1,263

n= 1263

3 Full-text articles assessed for eligibility

3,301 records after duplicates removed

4,564 records identified through database searching

4,564 records identified through database searching

Medline via PubMed n=2,411

CENTRAL n=2,056

Igaku-Chuo-Zasshi n=97

0 additional records identified through other sources

1 Full-text articles excluded, with reasons:

・Wrong outcome (n=1)

2 Studies included in quantitative synthesis (meta-analysis)

**Included**

**Eligibility**

**Screening**

3,298 records excluded

1. Risk of bias


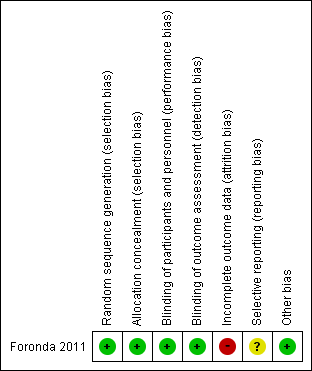
Mortality Duration of mechanical ventilation


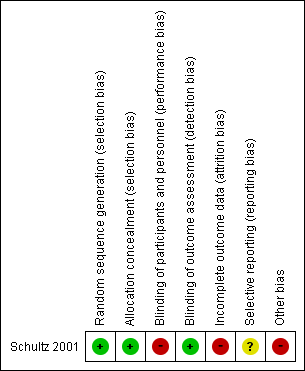


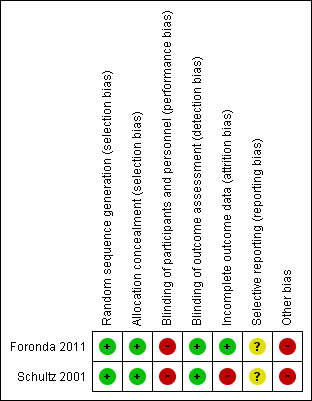
re-intubation Unplanned extubation


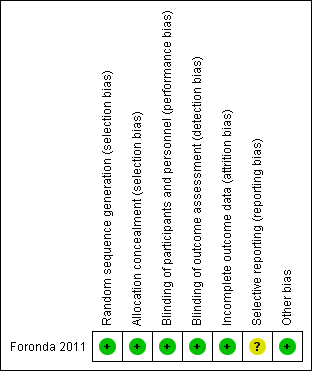


1. Forest plot

Mortality


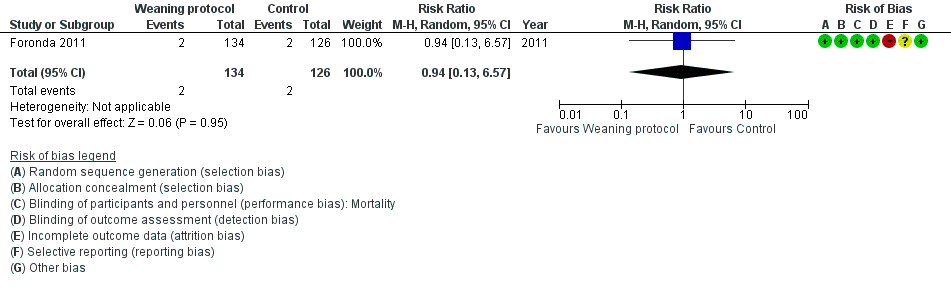


Duration of mechanical ventilation


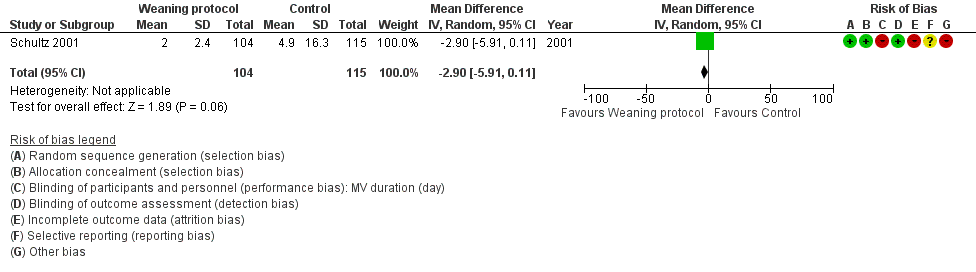


Re-intubation


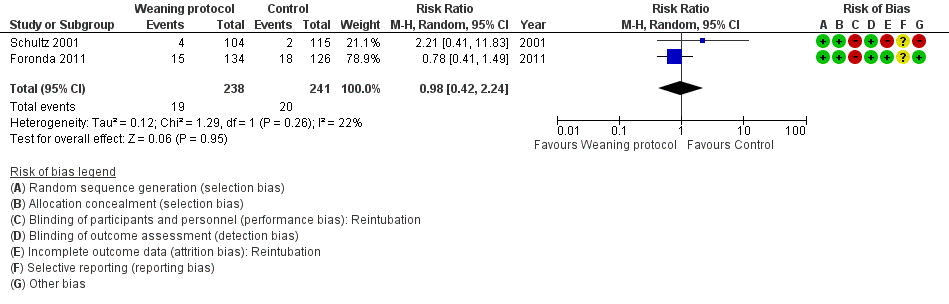


Unplanned extubation


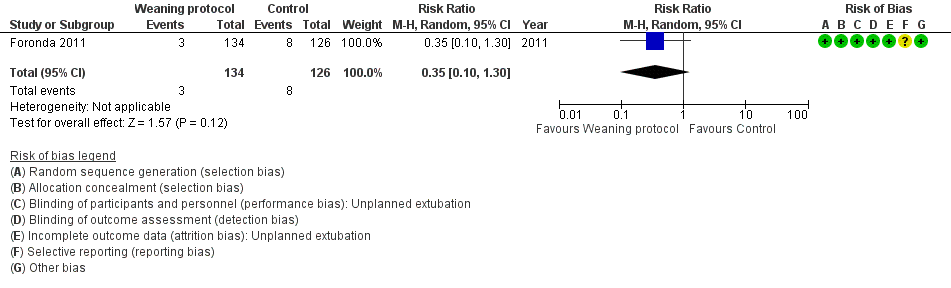


1. Evidence profile

| **Assessment of certainty** | | | | | | | **No. of patients** | | **Efficacy** | | **Certainty of the evidence** | **Importance** |
| --- | --- | --- | --- | --- | --- | --- | --- | --- | --- | --- | --- | --- |
| **No. of studies** | **Study design** | **Risk of bias** | **Inconsistency** | **Indirectness** | **Imprecision** | **Others** | **Weaning protocol** | **Control** | **Relative index (95% CI)** | **Absolute index (95% CI)** |  |  |
| **Mortality** | | | | | | | | | | | | |
| 1 | RCT | Very Serious ^a^ | Not Serious | Not Serious | Very Serious ^b^ | None | 2/134 (1.5%) | 2/126 (1.6%) | **RR 0.94** (0.13 to 6.57) | **-1 per 1000 patients**  (-14 to +88) | ⨁◯◯◯ Very Low | Critical |
| **Developmental prognosis** | | | | | | | | | | | | |
| 0 |  |  |  |  |  |  |  |  |  |  |  | Critical |
| **Duration of mechanical ventilation (days)** | | | | | | | | | | | | |
| 1 | RCT | Very Serious ^c^ | Not Serious | Not Serious | Serious ^d^ | None | 104 | 115 | - | **MD 2.9 days shorter**  (-5.91 to +0.11) | ⨁◯◯◯ Very Low | Critical |
| **Length of hospital stay** | | | | | | | | | | | | |
| 0 |  |  |  |  |  |  |  |  |  |  |  | Critical |
| **Length of ICU stay** | | | | | | | | | | | | |
| 0 |  |  |  |  |  |  |  |  |  |  |  | Important |
| **Tracheostomy** | | | | | | | | | | | | |
| 0 |  |  |  |  |  |  |  |  |  |  |  | Critical |
| **Re-intubation** | | | | | | | | | | | | |
| 2 | RCT | Very Serious ^e^ | Not Serious | Not Serious | Very Serious ^b^ | None | 19/238 (8.0%) | 20/241 (8.3%) | **RR 0.98** (0.42 to 2.24) | **-2 per 1000 patients** (-48 to +103) | ⨁◯◯◯ Very low | Important |
| **Unplanned extubation** | | | | | | | | | | | | |
| 1 | RCT | Not Serious | Not Serious | Not Serious | Very Serious ^b,^ | None | 3/134 (2.2%) | 8/126 (6.3%) | **RR 0.35** (0.10 to 1.30) | **-41 per 1000 patients** (-57 to +19) | ⨁⨁◯◯ Low | Important |

**CI:** Confidence interval; **RR:** Relative risk; **MD:** Mean difference; RCT: Randomized controlled trial; ICU: Intensive care unit

Note: No studies reported the outcomes including developmental prognosis, length of ICU stay, length of hospital stay, or tracheostomy.

#### Explanations

a. Thirty-four patients who died before weaning had deviated from the analysis after randomization, therefore the grade of certainty was lowered by two levels.

b. The sample size of 260 did not meet the optimal information size (OIS), the 95% confidence interval was wide, and the 95% confidence interval included both clinically meaningful thresholds for benefit and harm, making the grade of certainty lowered by two levels.

c. The grade of certainty was lowered by two levels due to the study participants and treatment providers not being blinded, a significant number of patients in the intervention group changing their treatment to that of the control group during the course of the study, and baseline imbalance.

d. The total sample size is 219, which does not meet OIS. Therefore, lacking sufficient detection power, the grade of certainty was lowered by one level.

e. In two studies, study participants and treatment providers were not blinded. Moreover, in one study, a significant number of patients in the intervention group changed their treatment to that of the control group during the course of the study. Therefore, the grade of certainty is lowered by two levels.

1. Evidence-to-Decision table

| QUESTION | |
| --- | --- |
| **PCQ5：** Should a protocol be used when liberating pediatric patients with acute respiratory failure from mechanical ventilators? | |
| **POPULATION:** | Pediatric patients (as defined in the article, 20 years old or younger if not specified) requiring ventilatory management for more than 24 hours with or without ARDS |
| **INTERVENTION:** | Use protocols in liberation from ventilators |
| **COMPARISON:** | Clinician’s discretion |
| **MAIN OUTCOMES:** | Mortality, Developmental prognosis, Duration of mechanical ventilation, Length of hospital stay, Tracheostomy |
| **SETTINGS:** | Emergency department or intensive care unit (ICU) |
| **PERSPECTIVE:** | Individual |
| **BACKGROUND:** | The longer the duration of mechanical ventilation, the more adverse events such as ventilator-associated pneumonia increase. In contrast, premature extubation may increase reintubation and mortality rates. Therefore, it is important to recognize the timing of ventilator liberation early and reliably. Although ventilator liberation is often performed at the discretion of the clinician, protocol-based liberation is considered effective in shortening the duration of mechanical ventilation. Thus, it is necessary to verify whether protocol-based weaning reduces mortality and whether there are any associated adverse events.  In this CQ, we did not limit the target population to patients with ARDS because the respiratory condition improved in patients at the stage of liberation from ventilators. |
| **CONFLICT OF INTERESTS:** | None |

# ASSESSMENT

| Problem Is the problem a priority? | | |
| --- | --- | --- |
| Judgement | Research evidence | Additional considerations |
| ○ No  ○ Probably no  ○ Probably yes  ● Yes  ○ Varies  ○ Do not know | The longer the duration of mechanical ventilation, the more adverse events such as ventilator-associated pneumonia increase. In contrast, premature extubation may increase reintubation and mortality rates. Therefore, it is important to recognize the timing of ventilator liberation early and reliably. Although ventilator liberation is often performed at the discretion of the clinician, protocol-based liberation is considered effective in shortening the duration of mechanical ventilation. Thus, it is an important clinical issue and a high priority to verify whether protocol-based liberation reduces mortality and whether there are any associated adverse events. |  |
| Desirable Effects How substantial are the desirable anticipated effects? | | |
| Judgement | Research evidence | Additional considerations |
| ○ Trivial  ● Small  ○ Moderate  ○ Large  ○ Varies  ○ Do not know | Two randomized controlled trials (RCTs)^1,2^ consistent with PICO were included in the systematic review and used in the meta-analysis.  As a beneficial outcome, the estimate of effect for the 30-day mortality (1 RCT, N=260) was 1 fewer per 1,000 (95% CI: 14 fewer to 88 more) in the intervention group than that in the control group. The estimate of effect for duration of mechanical ventilation (1 RCT, N=219) was the mean difference of 2.9 days shorter (95% CI: 5.91 shorter to 0.11 longer). Note that the outcomes of developmental prognosis and length of hospital stay were not reported. Therefore, we considered the desirable effect of the intervention to be "small.” |  |
| Undesirable Effects How substantial are the undesirable anticipated effects? | | |
| Judgement | Research evidence | Additional considerations |
| ○ Large  ○ Moderate  ○ Small  ● Trivial  ○ Varies  ○ Do not know | There were no reports of tracheostomy, which is a harmful outcome with an importance level of 7 or higher. Therefore, when considered together with the description in the additional considerations, the undesirable effect was deemed "trivial.” | For other adverse outcomes with importance level 6 or less, the estimate of effect for reintubation (2 RCTs^1,2^, N=479) was 2 fewer per 1,000 (95% CI: 48 fewer to 103 more) in the intervention group than that in the control group. The estimate of effect for unplanned extubation (1 RCT, N=260) was 41 fewer per 1,000 persons in the intervention group than that in the control group (95% CI: 57 fewer to 19 more). |
| Certainty of evidence What is the overall certainty of the evidence of effects? | | |
| Judgement | Research evidence | Additional considerations |
| ● Very low  ○ Low  ○ Moderate  ○ High  ○ No included studies | **The relative importance or values of the main outcomes of interest:**   \| **Outcome** \| **Importance** \| **Certainty of the Evidence**  **(GRADE)** \| \| --- \| --- \| --- \| \| \| \| 30-day mortality \| Critical \| ⨁◯◯◯ \| \| Very low \| \| Developmental prognosis* \| Critical \| - \| \| Duration of mechanical ventilation \| Critical \| ⨁◯◯◯ \| \| Very low \| \| Length of hospital stay* \| Critical \| - \| \| Tracheostomy* \| Critical \| - \|   *There were no outcomes reported on developmental prognosis, length of hospital stay, or tracheostomy in the adopted literature.  **Overall certainty of evidence**  The desirable effect was that the intervention would reduce "30-day mortality" by 1 per 1,000 and "duration of mechanical ventilation" by 2.9 days. The certainty of evidence was "very low" for both. Conversely, for the undesirable effect, there were no reports of tracheostomy, a critical outcome. The directions of the desirable and undesirable effects were consistent, and the certainty of evidence for the overall outcome was judged to be "very low.” |  |
| Values Is there important uncertainty about or variability in how much people value the main outcomes? | | |
| Judgement | Research evidence | Additional considerations |
| ○ Important uncertainty or variability  ○ Possibly important uncertainty or variability  ○ Probably no important uncertainty or variability  ● No important uncertainty or variability | “Mortality” is generally a critical outcome, and there is no great uncertainty or diversity of values about this. |  |
| Balance of effects Does the balance between desirable and undesirable effects favor the intervention or the comparison? | | |
| Judgement | Research evidence | Additional considerations |
| ○ Favors the comparison  ○ Probably favors the comparison  ○ Does not favor either the intervention or the comparison  ● Probably favors the intervention  ○ Favors the intervention  ○ Varies  ○ Do not know | **Summary of evidence:**   \| **Outcome** \| **Comparison** \| **Intervention** \| **Absolute difference**  **(95% CI)** \| **Risk ratio (RR)**  **(95% CI)** \| \| --- \| --- \| --- \| --- \| --- \| \| 30-day mortality \| 2/126  (1.6%) \| 2/134  (1.5%) \| 1fewer per 1,000 (14 fewer to 88 more) \| RR 0.94 (0.13 to 6.57) \| \| Developmental prognosis \| - \| - \| - \| - \| \| Duration of mechanical ventilation \| - \| - \| MD 2.9 day shorter (5.91 shorter to 0.11 longer) \| - \| \| Length of hospital stay \| - \| - \| - \| - \| \| Tracheostomy \| - \| - \| - \| - \|   Based on the above, we determined that the balance between desirable and undesirable effects was "probably favors the intervention.” |  |
| Acceptability Is the intervention acceptable to key stakeholders? | | |
| Judgement | Research evidence | Additional considerations |
| ○ No  ○ Probably no  ● Probably yes  ○ Yes  ○ Varies  ○ Do not know | This intervention has been implemented in daily clinical practice and is probably acceptable to patients and their families. |  |
| Feasibility Is the intervention feasible to implement? | | |
| Judgement | Research evidence | Additional considerations |
| ○ No  ○ Probably no  ○ Probably yes  ○ Yes  ● Varies  ○ Do not know | In ICUs that do not specialize in pediatric patients, it is not easy to assess the level of sedation. Therefore, the feasibility of this intervention varies from facility to facility. |  |

# Summary of Judgement

|  | **JUDGMENT** | | | | | | |
| --- | --- | --- | --- | --- | --- | --- | --- |
| **PROBLEM** | No | Probably No | Probably Yes | **Yes** |  | Varies | Do not know |
| **DESIRABLE EFFECTS** | Trivial | **Small** | Moderate | Large |  | Varies | Do not know |
| **UNDESIRABLE EFFECTS** | Large | Moderate | Small | **Trivial** |  | Varies | Do not know |
| **CERTAINTY OF EVIDENCE** | **Very low** | Low | Moderate | High |  |  | No included studies |
| **VALUES** | Important uncertainty or variability | Possibly important uncertainty or variability | Probably no important uncertainty of variability | **No important uncertainty of variability** |  |  |  |
| **BALANCE OF EFFECTS** | Favors the comparison | Probably favors the comparison | Does not favor either the intervention or the comparison | **Probably favors the intervention** | Favors the intervention | Varies | Do not know |
| **ACCEPTABILITY** | No | Probably No | **Probably Yes** | Yes |  | Varies | Do not know |
| **FEASIBILITY** | No | Probably No | Probably Yes | Yes |  | **Varies** | Do not know |

# Type of Recommendation

| Strong recommendation against the intervention | Conditional recommendation against the intervention | Conditional recommendation for either the intervention or the comparison | Conditional recommendation for the intervention | Strong recommendation for the intervention |
| --- | --- | --- | --- | --- |
| ○ | ○ | ○ | ● | ○ |

# CONCLUSION

| Recommendation |
| --- |
| We suggest the use of a protocol when liberating pediatric patients with acute respiratory failure who have been mechanically ventilated for more than 24 hours from ventilators (weak recommendation / very low certainty of evidence: GRADE 2D）.  Supplementary item:  None |
|  |
| Justification |
| **Question:**　Should a protocol be used when liberating pediatric patients with acute respiratory failure from mechanical ventilators?  **Population:**　Pediatric patients (as defined in the article, 20 years old or younger if not specified) requiring ventilatory management for more than 24 hours with or without ARDS  **Intervention:**　Use protocols in libaretion from ventilators  **Comparison:**　Clinician’s discretion  **Main outcomes:**　Mortality, Developmental prognosis, Duration of mechanical ventilation, Length of hospital stay, Tracheostomy, re-intubation, unplanned extubation  **Summary of evidence:**  Two randomized controlled trials (RCTs)^1,2^ consistent with PICO were included in the systematic review and used in the meta-analysis.  As a beneficial outcome, the estimate of effect for the 30-day mortality (1 RCT, N=260) was 1 fewer per 1,000 (95% CI: 14 fewer to 88 more) in the intervention group than that in the control group. The estimate of effect for duration of mechanical ventilation (1 RCT, N=219) was the mean difference of 2.9 days shorter (95% CI: 5.91 shorter to 0.11 longer). Note that the outcomes of developmental prognosis and length of hospital stay were not reported. Therefore, we considered the desirable effect of the intervention to be "small.”  There were no reports of tracheostomy, which is a harmful outcome with an importance level of 7 or higher. Therefore, when considered together with the description in the additional considerations, the undesirable effect was deemed "trivial.” For other adverse outcomes with importance level 6 or less, the estimate of effect for reintubation (2 RCTs^1,2^, N=479) was two fewer per 1,000 (95% CI: 48 fewer to 103 more) in the intervention group than that in the control group. The estimate of effect for unplanned extubation (1 RCT, N=260) was 41 fewer per 1,000 persons in the intervention group than that in the control group (95% CI: 57 fewer to 19 more).  Based on the above, we determined that the balance between desirable and undesirable effects was "probably favors the intervention.”  **Certainty of evidence:**  The desirable effect was that the intervention would reduce "30-day mortality" by 1 per 1,000 and "duration of mechanical ventilation" by 2.9 days. The certainty of evidence was "very low" for both. Conversely, for the undesirable effect, there were no reports of tracheostomy, a critical outcome. The directions of the desirable and undesirable effects were consistent, and the certainty of evidence for the overall outcome was judged to be "very low.”  **Balance of effects, Acceptability, Feasibility**  In ICUs that do not specialize in pediatric patients, it is not easy to assess the level of sedation. Therefore, the feasibility of this intervention varies from facility to facility.  **Panel meeting:**  In the pre-vote, using the modified Delphi method, "we propose that the protocol be used in ventilator weaning of pediatric patients with respiratory failure (grade 2D, weak recommendation/certainty of evidence “very low" was agreed upon with a median score of 8 and a disagreement index of 0.2920.  At the panel meeting, it was agreed that the following additional considerations, which were originally included in the recommendation column, should be deleted. Therefore, supplementary items were only included in the reason column and re-voted. Using a modified Delphi method, " We suggest the use of a protocol when liberating pediatric patients with acute respiratory failure who have been mechanically ventilated for more than 24 hours (weak recommendation / very low certainty of evidence: GRADE 2D）." was agreed upon with a median score of 8 and a disagreement index of 0.1316.  **Additional considerations**  Populations were pediatric patients requiring ventilatory management for more than 24 hours with or without ARDS (We thought that the liberation process would not be significantly different unless the patient had chronic lung disease).  The protocols adopted were as follows, however, they vary from study to study and cannot be universalized.  Foronda2011：Patients who meet the criteria for daily evaluation are given a spontaneous breathing trial (SBT) (PEEP 5 cmH2O, PS 10 cmH_2_O, FIO2 unchanged, 2 hours) and extubate if there are no signs of intolerance. Discontinue SBT if patient has signs of intolerance, such as increase in respiratory rate or heart rate by 20% or more, signs of increased respiratory workload (use of respiratory support muscles or seesaw breathing), changes in level of consciousness, hypotension below the 5th percentile of blood pressure for age, arterial oxygen saturation 90%, or PaCO_2_ >50 mmHg (or an increase of 10 mmHg per hour in patients with chronic lung disease), and reassess in 24 hours. If SBT is cleared, proceed to extubation.  Schultz 2001：Once the respiratory conditions meet certain criteria, the nurse or physical therapist will change the ventilation conditions according to the protocol every hour, decreasing PS by 2 cmH_2_O and PEEP by 1 cmH_2_O, stopping the liberation once the patient experiences respiratory distress. If the patient is still breathing steadily when PS<5cmH_2_O and PEEP<5cmH_2_O are reached, liberation should be terminated. |

| Subgroup considerations |
| --- |
| It is difficult to use protocols in ventilator liberation for patients with low baseline SpO_2_, such as those with cyanotic heart disease. |
| Implementation considerations |
| Level of consciousness and dyspnea are difficult to assess in children.  In addition to ARDS, we also included children who required ventilatory management for more than 24 h since we believe that the liberation process would not be significantly different unless chronic lung disease was present. |

| Monitoring and evaluation |
| --- |
| There is a need to investigate the percentage of facilities where ventilator liberation is already protocolized. After publishing this recommendation, it is necessary to monitor the prognosis changes in facilities that implement protocols. |
| Research priorities |
| Studies comparing protocols for ventilator liberation are expected. |

References

1. Foronda FK, Troster EJ, Farias JA, et al. The impact of daily evaluation and spontaneous breathing test on the duration of pediatric mechanical ventilation: a randomized controlled trial. *Crit Care Med*. 2011; 39: 2526-33.
2. Schultz TR, Lin RJ, Watzman HM, et al. Weaning children from mechanical ventilation: a prospective randomized trial of protocol-directed versus physician-directed weaning. *Respir care*. 2001; 46: 772-82.

**PCQ6 Should HFOV be used for pediatric patients with moderate to severe ARDS?**

1.Search strategy

MEDLINE via Pubmed (Search date: 2021/3/23)

| #1 | Respiratory Distress Syndrome, Adult [MH] OR acute respiratory distress syndrome[tiab] OR adult respiratory distress syndrome[tiab] OR respiratory distress syndrome[tiab] OR ARDS[tiab] |
| --- | --- |
| #2 | Acute Lung Injury [MH] OR acute lung injury[tiab] OR acute lung injuries[tiab] OR lung injury[tiab] OR lung injuries[tiab] OR ALI [tiab] OR shock lung[tiab] |
| #3 | respiratory insufficiency[MH] OR respiratory insufficiency[tiab] OR acute respiratory failure[tiab] OR respiratory depression[tiab] OR ventilatory depression[tiab] |
| #4 | #1 OR #2 OR #3 |
| #5 | High frequency ventilation[MH] OR High-Frequency Oscillation Ventilation [tiab] OR High-Frequency Oscillation Ventilations [tiab] OR High-Frequency Positive Pressure Ventilation [tiab] OR High-Frequency Positive Pressure Ventilations [tiab] OR HFO [tiab] OR HFOV [tiab] |
| #6 | Respiration, Artificial[MH] OR Artificial respiration[tiab] OR Mechanical ventilation[tiab] OR Mechanical ventilations[tiab] |
| #7 | #5 AND #6 |
| #8 | Clinical trial[pt] OR trial[ti] OR randomized controlled trial[pt] OR(controlled clinical trial[pt] OR randomized[tiab]) OR placebo[tiab] OR clinical trials as topic[MH] OR randomly[tiab] |
| #9 | Animals[MH] NOT Humans[MH] |
| #10 | #8NOT#9 |
| #11 | #4 AND #7 AND #10 |

CENTRAL (Search date: 2021/3/23)

| #1 | MeSH descriptor: [Respiratory Distress Syndrome, Adult] explode all trees |
| --- | --- |
| #2 | ("acute respiratory distress syndrome"):ti,ab,kw |
| #3 | ("adult respiratory distress syndrome"):ti,ab,kw |
| #4 | ("respiratory distress syndrome"):ti,ab,kw |
| #5 | (ARDS):ti,ab,kw |
| #6 | {OR #1-#5} |
| #7 | MeSH descriptor: [Acute Lung Injury] explode all trees |
| #8 | ("acute lung injury"):ti,ab,kw |
| #9 | ("acute lung injuries"):ti,ab,kw |
| #10 | (ALI):ti,ab,kw |
| #11 | ("shock lung"):ti,ab,kw |
| #12 | (" lung injury "):ti,ab,kw |
| #13 | (" lung injuries "):ti,ab,kw |
| #14 | {OR #7-#13} |
| #15 | MeSH descriptor: [Respiratory Insufficiency] explode all trees |
| #16 | ("Respiratory Insufficiency"):ti,ab,kw |
| #17 | ("acute respiratory failure"):ti,ab,kw |
| #18 | ("respiratory depression"):ti,ab,kw |
| #19 | ("ventilatory depression"):ti,ab,kw |
| #20 | {OR #15-#20} |
| #21 | MeSH descriptor: [High-Frequency Ventilation] explode all trees |
| #22 | ("high frequency ventilation"):ti,ab,kw |
| #23 | ("high frequency oscillatory ventilation"):ti,ab,kw |
| #24 | ("high frequency oscillation"):ti,ab,kw |
| #25 | ("high frequency positive pressure ventilation"):ti,ab,kw |
| #26 | (HFOV):ti,ab,kw |
| #27 | (HFO):ti,ab,kw |
| #28 | {OR #21-#27} |
| #29 | MeSH descriptor: [Respiration, Artificial] explode all trees |
| #30 | ("artificial respiration"):ti,ab,kw |
| #31 | ("mechanical ventilation"):ti,ab,kw |
| #32 | ("mechanical ventilatory support"):ti,ab,kw |
| #33 | ("mechanical ventilations"):ti,ab,kw |
| #34 | {OR #29-#33} |
| #35 | #6 OR #14 OR #20 |
| #36 | #28 AND #34 |
| #37 | #35 AND #36 |

Igaku-Chuo-Zasshi (Search date: 2021/3/23)

| #1 | 呼吸窮迫症候群-急性/TH |
| --- | --- |
| #2 | 呼吸促迫症候群/AL |
| #3 | 呼吸窮迫症候群/AL |
| #4 | 急性呼吸窮迫症候群/AL |
| #5 | 成人呼吸窮迫症候群/AL |
| #6 | acute respiratory distress syndrome/AL |
| #7 | ARDS/AL |
| #8 | PT=会議録除く |
| #9 | #1or#2or#3or#4or#5or#6or#7and#8 |
| #10 | 急性肺損傷/TH |
| #11 | 急性肺損傷/AL |
| #12 | 急性肺障害/AL |
| #14 | acute lung injuries/AL |
| #15 | acute lung injury/AL |
| #16 | PT=会議録除く |
| #17 | #10or#11or#12or#13or#14or#15and#16 |
| #18 | 高頻度換気/TH |
| #19 | 高頻度振動換気/AL |
| #20 | high frequency oscillatory ventilation |
| #21 | HFOV/AL |
| #22 | PT=会議録除く |
| #23 | #18or#19or#20or#21and#22 |
| #24 | RD=ランダム化比較試験 |
| #25 | CK=ヒト |
| #26 | CK=動物 |
| #27 | CK=成人(19～44),中年(45～64),高齢者(65～) |
| #28 | #25and#27not#26 |
| #29 | #9or#17or#23and#24and#28 |

1. Flow diagram

**Identification**

4 Studies included in qualitative synthesis

Duplicates

n=220

n= 1263

10 Full-text articles assessed for eligibility

265 records after duplicates removed

485 records identified through database searching

485 records identified through database searching

Medline via PubMed n=265

CENTRAL n=190

Igaku-Chuo-Zasshi n=30

0 additional records identified through other sources

6 Full-text articles excluded, with reasons:

・Wrong study design (n=4)

・Wrong publication type (n=2)

4 Studies included in quantitative synthesis (meta-analysis)

**Included**

**Eligibility**

**Screening**

255 records excluded

1. Risk of bias

Mortality Ventilator-free days (VFD)


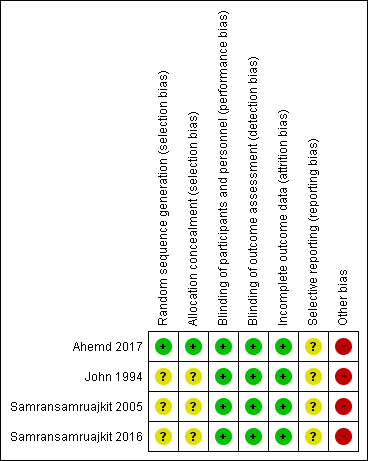

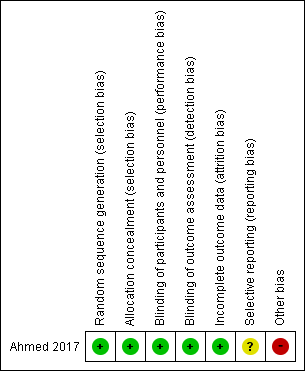


Length of ICU stay Reduction of barotrauma


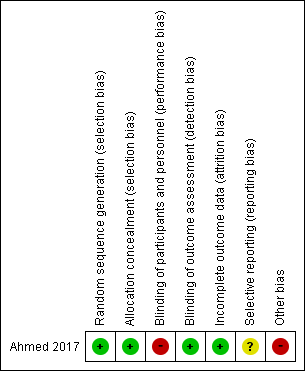

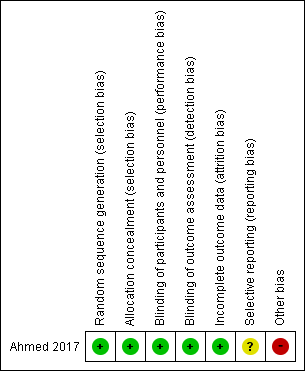


PF ratio improvement rate OI improvement rate


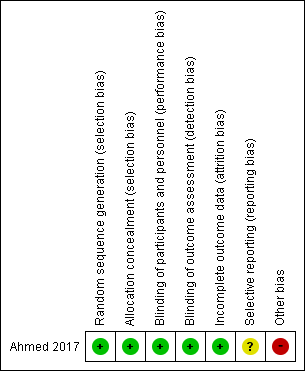

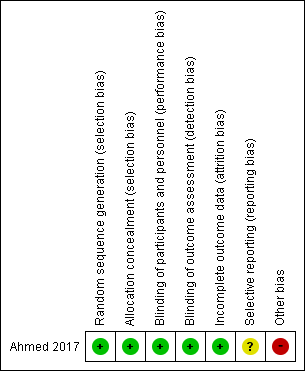


Hemodynamic deterioration


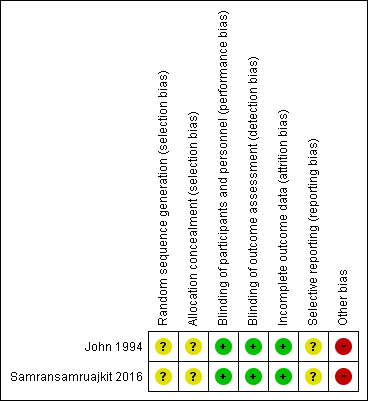


1. Forest plot

Mortality


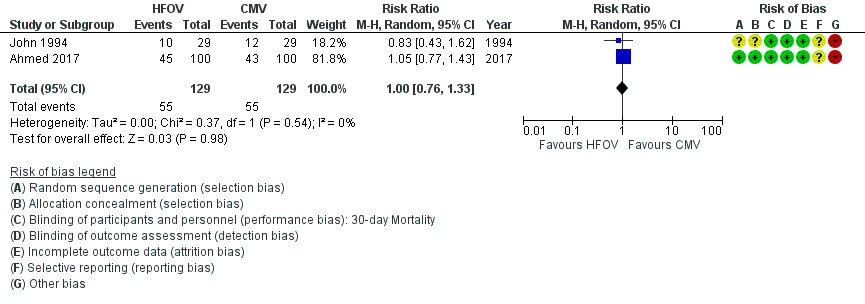


Ventilator-free days (VFD)


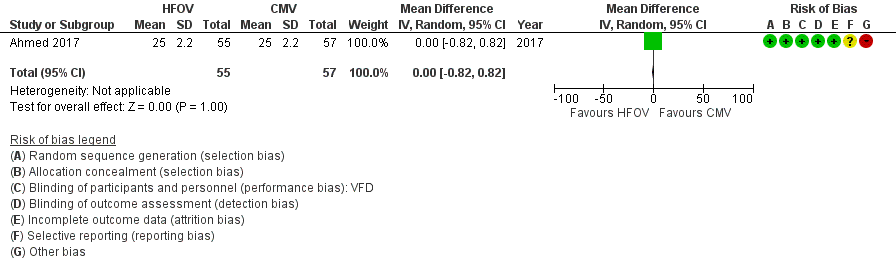


Length of ICU stay


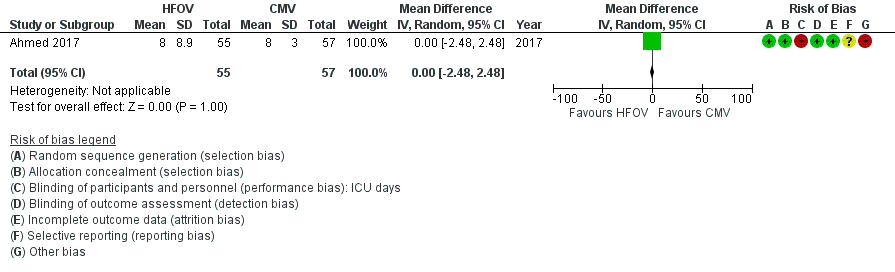


Reduction of barotrauma


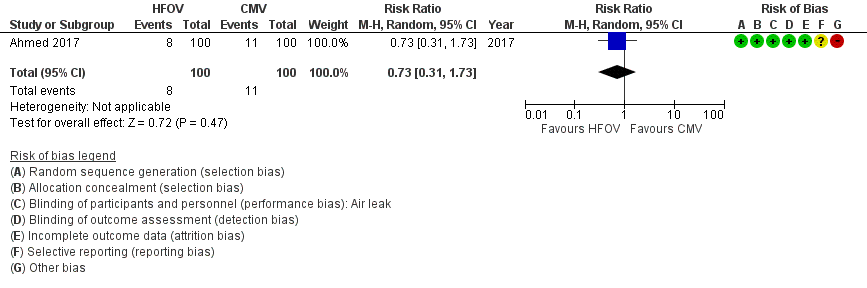


PF ratio improvement rate


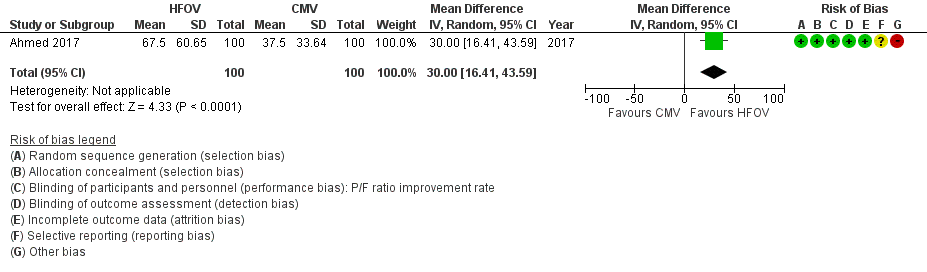


OI improvement rate


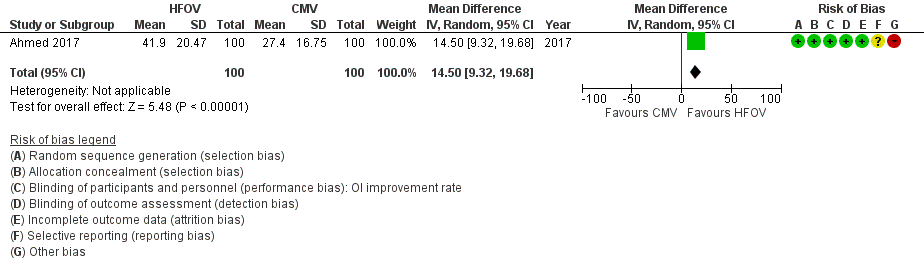


Hemodynamic deterioration


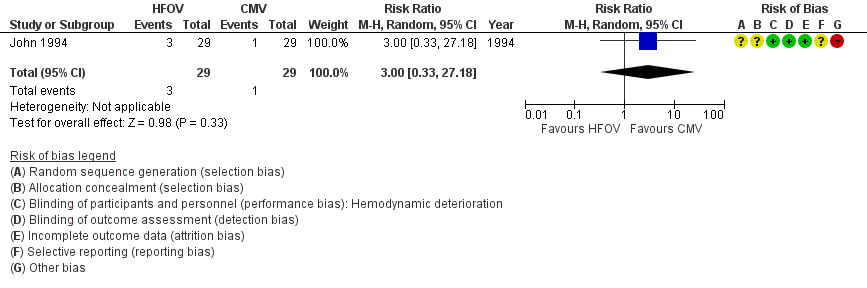


1. Evidence profile

| **Assessment of certainty** | | | | | | | **No. of patients** | | **Efficacy** | | **Certainty of the evidence** | **Importance** |
| --- | --- | --- | --- | --- | --- | --- | --- | --- | --- | --- | --- | --- |
| **No. of studies** | **Study design** | **Risk of bias** | **Inconsistency** | **Indirectness** | **Imprecision** | **Others** | **HFO** | **CMV** | **Relative index (95% CI)** | **Absolute index (95% CI)** |  |  |
| **Mortality （Including 30-day and unspecified mortality）** | | | | | | | | | | | | |
| 4 | RCT | Serious ^a^ | Not Serious | Serious ^b^ | Very Serious ^c^ | None | 58/145 (40.0%) | 61/147 (41.5%) | **RR 0.97** (0.74 to 1.28) | **-12 per 1000 patients** (-108 to +116) | ⨁◯◯◯ Very Low | Critical |
| **Developmental prognosis** | | | | | | | | | | | | |
| 0 |  |  |  |  |  |  |  |  |  |  |  | Critical |
| **Ventilator-free days (VFD)** | | | | | | | | | | | | |
| 1 | RCT | Serious ^d^ | Not Serious | Not Serious | Serious ^e^ | None | 55 | 57 | - | **MD 0 day shorter**  (-0.82 to +0.82) | ⨁⨁◯◯ Low | Critical |
| **Length of hospital stay** | | | | | | | | | | | | |
| 0 |  |  |  |  |  |  |  |  |  |  |  | Critical |
| **Length of ICU stay** | | | | | | | | | | | | |
| 1 | RCT | Serious ^f^ | Not Serious | Not Serious | Serious ^e^ | None | 55 | 57 | - | **MD 0 day shorter**  (-2.48 to +2.48) | ⨁⨁◯◯ Low | Important |
| **Reduction of Barotrauma** | | | | | | | | | | | | |
| 1 | RCT | Serious ^d^ | Not Serious | Not Serious | Very Serious ^g^ | None | 8/100 (8.0%) | 11/100 (11.0%) | **RR 0.73** (0.31 to 1.73) | **-30 per 1000 patients** (-76 to +80) | ⨁⨁◯◯ Low | Important |
| **PF ratio improvement rate after 24 h of treatment (%)** | | | | | | | | | | | | |
| 1 | RCT | Serious ^d^ | Not Serious | Not Serious | Serious ^h^ | None | 100 | 100 | - | **MD 30% higher**  (+16.41 to +43.59) | ⨁⨁◯◯ Low | Important |
| **OI improvement rate after 24 h of treatment (%)** | | | | | | | | | | | | |
| 1 | RCT | Serious ^d^ | Not serious | Not serious | Serious ^h^ | None | 100 | 100 | - | **MD 14.5% higher** (+9.32 to +19.68) | ⨁⨁◯◯ Low | Important |
| **Hemodynamic deterioration** | | | | | | | | | | | | |
| 2 | RCT | Serious ^i^ | Not serious | Serious ^j^ | Very serious ^k^ | None | 3/38 (7.9%) | 1/38 (2.6%) | **RR 3.00** (0.33 to 27.18) | **+53 per 1000 patients** (-18 to +689) | ⨁◯◯◯ Very Low | Critical |

**CI:** Confidence interval; **RR:** Relative risk; **MD:** mean difference; **RCT**: randomized controlled trial

Note: No studies reported the outcomes including developmental prognosis or length of hospital stay*.*

#### Explanations

a. Both studies are affected by crossover. In one study, allocation concealment is unclear. In one study, there is baseline imbalance.

b. In one study, the target population included patients with pneumothorax who did not have ARDS.

c. The sample size of 292 did not meet the optimal information size (OIS). Additionally, the 95% confidence interval is wide and spans both the threshold for choosing treatment and the threshold for not choosing treatment. Based on the above, the grade of certainty is lowered by two levels.

d. Baseline imbalance is observed. The outcome is affected by crossover.

e. The sample size of 112 did not meet the OIS. Therefore, lacking sufficient detection power, the grade of certainty was lowered by one level.

f. Treatment providers are not blinded and ICU exit criteria are not specified. Baseline imbalance is observed. The outcome is affected by crossover.

g. The sample size of 200 did not meet OIS. Additionally, the 95% confidence interval is wide and spans both the threshold for choosing treatment and the threshold for not choosing treatment. Based on the above, the grade of certainty was lowered by two levels.

h. The sample size of 200 did not meet OIS. Therefore, lacking sufficient detection power, the grade of certainty is lowered by one level.

i. The concealment of the allocation is unclear.

j. The target population included patients with pneumothorax who did not have ARDS.

k. The sample size of 76 did not meet OIS. Additionally, the 95% confidence interval was wide and spanned both the threshold for choosing treatment and the threshold for not choosing treatment. Based on the above, the grade of certainty was lowered by two levels.

1. Evidence-to-Decision table

| **QUESTION** | |
| --- | --- |
| **PCQ6：** Should high frequency oscillatory ventilation (HFOV) be used for pediatric patients with moderate to severe ARDS? | |
| **POPULATION:** | Pediatric patients (as defined in the article, 20 years old or younger if not specified) on ventilators for moderate to severe ARDS (as defined in the article) |
| **INTERVENTION:** | HFOV |
| **COMPARISON:** | Conventional mechanical ventilation |
| **MAIN OUTCOMES:** | Mortality, Developmental prognosis, Ventilator-free days (VFD), Length of hospital stay, Hemodynamic deterioration |
| **SETTINGS:** | Emergency department or intensive care unit (ICU) |
| **PERSPECTIVE:** | Individual |
| **BACKGROUND:** | HFOV is a mode of ventilation that allows lung recruitment by limiting tidal volume. HFOV has not yet been a common method for pediatric ventilation management. Previous guidelines (2016) stated that in adults, mortality and VFD were not significantly different from conventional ventilation, although the adverse event, pressure injury, was increased.  Evaluating the benefits and harms of the planned use of HFOV in children with moderate to severe ARDS is an important clinical issue. |
| **CONFLICT OF INTERESTS:** | None |

**ASSESSMENT**

| **Problem**  Is the problem a priority? | | |
| --- | --- | --- |
| **Judgement** | **Research evidence** | **Additional considerations** |
| ○ No  ○ Probably no  ○ Probably yes  ● Yes  ○ Varies  ○ Do not know | HFOV is a mode of ventilation that allows lung recruitment by limiting tidal volume. HFOV is not yet common in pediatric ventilatory management. HFOV has no longer been used, as in the previous guideline (2016) which states that mortality and duration of non-ventilation were not significantly different from conventional ventilatory management in adults, although the adverse event, pressure injury, was increased. On the other hand, in the neonatal field, HFOV is used as a mode of ventilation during respiratory failure. Therefore, this issue is of high priority. |  |
| **Desirable Effects**  How substantial are the desirable anticipated effects? | | |
| **Judgement** | **Research evidence** | **Additional considerations** |
| ● Trivial  ○ Small  ○ Moderate  ○ Large  ○ Varies  ○ Do not know | Four randomized controlled trials (RCTs)^1-4^ consistent with PICO were included in the systematic review and used in the meta-analysis.  As a beneficial outcome, the estimate of effect for mortality (4 RCTs, N=292) was 12 fewer per 1,000 (95% CI: 108 fewer to 116 more) in the intervention group than that in the control group. The estimate of effect for VFD (1 RCT, N=102) was the mean difference of 0 days shorter (95% CI: 0.82 shorter to 0.82 longer). The outcomes of developmental prognosis and length of hospital stay were not reported. Therefore, the desirable effect of the intervention was judged to be "trivial." |  |
| **Undesirable Effects**  How substantial are the undesirable anticipated effects? | | |
| **Judgement** | **Research evidence** | **Additional considerations** |
| ○ Large  ○ Moderate  ● Small  ○ Trivial  ○ Varies  ○ Do not know | As a harmful outcome, the estimate of the effect of hemodynamic deterioration (2 RCTs, N=76) was 53 more per 1,000 (95% CI: 18 fewer to 689 more) in the intervention group than in the control group. This undesirable effect was considered "small." |  |
| **Certainty of evidence**  What is the overall certainty of the evidence of effects? | | |
| **Judgement** | **Research evidence** | **Additional considerations** |
| ● Very low  ○ Low  ○ Moderate  ○ High  ○ No included studies | **The relative importance or values of the main outcomes of interest:**   \| **Outcome** \| **Importance** \| **Certainty of the Evidence**  **(GRADE)** \| \| --- \| --- \| --- \| \| \| \| Mortality* \| Critical \| ⨁◯◯◯ \| \| Very low \| \| Developmental prognosis** \| Critical \| - \| \| VFD \| Critical \| ⨁⨁◯◯ \| \| Low \| \| Length of hospital stay** \| Critical \| - \| \| Hemodynamic deterioration \| Critical \| ⨁◯◯◯ \| \| Very low \|   * Mortality is a combination of 30-day mortality and mortality for which the time period is not stated  **There were no outcome reports on developmental prognosis and length of hospital stay in the accepted literature.  **Overall certainty of evidence**  The desirable effect was that the intervention would reduce “mortality” by 12 per 1,000 and “VFD” by 0 days. The certainty of the evidence was "very low" and "low." On the other hand, the undesirable effect was that the intervention increased "hemodynamic deterioration" by 53 per 1,000. The certainty of the evidence was "very low." Therefore, the direction of the desirable and undesirable effects was not consistent, and the certainty of evidence for the overall outcome was judged to be "very low," since the lowest certainty of evidence was adopted. |  |
| **Values**  Is there important uncertainty about or variability in how much people value the main outcomes? | | |
| **Judgement** | **Research evidence** | **Additional considerations** |
| ○ Important uncertainty or variability  ○ Possibly important uncertainty or variability  ○ Probably no important uncertainty or variability  ● No important uncertainty or variability | “Mortality” is generally a critical outcome, and there is no great uncertainty or diversity of values about this. |  |
| **Balance of effects**  Does the balance between desirable and undesirable effects favor the intervention or the comparison? | | |
| **Judgement** | **Research evidence** | **Additional considerations** |
| ○ Favors the comparison  ● Probably favors the comparison  ○ Does not favor either the intervention or the comparison  ○ Probably favors the intervention  ○ Favors the intervention  ○ Varies  ○ Do not know | **Summary of evidence:**   \| **Outcome** \| **Comparison** \| **Intervention** \| **Absolute difference**  **(95% CI)** \| **Risk ratio (RR)**  **(95% CI)** \| \| --- \| --- \| --- \| --- \| --- \| \| Mortality \| 61/147 (41.5%) \| 58/145 (40.0%) \| 12 fewer per 1,000 (108 fewer to 116 more) \| RR 0.97 (0.74 to 1.28) \| \| Developmental prognosis \| - \| - \| - \| - \| \| VFD \| - \| - \| MD 0 day shorter (0.82 shorter to 0.82 longer) \| - \| \| Length of hospital stay \| - \| - \| - \| - \| \| Hemodynamic deterioration \| 1/38 (2.6%) \| 3/38 (7.9%) \| 53 more per 1,000 (18 fewer to 689 more) \| RR 3.00 (0.33 to 27.18) \|   Based on the above, we judged that the balance between desirable and undesirable effects was "probably favors the comparison.” |  |
| **Acceptability**  Is the intervention acceptable to key stakeholders? | | |
| **Judgement** | **Research evidence** | **Additional considerations** |
| ○ No  ○ Probably no  ● Probably yes  ○ Yes  ○ Varies  ○ Do not know | If HFOV is therapeutically necessary, it may be acceptable to patients and their families. |  |
| **Feasibility**  Is the intervention feasible to implement? | | |
| **Judgement** | **Research evidence** | **Additional considerations** |
| ○ No  ● Probably no  ○ Probably yes  ○ Yes  ○ Varies  ○ Do not know | In facilities that have ventilators dedicated to HFOV, HFOV is considered to be routinely used, so HFOV can be selected; however, it is presumed that there are few such facilities. In addition, it is difficult to perform HFOV in facilities that do not have dedicated ventilators. |  |

**Summary of Judgement**

|  | **JUDGMENT** | | | | | | |
| --- | --- | --- | --- | --- | --- | --- | --- |
| **PROBLEM** | No | Probably No | Probably Yes | **Yes** |  | Varies | Do not know |
| **DESIRABLE EFFECTS** | **Trivial** | Small | Moderate | Large |  | Varies | Do not know |
| **UNDESIRABLE EFFECTS** | Large | Moderate | **Small** | Trivial |  | Varies | Do not know |
| **CERTAINTY OF EVIDENCE** | **Very low** | Low | Moderate | High |  |  | No included studies |
| **VALUES** | Important uncertainty or variability | Possibly important uncertainty or variability | Probably no important uncertainty of variability | **No important uncertainty of variability** |  |  |  |
| **BALANCE OF EFFECTS** | Favors the comparison | **Probably favors the comparison** | Does not favor either the intervention or the comparison | Probably favors the intervention | Favors the intervention | Varies | Do not know |
| **ACCEPTABILITY** | No | Probably No | **Probably Yes** | Yes |  | Varies | Do not know |
| **FEASIBILITY** | No | **Probably No** | Probably Yes | Yes |  | Varies | Do not know |

**Type of Recommendation**

| Strong recommendation against the intervention | Conditional recommendation against the intervention | Conditional recommendation for either the intervention or the comparison | Conditional recommendation for the intervention | Strong recommendation for the intervention |
| --- | --- | --- | --- | --- |
| ○ | ● | ○ | ○ | ○ |

**CONCLUSION**

| **Recommendation** |
| --- |
| We suggest against implementing HFOV for pediatric patients with ARDS.  (weak recommendation / very low certainty of evidence: GRADE 2D）  Supplementary item:  This recommendation does not reject the implementation of this procedure in facilities that already have an HFOV-specialized ventilator and are familiar with its use. |
|  |
| **Justification** |
| **Question:** Should HFOV be used for pediatric patients with moderate to severe ARDS?  **Population:**　 Pediatric patients (as defined in the article, 20 years old or younger if not specified) on ventilators for moderate to severe ARDS (as defined in the article)  **Intervention:**　HFOV  **Comparison:**　 Conventional mechanical ventilation  **Main outcomes:**　 Mortality, Developmental prognosis, Ventilator-free days (VFD), Length of hospital stay, Hemodynamic deterioration  **Summary of evidence:**  Four randomized controlled trials (RCTs)^1-4^ consistent with PICO were included in the systematic review and used in the meta-analysis.  As a beneficial outcome, the estimate of effect for mortality (4 RCTs, N=292) was 12 fewer per 1,000 (95% CI: 108 fewer to 116 more) in the intervention group than that in the control group. The estimate of effect for VFD (1 RCT, N=102) was the mean difference of 0 days shorter (95% CI: 0.82 shorter to 0.82 longer). The outcomes of developmental prognosis and length of hospital stay were not reported. Therefore, the desirable effect of the intervention was judged to be "trivial."  As a harmful outcome, the estimate of the effect of hemodynamic deterioration (2 RCTs, N=76) was 53 more per 1,000 (95% CI: 18 fewer to 689 more) in the intervention group than in the control group. This undesirable effect was considered "small."  **Certainty of evidence:**  The desirable effect was that the intervention would reduce “mortality” by 12 per 1,000 and “VFD” by 0 days. The certainty of the evidence was "very low" and "low." On the other hand, the undesirable effect was that the intervention increased "hemodynamic deterioration" by 53 per 1,000. The certainty of the evidence was "very low." Therefore, the direction of the desirable and undesirable effects was not consistent, and the certainty of evidence for the overall outcome was judged to be "very low," since the lowest certainty of evidence was adopted.  **Balance of effects, Acceptability, Feasibility**  The harms of this intervention probably outweigh the benefits. In addition, it cannot be achieved without a dedicated HFOV ventilator, and purchasing an expensive dedicated HFOV ventilator and educating physicians on how to use it in facilities that do not normally use HFOV will increase the burden on the field.  **Panel meeting:**  At the panel meeting, the recommendation "We suggest against implementing HFOV for pediatric patients with ARDS. (weak recommendation / very low certainty of evidence: GRADE 2D） " was agreed upon with a median score of 9.0 and a disagreement index of 0.00, using the modified Delphi method.  **Additional considerations**  This does not preclude implementation in facilities that already have dedicated HFOV ventilators and are familiar with their use. |

| **Subgroup considerations** |
| --- |
| There may be room for further study, such as subgroup analysis by age. |
| **Implementation considerations** |
| If sedatives or muscle relaxants need to be used or increased, their detrimental effects should also be considered.  In addition to the usual monitoring, blood gas should be collected and evaluated periodically, as it is difficult to assess ventilation using capnography. |

| **Monitoring and evaluation** |
| --- |
| One of the clinical challenges in implementing the recommendation is the need to collect more information, such as whether clinicians have a ventilator dedicated to HFOV and whether they use HFOV on a regular basis. In addition, even after the guidelines are published, we need to monitor the implementation of the guidelines through questionnaires to determine if there are any other clinical issues. |
| **Research priorities** |
| Few studies have been incorporated into meta-analyses, and higher quality studies are expected in the future. On that occasion, we need to clarify what kind of patients to include (consider factors such as patient age and disease severity), when to introduce HFOV (example: either before or after prone), and which method to compare with other methods (extracorporeal membrane oxygenation [ECMO] or airway pressure-release ventilation [APRV]). We also need to ensure the technical level of HFOV. |

References

1. El-Nawawy A, Moustafa A, Heshmat H, et al. High frequency oscillatory ventilation versus conventional mechanical ventilation in pediatric acute respiratory distress syndrome: A randomized controlled study. *Turk J Pediatr*. 2017; 59: 130-43.
2. Arnold JH, Hanson JH, Toro-Figuero LO, et al. Prospective, randomized comparison of high-frequency oscillatory ventilation and conventional mechanical ventilation in pediatric respiratory failure. *Crit Care Med*. 1994; 22:1530-9.
3. Samransamruajkit R, Rassameehirun C, Pongsanon K, et al. A comparison of clinical efficacy between high frequency oscillatory ventilation and conventional ventilation with lung volume recruitment in pediatric acute respiratory distress syndrome: A randomized controlled trial. *Indian J Crit Care Med*. 2016; 20: 72-7.
4. Samransamruajkit R, Prapphal N, Deelodegenavong J et al. Plasma soluble intercellular adhesion molecule-1 (sICAM-1) in pediatric ARDS during high frequency oscillatory ventilation: a predictor of mortality. *Asian Pac J Allergy Immunol*. 2005; 23: 181-8.

**PCQ7 Should APRV be used for pediatric patients with ARDS?**

1.Search strategy

MEDLINE via Pubmed (Search date: 2020/7/8）

| #1 | respiratory distress syndrome, adult[mh] OR acute respiratory distress syndrom*[tiab] OR respiratory insufficiency[mh] OR acute lung injury[mh] OR acute lung injur*[tiab] OR acute respiratory failure*[tiab] OR ALI[tiab] OR ARDS[tiab] |
| --- | --- |
| #2 | Respiration, Artificial[mh] OR artificilal respiration*[tiab] OR Pulmonary Ventilation[mh] OR pulmonary ventilat*[tiab] OR Ventilators, Mechanical[mh] OR mechanical ventilat*[tiab] OR positive-pressure respiration*[tiab] OR positive pressure ventilat*[tiab] |
| #3 | pressure control*[tiab] OR PCV[tiab] OR volume control*[tiab] OR VCV[tiab] |
| #4 | airway pressure release ventilat*[tiab] OR APRV[tiab] |
| #5 | synchronized intermittent mandatory ventilation[tiab] OR intermittent mandatory ventilation[tiab] OR SIMV[tiab] OR IMV[tiab] |
| #6 | PSV[tiab] OR pressure support[tiab] |
| #7 | #2 OR #3 OR #4 OR #5 OR #6 |
| #8 | (randomized controlled trial [pt] OR controlled clinical trial [pt] OR randomized [tiab] OR placebo [tiab] OR clinical trials as topic [mesh: noexp] OR randomly [tiab] OR trial [ti]) NOT (animals[mh] NOT humans[mh]) |
| #9 | #1 AND #7 AND #8 |

CENTRAL (Search date: 2020/7/8）

| #1 | [mh "respiratory distress syndrome, adult"] OR "acute respiratory distress syndrom":ti,ab OR [mh "respiratory insufficiency"] OR [mh "acute lung injury"] OR "acute lung injury":ti,ab OR "acute respiratory failure":ti,ab OR ALI:ti,ab OR ARDS:ti,ab |
| --- | --- |
| #2 | [mh "Respiration, Artificial"] OR "artificilal respiration":ti,ab OR [mh "Pulmonary Ventilation"] OR "pulmonary ventilation":ti,ab OR [mh "Ventilators, Mechanical"] OR "mechanical ventilation":ti,ab OR "positive-pressure respiration":ti,ab OR "positive pressure ventilation":ti,ab |
| #3 | "pressure control":ti,ab OR PCV:ti,ab OR "volume control":ti,ab OR VCV:ti,ab |
| #4 | "airway pressure release ventilation":ti,ab OR APRV:ti,ab |
| #5 | "synchronized intermittent mandatory ventilation":ti,ab OR "intermittent mandatory ventilation":ti,ab OR SIMV:ti,ab OR IMV:ti,ab |
| #6 | PSV:ti,ab OR "pressure support":ti,ab |
| #7 | {OR #2-#6} |
| #8 | #1 AND #7 |
| #9 | [mh animals] NOT [mh humans] |
| #10 | #8 NOT #9 |

Igaku-Chuo-Zasshi (Search date: 2020/7/8）

| #1 | 呼吸窮迫症候群-急性/TH or 呼吸促迫症候群/AL or ARDS/AL or "acute respiratory distress syndrome"/AL or 急性肺損傷/TH or 急性肺損傷/AL or 急性肺障害/AL or 急性肺傷害/AL or "acute lung Injury"/AL or 呼吸不全/TH or 呼吸不全/AL or 呼吸機能不全/AL |
| --- | --- |
| #2 | 人工呼吸/TH or 人工呼吸/AL or 人工換気/AL or レスピレータ/AL or ベンチレータ/AL or 機械換気/AL or 機械的換気/AL or 人工換気/AL or 調節呼吸/AL or 調節換気/AL or 陽圧呼吸/AL or 陽圧換気/AL |
| #3 | 従圧/AL or 圧規定/AL or 従量/AL or 量規定/AL or PCV/AL or VCV/AL |
| #4 | APRV/AL or 気道内圧開放式/AL or 気道内圧解放式/AL or "Airway Pressure Release Ventilation"/AL |
| #5 | 間欠性強制換気/TH or 同期式間欠的強制/AL or 間欠的強制/AL or SIMV/AL or IMV/AL or "synchronized intermittent mandatory"/AL or "intermittent mandatory"/AL |
| #6 | プレッシャーサポート/AL or PSV/AL or "pressure support"/AL or "pressure-support"/AL |
| #7 | #2 or #3 or #4 or #5 or #6 |
| #8 | (((RD=ランダム化比較試験,準ランダム化比較試験,比較研究) or (ランダム化比較試験/TH or 準ランダム化比較試験/TH or ランダム化/AL or 無作為化/AL or 比較試験/AL or 臨床試験/AL or プラセボ/AL or 対照/AL or コントロール/AL or 臨床研究/AL)) not (CK=動物 not CK=ヒト)) and (PT=会議録除く) |
| #9 | #1 and #7 and #8 |

1. Flow diagram

0 additional records identified through other sources

5,933 records identified through database searching

Medline via PubMed (n=2,824)

CENTRAL (n=2,259)

Igaku-Chuo-Zasshi (n=550)

**Identification**

**Screening**

**Eligibility**

**Included**

87 records assessing eligibility of children

4,278 records excluded

Duplicates

n=1,568

5,933 records identified through database searching

4,365 records after duplicates removed

54 records excluded

32 Full-text articles excluded, with reasons:

・Wrong language (n=5)

・Wrong publication type (n=14)

・Wrong population (n=13)

33 Full-text articles assessed for eligibility

1 Studies included in qualitative synthesis

1 Studies included in quantitative synthesis (meta-analysis)

1. Risk of bias

28-day Mortality Developmental prognosis


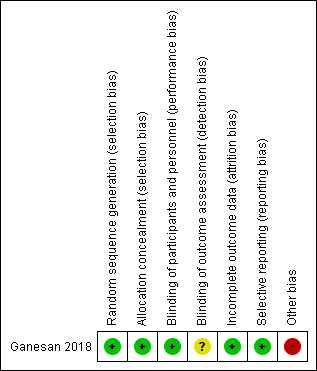

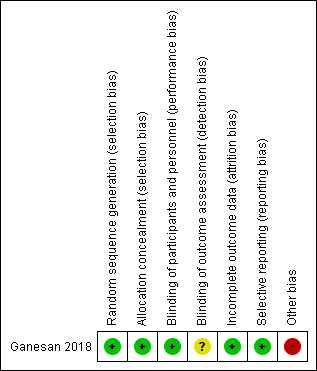


Ventilator-free days (VFD) Length of hospital stay


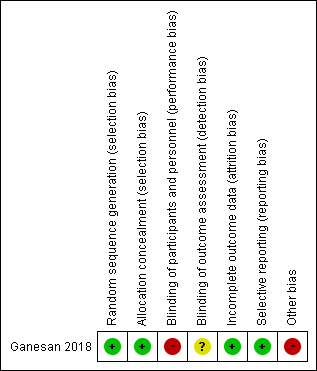

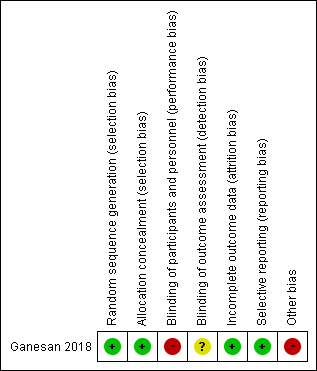


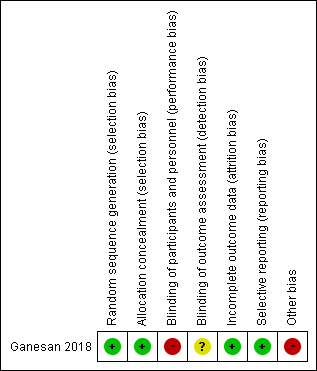
Hemodynamic deterioration Barotrauma


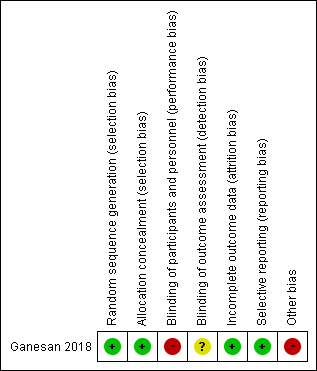


Oxygenation


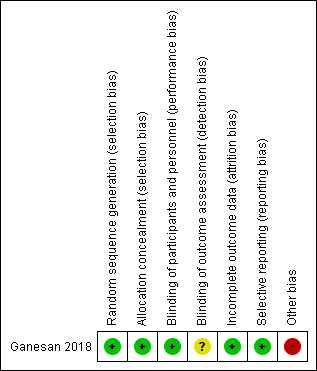


1. Forest plot

28-day mortality


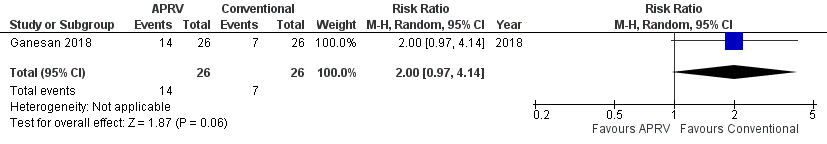


Developmental prognosis


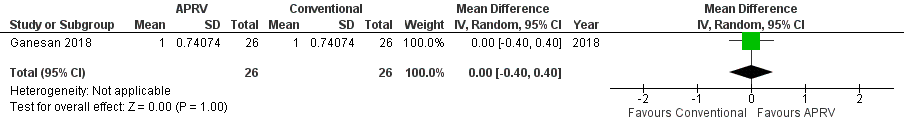


Ventilator-free days (VFD)


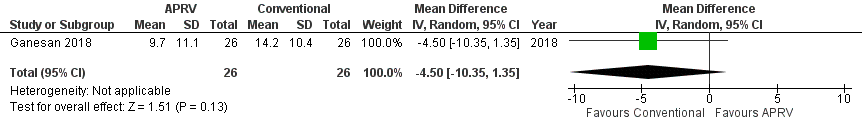


Length of ICU stay


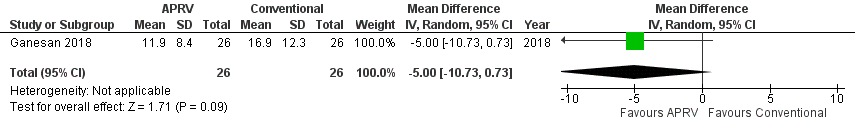


Hemodynamic deterioration


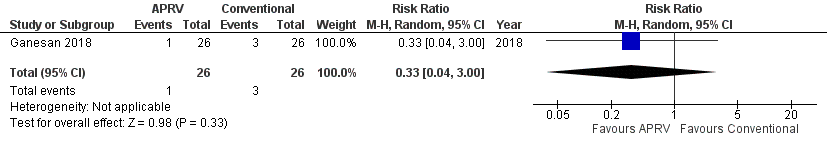


Barotrauma


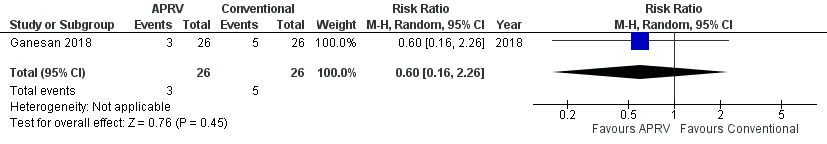


Oxygenation


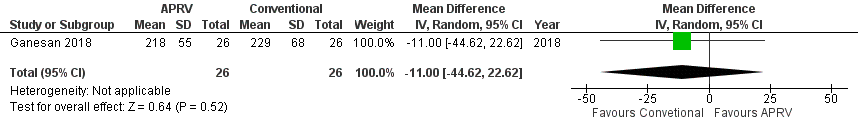


1. Evidence profile

| **Assessment of certainty** | | | | | | | **No. of patients** | | **Efficacy** | | **Certainty of the Evidence** | **Importance** |
| --- | --- | --- | --- | --- | --- | --- | --- | --- | --- | --- | --- | --- |
| **No. of studies** | **Study design** | **Risk of bias** | **Inconsistency** | **Indirectness** | **Imprecision** | **Others** | **APRV** | **placebo** | **Relative index** **(95% CI)** | **Relative index** **(95% CI)** |  |  |
| **28-day mortality** | | | | | | | | | | | | |
| 1 | RCT | Serious^a^ | Not serious | Not serious | Serious^b^ | None | 14/26 (53.8%) | 7/26 (26.9%) | **RR 2.00** (0.97 to 4.14) | **+269 per 1000 patients** (-8 to +845) | ⨁⨁◯◯ Low | Critical |
| **Developmental prognosis (PCPC^c^ at 180 days)** | | | | | | | | | | | | |
| 1 | RCT | Serious^a^ | Not serious | Not serious | Serious^d^ | None | 26 | 26 | - | **MD ±0** (-0.4 to +0.4) | ⨁⨁◯◯ Low | Critical |
| **Ventilator-free days (VFD)** | | | | | | | | | | | | |
| 1 | RCT | Serious^e^ | Not serious | Not serious | Serious^d^ | None | 26 | 26 | - | **MD 4.5 days shorter**  (-10.35 to +1.35) | ⨁⨁◯◯ Low | Critical |
| **Length of hospital stay** | | | | | | | | | | | | |
| 1 | RCT | Serious^e^ | Not serious | Not serious | Serious^d^ | None | 26 | 26 | - | **MD 5 days shorter**  (-10.73 to +0.73) | ⨁⨁◯◯ Low | Critical |
| **Hemodynamic deterioration** | | | | | | | | | | | | |
| 1 | RCT | Serious^e^ | Not serious | Not serious | Very serious^f^ | None | 1/26 (3.8%) | 3/26 (11.5%) | **RR 0.33** (0.04 to 3.00) | **-77 per 1000 patients** (-111 to +231) | ⨁◯◯◯ Very low | Critical |
| **Barotrauma** | | | | | | | | | | | | |
| 1 | RCT | Serious^e^ | Not serious | Not serious | Very serious^f^ | None | 3/26 (11.5%) | 5/26 (19.2%) | **RR 0.60** (0.16 to 2.26) | **-77 per 1000 patients** (-162 to +242) | ⨁◯◯◯ Very low | Important |
| **Oxygenation (P/F ratio after 24 h)** | | | | | | | | | | | | |
| 1 | RCT | Serious^g^ | Not serious | Not serious | Serious^d^ | None | 26 | 26 | - | **MD 11 lower**  (-44.62 to +22.62) | ⨁⨁◯◯ Low | Important |

CI: confidence interval; RR: risk ratio; RCT: randomized controlled trial; MD: mean difference

**Explanations**

a. One of the included studies had a high risk of other bias (the baseline P/F ratio differed significantly between the two groups) and was downgraded by one level.

b. Total sample size of 52 patients does not meet the optimal information size (OIS). The 95% confidence interval of the outcome estimate straddles the treatment threshold for harm. Therefore, the inaccuracy was judged to be serious and the grade was downgraded by one level.

c. PCPC, Pediatric Cerebral Performance Category Scale.

d. The total sample size of 52 patients does not meet the optimal information size (OIS), and the direction of the recommendation may differ within the 95% confidence interval of the estimated effect on the outcome. Therefore, the inaccuracy was judged to be serious and the grade was downgraded by one level.

e. One of the included studies had a high risk of other bias (large difference in baseline P/F ratio between the two groups), and blinding of participants and treatment providers was a high risk of bias; therefore, it was downgraded by one level.

f. Two-stage grade down because the total sample size of 52 participants did not meet the OIS, and the 95% confidence interval for the estimated effect of the outcome straddled the treatment threshold for benefit and harm and was considered very serious.

g. One study that was inclusion had a high risk of other bias (baseline P/F ratio differed significantly between the two groups), and the P/F ratio at 24 hours post-intervention was judged to denote a serious risk of bias due to the outcome and was one step down.

1. Evidence-to-Decision table

| SQUESTION | |
| --- | --- |
| **PCQ7：** Should APRV be used for pediatric patients with ARDS? | |
| **POPULATION:** | Pediatric patients (as defined in the article, 20 years old or younger if not specified) on ventilators for ARDS (as defined in the article) |
| **INTERVENTION:** | APRV |
| **COMPARISON:** | Volume control ventilation (VCV) or Pressure control ventilation (PCV) |
| **MAIN OUTCOMES:** | Mortality, Developmental prognosis, Ventilator-free days (VFD), Length of hospital stay, Hemodynamic deterioration |
| **SETTINGS:** | Emergency department or intensive care unit (ICU) |
| **PERSPECTIVE:** | Individual |
| **BACKGROUND:** | Ventilatory management of patients with acute respiratory distress syndrome (ARDS) is as important as the treatment of the primary disease. Airway pressure-release ventilation (APRV) is often used in ARDS because it can maintain high airway pressure, although its efficacy in pediatric patients is not clear. |
| **CONFLICT OF INTERESTS:** | None |

# ASSESSMENT

| Problem Is the problem a priority? | | |
| --- | --- | --- |
| Judgement | Research evidence | Additional considerations |
| ○ No  ○ Probably no  ○ Probably yes  ● Yes  ○ Varies  ○ Do not know | Ventilatory management of patients with ARDS is as important as the treatment of the primary disease. APRV is used in some cases of ARDS because it can maintain high airway pressure, although its efficacy in pediatric patients with ARDS is not clear. Therefore, this issue is of high priority. |  |
| Desirable Effects How substantial are the desirable anticipated effects? | | |
| Judgement | Research evidence | Additional considerations |
| ○ Trivial  ● Small  ○ Moderate  ○ Large  ○ Varies  ○ Do not know | One randomized controlled trial (RCT)^1^ consistent with PICO was included in the systematic review and used in the meta-analysis.  As beneficial outcomes, an estimate of effect for the developmental prognosis (PCPC at 180 days) (1 RCT^1^, N=52) was the mean difference of 0 (95% CI: 0.4 worse to 0.4 better) and for the length of hospital stay, it was the mean difference of 5 days shorter (10.73 shorter to 0.73 longer) in the intervention group compared with that in the comparison group. The 28-day mortality and VFD were assumed to have desirable effects. However, they were treated as undesirable effects because mortality increased and VFD was shortened, as discussed below. Based on the above, the desirable effect of the intervention was considered "small." |  |
| Undesirable Effects How substantial are the undesirable anticipated effects? | | |
| Judgement | Research evidence | Additional considerations |
| ○ Large  ● Moderate  ○ Small  ○ Trivial  ○ Varies  ○ Do not know | As a harmful outcome, the estimate of the effect for the 28-day mortality (one RCT^1^, N=52) was 269 more per 1,000 (95% CI: 8 fewer to 845 more); for VFD (one RCT^1^, N=52), it has a mean difference of 4.5 days shorter (95% CI: 10.35 fewer to 1.35 more); and for hemodynamic deterioration (one RCT^1^, N=52), it was 77 fewer per 1,000 (95% CI: 111 fewer to 231 more) in the intervention group than that in the comparison group. Therefore, the undesirable effect was considered "moderate." |  |
| Certainty of evidence What is the overall certainty of the evidence of effects? | | |
| Judgement | Research evidence | Additional considerations |
| ● Very low  ○ Low  ○ Moderate  ○ High  ○ No included studies | **The relative importance or values of the main outcomes of interest:**   \| **Outcome** \| **Importance** \| **Certainty of the Evidence**  **(GRADE)** \| \| --- \| --- \| --- \| \| 28-day mortality \| Critical \| ⨁⨁◯◯ Low \| \| Developmental prognosis \| Critical \| ⨁⨁◯◯ Low \| \| VFD \| Critical \| ⨁⨁◯◯ Low \| \| Length of hospital stay \| Critical \| ⨁⨁◯◯ Low \| \| Hemodynamic deterioration \| Critical \| ⨁◯◯◯ Very low \|   **Overall certainty of evidence**  The desirable effect results were that the intervention resulted in a mean difference of 0 change in "developmental prognosis (PCPC at 180 days)" and the mean difference of 5 days shortening in "length of hospital stay." The certainty of the evidence was "low" and "low,” respectively. In contrast, the undesirable effect results were that the intervention resulted in an increase in "28-day mortality" by 269 per 1,000, a reduction in "VFD" by the mean difference of 4.5 days, and a decrease in "hemodynamic deterioration" by 77 per 1,000. The certainty of the evidence was "low," "low," and "very low,” respectively. The direction of the desirable and undesirable effects was not consistent, and the certainty of evidence for the entire outcome was judged to be "very low," since the certainty of the outcome with the lowest certainty was adopted. |  |
| Values Is there important uncertainty about or variability in how much people value the main outcomes? | | |
| Judgement | Research evidence | Additional considerations |
| ○ Important uncertainty or variability  ○ Possibly important uncertainty or variability  ○ Probably no important uncertainty or variability  ● No important uncertainty or variability | “Mortality” is generally a critical outcome, and there is no great uncertainty or diversity of values about this. |  |
| Balance of effects Does the balance between desirable and undesirable effects favor the intervention or the comparison? | | |
| Judgement | Research evidence | Additional considerations |
| ○ Favors the comparison  ● Probably favors the comparison  ○ Does not favor either the intervention or the comparison  ○ Probably favors the intervention  ○ Favors the intervention  ○ Varies  ○ Do not know | **Summary of evidence:**   \| **Outcome** \| **Control** \| **Intervention** \| **Absolute difference**  **(95% CI)** \| **Risk ratio (RR)**  **(95% CI)** \| \| --- \| --- \| --- \| --- \| --- \| \| 28-day mortality \| 7/26  (26.9%) \| 14/26  (53.8%) \| 269 more per 1,000  (8 fewer to 845 more) \| RR 2.00  (0.97 to 4.14) \| \| Developmental prognosis  (PCPC at 180 days) \| - \| - \| MD 0  (0.4 worse to 0.4 better) \| - \| \| VFD \| - \| - \| MD 4.5 day shorter  (10.35 shorter to 1.35 longer) \| - \| \| Length of hospital stay \| - \| - \| MD 5 day shorter  (10.73 shorter to 0.73 longer) \| - \| \| Hemodynamic deterioration \| 3/26  (11.5%) \| 1/26  (3.8%) \| 77 fewer per 1,000  (111 fewer to 231 more) \| RR 0.33  (0.04 to 3.00) \|   Based on the above, we consider that the balance between desirable and undesirable effects was "probably favors the comparison.” |  |
| Acceptability Is the intervention acceptable to key stakeholders? | | |
| Judgement | Research evidence | Additional considerations |
| ○ No  ○ Probably no  ○ Probably yes  ○ Yes  ● Varies  ○ Do not know | Acceptability can vary, given that some healthcare providers are concerned about the possibility of lung injury from excessive effort of spontaneous breathing. |  |
| Feasibility Is the intervention feasible to implement? | | |
| Judgement | Research evidence | Additional considerations |
| ○ No  ○ Probably no  ○ Probably yes  ○ Yes  ● Varies  ○ Do not know | Feasibility can vary since APRV is only possible if an APRV dedicated ventilator is available. |  |

# Summary of Judgement

|  | **JUDGEMENT** | | | | | | |
| --- | --- | --- | --- | --- | --- | --- | --- |
| **PROBLEM** | No | Probably no | Probably yes | **Yes** |  | Varies | Do not know |
| **DESIRABLE EFFECTS** | Trivial | **Small** | Moderate | Large |  | Varies | Do not know |
| **UNDESIRABLE EFFECTS** | Large | **Moderate** | Small | Trivial |  | Varies | Do not know |
| **CERTAINTY OF EVIDENCE** | **Very low** | Low | Moderate | High |  |  | No included studies |
| **VALUES** | Important uncertainty or variability | Possibly important uncertainty or   variability | Probably no important uncertainty or variability | **No important uncertainty or variability** |  |  |  |
| **BALANCE OF EFFECTS** | Favors the comparison | **Probably favors the comparison** | Does not favor either the intervention or the comparison | Probably favors the intervention | Favors the intervention | Varies | Do not know |
| **ACCEPTABILITY** | No | Probably no | Probably yes | Yes |  | **Varies** | Do not know |
| **FEASIBILITY** | No | Probably no | Probably yes | Yes |  | **Varies** | Do not know |

# Type of Recommendation

| Strong recommendation against the intervention | Conditional recommendation against the intervention | Conditional recommendation for either the intervention or the comparison | Conditional recommendation for the intervention | Strong recommendation for the intervention |
| --- | --- | --- | --- | --- |
| ○ | ● | ○ | ○ | ○ |

# CONCLUSION

| Recommendation |
| --- |
| We suggest against implementing APRV as a ventilation mode in pediatric patients with ARDS (weak recommendation / very low certainty of evidence：GRADE 2D）.  Supplementary item:  This recommendation does not reject the implementation of APRV in facilities familiar with its use. |
|  |
| Justification |
| **Question:** Should APRV be used for pediatric patients with ARDS?  **Population:** Pediatric patients (as defined in the article, 20 years old or younger if not specified) on ventilators for ARDS (as defined in the article)  **Intervention:** APRV  **Comparison:** Volume control ventilation (VCV) or Pressure control ventilation (PCV)  **Outcome:** 28 days mortality, Developmental prognosis, Ventilator-free days (VFD), Length of hospital stay, Hemodynamic deterioration  **Summary of evidence:**  A systematic review found one randomized controlled trial consistent with PICO^1^ (N=52).  As desirable outcomes, the estimated effect on developmental prognosis (PCPC at 180 days) was a mean change of 0 (95% CI: 0.4 worse-0.4 better), the ventilator-free days (VFD) was reduced by mean 4.5 days (10.35 days shorter-1.35 days longer), and the length of hospital stay was reduced by mean 5 days (10.73 days shorter-0.73 days longer). Although 28-day mortality was assumed to be a desirable effect, it was treated as an undesirable effect because of the increase in mortality, as described below. In summary, considering the developmental prognosis, non-ventilator duration, and hospital stay as desirable effects, we judged the desirable effect of the intervention to be "small".  As undesirable effects, the estimated effect on 28-day mortality was a risk difference of 269 more /1000 patients (95% CI: 8 fewer - 845 more) for the intervention compared to the comparison, and hemodynamic deterioration was a risk difference of 77 fewer /1000 patients (95% CI: 111 fewer - 231 more). Therefore, the undesirable effect was judged to be "moderate".  Therefore, we concluded that the balance between desirable and undesirable effects was "probably favors comparison".  **Quality of evidence:**  The desirable effect results were that the intervention resulted in a mean difference of 0 change in "developmental prognosis (PCPC at 180 days)" and the mean difference of 5 days shortening in "length of hospital stay." The certainty of the evidence was "low" and "low,” respectively. In contrast, the undesirable effect results were that the intervention resulted in an increase in "28-day mortality" by 269 per 1,000, a reduction in "VFD" by the mean difference of 4.5 days, and a decrease in "hemodynamic deterioration" by 77 per 1,000. The certainty of the evidence was "low," "low," and "very low,” respectively. The direction of the desirable and undesirable effects was not consistent, and the certainty of evidence for the entire outcome was judged to be "very low," since the certainty of the outcome with the lowest certainty was adopted.  **Balance of effects, Acceptability, Feasibility:**  The balance between desired and undesired effects probably favors comparison, even though it may have a negative effect on mortality and benefit other outcomes. For this reason, interventions may not be acceptable and are not likely to be feasible given the specific ventilator requirements.  **Panel meeting:**  At the panel meeting, the recommendation "We suggest against implementing APRV as a ventilation mode in pediatric patients with ARDS (weak recommendation / very low certainty of evidence：GRADE 2D）." was agreed upon with a median of 8.0 and a disagreement index of 0.2920, using the modified Delphi method.  **Additional considerations:**  This does not preclude implementation in facilities that already have dedicated APRV ventilators and are familiar with their use. |

| Subgroup considerations |
| --- |
| The effect of APRV may be different in patients with high lung recruitability. On the other hand, patients with shock, congenital heart disease (especially single ventricle physiology, right heart bypass), and elevated intracranial pressure require more careful monitoring. |
| Implementation considerations |
| The safe management of uncooperative pediatric patients while maintaining adequate spontaneous breathing requires analgesic and sedation techniques. Intrinsic PEEP may occur in children due to greater airway resistance and greater chest compliance, which vary greatly with age. In addition, some facilities may incur new purchase costs. |

| Monitoring and evaluation |
| --- |
| None |
| Research priorities |
| The effect of APRV may depend on the lung capacity that can be recruited and the management of strong spontaneous breathing. Future studies should be well-designed and carefully monitored. |

Reference

1. Lalgudi Ganesan S, Jayashree M, Chandra Singhi S, Bansal A. Airway Pressure Release Ventilation in Pediatric Acute Respiratory Distress Syndrome. A Randomized Controlled Trial. American journal of respiratory and critical care medicine 2018;198:1199-207.

**PCQ8 How should target SpO_2_ values be set in pediatric patients with ARDS?**

1.Search strategy

MEDLINE via Pubmed (Search date: 2020/7/2)

| #1 | Respiratory distress syndrome,adult[mh] OR adult respiratory distress syndrom*[tiab] OR acute respiratory distress syndrom*[tiab] OR ARDS[tiab] |
| --- | --- |
| #2 | acute lung injury[mh] OR acute lung injur*[tiab] OR ALI[tiab] |
| #3 | acute lung fail*[tiab] OR acute lung depression[tiab] |
| #4 | Critical Illness[mh] OR critically ill*[tiab] OR critical ill*[tiab] |
| #5 | Respiratory insufficiency[mh] OR Respiratory insufficiency[tiab] |
| #6 | #1 OR #2 OR #3 OR #4 OR #5 |
| #7 | Oxygen Inhalation Therapy[mh] OR oxygen therap*[tiab] |
| #8 | Oximetry[mh] OR Oximetr*[tiab] |
| #9 | P/F[tiab] OR PaO2/FIO2[tiab] OR Oxygen Saturation[tiab] OR SpO2[tiab] |
| #10 | #7 OR #8 OR #9 |
| #11 | #6 AND #10 |
| #12 | randomized controlled trial [pt] OR controlled clinical trial [pt] OR randomized [tiab] OR placebo [tiab] OR clinical trials as topic [mesh: noexp] OR randomly [tiab] OR trial [ti] |
| #13 | #12 NOT (animals[mh] NOT humans[mh]) |
| #14 | #11 AND #13 |

CENTRAL (Search date: 2020/7/2)

| #1 | [mh "Respiratory distress syndrome,adult"] OR "adult respiratory distress syndrom":ti,ab OR "acute respiratory distress syndrom":ti,ab OR ARDS:ti,ab |
| --- | --- |
| #2 | [mh "acute lung injury"] OR "acute lung injury":ti,ab OR ALI:ti,ab |
| #3 | "acute lung failure":ti,ab OR "acute lung depression":ti,ab |
| #4 | [mh "Critical Illness"] OR "critically ill":ti,ab OR "critical ill":ti,ab OR "critically illness":ti,ab OR "critical illness":ti,ab |
| #5 | [mh "Respiratory insufficiency"] OR "Respiratory insufficiency":ti,ab |
| #6 | {OR #1-#5} |
| #7 | [mh "Oxygen Inhalation Therapy"] OR "oxygen therapy":ti,ab |
| #8 | [mh Oximetry] OR Oximetry:ti,ab |
| #9 | "P/F":ti,ab OR "PaO2/FIO2" OR "Oxygen Saturation":ti,ab OR SpO2:ti,ab |
| #10 | {OR #7-#9} |
| #11 | #6 AND #10 |
| #12 | [mh Animals] NOT [mh Humans] |
| #13 | #11 NOT #12 |

Igaku-Chuo-Zasshi (Search date: 2020/7/2)

| #1 | 呼吸窮迫症候群‐急性/TH or 呼吸窮迫症候群/TA or 急性呼吸窮迫症候群/TA or ARDS/TA or RDS/TA |
| --- | --- |
| #2 | 急性肺損傷/TH or 急性肺損傷/TA or 急性肺障害/TA or 急性肺傷害/TA |
| #3 | 危篤/TH or 危篤/TA or 重症/TA |
| #4 | 呼吸不全/TH or 呼吸不全/TA |
| #5 | #1 or #2 or #3 or #4 |
| #6 | 酸素吸入療法/TH or 酸素吸入療法/TA |
| #7 | 酸素飽和度測定/TH or 動脈血酸素飽和度測定/TH or 酸素飽和度/TA or SpO2/TA |
| #8 | パルスオキシメーター/TA |
| #9 | "P/F"/TA or "PaO2/FIO2"/TA |
| #10 | #6 or #7 or #8 or #9 |
| #11 | #5 and #10 |
| #12 | (ランダム化比較試験/TH or 準ランダム化比較試験/TH or ランダム化/AL or 無作為化/AL or 比較試験/AL or 臨床試験/AL or プラセボ/AL or 対照/AL or コントロール/AL or 臨床研究/AL) and (PT=会議録除く) |
| #13 | #11 and #12 |

1. Flow diagram

**Identification**

0 Studies included in qualitative synthesis

5

Full-text articles assessed for eligibility

2,016 records after duplicates removed

2,408 records identified through database searching

2,408 records identified through database searching

Medline via PubMed (n=1227)

CENTRAL (n=959)

Igaku-Chuo-Zasshi (n=222)

0 additional records identified through other sources

0 Studies included in quantitative synthesis (meta-analysis)

5 Full-text articles excluded, with reasons:

・Wrong study design (n=2)

・Wrong population (n=2)

・Wrong Publication type (n=1)

Duplicates

n=392

2,011 records excluded

**Included**

**Eligibility**

**Screening**

1. Risk of bias

Not applicable

1. Forest plot

Not applicable

1. Evidence profile

Not applicable

1. Evidence-to-Decision table

| **QUESTION** | |
| --- | --- |
| **PCQ8：** How should target SpO_2_ values be set in pediatric patients with ARDS? | |
| **POPULATION:** | Pediatric patients (as defined in the article, 20 years old or younger if not specified) on ventilators for ARDS (as defined in the article) |
| **INTERVENTION:** | Low SpO_2_ management |
| **COMPARISON:** | Normal SpO_2_ management |
| **MAIN OUTCOMES:** | Mortality, Developmental prognosis, Ventilator-free days (VFD), Length of hospital stay, Severe hypoxemia |
| **SETTINGS:** | Emergency department or intensive care unit (ICU) |
| **PERSPECTIVE:** | Individual |
| **BACKGROUND:** | Management with high SpO_2_ has been reported to cause adverse events that may contribute to pulmonary fibrosis. However, it is unclear whether management with low SpO_2_ improves survival or worsens prognosis due to adverse events. To date, there have been no interventional studies or systematic reviews on oxygen saturation goals in pediatric patients with acute respiratory distress syndrome (ARDS). Therefore, the efficacy of low SpO_2_ management and the certainty of the evidence are unknown. |
| **CONFLICT OF INTERESTS:** | None |

**ASSESSMENT**

| **Problem**  Is the problem a priority? | | |
| --- | --- | --- |
| **Judgement** | **Research evidence** | **Additional considerations** |
| ○ No  ○ Probably no  ○ Probably yes  ● Yes  ○ Varies  ○ Do not know | Adverse events contributing to pulmonary fibrosis have been reported with the management of hyperbaric oxygen therapy. To date, there have been no interventional studies or systematic reviews in pediatric ARDS patients to determine whether low SpO_2_ management contributes to improved survival and whether low SpO_2_ management is associated with (or acceptable for) adverse events. The efficacy of low SpO_2_ management in pediatric ARDS patients and the certainty of the evidence are unknown. Therefore, this issue is of high priority. |  |
| **Desirable Effects**  How substantial are the desirable anticipated effects? | | |
| **Judgement** | **Research evidence** | **Additional considerations** |
| ○ Trivial  ○ Small  ○ Moderate  ○ Large  ○ Varies  ● Do not know | Integrated into the “Certainty of evidence”. |  |
| **Undesirable Effects**  How substantial are the undesirable anticipated effects? | | |
| **Judgement** | **Research evidence** | **Additional considerations** |
| ○ Large  ○ Moderate  ○ Small  ○ Trivial  ○ Varies  ● Do not know | Integrated into the “Certainty of evidence”. |  |
| **Certainty of evidence**  What is the overall certainty of the evidence of effects? | | |
| **Judgement** | **Research evidence** | **Additional considerations** |
| ○ Very low  ○ Low  ○ Moderate  ○ High  ● No included studies | Management that permits hypoxemia is expected to have benefits such as avoiding high inspiratory oxygen concentrations during ventilatory management and reducing ventilator-induced lung injury (VILI/VALI). On the other hand, there is concern that low SpO_2_ management may narrow the safety margin and increase organ damage due to hypoxemia. Since there is no comparison between normal SpO_2_ management and low SpO_2_ management, the certainty of the evidence cannot be described. |  |
| **Values**  Is there important uncertainty about or variability in how much people value the main outcomes? | | |
| **Judgement** | **Research evidence** | **Additional considerations** |
| ○ Important uncertainty or variability  ○ Possibly important uncertainty or variability  ○ Probably no important uncertainty or variability  ● No important uncertainty or variability | “Mortality” is generally a critical outcome, and there is no great uncertainty or diversity of values about this. |  |
| **Balance of effects**  Does the balance between desirable and undesirable effects favor the intervention or the comparison? | | |
| **Judgement** | **Research evidence** | **Additional considerations** |
| ○ Favors the comparison  ○ Probably favors the comparison  ○ Does not favor either the intervention or the comparison  ○ Probably favors the intervention  ○ Favors the intervention  ○ Varies  ● Do not know | No relevant studies. |  |
| **Acceptability**  Is the intervention acceptable to key stakeholders? | | |
| **Judgement** | **Research evidence** | **Additional considerations** |
| ○ No  ○ Probably no  ● Probably yes  ○ Yes  ○ Varies  ○ Do not know | It is possible that some patients, family members, and health care providers may not accept lower SpO_2_ than normal. There needs to be an explanation that the patient understands and accepts that SpO_2_ must be managed at a lower-than-normal level. |  |
| **Feasibility**  Is the intervention feasible to implement? | | |
| **Judgement** | **Research evidence** | **Additional considerations** |
| ○ No  ○ Probably no  ○ Probably yes  ● Yes  ○ Varies  ○ Do not know | Maintaining a patient’s SpO_2_ below normal can be achieved by adjusting the ventilator. It is also described in the consensus statement of the Pediatric Acute Lung Injury Consensus Conference (PALICC) and has already been implemented in daily clinical practice. This seems feasible even in consideration of its harm. |  |

**Summary of Judgement**

|  | **JUDGMENT** | | | | | | |
| --- | --- | --- | --- | --- | --- | --- | --- |
| **PROBLEM** | No | Probably No | Probably Yes | **Yes** |  | Varies | Do not know |
| **DESIRABLE EFFECTS** | Trivial | Small | Moderate | Large |  | Varies | **Do not know** |
| **UNDESIRABLE EFFECTS** | Large | Moderate | Small | Trivial |  | Varies | **Do not know** |
| **CERTAINTY OF EVIDENCE** | Very low | Low | Moderate | High |  |  | **No included studies** |
| **VALUES** | Important uncertainty or variability | Possibly important uncertainty or variability | Probably no important uncertainty of variability | **No important uncertainty of variability** |  |  |  |
| **BALANCE OF EFFECTS** | Favors the comparison | Probably favors the comparison | Does not favor either the intervention or the comparison | Probably favors the intervention | Favors the intervention | Varies | **Do not know** |
| **ACCEPTABILITY** | No | Probably No | **Probably Yes** | Yes |  | Varies | Do not know |
| **FEASIBILITY** | No | Probably No | Probably Yes | **Yes** |  | Varies | Do not know |

**Type of Recommendation**

| Strong recommendation against the intervention | Conditional recommendation against the intervention | Conditional recommendation for either the intervention or the comparison | Conditional recommendation for the intervention | Strong recommendation for the intervention |
| --- | --- | --- | --- | --- |
| ○ | ○ | ○ | ○ | ○ |

**CONCLUSION**

| **Recommendation** |
| --- |
| We cannot provide a specific target SpO_2_ value for pediatric patients with ARDS, but target SpO_2_ value are set so as to avoid excessively high or low oxygen levels, which could cause organ damage (in our practice statement).  Supplementary item:  None |
|  |
| **Justification** |
| **Question：** How should target SpO_2_ values be set in pediatric patients with ARDS?  **Population：**Pediatric (as defined in the article, under 20 years old if not stated), ARDS patients (as defined in the article)  **Intervention：**low SpO_2_ management  **Comparison：**normal SpO_2_ management  **Explanation：**  The ventilation strategy for patients with ARDS is to reduce the incidence of oxygen toxicity and ventilator induced lung injury while maintaining oxygen supply to tissues and organs. The concept of tolerating low oxygen saturation is referred to as "permissive hypoxemia.  For pediatric patients with ARDS, temporarily improving oxygenation is not associated with improved long-term outcomes.^1-3^ In addition, inhalation of high concentrations of oxygen and hyperoxemia may lead to cellular injury, including to alveolar epithelial cells, from the production of reactive oxygen species.1-3 Pediatric Acute Lung Injury The Pediatric Acute Lung Injury Consensus Conference (PALICC) recommends a target SpO_2_ of 92-97% and a PEEP of less than 10 cmH2O for mild pediatric ARDS. 88-92% SpO_2_ is the target for low SpO_2_ management for pediatric ARDS patients who require a PEEP of 10 cmH2O or higher. It also recommends considering low SpO_2_ management with a goal of 88-92% SpO_2_ for pediatric patients with ARDS requiring PEEP of 10 cmH2O or higher, but notes that there is no evidence to support this. They also recommend that oxygenation and ventilation goals should be fine-tuned to account for the benefits and harms of assisted ventilation. Because of the organ damage concerns associated with permissive hypoxemia, it is recommended that indices of oxygen supply and demand, such as central venous oxygen saturation, be monitored when SpO_2_ is managed below 92%. ^4^ The SSCG in children, published in 2020, does not provide oxygen saturation targets for pediatric patients with ARDS due to sepsis.  It is also important to consider comorbidities when managing low SpO_2_. In pulmonary hypertension without congenital heart disease, oxygen is appropriate for children with SpO_2_ <92% and PaO2 <60 mmHg.6 Care should also be taken with diseases such as acute intracranial disease, where the harm of hypoxemia is greater.  In the absence of high-quality evidence comparing low SpO_2_ management with usual management in pediatric patients with ARDS, no clear recommendations can be offered in response to this CQ. Therefore, this CQ is not an evidence-based recommendation, but rather a description of current practice.  **Summary of evidence**：  No relevant studies  **Quality of evidence**：  Since there are no relevant studies, the quality of evidence cannot be assessed.  **Balance of effects, Acceptability, Feasibility：**  There are no relevant studies, and it is impossible to determine the balance of effects. Since management targeting a specific SpO_2_ can be achieved only by changing the ventilator settings and is already implemented in daily clinical practice, there is no problem with acceptance or feasibility.  **Panel meeting**：  At a prior meeting, a modified Delphi method was used to determine that "specific SpO_2_ targets cannot be recommended for pediatric patients with ARDS, but SpO_2_ targets are set to avoid hyperoxia and hypoxemia that could cause organ failure" was agreed upon as the in our practice statement with a median score of 8 and a disagreement index of 0.0000.  At the panel meeting, there was an opinion that the meaning of hyperoxia was ambiguous. Therefore, the recommendation was changed to "We cannot provide a specific target SpO_2_ value for pediatric patients with ARDS, but target SpO_2_ value are set so as to avoid excessively high or low oxygen levels, which could cause organ damage (in our practice statement). ” and re-voted. Using the modified Delphi method, a median score of 9 and a disagreement index of 0.1316 were agreed upon, as in our practice statement.  **Additional considerations**：  None |

| **Subgroup considerations** |
| --- |
| In cases where hypoxemia is a significant risk, such as cyanotic heart disease, pulmonary hypertension, and acute intracranial disease, the risks and benefits should be carefully weighed. |
| **Implementation considerations** |
| Although there is no evidence to examine in this systematic review, lower oxygenation targets are already being implemented in daily practice. Cost is not likely to be an issue. The harm and acceptability of hypoxemia need to be considered on a case-by-case basis, as they may vary depending on the patient's background.  It is important to note that if SpO_2_ is managed at 100%, it is difficult to quickly identify changes in PaO_2_. On the other hand, if SpO_2_ is less than 92%, indicators of oxygen supply and demand, such as central venous blood oxygen saturation and lactate levels, should be monitored.  In addition, when managing low SpO_2_, it is necessary to be able to intervene quickly when SpO_2_ falls below the target value. |

| **Monitoring and evaluation** | |
| --- | --- |
| More information needs to be collected to determine whether organ damage due to hypoxemia is increasing. It is also necessary to monitor the implementation status of the guidelines through questionnaires and other means after publication to determine if there are other clinical problems. | |
| **Research priorities** | |
| The Oxy-PICU^7^ study investigated the safety and appropriateness of targeted management of low SpO_2_ in pediatric patients. The study was designed to compare the management of SpO_2_ >94% with SpO_2_ 88%–92% in patients requiring oxygen, suggesting that targeted management of low SpO_2_ is safe. However, this study did not include patients with ARDS. The mean SpO_2_ of the low-SpO_2_ group was 94%, which is above the target for patients with severe ARDS and not applicable to pediatric patients with ARDS. In other words, there have been no large studies evaluating tolerable hypoxemia in pediatric ARDS.  Studies of pediatric ARDS may include patients between the ages of 0 and 18 years. However, the anatomical background among patients varies widely, with some groups of patients having a potentially problematic immaturity and others having a body size comparable to that of adults. Therefore, in studies of pediatric ARDS, the age of the patients should be standardized. In addition, RCTs comparing pediatric ARDS patient groups divided by oxygenation targets are needed. Thus, we need to redefine permissive hypoxia and determine oxygenation targets based on a uniform approach to ventilatory management of ARDS. |  |

Reference

1. Curley MA, Hibberd PL, Fineman LD, et al. Effect of prone positioning on clinical outcomes in children with acute lung injury: a randomized controlled trial. JAMA 2005;294:229-37.
2. Dobyns EL, Anas NG, Fortenberry JD, et al. Interactive effects of high-frequency oscillatory ventilation and inhaled nitric oxide in acute hypoxemic respiratory failure in pediatrics. Crit Care Med 2002;30:2425-9.
3. El-Nawawy A, Moustafa A, Heshmat H, Abouahmed A. High frequency oscillatory ventilation versus conventional mechanical ventilation in pediatric acute respiratory distress syndrome: A randomized controlled study. Turk J Pediatr 2017;59:130-43.
4. Rimensberger PC, Cheifetz IM. Ventilatory support in children with pediatric acute respiratory distress syndrome: proceedings from the Pediatric Acute Lung Injury Consensus Conference. Pediatr Crit Care Med 2015;16:S51-60.
5. Weiss SL, Peters MJ, Alhazzani W, et al. Surviving sepsis campaign international guidelines for the management of septic shock and sepsis-associated organ dysfunction in children. Intensive Care Med 2020;46:10-67.
6. Hansmann G, Koestenberger M, Alastalo T-P, et al. 2019 updated consensus statement on the diagnosis and treatment of pediatric pulmonary hypertension: The European Pediatric Pulmonary Vascular Disease Network (EPPVDN), endorsed by AEPC, ESPR and ISHLT. The Journal of Heart and Lung Transplantation 2019;38:879-901.
7. Peters MJ, Jones GAL, Wiley D, et al. Conservative versus liberal oxygenation targets in critically ill children: the randomised multiple-centre pilot Oxy-PICU trial. Intensive Care Med 2018;44:1240-8.

**PCQ9 Should muscle relaxants be used at an early stage in pediatric patients with moderate to severe ARDS?**

1.Search strategy

MEDLINE via PubMed （Search date: 2020/6/23）

| #1 | Respiratory Distress Syndrome, Adult[mh] OR Respiratory Insufficiency[mh] OR Severe Acute Respiratory Syndrome[mh] OR respiratory distress syndrome[tiab] OR respiratory failure[tiab] OR ARDS[tiab] OR Acute Lung Injury[mh] OR acute lung injury[tiab] |
| --- | --- |
| #2 | neuromuscular blockade[mh] OR neuromuscular blocking agents[mh] OR muscle relaxants, central[mh] OR neuromuscular blocker[tiab] OR neuromuscular blockade[tiab] OR neuromuscular blocking drug*[tiab] OR neuromuscular blocking agent*[tiab] OR muscle relaxant[tiab] OR paralytics[tiab] OR respiratory paralysis[tiab] |
| #3 | vecuronium OR pancuronium OR rocuronium OR atracurium OR cisatracurium OR succinylcholine OR curare OR rapacuronium OR mivacurium OR mivacron OR tracrium OR doxacurium OR nuromax OR bex OR norcuron OR zemuron OR pavulon OR tubocurarine OR gallamine OR flaxedil OR pipecuronium OR alcuronium OR toxiferine OR suxamethonium OR raplon |
| #4 | #2 OR #3 |
| #5 | #1 AND #4 |
| #6 | animals[mh] NOT humans[mh] |
| #7 | #5 AND #6 |

CENTRAL （Search date: 2020/6/23）

| #1 | Respiratory Distress Syndrome, Adult[mh] OR Respiratory Insufficiency[mh] OR Severe Acute Respiratory Syndrome[mh] OR respiratory distress syndrome[tiab] OR respiratory failure[tiab] OR ARDS[tiab] OR Acute Lung Injury[mh] OR acute lung injury[tiab] |
| --- | --- |
| #2 | neuromuscular blockade[mh] OR neuromuscular blocking agents[mh] OR muscle relaxants, central[mh] OR neuromuscular blocker[tiab] OR neuromuscular blockade[tiab] OR neuromuscular blocking drug*[tiab] OR neuromuscular blocking agent*[tiab] OR muscle relaxant[tiab] OR paralytics[tiab] OR respiratory paralysis[tiab] |
| #3 | vecuronium OR pancuronium OR rocuronium OR atracurium OR cisatracurium OR succinylcholine OR curare OR rapacuronium OR mivacurium OR mivacron OR tracrium OR doxacurium OR nuromax OR bex OR norcuron OR zemuron OR pavulon OR tubocurarine OR gallamine OR flaxedil OR pipecuronium OR alcuronium OR toxiferine OR suxamethonium OR raplon |
| #4 | #2 OR #3 |
| #5 | #1 AND #4 |

Igaku-Chuo-Zasshi （Search date: 2020/6/23）

| #1 | 呼吸窮迫症候群-急性/TH or 急性呼吸窮迫症候群/TA or ARDS/TA |
| --- | --- |
| #2 | 急性肺損傷/TH or 急性肺損傷/TA |
| #3 | 重症急性呼吸器症候群/TH or SARS/TA |
| #4 | 呼吸不全/TH or 呼吸不全/TA |
| #5 | #1 or #2 or #3 or #4 |
| #6 | 神経筋遮断/TH or 神経筋遮断剤/TH or 中枢性筋弛緩剤/TH or 神経筋遮断/TA or 弛緩/TA |
| #7 | ("Vecuronium Bromide"/TH or vecuronium/AL) or (Pancuronium/TH or pancuronium/AL) or ("Rocuronium Bromide"/TH or rocuronium/AL) or ("Atracurium Besilate"/TH or atracurium/AL) or ("Cisatracurium Besilate"/TH or cisatracurium/AL) or (Succinylcholine/TH or succinylcholine/AL) or (Curare/TH or curare/AL) or ("Rapacuronium Bromide"/TH or rapacuronium/AL) or ("Mivacurium Chloride"/TH or mivacurium/AL) or ("Mivacurium Chloride"/TH or mivacron/AL) or ("Atracurium Besilate"/TH or tracrium/AL) or ("Doxacurium Chloride"/TH or doxacurium/AL) or ("Doxacurium Chloride"/TH or nuromax/AL) or bex/AL or ("Vecuronium Bromide"/TH or norcuron/AL) or ("Rocuronium Bromide"/TH or zemuron/AL) or (Pancuronium/TH or pavulon/AL) or (Tubocurarine/TH or tubocurarine/AL) or ("Gallamine Triethiodide"/TH or gallamine/AL) or ("Gallamine Triethiodide"/TH or flaxedil/AL) or (Pipecuronium/TH or pipecuronium/AL) or (Alcuronium/TH or alcuronium/AL) or (Toxiferine/TH or toxiferine/AL) or (Succinylcholine/TH or suxamethonium/AL) or ("Rapacuronium Bromide"/TH or raplon/AL) |
| #8 | #6 or #7 |
| #9 | #5 and #8 |
| #10 | (#9) and (PT=会議録除く) |

1. Flow diagram

**Identification**

0 Studies included in qualitative synthesis

7 Full-text articles assessed for eligibility

1,880 records after duplicates removed

1,885 records identified through database searching

1,885 records identified through database searching

Medline via PubMed (n=1,337)

CENTRAL (n=171)

Igaku-Chuo-Zasshi (n=377)

0 additional records identified through other sources

0 Studies included in quantitative synthesis (meta-analysis)

7 Full-text articles excluded, with reasons:

・Wrong study design (n=6)

・Wrong population (n=1)

Duplicates

n=5

1,873 records excluded

**Included**

**Eligibility**

**Screening**

1. Risk of bias

Not applicable

1. Forest plot

Not applicable

1. Evidence profile

Not applicable

1. Evidence-to-Decision table

| **QUESTION** | |
| --- | --- |
| **PCQ9：** Should muscle relaxants be used at an early stage in pediatric patients with moderate to severe ARDS? | |
| **POPULATION:** | Pediatric patients (as defined in the article, 20 years old or younger if not specified) on ventilators for moderate to severe ARDS (as defined in the article) |
| **INTERVENTION:** | Administer muscle relaxants (within 48 hours of diagnosis) |
| **COMPARISON:** | No muscle relaxants administered |
| **MAIN OUTCOMES:** | Mortality, Developmental prognosis, Ventilator-free days (VFD), Length of hospital stay, Ventilator-associated pneumonia |
| **SETTINGS:** | Emergency department or intensive care unit (ICU) |
| **PERSPECTIVE:** | Individual |
| **BACKGROUND:** | The use of muscle relaxants in patients with acute respiratory distress syndrome (ARDS)　has been reported to avoid excessive stress on the alveoli, reduce barotrauma, and improve oxygenation, suggesting an improved prognosis. However, there are many reports of complications from the use of therapies that decrease or eliminate spontaneous breathing. Clarifying whether ventilatory management using muscle relaxants to decrease or eliminate spontaneous breathing is an important clinical issue. |
| **CONFLICT OF INTERESTS:** | None |

**ASSESSMENT**

| **Problem**  Is the problem a priority? | | |
| --- | --- | --- |
| **Judgement** | **Research evidence** | **Additional considerations** |
| ○ No  ○ Probably no  ○ Probably yes  ● Yes  ○ Varies  ○ Do not know | The use of muscle relaxants in adult patients with ARDS has been reported to avoid excessive stress on the alveoli, reduce barotrauma, and improve oxygenation, suggesting an improved prognosis. However, there are many reports of complications from the use of therapies that decrease or eliminate spontaneous breathing. Clarifying whether ventilatory management using muscle relaxants to decrease or eliminate spontaneous breathing is an important clinical issue in pediatric ARDS patients. There have been no interventional studies or systematic reviews in pediatric ARDS patients, and the efficacy of muscle relaxants and certainty of the evidence are unknown. Therefore, this issue is of high priority. |  |
| **Desirable Effects**  How substantial are the desirable anticipated effects? | | |
| **Judgement** | **Research evidence** | **Additional considerations** |
| ○ Trivial  ○ Small  ○ Moderate  ○ Large  ○ Varies  ● Do not know | Integrated into the “Certainty of evidence”. |  |
| **Undesirable Effects**  How substantial are the undesirable anticipated effects? | | |
| **Judgement** | **Research evidence** | **Additional considerations** |
| ○ Large  ○ Moderate  ○ Small  ○ Trivial  ○ Varies  ● Do not know | Integrated into the “Certainty of evidence”. |  |
| **Certainty of evidence**  What is the overall certainty of the evidence of effects? | | |
| **Judgement** | **Research evidence** | **Additional considerations** |
| ○ Very low  ○ Low  ○ Moderate  ○ High  ● No included studies | Muscle relaxants are thought to be effective in decreasing patient-ventilator synchrony,　decreasing oxygen consumption, increasing respiratory compliance and functional residual air volume, and preventing hyperinflation in compliant lung regions in ARDS patients requiring ventilator management. However, the side effects of muscle relaxants may include ICU-acquired weakness and the development of ventilator-associated pneumonia. However, there is no comparison of the balance of benefits and harms of muscle relaxants in pediatric ARDS patients, and the certainty of the evidence cannot be described. |  |
| **Values**  Is there important uncertainty about or variability in how much people value the main outcomes? | | |
| **Judgement** | **Research evidence** | **Additional considerations** |
| ○ Important uncertainty or variability  ○ Possibly important uncertainty or variability  ○ Probably no important uncertainty or variability  ● No important uncertainty or variability | “Mortality” is generally a critical outcome, and there is no great uncertainty or diversity of values regarding this. |  |
| **Balance of effects**  Does the balance between desirable and undesirable effects favor the intervention or the comparison? | | |
| **Judgement** | **Research evidence** | **Additional considerations** |
| ○ Favors the comparison  ○ Probably favors the comparison  ○ Does not favor either the intervention or the comparison  ○ Probably favors the intervention  ○ Favors the intervention  ○ Varies  ● Do not know | No relevant studies. |  |
| **Acceptability**  Is the intervention acceptable to key stakeholders? | | |
| **Judgement** | **Research evidence** | **Additional considerations** |
| ○ No  ○ Probably no  ● Probably yes  ○ Yes  ○ Varies  ○ Do not know | The use of muscle relaxants, while potentially improving prognosis, was deemed "probably yes" for patients and their families to accept, given the potential for long-term adverse events such as ICU-acquired weakness. |  |
| **Feasibility**  Is the intervention feasible to implement? | | |
| **Judgement** | **Research evidence** | **Additional considerations** |
| ○ No  ○ Probably no  ○ Probably yes  ● Yes  ○ Varies  ○ Do not know | Muscle relaxants are already commonly used for ventilator management and seems feasible. |  |

**Summary of Judgement**

|  | **JUDGMENT** | | | | | | |
| --- | --- | --- | --- | --- | --- | --- | --- |
| **PROBLEM** | No | Probably No | Probably Yes | **Yes** |  | Varies | Do not know |
| **DESIRABLE EFFECTS** | Trivial | Small | Moderate | Large |  | Varies | **Do not know** |
| **UNDESIRABLE EFFECTS** | Large | Moderate | Small | Trivial |  | Varies | **Do not know** |
| **CERTAINTY OF EVIDENCE** | Very low | Low | Moderate | High |  |  | **No included studies** |
| **VALUES** | Important uncertainty or variability | Possibly important uncertainty or variability | Probably no important uncertainty of variability | **No important uncertainty of variability** |  |  |  |
| **BALANCE OF EFFECTS** | Favors the comparison | Probably favors the comparison | Does not favor either the intervention or the comparison | Probably favors the intervention | Favors the intervention | Varies | **Do not know** |
| **ACCEPTABILITY** | No | Probably No | **Probably Yes** | Yes |  | Varies | Do not know |
| **FEASIBILITY** | No | Probably No | Probably Yes | **Yes** |  | Varies | Do not know |

**Type of Recommendation**

| Strong recommendation against the intervention | Conditional recommendation against the intervention | Conditional recommendation for either the intervention or the comparison | Conditional recommendation for the intervention | Strong recommendation for the intervention |
| --- | --- | --- | --- | --- |
| ○ | ○ | ○ | ○ | ○ |

**CONCLUSION**

| **Recommendation** |
| --- |
| We cannot provide a recommendation on the early use of muscle relaxants for pediatric patients with moderate to severe ARDS, but muscle relaxants have been used at an early stage in accordance with the treatment strategy of adult patients with moderate to severe ARDS (in our practice statement).  Supplementary item:  None |
|  |
| **Justification** |
| **Question:** Should muscle relaxants be used at an early stage in pediatric patients with moderate to severe ARDS?  **Population:** Pediatric patients (as defined in the article, 20 years old or younger if not specified) on ventilators for moderate to severe ARDS (as defined in the article)  **Intervention:** Administer muscle relaxants (within 48 hours of diagnosis)  **Comparison:** No muscle relaxants administered  **Explanation:**  In adults with ARDS, muscle relaxants have been used early to minimize ventilator-associated lung injury, patient-ventilator dyssynchrony, and reduce lung volumes and oxygen consumption.^1^ Multiple landmark studies have been conducted.^2,3^ This guideline proposes the early use of muscle relaxants in adults with moderate-to-severe ARDS. This guideline proposes the early use of muscle relaxants in adults with moderate-to-severe ARDS. This guideline proposes the early use of muscle relaxants in adult patients with moderate-to-severe ARDS (Weak recommendation, Very low certainty of evidence: GRADE 2D).  On the other hand, there are no randomized controlled trials (RCTs) investigating the efficacy of early use of muscle relaxants in pediatric ARDS patients, and the accumulated knowledge is insufficient. To date, the following recommendations have been made regarding the use of muscle relaxants in pediatric ARDS. In 2015, the Pediatric Acute Lung Injury Consensus Conference (PALICC) recommended that muscle relaxants be used at the minimum required dose, with monitoring of their effects, when sedation alone does not provide effective ventilation.^4^ In 2020, the Surviving Sepsis Campaign Guidelines (SSCG) recommended the use of muscle relaxants for severe ARDS due to sepsis (Weak recommendation, Very low quality of evidence)^5^, although only one retrospective study of pediatric patients of mechanical ventilation^6^ was referenced for this recommendation. Regarding the use of muscle relaxants for pediatric ARDS, it has been reported that 31% of all pediatric ARDS patients and 50% of pediatric patients with severe ARDS are receiving continuous muscle relaxants within 72 hours of diagnosis.^7^  Thus, at this time, there is no clear recommendation for this CQ because there is no high-quality evidence comparing the early use of muscle relaxants in pediatric patients with moderate-to-severe ARDS. Therefore, this CQ is not an evidence-based recommendation, but rather a description of current practice.  **Summary of Evidence:** No relevant studies.  **Quality of Evidence:** Since there are no relevant studies, the quality of the evidence cannot be assessed.  **Balance of effects, Acceptability, Feasibility:**  Muscle relaxants are thought to reduce patient-ventilator dyssynchrony, decrease oxygen consumption, increase respiratory compliance and functional residual capacity, and prevent hyperinflation of compliant lung areas in ARDS patients requiring ventilator management. On the other hand, muscle relaxants may have adverse effects such as the development of ICU-acquired weakness. However, the balance of benefits and harms of muscle relaxants in pediatric ARDS patients has not been compared, and the confidence of the evidence cannot be stated. Muscle relaxants are used in daily clinical practice and there is no problem of acceptability and feasibility considering the harms.  **Panel meeting:**  In the pre-vote, the modified Delphi method resulted in the following recommendation: "Although we cannot provide a recommendation for the early use of muscle relaxants in pediatric patients with moderate to severe ARDS, muscle relaxants are currently being used at the early phase in accordance with treatment strategies for adult patients with moderate to severe ARDS. Supplementary items: "We note that the muscle relaxant currently used in RCTs in adult patients with ARDS is cisatracurium, which is unapproved in Japan as of January 2021.” was agreed upon in our practice statement with a median score of 8 and a disagreement index of 0.1316.  At the panel meeting, the opinion was expressed that the frequency of use of muscle relaxants may be overestimated and that cisatracurium should not be mentioned in the recommendation. Therefore, the recommendation was changed to "We cannot provide a recommendation on the early use of muscle relaxants for pediatric patients with moderate to severe ARDS, but muscle relaxants have been used at an early stage in accordance with the treatment strategy of adult patients with moderate to worse ARDS (in our practice statement)." and re-voted without supplementary items. Using the modified Delphi method, a median score of 9 and a disagreement index of 0.1316 were agreed upon, as in our practice statement.  **Supplementary note:**  None |

| **Subgroup considerations** |
| --- |
| As a subgroup, the classification of ARDS according to the severity of oxygenation impairment (mild, moderate, and severe) in the PALICC definition needs to be considered. |
| **Implementation considerations** |
| Cisatracurium is a muscle relaxant currently being used in RCTs in adults with ARDS, although as of January 2021, it has not been approved in Japan. Patients on muscle relaxants should be carefully monitored for respiratory and cardiovascular changes. |

| **Monitoring and evaluation** |
| --- |
| It is necessary to monitor whether the use of muscle relaxants increases the occurrence of post-traumatic stress disorder (PTSD) and other problems that occur when sedation levels are inadequate, and whether the frequency, severity and recovery periods of neurological disorders and muscle atrophy occur as long-term side effects. In addition, it is necessary to monitor the implementation status through questionnaires and other means after the guidelines are published to see if there are any other clinical problems. |
| **Research priorities** |
| Multiple RCTs have suggested that the benefits of muscle relaxants in adults with moderate to severe ARDS may outweigh the harm. In contrast, there is no high-quality evidence in children, and its efficacy is unknown. There are unresolved issues such as whether different types of muscle relaxants have different effects, what depth of muscle relaxation is required, and whether there is a relationship with ventilator-induced diaphragm dysfunction. The impact on developmental prognosis should also be considered as an important issue specific to children. Clinical trials and RCTs in pediatric patients with moderate to severe ARDS are needed. |

**Reference**

1. Rosenberg L, Traube C. Sedation strategies in children with pediatric acute respiratory distress syndrome (PARDS). Annals of translational medicine 2019;7:509.

2. Papazian L, Forel JM, Gacouin A, et al. Neuromuscular blockers in early acute respiratory distress syndrome. The New England journal of medicine 2010;363:1107-16.

3. Moss M, Huang DT, Brower RG, et al. Early Neuromuscular Blockade in the Acute Respiratory Distress Syndrome. The New England journal of medicine 2019;380:1997-2008.

4. Pediatric acute respiratory distress syndrome: consensus recommendations from the Pediatric Acute Lung Injury Consensus Conference. Pediatric critical care medicine : a journal of the Society of Critical Care Medicine and the World Federation of Pediatric Intensive and Critical Care Societies 2015;16:428-39.

5. Weiss SL, Peters MJ, Alhazzani W, et al. Surviving Sepsis Campaign International Guidelines for the Management of Septic Shock and Sepsis-Associated Organ Dysfunction in Children. Pediatric critical care medicine : a journal of the Society of Critical Care Medicine and the World Federation of Pediatric Intensive and Critical Care Societies 2020;21:e52-e106.

6. Da Silva PS, Neto HM, de Aguiar VE, Lopes E, Jr., de Carvalho WB. Impact of sustained neuromuscular blockade on outcome of mechanically ventilated children. Pediatrics international : official journal of the Japan Pediatric Society 2010;52:438-43.

7. Rowan CM, Klein MJ, Hsing DD, et al. Early Use of Adjunctive Therapies for Pediatric Acute Respiratory Distress Syndrome: A PARDIE Study. American journal of respiratory and critical care medicine 2020;201:1389-97.

**PCQ10 Should pediatric patients with moderate to severe ARDS be placed in the prone position?**

1.Search strategy

Medline via PubMed（Search date: 2020/6/28）

| #1 | "Respiratory Distress Syndrome, Adult"[Mesh] OR "Acute Respiratory Distress"[Title/Abstract] OR ARDS[Title/Abstract] OR "Acute Lung Injury"[Mesh] OR Acute Lung Injur*[Title/Abstract] |
| --- | --- |
| #2 | "Respiratory Distress Syndrome, Newborn"[Mesh] |
| #3 | #1 OR #2 |
| #4 | "Prone Position"[Mesh] OR Prone Position*[Title/Abstract] |
| #5 | #3 AND #4 |

CENTRAL（Search date: 2020/6/22）

| #1 | [mh "Respiratory Distress Syndrome, Adult"] OR "acute respiratory distress":ti,ab OR ARDS:ti,ab OR [mh "Acute Lung Injury"] OR "acute lung injury":ti,ab |
| --- | --- |
| #2 | [mh "Respiratory Distress Syndrome, Newborn"] |
| #3 | #1 OR #2 |
| #4 | [mh "Prone Position"] OR "prone position":ti,ab |
| #5 | #3 AND #4 |
|  | Trials |

Igaku-Chuo-Zasshi （Search date: 2020/6/22）

| #1 | 呼吸窮迫症候群-急性/TH or 急性呼吸窮迫症候群/TA or ARDS/TA |
| --- | --- |
| #2 | 急性肺損傷/TH or 急性肺損傷/TA |
| #3 | #1 or #2 |
| #4 | 腹臥位/TH or 腹臥位/TA |
| #5 | #3 and #4 |
| #6 | (#5) and (PT=会議録除く) |

1. Flow diagram

**Identification**

1 Studies included in qualitative synthesis

27 Full-text articles assessed for eligibility

1330 records after duplicates removed

1,362 records identified through database searching

1,362 records identified through database searching

Medline via PubMed (n=1,008)

CENTRAL (n=118)

Igaku-Chuo-Zasshi (n=236)

0 additional records identified through other sources

1 Studies included in quantitative synthesis (meta-analysis)

26 Full-text articles excluded, with reasons:

・Wrong study design (n=15)

・Wrong population (n=9)

・Wrong intervention (n=2)

Duplicates

n=32

1303 records excluded

**Included**

**Eligibility**

**Screening**

1. Risk of bias

Mortality Ventilator-free days (VFD)

Developmental prognosis Tracheal tube problems

Decubitus ulcer

1. Forest plot

Mortality

Ventilator-free days (VFD)

Developmental prognosis

Tracheal tube problems

Decubitus ulcer

1. Evidence profile

| **Assessment of certainty** | | | | | | | **No. of patients** | | **Efficacy** | | **Certainty of the evidence** | **Importance** |
| --- | --- | --- | --- | --- | --- | --- | --- | --- | --- | --- | --- | --- |
| **No. of studies** | **Study design** | **Risk of bias** | **Inconsistency** | **Indirectness** | **Imprecision** | **Others** | **Weaning protocol** | **Control** | **Relative index (95% CI)** | **Absolute index (95% CI)** |  |  |
| **Mortality** ^a^ | | | | | | | | | | | | |
| 1 | RCT | Not serious | Not serious | Not serious | Very serious ^b^ | None | 4/51 (7.8%) | 4/50 (8.0%) | **RR 0.98** (0.26 to 3.71) | **-2 per 1000 patients** (-59 to +217) | ⨁⨁◯◯ Low | Critical |
| **Ventilator-free days (VFD)** | | | | | | | | | | | | |
| 1 | RCT | Serious ^c^ | Not serious | Not serious | Very serious ^b^ | None | 51 | 50 | - | **MD 0.3 days shorter** (-3.63 to +3.03) | ⨁◯◯◯ Very Low | Critical |
| **Developmental prognosis** ^d^ | | | | | | | | | | | | |
| 1 | RCT | Not serious | Not serious | Not serious | Very serious ^b^ | None | 6/51 (11.8%) | 11/50 (22.0%) | **RR 0.53** (0.21 to 1.34) | **-103 per 1000 patients** (-174 to +75) | ⨁⨁◯◯ Low | Critical |
| **Tracheal tube problems** | | | | | | | | | | | | |
| 1 | RCT | Not serious | Not serious | Not serious | Very serious ^b^ | None | 4/51 (7.8%) | 5/50 (10.0%) | **RR 0.78** (0.22 to 2.75) | **-22 per 1000 patients** (-78 to +175) | ⨁⨁◯◯ Low | Critical |
| **Decubitus ulcer** | | | | | | | | | | | | |
| 1 | RCT | Not serious | Not serious | Not serious | Very serious ^b^ | None | 13/50 (26.0%) | 13/51 (25.5%) | **RR 1.02** (0.53 to 1.98) | **+5 per 1000 patients** (-120 to +250) | ⨁⨁◯◯ Low | Important |

**CI:** Confidence interval; **RR:** Relative risk; **MD:** Mean difference; RCT: Randomized controlled trial

Note: No studies reported the outcomes including length of hospital stay or oxygenation improvement.

**Explanations**

a. The mortality is 28-day one.

b. The sample size of 101 did not meet the optimal information size (OIS), and the 95% confidence interval included both clinically meaningful thresholds for benefit and harm.

c. Rated as “Serious” for one risk of bias (RoB) item (blinding of study participants and treatment providers).

d. Developmental prognosis was the worsening of pediatric cerebral performance category (PCPC) or pediatric overall performance category (POPC) at the time of discharge compared to the time of admission.

e. No studies reported outcomes related to length of hospital stay or improvement in oxygenation.

1. Evidence-to-Decision table

| **QUESTION** | |
| --- | --- |
| **PCQ10：** Should pediatric patients with moderate to severe ARDS be placed in the prone position? | |
| **POPULATION:** | Pediatric patients (as defined in the article, 20 years old or younger if not specified) on ventilators for moderate to severe ARDS (as defined in the article) |
| **INTERVENTION:** | Perform prone position |
| **COMPARISON:** | No prone position |
| **MAIN OUTCOMES:** | Mortality, Developmental prognosis, Ventilator-free days (VFD), Length of hospital stay, Tracheal tube problems (unplanned extubation, occlusion, displacement) |
| **SETTINGS:** | Emergency department or intensive care unit (ICU) |
| **PERSPECTIVE:** | Individual |
| **BACKGROUND:** | Prone position has the potential to be an effective treatment for acute respiratory distress syndrome (ARDS) because of its potential pathophysiological benefits, including improvements in respiratory mechanics, oxygenation, hemodynamics, and prevention of ventilator-induced lung injury (VILI/VALI). Although many randomized controlled trials (RCTs) and meta-analyses have been conducted, the results have been inconsistent and the clinical efficacy is controversial. Prone position is a treatment that can be implemented without special equipment, and it is considered a high priority to examine its effectiveness. |
| **CONFLICT OF INTERESTS:** | None |

**ASSESSMENT**

| **Problem**  Is the problem a priority? | | |
| --- | --- | --- |
| **Judgement** | **Research evidence** | **Additional considerations** |
| ○ No  ○ Probably no  ○ Probably yes  ● Yes  ○ Varies  ○ Do not know | The prone position has the potential to be an effective treatment for ARDS because of its potential pathophysiological benefits, including improvements in respiratory mechanics, oxygenation, hemodynamics, and prevention of VILI/VALI. Since the prone position has been shown to be effective in adult ARDS, it can be performed without special equipment, and is relatively easy to perform in children, investigating its effectiveness in pediatric ARDS is a high priority. |  |
| **Desirable Effects**  How substantial are the desirable anticipated effects? | | |
| **Judgement** | **Research evidence** | **Additional considerations** |
| ○ Trivial  ● Small  ○ Moderate  ○ Large  ○ Varies  ○ Do not know | An RCT^1^ consistent with PICO was included in the systematic review and used in the meta-analysis. The study included intubated patients with a P/F ratio of less than 300 (mean [SD] P/F ratio at study entry was 105 [48] in the prone group and 94 [41] in the prone group).  As a beneficial outcome, the estimate of effect for the 28-day mortality (one RCT^1^, N=101) was two fewer per 1,000 (95% CI: 59-217 more) in the intervention group than that in the control group. The estimate of the effect for poor developmental prognosis (one RCT^1^, N=101) was 103 fewer per 1,000 (95% CI: 174 fewer to 75 more). The estimate of effect for VFD (1 RCT^1^, N=101) was the mean difference of 0.3 day shorter (95% CI: 3.63 shorter to 3.03 longer). Therefore, the desirable effect of the intervention was judged to be "small". |  |
| **Undesirable Effects**  How substantial are the undesirable anticipated effects? | | |
| **Judgement** | **Research evidence** | **Additional considerations** |
| ○ Large  ○ Moderate  ○ Small  ● Trivial  ○ Varies  ○ Do not know | As for harmful outcomes, the estimate of the effect of tracheal tube problems (one RCT^1^, N=101) was 22 fewer per 1,000 (95% CI: 78 fewer to 175 more) in the intervention group than that in the control group. The expected undesirable effect was judged to be "trivial". |  |
| **Certainty of evidence**  What is the overall certainty of the evidence of effects? | | |
| **Judgement** | **Research evidence** | **Additional considerations** |
| ● Very low  ○ Low  ○ Moderate  ○ High  ○ No included studies | **The relative importance or values of the main outcomes of interest:**   \| **Outcome** \| **Importance** \| **Certainty of the Evidence**  **(GRADE)** \| \| --- \| --- \| --- \| \| 28-day mortality \| Critical \| ⨁⨁◯◯ Low \| \| Developmental prognosis \| Critical \| ⨁⨁◯◯ Low \| \| VFD \| Critical \| ⨁◯◯◯ Very low \| \| Tracheal tube problems (unplanned extubation, occlusion, displacement) \| Critical \| ⨁⨁◯◯ Low \|   **Overall certainty of evidence**  The desirable effect results showed that the intervention reduced the “28-day mortality" by two per 1,000 and "poor developmental prognosis" by 103 per 1,000. In addition, “VFD” decreased by 0.3 days. The certainty of the evidence was "low," "low," and "very low," respectively. On the other hand, the undesirable effect of the intervention was a decrease in "tracheal tube problems" by 22 per 1,000, and the certainty of evidence was "low. Therefore, the directions of the desirable and undesirable effects were not consistent, and the certainty of the evidence for the entire outcome was judged to be "very low," since the evidence with the lowest certainty was adopted. |  |
| **Values**  Is there important uncertainty about or variability in how much people value the main outcomes? | | |
| **Judgement** | **Research evidence** | **Additional considerations** |
| ○ Important uncertainty or variability  ○ Possibly important uncertainty or variability  ○ Probably no important uncertainty or variability  ● No important uncertainty or variability | “Mortality” is generally a critical outcome, and there is no great uncertainty or diversity of values regarding this. |  |
| **Balance of effects**  Does the balance between desirable and undesirable effects favor the intervention or the comparison? | | |
| **Judgement** | **Research evidence** | **Additional considerations** |
| ○ Favors the comparison  ○ Probably favors the comparison  ○ Does not favor either the intervention or the comparison  ● Probably favors the intervention  ○ Favors the intervention  ○ Varies  ○ Do not know | **Summary of evidence:**   \| **Outcome** \| **Comparison** \| **Intervention** \| **Absolute difference**  **(95% CI)** \| **Risk ratio (RR)**  **(95% CI)** \| \| --- \| --- \| --- \| --- \| --- \| \| 28-day mortality \| 4/50  (8.0%) \| 4/51  (7.8%) \| 2 fewer per 1,000 (59 fewer to 217 more) \| RR 0.98 (0.26 to 3.71) \| \| Poor developmental prognosis \| 11/50  (22.0%) \| 6/51  (11.8%) \| 103 fewer per 1,000 (174 fewer to 75 more) \| RR 0.53 (0.21 to 1.34) \| \| VFD \|  \|  \| MD 0.3 day shorter (3.63 shorter to 3.03 longer) \|  \| \| Tracheal tube problems (unplanned extubation, occlusion, displacement) \| 5/50  (10.0%) \| 4/51  (7.8%) \| 22 fewer per 1,000 (78 fewer to 175 more) \| RR 0.78 (0.22 to 2.75) \|   Based on the above, we surmise that the balance between desirable and undesirable effects was "probably favors the intervention”. |  |
| **Acceptability**  Is the intervention acceptable to key stakeholders? | | |
| **Judgement** | **Research evidence** | **Additional considerations** |
| ○ No  ○ Probably no  ● Probably yes  ○ Yes  ○ Varies  ○ Do not know | Although the prone position is a treatment that requires only repositioning, the facility needs to be proficient in performing it safely. Therefore, the intervention would probably be acceptable to both patients and their families. |  |
| **Feasibility**  Is the intervention feasible to implement? | | |
| **Judgement** | **Research evidence** | **Additional considerations** |
| ○ No  ○ Probably no  ● Probably yes  ○ Yes  ○ Varies  ○ Do not know | In children, who are smaller than adults, the human resources required for repositioning to the prone position may not be as great. However, the management of children in the prone position requires facility proficiency, and different indications of the intervention in each facility should be considered. In addition, although the prone position under light sedation is dangerous, the adverse effects of using deep sedatives and muscle relaxants should be considered. Based on the above, intervention is likely to be feasible. |  |

**Summary of Judgement**

|  | **JUDGMENT** | | | | | | |
| --- | --- | --- | --- | --- | --- | --- | --- |
| **PROBLEM** | No | Probably no | Probably yes | **Yes** |  | Varies | Do not know |
| **DESIRABLE EFFECTS** | Trivial | **Small** | Moderate | Large |  | Varies | Do not know |
| **UNDESIRABLE EFFECTS** | Large | Moderate | Small | **Trivial** |  | Varies | Do not know |
| **CERTAINTY OF EVIDENCE** | **Very low** | Low | Moderate | High |  |  | No included studies |
| **VALUES** | Important uncertainty or variability | Possibly important uncertainty or variability | Probably no important uncertainty of variability | **No important uncertainty of variability** |  |  |  |
| **BALANCE OF EFFECTS** | Favors the comparison | Probably favors the comparison | Does not favor either the intervention or the comparison | **Probably favors the intervention** | Favors the intervention | Varies | Do not know |
| **ACCEPTABILITY** | No | Probably no | **Probably yes** | Yes |  | **Varies** | Do not know |
| **FEASIBILITY** | No | Probably no | **Probably yes** | Yes |  | **Varies** | Do not know |

**Type of Recommendation**

| Strong recommendation against the intervention | Conditional recommendation against the intervention | Conditional recommendation for either the intervention or the comparison | Conditional recommendation for the intervention | Strong recommendation for the intervention |
| --- | --- | --- | --- | --- |
| ○ | ○ | ○ | ● | ○ |

**CONCLUSION**

| **Recommendation** |
| --- |
| We suggest placing pediatric patients with moderate to severe ARDS in the prone position (weak recommendation / very low certainty of evidence：GRADE 2D）.  Supplementary item:  Placing children in the prone position requires the facility to be familiar with the procedure, and applications need to be considered for each facility, including the degree of sedation. |
|  |
| **Justification** |
| **Question**：Should pediatric patients with moderate to severe ARDS be placed in the prone position?  **Population**：Pediatric patients (as defined in the article, 20 years old or younger if not specified) on ventilators for moderate to severe ARDS (as defined in the article)  **Intervention：**Perform prone position  **Main outcomes**：Mortality, Developmental prognosis, Ventilator-free days (VFD), Length of hospital stay, Tracheal tube problems (unplanned extubation, occlusion, displacement)  **Summary of evidence**：  One RCT^1^ consistent with PICO was included in the systematic review and used in the meta-analysis. This study included intubated patients with a P/F ratio of less than 300 (mean [SD] P/F ratio at study entry was 105 [48] in the prone group and 94 [41] in the prone group).  As a beneficial outcome, the estimate of effect for the 28-day mortality (one RCT^1^, N=101) was two fewer per 1,000 (95% CI: 59-217 more) in the intervention group than that in the control group. The estimate of the effect for poor developmental prognosis (one RCT^1^, N=101) was 103 fewer per 1,000 (95% CI: 174 fewer to 75 more). The estimate of effect for VFD (1 RCT^1^, N=101) was the mean difference of 0.3 day shorter (95% CI: 3.63 shorter to 3.03 longer). Therefore, the desirable effect of the intervention was judged to be "small".  As for harmful outcomes, the estimate of the effect of tracheal tube problems (one RCT^1^, N=101) was 22 fewer per 1,000 (95% CI: 78 fewer to 175 more) in the intervention group than that in the control group. The expected undesirable effect was judged to be "trivial".  Based on the above, we surmise that the balance between desirable and undesirable effects was "probably favors the intervention”.  **Certanity of evidence**：  The desirable effect results showed that the intervention reduced the “28-day mortality" by two per 1,000 and "poor developmental prognosis" by 103 per 1,000. In addition, “VFD” decreased by 0.3 days. The certainty of the evidence was "low," "low," and "very low," respectively. On the other hand, the undesirable effect of the intervention was a decrease in "tracheal tube problems" by 22 per 1,000, and the certainty of evidence was "low. Therefore, the directions of the desirable and undesirable effects were not consistent, and the certainty of the evidence for the entire outcome was judged to be "very low," since the evidence with the lowest certainty was adopted.    **Balance of effects,Acceptability、Feasibility**：  The prone position reduces poor developmental prognosis and doesn't increase tracheal tube problems..In children, who are smaller in stature than adults, the human resources required for repositioning to the prone position may not be as great. Though the management of children in the prone position requires facility proficiency、the intervention would probably be acceptable and feasible.  **Panel meeting**：  In the pre-vote, the modified Delphi method resulted in the following recommendation: "We suggest placing pediatric patients with moderate ARDS in the prone position (weak recommendation / very low certainty of evidence：GRADE 2D）. " and the supplementary items "Unlike adults, the body size of children ranges from neonates to adults; therefore, the human resources for prone position management, including position changes, may vary from case to case. The risk of pressure ulcers due to prone positioning is deemed to be lower in pediatric patients than in adults; hence prone position management for 12–24 h is possible” was agreed upon with a median score of 8 and a disagreement index of 0.1639.  At the panel meeting, it was suggested that as the supplementary items, the fact that the prone position is a management method that requires proficiency and that it is important to achieve an appropriate level of sedation and monitoring should be mentioned. Concerns were also expressed about the strength of the rationale for the 12–24 h prone position management time. Therefore, the supplementary items were revised to as follows: "Placing children in the prone position requires the facility to be familiar with the procedure, and applications need to be considered for each facility, including the degree of sedation.” and re- voted. The modified Delphi method resulted in a median score of eight and a disagreement index of 0.1316.  **Additional considerations**：  The management of children in the prone position requires facility proficiency, and different indications of the intervention in each facility should be considered. In addition, although the prone position under light sedation is dangerous, the adverse effects of using deep sedatives and muscle relaxants should be considered. |

| **Subgroup considerations** |
| --- |
| Since infants can be managed in the prone position for a longer period of time than adults, the effectiveness of the therapy can be enhanced by employing infants as a subgroup. |
| **Implementation considerations** |
| It should be noted that the evidence used in this study indicates that the prone position is used in PICUs in the United States that belong to the Pediatric Acute Lung Injury and Sepsis Investigators Network, and that institutional proficiency is required for the management of children in the prone position. The prone position was used in the PICU in the United States. It should be noted that institutional proficiency is required for prone management of children. It can be dangerous to perform these procedures under light sedation. There is room for consideration of harm when using deep sedation/muscle relaxants. Body size may make a difference in the human resources available to perform prone management, such as posture change. |

| **Monitoring and evaluation** |
| --- |
| After the publication of the guidelines, it is necessary to monitor the following outcomes using questionnaires and other means: whether sufficient duration is secured when the prone position is used, whether unplanned extubation, pressure ulcers, or nerve paralysis occurs, whether the amount of sedative medication administered increases, and whether problems arise in the surrounding patients due to large amounts of human resources being invested in the target patients. |
| **Research priorities** |
| The article selected for this systematic review was based on pediatric patients who were intubated and had a P/F ratio of less than 300, and multicenter RCTs in pediatric patients with moderate to severe ARDS are needed. We await the results of the ongoing RCT PROSpect (NCT03896763). |

Reference

1. Curley MA, Hibberd PL, Fineman LD, et al. Effect of prone positioning on clinical outcomes in children with acute lung injury: a randomized controlled trial. Jama 2005;294:229-37.

**PCQ11 Should nitric oxide inhalation therapy be used in pediatric patients with ARDS?**

1.Search strategy

MEDLINE via PubMed (Search date: 2020/7/4)

| #1 | Respiratory Distress Syndrome, Adult[mh] OR Respiratory Distress Syndrome, Newborn[mh] OR ARDS[tiab] OR Acute Lung Injury[tiab] OR Acute Hypoxemic Respiratory Failure[tiab] |
| --- | --- |
| #2 | Nitric oxide[mh] OR Nitric oxide[tiab] |
| #3 | #1 AND #2 |
| #4 | (randomized controlled trial [pt] OR controlled clinical trial [pt] OR randomized [tiab] OR placebo [tiab] OR drug therapy [sh] OR randomly [tiab] OR trial [tiab] OR groups [tiab]) NOT (animals[mh] NOT humans[mh]) |
| #5 | #1 AND #2 AND #4 |

CENTRAL (Search date: 2020/7/4)

| #1 | MeSH descriptor: [Respiratory Distress Syndrome, Adult] explode all trees |
| --- | --- |
| #2 | MeSH descriptor: [Respiratory Distress Syndrome, Newborn] explode all trees |
| #3 | (ARDS):ti,ab,kw |
| #4 | ("Acute Lung Injury"):ti,ab,kw |
| #5 | ("Acute Hypoxemic Respiratory Failure"):ti,ab,kw |
| #6 | ("acute respiratory distress syndrome"):ti,ab,kw |
| #7 | {OR #1-#6} |
| #8 | MeSH descriptor: [Nitric Oxide] explode all trees |
| #9 | ("nitric oxide"):ti,ab,kw |
| #10 | #8 OR #9 |
| #11 | #7 AND #10 |

Igaku-Chuo-Zasshi (Search date: 2020/7/4)

| #1 | 呼吸窮迫症候群-急性/TH or 急性呼吸窮迫症候群/TA or ARDS/TA |
| --- | --- |
| #2 | 急性肺損傷/TH or 急性肺損傷/TA |
| #3 | #1 or #2 |
| #4 | 一酸化窒素/TH or 一酸化窒素/TA |
| #5 | #3 and #4 |
| #6 | (ランダム化比較試験/TH or 準ランダム化比較試験/TH or ランダム化/AL or 無作為化/AL or 比較試験/AL or 臨床試験/AL or プラセボ/AL or 対照/AL or コントロール/AL or 臨床研究/AL) and (PT=会議録除く) |
| #7 | #5 and #6 |

1. Flow diagram

**Identification**

3 Studies included in qualitative synthesis

31 Full-text articles assessed for eligibility

720 records after duplicates removed

828 records identified through database searching

828 records identified through database searching

Medline via PubMed (n=643)

CENTRAL (n=170)

Igaku-Chuo-Zasshi (n=15)

0 additional records identified through other sources

3 Studies included in quantitative synthesis (meta-analysis)

28 Full-text articles excluded, with reasons:

・Wrong language (n=2)

・Wrong publication type (n=17)

・Wrong population (n=9)

Duplicates

n=108

689 records excluded

**Included**

**Eligibility**

**Screening**

1. Risk of bias


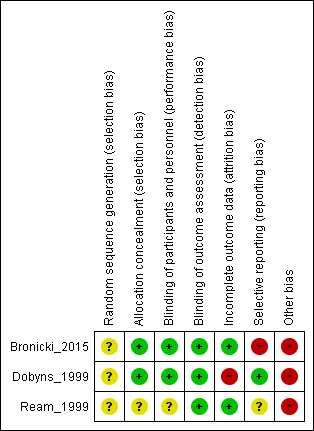
Mortality Ventilator-free days (VFD)


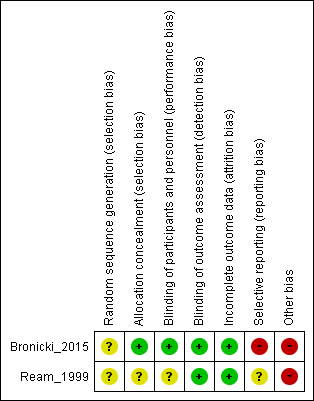


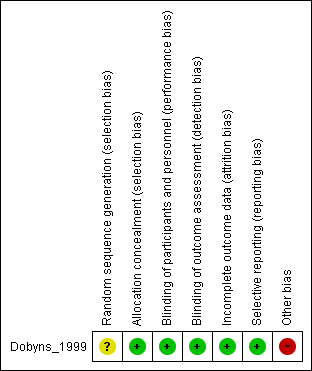
Improvement of oxygenation Severe adverse events


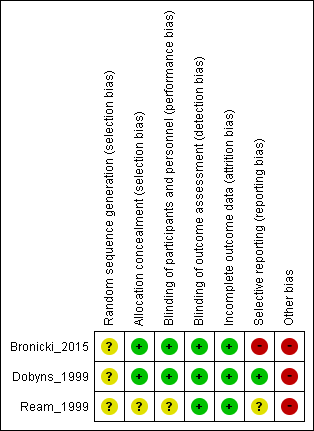


1. Forest plot

Mortality


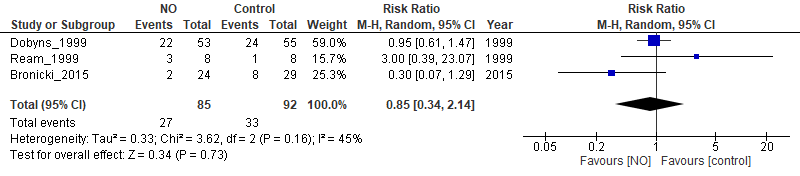


Ventilator-free days (VFD)


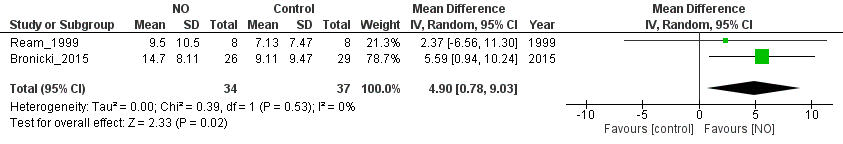


Improvement of oxygenation


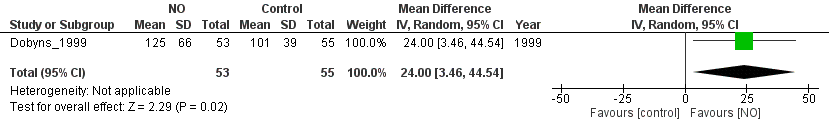


Severe adverse events


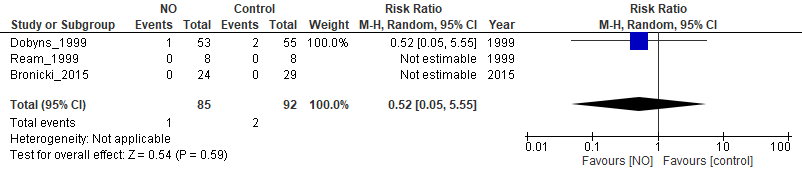


1. Evidence profile

| Assessment of certainty | | | | | | | | | | of patients | | | | Efficacy | | | Certainty of the evidence | Importance |
| --- | --- | --- | --- | --- | --- | --- | --- | --- | --- | --- | --- | --- | --- | --- | --- | --- | --- | --- |
| No. of studies | Study design | Risk of bias | Inconsistency | | Indirectness | | Imprecision | | Others | Nitric Oxide | | Control | | Relative index (95% CI) | | Absolute index (95% CI) |  |  |
| Mortality ^a^ | | | | | | | | | | | | | | | | | | |
| 3 | RCT | Serious ^b^ | | Serious ^c^ | | Not serious | | Serious ^d^ | None | 27/85 (31.8%) | 33/92 (35.9%) | | **RR 0.85** (0.34 to 2.14) | | **-54 per 1,000 patients** (-237 to +409) | | ⨁◯◯◯  Very low | Critical |
| Developmental prognosis | | | | | | | | | | | | | | | | | | |
| 0 | - | - | - | | - | | - | | - | - | | - | | - | | - | - | Critical |
| Ventilator-free days (VFD) | | | | | | | | | | | | | | | | | | |
| 2 | RCT | Serious ^e^ | Not serious | | Not serious | | Serious ^f^ | | None | 34 | | 37 | | - | | **MD 4.90 days longer** (+0.78 to +9.03) | ⨁⨁◯◯  low | Critical |
| Length of hospital stay | | | | | | | | | | | | | | | | | | |
| 0 | - | - | - | | - | | - | | - | - | | - | | - | | - | - | Critical |
| Length of ICU stay | | | | | | | | | | | | | | | | | | |
| 0 | - | - | - | | - | | - | | - | - | | - | | - | | - | - | Important |
| Improvement of oxygenation (P/F ratio at 12 h) | | | | | | | | | | | | | | | | | | |
| 1 | RCT | Serious ^g^ | Not serious | | Not serious | | Serious ^h^ | | None | 53 | | 55 | | - | | **MD 24 higher** (+3.46 to +44.54) | ⨁⨁◯◯  low | Important |
| Severe adverse events | | | | | | | | | | | | | | | | | | |
| 3 | RCT | Serious ^i^ | Not serious | | Not serious | | Serious ^d^ | | None | 1/85 (1.2%) | | 2/92 (2.2%) | | **RR 0.52** (0.05 to 5.55) | | **-10 per 1,000 patients** (-21 to +99) | ⨁⨁◯◯  low | Critical |

CI: confidence interval; RR: relative risk; RCT: randomized controlled trial; ICU: intensive care unit

#### Explanations

1. The mortality rate is the combined 28-day mortality rate and mortality rate with no stated duration.
2. The risk of bias was considered to be “serious” and downgrade by one level, considering a study (Bronicki_2015) with a large sample size was withdrawn, and the fact that there was no significant difference in the patient background; however, there were variations among institutions in the respiratory management and indication of extracorporeal membrane oxygenation (ECMO).
3. The I-squared test is 45%, which was judged to be “serious” and downgraded by one level based on a forest plot of the variability of the results of each study included in the systematic review.
4. The total sample size was 177, which did not meet the optimal information size (OIS) Therefore, it was not considered to have sufficient power. In addition, since the 95% CI crossed the treatment threshold, we judged it to be “very serious” and downgraded it by two levels.
5. The study (Bronicki_2015) with large sample size, was judged as “serious” and downgraded by one level because there was no significant difference in the patient background; however, there were variations among institutes in respiratory management and an indication of ECMO.
6. The total sample size was 71, which did not meet the OIS. Therefore, it was not considered to have sufficient power and it was judged to be 'serious' and downgraded by one level.
7. While the data of pH is within the normal range compared to the severity, the data of PaCO_2_ is quite different. There are some items for which the numbers are too large even though they are reported as SEM. Therefore, it was judged as “serious” and downgraded by one level.
8. The total sample size is 108, which does not meet the OIS. Therefore, it is not considered to have sufficient power and it was judged to be "serious" and downgraded by one level.
9. The risk of bias was judged to be “serious” and was downgraded by one level because the research (Bronicki_2015) was withdrawn.
10. No studies reported outcomes for developmental prognosis, length of hospital stay, or ICU stay.
11. Evidence-to-profile

| **QUESTION** | |
| --- | --- |
| **PCQ11：** Should nitric oxide (NO) inhalation therapy be used in pediatric patients with ARDS? | |
| **POPULATION:** | Pediatric patients (as defined in the article, 20 years old or younger if not specified) on ventilators for ARDS (as defined in the article) |
| **INTERVENTION:** | Inhaled nitric oxide (iNO) |
| **COMPARISON:** | No iNO |
| **MAIN OUTCOMES:** | Mortality, Developmental prognosis, Ventilator-free days (VFD), Length of hospital stay, Severe adverse events |
| **SETTINGS:** | Emergency department or intensive care unit (ICU) |
| **PERSPECTIVE:** | Individual |
| **BACKGROUND:** | ARDS is caused by inflammation that spreads to the lungs from a variety of sources. Various factors are involved in its pathogenesis, including damage to the alveolar epithelium, increased pulmonary vascular resistance due to hypoxic pulmonary vasoconstriction, imbalance in the ventilation-perfusion ratio, and alveolar surfactant dysfunction. Therefore, iNO, which has a pulmonary vasodilator effect, is considered effective. Some studies demonstrated improved oxygenation after iNO use. Other studies have reported that iNO does not decrease the mortality or the duration of mechanical ventilation. Considering these inconsistencies, a systematic review of iNO is important to address this clinical issue. |
| **CONFLICT OF INTERESTS:** | None |

**ASSESSMENT**

| **Problem**  Is the problem a priority? | | |
| --- | --- | --- |
| **Judgement** | **Research evidence** | **Additional considerations** |
| ○ No  ○ Probably no  ○ Probably yes  ● Yes  ○ Varies  ○ Do not know | ARDS is a disease caused by inflammation that spreads to the lungs from a variety of sources. Various factors are involved in its pathogenesis, including damage to the alveolar epithelium, increased pulmonary vascular resistance due to hypoxic pulmonary vasoconstriction, imbalance in the ventilation-perfusion ratio, and alveolar surfactant dysfunction. Therefore, iNO, which has a pulmonary vasodilator effect, is considered effective. Some studies demonstrated improved oxygenation after iNO use. Other studies have reported that iNO does not decrease the mortality or the duration of mechanical ventilation. Considering these inconsistencies, a systematic review of iNO is important to address this clinical issue. |  |
| **Desirable Effects**  How substantial are the desirable anticipated effects? | | |
| **Judgement** | **Research evidence** | **Additional considerations** |
| ○ Trivial  ● Small  ○ Moderate  ○ Large  ○ Varies  ○ Do not know | Three randomized controlled trials (RCTs)^1-3^ consistent with PICO were included in the systematic review and used in the meta-analysis.  As beneficial outcomes, the estimated short-term effect on mortality (3 RCTs ^1-3^, N=177) was 54 fewer deaths per 1,000 (95% confidence interval [CI]: 237 fewer to 409 more) and the VFD_28_ (2 RCTs ^1,3^, N=71) was on average 4.90 days longer (95% CI: 0.78 longer to 9.03 longer) in the intervention group than in the control group. Clinically meaningful benefits could be achieved even though the 95% CIs were wide while there was a potential increase in mortality of 409 per 1,000. The expected desirable effect was considered "small”. |  |
| **Undesirable Effects**  How substantial are the undesirable anticipated effects? | | |
| **Judgement** | **Research evidence** | **Additional considerations** |
| ○ Large  ○ Moderate  ○ Small  ● Trivial  ○ Varies  ○ Do not know | As a harmful outcome, the estimate of severe adverse events (3 RCTs 1-3, N=177) was 10 fewer per 1,000 (95% CI: 21 fewer to 99 more) in the intervention group than in the control group. However, since the intervention could potentially increase severe adverse events by 99 per 1,000, the expected undesirable effect was considered "trivial.” |  |
| **Certainty of evidence**  What is the overall certainty of the evidence of effects? | | |
| **Judgement** | **Research evidence** | **Additional considerations** |
| ○ Very low  ● Low  ○ Moderate  ○ High  ○ No included studies | **The relative importance or values of the main outcomes of interest:**   \| **Outcome** \| **Importance** \| **Certainty of the Evidence**  **(GRADE)** \| \| --- \| --- \| --- \| \| Mortality \| Critical \| ⨁◯◯◯  Very low \| \| Developmental prognosis \| Critical \| ‐ \| \| VFD_28_ \| Critical \| ⨁⨁◯◯ Low \| \| Length of hospital stay \| Critical \| ‐ \| \| Severe adverse events (acute kidney injury, hemodynamic deterioration, pulmonary hemorrhage) \| Critical \| ⨁⨁◯◯ Low \|   **Overall certainty of evidence**  The desirable effects were a reduction in mortality by 54 per 1,000 and an increase in VFD by 4.90 days. The certainty of evidence for these effects were "very low" and "low," respectively. - The undesirable effect, in contrast, was a reduction in serious adverse events by 10 per 1,000, with a "low” certainty of evidence. Therefore, since all directions of the desirable and undesirable effects were consistent, the highest certainty of evidence for the overall outcome ("low") was adopted. |  |
| **Values**  Is there important uncertainty about or variability in how much people value the main outcomes? | | |
| **Judgement** | **Research evidence** | **Additional considerations** |
| ○ Important uncertainty or variability  ○ Possibly important uncertainty or variability  ○ Probably no important uncertainty or variability  ● No important uncertainty or variability | “Mortality” is generally a critical outcome. There was no significant uncertainty or diversity of values for this outcome. |  |
| **Balance of effects**  Does the balance between desirable and undesirable effects favor the intervention or the comparison? | | |
| **Judgement** | **Research evidence** | **Additional considerations** |
| ○ Favors the comparison  ○ Probably favors the comparison  ○ Does not favor either the intervention or the comparison  ● Probably favors the intervention  ○ Favors the intervention  ○ Varies  ○ Do not know | **Summary of evidence:**   \| **Outcome** \| **Control** \| **Intervention** \| **Absolute difference**  **(95% CI)** \| **Risk ratio (RR)**  **(95% CI)** \| \| --- \| --- \| --- \| --- \| --- \| \| Mortality \| 33/92  (35.9%) \| 27/85  (31.8%) \| 54 fewer per 1,000 (237 fewer to 409 more) \| RR 0.85 (0.34 to 2.14) \| \| Developmental prognosis \| ‐ \| ‐ \| ‐ \| ‐ \| \| VFD_28_ \| ‐ \| ‐ \| MD 4.90 day longer (0.78 longer to 9.03 longer) \| ‐ \| \| Length of hospital stay \| ‐ \| ‐ \| ‐ \| ‐ \| \| Severe adverse events \| 2/92  (2.2%) \| 1/85  (1.2%) \| 10 fewer per 1,000 (21 fewer to 99 more) \| RR 0.52 (0.05 to 5.55) \|   Based on the aforementioned findings, we determined that the balance between desirable and undesirable effects was "probably favors the intervention”. |  |
| **Acceptability**  Is the intervention acceptable to key stakeholders? | | |
| **Judgement** | **Research evidence** | **Additional considerations** |
| ○ No  ○ Probably no  ● Probably yes  ○ Yes  ○ Varies  ○ Do not know | Although no evidence has been reviewed for acceptability, we estimate that patients and patient families would probably accept the intervention. |  |
| **Feasibility**  Is the intervention feasible to implement? | | |
| **Judgement** | **Research evidence** | **Additional considerations** |
| ○ No  ○ Probably no  ○ Probably yes  ○ Yes  ● Varies  ○ Do not know | Implementing this intervention would require investment in inhalation equipment, which is likely difficult for many facilities across the country. In addition, since it is not covered by insurance, it requires approval from the institutional ethics committee. In contrast, in pediatric intensive care units, iNO is standard care for other diseases (e.g. neonatal pulmonary hypertension), and resources are often available. Overall, the feasibility of the intervention varies from facility to facility. |  |

**Summary of Judgement**

|  | **JUDGMENT** | | | | | | |
| --- | --- | --- | --- | --- | --- | --- | --- |
| **PROBLEM** | No | Probably no | Probably yes | **Yes** |  | Varies | Do not know |
| **DESIRABLE EFFECTS** | Trivial | **Small** | Moderate | Large |  | Varies | Do not know |
| **UNDESIRABLE EFFECTS** | Large | Moderate | Small | **Trivial** |  | Varies | Do not know |
| **CERTAINTY OF EVIDENCE** | Very low | **Low** | Moderate | High |  |  | No included studies |
| **VALUES** | Important uncertainty or variability | Possibly important uncertainty or variability | Probably no important uncertainty of variability | **No important uncertainty of variability** |  |  |  |
| **BALANCE OF EFFECTS** | Favors the comparison | Probably favors the comparison | Does not favor either the intervention or the comparison | **Probably favors the intervention** | Favors the intervention | Varies | Do not know |
| **ACCEPTABILITY** | No | Probably no | **Probably yes** | Yes |  | Varies | Do not know |
| **FEASIBILITY** | No | Probably no | Probably yes | Yes |  | **Varies** | Do not know |

**Type of Recommendation**

| Strong recommendation against the intervention | Conditional recommendation against the intervention | Conditional recommendation for either the intervention or the comparison | Conditional recommendation for the intervention | Strong recommendation for the intervention |
| --- | --- | --- | --- | --- |
| ○ | ● | ○ | ○ | ○ |

**CONCLUSION**

| **Recommendation** |
| --- |
| We suggest against routinely implementing NO inhalation therapy for pediatric patients with ARDS (weak recommendation / very low certainty of evidence：GRADE 2C）.  Supplementary item:  This treatment can be acceptable in limited circumstances. Examples include temporary use until the introduction of extracorporeal membrane oxygenation, and situations where there are no other treatments and a high mortality rate is predicted. It should be noted that NO inhalation therapy for ARDS is not covered by insurance. |
|  |
| **Justification** |
| **Clinical Question:** Should NO inhalation therapy be used in pediatric patients with ARDS?  **Population:** Pediatric patients (as defined in the article, 20 years old or younger if not specified) on ventilators for ARDS (as defined in the article)  **Intervention:** Inhaled nitric oxide  **Outcomes:** Mortality, VFD^28^_,_ Severe adverse events  **Summary of evidence:**  The meta-analysis for the included three randomized controlled trials (RCTs)^1-3^ showed that as beneficial outcomes, the estimated short-term effect on mortality (3 RCTs ^1-3^, N=177) was 54 fewer deaths per 1,000 (95% confidence interval [CI]: 237 fewer to 409 more) and the VFD_28_ (2 RCTs ^1,3^, N=71) was on average 4.90 days longer (95% CI: 0.78 longer to 9.03 longer) in the intervention group. Clinically meaningful benefits could be achieved even though the 95% CIs were wide while there was a potential increase in mortality of 409 per 1,000. The expected desirable effect was considered "small”.  As a harmful outcome, the estimate of severe adverse events (3 RCTs 1-3, N=177) was 10 fewer per 1,000 (95% CI: 21 fewer to 99 more) in the intervention group. However, since the intervention could potentially increase severe adverse events by 99 per 1,000, the expected undesirable effect was considered "trivial.”  **Quality of evidence:**  Since all directions of the desirable and undesirable effects were consistent, the highest certainty of evidence for the overall outcome ("low") was adopted.  **Balance of effects, acceptability, Feasibility:**  Based on the findings of the meta-analysis, we determined that the balance between desirable and undesirable effects was "probably favors the intervention”. Though, given the uncertainty of the expected net benefit and the limited feasibility of the implementation of the NO inhalation equipment, the recommendation was decided as “against using NO inhalation routinely”.  **Panel meeting:**  In a pre-ballot, the agreed upon recommendation was: "We suggest against routinely implementing NO inhalation therapy for pediatric patients with ARDS (weak recommendation / very low certainty of evidence：GRADE 2C）. ” with supplementary items: “This treatment can be acceptable in limited circumstances. Examples include temporary use until the introduction of extracorporeal membrane oxygenation, and situations where there are no other treatments and a high mortality rate is predicted. It should be noted that NO inhalation therapy for ARDS is not covered by insurance.” This recommendation was agreed upon by the panel with a median score of 7.0 and a disagreement index of 0.3738 using the modified Delphi method. A re-vote was held at the panel meeting due to minor revisions to the wording in the supplementary items. Using the modified Delphi method, a consensus was reached with a median score of 7.0 and a disagreement index of 0.3738.  **Additional considerations:**  The therapy may be rationalized in limited conditions including the short-term use of iNO to stabilize patients until ECMO was commenced, and cases with progressive ARDS regardless of all other available therapies cases. As iNO is not covered by insurance, it is recommended to obtain an approval from the institutional ethics committee priori. |

| **Subgroup considerations** |
| --- |
| None. |
| **Implementation considerations** |
| When introducing iNO, it is desirable to introduce the therapy in ICUs in which therapy-related adverse events (renal dysfunction, circulatory compromise, pulmonary hemorrhage, etc.) could be handled and children requiring the rescue therapy for pediatric ARDS have been cared (i.e. Pediatric intensive care units). This also indicates the need for the pediatric-specialized medical transport service to transfer critically-ill children to the tertiary center.  One of the complications of this intervention is methemoglobinemia, which should be monitored with blood gas analysis. In addition, this intervention for ARDS is not covered by insurance and requires approval from the institutional ethics committee. |

| **Monitoring and evaluation** |
| --- |
| Mortality, therapy-related adverse events, costs, reduction in the ECMO use, and need for interhospital transport should be monitored in a registry. In particular, acute kidney injury associated with the therapy should be monitored since it has not been adequately assessed in the RCTs we have evaluated. |
| **Research priorities** |
| Two of the three studies included in the meta-analysis were conducted before this lung protection strategy became the standard care. Therefore, the effect of iNO in the modern era with the standardized ventilation strategy for ARDS has not been examined. Recently, muscle relaxants, prone positioning, and ECMO have been increasingly used in children with ARDS. Though, there is insufficient data to rank iNO among these supportive therapies. Considering these findings, we suggest that future studies should focus on the population that is expected to benefit from iNO, such as patients with right heart failure and patients who respond to iNO. |

Reference

1. Dobyns EL, Cornfield DN, Anas NG, et al. Multicenter randomized controlled trial of the effects of inhaled nitric oxide therapy on gas exchange in children with acute hypoxemic respiratory failure. The Journal of pediatrics 1999;134:406-12.

2. Ream RS, Hauver JF, Lynch RE, Kountzman B, Gale GB, Mink RB. Low-dose inhaled nitric oxide improves the oxygenation and ventilation of infants and children with acute, hypoxemic respiratory failure. Critical care medicine 1999;27:989-96.

3. Bronicki RA, Fortenberry J, Schreiber M, Checchia PA, Anas NG. Multicenter randomized controlled trial of inhaled nitric oxide for pediatric acute respiratory distress syndrome. The Journal of pediatrics 2015;166:365-9.e1.

**PCQ12 Should surfactant be used in pediatric patients with ARDS?**

1.Search strategy

MEDLINE via PubMed (Search date: 2020/7/3）

| #1 | "respiratory distress syndrome, adult"[mh] OR "respiratory distress syndrome, newborn"[mh] OR respiratory distress syndrome[tiab] OR ARDS[tiab] |
| --- | --- |
| #2 | "lung injury"[mh] OR "lung injur*"[tiab] |
| #3 | “respiratory failure”[tiab] |
| #4 | #1 OR #2 OR #3 |
| #5 | "Surface-Active Agents"[mh] OR "Surface Active Agents"[tiab] OR tensides[tiab] OR "amphiphilic agent"[tiab] |
| #6 | "Pulmonary surfactants"[mh] OR Surfactant*[tiab] |
| #7 | poractant*[tiab] OR curosurf[tiab] OR "poractant alfa" [Supplementary Concept] |
| #8 | beractant*[tiab] OR "survanta"[tiab] OR "beractant" [Supplementary Concept] |
| #9 | "calfactant*" [tiab] OR "calfactant" [Supplementary Concept] |
| #10 | infasurft*[tiab] |
| #11 | venticute*[tiab] OR "Venticute" [Supplementary Concept] |
| #12 | exosurf[tiab] OR "dipalmitoylphosphatidylcholine, hexadecanol, tyloxapol drug combination" [Supplementary Concept] |
| #13 | alec[tiab] OR "artificial lung expanding compound" [Supplementary Concept] |
| #14 | #5 OR #6 OR #7 OR #8 OR #9 OR #10 OR #11 OR #12 OR #13 |
| #15 | #4 AND #14 |
| #16 | adult[mh] NOT child[mh] |
| #17 | #15 NOT #16 |
| #18 | “randomized controlled trial”[pt] OR “controlled clinical trial”[pt] OR randomized[tiab] OR placebo[tiab] OR randomly[tiab] OR trial[tiab] OR groups[tiab] NOT (animals [mh] NOT humans [mh]) |
| #19 | #17 AND #18 |

CENTRAL (Search date: 2020/7/3）

| #1 | [mh "respiratory distress syndrome, adult"] OR [mh "respiratory distress syndrome, newborn"] OR "respiratory distress syndrome":ti,ab OR ARDS:ti,ab |
| --- | --- |
| #2 | [mh "lung injury"] OR "lung injury":ti,ab |
| #3 | "respiratory failure":ti,ab |
| #4 | {OR #1-#3} |
| #5 | [mh "Surface-Active Agents"] OR "Surface Active Agents":ti,ab OR tensides:ti,ab OR "amphiphilic agent":ti,ab |
| #6 | [mh "Pulmonary surfactants"] OR Surfactant:ti,ab OR Surfactants:ti,ab |
| #7 | poractant:ti,ab OR curosurf:ti,ab |
| #8 | beractant*:ti,ab OR survanta:ti,ab |
| #9 | calfactant:ti,ab |
| #10 | exosurf:ti,ab |
| #11 | alec:ti,ab OR "artificial lung expanding compound":ti,ab |
| #12 | {OR #5-#11} |
| #13 | #4 AND #12 |
| #14 | [mh animals] NOT [mh humans] |
| #15 | #13 NOT #14 |

Igaku Chuo Zasshi (Search date: 2020/7/3）

| #1 | 呼吸窮迫症候群-急性/TH or 急性呼吸窮迫症候群/AL or ARDS/AL or ショック肺/AL |
| --- | --- |
| #2 | 急性肺損傷/TH or 急性肺損傷/AL |
| #3 | 呼吸不全/TH or 呼吸不全/AL |
| #4 | 重症急性呼吸器症候群/TH or SARS/AL |
| #5 | #1 or #2 or #3 or #4 |
| #6 | 界面活性剤/TH or 界面活性剤/AL or サーファクタント/AL |
| #7 | Pulmonary/AL and surfactant/AL |
| #8 | poractant*/AL or curosurf/AL or ボラクタント/AL |
| #9 | beractant/AL or Beractant/TH or survanta/AL or ベラクタント/AL |
| #10 | Calfactant/TH or calfactant/AL or カルファクタント/AL |
| #11 | Exosurf/TH or exosurf/AL |
| #12 | Alectinib/TH or Alectinib/AL or アレセンサ/AL or アレクチニブ/AL or (artificial/AL and (肺/TH or lung/AL) and expanding/AL and compound/AL) |
| #13 | #6 or #7 or #8 or #9 or #10 or #11 or #12 |
| #14 | #5 and #13 |
| #15 | (CK=成人(19～44),中年(45～64),高齢者(65～)) not (CK=新生児,乳児(1～23ヶ月),幼児(2～5),小児(6～12),青年期(13～18)) |
| #16 | #14 not #15 |
| #17 | (ランダム化比較試験/TH or ランダム化/AL or 無作為化/AL) or (比較試験/AL) or (臨床試験/TH or 臨床試験/AL) or (プラセボ/TH or プラセボ/AL) or (対照/AL) or (コントロール/AL) or (臨床研究・疫学研究/TH or 臨床研究/AL) |
| #18 | #16 and #17 |

1. Flow diagram

**Identification**

9 Studies included in qualitative synthesis

54 Full-text articles assessed for eligibility

1,905 records after duplicates removed

2,500 records identified through database searching

2,500 records identified through database searching

Medline via PubMed (n=1,405)

CENTRAL (n=1,052)

Igaku-Chuo-Zasshi (n=43)

0 additional records identified through other sources

9 Studies included in quantitative synthesis (meta-analysis)

45 Full-text articles excluded, with reasons:

・Wrong language (n=3)

・Wrong study design (n=1)

・Wrong publication type (n=27)

・Wrong population (n=14)

Duplicates

n=595

1,851 records excluded

**Included**

**Eligibility**

**Screening**

1. Risk of bias


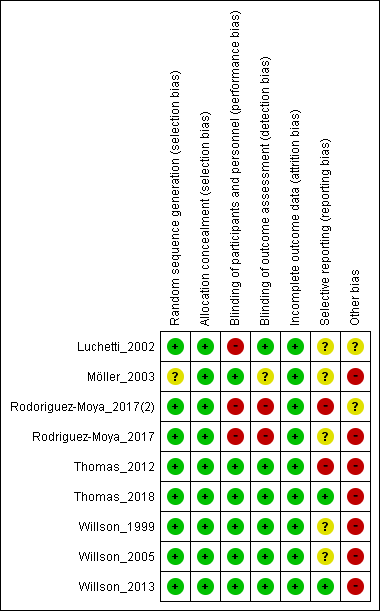
Mortality Ventilator-free days (VFD)


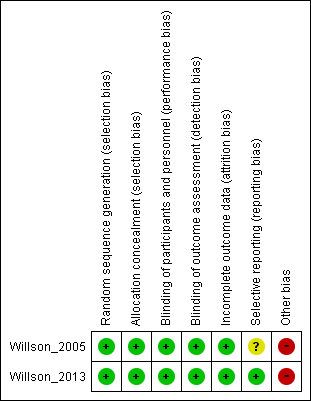


Length of hospital stat Length of ICU stay


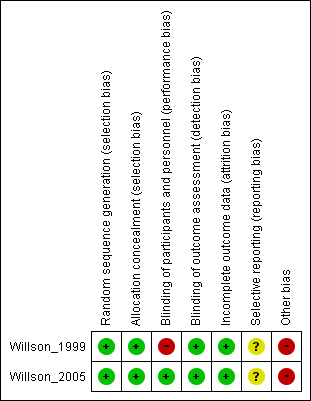

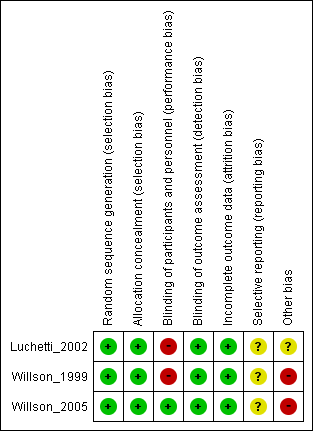


Drug-related adverse evnets


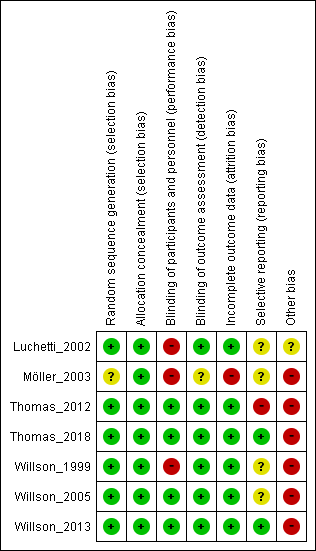


1. Forest plot

Mortality


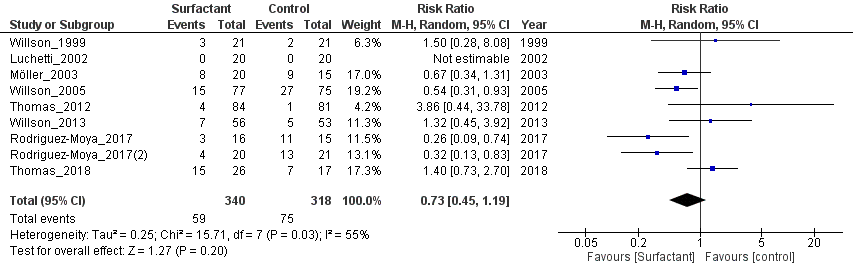


Ventilator-free days (VFD)


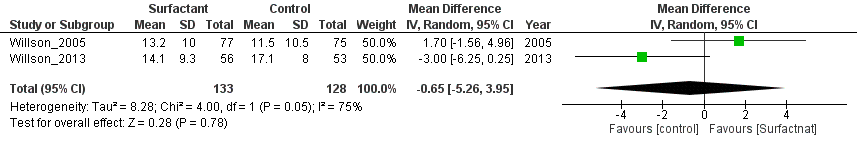


Length of hospital stay


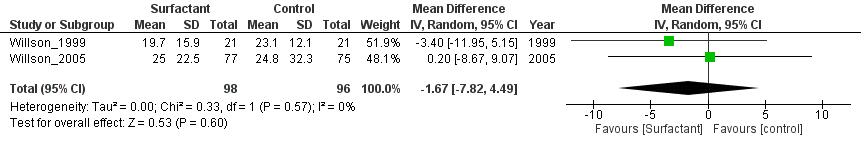


Length of ICU stay


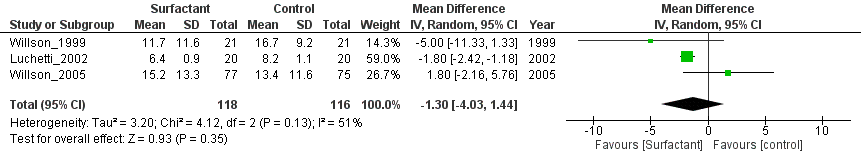


Drug-related adverse evnets


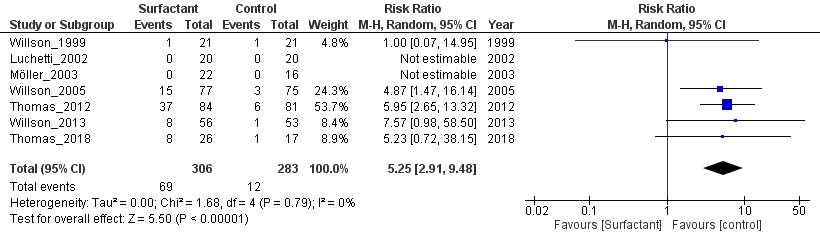


1. Evidence profile

| Assessment of certainty | | | | | | | No of patients | Efficacy | | | Certainty of the evidence | Importance |
| --- | --- | --- | --- | --- | --- | --- | --- | --- | --- | --- | --- | --- |
| No of studies | Study design | Risk of bias | Inconsistency | Indirectness | Imprecision | Others | Surfactant | Control | Relative index (95% CI) | Absolute index (95% CI) |  |  |
| **Mortality** ^a^ | | | | | | | | | | | | |
| 9 | RCT | Serious ^b^ | Serious ^c^ | Not serious | Serious ^d^ | None | 59/340 (17.4%) | 75/318 (23.6%) | **RR 0.73** (0.45 to 1.19) | **-64 per 1,000 patients** (-130 to +45) | ⨁◯◯◯  Very low | Critical |
| **Developmental prognosis** | | | | | | | | | | | | |
| 0 | - | - | - | - | - | - | - | - | - | - | - | Critical |
| **Ventilator-free days (VFD)** | | | | | | | | | | | | |
| 2 | RCT | Serious ^e^ | Serious ^f^ | Not serious | Very Serious ^g^ | None | 133 | 128 | - | **MD**  **0.65 day shorter** (-5.26 to +3.95) | ⨁◯◯◯  Very low | Critical |
| **Length of hospital stay** | | | | | | | | | | | | |
| 2 | RCT | Serious ^h^ | Not serious | Not serious | Very Serious ^i^ | None | 98 | 96 | - | **MD 1.67 day shorter** (-7.82 to +4.49) | ⨁◯◯◯  Very low | Critical |
| **Length of ICU stay** | | | | | | | | | | | | |
| 3 | RCT | Serious ^j^ | Serious ^k^ | Not serious | Very Serious ^l^ | None | 118 | 116 | - | **MD 1.3 day shorter** (-4.03 to +1.44) | ⨁◯◯◯  Very low | Important |
| **Improvement of oxygenation** | | | | | | | | | | | | |
| 0 | - | - | - | - | - | - | - | - | - | - | - | Important |
| **Drug-related adverse events** | | | | | | | | | | | | |
| 7 | RCT | Serious ^m^ | Not serious | Not serious | Serious ^n^ | None | 69/306 (22.5%) | 12/283 (4.2%) | **RR 5.25** (2.91 to 9.48) | **+180 per 1,000 patients** (+81 to +360) | ⨁⨁◯◯  low | Critical |

**CI:** confidence interval; **RCT**: randomized controlled trial; **RR**: relative risk

**Explanations**

1. Mortality is the combined 48-h, 14-day, 28-day, 30-day, 90-day, and 120-day mortality rates.
2. In the study by Thomas et al. 2012, pertussis was added to the exclusion criteria in the middle of the study. It is judged to be "serious" and downgraded by one level because it did not respond to surfactants and had a high mortality rate.
3. The I-squared test was 53%, which was judged to be "serious" and downgraded by one level based on a forest plot of the variability of the results of each study included in the systematic review.
4. The total sample size is 658, which does not meet the optimal information size (OIS). Additionally, since the 95% CI crossed the treatment threshold for benefit, we judged it to be "serious" and downgraded it by one level.
5. As the first author received a grant from a pharmaceutical company for calfactant, it was judged as "serious" and downgraded by one level.
6. The I square test was 75%, which was judged to be "serious" and downgraded by one level based on a forest plot of the variability of the results of each study included in the systematic review.
7. The total sample size is 261, which does not meet the OIS. Additionally, since the 95% CI straddles the treatment thresholds for benefit and harm, we judged it to be "very serious" and downgraded it by two levels.
8. Block randomization was performed in unblinded clinical trials. Unscheduled interim analysis and sample size review was performed. As a result, it was judged as "serious" and downgraded by one level.
9. The total sample size is 194, which does not meet the OIS. Therefore, it is not considered to have sufficient power and it was judged to be "serious" and downgraded by one level.
10. Since the treatment provider was not blinded, it was judged as "serious" and downgraded by one level.
11. The I-squared test was 51%, which was judged to be "serious" and downgraded by one level based on a forest plot of the variability of the results of each study included in the systematic review.
12. The total sample size was 234, which did not meet the OIS. Therefore, it was not considered to have sufficient power, and it was judged as "serious" and downgraded by one level.
13. Owing to a large number of unblinded studies, they were judged as "serious" and downgraded by one level.
14. The total sample size is 589, which does not meet the OIS. Additionally, since the 95% CI did not straddle the treatment threshold for benefit and harm, we judged it to be "serious" and downgraded it by one level.
15. No studies reported developmental prognosis or improvement associated with oxygenation.
16. Evidence-to-Decision table

| **QUESTION** | |
| --- | --- |
| **PCQ12：** Should surfactant be used in pediatric patients with ARDS? | |
| **POPULATION:** | Pediatric patients (as defined in the article, 20 years old or younger if not specified) on ventilators for ARDS (as defined in the article) |
| **INTERVENTION:** | Administer surfactant |
| **COMPARISON:** | No surfactant administered |
| **MAIN OUTCOMES:** | Mortality, Developmental prognosis, Ventilator-free days (VFD), Length of hospital stay, Drug-related adverse events |
| **SETTINGS:** | Emergency department or intensive care unit (ICU) |
| **PERSPECTIVE:** | Individual |
| **BACKGROUND:** | Acute respiratory distress syndrome (ARDS) is a disease caused by lung inflammation from a variety of sources. Various factors are involved in its pathogenesis, including damage to the alveolar epithelium, increased pulmonary vascular resistance due to hypoxic pulmonary vasoconstriction, imbalance in the ventilation-perfusion ratio, and dysfunction of alveolar surfactants. Some researchers have reported that the administration of surfactants improves oxygenation and shortens the duration of mechanical ventilation. Others have reported that surfactant administration does not improve oxygenation, duration of mechanical ventilation, or mortality. Therefore, a systematic review of surfactant administration is an important clinical issue. |
| **CONFLICT OF INTERESTS:** | None |

**ASSESSMENT**

| **Problem**  Is the problem a priority? | | |
| --- | --- | --- |
| **Judgement** | **Research evidence** | **Additional considerations** |
| ○ No  ○ Probably no  ○ Probably yes  ● Yes  ○ Varies  ○ Do not know | ARDS is a disease caused by inflammation that spreads to the lungs from a variety of sources. Various factors are involved in its pathogenesis, including damage to the alveolar epithelium, increased pulmonary vascular resistance due to hypoxic pulmonary vasoconstriction, imbalance in the ventilation-perfusion ratio, and dysfunction of alveolar surfactants. Some researchers have reported that the administration of surfactants improves oxygenation and shortens the duration of mechanical ventilation. Others have reported that surfactant administration does not improve oxygenation, duration of mechanical ventilation, or mortality. Therefore, conducting a systematic review of surfactant administration is an important clinical issue with high priority. |  |
| **Desirable Effects**  How substantial are the desirable anticipated effects? | | |
| **Judgement** | **Research evidence** | **Additional considerations** |
| ○ Trivial  ● Small  ○ Moderate  ○ Large  ○ Varies  ○ Do not know | Nine randomized controlled trials (RCTs)^1-9^ consistent with PICO were included in the systematic review and used in the meta-analysis.  As beneficial outcomes, the estimate of effect for mortality (9 RCTs^1-9^, N=658) was 64 fewer per 1,000 (95% CI: 130 fewer to 45 more); for the 28-day VFD (2 RCTs^4,6,^ N=261), it has a mean difference of 0.65 days shorter (95% CI: 5.26 shorter to 3.95 longer); for length of hospital stay (2 RCTs^1,4^, N=194), it has a mean difference of 1.67 days shorter (95% CI: 7.82 shorter to 4.49 longer) in the intervention group compared with that in the control group. Therefore, the expected desirable effect was judged to be "small." |  |
| **Undesirable Effects**  How substantial are the undesirable anticipated effects? | | |
| **Judgement** | **Research evidence** | **Additional considerations** |
| ○ Large  ○ Moderate  ● Small  ○ Trivial  ○ Varies  ○ Do not know | As a harmful outcome, the estimate of the effect of drug-related adverse effects (7 RCTs^1-6, 9^, N=589) was 180 more per 1,000 (95% CI: 81 more to 360 more) in the intervention group than that in the control group. Since most of the drug-related adverse effects were transient hypoxemia and hemodynamic changes, which were not serious, the undesirable effect was judged to be "small.” |  |
| **Certainty of evidence**  What is the overall certainty of the evidence of effects? | | |
| **Judgement** | **Research evidence** | **Additional considerations** |
| ● Very low  ○ Low  ○ Moderate  ○ High  ○ No included studies | **The relative importance or values of the main outcomes of interest:**   \| **Outcome** \| **Importance** \| **Certainty of the Evidence**  **(GRADE)** \| \| --- \| --- \| --- \| \| Mortality \| Critical \| ⨁◯◯◯ Very low \| \| Developmental prognosis* \| Critical \| - \| \| VFD \| Critical \| ⨁◯◯◯ Very low \| \| Length of hospital stay \| Critical \| ⨁◯◯◯ Very low \| \| Drug-related adverse events (bronchoconstriction, pneumothorax, transient hypoxemia, and transient changes in circulatory dynamics, etc) \| Critical \| ⨁⨁◯◯ Low \|   * There was no outcome report on developmental prognosis in the accepted literature.  **Overall certainty of evidence**  The desirable effect was the reduction in mortality by 64 per 1,000, the decrease in VFD and length of hospital stay by 0.65 days and 1.67 days, respectively. The certainty of evidence was "very low," "very low," and "very low," respectively. On the other hand, the undesirable effect was the increase in drug-related adverse events by 180 per 1,000, and the certainty of evidence was "low.” Since the directions of the desirable and undesirable effects did not match, the lowest certainty of evidence for the overall outcome, "very low," was adopted. |  |
| **Values**  Is there important uncertainty about or variability in how much people value the main outcomes? | | |
| **Judgement** | **Research evidence** | **Additional considerations** |
| ○ Important uncertainty or variability  ○ Possibly important uncertainty or variability  ○ Probably no important uncertainty or variability  ● No important uncertainty or variability | “Mortality” is generally a critical outcome, and there is no uncertainty or diversity of values regarding this. |  |
| **Balance of effects**  Does the balance between desirable and undesirable effects favor the intervention or the comparison? | | |
| **Judgement** | **Research evidence** | **Additional considerations** |
| ○ Favors the comparison  ○ Probably favors the comparison  ● Does not favor either the intervention or the comparison  ○ Probably favors the intervention  ○ Favors the intervention  ○ Varies  ○ Do not know | **Summary of evidence:**   \| **Outcome** \| **Control** \| **Intervention** \| **Absolute difference**  **(95% CI)** \| **Risk ratio (RR)**  **(95% CI)** \| \| --- \| --- \| --- \| --- \| --- \| \| Mortality \| 75/318  (23.6%) \| 59/340  (17.4%) \| 64 fewer per 1,000 (130 fewer to 45 more) \| RR 0.73  (0.45 to 1.19) \| \| Developmental prognosis \| - \| - \| - \| - \| \| VFD \| - \| - \| MD 0.65 day shorter (5.26 shorter to 3.95 longer) \| - \| \| Length of hospital stay \| - \| - \| MD 1.67 day shorter  (7.82 shorter to 4.49 longer) \| - \| \| Drug-related adverse events \| 12/283  (4.2%) \| 69/306  (22.5%) \| 180 more per 1,000 (81 more to 360 more) \| RR 5.25  (2.91 to 9.48) \|   Based on the above, we determined that the balance between desirable and undesirable effects was "Does not favor either the intervention or control.” |  |
| **Acceptability**  Is the intervention acceptable to key stakeholders? | | |
| **Judgement** | **Research evidence** | **Additional considerations** |
| ○ No  ● Probably no  ○ Probably yes  ○ Yes  ○ Varies  ○ Do not know | Although there is no evidence to examine the acceptability of surfactants, we decided that it would probably be an unacceptable treatment for patients and their families due to the competing desirable and undesirable effects and the increased side effects secondary to the drug. |  |
| **Feasibility**  Is the intervention feasible to implement? | | |
| **Judgement** | **Research evidence** | **Additional considerations** |
| ○ No  ● Probably no  ○ Probably yes  ○ Yes  ○ Varies  ○ Do not know | Cases have been reported that required the use of resuscitation drugs and ventilator adjustments to deal with drug-related adverse events. It is desirable to administer surfactant in ICUs staffed by intensivists experienced in the use of surfactants. Since the number of facilities that meet these requirements is limited, it is "probably difficult" to administer surfactants for ARDS. In addition, surfactants are expensive drugs. Since this intervention is not covered by insurance for ARDS, if it is to be used, it is preferred to have it approved by the ethics committee of the institution, including how to explain the therapy to the family. | The drug price for one vial of alveolar surfactant, 120 mg, was 76488.3 yen. The insured dose for neonatal respiratory distress syndrome is 60–120 mg/kg. In a pediatric ARDS study, approximately 100 mg/kg was administered. |

**Summary of Judgement**

|  | **JUDGMENT** | | | | | | |
| --- | --- | --- | --- | --- | --- | --- | --- |
| **PROBLEM** | No | Probably no | Probably yes | **Yes** |  | Varies | Do not know |
| **DESIRABLE EFFECTS** | Trivial | **Small** | Moderate | Large |  | Varies | Do not know |
| **UNDESIRABLE EFFECTS** | Large | Moderate | **Small** | Trivial |  | Varies | Do not know |
| **CERTAINTY OF EVIDENCE** | **Very low** | Low | Moderate | High |  |  | No included studies |
| **VALUES** | Important uncertainty or variability | Possibly important uncertainty or variability | Probably no important uncertainty of variability | **No important uncertainty of variability** |  |  |  |
| **BALANCE OF EFFECTS** | Favors the comparison | Probably favors the comparison | **Does not favor either the intervention or the comparison** | Probably favors the intervention | Favors the intervention | Varies | Do not know |
| **ACCEPTABILITY** | No | **Probably no** | Probably yes | Yes |  | Varies | Do not know |
| **FEASIBILITY** | No | **Probably no** | Probably yes | Yes |  | Varies | Do not know |

**Type of Recommendation**

| Strong recommendation against the intervention | Conditional recommendation against the intervention | Conditional recommendation for either the intervention or the comparison | Conditional recommendation for the intervention | Strong recommendation for the intervention |
| --- | --- | --- | --- | --- |
| ○ | ● | ○ | ○ | ○ |

**CONCLUSION**

| **Recommendation** |
| --- |
| We suggest against using surfactants for pediatric patients with ARDS.  (weak recommendation / very low certainty of evidence：GRADE 2D）  Supplementary item:  None |
|  |
| **Justification** |
| **Question:** Should surfactant be used in pediatric patients with ARDS?  **Population:** Pediatric patients (as defined in the article, 20 years old or younger if not specified) on ventilators for ARDS (as defined in the article)  **Intervention:** Surfactant  **Outcomes:** Mortality, Ventilator-free days (VFD), Length of hospital stay, Drug-related adverse events  **Summary of evidence:**  The meta-analysis for the included three randomized controlled trials (RCTs)^1-9^ showed that as beneficial outcomes, the estimate of effect for mortality (9 RCTs^1-9^, N=658) was 64 fewer per 1,000 (95% CI: 130 fewer to 45 more); for the 28-day VFD (2 RCTs4,6, N=261), it has a mean difference of 0.65 days shorter (95% CI: 5.26 shorter to 3.95 longer); for length of hospital stay (2 RCTs^1,4^, N=194), it has a mean difference of 1.67 days shorter (95% CI: 7.82 shorter to 4.49 longer) in the intervention group compared with that in the control group. Therefore, the expected desirable effect was judged to be "small."  As a harmful outcome, the estimate of the effect of drug-related adverse effects (7 RCTs^1-6,9^, N=589) was 180 more per 1,000 (95% CI: 81 more to 360 more) in the intervention group than that in the control group. Since most of the drug-related adverse effects were transient hypoxemia and hemodynamic changes, which were not serious, the undesirable effect was judged to be "small.”  **Quality of evidence:**  Since the directions of the desirable and undesirable effects did not match, the lowest certainty of evidence for the overall outcome, "very low," was adopted.  **Balance of effects, acceptability, Feasibility:**  Based on the findings of the meta-analysis, we determined that the balance between desirable and undesirable effects was "Does not favor either the intervention or control". Though, given that (a) the cost of the drug is substantial, (b) drug-related side effects requiring the administration of resuscitation drugs and adjustment of ventilation could happen, and (c) the feasibility of the therapy is limited considering the availability of the environment desirable to perform this therapy, we concluded “the condition recommendation against using surfactant”.  **Panel meeting:**  In the pre-vote, a modified Delphi method was used, with the recommendation "We suggest against using surfactants for pediatric patients with ARDS.  (weak recommendation / very low certainty of evidence：GRADE 2D）” and supplementary items "Alveolar surfactant has a small prognostic benefit, while drug-related adverse effects are increased. In addition, it is an expensive drug for uninsured use. Overall, “we suggest that surfactants should not be used for pediatric ARDS" was agreed upon, with a median score of 9 and a disagreement index of 0.2920.  At the panel meeting, it was expressed that the supplementary items in the recommendation column were unnecessary. Therefore, the supplementary conditions were removed from the recommendation column and re-voted to be included only in the reason column. Using the modified Delphi method, agreement was reached with a median score of 9 and a disagreement index of 0.0000.  **Additional considerations:**  While the net effect by the administration of pulmonary surfactant was limited, there was an increase in the drug-related side effects. In addition, the cost of surfactant is substantial. |

| **Subgroup considerations** |
| --- |
| None |
| **Implementation considerations** |
| For children with ARDS severe enough to be considered for this drug, the patient should be transferred to a facility (e.g., pediatric ICU) with experience in rescue therapy for ARDS (e.g., special ventilation or extracorporeal membrane ventilation). If a surfactant is to be administered, it should be discussed with the ethics committee of the institution, including how to explain the procedure to the family, since insurance does not cover ARDS. |

| **Monitoring and evaluation** |
| --- |
| When surfactant administration is implemented, the efficacy, toxicity, and cost of the treatment must be carefully monitored. |
| **Research priorities** |
| Many of the studies included in the meta-analysis were funded or supported by pharmaceutical companies, leading to the weakened reliability of studies due to the high risk of bias. Despite trial and error in studies over the past two decades, no reliable therapeutic benefits have been demonstrated. It is expected that studies that show new evidence will continue to be difficult. |

Reference

1. Willson DF, Zaritsky A, Bauman LA, et al. Instillation of calf lung surfactant extract (calfactant) is beneficial in pediatric acute hypoxemic respiratory failure. Members of the Mid-Atlantic Pediatric Critical Care Network. Critical care medicine 1999;27:188-95.

2. Luchetti M, Ferrero F, Gallini C, et al. Multicenter, randomized, controlled study of porcine surfactant in severe respiratory syncytial virus-induced respiratory failure. Pediatric critical care medicine : a journal of the Society of Critical Care Medicine and the World Federation of Pediatric Intensive and Critical Care Societies 2002;3:261-8.

3. Möller JC, Schaible T, Roll C, et al. Treatment with bovine surfactant in severe acute respiratory distress syndrome in children: a randomized multicenter study. Intensive care medicine 2003;29:437-46.

4. Willson DF, Thomas NJ, Markovitz BP, et al. Effect of exogenous surfactant (calfactant) in pediatric acute lung injury: a randomized controlled trial. Jama 2005;293:470-6.

5. Thomas NJ, Guardia CG, Moya FR, et al. A pilot, randomized, controlled clinical trial of lucinactant, a peptide-containing synthetic surfactant, in infants with acute hypoxemic respiratory failure. Pediatric critical care medicine : a journal of the Society of Critical Care Medicine and the World Federation of Pediatric Intensive and Critical Care Societies 2012;13:646-53.

6. Willson DF, Thomas NJ, Tamburro R, et al. Pediatric calfactant in acute respiratory distress syndrome trial. Pediatric critical care medicine : a journal of the Society of Critical Care Medicine and the World Federation of Pediatric Intensive and Critical Care Societies 2013;14:657-65.

7. Rodríguez-Moya VS, Gallo-Borrero CM, Santos-Áreas D, Prince-Martínez IA, Díaz-Casañas E, López-Herce Cid J. Exogenous surfactant and alveolar recruitment in the treatment of the acute respiratory distress syndrome. The clinical respiratory journal 2017;11:1032-9.

8. Rodríguez-Moya VS M-LM, Barrese-Pérez Y, et al. Cuban exogenous pulmonary surfactant in treatment of pediatric acute respiratory distress syndrome. MEDICC Review 2017;19:24-31.

9. Thomas NJ, Spear D, Wasserman E, et al. CALIPSO: A Randomized Controlled Trial of Calfactant for Acute Lung Injury in Pediatric Stem Cell and Oncology Patients. Biology of blood and marrow transplantation : journal of the American Society for Blood and Marrow Transplantation 2018;24:2479-86.

**PCQ13 Should corticosteroids be used in pediatric patients with ARDS?**

1.Search strategy

MEDLINE via Pubmed (Search date: 2020/7/3)

| #1 | "respiratory distress syndrome, adult"[MeSH Terms] OR "ARDS"[Title/Abstract] OR "respiratory distress syndrome"[Title/Abstract] OR "acute lung injury"[MeSH Terms] OR "acute lung injur*"[Title/Abstract] OR "ALI"[Title/Abstract] |
| --- | --- |
| #2 | "respiratory distress syndrome, newborn"[MeSH Terms] |
| #3 | #1 OR #2 |
| #4 | "steroids"[MeSH Terms] OR "adrenal cortex hormones"[MeSH Terms] |
| #5 | "methylprednisolone"[Title/Abstract] OR "hydrocortisone"[Title/Abstract] OR "glucocorticoid*"[Title/Abstract] OR "dexamethasone"[Title/Abstract] OR "corticosteroid*"[Title/Abstract] OR "cortiso*"[Title/Abstract] |
| #6 | #4 OR #5 |
| #7 | #3 AND #6 |
| #8 | ((((((("randomized controlled trial"[Publication Type] OR "controlled clinical trial"[Publication Type]) OR "randomized"[Title/Abstract]) OR "placebo"[Title/Abstract]) OR "drug therapy"[MeSH Subheading]) OR "randomly"[Title/Abstract]) OR "trial"[Title/Abstract]) OR "groups"[Title/Abstract]) NOT ("animals"[MeSH Terms] NOT "humans"[MeSH Terms]) |
| #9 | #7 AND #8 |

CENTRAL (Search date: 2020/7/3)

| #1 | [mh "Respiratory Distress Syndrome, Adult"] OR ARDS:ti,ab OR "respiratory distress syndrome":ti,ab OR [mh "Acute Lung Injury"] OR "acute lung injury":ti,ab OR ALI:ti,ab |
| --- | --- |
| #2 | [mh "Respiratory Distress Syndrome, Newborn"] |
| #3 | #1 OR #2 |
| #4 | [mh Steroids] OR [mh "adrenal cortex hormones"] |
| #5 | methyl prednisolone:ti,ab OR hydrocortisone:ti,ab OR glucocorticoid*:ti,ab OR dexamethasone:ti,ab OR corticosteroid*:ti,ab OR cortiso*:ti,ab |
| #6 | #4 OR #5 |
| #7 | #3 AND #6 |

Igaku Chuo Zasshi (Search date: 2020/6/26)

| #1 | 呼吸窮迫症候群-急性/TH or 急性呼吸窮迫症候群/TA or ARDS/TA |
| --- | --- |
| #2 | 急性肺損傷/TH or 急性肺損傷/TA |
| #3 | 呼吸窮迫症候群-新生児/TH or 新生児呼吸窮迫症候群/TA |
| #4 | #1 or #2 or #3 |
| #5 | Steroids/TH or "Fluorinated Steroids"/TA |
| #6 | 副腎皮質ホルモン/TH or 副腎皮質ホルモン/TA |
| #7 | "Methylprednisolone Succinate"/TH or "Methylprednisolone Succinate"/TA |
| #8 | Hydroxycorticosteroids/TH or Hydroxycorticosteroids/TA |
| #9 | Methylprednisolone/TH or Methylprednisolone/TA or メチルプレドニゾロン/TA |
| #10 | Hydrocortisone/TH or Hydrocortisone/TA or ヒドロコルチゾン/TA or ハイドロコルチゾン/TA |
| #11 | Dexamethasone/TA or デキサメタゾン/TA |
| #12 | Betamethasone/TA or ベタメタゾン/TA |
| #13 | Cortisone/TH or Cortisone/TA or コルチゾン/TA |
| #14 | Glucocorticoids/TH or Glucocorticoids/TA or グルココルチコイド/TA |
| #15 | #5 or #6 or #7 or #8 or #9 or #10 or #11 or #12 or #13 or #14 |
| #16 | #4 and #15 |
| #17 | (ランダム化比較試験/TH or 準ランダム化比較試験/TH or ランダム化/AL or 無作為化/AL or 比較試験/AL or 臨床試験/AL or プラセボ/AL or 対照/AL or コントロール/AL or 臨床研究/AL) and (PT=会議録除く) |
| #18 | #16 and #17 |

1. Flow diagram

**Identification**

1 Studies included in qualitative synthesis

26 Full-text articles assessed for eligibility

2,161 records after duplicates removed

2,443 records identified through database searching

2,443 records identified through database searching

Medline via PubMed (n=1,827)

CENTRAL (n=519)

Igaku-Chuo-Zasshi (n=97)

0 additional records identified through other sources

1 Study included in quantitative synthesis (meta-analysis)

25 Full-text articles excluded, with reasons:

・Wrong study design (n=4)

・Wrong publication type (n=4)

・Wrong population (n=13)

・Wrong outcome (n=1)

・Duplicates (n=3)

Duplicates

n=282

2,135 records excluded

**Included**

**Eligibility**

**Screening**

1. Risk of bias

Mortality Ventilator-free days (VFD)


Length of hospital stay Nosocomial inection

Hyperglycemia

1. Forest plot

Mortality

Ventilator-free days (VFD)

Length of hospital stay

Nosocomial infection

Hyperglycemia

1. Evidence profile

| Assessment of certainty | | | | | | | | | | No. of patients | | | | Efficacy | | | | | Certainty of the Evidence | | Importance |
| --- | --- | --- | --- | --- | --- | --- | --- | --- | --- | --- | --- | --- | --- | --- | --- | --- | --- | --- | --- | --- | --- |
| No. of studies | | Study design | | Risk of bias | | Inconsistency | Indirectness | Imprecision | Others | Corticosteroid | | Placebo | | Relative index (95% CI) | | | Absolute index (95% CI) | |  |  |  |
| **Mortality ^a^** | | | | | | | | | | | | | | | | | | | | | |
|  | 1 | | RCT | | Very serious ^b^ | Not serious | Not serious | Very　serious ^c^ | None | | 0/17 (0.0%) | | 2/18 (11.1%) | | **RR 0.21** (0.01–4.10) | **-88 per 1000 patients** (-110 to +344) | | ⨁◯◯◯ very low | | Critical | |
| **Developmental prognosis** | | | | | | | | | | | | | | | | | | | | | |
| 0 | | - | | - | | - | - | - | - | - | | - | | - | | | - | | - | | Critical |
| **Ventilator-free days (VFD)** | | | | | | | | | | | | | | | | | | | | | |
| 1 | | RCT | | Very serious ^b^ | | Not serious | Not serious | Very　serious ^d^ | None | 17 | | 18 | | - | | | **MD 1.32 days longer** (-3.32 to +5.96) | | ⨁◯◯◯ very low | | Critical |
| **Length of hospital stay** | | | | | | | | | | | | | | | | | | | | | |
| 1 | | RCT | | Very serious ^b^ | | Not serious | Not serious | Very　serious ^d^ | None | 17 | | 18 | | - | | | **MD 6.87 days shorter**  (-15.32 shorter to +1.58) | | ⨁◯◯◯ very low | | Critical |
| **Nosocomial infection** | | | | | | | | | | | | | | | | | | | | | |
| 1 | | RCT | | Very serious ^b^ | | Not serious | Not serious | Very serious ^c^ | None | 1/17 (5.9%) | | 5/18 (27.8%) | | **RR 0.21** (0.03 to 1.63) | | | **-219 per 1000 patients** (-296 to +175 ) | | ⨁◯◯◯ very low | | Critical |
| **Hyperglycemia** | | | | | | | | | | | | | | | | | | | | | |
| 1 | | RCT | | Very serious ^b^ | | Not serious | Not serious | Very serious ^c^ | None | 10/17 (58.8%) | | 8/18 (44.4%) | | **RR 1.32** (0.69 to 2.54) | | | **+142 per 1000 patients** (-138 to +684) | | ⨁◯◯◯ very low | | Important |
| **Myopathy /Neuropathy** | | | | | | | | | | | | | | | | | | | | | |
| 0 | | - | | - | | - | - | - | - | - | | - | | - | | | - | | - | | Critical |

CI: confidence interval; RR: risk ratio; MD: mean difference; RCT: randomized controlled trial

1. The follow-up period for mortality is uncertain.
2. One eligible study was deemed “serious” and downgraded one level because the risk of bias was determined to be uncertain.
3. The total sample size is 35, which does not meet the optimal information scale (OIS). Additionally, since the 95% CI straddles the treatment thresholds for benefit and harm, we judged it to be ‘very serious’ and downgraded it by two levels.
4. The total sample size is 35, and the 95% CI was wide enough to override the clinical decision; therefore, it is downgraded by two levels.

Note: Developmental prognosis and myopathy were set as outcomes for CQ; however, no studies reported these.

1. Evidence-to-Decision table

| **QUESTION** | |
| --- | --- |
| **PCQ13：** Should corticosteroids be used in pediatric patients with ARDS? | |
| **POPULATION:** | Pediatric patients (as defined in the article, 20 years old or younger if not specified) on ventilators for ARDS (as defined in the article) |
| **INTERVENTION:** | Systemic administration of corticosteroids (including oral and intravenous administration, regardless of the drug administered, the dose administered, or the duration of administration). |
| **COMPARISON:** | No corticosteroids administered |
| **MAIN OUTCOMES:** | Mortality, Developmental prognosis, Ventilator-free days (VFD), Length of hospital stay, Nosocomial infection, Myopathy / Neuropathy |
| **SETTINGS:** | Emergency department or intensive care unit (ICU) |
| **PERSPECTIVE:** | Individual |
| **BACKGROUND:** | Acute respiratory distress syndrome (ARDS) is a disease caused by inflammation that spreads to lungs from a variety of sources. Corticosteroids are deemed effective because they inactivate the inflammatory mediators involved in ARDS. In contrast, corticosteroids can suppress immune function and cause infections. Whether corticosteroids improve mortality and other clinical outcomes remains controversial, as previous systematic reviews have reported conflicting results. Therefore, this clinical question is important. |
| **CONFLICT OF INTERESTS:** | None |

**ASSESSMENT**

| **Problem**  Is the problem a priority? | | |
| --- | --- | --- |
| **Judgement** | **Research evidence** | **Additional considerations** |
| ○ No  ○ Probably no  ○ Probably yes  ● Yes  ○ Varies  ○ Do not know | ARDS is a disease caused by diffuse lung inflammation from a variety of sources. Corticosteroids are believed to be effective because they inactivate the inflammatory mediators involved in ARDS. In contrast, corticosteroids can suppress immune function and cause infections. Whether corticosteroids improve mortality and other clinical outcomes remains controversial, as previous systematic reviews have reported conflicting results. Therefore, this clinical question is an important issue and a high priority. |  |
| **Desirable Effects**  How substantial are the desirable anticipated effects? | | |
| **Judgement** | **Research evidence** | **Additional considerations** |
| ● Trivial  ○ Small  ○ Moderate  ○ Large  ○ Varies  ○ Do not know | One RCT^1^ consistent with PICO was included in the systematic review and used in the meta-analysis. In this study, the Pediatric Logistic Organ Dysfunction score (PELOD score) was almost twice as high in the control group, as in the intervention group.  As beneficial outcomes, the estimate of effect for mortality (one RCT^1^, N=35) was 88 fewer per 1,000 (95% CI: 110 fewer to 344 more), a mean difference of 1.32 days longer (95% CI: 3.32 shorter to 5.96 longer) for VFD (one RCT^1^, N=35), and a mean difference of 6.87 days shorter (95% CI: 15.32 shorter to 1.58 longer) for length of hospital stay (one RCT^1^, N=35) in the intervention group compared with that in the control group. However, the sample size was very small, and the CI was wide. Since the number of deaths, a critically important outcome, could increase up to 344 in some cases, we believe that the desirable effect was “trivial." |  |
| **Undesirable Effects**  How substantial are the undesirable anticipated effects? | | |
| **Judgement** | **Research evidence** | **Additional considerations** |
| ○ Large  ○ Moderate  ○ Small  ○ Trivial  ● Varies  ○ Do not know | As a harmful outcome, the estimate of the effect of nosocomial infection (one RCT^1^, N=35) was 219 fewer per 1,000 (95% CI: 296 fewer to 175 more) in the intervention group than that in the control group. On the other hand, the estimated effect of hyperglycemia (one RCT^1^, N=35) was 142 more (95% CI: 138 fewer to 684 more). Developmental prognosis and myopathy/neuropathy, which are critically important outcomes, were not assessed as undesirable effects. Therefore, undesirable effects were categorized as "varies." |  |
| **Certainty of evidence**  What is the overall certainty of the evidence of effects? | | |
| **Judgement** | **Research evidence** | **Additional considerations** |
| ● Very low  ○ Low  ○ Moderate  ○ High  ○ No included studies | **The relative importance or values of the main outcomes of interest:**   \| **Outcome** \| **Importance** \| **Certainty of the Evidence**  **(GRADE)** \| \| --- \| --- \| --- \| \| Mortality \| Critical \| ⨁◯◯◯ Very low \| \| Developmental prognosis* \| Critical \| - \| \| VFD \| Critical \| ⨁◯◯◯ Very low \| \| Length of hospital stay \| Critical \| ⨁◯◯◯ Very low \| \| Nosocomial infection \| Critical \| ⨁◯◯◯ Very low \| \| Myopathy/Neuropathy* \| Critical \| - \|   *There were no outcome reports on developmental prognosis or myopathy/neuropathy in the adopted literature.  **Overall certainty of evidence**  There was only one RCT^1^ with a very small sample size of 35, and the patient backgrounds were different between the corticosteroid and placebo groups (plateau pressure was significantly higher in the corticosteroid group, and the severity score (PELOD score) was about twice as high in the placebo group). Although there were no significant differences, some desirable effects favored corticosteroid administration, which may be due to differences in the patient background. Given that corticosteroids may have only trivial positive effects and that undesirable effects may be inadequately assessed, it would be difficult to make a recommendation as to whether corticosteroids should be administered. The Pediatric Acute Lung Injury Consensus Statement^2^ does not recommend the routine use of corticosteroids in pediatric patients with ARDS. |  |
| **Values**  Is there important uncertainty about or variability in how much people value the main outcomes? | | |
| **Judgement** | **Research evidence** | **Additional considerations** |
| ○ Important uncertainty or variability  ○ Possibly important uncertainty or variability  ○ Probably no important uncertainty or variability  ● No important uncertainty or variability | “Mortality” is generally a critical outcome, and there is no great uncertainty or diversity of values regarding this. |  |
| **Balance of effects**  Does the balance between desirable and undesirable effects favor the intervention or the comparison? | | |
| **Judgement** | **Research evidence** | **Additional considerations** |
| ○ Favors the comparison  ○ Probably favors the comparison  ○ Does not favor either the intervention or the comparison  ○ Probably favors the intervention  ○ Favors the intervention  ● Varies  ○ Do not know | **Summary of evidence:**   \| **Outcome** \| **Comparison** \| **Intervention** \| **Absolute difference**  **(95% CI)** \| **Risk ratio**  **(RR)**  **(95% CI)** \| \| --- \| --- \| --- \| --- \| --- \| \| Mortality \| 2/18  (11.1%) \| 0/17  (0.0%) \| 88 fewer per 1,000  (110 fewer to 344 more) \| RR 0.21 (0.01 to 4.10) \| \| Developmental prognosis \| - \| - \| - \| - \| \| VFD \| - \| - \| MD 1.32 day longer (3.32 shorter to 5.96 longer) \| - \| \| Length of hospital stay \| - \| - \| MD 6.87 day shorter (15.32 shorter to 1.58 longer) \| - \| \| Nosocomial infection \| 5/18  (27.8%) \| 1/17  (5.9%) \| 219 fewer per 1,000  (296 fewer to 175 more) \| RR 0.21 (0.03 to 1.63) \| \| Myopathy/Neuropathy \| - \| - \| - \| **-** \|     Because the desirable effect of the intervention was trivial and the undesirable effect was not fully assessed, the balance of effects was "varies.” |  |
| **Acceptability**  Is the intervention acceptable to key stakeholders? | | |
| **Judgement** | **Research evidence** | **Additional considerations** |
| ○ No  ○ Probably no  ○ Probably yes  ○ Yes  ● Varies  ○ Do not know | The perception of harm from the intervention may vary from stakeholder to stakeholder. |  |
| **Feasibility**  Is the intervention feasible to implement? | | |
| **Judgement** | **Research evidence** | **Additional considerations** |
| ○ No  ○ Probably no  ○ Probably yes  ● Yes  ○ Varies  ○ Do not know | The intervention seems feasible because it is already being implemented in daily practice. |  |

**Summary of judgement**

|  | **JUDGEMENT** | | | | | | |
| --- | --- | --- | --- | --- | --- | --- | --- |
| **PROBLEM** | No | Probably no | Probably yes | **Yes** |  | Varies | Do not know |
| **DESIRABLE EFFECTS** | **Trivial** | Small | Moderate | Large |  | Varies | Do not know |
| **UNDESIRABLE EFFECTS** | Large | Moderate | Small | Trivial |  | **Varies** | Do not know |
| **CERTAINTY OF EVIDENCE** | **Very low** | Low | Moderate | High |  |  | No included studies |
| **VALUES** | Important uncertainty or variability | **Possibly important uncertainty or**  **variability** | Probably no important uncertainty or variability | No important uncertainty or variability |  |  |  |
| **BALANCE OF EFFECTS** | Favors the comparison | Probably favors the comparison | Does not favor either the intervention or the comparison | Probably favors the intervention | Favors the intervention | **Varies** | Do not know |
| **ACCEPTABILITY** | No | Probably no | Probably yes | Yes |  | **Varies** | Do not know |
| **FEASIBILITY** | No | Probably no | Probably yes | **Yes** |  | Varies | Do not know |

**Type of Recommendation**

| Strong recommendation against the intervention | Conditional recommendation against the intervention | Conditional recommendation for either the intervention or the comparison | Conditional recommendation for the intervention | Strong recommendation for the intervention |
| --- | --- | --- | --- | --- |
| ○ | ○ | ○ | ○ | ○ |

**CONCLUSION**

| **Recommendation** |
| --- |
| We cannot provide a recommendation on the administration of corticosteroids for pediatric patients with ARDS. Corticosteroid administration is examined in consideration of patient background and pathophysiology (in our practice statement).  Supplementary item:  This is a description of the current clinical practice, and this does not reject the use of corticosteroids. |
|  |
| **Justification** |
| **Question：** Should corticosteroids be used in pediatric patients with ARDS?  **Population：** Pediatric patients (as defined in the article, 20 years old or younger if not specified) on ventilators for ARDS (as defined in the article)  **Intervention:** Systemic administration of corticosteroids (including oral and intravenous administration, regardless of the drug administered, the dose administered, or the duration of administration).  **Comparison**： No corticosteroids administered  **Main Outcomes：** Mortality, Developmental prognosis, Ventilator-free days (VFD), Length of hospital stay, Nosocomial infection, Myopathy / Neuropathy  One RCT^1^ consistent with PICO was included in the systematic review and used in the meta-analysis. In this study, the Pediatric Logistic Organ Dysfunction score (PELOD score) was almost twice as high in the control group, as in the intervention group.  As beneficial outcomes, the estimate of effect for mortality (one RCT^1^, N=35) was 88 fewer per 1,000 (95% CI: 110 fewer to 344 more), a mean difference of 1.32 days longer (95% CI: 3.32 shorter to 5.96 longer) for VFD (one RCT^1^, N=35), and a mean difference of 6.87 days shorter (95% CI: 15.32 shorter to 1.58 longer) for length of hospital stay (one RCT^1^, N=35) in the intervention group compared with that in the control group. However, the sample size was very small, and the CI was wide. Since the number of deaths, a critically important outcome, could increase up to 344 in some cases, we believe that the desirable effect was “trivial."  As a harmful outcome, the estimate of the effect of nosocomial infection (one RCT^1^, N=35) was 219 fewer per 1,000 (95% CI: 296 fewer to 175 more) in the intervention group than that in the control group. On the other hand, the estimated effect of hyperglycemia (one RCT^1^, N=35) was 142 more (95% CI: 138 fewer to 684 more). Developmental prognosis and myopathy/neuropathy, which are critically important outcomes, were not assessed as undesirable effects. Therefore, undesirable effects were categorized as "varies."  **Quality of evidence**：  There was only one RCT^1^ with a very small sample size of 35, and the patient backgrounds were different between the corticosteroid and placebo groups (plateau pressure was significantly higher in the corticosteroid group, and the severity score (PELOD score) was about twice as high in the placebo group). Although there were no significant differences, some desirable effects favored corticosteroid administration, which may be due to differences in the patient background. Given that corticosteroids may have only trivial positive effects and that undesirable effects may be inadequately assessed, it would be difficult to make a recommendation as to whether corticosteroids should be administered. The Pediatric Acute Lung Injury Consensus Statement^2^ does not recommend the routine use of corticosteroids in pediatric patients with ARDS.  **Balance of effects, Acceptability, Feasibility**:  It is impossible to assess the balance of effects because the desirable effect of the intervention was trivial and the undesirable effect was not fully assessed. While we believe that this is already being implemented in daily clinical practice and there is no problem with feasibility, we thought that acceptance of the undesirable effects of corticosteroid administration would vary among stakeholders.  **Panel meeting:** In a pre-vote, the modified Delphi method resulted in the following recommendation: “We cannot provide a recommendation on the administration of corticosteroids for pediatric patients with ARDS. Corticosteroid administration is examined in consideration of patient background and pathophysiology (in our practice statement)." was agreed upon in our practice statement with a median score of 8.0 and a disagreement index of 0.2920.  In the panel meeting, a discussion on whether to adopt the extracted RCT and include it in our practice statement or not transpired. The final decision was to adopt it and include it in our practice statement. There was no change in the recommendation statement, but a re-vote was conducted because of the possibility of a change in the result from the prior vote based on the discussion. Using the modified Delphi method, a median score of 9 and a disagreement index of 0.1316 were agreed upon, as in our practice statement.  **Additional considerations：**  This is only a description of current practice and does not negate the use of corticosteroids, including the results of this RCT. |

| **Subgroup considerations** |
| --- |
| Although subgroup analysis has not been performed, patients with underlying diseases such as hypertension/diabetes/gastrointestinal bleeding/immunodeficiency/predisposition to thrombosis need to be more careful about the side effects of corticosteroids. |
| **Implementation considerations** |
| Corticosteroids have various side effects, such as infections due to decreased immunity, glucose intolerance, hypertension, gastrointestinal bleeding, and thrombus formation. Therefore, monitoring of blood glucose and blood pressure, gastric juice, physical findings of deep venous thrombosis, echocardiographic evaluation, and nutritional management and rehabilitation should be encouraged.  The dosage of corticosteroids described in the literature was 2 mg/kg of methylprednisolone intravenously followed by 1 mg/kg/day of methylprednisolone intravenously on a continuous basis (administered for 7 days and then tapered off over a week). |

| **Monitoring and evaluation** |
| --- |
| In order to implement the recommendation, it may be necessary to examine in more detail whether corticosteroids should be used in individual cases. There are still many unresolved issues, such as indications for administration, timing of administration, and duration of administration. Therefore, after the publication of this guideline, it will be necessary to closely monitor the situation when corticosteroids are unavoidably administered based on clinical judgment. |
| **Research priorities** |
| The appropriate type, dosage, and duration of corticosteroid administration in pediatric ARDS are still unclear; clinical studies that consider the causes of ARDS and the age of the patient group are warranted. |

Reference

1. Drago BB, Kimura D, Rovnaghi CR, et al. Double-blind, placebo-controlled pilot randomized trial of methylprednisolone infusion in pediatric acute respiratory distress syndrome. Pediatric critical care medicine : a journal of the Society of Critical Care Medicine and the World Federation of Pediatric Intensive and Critical Care Societies 2015;16:e74-81.

2. Tamburro RF, Kneyber MC. Pulmonary specific ancillary treatment for pediatric acute respiratory distress syndrome: proceedings from the Pediatric Acute Lung Injury Consensus Conference. Pediatric critical care medicine : a journal of the Society of Critical Care Medicine and the World Federation of Pediatric Intensive and Critical Care Societies 2015;16:S61-72.

**PCQ14 Should a protocol be used for the sedation of pediatric respiratory failure patients?**

1.Search strategy

MEDLINE via Pubmed (Search date: 2020/6/18）

| #1 | Respiratory Distress Syndrome, Adult[mh] OR ARDS[tiab] OR respiratory distress syndrome[tiab] OR Acute Lung Injury[mh] OR acute lung injury[tiab] OR ALI[tiab] |
| --- | --- |
| #2 | Critical Illness[mh] OR Critical Care[mh] OR Intensive Care Units[mh] OR critically ill[tiab] OR critical care[tiab] OR intensive care[tiab] |
| #3 | #1 OR #2 |
| #4 | Respiration, Artificial[mh] OR ventilators, mechanical[mh] OR ventilator[tiab] OR Ventilators[tiab] OR ventilation[tiab] OR ventilations[tiab] |
| #5 | sedation*[tiab] OR deep sedation[mh] OR Conscious Sedation[mh] OR Hypnotics and Sedatives[mh] |
| #6 | #3 AND #4 AND #5 |
| #7 | (randomized controlled trial[pt] OR controlled clinical trial[pt] OR randomized[tiab] OR placebo[tiab] OR clinical trials as topic[mesh:noexp] OR randomly[tiab] OR trial[ti]) NOT (animals[mh] NOT humans[mh]) |
| #8 | #6 AND #7 |

CENTRAL (Search date: 2020/6/18）

| #1 | [mh "Respiratory Distress Syndrome, Adult"] OR ARDS:ti,ab OR "respiratory distress syndrome":ti,ab OR [mh "Acute Lung Injury"] OR "acute lung injury":ti,ab |
| --- | --- |
| #2 | [mh "Critical Illness"] OR [mh "Critical Care"] OR [mh "Intensive Care Units"] OR "critically ill":ti,ab OR "critical care":ti,ab OR "intensive care":ti,ab |
| #3 | #1 OR #2 |
| #4 | [mh "Respiration, Artificial"] OR [mh "ventilators, mechanical"] OR ventilator:ti,ab OR Ventilators:ti,ab OR ventilation:ti,ab OR ventilations:ti,ab |
| #5 | sedation*:ti,ab OR [mh "deep sedation"] OR [mh "Conscious Sedation"] OR [mh "Hypnotics and Sedatives"] |
| #6 | #3 AND #4 AND #5 |

Igaku Chuo Zasshi (Search date: 2020/6/18）

| #1 | 呼吸窮迫症候群-急性/TH or 急性呼吸窮迫症候群/TA or ARDS/TA |
| --- | --- |
| #2 | クリティカルケア/TH or クリティカルケア/TA |
| #3 | ICU/TH or ICU/TA |
| #4 | 危篤/TH or 危篤/TA |
| #5 | #1 or #2 or #3 or #4 |
| #6 | 人工呼吸/TH or 人工呼吸器/TH or 人工呼吸/TA |
| #7 | 鎮静度/TH or 催眠剤と鎮静剤/TH or 鎮静/TA |
| #8 | #5 and #6 and #7 |
| #9 | (ランダム化比較試験/TH or 準ランダム化比較試験/TH or ランダム化/AL or 無作為化/AL or 比較試験/AL or 臨床試験/AL or プラセボ/AL or 対照/AL or コントロール/AL or 臨床研究/AL) and (PT=会議録除く) |
| #10 | #8 and #9 |

1. Flow diagram

1,138 records after duplicates removed

1 Studies included in qualitative synthesis

1,114 records excluded

1,597 records identified through database searching

Medline via PubMed (n=628)

CENTRAL (n=926)

Igaku-Chuo-Zasshi (n=43)

23 Full-text articles excluded, with reasons:

・Wrong study design (n=7)

・Wrong publication type (n=5)

・Wrong population (n=8)

・Wrong intervention (n=3)

0 additional records identified through other sources

1,597 records identified through database searching

Duplicates

n=459

24 Full-text articles assessed for eligibility

**Identification**

**Screening**

**Eligibility**

1 Studies included in quantitative synthesis (meta-analysis)

**Included**

1. Risk of bias

Mortality Duration of mechanical ventilation


Length of hospital stay Length of ICU stay

Withdrawal syndrome

1. Forest plot

Mortality

Duration of mechanical ventilation

Length of hospital stay

Length of ICU stay

Withdrawal syndrome

1. Evidence profile

| **Assessment of certainty** | | | | | | | **No. of patients** | | **Efficacy** | | **Certainty of the evidence** | **Importance** |
| --- | --- | --- | --- | --- | --- | --- | --- | --- | --- | --- | --- | --- |
| **No. of studies** | **Study design** | **Risk of bias** | **Inconsistency** | **Indirectness** | **Imprecision** | **Others** | **Sedation protocol** | **Control** | **Relative index (95% CI)** | **Absolute index (95% CI)** |  |  |
| **Mortality^a^** | | | | | | | | | | | | |
| 1 | RCT | Serious ^b^ | Not serious | Not serious | Serious ^c^ | None | 47/1225 (3.8%) | 63/1224 (5.1%) | **RR 0.75** (0.52 to 1.08) | **-13 per 1000 patients**  (-25 to +4) | ⨁⨁◯◯ Low | Critical |
| **Developmental prognosis** | | | | | | | | | | | | |
| 0 |  |  |  |  |  |  |  |  | - | - | - | Critical |
|  | | | | | | | | | | | | |
| **Duration of mechanical ventilation** | | | | | | | | | | | | |
| 1 | RCT | Serious ^b^ | Not serious | Not serious | Not serious | None | 1225 | 1224 | - | **MD ±0 day** (-0.46 to +0.46) | ⨁⨁⨁◯ Moderate | Critical |
| **Length of hospital stay** | | | | | | | | | | | | |
| 1 | RCT | Serious ^b^ | Not serious | Not serious | Not serious | None | 1225 | 1224 | - | **MD 2 days shorter** (-3.04 to +0.96) | ⨁⨁⨁◯ Moderate | Critical |
| **Length of ICU stay** | | | | | | | | | | | | |
| 1 | RCT | Serious ^b^ | Not serious | Not serious | Not serious | None | 1225 | 1224 | - | **MD ±0day**  (-0.6 to +0.6) | ⨁⨁⨁◯ Moderate | Important |
| **Withdrawal syndrome** | | | | | | | | | | | | |
| 1 | RCT | Serious ^b^ | Not serious | Not serious | Not serious | None | 149/1225 (12.2%) | 114/1224 (9.3%) | **RR 1.31** (1.04 to 1.64) | **+29 per 1,000 patients**  (+4 to +60) | ⨁⨁⨁◯ Moderate | Important |

**Adverse events**

| 0 |  |  |  |  |  |  |  |  | - | - | - | Critical |
| --- | --- | --- | --- | --- | --- | --- | --- | --- | --- | --- | --- | --- |

**CI:** Confidence interval; **RR:** Risk ratio; **RCT**: randomized controlled trial; **ICU**: intensive care unit

**Explanations**

a. Mortality rate at 28 days

b. Downgraded by one level due to one target literature and a high risk of bias

c. The total sample size was 2449, the optimal information size (OIS) was not met, and the 95% confidence interval included the treatment area outside, which was downgraded by one level.

No articles reported developmental outcomes and adverse events.

1. Evidence-to-Decision table

| **QUESTION** | |
| --- | --- |
| **PCQ14:** Should a protocol be used for the sedation of pediatric respiratory failure patients? | |
| **POPULATION:** | Pediatric patients (as defined in the article, 20 years old or younger if not specified) on ventilators for ARDS who meet one of the following criteria: AECC criteria, Berlin criteria, or PALICC criteria  or  Pediatric patients requiring ventilatory management for more than 12 hours in intensive care unit |
| **INTERVENTION:** | Protocolized sedation (with or without Spontaneous awakening trial or Daily sedation interruption) |
| **COMPARISON:** | Conventional sedation without any protocols |
| **MAIN OUTCOMES:** | Mortality, Developmental prognosis, Duration of mechanical ventilation, Length of hospital stay, Adverse events |
| **SETTINGS:** | Emergency department or intensive care unit (ICU) |
| **PERSPECTIVE:** | Individual |
| **BACKGROUND:** | No sedation/light sedation/protocolized sedation is expected to effectively avoid complications of deep sedation because the use of large doses of sedatives increases the risk of prolonged ventilation, delirium, and withdrawal symptoms, although adequate sedation is sometimes necessary for ventilatory management. In the ventilatory management of pediatric Acute respiratory distress syndrome (ARDS) patients, no sedation/light sedation/protocolized sedation is not a well-established treatment. Therefore, clarifying its efficacy is an important clinical issue.  We did not limit the target population to ARDS patients because the sedation protocol is an intervention based on ventilator weaning, and its usefulness is not expected to differ between ARDS and non-ARDS patients. |
| **CONFLICT OF INTERESTS:** | None |

**ASSESSMENT**

| **Problem**  Is the problem a priority? | | |
| --- | --- | --- |
| **Judgement** | **Research evidence** | **Additional considerations** |
| ○ No  ○ Probably no  ○ Probably yes  ● Yes  ○ Varies  ○ Do not know | Ventilatory management requires daily assessment of the patient's ability to be weaned from the ventilator. In pediatric ventilatory management, large doses of sedatives may be required if the patient does not cooperate with rest, which may interfere with the assessment of extubation feasibility. Since the use of large doses of sedatives increases the risk of prolonged ventilation, delirium, and withdrawal symptoms, the use of protocolized sedation is expected to effectively avoid complications. Protocolized sedation is not an established therapy in the ventilatory management of pediatric ARDS patients, and clarifying its efficacy is an important clinical issue and a high priority. |  |
| **Desirable Effects**  How substantial are the desirable anticipated effects? | | |
| **Judgement** | **Research evidence** | **Additional considerations** |
| ○ Trivial  ● Small  ○ Moderate  ○ Large  ○ Varies  ○ Do not know | One RCT^1^ consistent with PICO was included in the systematic review and used in the meta-analysis.  As a beneficial outcome, the estimate of effect for the 28-day mortality (1 RCT^1^, N=2449) was 13 fewer per 1,000 (95% CI: 25 fewer to 4 more) in the intervention group than that in the control group. The estimate of the effect for the duration of mechanical ventilation (1 RCT^1^, N=2449) was the mean difference of 0 day (95% CI: 0.46 shorter to 0.46 longer) and for duration of length of hospital stay (1 RCT^1^, N=2449) was the mean difference of 2 days shorter (95% CI: 3.04 shorter to 0.96 longer). Although developmental prognosis is considered a beneficial outcome, no studies have reported this. Therefore, considering the decrease in mortality, duration of mechanical ventilation, and length of hospital stays, we judged the desirable effect of the intervention to be "small.” |  |
| **Undesirable Effects**  How substantial are the undesirable anticipated effects? | | |
| **Judgement** | **Research evidence** | **Additional considerations** |
| ○ Large  ○ Moderate  ○ Small  ● Trivial  ○ Varies  ○ Do not know | Although adverse events such as unplanned extubation and worsening respiratory status were examined, no studies have reported the　absolute numbers of those outcomes. Considering this result together with the additional considerations, the undesirable effect of the intervention was judged to be "trivial.”  The literature reviewed in this systematic review (Curley 2015)^1^ found that the estimate of the effect of withdrawal syndrome (1 RCT^1^, N=2449), an important adverse event, was 29 more per 1,000 (95% CI: 4 more to 60 more) in the intervention group than that in the control group. | The literature reviewed in this systematic review (Curley 2015)^1^ found that the estimate of effect for the withdrawal syndrome (1RCT, N=2449), an important adverse event, was 29 more per 1,000 (95% CI: 4 more to 60 more) in the intervention group compared to the control group. |
| **Certainty of evidence**  What is the overall certainty of the evidence of effects? | | |
| **Judgement** | **Research evidence** | **Additional considerations** |
| ○ Very low  ○ Low  ● Moderate  ○ High  ○ No included studies | **The relative importance or values of the main outcomes of interest:**   \| **Outcome** \| **Importance** \| **Certainty of the Evidence**  **(GRADE)** \| \| --- \| --- \| --- \| \| Mortality \| Critical \| ⨁⨁◯◯ \| \| Low \| \| Duration of mechanical ventilation \| Critical \| ⨁⨁⨁◯ \| \| Moderate \| \| Length of hospital stay \| Critical \| ⨁⨁⨁◯ \| \| Moderate \| \| Developmental prognosis* \| Critical \| - \| \| Adverse events (unplanned extubation、worsening respiratory status, etc.)* \| Critical \| - \|   *There were no outcome reports on developmental prognosis and the absolute numbers of adverse events in the accepted literature.  **Overall certainty of evidence**  Since the direction of the critically important outcomes extracted in the systematic review was consistent, the highest certainty of evidence for the overall outcome, "moderate," was adopted. |  |
| **Values**  Is there important uncertainty about or variability in how much people value the main outcomes? | | |
| **Judgement** | **Research evidence** | **Additional considerations** |
| ○ Important uncertainty or variability  ○ Possibly important uncertainty or variability  ○ Probably no important uncertainty or variability  ● No important uncertainty or variability | Currently, there is no evidence of the relative importance that children and their parents attach to each outcome of this intervention. However, “Mortality” is generally an important outcome, and there is no great uncertainty or diversity of values about this. |  |
| **Balance of effects**  Does the balance between desirable and undesirable effects favor the intervention or the comparison? | | |
| **Judgement** | **Research evidence** | **Additional considerations** |
| ○ Favors the comparison  ○ Probably favors the comparison  ○ Does not favor either the intervention or the comparison  ● Probably favors the intervention  ○ Favors the intervention  ○ Varies  ○ Do not know | **Summary of evidence**   \| **Outcome** \| **Control** \| **Intervention** \| **Absolute difference**  **(95% CI)** \| **Risk ratio (RR)**  **(95% CI)** \| \| --- \| --- \| --- \| --- \| --- \| \| Mortality \| 63/1224  (5.1%) \| 47/1225  (3.8%) \| 13 fewer per 1,000  (25 fewer to 4 more) \| RR 0.75  (0.52-1.08) \| \| Duration of mechanical ventilation \| - \| - \| MD 0 day  (0.46 shorter to 0.46 longer) \| - \| \| Length of hospital stay \| - \| - \| MD 2 day shorter  (3.04 shorter to 0.96 longer) \| - \| \| Developmental prognosis \| - \| - \| - \| - \| \| Adverse events \| - \| - \| - \| - \|   Based on the above, we determined that the balance between desirable and undesirable effects was "probably favors the intervention.” |  |
| **Acceptability**  Is the intervention acceptable to key stakeholders? | | |
| **Judgement** | **Research evidence** | **Additional considerations** |
| ○ No  ○ Probably no  ● Probably yes  ○ Yes  ○ Varies  ○ Do not know | No serious adverse events occurred. Thus, there would probably be no major problems with patient and patient family acceptance. |  |
| **Feasibility**  Is the intervention feasible to implement? | | |
| **Judgement** | **Research evidence** | **Additional considerations** |
| ○ No  ○ Probably no  ○ Probably yes  ○ Yes  ● Varies  ○ Do not know | Implementation of the protocol requires sedation and pain scales for pediatric patients. Although it may be possible to introduce these scales in PICUs by adjusting protocols or solving problems related to the authority to change drug doses, the workload for education and dissemination of these scales is expected to be high in ICUs that do not specialize in pediatric patients. Therefore, the feasibility of the intervention may vary from facility to facility. |  |

**Summary of Judgement**

|  | **JUDGEMENT** | | | | | | |
| --- | --- | --- | --- | --- | --- | --- | --- |
| **PROBLEM** | No | Probably no | **Probably yes** | Yes |  | Varies | Do not know |
| **DESIRABLE EFFECTS** | Trivial | **Small** | Moderate | Large |  | Varies | Do not know |
| **UNDESIRABLE EFFECTS** | Large | Moderate | Small | **Trivial** |  | Varies | Do not know |
| **CERTAINTY OF EVIDENCE** | Very low | Low | **Moderate** | High |  |  | No included studies |
| **VALUES** | Important uncertainty or variability | Possibly important uncertainty or   variability | Probably no important uncertainty or variability | **No important uncertainty or variability** |  |  |  |
| **BALANCE OF EFFECTS** | Favors the comparison | Probably favors the comparison | Does not favor either the intervention or the comparison | **Probably favors the intervention** | Favors the intervention | Varies | Do not know |
| **ACCEPTABILITY** | No | Probably no | **Probably yes** | Yes |  | Varies | Do not know |
| **FEASIBILITY** | No | Probably no | Probably yes | Yes |  | **Varies** | Do not know |

**Type of Recommendation**

| Strong recommendation against the intervention | Conditional recommendation against the intervention | Conditional recommendation for either the intervention or the comparison | Conditional recommendation for the intervention | Strong recommendation for the intervention |
| --- | --- | --- | --- | --- |
| ○ | ○ | ○ | ● | ○ |

**CONCLUSION**

| **Recommendation** |
| --- |
| We suggest the use of protocols for the sedation of pediatric respiratory failure patients (weak recommendation / moderate certainty of evidence: GRADE 2B).  Supplementary item:  The sedation protocol examined is based on an algorithm in which nurses assess and adjust sedation and analgesia on a scale. |
|  |
| **Justification** |
| **Question：**Should a protocol be used for the sedation of pediatric respiratory failure patients?  **Population：** Pediatric patients (as defined in the article, 20 years old or younger if not specified) on ventilators for ARDS who meet one of the following criteria: AECC criteria, Berlin criteria, or PALICC criteria  or  Pediatric patients requiring ventilatory management for more than 12 hours in intensive care unit  **Intervention：**Protocolized sedation (with or without Spontaneous awakening trial or Daily sedation interruption)  **Comparison：**Conventional sedation without any protocols  **Main outcomes**：Mortality, Developmental prognosis, Duration of mechanical ventilation, Length of hospital stay, Adverse events  **Summary of evidence**：  The results of the systematic review showed that there was one RCT1 (N=2449 patients) comparing protocolized sedation with business-as-usual, and although it was not reported to be limited to patients with ARDS and included children who required ventilatory management, it was deemed appropriate for consideration in this CQ. Outcomes of significant benefit of protocolized sedation for pediatric ventilatory management included death with a risk difference of 13 fewer deaths/1000 patients, (95% CI: 25 fewer to 4 more deaths), duration of ventilation with a mean difference of ±0 days (95% CI: 0.46 days shorter to 0.46 days longer), and hospital stay with a mean difference of 2 days shorter (95% CI: 3.04 days shorter to 0.96 days longer), and developmental outcomes were not reported. Based on the above, we judged the effect to be "small. On the other hand, there were no reports of absolute numbers of adverse events such as unplanned extubation or worsening of respiratory status as serious harm outcomes. Therefore, we reviewed the literature (Curley 2015),^1^ which was included in this systematic review, and determined that the undesirable effect of withdrawal syndrome as a significant harm outcome was "slight," with a risk difference of 29 more patients/1000 (95% CI: 4 fewer to 60 more patients). Therefore, a weak recommendation was made.  **Overall certainty of evidence**：  Since the direction of the critically important outcomes extracted in the systematic review was consistent, the highest certainty of evidence for the overall outcome, "moderate," was adopted.  **Balance of effect, acceptability and feasibility**：  The predicted benefits of this intervention may outweigh the harms, but they are not significant, and the balance of effects was judged to be "probably in favor of the intervention. The intervention does not require any new drugs or equipment to be used in daily practice, and the cost burden is estimated to be negligible because of the possibility that medical costs themselves will be reduced due to the decrease in hospital stays. On the other hand, doctors and nurses need to be trained and human resources are needed. The introduction of a sedation/analgesia scale for pediatric patients may be a significant burden, especially in ICUs where there are few opportunities to treat children. Based on the above, the feasibility of the project was judged to be "difficult to say”. Acceptance is not expected to be a problem.  **Panel meeting**：  In the pre-vote, the modified Delphi method resulted in the following recommendation "We suggest the use of protocols for the sedation of pediatric respiratory failure patients (weak recommendation / moderate certainty of evidence: GRADE 2B). " and supplementary items: "It should be noted that this RCT was conducted in children who required ventilatory management, not in patients with ARDS. In addition, the sedation protocol used in the RCT was based on an algorithm in which nurses assessed sedation depth and pain using a scale and adjusted analgesia and sedation toward defined goals." was agreed upon, with a median score of 8 and a disagreement index of 0.0000.  At the panel meeting, it was suggested that the need to introduce a sedation/analgesia scale for pediatric patients should be added as a supplementary note to the protocol. Therefore, the supplementary items should be changed as follows: "The sedation protocol examined is based on an algorithm in which nurses assess and adjust sedation and analgesia on a scale.” and re-voted. Using the modified Delphi method, a consensus was reached with a median score of 9 and a disagreement index of 0.1316.  **Additional considerations**：  It is important to note that the RCT was conducted in children who required mechanical ventilation, not in patients with ARDS. In addition, the sedation protocol used in the RCT was based on an algorithm in which nurses assessed the depth of sedation and pain using scales, and adjusted the analgesia and sedation to achieve a set goal. |

| **Subgroup considerations** |
| --- |
| None. |
| **Implementation considerations** |
| Although we were unable to find any RCTs limited to pediatric ARDS patients, we believe that it would not be a major problem in targeting children requiring ventilatory management, because the sedation protocol assumed that the patients were weaned from the ventilator and were out of the acute phase of ARDS.  There have been no reports of absolute numbers of unplanned extubation or worsening of respiratory status as outcomes of harm; therefore, caution is needed. In addition to avoiding overdose of sedatives, it is also important to consider the possibility of ventilator weaning on a daily basis.^2^ Furthermore, sedation protocols are not uniformly applicable to all patients, and the content of the protocol and the indicated patient group should be considered on an individual basis. |

| **Monitoring and evaluation** |
| --- |
| In order to implement the recommendations, it is necessary to evaluate whether the total amount of sedatives used is decreasing, the degree of decrease in complications (such as duration of ventilation, delirium and withdrawal syndrome) due to the review of excessive sedation, and the increase or decrease in medical costs. It is also necessary to monitor how well protocols are followed through questionnaires. |
| **Research priorities** |
| Since there was only one RCT in this study, we did not examine whether the content of the protocol affected the results. It is necessary to conduct similar studies in the future and conduct another meta-analysis. We also defined developmental prognosis and adverse events as outcomes, but no studies have reported these outcomes. |

References

1. Curley MA, Wypij D, Watson RS, et al. Protocolized sedation vs usual care in pediatric patients mechanically ventilated for acute respiratory failure: a randomized clinical trial. Jama 2015;313:379-89.

2. Pediatric acute respiratory distress syndrome: consensus recommendations from the Pediatric Acute Lung Injury Consensus Conference. Pediatric critical care medicine : a journal of the Society of Critical Care Medicine and the World Federation of Pediatric Intensive and Critical Care Societies 2

**PCQ15 Should daily sedation interruption (DSI) be implemented for pediatric respiratory failure patients?**

1.Search strategy

MEDLINE via Pubmed (Search date: 2020/6/18）

| #1 | Respiratory Distress Syndrome, Adult[mh] OR ARDS[tiab] OR respiratory distress syndrome[tiab] OR Acute Lung Injury[mh] OR acute lung injury[tiab] OR ALI[tiab] |
| --- | --- |
| #2 | Critical Illness[mh] OR Critical Care[mh] OR Intensive Care Units[mh] OR critically ill[tiab] OR critical care[tiab] OR intensive care[tiab] |
| #3 | #1 OR #2 |
| #4 | Respiration, Artificial[mh] OR ventilators, mechanical[mh] OR ventilator[tiab] OR Ventilators[tiab] OR ventilation[tiab] OR ventilations[tiab] |
| #5 | sedation*[tiab] OR deep sedation[mh] OR Conscious Sedation[mh] OR Hypnotics and Sedatives[mh] |
| #6 | #3 AND #4 AND #5 |
| #7 | (randomized controlled trial[pt] OR controlled clinical trial[pt] OR randomized[tiab] OR placebo[tiab] OR clinical trials as topic[mesh:noexp] OR randomly[tiab] OR trial[ti]) NOT (animals[mh] NOT humans[mh]) |
| #8 | #6 AND #7 |

CENTRAL (Search date: 2020/6/18）

| #1 | [mh "Respiratory Distress Syndrome, Adult"] OR ARDS:ti,ab OR "respiratory distress syndrome":ti,ab OR [mh "Acute Lung Injury"] OR "acute lung injury":ti,ab |
| --- | --- |
| #2 | [mh "Critical Illness"] OR [mh "Critical Care"] OR [mh "Intensive Care Units"] OR "critically ill":ti,ab OR "critical care":ti,ab OR "intensive care":ti,ab |
| #3 | #1 OR #2 |
| #4 | [mh "Respiration, Artificial"] OR [mh "ventilators, mechanical"] OR ventilator:ti,ab OR Ventilators:ti,ab OR ventilation:ti,ab OR ventilations:ti,ab |
| #5 | sedation*:ti,ab OR [mh "deep sedation"] OR [mh "Conscious Sedation"] OR [mh "Hypnotics and Sedatives"] |
| #6 | #3 AND #4 AND #5 |

Igaku-Chuo-Zasshi (Search date: 2020/6/18）

| #1 | 呼吸窮迫症候群-急性/TH or 急性呼吸窮迫症候群/TA or ARDS/TA |
| --- | --- |
| #2 | クリティカルケア/TH or クリティカルケア/TA |
| #3 | ICU/TH or ICU/TA |
| #4 | 危篤/TH or 危篤/TA |
| #5 | #1 or #2 or #3 or #4 |
| #6 | 人工呼吸/TH or 人工呼吸器/TH or 人工呼吸/TA |
| #7 | 鎮静度/TH or 催眠剤と鎮静剤/TH or 鎮静/TA |
| #8 | #5 and #6 and #7 |
| #9 | (ランダム化比較試験/TH or 準ランダム化比較試験/TH or ランダム化/AL or 無作為化/AL or 比較試験/AL or 臨床試験/AL or プラセボ/AL or 対照/AL or コントロール/AL or 臨床研究/AL) and (PT=会議録除く) |
| #10 | #8 and #9 |

1. Flow diagram

1,138 records after duplicates removed

3 Studies included in qualitative synthesis

1,114 records excluded

1,597 records identified through database searching

Medline via PubMed (n=628)

CENTRAL (n=926)

Igaku-Chuo-Zasshi (n=43)

21 Full-text articles excluded, with reasons:

・

・Wrong study design (n=7)

・Wrong publication type (n=5)

・Wrong population (n=8)

・Wrong intervention (n=1)

0 additional records identified through other sources

1,597 records identified through database searching

Duplicates

n=459

24 Full-text articles assessed for eligibility

**Identification**

**Screening**

**Eligibility**

3 Studies included in quantitative synthesis (meta-analysis)

**Included**

1. Risk of bias

Mortality Duration of mechanical ventilation


Length of hospital stay Length of ICU stay


Withdrawal syndrome Unplanned extubation

1. Forest plot

Mortality

Duration of mechanical ventilation

Length of hospital stay

Length of ICU stay

Withdrawal syndrome

Unplanned extubation

1. Evidence profile

| **Assessment of certainty** | | | | | | | **No. of patients** | | **Efficacy** | | **Certainty of the evidence** | **Importance** |
| --- | --- | --- | --- | --- | --- | --- | --- | --- | --- | --- | --- | --- |
| **No. of studies** | **Study design** | **Risk of bias** | **Inconsitency** | **Indirectness** | **Imprecision** | **Others** | **Daily sedation interruption** | **control** | **Relative index (95% CI)** | **Absolute index (95% CI)** |  |  |
| **Mortality** | | | | | | | | | | | | |
| 3 | RCT | Not serious | Not serious | Not serious | Very serious ^a^ | None | 18/127 (14.2%) | 15/134 (11.2%) | **RR 2.48** (0.18 to 33.68) | **+166 per 1,000patients**  (-92 to +1000) | ⨁⨁◯◯ 低Low | Critical |
| \|  \| **Developmental prognosis** \| \| \| --- \| --- \| --- \| \|  \| \| Critical \|   **Duration of mechanical ventilation** | | | | | | | | | | | | |
| 3 | RCT | Serious ^b^ | Serious ^c^ | Not serious | Serious ^d^ | None | 127 | 134 | - | **MD 1.48 days shorter** (-3.48 to +0.51) | ⨁◯◯◯ Very low | Critical |
| **Length of Hospital stay** | | | | | | | | | | | | |
| 1 | RCT | Not serious | Not serious | Not serious | Serious ^e^ | None | 66 | 63 | - | **MD 3.4 days shorter** (-8.84 to +2.04) | ⨁⨁⨁◯ Moderate | Critical |
| **Length of ICU stay** | | | | | | | | | | | | |
| 3 | RCT | Serious ^b^ | Serious ^f^ | Not serious | Serious ^d^ | None | 127 | 134 | - | **MD 2.25 days shorter**  (-4.64 to +0.14) | ⨁◯◯◯ Very low | Important |
| **Withdrawal syndrome** | | | | | | | | | | | | |
| 1 | RCT | Serious ^g^ | Not serious | Not serious | Serious ^h^ | None | 32/317 (10.1%) | 66/540 (12.2%) | **RR 0.83** (0.55 to 1.23) | **-21 per 1,000 patients**  (-55 to +28) | ⨁⨁◯◯ Low | Important |
| **Unplanned extubation** | | | | | | | | | | | | |
| 3 | RCT | Not serious | Not serious | Not serious | Very serious ^i^ | None | 2/127 (1.6%) | 6/134 (4.5%) | **RR 0.42** (0.09 to 1.86) | **-26 per 1,000 patients**  (-41 to +39) | ⨁⨁◯◯ Low | Critical |

**CI:** Confidence interval; **RR:** Risk ratio; RCT: randomized controlled trial, MD: mean difference

**Explanations**

a. The total sample size is 216, which did not meet the optimal information size (OIS). The overall imprecision is downgraded by two levels due to the wide range of the 95% confidence interval across the treatment thresholds for benefit and harm.

b. Since two of the three studies were judged to be high risk, they were downgraded by one grade.

c. I2 = 63%, which was judged to be "serious" and downgraded by one level based on a visual forest plot of the variability of the results of each study included in the systematic review.

d. The total sample size was 261, which did not meet the OIS and was therefore downgraded by one level.

e. The total sample size was 129, which did not meet the OIS and was therefore downgraded by one level.

f. I2 = 57%, which was judged to be "serious" and downgraded by one level based on a visual forest plot of the variability of the results of each study included in the systematic review.

g. One target article was downgraded by one level because it contained seven items, one of which was high risk and one of which was an unknown risk.

h. The total sample size is 857, which did not meet the OIS; the width of the 95% confidence interval straddles the benefits treatment threshold; therefore, the overall imprecision was downgraded by one level.

i. The total sample size was 261, which did not meet the OIS; the overall imprecision was downgraded by two levels due to the wide range of the 95% confidence interval, which spans the treatment thresholds for benefit and harm.

No articles reported developmental prognosis.

1. Evidence-to-Decision table

| **QUESTION** | |
| --- | --- |
| **PCQ15：** Should daily sedation interruption (DSI) be implemented for pediatric respiratory failure patients? | |
| **POPULATION:** | Pediatric patients (as defined in the article, 20 years old or younger if not specified) on ventilators for ARDS who meet one of the following criteria: AECC criteria, Berlin criteria, or PALICC criteria  or  Pediatric patients requiring ventilatory management for more than 12 hours in intensive care unit |
| **INTERVENTION:** | DSI |
| **COMPARISON:** | Conventional sedation without DSI |
| **MAIN OUTCOMES:** | Mortality, Developmental prognosis, Duration of mechanical ventilation, Length of hospital stay, Unplanned extubation |
| **SETTINGS:** | Emergency department or intensive care unit (ICU) |
| **PERSPECTIVE:** | Individual |
| **BACKGROUND:** | No sedation/light sedation/protocolized sedation is expected to effectively avoid complications of deep sedation because the use of large doses of sedatives increases the risk of prolonged ventilation, delirium, and withdrawal symptoms, although adequate sedation is sometimes necessary for ventilatory management. In the ventilatory management of pediatric acute respiratory distress syndrome (ARDS)　patients, no sedation/light sedation/protocolized sedation is not a well-established treatment. Therefore, clarifying its efficacy is an important clinical issue.  We did not limit the target population to ARDS patients because daily sedation interruption (DSI) is an intervention based on ventilator weaning, and its usefulness is not expected to differ between ARDS and non-ARDS patients. |
| **CONFLICT OF INTERESTS:** | None |

**ASSESSMENT**

| **Problem**  Is the problem a priority? | | |
| --- | --- | --- |
| **Judgement** | **Research evidence** | **Additional considerations** |
| ○ No  ○ Probably no  ○ Probably yes  ● Yes  ○ Varies  ○ Do not know | Ventilatory management requires daily assessment of the patient's ability to be weaned from the ventilator. In pediatric ventilatory management, large doses of sedatives may be required if the patient does not cooperate with rest, which may interfere with the assessment of extubation feasibility. In adult patients, DSI is effective for early weaning from ventilation. However, in pediatric patients, when DSI increases arousal, unscheduled extubation can be expected. Therefore, clarifying the effectiveness of DSI in the ventilatory management of pediatric patients with ARDS is an important clinical issue and of high priority. |  |
| **Desirable Effects**  How substantial are the desirable anticipated effects? | | |
| **Judgement** | **Research evidence** | **Additional considerations** |
| ○ Trivial  ● Small  ○ Moderate  ○ Large  ○ Varies  ○ Do not know | Three randomized controlled trials (RCT)^1-3^ consistent with PICO were included in the systematic review and used in the meta-analysis.  As beneficial outcomes, the estimate of the effect for the duration of mechanical ventilation (3 RCTs^1-3^, N=261) has a mean difference of 1.48 days shorter (95% CI: 3.48 shorter to 0.51 longer) and for length of hospital stay (1 RCT^3^, N=129), with a mean difference of 3.4 days shorter (95% CI: 8.84 shorter to 2.04 longer).  We assumed that a reduction in mortality was a beneficial outcome. However, as discussed below, mortality increased in the intervention group. No studies have reported the outcomes of the developmental prognosis.  Therefore, considering the decrease in duration of mechanical ventilation and the length of hospital stay, we deemed the desirable effect of the intervention to be "small.” |  |
| **Undesirable Effects**  How substantial are the undesirable anticipated effects? | | |
| **Judgement** | **Research evidence** | **Additional considerations** |
| ○ Large  ● Moderate  ○ Small  ○ Trivial  ○ Varies  ○ Do not know | As harmful outcomes, the estimate of the effect for mortality (3 RCTs^1-3^, N=261) was 166 more per 1,000 (95% CI: 92 fewer to 1000 more) and for unplanned extubation (3 RCTs^1-3^, N=261), it was 26 fewer per 1,000 (95% CI: 41 fewer to 39 more) in the intervention group compared with that in the control group. Therefore, the undesirable effect of the intervention was considered "moderate." |  |
| **Certainty of evidence**  What is the overall certainty of the evidence of effects? | | |
| **Judgement** | **Research evidence** | **Additional considerations** |
| ● Very low  ○ Low  ○ Moderate  ○ High  ○ No included studies | **The relative importance or values of the main outcomes of interest:**   \| **Outcome** \| **Importance** \| **Certainty of the Evidence**  **(GRADE)** \| \| --- \| --- \| --- \| \| Mortality \| Critical \| ⨁⨁◯◯ \| \| Low \| \| Duration of mechanical ventilation \| Critical \| ⨁◯◯◯ \| \| Very low \| \| Length of hospital stay \| Critical \| ⨁⨁⨁◯ \| \| Moderate \| \| Adverse events (unplanned extubation、worsening respiratory status, etc.) \| Critical \| ⨁⨁◯◯ \| \| Low \| \| Developmental prognosis* \| Critical \| - \|   *There were no outcome reports on developmental prognosis in the accepted literature.  **Overall certainty of evidence**  Since the direction of the critically important outcomes extracted in the systematic review was inconsistent, the lowest certainty of evidence for the overall outcome, "very low," was adopted. |  |
| **Values**  Is there important uncertainty about or variability in how much people value the main outcomes? | | |
| **Judgement** | **Research evidence** | **Additional considerations** |
| ○ Important uncertainty or variability  ○ Possibly important uncertainty or variability  ○ Probably no important uncertainty or variability  ● No important uncertainty or variability | Currently, there is no evidence of the relative importance that children and their parents attach to each outcome of this intervention. However, “mortality” is generally a critical outcome, and there is no great uncertainty or diversity of values regarding this. |  |
| **Balance of effects**  Does the balance between desirable and undesirable effects favor the intervention or the comparison? | | |
| **Judgement** | **Research evidence** | **Additional considerations** |
| ○ Favors the comparison  ● Probably favors the comparison  ○ Does not favor either the intervention or the comparison  ○ Probably favors the intervention  ○ Favors the intervention  ○ Varies  ○ Do not know | **Summary of evidence**   \| **Outcome** \| **Control** \| **Intervention** \| **Absolute difference**  **(95% CI)** \| **Risk ratio (RR)**  **(95% CI)** \| \| --- \| --- \| --- \| --- \| --- \| \| Mortality \| 15/134  (11.2%) \| 18/127  (14.2%) \| 166 more per 1,000  (92 fewer to 1,000 more) \| RR 2.48  (0.18-33.68) \| \| Duration of mechanical ventilation \| - \| - \| MD 1.48 day shorter  (3.48 shorter to 0.51 longer) \| - \| \| Length of hospital stay \| - \| - \| MD 3.4 day shorter  (8.84 shorter to 2.04 longer) \| - \| \| Adverse events \| 6/134  (4.5%) \| 2/127  (1.6%) \| 26 fewer per 1,000  (41 fewer to 39 more) \| RR 0.42  (0.09-1.86) \| \| Developmental prognosis \| - \| - \| - \| - \|   Based on the above, we determined that the balance between desirable and undesirable effects was "probably favors the comparison.” |  |
| **Acceptability**  Is the intervention acceptable to key stakeholders? | | |
| **Judgement** | **Research evidence** | **Additional considerations** |
| ○ No  ○ Probably no  ○ Probably yes  ○ Yes  ● Varies  ○ Do not know | If the intervention is expected to have desirable effects, it is considered acceptable to patients. On the other hand, the patient's family may feel distressed when they see their children showing discomfort with the tracheal tube. Based on the above, it was determined that the acceptability of key stakeholders can vary. |  |
| **Feasibility**  Is the intervention feasible to implement? | | |
| **Judgement** | **Research evidence** | **Additional considerations** |
| ○ No  ○ Probably no  ○ Probably yes  ○ Yes  ● Varies  ○ Do not know | The introduction of DSI requires sedation and pain scales for pediatric patients, which may be feasible in PICUs by adjusting protocols or solving problems related to the authority to change drug doses. However, in ICUs that do not specialize in pediatric patients, the human burden of educating and disseminating these scales is expected to be high. Therefore, the feasibility of this intervention may vary from facility to facility. |  |

**SUMMARY OF JUDGEMENT**

|  | **JUDGEMENT** | | | | | | |
| --- | --- | --- | --- | --- | --- | --- | --- |
| **PROBLEM** | No | Probably no | Probably yes | **Yes** |  | Varies | Do not know |
| **DESIRABLE EFFECTS** | Trivial | **Small** | Moderate | Large |  | Varies | Do not know |
| **UNDESIRABLE EFFECTS** | Large | **Moderate** | Small | Trivial |  | Varies | Do not know |
| **CERTAINTY OF EVIDENCE** | **Very low** | Low | Moderate | High |  |  | No included studies |
| **VALUES** | Important uncertainty or variability | Possibly important uncertainty or   variability | Probably no important uncertainty or variability | **No important uncertainty or variability** |  |  |  |
| **BALANCE OF EFFECTS** | Favors the comparison | **Probably favors the comparison** | Does not favor either the intervention or the comparison | Probably favors the intervention | Favors the intervention | Varies | Do not know |
| **ACCEPTABILITY** | No | Probably no | Probably yes | Yes |  | **Varies** | Do not know |
| **FEASIBILITY** | No | Probably no | Probably yes | Yes |  | **Varies** | Do not know |

**Type of Recommendation**

| Strong recommendation against the intervention | Conditional recommendation against the intervention | Conditional recommendation for either the intervention or the comparison | Conditional recommendation for the intervention | Strong recommendation for the intervention |
| --- | --- | --- | --- | --- |
| ○ | ● | ○ | ○ | ○ |

**CONCLUSION**

| **Recommendation** |
| --- |
| We suggest against implementing DSI for pediatric respiratory failure patients (weak recommendation / very low certainty of evidence：GRADE 2D).  Supplementary item:  There is currently insufficient evidence for providing a recommendation for DSI. It is important to evaluate on a daily basis whether withdrawal from a ventilator is possible. |
|  |
| **Justification** |
| **Question**：　Should DSI be implemented for pediatric respiratory failure patients?  **Population**：　Pediatric patients (as defined in the article, 20 years old or younger if not specified) on ventilators for ARDS who meet one of the following criteria: AECC criteria, Berlin criteria, or PALICC criteria  or  Pediatric patients requiring ventilatory management for more than 12 hours in intensive care unit  **Intervention**：　DSI  **Comparison**：　Conventional sedation without DSI  **Main outcomes**：　Mortality, Developmental prognosis, Duration of mechanical ventilation, Length of hospital stay, Unplanned extubation  **Summary of evidence**：  The results of the systematic review showed that there were 3 RCTs^1-3^ (N=261) comparing sedation management with and without DSI. The outcomes of benefit of sedation with DSI for pediatric ventilatory management were 1.48 days shorter duration of ventilation (95% CI: 3.48 days shorter to 0.51 days longer), 3.4 days shorter hospital stay (95% CI: 8.84 days shorter to 2.04 days longer), and no developmental outcomes were reported. Mortality was assumed to be an outcome of benefit, but it increased with intervention. Therefore, the desired effect was judged to be "small. On the other hand, the undesirable effect outcomes were 166 more deaths/1000 patients (95% CI: 92 fewer to 1000 more) and 26 fewer unplanned extubations/1000 patients (95% CI: 41 fewer to 39 more). Thus, the undesirable effect was judged to be "moderate".  It should be noted that the increase in deaths in this meta-analysis was influenced by one RCT (Vet 2016)^3^, which showed no increase in adverse events in the DSI group, a decreasing trend in ventilatory duration and hospital stay, and a decreasing rate of reintubation, making it difficult to explain the causal effect that led to the increase in deaths. RCTs in adults do not show a significant increase in mortality due to DSI^4^.  **Overall certainty of evidence**：  Since the direction of the critically important outcomes extracted in the systematic review was inconsistent, the lowest certainty of evidence for the overall outcome, "very low," was adopted.  **Balance of effect, acceptability and feasibility**：  Although the decrease in drug costs is not expected to be large, the introduction of a sedation scale for children is necessary, and the burden of observation after interruption of sedation is estimated to be large in the ICU, which has few opportunities to see children. In addition, there is a concern about an increase in deaths, although this is not significant, so the undesirable effects are greater in the intervention group in terms of the balance of effects including costs. In addition, the family of the child may have difficulty accepting the situation in which the child is uncomfortable with the tracheal tube due to the interruption of sedation. Based on the above, we judged that the feasibility of the intervention was not entirely clear.  **Panel meeting**：  In the pre-vote, a modified Delphi method resulted in a recommendation “We suggest against implementing DSI for pediatric respiratory failure patients (weak recommendation / very low certainty of evidence：GRADE 2D). " with supplementary conditions: "The RCTs included in this study were not in patients with ARDS but in children who required ventilatory management, and there is a lack of evidence in patients with ARDS. Of the three RCTs according to mortality, one RCT (Vet 2016) with a large sample size found 0/63 deaths in the control group versus 6/66 in the intervention group. The meta-analysis was heavily influenced by this result. The authors state that the causal relationship between the intervention and increased mortality is unclear." was agreed upon, with a median score of 8 and a disagreement index of 0.0000.  At the panel meeting, it was suggested that ancillary information should not refer only to specific RCTs, and that the need to assess whether daily ventilator weaning is feasible should be mentioned. Therefore, the supplementary conditions should be changed as follows: "There is currently insufficient evidence for providing a recommendation for DSI. It is important to evaluate on a daily basis whether withdrawal from a ventilator is possible.” and re-voted. Using the modified Delphi method, a consensus was reached with a median score of 9 and a disagreement index of 0.0000.  **Additional considerations**：  This RCT was not in patients with ARDS but in children who required ventilatory management. At this time, there is insufficient evidence to recommend complete sedation interruption in pediatric patients with ARDS, but it is necessary to evaluate whether daily ventilator weaning is feasible. |

| **Subgroup considerations** |
| --- |
| None |
| **Implementation considerations** |
| Before implementing DSI, it is necessary to consider whether weaning from the ventilator is possible.  In all RCTs reviewed in this CQ, DSI means complete interruption of sedatives and analgesics. Although this meta-analysis did not increase the number of unplanned extubations, all of the included RCTs were performed in the PICU, which may increase the risk of DSI depending on the medical and nursing systems of each institution. When implementing DSI, consideration must be given to safety, including the establishment of a nursing system. It is also necessary to evaluate whether a sufficient level of analgesia has been achieved or explained to the patients’ families that the patients may temporarily show discomfort with the tracheal tube during DSI.  Although RCTs limited to pediatric ARDS patients were not found in the search, we surmise that it would not be a hindrance in targeting children requiring mechanical ventilation because DSI is an intervention based on ventilator weaning and its usefulness is not expected to differ between ARDS and non-ARDS patients. |

| **Monitoring and evaluation** |
| --- |
| When implementing this intervention, it is necessary to ensure that there is no increase in adverse events or mortality after implementation. |
| **Research priorities** |
| One of the three RCTs used in this study (Vet 2016)^3^ found an increase in mortality of 6/66 in the intervention group compared to 0/63 in the control group. The authors note that similar studies involving adults have not previously reported an increase in mortality with intervention, making it difficult to explain the causal effect of increased mortality in the intervention group. In addition, in all three RCTs, mortality was not the primary endpoint, and the increase in mortality due to the intervention was not significant. Therefore, there is insufficient evidence to conclude whether this intervention increases mortality in children. The direction of the recommendation may change as evidence from future studies accumulates. |

References

1. Gupta K, Gupta VK, Jayashree M, Singhi S. Randomized controlled trial of interrupted versus continuous sedative infusions in ventilated children. Pediatric critical care medicine : a journal of the Society of Critical Care Medicine and the World Federation of Pediatric Intensive and Critical Care Societies 2012;13:131-5.

2. Verlaat CW, Heesen GP, Vet NJ, et al. Randomized controlled trial of daily interruption of sedatives in critically ill children. Paediatric anaesthesia 2014;24:151-6.

3. Vet NJ, de Wildt SN, Verlaat CW, et al. A randomized controlled trial of daily sedation interruption in critically ill children. Intensive care medicine 2016;42:233-44.

4. Burry L, Rose L, McCullagh IJ, Fergusson DA, Ferguson ND, Mehta S. Daily sedation interruption versus no daily sedation interruption for critically ill adult patients requiring invasive mechanical ventilation. The Cochrane database of systematic reviews 2014;2014:Cd009176.
