# Supplementary material for: ARDS Clinical Practice Guideline 2021
Source: J Intensive Care. 2022 Jul 8;10:32. doi: 10.1186/s40560-022-00615-6 (PMC9263056; doi:10.1186/s40560-022-00615-6)
Supplement: Supplementary file 7 — Additional file 7. Contains tables disclosing intellectual and financial conflicts of interest for each person who participated in the creation of this guideline [file 40560_2022_615_MOESM7_ESM.docx]

**Additional file 7**

**Disclosure of intellectual and financial conflicts of interest for each person who participated in the creation of this guideline**

■Japanese ARDS clinical practice guideline 2021 members

□Committee chair

Masamitsu Sanui, Department of Anesthesiology and Critical Care Medicine, Jichi Medical University Saitama Medical Center

Sadatomo Tasaka, Department of Respiratory Medicine, Hirosaki University Graduate School of Medicine

□ Vice chair

Shinichiro Ohshimo, Department of Emergency and Critical Care Medicine,Graduate School of Biomedical and Health Sciences, Hiroshima University

Muneyuki Takeuchi, Department of Intensive Care Medicine, Osaka Women's and Children's Hospital

□ Committee members

Hideto Yasuda, Department of Emergency and Critical Care Medicine, Jichi Medical University, Saitama Medical Center

Kazuya Ichikado, Division of Respiratory Medicine, Saiseikai Kumamoto Hospital

Kenji Tsushima, International University of Health and Welfare

Moritoki Egi, Department of anesthesiology, Kobe University hospital

Satoru Hashimoto, Department of Anesthesiology and Intensive Care Medicine, Kyoto Prefectural University of Medicine

□ Pediatrics committee

Nobuaki Shime, Department of Emergency and Critical Care Medicine, Graduate School of Biomedical & Health Sciences, Hiroshima University

Osamu Saito, Department of Pediatric Emergency and Critical Care Medicine, Tokyo Metropolitan Children’s Medical Center

Shotaro Matsumoto, Division of Critical Care Medicine, National Center for Child Health and Development

□ Methodological consultant

Eishu Nango, Department of Family Medicine, Seibo International Catholic Hospital

□ Supporting team members

Yohei Okada (Leader), Department of Primary care and Emergency medicine, Graduate school of medicine, Kyoto University

Kenichiro Hayashi, Department of Pediatrics, The University of Tokyo Hospital

Chihiro Narita, Department of Emergency medicine, Shizuoka General Hospital

Daisuke Kawakami, Department of Anesthesia and Critical Care, Kobe City Medical Center General Hospital

Hiromu Okano , Department of Critical care and Emergency Medicine, National Hospital Organization Yokohama Medical Center

Jun Takeshita, Department of Anesthesiology, Osaka Women's and Children's Hospital

Keisuke Anan, Department of Healthcare Epidemiology, Kyoto University, Graduate School of Medicine, Division of Respiratory Medicine, Saiseikai Kumamoto Hospital

Satoru Robert Okazaki, Department of Intensive Care Medicine, Kameda Medical Center

Shinya Miura, Paediatric Intensive Care Unit, The Royal Children's Hospital Melbourne

Shunsuke Taito, Division of Rehabilitation, Department of Clinical Practice and Support, Hiroshima University Hospital

Tadashi Ishihara, Department of Emergency and Critical Care Medicine, Juntendo University, Urayasu Hospital

Takuya Hayashi, Pediatric emergency and critical care center、Saitama Children's medical center a

Takuya Mayumi, Department of Cardiovascular Medicine, Graduate School of Medical Science, Kanazawa University

Tetsuro Terayama, Department of Psychiatry, National Defense Medical College

Yoshifumi Kubota, Kameda medical center department of infectious diseases

Yoshinobu Abe, Division of Emergency and Disaster Medicine Tohoku Medical and Pharmaceutical University

Yudai Iwasaki, Department of Anesthesiology and Perioperative Medicine, Tohoku University Graduate School of Medicine

Yuki Kishihara, Department of emergency medicine, Japanese Red Cross Musashino Hospital

■Panel committee

□Clinical question for adult

Eriko Takezawa, Patient’s family

Hiroshi Okuda, Department of General Internal Medicine, Obihiro Dai-ichi Hospital / Tohoku University Tohoku Medical Megabank Organization

Hiroshi Yoshikawa, Department of Pharmaceutical Services, Hiroshima University Hospital

Hitoshi Yokoyama, Department of Rehabilitation Center, St. Marianna University School of Medicine Hospital

Keiko Ishimura, Department of Public Health, Osaka University Graduate School of Medicine

Kokichi Andoh , Division of Anesthesiology,Division of Intensive Care

Makoto Miki, Department of Respiratory medicine, Japanese Red Cross Sendai Hospital

Masashi Morizane, Department of Clinical Engineering, Saiseikai Yokohamashi Tobi Hospital

Nana Arai, Department of Biomedical Ethics, Graduate School of Medicine, The University of Tokyo

Ryo Kozu, Department of Physical Therapy Science, Nagasaki University Graduate School of Biomedical Sciences

Ryutaro Seo, Department of Emergency Medicine, Kobe City Medical Center General Hospital

Satoshi Doi, Bachelor of Science in Nursing / Nursing Department , Tokushima University Hospital

Takeshi Yoshida, Department of Anesthesiology and Intensive Care Medicine, Osaka University Graduate School of Medicine, Suita, Japan

Yusuke Iizuka, Department of critical care and anesthesiology, Saitama medical center, Jichi medical university

□Recommendation for pediatrics

Nana Arai, Department of Biomedical Ethics, Graduate School of Medicine, The University of Tokyo

Akimasa Yamatani, Department of Pharmacy, National Center for Child Health and Development

Eriko Takezawa, Patient’s family representative

Junichi Ishikawa, Emergency and Critical Care Medical Center & Pediatric emergency medicine Osaka City General Hospital

Kensuke Yagi, Tokyo Metropolitan Children's Medical Center

Osamu Saito, Department of Pediatric Emergency and Critical Care Medicine, Tokyo Metropolitan Children’s Medical Center

Satoshi Doi, Bachelor of Science in Nursing/ Nursing Department , Tokushima University Hospital

Satoshi Nakagawa, Critical Care Medicine, National Center for Child Health and Development

Shinya Miura, Paediatric Intensive Care Unit, The Royal Children's Hospital Melbourne

Shunsuke Nosaka, National Center for Child Health and Development

Tatsuya Kawasaki, Department of Pediatric Critical Care, Shizuoka Children's Hospital

Yuki Enomoto, Department of Emergency and Critical care medicine, University of Tsukuba

Yusuke Iizuka, Department of critical care and anesthesiology, Saitama medical center, Jichi medical university

■Systematic review managing committee

□Committee chair

Shinichiro Ohshimo, Department of Emergency and Critical Care Medicine,Graduate School of Biomedical and Health Sciences, Hiroshima University

□Vice chair

Hideto Yasuda, Department of Emergency and Critical Care Medicine, Jichi Medical University, Saitama Medical Center

□Committee members

Kenichiro Hayashi, Department of Pediatrics, The University of Tokyo Hospital

Masaaki Sakuraya, Department of Emergency and Intensive Care Medicine, JA Hiroshima General Hospital

Mikio Nakajima, Emergency and Critical Care Center, Tokyo Metropolitan Hiroo Hospital, Tokyo, Japan

Satoshi Okamori, Keio University School of Medicine, Division of Pulmonary Medicine, Department of Medicine

Shinya Miura, Paediatric Intensive Care Unit, The Royal Children's Hospital Melbourne

Tatsuma Fukuda, Department of Emergency and Critical Care Medicine, Graduate School of Medicine, University of the Ryukyus

Tadashi Ishihara, Department of Emergency and Critical Care Medicine, Juntendo University, Urayasu Hospital

Tetsuro Kamo, Department of Critical Care Medicine, Tokyo Metropolitan Hospital

Tomoaki Yatabe, Department of Anesthesiology , Nishichita General Hospital

Yasuhiro Norisue, Tokyo Bay Urayasu Ichikawa Medical Center

Yoshitaka Aoki, Department of Anesthesiology and Intensive Care Medicine, Hamamatsu University School of Medicine

Yusuke Iizuka, Department of critical care and anesthesiology, Saitama medical center, Jichi medical university

Yutaka Kondo, Department of Emergency and Critical Care Medicine, Juntendo University Urayasu Hospital

□Systematic review supervising committee members

Chihiro Narita, Department of Emergency medicine, Shizuoka General Hospital

Jun Kataoka, Department of Critical Care Medicine, Nerima Hikarigaoka Hospital

Masayuki Ozaki, Department of Emergency and Critical Care Medicine, Komaki City Hospital

Tetsuro Nishimura, Department of Traumatology and Critical Care Medicine Osaka City University Graduate School of Medicine

Hiroshi Yonekura, Department of Anesthesiology and Pain Medicine, Fujita Health University Bantane Hospital

Koichi Ando, Division of Respiratory Medicine and Allergology, Department of Medicine, Showa University School of Medicine.

Shunsuke Taito, Division of Rehabilitation, Department of Clinical Practice and Support, Hiroshima University Hospital

Takuo Yoshida, Intensive Care Unit, Department of Anesthesiology, Jikei University School of Medicine

Tomoyuki Masuyama, Department of Emergency and Critical Care Medicine, Jichi Medical University Saitama Medical Center,

Yohei Okada, Department of Primary care and Emergency medicine, Graduate school of medicine,Kyoto University

□Systematic review team

Takuro Nakashima, Kumamoto University Hospital, Department of Intensive Care

Aiko Masunaga, Department of Respiratory Medicine, Kumamoto University Hospital

Aiko Tanaka, Department of Anesthesiology and Intensive Care Medicine, Graduate School of Medicine, Osaka University

Akihiko Inoue, Department of Emergency and Critical Care Medicine, Hyogo Emergency Medical Center

Akiko Higashi, Department of Emergency and Critical Care Medicine / Chiba University Graduate School of Medicine

Atsushi Tanikawa, Department of Emergency and Critical Care, Tohoku University Hospital

Atsushi Ujiro, Department of critical care, Osaka city general hospital

Chihiro Takayama, Takatsuki　General Hospital

Daisuke Kasugai, Department of Emergency and Critical Care Medicine, Nagoya University Graduate School of Medicine

Daisuke Kawakami, Department of Anesthesia and Critical Care, Kobe City Medical Center General Hospital

Daisuke Ueno, Department of Acute Medicine, Kawasaki Medical School

Daizoh Satoh, Department of Anesthesiology and Pain Medicine, Juntendo University School of Medicine

Shinichi Kai, Department of Anesthesia, Kyoto University Hospital

Kohei Ohta, Department of Emergency and Critical Care Medicine,  Graduate School of Biomedical and Health Sciences,  Hiroshima University

Yoshihiro Hagiwara, Department of Emergency and Critical Care Medicine, Saiseikai Utsunomiya Hospital

Jun Hamaguchi, Department of Emergency and Critical Care Medicine, Tokyo Metropolitan Tama Medical Center

Ryo Fujii, Department of Emergency Medicine and Critical Care Medicine, Tochigi prefectural emergency and critical care center, Imperial Foundation Saiseikai Utsunomiya Hospital

Takashi Hongo, Department of Emergency Medicine Okayama Saiseikai General Hospital

Yuki Kishihara, Department of emergency medicine, Japanese Red Cross Musashino Hospital

Naohisa Masunaga, Department of Healthcare Epidemiology, School of Public Health in the Graduate School of Medicine, Kyoto University

Ryohei Yamamoto, Department of Healthcare Epidemiology, School of Public Health in the Graduate School of Medicine, Kyoto University, Kyoto, Japan

Satoru Robert Okazaki, Department of Intensive Care Medicine, Kameda Medical Center

Ryo Uchimido, Department of Intensive Care Medicine, Tokyo Medical and Dental School

Tetsuro Terayama, Department of Psychiatry, National Defense Medical College

Satoshi Hokari, Department of Respiratory Medicine and Infectious Diseases, Niigata University Graduate School of Medical and Dental Sciences

Hitoshi Sakamoto, Department of Thoracic Surgery, Shimane Prefectural Central Hospital

Dongli, Department of Anesthesia, Kyoto University Hospital; Department of Healthcare Epidemiology, Kyoto University Graduate School of Medicine and Public Health

Emiko Nakataki, Department of Critical care medicine, Tokushima Central Prefectural Hospital

Erina Tabata, Department of Respiratory Medicine, Kanagawa Cardiovascular and Respiratory Medicine

Seisuke Okazawa, First Department of Internal Medicine, Toyama University Hospital, Toyama

Futoshi Kotajima, Department of Intensive Care Medicine, Saitama Medical School International Medical Center

Go Ishimaru, Soka Municipal Hospital

Haruhiko Hoshino, Department of nursing, International University of Health and Welfare

Hideki Yoshida, Department of Emergency and Critical Care Medicine, St. Marianna University School of Medicine

Hidetaka Iwai, Department of Anesthesia, Social medical corporation Hakuaikai Kaisei Hospital

Hiroaki Nakagawa, Division of Respiratory Medicine, Department of Internal Medicine, Shiga University of Medical Science

Hiroko Sugimura, Department of Critical Care, Chiba Children's Hospital

Hiromichi Narumiya, Division of Emergency and Critical Care, Japanese Red Cross Kyoto Daini Hospital

Hiromu Okano, Department of Critical care and Emergency Medicine, National Hospital Organization Yokohama Medical Center

Hiroshi Nakamura, Department of General Medicine, Kure Medical Center and Chugoku Cancer Center

Hiroshi Sugimoto, Department of Respiratory Medicine, Konan Medical Center

Hiroyuki Hashimoto, Department of Pharmacoepidemiology, Graduate School of Medicine and Public Health, Kyoto University

Hiroyuki Ito, Department of Pulmonology, Kameda Medical Center

Hisashi Dote, Department of Emergency and Critical Care Medicine, Seirei Hamamatsu General Hospital.

Hisashi Imahase, Graduate school of medicine, Tokyo University

Hitoshi Sato, Department of critical care, National Cerebral and Cardiovascular Center

Masahiro Katsurada, Hyogo Prefectural Tamba Medical Center

Ichiro Osawa, Department of Critical Care and Anesthesia, National Center for Child Health and Development

Jun Kamei, Emergency and Critical Care Center, Kurashiki Central Hospital

Jun Maki, Department of Critical Care Medicine, Kyushu University Hospital

Jun Sugihara, Department of pulmonology, Kashiwa municipal hospital.

Jun Takeshita, Department of Anesthesiology, Osaka Women's and Children's Hospital

Junichi Fujimoto, Department of intensivei care medicine, Yokohama Rosai Hospital

Junichi Ishikawa, Emergency and Critical Care Medical Center & Pediatric emergency medicine Osaka City General Hospital

Junko Kosaka, Department of Anesthesiology and Resuscitology, Okayama University Hospital

Junpei Shibata, Department of Anesthesiology and Critical Care Medicine, Fujita Health University School of Medicine

Katsuhiko Hashimoto, Department of Minimally Invasive Surgical and Medical Oncology, Fukushima Medical University, Fukushima, Japan

Yasushi Nakano, Kawasaki Municipal Ida Hospital

Kazuki Kikuyama, Department of Intensive Care Unit, Showa University Hospital

Kazushige Shimizu, Department of Clinical Engineer

Kazuya Okada, Department of intensive care, Yokosuka General Hospital Uwamachi

Keishi Kawano , Department of Anesthesiology and Resuscitology, Okayama University Hospital

Keisuke Anan, Department of Healthcare Epidemiology, Kyoto University, Graduate School of Medicine, Division of Respiratory Medicine, Saiseikai Kumamoto Hospital

Keisuke Ota, Intensive care center,Shizuoka General Hospital

Ken-ichi Kano, Department of Emergency medicine, Fukui Prefectural Hospital

Kengo Asano, Intensive Care Unit, Department of Anesthesiology, Jikei University School of Medicine

Kenichi Hondo, Department of Trauma and Acute Critical Care Center, Tokyo Medical and Dental University Hospital

Kenji Ishii, Intesive Care unit , St Lukes international hospital

Kensuke Fujita, Department of Emergency Medicine and Critical Care Medicine, Tochigi prefectural emergency and critical care center, Imperial Foundation Saiseikai Utsunomiya Hospital

Kenta Ogawa, Intensive Care Unit, Department of Anesthesiology, Jikei University School of Medicine

Kentaro Ito, Respiratory Center, Matsusaka Municipal Hospital

Kentaro Tokunaga, Department of Intensive Care Medicine, Kumamoto University Hospital

Kenzo Ishii, Department of Anesthesiology, Intensive Care Unit, Fukuyama City Hospital

Kohei Kusumoto, Department of pediatric critical care medicine, Hyogo prefectural Amagasaki General Medical Center

Kohei Takimoto, Department of Intensive Care Medicine, Kameda Medical Center

Kohei Yamada, Department of Traumatology and Critical Care Medicine, National Defense Medical College

Koichi Naito, Department of Cardiac Rehabilitation, Iwama Cardiovascular and Dental Clinic for Prevention and Care

Koichi Yamashita, Division of Critical Care Center, Kochi Red Cross Hospital

Koichi Yoshinaga, Department of Anesthesiology and Critical Care, Jichi Medical University, Saitama Medical Center

Kota Yamauchi, Department of Rehabilitation, Steel Memorial Yawata Hospital

Maki Murata, Department of Healthcare Epidemiology, Kyoto University of Graduate School of Medicine

Makiko Konda, Department of Anesthesiology , Nara Medical University

Manabu Hamamoto, Division of Emergency & Critical Care Medicine, Saitma Children's Medical Center

Masaharu Aga, Department of Respiratory Medicine, Yokohama Municipal Citizen's Hospital

Masahiro Kashiura, Department of Emergency and Critical Care Medicine, Jichi Medical University Saitama Medical Center

Masami Ishikawa, Department of Anesthesiology and Critical Care Medicine of Kure Kyousai Hospital

Michihiko Kono, Department of intensive Care Unit ,Sakai City Medical CenterMedicalcare unit

Michihito Kyo, Department of Emergency and Critical Care Medicine, Graduate School of Biomedical and Health Sciences, Hiroshima University

Minoru Hayashi, Fukui Prefectural Hospital

Mitsuhiro Abe, Respiratory Medicine, Chiba University Hospital

Mitsunori Sato, Department of pediatric critical care, Shizuoka children's hospital

Mizu Sakai, Department of Respiratory medicine and Allergology, Kochi Medical School, Kochi University

Motoshi Kainuma, Anesthesiology, Emergency Medicine, and Intensive Care Division Inazawa Municipal Hospital

Naoki Tominaga, Department of Emergency and Critical Care Medicine, Nippon Medical School

Naoya Iguchi, Department of Anesthesiology and Intensive Care Medicine, Graduate School of Medicine, Osaka University

Natsuki Nakagawa, Department of respiratory medicine, Kanto Central Hospital of the Mutual Aid of Association of Public School Teachers

Nobumasa Aoki, Department of Respiratory medicine and Infectious diseases, Niigata University Medical School

Norihiro Nishioka, Department of Preventive Services, Kyoto University Graduate School of Medicine

Norihisa Miyashita, Division of Pediatric Critical Care Medicine, Hyogo Prefectural Kobe Children's Hospital

Nozomu Seki, Department of Emergency, Toyama University Hospital

Ryo Ikebe, Department of Nursing, Osaka Women’s and Children’s Hospital

Ryosuke Imai, Department of Pulmonary Medicine, St. Luke’s International Hospital

Ryota Tate, Department of Critical Care Medicine, Tokyo Bay Urayasu-Ichikawa medical center

Ryuhei Sato, Department of Critical Care Nursing, Graduate School of Medicine Kyoto University

Sachiko Miyakawa, Emergency Life-Saving Technique Academy of Kyushu, Professor

Satoshi Kazuma, Department of Intensive Care Medicine, Sapporo Medical University School of Medicine

Satoshi Nakano, Department of emergency medicine, Saitama children’s medical center

Satoshi Tetsumoto, Department of Respiratory Medicine and Clinical Immunology, Suita Municipal Hospital

Satoshi Yoshimura, Department of Preventive Services, Kyoto University Graduate School of Medicine

Shigenori Yoshitake, Department of Health Science, Kyusyu University of Health and Welfare

Shin-etsu Hoshi, Iwate Prefectural Kamaishi Public Health Center

Shingo Ohki, Department of Emergency and Critical Care Medicine, Graduate School of Biomedical and Health Sciences, Hiroshima University

Shintaro Sato, Department of Respirology, Saitama Red Cross Hospital

Shodai Yoshihiro, Pharmaceutical department, JA Hiroshima General Hospital

Shoichi Ihara, Osaka Police Hospiral, Respiratory medicine

Shota Yamamoto, Department of Radiology, Tokai University Hachioji Hospital, Tokyo, Japan

Shunichi Koide, Department of Emergency and Critical Care Medicine, Urasoe General Hospital

Shunsuke Kimata, Department of Preventive Medicine,Kyoto University Graduate School of Public Health

Shunsuke Saito, Department of emergency, Okinawa Prefectural Chubu Hospital

Shunsuke Yasuo, Department of emergency medicine, Kyoto Katsura Hospital

Shusuke Sekine, Department of Anesthesiology, Tokyo Medical University

Soichiro Mimuro, Department of Anesthesiology and Intensive Care Hamamatsu University School of Medicine

Soichiro Wada, Department of pediatrics, Teine Keijinkai Hospital

Sosuke Sugimura, Department of Medical Engineering, Faculty of Health Care Science, Himeji Dokkyo University

Tadashi Ishihara, Department of Emergency and Critical Care Medicine, Juntendo University, Urayasu Hospital

Tadashi Kaneko, Emergency and Critical Care Center, Mie University Hospital

Tadashi Nagato, Department of Respiratory Medicine,JCHO Tokyo Yamate Medical Center

Takaaki Maruhashi, Department　of　Emergency　and　Critical　care　Medicine,　Kitasato　University school of Medicine

Takahiro Tamura, Department of Anesthesiology, Nagoya University Graduate School of Medicine

Takanori Ohno, Department of Emergency ,SHOWA University Fujigaoka hospital

Takashi Ichiyama, Intensive Care Unit, Shinshu University Hospital

Takashi Niwa, Department of Respiratory Medicine, Kanagawa Cardiovascular and Respiratory Center

Takashi Ueji, National Hospital Organization Osaka National Hospital

Takayuki Ogura, Department of Emergency Medicine and Critical Care Medicine, Tochigi Prefectural Emergency and Critical Care Center, Imperial Gift Foundation SAISEIKAI, Utsunomiya Hospital

Takeshi Kawasaki, Department of Respirology, Graduate School of Medicine, Chiba University

Takeshi Tanaka, Infection Control and Education Center, Nagasaki University Hospital

Takeshi Umegaki, Department of Anesthesiology, Kansai Medical University

Taku Furukawa, Department of Anesthesiology and Critical Care, Jichi Medical University Saitama Medical Center

Taku Omura, Department of Emergency and Critical Care Medicine, Chiba University Graduate School of Medicine

Takumi Nagao, Department of Sakakibara Heart Institute

Takuya Mayumi, Department of Cardiovascular Medicine, Graduate School of Medical Science, Kanazawa University

Takuya Taniguchi, Department of Cardiovascular Medicine, Otsu City Hospital

Takuya Yoshida, Department of Emergency and Critical Care Medicine, Komaki City Hospital

Tatsutoshi Shimatani, Chugoku Rosai Hospital

Teppei Murata , Department of Cardiology Tokyo Metropolitan Geriatric Hospital and Institute of Gerontology

Tetsuya Sato, Department of Emergency and Critical Care Medicine, Tohoku University Hospital

Tohru Sawamoto, Department of Emergency and Critical Care Medicine, Tokai University School of Medicine

Yoshifumi Koukei, Tokyo Metropolitan Tama Medical Center Department of Emergency Medicine Trauma and Resuscitation Center

Tomohiro Takehara, Division of Pulmonary Medicine, Department of Medicine, Keio University School of Medicine

Tomomi Ueda, Department of Anesthesiology, Saiseikai Yokohamashi Tobu Hospital

Tomoya Katsuta, Department of respiratory medicine, Ehime Prefecture Central Hospital

Tomoya Nishino, Department of Emergency and Critical Care, Tokai University School of Medicine

Toshiki Yokoyama, Department of Respiratory Medicine and Allergy and Department of Emergency and Intensive Care Medicine, Intensive Care Unit / Tosei General Hospital

Ushio Higashijima, Department of Anesthesiology and Intensive Care Medicine, Nagasaki University Graduate school of Biomedical Sciences

Wataru Iwanaga, Department of emergency and critical care medicine, Urasoe general hospital

Yasushi Inoue, International University of Health and Welfare, Mita Hospital, Respiratory Diseases Center

Yoshiaki Iwashita, Emergency and Critical Care Medicine, Shimane University

Yoshie Yamada, Department of Healthcare Epidemiology, School of Public Health in the Graduate School of Medicine, Kyoto University

Yoshifumi Kubota, Kameda medical center department of infectious diseases

Yoshihiro Suido, Department of Respiratory Medicine, Asao General Hospital

Yoshihiro Tomioka, Department of Anesthesiology, Todachuo General Hospital

Yoshihisa Fujimoto, TMG Asaka Medical Center

Yoshihito Fujita, Department of Anesthesiology and Intensive Care Medicine, Aichi Medical University

Yoshikazu Yamaguchi, Department of Anesthesiology, Yokohama Municipal Citizen's Hospital

Yoshimi Nakamura, Department of Emergency and Critical Care Medicine, Japanese Red Cross Kyoto Daini Hospital

Yoshinobu Abe, Division of Emergency and Disaster Medicine Tohoku Medical and Pharmaceutical University

Yoshitomo Eguchi, Saiseikai Kumamoto Hospital

Yoshiyasu Oshima, Bachelor of Pharmacy/Department of Pharmacy, Kobe Tokushukai Hospital

Yosuke Fukuda, Department of Medicine, Division of Respiratory Medicine and Allergology, Showa University School of Medicine

Yudai Iwasaki, Department of Anesthesiology and Perioperative Medicine, Tohoku University Graduate School of Medicine

Yuichi Yasufuku, Department of Social Preventive Medical Sciences, Center for Preventive Medical Sciences, Chiba University

Yuji Shono, Emergency and Critical Care Center, Kyushu University Hospital

Yuka Nakatani , Department of Internal Medicine, Showa Inan General Hospital

Yuki Nakamori, Department of Molecular Pathobiology and Cell Adhesion Biology, Mie University Graduate School of Medicine

Yukie Ito, Department of Intensive Care Medicine, Osaka Women's and Children's Hospital

Yuko Tanabe, Department of Clinical Oncology, Toranomon hospital

Yusuke Nagamine, Department of Anesthesiology and Critical Care Medicine, Yokohama City University Hospital

Yuta Nakamura, Emergency and Critical Care Centre, Saiseikai Kumamoto Hospital

Yutaro Kurihara, Department of Emergency and Critical Care Medicine, Kitasato University School of Medicine

■External evaluation committee

Erika Ota, Global Health Nursing, Graduate School of Nursing Science, St. Luke's international university

Hidemichi Yuasa, Department of Oral and Maxillofacial Surgery, National Hospital Organization Toyohashi Medical Center

Kazuma Yamakawa, Department of Emergency Medicine, Osaka Medical and Pharmaceutical University

Toshio Fukuoka, Department of General Medicine/ Kurashiki Central Hospital

■Plane language summary for patients and patient’s family

Yohei Okada (Manager), Department of Primary care and Emergency medicine, Graduate school of medicine, Kyoto University

Hiroshi Sugimoto (Leader), Department of Respiratory Medicine, Konan Medical Center

Aiko Masunaga, Department of Respiratory Medicine, Kumamoto University Hospital

Chihiro Takayama, Takatsuki　General Hospital

Sosuke Sugimura, Department of Medical Engineering, Faculty of Health Care Science, Himeji Dokkyo University

Yoshiyasu Oshima, Bachelor of Pharmacy/Department of Pharmacy, Kobe Tokushukai Hospital

Kohei Yamada, Department of Traumatology and Critical Care Medicine, National Defense Medical College

Ken-ichi Kano, Department of Emergency medicine, Fukui Prefectural Hospital

Masaaki Sakuraya, Department of Emergency and Intensive Care Medicine, JA Hiroshima General Hospital

Takumi Nagao, Department of Sakakibara Heart Institute

Eriko Takezawa, Patient’s family representative

Kaoru Shintani, Illsutrator

■Visual abstract

Yohei Okada, Department of Primary care and Emergency medicine, Graduate school of medicine, Kyoto University

Tadashi Ishihara, Department of Emergency and Critical Care Medicine, Juntendo University, Urayasu Hospital

Eriko Takezawa, Patient’s family representative

**The policy for disclosure of conflicts of interest (COI) in this clinical practice guideline**

Financial conflicts of interest (COI) and intellectual COI are disclosed in this appendix file.

COI type

This clinical practice guideline classifies COI into financial COI and intellectual (academic) COI and classifies them into Primary COI and Secondary COI based on their importance. Members with primary COI are not allowed to participate in discussions or voting on the recommendations, and secondary COI is only for disclosure (no restriction). Further, the following criteria for financial COI and intellectual COI are applied. Financial interest is payments received in exchange for services or any financial value derived from holdings. Intellectual interest is attachment to a certain academic ideas or activities that create the potential for cognitive biases, or attachment to a specific viewpoint.

| Category | Financial COI | Intellectual COI | Restriction |
| --- | --- | --- | --- |
| Primary COI | Active relationship within 3 years met the following criteria | Authorship of original studies, directly bearing on a recommendation  Peer-reviewed grant funding, directly bearing on a recommendation. | Restriction from all discussion and voting about recommendation |
| Secondary COI | Inactive relationship that meets the following criteria but have already expired (e.g., contracts have been terminated) | Other than the above | No restrictions |

Financial COI

The following items are to be disclosed in accordance with criteria A, B and C below. Disclose retrospectively for the past three years from January 2018 to December 2021. Peer-reviewed grant funding from pharmaceutical manufacturers, etc. are also subject to COI. In cases where the person is in a position such as chief professor or department director, if the fund is obtained as for the entire department, they should be disclosed as COI (*see item C below).

1. Declarant's own declaration

Regarding the COI declaration of an individual researcher, disclosure must be made for the following items A-1 to A-9.

A-1

When the compensation for a leadership position and/or an advisory role in one private company, corporate body, or for-profit organization (hereinafter referred to as “private company, corporate body, or organization”) involved in medical research is one million yen or more per year.

A-2．

When the profit from stocks (total dividend and gain-on-sale) in one private company is one million yen or more per year, or when the ratio of stocks held is 5% or more of all shares in one private company.

A-3

When patent royalty and/or licensing fees for one patent and/or license from one private company, corporate body, or organization are one million yen or more per year.

A-4

When the daily allowance or honoraria (e.g. lecture fees) paid as compensation for the hours detained or labor for attending (or presenting at or providing advice at) meetings by one private company, corporate body, or organization is five hundred thousand yen or more per year.

A-5

When the total manuscript fee for writing brochures, articles on discussions, or other publications paid by one private company, corporate body, or organization is five hundred thousand yen or more per year.

A-6

When the total contract-based research funding (including funding for collaborative research, funded research, and drug trials) for medical research that the disclosing party may substantially make a decision on how to use paid by one private company, corporate body, or organization is one million yen or more per year.

A-7

When the total of contributions for scholarships provided by one private company, corporate body, or organization to a disclosing party, his/her course/class, or laboratory that the disclosing party may substantially make a decision on how to use is one million yen or more per year.

A-8

When a presenter belongs to a study group sponsored by a private company, corporate body, or organization; provided, however, that when the total contributions that the disclosing party may substantially make a decision on how to use is one million yen or more per year.

A-9

When the total amount paid by one private company, corporate body, or organization for travel, gifts or anything directly unrelated to research is fifty thousand yen or more per year.

【B. Spouses, first-degree relatives, and anyone who shares the income and assets of the people】

If spouses, first-degree relatives, and anyone who shares the income and assets of the people of a member met the following criteria, it should be indicated.

B-1

When the compensation for a leadership position and/or an advisory role in one private company, corporate body, or for-profit organization (hereinafter referred to as “private company, corporate body, or organization”) involved in medical research is one million yen or more per year.

B-2．

When the profit from stocks (total dividend and gain-on-sale) in one private company is one million yen or more per year, or when the ratio of stocks held is 5% or more of all shares in one private company.

B-3

When patent royalty and/or licensing fees for one patent and/or license from one private company, corporate body, or organization are one million yen or more per year.

【C. Institutional COI】

When the disclosing party is currently or was in the past in a relationship of joint researcher or co-researcher with the chief executive of the research institute to which the disclosing party belongs or the chief of the department of such institute (university, hospital, faculty, or center), the COI must be disclosed in accordance with the designated form (Form 3-C) for the following matters. Regarding the amounts that require COI disclosure, criteria have been set for each item requiring disclosure as follows:

C-1

When the total contract based research funding (including funding for collaborative research, funded research, and drug trials) for medical research that the disclosing party may substantially make a decision on how to use paid by one private company, corporate body, or organization is ten million yen or more per year.

C-2

When the total contributions provided by one private company, corporate body, or organization to a disclosing party, the affiliate institute or department to which the disclosing party belongs or chief of such institute or department for which the disclosing party may substantially make a decision on how to use is two million yen or more per year

C-3

When there are any shares owned by the research institute or department to which the disclosing party belongs or the head thereof (in a joint researcher or co-researcher relationship within the past three years), patent royalties, or investment in a venture company, include such as institutional COI.

**Intellectual COI**

The following items are to be disclosed in accordance with criteria A, B and C below. The items in C-1 are treated as Primary COI. For items C-3 and C-4, if the research is based on acquired research funds, both the source of research funds and the name of the research should be disclosed and treated as a Primary COI.

A. Positions in academic organizations

If a member has a position of board member in a national academic organizations or equivalents including societies in all areas within 3 years (between January 1, 2018 and December 2021), the position and society name should be listed.

B. Involvement in the clinical guidelines

B-1

If a member has been involved in any medical guideline or its equivalent between January, 2018, and December 2021, the name and position of the guideline should be indicated.

B-2

The member of a panel committee in the previous edition of the ARDS Clinical Practice Guidelines 2016, it should be indicated.

C. Involvement in related research

C-1

If a member is an author (co-author) of a paper that is included as primary original study in a systematic review for developing recommendation, the name of the paper and journal should be indicated. (It includes the period before the last three years.)

C-2

If a member is an author (or coauthor) of systematic review for developing recommendation, the name of the paper and the journal should be disclosed. (It includes the period before the last three years.)

C-3

If a member is an author of the study or speaker (including co-author or co-speaker) in a conference presentation related to clinical question in this guideline published or held between January 2018 and December 2021, the name of the article, journal or conference should be indicated.

C-4

If a member is involved in the research related to the clinical questions in this guideline performed between January 2018 and December 2021, the name of the research should be disclosed.

COI List (Financial COI）

| Name | A-1 | A-2 | A-3 | A-4 | A-5 | A-6 | A-7 | A-8 | A-9 | B-1 | B-2 | B-3 | C-1 | C-2 | C-3 |
| --- | --- | --- | --- | --- | --- | --- | --- | --- | --- | --- | --- | --- | --- | --- | --- |
| Masamitsu Sanui | NA | NA | NA | NA | NA | NA | NA | NA | NA | NA | NA | NA | NA | NA | NA |
| Sadatomo Tasaka | NA | NA | NA | NA | NA | NA | NA | NA | NA | NA | NA | NA | NA | NA | NA |
| Shinichiro Ohshimo | NA | NA | NA | NA | NA | NA | NA | NA | NA | NA | NA | NA | NA | NA | NA |
| Muneyuki Takeuchi | NA | NA | NA | NA | NA | NA | NA | NA | NA | NA | NA | NA | NA | NA | NA |
| Hideto Yasuda | NA | NA | NA | NA | NA | NA | NA | NA | NA | NA | NA | NA | NA | NA | NA |
| Kazuya Ichikado | NA | NA | NA | *1 | NA | NA | NA | NA | NA | NA | NA | NA | NA | NA | NA |
| Kenji Tsushima | NA | NA | NA | *2 | NA | NA | NA | NA | NA | NA | NA | NA | NA | NA | NA |
| Moritoki Egi | NA | NA | NA | NA | NA | NA | NA | NA | NA | NA | NA | NA | NA | NA | NA |
| Nobuaki Shime | NA | NA | NA | *3 | NA | NA | *4 | NA | NA | NA | NA | NA | NA | NA | NA |
| Satoru Hashimoto | NA | NA | NA | NA | NA | NA | NA | NA | NA | NA | NA | NA | NA | NA | NA |
| Osamu Saito | NA | NA | NA | NA | NA | NA | NA | NA | NA | NA | NA | NA | NA | NA | NA |
| Shotaro Matsumoto | NA | NA | NA | NA | NA | NA | NA | NA | NA | NA | NA | NA | NA | NA | NA |
| Eishu Nango | NA | NA | NA | NA | NA | NA | NA | NA | NA | NA | NA | NA | NA | NA | NA |
| Kenichiro Hayashi | NA | NA | NA | NA | NA | NA | NA | NA | NA | NA | NA | NA | NA | NA | NA |
| Masaaki Sakuraya | NA | NA | NA | NA | NA | NA | NA | NA | NA | NA | NA | NA | NA | NA | NA |
| Mikio Nakajima | NA | NA | NA | NA | NA | NA | NA | NA | NA | NA | NA | NA | NA | NA | NA |
| Satoshi Okamori | NA | NA | NA | NA | NA | NA | NA | NA | NA | NA | NA | NA | NA | NA | NA |
| Shinya Miura | NA | NA | NA | NA | NA | NA | NA | NA | NA | NA | NA | NA | NA | NA | NA |
| Tadashi Ishihara | NA | NA | NA | NA | NA | NA | NA | NA | NA | NA | NA | NA | NA | NA | NA |
| Tatsuma Fukuda | NA | NA | NA | NA | NA | NA | NA | NA | NA | NA | NA | NA | NA | NA | NA |
| Tetsuro Kamo | NA | NA | NA | NA | NA | NA | NA | NA | NA | NA | NA | NA | NA | NA | NA |
| Tomoaki Yatabe | NA | NA | NA | NA | NA | NA | NA | NA | NA | NA | NA | NA | NA | NA | NA |
| Yasuhiro Norisue | NA | NA | NA | NA | NA | NA | NA | NA | NA | NA | NA | NA | NA | NA | NA |
| Yoshitaka Aoki | NA | NA | NA | NA | NA | NA | NA | NA | NA | NA | NA | NA | NA | NA | NA |
| Yusuke Iizuka | NA | NA | NA | NA | NA | NA | NA | NA | NA | NA | NA | NA | NA | NA | NA |
| Yutaka Kondo | NA | NA | NA | NA | NA | NA | NA | NA | NA | NA | NA | NA | NA | NA | NA |
| Chihiro Narita | NA | NA | NA | NA | NA | NA | NA | NA | NA | NA | NA | NA | NA | NA | NA |
| Hiroshi Yonekura | NA | NA | NA | NA | NA | NA | NA | NA | NA | NA | NA | NA | NA | NA | NA |
| Jun Kataoka | NA | NA | NA | NA | NA | NA | NA | NA | NA | NA | NA | NA | NA | NA | NA |
| Koichi Ando | NA | NA | NA | NA | NA | NA | NA | NA | NA | NA | NA | NA | NA | NA | NA |
| Masayuki Ozaki | NA | NA | NA | NA | NA | NA | NA | NA | NA | NA | NA | NA | NA | NA | NA |
| Shunsuke Taito | NA | NA | NA | NA | NA | NA | NA | NA | NA | NA | NA | NA | NA | NA | NA |
| Takuo Yoshida | NA | NA | NA | NA | NA | NA | NA | NA | NA | NA | NA | NA | NA | NA | NA |
| Tetsuro Nishimura | NA | NA | NA | NA | NA | NA | NA | NA | NA | NA | NA | NA | NA | NA | NA |
| Tomoyuki Masuyama | NA | NA | NA | NA | NA | NA | NA | NA | NA | NA | NA | NA | NA | NA | NA |
| Yohei Okada | NA | NA | NA | NA | NA | NA | NA | NA | NA | NA | NA | NA | NA | NA | NA |
| Aiko Masunaga | NA | NA | NA | NA | NA | NA | NA | NA | NA | NA | NA | NA | NA | NA | NA |
| Aiko Tanaka | NA | NA | NA | NA | NA | NA | NA | NA | NA | NA | NA | NA | NA | NA | NA |
| Akihiko Inoue | NA | NA | NA | NA | NA | NA | NA | NA | NA | NA | NA | NA | NA | NA | NA |
| Akiko Higashi | NA | NA | NA | NA | NA | NA | NA | NA | NA | NA | NA | NA | NA | NA | NA |
| Atsushi Tanikawa | NA | NA | NA | NA | NA | NA | NA | NA | NA | NA | NA | NA | NA | NA | NA |
| Atsushi Ujiro | NA | NA | NA | NA | NA | NA | NA | NA | NA | NA | NA | NA | NA | NA | NA |
| Chihiro Takayama | NA | NA | NA | NA | NA | NA | NA | NA | NA | NA | NA | NA | NA | NA | NA |
| Daisuke Kasugai | NA | NA | NA | NA | NA | NA | NA | NA | NA | NA | NA | NA | NA | NA | NA |
| Daisuke Kawakami | NA | NA | NA | NA | NA | NA | NA | NA | NA | NA | NA | NA | NA | NA | NA |
| Daisuke Ueno | NA | NA | NA | NA | NA | NA | NA | NA | NA | NA | NA | NA | NA | NA | NA |
| Daizoh Satoh | NA | NA | NA | NA | NA | NA | NA | NA | NA | NA | NA | NA | NA | NA | NA |
| Dongli | NA | NA | NA | NA | NA | NA | NA | NA | NA | NA | NA | NA | NA | NA | NA |
| Emiko Nakataki | NA | NA | NA | NA | NA | NA | NA | NA | NA | NA | NA | NA | NA | NA | NA |
| Erina Tabata | NA | NA | NA | NA | NA | NA | NA | NA | NA | NA | NA | NA | NA | NA | NA |
| Futoshi Kotajima | NA | NA | NA | NA | NA | NA | NA | NA | NA | NA | NA | NA | NA | NA | NA |
| Go Ishimaru | NA | NA | NA | NA | NA | NA | NA | NA | NA | NA | NA | NA | NA | NA | NA |
| Haruhiko Hoshino | NA | NA | NA | NA | NA | NA | NA | NA | NA | NA | NA | NA | NA | NA | NA |
| Hideki Yoshida | NA | NA | NA | NA | NA | NA | NA | NA | *5 | NA | NA | NA | NA | NA | NA |
| Hidetaka Iwai | NA | NA | NA | NA | NA | NA | NA | NA | NA | NA | NA | NA | NA | NA | NA |
| Hiroaki Nakagawa | NA | NA | NA | NA | NA | NA | NA | NA | NA | NA | NA | NA | NA | NA | NA |
| Hiroko Sugimura | NA | NA | NA | NA | NA | NA | NA | NA | NA | NA | NA | NA | NA | NA | NA |
| Hiromichi Narumiya | NA | NA | NA | NA | NA | NA | NA | NA | NA | NA | NA | NA | NA | NA | NA |
| Hiromu Okano | NA | NA | NA | NA | NA | NA | NA | NA | NA | NA | NA | NA | NA | NA | NA |
| Hiroshi Nakamura | NA | NA | NA | NA | NA | NA | NA | NA | NA | NA | NA | NA | NA | NA | NA |
| Hiroshi Sugimoto | NA | NA | NA | NA | NA | NA | NA | NA | NA | NA | NA | NA | NA | NA | NA |
| Hiroyuki Hashimoto | NA | NA | NA | NA | NA | NA | NA | NA | NA | NA | NA | NA | NA | NA | NA |
| Hiroyuki Ito | NA | NA | NA | NA | NA | NA | NA | NA | NA | NA | NA | NA | NA | NA | NA |
| Hisashi Dote | NA | NA | NA | NA | NA | NA | NA | NA | NA | NA | NA | NA | NA | NA | NA |
| Hisashi Imahase | NA | NA | NA | NA | NA | NA | NA | NA | NA | NA | NA | NA | NA | NA | NA |
| Hitoshi Sakamoto | NA | NA | NA | NA | NA | NA | NA | NA | NA | NA | NA | NA | NA | NA | NA |
| Hitoshi Sato | NA | NA | NA | NA | NA | NA | NA | NA | NA | NA | NA | NA | NA | NA | NA |
| Ichiro Osawa | NA | NA | NA | NA | NA | NA | NA | NA | NA | NA | NA | NA | NA | NA | NA |
| Jun Hamaguchi | NA | NA | NA | NA | NA | NA | NA | NA | NA | NA | NA | NA | NA | NA | NA |
| Jun Kamei | NA | NA | NA | NA | NA | NA | NA | NA | NA | NA | NA | NA | NA | NA | NA |
| Jun Maki | NA | NA | NA | NA | NA | NA | NA | NA | NA | NA | NA | NA | NA | NA | NA |
| Jun Sugihara | NA | NA | NA | NA | NA | NA | NA | NA | NA | NA | NA | NA | NA | NA | NA |
| Jun Takeshita | NA | NA | NA | NA | NA | NA | NA | NA | NA | NA | NA | NA | NA | NA | NA |
| Junichi Fujimoto | NA | NA | NA | NA | NA | NA | NA | NA | NA | NA | NA | NA | NA | NA | NA |
| Junichi Ishikawa | NA | NA | NA | NA | NA | NA | NA | NA | NA | NA | NA | NA | NA | NA | NA |
| Junko Kosaka | NA | NA | NA | NA | NA | NA | NA | NA | NA | NA | NA | NA | NA | NA | NA |
| Junpei Shibata | NA | NA | NA | NA | NA | NA | NA | NA | NA | NA | NA | NA | NA | NA | NA |
| Katsuhiko Hashimoto | NA | NA | NA | NA | NA | NA | NA | NA | NA | NA | NA | NA | NA | NA | NA |
| Kazuki Kikuyama | NA | NA | NA | NA | NA | NA | NA | NA | NA | NA | NA | NA | NA | NA | NA |
| Kazushige Shimizu | NA | NA | NA | NA | NA | NA | NA | NA | NA | NA | NA | NA | NA | NA | NA |
| Kazuya Okada | NA | NA | NA | NA | NA | NA | NA | NA | NA | NA | NA | NA | NA | NA | NA |
| Keishi Kawano | NA | NA | NA | NA | NA | NA | NA | NA | NA | NA | NA | NA | NA | NA | NA |
| Keisuke Anan | NA | NA | NA | NA | NA | NA | NA | NA | NA | NA | NA | NA | NA | NA | NA |
| Keisuke Ota | NA | NA | NA | NA | NA | NA | NA | NA | NA | NA | NA | NA | NA | NA | NA |
| Ken-ichi Kano | NA | NA | NA | NA | NA | NA | NA | NA | NA | NA | NA | NA | NA | NA | NA |
| Kengo Asano | NA | NA | NA | NA | NA | NA | NA | NA | NA | NA | NA | NA | NA | NA | NA |
| Kenichi Hondo | NA | NA | NA | NA | NA | NA | NA | NA | NA | NA | NA | NA | NA | NA | NA |
| Kenji Ishii | NA | NA | NA | NA | NA | NA | NA | NA | NA | NA | NA | NA | NA | NA | NA |
| Kensuke Fujita | NA | NA | NA | NA | NA | NA | NA | NA | NA | NA | NA | NA | NA | NA | NA |
| Kenta Ogawa | NA | NA | NA | NA | NA | NA | NA | NA | NA | NA | NA | NA | NA | NA | NA |
| Kentaro Ito | NA | NA | NA | *6 | NA | NA | NA | NA | NA | NA | NA | NA | NA | NA | NA |
| Kentaro Tokunaga | NA | NA | NA | NA | NA | NA | NA | NA | NA | NA | NA | NA | NA | NA | NA |
| Kenzo Ishii | NA | NA | NA | NA | NA | NA | NA | NA | NA | NA | NA | NA | NA | NA | NA |
| Kohei Kusumoto | NA | NA | NA | NA | NA | NA | NA | NA | NA | NA | NA | NA | NA | NA | NA |
| Kohei Ohta | NA | NA | NA | NA | NA | NA | NA | NA | NA | NA | NA | NA | NA | NA | NA |
| Kohei Takimoto | NA | NA | NA | NA | NA | NA | NA | NA | NA | NA | NA | NA | NA | NA | NA |
| Kohei Yamada | NA | NA | NA | NA | NA | NA | NA | NA | NA | NA | NA | NA | NA | NA | NA |
| Koichi Naito | NA | NA | NA | NA | NA | NA | NA | NA | NA | NA | NA | NA | NA | NA | NA |
| Koichi Yamashita | NA | NA | NA | NA | NA | NA | NA | NA | NA | NA | NA | NA | NA | NA | NA |
| Koichi Yoshinaga | NA | NA | NA | NA | NA | NA | NA | NA | NA | NA | NA | NA | NA | NA | NA |
| Kota Yamauchi | NA | NA | NA | NA | NA | NA | NA | NA | NA | NA | NA | NA | NA | NA | NA |
| Maki Murata | NA | NA | NA | NA | NA | NA | NA | NA | NA | NA | NA | NA | NA | NA | NA |
| Makiko Konda | NA | NA | NA | NA | NA | NA | NA | NA | NA | NA | NA | NA | NA | NA | NA |
| Manabu Hamamoto | NA | NA | NA | NA | NA | NA | NA | NA | NA | NA | NA | NA | NA | NA | NA |
| Masaharu Aga | NA | NA | NA | NA | NA | NA | NA | NA | NA | NA | NA | NA | NA | NA | NA |
| Masahiro Kashiura | NA | NA | NA | NA | NA | NA | NA | NA | NA | NA | NA | NA | NA | NA | NA |
| Masahiro Katsurada | NA | NA | NA | NA | NA | NA | NA | NA | NA | NA | NA | NA | NA | NA | NA |
| Masami Ishikawa | NA | NA | NA | NA | NA | NA | NA | NA | NA | NA | NA | NA | NA | NA | NA |
| Michihiko Kono | NA | NA | NA | NA | NA | NA | NA | NA | NA | NA | NA | NA | NA | NA | NA |
| Michihito Kyo | NA | NA | NA | NA | NA | NA | NA | NA | NA | NA | NA | NA | NA | NA | NA |
| Minoru Hayashi | NA | NA | NA | NA | NA | NA | NA | NA | NA | NA | NA | NA | NA | NA | NA |
| Mitsuhiro Abe | NA | NA | NA | NA | NA | NA | NA | NA | NA | NA | NA | NA | NA | NA | NA |
| Mitsunori Sato | NA | NA | NA | NA | NA | NA | NA | NA | NA | NA | NA | NA | NA | NA | NA |
| Mizu Sakai | NA | NA | NA | NA | NA | NA | NA | NA | NA | NA | NA | NA | NA | NA | NA |
| Motoshi Kainuma | NA | NA | NA | NA | NA | NA | NA | NA | NA | NA | NA | NA | NA | NA | NA |
| Naohisa Masunaga | NA | NA | NA | NA | NA | NA | NA | NA | NA | NA | NA | NA | NA | NA | NA |
| Naoki Tominaga | NA | NA | NA | NA | NA | NA | NA | NA | NA | NA | NA | NA | NA | NA | NA |
| Naoya Iguchi | NA | NA | NA | NA | NA | *7 | NA | NA | NA | NA | NA | NA | NA | NA | NA |
| Natsuki Nakagawa | NA | NA | NA | NA | NA | NA | NA | NA | NA | NA | NA | NA | NA | NA | NA |
| Nobumasa Aoki | NA | NA | NA | NA | NA | NA | NA | NA | NA | NA | NA | NA | NA | NA | NA |
| Norihiro Nishioka | NA | NA | NA | NA | NA | NA | NA | NA | NA | NA | NA | NA | NA | NA | NA |
| Norihisa Miyashita | NA | NA | NA | NA | NA | NA | NA | NA | NA | NA | NA | NA | NA | NA | NA |
| Nozomu Seki | NA | NA | NA | NA | NA | NA | NA | NA | NA | NA | NA | NA | NA | NA | NA |
| Ryo Fujii | NA | NA | NA | NA | NA | NA | NA | NA | NA | NA | NA | NA | NA | NA | NA |
| Ryo Ikebe | NA | NA | NA | NA | NA | NA | NA | NA | NA | NA | NA | NA | NA | NA | NA |
| Ryo Uchimido | NA | NA | NA | NA | NA | NA | NA | NA | NA | NA | NA | NA | NA | NA | NA |
| Ryohei Yamamoto | NA | NA | NA | NA | NA | NA | NA | NA | NA | NA | NA | NA | NA | NA | NA |
| Ryosuke Imai | NA | NA | NA | NA | NA | NA | NA | NA | NA | NA | NA | NA | NA | NA | NA |
| Ryota Tate | NA | NA | NA | NA | NA | NA | NA | NA | NA | NA | NA | NA | NA | NA | NA |
| Ryuhei Sato | NA | NA | NA | NA | NA | NA | NA | NA | NA | NA | NA | NA | NA | NA | NA |
| Sachiko Miyakawa | NA | NA | NA | NA | NA | NA | NA | NA | NA | NA | NA | NA | NA | NA | NA |
| Satoru Robert Okazaki | NA | NA | NA | NA | NA | NA | NA | NA | NA | NA | NA | NA | NA | NA | NA |
| Satoshi Hokari | NA | NA | NA | NA | NA | NA | NA | NA | NA | NA | NA | NA | NA | NA | NA |
| Satoshi Kazuma | NA | NA | NA | NA | NA | NA | NA | NA | NA | NA | NA | NA | NA | NA | NA |
| Satoshi Nakano | NA | NA | NA | NA | NA | NA | NA | NA | NA | NA | NA | NA | NA | NA | NA |
| Satoshi Tetsumoto | NA | NA | NA | NA | NA | NA | NA | NA | NA | NA | NA | NA | NA | NA | NA |
| Satoshi Yoshimura | NA | NA | NA | NA | NA | NA | NA | NA | NA | NA | NA | NA | NA | NA | NA |
| Seisuke Okazawa | NA | NA | NA | NA | NA | NA | NA | NA | NA | NA | NA | NA | NA | NA | NA |
| Shigenori Yoshitake | NA | NA | NA | NA | NA | NA | NA | NA | NA | NA | NA | NA | NA | NA | NA |
| Shin-etsu Hoshi | NA | NA | NA | NA | NA | NA | NA | NA | NA | NA | NA | NA | NA | NA | NA |
| Shingo Ohki | NA | NA | NA | NA | NA | NA | NA | NA | NA | NA | NA | NA | NA | NA | NA |
| Shinichi Kai | NA | NA | NA | NA | NA | NA | NA | NA | NA | NA | NA | NA | NA | NA | NA |
| Shintaro Sato | NA | NA | NA | NA | NA | NA | NA | NA | NA | NA | NA | NA | NA | NA | NA |
| Shodai Yoshihiro | NA | NA | NA | NA | NA | NA | NA | NA | NA | NA | NA | NA | NA | NA | NA |
| Shoichi Ihara | NA | NA | NA | NA | NA | NA | NA | NA | NA | NA | NA | NA | NA | NA | NA |
| Shota Yamamoto | NA | NA | NA | NA | NA | NA | NA | NA | NA | NA | NA | NA | NA | NA | NA |
| Shunichi Koide | NA | NA | NA | NA | NA | NA | NA | NA | NA | NA | NA | NA | NA | NA | NA |
| Shunsuke Kimata | NA | NA | NA | NA | NA | NA | NA | NA | NA | NA | NA | NA | NA | NA | NA |
| Shunsuke Saito | NA | NA | NA | NA | NA | NA | NA | NA | NA | NA | NA | NA | NA | NA | NA |
| Shunsuke Yasuo | NA | NA | NA | NA | NA | NA | NA | NA | NA | NA | NA | NA | NA | NA | NA |
| Shusuke Sekine | NA | NA | NA | NA | NA | NA | NA | NA | NA | NA | NA | NA | NA | NA | NA |
| Soichiro Mimuro | NA | NA | NA | NA | NA | NA | NA | NA | NA | NA | NA | NA | NA | NA | NA |
| Soichiro Wadal | NA | NA | NA | NA | NA | NA | NA | NA | NA | NA | NA | NA | NA | NA | NA |
| Sosuke Sugimura | NA | NA | NA | NA | NA | NA | NA | NA | NA | NA | NA | NA | NA | NA | NA |
| Tadashi Kaneko | NA | NA | NA | NA | NA | NA | NA | NA | NA | NA | NA | NA | NA | NA | NA |
| Tadashi Nagatomo | NA | NA | NA | NA | NA | NA | NA | NA | NA | NA | NA | NA | NA | NA | NA |
| Takaaki Maruhashi | NA | NA | NA | NA | NA | NA | NA | NA | NA | NA | NA | NA | NA | NA | NA |
| Takahiro Tamura | NA | NA | NA | NA | NA | NA | NA | NA | *8 | NA | NA | NA | NA | NA | NA |
| Takanori Ohno | NA | NA | NA | NA | NA | NA | NA | NA | NA | NA | NA | NA | NA | NA | NA |
| Takashi Ichiyama | NA | NA | NA | NA | NA | NA | NA | NA | NA | NA | NA | NA | NA | NA | NA |
| Takashi Hongo | NA | NA | NA | NA | NA | NA | NA | NA | NA | NA | NA | NA | NA | NA | NA |
| Takashi Niwa | NA | NA | NA | NA | NA | NA | NA | NA | NA | NA | NA | NA | NA | NA | NA |
| Takashi Ueji | NA | NA | NA | NA | NA | NA | NA | NA | NA | NA | NA | NA | NA | NA | NA |
| Takayuki Ogura | NA | NA | NA | NA | NA | NA | NA | NA | NA | NA | NA | NA | NA | NA | NA |
| Takeshi Kawasaki | NA | NA | NA | NA | NA | NA | NA | NA | NA | NA | NA | NA | NA | NA | NA |
| Takeshi Tanaka | NA | NA | NA | NA | NA | NA | NA | NA | NA | NA | NA | NA | NA | NA | NA |
| Takeshi Umegaki | NA | NA | NA | NA | NA | NA | NA | NA | NA | NA | NA | NA | NA | NA | NA |
| Taku Furukawa | NA | NA | NA | NA | NA | NA | NA | NA | NA | NA | NA | NA | NA | NA | NA |
| Taku Omura | NA | NA | NA | NA | NA | NA | NA | NA | NA | NA | NA | NA | NA | NA | NA |
| Takumi Nagao | NA | NA | NA | NA | NA | NA | NA | NA | NA | NA | NA | NA | NA | NA | NA |
| Takuro Nakashima | NA | NA | NA | NA | NA | NA | NA | NA | NA | NA | NA | NA | NA | NA | NA |
| Takuya Mayumi | NA | NA | NA | NA | NA | NA | NA | NA | NA | NA | NA | NA | NA | NA | NA |
| Takuya Taniguchi | NA | NA | NA | NA | NA | NA | NA | NA | NA | NA | NA | NA | NA | NA | NA |
| Takuya Yoshida | NA | NA | NA | NA | NA | NA | NA | NA | NA | NA | NA | NA | NA | NA | NA |
| Tatsutoshi Shimatani | NA | NA | NA | NA | NA | NA | NA | NA | NA | NA | NA | NA | NA | NA | NA |
| Teppei Murata | NA | NA | NA | NA | NA | NA | NA | NA | NA | NA | NA | NA | NA | NA | NA |
| Tetsuro Terayama | NA | NA | NA | NA | NA | NA | NA | NA | NA | NA | NA | NA | NA | NA | NA |
| Tetsuya Sato | NA | NA | NA | NA | NA | NA | NA | NA | NA | NA | NA | NA | NA | NA | NA |
| Tohru Sawamoto | NA | NA | NA | NA | NA | NA | NA | NA | NA | NA | NA | NA | NA | NA | NA |
| Tomohiro Takehara | NA | NA | NA | NA | NA | NA | NA | NA | NA | NA | NA | NA | NA | NA | NA |
| Tomomi Ueda | NA | NA | NA | NA | NA | NA | NA | NA | NA | NA | NA | NA | NA | NA | NA |
| Tomoya Katsuta | NA | NA | NA | NA | NA | NA | NA | NA | NA | NA | NA | NA | NA | NA | NA |
| Tomoya Nishino | NA | NA | NA | NA | NA | NA | NA | NA | NA | NA | NA | NA | NA | NA | NA |
| Toshiki Yokoyama | NA | NA | NA | NA | NA | NA | NA | NA | NA | NA | NA | NA | NA | NA | NA |
| Ushio Higashijima | NA | NA | NA | NA | NA | NA | NA | NA | NA | NA | NA | NA | NA | NA | NA |
| Wataru Iwanaga | NA | NA | NA | NA | NA | NA | NA | NA | NA | NA | NA | NA | NA | NA | NA |
| Yasushi Inoue | NA | NA | NA | NA | NA | NA | NA | NA | NA | NA | NA | NA | NA | NA | NA |
| Yasushi Nakano | NA | NA | NA | NA | NA | NA | NA | NA | NA | NA | NA | NA | NA | NA | NA |
| Yoshiaki Iwashita | NA | NA | NA | NA | NA | NA | NA | NA | NA | NA | NA | NA | NA | NA | NA |
| Yoshie Yamada | NA | NA | NA | NA | NA | NA | NA | NA | NA | NA | NA | NA | NA | NA | NA |
| Yoshifumi Koukei | NA | NA | NA | NA | NA | NA | NA | NA | NA | NA | NA | NA | NA | NA | NA |
| Yoshifumi Kubota | NA | NA | NA | NA | NA | NA | NA | NA | NA | NA | NA | NA | NA | NA | NA |
| Yoshihiro Hagiwara | NA | NA | NA | NA | NA | NA | NA | NA | NA | NA | NA | NA | NA | NA | NA |
| Yoshihiro Suido | NA | NA | NA | NA | NA | NA | NA | NA | NA | NA | NA | NA | NA | NA | NA |
| Yoshihiro Tomioka | NA | NA | NA | NA | NA | NA | NA | NA | NA | NA | NA | NA | NA | NA | NA |
| Yoshihisa Fujimoto | NA | NA | NA | NA | NA | NA | NA | NA | NA | NA | NA | NA | NA | NA | NA |
| Yoshihito Fujita | NA | NA | NA | NA | NA | NA | NA | NA | NA | NA | NA | NA | NA | NA | NA |
| Yoshikazu Yamaguchi | NA | NA | NA | NA | NA | NA | NA | NA | NA | NA | NA | NA | NA | NA | NA |
| Yoshimi Nakamura | NA | NA | NA | NA | NA | NA | NA | NA | NA | NA | NA | NA | NA | NA | NA |
| Yoshinobu Abe | NA | NA | NA | NA | NA | NA | NA | NA | NA | NA | NA | NA | NA | NA | NA |
| Yoshitomo Eguchi | NA | NA | NA | NA | NA | NA | NA | NA | NA | NA | NA | NA | NA | NA | NA |
| Yoshiyasu Oshimal | NA | NA | NA | NA | NA | NA | NA | NA | NA | NA | NA | NA | NA | NA | NA |
| Yosuke Fukuda | NA | NA | NA | NA | NA | NA | NA | NA | NA | NA | NA | NA | NA | NA | NA |
| Yudai Iwasaki | NA | NA | NA | NA | NA | NA | NA | NA | NA | NA | NA | NA | NA | NA | NA |
| Yuichi Yasufuku | NA | NA | NA | NA | NA | NA | NA | NA | NA | NA | NA | NA | NA | NA | NA |
| Yuji Shono | NA | NA | NA | NA | NA | NA | NA | NA | NA | NA | NA | NA | NA | NA | NA |
| Yuka Nakatani | NA | NA | NA | NA | NA | NA | NA | NA | NA | NA | NA | NA | NA | NA | NA |
| Yuki Kishihara | NA | NA | NA | NA | NA | NA | NA | NA | NA | NA | NA | NA | NA | NA | NA |
| Yuki Nakamori | NA | NA | NA | NA | NA | NA | NA | NA | NA | NA | NA | NA | NA | NA | NA |
| Yukie Ito | NA | NA | NA | NA | NA | NA | NA | NA | NA | NA | NA | NA | NA | NA | NA |
| Yuko Tanabe | NA | NA | NA | NA | NA | *9 | NA | NA | NA | NA | NA | NA | NA | NA | NA |
| Yusuke Nagamine | NA | NA | NA | NA | NA | NA | NA | NA | NA | NA | NA | NA | NA | NA | NA |
| Yuta Nakamura | NA | NA | NA | NA | NA | NA | NA | NA | NA | NA | NA | NA | NA | NA | NA |
| Yutaro Kurihara | NA | NA | NA | NA | NA | NA | NA | NA | NA | NA | NA | NA | NA | NA | NA |
| Eriko Takezawa | NA | NA | NA | NA | NA | NA | NA | NA | NA | NA | NA | NA | NA | NA | NA |
| Hiroshi Okuda | NA | NA | NA | NA | NA | NA | NA | NA | NA | NA | NA | NA | NA | NA | NA |
| Hiroshi Yoshikawa | NA | NA | NA | NA | NA | NA | NA | NA | NA | NA | NA | NA | NA | NA | NA |
| Hitoshi Yokoyama | NA | NA | NA | NA | NA | NA | NA | NA | NA | NA | NA | NA | NA | NA | NA |
| Keiko Ishimura | NA | NA | NA | NA | NA | NA | NA | NA | NA | NA | NA | NA | NA | NA | NA |
| Kokichi Andoh | NA | NA | NA | NA | NA | NA | NA | NA | NA | NA | NA | NA | NA | NA | NA |
| Makoto Miki | NA | NA | NA | NA | NA | NA | NA | NA | NA | NA | NA | NA | NA | NA | NA |
| Masashi Morizane | NA | NA | NA | NA | NA | NA | NA | NA | NA | NA | NA | NA | NA | NA | NA |
| Nana Arai | NA | NA | NA | NA | NA | NA | NA | NA | NA | NA | NA | NA | NA | NA | NA |
| Ryo Kozu | NA | NA | NA | *10 | NA | NA | NA | NA | NA | NA | NA | NA | NA | NA | NA |
| Ryutaro Seo | NA | NA | NA | NA | NA | NA | NA | NA | NA | NA | NA | NA | NA | NA | NA |
| Satoshi Doi | NA | NA | NA | NA | NA | NA | NA | NA | NA | NA | NA | NA | NA | NA | NA |
| Takeshi Yoshida | NA | NA | NA | NA | NA | NA | NA | NA | NA | NA | NA | NA | NA | NA | NA |
| Akimasa Yamatani | NA | NA | NA | NA | NA | NA | NA | NA | NA | NA | NA | NA | NA | NA | NA |
| Kensuke Yagi | NA | NA | NA | NA | NA | NA | NA | NA | NA | NA | NA | NA | NA | NA | NA |
| Satoshi Nakagawa | NA | NA | NA | NA | NA | NA | NA | NA | NA | NA | NA | NA | NA | NA | NA |
| Shunsuke Nosaka | NA | NA | NA | NA | NA | NA | NA | NA | NA | NA | NA | NA | NA | NA | NA |
| Tatsuya Kawasaki | NA | NA | NA | NA | NA | NA | NA | NA | NA | NA | NA | NA | NA | NA | NA |
| Yuki Enomoto | NA | NA | NA | NA | NA | NA | NA | NA | NA | NA | NA | NA | NA | NA | NA |
| Takuya Hayashi | NA | NA | NA | NA | NA | NA | NA | NA | NA | NA | NA | NA | NA | NA | NA |
| Erika Ota | NA | NA | NA | NA | NA | NA | NA | NA | NA | NA | NA | NA | NA | NA | NA |
| Hidemichi Yuasa | NA | NA | NA | NA | NA | NA | NA | NA | NA | NA | NA | NA | NA | NA | NA |
| Kazuma Yamakawa | NA | NA | NA | NA | NA | *11 | *12 | NA | NA | NA | NA | NA | NA | NA | NA |
| Toshio Fukuoka | NA | NA | NA | NA | NA | NA | NA | NA | NA | NA | NA | NA | NA | NA | NA |

NA: Not applicable

*1 Boehringer Ingelheim Japan, 2020, 1900,000 JPY

*2 Boehringer Ingelheim Japan, 2019, 2100,000 JPY

*3 Pfizer 2017, Sumitomo Dainippon Pharma 2017, MSD2018, 2019: All of them are between 500,000 and 1,000,000 yen.

*4 Pfizer, 2017, 2018 Asahi Kasei Pharm, 2017

*5 bioMérieux Japan, Lecture fee, 2020, 70,000JPY

*6 Eli Lilly, Boehringer Ingelheim Japan

*7 Baxter Healthcare Corporation, Competitive fund, 2021, 38000 USD

*8 Haemonetics Japan, Lecture fee, 2020, 50,000 JPY

*9 Taiho Pharma, Clinical trial, 2020, 1,557,600JPY、Ono Pharmaceutical, Clinical trial, 2020, 5,472,000JPY

*10 Teijin Pharma，Lecture fee，2019, 670,000JPY

*11 JIMRO, Collaborating research, 2020, 4,000,000JPY

*12 Asahi Kasei Pharma, 2020, 2,000,000JPY, Nihon Pharmaceutical, 2020, 1,000,000JPY

COI List（Intellectual COI）　(Items without names in English are listed in Japanese. )

| Name | A  Society, Role | B-1  Guideline, Role | B-2  Panel member of previous version | C-1  Title, Journal | C-2: Title, Journal, C-3: Title, Conference | C-4: Related research |
| --- | --- | --- | --- | --- | --- | --- |
| Masamitsu Sanui | 日本呼吸療法医学会、理事、2020年10月 | ARDS診療ガイドライン、作成統括委員長 | 有 | NA | デクスメデトミジンは本当に最良な鎮静剤なのか？Consの立場からの考察、第45回日本集中治療医学会学術集会 重症肺胞出血に対してVVEVMOを使用した1例、第45回日本集中治療医学会学術集会  急性呼吸促迫症候群に対する個別化医療、THE LUNG-perspectives | NA |
| Sadatomo Tasaka | ①日本呼吸器学会、②理事、③2018年4月～現在 | NA | NA | ①Prognostic values of the Berlin definition criteria, blood lactate level, and fibroproliferative changes on high-resolution computed tomography in ARDS patients、② BMC Pulmonary Medicine | NA | NA |
| Shinichiro Ohshimo | NA | J-SSCG2020，アカデミック委員．日本蘇生協議会（JRC) 蘇生ガイドライン，委員．国際蘇生連絡委員会（ILCOR）心肺蘇生ガイドライン，委員 | 有 | NA | NA | NA |
| Muneyuki Takeuchi | ①日本小児集中治療研究会、②理事、③2018年から2020年 | the international Pediatric Acute Lung Injury Consensus Conference update (PALICC 2)、作成委員 | NA | NA | NA | NA |
| Kazuya Ichikado | NA | 特発性間質性肺炎　診断と治療の手引き第3版、分担執筆者 | NA | Prediction of prognosis for acute respiratory distress syndrome with thin-section CT: validation in 44 cases. PMID: 16293804、Radiology  Fibroproliferative changes on high-resolution CT in the acute respiratory distress syndrome predict mortality and ventilator dependency: a prospective observational cohort study. BMJ Open | NA | NA |
| Moritoki Egi | NA | J-SSCG2020, 日本医学会連合COVID-19 expert opinion, COVID-19薬物療法に関するRapid/Living recommendations | 有 | NA | NA | NA |
| Nobuaki Shime | 理事：日本集中治療医学会、日本呼吸療法医学会、日本小児集中治療研究会　2018-2020 | J-SSCG2020委員　肺炎診療ガイドライン、委員　深在性真菌症診療ガイドライン、委員　J-SSCG2020 COVID-19, Rapid/Living recommendations, Advisory board, 　JAID/JSC感染症治療ガイドライン、委員 | NA | NA | NA | NA |
| Kenji Tsushima | NA |  | NA | NA | NA | NA |
| Satoru Hashimoto | 日本集中治療医学会理事 2018-現在　日本呼吸療法医学会監事　2018-現在　日本ショック学会理事 2018-2021 | J-SSCG2020 委員 | NA | NA | NA | NA |
| Hideto Yasuda | 日本集中治療教育研究会(JSEPTIC)　理事　2019年4月-2021年3月 | J-SSCG2020アカデミック班、JRC Guideline 2020　SR Member | NA | NA | NA | NA |
| Osamu Saito | NA |  | NA | NA | NA | NA |
| Shotaro Matsumoto | NA |  | NA | NA | NA | NA |
| Eishu Nango | 日本プライマリ・ケア連合学会，理事，2016年～，コクランジャパン，監事，2017年～ | てんかん診療ガイドライン2018，外部委員 パーキンソン病診療ガイドライン2018，外部委員 リハビリテーション栄養学会診療ガイドライン2018，診療ガイドライン作成協力委員 特発性肺線維症の治療ガイドライン2017，協力者 腹膜透析ガイドライン2019，外部評価委員 | NA | NA | NA | NA |
| Yoshitaka Aoki | NA | J-SSCG2020、アカデミックガイドライン推進班 兼 PAD班WG Member | NA | NA | ARDS診療ガイドライン2021へ向けて GRADEシステムの課題と展望、第46回日本集中治療医学会学術集会 | NA |
| Yusuke Iizuka | NA | NA | NA | NA | NA | NA |
| Tadashi Ishihara | NA | J-SSCG2020、SR班班長、サポート委員、J-SSCG2020 COVID-19, Rapid/Living recommendations, CQ班長 | NA | NA | NA | NA |
| Satoshi Okamori | NA | NA | NA | NA | *5-10参照 | NA |
| Tetsuro Kamo | NA | NA | NA | ①Prognostic values of the Berlin definition criteria, blood lactate level, and fibroproliferative changes on high-resolution computed tomography in ARDS patients、②BMC Pulm Med. 2019 Feb 11;19(1):37. | NA | NA |
| Yutaka Kondo | NA | J-SSCG2020、WG委員；JRC Guideline 020,Neuro-resuscitation, 2022年改訂版非心臓手術における合併心疾患の評価と管理に関するガイドライン、協力委員：熱中症診療ガイドライン、作成委員 | NA | NA | NA | NA |
| Masaaki Sakuraya | NA | J-SSCG2020、アカデミック推進班 | 有 | NA | ＊３、４ | NA |
| Mikio Nakajima | NA | ARDS2016 SR委員 | NA | NA | NA | NA |
| Yasuhiro Norisue | NA | J-SSCG2020、WG | 有 | NA | NA | NA |
| Kenichiro Hayashi | NA | NA | NA | NA | NA | NA |
| Tatsuma Fukuda | NA | J-SSCG2020 アカデミックガイドライン推進班 JRC guideline 2020 ALS | NA | NA | NA | NA |
| Shinya Miura | NA | J-SSCG2020、アカデミック班、WG、SRグループ | NA | NA | NA | NA |
| Tomoaki Yatabe | NA | J-SSCG2020、委員 | NA | NA | NA | NA |
| Koichi Ando | NA | NA | NA | NA | *5-10 | NA |
| Yohei Okada | NA | J-SSCG2020, SR Team member, 「新型コロナウイルス感染症の流行を踏まえた 熱中症予防に関する提言」、日本救急医学会　熱中症及び低体温症に関する委員会　委員 | NA | NA | *5-11 | NA |
| Masayuki Ozaki | NA | J-SSCG2020、WG Member　SR Member | NA | NA | NA | NA |
| Jun Kataoka | NA | NA | NA | NA | NA | NA |
| Shunsuke Taito | NA | ①理学療法ガイドライン第2版、②SR班 / ①J-SSCG2020②アカデミックガイドライン推進班、WG、SR/ ①日本版重症患者リハビリテーションガイドライン2022に②委員 | NA | NA | NA | NA |
| Chihiro Narita | NA | J-SSCG2020　SR Team member, J-SSCG2020 COVID-19, Rapid/Living recommendations Task force Member | NA | NA | NA | NA |
| Tetsuro Nishimura | NA | J-SSCG2020　SR Team member | NA | NA | NA | NA |
| Tomoyuki Masuyama | NA | NA | NA | NA | *3, 4 | NA |
| Takuo Yoshida | ABCD-sonography, 理事, 2014年5月～現在 | NA | NA | NA | *5-10 | NA |
| Hiroshi Yonekura | NA | ・J-SSCG2020　SR Team member  ・周術期禁煙ガイドライン、WG Member ・ 術後痛ガイドライン（プラクティカルガイド）、WG Member ・医療情報データベースを用いる研究のためのOutcome Definition Repository Task force委員、WG Member | NA | NA | Early versus late tracheostomy in patients with traumatic brain injury、Intensive Care Medicine | NA |
|  |  |  |  |  |  |  |
|  |  |  |  |  |  |  |
| Aiko Masunaga | NA | NA | NA | NA | NA | NA |
| Aiko Tanaka | NA | NA | NA | NA | NA | NA |
| Akihiko Inoue | NA | NA | NA | NA | NA | NA |
| Akiko Higashi | NA | NA | NA | NA | *6 | NA |
| Atsushi Tanikawa | NA |  | NA | NA | NA | NA |
| Atsushi Ujiro | NA | NA | NA | NA | NA | NA |
| Chihiro Takayama | NA | NA | NA | NA | NA | NA |
| Daisuke Kasugai | NA | J-SSCG2020　SR Team member | NA | NA | NA | NA |
| Daisuke Kawakami | NA |  | NA | NA | NA | NA |
| Daisuke Ueno | NA | NA | NA | NA | NA | NA |
| Daizoh Satoh | NA | NA | NA | NA | NA | NA |
| Dongli | NA | NA | NA | NA | NA | NA |
| Emiko Nakataki | NA | NA | NA | NA | NA | NA |
| Erina Tabata | NA |  | NA | NA | *7 | NA |
| Futoshi Kotajima | NA | NA | NA | NA | NA | NA |
| Go Ishimaru | NA | J-SSCG2020　SR Team member, J-SSCG2020 COVID-19, Rapid/Living recommendations 2．Task force Member | NA | NA | NA | NA |
| Haruhiko Hoshino | NA | NA | NA | NA | NA | NA |
| Hideki Yoshida | NA | NA | NA | NA | NA | NA |
| Hidetaka Iwai | NA | NA | NA | NA | NA | NA |
| Hiroaki Nakagawa | NA | NA | NA | NA | *7 | NA |
| Hiroko Sugimura | NA | NA | NA | NA | NA | NA |
| Hiromichi Narumiya | NA | NA | NA | NA | NA | NA |
| Hiroshi Nakamura | ①中性脂肪学会、②デジタル担当理事、③2020年〜現在 | 中性脂肪蓄積心筋血管症　成果報告2020年度版－診断基準2020・分類・鑑別診断－、委員 | NA | NA | ①情動と痛みの数値化を目的としたストレスセンサの開発とオンライン診療への期待、②日本病院総合診療医学会雑誌2021 | 運動と食事による行動変容の観察研究 |
| Hiroshi Sugimoto | NA | NA | NA | NA | *5 | NA |
| Hiroyuki Hashimoto | NA | NA | NA | NA | *7 | NA |
| Hiroyuki Ito | NA | NA | NA | NA | *5 | NA |
| Hisashi Dote | NA |  | NA | NA | NA | NA |
| Hisashi Imahase | NA | J-SSCG2020　SR Team member | NA | NA | NA | NA |
| Hitoshi Sakamoto | NA | NA | NA | NA | NA | NA |
| Hitoshi Sato | NA | NA | NA | NA | NA | NA |
| Ichiro Osawa | NA | NA | NA | NA | NA | NA |
| Jun Hamaguchi | NA | NA | NA | NA | NA | NA |
| Jun Kamei | NA | J-SSCG2020　SR Team member | NA | NA | NA | NA |
| Jun Maki | NA | J-SSCG2020　SR Team member | NA | NA | NA | NA |
| Jun Sugihara | NA | NA | NA | NA | NA | NA |
| Jun Takeshita | NA | J-SSCG2020　SR Team member | NA | NA | NA | NA |
| Junichi Fujimoto | NA | NA | NA | NA | NA | NA |
| Junichi Ishikawa | NA | NA | NA | NA | NA | NA |
| Junko Kosaka | NA | NA | NA | NA | NA | NA |
| Junpei Shibata | NA | NA | NA | NA | NA | NA |
| Katsuhiko Hashimoto | NA | J-SSCG2020　SR Team member | NA | NA | NA | NA |
| Kazuki Kikuyama | NA | NA | NA | NA | NA | NA |
| Kazushige Shimizu | NA | NA | NA | NA | NA | NA |
| Kazuya Okada | NA | NA | NA | NA | NA | NA |
| Keishi Kawano | NA | NA | NA | NA | NA | NA |
| Keisuke Anan | NA | IPFの治療ガイドライン, 協力者, 肺がん検診エビデンスレポート作成会議, SR Member | NA | NA | *6, 10 | NA |
| Keisuke Ota | NA | NA | NA | NA | NA | NA |
| Ken-ichi Kano | NA | J-SSCG2020　SR Team member | NA | NA | NA | NA |
| Kengo Asano | NA | NA | NA | NA | NA | NA |
| Kenichi Hondo | NA | NA | NA | NA | NA | NA |
| Kenji Ishii | NA | NA | NA | NA | NA | NA |
| Kensuke Fujita | NA | NA | NA | NA | *6 | NA |
| Kenta Ogawa | NA | J-SSCG2020　SR Team member | NA | NA | *3,4 | NA |
| Kentaro Ito | NA | NA | NA | NA | NA | NA |
| Kentaro Tokunaga | NA | J-SSCG2020　SR Team member | NA | NA | NA | NA |
| Kenzo Ishii | NA | NA | NA | NA | NA | NA |
| Kohei Kusumoto | NA | NA | NA | NA | NA | NA |
| Kohei Ohta | NA | J-SSCG2020　SR Team member | NA | NA | NA | NA |
| Kohei Takimoto | NA | NA | NA | NA | NA | NA |
| Kohei Yamada | NA | J-SSCG2020　SR Team member | NA | NA | NA | The efficacy of albumin with diuretics in the mechanically ventilated patients with hypoalbuminemia: systematic review and meta-analysis |
| Koichi Naito | NA | NA | NA | NA | NA | NA |
| Koichi Yamashita | NA | NA | NA | NA | NA | NA |
| Koichi Yoshinaga | NA | NA | NA | NA | NA | NA |
| Kota Yamauchi | NA | NA | NA | NA | NA | NA |
| Maki Murata | NA | NA | NA | NA | *8,9 | NA |
| Makiko Konda | NA | NA | NA | NA | NA | NA |
| Manabu Hamamoto | NA | NA | NA | NA | NA | NA |
| Masaharu Aga | NA | NA | NA | NA | NA | NA |
| Masahiro Kashiura | NA | J-SSCG2020　 ②JRC Guideline 2020 | NA | NA | NA | NA |
| Masahiro Katsura | NA | NA | NA | NA | NA | NA |
| Masami Ishikawa | NA | J-SSCG2020　SR Team member, JRC Guideline 2020、BLS part chair | NA | NA | NA | NA |
| Michihiko Kono | NA | NA | NA | NA | NA | NA |
| Minoru Hayashi | NA | J-SSCG2020　SR Team member | NA | NA | NA | NA |
| Mitsuhiro Abe | NA | NA | NA | NA | NA | NA |
| Mitsunori Sato | NA | NA | NA | NA | NA | NA |
| Mizu Sakai | NA | NA | NA | NA | NA | NA |
| Motoshi Kainuma | NA | NA | NA | NA | NA | NA |
| Naohisa Masunaga | NA | J-SSCG2020　SR Team member | NA | NA | NA | NA |
| Naoki Tominaga | NA | J-SSCG2020　SR Team member | NA | NA | NA | NA |
| Naoya Iguchi | NA | J-SSCG2020　SR Team member | NA | NA | NA | NA |
| Natsuki Nakagawa | NA | がん薬物療法に伴う神経障害診療ガイドライン2022年版、SR委員 | NA | NA | *8,9 | NA |
| Nobumasa Aoki | NA | NA | NA | NA | NA | NA |
| Norihiro Nishioka | NA | J-SSCG2020　SR Team member ①Guidelines for Management of Vasculitis Syndrome2022, ②SR Member | NA | NA | NA | NA |
| Norihisa Miyashita | NA | NA | NA | NA | NA | NA |
| Nozomu Seki | NA | NA | NA | NA | NA | NA |
| Ryo Fujii | NA | J-SSCG2020　SR Team member | NA | NA | NA | NA |
| Ryo Ikebe | NA | NA | NA | NA | NA | NA |
| Ryo Uchimido | NA | NA | NA | NA | NA | NA |
| Ryohei Yamamoto | NA | NA | NA | NA | NA |  |
| Ryosuke Imai | NA | NA | NA | NA | *10 | NA |
| Ryota Tate | NA | NA | NA | NA | NA | NA |
| Ryuhei Sato | NA | NA | NA | NA | NA | NA |
| Sachiko Miyakawa | NA | NA | NA | NA | NA | NA |
| Satoru Robert Okazaki | NA | NA | NA | NA | NA | NA |
| Satoshi Hokari | NA | NA | NA | NA | *3, 4 | NA |
| Satoshi Kazuma | NA | NA | NA | NA | NA | NA |
| Satoshi Nakano | NA | NA | NA | NA | NA | NA |
| Satoshi Tetsumoto | NA |  | NA | NA | NA | NA |
| Satoshi Yoshimura | NA | NA | NA | NA | NA | NA |
| Seisuke Okazawa | NA | NA | NA | NA | NA | NA |
| Shigenori Yoshitake | NA | NA | NA | NA | *7 | NA |
| Shin-etsu Hoshi | NA | NA | NA | NA | NA | NA |
| Shingo Ohki | NA | NA | NA | NA | NA | NA |
| Shinichi Kai | NA | NA | NA | NA | *6 | NA |
| Shintaro Sato | NA | NA | NA | NA | NA | NA |
| Shodai Yoshihiro | NA | J-SSCG2020　SR Team member, 重症患者におけるリハビリテーション診療ガイドラインJ-ReCIP 2022,アカデミック班 | NA | NA | NA | NA |
| Shoichi Ihara | NA | NA | NA | NA | NA | NA |
| Shota Yamamoto | NA | 門脈圧亢進症診療における部分脾動脈塞栓術の手技に関するガイドライン、ガイドライン作成Member（システマティックレビュー担当） | NA | NA | *7 | NA |
| Shunichi Koide | NA | NA | NA | NA | NA | NA |
| Shunsuke Kimata | NA | NA | NA | NA | NA | NA |
| Shunsuke Saito | NA | NA | NA | NA | NA | NA |
| Shunsuke Yasuo | NA | NA | NA | NA | *8,9 | NA |
| Shusuke Sekine | NA | J-SSCG2020　SR Team member | NA | NA | NA | NA |
| Soichiro Mimuro | NA | NA | NA | NA | NA | NA |
| Soichiro Wada | NA | NA | NA | NA | NA | NA |
| Sosuke Sugimura | NA | NA | NA | NA | NA | NA |
| Tadashi Kaneko | NA | NA | NA | NA | NA | NA |
| Tadashi Nagatomo | NA | J-SSCG2020　SR Team member | NA | NA | NA | NA |
| Takaaki Maruhashi | NA | ①JRC Guideline 2020②ALS WG | NA | NA | NA | NA |
| Takahiro Tamura | NA | ①大量出血症例に対する血液製剤の適正な使用のガイドライン、②SR team member | NA | NA | NA | NA |
| Takanori Ohno | NA | J-SSCG2020, WG | NA | NA | NA | NA |
| Takashi Hongo | NA | NA | NA | NA | NA | NA |
| Takashi Ichiyama | NA | NA | NA | NA | NA | NA |
| Takashi Niwa | NA | NA | NA | NA | NA | NA |
| Takashi Ueji | NA | NA | NA | NA | NA | NA |
| Takayuki Ogura | NA | NA | NA | NA | NA | NA |
| Takeshi Kawasaki | NA | 結核診療ガイドライン, SR班 | NA | NA | *8, 9 | NA |
| Takeshi Tanaka | NA | NA | NA | NA | *5, ①演題名:(1) Serotonin and vascular permeability (Serotonin beyond the brain) Analysis of Serotonin in Sepsis and ARDS.、②発表学会：APSR 2018 in Taipei, Taiwan | 急性肺障害患者におけるセロトニンの動態解析 |
| Takeshi Umegaki | NA | J-SSCG2020　SR Team member | NA | NA | NA | NA |
| Taku Furukawa | NA | NA | NA | NA | NA | NA |
| Taku Omura | NA | NA | NA | NA | NA | NA |
| Takumi Nagao | NA | NA | NA | NA | NA | NA |
| Takuro Nakashima | NA | NA | NA | NA | NA | NA |
| Takuya Mayumi | NA | J-SSCG2020　SR Team member ①COVID-19リビングガイドライン②Task force Member | NA | NA | NA | NA |
| Takuya Taniguchi | NA |  | NA | NA | *10 | NA |
| Takuya Yoshida | NA | NA | NA | NA | NA | NA |
| Tatsutoshi Shimatani | NA | NA | NA | NA | NA | NA |
| Tatsuya Kawasaki | NA | NA | NA | NA | NA | NA |
| Teppei Murata | NA | J-SSCG2020　SR Team member | NA | NA | NA | NA |
| Tetsuro Kamo | NA |  | NA | NA | NA | NA |
| Tetsuro Terayama | NA | J-SSCG2020, アカデミック班 | NA | NA | *10 | NA |
| Tetsuya Sato | NA | J-SSCG2020　SR Team member | NA | NA | NA | NA |
| Tohru Sawamoto | NA | NA | NA | NA | NA | NA |
| Tomohiro Takehara | NA | NA | NA | NA | NA | NA |
| Tomomi Ueda | NA | NA | NA | NA | NA | NA |
| Tomoya Katsuta | NA | NA | NA | NA | NA | NA |
| Tomoya Nishino | NA | NA | NA | NA | NA | NA |
| Toshiki Yokoyama |  |  |  |  |  |  |
| Ushio Higashijima | NA | NA | NA | NA | NA | NA |
| Wataru Iwanaga | NA | NA | NA | NA | NA | NA |
| Yasushi Inoue | NA | NA | NA | NA | NA | NA |
| Yasushi Nakano | NA | Clinical guidelines for respiratory symptoms in Cancer patients, WG member | NA | NA | NA | NA |
| Yoshiaki Iwashita | NA | NA | NA | NA | *6 | NA |
| Yoshie Yamada | NA | NA | NA | NA | *5 |  |
| Yoshifumi Koukei | NA | NA | NA | NA | NA | NA |
| Yoshifumi Kubota | NA | NA | NA | NA | NA | NA |
| Yoshihiro Hagiwara | NA | NA | NA | NA | NA | NA |
| Yoshihiro Suido | NA | NA | NA | NA | *7 | NA |
| Yoshihiro Tomioka | NA | J-SSCG2020　SR Team member | NA | NA | NA | NA |
| Yoshihisa Fujimoto | NA | JRC Guideline 2020, Neuro-resuscitation, SR member | NA | NA | NA | NA |
| Yoshihito Fujita | NA | NA | NA | NA | NA | NA |
| Yoshikazu Yamaguchi | NA | NA | NA | NA | NA | NA |
| Yoshimi Nakamura | NA | J-SSCG2020　SR Team member | NA | NA | NA | NA |
| Yoshinobu Abe | NA | NA | NA | NA | NA | NA |
| Yoshitomo Eguchi | NA | NA | NA | NA | NA | NA |
| Yoshiyasu Oshimal | NA | NA | NA | NA | *6 | NA |
| Yosuke Fukuda | NA | NA | NA | NA | *5 | NA |
| Yudai Iwasaki | NA | NA | NA | NA | NA | NA |
| Yuichi Yasufuku | NA | ①理学療法ガイドライン第2版、②システマティックレビュー委員（間質性肺炎） | NA | NA | NA | NA |
| Yuji Shono | NA | NA | NA | NA | NA | NA |
| Yuka Nakatani | NA | NA | NA | NA | NA | NA |
| Yuki Kishihara | NA | NA | NA | NA | *3, 4 | NA |
| Yuki Nakamori | NA | J-SSCG2020　SR Team member | NA | NA | ①右室- 肺動脈カップリングの視点による循環管理で右室機能不全を併発したARDS 患者を救命する ②循環制御 | NA |
| Yukie Ito | NA | NA | NA | NA | NA | NA |
| Yuko Tanabe | NA | 乳癌診療ガイドライン、病理小委員会委員 | NA | NA | *6 | NA |
| Yusuke Nagamine | NA | NA | NA | NA | NA | NA |
| Yuta Nakamura | NA | NA | NA | NA | NA | NA |
|  |  |  |  |  |  |  |
|  |  |  |  |  |  |  |
|  |  |  |  |  |  |  |
| Nana Arai | NA | NA | NA | NA | NA | NA |
| Kokichi Andoh | NA | J-SSCG, WG member | NA | NA | NA | NA |
| Keiko Ishimura |  |  | NA | NA | NA | NA |
| Hiroshi Okuda | NA | J-SSCG2020　SR Team member | NA | NA | NA | NA |
| Ryo Kozu | 日本呼吸療法医学会，理事2018-2020，日本呼吸ケア・リハビリテーション学会，理事2018-2020 | 理学療法ガイドライン第2版（日本理学療法士学会），作成統括委員 | 有 | NA | NA | NA |
| Ryutaro Seo | NA | NA | 有 | NA | NA | NA |
| Eriko Takezawa | NA | NA | 有 | NA | NA | NA |
| Satoshi Doi | NA | NA | NA | NA | ① 論文名：長期間の多職種連携による離床により人工呼吸器からの離脱に成功したacute respiratory distress syndrome（ARDS）の1症例、②雑誌名：四国医学雑誌 | NA |
| Makoto Miki | 日本結核・非結核性抗酸菌症学会、理事、2017年から現在 | 日本結核・非結核性抗酸菌症学会結核診療ガイドライン、作成医員 | NA | NA | NA | NA |
| Masashi Morizane | NA | NA | NA | NA | NA | NA |
| Hitoshi Yokoyama | NA | NA | NA | NA | NA | NA |
| Hiroshi Yoshikawa | NA | NA | NA | NA | NA | NA |
| Takeshi Yoshida | NA | J-SSCG2020、WG Member / the Pleural Pressure Working Group, Acute Respiratory Failure Section of the European Society of Intensive Care Medicine, Panelist | NA | NA | ＊１ | ＊2 |
| Yuki Enomoto | NA | J-SSCG2020　SR Team member | NA | NA | NA | NA |
| Tatsuya Kawasaki | NA | ①J-SSCG2020②委員；①J-SSCG2020特別編 COVID-19薬物療法に関するRapid/Living recommendations、②アドバイザリーボード；①COVID―19急性呼吸不全への人工呼吸とECMO　小児における基本的注意事項、②WG Member | NA | NA | NA | NA |
| Satoshi Nakagawa | 1. World Federation of Pediatric Intensive and Critical Care Societies, 2. President, 3, from December 2020 to present time | 1. COVID-19 PICU guidelines: for high- and limited-resource settings, 2. Author | NA | NA | NA | NA |
| Shunsuke Nosaka | ①一般社団法人日本救急放射線研究会、②理事、③2018年4～現在 | ①胆道閉鎖症診療ガイドライン、②ガイドライン作成グループ | NA | NA | NA | NA |
| Kensuke Yagi | NA | NA | NA | NA | NA | NA |
| Akimasa Yamatani | NA | NA | NA | NA | NA | NA |
| Takuya Hayashi | NA | J-SSCG2020 | NA | NA | NA | NA |
| Erika Ota | ①コクランジャパン②副理事長③2018-2020 | ①看護ケアのための摂食・嚥下時の誤嚥・咽頭残留に関するアセスメントに関する診療ガイドライン②診療ガイドライン作成の専門家 ①う蝕に関する診療ガイドライン②診療ガイドライン作成の専門家 ①WHO antenatal care guideline ②WHO Guideline development group | NA | NA | NA | NA |
| Toshio Fukuoka | NA | ①JRC Guideline 2020, BLS/ALS②外部評価委員 | NA | NA | NA | NA |
| Kazuma Yamakawa | NA | J-SSCG2020委員長補佐 | NA | NA | NA | NA |
| Hidemichi Yuasa | NA | 顎関節症診療ガイドライン、副委員長・腹膜透析ガイドライン、外部委員、口腔癌診療ガイドライン、アドバイザー | NA | NA | NA | NA |

(Abbreviation)

J-SSCG2020: Japanese Surviving Sepsis Clinical Guideline 2020, NA: Not applicable, SR: Systematic review, WG: Working group, JRC:Japan Resuscitation council

＜Detail of Intellectual COI＞

＊1

C-2)

Takeshi Yoshida, Yuji Fujino. Monitoring the patient for a safe-assisted ventilation Current Opinion in Critical Care Publish Ahead of Print 2020年12月3日 4) Sami Hraiech, Takeshi Yoshida, Djillali Annane, Abhijit Duggal, Vito Fanelli, Arnaud Gacouin, Leo Heunks, Samir Jaber, Peter D. Sottile, Laurent Papazian. Myorelaxants in ARDS patients. Intensive Care Medicine 2020年11月7日 5) Mario Menk, Elisa Estenssoro, Sarina K. Sahetya, Ary Serpa Neto, Pratik Sinha, Arthur S. Slutsky, Charlotte Summers, Takeshi Yoshida, Thomas Bein, Niall D. Ferguson. Current and evolving standards of care for patients with ARDS. Intensive Care Medicine 2020年11月6日. 6) Ewan C. Goligher, Annemijn H. Jonkman, Jose Dianti, Katerina Vaporidi, Jeremy R. Beitler, Bhakti K. Patel, Takeshi Yoshida, Samir Jaber, Martin Dres, Tommaso Mauri, Giacomo Bellani, Alexandre Demoule, Laurent Brochard, Leo Heunks. Clinical strategies for implementing lung and diaphragm-protective ventilation: avoiding insufficient and excessive effort. Intensive Care Medicine 2020年11月2日 7) Luca Bastia, Doreen Engelberts, Kohei Osada, Bhushan H Katira, L Felipe Damiani, Takeshi Yoshida, Lu Chen, Niall D Ferguson, Marcelo B P Amato, Martin Post, Brian P Kavanagh, Laurent Brochard. Role of PEEP and Regional Transpulmonary Pressure in Asymmetrical Lung Injury. American Journal of Respiratory and Critical Care Medicine 2020年10月22日 8) Yusuke Enokidani, Akinori Uchiyama, Takeshi Yoshida, Ryuichiro Abe, Tomonori Yamashita, Yukiko Koyama, Yuji Fujino. Effects of Ventilatory Settings on Pendelluft Phenomenon During Mechanical Ventilation. Respiratory Care 2021;66(1) 1-10. 9) Ewan C. Goligher, Martin Dres, Bhakti K. Patel, Sarina K. Sahetya, Jeremy R. Beitler, Irene Telias, Takeshi Yoshida, Katerina Vaporidi, Domenico Luca Grieco, Tom Schepens, Giacomo Grasselli, Savino Spadaro, Jose Dianti, Marcelo Amato, Giacomo Bellani, Alexandre Demoule, Eddy Fan, Niall D. Ferguson, Dimitrios Georgopoulos, Claude Guérin, Robinder G. Khemani, Franco Laghi, Alain Mercat, Francesco Mojoli, Coen A. C. Ottenheijm, Samir Jaber, Leo Heunks, Jordi Mancebo, Tommaso Mauri, Antonio Pesenti, Laurent Brochard. Lungand Diaphragm-Protective Ventilation. American Journal of Respiratory and Critical Care Medicine 2020;202(7) 950-961. 10) Tomonori Yamashita, Akinori Uchiyama, Yukiko Koyama, Takeshi Yoshida, Aiko Tanaka, Yuji Fujino. Effects of alkaline agents on respiratory characteristics in rabbit models of respiratory failure. Respiratory Physiology & Neurobiology 2020;280 103485. 11) Takeshi Yoshida. The Dark Side of Spontaneous Breathing during Noninvasive Ventilation. From Hypothesis to Theory. American Journal of Respiratory and Critical Care Medicine 2020;202(4) 482-484. 12) 吉田 健史. 【最新 救急医療機器総覧 2020】食道内圧モニター 救急医学 2020;44(7) 802-809. 13) 橋本 明佳,吉田 健史.【ICU治療指針 III】手術・麻酔と周術期管理 呼吸器外科手術の周術期管理 救急・集中治療 2020;31(4) 1515-1516. 14) 妙中 浩紀,吉田 健史.【ICU治療指針 III】手術・麻酔と周術期管理 非心臓手術における合併心疾患の術前評価と管理 救急・集中治療 2020;31(4) 1539-1543. 15) 小山 有紀子,吉田 健史.【ICU治療指針 III】手術・麻酔と周術期管理 非肺手術における低肺機能患者の術前評価と管理 救急・集中治療 2020;31(4) 1544-1548. 16) Takeshi Yoshida, Domenico L. Grieco, Laurent Brochard, Yuji Fujino. Patient self-inflicted lung injury and positive end-expiratory pressure for safe spontaneous breathing Current Opinion in Critical Care 2020;26(1) 59-65. 17) Laurent Brochard, Takeshi Yoshida, Marcelo Amato. Reply to Frerichs et al.: Simple Electrical Impedance Tomography Measures for the Assessment of Ventilation Distribution American Journal of Respiratory and Critical Care Medicine 2020;201(3) 388-388. 18) Arnaud W. Thille, Takeshi Yoshida. High-pressure versus high-flow: What should we target in acute respiratory failure? American Journal of Respiratory and Critical Care Medicine. 2020; 201:265-266. 19) 吉田健史. 呼吸管理 2020-’21-ガイドライン,スタンダード,論点そして私見-IV.人工呼吸 肺保護換気 救急・集中治療 2020;32(1) 20) Sourav Kumar Mukhopadhyay, Michael Zara, Irene Telias, Lu Chen, Remi Coudroy, Takeshi Yoshida, Laurent Brochard, Sridhar Krishnan. A Singular Spectrum Analysis-Based Data-Driven Technique for the Removal of Cardiogenic Oscillations in Esophageal Pressure Signals IEEE Journal of Translational Engineering in Health and Medicine 2020;30(8):3300211 21) Maurizio Cereda, Yi Xin, Alberto Goffi, Jacob Herrmann, David W. Kaczka, Brian P. Kavanagh, Gaetano Perchiazzi, Takeshi Yoshida, Rahim R. Rizi. Imaging the Injured Lung Anesthesiology 2019;131(3) 716-749. 22) Yoshida T, Piraino T, Lima CAS, Kavanagh BP, Amato MBP, Brochard L. Regional ventilation displayed by electrical impedance tomography as an incentive to decrease positive end-expiratory pressure. American Journal of Respiratory and Critical Care Medicine. 2019;200:933-937. 23) Yoshida T, Kavanagh BP, Brochard L. Early Neuromuscular Blockade in the Acute Respiratory Distress Syndrome New England Journal of Medicine. 2019 Aug 22;381(8):786-787 24) 妙中 浩紀,吉田 健史,内山 昭則,藤野 裕士. 食道内圧の測定方法 人工呼吸 2019;36(2) 151-157. 25) 髭野 亮太,吉田 健史. 【換気モードを整理する】肺保護換気 ICUとCCU 2019;43(6) 317-322. 26) Yoshida T, Amato MBP, Kavanagh BP, Fujino Y. Impact of spontaneous breathing during mechanical ventilation in acute respiratory distress syndrome. Current Opinion in Critical Care 2019;25(2):192-198. 27) Cereda M, Xin Y, Goffi A, Herrmann J, Kaczka DW, Kavanagh BP, Perchiazzi G, Yoshida T, Rizi RR. Imaging the Injured Lung: Mechanisms of Action and Clinical Use. Anesthesiology. 2019;131(3):716-749 28) Yoshida T, Grieco DL, Brochard L. Guiding ventilation with transpulmonary pressure. Intensive Care Medicine 2019;45(4):535-538. 29) Koide M, Uchiyama A, Yamashita T, Yoshida T, Fujino Y. Attaining low tidal volume ventilation during assisted ventilation in sedated subjects. Respir Care 2019;64:890-898. 30) Yoshida T, Nakamura MAM, Morais CCA, Amato MBP, Kavanagh BP. Reverse Triggering Causes Injurious Inflation during Mechanical Ventilation. American Journal of Respiratory and Critical Care Medicine 2018;198:1096-1099. 31) Pham T, Telias I, Piraino T, Yoshida T, Brochard L. Asynchrony Consequences & Management Critical Care Clinics 2018 Jul;34(3):325-341. 32) Koyama Y, Uchiyama A, Yoshida J, Yoshida T, Yamashita T, Fujino Y. A Comparison of the Adjustable Ranges of Inspiratory Pressurization During Pressure Controlled Continuous Mandatory Ventilation of 5 ICU Ventilators. Respir Care 2018 Jul;63(7):849-858. 33) Yoshida T, Engelberts D, Otulakowski G, Katira B, Ferguson ND, Brochard L, Amato MBP, Kavanagh BP. Continuous Abdominal Negative Pressure: Mechanism of Action and Comparison with Prone Position Journal of Applied Physiology 2018 Jul 1;125(1):107-116. 34) Yoshida T, Engelberts D, Otulakowski G, Katira B, Post M, Ferguson ND, Brochard L, Amato MBP, Kavanagh BP. Continuous Abdominal Negative Pressure Reduces Ventilator-Induced Lung Injury in a Porcine Model Anesthesiology 2018 Jul;129(1):163-172 35) Alex Gordon, Kevin Ai Xin Jue Luo, Rami Saab, Doreen Engelberts, Brian Kavanagh, Takeshi Yoshida, Thomas Looi. A Device for Improving Oxygenation in Patients With Acute Respiratory Distress Syndrome BIOMED 2018;V001T03A006. 36) Yoshida T, Amato MBP, Kavanagh BP. Understanding Spontaneous vs. Ventilator Breaths: Impact and Monitoring. Intensive Care Medicine 2018 Dec;44(12):2235-2238. 37) Yoshida T, Brochard L. Esophageal Pressure Monitoring: Why, when and how? Current Opinion in Critical Care 2018 Jun;24(3):216-222. 38) Katira BH, Engelberts D, Otulakowski G, Giesinger RE, Yoshida T, Post M, Kuebler WM, Connelly KA, Kavanagh BP. Abrupt Deflation after Sustained Inflation Causes Lung Injury. American Journal of Respiratory and Critical Care Medicine 2018;198(9) 1165-1176. 39) Yoshida T, Amato MBP, Grieco DL, Chen L, Lima CAS, Roldan R, Morais CCA, Gomes S, Costa ELV, Cardoso PFG, Charbonney E, Richard JM, Brochard L, Kavanagh BP. Esophageal Manometry and Regional Transpulmonary Pressure in Lung Injury. American Journal of Respiratory and Critical Care Medicine 2018;197:1018-1026. 40) Morais CCA*, Koyama Y*, Yoshida T*, Plens GM, Gomes S, Lima CLAS, Ramos OP, Pereira SM, Kawaguchi N, Yamamoto H, Uchiyama A, Borges JB, Vidal Melo MF, Tucci MR, Amato MBP, Kavanagh BP, Costa ELV, Fujino Y. High Positive End-Expiratory Pressure Renders Spontaneous Effort Non-Injurious. *equally contributed. American Journal of Respiratory and Critical Care Medicine 2018;197:1285-1296. 41) Yoshida T, Engelberts D, Otulakowski G, Katira B, Post M, Ferguson ND, Brochard L, Amato MBP, Kavanagh BP. Continuous Negative Abdominal Pressure Recruits Lungs at Lower Distending Pressures. American Journal of Respiratory and Critical Care Medicine 2018;197:534-537. 42) Yoshida T, Brochard L. Ten tips to facilitate understanding and clinical use of esophageal pressure manometry. Intensive Care Medicine 2018 Feb;44(2):220-222 43) 海老島 宏典,吉田 健史,内山 昭則. 人工呼吸管理中の自発呼吸の功罪 日本集中治療医学会雑誌 2018;25(4) 243-248. 44) 吉田健史.【人工呼吸器】人工呼吸器関連肺傷害 不均一な肺含気分布から病態生理を理解する Intensivist 2018;10(3) 499-507. 45) 吉田健史.呼吸器病学TOPICS 2017‐18 8.呼吸管理 人工呼吸管理下の自発呼吸と肺傷害の関係

C-3）

常に肺に寄り添ってますか? 吉田健史 第 42 回日本呼吸療法医学会学術集会 2020年12月20日 自発呼吸関連肺傷害とそれに対する治療法 吉田健史 第 42 回日本呼吸療法医学会学術集会 2020年12月20日 Impact of spontaneous breathing during mechanical ventilation in acute respiratory distress syndrome Takeshi Yoshida 38° CHILEAN INTENSIVE CARE CONGRESS 2020年11月25日 How to Evaluate Spontaneous Breathing Effort without Esophageal Catheter TAKESHI YOSHIDA 2020 Annual Critical Care Quality Control Congress of China 2020年11月13日 Spontaneous breathing caused lung injury assessed by EIT Takeshi Yoshida 2020 Chinese Critical Care Congress 2020年8月9日 The Risk of Spontaneous Breathing in ARDS Takeshi Yoshida The 40th KSCCM Annual Congress 2020年8月1日 自発呼吸関連肺傷害 吉田健史 日本集中治療医学会第4回中国・四国支部学術集会 2020年7月18日 肺保護換気 吉田健史 日本COVID-19対策ECMOnet 人工呼吸/ECMO講習会 2020年6月13日 Spontaneous Breathing in ARDS 吉田健史 第47回日本集中治療医学会学術集会 2020年3月6日 Brian Kavanagh先生追悼講演 「A New Concept for Lung Recruitment: Negative pressure is better than PEEP」 吉田健史 第47回日本集中治療医学会学術集会 2020年3月6日 経肺圧 吉田健史 第4回Okayama Young Intensivist Seminar 2020年1月11日 ARDSに対する肺保護換気戦略 吉田健史 第18回大阪人工呼吸器セミナー 2019年12月21日 急性呼吸不全のモニタリングと対応方法: 一般病棟でできること、集中治療医にまかせること 吉田健史 第14回医療の質・安全学会学術集会 2019年11月29日 CNAPNovel Approach to Prevent VILI TAKESHI YOSHIDA Critical Care Canada Forum2019 2019年11月12日 Spontaneous Breathing & VILI TAKESHI YOSHIDA Critical Care Canada Forum2019 2019年11月11日 ARDS: Close the Lung and Keep It Closed TAKESHI YOSHIDA 2019 Annual Critical Care Quality Control Congress of China 2019年11月8日 Lung Protection: Continuous Negative Abdominal Pressure (CNAP) Is Better Than PEEP TAKESHI YOSHIDA 2019 Annual Critical Care Quality Control Congress of China 2019年11月8日 急性肺傷害に対する呼吸管理法をupdate する 吉田健史 第30回日本急性血液浄化学会学術集会 2019年10月26日 成人ICUの常識 大阪から世界へ！ 新しい呼吸管理を知る自発呼吸関連肺傷害 吉田健史 第35回日本小児外科学会秋季シンポジウム/第27回小児集中治療ワークショップ 2019年10月17日 CNAP Is Better Than PEEP Takeshi Yoshida International Symposium of Mechanical Ventilation, Lima, Peru 2019年9月7日 The darkside of spontaneous breathing International Symposium of Mechanical Ventilation, Lima, Peru 2019年9月6日 人工呼吸管理中の自発呼吸を再考する 「自発呼吸の害」 吉田健史 第 41 回日本呼吸療法医学会学術集会 2019年8月4日 新しい人工呼吸器関連肺傷害の概念 「自発呼吸関連肺傷害」 吉田健史 第 41 回日本呼吸療法医学会学術集会 2019年8月4日 なぜHigh PEEP TrialはNegativeになるのか 吉田健史 第 41 回日本呼吸療法医学会学術集会 2019年8月4日 ARDS: Close the Lung and Keep It Closed Takeshi Yoshida The 13th Congress of Chinese Society of Critical Care Medicine 2019年5月25日 VILI: Spontaneous Breathing Takeshi Yoshida The 13th Congress of Chinese Society of Critical Care Medicine 2019年5月25日 VILI: Protect the Lung with Esophageal Balloon Manometry Takeshi Yoshida The 13th Congress of Chinese Society of Critical Care Medicine 2019年5月25日 Pleural and Esophageal Pressure, What is the Difference? Takeshi Yoshida 30° Smart Meeting Anesthesia Resuscitation Intensive Care, Milan, Italy 2019年5月8日 Protecting the lungs during spontaneous breathing: can esophageal pressure help? Takeshi Yoshida 30° Smart Meeting Anesthesia Resuscitation Intensive Care, Milan, Italy 2019年5月8日 Spontaneous breathing in acute respiratory failure: experimental insights Takeshi Yoshida 30° Smart Meeting Anesthesia Resuscitation Intensive Care, Milan, Italy 2019年5月8日 Positive end-expiratory pressure for safe spontaneous breathing Takeshi Yoshida Lung and Diaphragm-Protective Ventilation Consensus Conference, European Society of Intensive Care Medicine 2019年5月7日 Monitoring Techniques: Electrical Impedance Tomography Takeshi Yoshida Mechanical Ventilation Course 2019 2019年4月10日 Spontaneous Breathing in ARDS Takeshi Yoshida Mechanical Ventilation Course 2019 2019年4月9日 人工呼吸器関連肺傷害 吉田健史 第35回JSEPTICセミナー 2019年3月23日 自発呼吸による肺傷害・非同調による肺傷害 吉田健史 第47回日本集中治療医学会学術集会 ジョイントシンポジウム (日本集中治療医学会・日本呼吸器学会) 2019年3月1日 Spontaneous Breathing in ARDS Takeshi Yoshida 第47回日本集中治療医学会学術集会 TSCCM-JSICM Symposium 2019年3月1日 ARDSに対する肺保護換気戦略 吉田健史 第46回日本集中治療医学会学術集会 リフレッシャーセミナー 2019年2月28日 自発呼吸関連肺傷害とその管理法 吉田健史 第6回ART若手セミナー -Acute Respiratory Treatment 2019年2月14日 自発呼吸関連肺傷害 吉田健史 第17回大阪人工呼吸器セミナー 2018年12月15日 Targeting the abdomen in acute lung injury Takeshi Yoshida Critical Care Canada Forum 2018 2018年11月6日 CNAP is better than PEEP 吉田 健史 Critical Care Canada Forum 2018年11月7日 The darkside of spontaneous breathing 吉田 健史 Critical Care Canada Forum 2018年11月7日 Regional Transpulmonary Pressure in ARDS TAKESHI YOSHIDA 2018Chinese Critical Care Congress 2018年9月 The Darkside of Spontaneous Breathing in ARDS TAKESHI YOSHIDA 2018 Chinese Critical Care Congress 2018年9月 これぞ臨床のコツ これで明日から君も一流だ こんな時は筋弛緩剤を使いなさい 吉田 健史 第40回日本呼吸療法医学会学術集会 2018年8月4日 肺の状態に適した換気設定を探る: 食道内圧測定と経肺圧 吉田 健史 第40回日本呼吸療法医学会学術集会 2018年8月4日 Continuous Negative Abdominal Pressure A New Concept for Critically Injured Lungs 吉田 健史 Andrew Sass-Kortsak Memorial Lecture 2018年6月19日 Spontaneous breathing in ARDS 吉田 健史 Mechanical ventilation Course 2018 2018年6月12日 Patient Self-inflicted Lung Injury 吉田 健史 Lives Forum-Monitoring in Acute Respiratory Failure, European Society of Intensive Care Medicine 2018年5月4日 Shaping the Future 吉田 健史 50th anniversary Practical Day 2018年2月21日 Recent Advances in ARDSthe darkside of spontaneous breathing吉田 健史 分子呼吸器病 2018;22(1) 56‐59.

＊２

１）PRONE POSITIONING AND SPONTANEOUS BREATHING: A FEASIBILITY STUDY (The PROSE study)

２）HIGH FLoW NAsaL CaNNULA IN PEDIATRIC PATIENTS AFTER CaRDIOTHORACIC SURGERY: A RANDOMIZED CONTROLLED TRIAL (OSACA CATS TRIAL)

３）(In Japanese) 電気インピーダンストモグラフィを用いた新型コロナウイルス肺炎による急性呼吸窮迫症候群の呼吸メカニクス解明：前向き観察研究

４）(In Japanese) 急性呼吸窮迫症候群患者における患者-人工呼吸器非同調に対する呼気終末陽圧の効果：クロスオーバーNA作為化比較試験

*3

Efficacy of non-invasive and invasive respiratory management strategies in adult patients with acute hypoxaemic respiratory failure: a systematic review and network meta-analysis. Crit Care. 2021 Nov 29;25(1):414. Sakuraya M, Okano H, Masuyama T, Kimata S, Hokari S.

*4

Respiratory support strategy in adults with acute hypoxemic respiratory failure: A systematic review and network meta-analysis, Hiromu Okano, Masaaki Sakuraya, Tomoyuki Masuyama, Shunsuke Kimata, Satoshi Hokari

*5

Safety and feasibility of lung biopsy in diagnosis of acute respiratory distress syndrome: protocol for a systematic review and meta-analysis. BMJ Open. 2021 Feb 12;11(2):e043600. Fukuda Y, Sugimoto H, Yamada Y, Ito H, Tanaka T, Yoshida T, Okamori S, Ando K, Okada Y.

*6

Safety and harms of bronchoalveolar lavage for acute respiratory failure: A systematic review and meta-analysis. Respir Investig. 2021 Sep 3:S2212-5345(21)00145-3. Anan K, Oshima Y, Ogura T, Tanabe Y, Higashi A, Iwashita Y, Fujita K, Yoshida T, Ando K, Okamori S, Okada Y.

*7

Clinical Utility of Surgical Lung Biopsy for Patients with Acute Respiratory Distress Syndrome: A Systematic Review and Meta-Analysis, Hiroyuki Hashimoto, Shota Yamamoto, Hiroaki Nakagawa, Yoshihiro Suido, Shintaro Sato, Erina Tabata, Satoshi Okamori, Takuo Yoshida, Koichi Ando, Shigenori Yoshitake, Yohei Okada

*8

Safety and adverse events during intrahospital transport of critically ill patients: a systematic review and meta-analysis, Maki Murata, Natsuki Nakagawa, Takeshi Kawasaki, Shunsuke Yasuo, Takuo Yoshida, Koichi Ando, Satoshi Okamori, Yohei Okada

*9

Diagnostic accuracy of urinary antigen tests for legionellosis: A systematic review and meta-analysis, Takeshi Kawasaki, Natsuki Nakagawa, Maki Murata, Shunsuke Yasuo, Takuo Yoshida, Koichi Ando, Satoshi Okamori, Yohei Okada, Respiratory Investigation, 2021

*10

Protocol for a systematic review and meta-analysis of studies on the use of brain natriuretic peptide and N-terminal brain natriuretic peptide levels in the diagnosis of cardiopulmonary edema in acute respiratory failure. Syst Rev. 2021 Dec 16;10(1):314. Terayama T, Taniguchi T, Imai R, Anan K, Yoshida T, Ando K, Okamori S, Okada Y.

*11

Early versus delayed mobilization for in-hospital mortality and health-related quality of life among critically ill patients: a systematic review and meta-analysis, Journal of intensive care 2019
